# Evaluating the quality of shared decision making during the patient-carer encounter: a systematic review of tools

**DOI:** 10.1186/s13104-016-2164-6

**Published:** 2016-08-02

**Authors:** Nathalie Bouniols, Brice Leclère, Leïla Moret

**Affiliations:** 1 Medical Evaluation and Epidemiology Department, PHU11, Saint-Jacques University Hospital, 85, rue Saint-Jacques, 44093 Nantes Cedex, France; 2 EA 4275 SPHERE: biostatistics, Pharmacoepidemiology and Human sciences Research team, Faculty of Pharmaceutical Sciences, University of Nantes, Rue Gaston Veil, 44000 Nantes, France

**Keywords:** Shared decision making, Patient involvement, Measurement, Questionnaire, Methodology, Systematic review

## Abstract

**Background:**

The concept of shared decision making (SDM) has been developing in many countries since the 1990s. The main challenge of SDM, based on the principles of respect for the person’s autonomy, is to improve patients’ participation, should they so wish, in decisions concerning their personal health. To our knowledge, there is only one SDM evaluation tool validated in metropolitan French that does not measure the entire SDM construct. The aim of this review was to identify existing and validated SDM measurement tools to determine which of them could be adapted in French to cover all the dimensions of SDM.

**Methods:**

A systematic literature review was conducted based on articles found in the PubMed and PsycINFO bibliographic databases and published between 2010 and 2014. Studies were included if the main goal of the article was the development and psychometric validation of an SDM measurement tool, not specific to any given disease or situation, in English, French and Spanish. We used the nine essential elements of the Makoul and Clayman’s integrative model to describe the different existing tools.

**Results:**

Nineteen studies were included. Seven new tools had been published since Scholl’s previous review in 2011. We observed a recent spread of the multi-appraiser approach, which combines points of view of patients, healthcare professionals and sometimes external observers. Several models were used for the development of the seven newly identified tools. None of the identified tools assessed the nine elements of the Makoul’s model. Three of these elements, however, were systematically measured in each of the new tools: “defining/explaining the problem”, “patient values/preferences”, and “checking/clarifying understanding”.

**Conclusions:**

We identified several potentially interesting tools for the French context which could cover the whole elements of Makoul’s model. The next step will be the development of a French-language instrument based on these tools.

**Electronic supplementary material:**

The online version of this article (doi:10.1186/s13104-016-2164-6) contains supplementary material, which is available to authorized users.

## Background

The concept of shared decision making (SDM) has been developing both in France [1] and in Anglo-Saxon countries [2] since the 1990s. This concept describes the process during which a decision concerning a patient’s health must be made using a decision making model consisting of two key steps in the relationship between a healthcare professional and a patient: information exchange and deliberation, before making a mutually accepted decision [2]. The healthcare professional and patient share medical information, in particular scientific evidence, and the patient receives the support required to express his/her preferences and to consider the various healthcare-related options, in order to reach an informed joint decision [3, 4].

In France, patients’ demand for information and participation in medical decision were introduced in the eighties by AIDS associations, then reinforced by the tainted blood scandal and in the nineties by cancer patients’ associations [5]. These movements are commonly associated with the rise of what has been called in France ‘‘Democracy in healthcare’’, a concept originating from the growing awareness of the civil society [6]. In the French context, while the Patients’ rights healthcare system quality act of 04 March, 2002 introduced the notion of a patient’s right to know and to decide [1], effective patient participation in SDM remains one of the stakes highlighted in 2013 by the HAS (Haute Autorité de Santé, French National Authority for Health) in a guide entitled “Patient and healthcare professionals: deciding together” [2]. Moreover, SDM represents a growing research field and increasing interest for French research teams. Indeed some studies have been conducted in France and papers published both at the national and international level. They were conducted by multidisciplinary research groups, in various domains in particular cancerology [7–13], psychiatry [14, 15], and SDM itself [16]. Furthermore seminars and congresses in the field of SDM are regularly organized in France for example in psychiatry [17, 18] or oncology [19, 20].

The main interest of SDM resides in improving the participation of patients who so wish, though without imposing, in the decisions concerning them, highlighting the fundamental right of each patient to be involved in the decision making process concerning his/her health [21, 22]. Moreover SDM contributes to improving healthcare quality and safety and to reducing inappropriate care, based on the principle of solidarity.

As specified in 2007 by Moumjid and colleagues [23], there is no consensual definition of SDM and a systematic review focusing on the definition of SDM is currently under way, conducted by a Danish team (Prospero no. CRD42015019740). Several models have been developed in the past 30 years, as described by Makoul and Clayman in an overview published in 2006 [24]. These authors defined nine essential elements for SDM which are: defining and explaining the problem, presenting the options, discussing the pros and cons (benefits/risks/cost), discussing the patient’s values and preferences, discussing the patient ability/self-efficacy, considering the physician’s knowledge and recommendations, checking/clarifying understanding, making a decision or explicitly postponing it, and organising the follow-up. In 2013, building in particular on Makoul’s model, Elwyn et al. proposed the “talk model”, that summarised existing models [25]. The “talk model” combines three successive and co-dependent steps in the establishment of a relationship between a healthcare professional and a patient: patient information by the healthcare professional about the nature of the problem and the various possible options, patient questioning concerning his/her preferences in terms of goals and treatments, and finally integration of the informed preferences into the decision making process. Despite its simplicity, this model seems to be less frequently cited than Makoul’s model in the articles of the field of SDM.

Beyond the models, many patient decision support or healthcare professional SDM implementation tools have been developed [26]. Most SDM best practices evaluation tools have been developed in English or German [27]. A survey of existing evaluation tools, now representing a research field in their own right, was conducted through two systematic reviews: one by Légaré et al. [28] and the other by Scholl et al. [27], both being reference teams in this field. very few evaluation tools validated in metropolitan French have been published. The only one found to date in the literature is the Decisional Conflict Scale (DCS) that was translated and validated in 2006 [29]. The DCS contains 16 items, grouped into 5 dimensions and has been validated in many languages. It pertains to decisional conflict and evaluates the uncertainty construct, which is not included in the Makoul’s model. To meet the stakes identified by the HAS for the development of SDM best practices, this is a need to develop standardised evaluation tools.

The objective of this work was to update the systematic review (dating back to 2011) of English, French and Spanish language SDM evaluation tools. The perspective is to develop an SDM evaluation tool (de novo creation or trans-cultural adaptation of an existing tool) validated in French, measuring all the SDM elements identified during a doctor-patient encounter, according to the Makoul’s model.

## Methods

The systematic review was registered with PROSPERO (No. CRD42015017101).

### Selection criteria

The following inclusion criteria were used:

1. The full text is available
2. The article is written in English, French or Spanish
3. The article was published between January 1, 2010 and December 31, 2014
4. The main goal of the article was the development and psychometric validation of a measurement tool
5. The measured construct was SDM
6. Psychometric validation data are presented
7. The measurement was performed during a healthcare professional-patient encounter (real or fictitious).

The exclusion criteria were the following:

1. The tool was not specifically dedicated to SDM
2. The tool was specific to a given disease or situation and does not appear readily adaptable to other diseases
3. The study used a tool (whether validated or not) applied to a specific situation, without contributing new psychometric measurements
4. The study was exclusively qualitative.

### Search strategy

The document search strategy was based on the method recommended by the Cochrane Collaboration [30], as described in a guide by the “Institut national d’excellence en santé et en services sociaux du Québec” [31]. It was performed using the PubMed and PsycINFO databases.

For each database, a search query was formalised by combining Medical Subject Heading (MeSH) keywords with the following free text terms: “shared decision making”, “shared medical decision”, “patient participation”, “patient involvement”, questionnaire, “self-report”, “scale”, “tool”, “survey”, “test”, “instrument”, “validation”, “psychometric”, “measure*”, “assess*”. In order to search for the relevant keywords, the search query was based on the strategy used within the two previously identified systematic reviews [27, 28], along with their references.

The search query used in PubMed is described in Appendix.

### Study selection

The study selection process consisted of several successive steps. First, the results obtained from the two databases were grouped into a single file. Duplicate records for a single study were eliminated before starting the selection process. An additional table file shows this in more details (see Additional file 1). The study selection form was tested on fifty randomly drawn studies in order to ascertain selection criterion relevance and discrimination.

We then examined the titles in order to exclude any irrelevant article. At this point, the studies were independently selected by the two observers LM and NB. NB examined all selected titles, while LM examined a 20 % random sample. The calculated coefficient of concordance κ1 was of 0.38. Discussions between the two observers helped to clarify the selection strategy when reading titles and to reach an agreement. A second coefficient of concordance κ2 estimated on a 10 % sample was of 0.63. An additional table file shows this in more details (see Additional file 2). During the third phase, the selected abstracts were examined by NB using the same process. Finally, examination of the full articles by NB enabled a decision of whether to include each article in the systematic review to be made. An additional table file shows this in more details (see Additional file 3). All bibliographic references were managed using the Zotero bibliography management application [32].

### Data extraction

The two observers, LM and NB, collected the data independently. Initial divergences were discussed among the two observers and a consensus was reached. For each of the selected articles corresponding to new tools, the following data were gathered: tool’s name, first author’s name, publication date, point of view adopted (patient, professional, observer), language of validation, type of healthcare environment (hospital, outpatient), number and types of dimensions evaluated, number and labels of items, response scale, recording type (audio or video) where applicable, description of the physician, patient and observer samples (population size, disease or clinical situation), psychometric properties (validity, reliability), and model if it does exist.

As the name of one of the new tools, developed by Légaré et al. [33] could not be clearly determined, we used the following description proposed by the authors as a name in our study: “Dyadic measure of SDM”.

For each of the selected articles corresponding to tools presenting new psychometrics properties, the following data were gathered: tool’s name, name of the tool’s version, first author’s name, publication date, point of view adopted, study type, number of dimensions, number of items, response scale, and psychometric properties (validity, reliability).

### Results analysis strategy

We first studied the characteristics of all included studies. They were classified in three groups: articles already identified in the previous reviews, new psychometric properties of previously identified tools and newly identified tools.

Regarding the latter, we studied their general characteristics, and then focused on the appraiser’s point of view. Finally they were analysed in light of Makoul’s model [24]. We classified each item into one of the nine essential elements described by Makoul and Clayman, in order to determine the extent to which the included tools measured SDM according to this model. Dimension names were not taken into consideration, only the item labels. Each item could match with only one element.

## Results

### Sample

**Fig. 1:**
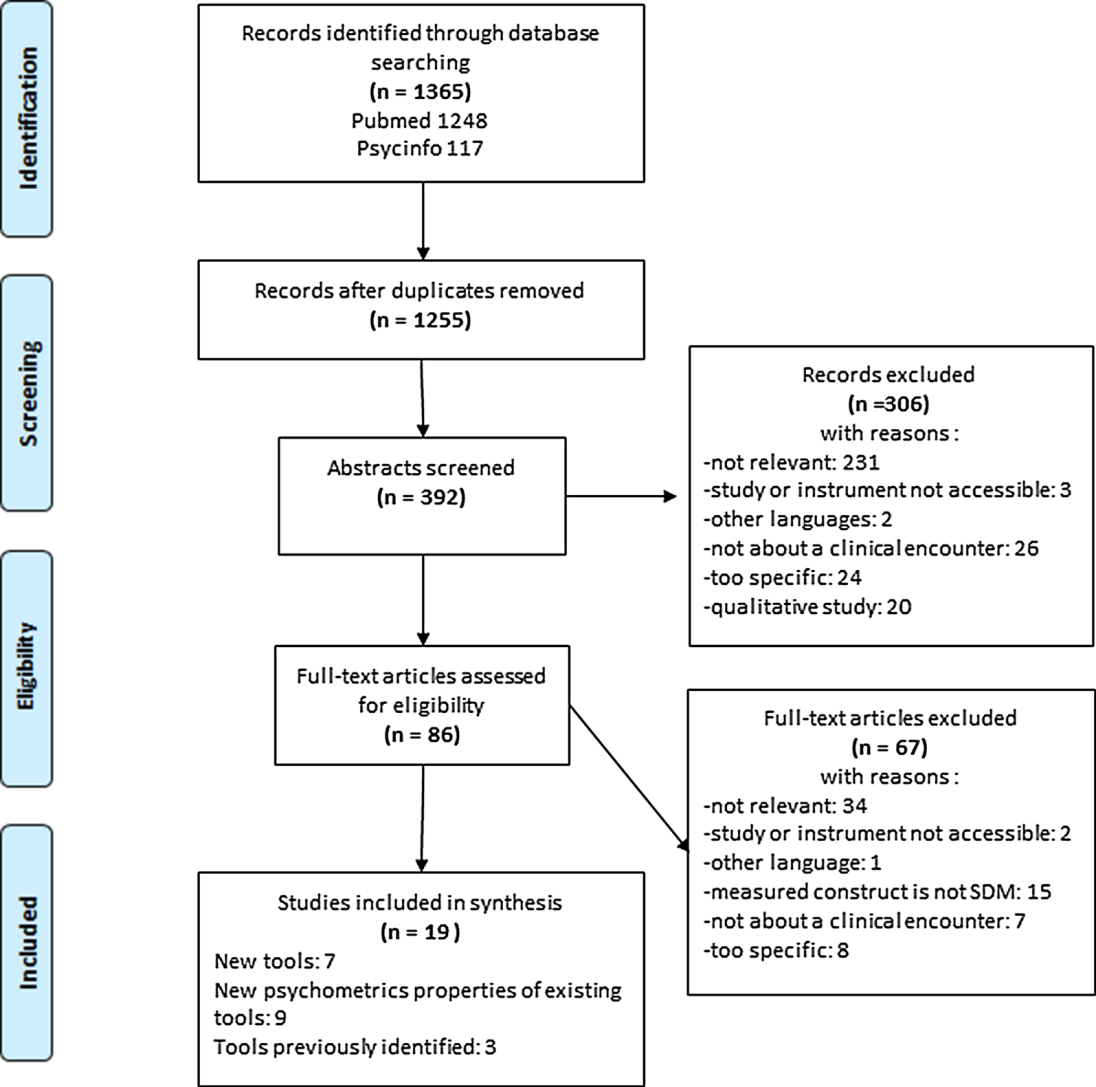
Flowchart of the study

The study selection steps are documented in the flowchart in Fig. 1.

### Characteristics of included studies (n = 19)

Overall, nineteen studies were included in this review. Table 1 gives a general description. Amongst the included articles, seven [33–39] described new tools published since the last systematic review conducted by Scholl et al. [27]. Three articles related to tools that had previously been identified by this review [40–42]. Nine studies presented results of new psychometric properties for tools identified [43–51].

**Table 1:** General characteristics of the included articles (n = 19)

Tool’s name	Version	Author, year of publication	Study type
OPTION scale	Revised-scale of OPTION	Kasper, 2012 [43]	Specific psychometric properties (criterion validity)
	MAPPIN’SDM	Kasper, 2012 [43]	New instrument
	SDM’MASS (meeting its concept’s assumptions)	Geiger, 2012 [37]	New instrument
	German version of the OPTION scale	Hirsch, 2012 [45]	Transcultural validation in german language
	OPTION scale	Nicolai, 2012 [46]	New psychometric properties
	Modified-OPTION scale	Keller, 2013 [47]	Specific psychometric properties (criterion validity, ICC, sensitivity analysis)
SDM-Q-9	SDM-Q-Doc	Schöll, 2012 [34]	New instrument
	SDM-Q-9	Kriston, 2010 [42]	Instrument previously identified in Scholl’s review
	SDM-Q-9	De Las Cuevas, 2014 [50]	Transcultural validation in spanish language
Decisional Conflict Scale	DCS	Kawaguchi, 2013 [44]	Transcultural validation in japanese language
	DCS-LL (low literacy version)	Linder, 2011 [51]	Transcultural validation in low literacy version
IDM (Informed decision making) instrument		Leader, 2012 [36]	New instrument
CICAA-Patient Centered	CICAA-D	Ruiz Moral, 2010 [38]	New instrument
Dyadic measure of SDM		Légaré, 2012 [33]	New instrument
CollaboRATE		Barr, 2014 [39]	New instrument
SURE		Ferron Parayre, 2014 [48]	Specific psychometric properties (criterion validity, sensitivity analysis)
I-PICS		Jonsdottir, 2013 [49]	Transcultural validation in icelandic language
SDM scale		Singh, 2010 [40]	Instrument previously identified in Scholl’s review
DAS-O		Brown, 2011 [41]	Instrument previously identified in Scholl’s review

More than half of the included studies were conducted in Europe [34, 35, 37, 38, 42, 43, 45–47, 49, 50], most of which in Germany. Seven were conducted in North America [33, 36, 39–41, 48, 51] and one in Japan [44]. For most of the studies, the first author was affiliated with a faculty of Medicine, or with a medical organisation (n = 17) [33–43, 45–48, 50, 51]. One of the tools was developed by researchers of a nursing school (n = 1) [49] and one by a team affiliated to a faculty of pharmacy (n = 1) [44].

Concerning the point of view used to evaluate SDM, six studies used the patient’s point of view alone [42, 44, 48–51], eight that of an external observer alone [36, 38–41, 45–47], and one that of a healthcare professional alone [34]. In three studies, the recently published tools focused on the confrontation of points of view by evaluating the patient-professional dyad [33], or the patient-professional-observer triad [35, 37].

### Characteristics of tools presenting new psychometric properties (n = 9)

Nine studies presented results of new psychometric properties of already known tools. Table 2 presents the characteristics of these instruments. Amongst them, five articles presented trans-cultural validations, with analysis of the usual psychometric properties. Four of which involved another language (Japanese [44], german [45], Icelandic [49], Spanish [50] and one a specific type of patient (DCS-Low literacy) [51]. Four other articles presented the results of specific analyses of criterion validity [43, 46–48], two of which also presented sensitivity analyses [47, 48].

**Table 2:** Characteristics of tools presenting new psychometric properties (n = 9)

Name of the tool	Name of the tool’s version	First author, year of publication (reference no)	Study type	Point of view	Number of dimensions and items of SDM	Response-scale	Reliability	Validity
Decisional Conflict Scale	Japanese version of DCS	Kawaguchi, 2013 [44]	Transcultural validation in Japanese language	Patient	5 dimensions; 16 items Uncertainty (3) Informed (3) Values clarity (3) Support (3) Effective decision (4)	5-point scale	Cronbach’s alpha = 0.84–0.96 ICC = 0.63–0.93	Content validity: Yes Face validity: No Construct validity: Yes Convergent/discriminant validity: Yes
OPTION scale	German version of the OPTION scale	Hirsch, 2012 [45]	Transcultural validation in German language	Observer	1 dimension; 12 items	5-point scale	Cronbach’s alpha = 0.90 ICC = 0.68–0.82	Face validity: No Construct validity: Yes Convergent/discriminant validity: No Criterion validity: Yes
PICS	I-PICS	Jonsdottir, 2013 [49]	Transcultural validation in Icelandic language	Patient	4 dimensions; 20 items Health care professional (HCP)information (6) Patient information (5) Patient participation in decision making (4) HCP facilitation (5)	5-point Likert scale	Cronbach’s alpha = 0.86	Face validity: No Construct validity: Yes Convergent/discriminant validity: No Criterion validity: Yes
SDM-Q-9	Spanish version of SDM-Q-9	De Las Cuevas, 2014 [50]	Transcultural validation in Spanish language	Patient	1 dimension; 9 items	6-point scale	Cronbach’s alpha = 0.89 ICC = 0.52–0.82 (item 1: 0.27)	Face validity: Yes Construct validity: Yes Convergent/discriminant validity: Yes
Decisional conflict Scale	DCS-LL (low literacy version of decisional conflict scale)	Linder, 2011 [51]	Transcultural validation in low literacy version	Patient	4 dimensions; 10 items Uncertainty (2) Informed (3) Values clarity (2) Supported (3)	3-point scale	Cronbach’s alpha = 0.80 ICC = 0.43–0.87	Content validity: No Face validity: No Construct validity: Yes Convergent/discriminant validity: Yes
OPTION scale	Revised-scale of OPTION scale	Kasper, 2012 [43]	Specific psychometric properties (criterion validity)	Patient, professional	1 dimension; 12 items	5-point scale	Cronbach’s alpha = Yes	Criterion validity: Yes
OPTION scale	Modified-OPTION scale	Keller, 2013 [47]	Specific psychometric properties (criterion validity, sensitivity analysis)	Observer	1 dimension; 12 items	5-point scale	Cronbach’s alpha = 0.90 ICC = 0.43–0.82	Criterion validity: Yes Sensitivity analyses: Yes
SURE	SURE	Ferron Parayre, 2014 [48]	Specific psychometric properties (criterion validity, sensitivity analysis)	Patient	1 dimension; 4 items	5-point scale	Cronbach’s alpha = 0.70	Content validity: Yes Face validity: No Construct validity: No Convergent/discriminant validity: No Criterion validity: Yes Sensitivity analyses: Yes
OPTION scale	OPTION scale	Nicolai, 2012 [46]	New psychometric properties	Observer	1 dimension; 12 items	5-point scale	Cronbach’s alpha = Yes	Criterion validity: Yes Convergent/discriminant validity: unknown

Amongst these nine studies, four described properties of the OPTION scale [43, 45–47], two those of the decisional conflict scale [44, 51], one those of the PICS [49], one those of the SDM-Q-9 [50] and one those of the SURE tool [48].

Most of these instruments were unidimensional (n = 6) [43, 45–48, 50]. Three tools were multidimensional [44, 49, 51]. The three multidimensional tools comprised 4–5 dimensions. The mean number of items per tool was of 12 (4–20). The response scale used was mainly of the Likert-5 type (n = 7) [43–49]. The other instruments used a 3-point response scale [51], and a 6-point response scale [50].

Concerning reliability, the Cronbach’s alpha coefficient was calculated in all nine studies and the interclass correlation coefficient (ICC) was calculated for five studies [44, 45, 47, 50, 51]. Regarding validity, five of the nine articles presented a construct validity [44, 45, 49–51], one a face validity [50], and three a convergent/discriminating validity [44, 50, 51].

Amongst the nine tools presenting new psychometric properties, one evaluated two points of view simultaneously: that of the patient and healthcare professional (dyad) [43]. Five instruments evaluated SDM according to the patient point of view only [44, 48–51]. Three tools assessed SDM from the external observer’s perspective alone [45–47]. None of the instruments evaluated the healthcare professional’s point of view alone.

### Characteristics of newly included tools (n = 7)

#### General description

Among the seven newly identified tools, two had in fact previously been identified by Scholl et al. work [27], though at the time they were “work in progress”, i.e. with no published psychometric data [33, 34]. As the presence of psychometric data was one of our inclusion criteria, we made the choice to include them in this subgroup. Table 3 presents the characteristics of the seven newly identified instruments: SDM-Q-Doc [34], Mappin’SDM [35], informed decision making instrument [36], SDM’Mass [37], CICAA-Decision [38], Dyadic measure of SDM [33], and collaborate [39].

**Table 3:** Characteristics of the newly developed instruments (n=7)

Name of the tool	First author, year of publication (reference no)	Point of view	Language of validation	Inpatients and/or outpatients	Number of dimensions and items of SDM	Response-scale	Methodology	Sample	Reliability	Validity	Model and items generation process
SDM-Q-Doc	Scholl I, 2012 [34]	Professional	German	Inpatients and outpatients	1 dimension; 9 items	6-point scale	Real consultations	PHYSICIANS: N = 29/General practitioners 51.7 %, orthopaedists 13.8 %, psychiatrists 13.8 %, diabetologists 20.7 % PATIENTS: N = 324/external patients of primary and secondary care with a chronic back pain, type 2 diabetes, or depression	Cronbach’s alpha = 0.88 ICC = 0.35–0.76	Content validity: unknown Face validity: Yes Construct validity: Yes Convergent/Discriminant validity: Yes	Pre-existent tool (SDM-Q, 2006) and theory-driven: Nine practical steps of the SDM process defined by the authors: disclosure that a decision needs to be made, formulation of equality of partners, presentation of treatment options, informing on the benefits and risks of the options, investigation of patient’s understanding and expectations, identification of both parties’ preferences, negotiation, reaching a shared decision, arrangement of follow-up
Mappin’SDM	Kasper J, 2012 [35]	Patient, professional, observer	German	Inpatients and outpatients	15 items	5-point Likert scale	Real consultations	(video recording) 40 consultations physician-patient videorecorded (Hambourg)/average duration 19.5 min (2.5–51 min)/average duration of decision sequence 15 min (2.5 to 38.8 min)// PHYSICIANS: N = 10/neurologists and internal medicine 40 %, dentists 30 %, general practitioners 30 % PATIENTS: N = 40/55 % of men	Cronbach’s alpha = 0.91–0.94 ICC = Yes	Face validity: unknown Construct validity: No Convergent/Discriminant validity: Yes	Theory-driven (created by the authors): three perspectives, two constructs, three units and seven focus result in a set of three tools, each of them measuring the same fifteen items
Informed decision making instrument	Leader A, 2012 [36]	Observer	English	Inpatients and outpatients	3 dimensions 9 items Patient empowerment (1) Information sharing (4) Active engagement in preference clarification (4)	2 point-scale	Real consultations audio recorded N = 146	PHYSICIANS: N = 22 PATIENTS: N = 146 men candidates screening of prostate cancer	Cronbach’s alpha = 0.80 ICC = 0.81	Construct validity: use of an existing instrument	Theory-driven: Nine elements of Informed Decision Making developed by Dr Braddock [56]: the patient’s role in decision making, the impact of the decision on the patient’s daily life (context of decision), the nature of the decision or clinical issue, alternatives, pros and cons surrounding alternatives, uncertainties regarding alternatives, physician assessment of the patient’s understanding, physician assessment of the patient’s desire for input from trusted others, physician solicitation and exploration of the patient’s preference
SDM’Mass (SDM Meeting its concept’s ASSumptions)	Geiger F, 2012 [37]	Patient, professional, observer	German	Inpatients and outpatients	15 items	5-point Likert scales	Real consultations video recorded N = 40	Average duration 20 min (2.5–51 min; SD = 11)/Average duration of decision sequence 15 min (3 to 39 min; SD = 8)// PHYSICIANS: N = 10/neurologists and internal medicine 40 %, dentists 30 %, general practitioners 30 % PATIENTS: N = 40/55 % of men	Cronbach’s alpha = 0.94 ICC = 0.74–0.87	Face validity: unknown Construct validity: No Convergent/discriminant validity: No	Theory-driven (created by the authors): Three perspectives, two constructs, three units and seven focus result in a set of three tools, each of them measuring the same fifteen items
CICAA-Decision	Ruiz Moral R, 2010 [38]	Observer (on professional ‘s behaviour)	Spanish	Outpatient	3 dimensions; 17 items Identifying and understanding problems (2) Reach an agreement and help to act (11) Decisions with options (4)	3-point scale	Real and fictional consultations N = 111 real patients and N = 50 simulated patients	(Video recording) 161 consultations videorecorded: 61 consultations between “professional” and patient with chronic disease (diabetes et chronic pain) + 100 consultations between last year’s residents and new patients (50) or simulated patients (50)// Then selection of 32 consultations (20 % where item 25 is positive = a bit of participation is detected)/average duration = 11.3 min (SD = 5.6; IC95 = 9.2-13.3)	Cronbach’s alpha = 0.60–0.51 (1st and 2nd encounter) ICC global = 0.96	Content validity: Yes Face validity: Yes Construct validity: Yes Convergent/discriminant validity: No	Pre-existent tool (CICAA-CP) and literature research (review of pre-existing conceptual frameworks)
Dyadic measure of SDM	Légaré F, 2012 [33]	Patient, professional	English and Quebec French (patient and doctors recruited in Ontario and Québec)	Outpatient	7dimensions; 30 items Information giving (9) Values clarification (3) Doctor recommendations (5) Self-efficacy (3) Feeling uninformed (3) Information verifying (4) Uncertainty (3)	5-point scale and 10-point scale (different subscales)	Real consultations	PHYSICIANS: N = 272/english language N = 109, french N = 163 PATIENTS: N = 269/english language N = 108, french language N = 161/69 % of women/average age 49 (SD = 18) Complete DYAD: N = 259 (after consultation)	Cronbach’s alpha = 0.90 ICC = 0.43–0.82	Face validity: No Construct validity: Yes Convergent/discriminant validity: No Criterion validity: correlation with OPTION scale: Yes sensitivity analysis (AUC and ROC) Agreement across raters: ICC	Pre-existent tools and theory-driven (created by the authors): Based on Makoul and Clayman model, creation of a dyadic model that conceptualized the interpersonal and interdependent elements of the relationship between physicians and patients Then identification of instruments tested on both physicians and patients. Finally cross-cultural adaptation of the identified subscales that mapped the essential elements of SDM included in their dyadic model
Collaborate	Barr PJ, 2014 [39]	Observer (citizen; has to put in patient’s place)	English	Inappropriate	3 dimensions; 3 items Explanation of the health issue Elicitation of patient preferences Integration of patient preferences	2 versions: 5-point scale, and 10-point scale	Fictional consultations N = 6 simulated videos of encounters physician-patient	OBSERVERS: N = 1341 in study 1/N = 251 in study 2 (1–2 weeks after first answer)/On N = 1341: 46 % of men/47 % of 18-44 years, 33 % of 45-64 years, 20 % of 65 years and more/public in general population acting as the observer. Recruited by the 2010 US Census/representative sample of general population of USA	Cronbach’s alpha: No ICC = 0.76–0.90	Convergent/Discriminant validity: Yes Criterion validity: Yes Sensitivity to change: Yes	Theory-driven (created by the authors): the “talk model” developed by authors in a previous study [25]

In most cases, the validation language was German (n = 3) [34, 35, 37], followed by English (n = 2) [36, 39] and Spanish (n = 1) [38]. One study presented a tool with a dual French–English version [33]. The first author was always affiliated with a faculty of Medicine or a medical organisation. Patient sample sizes ranged from 40 to 324, with a mean of 163 patients (median = 153.5). The healthcare professionals involved in the studies were explicitly physicians in six of the studies. One study used the terms “professionals” and “residents” [38]. The number of physicians ranged between 10 and 272, with a mean of 67 (median = 22). In four studies, the patient sample consisted of both inpatients and outpatients [34–37] whereas two studies focused on outpatients alone [33, 38].

Most of the newly included tools were multidimensional (n = 4). The four multidimensional tools comprised a median of 3 dimensions (3–7). Only one tool was unidimensional [34]. Two tools described a total of fifteen items, without specifying their grouping into dimensions [35, 37]. The mean number of items per tool was of 14 (3–30). The response scale used was mainly of the Likert-5 type (n = 4) [33, 35, 37, 39] of which two tools used a 5- or 10-point response scale depending on the sub-scales [33] or versions [39]. The other tools used a 2-point [36], 3-point [38], or 6-point [34] response scale.

Regarding reliability, the Cronbach’s alpha coefficient was calculated for six studies [33–38], while the interclass correlation coefficient (ICC) was calculated in all seven studies. As for validity, three of the seven articles reported a construct validity [33, 34, 38], two a face validity [34, 38], and three a convergent/discriminating validity [34, 35, 39]. The criterion validity was presented in two articles [33, 39], and content validity in one article [38].

#### Evaluated point of view

Amongst the seven newly included tools, two evaluated all three points of view simultaneously (triad) [35, 37], while one evaluated that of the patient and healthcare professional (dyad) [33]. Three instruments evaluated SDM according to the external observer’s point of view only [36, 38, 39]. Amongst them, one study used a sample of patients taken from the general population who were asked to place themselves in the patient’s place, thus assuming a role of external observer [39]. One tool evaluated SDM from the healthcare professional’s perspective alone [34]. None of the instruments explored the patient’s point of view alone.

Amongst the tools evaluating the perspective of an external observer (alone, or jointly with other points of view), the evaluation always involved recordings. Most of these were videos (n = 4) [35, 37–39], while one study used audio recordings [36]. Most of the tools were based on real consultations (n = 5). One tool used only fictitious consultations [39], while another was based on encounters with both real and fictitious patients [38].

#### Model and comparison to Makoul and Clayman’s integrative model of SDM

The design of six out of seven tools was based on a model:

- Four authors had created the model themselves [33, 35, 37, 39].
- Two used, in addition to the theory, one or several pre-existent tools [33, 34].

The development of one of the seven tools was not based on a precise model, but rather on a pre-existent tool combined with a literature review [38].

**Fig. 2:**
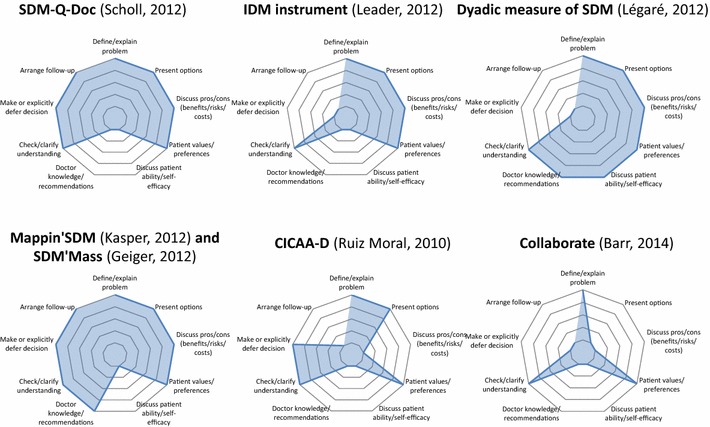
Distribution diagrams of the essential elements of SDM according to Makoul in newly developed tools

The nine elements defining SDM according to Makoul [24] were inconsistently met in the seven newly identified tools. The distribution is presented in Fig. 2. Three elements were found in all seven tools: “define/explain problem”, “patient values/preferences”, and “check/clarify understanding”. The element “present options” was found in every tool except the collaborate tool.

Still according on the Makoul’s model, Mappin’SDM [35] and SDM’Mass [37] were the tools that evaluated the greatest number of essential SDM elements: only one “discuss patient ability/self-efficacy” was not explored by these two tools. It is noteworthy that the collaborate tool [39] contained only three of the essential elements described by Makoul and Clayman.

## Discussion

This literature review covering the 2010–2014 period identified a total of nineteen publications since the last systematic review by Scholl et al. [27]. Seven articles presented psychometric validations of new tools: SDM-Q-Doc, Mappin’SDM, SDM’Mass, Informed Decision Making Instrument, CICAA-Decision, Dyadic measure of SDM, and Collaborate. Our results partially overlap those of Scholl et al.: indeed, three studies had already been identified in the previous review and nine studies covered new psychometric validations of previously identified tools, including five trans-cultural validations. It shows the growing effort of SDM research in various countries including France. Indeed, the interest paid to the field of SDM is shared internationally both by clinicians and researchers [2], as shown by the multiple languages of the identified evaluation tools.

We have found that there was a recent breakthrough in the field of SDM research with the multiplicity of appraiser points of view. The old approach involving a single appraiser is progressively tending towards a multiple, dyadic [27] or even triadic approach, in accordance to recent recommendations [52]. In the field of clinical practice, SDM evaluation is performed by the healthcare professional or the patient. This approach is helpful to provide an image of the interdependence between the members of this dyad during the decision making process [53, 54]. In the field of research, a third player—the external observer—has emerged, most often using video or audio recordings.

Regarding the different existing models subtending SDM, it is not unequivocal. Several models coexist [24, 25, 52, 55]. The Makoul’s model [24] based on a systematic and broadly acknowledged review in the field of SDM research was published in 2006. We chose this model as a basis for comparison between SDM evaluation tools for three main reasons. First, it is one of the most frequently cited models in the articles of the field. Second, it is based on a systematic review and is therefore a synthesis of other existing models. Third, compared to the “talk model”, also based on a literature review, Makoul’s model describes more elements, and thus provides a broader description of the SDM process. Although some aspects of this process might still be overlooked by Makoul’s model, it seemed to be the most relevant model for standardized comparisons of the seven newly identified tools.

The originality of our work partly lies in these standardized comparisons. They showed notably that none of the newly identified tool covered the whole nine essential elements of Makoul’s model. However, three elements (“define/explain problem”, “patient values/preferences”, and “check/clarify understanding”) were identified in each of the tools, and might therefore constitute necessary—and maybe sufficient—dimensions of the SDM construct. Conversely, the element “discuss patient ability/self-efficacy” was missing in every tool except for the Dyadic measure of SDM [33] and might therefore be more difficult to measure. It is interesting to note that the collaborate tool [39], which contains only three essential elements of Makoul’s model, was specifically designed for a clinical use. For practical reasons, indeed, it might be reasonable to limit the number of elements assessed by clinical tools, as opposed to research tools, which could be more comprehensive.

Five tools were particularly interesting, because of the multiplicity of SDM dimensions and/or points of view that they explored. Mappin’SDM [35] and SDM’Mass [37] cover eight SDM elements according to Makoul’s model and evaluate all three points of view; the Dyadic measure of SDM [33] covers seven dimensions and measures the points of view of both the patient and the healthcare professional; SDM-Q-Doc [34] covers seven dimensions of SDM, and collaborate [39], covers the three dimensions defined by the “talk model” from which it is derived.

There are several limitations in our study that could have prevented relevant studies to be included in our review. For instance, the grey literature was not examined, experts in the SDM field were not contacted and their internet pages and social networks were not consulted. This selection strategy probably resulted in a lack of relevant sources. The authors take into account the fact that it might have led to a selection bias. The articles were obtained by searching solely from the PubMed and PsycINFO databases. However, this latter limitation might not be that important. Indeed, after having tested the search equations on five databases (PubMed, PsycINFO, BDSP, Cairn and Pascal), we decided to use the first two only due to the lack of relevant results in the others. We have also limited our review to articles in French, English and Spanish. Therefore, tools presented in article written in another language would be missing from our review. This language bias in fact occurred in the two previous reviews [27, 28]. Indeed, the inclusion of articles in Spanish allowed us to identify one tool that was missed by these studies [38]. Moreover, while the previous literature reviews focused on several dimensions of the patient-carer relationship, including SDM and communication [27, 28], we deliberately chose to exclude tools that were not exclusively dedicated to SDM, for reasons of clarity. Some interesting tools might have been missed due to this selection process.

Finally, the double independent observers strategy was adopted for a fraction of the titles read only, not for reading the abstracts and full-text articles.

In spite of these limitations, our results are consistent with the already published systematic reviews.

## Conclusions

The stakes of SDM are major, whether it be in regard to patient participation improvement, healthcare quality or safety, or inappropriate use of healthcare reduction. Evaluating the quality of SDM between patient and professional for the needs of improvement and training requires the use of subjective measurement tools with good psychometric properties. This review enabled us to identify several interesting tools. The next step of our project will be to develop and validate a French language instrument by operating a trans-cultural adaptation of one or several identified tools, since it could allow both to develop an SDM evaluation tool in French, and to make a cultural comparison in the field.

## Appendix: search query on PubMed

- Filter: publication date from January 1, 2010 to December 31, 2014
- ((“shared decision making”[Title] OR “shared decision-making”[Title] OR “shared decision making”[TW] OR “shared medical decision”[Title] OR “patient participation”[Title] OR “patient participation”[MH] OR “patient participation”[TW] OR “patient involvement”[Title])) AND (Questionnaire[Title] OR Questionnaires[Title] OR “self report”[Title] OR “self reports”[Title] OR scale[Title] OR scales[Title] OR tool[Title] OR tools[Title] OR measur*[Title] OR assess*[Title] OR survey[Title] OR surveys[Title] OR test[Title] OR tests[Title] OR instrument[Title] OR instruments[Title] OR questionnaires[MH] OR “self report”[MH] OR validation[Title] OR psychometrics[MH] OR Psychometric[Title] OR psychometrics[Title])

## Additional files

10.1186/s13104-016-2164-6 List of records identified trough database searching (n=1365) and after removed (n=1255). Description of data: title, author 1 to 5 (first and last name), name of journal, date of publication, abstract, tag 1 to 4, selection’s status (included or duplicate).
10.1186/s13104-016-2164-6 List of records of 20% and 10 % random sample selected by two independent observers and calcul of coefficients of concordance κ1 and κ2. Description of data: Sheet 1: title, selection’s status NB reading 1 20 % titles (excluded/included), selection’s status LM reading 1 20 % titles (excluded/included), comparison between 2 observers, kappa 1. Sheet 2: title, selection’s status NB reading 2 10 % titles (excluded/included), selection’s status LM reading 2 10 % titles (excluded/included), comparison between 2 observers, selection’s status adjustment after discussions (excluded/included), kappa 2.
10.1186/s13104-016-2164-6 List of full-text articles assessed for eligibility (n=86) and full-text articles excluded (n=67). Description of data: title, first author (last name), name of journal, date of publication, abstract, selection’s status (included or excluded), not relevant, study or instrument not accessible, other language, measured construct is not SDM, not about a clinical encounter, too specific.

## Abbreviations

DCS: decisional conflict scale
HAS: Haute Autorité de Santé
ICC: interclass correlation coefficient
SDM: shared decision making
