# Supplementary material for: Evaluating the quality of shared decision making during the patient-carer encounter: a systematic review of tools
Source: BMC Res Notes. 2016 Aug 2;9:382. doi: 10.1186/s13104-016-2164-6 (PMC4971727; doi:10.1186/s13104-016-2164-6)
Supplement: Supplementary file 1 — 10.1186/s13104-016-2164-6 List of records identified trough database searching (n=1365) and after removed (n=1255). Description of data: title, author 1 to 5 (first and last name), name of journal, date of publication, abstract, tag 1 to 4, selection’s status (included or duplicate). [file 13104_2016_2164_MOESM1_ESM.docx]

| **Author3_First** | **Author3_Last** | **Author4_First** | **Author4_Last** | **Author5_First** | **Author5_Last** | **Name of journal** | **Date of publication** | **Abstract** | **TAG_1** | **TAG_2** | **TAG_3** | **TAG_4** | **Selection's status (Included or Duplicate)** |
| --- | --- | --- | --- | --- | --- | --- | --- | --- | --- | --- | --- | --- | --- |
| Sarah | Collins | Tineke A. | Abma | John | Kirwan | The Patient | 2013-00-00 2013 | BACKGROUND: Patients are incidentally involved in scientific conferences as collaborating partners. Little is known about how they engage with researchers. OBJECTIVE: The purpose of this study was to explore the expectations and experiences of new patients to better understand the specific features of collaborative research during conferences in its complexity. STUDY DESIGN: After a thematic literature review, we conducted fourteen interviews with eight delegates: four men and four women with three rheumatic diseases and representing five countries. They participated for the first time in the biannual conference on Outcome Measures in Rheumatology (OMERACT) in 2010. Data were subjected to a thematic content analysis. RESULTS: Before the conference, patient participants had felt privileged to be invited but felt insufficiently prepared and uncertain about what was expected from their participation. They had anticipated a learning experience and had hoped to be able to make a contribution. Most experienced the conference program as physically and mentally challenging, partially due to poor moderation or lack of individual support. They doubted their input had been beneficial. After the conference these patients also described their participation as having been a valuable, meaningful, and learning experience. Although they presumed that they had not been very productive, they expected their contribution would be more effective at future conferences. CONCLUSIONS: Patient delegates attending a scientific conference need clear information about their role prior to the event. Personalized support and a facilitative moderation style during sessions are advantageous for maximizing valuable contributions. Participation leads to personal learning curves and various benefits. | Male | Humans | Patient Participation | Patient Satisfaction | OK |
| James M. | Mason | Jerry J. | Murphy | Ahmet | Fuat | BMC geriatrics | 2013-00-00 2013 | BACKGROUND: Older people in care-facilities may be less likely to access gold standard diagnosis and treatment for heart failure (HF) than non residents; little is understood about the factors that influence this variability. This study aimed to examine the experiences and expectations of clinicians, care-facility staff and residents in interpreting suspected symptoms of HF and deciding whether and how to intervene. METHODS: This was a nested qualitative study using in-depth interviews with older residents with a diagnosis of heart failure (n=17), care-facility staff (n=8), HF nurses (n=3) and general practitioners (n=5). RESULTS: Participants identified a lack of clear lines of responsibility in providing HF care in care-facilities. Many clinical staff expressed negative assumptions about the acceptability and utility of interventions, and inappropriately moderated residents' access to HF diagnosis and treatment. Care-facility staff and residents welcomed intervention but experienced a lack of opportunity for dialogue about the balance of risks and benefits. Most residents wanted to be involved in healthcare decisions but physical, social and organisational barriers precluded this. An onsite HF service offered a potential solution and proved to be acceptable to residents and care-facility staff. CONCLUSIONS: HF diagnosis and management is of variable quality in long-term care. Conflicting expectations and a lack of co-ordinated responsibility for care, contribute to a culture of benign neglect that excludes the wishes and needs of residents. A greater focus on rights, responsibilities and co-ordination may improve healthcare quality for older people in care. TRIAL REGISTRATION ISRCTN: ISRCTN19781227. | Male | Humans | Patient Participation | Questionnaires | OK |
| A. | Daley | R. | Walisser | R. | Halpenny | Vaccine | 2011-01-10 Jan 10, 2011 | The Step Study phase IIb HIV-1 vaccine trial was terminated early due to futility; subsequent analyses revealed increased susceptibility to HIV infection among a subset of test vaccine recipients. We conducted a mixed methods investigation, including a brief, self-administered baseline questionnaire and in-depth, semi-structured, 1-h interviews after unblinding, to explore experiences and perspectives among trial participants and key informants. Interviews were digitally recorded, transcribed, and analyzed using NVivo and thematic techniques. Forty-eight trial participants (46 gay/bisexual men) completed baseline surveys; 15 (14 gay/bisexual men) engaged in post-trial interviews. Participants indicated surprise and disappointment about the early trial termination and unexpected risks. Some articulated understanding the uncertainties of clinical trials, steadfast support and willingness to participate in the future; others reported greater risks than they deemed acceptable and unlikelihood of volunteering again. A few indicated mistrust of trial sponsors and ethics. Participants' most profound criticism was not about unexpected results, but perceived delays in unblinding and gaps in post-trial dissemination of information. Future HIV vaccine trials may benefit from increased emphasis on: (1) communication mechanisms among participants, investigators and trial sponsors, and (2) post-trial dissemination of information and psychosocial support. | Male | Humans | Patient Participation | Questionnaires | OK |
|  |  |  |  |  |  | Gesundheitswesen (Bundesverband Der Ärzte Des Öffentlichen Gesundheitsdienstes (Germany)) | 2011-01-00 Jan 2011 | The great significance of the concept of participation in health care policy is in contrast with the comparatively low resonance that the participation construct ("Teilhabe" in German) has found in scientific circles. It can be argued that this is due in part to the insufficient specification of the term in the ICF ("International Classification of Functioning, Disability and Health") and the lack of suitable measuring instruments. This article deals with the question of what approaches to defining participation currently exist and what methods are conceivable for facilitating the integration of the construct in health services research. Based on a review of German and international literature on participation, the construct is differentiated from related concepts such as "social capital," "social network," "social support," and "community integration". It is recommended that participation should be understood as "social role participation". The possibility this entails of referring to existing research traditions and available studies leads to the necessity that a comprehensive measurement of participation should include five dimensions of this construct: performance, capability, importance, context factors, and satisfaction. A review of the available instruments for measuring participation shows that most of them cover the ICF domains that are important in this context to a sufficient extent. However, there are the following areas for improvement: a) No measuring instrument includes all five relevant dimensions of participation, b) None of the instruments take non-health-related obstacles to participation (context factors) into consideration, c) The possibility of a version with parallel content for proxy assessment is rarely used, d) The published methods available to German-speaking users cover participation only globally or are older and do not incorporate experience with the ICF. In view of the significance of the participation construct in the German health care system, studies on new or ongoing developments of assessment instruments that meet these challenges would be welcomed. | Humans | Patient Participation | health policy | Clinical Trials as Topic | OK |
| Kathleen | Ardis |  |  |  |  | Journal of Rehabilitation Medicine | 2011-10-00 Oct 2011 | OBJECTIVE: To explore the perceptions of being discharged home following lower limb orthopaedic surgery in older adults. METHODS: Qualitative interviews with 11 patients over the age of 65 years were conducted between 6 and 12 weeks afterdischarge home and analysed using interpretative phenomenological analysis (IPA). RESULTS: Three themes were identified from analysis of the participants’ experiences of rehabilitation during the 6–12 weeks following discharge: (i) lack of a shared decision on when to go home; (ii) dependent on family to go home and to feel confident there; and (iii) trial and error rehabilitation.A further theme: a paternalistic medical model was also identified in participants’ experiences of contact with health professionals. CONCLUSION: Participants had positive experiences of being discharged home from hospital. However, few participants played an active role in their discharge, all required the support of family to go home, and many were left unsure of how and when to return to usual activities. A paternalistic medical model was apparent. Family support, not without costs,was integral to discharge and rehabilitation at home. | Male | Humans | Patient Participation | Patient Satisfaction | OK |
| Jordan E. | Roberts | Joseph T. | Cooke | M. Carrington | Reid | Journal of Palliative Medicine | 2013-05-00 May 2013 | BACKGROUND: Effective communication is essential for shared decision making with families of critically ill patients in the intensive care unit (ICU), yet there is limited evidence on effective strategies to teach these skills. OBJECTIVE: The study's objective was to pilot test an educational intervention to teach internal medicine interns skills in discussing goals of care and treatment decisions with families of critically ill patients using the shared decision making framework. DESIGN: The intervention consisted of a PowerPoint online module followed by a four-hour workshop implemented at a retreat for medicine interns training at an urban, academic medical center. MEASUREMENTS: Participants (N=33) completed post-intervention questionnaires that included self-assessed skills learned, an open-ended question on the most important learning points from the workshop, and retrospective pre- and post-workshop comfort level with ICU communication skills. Participants rated their satisfaction with the workshop. RESULTS: Twenty-nine interns (88%) completed the questionnaires. Important self-assessed communication skills learned reflect key components of shared decision making, which include assessing the family's understanding of the patient's condition (endorsed by 100%) and obtaining an understanding of the patient/family's perspectives, values, and goals (100%). Interns reported significant improvement in their comfort level with ICU communication skills (pre 3.26, post 3.73 on a five-point scale, p=0.004). Overall satisfaction with the intervention was high (mean 4.45 on a five-point scale). CONCLUSIONS: The findings suggest that a brief intervention designed to teach residents communication skills in conducting goals of care and treatment discussions in the ICU is feasible and can improve their comfort level with these conversations. | Male | Decision Making | Humans | Questionnaires | OK |
| Sadaaki | Fukui | Mark C. | Holter | Linda | Collins | Psychiatric Services | 2012-08-01 août 1, 2012 | Objective: Shared decision making is widely recognized to facilitate effective health care. The purpose of this study was to assess the applicability and usefulness of a scale to measure the presence and extent of shared decision making in clinical decisions in psychiatric practice. Methods: A coding scheme assessing shared decision making in general medical settings was adapted to mental health settings, and a manual for using the scheme was created. Trained raters used the adapted scale to analyze 170 audio-recordings of medication check-up visits with either psychiatrists or nurse practitioners. The scale assessed the level of shared decision making based on the presence of nine specific elements. Interrater reliability was examined, and the frequency with which elements of shared decision making were observed was documented. The association between visit length and extent of shared decision making was also examined. Results: Interrater reliability among three raters on a subset of 20 recordings ranged from 67% to 100% agreement for the presence of each of the nine elements of shared decision making and 100% for the agreement between provider and consumer on decisions made. Of the 170 sessions, 128 (75%) included a clinical decision. Just over half of the decisions (53%) met minimum criteria for shared decision making. Shared decision making was not related to visit length after the analysis controlled for the complexity of the decision. Conclusions: The rating scale appears to reliably assess shared decision making in psychiatric practice and could be helpful for future research, training, and implementation efforts. (PsycINFO Database Record (c) 2012 APA, all rights reserved). (journal abstract) | Decision Making | clinical practice | Health Care Services | coding system | Doublon |
| Sadaaki | Fukui | Mark C. | Holter | Linda | Collins | Psychiatric Services (Washington, D.C.) | 2012-08-00 Aug 2012 | OBJECTIVE: Shared decision making is widely recognized to facilitate effective health care. The purpose of this study was to assess the applicability and usefulness of a scale to measure the presence and extent of shared decision making in clinical decisions in psychiatric practice. METHODS: A coding scheme assessing shared decision making in general medical settings was adapted to mental health settings, and a manual for using the scheme was created. Trained raters used the adapted scale to analyze 170 audio-recordings of medication check-up visits with either psychiatrists or nurse practitioners. The scale assessed the level of shared decision making based on the presence of nine specific elements. Interrater reliability was examined, and the frequency with which elements of shared decision making were observed was documented. The association between visit length and extent of shared decision making was also examined. RESULTS: Interrater reliability among three raters on a subset of 20 recordings ranged from 67% to 100% agreement for the presence of each of the nine elements of shared decision making and 100% for the agreement between provider and consumer on decisions made. Of the 170 sessions, 128 (75%) included a clinical decision. Just over half of the decisions (53%) met minimum criteria for shared decision making. Shared decision making was not related to visit length after the analysis controlled for the complexity of the decision. CONCLUSIONS: The rating scale appears to reliably assess shared decision making in psychiatric practice and could be helpful for future research, training, and implementation efforts. | Male | Decision Making | Humans | Patient Participation | OK |
| Benedict C. | Nwomeh |  |  |  |  | Patient Education and Counseling | 2013-10-00 Oct 2013 | OBJECTIVE: It was hypothesized that parents exposed to a communication skills intervention would participate (e.g., ask questions, express concerns) in a pre-surgical consultation more than parents in the control group. METHODS: Sixty-five parents of prospective pediatric surgery patients were randomly assigned to either a communication skills intervention (booklet mailed prior to the consultation) or control group. Only initial consultation parents were included, no follow-ups. Audio-recordings of the medical consultations were obtained and the transcripts analyzed utilizing a content-coding system to determine patient participation. Also, parents were given a health literacy test (s-TOFHLA) and a post-interview questionnaire to gather demographic data. RESULTS: Intervention parents participated overall significantly more than control parents. Follow-up analyses revealed that intervention parents asked significantly more questions, and engaged in significantly more information verifying and expressing of concerns. There were no significant differences for parents' assertive statements or information provision. Other significant predictors of parents' participation were consultation length and parents' income. CONCLUSION: The intervention tested in this study promoted parents' participation in a pre-surgical consultation. As such, it has the potential to improve information exchange between parents and physicians with positive implications for informed consent. PRACTICE IMPLICATIONS: Providing parents with communication guidelines prior to a surgical consultation may improve physician-patient communication. | Male | Humans | Questionnaires | Female | OK |
| Michelle A. | Mathiason | Kristen A. | Marcou | Gayle S. | Jago | The Breast Journal | 2010-10-00 2010 Sep-Oct | Measurement of quality indicators and peer comparison has been demonstrated to improve quality of care. The goal of this study was to determine whether a community breast center, in collaboration with the National Consortium of Breast Centers (NCBC), could voluntarily audit the quality of breast cancer care, confidentially transmit quality information to the NCBC, and receive peer performance comparisons. Quality indicator metrics from consecutive breast cancer patients undergoing care at a community interdisciplinary breast center were entered into a prospective database of quality measures that were defined by the NCBC. Retrospective review of patients from 2004 to 2006 was performed and subsequent quality indicator data was submitted electronically to the NCBC National Quality Measures for Breast Centers (NQMBC(TM) ) program. The percentage of new cancer diagnoses made by needle biopsy techniques was 94%, 95% and 96% from 2004 to 2006. Sentinel lymph node utilization in eligible patients was 93%, 96% and 91% from 2004 to 2006 and the immediate intraoperative pathologic frozen section false negative rate of the sentinel lymph node was 6.5%, 4.7% and 4%. Chart documentation of "patient participation in shared decision making for breast conserving therapy versus mastectomy" improved from 74% to 99% (p<0.05) from 2004 to 2006. Adjuvant systemic treatment for stage 2 breast cancer occurred in 76%, 89% and 77% of patients from 2004 to 2006. Neutropenia requiring hospital admission occurred in no patients in 2004 but in 4.8% and 2.9% in 2005 and 2006. The re-excision lumpectomy rates for stage 0, 1, 2, and 3 breast cancer patients from 2004 to 2006 was 14.2%, 22% and 24.8%. Quality indicator data was submitted to the NQMBC(TM) with successful confidential receipt of peer performance comparisons. Voluntary interdisciplinary institutional audits of breast cancer quality can be successfully submitted to the NQMBC(TM) with confidential peer performance comparison. | Humans | Female | Breast Neoplasms | Prospective Studies | OK |
|  |  |  |  |  |  | Archives of Physical Medicine and Rehabilitation | 2010-09-00 Sep 2010 | OBJECTIVES: To provide a review of contemporary participation measures' conceptual foundations, psychometric properties and linkage to the International Classification of Functioning, Disability and Health (ICF). DATA SOURCES: Major medical databases, including PubMed, Medline, PsychInfo, and CINAHL. STUDY SELECTION: Articles that described the psychometric properties of generic measures of adult participation published in English between 1998 and 2008 were included. DATA EXTRACTION: Two reviewers independently reviewed each measure using recognized quality criteria for health questionnaires. Individual items were linked to the ICF using established linking rules. DATA SYNTHESIS: Eight measures met the inclusion criteria: Impact on Participation and Autonomy, ICF Measure of Participation and Activities, Keele Assessment of Participation, Assessment of Life Habits, Participation Profile, Participation Survey/Mobility, Participation Scale, and the Participation Measure for Post-Acute Care. The selected measures were based primarily on the ICF and demonstrated moderate to good validity and reliability, but psychometric information was often incomplete. The most commonly addressed ICF domains were mobility; domestic life; social interactions; major life domains; and community, social, and civic life. CONCLUSIONS: This review provides tools--a detailed review of individual participation measures, a comparative table of the measures' psychometric properties, and ICF linkages-and a set of 3 guiding questions to help users select appropriate participation measures. | Humans | Patient Participation | Questionnaires | Psychometrics | OK |
| Laura | Koopman | Peter | Spreeuwenberg | Jany | Rademakers | Journal of Medical Internet Research | 2011-00-00 2011 | BACKGROUND: The Internet is increasingly considered to be an efficient medium for assessing the quality of health care seen from the patients' perspective. Potential benefits of Internet surveys such as time efficiency, reduced effort, and lower costs should be balanced against potential weaknesses such as low response rates and accessibility for only a subset of potential participants. Combining an Internet questionnaire with a traditional paper follow-up questionnaire (mixed-mode survey) can possibly compensate for these weaknesses and provide an alternative to a postal survey. OBJECTIVE: To examine whether there are differences between a mixed-mode survey and a postal survey in terms of respondent characteristics, response rate and time, quality of data, costs, and global ratings of health care or health care providers (general practitioner, hospital care in the diagnostic phase, surgeon, nurses, radiotherapy, chemotherapy, and hospital care in general). METHODS: Differences between the two surveys were examined in a sample of breast care patients using the Consumer Quality Index Breast Care questionnaire. We selected 800 breast care patients from the reimbursement files of Dutch health insurance companies. We asked 400 patients to fill out the questionnaire online followed by a paper reminder (mixed-mode survey) and 400 patients, matched by age and gender, received the questionnaire by mail only (postal survey). Both groups received three reminders. RESULTS: The respondents to the two surveys did not differ in age, gender, level of education, or self-reported physical and psychological health (all Ps > .05). In the postal survey, the questionnaires were returned 20 days earlier than in the mixed-mode survey (median 12 and 32 days, respectively; P < .001), whereas the response rate did not differ significantly (256/400, 64.0% versus 242/400, 60.5%, respectively; P = .30). The costs were lower for the mixed-mode survey (€2 per questionnaire). Moreover, there were fewer missing items (3.4% versus 4.4%, P = .002) and fewer invalid answers (3.2% versus 6.2%, P < .001) in the mixed-mode survey than in the postal survey. The answers of the two respondent groups on the global ratings did not differ. Within the mixed-mode survey, 52.9% (128/242) of the respondents filled out the questionnaire online. Respondents who filled out the questionnaire online were significantly younger (P < .001), were more often highly educated (P = .002), and reported better psychological health (P = .02) than respondents who filled out the paper questionnaire. Respondents to the paper questionnaire rated the nurses significantly more positively than respondents to the online questionnaire (score 9.2 versus 8.4, respectively; χ²₁ = 5.6). CONCLUSIONS: Mixed-mode surveys are an alternative method to postal surveys that yield comparable response rates and groups of respondents, at lower costs. Moreover, quality of health care was not rated differently by respondents to the mixed-mode or postal survey. Researchers should consider using mixed-mode surveys instead of postal surveys, especially when investigating younger or more highly educated populations. | Humans | Patient Participation | Patient Satisfaction | Questionnaires | OK |
| Robert D. | Reid | Treva | McCumber | Sherry L. | Grace | Journal of Cardiopulmonary Rehabilitation and Prevention | 2013-10-00 2013 Sep-Oct | PURPOSE: Despite the established benefits of cardiac rehabilitation (CR), it remains significantly underutilized. It is unknown whether patient barriers to enrollment and adherence are addressed by offering choice of program type. The purpose of this study was to examine barriers to participation in CR by program type (site- vs home-based program) and the relation of these barriers to degree of program participation and exercise behavior. METHODS: One thousand eight hundred nine cardiac patients from 11 hospitals across Ontario completed a sociodemographic survey inhospital, and clinical data were extracted from medical records. They were mailed a followup survey 1 year later, which included the Cardiac Rehabilitation Barriers Scale and the Physical Activity Scale for the Elderly. Participants were also asked whether they attended CR, the type of program model attended, and the percentage of prescribed sessions completed. RESULTS: Overall, 939 patients (51.9%) participated in CR, with 96 (10.3%) participating in a home-based program. Home-based participants reported significantly greater CR barriers, including distance, than site-based participants (P < .001). Mean barrier scores were significantly and negatively related to session completion and physical activity among site-based (Ps < .05), but not home-based (NS), CR participants. CONCLUSION: The barriers to CR are significantly different among patients attending site- versus home-based programs, suggesting appropriate use of alternative models of care. Patient preferences should be considered when allocating patients to program models. Once in CR, programs should work toward identifying and tackling barriers among site-based participants. | Male | Humans | Patient Participation | Questionnaires | OK |
| Margareta | Ehnfors |  |  |  |  | International journal of nursing terminologies and classifications: the official journal of NANDA International | 2010-03-00 2010 Jan-Mar | PURPOSE. To depict what patients describe as patient participation and whether descriptions of patient participation are affected by gender, age, healthcare contact, and duration of disease. DATA SOURCES. Current patients (n= 362) responded to a questionnaire on participation. DATA SYNTHESIS. Patients' descriptions focused on having knowledge, rather than being informed, and on interacting with health professionals, rather than merely partaking in decision making. CONCLUSIONS. Patients' descriptions of participation correspond with the International Classification of Functioning, Disability and Health's definition, which includes "being involved in a life situation." Healthcare legislation and professionals employ a narrower concept of patient participation as defined by, e.g., Medical Subject Headings. PRACTICE IMPLICATIONS. Findings suggest that health professionals need to embrace what patients describe as participation. | Humans | Patient Participation | Questionnaires | Delivery of health care | OK |
| Robert | Beyer | Samanta | Viana | Han-Seok | Seo | European archives of oto-rhino-laryngology: official journal of the European Federation of Oto-Rhino-Laryngological Societies (EUFOS): affiliated with the German Society for Oto-Rhino-Laryngology - Head and Neck Surgery | 2011-09-00 Sep 2011 | Odor identification tests are widely used for the general screening of olfactory function. However, the administration of odor identification tests is often limited due to a lack of investigators' time. Therefore, we attempted to design a computer-controlled olfactometer to present a self-administered odor identification test. The results produced by means of this olfactometer were evaluated in terms of validity and test-retest reliability. To test the validity, participants' performance in the odor identification test using the olfactometer was compared with their performance in the odor identification test using the validated assessment of the "Sniffin' Sticks" test. The ten-item odor identification test was performed two times using two different methods: (1) the self-administered test using the computer-controlled olfactometer and (2) the foreign-administered test using the "Sniffin' sticks." To examine test-retest reliability, 20 participants were asked to repeat these tests on a different day. Participants reached significantly higher scores on a foreign-administered odor identification test using the "Sniffin' sticks" than on the olfactometer-based test; however, this effect was driven by two less correctly identified odors in the olfactometer-based test. The significant difference between both methods in the mean scores disappeared after excluding two odors from the analysis. In addition, both methods showed no significant difference in scores obtained during the first and second session, indicating that results were consistent between sessions. In conclusion, our findings demonstrate that the computer-controlled olfactometer designed in this study can be used for a self-administered odor identification test. | Male | Humans | Patient Participation | Young Adult | OK |
| Junji | Furuse | Hiroshi | Kasugai | Masaru | Konishi | Japanese Journal of Clinical Oncology | 2010-10-00 Oct 2010 | OBJECTIVE: The aim of this study was to explore why patients accepted or declined to participate in a randomized clinical trial, which was subsequently discontinued because of a low recruitment rate. METHODS: Forty-one patients were invited to participate in a randomized clinical trial that aimed to compare local ablation therapies and surgery to treat small asymptomatic hepatocellular carcinomas. These patients were then asked to answer a questionnaire that assessed patient perception and reasons for accepting or declining to enroll in the randomized clinical trial. When patients had a strong preference for a specific treatment, the questionnaire assessed why, how and when they had chosen it. RESULTS: The response rate was 6/6 (100%) and 30/35 (86%) for the participant and non-participant groups, respectively. Among the 30 non-participants, 23 had a strong preference for local ablation therapies, which was less invasive and offered shorter hospitalization. Patient preference for a specific treatment often stemmed from their consultations with a clinician who referred them to a specialist hospital. Patients without strong preference for a specific treatment participated in the randomized clinical trial because of altruistic motivations. CONCLUSION: When new treatments that are innovative and less burdensome become widespread, they are difficult to compare with standard therapy utilizing a well-designed randomized clinical trial. Consequently, when an innovative treatment is developed, investigators should consider designing a randomized clinical trial as early as possible. | Humans | Patient Participation | Questionnaires | patient preference | OK |
|  |  |  |  |  |  | Disability and Health Journal | 2014-01-00 Jan 2014 | Supported decision-making is increasingly being promoted as an alternative to guardianship for persons aging with intellectual disabilities. Proponents argue that supported decision-making, unlike guardianship, empowers persons with disabilities by providing them with help in making their own decisions, rather than simply providing someone else to make decisions for them. To evaluate the empirical support for these claims, we reviewed the evidence base on supported decision-making. Our review found little such empirical research, suggesting that significant further research is warranted to determine whether--and under what conditions--supported decision-making can benefit persons with intellectual disabilities. Indeed, without more empirical evidence as to how supported decision-making functions in practice, it is too early to rule out the possibility it may actually disempower individuals with disabilities by facilitating undue influence by their alleged supporters. We therefore suggest several key areas for future research. | Decision Making | Humans | Patient Participation | Aging | OK |
| Lauren | Levitz | Mali | Rochas | Kotou | Sangare | PloS One | 2013-00-00 2013 | Despite a high prevalence of oncogenic human papilloma virus (HPV) infection and cervical cancer mortality, HPV vaccination is not currently available in Mali. Knowledge of HPV and cervical cancer in Mali, and thereby vaccine readiness, may be limited. Research staff visited homes in a radial pattern from a central location to recruit adolescent females and males aged 12-17 years and men and women aged ≥ 18 years (N = 51) in a peri-urban village of Bamako, Mali. Participants took part in structured interviews assessing knowledge, attitudes, and practices related to HPV, cervical cancer, and HPV vaccination. We found low levels of HPV and cervical cancer knowledge. While only 2.0% of respondents knew that HPV is a sexually transmitted infection (STI), 100% said they would be willing to receive HPV vaccination and would like the HPV vaccine to be available in Mali. Moreover, 74.5% said they would vaccinate their child(ren) against HPV. Men were found to have significantly greater autonomy in the decision to vaccinate themselves than women and adolescents (p = 0.005), a potential barrier to be addressed by immunization campaigns. HPV vaccination would be highly acceptable if the vaccine became widely available in Bamako, Mali. This study demonstrates the need for a significant investment in health education if truly informed consent is to be obtained for HPV vaccination. Potential HPV vaccination campaigns should provide more information about HPV and the vaccine. Barriers to vaccination, including the significantly lower ability of the majority of the target population to autonomously decide to get vaccinated, must also be addressed in future HPV vaccine campaigns. | Male | Decision Making | Humans | Patient Participation | OK |
| M. Chris | Runken | Mechele | Lee | Ellen | Sulcs | Headache | 2013-08-00 2013 Jul-Aug | OBJECTIVES: To investigate the factors that influence a migraineur's beliefs regarding oral triptans for the acute treatment of migraines and to provide further insight into patients' decision-making process when faced with migraine. METHODS: A multicenter, cross-sectional, observational study of subjects currently prescribed an oral triptan medication for the acute treatment of migraine headaches. Subjects were recruited from 6 headache clinics and one primary care practice in the United States. Enrolled subjects completed a questionnaire that could be completed either at the site as part of the visit or at home. The questionnaire comprised 27 questions assessing demographic characteristics, migraine history, migraine frequency and severity, and general beliefs about migraine treatments. The study population was stratified into 2 cohorts (Early Treatment and Delayed Treatment) based on how they typically use their oral triptan to treat a typical migraine. RESULTS: A total 506 subjects were enrolled in the study, of which 502 were stratified into the Early Treatment cohort (41.2%) and Delayed Treatment cohort (58.8%). Demographic and clinical characteristics were generally similar between the 2 cohorts. In terms of general treatment patterns, there were notable differences between the Delayed and Early Treatment cohorts, with the Delayed Treatment cohort significantly more likely to take an over-the-counter (OTC) or non-triptan medication first (P ≤ .001) and only take a triptan if the OTC or non-triptan medication did not work (P ≤ .001). Furthermore, 55% of the Delayed Treatment cohort delayed taking a triptan to be certain that the headache was a migraine (vs 32% of the Early Treatment cohort; P ≤ .001). When asked to specify the reasons for delaying treatment with a triptan, the Delayed Treatment cohort had, in general, greater concerns about using their oral triptan in comparison with the Early Treatment cohort. In particular, respondents were primarily concerned with running out of their triptan medication with 35% of the Delayed Treatment cohort expressing this concern compared with 22% of the Early Treatment cohort (P ≤ .001). Statistically significant differences were also noted for concerns about taking medications (P ≤ .001), side effects (P ≤ .05), expense (P ≤ .01), and taking prescription medications (P ≤ .001). CONCLUSIONS: Results build upon previously published studies and suggest that patient beliefs directly influence how migraineurs manage their migraines and have implications for patient outcomes. Such insights should be used to facilitate physician-patient communication and reinforce the need for patient-centered care to improve patient outcomes. | Male | Decision Making | Humans | Patient Participation | OK |
| D. T. | Ubbink | R. | Balm | M. J. W. | Koelemay | European Journal of Vascular and Endovascular Surgery: The Official Journal of the European Society for Vascular Surgery | 2014-09-00 Sep 2014 | OBJECTIVE: Abdominal aortic aneurysm patients tend to be informed inconsistently and incompletely about their disorder and the treatment options open to them. The objective of this trial was to evaluate whether these patients are better informed and experience less decisional conflict regarding their treatment options after viewing a decision aid. DESIGN: A six-centre, randomised clinical trial comparing a decision aid plus regular information versus regular information from the surgeon. METHODS: Included patients had recently been diagnosed with an asymptomatic abdominal aortic aneurysm at least 4 cm in diameter. The decision aid consisted of a one-time viewing of an interactive CD-ROM elaborating on elective surgery versus watchful waiting. Generally, the decision aid advised patients with aneurysms less than 5.5 cm to agree to watchful waiting, for larger aneurysms the decision aid provided insight into the balance of benefit and harm of surgical and conservative approaches, taking into account age, co-morbidity and size of the aneurysm. The primary outcome was patient decisional conflict measured at 1 month follow-up (Decisional Conflict Scale). Secondary outcomes were patient knowledge, anxiety and satisfaction. RESULTS: In 178 aneurysm patients, decisional conflict scores did not differ significantly between the decision aid and the regular information groups (22 vs. 24 on the 0-100 Decisional Conflict Scale; p = .33). Patients in the decision aid group had significantly better knowledge (10.0 vs. 9.4 out of 13 points; p = .04), whereas anxiety levels (4.4 and 5.0 on a 0-21 scale; p = .73) and satisfaction scores (74 and 73 on a 0-100 scale; p = .81) were similar in both groups. CONCLUSION: In addition to regular patient-surgeon communication, a decision aid helps to share treatment decisions with abdominal aortic aneurysm patients by increasing their knowledge about the disorder and available treatment options without raising anxiety levels; however, it does not reduce decisional conflict, nor does it improve satisfaction. | Male | Decision Support Techniques | Humans | Patient Participation | OK |
| Lior | Fink |  |  |  |  | Health Expectations: An International Journal of Public Participation in Health Care & Health Policy | 2014-12-00 décembre 2014 | Background: Shared decision making (SDM) encourages the patient to play a more active role in the process of medical consultation and its primary objective is to find the best treatment for a specific patient. Recent findings, however, show that patient preferences cannot be easily or accurately judged on the basis of communicative exchange during routine office visits, even for patients who seek to expand their role in medical decision making (MDM). Objective: The objective of this study is to improve the quality of patient–physician communication by developing a novel design process for SDM and then demonstrating, through a case study, the applicability of this process in enabling the use of a normative model for a specific medical situation. Design: Our design process goes through the following stages: definition of medical situation and decision problem, development/identification of normative model, adaptation of normative model, empirical analysis and development of decision support systems (DSS) tools that facilitate the SDM process in the specific medical situation. Case study: This study demonstrates the applicability of the process through the implementation of the general normative theory of MDM under uncertainty for the medical–financial dilemma of choosing a physician to perform amniocentesis. Discussion: The use of normative models in SDM raises several issues, such as the goal of the normative model, the relation between the goals of prediction and recommendation, and the general question of whether it is valid to use a normative model for people who do not behave according to the model's assumptions. (PsycINFO Database Record (c) 2014 APA, all rights reserved). (journal abstract) | Shared decision making | Decision Making | physicians | Cooperation | OK |
| Charlotte | Delmar | Elizabeth | Cummings | Christian | Nøhr | BMC medical informatics and decision making | 2012-00-00 2012 | BACKGROUND: In today's short stay hospital settings the contact time for patients is reduced. However, it seems to be more important for the patients that the healthcare professionals are easy to get in contact with during the whole course of treatment, and to have the opportunity to exchange information, as a basis for obtaining individualized information and support. Therefore, the aim was to explore the ability of a dialogue-based application to contribute to accessibility of the healthcare professionals and exchangeability of information. METHOD: An application for online written and asynchronous contacts was developed, implemented in clinical practice, and evaluated. The qualitative effect of the online contact was explored using a Web-based survey comprised of open-ended questions. RESULTS: Patients valued the online contacts and experienced feelings of partnership in dialogue, in a flexible and calm environment, which supported their ability to be active partners and feelings of freedom and security. CONCLUSION: The online asynchronous written environment can contribute to accessibility and exchangeability, and add new possibilities for dialogues from which the patients can benefit. The individualized information obtained via online contact empowers the patients. The Internet-based contacts are a way to differentiate and expand the possibilities for contacts outside the few scheduled face-to-face hospital contacts. | Male | Humans | Patient Participation | Patient Satisfaction | OK |
| Isabelle | Scholl |  |  |  |  | Zeitschrift Für Evidenz, Fortbildung Und Qualität Im Gesundheitswesen | 2012-00-00 2012 | The aim of our study is to introduce a statistical framework using latent variable modeling for the investigation of correspondence between patients' and physicians' perceptions that were measured using dyadic instruments. This statistical approach combines multitrait-multimethod and measurement invariance methodologies. In an illustrative example we used a sample of 285 primary care consultations on chronic diseases to test correspondence between patients' and physicians' views on the process of shared decision-making (SDM), which was measured by the patient and physician version of the nine-item Shared Decision-Making Questionnaire (SDM-Q-9 and SDM-Q-Doc), respectively. We revealed that while patients and physicians seem to agree on what is the core of SDM, they differ in their ratings regarding to which extent it is present in a certain consultation. The described statistical approach provides important insights into correspondence between perceptions of different stakeholders that cannot be gained using traditional approaches. However, its generalizability is questionable due to the partly explorative data-driven approach and its flexibility is limited by the requirement of samples including at least 200 cases. | Male | Decision Making | Humans | Patient Satisfaction | OK |
| René | Nielsen | Eva | Tuninger | Sten | Levander | European Neuropsychopharmacology: The Journal of the European College of Neuropsychopharmacology | 2013-10-00 Oct 2013 | The self-report Drug Attitude Inventory (DAI), in 30- and 10-item versions, provides unique information of clinical relevance for monitoring treatment adherence among people diagnosed with schizophrenia. The primary purpose of this paper was to evaluate the 10-item version among patients living in sheltered housing. Data were collected among 68 persons living in sheltered housing, most of them (82%) diagnosed with schizophrenia, 6% with non-organic psychoses, and 12% with other diagnoses. The dichotomic response format of the original DAI-10 was replaced by a 4-point Likert scale, in order to improve the resolution of the scale. Over 90% of the participants produced meaningful scores. A factor analysis suggested a 2-factor orthogonal structure: one highly homogenous factor (5 items) reflected wanted effects of the drug and displayed a bimodal distribution; one factor (3 items) reflected side effects. One item concerned the perceived control over one's drug treatment, which is a key clinical issue. One item was conceptually ambiguous and displayed no correlations with the other items. On the basis of the results we suggest cut-off scores which indicate the need for three kinds of adherence-improving interventions. Summing up, by dropping one item and using a Likert scale response format, the resulting instrument, DAI-9, appears to be an easy-to-use self-report instrument for monitoring drug attitudes and to identify needs for treatment adherence interventions among seriously ill patients. | Humans | Patient Participation | Young Adult | Aged | OK |
| Omar A. | Ibrahimi | Natalie | Kim | Jeremy | Bordeaux | JAMA dermatology | 2014-05-00 May 2014 | IMPORTANCE: Excisional skin cancer surgery is a common procedure, with no formal consensus for mitigating the risk of wrong-site cutaneous surgery. OBJECTIVE: To systematically consider the usefulness and feasibility of proposed methods for correct biopsy site identification in dermatology. EVIDENCE REVIEW: Survey study with a formal consensus process. Item development was via a literature review and expert interviews, followed by 2 stages of a Delphi process to develop consensus recommendations. FINDINGS: In total, 2323 articles were reviewed in the literature search, with data extraction from 14. Twenty-five experts underwent 30-minute structured interviews, which were transcribed and coded. The resulting survey was composed of 42 proposed interventions by multiple stakeholders (biopsying physicians, operating physicians, nurses, ancillary staff, patients, caregivers, and family members) at 3 time points (day of biopsy, delay and consultation period, and day of definitive surgery). Two rounds of a Delphi process with 59 experts (25 academic and 34 private practice) scored the survey. Strong consensus was obtained on 14 behaviors, and moderate consensus was obtained on 21 other behaviors. In addition, a 2-state simultaneous algorithm was developed to model surgeon behavior on the day of definitive surgery based on surgeon and patient perceptions. CONCLUSIONS AND RELEVANCE: When definitive surgery is performed after the initial biopsy and by a different surgeon, procedures can be implemented at several time points to increase the likelihood of correct site identification. The specific circumstances of a case suggest which methods may be most appropriate and feasible, and some may be implemented. The risk of wrong-site cutaneous surgery can be reduced but not eliminated. | Male | Humans | Patient Participation | Female | OK |
|  |  |  |  |  |  | Psychiatria Danubina | 2011-09-00 Sep 2011 | Effective communication between patients, their families, their carers and health care professionals is paramount to the delivery of high quality care. Addressing the ideas, concerns and expectations of these groups may improve their healthcare experience. We propose that opening a new channel of communication between patients, families, carers and healthcare professionals on the wards would improve the delivery of healthcare. We present a novel written communication aid- the Care Communication Aid (CCA), with preliminary data from secondary and tertiary healthcare trials demonstrating its efficacy and shortcomings, and the reaction of both recipients and providers of healthcare to this novel approach. | Humans | Patient Participation | communication | Family | OK |
| Sarah | Krein |  |  |  |  | The American Journal of Nursing | 2013-01-00 Jan 2013 | | Decision Support Techniques | Humans | Patient Participation | United States | OK |
| G. | Matthew | M. | Mercer |  |  | Journal of Medical Ethics | 2010-09-00 Sep 2010 | The Treatment Escalation Plan (TEP) was introduced into our trust in an attempt to improve patient involvement and experience of their treatment in hospital and to embrace and clarify a wider remit of treatment options than the Do Not Resuscitate (DNR) order currently offers. Our experience suggests that the patient and family are rarely engaged in DNR discussions. This is acutely relevant considering that the Mental Capacity Act (MCA) now obliges these discussions to take place. The TEP is a form that the doctor completes, ideally with the competent patient or close relative, documenting what treatment options would be appropriate if that patient were to become acutely unwell. Ventilation of the lungs, cardiac resuscitation, renal replacement therapy, intravenous fluids and antibiotics are all discussed. The study evaluated patient and relative experiences with the TEP. 55 patients or their relatives were interviewed regarding their experience of the TEP and thoughts regarding the process. 96% of patients and relatives evaluated thought that the TEP was a good idea. Free text comments were all positive and only 34% of patients claimed to feel anxious when completing the form. Following this study, the TEP has been expanded hospital wide and into the community within our trust. Discussions are currently taking place in hospitals within our region to introduce the TEP form into other local trusts. | Humans | Patient Participation | Questionnaires | Aged | OK |
| Nelda P. | Wray |  |  |  |  | Patient Education and Counseling | 2010-11-00 Nov 2010 | OBJECTIVE: To develop a preliminary version of a post-doctor visit self-assessment tool that patients with hypertension can use to evaluate their communication behaviors. High-quality communication between patient and doctor may have a positive effect on blood pressure control in hypertensive patients. Patients' communicative behaviors such as asking questions influence those of doctors, but most existing measurement tools assess doctors' behaviors rather than patients'. METHODS: The tool is intended for use by African American or Caucasian American adults with hypertension, regardless of literacy level. The project included theory-based development of the item pool, usability testing (8 individuals), and cognitive response testing (13 additional individuals). MAIN RESULTS: After multiple iterations, the preliminary version includes 138 items in 7 theory-based domains. CONCLUSION AND PRACTICE IMPLICATIONS: The self-assessment tool is ready for testing of item and scale reliability and validity and consequent item reduction. This tool could prove useful in trials evaluating whether patients with hypertension who learn to be better communicators are more likely to achieve blood pressure control. In addition, because it asks patients to reflect on their use of specific behaviors that can be learned, the tool might also help patients in clinical practice to assume more active roles during their medical interactions. | Male | Humans | Patient Participation | Physician-Patient Relations | OK |
|  |  |  |  |  |  | Perspectives in health information management / AHIMA, American Health Information Management Association | 2010-00-00 2010 | BACKGROUND: The Emergency Care Summary (ECS) was introduced in 2006 to allow aspects of the general practitioner (GP; family doctor, equivalent to primary care physician) medical record to be viewed in hospitals and out-of-hours centers in Scotland. Records were automatically uploaded unless patients actively opted out. This study investigated patient awareness and acceptance of this process. METHODS: This was a questionnaire survey of patients in a GP surgery (office) in Paisley, Scotland. RESULTS: Survey results indicated that 42 percent of patients were aware of the ECS, and 16 percent said that they recognized the leaflet posted to households. Of those who recognized the leaflet, 92 percent said they were happy for their record to be part of the system, while the others did not realize their record was to be included. Having read the leaflet, 97 percent said that they were happy for their record to be included in the ECS. CONCLUSIONS: This study shows that most patients were not aware of the Emergency Care Summary or did not remember seeing the leaflet posted to households. Having read the leaflet, the vast majority of patients were happy for their records to be included in the system. The low awareness of the ECS calls into question the validity of an implied consent model using an information leaflet distributed by post. | Male | Humans | Patient Participation | Questionnaires | OK |
|  |  |  |  |  |  | Archives of Disease in Childhood | 2013-04-00 Apr 2013 | | Humans | Patient Participation | Child | Health Care Surveys | OK |
| Francisco | Lopez-Jimenez | Randal J. | Thomas | Thomas G. | Allison | Mayo Clinic Proceedings | 2014-09-00 Sep 2014 | The recently published American College of Cardiology (ACC)/American Heart Association (AHA) guidelines for cardiovascular risk assessment provide equations to estimate the 10-year and lifetime atherosclerotic cardiovascular disease (ASCVD) risk in African Americans and non-Hispanic whites, include stroke as an adverse cardiovascular outcome, and emphasize shared decision making. The guidelines provide a valuable framework that can be adapted on the basis of clinical judgment and individual/institutional expertise. In this review, we provide a perspective on the new guidelines, highlighting what is new, what is controversial, and potential adaptations. We recommend obtaining family history of ASCVD at the time of estimating ASCVD risk and consideration of imaging to assess subclinical disease burden in patients at intermediate risk. In addition to the adjuncts for ASCVD risk estimation recommended in the guidelines, measures that may be useful in refining risk estimates include carotid ultrasonography, aortic pulse wave velocity, and serum lipoprotein(a) levels. Finally, we stress the need for research efforts to improve assessment of ASCVD risk given the suboptimal performance of available risk algorithms and suggest potential future directions in this regard. | Male | Decision Making | Humans | Female | OK |
| Margaret | Brinich |  |  |  |  | Journal of Medical Ethics | 2010-10-00 Oct 2010 | STUDY OBJECTIVES: The emergency department (ED) provides an arena for patient enrollment into a variety of research studies even for non-critically ill patients. Given the types of illness, time constraints and sense of urgency that exists in the ED environment, concern exists about whether research subjects in the ED can provide full consent for participation. We sought to identify enrolled research subjects' perspectives on the informed consent process for research conducted in the ED. METHODS: This was a prospective, observational study of ED subjects, 18&emsp14;years or older, who had been approached to participate in research in the ED and who were judged to have decision-making capacity. Exclusions were critical illness and refusal to participate. Subjective were followed up within 1&emsp14;week after enrolling using structured phone interviews by trained interviewers. RESULTS: During the study period, 229 eligible patients were approached to participate in both a target study and this study. Of these, 66% (150/229) agreed to participate in this study, at least to the extent of allowing us access to their demographic data. The study participant group was similar in terms of gender to this particular ED's patient population but had significantly more African-Americans and persons older than 45. CONCLUSION: Despite rigorous time constraints and rapid throughput times, the majority of subjects who consented to research participation in the ED felt that they were sufficiently informed and had adequate time to decide to participate. | Male | Decision Making | Humans | Patient Participation | OK |
| Arnold L. | Potosky | Anita | Ambs | Yulei | He | Journal of the National Cancer Institute | 2011-03-02 Mar 2, 2011 | BACKGROUND: Clinical trials are critical for evaluating new cancer therapies, but few adult patients participate in them. Physicians have an important role in facilitating patient participation in clinical trials. We examined the characteristics of specialty physicians who participate in clinical trials by enrolling or referring patients, the types of trials in which they participate, and factors associated with physicians who report greater involvement in clinical trials. METHODS: We analyzed data from the Cancer Care Outcomes Research and Surveillance Consortium. The study included 1533 specialty physicians who cared for colorectal and lung cancer patients (496 medical oncologists, 228 radiation oncologists, and 809 surgeons) and completed a survey conducted during 2005-2006 (response rate = 61.0%). Descriptive statistics were used to characterize physicians' personal and practice characteristics, and regression models were used to examine associations between these characteristics and physician participation in clinical trials. All statistical tests were two-sided. RESULTS: A total of 87.8% of medical oncologists, 66.1% of radiation oncologists, and 35.0% of surgeons reported referring or enrolling one or more patients in clinical trials during the previous 12 months. The mean number of patients referred or enrolled by these physicians was 17.2 (95% confidence interval [CI] = 15.5 to 18.9) for medical oncologists, 9.5 (95% CI = 7.7 to 11.3) for radiation oncologists, and 12.2 (95% CI = 9.8 to 14.6) for surgeons (P < .001). Specialty type, involvement in teaching, and affiliation with a Community Clinical Oncology Program (CCOP) and/or a National Cancer Institute-designated cancer center were associated with physician trial participation and enrolling more patients (all Ps < .05). Two-thirds of physicians with a CCOP or National Cancer Institute-designated cancer center affiliation reported participating in trials. CONCLUSIONS: Features of specialty physicians' practice environments are associated with their trial participation, but many physicians at CCOPs and cancer centers do not participate. | Male | Humans | Physician-Patient Relations | physicians | OK |
| Julie R. | Gralow | Carol M. | Moinpour | Antoinette J. | Wozniak | The Oncologist | 2012-00-00 2012 | PURPOSE: Patients older than 65 years are underrepresented in clinical trials. We conducted a prospective study (SWOG S0316) to determine physician- and patient-perceived barriers to breast cancer clinical trial enrollment for older patients. METHODS: Eight geographically diverse SWOG institutions participated. The study assessed patients' and physicians' decisions to enroll in or decline clinical treatment trials, including demographics, trial availability, and eligibility. Patient and physician questionnaires elicited concerns related to treatment, medical status, age, family, and financial or transportation concerns. RESULTS: A total of 1,079 patients were registered and eligible and 909 (84%) returned for follow-up. The major reason for nonaccrual was either trial unavailability or ineligibility (60%). Older patients were less likely to be eligible for trials (65% for age ≥65 years vs. 78% for age <65 years). If eligible, trial participation rates did not differ significantly by age (34% for age ≥65 years vs. 40% for age <65 years). Patients ≥65 years more often were concerned about side effects, had friends opposed to participation, or believed that participation would not benefit other generations. When trials were available and patients were eligible, physicians discussed trial participation with 76% of patients <65 years versus 58% of patients ≥65 years of age. For patients ≥65 years, 11% of physicians indicated age as a reason they did not enroll a patient in a clinical trial. CONCLUSION: Trial unavailability or patient ineligibility were the major reasons for lack of enrollment in breast cancer clinical trials for patients of all ages in this prospective study. Older patients were less likely to be eligible for trials, but if eligible they participated at similar rates to younger patients. | Decision Making | Humans | Patient Participation | Questionnaires | OK |
| Jee Hyun | Kwag | Yeon Deok | Kim | Hye Bin | Yim | Graefe's Archive for Clinical and Experimental Ophthalmology = Albrecht Von Graefes Archiv Für Klinische Und Experimentelle Ophthalmologie | 2010-05-00 May 2010 | PURPOSE: To assess the validity of written informed consent taken from patients prior to undergoing glaucoma surgery by testing their ability to understand the information offered to them during the consent-taking process. METHODS: Seventy-three patients were asked to complete a standardised confidential questionnaire after giving a written informed consent. Surgeons who were taking the consent were also requested to submit their self-evaluation form. Patients' understanding of the information they were given was evaluated using a standardised point scoring system. RESULTS: Fifty patients (68.5%) agreed that they were given enough time to make an informed decision, while 67 doctors (91.8%) claimed that they had allocated enough time to explain the procedures. Fifty-two patients (71.2%) reported that they were given adequate information on the details or diagnosis of their problems, 65 patients (89.0%) on the details of the procedure and 69 patients (94.5%) on the risks and complications. Thirty-four patients (46.6%) were not sure, or refused information on the risks and complications of the procedure. Only half of the patients (57.5%) had overall moderate understanding of their surgical problem, and only 13 patients (17.8%) were able to demonstrate a good overall understanding of their surgical problem. CONCLUSIONS: Although most patients acknowledged that they received sufficient information to give consent, few could objectively recall the information given to them. This study thus raises some doubts on the validity and quality of written informed consent, and highlights the importance of giving clear information to patients undergoing glaucoma surgery. | Male | Humans | Patient Participation | Questionnaires | OK |
| Thongchai | Pratipanawatr | Verawan | Uchaipichat | Narumol | Jarernsiripornkul | European Journal of Clinical Pharmacology | 2014-05-00 May 2014 | PURPOSE: To explore how Thai patients assess symptoms as adverse drug reactions (ADRs). METHODS: Out-patients at two hospitals in Thailand previously reporting suspected ADRs to statins were purposively selected to cover factors relevant to the accuracy of ADR reports. Semi-structured interviews explored the mechanisms participants used to work out whether their symptoms were related to their statin. All interviews were audio-recorded, transcribed and independently thematically analyzed by two researchers. RESULTS: One hundred interviews were suitable for analysis; 52 were male, age range was 36 to 77 years (mean ± S.D.: 59.83 ± 9.14) and most (92) were taking other medicines in addition to statins. Patient assessment of symptoms as ADRs fell into two major themes: medicine-related factors and external factors. Timing relationships were mentioned most frequently (74), followed by information received (55), seeing similar symptoms in others (7) and diagnosis through blood tests (4). Use of multiple medicines, consideration of the medicine versus diseases, symptoms occurring with more than one medicine or relieved through treatment reduced confidence in ADR attribution. Many participants proposed alternative explanations for symptoms, including old age. Lack of information and knowledge were obstacles to the assessment process. CONCLUSIONS: Patients assessed possible ADRs most often by considering timing relationships. While they also used medicine information, Thai patients received inadequate information to help them assess their symptoms. Patients expressed uncertainty and difficulties in deciding attribution when concomitant medicines and diseases were involved. The findings could support the development of a patient-friendly systematic tool for identifying and assessing possible ADRs. | Male | Humans | Patient Participation | Questionnaires | OK |
| Harry J. | de Koning |  |  |  |  | European Spine Journal: Official Publication of the European Spine Society, the European Spinal Deformity Society, and the European Section of the Cervical Spine Research Society | 2010-05-00 May 2010 | Trials often do not succeed in including as many patients as anticipated beforehand. The aim of this paper was to describe why we were not able to include more than a few patients in our randomized controlled treatment trial on the effectiveness of bracing patients with idiopathic scoliosis, and to describe which lessons can be learnt. A pilot study on the willingness to participate in such a trial was conducted amongst 21 patients and their parents. A description of how we prepared and designed this trial, the problems we faced and how we tried to improve the inclusion are given. A total of four patients were included, and 14 refused to participate in an 18-month period. There were a lot less eligible patients than anticipated (40 instead of 100 per year), and the patients' participation rate was much lower than we had found in our pilot study (21% instead of 70%). The trial failed to include more than a few patients because of an overestimation of the number of eligible patients and because a lot less eligible patients were willing to participate compared to our pilot study. One reason for a low participation rate could be that this trial evaluated a frequently used existing treatment instead of a new treatment, and patients and parents might be afraid of not being treated (despite an intensive secure system for the control arm). | Humans | Patient Participation | Questionnaires | Patient Selection | OK |
| Benjamin | Wilfond | Randa | Sifri | Barry | Ziring | Contemporary Clinical Trials | 2011-01-00 Jan 2011 | PURPOSE: This paper describes an ongoing randomized controlled trial designed to assess the impact of genetic and environmental risk assessment (GERA) on colorectal cancer (CRC) screening. METHODS: The trial includes asymptomatic patients who are 50-79years and are not up-to-date with CRC screening guidelines. Patients who responded to a baseline telephone survey are randomized to a GERA or Control group. GERA group participants meet with a nurse, decide whether to have a GERA blood test (a combination of genetic polymorphism and folate), and, if tested, receive GERA feedback. Follow-up telephone surveys are conducted at 1 and 6months. A chart audit is performed at 6months. RESULTS: Of 2,223 eligible patients, 562 (25%) have enrolled. Patients who enrolled in the study were significantly younger than those who did not (p<0.001). Participants tended to be 50-59years (64%), female (58%), white (52%), married (51%), and have more than a high school education (67%). At baseline, most participants had some knowledge of CRC screening and GERA, viewed CRC screening favorably, and reported that they had decided to do screening. Almost half had worries and concerns about CRC. CONCLUSIONS: One in four eligible primary care patients enrolled in the study. Age was negatively associated with enrollment. Prospective analyses using data for all participants will provide more definitive information on GERA uptake and the impact of GERA feedback. | Male | Humans | Patient Participation | Female | OK |
| Julie | Brown | Elizabeth | Clarke | Soufiane | Boufous | BMC public health | 2013-00-00 2013 | BACKGROUND: There are concerns over safety of older drivers due to increased crash involvement and vulnerability to injury. However, loss of driving privileges can dramatically reduce independence and quality of life for older members of the community. The aim of this trial is to examine the effectiveness of a safe transport program for drivers aged 75 years and older at reducing driving exposure but maintaining mobility. METHODS AND DESIGN: A randomised trial will be conducted, involving 380 drivers aged 75 years and older, resident in urban and semi-rural areas of North-West Sydney. The intervention is an education program based on the Knowledge Enhances Your Safety (KEYS) program, adapted for the Australian context. Driving experience will be measured objectively using an in-vehicle monitoring device which includes a global positioning system (GPS) to assess driving exposure and an accelerometer to detect rapid deceleration events. Participation will be assessed using the Keele Assessment of Participation (KAP). Data will be analysed on an intention-to-treat basis; the primary outcomes include driving exposure, rapid deceleration events and scores for KAP. Secondary outcomes include self-reported measures of driving, socialisation, uptake of alternative forms of transport, depressive symptoms and mood. A detailed process evaluation will be conducted, including examination of the delivery of the program and uptake of alternative forms of transport. A subgroup analysis is planned for drivers with reduced function as characterized by established cut-off scores on the Drivesafe assessment tool. DISCUSSION: This randomised trial is powered to provide an objective assessment of the efficacy of an individually tailored education and alternative transportation program to promote safety of older drivers but maintain mobility. TRIAL REGISTRATION: Australian New Zealand Clinical Trials Registry ACTRN12612000543886. | Male | Humans | Patient Participation | Female | OK |
| Nancy | Stark | Susan | Williford | Jeff | Giguere | The Journal of Supportive Oncology | 2013-03-00 Mar 2013 | BACKGROUND: Coenzyme Q10 (CoQ10) is a common antioxidant supplement with known cardioprotective effects and potential anticancer benefits. OBJECTIVES: We performed a randomized, double-blind, placebo-controlled study of oral CoQ10 in female breast cancer patients with the primary objective of determining CoQ10's effects on self-reported fatigue, depression, and quality of life (QOL). Methods Eligible women with newly diagnosed breast cancer and planned adjuvant chemotherapy were randomized to oral supplements of 300 mg CoQ10 or placebo, each combined with 300 IU vitamin E, divided into 3 daily doses. Treatment was continued for 24 weeks. Blood tests, QOL measures, and levels of plasma CoQ10 and vitamin E were obtained at baseline and at 8, 16, and 24 weeks. Mixed-effects models were used to assess treatment differences in outcomes over time. RESULTS: Between September 2004 and March 2009, 236 women were enrolled. Treatment arms were well balanced with respect to age (range, 28-85 years), pathologic stage (stage 0, 91%; stage 1, 8%; stage II, 1%), ethnicity (white, 87%; black, 11%; Hispanic, 2%), and planned therapy. Baseline CoQ10 levels in the CoQ10 and placebo arms were 0.70 and 0.73 microg/mL, respectively; the 24-week CoQ10 levels were 1.83 and 0.79 microg/mL, respectively. There were no significant differences between the CoQ10 and placebo arms at 24 weeks for scores on the Profile of Mood States-Fatigue questionnaire (least squares means, 7.08 vs 8.24, P = .257), the Functional Assessment of Chronic Illness Therapy-Fatigue tool (37.6 vs 37.6, P = .965), the Functional Assessment of Cancer Therapy-Breast Cancer instrument (111.9 vs 110.4, P = .577), or the Center for Epidemiologic Studies-Depression scale (11.6 vs 12.3, P = .632). CONCLUSIONS: Supplementation with conventional doses of CoQ10 led to sustained increases in plasma CoQ10 levels but did not result in improved self-reported fatigue or QOL after 24 weeks of treatment. | Humans | Patient Participation | Female | Aged | OK |
| Lynne | Stobbart | Helen | Rodgers | Madeleine J. | Murtagh | BMC health services research | 2013-00-00 2013 | BACKGROUND: Tools to support clinical or patient decision-making in the treatment/management of a health condition are used in a range of clinical settings for numerous preference-sensitive healthcare decisions. Their impact in clinical practice is largely dependent on their quality across a range of domains. We critically analysed currently available tools to support decision making or patient understanding in the treatment of acute ischaemic stroke with intravenous thrombolysis, as an exemplar to provide clinicians/researchers with practical guidance on development, evaluation and implementation of such tools for other preference-sensitive treatment options/decisions in different clinical contexts. METHODS: Tools were identified from bibliographic databases, Internet searches and a survey of UK and North American stroke networks. Two reviewers critically analysed tools to establish: information on benefits/risks of thrombolysis included in tools, and the methods used to convey probabilistic information (verbal descriptors, numerical and graphical); adherence to guidance on presenting outcome probabilities (IPDASi probabilities items) and information content (Picker Institute Checklist); readability (Fog Index); and the extent that tools had comprehensive development processes. RESULTS: Nine tools of 26 identified included information on a full range of benefits/risks of thrombolysis. Verbal descriptors, frequencies and percentages were used to convey probabilistic information in 20, 19 and 18 tools respectively, whilst nine used graphical methods. Shortcomings in presentation of outcome probabilities (e.g. omitting outcomes without treatment) were identified. Patient information tools had an aggregate median Fog index score of 10. None of the tools had comprehensive development processes. CONCLUSIONS: Tools to support decision making or patient understanding in the treatment of acute stroke with thrombolysis have been sub-optimally developed. Development of tools should utilise mixed methods and strategies to meaningfully involve clinicians, patients and their relatives in an iterative design process; include evidence-based methods to augment interpretability of textual and probabilistic information (e.g. graphical displays showing natural frequencies) on the full range of outcome states associated with available options; and address patients with different levels of health literacy. Implementation of tools will be enhanced when mechanisms are in place to periodically assess the relevance of tools and where necessary, update the mode of delivery, form and information content. | Humans | Patient Participation | communication | risk assessment | OK |
| Marci | Clark | Carla | DeMuro | Sheri | Fehnel | Value in Health: The Journal of the International Society for Pharmacoeconomics and Outcomes Research | 2012-05-00 May 2012 | OBJECTIVE: In 2004, Willke and colleagues reviewed the efficacy endpoints reported in the labels of new drugs approved in the United States from 1997 through 2002 to evaluate the use of patient-reported outcome (PRO) endpoints. Of the labels reviewed, 30% included PROs. Our study aimed to build on this work by describing the current state of PRO label claims granted for new molecular entities (and biologic license applications since February 2006 after the release of the US Food and Drug Administration (FDA) draft PRO guidance. METHODS: All new molecular entities and biologic license applications approved by the FDA from January 2006 through December 2010 were identified by using the Web page of the FDA Drug Approval Reports. For all identified products, drug approval packages and approved product labels were reviewed to identify PRO endpoint status and to determine the number and type of PRO claims. RESULTS: Of the 116 products identified, 28 (24%) were granted PRO claims; 24 (86%) were for symptoms, and, of these, 9 (38%) claims were pain related. Of the 28 products with PRO claims, a PRO was a primary endpoint for 20 (71%), all symptom related. CONCLUSIONS: The FDA continues to approve PRO claims, with 24% of new molecular entities and biologic license applications being granted. Successful PRO label claims over the past 5 years have generally supported treatment benefit for symptoms specified as primary endpoints. | Humans | Patient Participation | United States | self report | OK |
| John B. | Carlin | Dallas R. | English | Julie A. | Simpson | BMC medical research methodology | 2012-00-00 2012 | BACKGROUND: Retaining participants in cohort studies with multiple follow-up waves is difficult. Commonly, researchers are faced with the problem of missing data, which may introduce biased results as well as a loss of statistical power and precision. The STROBE guidelines von Elm et al. (Lancet, 370:1453-1457, 2007); Vandenbroucke et al. (PLoS Med, 4:e297, 2007) and the guidelines proposed by Sterne et al. (BMJ, 338:b2393, 2009) recommend that cohort studies report on the amount of missing data, the reasons for non-participation and non-response, and the method used to handle missing data in the analyses. We have conducted a review of publications from cohort studies in order to document the reporting of missing data for exposure measures and to describe the statistical methods used to account for the missing data. METHODS: A systematic search of English language papers published from January 2000 to December 2009 was carried out in PubMed. Prospective cohort studies with a sample size greater than 1,000 that analysed data using repeated measures of exposure were included. RESULTS: Among the 82 papers meeting the inclusion criteria, only 35 (43%) reported the amount of missing data according to the suggested guidelines. Sixty-eight papers (83%) described how they dealt with missing data in the analysis. Most of the papers excluded participants with missing data and performed a complete-case analysis (n=54, 66%). Other papers used more sophisticated methods including multiple imputation (n=5) or fully Bayesian modeling (n=1). Methods known to produce biased results were also used, for example, Last Observation Carried Forward (n=7), the missing indicator method (n=1), and mean value substitution (n=3). For the remaining 14 papers, the method used to handle missing data in the analysis was not stated. CONCLUSIONS: This review highlights the inconsistent reporting of missing data in cohort studies and the continuing use of inappropriate methods to handle missing data in the analysis. Epidemiological journals should invoke the STROBE guidelines as a framework for authors so that the amount of missing data and how this was accounted for in the analysis is transparent in the reporting of cohort studies. | Humans | Patient Participation | Observer Variation | Cohort Studies | OK |
| Janine | Davis | Bahman | Guyuron |  |  | Plastic and Reconstructive Surgery | 2012-04-00 Apr 2012 | BACKGROUND: This study is meant to compare the direct and indirect cost of migraine headache care before and after migraine surgery and to evaluate any postoperative changes in patient participation in daily activities. METHODS: Eighty-nine patients enrolled in a migraine surgery clinical trial completed the Migraine-Specific Quality-of-Life Questionnaire, the Migraine Disability Assessment questionnaire, and a financial cost report preoperatively and 5 years postoperatively. RESULTS: Mean follow-up was 63.0 months (range, 56.9 to 72.6 months). Migraine medication expenses were reduced by a median of $1997.26 annually. Median cost reduction for alternative treatment expenses was $450 annually. Patients had a median of three fewer annual primary care visits for the migraine headache treatment, resulting in a median cost reduction of $320 annually. Patients missed a median of 8.5 fewer days of work or childcare annually postoperatively, with a median regained income of $1525. The median total cost spent on migraine headache treatment was $5820 per year preoperatively, declining to $900 per year postoperatively. Total median cost reduction was $3949.70 per year postoperatively. The mean surgical cost was $8378. Significant improvements were demonstrated in all aspects of the Migraine-Specific Quality-of-Life Questionnaire and the Migraine Disability Assessment questionnaire. CONCLUSIONS: Surgical deactivation of migraine trigger sites has proven to be effective for the treatment of severe migraine headache. This study illustrates that the surgical treatment is a cost-effective modality, reducing direct and indirect costs. Patients may also expect improvements in the performance of and increased participation in activities of daily living. CLINICAL QUESTION/LEVEL OF EVIDENCE: Therapeutic, IV. | Male | Humans | Questionnaires | United States | OK |
| Lawrence H. | Kushi | Lois | Lamerato | Christine B. | Ambrosone | Breast (Edinburgh, Scotland) | 2013-08-00 Aug 2013 | PURPOSE: Shared breast cancer treatment decision-making between patients and physicians increases patient treatment satisfaction and compliance and is influenced by physician-related factors. Attitudes and behaviors about patient involvement in breast cancer treatment decisions and treatment-related communication were assessed by specialty among breast cancer physicians of women enrolled in the Breast Cancer Quality of Care Study (BQUAL). RESULTS: Of 275 BQUAL physicians identified, 50.0% responded to the survey. Most physicians spend 46-60 min with the patient during the initial consult visit and 51.5% report that the treatment decision is made in one visit. Oncologists spend more time with new breast cancer patients during the initial consult (p = 0.021), and find it more difficult to handle their own feelings than breast surgeons (p = <0.001). CONCLUSION: Breast surgeons and oncologists share similar attitudes and behaviors related to patient involvement in treatment decision-making, yet oncologists report more difficulty managing their own feelings during the decision-making process. | Male | Decision Making | Humans | Patient Participation | OK |
| Judy | Caesley | Alice | Peterson | Nicholas | Ambler | Musculoskeletal Care | 2014-09-00 Sep 2014 | OBJECTIVES: The consequences of inflammatory arthritis can include depression, anxiety and low mood, reducing patients' quality of life and increasing pressure on the healthcare system. Treatment guidelines recommend psychological support, but data are lacking on the provision available. METHODS: A postal survey concerning psychological support provision was sent to rheumatology units in 143 acute trusts across England. Nurses from 73 rheumatology units (51%) responded. RESULTS: Overall, 73% rated their unit's psychological support provision as 'inadequate' and only 4% rated it as 'good'. Few units believed that psychological support did not fall within their remit (12%), yet only 8% had a psychologist in the team. Most units (68%) did not routinely screen patients to identify psychological difficulties. Referral to other service providers was reported in 42% of units, with 3% very satisfied with this provision. Within units, services containing elements of psychological support ranged from occupational therapy (81%) to psychology/counselling (14%). Psychological approaches used by team members ranged from shared decision making (77%) to cognitive-behavioural approaches (26%). The current barriers to providing psychological support were lack of clinical time and available training (86% and 74%, respectively), and delivery costs (74%). Future facilitators included management support (74%) and availability of skills training (74%). CONCLUSIONS: Rheumatology units viewed psychological support provision as part of their remit but rated their overall provision as inadequate, despite some team members using psychological skills. To improve provision, clinicians' training needs must be addressed and organizational support generated, and further research needs to define adequate psychological support provision from the patient perspective. | | | | | OK |
|  |  |  |  |  |  | Nursing Inquiry | 2014-09-12 Sep 12, 2014 | This systematic review identified and evaluated instruments measuring patients' perceptions of patient-centred nursing care. Of 2629 studies reviewed, 12 were eligible for inclusion. Four instruments were reported: The Individualized Care Scale, the Client-Centred Care Questionnaire, the Oncology patients' Perceptions of the Quality of Nursing Care Scale and the Smoliner scale. These instruments cover themes addressing patient participation and the clinician-patient relationship. Instruments were shown to have satisfactory psychometric properties, although not all were adequately assessed. More research is needed regarding test-retest reliability, convergent and discriminant validity, validity with known groups and structural validity using confirmatory factor analysis. | | | | | OK |
| Lyndal | Trevena | Judy M. | Simpson | Jesse | Jansen | Patient Education and Counseling | 2012-11-00 Nov 2012 | OBJECTIVE: To describe a theoretical framework for assessing knowledge about the possible outcomes of participating in bowel cancer screening for the faecal occult blood test. METHODS: The content of the knowledge measure was based on the UK General Medical Council's screening guidelines and a theory-based approach to assessing gist knowledge (Fuzzy Trace Theory). It comprised conceptual and numeric questions to assess knowledge of the underlying construct (e.g. false positive concept) and the approximate numbers affected (e.g. likelihood of a false positive). The measure was used in a randomised controlled trial involving 530 adults with low education, to compare the impact of a bowel screening decision aid with a screening information booklet developed for the Australian Government National Bowel Cancer Screening Program. RESULTS: The numeric knowledge scale was particularly responsive to the effects of the decision aid; at follow-up decision aid participants' numeric knowledge was significantly greater than the controls (P<0.001). This contrasts with the conceptual knowledge scale which improved significantly in both groups from baseline to follow-up (P<0.001). CONCLUSION: Our theory-based knowledge measure was responsive to change in conceptual knowledge and to the effect on numeric knowledge of a decision aid. PRACTICE IMPLICATIONS: This theoretical framework has the potential to guide the development of knowledge measures in other screening settings. | Male | Decision Making | Decision Support Techniques | Humans | OK |
| Rachel E. | Davis |  |  |  |  | Journal of Evaluation in Clinical Practice | 2013-10-00 Oct 2013 | BACKGROUND: Various authorities recommend the participation of patients in promoting patient safety, but little is known about health care professionals' (HCPs') attitudes towards patients' involvement in safety-related behaviours. OBJECTIVE: To investigate how HCPs evaluate patients' behaviours and HCP responses to patient involvement in the behaviour, relative to different aspects of the patient, the involved HCP and the potential error. DESIGN: Cross-sectional fractional factorial survey with seven factors embedded in two error scenarios (missed hand hygiene, medication error). Each survey included two randomized vignettes that described the potential error, a patient's reaction to that error and the HCP response to the patient. SETTING: Twelve hospitals in Switzerland. PARTICIPANTS: A total of 1141 HCPs (response rate 45%). MEASUREMENTS: Approval of patients' behaviour, HCP response to the patient, anticipated effects on the patient-HCP relationship, HCPs' support for being asked the question, affective response to the vignettes. Outcomes were measured on 7-point scales. RESULTS: Approval of patients' safety-related interventions was generally high and largely affected by patients' behaviour and correct identification of error. Anticipated effects on the patient-HCP relationship were much less positive, little correlated with approval of patients' behaviour and were mainly determined by the HCP response to intervening patients. HCPs expressed more favourable attitudes towards patients intervening about a medication error than about hand sanitation. CONCLUSIONS: This study provides the first insights into predictors of HCPs' attitudes towards patient engagement in safety. Future research is however required to assess the generalizability of the findings into practice before training can be designed to address critical issues. | Male | Humans | Patient Participation | Questionnaires | OK |
| C. | McGuigan | M. | Hutchinson | N. | Tubridy | Irish Medical Journal | 2012-10-00 Oct 2012 | Ireland has the lowest number of consultant neurologists per capita in Europe. This results in long waiting lists, overbooked clinics, unnecessary emergency department presentations and patient frustration. In 2006, the neurology department in St. Vincent's University Hospital and the National Healthlink project, launched an internet referral system (Neurolink) for GPs, to alleviate the administrative burden on staff, reduce unnecessary visits for patients, shorten waiting lists and improve patient care. 710 electronic referrals from GPs between December 2006 and January 2011 were analysed. The average time taken to for a consultant to reply to a GP referral was 19hours 8minutes. When asked their opinion as to the suspected aetiology 33.7% (239/710) of GPs selected the option "unknown", followed by epilepsy 12.1% (86/710), migraine 12% (85/710), and multiple sclerosis 7.6% (54/710). Significantly, 19% (127/662) of referrals did not require a neurology outpatient appointment and the GP was given advice. The results highlight the benefits of using an electronic communication system with primary care; allowing prompt response to GP enquires, early initiation of treatment and reducing the number of patients attending hospital clinics. | Male | Humans | Patient Participation | Female | OK |
| Nynke | Boonstra | Lex | Wunderink | Peter | de Jonge | Journal of Medical Internet Research | 2013-10-00 octobre 2013 | Background: Mental health policy makers encourage the development of electronic decision aids to increase patient participation in medical decision making. Evidence is needed to determine whether these decision aids are helpful in clinical practice and whether they lead to increased patient involvement and better outcomes. Objective: This study reports the outcome of a randomized controlled trial and process evaluation of a Web-based intervention to facilitate shared decision making for people with psychotic disorders. Methods: The study was carried out in a Dutch mental health institution. Patients were recruited from 2 outpatient teams for patients with psychosis (N = 250). Patients in the intervention condition (n = 124) were provided an account to access a Web-based information and decision tool aimed to support patients in acquiring an overview of their needs and appropriate treatment options provided by their mental health care organization. Patients were given the opportunity to use the Web-based tool either on their own (at their home computer or at a computer of the service) or with the support of an assistant. Patients in the control group received care as usual (n = 126). Half of the patients in the sample were patients experiencing a first episode of psychosis; the other half were patients with a chronic psychosis. Primary outcome was patient-perceived involvement in medical decision making, measured with the Combined Outcome Measure for Risk Communication and Treatment Decision-making Effectiveness (COMRADE). Process evaluation consisted of questionnaire-based surveys, open interviews, and researcher observation. Results: In all, 73 patients completed the follow-up measurement and were included in the final analysis (response rate 29.2%). More than one-third (48/124, 38.7%) of the patients who were provided access to the Web-based decision aid used it, and most used its full functionality. No differences were found between the intervention and control conditions on perceived involvement in medical decision making (COMRADE satisfaction with communication: F1,68 = 0.422, P = .52; COMRADE confidence in decision: F1,67 = 0.086, P = .77). In addition, results of the process evaluation suggest that the intervention did not optimally fit in with routine practice of the participating teams. Conclusions: The development of electronic decision aids to facilitate shared medical decision making is encouraged and many people with a psychotic disorder can work with them. This holds for both first-episode patients and long-term care patients, although the latter group might need more assistance. However, results of this paper could not support the assumption that the use of electronic decision aids increases patient involvement in medical decision making. This may be because of weak implementation of the study protocol and a low response rate. (PsycINFO Database Record (c) 2014 APA, all rights reserved). (journal abstract) | Decision Making | Mental health | web based tool | psychotic disorder | Doublon |
| Nynke | Boonstra | Lex | Wunderink | Peter | de Jonge | Journal of Medical Internet Research | 2013-00-00 2013 | BACKGROUND: Mental health policy makers encourage the development of electronic decision aids to increase patient participation in medical decision making. Evidence is needed to determine whether these decision aids are helpful in clinical practice and whether they lead to increased patient involvement and better outcomes. OBJECTIVE: This study reports the outcome of a randomized controlled trial and process evaluation of a Web-based intervention to facilitate shared decision making for people with psychotic disorders. METHODS: The study was carried out in a Dutch mental health institution. Patients were recruited from 2 outpatient teams for patients with psychosis (N=250). Patients in the intervention condition (n=124) were provided an account to access a Web-based information and decision tool aimed to support patients in acquiring an overview of their needs and appropriate treatment options provided by their mental health care organization. Patients were given the opportunity to use the Web-based tool either on their own (at their home computer or at a computer of the service) or with the support of an assistant. Patients in the control group received care as usual (n=126). Half of the patients in the sample were patients experiencing a first episode of psychosis; the other half were patients with a chronic psychosis. Primary outcome was patient-perceived involvement in medical decision making, measured with the Combined Outcome Measure for Risk Communication and Treatment Decision-making Effectiveness (COMRADE). Process evaluation consisted of questionnaire-based surveys, open interviews, and researcher observation. RESULTS: In all, 73 patients completed the follow-up measurement and were included in the final analysis (response rate 29.2%). More than one-third (48/124, 38.7%) of the patients who were provided access to the Web-based decision aid used it, and most used its full functionality. No differences were found between the intervention and control conditions on perceived involvement in medical decision making (COMRADE satisfaction with communication: F1,68=0.422, P=.52; COMRADE confidence in decision: F1,67=0.086, P=.77). In addition, results of the process evaluation suggest that the intervention did not optimally fit in with routine practice of the participating teams. CONCLUSIONS: The development of electronic decision aids to facilitate shared medical decision making is encouraged and many people with a psychotic disorder can work with them. This holds for both first-episode patients and long-term care patients, although the latter group might need more assistance. However, results of this paper could not support the assumption that the use of electronic decision aids increases patient involvement in medical decision making. This may be because of weak implementation of the study protocol and a low response rate. | Decision Making | Humans | Internet | Netherlands | OK |
| Brian P. | Butz | Natan | Bar-Chama | Richard | Stock | Journal of Medical Internet Research | 2012-00-00 2012 | BACKGROUND: Prostate cancer is the most common cancer affecting men in the United States. Management options for localized disease exist, yet an evidence-based criterion standard for treatment still has to emerge. Although 5-year survival rates approach 98%, all treatment options carry the possibility for significant side effects, such as erectile dysfunction and urinary incontinence. It is therefore recommended that patients be actively involved in the treatment decision process. We have developed an Internet/CD-ROM-based multimedia Prostate Interactive Educational System (PIES) to enhance patients' treatment decision making. PIES virtually mirrors a health center to provide patients with information about prostate cancer and its treatment through an intuitive interface, using videos, animations, graphics, and texts. OBJECTIVES: (1) To examine the acceptability and feasibility of the PIES intervention and to report preliminary outcomes of the program in a pilot trial among patients with a new prostate cancer diagnosis, and (2) to explore the potential impact of tailoring PIES treatment information to participants' information-seeking styles on study outcomes. METHODS: Participants (n = 72) were patients with newly diagnosed localized prostate cancer who had not made a treatment decision. Patients were randomly assigned to 3 experimental conditions: (1) control condition (providing information through standard National Cancer Institute brochures; 26%), and PIES (2) with tailoring (43%) and (3) without tailoring to a patient's information-seeking style (31%). Questionnaires were administrated before (t1) and immediately after the intervention (t2). Measurements include evaluation and acceptability of the PIES intervention, monitoring/blunting information-seeking style, psychological distress, and decision-related variables (eg, decisional confidence, feeling informed about prostate cancer and treatment, and treatment preference). RESULTS: The PIES program was well accepted by patients and did not interfere with the clinical routine. About 79% of eligible patients (72/91) completed the pre- and post-PIES intervention assessments. Patients in the PIES groups compared with those in the control condition were significantly more likely to report higher levels of confidence in their treatment choices, higher levels of helpfulness of the information they received in making a treatment decision, and that the information they received was emotionally reassuring. Patients in the PIES groups compared with those in the control condition were significantly less likely to need more information about treatment options, were less anxious about their treatment choices, and thought the information they received was clear (P < .05). Tailoring PIES information to information-seeking style was not related to decision-making variables. CONCLUSIONS: This pilot study confirms that the implementation of PIES within a clinical practice is feasible and acceptable to patients with a recent diagnosis of prostate cancer. PIES improved key decision-making process variables and reduced the emotional impact of a difficult medical decision. | Male | Decision Making | Humans | Patient Participation | OK |
| Jan E. A. M. | van Bergen | Elfi E. H. G. | Brouwers | Han J. S. A. | Fennema | Sexually Transmitted Diseases | 2011-06-00 Jun 2011 | BACKGROUND: The study assessed the acceptability of internet-based Chlamydia screening using home-testing kits among 16- to 29-year-old participants and nonparticipants in the first year of a Chlamydia Screening Implementation program in the Netherlands. METHODS: Questionnaire surveys were administered to randomly selected participants (acceptability survey) and nonparticipants (nonresponse survey) in 3 regions of the Netherlands where screening was offered. Participants received email invitations to an online survey; nonparticipants received postal questionnaires. Both surveys enquired into opinions on the screening design, reasons for (non-) participation and future willingness to be tested. RESULTS: The response rate was 63% (3499/5569) in the acceptability survey and 15% (2053/13,724) in the nonresponse survey. Primary motivation for participating in the screening was "for my health" (63%). The main reason for nonresponse given by sexually active nonparticipants was "no perceived risk of infection" (40%). Only 2% reported nonparticipation due to no internet access. Participants found the internet (93%) and home-testing (97%) advantages of the program, regardless of test results. Two-thirds of participants would test again, 92% via the screening program. Half of nonparticipants were appreciative of the program design, while about 1 in 5 did not like internet usage, home-testing, or posting samples. CONCLUSIONS: The screening method was highly acceptable to participants. Nonparticipants in this survey were generally appreciative of the program design. Both groups made informed choices about participation and surveyed low-risk nonparticipants accurately perceived their low-risk status. Although many nonparticipants were not reached by the nonresponse survey, current insights on acceptability and nonresponse are undoubtedly valuable for evaluation of the current program. | Male | Humans | Patient Participation | Questionnaires | OK |
| Long-Lung | Tsao | Tzung-Yi | Tsai |  |  | BMC neurology | 2013-00-00 2013 | BACKGROUND: Modern medicine has increased the survival rate for stroke patients; however, the patient's psychosocial adaptation after stroke onset may be related to the clinical outcomes. This study aimed to investigate patients' acceptance of disability (AOD) and its predictors in stroke patients. METHODS: This cross-sectional study used a purposive sampling method to recruit 175 stroke patients from a hospital in southern Taiwan. A structured questionnaire gathered data on respondent demographics and disease characteristics, and included the Chinese version of the AOD Scale-Revised. Factors associated with AOD were examined by a multiple linear regression analysis. RESULTS: The mean AOD score was 71.72, which indicated a lower level of disease acceptance (range, 32-128). Our findings showed that patients who reported no religious beliefs, shorter disease duration, recurrent stroke episodes, and poorer physical functioning also reported lower levels of disability acceptance. These factors accounted for 38.2% of the variance in AOD among participants. CONCLUSIONS: The findings are beneficial to healthcare providers by identifying those stroke patients with predisposition of having lower disability acceptance, which could then facilitate the provision of appropriate rehabilitation interventions within six months after the diagnosis of stroke to support their adaptation process. | Male | Humans | Patient Participation | Questionnaires | OK |
| S. | Rozenberg | P. | Barlow | M. | Hainaut | Maturitas | 2010-01-00 Jan 2010 | OBJECTIVE: To assess the adequacy of a multidisciplinary approach providing information to couples affected by HIV before ART. DESIGN: Prospective observational study. SETTING: RT centre and infectious disease clinic, public university hospital. PATIENTS: 50 couples with at least one HIV-infected partner. INTERVENTIONS: Multidisciplinary approach towards ART by various intervening physicians (specialist in fertility, infectious diseases, paediatrics, obstetrics, psychiatry). MAIN OUTCOME MEASURED: We analysed specifically the cases in which the staff did not accept and the patient's compliance to the counselling procedures. RESULTS: Among the 150 couples, 30 did not complete the procedure and were lost to follow-up. The remaining 120 couples were evaluated: 89 couples were accepted, 5 were temporarily refused and 26 were refused definitively. The major reasons for refusal were medical reasons (n=13). CONCLUSION: Because of the high refusal rate and the drop of rate, a multidisciplinary approach is mandatory before initiating ART in seropositive couples. | Male | Humans | Patient Participation | Questionnaires | OK |
| Tanja | Krones | Norbert | Donner-Banzhoff | |  | Implementation science: IS | 2011-00-00 2011 | BACKGROUND: Decision aids based on the philosophy of shared decision making are designed to help patients make informed choices among diagnostic or treatment options by delivering evidence-based information on options and outcomes. A patient decision aid can be regarded as a complex intervention because it consists of several presumably relevant components. Decision aids have rarely been field tested to assess patients' and physicians' attitudes towards them. It is also unclear what effect decision aids have on the adherence to chosen options. METHODS: The electronic library of decision aids (arriba-lib) to be used within the clinical encounter has a modular structure and contains evidence-based decision aids for the following topics: cardiovascular prevention, atrial fibrillation, coronary heart disease, oral antidiabetics, conventional and intensified insulin therapy, and unipolar depression. We conducted an evaluation study in which 29 primary care physicians included 192 patients. After the consultation, patients filled in questionnaires and were interviewed via telephone two months later. We used generalised estimation equations to measure associations within patient variables and traditional crosstab analyses. RESULTS: Patients were highly satisfied with arriba-lib and the process of shared decision making. Two-thirds of patients reached in the telephone interview wanted to be counselled again with arriba-lib. There was a high congruence between preferred and perceived decision making. Of those patients reached in the telephone interview, 80.7% said that they implemented the decision, independent of gender and education. Elderly patients were more likely to say that they implemented the decision. CONCLUSIONS: Shared decision making with our multi-modular electronic library of decision aids (arriba-lib) was accepted by a high number of patients. It has positive associations to general aspects of decision making in patients. It can be used for patient groups with a wide range of individual characteristics. | Male | Decision Making | Decision Support Techniques | Humans | OK |
|  |  |  |  |  |  | Die Rehabilitation | 2014-04-00 Apr 2014 | BACKGROUND AND GOALS: In a multicenter study, patients of the German statutory insurance were encouraged to deal with their own rehabilitation goals using a questionnaire sent in advance of rehabilitation. How patients and physicians assess the benefits and practicability of this method should, in particular, be examined. METHODS: In a randomized controlled study a questionnaire, based on the preparation booklet of the DRV Bund, was sent by the German statutory insurance to 2782 rehabilitation patients (n(ortho)=1406, n(onco)714, n(psy)=662). For the intervention group, the questionnaire included, in addition to general information about rehabilitation goals, free text fields for the formulation of own goals. Patients were asked to bring the completed questionnaire to the admission interview. At the end of rehabilitation, satisfaction with participation in the goal-setting process and perceived usefulness of information on rehabilitation goals were captured. The doctors/psychologists responded to standardized items regarding various aspects of utility and practicability of the questionnaire. RESULTS: 34% of the targeted rehabilitation patients brought the completed questionnaire to the admission interview. For most of the rehabilitation patients goal setting is important; 31.4% reported the information obtained to rehabilitation goals to be very, and 62.9% particularly helpful. For practitioners, the questionnaire is likely to orient rehabilitation patients to rehabilitation goals: they reported that 56.6% of the patients reflected about their goals and that 50% were more familiar with the subject. Physicians rated the handling and integration of the questionnaire into the interview, its format and layout as well as its benefits more positively than the surveyed psychologists. These were more optimistic about the ability of rehabilitation patients to answer the questionnaire (62.5%, physicians 47.2%) and define own goals (77.8%, physicians 41.7%). CONCLUSIONS: The applied questionnaire has been well accepted by the majority of rehabilitation patients and practitioners as well as was judged to be practical and useful in important aspects. A comprehensive implementation of the questionnaire proves to be promising, particularly in the context of oncological and orthopedic rehabilitation. To increase the return of the questionnaire, alternative delivery options should be explored in future. Against the background that rehabilitation patients and practitioners of psychosomatic medicine perceived the questionnaire as less helpful, it is also prudent to investigate whether indication-specific implementation strategies would be advantageous. | Male | Humans | Patient Participation | Patient Satisfaction | OK |
| Jason J. | Saleem | Scott | Russell | Joshua J. | Robinson | Health Informatics Journal | 2014-12-00 Dec 2014 | This article reports redesign strategies identified to create a Web-based user-interface for the Self-management TO Prevent (STOP) Stroke Tool. Members of a Stroke Quality Improvement Network (N = 12) viewed a visualization video of a proposed prototype and provided feedback on implementation barriers/facilitators. Stroke-care providers (N = 10) tested the Web-based prototype in think-aloud sessions of simulated clinic visits. Participants' dialogues were coded into themes. Access to comprehensive information and the automated features/systematized processes were the primary accessibility and usability facilitator themes. The need for training, time to complete the tool, and computer-centric care were identified as possible usability barriers. Patient accountability, reminders for best practice, goal-focused care, and communication/counseling themes indicate that the STOP Stroke Tool supports the paradigm of patient-centered care. The STOP Stroke Tool was found to prompt clinicians on secondary stroke-prevention clinical-practice guidelines, facilitate comprehensive documentation of evidence-based care, and support clinicians in providing patient-centered care through the shared decision-making process that occurred while using the action-planning/goal-setting feature of the tool. | | | | | OK |
| Jennifer | Mongoven | Margaret | McDonald | Ernesto | Henriquez | The Journal of Ambulatory Care Management | 2011-09-00 2011 Jul-Sep | Successful chronic care ideally involves patient engagement, but little is known about chronically ill older adults' ability to self-manage their health. This study examines activation among hypertensive patients older than 65 years. Almost 60% of participants scored in the bottom half of the activation scale; only 8% scored at the highest level. Higher activation was associated with higher self-ratings of health, health literacy, and receipt of patient-centered care, shorter lengths of stay, and lower depression and hearing impairment levels. Effective self-management support for chronically ill elders will likely require varied strategies and may need to address depression, health literacy, and/or hearing impairments. | Male | Humans | Patient Participation | Questionnaires | OK |
|  |  |  |  |  |  | Journal of Palliative Medicine | 2011-07-00 juillet 2011 | A 69-year-old female was receiving renal replacement therapy (RRT) for acute renal failure (ARF) in an intensive care unit (ICU). Consultation was requested from the palliative medicine service to facilitate a shared decision-making process regarding goals of care. Clinician responsibility in shared decision making includes the formulation and expression of a prognostic assessment providing the necessary perspective for a spokesperson to match patient values with treatment options. For this patient, ARF requiring RRT in the ICU was used as a focal point for preparing a prognostic assessment. A prognostic assessment should include the outcomes of most importance to a discussion of goals of care: mortality risk and survivor functional status, in this case including renal recovery. A systematic review of the literature was conducted to document published data regarding these outcomes for adult patients receiving RRT for ARF in the ICU. Forty-one studies met the inclusion criteria. The combined mean values for short-term mortality, long-term mortality, renal-function recovery of short-term survivors, and renal-function recovery of long-term survivors were 51.7%, 68.6%, 82.0%, and 88.4%, respectively. This case example illustrates a process for formulating and expressing a prognostic assessment for an ICU patient requiring RRT for ARF. Data from the literature review provide baseline information that requires adjustment to reflect specific patient circumstances. The nature of the acute primary process, comorbidities, and severity of illness are key modifiers. Finally, the prognostic assessment is expressed during a family meeting using recommended principles of communication. (PsycINFO Database Record (c) 2012 APA, all rights reserved). (journal abstract) | Shared decision making | Decision Making | acute renal failure | renal replacement therapy | Doublon |
|  |  |  |  |  |  | Journal of Palliative Medicine | 2011-07-00 Jul 2011 | A 69-year-old female was receiving renal replacement therapy (RRT) for acute renal failure (ARF) in an intensive care unit (ICU). Consultation was requested from the palliative medicine service to facilitate a shared decision-making process regarding goals of care. Clinician responsibility in shared decision making includes the formulation and expression of a prognostic assessment providing the necessary perspective for a spokesperson to match patient values with treatment options. For this patient, ARF requiring RRT in the ICU was used as a focal point for preparing a prognostic assessment. A prognostic assessment should include the outcomes of most importance to a discussion of goals of care: mortality risk and survivor functional status, in this case including renal recovery. A systematic review of the literature was conducted to document published data regarding these outcomes for adult patients receiving RRT for ARF in the ICU. Forty-one studies met the inclusion criteria. The combined mean values for short-term mortality, long-term mortality, renal-function recovery of short-term survivors, and renal-function recovery of long-term survivors were 51.7%, 68.6%, 82.0%, and 88.4%, respectively. This case example illustrates a process for formulating and expressing a prognostic assessment for an ICU patient requiring RRT for ARF. Data from the literature review provide baseline information that requires adjustment to reflect specific patient circumstances. The nature of the acute primary process, comorbidities, and severity of illness are key modifiers. Finally, the prognostic assessment is expressed during a family meeting using recommended principles of communication. | Humans | Patient Participation | Female | Aged | OK |
| Peter | Vedsted |  |  |  |  | European Journal of Public Health | 2012-02-00 Feb 2012 | BACKGROUND: The Patient Assessment of Chronic Illness Care (PACIC) 20-item questionnaire measures how chronic care patients perceive their involvement in care. We aimed to adapt the measure into Danish and to assess data quality, internal consistency and the proposed factorial structure. METHODS: The PACIC was translated by a standardised forward-backward procedure, and filled in by 560 patients receiving type 2 diabetes care. Data quality was assessed by mean, median, item response, missing values, floor and ceiling effects, internal consistency (Cronbach's α and average inter-item correlation), item-rest correlations and factorial structure was assessed by confirmatory factor analysis (CFA). RESULTS: The item response was high (missing answers: 0.5-2.9%). Floor effect was 2.7-69.2%, above 15% for 17 items. Ceiling effect was 4.0-40.4%, above 15% for 12 items. The subscales had average inter-item correlations over 0.30 and CFA showed high factor loadings (range 0.67-0.77). All had α over 0.7 and included items with both high and low loadings. The CFA model fit was good for two indices out of six (TLI and SRMR). CONCLUSIONS: Danish PACIC is now available and validated in primary care in a type 2 diabetes population. The psychometric properties were satisfactory apart from ceiling and floor effects. We endorse the proposed five scale structure. All the subscales showed good model fit, and may be used for separate sum scores. | Male | Humans | Patient Participation | Patient Satisfaction | OK |
| Jamil | Jivraj |  |  |  |  | International Journal of Technology Assessment in Health Care | 2014-07-00 Jul 2014 | BACKGROUND: Adaptive approaches to the introduction of drugs and medical devices involve the use of an evolving evidence base rather than conventional single-point-in-time evaluations as a proposed means to promote patient access to innovation, reduce clinical uncertainty, ensure effectiveness, and improve the health technology development process. METHODS: This report summarizes a Health Technology Assessment International (HTAi) Policy Forum discussion, drawing on presentations from invited experts, discussions among attendees about real-world case examples, and background paper. RESULTS: For adaptive approaches to be understood, accepted, and implemented, the Forum identified several key issues that must be addressed. These include the need to define the goals of and to set priorities for adaptive approaches; to examine evidence collection approaches; to clarify the roles and responsibilities of stakeholders; to understand the implications of adaptive approaches on current legal and ethical standards; to determine costs of such approaches and how they will be met; and to identify differences in applying adaptive approaches to drugs versus medical devices. The Forum also explored the different implications of adaptive approaches for various stakeholders, including patients, regulators, HTA/coverage bodies, health systems, clinicians, and industry. CONCLUSIONS: A key outcome of the meeting was a clearer understanding of the opportunities and challenges adaptive approaches present. Furthermore, the Forum brought to light the critical importance of recognizing and including a full range of stakeholders as contributors to a shared decision-making model implicit in adaptive pathways in future discussions on, and implementation of, adaptive approaches. | | | | | OK |
| Monique C. M. | Baas-Thijssen | Corrie A. M. | Marijnen | Anne M. | Stiggelbout | Patient Education and Counseling | 2010-02-00 Feb 2010 | OBJECTIVE: Patient values are not routinely assessed in clinical practice. Adaptive Conjoint Analysis (ACA) is increasingly applied in studies assessing treatment preferences, and could provide a means to routinely assess individual patients' treatment preferences. METHODS: An ACA-questionnaire was administered three times (7-10 days apart) to 98 long-term rectal cancer survivors either on a portable computer or through internet, to assess whether (a) responses differ according to administration mode, (b) relative importances of rectal cancer treatment outcomes (survival, local control, incontinence, sexual problems) consolidate over time, (c) ACA-outcomes are sufficiently reliable (ICC) for use in individual decision-making. We also evaluated patients' acceptance of ACA. RESULTS: Mode did not affect ACA-completion or evaluation. Importance scores did not consolidate over time. ICCs were poor for sexual problems and fair for the other outcomes, and were at least equal or higher from first to second retest. Most participants valued completing the ACA-questionnaire and learning their results. CONCLUSION: Values did not show consolidation over time. ACA-derived preferences should not determine which treatment patients should choose. PRACTICE IMPLICATIONS: Findings extend ACA-validation studies to the health care setting and suggest that ACA-questionnaires might be appreciated as adjuncts to treatment decision-making in newly diagnosed patients. | Male | Decision Support Techniques | Humans | Patient Participation | OK |
| Patricia S. | Goode | Alayne D. | Markland | Kimberly | Kenton | Physical Therapy | 2010-10-00 Oct 2010 | BACKGROUND: Behavioral intervention outcomes for urinary incontinence (UI) depend on active patient participation. OBJECTIVE: The purpose of this study was to describe adherence to behavioral interventions (pelvic-floor muscle [PFM] exercises, UI prevention strategies, and delayed voiding), patient-perceived exercise barriers, and predictors of exercise adherence in women with urge-predominant UI. DESIGN: This was a prospectively planned secondary data analysis from a 2-stage, multicenter, randomized clinical trial. PATIENTS AND INTERVENTION: Three hundred seven women with urge-predominant UI were randomly assigned to receive either 10 weeks of drug therapy only or 10 weeks of drug therapy combined with a behavioral intervention for UI. One hundred fifty-four participants who received the combined intervention were included in this analysis. MEASUREMENTS: Pelvic-floor muscle exercise adherence and exercise barriers were assessed during the intervention phase and 1 year afterward. Adherence to UI prevention strategies and delayed voiding were assessed during the intervention only. RESULTS: During intervention, 81% of women exercised at least 5 to 6 days per week, and 87% performed at least 30 PFM contractions per day. Ninety-two percent of the women used the urge suppression strategy successfully. At the 12-month follow-up, only 32% of the women exercised at least 5 to 6 days per week, and 56% performed 15 or more PFM contractions on the days they exercised. The most persistent PFM exercise barriers were difficulty remembering to exercise and finding time to exercise. Similarly, difficulty finding time to exercise persisted as a predictor of PFM exercise adherence over time. LIMITATIONS: Co-administration of medication for UI may have influenced adherence. CONCLUSIONS: Most women adhered to exercise during supervised intervention; however, adherence declined over the long term. Interventions to help women remember to exercise and to integrate PFM exercises and UI prevention strategies into daily life may be useful to promote long-term adherence. | Humans | Questionnaires | United States | Female | OK |
| Angela C. | Leek | Sabahat | Hizlan | Susan R. | Rheingold | Pediatric Blood & Cancer | 2013-05-00 May 2013 | BACKGROUND: The aim of this study was to examine adolescent patients' perspectives on their understanding and decision making about a pediatric phase I cancer study. PROCEDURE: Participants included adolescents ages 14-21 years with cancer (N = 20), all of whom attended a phase I study consent conference. Participants responded to closed- and open-ended questions on a verbally administered structured interview, which assessed aspects of understanding and decision making about the phase I study. RESULTS: All participants decided to enroll in the phase I study. The majority of participants understood that participation was voluntary, entailed risks, and that they could withdraw. Most also believed that participation in the phase I study would increase the length of their lives. The most frequent reasons for enrolling were positive clinical benefit, needing an option, impact on quality of life, and few side effects or fewer than those of current or past treatments. Eighty-five percent of participants reported that they themselves made the final decision about enrollment in the phase I study. CONCLUSIONS: Most participants hoped or expected that the phase I study would provide a direct benefit (increased survival time or cure) and reported that they themselves were the final decision-maker about enrollment. Clinicians may underestimate the role of adolescents, especially if they believe that parents typically make such decisions. Future research should assess the actual participation of children and adolescents during the informed consent process and explore the role of hope in their decision making about phase I studies. | Male | Decision Making | Humans | Patient Participation | OK |
| Patrick Kim Cheng | Low | Zaw | Wint | Mustafa Z. | Younis | Journal of Health Care Finance | 2013-00-00 2013 | The aim of this article is to present an e-health model that embeds empowerment and social network intervention that may extend the role of customers in health care settings. A 25-item Likert-type survey instrument was specifically developed for this study and administered to a sample of 108 participants in Indonesia from October to November 2012. The data were analyzed to provide ideas on how to move forward with the e-health initiative as a means to improve e-health services. The survey revealed that there is a high demand for customers' empowerment and involvement in social networks to improve their health literacy and customer satisfaction. Regardless of the limitations of the study, the participants have responded with great support for the abilities of the prototype systems drawn from the survey. The survey results were used as requirements to develop a system prototype that incorporates the expectations of the people. The prototype (namely Clinic 2.0) was derived from the model and confirmed from the survey. Participants were selected to use the system for three months, after which we measured its impact towards their health literacy and customer satisfaction. The results show that the system intervention through Clinic 2.0 leads to a high level of customer satisfaction and health literacy. | Male | Humans | Patient Participation | Questionnaires | OK |
| Joanne | Tyler | Joan | Schreiner | Mary Jo | Deering | Journal of Medical Internet Research | 2012-00-00 2012 | BACKGROUND: Less than 5% of breast cancer patients participate in clinical trials. To increase patients' awareness and access to trials, we created BreastCancerTrials.org, a clinical trial matching website. BreastCancerTrials.org matched patients to trials based on their self-reported breast cancer history. It also provided a messaging platform through which patients could self-refer themselves to participating research sites. OBJECTIVE: To assess adoption by research sites, acceptability to patients, and patients' accuracy in providing information to BreastCancerTrials.org. METHODS: We approached 13 research sites in Northern California to list their trials on BreastCancerTrials.org. For adoption, we examined the willingness of contacted research sites to collaborate with BreastCancerTrials.org. For acceptability, we analyzed usage statistics of visitors who completed the BreastCancerTrials.org health history questionnaire in the first 14 months after launch and surveyed users who visited the website during its first year about their experience. For accuracy, we compared the self-reported health history of 20 patients against their medical records. The health history questionnaire was divided into four sections: About Me, personal information including date of birth and sex; My Health as of Today, current status including cancer stage, menopausal status, and sites with evidence of disease; My Cancer, diagnostic information such as hormone and human epidermal growth factor receptor 2 status; and My Treatment, an itemized record of past treatment including responses to therapy. RESULTS: A total of 12 sites contributed 55 trials. Regarding acceptability, 733 visitors registered on the website; 428 reported their health history; and 407 matched to at least one trial. Of 375 patients who were sent a survey, 75 responded (20%); 23 of the 75 (31%) contacted a research site, 12 of the 23 (52%) were eligible for a trial, and 5 of the 12 (42%) reported enrolling. As for accuracy, 20 clinic visitors reported 1456 health history items, 1324 of which matched their clinic record (90.93%). CONCLUSIONS: BreastCancerTrials.org was adopted by research sites. Patients found it acceptable and were able to provide accurate information for trial matching. Based on our findings, we launched an upgraded version of BreastCancerTrials.org as a national service in October 2008. | Humans | Patient Participation | Questionnaires | Female | OK |
| Joseph B. | Straton | Jennifer M. | Kapo |  |  | Journal of Palliative Medicine | 2010-05-00 May 2010 | PURPOSE: The purpose of this study was to assess patient participation in advance care planning (ACP) and the decision to enroll in hospice. METHODS: One hundred sixty-five family members of patients who died in hospice between January 2004 and September 2004 returned an anonymous survey (165/380; 43% response rate). RESULTS: Forty-nine percent of family members reported that the patient was not involved in the hospice enrollment decision. The majority of respondents (78%) reported one or more people helped make the decision to enroll in hospice. For patients reported as being involved in the decision to enroll in hospice (either independently or in a shared capacity) they were more likely to have cancer (odds ratio [OR] = 2.3, p = 0.02), die at home (OR = 3.3, p = 0.006), have a length of stay in hospice greater than 7 days (OR = 2.1, p = 0.03), and less likely to have dementia (OR = 0.43, p = 0.001). White respondents were more likely to report having ACP discussions with the patient about: feeding tubes (OR = 4.7; p = 0.001), cardiopulmonary resuscitation (CPR; OR = 3.9; p = 0.002), or mechanical ventilation (OR = 2.7; p = 0.02) than non-white respondents. White respondents were more likely than non-white respondents to report that the patient had a written advance directive (OR = 4.2, p = 0.001). DISCUSSION: These data indicate that some patients are not actively involved in the decision to enroll in hospice and that others, often physicians and family members, are making these decisions for the patient collaboratively. These data support the need for early education and interventions that assist patients and families in discussing ACP preferences and the need for greater understanding of how involved patients want to be with the decision to enroll in hospice. | Male | Decision Making | Humans | Patient Participation | OK |
| Elizabeth A. | Jacobs | Torsten B. | Neilands | Robert | Weech-Maldonado | Medical Care | 2012-09-00 Sep 2012 | BACKGROUND: Providing culturally competent care shows promise as a mechanism to reduce health care inequalities. Until the recent development of the Consumer Assessment of Healthcare Providers and Systems Cultural Competency Item Set (CAHPS-CC), no measures capturing patient-level experiences with culturally competent care have been suitable for broad-scale administration. METHODS: We performed confirmatory factor analysis and internal consistency reliability analysis of CAHPS-CC among patients with type 2 diabetes (n=600) receiving primary care in safety-net clinics. CAHPS-CC domains were also correlated with global physician ratings. RESULTS: A 7-factor model demonstrated satisfactory fit (χ²₂₃₁=484.34, P<0.0001) with significant factor loadings at P<0.05. Three domains showed excellent reliability-Doctor Communication-Positive Behaviors (α=0.82), Trust (α=0.77), and Doctor Communication-Health Promotion (α=0.72). Four domains showed inadequate reliability either among Spanish speakers or overall (overall reliabilities listed): Doctor Communication-Negative Behaviors (α=0.54), Equitable Treatment (α=0.69), Doctor Communication-Alternative Medicine (α=0.52), and Shared Decision-Making (α=0.51). CAHPS-CC domains were positively and significantly correlated with global physician rating. CONCLUSIONS: Select CAHPS-CC domains are suitable for broad-scale administration among safety-net patients. Those domains may be used to target quality-improvement efforts focused on providing culturally competent care in safety-net settings. | Male | Humans | Patient Satisfaction | Physician-Patient Relations | OK |
| Allen W. | Heinemann |  |  |  |  | Archives of Physical Medicine and Rehabilitation | 2011-04-00 Apr 2011 | The authors of 3 articles in this issue have collaborated in an effort to advance the conceptualization and measurement of participation. These articles offer (1) a new tool for measuring participation, the Participation Assessment with Recombined Tools-Objective (PART-O), which combines items from widely used instruments in traumatic brain injury rehabilitation research; (2) 2 methods of scoring 17 items of PART-O, assessing relatively objective social role performance and yielding 3 subscale scores, as well as 2 alternative total scores (including 1 incorporating the concept of balance among types of participation), and (3) 19 enfranchisement items assessing the degree to which people with disability perceive they have the freedom to engage in social roles of their choosing while being accepted and valued by others. | Humans | Patient Participation | Questionnaires | Psychometrics | OK |
| Jennifer R. | Byrd | Christopher L. | Edwards | Margaret | Pericak-Vance | Journal of public health management and practice: JPHMP | 2013-04-00 2013 Mar-Apr | OBJECTIVE: To elucidate factors that influence African American willingness to participate in health-related research studies. METHODS: The African American Alzheimer disease research study group at North Carolina A&T State University designed an in-person questionnaire and surveyed more than 700 African American adults on their willingness to participate in health-related research studies. The questionnaire was distributed and collected in a nonclinical setting during the years 2008 and 2009. This study was approved by the North Carolina A&T State University Institutional Review Board. RESULTS: Of the 733 valid respondents, 16% had previously participated in a health-related research study. Of these, more than 90% were willing to participate again in future research studies. Of the 614 who had never participated in a research study, more than 70% expressed willingness to participate. The majority (75%) of experienced research study participants (RSP) were older than 40 years compared with 45% of non-research study participants. Experienced research participants were also twice as likely to have a college degree compared with non-research study participants. Seventy-three percent of non-research study participants were willing to participate in research studies in the future. The factors that were probable impediments to participation included lack of time and trust. Men with knowledge of the Tuskegee Syphilis Study were 50% less likely to be willing to participate compared with those who had not heard of Tuskegee Syphilis Study. CONCLUSIONS: African Americans are willing to participate in health-related research studies. Several factors such as the appropriate incentives, community trust building, outreach, and community partnership creation are necessary for engaging minority participants. Incorporating factors that target African American enrollment in research design and implementation, such as increased training of minority health ambassadors and African American researchers and public health specialists, are needed to better engage minorities across generations, in research. | Male | Humans | Patient Participation | Questionnaires | OK |
| Aubrey R. | Turner | Jianfeng | Xu | Nancy E. | Avis | Patient Education and Counseling | 2013-01-00 Jan 2013 | OBJECTIVE: To examine African-American prostate cancer (PCa) survivors' involvement in treatment decision-making (TDM), and examine the association between TDM and quality of life (QOL), using secondary data. METHODS: African-American PCa survivors (181) were recruited from the North Carolina Central Cancer Registry. Participants completed a cross-sectional survey that asked about their chosen cancer treatment, TDM factors, and PCa-specific QOL (using the Expanded Prostate Cancer Index Composite--EPIC). Multivariate analysis of covariance was conducted to determine the association between TDM and QOL, controlling for confounders. RESULTS: Most men reported being active (44.2%) or collaborative (38.1%) in TDM, while 14.4% preferred a passive role. Adjusting for marital status, education and treatment, passive patients reported somewhat better QOL compared to active patients in the following QOL domains: urinary summary (p=0.04), urinary function (p=0.01), and urinary incontinence (p=0.03). CONCLUSION: Most African-American PCa survivors preferred to be, and were, actively or collaboratively involved in TDM. However, those who preferred a passive role reported better PCa-specific QOL for the urinary domain compared to others. PRACTICE IMPLICATIONS: It is important to assess patients' TDM preference. Patients' QOL may differ by their TDM role, such that active patients may be more bothered by treatment side effects than other patients. | Male | Decision Making | Humans | Patient Participation | OK |
| Monica W. | Parker | Joyce E. | Balls-Berry | Michele Y. | Halyard | Journal of Women's Health (2002) | 2014-08-00 Aug 2014 | OBJECTIVE: To examine perceptions and attitudes toward health-related research participation among professional African American women. METHODS: Participants were members of an African American women's service organization, The Links, Incorporated. Data were collected via self-administered questionnaires at The Links, Incorporated 2012 National Assembly. Sociodemographics, prior research experience, intention to participate (ITP), willingness to participate (WTP) in a variety of research studies and attitudes about research participation were measured. RESULTS: A total of 381 surveys were analyzed. A majority of respondents were married (66%), employed (69%), and college educated (96%). Median age was 59; 38% reported prior research participation. Overall, 78% agreed with the statement, "Participation in research will mean better care," 24% agreed "Participation in research is risky" and 3% agreed "Scientists cannot be trusted." Fifty-two percent agreed with the statement, "Research conducted in the U.S. is ethical." Mean ITP in research was 4.9±1.7 on a rating scale of 1 ("definitely no") to 7 ("definitely yes"). WTP was highest for an interview study and providing a blood sample, and lowest for clinical trial and medical record review. CONCLUSION: Attitudes toward research participation were generally favorable among professional African American women; many expressed WTP in a variety of research study types. | Humans | Patient Participation | Questionnaires | Young Adult | OK |
|  |  |  |  |  |  | AIDS care | 2013-00-00 2013 | Most studies of cultural competence in healthcare examine healthcare providers' definitions of cultural competence practices. This study is unique in that it examines the relationship between African-American patients' perceptions of the cultural competence of their HIV healthcare providers and the adherence of these patients to medical self-care and antiretroviral therapy (ART). This cross-sectional, exploratory, descriptive study was conducted at the Ruth Rothstein CORE Center in Chicago, Illinois. The sample consisted of 202 HIV-positive African-Americans who completed surveys during clinic visits. Multiple measures were used, including the Patient Assessments of Cultural Competency survey instrument developed by the Department of Health and Human Services Agency for Healthcare Research and Quality. Medical self-care was measured using the advice and instructions scale and the self-care symptom management for people living with HIV/AIDS categorical scale. ART adherence was measured using the Adherence Behaviors Self-Report and Adherence Self-Report scales. The data revealed many significant correlations between variables. The more patients believed that providers should integrate culture in HIV treatment; the better their reported health (F1,138=0.151, P=0.05) and the more they followed their provider's advice and instructions (medical self-care; F1,138=0.029, P=0.05). Participants who trusted their providers engaged in more medical self-care (F1,138=0.280, P=0.01). More shared treatment decisions were reported among participants who had higher levels of education (F1,127=0.337, P=0.05). Findings of this study indicate the need for increased attention to the role of cultural competence in HIV/AIDS care. Understanding patient perceptions of provider cultural competence has the potential to improve HIV treatment adherence and health outcomes. | Male | Humans | Patient Participation | Physician-Patient Relations | OK |
| Laurence Foix | L'Hélias | Jean | Bouyer | Elise | de La Rochebrochard | Early Human Development | 2011-10-00 Oct 2011 | BACKGROUND: The Ages and Stages Questionnaire (ASQ), completed by parents and caregivers, has been shown to be an accurate tool for screening children who need further developmental assessment. AIMS: To assess the feasibility of using the French Canadian translation of the ASQ in an epidemiological cohort of children from the French general population. STUDY DESIGN: Follow-up study by postal questionnaire at 12 and 36 months, using the ASQ. SUBJECTS: 339 French families recruited at the birth of their child in 2006 in two hospitals in the Paris suburbs. OUTCOME MEASURE: Response rates and French ASQ results at 12 and 36 months. The ASQ was scored as indicated in the manual. RESULTS: A high response rate of 79% was observed at the children's 1st and 3rd birthdays. Parents were enthusiastic about participating; half of them wrote comments on the questionnaires, most of them positive. Low scores at the 12-month assessment were associated with birth characteristics such as prematurity and transfer to the neonatology unit after birth, whereas at 36 months they tended to be associated with both birth and family socio-demographic characteristics. CONCLUSIONS: Use of the French ASQ in a research cohort appears feasible as response rates were high. Moreover, known links between child development measured by ASQ and birth and social characteristics were observed. However, further French studies are needed to understand differences observed in 12-month ASQ gross motor scores compared with US norms. For research purposes, further analysis of the ASQ in innovative, quantitative approaches, is needed. | Humans | Patient Participation | Questionnaires | Female | OK |
| Catherine C. | Vick | Christopher W. | Snyder | Rhiannon J. | Deierhoi | Surgery | 2011-09-00 Sep 2011 | BACKGROUND: Little information is available on agreement between patient-reported outcomes and data collected from medical chart abstraction (MCA) for recurring events. Recurring conditions pose a risk of misclassification, especially when events occur relatively close together in time. We examined agreement, predictors of agreement, and relative accuracy of patient survey and MCA for assessment of outcomes of incisional hernia repair (IHR). METHODS: Surveys to assess hernia outcomes were mailed to 1,124 living patients who underwent ≥ 1 IHR during 1998-2002 at 16 Veteran's Affairs Medical Center study sites. Patients were asked if they developed a recurrence or an infection at their hernia site. Physician-abstracted data from the medical chart were compared with patient response. Chi-square tests were used to assess significance. RESULTS: Of 487 (43.3%) individuals responding to the survey, 33 (6.8%) with >1 re-repair during 1998-2002 and 98 (20.5%) with a repair before the 1998-2002 period were excluded from the analysis. Although recurrence rates derived from self-reported data and data abstracted from the medical chart were similar (29.3% and 26.1%, respectively), overall concordance was low. Only 49% (n = 54) of self-reported recurrences were confirmed by data abstracted from the medical chart. In addition, 16 (8.3%) recurrences abstracted from the medical chart were not reported by the patient. Factors associated with discordance were high reported pain intensity (P = .02), poorer general health (P = .03), and poorer perceived repair results (P < .0001). CONCLUSION: Multiple recurrences and subsequent operations across the study period complicate the interpretation of both patient response and data abstracted from the medical chart when referring to a hernia repair. Further study on how best to assess treatment outcomes for recurring conditions is warranted. | Male | Humans | Patient Participation | United States | OK |
| Kristian | Tambs |  |  |  |  | Social Psychiatry and Psychiatric Epidemiology | 2012-05-00 May 2012 | PURPOSE: To investigate to what degree alcohol use and mental distress are associated with non-response in a population-based health study. METHODS: From 1995 to 1997, 91,488 persons were invited to take part in a health study at Nord-Trøndelag, Norway, and the response rate was 69.2%. Demographics were available for everyone. Survey answers from a previous survey were available for most of the participants and a majority of non-participants. In addition, the survey responses from spouses and children of the invitees were used to predict participation in the aforementioned study. Crude and adjusted ORs for a number of predictors, among these alcohol consumption and mental distress, are reported. RESULTS: Both heavy drinkers (OR = 1.27) and abstainers (OR = 1.64) had a higher probability of dropping out in comparison to people who usually do not drink. High levels of mental distress (OR = 1.84) also predicted attrition. CONCLUSION: Alcohol use and mental distress are moderately associated with non-response, though probably not a major cause, as controlling for other variables weakened the associations. Nevertheless, the moderate but clear underrepresentation at the crude level of people with high alcohol consumption, abstainers and people with poor mental health should be taken into consideration when interpreting results from health surveys. | Male | Humans | Patient Participation | Questionnaires | OK |
| Kathleen Kara | Fitzpatrick | Sarah | Forsberg | Linsey | Utzinger | European Eating Disorders Review: The Journal of the Eating Disorders Association | 2010-08-00 2010 Jul-Aug | The purpose of this study was to explore how individuals with anorexia nervosa (AN) engage in treatment and define recovery. A mixed methods design was used to triangulate the experience of 20 women with a history of AN. Interview data were analysed thematically to explore frequency of emergent themes and current eating disorder psychopathology was assessed using standardized self-report measures. Participants' mean age was 29.35 (SD = 12.11). Participants' scores were indicative of persistent psychopathology. Those with more involvement in treatment choice had better motivation to change and normalized eating. Participants' definition of recovery mapped on well to current research conceptualizations, though a substantial proportion of the group expressed some ambivalence around the concept. Results are interpreted in the context of self-determination theory of motivation and suggest that patients should be involved collaboratively in the formulation of shared goals and concepts of recovery in treatment settings. | Humans | Patient Participation | Questionnaires | Female | OK |
|  |  |  |  |  |  | Sport, Education and Society | 2013-01-00 janvier 2013 | Assessment is an integral feature of the work of all Higher Education institutions. It is, moreover, a process that makes transparent the power relations and hierarchical structures that are part of the fabric of academic fields and disciplines. The importance of assessment as a high stakes practice is increasingly being recognised, with a movement towards alternative practices driven by a broad range of forces, including perversely the corporatisation of the university. In recognition of these powerful trends towards alternative assessment and the marked absence of published research on this topic in physical education teacher education (PETE), the purpose of this study is to reveal some lessons learned through action research by a teacher educator as she sought to apply alternative, democratic assessment practices in a PETE programme. The study aimed to reflect on and deconstruct current educational assessment processes and principles and to investigate what happens in a pedagogical process that is not only based on teacher student agreement, dialogue, mutual respect, shared decision-making and personal responsibility, but also is accompanied by forms of assessment that are consistent with these democratic principles. We consider briefly some of the literature that has informed our thinking about alternative forms of assessment in Higher Education, with a particular focus on student participation in assessment. In this context we consider some strategies for participative assessment and some of the benefits, risks and challenges that have been proposed by researchers. We outline the methodology of the study, noting that this long-term action research project was carried out within the context of the Shared and Formative Assessment Network before presenting the main findings and discussion, and some lessons learned from reflecting on this study. We conclude that exploration and implementation of alternative forms of assessment is one crucially important site for challenging the process of corporatisation of the university and its undesirable and often unintended effects. (PsycINFO Database Record (c) 2013 APA, all rights reserved). (journal abstract) | Decision Making | Educational Measurement | action research | exploring risks | OK |
| E. Francis | Cook | Steven | Joffe |  |  | Clinical Trials (London, England) | 2011-10-00 Oct 2011 | BACKGROUND: Patients' motivations for participation in cancer clinical trials are incompletely understood. Even less is known about the factors that influence participants' motivations for enrolling in trials. PURPOSE: We studied the reasons why adult patients and parents of pediatric patients agree to participate in cancer trials. We focused on the role of altruism across all phases of trial. METHODS: We surveyed adult patients and parents of pediatric patients participating in phase I, II, or III cancer clinical trials. We asked respondents why they agreed to enroll, and examined correlates of altruistic motivation using univariate and multivariate analyses. RESULTS: Among 205 adults and 48 parents of children participating in cancer trials, 47% reported that altruistic motivations were 'very important' to their decisions to enroll. In multivariate analysis with phase III trial participants as the reference group, phase I trial participants least often identified altruism as a 'very important' motivation for enrolling (phase I OR 0.4, 95% CI (confidence interval) 0.2-0.8; phase II OR 0.9, 95% CI 0.5-1.5, overall P = 0.017). Thirty-three respondents (13%) reported being motivated primarily by altruism. In multivariate analysis, participants with poor prognoses-defined as an expected 5-year disease-free survival of ≤ 10%-reported altruism as their primary motivation less often than those with better prognoses (OR 0.2, 95% CI 0.1-0.5, P = 0.001). Altruistic motivations did not differ between adult patients and parents of pediatric participants. LIMITATIONS: The data are derived from related academic medical centers in one city, and the study sample reflects limited sociodemographic diversity, thereby limiting generalizability to other settings. CONCLUSIONS: Although cancer trial participants commonly report that altruism contributed to their decision to enroll, it is rarely their primary motivation for study participation. Participants in early phase trials and those with poor prognoses are least often motivated by altruism. | Male | Humans | Patient Participation | Questionnaires | OK |
| Kamil | Ugurbil | Noam | Harel |  |  | Neurosurgery | 2010-12-00 Dec 2010 | BACKGROUND: Deep brain stimulation (DBS) surgery is used for treating movement disorders, including Parkinson disease, essential tremor, and dystonia. Successful DBS surgery is critically dependent on precise placement of DBS electrodes into target structures. Frequently, DBS surgery relies on normalized atlas-derived diagrams that are superimposed on patient brain magnetic resonance imaging (MRI) scans, followed by microelectrode recording and macrostimulation to refine the ultimate electrode position. Microelectrode recording carries a risk of hemorrhage and requires active patient participation during surgery. OBJECTIVE: To enhance anatomic imaging for DBS surgery using high-field MRI with the ultimate goal of improving the accuracy of anatomic target selection. METHODS: Using a 7-T MRI scanner combined with an array of acquisition schemes using multiple image contrasts, we obtained high-resolution images of human deep nuclei in healthy subjects. RESULTS: Superior image resolution and contrast obtained at 7 T in vivo using susceptibility-weighted imaging dramatically improved anatomic delineation of DBS targets and allowed the identification of internal architecture within these targets. A patient-specific, 3-dimensional model of each target area was generated on the basis of the acquired images. CONCLUSION: Technical developments in MRI at 7 T have yielded improved anatomic resolution of deep brain structures, thereby holding the promise of improving anatomic-based targeting for DBS surgery. Future study is needed to validate this technique in improving the accuracy of targeting in DBS surgery. | Humans | Brain | Magnetic Resonance Imaging | Brain Mapping | OK |
| Keith | Reed | Amanda | Burls | Taunton R. | Southwood | Journal of Medical Ethics | 2011-09-00 Sep 2011 | BACKGROUND: Informed consent is a requirement for all research. It is not, however, clear how much information is sufficient to make an informed decision about participation in research. Information on an online questionnaire about childhood development was provided through an unfolding electronic participant sheet in three levels of information. METHODS: 552 participants, who completed the web-based survey, accessed and spent time reading the participant information sheet (PIS) between July 2008 and November 2009. The information behaviour of the participants was investigated. The first level contained less information than might be found on a standard PIS, the second level corresponded to a standard PIS, and the third contained more information than on a standard PIS. The actual time spent on reading the information provided in three incremental levels and the participants' evaluation of the information were calculated. RESULTS: 77% of the participants chose to access the first level of information, whereas 12% accessed the first two levels, 6% accessed all three levels of information and 23% participated without accessing information. The most accessed levels of information were those that corresponded to the average reading times. CONCLUSION: The brief information provided in the first level was sufficient for participants to make informed decisions, while a sizeable minority of the participants chose not to access any information at all. This study adds to the debate about how much information is required to make a decision about participation in research and the results may help inform the future development of information sheets by providing data on participants' actual needs when deciding about questionnaire surveys. | Humans | Patient Participation | Questionnaires | Internet | OK |
|  |  |  |  |  |  | Journal of Nursing Management | 2013-03-00 mars 2013 | Aim: The environmental scan aimed to deepen our understanding of the aged care work culture and to ascertain the readiness of the workers to advance towards team‐based quality care provision. Background: The workplace context was a high‐care unit within a large residential aged care facility. Methods: We used the Promoting Action on Research Implementation in Health Services (PARiHS) framework to assess workplace readiness via interviews, individual surveys and observation of practice. Results: A profile of current culture emerged as mutually supportive and task focused, but at the same time lacking corporate team features of shared decision‐making and feedback for practice improvement. However, latent within the frontline leaders and personal care staff, there was evidence of some embedded knowledge and capacity for corporate team performance. Conclusions: This study has validated an evidence‐based method for conducting environmental scanning in aged care, recommended before any major change is introduced. Implications for nursing management: Environmental scanning helps gauge workforce capacity and limitations; this information can enable managers to capitalize on identified cultural strengths to fortify change and avoid pitfalls of personal and collective vulnerabilities. (PsycINFO Database Record (c) 2014 APA, all rights reserved). (journal abstract) | Decision Making | Health Care Services | quality of care | environmental scan | Doublon |
|  |  |  |  |  |  | Journal of Nursing Management | 2013-03-00 Mar 2013 | AIM: The environmental scan aimed to deepen our understanding of the aged care work culture and to ascertain the readiness of the workers to advance towards team-based quality care provision. BACKGROUND: The workplace context was a high-care unit within a large residential aged care facility. METHODS: We used the Promoting Action on Research Implementation in Health Services (PARiHS) framework to assess workplace readiness via interviews, individual surveys and observation of practice. RESULTS: A profile of current culture emerged as mutually supportive and task focused, but at the same time lacking corporate team features of shared decision-making and feedback for practice improvement. However, latent within the frontline leaders and personal care staff, there was evidence of some embedded knowledge and capacity for corporate team performance. CONCLUSIONS: This study has validated an evidence-based method for conducting environmental scanning in aged care, recommended before any major change is introduced. IMPLICATIONS FOR NURSING MANAGEMENT: Environmental scanning helps gauge workforce capacity and limitations; this information can enable managers to capitalize on identified cultural strengths to fortify change and avoid pitfalls of personal and collective vulnerabilities. | Humans | Aged, 80 and over | Focus Groups | Models, Theoretical | OK |
| Nefyn | Williams | Larry | Raisanen | Graham | Moore | Journal of Epidemiology and Community Health | 2012-08-00 Aug 2012 | BACKGROUND: The Wales National Exercise Referral Scheme (NERS) is a 16-week programme including motivational interviewing, goal setting and relapse prevention. METHOD: A pragmatic randomised controlled trial with nested economic evaluation of 2160 inactive participants with coronary heart disease risk (CHD, 1559, 72%), mild to moderate depression, anxiety or stress (79, 4%) or both (522, 24%) randomised to receive (1) NERS or (2) normal care and brief written information. Outcome measures at 12 months included the 7-day physical activity recall, the hospital anxiety and depression scale. RESULTS: Ordinal regression identified increased physical activity among those randomised to NERS compared with those receiving normal care in all participants (OR 1.19, 95% CI 0.99 to 1.43), and among those referred for CHD only (OR 1.29, 95% CI 1.04 to 1.60). For those referred for mental health reason alone, or in combination with CHD, there were significantly lower levels of anxiety (-1.56, [corrected] 95% CI -2.75 to -0.38) and depression (-1.39, [corrected] 95% CI -2.60 to -0.18), but no effect on physical activity. The base-case incremental cost-effectiveness ratio was £12,111 per quality adjusted life year, falling to £9741 if participants were to contribute £2 per session. CONCLUSIONS: NERS was effective in increasing physical activity among those referred for CHD risk only. Among mental health referrals, NERS did not influence physical activity but was associated with reduced anxiety and depression. Effects were dependent on adherence. NERS is likely to be cost effective with respect to prevailing payer thresholds. Trial registration Current Controlled Trials ISRCTN47680448. | Male | Humans | Patient Participation | Female | OK |
| Daisy | Yoo | Rafae | Aziz | Hilaire J. | Thompson | Telemedicine Journal and E-Health: The Official Journal of the American Telemedicine Association | 2014-11-17 Nov 17, 2014 | Abstract Background: Smart home technologies provide a valuable resource to unobtrusively monitor health and wellness within an older adult population. However, the breadth and density of data available along with aging associated decreases in working memory, prospective memory, spatial cognition, and processing speed can make it challenging to comprehend for older adults. We developed visualizations of smart home health data integrated into a framework of wellness. We evaluated the visualizations through focus groups with older adults and identified recommendations to guide the future development of visualizations. Materials and Methods: We conducted four focus groups with older adult participants (n=31) at an independent retirement community. Participants were presented with three different visualizations from a wellness pilot study. A qualitative descriptive analysis was conducted to identify thematic content. Results: We identified three themes related to processing and application of visualizations: (1) values of visualizations for wellness assessment, (2) cognitive processing approaches to visualizations, and (3) integration of health data for visualization. In addition, the focus groups highlighted key design considerations of visualizations important towards supporting decision-making and evaluation assessments within integrated health displays. Conclusions: Participants found inherent value in having visualizations available to proactively engage with their healthcare provider. Integrating the visualizations into a wellness framework helped reduce the complexity of raw smart home data. There has been limited work on health visualizations from a consumer perspective, in particular for an older adult population. Creating appropriately designed visualizations is valuable towards promoting consumer involvement within the shared decision-making process of care. | | | | | OK |
| John | Benson |  |  |  |  | Patient Education and Counseling | 2012-04-00 avril 2012 | Objective: To examine the validity and utility of the Explanation and Planning Scale (EPSCALE) instrument, a widely used scale for teaching and assessment of explanation and planning skills used by clinicians during the medical interview. Methods: Data obtained across 4 OSCE stations during medical student final MB examinations. Exploratory factor analysis, using a single factor and two factor models (based on prior theory) and a six factor empirical model, suggested by parallel analysis. Participants: 124 medical students sitting final MB examinations at the University of Cambridge. Results: A single factor model represented a very poor fit. A two factor model with factors labelled ‘Explanation’ and ‘Planning’ produced an improved fit, but the best was seen with a six factor model, with factors which broadly corresponded to the domains of the Calgary–Cambridge guide. Conclusions: These factor models provide supportive evidence for the construct validity of EPSCALE. Practice implications: EPSCALE can justifiably be used in the assessment of shared-decision making skills. (PsycINFO Database Record (c) 2012 APA, all rights reserved). (journal abstract) | Decision Making | Psychometrics | test validity | medical students | Doublon |
| John | Benson |  |  |  |  | Patient Education and Counseling | 2012-04-00 Apr 2012 | OBJECTIVE: To examine the validity and utility of the Explanation and Planning Scale (EPSCALE) instrument, a widely used scale for teaching and assessment of explanation and planning skills used by clinicians during the medical interview. METHODS: Data obtained across 4 OSCE stations during medical student final MB examinations. Exploratory factor analysis, using a single factor and two factor models (based on prior theory) and a six factor empirical model, suggested by parallel analysis. PARTICIPANTS: 124 medical students sitting final MB examinations at the University of Cambridge. RESULTS: A single factor model represented a very poor fit. A two factor model with factors labelled 'Explanation' and 'Planning' produced an improved fit, but the best was seen with a six factor model, with factors which broadly corresponded to the domains of the Calgary-Cambridge guide. CONCLUSIONS: These factor models provide supportive evidence for the construct validity of EPSCALE. PRACTICE IMPLICATIONS: EPSCALE can justifiably be used in the assessment of shared-decision making skills. | Male | Humans | Physician-Patient Relations | Questionnaires | OK |
|  |  |  |  |  |  | Australian Occupational Therapy Journal | 2010-06-00 Jun 2010 | AIM: The purpose of this study was to illuminate the experiences of older adults' return to leisure activities, following rehabilitation, post-stroke. METHOD: A phenomenological approach was used to explore the experiences of re-engaging in leisure occupations post-stroke. In-depth interviews were conducted with five community-dwelling individuals (three men, two women) aged 68-74 years who had experienced a stroke in the past year. Data were analysed using thematic analysis. FINDINGS: Four themes emerged: (i) Re-engaging in leisure activities, (ii) acceptance of physical limitations post-stroke, (iii) gratitude for help and support and (iv) looking forward to the future. CONCLUSION: Implications revealed that post-stroke interventions need a more occupation-focussed approach, including return to leisure at an earlier stage of stroke rehabilitation. | Time Factors | Male | Humans | Patient Participation | OK |
| David | Morrison | Kirsty | Buising |  |  | Studies in Health Technology and Informatics | 2012-00-00 2012 | INTRODUCTION: Information systems with clinical decision support (CDS) offer great potential to assist the co-ordination of patients with chronic diseases and to improve patient care. Despite this, few have entered routine clinical use. BACKGROUND: Tuberculosis (TB) is an infection of public health importance. It has complex interactions with many comorbid conditions, requires close supervised care and prolonged treatment for effective cure. These features make it suitable for use with an information management system with CDS features. In close consultation with key stakeholders, a clinical application was developed for the management of TB patients in Victoria. METHODS: A formal usability assessment using semi-structured case-scenario based exercises was performed. Subjects were 12 individuals closely involved in the care of TB patients, including Infectious Diseases and Respiratory Physicians, and Public Health Nurses. Two researchers conducted the sessions, independently analysed responses and discrepancies compared to the voice record for validity. RESULTS: Despite varied computer experience, responses were positive regarding user interface and content. Data location was not always intuitive, however this improved with familiarity of the program. Decision support was considered valuable, with useful suggestions for expansion of these features. Automated reporting for correspondence and notification to the Health Department were felt worth the initial investment in data entry. An important workflow-based issue regarding dismissal of alerts and several errors were detected. CONCLUSION: Usability assessment validated many design elements of the system, provided a unique insight into workflow issues faced by users and hopefully will impact on its ultimate clinical utility. | Humans | Patient Participation | Self Care | User-Computer Interface | OK |
|  |  |  |  |  |  | Health Education & Behavior | 2011-06-00 juin 2011 | The Community Coalition Action Theory (CCAT) blends practice wisdom with empirical data to explain how community coalitions achieve community change and community capacity outcomes. The current study uses data from an evaluation of 20 California Healthy Cities and Communities coalitions to test relationships between coalition factors and outcomes as predicted by CCAT in two stages of coalition development. Data are from two rounds of coalition member surveys, interviews with local coalition coordinators, and semiannual progress reports. Consistent with CCAT predictions and prior research, shared decision making and leadership were correlated with participation; staff competence, task focus, and cohesion were correlated with member satisfaction. Coalition size was associated with participation and dollars leveraged. Also, consistent with CCAT, diversity of funding sources was associated with new leadership opportunities and program expansion; dollars leveraged was correlated with new leadership opportunities and new partners. Findings provide preliminary support for many, but not all, of the relationships predicted by CCAT. (PsycINFO Database Record (c) 2012 APA, all rights reserved). (journal abstract) | Decision Making | Testing | operationalization | community coalition action theory | OK |
| Andrea | Glässel | Alarcos | Cieza |  |  | Journal of Occupational Rehabilitation | 2011-06-00 juin 2011 | Background :Vocational rehabilitation (VR) is a key process in work disability (WD) management which aims to engage or re-engage individuals to work and employment. The International Classification of Functioning, Disability and Health (ICF) by the World Health Organization (WHO) can be interfaced with VR but there is a lack of evidence of what ICF contents experts in the field consider. The objective of this study is to survey the experts in the VR field with regard to what factors are considered important to patients participating in VR using the ICF as the language to summarize the results. Methods: An internet-based survey was conducted with experts from six WHO Regions (Africa, the Americas, Eastern Mediterranean, Europe, South-East Asia, and Western Pacific). Experts were asked six open-ended questions on factors that are important in VR. Each question was related to a component of the ICF (body functions, body structures, activities and, environmental factors, and personal factors). Responses were linked to the ICF. Results: Using a modified stratified randomized sampling, 201 experts were sent the survey and 151 experts responded (75% response rate). We identified 101 ICF categories: 22 (21.8%) for body functions, 13 (12.9%) for body structures, 36 (35.6%) for activities and participation, and 30 (29.7%) for environmental factors. Conclusions: There was a multitude of ICF functioning domains according to the respondents which indicates the complexity of VR. This expert survey has provided a list of ICF categories which could be considered in VR. (PsycINFO Database Record (c) 2012 APA, all rights reserved). (journal abstract) | Patient Participation | Client Participation | Health | Health Personnel | OK |
|  |  |  |  |  |  | Value in Health: The Journal of the International Society for Pharmacoeconomics and Outcomes Research | 2013-02-00 2013 Jan-Feb | OBJECTIVES: Many jurisdictions are moving toward greater public involvement in health technology assessment (HTA) processes. This study aims to provide a broad, cross-sectional indication of the extent and methods of public engagement in HTA, with a focus on which public are engaged, by what mechanisms, and the purpose of public engagement. METHODS: An international Web-based survey of 217 organizations involved in HTA was undertaken. Contact e-mail addresses for targeted organizations were identified from the Internet. RESULTS: Individuals from 39 (18%) of the contacted organizations completed a survey. The majority (67%) of responding HTA organizations undertake public engagement activities, predominantly involving lay representatives of organized groups (81%), and to a lesser extent individual patients/consumers (54%) or citizens/community members (54%). For organizations undertaking public engagement, mechanisms based on communication or consultation were the most common, although some organizations have used or intend to use participatory approaches, particularly the Citizens' Jury (8%) or Consensus Council (20%) methods. Respondents identified with a number of rationales and barriers for undertaking public engagement. CONCLUSIONS: This survey provides further insight into the public engagement approaches that are used by HTA organizations in practice. In particular, it suggests a limited adoption of participatory methods to date, and interest in the use of social media. Study findings require further confirmation, due to limitations related to survey response. There is considerable opportunity for further research into pragmatic, robust, and meaningful approaches to public engagement to strengthen HTA policy and decision-making frameworks. An agenda for future research evolving from the survey responses is proposed. | Decision Making | Humans | Patient Participation | Internet | OK |
|  |  |  |  |  |  | Patient Education and Counseling | 2011-04-00 Apr 2011 | OBJECTIVE: The current research investigates the potential of online support groups (OSGs) to foster empowerment and how membership might affect the patient/health professional relationship. METHODS: 246 participants across 33 OSGs completed an online questionnaire. RESULTS: All empowerment processes and outcomes identified by van Uden-Kraan et al. [1] were found to be present. All empowerment outcomes were adequately predicted by empowerment processes. The majority (82.2%) of participants had discussed information found online with their health professional and most (74.2%) were satisfied with the response. Around 60% of participants felt membership of an OSG had affected the relationship with their health professional and from qualitative responses the effects were mostly positive. CONCLUSION: OSGs have the potential to produce empowerment outcomes for those who choose to use them. Furthermore, users report a positive reaction to information found online from their health professionals. PRACTICE IMPLICATIONS: Although not all patients will benefit from using OSGs, health professionals suggesting their use could ensure that they reach the maximum receptive audience. Furthermore, this research could be used to encourage a more 'net friendly' attitude amongst health professionals. | Male | Humans | Patient Participation | Questionnaires | OK |
| Göran | Isacsson | Ulf | Brodin |  |  | BMC medical research methodology | 2012-00-00 2012 | BACKGROUND: This study investigates whether an analysis, based on Item Response Theory (IRT), can be used for initial evaluations of depression assessment instruments in a limited patient sample from an affective disorder outpatient clinic, with the aim to finding major advantages and deficiencies of the instruments. METHODS: Three depression assessment instruments, the depression module from the Patient Health Questionnaire (PHQ9), the depression subscale of Affective Self Rating Scale (AS-18-D) and the Montgomery-Åsberg Depression Rating Scale (MADRS) were evaluated in a sample of 61 patients with affective disorder diagnoses, mainly bipolar disorder. A '3- step IRT strategy' was used. RESULTS: In a first step, the Mokken non-parametric analysis showed that PHQ9 and AS-18-D had strong overall scalabilities of 0.510 [C.I. 0.42, 0.61] and 0,513 [C.I. 0.41, 0.63] respectively, while MADRS had a weak scalability of 0.339 [C.I. 0.25, 0.43]. In a second step, a Rasch model analysis indicated large differences concerning the item discriminating capacity and was therefore considered not suitable for the data. In third step, applying a more flexible two parameter model, all three instruments showed large differences in item information and items had a low capacity to reliably measure respondents at low levels of depression severity. CONCLUSIONS: We conclude that a stepwise IRT-approach, as performed in this study, is a suitable tool for studying assessment instruments at early stages of development. Such an analysis can give useful information, even in small samples, in order to construct more precise measurements or to evaluate existing assessment instruments. The study suggests that the PHQ9 and AS-18-D can be useful for measurement of depression severity in an outpatient clinic for affective disorder, while the MADRS shows weak measurement properties for this type of patients. | Male | Humans | Patient Participation | Questionnaires | OK |
|  |  |  |  |  |  | Studies in Health Technology and Informatics | 2010-00-00 2010 | New services devoted to improve personalized healthcare are emerging from information technology developments. Personal health record systems allow the patients to participate actively in their healthcare process. However, the dissemination and use of personal health record systems face with some barriers, for example low health literacy that leads to discrepancy in understanding medical concepts. While it is important to present health information using consumer-familiar terms in consumer applications, consistently converting medical terms to consumer-familiar ones is a challenging task. We designed and developed both an ontology-like taxonomic structure devoted to the Geriatrics domain for the outpatient and a software tool, for carrying out the matching between the medical vocabulary of the consumer and that of the doctor from the outpatient's and their family point of view. | Humans | Patient Participation | communication | Outpatients | OK |
| Christine | Cosby | John | Zajicek |  |  | Clinical Medicine (London, England) | 2012-06-00 Jun 2012 | Promotion of research is a key strategy of the National Health Service (NHS). Currently, many patients are not afforded the opportunity to participate in clinical studies. A register of research-interested patients has the potential to maximise inclusivity. We have established a register of research-interested patients with Parkinson's disease within the South West of England, with pragmatic inclusion criteria and multiple recruitment routes. We undertook an analysis of the register, investigation of its utility as a recruitment tool and a survey of recruiters. There were 529 active participants; 30% were self-referred and 70% were recruited by a healthcare practitioner. Response rate to annual questionnaires was 86.5%. Staff time required for pack preparation, recruitment and data entry was 15 min per new recruit and 5 min per follow-up questionnaire. In total, 85% of recruiters viewed the register positively. A single mailing to participants resulted in a recruitment rate that significantly exceeded that achieved by traditional recruitment methods. | Male | Humans | Patient Participation | Female | OK |
|  |  |  |  |  |  | Polski Merkuriusz Lekarski: Organ Polskiego Towarzystwa Lekarskiego | 2014-02-00 Feb 2014 | Prophylactic examinations allowing for the evaluation of the medical condition and the diagnosis of symptoms of illness in the preclinical stage is a crucial element of the prevention. The early diagnosis of disorders is improving the effectiveness of medicinal actions and rehabilitation, enhancing the possibility of getting well. It is of special importance in cardiovascular diseases which in Poland are recognised late and have become a fundamental health hazard. The aim of study was to evaluate causes of the participation in open days in the aspect of sociodemographic conditioning of residents of Sanok district and conducting the evaluation of their body mass and values of the blood pressure. MATERIALS AND METHODS: The research was conducted in 2009 and in 2010 including the group of 215 persons during open days of the special hospital of the Independent Public Centre of Health Care in Sanok. In the accumulation of empirical material method of the diagnostic survey was used with applying the author's questionnaire of the survey form. The following techniques were applied: conducting a survey and the measurement of biophysical features (of body mass, height, blood pressure) with using measuring devices. Collected data were subjected to a statistical analysis with applying the chi-square test. RESULTS: The ease of access and desire for performing examinations and expectation of the evaluation of the medical condition decided in the main measure about the participation in this initiative. The greatest demand was for advice of the cardiologist. The correct BMI indicator has most often been evaluated in the group of people of the singleness and country dwellers. The obesity has more often been identified at persons after divorce, being married and of residents of cities. Almost at the every second person an arterial hypertension of blood was stated. Majority of the examined had the correct concentration of glucose in the blood on an empty stomach. CONCLUSIONS: Risk factors of cardiovascular diseases identified at examined persons are an arterial hypertension and different degrees of the excess mass. During open days the greatest demand was stated to advice of consultants in cardiology. Obtained findings constitute the argument being enough to continue further diagnostic investigation at patients, at whom alarming manifestations were identified, in order to confirm illness and early beginning the treatment. | Male | Humans | Patient Participation | Questionnaires | OK |
| Yves | Longtin | Cécile | Michaud |  |  | Perspective Infirmière: Revue Officielle De l'Ordre Des Infirmières Et Infirmiers Du Québec | 2014-04-00 2014 Mar-Apr | | Humans | Patient Participation | Questionnaires | Female | OK |
| Amy | Herring | Fred | Spielman | Karamarie | Fecho | Journal of Midwifery & Women's Health | 2011-08-00 2011 Jul-Aug | INTRODUCTION: This study described anesthesia and analgesia-related preferences and outcomes of women who used a birth plan for labor and birth. METHODS: A prospective cohort study was conducted (N = 63). Data were abstracted from medical records, birth plans, and a follow-up survey. Descriptive statistics were used for analysis. RESULTS: Women who elected birth plans were primarily white, college-educated, primigravida, and under the care of a certified nurse-midwife. One-third of births were induced, 10% required instrumentation, and 29% were cesarean births. Nearly every birth was associated with at least 1 labor and birth complication, although most complications were minor. Analgesic preferences were reported to be the most important birth plan request. Greater than 50% of women requested to avoid epidural analgesia; however, 65% of women received epidural analgesia. On follow-up, greater than 90% of women who received epidural analgesia reported being pleased. The majority of women agreed that the birth plan enhanced their birth experiences, added control, clarified their thoughts, and improved communication with their health care providers. DISCUSSION: Anesthesia and analgesia-related preferences were an important component of the birth plans. The majority of women favorably viewed the use of a birth plan, whether or not preferences were fulfilled or complications occurred. | Humans | Patient Participation | Questionnaires | Female | OK |
| Stefanie | Wilke | Mechthild | Hartmann | Wolfgang | Herzog | Pacing and clinical electrophysiology: PACE | 2014-01-00 Jan 2014 | OBJECTIVES: To determine (1) the frequency and course of anxiety disorders in patients with implantable cardioverter defibrillators (ICDs), (2) the predictors of anxiety, (3) the treatment situation and patients' requests for therapy. METHODS: Quantitative and qualitative methods in a prospective design. At baseline, 327 ICD outpatients completed validated self-report questionnaires (participation rate = 77%). Five months later, a predefined subsample of patients (n = 108, participation rate = 81%) consisting of all patients with (n = 58) and a randomly selected subsample of patients without (n = 50) elevated symptoms of anxiety at baseline, was reassessed using a structured diagnostic interview, the baseline questionnaires, and open-ended questions. RESULTS: At baseline, 19.2% of patients suffered from at least some form of clinically relevant anxiety with an overall remission rate of 56.5% at follow-up. Predictive for anxiety at follow-up were higher levels of stress (odds ratio [OR], 1.52, P < 0.001), depression (OR, 1.26, P < 0.001), somatic symptom severity (OR, 1.25, P < 0.001), more perceived ICD-related constraints (OR, 2.4, P = 0.007), lower quality of life (physical health: OR, 0.91, P = 0.004; mental health: OR, 0.87, P = 0.001), and a higher New York Heart Association class (OR, 7.99, P = 0.002) at baseline. Only 35.3% of patients received an evidenced-based treatment for their anxiety disorder. A supervised ICD patient group was the most preferred treatment (51.1%). CONCLUSIONS: Most patients seemed to adapt well to ICD therapy. Patients suffering from additional psychological strains and reporting more negative ICD-related attitudes were at risk for developing an anxiety disorder. Special tailored interventions, such as a supervised ICD patient group, could reduce the gap between treatment needs and the treatment situation. | Male | Humans | Patient Participation | Questionnaires | OK |
|  |  |  |  |  |  | Archives of Physical Medicine and Rehabilitation | 2010-09-00 Sep 2010 | Measurement of participation in people with disability can pose psychometric and conceptual challenges. Ambiguous or paradoxical findings can occur because of differences among people or changes within people regarding internal standards, values, or conceptualization of participation. These response shifts can affect standard psychometric indices, such as reliability and validity. We focus herein on the interpretation of patient-reported outcomes and, in particular, on the cognitive appraisal processes known as response shift. We present theoretical and conceptual distinctions building on response shift theory and other current developments in health-related quality of life research to inform participation measurement research. We discuss how response shifts can influence the interpretation of reliability, validity, and responsiveness of participation measures. We then discuss the evidence for the clinical significance of response shift phenomena and describe current design, statistical, and individualized approaches for detecting response shift phenomena. | Humans | Patient Participation | Psychometrics | quality of life | OK |
| Korbinian | Fischer | Nina | Schäffler | Imke | Backhus | BMC medical informatics and decision making | 2012-00-00 2012 | BACKGROUND: Patients making important medical decisions need to evaluate complex information in the light of their own beliefs, attitudes and priorities. The process can be considered in terms of the theory of planned behaviour. Decision support technologies aim at helping patients making informed treatment choices. Instruments assessing informed choices need to include risk knowledge, attitude (towards therapy) and actual uptake. However, mechanisms by which decision support achieves its goals are poorly understood.Our aim was therefore to develop and validate an instrument modeling the process of multiple sclerosis (MS) patients' decision making about whether to undergo disease modifying (immuno-)therapies (DMT). METHODS: We constructed a 30-item patient administered questionnaire to access the elaboration of decisions about DMT in MS according to the theory of planned behaviour. MS-patients' belief composites regarding immunotherapy were classified according to the domains "attitude", "subjective social norm" and "control beliefs" and within each domain to either "expectations" or "values" yielding 6 sub-domains. A randomized controlled trial (n = 192) evaluating an evidence based educational intervention tested the instrument's predictive power regarding intention to use immunotherapy and its sensitivity to the intervention. RESULTS: The psychometric properties of the questionnaire were satisfactory (mean item difficulty 62, mean SD 0.9, range 0-3). Responses explain up to 68% of the variability in the intention to use DMT was explained by up to 68% in the total sample. Four weeks after an educational intervention, predictive power was higher in the intervention (IG) compared to the control group (CG) (intention estimate: CG 56% / IG 69%, p = .179; three domains CG 56% / IG 74%, p = .047; six sub-domains CG 64% / IG 78%, p = .073). The IG held more critical beliefs towards immunotherapy (p = .002) and were less willing to comply with social norm (p = .012). CONCLUSIONS: The questionnaire seems to provide a valid way of explaining patients' inherent decision processes and to be sensitive towards varying levels of elaboration. Similar tools based on the theory of planned behaviour could be applied to other decision making scenarios. | Male | Decision Making | Decision Support Techniques | Humans | OK |
| Ann-Britt | Wiréhn | Maria | Friedrichsen |  |  | BMC geriatrics | 2011-00-00 2011 | BACKGROUND: Medical decision making has long been in focus, but little is known of the preferences and conditions for elderly people with co-morbidities to participate in medical decision making. The main objective of the present study was to investigate the preferred and the actual degree of control, i.e. the role elderly people with co-morbidities wish to assume and actually had with regard to information and participation in medical decision making during their last stay in hospital.This study was a cross-sectional survey including three Swedish hospitals with acute admittance. The participants were patients aged 75 years and above with three or more diagnoses according to the International Classification of Diseases (ICD-10) and three or more hospitalisations during the last year. METHODS: We used a questionnaire combined with a telephone interview, using the Control Preference Scale to measure each participant's preferred and actual role in medical decision making during their last stay in hospital. Additional questions were asked about barriers to participation in decision making and preferred information seeking role. The results are presented with descriptive statistics with kappa weights. RESULTS: Of the 297 elderly patients identified, 52.5% responded (n = 156, 46.5% male). Mean age was 83.1 years. Of the respondents, 42 of 153 patients said that they were not asked for their opinion (i.e. no shared decision making). Among the other 111 patients, 49 had their exact preferred level of participation, 37 had less participation than they would have preferred, and 23 had more responsibility than they would have preferred. Kappa statistics showed a moderate agreement between preferred and actual role (κw = 0.57; 95% CI: 0.45-0.69). Most patients wanted to be given more information without having to ask. There was no correlation between age, gender, or education and preferred role. 35% of the patients agreed that they experienced some of the various barriers to decision making that they were asked about: 1) the severity of their illness, 2) doctors with different treatment strategies, 3) difficulty understanding the medical information, and 4) difficulty understanding doctors who did not speak the patient's own language. CONCLUSIONS: Physicians are not fully responsive to patient preferences regarding either the degree of communication or the patient's participation in decision making. Barriers to participation can be a problem, and should be taken into account more often when dealing with hospitalised elderly people. | Male | Decision Making | Humans | Patient Participation | OK |
|  |  |  |  |  |  | Harvard Health Letter / from Harvard Medical School | 2013-01-00 Jan 2013 | | Humans | Patient Participation | Arrhythmias, Cardiac | Health Education | OK |
| Sahar | Borairi |  |  |  |  | The International Journal of Eating Disorders | 2014-01-00 Jan 2014 | Compassion-focused therapy (CFT; Gilbert, 2005, 2009) is a transdiagnostic treatment approach focused on building self-compassion and reducing shame. It is based on the theory that feelings of shame contribute to the maintenance of psychopathology, whereas self-compassion contributes to the alleviation of shame and psychopathology. We sought to test this theory in a transdiagnostic sample of eating disorder patients by examining whether larger improvements in shame and self-compassion early in treatment would facilitate faster eating disorder symptom remission over 12 weeks. Participants were 97 patients with an eating disorder admitted to specialized day hospital or inpatient treatment. They completed the Eating Disorder Examination-Questionnaire, Experiences of Shame Scale, and Self-Compassion Scale at intake, and again after weeks 3, 6, 9, and 12. Multilevel modeling revealed that patients who experienced greater decreases in their level of shame in the first 4 weeks of treatment had faster decreases in their eating disorder symptoms over 12 weeks of treatment. In addition, patients who had greater increases in their level of self-compassion early in treatment had faster decreases in their feelings of shame over 12 weeks, even when controlling for their early change in eating disorder symptoms. These results suggest that CFT theory may help to explain the maintenance of eating disorders. Clinically, findings suggest that intervening with shame early in treatment, perhaps by building patients' self-compassion, may promote better eating disorders treatment response. | Male | Humans | Patient Participation | Questionnaires | OK |
| Robert A. C. | Ruiter | Herman P. | Schaalma | Wim L. A. M. | de Kort | Transfusion | 2012-06-00 Jun 2012 | BACKGROUND: This study investigated the possibility of rerecruiting lapsed blood donors. Reasons for donation cessation, motivation to restart donation, and modifiable components of donation motivation were examined. We distinguished between lapsed donors who had passively withdrawn by merely not responding to donation invitations and donors who had contacted the blood bank to actively withdraw. STUDY DESIGN AND METHODS: A cross-sectional survey was sent to 400 actively lapsed donors and to 400 passively lapsed donors, measuring intention to restart donation and psychological correlates of restart intention. The data were analyzed using multiple regression analyses. RESULTS: The response rate among actively lapsed donors was higher than among passively lapsed donors (37% vs. 25%). Actively lapsed donors typically ceased donating because of physical reactions, while passively lapsed donors quit because of a busy lifestyle. Nonetheless, 51% of actively lapsed responders and 80% of passively lapsed responders were willing to restart donations. Multiple regression analysis showed that, for passively lapsed donors, cognitive attitude was the strongest correlate of intention to donate in the future (β=0.605, p<0.001), with affective attitude (β=0.239, p<0.05) and self-efficacy (β=0.266, p<0.001) explaining useful proportions of the variance as well. For actively lapsed donors, cognitive attitude was also the strongest correlate of intention (β=0.601, p<0.001), with affective attitude (β=0.345, p<0.001) and moral norm (β=-0.118, p<0.05) explaining smaller proportions of the variance. CONCLUSION: The majority of lapsed donors indicated a moderate to high intention to restart donations. Interventions focusing on boosting cognitive and affective attitudes and self-efficacy could further raise such intentions. | Male | Humans | Patient Participation | Questionnaires | OK |
| Robert | Vander Stichele | Jan | Bernheim | Freddy | Mortier | Palliative Medicine | 2011-01-00 Jan 2011 | We examined the degree to which newly diagnosed patients with advanced lung cancer wanted to be informed and involved in medical decision-making, and whether the patients felt their preferences were met. Patients from 13 hospitals in Flanders were interviewed with a standard questionnaire. A total of 128 patients (68%) participated. Of the patients who wanted to be informed about life expectancy, half (53%) reported they were informed, and of those who wanted to be informed about palliative care and end-of-life decisions, 25% and 31% said they were informed, respectively. With regard to participation in medical decision-making (in general, about treatment, transfer or end-of-life), patients who preferred the doctor to make decisions or those who preferred to make the decision themselves often achieved this (in their perception), while patients who wanted an in-between position with some involvement, often did not. To conclude, preferences of patients with lung cancer for information concerning delicate topics and for shared decision-making with the physician were not well met. | Male | Decision Making | Humans | Patient Satisfaction | OK |
| Arash | Taheri | Laura F. | Sandoval | Scott A. | Davis | The Journal of Dermatological Treatment | 2014-10-00 Oct 2014 | | Male | Humans | Patient Participation | Female | OK |
| Milan | Milošević | Jadranka | Mustajbegović | Ana | Borovečki | Croatian Medical Journal | 2013-04-00 Apr 2013 | AIM: To explore physician-patient communication practices during the process of obtaining informed consent in a hospital setting in Croatia. METHODS: Two hundred and fifty patients (response rate 78%) from five tertiary level hospitals in Zagreb, Croatia, anonymously filled in the questionnaire on informed consent and communication practices by Nemcekova et al in the period from April to December 2011. RESULTS: Eighty five percent of patients received complete, understandable information, presented in a considerate manner. Patients in surgical departments received a higher level of information than those in internal medicine departments. Patients were informed about health risks of the proposed treatments (in 74% of cases) and procedures (76%), health consequences of refusing a medical intervention (69%), and other methods of treatment (46%). However, patients pointed out a number of problems in physician-patient communication. CONCLUSION: Communication practices during informed consent-obtaining process in hospitals in Zagreb are based on a model of shared decision-making, but paternalistic physician-patient relationship is still present. Our results indicate that Croatia is undergoing a transition in the physician-patient relationship and communication. | Male | Decision Making | Humans | Physician-Patient Relations | OK |
| Malcolm | Campbell | Chris | Todd |  |  | Journal of Aging and Physical Activity | 2012-01-00 Jan 2012 | Little is known about the relationship between attitudes and characteristics of instructors and uptake and adherence of older people to exercise classes. This article explores these issues. METHODS: The authors surveyed 731 UK exercise instructors with specialist older adult exercise qualifications. A questionnaire investigated instructors' characteristics and attitudes toward older adults' participation in exercise. RESULTS: For mostly seated classes, EXTEND qualification (B = 0.36, p = .005) had a positive effect on instructors' attitudes. Later Life Training qualification (B = -2.80, p = .003), clinical background (B = -3.99, p = .005), and delivering classes in National Health Services (B = -3.12, p < .001), leisure centers (B = -2.75, p = .002), or nursing homes (B = -2.29, p = .005) had a negative effect on attitudes. For mostly standing classes, experience (B = 0.20, p = .003) and delivering in leisure centers (B = 0.46, p = .032) had a positive and clinical background (B = -1.78, p = .018) had a negative effect on instructors' attitudes. CONCLUSIONS: Most instructors have positive attitudes, but training and work context can influence attitudes toward older people's participation in exercise classes both positively and negatively. | Male | Humans | Patient Participation | Questionnaires | OK |
| Algernon | Cargill | Marshall H. | Chin |  |  | Medical Decision Making: An International Journal of the Society for Medical Decision Making | 2011-06-00 2011 May-Jun | BACKGROUND: In the United States, African Americans are more likely to experience lower quality patient/provider communication and less shared decision making (SDM) than whites, which may be an important contributor to racial health disparities. Patient factors have not been fully explored as a potential contributor to communication disparities. METHODS: The authors analyzed cross-sectional data from a survey of 974 patients with diabetes seen at 34 community health centers (HC) in 17 midwestern and west-central states. They used ordinal and logistic regression models to investigate racial differences in patients' preferences for SDM and in patients' behaviors that may facilitate SDM (initiating discussions about diabetes care). RESULTS: The response rate was 67%. In bivariate and multivariate analyses, race was not associated with patient preference for a shared role in the 3 measured SDM domains: agenda setting (odds ratio [OR]: 1.13 [0.86, 1.49]), information sharing (OR: 1.26 [0.97, 1.64]), or decision making (OR: 1.16 [0.85, 1.59]). African Americans were more likely to report initiating discussions with their physicians about 4 of 6 areas of diabetes care-blood pressure measurement (66% v. 52%, P < 0.001), foot examination (54% v. 47%, P = 0.04), eye examination (57% v. 46%, P = 0.002), and microalbumin testing (38% v. 29%, P = 0.01)-but not HbA1c testing (39% v. 43%, P = 0.31) or cholesterol testing (53% v. 51%, P = 0.52). In multivariate analysis, African Americans were still more likely to report initiating conversations about diabetes care (OR: 1.78 [1.10, 2.89]). CONCLUSIONS: The authors found that African Americans in this study preferred shared decision making as much as whites and were more likely to report initiating more discussions with their doctors about their diabetes care. This research suggests that, among diabetes patients receiving care at community health centers, patient preference or patient behaviors may be an unlikely cause of racial differences in shared decision making. | Decision Making | Humans | Patient Satisfaction | Physician-Patient Relations | OK |
|  |  |  |  |  |  | Canadian Oncology Nursing Journal = Revue Canadienne De Nursing Oncologique | 2013-00-00 2013 | | Humans | Patient Participation | Neoplasms | Nurse-Patient Relations | OK |
| Tanja | Krones | Norbert | Donner-Banzhoff | |  | BMC medical informatics and decision making | 2012-00-00 2012 | BACKGROUND: The successful implementation of decision aids in clinical practice initially depends on how clinicians perceive them. Relatively little is known about the acceptance of decision aids by physicians and factors influencing the implementation of decision aids from their point of view. Our electronic library of decision aids (arriba-lib) is to be used within the encounter and has a modular structure containing evidence-based decision aids for the following topics: cardiovascular prevention, atrial fibrillation, coronary heart disease, oral antidiabetics, conventional and intensified insulin therapy, and unipolar depression. The aim of our study was to evaluate the acceptance of arriba-lib in primary care physicians. METHODS: We conducted an evaluation study in which 29 primary care physicians included 192 patients. The physician questionnaire contained information on which module was used, how extensive steps of the shared decision making process were discussed, who made the decision, and a subjective appraisal of consultation length. We used generalised estimation equations to measure associations within patient variables and traditional crosstab analyses. RESULTS: Only a minority of consultations (8.9%) was considered to be unacceptably extended. In 90.6% of consultations, physicians said that a decision could be made. A shared decision was perceived by physicians in 57.1% of consultations. Physicians said that a decision was more likely to be made when therapeutic options were discussed "detailed". Prior experience with decision aids was not a critical variable for implementation within our sample of primary care physicians. CONCLUSIONS: Our study showed that it might be feasible to apply our electronic library of decision aids (arriba-lib) in the primary care context. Evidence-based decision aids offer support for physicians in the management of medical information. Future studies should monitor the long-term adoption of arriba-lib in primary care physicians. | Male | Decision Support Techniques | Humans | Patient Participation | OK |
| Dan | Rothstein | Luz | Santana | Marthe R. | Gold | Patient Education and Counseling | 2011-08-00 Aug 2011 | OBJECTIVE: To evaluate the impact of a patient activation intervention (PAI) focused on building question formulation skills that was delivered to patients in community health centers prior to their physician visit. METHODS: Level of patient activation and patient preferred role were examined using the patient activation measure (PAM) and the patient preference for control (PPC) measure. RESULTS: More of the 252 patients evaluated were at lower levels of activation (PAM levels 1 or 2) than U.S. population norms before the intervention. Paired-samples t-test revealed a statistically significant increase from pre-intervention to post-visit PAM scores. One-third of participants moved from lower levels of activation to higher levels (PAM levels 3 or 4) post-intervention. Patients preferring a more passive role had lower initial PAM scores and greater increases in their post-intervention PAM scores than did those who preferred a more active role. CONCLUSION: Patients exposed to the PAI demonstrated significant improvement on a measure of activation. The PAI may be useful in helping patients prepare for more effective encounters with their physicians. PRACTICE IMPLICATIONS: The PAI was feasible to deliver in the health center setting and may be a useful method for activating low-income, racial/ethnic minority patient populations. | Male | Decision Making | Humans | Patient Participation | OK |
| Ali A. | Mokdad | Danny D. | Shen | David W. | Fleming | BMC cardiovascular disorders | 2014-00-00 2014 | BACKGROUND: Little is known about the relationship between cardiovascular risk, disease and actual use of aspirin in the community. METHODS: The Measuring Disparities in Chronic Conditions (MDCC) study is a community and health facility-based survey designed to track disparities in the delivery of health interventions for common chronic diseases. MDCC includes a survey instrument designed to collect detailed information about aspirin use. In King County, WA between 2011 and 2012, we surveyed 4633 white, African American, or Hispanic adults (45% home address-based sample, 55% health facility sample). We examined self-reported counseling on, frequency of use and risks of aspirin for all respondents. For a subgroup free of CAD or cerebral infarction that underwent physical examination, we measured 10-year coronary heart disease risk and blood salicylate concentration. RESULTS: Two in five respondents reported using aspirin routinely while one in five with a history of CAD or cerebral infarction and without contraindication did not report routine use of aspirin. Women with these conditions used less aspirin than men (65.0% vs. 76.5%) and reported more health problems that would make aspirin unsafe (29.4% vs. 21.2%). In a subgroup undergoing phlebotomy a third of respondents with low cardiovascular risk used aspirin routinely and only 4.6% of all aspirin users had no detectable salicylate in their blood. CONCLUSIONS: In this large urban county where health care delivery should be of high quality, there is insufficient aspirin use among those with high cardiovascular risk or disease and routine aspirin use by many at low risk. Further efforts are needed to promote shared-decision making between patients and clinicians as well as inform the public about appropriate use of routine aspirin to reduce the burden of atherosclerotic vascular disease. | Male | Humans | Questionnaires | Female | OK |
| H. | Raspe |  |  |  |  | Zeitschrift Für Gastroenterologie | 2013-03-00 Mar 2013 | BACKGROUND: Health care for inflammatory bowel diseases (IBD), to be comprehensive, takes a broad range of patients' somatic and psychosocial problems into account. Patients should be actively involved in health-care planning and organisation. METHODS: 431 adult patients with Crohn's disease (50 %) or ulcerative colitis participated in a postal questionnaire survey; 6 months apart it twice assessed the patients' individual problem profiles. The results of the assessment were back-reported to each patient combined with targeted (though standardised) recommendations for future care. This publication is the first of a series. It presents the basic study design, describes the prevalence of 16 psychosocial problem domains and analyses their association with socio-demographic and disease variables. RESULTS: Participants had a mean age of 46 years; 61 % were female; 57 % in remission (GIBDI ≤ 3). The most prevalent problems reported addressed sexual impairments (27 %), high stress (26 %) and depression (21 %). 27 % of the respondents did not report any psychosocial problem. One in five (21 %) described highly complex problem profiles (≥ 5 "active" problems) combined with a mean of 28 disability days within the past 3 months. Complex psychosocial profiles were associated with active disease, Crohn's disease and low educational level. CONCLUSIONS: IBD patients show a highly variable spectrum of psychosocial problems. Their number is closely associated with disease activity and a social status variable (school education). The gradient could complicate efforts to increase patient participation in care and to enhance self-management. | Male | Humans | Questionnaires | Young Adult | OK |
| T. | Le | H. | Thompson | G. | Demiris | Methods of Information in Medicine | 2014-00-00 2014 | INTRODUCTION: This article is part of the Focus Theme of Methods of Information in Medicine on "Using Data from Ambient Assisted Living and Smart Homes in Electronic Health Records". OBJECTIVES: Our objectives were to: 1) characterize older adult participants' perceived usefulness of in-home sensor data and 2) develop novel visual displays for sensor data from Ambient Assisted Living environments that can become part of electronic health records. METHODS: Semi-structured interviews were conducted with community-dwelling older adult participants during three and six-month visits. We engaged participants in two design iterations by soliciting feedback about display types and visual displays of simulated data related to a fall scenario. Interview transcripts were analyzed to identify themes related to perceived usefulness of sensor data. RESULTS: Thematic analysis identified three themes: perceived usefulness of sensor data for managing health; factors that affect perceived usefulness of sensor data and; perceived usefulness of visual displays. Visual displays were cited as potentially useful for family members and health care providers. Three novel visual displays were created based on interview results, design guidelines derived from prior AAL research, and principles of graphic design theory. CONCLUSIONS: Participants identified potential uses of personal activity data for monitoring health status and capturing early signs of illness. One area for future research is to determine how visual displays of AAL data might be utilized to connect family members and health care providers through shared understanding of activity levels versus a more simplified view of self-management. Connecting informal and formal caregiving networks may facilitate better communication between older adults, family members and health care providers for shared decision-making. | | | | | OK |
| Annie | Lapointe | Sarah‐Maude | Deschênes | Sophie | Desroches | Health Expectations: An International Journal of Public Participation in Health Care & Health Policy | 2014-08-00 août 2014 | Background: Shared decision making (SDM) represents an interesting approach to optimize the impact of dietary treatment, but there is no evidence that SDM is commonly integrated into diet‐related health care. Objective: To assess the extent to which dietitians involve patients in decisions about dietary treatment. Methods: We audiotaped dietitians conducting nutritional consultations with their patients, and we transcribed the tapes verbatim. Three trained raters independently evaluated the content of the nutritional consultations using a coding frame based on the 12 items of the French‐language version of the OPTION scale, a validated and reliable third‐observer instrument designed to assess patients’ involvement by examining specific health professionals’ behaviours. Coding was facilitated by the qualitative research software NVivo 8. We assessed internal consistency with Cronbach’s alpha and inter‐rater reliability with the intraclass correlation coefficient (ICC). Results: Of the 40 dietitians eligible to participate in the study, 19 took part. We recruited one patient per participating dietitian. The overall mean OPTION score was 29 ± 8% [range, 0% (no patient involvement in the decision] to 100% [high patient involvement)]. The mean duration of consultations was 50 ± 26 min. The OPTION score was positively correlated with the duration of the consultation (r = 0.65, P &lt; 0.01). Internal consistency and inter‐rater reliability were both good (Cronbach’s alpha = 0.72; ICC = 0.65). Conclusion: This study is the first to use a framework based on the OPTION scale to report on dietitians’ involvement of patients in decisions about patients’ dietary treatment. The results suggest that involvement is suboptimal. Interventions to increase patients’ involvement in diet‐related decision making are indicated. (PsycINFO Database Record (c) 2014 APA, all rights reserved). (journal abstract) | Shared decision making | Decision Making | patients | Therapeutic Processes | Doublon |
| Annie | Lapointe | Sarah-Maude | Deschênes | Sophie | Desroches | Health Expectations: An International Journal of Public Participation in Health Care and Health Policy | 2014-08-00 Aug 2014 | BACKGROUND: Shared decision making (SDM) represents an interesting approach to optimize the impact of dietary treatment, but there is no evidence that SDM is commonly integrated into diet-related health care. OBJECTIVE: To assess the extent to which dietitians involve patients in decisions about dietary treatment. METHODS: We audiotaped dietitians conducting nutritional consultations with their patients, and we transcribed the tapes verbatim. Three trained raters independently evaluated the content of the nutritional consultations using a coding frame based on the 12 items of the French-language version of the OPTION scale, a validated and reliable third-observer instrument designed to assess patients' involvement by examining specific health professionals' behaviours. Coding was facilitated by the qualitative research software NVivo 8. We assessed internal consistency with Cronbach's alpha and inter-rater reliability with the intraclass correlation coefficient (ICC). RESULTS: Of the 40 dietitians eligible to participate in the study, 19 took part. We recruited one patient per participating dietitian. The overall mean OPTION score was 29 ± 8% [range, 0% (no patient involvement in the decision] to 100% [high patient involvement)]. The mean duration of consultations was 50 ± 26 min. The OPTION score was positively correlated with the duration of the consultation (r = 0.65, P < 0.01). Internal consistency and inter-rater reliability were both good (Cronbach's alpha = 0.72; ICC = 0.65). CONCLUSION: This study is the first to use a framework based on the OPTION scale to report on dietitians' involvement of patients in decisions about patients' dietary treatment. The results suggest that involvement is suboptimal. Interventions to increase patients' involvement in diet-related decision making are indicated. | | | | | OK |
| Lisa H. | Jaycox | Aaron | Kofner | Dana | Schultz | Journal of Experimental Criminology | 2013-09-00 septembre 2013 | Objectives: The study tests whether participation in interventions offered by a subset of sites from the National Safe Start Promising Approaches for Children Exposed to Violence initiative improved outcomes for children relative to controls. Methods: The study pools data from the nine Safe Start sites that randomized families to intervention and control groups, using a within-site block randomization strategy based on child age at baseline. Caregiver-reported outcomes, assessed at baseline, 6 and 12 months, included caregiver personal problems, caregiver resource problems, parenting stress, child and caregiver victimization, child trauma symptoms, child behavior problems, and social-emotional competence. Results: Results revealed no measurable intervention impact in intent-to-treat analyses at either 6- or 12-month post-baseline. In 6-month as-treated analyses, a medium to high intervention dose was associated with improvement on two measures of child social-emotional competence: cooperation and assertion. Overall, there is no reliable evidence of significant site-to-site effect variability, even in the two cases of significant intervention effect. Conclusions: Since families in both the intervention and control groups received some degree of case management and both groups improved over time, it may be advantageous to explore the potential impacts of crisis and case management separately from mental health interventions. It may be that, on average, children in families whose basic needs are being attended to improve substantially on their own. (PsycINFO Database Record (c) 2013 APA, all rights reserved). (journal abstract) | Client Participation | Intervention | At Risk Populations | Program Evaluation | OK |
| Jennifer | Yessis | Barry S. | Coller | David K. | Henderson | Clinical and Translational Science | 2011-12-00 Dec 2011 | INTRODUCTION: Participants' perceptions of their research experiences provide valuable measures of ethical treatment, yet no validated instruments exist to measure these experiences. We conducted focus groups of research participants and professionals as the initial step in developing a validated instrument. METHODS: Research participants enrolled in 12 focus groups, consisting of: (1) individuals with disorders undergoing interventions; (2) in natural history studies; or (3) healthy volunteers. Research professionals participated in six separate groups of: (1) institutional review board members, ethicists, and Research Subject Advocates; (2) research nurses/coordinators; or (3) investigators. Focus groups used standard methodologies. RESULTS: Eighty-five participants and 29 professionals enrolled at eight academic centers. Altruism and personal relevance of the research were commonly identified motivators; financial compensation was less commonly mentioned. Participants were satisfied with informed consent processes but disappointed if not provided test results, or study outcomes. Positive relationships with research teams were valued highly. Research professionals were concerned about risks, undue influence, and informed consent. CONCLUSIONS: Participants join studies for varied, complex reasons, notably altruism and personal relevance. They value staff relationships, health gains, new knowledge, and compensation, and expect professionalism and good organization. On the basis of these insights, we propose specific actions to enhance participant recruitment, retention, and satisfaction. | Male | Humans | Patient Participation | Patient Satisfaction | OK |
| Paul W. | Newacheck | Christina D. | Bethell | Stephen J. | Blumberg | Maternal and Child Health Journal | 2014-06-10 Jun 10, 2014 | To provide a national, population-based assessment of the quality of the health care system for children and youth with special health care needs using a framework of six health care system quality indicators. 49,242 interviews with parents of children with special health care needs from the 2009-10 National Survey of Children with Special Health Care Needs (NS-CSHCN) were examined to determine the extent to which CSHCN had access to six quality indicators of a well-functioning system of services. Criteria for determining access to each indicator were established and applied to the survey data to estimate the proportion of CSHCN meeting each quality indicator by socio-demographic status and functional limitations. 17.6 % of CSHCN received care consistent with all six quality indicators. Results for each component of the system quality framework ranged from a high of 70.3 % of parents reporting that they shared decision-making with healthcare providers to a low of 40 % of parents reporting receipt of services needed for transition to adult health care. Attainment rates were lower for CSHCN of minority racial and ethnic groups, those residing in households where English was not the primary language, those in lower income households, and those most impacted by their health condition. Only a small proportion of CSHCN receive all identified attributes of a high-quality system of services. Moreover, significant disparities exist whereby those most impacted by their conditions and those in traditionally disadvantaged groups are served least well by the current system. A small proportion of CSHCN appear to remain essentially outside of the system, having met few if any of the elements studied. | | | | | OK |
| Aleksandra | Torbica |  |  |  |  | Value in Health | 2013-01-00 janvier 2013 | Providing universal access to innovative, high-cost technologies leads to tensions in today’s health care systems. The tension becomes particularly evident in the context of scarce resources, where the risk of taking contentious coverage decisions increases rapidly. To ensure economic sustainability, the payers of health care think that the benefits from the use of the new technologies need to be commensurate with the costs. Therefore, many jurisdictions have programs of health technology assessment, which often results in restrictions of access to care, either through complete refusal to reimburse the technology or its restriction of use to only a subset of the eligible patient population. However, manufacturers feel that they should be adequately rewarded for their innovations and require sufficient funds to invest in further research. Finally, patients perceive these technologies to have added benefits, and so they are concerned when they are denied access. If sustainable access to health care is to be maintained in the future, approaches are needed to reconcile these different perspectives. This article explores the approaches, in both methods and policy, to help bring about this reconciliation. These include rethinking the notion of social value (on the part of payers), aligning manufacturers’ research more closely with societal objectives, and increasing patient participation in health technology assessment. (PsycINFO Database Record (c) 2014 APA, all rights reserved). (journal abstract) | Patient Participation | Client Participation | Health Care Services | health technology assessment | Doublon |
| Aleksandra | Torbica |  |  |  |  | Value in Health: The Journal of the International Society for Pharmacoeconomics and Outcomes Research | 2013-02-00 2013 Jan-Feb | Providing universal access to innovative, high-cost technologies leads to tensions in today's health care systems. The tension becomes particularly evident in the context of scarce resources, where the risk of taking contentious coverage decisions increases rapidly. To ensure economic sustainability, the payers of health care think that the benefits from the use of the new technologies need to be commensurate with the costs. Therefore, many jurisdictions have programs of health technology assessment, which often results in restrictions of access to care, either through complete refusal to reimburse the technology or its restriction of use to only a subset of the eligible patient population. However, manufacturers feel that they should be adequately rewarded for their innovations and require sufficient funds to invest in further research. Finally, patients perceive these technologies to have added benefits, and so they are concerned when they are denied access. If sustainable access to health care is to be maintained in the future, approaches are needed to reconcile these different perspectives. This article explores the approaches, in both methods and policy, to help bring about this reconciliation. These include rethinking the notion of social value (on the part of payers), aligning manufacturers' research more closely with societal objectives, and increasing patient participation in health technology assessment. | Humans | Patient Participation | health policy | Health Services Accessibility | OK |
| Anne-Michelle | Noone | Larisa | Caicedo | Maria | Lopez-Class | Journal of Community Health | 2012-04-00 Apr 2012 | Clinical trials are considered the gold standard of evidence about the efficacy of cancer prevention, early detection, and treatment interventions. A paucity of data exists on determinants of clinical trial participation in the growing US Latino population despite poor cancer outcomes in this group. This study seeks to describe correlates of awareness of and willingness to participate in clinical trials among largely Central, North, and South American Latinos using safety-net clinics. Between June 2007 and November 2008, we conducted an interviewer-administered, Spanish-language cross-sectional survey (n = 944). Logistic regression was used to assess effects of health information sources and psychosocial variables on awareness of and intention to participate in clinical trials. Analyses were completed in spring 2010. While only 48% knew what a clinical trial was, when explained, 65% indicated a willingness to participate. Providers were the most common source of health information. Use of Internet for health information, trust in health information, and higher education each independently increased the odds of clinical trial awareness, but obtaining information from providers did not. Contacting the Cancer Information Service and psychosocial factors were each independently associated with intent to join a clinical trial, while demographic factors were not. Information channels such as the Internet may be effective in conveying clinical trial information to Latinos. Providers being cited as the most common source of health information but not being associated with knowledge about or intent to participate in trials suggests a missed opportunity for communication to this population. | Male | Humans | Patient Participation | Young Adult | OK |
| Amiram | Gafni |  |  |  |  | Health Expectations: An International Journal of Public Participation in Health Care & Health Policy | 2014-04-00 avril 2014 | Context: Promoting patient participation in treatment decision making is of increasing interest to researchers, clinicians and policy makers. Decision aids (DAs) are advocated as one way to help achieve this goal. Despite their proliferation, there has been little agreement on criteria or standards for evaluating these tools. To fill this gap, an international collaboration of researchers and others interested in the development, content and quality of DAs have worked over the past several years to develop a checklist and, based on this checklist, an instrument for determining whether any given DA meets a defined set of quality criteria. Objective/Methods: In this paper, we offer a framework for assessing the conceptual clarity and evidence base used to support the development of quality criteria/standards for evaluating DAs. We then apply this framework to assess the conceptual clarity and evidence base underlying the International Patient Decision Aids Standards (IPDAS) checklist criteria for one of the checklist domains: how best to present in DAs probability information to patients on treatment benefits and risks. Conclusion: We found that some of the central concepts underlying the presenting probabilities domain were not defined. We also found gaps in the empirical evidence and theoretical support for this domain and criteria within this domain. Finally, we offer suggestions for steps that should be undertaken for further development and refinement of quality standards for DAs in the future. (PsycINFO Database Record (c) 2014 APA, all rights reserved). (journal abstract) | decision aids | Decision Making | Patient Participation | Client Participation | Doublon |
| Amiram | Gafni |  |  |  |  | Health Expectations: An International Journal of Public Participation in Health Care and Health Policy | 2014-04-00 Apr 2014 | CONTEXT: Promoting patient participation in treatment decision making is of increasing interest to researchers, clinicians and policy makers. Decision aids (DAs) are advocated as one way to help achieve this goal. Despite their proliferation, there has been little agreement on criteria or standards for evaluating these tools. To fill this gap, an international collaboration of researchers and others interested in the development, content and quality of DAs have worked over the past several years to develop a checklist and, based on this checklist, an instrument for determining whether any given DA meets a defined set of quality criteria. OBJECTIVE/METHODS: In this paper, we offer a framework for assessing the conceptual clarity and evidence base used to support the development of quality criteria/standards for evaluating DAs. We then apply this framework to assess the conceptual clarity and evidence base underlying the International Patient Decision Aids Standards (IPDAS) checklist criteria for one of the checklist domains: how best to present in DAs probability information to patients on treatment benefits and risks. CONCLUSION: We found that some of the central concepts underlying the presenting probabilities domain were not defined. We also found gaps in the empirical evidence and theoretical support for this domain and criteria within this domain. Finally, we offer suggestions for steps that should be undertaken for further development and refinement of quality standards for DAs in the future. | Decision Support Techniques | Humans | Patient Participation | Evidence-based medicine | OK |
| Kate | Wilson | Sandra G. | García | Freddy | Tinajeros | Sexually Transmitted Infections | 2011-08-00 Aug 2011 | OBJECTIVE: Assess the feasibility and acceptability of a patient-led syphilis partner notification strategy among pregnant women with syphilis, their male partners and treatment completion in Bolivia. METHODS: In four provinces, women who had tested positive for syphilis while attending antenatal care visits were recruited to receive a partner notification (PN) intervention on how to notify partners of their positive diagnosis and encourage them to get tested/treated. All women who completed PN counselling and notified their male partners completed self-administered questionnaires regarding PN experiences. Sociodemographic characteristics associated with notification and partner treatment completion were assessed using bivariate and multivariate analyses. RESULTS: 144 women and 137 male partners participated; 78% women notified their partners. No women characteristics were significantly associated with PN. Significantly more male partners (85%) who were notified by women completed syphilis treatment (p<0.05) compared to those notified by providers (66%). In multivariate analysis, men notified by female partners had a threefold greater odds of treatment completion compared to men who had not been notified by partners or by someone else (ie, healthcare worker) (OR 3.45, 95% CI 1.21 to 9.90). 86% of women and 80% of men completed syphilis treatment. Our results suggest that lack of time was considered a barrier to care among men who did not complete treatment. CONCLUSION: A patient-led partner notification strategy for pregnant women and their male partners appears to be feasible and acceptable, providing evidence for larger-scale effectiveness studies to improve male partner treatment compliance. | Male | Humans | Patient Participation | Patient Satisfaction | OK |
|  |  |  |  |  |  | Health Communication | 2012-01-00 janvier 2012 | This study examined the relationship between patient participation and recall of treatment recommendations among a sample of 96 patients at two primary care medical clinics. Using multilevel modeling analysis, the results of this study indicated a significant positive correlation (t(81)=8.84, p &lt; .0001) between patients’ participation and their ability to recall treatment recommendations discussed with their physicians. The results presented here suggest that patients who take a more active role in medical interviews may have an increased ability to recall important information discussed during these interactions. This is of utmost importance because the ability to recall treatment recommendations is an important predictor of health outcomes. (PsycINFO Database Record (c) 2012 APA, all rights reserved). (journal abstract) | Patient Participation | Client Participation | Treatment | Primary Health Care | Doublon |
|  |  |  |  |  |  | Health Communication | 2012-00-00 2012 | This study examined the relationship between patient participation and recall of treatment recommendations among a sample of 96 patients at two primary care medical clinics. Using multilevel modeling analysis, the results of this study indicated a significant positive correlation (t(81)= 8.84, p < .0001) between patients' participation and their ability to recall treatment recommendations discussed with their physicians. The results presented here suggest that patients who take a more active role in medical interviews may have an increased ability to recall important information discussed during these interactions. This is of utmost importance because the ability to recall treatment recommendations is an important predictor of health outcomes. | Male | Humans | Patient Participation | Female | OK |
| Winette T. A. | van der Graaf | Peep F. M. | Stalmeier |  |  | BMC medical informatics and decision making | 2011-00-00 2011 | BACKGROUND: There is a continuing debate on the desirability of informing patients with cancer and thereby involving them in treatment decisions. On the one hand, information uptake may be hampered, and additional stress could be inflicted by involving these patients. On the other hand, even patients with advanced cancer desire information on risks and prognosis. To settle the debate, a decision aid will be developed and presented to patients with advanced disease at the point of decision making. The aid is used to assess the amount of information desired. Factors related to information desire are explored, as well as the ability of the medical oncologist to judge the patient's information desire. The effects of the information on patient well-being are assessed by comparing the decision aid group with a usual care group. METHODS/DESIGN: This study is a randomized controlled trial of patients with advanced colorectal, breast, or ovarian cancer who have started treatment with first-line palliative chemotherapy. The trial will consist of 100 patients in the decision aid group and 70 patients in the usual care group. To collect complete data of 170 patients, 246 patients will be approached for the study. Patients will complete a baseline questionnaire on sociodemographic data, well-being measures, and psychological measures, believed to predict information desire. The medical oncologist will judge the patient's information desire. After disease progression is diagnosed, the medical oncologist offers the choice between second-line palliative chemotherapy plus best supportive care (BSC) and BSC alone. Randomization will take place to determine whether patients will receive usual care (n = 70) or usual care and the decision aid (n = 100). The aid offers information about the potential risks and benefits of both treatment options, in terms of adverse events, tumour response, and survival. Patients decide for each item whether they desire the information or not. Two follow-up questionnaires will evaluate the effect of the decision aid. DISCUSSION: This study attempts to settle the debate on the desirability of informing patients with cancer. In contrast to several earlier studies, we will actually deliver information on treatment options to patients at the point of decision making. | Decision Making | Decision Support Techniques | Humans | Patient Participation | OK |
|  |  |  |  |  |  | The Journal of Ambulatory Care Management | 2010-09-00 2010 Jul-Sep | Strengthening patients' meaningful involvement enhances the results of health-related projects and contributes toward patient-centered, equitable health care across the European Union (EU). Although current EU health policy reflects this premise, a concerted strategy to achieve patient involvement is, however, missing. European Patients' Forum-led Value+ project has represented in this respect the first effort ever made to produce an overarching EU-wide analysis of current practices and trends regarding patient involvement in EU health-related projects. Through the extensive use of qualitative, case-based research methods and effective exchange of experiences among key stakeholders, Value+ made an effective contribution to raising awareness about this important issue. | Humans | Patient Participation | Public health | health policy | OK |
| Joanne B. | Clovis | Mary E. | McNally | Mark J. | Filiaggi | Gerodontology | 2012-06-00 Jun 2012 | OBJECTIVES: To examine predictors of participation and to describe the methodological considerations of conducting a two-stage population-based oral health survey. METHODS: An observational, cross-sectional survey (telephone interview and clinical oral examination) of community-dwelling adults aged 45-64 and ≥65 living in Nova Scotia, Canada was conducted. RESULTS: The survey response rate was 21% for the interview and 13.5% for the examination. A total of 1141 participants completed one or both components of the survey. Both age groups had higher levels of education than the target population; the age 45-64 sample also had a higher proportion of females and lower levels of employment than the target population. Completers (participants who completed interview and examination) were compared with partial completers (who completed only the interview), and stepwise logistic regression was performed to examine predictors of completion. Identified predictors were as follows: not working, post-secondary education and frequent dental visits. CONCLUSION: Recruitment, communications and logistics present challenges in conducting a province-wide survey. Identification of employment, education and dental visit frequency as predictors of survey participation provide insight into possible non-response bias and suggest potential for underestimation of oral disease prevalence in this and similar surveys. This potential must be considered in analysis and in future recruitment strategies. | Male | Humans | Patient Participation | Female | OK |
| Sriharsha | Athreya |  |  |  |  | Cardiovascular and Interventional Radiology | 2013-04-00 Apr 2013 | PURPOSE: This study was designed to examine the best internet resources about uterine fibroid embolization (UFE) pertinent to medical trainees, radiologists, gynecologists, family physicians, and patients. METHODS: The terms "uterine fibroid embolization," "uterine fibroid embolization," and "uterine artery embolization" were entered into Google, Yahoo, and Bing search engines; the top 20 hits were assessed. The hits were categorized as organizational or nonorganizational. Additionally, 23 radiological and obstetrical organizations were assessed. The DISCERN instrument and Journal of the American Medical Association (JAMA) benchmarks (authorship, attribution, currency, disclosure) were used to assess the information critically. The scope, strength, weaknesses, and unique features were highlighted for the top five organizational and nonorganizational websites. RESULTS: A total of 203 websites were reviewed; 23 were removed in accordance with the exclusion criteria and 146 were duplicate websites, for a total of 34 unique sites. It was found that 35 % (12/34 websites) were organizational (family medicine, radiology, obstetrics/gynecology) and 65 % (22/34 websites) were nonorganizational (teaching or patient resources). The overall mean DISCERN score was 49.6 (10.7). Two-tailed, unpaired t test demonstrated no statistically significant difference between organizational and nonorganizational websites (p = 0.101). JAMA benchmarks revealed 44 % (15/34 websites) with authorship, 71 % (24/34 websites) with attribution, 68 % (23/34 websites) with disclosure, and 47 % (16/34 websites) with currency. CONCLUSIONS: The overall quality of websites for UFE is moderate, with important but not serious shortcomings. The best websites provided relevant information about the procedure, benefits/risks, and were interactive. DISCERN scores were compromised by sites failing to provide resources for shared decision-making, additional support, and discussing consequence of no treatment. JAMA benchmarks revealed lack of authorship and currency. | Humans | Female | Internet | Information Dissemination | OK |
| Rakesh M. | Suri | Martin B. | Leon | Vuyisile T. | Nkomo | Mayo Clinic Proceedings | 2014-10-00 Oct 2014 | Surgical aortic valve replacement had been the only definitive treatment of severe aortic stenosis before the availability of transcatheter valve technology. Historically, many patients with severe aortic stenosis had not been offered surgery, largely related to professional and patient perception regarding the risks of operation relative to anticipated benefits. Such patients have been labeled as "high risk" or "inoperable" with respect to their suitability for surgery. The availability of transcatheter aortic valve replacement affords a new treatment option for patients previously not felt to be optimal candidates for surgical valve replacement and allows for the opportunity to reexamine the methods for assessing operative risk in the context of more than 1 available treatment. Standardized risk assessment can be challenging because of both the imprecision of current risk scoring methods and the variability in ascertaining risk related to operator experience as well as local factors and practice patterns at treating facilities. Operative risk in actuality is not an absolute but represents a spectrum from very low to extreme, and the conventional labels of high risk and inoperable are incomplete with respect to their utility in clinical decision making. Moving forward, the emphasis should be on developing an individual assessment that takes into account procedure risk as well as long-term outcomes evaluated in a multidisciplinary fashion, and incorporating patient preferences and goals in a model of shared decision making. | Humans | risk assessment | Aortic Valve | Aortic Valve Stenosis | OK |
|  |  |  |  |  |  | Journal of Gerontological Nursing | 2010-05-00 May 2010 | Determination of a patient's capacity for medical decision making can be simple or complex, depending on the issue being considered; likewise, the degree of capacity required to make decisions varies with the complexity of the situation. A capacity examination is a focused and impartial evaluation of mental status and medical, surgical, psychiatric, and psychosocial histories. The examination is composed of several basic steps, beginning with an assessment of the patient's ability to communicate, followed by determination of his or her understanding of the proposed care, followed by the patient's rationale for decisions about that care. When a patient is deemed to lack capacity, mechanisms should be in place to ensure the patient receives the care needed. This article provides an overview of the concept of capacity, how it is determined, and how to proceed when the patient is found to lack it. | Decision Making | Humans | Patient Participation | Aged | OK |
| Neila | Chaari | Mohamed | Akrout | Hassen | Bacha | Genetic Testing and Molecular Biomarkers | 2013-09-00 Sep 2013 | Epidemiological studies suggest that cytogenetic biomarkers, such as micronuclei (MN) in peripheral blood lymphocytes may predict cancer risk because they indicate genomic instability. The objective of the present study was to evaluate the frequencies of MN and chromosome aberrations (CA) in peripheral blood lymphocytes of hospital workers exposed to ionizing radiation and healthy subjects. The study was conducted using peripheral blood lymphocytes from 30 workers from the radiology department and 30 from the cardiology department. This study included 27 healthy age- and sex-matched individuals as the control group. The assessment of chromosomal damage was carried out by the use of CA and micronucleus assays in peripheral lymphocytes. Our results show that CA and micronucleus frequencies were significantly higher among the exposed groups when compared to controls. Our finding of significant increase of CA and MN frequencies in peripheral lymphocytes in exposed workers indicates a potential cytogenetic hazard due to this exposure. The enhanced chromosomal damage of subjects exposed to genotoxic agents emphasizes the need to develop safety programs. | Humans | Patient Participation | Genome, Human | Exome | OK |
| Scott Y. H. | Kim |  |  |  |  | Psychosomatics: Journal of Consultation and Liaison Psychiatry | 2013-03-00 mars 2013 | Background: Decision-making capacity (DMC) assessments can have profound consequences for patients. With an aging population, an increasing emphasis on shared decision-making, and a rising number of potential medical interventions, the need for such assessments will continue to grow. Objective: To assess psychosomatic medicine clinicians’ training, experiences, and views about DMC assessments. Method: Online survey of members of the Academy of Psychosomatic Medicine (APM). Of 780 eligible members, 288 responded to the survey (36.9% response rate). Results: Approximately 1 in 6 psychiatric consultations are DMC assessments. Ninety percent of respondents reported that at least half of their capacity assessments involve patients older than 60 years. DMC assessments were seen as more challenging and time-consuming than other types of consultations; yet training in capacity evaluations was seen as suboptimal and half of respondents felt the evidence-base guiding DMC assessment is somewhat or much weaker than for other types of psychiatric consultations. In addition, the practice of capacity assessment seems to vary widely with no consistent approach among respondents. Respondents strongly endorsed multiple areas and topics for potential future research, indicating a desire for a stronger evidence-base. Conclusions: Members of the APM perceive capacity assessments as common and challenging. Yet they perceive having received subpar training with relatively weak evidence to guide their current practice. Future research should address these potential deficiencies, given the likelihood that DMC assessments will only become more common. (PsycINFO Database Record (c) 2013 APA, all rights reserved). (journal abstract) | Decision Making | Informed Consent | Professional Consultation | decision making capacity | Doublon |
| Scott Y. H. | Kim |  |  |  |  | Psychosomatics | 2013-04-00 2013 Mar-Apr | BACKGROUND: Decision-making capacity (DMC) assessments can have profound consequences for patients. With an aging population, an increasing emphasis on shared decision-making, and a rising number of potential medical interventions, the need for such assessments will continue to grow. OBJECTIVE: To assess psychosomatic medicine clinicians' training, experiences, and views about DMC assessments. METHOD: Online survey of members of the Academy of Psychosomatic Medicine (APM). Of 780 eligible members, 288 responded to the survey (36.9% response rate). RESULTS: Approximately 1 in 6 psychiatric consultations are DMC assessments. Ninety percent of respondents reported that at least half of their capacity assessments involve patients older than 60 years. DMC assessments were seen as more challenging and time-consuming than other types of consultations; yet training in capacity evaluations was seen as suboptimal and half of respondents felt the evidence-base guiding DMC assessment is somewhat or much weaker than for other types of psychiatric consultations. In addition, the practice of capacity assessment seems to vary widely with no consistent approach among respondents. Respondents strongly endorsed multiple areas and topics for potential future research, indicating a desire for a stronger evidence-base. CONCLUSIONS: Members of the APM perceive capacity assessments as common and challenging. Yet they perceive having received subpar training with relatively weak evidence to guide their current practice. Future research should address these potential deficiencies, given the likelihood that DMC assessments will only become more common. | Male | Decision Making | Humans | United States | OK |
| Ranapreet | Patel |  |  |  |  | The American Journal of Hospice & Palliative Care | 2011-02-00 Feb 2011 | OBJECTIVE: to evaluate satisfaction with inpatient hospice goals at the end of life and to determine steps for program improvement. STUDY DESIGN AND SETTING: prospective observational study of patients admitted to an inpatient Veterans Affairs Medical Center hospice unit, located in an urban setting with dedicated 16 hospice and 20 palliative care beds. Patients and their families are cared for by an interdisciplinary team. POPULATION: veteran population receiving care in an inpatient hospice unit whose goals are comfort care. INTERVENTION: two anonymous surveys of patient goals were distributed upon admission to hospice unit patients and families, who were asked to complete the first survey within 1 to 5 days of admission and the second survey 2 weeks after admission or later. OUTCOME MEASURE: patient and family centered end-of-life care outcomes, including meeting goals of care with desired symptom management, emotional support to the dying patient, coordinated care, shared decision making, and attending to the emotional needs of families. RESULTS: fifty patients were given surveys between June and September 2009. Response rate was 52% for the first survey and 36% for the second survey. Most important goals for 90% of veterans were control of symptoms and being with family. More than 90% of our patients' families, responding to our second survey, strongly agreed that these goals were achieved. We also identified other needs of hospice veterans, and family surveys showed that these were also accomplished. CONCLUSION: clarification and focus on goals of care improves satisfaction with end-of-life care. | Male | Humans | Patient Participation | Patient Satisfaction | OK |
| Ranapreet | Patel |  |  |  |  | American Journal of Hospice & Palliative Medicine | 2011-02-00 février 2011 | Objective: To evaluate satisfaction with inpatient hospice goals at the end of life and to determine steps for program improvement. Study design and setting: Prospective observational study of patients admitted to an inpatient Veterans Affairs Medical Center hospice unit, located in an urban setting with dedicated 16 hospice and 20 palliative care beds. Patients and their families are cared for by an interdisciplinary team. Population: Veteran population receiving care in an inpatient hospice unit whose goals are comfort care. Intervention: Two anonymous surveys of patient goals were distributed upon admission to hospice unit patients and families, who were asked to complete the first survey within 1 to 5 days of admission and the second survey 2 weeks after admission or later. Outcome measure: Patient and family centered end-of-life care outcomes, including meeting goals of care with desired symptom management, emotional support to the dying patient, coordinated care, shared decision making, and attending to the emotional needs of families. Results: Fifty patients were given surveys between June and September 2009. Response rate was 52% for the first survey and 36% for the second survey. Most important goals for 90% of veterans were control of symptoms and being with family. More than 90% of our patients’ families, responding to our second survey, strongly agreed that these goals were achieved. We also identified other needs of hospice veterans, and family surveys showed that these were also accomplished. Conclusion: Clarification and focus on goals of care improves satisfaction with end-of-life care. (PsycINFO Database Record (c) 2012 APA, all rights reserved). (journal abstract) | patients | palliative care | hospital programs | goals | Doublon |
| Hossein | Khalili | Mary Beth | Bezzina |  |  | The Journal of Continuing Education in the Health Professions | 2012-00-00 2012 | INTRODUCTION: Many health professionals believe they practice collaboratively. Providing insight into their actual level of collaboration requires a means to assess practice within health settings. This chapter reports on the development, testing, and refinement process for the Assessment of Interprofessional Team Collaboration Scale (AITCS). There is a paucity of literature and measurement tools addressing interprofessional collaborative team performance and the nature of effective teamwork processes and patient roles within collaborative teams. These gaps limit our knowledge about how health care teams form and function. Instruments are therefore needed to assess collaborative relationships. METHODS: The AITCS, with its 47 items within 4 subscales (partnership, cooperation, coordination, and shared decision making) and assessed on a 5-point Likert scale, was administered to a total of 125 practitioners from 7 health care teams practicing within a variety of settings, in 2 provinces in Canada. RESULTS: Principal components and factor analysis of data resulted in 37 items loading onto 3 factors, explaining 61.02% of the variance. The internal consistency estimates for reliability of each subscale ranged from 0.80 to 0.97, with an overall reliability of 0.98. Thus, the AITCS is a reliable and valid instrument. DISCUSSION: The psychometric analysis of this instrument supports its value in measuring collaboration within teams and when patients are included as team members. The AITCS can be applied to continuing professional education interventions to determine change over time. It has limitations to the Canadian context and within the settings where participants practiced. Further test and retest reliability and longitudinal study application is needed. | Male | Decision Making | Humans | Female | OK |
| Arianne | Teherani | Christy K. | Boscardin | George W. | Saba | Journal of General Internal Medicine | 2011-04-00 Apr 2011 | BACKGROUND: Shared decision-making, in which physicians and patients openly explore beliefs, exchange information, and reach explicit closure, may represent optimal physician-patient communication. There are currently no universally accepted methods to assess medical students' competence in shared decision-making. OBJECTIVE: To characterize medical students' shared decision-making with standardized patients (SPs) and determine if students' use of shared decision-making correlates with SP ratings of their communication. DESIGN: Retrospective study of medical students' performance with four SPs. PARTICIPANTS: Sixty fourth-year medical students. MEASUREMENTS: Objective blinded coding of shared decision-making quantified as decision moments (exploration/articulation of perspective, information sharing, explicit closure for a particular decision); SP scoring of communication skills using a validated checklist. RESULTS: Of 779 decision moments generated in 240 encounters, 312 (40%) met criteria for shared decision-making. All students engaged in shared decision-making in at least two of the four cases, although in two cases 5% and 12% of students engaged in no shared decision-making. The most commonly discussed decision moment topics were medications (n = 98, 31%), follow-up visits (71, 23%), and diagnostic testing (44, 14%). Correlations between the number of decision moments in a case and students' communication scores were low (rho = 0.07 to 0.37). CONCLUSIONS: Although all students engaged in some shared decision-making, particularly regarding medical interventions, there was no correlation between shared decision-making and overall communication competence rated by the SPs. These findings suggest that SP ratings of students' communication skill cannot be used to infer students' use of shared decision-making. Tools to determine students' skill in shared decision-making are needed. | Male | Decision Making | Humans | Physician-Patient Relations | OK |
| Arianne | Teherani | Christy K. | Boscardin | George W. | Saba | Journal of General Internal Medicine | 2011-04-00 avril 2011 | Background: Shared decision-making, in which physicians and patients openly explore beliefs, exchange information, and reach explicit closure, may represent optimal physician–patient communication. There are currently no universally accepted methods to assess medical students’ competence in shared decision-making. Objective: To characterize medical students’ shared decision-making with standardized patients (SPs) and determine if students’ use of shared decision-making correlates with SP ratings of their communication. Design: Retrospective study of medical students’ performance with four SPs. Participants: Sixty fourth-year medical students. Measurements: Objective blinded coding of shared decision-making quantified as decision moments (exploration/articulation of perspective, information sharing, explicit closure for a particular decision); SP scoring of communication skills using a validated checklist. Results: Of 779 decision moments generated in 240 encounters, 312 (40%) met criteria for shared decision-making. All students engaged in shared decision-making in at least two of the four cases, although in two cases 5% and 12% of students engaged in no shared decision-making. The most commonly discussed decision moment topics were medications (n=98, 31%), follow-up visits (71, 23%), and diagnostic testing (44, 14%). Correlations between the number of decision moments in a case and students’ communication scores were low (rho=0.07 to 0.37). Conclusion: Although all students engaged in some shared decision-making, particularly regarding medical interventions, there was no correlation between shared decision-making and overall communication competence rated by the SPs. These findings suggest that SP ratings of students’ communication skill cannot be used to infer students’ use of shared decision-making. Tools to determine students’ skill in shared decision-making are needed. (PsycINFO Database Record (c) 2012 APA, all rights reserved). (journal abstract) | Shared decision making | Decision Making | medical students | communication | Doublon |
| L. | McJames | J. N. | Mahlangu |  |  | Haemophilia: The Official Journal of the World Federation of Hemophilia | 2014-05-00 May 2014 | Effective healthcare delivery necessitates evaluation of the effect of interventions in the form of outcome assessment. Treatment effect includes measurement of how the patient feels, functions and survives following healthcare interventions. In haemophilia, which is a rare bleeding disorder, outcome assessment was characterized by a lack of validated outcome measurement tools and the challenges of hemophilia study design to collect outcome data. The aim of this communication is to share current thinking and, through practical examples, provide a state of the art practice in the assessment of hemophilia outcomes from a healthcare provider, patient/family and funder perspective. This discussion is timely and particularly relevant to the care of people with hemophilia on the eve of a number of novel hemophilia treatment products which are about to be licensed for use, specifically the long-acting factor VIII and factor IX concentrates. The first section by Dr Blanchet gives an overview of the tools currently available for assessment of structure/function, patient activities and patient participation in hemophilia healthcare delivery, pointing out the challenge of developing new tools and appropriate validation of currently available tools. The second section by Mr Brian O'Mahony emphasizes the essential collaboration and partnership between healthcare providers and people with hemophilia in collating the outcome data. In the third and final section, Mr Leigh McJames, gives a funder's perspective of the desirable outcomes of hemophilia care. | Humans | Patient Satisfaction | Australia | Capital Financing | OK |
| Richard F. | Ittenbach | J. William | Gaynor | Susan | Nicolson | Journal of Medical Ethics | 2010-02-00 Feb 2010 | OBJECTIVE: To assess parental permission for a neonate's research participation using the MacArthur competence assessment tool for clinical research (MacCAT-CR), specifically testing the components of understanding, appreciation, reasoning and choice. STUDY DESIGN: Quantitative interviews using study-specific MacCAT-CR tools. HYPOTHESIS: Parents of critically ill newborns would produce comparable MacCAT-CR scores to healthy adult controls despite the emotional stress of an infant with critical heart disease or the urgency of surgery. Parents of infants diagnosed prenatally would have higher MacCAT-CR scores than parents of infants diagnosed postnatally. There would be no difference in MacCAT-CR scores between parents with respect to gender or whether they did or did not permit research participation. PARTICIPANTS: Parents of neonates undergoing cardiac surgery who had made decisions about research participation before their neonate's surgery. METHODS: The MacCAT-CR. RESULTS: 35 parents (18 mothers; 17 fathers) of 24 neonates completed 55 interviews for one or more of three studies. Total scores: magnetic resonance imaging (mean 36.6, SD 7.71), genetics (mean 38.8, SD 3.44), heart rate variability (mean 37.7, SD 3.30). Parents generally scored higher than published subject populations and were comparable to published control populations with some exceptions. CONCLUSIONS: The MacCAT-CR can be used to assess parental permission for neonatal research participation. Despite the stress of a critically ill neonate requiring surgery, parents were able to understand study-specific information and make informed decisions to permit their neonate's participation. | Male | Decision Making | Humans | Patient Participation | OK |
| Bernard T. | Lee |  |  |  |  | Plastic and Reconstructive Surgery | 2014-12-00 Dec 2014 | BACKGROUND: Health literacy affects patient participation, compliance, and outcomes. Nearly half of American adults have inadequate functional health literacy. Identification and accommodation of patients with low literacy is an important goal of the American Medical Association, U.S. Department of Health and Human Services, and the Healthy People 2020 initiative. This study aims to assess plastic surgeons' perception of patient literacy. METHODS: A survey was distributed to American Society of Plastic Surgeons members about time devoted to patient counseling, use of techniques for evaluating and enhancing patient understanding, perception of level of education, and estimated literacy. Participation was voluntary and data were collected anonymously using an online survey tool. RESULTS: There were 235 participants in the survey (9.9 percent response rate). Patient literacy was most frequently assessed using their general impression (62.2 percent) and by asking patients about their employment (37.3 percent); 26.2 percent did not assess literacy. The majority of surgeons (62 percent) reported spending at least 20 minutes counseling new patients, and 37 percent reported spending more than 30 minutes. Lay terminology (94 percent) and pictures/diagrams (84.6 percent) were common patient education aids, whereas only 8.1 percent use teach-back methods. Plastic surgeons overestimated the level of education and reading level of their patients compared with national data. CONCLUSIONS: Formal assessment of health literacy is rarely performed, as most plastic surgeons use a general impression. Although plastic surgeons devote significant time to patient counseling, evidence-based communication methods, such as the teach-back method, are underused. Simple, directed questions can identify patients with low literacy skills, to accommodate their communication needs. | | | | | OK |
| Wim J. A. | van den Heuvel | |  |  |  | Rehabilitation Nursing: The Official Journal of the Association of Rehabilitation Nurses | 2014-04-07 Apr 7, 2014 | PURPOSE: In addition to the amount and intensity of rehabilitation interventions and the number of therapies, the degree of patient participation in physical rehabilitation activities is key. For this reason, adequate information regarding participation is necessary to evaluate patient performance. This article reviews instruments designed to assess participation in physical rehabilitation activities. DESIGN: Integrative review. METHODS: Pubmed, CINAHL, PsycInfo, Embase, and Cochrane Library database were searched for publications between January 1976 and July 2012. Secondary searches were also conducted and reference lists scanned for relevant publications. FINDINGS: Fourteen articles reporting on three instruments were found. They differ with regard to their underlying theoretical concepts. Each instrument was tested in medical inpatient rehabilitation settings. CONCLUSIONS/CLINICAL RELEVANCE: Each instrument appears to be useful for assessing specific aspects of patient participation in rehabilitation activities. More theoretical work is needed to clarify the underlying concepts as these instruments are not yet ready for clinical application. | | | | | OK |
| George C. | Patton | Lena A. | Sanci | Susan M. | Sawyer | The Journal of Adolescent Health: Official Publication of the Society for Adolescent Medicine | 2013-06-00 Jun 2013 | PURPOSE: To review the literature on young people's perspectives on health care with a view to defining domains and indicators of youth-friendly care. METHODS: Three bibliographic databases were searched to identify studies that purportedly measured young people's perspectives on health care. Each study was assessed to identify the constructs, domains, and indicators of adolescent-friendly health care. RESULTS: Twenty-two studies were identified: 15 used quantitative methods, six used qualitative methods and one used mixed methodology. Eight domains stood out as central to young people's positive experience of care. These were: accessibility of health care; staff attitude; communication; medical competency; guideline-driven care; age appropriate environments; youth involvement in health care; and health outcomes. Staff attitudes, which included notions of respect and friendliness, appeared universally applicable, whereas other domains, such as an appropriate environment including cleanliness, were more specific to particular contexts. CONCLUSION: These eight domains provide a practical framework for assessing how well services are engaging young people. Measures of youth-friendly health care should address universally applicable indicators of youth-friendly care and may benefit from additional questions that are specific to the local health setting. | Humans | Patient Participation | Patient Satisfaction | Adolescent | OK |
| Hubert | Robitaille | Hugues | Vaillancourt | Annie | Leblanc | Health Expectations: An International Journal of Public Participation in Health Care and Health Policy | 2013-03-04 Mar 4, 2013 | BACKGROUND: We have no clear overview of the extent to which health-care providers involve patients in the decision-making process during consultations. The Observing Patient Involvement in Decision Making instrument (OPTION) was designed to assess this. OBJECTIVE: To systematically review studies that used the OPTION instrument to observe the extent to which health-care providers involve patients in decision making across a range of clinical contexts, including different health professions and lengths of consultation. SEARCH STRATEGY: We conducted online literature searches in multiple databases (2001-12) and gathered further data through networking. INCLUSION CRITERIA: (i) OPTION scores as reported outcomes and (ii) health-care providers and patients as study participants. For analysis, we only included studies using the revised scale. DATA EXTRACTION: Extracted data included: (i) study and participant characteristics and (ii) OPTION outcomes (scores, statistical associations and reported psychometric results). We also assessed the quality of OPTION outcomes reporting. MAIN RESULTS: We found 33 eligible studies, 29 of which used the revised scale. Overall, we found low levels of patient-involving behaviours: in cases where no intervention was used to implement shared decision making (SDM), the mean OPTION score was 23 ± 14 (0-100 scale). When assessed, the variables most consistently associated with higher OPTION scores were interventions to implement SDM (n = 8/9) and duration of consultations (n = 8/15). CONCLUSIONS: Whatever the clinical context, few health-care providers consistently attempt to facilitate patient involvement, and even fewer adjust care to patient preferences. However, both SDM interventions and longer consultations could improve this. | | | | | OK |
| Michael J. | Ostacher | David J. | Miklowitz | Andrew A. | Nierenberg | Journal of Clinical Psychopharmacology | 2013-06-00 Jun 2013 | OBJECTIVES: We sought to understand the association of specific aspects of care satisfaction, such as patients' perceived relationship with their psychiatrist and access to their psychiatrist and staff, and therapeutic alliance with participants' likelihood to adhere to their medication regimens among patients with bipolar disorder. METHODS: We examined data from the multicenter Systematic Treatment Enhancement Program for Bipolar Disorder, an effectiveness study investigating the course and treatment of bipolar disorder. We expected that participants (n = 3037) with positive perceptions of their relationship with their psychiatrist and quality of psychopharmacologic care, as assessed by the Helping Alliance Questionnaire and Care Satisfaction Questionnaire, would be associated with better medication adherence. We utilized logistic regression models controlling for already established factors associated with poor adherence. RESULTS: Patients' perceptions of collaboration, empathy, and accessibility were significantly associated with adherence to treatment in individuals with bipolar disorder completing at least 1 assessment. Patients' perceptions of their psychiatrists' experience, as well as of their degree of discussing medication risks and benefits, were not associated with medication adherence. CONCLUSIONS: Patients' perceived therapeutic alliance and treatment environment impact their adherence to pharmacotherapy recommendations. This study may enable psychopharmacologists' practices to be structured to maximize features associated with greater medication adherence. | Male | Humans | Patient Participation | Patient Satisfaction | OK |
| David O. | Meltzer |  |  |  |  | JAMA internal medicine | 2013-07-08 Jul 8, 2013 | IMPORTANCE: Patient participation in medical decision making has been associated with improved patient satisfaction and health outcomes. However, there is little evidence concerning its effects on resource utilization. Patient participation in medical decision making has been hypothesized to decrease excess utilization but might be expected to increase utilization when other decision makers have incentives to reduce utilization, as under prospective payment systems for hospital care. OBJECTIVE: To examine the relationship between patient preferences for participation in medical decision making and health care utilization among hospitalized patients. DESIGN AND SETTING: Survey study in an academic research setting. PARTICIPANTS: A survey that included questions about preferences to receive medical information and to participate in medical decision making was administered to all patients admitted to the University of Chicago Medical Center general internal medicine service between July 1, 2003, and August 31, 2011, and completed by 21,754 (69.6%) of admitted patients. MAIN OUTCOMES AND MEASURES: The survey data were linked with administrative data, including length of stay and total hospitalization costs. We used generalized linear models to measure the association of patient preference for participation in decision making with length of stay and costs. RESULTS: The mean length of stay was 5.34 days, and the mean hospitalization costs were $14,576. While 96.3% of patients expressed a desire to receive information about their illnesses and treatment options, 71.1% of patients preferred to leave medical decision making to their physician. Preference to participate in decision making increased with educational level and with private health insurance. Compared with patients who had a strong desire to delegate decisions to their physician, patients who preferred to participate in decision making concerning their care had a 0.26-day (95% CI, 0.06-0.47 day) longer length of stay (P = .01) and $865 (95% CI, $155-$1575) higher total hospitalization costs (P = .02). CONCLUSIONS AND RELEVANCE: Patient preference to participate in decision making concerning their care may be associated with increased resource utilization among hospitalized patients. Variation in patient preference to participate in medical decision making and its effects on costs and outcomes in the presence of varying physician incentives deserve further examination. | Male | Decision Making | Humans | Patient Participation | OK |
| Seok Jin | Nam | Se Hyun | Ahn | Byeong Woo | Park | BMC health services research | 2010-00-00 2010 | BACKGROUND: Although some studies examined the association between shared decision-making (SDM) and type of breast cancer surgery received, it is little known how treatment decisions might be shaped by the information provided by physicians. The purpose of this study was to identify the associations between shared decision making (SDM) and surgical treatment received. METHODS: Questionnaires on SDM were administered to 1,893 women undergoing primary curative surgery for newly diagnosed stage 0-II localized breast cancer at five hospitals in Korea. Questions included being informed on treatment options and the patient's own opinion in decision-making. RESULTS: Patients more likely to undergo mastectomy were those whose opinions were respected in treatment decisions (adjusted odds ratio, aOR), 1.40; 95% confidence interval (CI), 1.14-1.72) and who were informed on chemotherapy (aOR, 2.57; CI, 2.20-3.01) or hormone therapy (aOR, 2.03; CI, 1.77-2.32). In contrast, patients less likely to undergo mastectomy were those who were more informed on breast surgery options (aOR, 0.34; CI, 0.27-0.42). In patients diagnosed with stage 0-IIa cancer, clinical factors and the provision of information on treatment by the doctor were associated with treatment decisions. In patients diagnosed with stage IIb cancer, the patient's opinion was more respected in treatment decisions. CONCLUSION: Our population-based study suggested that women's treatment decisions might be shaped by the information provided by physicians, and that women might request different information from their physicians based on their preferred treatment options. These results might need to be confirmed in other studies of treatment decisions. | Decision Making | Humans | Patient Participation | Questionnaires | OK |
| Solrun | Sigurdardottir | Juan Carlos | Arango-Lasprilla | Cecilie | Roe | Brain Injury | 2012-00-00 2012 | OBJECTIVE: To investigate associations between disability and employment 1 year after traumatic brain injury (TBI) using the International Classification of Functioning, Disability and Health (ICF) as a conceptual model. DESIGN AND METHODS: A prospective study including 93 patients with moderate-to-severe TBI (aged 16-55 year). Disability components of the ICF model (impairments, activity limitations and participation restrictions) and personal factors (age, gender, pre-injury employment status) were used as independent variables. The outcome measure was employment at 1 year post-injury categorized into unemployed and employed groups. RESULTS: Personal factors, impairments (brain injury severity, overall trauma severity and number of impaired body functions) and activity limitations (motor and cognitive abilities) accounted for 57% of the variance in employment outcome. Multivariate analyses showed that the probabilities of being employed 1 year post-injury were 95% lower for patients who were unemployed pre-injury (OR = 0.05), 74% lower for those with more severe brain injury (OR = 0.26) and 82% lower for those with more cognitive limitations (OR = 0.18). CONCLUSION: Rehabilitation professionals should take into account the importance of the ICF model when planning vocational rehabilitation interventions for individuals with TBI and focus on targeting modifiable aspects related to employment outcome, such as the individual's cognitive ability. | Male | Humans | Patient Participation | Questionnaires | OK |
| Hassan | Heidarnazhad | Saied | Bokaie | Mostafa | Moin | Archives of Iranian Medicine | 2011-09-00 Sep 2011 | BACKGROUND: Worldwide, numerous people of all ages and ethnicities experience asthma. The achievements of current medical regimens for patients frequently depend on three factors: 1) knowledge of patients regarding this disease, 2) patient's attitude about asthma, including willingness to collaborate with the therapeutic group for disease control and 3) patient's self-efficacy for controlling asthma. Therefore, this study examined the relationship between knowledge, attitudes, and self-efficacy with socio-economic factors in Iranian asthmatic patients during 2006-2008. METHODS: Participants consisted of 120 adults referred to Milad Hospital, Tehran, Iran during 2006 to 2008 whose physicians diagnosed their asthma. Socio-demographic factors such as sex, age, education level, occupation, marital status, family history of asthma, disease costs, and period of sickness were reviewed. Assessments of knowledge, attitudes and self-efficacy were performed by the Persian version of an international standard questionnaire (KASE-AQ). Data were analyzed by SPSS version 14. RESULTS: Among respondents, only 9 (7.5%) patients had good knowledge about asthma, 108 (90%) patients had a suitable attitude about their asthma and 103 (85.3%) patients had proper self-efficacy. We found a significant association between self-efficacy and attitude in asthmatic patients (P<0.05). CONCLUSION: Although specific knowledge about asthma is low, however, favorable attitudes toward asthma create opportunities to intervene and improve asthma management among patients. However, the use of educational tools depends on patients' educational levels. Therefore, we recommend elevating asthma knowledge. | Male | Humans | Patient Participation | Questionnaires | OK |
|  |  |  |  |  |  | Adapted physical activity quarterly: APAQ | 2011-07-00 Jul 2011 | This study examined interrelationships among athletic identity, sport participation, and psychological adjustment in a sample of people with spinal cord injury (SCI). Participants (N = 1,034) completed measures of athletic identity, life satisfaction, anxiety, depression, and demographic and sport participation variables. Current amount of weekly sport participation was positively related to athletic identity when statistically controlling for age, gender, and pre-SCI amount of weekly sport participation. Being able to practice one's favorite sport after SCI was associated with higher levels of athletic identity and better psychological adjustment. Team sport participants reported experiencing better psychological adjustment than individual sport participants did. The findings suggest that social factors are important in the link between sport participation and psychological adjustment in people with SCI. | Male | Humans | Patient Participation | Questionnaires | OK |
| George | Reed | Sherry | Pagoto | Rex | Graff | Vascular Medicine (London, England) | 2010-04-00 Apr 2010 | Among 355 peripheral arterial disease (PAD) patients with low density lipoprotein cholesterol (LDL-C) levels > or = 70 mg/dl, we assessed knowledge regarding optimal LDL levels and the importance of LDL-C-lowering therapy. We also assessed PAD participants' behaviors and attitudes regarding their engagement with their physician in treatment decisions for LDL-C lowering. The average baseline LDL-C level of participants was 103.4 mg/dl +/- 30.7 mg/dl. Seventy-six percent of participants were taking at least one cholesterol-lowering medication. Sixty-six percent were unable to define their optimal LDL-C. Only 47% strongly agreed that their own actions and decisions could reduce their LDL-C. Just 29.8% were aware that patients who request specific medications from their physician were more likely to receive them, and 16% had asked their physician whether they should be taking more cholesterol-lowering medication. These findings suggest that further study is needed to identify effective interventions to educate PAD patients and their physicians about the importance of cholesterol-lowering therapy and to encourage PAD patients to participate with their physician in decisions regarding cholesterol-lowering treatment. Clinical Trial Registration - URL: http://www.clinicaltrials.gov. Unique identifier: NCT00217919. | Male | Humans | Patient Participation | Questionnaires | OK |
| Clayton | Shamblin | Mulugeta | Gebregziabher | Gerard A. | Silvestri | Chest | 2013-12-00 Dec 2013 | BACKGROUND: Lung cancer (LC) is the leading cause of cancer-related death for veterans cared for by the US Veterans Health Administration. The LC burden among veterans is almost double that of the general population. Before implementation of an LC screening program, we set out to assess the role of beliefs and attitudes toward LC screening among veterans. METHODS: Veterans presenting to the Ralph H. Johnson VA Medical Center were invited to complete a self-administered survey. The survey comprised questions about demographics, smoking status, health status, and knowledge about LC and willingness to be screened. Responses from veteran ever and never smokers were compared. RESULTS: A total of 209 veterans completed the survey. Smokers were significantly (P &lt; .05) more likely than never smokers to be less educated, have a lower income, and report poorer health. Smokers were more likely than never smokers to have two or more comorbidities, which trended toward significance (P = .062). Smokers were more likely to have been told by a physician that they were at high risk for LC and to believe that they were at risk. Nearly all veterans surveyed (92.8%) would have a CT scan for LC screening, and 92.4% would have surgery for a screen-detected LC. CONCLUSIONS: Veterans are overwhelmingly willing to undergo screening for LC, and it seems that participation will not be a barrier to implementation of an LC screening program. The mortality benefit of LC screening, however, may not be generalizable to the veteran population because of a higher number of comorbid conditions. | Male | Humans | Patient Participation | Questionnaires | OK |
| C. C. | Michon | J. Y. | Hogrel | V. | Doppler | Revue Neurologique | 2013-09-00 2013 Aug-Sep | AIM: This study aimed to gain a better understanding of the psychological impact of participating in a clinical trial for patients with Pompe disease (Acid Maltase Deficiency). Attitudes and expectations of adult patients with neuromuscular diseases regarding medical trials are as yet unreported. In order to learn about the psychological consequences of participating in a clinical trial, we conducted a prospective assessment of patients with late-onset Pompe Disease, a rare genetic condition, for which no treatment had been available before. This psychological study was carried out as an ancillary study to the randomized double-blind placebo-controlled trial described elsewhere (van der Ploeg et al., 2010). SUBJECTS AND METHODS: We assessed patients (n=8) at inclusion, and at 12 and 18 months for six psychological dimensions: depression (Beck Depression Inventory, BDI), hopelessness (Beck Hopelessness Scale, BHS), anxiety (STAI A-B), quality of life (Whoqol-26), social adjustment (S.A.S-self-report) and locus of control (IPC Levenson). We produced a self-administered questionnaire in order to assess the attitudes, motivations and expectations of patients during the trial. RESULTS: At 12 months, mean social adjustment (SAS-SR, P=0.02) had improved, and at 18 months mean depression score had improved as well (BDI, P=0.03). The quality of life of patients (Whoqol-26) remained unchanged. Throughout the study, patients were more likely to have an internal locus of control than an external one (IPC Levenson). The self-administered questionnaire showed that patients' expectations were disproportionate compared to the medical information they had received starting the trial. For all patients, the first motivation for being enrolled in a clinical trial was "to help research", for half of them the motivation was to "improve their health". Whether patients believed to be part of one group or another (placebo or treatment) depended on their subjective perception of improvement during the trial. CONCLUSION: Given the small sample size, the conclusions of this study are preliminary. However, findings do suggest that there is a positive psychological impact of participating in a treatment trial. Moreover, the patients' reactions upon unblinding have led us to recommend that patients be asked whether they would like their group assignation disclosed to them or not. | Male | Humans | Patient Participation | Questionnaires | OK |
| H. | Silverman |  |  |  |  | Eastern Mediterranean Health Journal = La Revue De Santé De La Méditerranée Orientale = Al-Majallah Al-Ṣiḥḥīyah Li-Sharq Al-Mutawassiṭ | 2014-02-00 Feb 2014 | The attitudes of dental patients towards participation in research and the independent socioeconomic factors associated with these attitudes are not known. A self-administered questionnaire was distributed to 120 patients attending dental clinics in Amman, Jordan. For patients who had previously participated in research (n = 28), motivating factors for most (> 75%) included a desire to help others and to advance science. Most respondents (81.7%) showed interest in participating in questionnaire-based research, but fewer would participate in invasive research, e.g. biopsies (22.0%) and drilling teeth (21.2%). Reasons given for not participating in research included fear of infectious diseases (71.3%) and pain (62.1%). Factors considered important in enhancing research participation included being asked to give informed consent (98.2%), fairness in selection of participants (97.3%) and the prospect of humanitarian benefit (96.5%). Sex and education level were significantly associated with patients' perceptions for several of items. To enhance recruitment, researchers should be aware of people's perspectives regarding participation in research. | Male | Humans | Patient Participation | Questionnaires | OK |
| Caterina | Masino | Joseph A. | Cafazzo | Jan | Barnsley | Journal of Medical Internet Research | 2010-00-00 2010 | BACKGROUND: Mobile phone-based remote patient monitoring systems have been proposed for heart failure management because they are relatively inexpensive and enable patients to be monitored anywhere. However, little is known about whether patients and their health care providers are willing and able to use this technology. OBJECTIVE: The objective of our study was to assess the attitudes of heart failure patients and their health care providers from a heart function clinic in a large urban teaching hospital toward the use of mobile phone-based remote monitoring. METHODS: A questionnaire regarding attitudes toward home monitoring and technology was administered to 100 heart failure patients (94/100 returned a completed questionnaire). Semi-structured interviews were also conducted with 20 heart failure patients and 16 clinicians to determine the perceived benefits and barriers to using mobile phone-based remote monitoring, as well as their willingness and ability to use the technology. RESULTS: The survey results indicated that the patients were very comfortable using mobile phones (mean rating 4.5, SD 0.6, on a five-point Likert scale), even more so than with using computers (mean 4.1, SD 1.1). The difference in comfort level between mobile phones and computers was statistically significant (P< .001). Patients were also confident in using mobile phones to view health information (mean 4.4, SD 0.9). Patients and clinicians were willing to use the system as long as several conditions were met, including providing a system that was easy to use with clear tangible benefits, maintaining good patient-provider communication, and not increasing clinical workload. Clinicians cited several barriers to implementation of such a system, including lack of remuneration for telephone interactions with patients and medicolegal implications. CONCLUSIONS: Patients and clinicians want to use mobile phone-based remote monitoring and believe that they would be able to use the technology. However, they have several reservations, such as potential increased clinical workload, medicolegal issues, and difficulty of use for some patients due to lack of visual acuity or manual dexterity. | Male | Humans | Patient Participation | Questionnaires | OK |
| J. | Najita | T. | Openshaw | K. | Krag | Annals of oncology: official journal of the European Society for Medical Oncology / ESMO | 2013-07-00 Jul 2013 | BACKGROUND: Research studies involving human tissue are increasingly common. However, patients' attitudes toward research biopsies are not well characterized, particularly when the biopsies are carried out outside the context of therapeutic trials. PATIENTS AND METHODS: One hundred sixty patients with metastatic breast cancer (MBC) from two academic (n = 80) and two community (n = 80) hospitals completed a 29-item self-administered survey to evaluate their willingness to consider providing research purposes only biopsies (RPOBs) (as a stand-alone procedure) and additional biopsies (ABs) (additional needle passes at the time of a clinically indicated biopsy). RESULTS: Eighty-two (51%) of 160 patients would consider having RPOBs, of which 42 (53%) and 40 (50%) patients were from academic and community hospitals, respectively. Patients who had more prior biopsies were less likely to consider RPOBs (RR = 0.6, 95% CI: 0.4-1.0, P = 0.03). Of 160 patients, 115 (72%) patients would consider having ABs. Of these, 64 (80%) and 51 (64%) patients from academic and community hospitals, respectively, would consider ABs (RR = 1.2, 95% CI: 1.0-1.5, P = 0.03). CONCLUSIONS: Many patients with MBC in both academic and community settings report willingness to consider undergoing biopsies for research. Further research is needed to understand ethical, logistical and provider-based barriers to broader participation in such studies. | Humans | Patient Participation | Questionnaires | Female | OK |
| W. Wolfgang | Fleischhacker | Silvana | Galderisi | Janusz K. | Rybakowski | Social Psychiatry and Psychiatric Epidemiology | 2011-02-00 Feb 2011 | BACKGROUND: Relatively few studies have examined how patients with schizophrenia and depression view psychiatric research and what influences their readiness to participate. METHODS: A total of 763 patients (48% schizophrenia, 52% depression) from 7 European countries were examined using a specifically designed self-report questionnaire ["Hamburg Attitudes to Psychiatric Research Questionnaire" (HAPRQ)]. RESULTS: Most patients (98%) approved of psychiatric research, in general, at least "a little". There was a tendency to approve psychosocial rather than biological research topics (e.g. research on the role of the family by 91% of patients compared to 79% in genetics). Reasons to participate were mainly altruistic. Only a minority (28%) considered monetary incentives important. Patients wanted extensive background information and a feedback of the results; both were significantly more expressed by schizophrenia as compared to depressive patients, although these findings need to be interpreted with care because of age and gender differences between the diagnostic groups. CONCLUSION: While patients expressed discerning views of psychiatric research, only few differences were apparent between the two diagnostic groups. Patients' research priorities are not the same as those of many professionals and funding bodies. Their demonstrated critical appraisal should inform future research ensuring an increased patient role in the research process. | Male | Humans | Patient Participation | Questionnaires | OK |
| Lilisbeth | Perestelo-Pérez | Jeanette | Pérez-Ramos | Pedro | Serrano-Aguilar | BMC psychiatry | 2012-00-00 2012 | BACKGROUND: Concordance and Shared Decision-Making (SDM) are considered measures of the quality of care that improves communication, promotes patient participation, creates a positive relationship with the healthcare professional, and results in greater adherence with the treatment plan. METHODS: This study compares the attitudes of 225 mental health professionals (125 psychiatrists and 100 psychiatry registrars) and 449 psychiatric outpatients towards SDM and concordance in medicine taking by using the "Leeds Attitude toward Concordance Scale" (LATCon). RESULTS: The internal consistency of the scale was good in all three samples (Cronbach's α: patients = 0.82, psychiatrists = 0.76, and registrars = 0.82). Patients scored significantly lower (1.96 ± 0.48) than professionals (P < .001 in both cases), while no statistically significant differences between psychiatrists (2.32 ± 0.32) and registrars (2.23 ± 0.35) were registered; the three groups showed a positive attitude towards concordance in most indicators. Patients are clearly in favor of being informed and that their views and preferences be taken into account during the decision-making process, although they widely consider that the final decision must be the doctor's responsibility. Among mental health professionals, the broader experience provides a greater conviction of the importance of the patient's decision about treatment. CONCLUSIONS: We observed a positive attitude towards concordance in the field of psychotropic drugs prescription both in professionals and among patients, but further studies are needed to address the extent to which this apparently accepted model is reflected in the daily practice of mental health professionals. | Male | Decision Making | Humans | Patient Participation | OK |
| Michael | Siegrist |  |  |  |  | Medical Teacher | 2011-00-00 2011 | BACKGROUND: Health professionals' attitudes toward shared decision-making (SDM) are an important facilitator of SDM, but information on these attitudes is limited. AIMS: The purpose of this study is to examine attitudes, education and practices around SDM and risk communication in residents and their teachers. METHOD: A questionnaire was mailed to residents in Swiss hospitals in postgraduate medical training programs assessing risk communication education and SDM. In an Internet survey, teachers of the medical training programs answered questions on SDM and risk communication practices. Data were analyzed with ANOVAs and paired samples t-tests. RESULTS: Significant differences in residents' and teachers' opinions regarding SDM were found between specialties and number of residents in a residency (1-3, 4-10, ≥11 residents). Teachers showed a high use of verbal risk communication. Neither residents nor teachers expressed a strong feeling that they lacked the time for decision-making. Residents were significantly more negative about the ability of patients to participate in decision-making compared to their teachers. CONCLUSIONS: As residents are more negative about SDM compared to teachers and teachers do not always use the preferred and best methods for risk communication, more education for teachers and residents is needed to improve communication practices in the future. | Decision Making | Humans | Patient Participation | Questionnaires | OK |
| Petra | Buettner | Peter | Piliouras | Zulfiquer | Otty | Asia-Pacific Journal of Clinical Oncology | 2011-03-00 Mar 2011 | AIM: To assess the knowledge of randomized clinical trials and willingness and barriers to participation among rural, remote and regional cancer patients of North Queensland. METHODS: A survey was conducted in medical oncology outpatient clinics at the Townsville and Mt Isa hospitals on patients, following their informed consent, using questionnaires. Rurality was defined according to the rural remote and metropolitan area classification. RESULTS: Of the 180 patients approached, 178 participated. The median distance to the regional trial center for rural participants was 180 km (range 80-1300 km). 45.4% lived in rural or remote areas and the rest lived in Townsville, a regional metropolitan center. Their overall knowledge was low, with a median knowledge score of 3 (inter-quartile ranges n=2.5). For randomized controlled trials there were no significant relationships between willingness to participate and rurality or education level (P=0.981). Cost of travel (41.1% rural or remote; 23.5% regional; P<0.001) and the need for family or friends to accompany them (38.9% rural or remote; 24.1% regional, P=0.021) were more important for rural/remote than regional patients as factors affecting participation. CONCLUSION: Rural and remote patients are as interested in participating in randomized clinical trials as regional patients. Their knowledge of trials is poor and education earlier in the consultations is needed. Since cost of travel and the need for family members to accompany them are important for rural patients trial budgets should include the cost of travel to encourage participation. | Male | Humans | Patient Participation | Questionnaires | OK |
| Mark | Boyko | Jonathan | Berkowitz | Betty | Calam | Canadian Family Physician Médecin De Famille Canadien | 2012-04-00 Apr 2012 | OBJECTIVE: To assess outpatient understanding of and previous experiences with do-not-resuscitate (DNR) orders and to gauge patient preferences with respect to DNR discussions. DESIGN: Cross-sectional, self-administered survey. SETTING: Four urban primary care physician offices in Vancouver, BC. PARTICIPANTS: A total of 429 consecutive patients 40 years of age and older presenting for routine primary care between March and May 2009. MAIN OUTCOME MEASURES: Awareness of, knowledge about, and experiences with DNR decisions; when, where, and with whom patients wished to discuss DNR decisions; and differences in responses by sex, age, and ethnicity, assessed using χ² tests of independence. RESULTS: The response rate was 90%, with 386 of 429 patients completing the surveys. Most (84%) respondents had heard of the terms do not resuscitate or DNR. Eighty-six percent chose family physicians as among the people they most preferred to discuss DNR decisions with; 56% believed that initial DNR discussions should occur while they were healthy; and 46% thought the discussion should take place in the office setting. Of those who were previously aware of DNR orders, 70% had contemplated DNR for their own care, with those older than 60 years more likely to have done so (P = .02); however, only 8% of respondents who were aware of DNR orders had ever discussed the subject with a health care provider. Few patients (16%) found this topic stressful. CONCLUSION: Most respondents were well informed about the meaning of DNR, thought DNR discussions should take place when patients were still healthy, preferred to discuss DNR decisions with family physicians, and did not consider the topic stressful. Yet few respondents reported having had a conversation about DNR decisions with any health care provider. Disparity between patient preferences and experiences suggests that family physicians can and should initiate DNR discussions with younger and healthier patients. | Male | Decision Making | Humans | Patient Participation | OK |
|  |  |  |  |  |  | Acta Paediatrica (Oslo, Norway: 1992) | 2011-01-00 Jan 2011 | AIM: To determine attitudes of paediatric consultants and trainees towards immediate care of babies born at 22-24 weeks gestation. METHODS: A questionnaire was sent to one consultant and one trainee within each of 63 neonatal units in South East England. RESULTS: Response rate was 90% (111/123). At 22 weeks gestation, 51% of all respondents would not attend the delivery. At 23 weeks gestation, 63% of respondents would advise against resuscitation during antenatal counselling but 45% would resuscitate despite a parental request for comfort care. Forty-one percent of respondents would provide comfort care for a 23 week gestation baby born in poor condition. If required, 53% of respondents would use chest compressions and/or adrenaline at birth of a 24 week gestation infant (38% consultants, 54% trainees; p = 0.05). More experienced and older paediatricians were more likely to attend 22 week deliveries and be comfortable with parental requests for resuscitation. Fifty-four percent of units had written guidelines on care of these babies. Significantly more consultants (67%) than trainees (25%) correctly classified neonatal deaths. CONCLUSIONS: Paediatricians with greater experience and age demonstrated more shared decision-making with parents. Nearly half of paediatricians would defy parental wishes at 23 weeks gestation, contrary to ethical recommendations (Nuffield Council on Bioethics, http://www.nuffieldbioethics.org). | Time Factors | Humans | Questionnaires | Age Factors | OK |
| Roland | Grad | Michel | Cauchon | Matthew | Greenway | BMC medical informatics and decision making | 2012-00-00 2012 | BACKGROUND: Decision Boxes are summaries of the most important benefits and harms of health interventions provided to clinicians before they meet the patient, to prepare them to help patients make informed and value-based decisions. Our objective is to explore the barriers and facilitators to using Decision Boxes in clinical practice, more precisely factors stemming from (1) the Decision Boxes themselves, (2) the primary healthcare team (PHT), and (3) the primary care practice environment. METHODS/DESIGN: A two-phase mixed methods study will be conducted. Eight Decision Boxes relevant to primary care, and written in both English and in French, will be hosted on a website together with a tutorial to introduce the Decision Box. The Decision Boxes will be delivered as weekly emails over a span of eight weeks to clinicians of PHTs (family physicians, residents and nurses) in five primary care clinics located across two Canadian provinces. Using a web-questionnaire, clinicians will rate each Decision Box with the Information Assessment Method (cognitive impacts, relevance, usefulness, expected benefits) and with a questionnaire based on the Theory of Planned Behavior to study the determinants of clinicians' intention to use what they learned from that Decision Box in their patient encounter (attitude, social norm, perceived behavioral control). Web-log data will be used to monitor clinicians' access to the website. Following the 8-week intervention, we will conduct semi-structured group interviews with clinicians and individual interviews with clinic administrators to explore contextual factors influencing the use of the Decision Boxes. Data collected from questionnaires, focus groups and individual interviews will be combined to identify factors potentially influencing implementation of Decision Boxes in clinical practice by clinicians of PHTs. CONCLUSIONS: This project will allow tailoring of Decision Boxes and their delivery to overcome the specific barriers identified by clinicians of PHTs to improve the implementation of shared decision making in this setting. | Humans | Questionnaires | Primary Health Care | Professional-Patient Relations | OK |
| Anik | Giguère | Marie-Pierre | Gagnon | Jeremy | Grimshaw | Implementation science: IS | 2011-00-00 2011 | BACKGROUND: In North America, acute respiratory infections are the main reason for doctors' visits in primary care. Family physicians and their patients overuse antibiotics for treating acute respiratory infections. In a pilot clustered randomized trial, we showed that DECISION+, a continuing medical education program in shared decision making, has the potential to reduce the overuse of antibiotics for treating acute respiratory infections. DECISION+ learning activities consisted of three interactive sessions of three hours each, reminders at the point of care, and feedback to doctors on their agreement with patients about comfort with the decision whether to use antibiotics. The objective of this study is to identify the barriers and facilitators to physicians' participation in DECISION+ with the goal of disseminating DECISION+ on a larger scale. METHODS/DESIGN: This descriptive study will use mixed methods and retrospective and prospective components. All analyses will be based on an adapted version of the Ottawa Model of Research Use. First, we will use qualitative methods to analyze the following retrospective data from the pilot study: the logbooks of eight research assistants, the transcriptions of 15 training sessions, and 27 participant evaluations of the DECISION+ training sessions. Second, we will collect prospective data in semi-structured focus groups composed of family physicians to identify barriers and facilitators to the dissemination of a future training program similar to DECISION+. All 39 family physicians exposed to DECISION+ during the pilot project will be eligible to participate. We will use a self-administered questionnaire based on Azjen's Theory of Planned Behaviour to assess participants' intention to take part in future training programs similar to DECISION+. DISCUSSION: Barriers and facilitators identified in this project will guide modifications to DECISION+, a continuing medical education program in shared decision making regarding the use of antibiotics in acute respiratory infections, to facilitate its dissemination in primary care on a large scale. Our results should help continuing medical educators develop a continuing medical education program in shared decision making for other clinically relevant topics. This will help optimize clinical decisions in primary care. | Decision Making | Humans | Patient Participation | Questionnaires | OK |
| Erica | Scholten | Lotty | Hooft | Erja | Portegijs | Journal of the American Geriatrics Society | 2013-08-00 Aug 2013 | | Male | Humans | Patient Participation | Questionnaires | OK |
| Donna L. | LaVallie | Dedra | Buchwald |  |  | The Journal of Rural Health: Official Journal of the American Rural Health Association and the National Rural Health Care Association | 2013-00-00 2013 | PURPOSE: American Indians and Alaska Natives (AIs/ANs) have some of the highest cancer-related mortality rates of all US racial and ethnic groups, but they are underrepresented in clinical trials. We sought to identify factors that influence willingness to participate in cancer clinical trials among AI/AN tribal college students, and to compare attitudes toward clinical trial participation among these students with attitudes among older AI/AN adults. METHODS: Questionnaire data from 489 AI/AN tribal college students were collected and analyzed along with previously collected data from 112 older AI/AN adults. We examined 10 factors that influenced participation in the tribal college sample, and using chi-square analysis and these 10 factors, we compared attitudes toward research participation among 3 groups defined by age: students younger than 40, students 40 and older, and nonstudent adults 40 and older. FINDINGS: About 80% of students were willing to participate if the study would lead to new treatments or help others with cancer in their community, the study doctor had experience treating AI/AN patients, and they received payment. Older nonstudent adults were less likely to participate on the basis of the doctor's expertise than were students (73% vs 84%, P = .007), or if the study was conducted 50 miles away (24% vs 41%, P= .001). CONCLUSIONS: Finding high rates of willingness to participate is an important first step in increasing participation of AIs/ANs in clinical trials. More information is needed on whether these attitudes influence actual behavior when opportunities to participate become available. | Male | Humans | Patient Participation | Questionnaires | OK |
|  |  |  |  |  |  | Nephrology, Dialysis, Transplantation: Official Publication of the European Dialysis and Transplant Association - European Renal Association | 2012-03-00 Mar 2012 | BACKGROUND: Physical inactivity is a strong predictor of mortality in patients with end-stage renal disease and is associated with poor physical functioning. Patients with end-stage renal disease are inactive even compared to sedentary individuals without kidney disease. We sought to identify patient barriers to physical activity. METHODS: Adult patients on hemodialysis in the San Francisco Bay Area were recruited and asked to complete a study survey composed of questions about self-reported level of physical functioning, physical activity participation, patient physical activity preference and barriers to physical activity. Univariate and multivariable linear regression analyses were performed to study the association between barriers to physical activity and participation in physical activity. RESULTS: A total of 100 patients participated in the study, the majority of whom were male (73%), with a mean age of 60 ± 15 years. Twenty-seven percent identified themselves as white, 30% black and 21% Hispanic. The majority of participants strongly agreed that a sedentary lifestyle was a health risk (98%) and that increasing exercise was a benefit (98%). However, 92% of participants reported at least one barrier to physical activity. The most commonly reported barriers were fatigue on dialysis days and non-dialysis days (67 and 40%, respectively) and shortness of breath (48%). In multivariate analysis, a greater number of reported barriers was associated with lower levels of physical activity (P < 0.02). Post-dialysis fatigue was not associated with differences in activity level in multivariate analysis. Lack of motivation was associated with less physical activity. Endorsement of too many medical problems and not having enough time on dialysis days were also associated with less activity in adjusted analysis. CONCLUSION: We have identified a number of barriers to physical activity that can be addressed in studies aimed at increasing levels of physical activity. Inconsistent with nephrologists' reported assumptions, dialysis patients were interested in physical activity. | Male | Humans | Patient Participation | Female | OK |
| Laura | Schiesari | Véronique | Kolly | Thomas V. | Perneger | PloS One | 2011-00-00 2011 | BACKGROUND: A common weakness of patient satisfaction surveys is a suboptimal participation rate. Some patients may be unable to participate, because of language barriers, physical limitations, or mental problems. As the role of these barriers is poorly understood, we aimed to identify patient characteristics that are associated with non-participation in a patient satisfaction survey. METHODOLOGY: At the University Hospitals of Geneva, Switzerland, a patient satisfaction survey is regularly conducted among all adult patients hospitalized for >24 hours on a one-month period in the departments of internal medicine, geriatrics, surgery, neurosciences, psychiatry, and gynaecology-obstetrics. In order to assess the factors associated with non-participation to the patient satisfaction survey, a case-control study was conducted among patients selected for the 2005 survey. Cases (non respondents, n = 195) and controls (respondents, n = 205) were randomly selected from the satisfaction survey, and information about potential barriers to participation was abstracted in a blinded fashion from the patients' medical and nursing charts. PRINCIPAL FINDINGS: Non-participation in the satisfaction survey was independently associated with the presence of a language barrier (odds ratio [OR] 4.53, 95% confidence interval [CI95%]: 2.14-9.59), substance abuse (OR 3.75, CI95%: 1.97-7.14), cognitive limitations (OR 3.72, CI95%: 1.64-8.42), a psychiatric diagnosis (OR 1.99, CI95%: 1.23-3.23) and a sight deficiency (OR 2.07, CI95%: 0.98-4.36). The odds ratio for non-participation increased gradually with the number of predictors. CONCLUSIONS: Five barriers to non-participation in a mail survey were identified. Gathering patient feedback through mailed surveys may lead to an under-representation of some patient subgroups. | Humans | Patient Participation | Patient Satisfaction | Mental Disorders | OK |
| Vivian W. | Sung |  |  |  |  | American Journal of Obstetrics and Gynecology | 2011-08-00 Aug 2011 | OBJECTIVE: The purpose of this study was to estimate the effect of insurance status on pelvic floor physical therapy (PFPT) nonparticipation for the treatment of urinary incontinence. STUDY DESIGN: A cross-sectional study of women referred to PFPT for urinary incontinence between January 2009 and June 2010 was conducted. A telephone questionnaire was administered. Multiple logistic regression was used to identify risk factors for nonparticipation. RESULTS: Thirty-three percent of women with private insurance and 17% with other insurance were PFPT nonparticipants. On multiple logistic regression, women with Medicare were more likely to participate in PFPT (odds ratio [OR], 0.12; 95% confidence interval [CI], 0.01-0.72). Risk factors for nonparticipation included insurance noncoverage (OR, 103.85; 95% CI, 6.21-infinity) and a negative perception regarding the benefit of PFPT (OR, 5.07; 95% CI, 2.16-12.49). CONCLUSION: Among women who were referred to PFPT for urinary incontinence, insurance noncoverage and negative patient perception of efficacy were risk factors for nonparticipation, although having Medicare was protective. Improving patient education and insurance coverage for PFPT may increase usage. | Humans | Patient Participation | Questionnaires | United States | OK |
|  |  |  |  |  |  | Nurse Researcher | 2014-01-00 Jan 2014 | AIM: To determine what encourages or discourages AIDS/HIV patients from completing questionnaires. BACKGROUND: Data from surveys can provide important information but response rates can be poor. Factors that affect this in an HIV clinic population have not been assessed previously. REVIEW METHODS: The researchers used a qualitative approach and a structured interview schedule. Ten patients were recruited and participated in one-to-one interviews. DISCUSSION: Most patients saw the potential value of completing a questionnaire in the clinic. Lack of privacy during completion was raised as a concern, particularly if there were sensitive questions to respond to. Lack of technical competence was identified by some as a barrier to participation in electronic surveys. Tickboxes were preferred over rating scales or open answers. Participants did not expect a cash incentive or entry in a prize draw when asked to complete a questionnaire in-clinic. CONCLUSION: Generally, AIDS/HIV patients were willing to participate in surveys. Availability of a private area to complete the questionnaire, avoiding complex electronic systems, completion in the clinic, tickbox answers and a length of under two sides of A4 were the most important features that improved acceptability of the questionnaire to patients. Implications for research Surveys that evaluate care need to be short, easy to complete and have clear relevance to the patients' experience. | Humans | Patient Participation | Questionnaires | Data Collection | OK |
|  |  |  |  |  |  | Circulation. Cardiovascular Quality and Outcomes | 2014-01-00 Jan 2014 | | Time Factors | Humans | Patient Participation | Physician-Patient Relations | OK |
| Elisa | Weiss | Soye | Zaid-Muhammad | David | Lounsbury | AIDS patient care and STDs | 2010-06-00 Jun 2010 | Despite substantial data documenting the challenges in recruiting racial and ethnic minorities into research studies, relatively little is known about the attitudes and beliefs toward research that are held by racial and ethnic minorities living with HIV/AIDS. The present study assessed the research attitudes and beliefs of a racially and ethnically diverse group of persons living with HIV/AIDS, with research broadly defined as either psychosocial, behavioral, or clinical. Also assessed were factors that would encourage or discourage them from participating in a research study. Six hundred twenty-two participants were recruited from 22 points of service in New York City; data were gathered through a single in-person structured interview conducted in Spanish or English. Findings from a series of quantitative analyses indicated that attitudes about research were primarily neutral or positive, and different attitude and belief patterns were associated with different preferences regarding what would or would not incline one to participate in a research study. Results suggest that minorities with HIV/AIDS are open to the possibility participating in research; however, they also suggest that receptivity to research may not be uniform and indicated a variety of specific research design and implementation options that investigators should consider in order to ensure sufficient access and interest in participation. | Male | Humans | Patient Participation | Questionnaires | OK |
| Tommy | Haynes | Louise | Jones |  |  | British Journal of Social Work | 2011-01-00 janvier 2011 | The management and delivery of bereavement support services in palliative care settings present practical and ethical challenges. A national survey, conducted in 2007, examined bereavement practice in ten Marie Curie hospices across the UK. This qualitative study was undertaken using semi-structured telephone interviews with Bereavement Service Leaders located in each hospice. Although findings revealed that bereavement services were in operation and had been reviewed in response to the National Institute for Clinical Excellence Guidance (2004) and all bereaved families were offered support, there was no standardisation of service delivery across sites. Multidisciplinary team meetings facilitated shared decision making for bereavement follow-up and expanded and clarified documentation completed by nursing staff around the time of the patient’s death. However, there was ambiguity regarding professional ‘duty of care’ and agency responses to bereaved individuals who were suicidal. Questions were raised around clinical effectiveness, reliability and professional accountability. The study highlighted ethical issues centred on documentation, user participation and consent, and found staff training was variable across the ten hospices. The findings have informed the development of a post-bereavement service model that has been subsequently implemented across Marie Curie Cancer Care. (PsycINFO Database Record (c) 2013 APA, all rights reserved). (journal abstract) | Decision Making | Health Care Delivery | Social Workers | palliative care | OK |
| Francis D. | Pagani | Jeffrey | Teuteberg | Salpy V. | Pamboukian | The Journal of Heart and Lung Transplantation: The Official Publication of the International Society for Heart Transplantation | 2012-11-00 Nov 2012 | | Decision Making | Humans | Patient Participation | Questionnaires | OK |
|  |  |  |  |  |  | Value in Health: The Journal of the International Society for Pharmacoeconomics and Outcomes Research | 2012-05-00 May 2012 | | Humans | Patient Participation | United States | self report | OK |
|  |  |  |  |  |  | Journal of Religion and Health | 2012-06-00 Jun 2012 | This paper summarizes survey and interview results from a cross-sectional study of New Zealand health care chaplaincy personnel concerning their involvement in multiple bioethical issues encountered by patients, families and clinical staff within the health care context. Some implications of this study concerning health care chaplaincy, ecclesiastical institutions, health care institutions and government responsibilities are discussed and recommendations presented. | Male | Humans | Patient Participation | Questionnaires | OK |
| Maxine | Weinstein | Carol D. | Ryff |  |  | Journal of Aging and Health | 2010-12-00 Dec 2010 | OBJECTIVES: Midlife in the United States (MIDUS) is a national study of health and aging among individuals aged 25 to 74 at baseline (1995-1996). Longitudinal survey assessments (2004-2005), were followed by biological assessments on a subsample (aged 35-85).To facilitate public use, we describe the protocol, measures, and sample. METHOD: Respondents traveled to clinics for a 2-day data-collection protocol that included fasting blood specimens, 12-hr urine specimen, medical history, physical exam, bone densitometry, and a laboratory challenge (heart rate variability, blood pressure, respiration, salivary cortisol). RESULTS: Response rates for the biological protocol (N = 1,255) were 39.3% or 43.1% (adjusting for those who could not be located or contacted). Reasons for nonparticipation were travel, family obligations, and being too busy. Respondents were comparable to the recruitment pool on most demographic characteristics and health assessments. DISCUSSION: Strengths of the protocol vis-à-vis similar studies include opportunities to link biological factors with diverse content from other MIDUS projects. | Male | Humans | Patient Participation | Questionnaires | OK |
| Graziella | Jarabo | Michel | Boulvain | Olivier | Irion | Midwifery | 2011-12-00 Dec 2011 | OBJECTIVE: To explore women's perceptions of their experience of the diagnosis of breech presentation and decision-making processes regarding the choice of mode of childbirth. DESIGN: A qualitative study was conducted using semi-structured interviews. Data were analysed thematically. SETTING: Department of Gynaecology and Obstetrics, University Hospitals of Geneva, Switzerland. PARTICIPANTS: seven primiparous and five multiparous women experiencing a singleton breech presentation for childbirth were interviewed. FINDINGS: Two concomitant and interdependent processes were identified. First, an emotional response ranging from the hope that the fetus would return to a normal vertex position to the acceptance of breech presentation and its consequences. Second, a decision-making process related to childbirth mode for breech presentation with the complex management of intra- and extra-personal factor influences. Women perceive information about the risks of vaginal childbirth of paramount importance compared with those associated with caesarean childbirth. When women choose vaginal childbirth, influences related to their personality and life history appear to predominate. Women often have the feeling of being alone to assume the choice of childbirth mode and possible complications. KEY CONCLUSIONS: The diagnosis of breech presentation should not be treated as a commonplace event. The role of caregivers needs to go beyond information on the risks and benefits of both modes of childbirth. Emphasis should be placed on listening to the expectations of pregnant women for childbirth, creating spaces for dialogue, and allowing additional time for reflection. Useful information material should be provided to give the women a feeling of shared decision-making. | Decision Making | Humans | Patient Satisfaction | Questionnaires | OK |
| Daniel | Rock |  |  |  |  | The Journal of Neuropsychiatry and Clinical Neurosciences | 2012-00-00 2012 | Few tools assessing neurocognitive dysfunction in schizophrenia are able to measure integrated executive functions in the context of the problem-solving demands of a patient's everyday world. The authors evaluated the BRIEF-A (Behavior Rating Inventory of Executive Function-Adult version) Informant Report in 112 inpatients with chronic schizophrenia in a rehabilitation hospital. Factor analysis yielded a three-factor solution (Emotional Regulation, Problem-Solving, Orderliness). The BRIEF-A is psychometrically robust in this population and, in the absence of patient participation, provides an estimate of executive functions in a population that may otherwise be beyond the reach of formal neurocognitive testing. | Male | Humans | Female | Aged | OK |
| Nanne K. | de Vries |  |  |  |  | Journal of Medical Internet Research | 2011-00-00 2011 | BACKGROUND: Internet-delivered interventions can effectively change health risk behaviors, but the actual use of these interventions by the target group once they access the website is often very low (high attrition, low adherence). Therefore, it is relevant and necessary to focus on factors related to use of an intervention once people arrive at the intervention website. We focused on user perceptions resulting in e-loyalty (ie, intention to visit an intervention again and to recommend it to others). A background theory for e-loyalty, however, is still lacking for Internet-delivered interventions. OBJECTIVE: The objective of our study was to propose and validate a conceptual model regarding user perceptions and e-loyalty within the field of eHealth. METHODS: We presented at random 3 primary prevention interventions aimed at the general public and, subsequently, participants completed validated measures regarding user perceptions and e-loyalty. Time on each intervention website was assessed by means of server registrations. RESULTS: Of the 592 people who were invited to participate, 397 initiated the study (response rate: 67%) and 351 (48% female, mean age 43 years, varying in educational level) finished the study (retention rate: 88%). Internal consistency of all measures was high (Cronbach alpha > .87). The findings demonstrate that the user perceptions regarding effectiveness (beta(range) .21-.41) and enjoyment (beta(range) .14-.24) both had a positive effect on e-loyalty, which was mediated by active trust (beta(range) .27-.60). User perceptions and e-loyalty had low correlations with time on the website (r(range) .04-.18). CONCLUSIONS: The consistent pattern of findings speaks in favor of their robustness and contributes to theory validation regarding e-loyalty. The importance of a theory-driven solution to a practice-based problem (ie, low actual use) needs to be stressed in view of the importance of the Internet in terms of intervention development. Longitudinal studies are needed to investigate whether people will actually revisit intervention websites and whether this leads to changes in health risk behaviors. | Male | Humans | Patient Participation | Questionnaires | OK |
| Jean J. | Schensul | Anne M. | Bowen | Marc I. | Rosen | The Psychiatric Quarterly | 2013-03-00 Mar 2013 | Integrating information technology into healthcare has the potential to bring treatment to hard-to-reach people. Individuals with serious mental illness (SMI), however, may derive limited benefit from these advances in care because of lack of computer ownership and experience. To date, conclusions about the computer skills and attitudes of adults with SMI have been based primarily on self-report. In the current study, 28 psychiatric outpatients with co-occurring cocaine use were interviewed about their computer use and opinions, and 25 were then directly observed using task analysis and think aloud methods as they navigated a multi-component health informational website. Participants reported low rates of computer ownership and use, and negative attitudes towards computers. Self-reported computer skills were higher than demonstrated in the task analysis. However, some participants spontaneously expressed more positive attitudes and greater computer self-efficacy after navigating the website. Implications for increasing access to computer-based health information are discussed. | Male | Humans | Patient Participation | Young Adult | OK |
| Judy K. | Wendt | Leo G. | Visser | Daan | Breederveld | Journal of Travel Medicine | 2013-02-00 2013 Jan-Feb | BACKGROUND: Many studies have explored the risk perception of frequent business travelers (FBT) toward malaria. However, less is known about their knowledge of other infectious diseases. This study aimed to identify knowledge gaps by determining the risk perception of FBT toward 11 infectious diseases. METHODS: Our retrospective web-based survey assessed the accuracy of risk perception among a defined cohort of FBT for 11 infectious diseases. We used logistic regression and the chi-square test to determine the association of risk perception with source of travel advice, demographic variables, and features of trip preparation. RESULTS: Surveys were returned by 63% of the 608 self-registered FBT in Rijswijk, and only the 328 completed questionnaires that adhered to our inclusion criteria were used for analysis. The majority (71%) sought pre-travel health advice and used a company health source (83%). Participants seeking company travel health advice instead of external had significantly more accurate risk knowledge (p = 0.03), but more frequently overestimated typhoid risk (odds ratio = 2.03; 95% confidence interval = 1.23-3.34). While underestimation of disease risk was on average 23% more common than overestimation, HIV risk was overestimated by 75% of FBT. CONCLUSIONS: More accurate knowledge among FBT seeking company health advice demonstrates that access to in-company travel clinics can improve risk perception. However, there is an obvious need for risk knowledge improvement, given the overall underestimation of risk. The substantial overestimation of HIV risk is probably due to both public and in-company awareness efforts. Conversely, typhoid risk overestimation was statistically associated with seeking company health advice, and therefore specifically reflects the high focus on typhoid fever within Shell's travel clinic. This study serves as a reminder that a knowledge gap toward infectious diseases besides malaria still exists. Our article will explore the future requirements for more targeted education and research among FBT in companies worldwide. | Male | Humans | Patient Participation | Questionnaires | OK |
| R. | Brown |  |  |  |  | Family Practice | 2013-08-00 Aug 2013 | PURPOSE: Acute upper respiratory infections (URI) are the second most common diagnosis in primary care offices. As treatments have limited effectiveness, patient counseling regarding expectations for the course of the URI is an important aspect of care. It is unknown how accurate patients, clinicians or questionnaires such as the Wisconsin Upper Respiratory Symptom Survey (WURSS) instrument are at predicting URI severity and duration, and whether these predictions should be used to counsel patients. METHODS: Seven hundred and nineteen individuals with recent onset cold in community clinic settings participated. Participants and clinicians predicted the severity and duration of the URI and participants completed the WURSS instrument at initial visit. Subsequent URI global severity was calculated as area under the curve using an average of twice-daily WURSS-21 self-reports as the y-axis and illness duration as the x-axis. URI duration was determined by self-report of beginning and end of illness. Linear regression analysis was used to correlate baseline predictions with subsequent outcomes. Analyses by gender, age and income were also performed. RESULTS: There was no significant association between participant and clinician predictions of severity or duration. Initial WURSS values explained 0.119 (95% CI: 0.074-0.163) of the variance in subsequent severity outcomes. There were no significant differences in associations by age, gender or income. CONCLUSIONS: Clinicians should not use their predictive assessments or their patients' predictions when advising patients on the expected course of a URI. This study also suggests that the WURSS instrument could give some predictive information, but whether this is clinically useful is uncertain. | Male | Humans | Patient Participation | Questionnaires | OK |
| Thomas W. J. | Janssen | Lia C. C. | van der Maas | Heleen | Beckerman | Journal of Rehabilitation Research and Development | 2014-00-00 2014 | The construct validity and construct responsiveness of the performance scale of the Canadian Occupational Performance Measure (COPM) was measured in 87 newly admitted patients with chronic pain attending an outpatient rehabilitation clinic. At admission and after 12 wk, patients completed a COPM interview, the Pain Disability Index (PDI), and the RAND 36-Item Health Survey (RAND-36). We determined the construct validity of the COPM by correlations between the COPM performance scale (COPM-P), the PDI, and the RAND-36 at admission. Construct responsiveness was assessed by calculating the correlations between the change scores (n = 57). The COPM-P did not significantly correlate with the PDI (r = -0.260) or with any subscale of the RAND-36 (r = -0.007 to 0.248). Only a moderate correlation was found between change scores of the COPM-P and PDI (r = -0.380) and weak to moderate correlations were found between change scores of the COPM-P and the RAND-36 (r = -0.031 to 0.388), with the higher correlations for the physical functioning, social functioning, and role limitations (physical) subscales. In patients with chronic pain attending our rehabilitation program, the COPM-P measures something different than the RAND-36 or PDI. Therefore, construct validity of the COPM-P was not confirmed by our data. We were not able to find support for the COPM-P to detect changes in occupational performance. | | | | | OK |
| Jean | Bourbeau | Charles K. | Chan | Darcy D. | Marciniuk | Canadian Respiratory Journal: Journal of the Canadian Thoracic Society | 2013-04-00 2013 Mar-Apr | INTRODUCTION: Chronic obstructive pulmonary disease (COPD) is a common respiratory condition and the fourth leading cause of death in Canada. Optimal COPD management requires patients to participate in their care and physician knowledge of patients' perceptions of their disease. METHODS: A prospective study in which respiratory specialist physicians completed a practice assessment questionnaire and patient assessments for 15 to 20 consecutive patients with COPD. Patients also completed a questionnaire regarding their perceptions of COPD and its management. RESULTS: A total of 58 respiratory specialist physicians from across Canada completed practice assessments and 931 patient assessments. A total of 640 patients with COPD (96% with moderate, severe or very severe disease) completed questionnaires. Symptom burden was high and most patients had experienced a recent exacerbation. Potential COPD care gaps were identified with respect to appropriate medication prescription, lack of an action plan, and access to COPD educators and pulmonary rehabilitation. Perceived knowledge needs and gaps differed between physicians and patients. CONCLUSIONS: Despite the dissemination of Canadian and international COPD clinical practice guidelines for more than a decade, potential care gaps remain among patients seen by respiratory specialist physicians. Differing perceptions regarding many aspects of COPD among physicians and patients may contribute to these care gaps. | Male | Humans | Patient Participation | Questionnaires | OK |
| Tracy A. | Schroepfer | Douglas J. | Reding | Bruce | Campbell | Journal of Health Communication | 2010-06-00 Jun 2010 | Previous research has indicated that treatment staff often underestimate the informational needs of cancer patients. In this study, the authors determined the total number of information sources obtained and used to influence treatment decisions, and the clinical and demographic factors associated with the use of specific sources of information in cancer patients. Participants were identified by the statewide cancer registry and diagnosed in 2004 with breast, colorectal, lung, or prostate cancer. A self-administered mailed questionnaire elicited cancer treatments, demographics, and information sources used to make treatment decisions. Of those surveyed, 1,784 (66%) participated and responded to all questions regarding information use. Over 69% of study participants reported obtaining information from a source other than the treatment staff. Significant predictors of using additional information sources included younger age, higher income, higher education, complementary and alternative medicine (CAM) use, and reporting shared decision making (all p values <.01). Participants with a college degree were more likely to use the Internet (OR 3.7; 95% CI 1.5-9.0) and scientific research reports (OR 3.3; 95% CI 1.6-6.9) to influence treatment decisions compared with those without a high school degree. Support group use to influence treatment decisions was not associated with socioeconomic variables but did vary by cancer type and CAM use. The sources of information study participants obtained and used to influence treatment decisions varied strongly by socioeconomic and demographic variables. These findings provide a deeper understanding of the information needs of cancer patients and have implications for dissemination strategies that can minimize disparities in access to cancer information. | Male | Humans | Patient Participation | Questionnaires | OK |
| Sebastian | Belle | Axel | Gerhardt | Kirsten | Merx | Onkologie | 2013-00-00 2013 | BACKGROUND: In 2009, Germany enacted a new law supporting advance directives that led to heated discussions in the media and the public. 3 years after the law passed, we surveyed patients with malignant diseases with regards to their views on advance directives. PATIENTS AND METHODS: Between September 2011 and July 2012 an anonymous survey on advance directives was conducted among 617 patients at the hematology and oncology outpatient department of the University Hospital Mannheim, using a standardized questionnaire developed for this investigation. RESULTS: Of the 503 patients who returned the questionnaire, 31% (n = 157) indicated having an advance directive. Of these 157, 54% (n = 85) completed the advance directive after 2009. 56% (282 out of 503) desired more information on advance directives. Of these, 71% (201 out of 282) wanted their general physician and 45% (128 out of 282) their specialist, to provide more information about this issue. Of the 339 patients without an advance directive, 47% (n = 158) stated that they had 'not worried about that yet'. CONCLUSION: Although the percentage of patients with advance directives has increased since the legislative amendment, more information is still required by patients. It is recommended that physicians should discuss advance directives more frequently with their patients. | Male | Humans | Patient Participation | Questionnaires | OK |
| Kristen J. | Wells | Luis E. | Gonzalez | Cathy D. | Meade | Journal of Cancer Education: The Official Journal of the American Association for Cancer Education | 2012-06-00 Jun 2012 | Patient-related barriers have hindered cancer patients' abilities to participate in the decision-making processes to participate in clinical trials. However, little is known about patients' emotional barrier of fear and how physicians influence this barrier. We conducted 48 in-depth interviews with cancer patients to determine their knowledge and attitudes about participating in clinical trials, transcribed interviews verbatim, and qualitatively analyzed the transcripts using content analysis. For the purpose of this manuscript, we focused on findings related to the role of the emotional barrier of fear in cancer patients' perceptions of participating in clinical trials. The majority of cancer patients (n = 40, 83.3%) discussed fears surrounding clinical trials, particularly as it related to cancer diagnosis, clinical trial participation, and fear of the unknown. In conclusion, providers might consider addressing the role of fear in patients' considering participating in a clinical trial. | Male | Decision Making | Humans | Patient Participation | OK |
| Pia | Heußner | Markus W. | Haun | Anne M. | Stiggelbout | Oncology Research and Treatment | 2014-00-00 2014 | BACKGROUND: Decision-making with patients with incurable cancer often requires trade-offs between quality and length of life. The 'Quality and Quantity Questionnaire' (QQ) is an English-language measure of patients' preference for length or quality of life. The aim of this study was to translate and validate this questionnaire. MATERIALS AND METHODS: 1 new item was formulated to improve the 'Quality of life' scale. Construct validity including exploratory factor analysis, convergent and discriminant validity, and reliability was determined in n = 194 patients. RESULTS: The acceptability of the questionnaire among patients was high. The item-non-response rate was very low (2.5-4%). The 2 QQ scales 'Quality of life' (QL) and 'Length of life' (LL) had good and acceptable internal consistency (Cronbach's = 0.71 for LL and 0.59 for QL). Convergent validity was shown by significant correlation of the QL subscale with the CCAT (Cancer Communication Assessment Tool) subscale 'Limitation of treatment' (r = 0.37, p < 0.01) and the LL scale with the CCAT subscale 'Continuing treatment' (r = 0.24, p = 0.00). CONCLUSION: The German version of 'QQ' has satisfactory psychometric properties for measuring patients' preferences for LL or QL. It can be used in all research fields that should be informed by patients' preferences: shared decision-making, palliative care, and health services. | | | | | OK |
| Anita | Jhunjhunwala | Glyn C. | Jarvis | Chris | McVittie | Supportive Care in Cancer: Official Journal of the Multinational Association of Supportive Care in Cancer | 2011-07-00 Jul 2011 | PURPOSE: To explore the perspectives of cancer care centre users on participation in psychosocial research to inform research design and ethics. METHODS: The study is based on a qualitative research design. Fourteen semistructured interviews were carried in people diagnosed with cancer and carers. The interview included four main questions about practical barriers to participation, types of research design, motivating factors and the conduct of research in a cancer care support setting. The data were analysed using qualitative content analysis. RESULTS: Interviewees demonstrated a willingness to participate in psychosocial research within certain circumstances. There were no practical barriers identified, although they considered payment for research-related travel important. The most acceptable research design was the face-to-face interview and the least preferred was the randomised control trial. The factors that motivated participation were altruism, valuing research, and making a contribution to the centre. Interviewees supported the conduct of research in cancer care support centres conditional upon delaying recruitment during the initial months of users' visits and its need to be discreet to avoid deterring visitors from accessing the centre. CONCLUSIONS: The study concludes that the personal interaction between participants and researchers is the most important feature of decision-making by patients/carers to join studies. Taking into account the perspectives of people affected by cancer during the early stages of research design may enhance recruitment and retention and can contribute to the development of research protocols and ethics. | Decision Making | Humans | Patient Participation | Questionnaires | OK |
| Lisa W. | Le | Jane | Higgins | Andrea | Bezjak | Clinical Lung Cancer | 2013-03-00 Mar 2013 | BACKGROUND: Comprehensive and accurate assessment of symptoms experienced by patients undergoing lung radiotherapy (RT) is challenging. This study aims to evaluate the feasibility and utility of collecting acute toxicity information by using a patient-reported instrument, the Thoracic Symptom Self-Assessment Tool (TSSAT). METHODS: The TSSAT is based on the CTCAE v3.0(Common Toxicity Criteria of Adverse Events). All patients undergoing lung RT at our center from May 2008 to April 2009 were asked to complete the TSSAT on day 1 and weekly during RT. TSSAT scores were compared with clinician reporting of the same symptoms. Descriptive statistics and weighted kappa values were calculated to measure the agreement between patient- and clinician-reported acute toxicity. RESULTS: Of 300 consecutive patients approached, 49% (148/300) completed the TSSAT at least once. Patient participation and compliance were associated with treatment intent; radical (87%) vs. palliative (25%); P = <.0001. The average data completion rate by patients was 72%, and the average toxicity documentation rate by clinicians was 67%. Agreement between patients and clinicians was fair to moderate for most symptoms; the majority (>79%) of the differences were within one grade. Patients reported greater severity than clinicians for subjective symptoms. Clinicians graded greater severity than patients for the more observable symptoms. CONCLUSIONS: The TSSAT has been shown to be feasible and accepted by patients receiving radical dose RT. Patient-reported assessments may improve acute symptom management in the future. | Male | Humans | Female | Aged | OK |
| Diane C. | Bodurka | Angele K. | Saleeba | Charlotte C. | Sun | Journal of Pain and Symptom Management | 2013-12-00 Dec 2013 | CONTEXT: Experts in patient-reported outcome (PRO) measurement emphasize the importance of including patient input in the development of PRO measures. Although best methods for acquiring this input are not yet identified, patient input early in instrument development ensures that instrument content captures information most important and relevant to patients in understandable terms. OBJECTIVES: The M. D. Anderson Symptom Inventory (MDASI) is a reliable valid PRO instrument for assessing cancer symptom burden. We report a qualitative (open-ended, in-depth) interviewing method that can be used to incorporate patient input into PRO symptom measure development, with our experience in constructing an MDASI module for ovarian cancer (MDASI-OC) as a model. METHODS: Fourteen patients with ovarian cancer (OC) described symptoms experienced at the time of the study, at diagnosis, and during prior treatments. Researchers and clinicians used content analysis of interview transcripts to identify symptoms in patient language. Symptoms were ranked on the basis of the number of patients mentioning them and by clinician assessment of relevance. RESULTS: Forty-two symptoms were mentioned. Eight OC-specific items will be added to the 13 core symptom items and six interference items of the MDASI in a test version of the MDASI-OC on the basis of the number of patients mentioning them and clinician assessment of importance. The test version is undergoing psychometric evaluation. CONCLUSION: The qualitative interviewing process, used to develop the test MDASI-OC, systematically captures common symptoms important to patients with OC. This methodology incorporates the patient experience recommended by experts in PRO instrument development. | Humans | Patient Participation | Questionnaires | Female | OK |
| Margaret | Brum | Veola | Caruso | Caroline | Chessex | Journal of Cardiopulmonary Rehabilitation and Prevention | 2011-12-00 2011 Nov-Dec | PURPOSE: Cardiac rehabilitation (CR) is a proven effective means for secondary prevention of coronary heart disease. Timely access to CR services is key to promoting patient participation and ensuring optimal patient outcomes. Despite wait time benchmarks having been established, research regarding how long patients wait to enter CR following referral receipt is limited. The aim of this study was to (a) describe wait times from CR referral to intake assessment and (b) examine the association of wait time to CR enrollment rates. METHODS: Wait time from date of CR referral to date of intake assessment was calculated in days for 599 participants referred to CR from 2006 to 2009 inclusive. A descriptive examination of sociodemographic and clinical characteristics was performed, followed by logistic regression analysis to assess the wait time by enrollment relationship. RESULTS: Median wait time from referral receipt to CR intake was 42.0 days. Wait time had a negative effect on CR enrollment, such that for every 1-day increment in wait time, patients were 1% less likely to enroll. CONCLUSIONS: The time that patients wait to enroll in CR may affect the number of patients who choose to attend, and longer wait times may mean fewer patients will benefit from CR participation. Programs should be encouraged to undertake quality improvement initiatives to ensure wait times are not negatively impacting patient enrollment and ultimately preventing patients from benefiting from CR participation. Further research is needed to establish evidence-based wait time benchmarks and interventions to promote timely access to CR services. | Time Factors | Male | Humans | Questionnaires | OK |
|  |  |  |  |  |  | PLoS ONE | 2013-06-06 juin 6, 2013 | Purpose: End-of-life (EOL) measures are limited in capturing caregiver assessment of the quality of EOL care. Because none include caregiver perception of patient suffering or prolongation of death, we sought to develop and validate the Caregiver Evaluation of Quality of End-of-Life Care (CEQUEL) scale to include these dimensions of caregiver-perceived quality of EOL care. Patients and Methods: Data were derived from Coping with Cancer (CwC), a multisite, prospective, longitudinal study of advanced cancer patients and their caregivers (N = 275 dyads). Caregivers were assessed before and after patient deaths. CEQUEL’s factor structure was examined; reliability was evaluated using Cronbach’s α, and convergent validity by the strength of associations between CEQUEL scores and key EOL outcomes. Results: Factor analysis revealed four distinct factors: Prolongation of Death, Perceived Suffering, Shared Decision-Making, and Preparation for the Death. Each item loaded strongly on only a single factor. The 13-item CEQUEL and its subscales showed moderate to acceptable Cronbach’s α (range: 0.52–0.78). 53% of caregivers reported patients suffering more than expected. Higher CEQUEL scores were positively associated with therapeutic alliance (ρ = .13; p ≤ .05) and hospice enrollment (z = −2.09; p ≤ .05), and negatively associated with bereaved caregiver regret (ρ = −.36, p ≤ .001) and a diagnosis of Posttraumatic Stress Disorder (ρ = −2.06; p ≤ .05). Conclusion: CEQUEL is a brief, valid measure of quality of EOL care from the caregiver’s perspective. It is the first scale to include perceived suffering and prolongation of death. If validated in future work, it may prove a useful quality indicator for the delivery of EOL care and a risk indicator for poor bereavement adjustment. (PsycINFO Database Record (c) 2014 APA, all rights reserved). (journal abstract) | Psychometrics | test validity | test reliability | cancer | OK |
|  |  |  |  |  |  | PloS One | 2013-00-00 2013 | PURPOSE: End-of-life (EOL) measures are limited in capturing caregiver assessment of the quality of EOL care. Because none include caregiver perception of patient suffering or prolongation of death, we sought to develop and validate the Caregiver Evaluation of Quality of End-of-Life Care (CEQUEL) scale to include these dimensions of caregiver-perceived quality of EOL care. PATIENTS AND METHODS: Data were derived from Coping with Cancer (CwC), a multisite, prospective, longitudinal study of advanced cancer patients and their caregivers (N = 275 dyads). Caregivers were assessed before and after patient deaths. CEQUEL's factor structure was examined; reliability was evaluated using Cronbach's α, and convergent validity by the strength of associations between CEQUEL scores and key EOL outcomes. RESULTS: FACTOR ANALYSIS REVEALED FOUR DISTINCT FACTORS: Prolongation of Death, Perceived Suffering, Shared Decision-Making, and Preparation for the Death. Each item loaded strongly on only a single factor. The 13-item CEQUEL and its subscales showed moderate to acceptable Cronbach's α (range: 0.52-0.78). 53% of caregivers reported patients suffering more than expected. Higher CEQUEL scores were positively associated with therapeutic alliance (ρ = .13; p≤.05) and hospice enrollment (z = -2.09; p≤.05), and negatively associated with bereaved caregiver regret (ρ = -.36, p≤.001) and a diagnosis of Posttraumatic Stress Disorder (z = -2.06; p≤.05). CONCLUSION: CEQUEL is a brief, valid measure of quality of EOL care from the caregiver's perspective. It is the first scale to include perceived suffering and prolongation of death. If validated in future work, it may prove a useful quality indicator for the delivery of EOL care and a risk indicator for poor bereavement adjustment. | Male | Humans | Questionnaires | Female | Doublon |
| Kevin | Ju | Thierry G. | Guitton | Chaitanya S. | Mudgal | The Journal of Hand Surgery | 2014-09-00 Sep 2014 | PURPOSE: This study tested the null hypothesis that there are no differences between the preferences of hand surgeons and those patients with carpal tunnel syndrome (CTS) facing decisions about management of CTS (ie, the preferred content of a decision aid). METHODS: One hundred three hand surgeons of the Science of Variation Group and 79 patients with CTS completed a survey about their priorities and preferences in decision making regarding the management of CTS. The questionnaire was structured according the Ottawa Decision Support Framework for the development of a decision aid. RESULTS: Important areas on which patient and hand surgeon interests differed included a preference for nonpainful, nonoperative treatment and confirmation of the diagnosis with electrodiagnostic testing. For patients, the main disadvantage of nonoperative treatment was that it was likely to be only palliative and temporary. Patients preferred, on average, to take the lead in decision making, whereas physicians preferred shared decision making. Patients and physicians agreed on the value of support from family and other physicians in the decision-making process. CONCLUSIONS: There were some differences between patient and surgeon priorities and preferences regarding decision making for CTS, particularly the risks and benefits of diagnostic and therapeutic procedures. CLINICAL RELEVANCE: Information that helps inform patients of their options based on current best evidence might help patients understand their own preferences and values, reduce decisional conflict, limit surgeon-to-surgeon variations, and improve health. | | | | | OK |
| Snjezana | Stemberger-Papić | Suzana | Janković | Helena | Glibotić-Kresina | Collegium Antropologicum | 2010-03-00 Mar 2010 | The opportunistic cervical cancer screening has been conducted in Croatia since its introduction in the 1960s, in the context of a high quality gynaecological cytology with a long tradition and a wide network of primary care gynaecologists. In 2006, a pilot screening programme under the title "Early detection of cervical cancer was conducted in Primorsko-Goranska County (PGC)", as the first organised cervical cancer screening ever conducted in the Republic of Croatia. The pilot screening programme targeted women aged 20-64 years. The pilot group consisted of 6,000 randomly sampled primary care patients of six gynaecologists. The women were invited via a personal letter and were given a questionnaire. The results of the first and the second year of screening, as well as of both years together were analysed. The response rate to the anamnestic questionnaire was 49.1%. The participation rates to the screening were 35.2% in 2007, and 46.5% in 2008, total of 42.7%. The increase in participation between years 2007 and 2008 was statistically significant (p = 0.01). According to the age, the lowest participation rate of 33.3% was observed in the youngest group of women (20-29) and the highest of 60.7% in the oldest group (60-64). The detection rate of cytological abnormalities was 4.6% with 2.6% of borderline (ASCUS) cytology and referral rate of 1.2%. The highest abnormal Pap test frequencies of 6.8% and 7.1% were observed in the youngest age groups (20-29 and 30-39), and the lowest (2%) in the age group of 60-64. Specimen adequacy was generally of high quality with unsatisfactory rate of 0.8%, with statistically significant improvement in 2008, compared to the previous year (p = 0.001). Although to a limited extension, during two-year pilot cervical cancer screening programme in PGC the participation rates and Pap smear adequacy have improved. We expect that the continuation of the programme will result in further increase of participation and higher overall quality of the programme. | Humans | Patient Participation | Questionnaires | Young Adult | OK |
| Line | Haboubi | Julien | Le Breton |  |  | Santé Publique (Vandoeuvre-Lès-Nancy, France) | 2013-06-00 2013 May-Jun | OBJECTIVES: The purpose of this study was to assess women's knowledge of cervical cancer screening, to examine the relationship between knowledge and screening participation, and to gain a better insight into GP involvement in screening. METHOD: A telephone survey was conducted on a random sample of women aged 25-65 years living in Val-d'Oise, France. RESULTS: 117 women responded to the survey. The results show that women were often not aware of screening modalities, although participation was high in 87.9 % of cases. Knowing that cervical smears (CS) can be performed in a medical laboratory and that HPV-vaccinated women should undergo screening were significantly associated with high participation. Women who had consulted a gynaecologist in the last 12 months were also significantly more likely to participate in screening. According to 64.1% of the respondents, the question of screening had never been discussed in GP consultations. Only 6.8% of the women had had a cervical smear performed by a GP, although most of the women (59.8%) felt that GPs should be more involved in cervical smear testing. CONCLUSION: Increased knowledge of cervical cancer screening among women may help to increase participation in screening. The study suggests that GPs should be more involved in cervical cancer screening and that their role in providing prevention information to female patients should be promoted. | Humans | Patient Participation | Questionnaires | Female | Doublon |
| Line | Haboubi | Julien | Le Breton |  |  | Santé Publique (Vandoeuvre-Lès-Nancy, France) | 2013-06-00 2013 May-Jun | OBJECTIVES: The purpose of this study was to assess women's knowledge of cervical cancer screening, to examine the relationship between knowledge and screening participation, and to gain a better insight into GP involvement in screening. METHOD: A telephone survey was conducted on a random sample of women aged 25-65 years living in Val-d'Oise, France. RESULTS: 117 women responded to the survey. The results show that women were often not aware of screening modalities, although participation was high in 87.9 % of cases. Knowing that cervical smears (CS) can be performed in a medical laboratory and that HPV-vaccinated women should undergo screening were significantly associated with high participation. Women who had consulted a gynaecologist in the last 12 months were also significantly more likely to participate in screening. According to 64.1% of the respondents, the question of screening had never been discussed in GP consultations. Only 6.8% of the women had had a cervical smear performed by a GP, although most of the women (59.8%) felt that GPs should be more involved in cervical smear testing. CONCLUSION: Increased knowledge of cervical cancer screening among women may help to increase participation in screening. The study suggests that GPs should be more involved in cervical cancer screening and that their role in providing prevention information to female patients should be promoted. | Humans | Patient Participation | Questionnaires | Female | OK |
|  |  |  |  |  |  | Birth (Berkeley, Calif.) | 2010-06-00 Jun 2010 | | Humans | Patient Participation | Female | Data Collection | OK |
| Morteza | Meftah | Maureen K. | Dwyer |  |  | Clinical Orthopaedics and Related Research | 2013-11-00 Nov 2013 | BACKGROUND: Some orthopaedic procedures, including TKA, enjoy high survivorship but leave many patients dissatisfied because of residual pain and functional limitations. An important cause of patient dissatisfaction is unfulfilled preoperative expectations. This arises, in part, from differences between provider and patient in their definition of a successful outcome. WHERE ARE WE NOW?: Patients generally are less satisfied with their outcomes than surgeons. While patients are initially concerned with symptom relief, their long-term expectations include return of symptom-free function, especially in terms of activities that are personally important. While surgeons share their patients' desire to achieve their goals, they are aware this will not always occur. Conversely, patients do not always realize some of their expectations cannot be met by current orthopaedic procedures, and this gap in understanding is an important source of discrepancies in expectations and patient dissatisfaction. WHERE DO WE NEED TO GO?: An essential prerequisite for mutual understanding is information that is accurate, objective, and relevant to the patient's condition and lifestyle. This critical information must also be understandable within the educational and cultural background of each patient to enable informed participation in a shared decision making process. Once this is achieved, it will become easier to formulate similar expectations regarding the likely level of function and symptom relief and the risk of adverse events, including persistent pain, complications, and revision surgery. HOW DO WE GET THERE?: Predictive models of patient outcomes, based on objective data, are needed to inform decision making on the individual level. This can be achieved once comprehensive data become available capturing the lifestyles of patients of diverse ages and backgrounds, including data documenting the frequency and intensity of participation in sporting and recreational activities. There is also a need for greater attention to the process of informing patients of the outcome of orthopaedic procedures, not simply for gaining more meaningful consent, but so that patients and providers may achieve greater alignment of expectations and increased acceptance of both the benefits and limitations of alternative treatments. | Humans | Patient Satisfaction | Questionnaires | risk assessment | OK |
| Hongkun | Wang | Monika J. | Thielen | Daniel | Sargent | Clinical Trials (London, England) | 2011-10-00 Oct 2011 | BACKGROUND: Research on barriers to accrual has typically emphasized factors influencing participation after trial activation. PURPOSE: We sought to identify factors influencing trial design and accrual predictions prior to trial activation associated with sufficient accrual. METHODS: A 30-question web-based survey was sent to the study chair and lead statistician for all 248 phase III trials open in 1993-2002 by five Clinical Trials Cooperative Groups. Questions addressed prior trial experience, trial design elements, accrual predictions, and perceived accrual influences. Accrual sufficiency categorization was derived from Clinical Trials Cooperative Group records: sufficient accrual included trials closed with complete accrual or at interim analysis, insufficient accrual included trials closed with inadequate accrual. Responses were analyzed by respondent role (study chair/lead statistician) and accrual sufficiency. RESULTS: Three hundred and nine eligible responses were included (response rate, 63%; lead statisticians, 81%; and study chairs, 45%), representing trials with sufficient (63%) and insufficient accruals (37%). Study chair seniority or lead statistician experience was not linked to accrual sufficiency. Literature review, study chair's personal experience, and expert opinion within Clinical Trials Cooperative Group most commonly influenced control arm selection. Clinical Trials Cooperative Group experience most influenced accrual predictions. These influences were not associated with accrual sufficiency. Among respondents citing accrual difficulties (41%), factors negatively influencing accrual were not consistently identified. Respondents credited three factors with positively influencing accrual: clinical relevance of study, lack of competing trials, and protocol paralleling normal practice. LIMITATIONS: Perceptions of lead statisticians and study chairs may not accurately reflect accrual barriers encountered by participating physicians or patients. Survey responses may be subject to recall bias. CONCLUSION: Consistent factors explaining poor accrual were not identified, suggesting reasons for poor accrual are not well understood and warrant further study. Alternate strategies for accrual prediction are needed since Clinical Trials Cooperative Group experience is linked to successful and unsuccessful accrual. | Humans | Patient Participation | Questionnaires | United States | OK |
| Joan | Smith | Adina | Gociu | Justin | Young | Journal of Health Care for the Poor and Underserved | 2010-02-00 Feb 2010 | We assessed the efficacy of a pilot questionnaire designed to elicit information about external risk factors for breast cancer in sub-Saharan African women. Preliminary analysis identified areas of the questionnaire and interviewing process that required modification, as well as socioeconomic factors that contribute to reduced participation among these understudied populations. | Humans | Patient Participation | Questionnaires | Female | OK |
|  |  |  |  |  |  | Medical Decision Making: An International Journal of the Society for Medical Decision Making | 2010-06-00 2010 May-Jun | BACKGROUND: Medical choices often evoke great value uncertainty, as patients face difficult, unfamiliar tradeoffs. Those seeking to aid such choices must be able to assess patients' ability to reduce that uncertainty, to reach stable, informed choices. OBJECTIVE: The authors demonstrate a new method for evaluating how well people have articulated their preferences for difficult health decisions. The method uses 2 evaluative criteria. One is internal consistency, across formally equivalent ways of posing a choice. The 2nd is compliance with principles of prospect theory, indicating sufficient task mastery to respond in predictable ways. METHOD: Subjects considered a hypothetical choice between noncurative surgery and palliative care, posed by a brain tumor. The choice options were characterized on 6 outcomes (e.g., pain, life expectancy, treatment risk), using a drug facts box display. After making an initial choice, subjects indicated their willingness to switch, given plausible changes in the outcomes. These changes involved either gains (improvements) in the unchosen option or losses (worsening) in the chosen one. A 2 x 2 mixed design manipulated focal change (gains v. losses) within subjects and change order between subjects. RESULTS: In this demonstration, subjects' preferences were generally consistent 1) with one another: with similar percentages willing to switch for gains and losses, and 2) with prospect theory, requiring larger gains than losses, to make those switches. CONCLUSION: Informed consent requires understanding decisions well enough to articulate coherent references. The authors' method allows assessing individuals' success in doing so. | Male | Decision Making | Decision Support Techniques | Humans | OK |
| Pamela S. | Hinds |  |  |  |  | Pediatric Critical Care Medicine: A Journal of the Society of Critical Care Medicine and the World Federation of Pediatric Intensive and Critical Care Societies | 2013-03-00 Mar 2013 | OBJECTIVE: To compare characteristics of family conferences at the bedside vs. the conference room in the PICU. DESIGN: Single-site, cohort survey study. SETTING: Thirty-three bed academic PICU in an urban setting. PARTICIPANTS: Ten PICU physicians (90.9%) providing care to 29 patients whose families participated in 58 family conferences. MEASUREMENTS AND MAIN RESULTS: Family conferences, defined as a meeting involving the parent(s) of a PICU patient and the critical care attending physician to discuss a treatment decision, redirection of care from curative to palliative, or deliver bad news, occurred most commonly among families of the sickest patients. Conferences were conducted at the bedside 20 times out of 58 (33%). Although physicians stated a general preference to discuss withdrawal or withholding care in the conference room, there was no difference in location during actual conferences. Physicians preferred the bedside when they wanted the patient to participate (p = 0.01) or because it was perceived to be easier (p < 0.0005) or faster (p = 0.016) to conduct, while the conference room was preferred when additional space was needed (p < 0.0005). Family conferences at the bedside were less likely to include a social worker (p < 0.0005), consultant physicians (p = 0.043), or father of the patient (p = 0.006) as compared with conferences in a conference room. Family conferences convened to discuss a treatment were followed by a decision within 24 hours (42% of the time) and a change in code status (32% of the time). In 32 of 58 family conferences (55%), the attending physician did not have a prior relationship with the family. CONCLUSION: Family conferences in the PICU are common both at the bedside and in conference rooms in a subpopulation of the most critically ill children and frequently result in a treatment decision or change in code status. | Male | Decision Making | Humans | Patient Participation | OK |
| C. D'Arcy J. | Holman |  |  |  |  | Asian Pacific journal of cancer prevention: APJCP | 2011-00-00 2011 | OBJECTIVE: Studies on participants' willingness to be interviewed in-person and donate blood specimens for genetic cancer research are few and most have been conducted in Western countries. Little information exists about the willingness to participate in genetic cancer research in China. METHODS: In 2009-2010, 560 hospital controls, matched to incidence cases by age, gender and residency, were randomly selected from outpatients attending the Health Examination Centre at the China Medical University's teaching hospital in Northeast China. Demographic and lifestyle characteristics were measured using a validated questionnaire by face-to-face interview and 5 ml blood samples were collected from consenting participants. A 7-point 'willingness to participate' scale was developed for use by the interviewer to record the levels of ease or difficulty experienced in recruiting each participant. The willingness to participate was compared between different subgroups of participants. RESULTS: The participation rate was 96.1% among the hospital controls. Characteristics associated with willingness to participate were age (≤60 years) and tertiary education. Weaker associations with gender and malignancies in first degree relatives were not statistically significant. The factors not strongly or significantly associated with willingness to participate were income, marital status, body mass index, smoking, passive smoking, alcohol consumption, tea drinking, or physical activity. CONCLUSION: This study suggests that while there is general acceptance of participation in genetic cancer epidemiologic research in China across subgroups of outpatient hospital controls, younger age and education are associated with increased willingness to participate, while lifestyle factors generally had little impact. | Humans | Patient Participation | Questionnaires | Neoplasms | OK |
| Toril | Rannestad | Rolf | Walstad |  |  | Journal of Rehabilitation Medicine | 2010-04-00 Apr 2010 | OBJECTIVE: To identify and compare objective and self-perceived characteristics of patients with chronic obstructive pulmonary disease, who do and do not choose rehabilitation. SUBJECTS: The study comprised 205 consecutive patients with mild to very severe chronic obstructive pulmonary disease. They chose either inpatient rehabilitation (n = 161) or ordinary outpatient consultations (n = 44). MEASUREMENTS: Disease severity was assessed with spirometric tests, health-related quality of life was assessed with the St George's Respiratory Questionnaire, and mental status was measured using the Hospital Anxiety and Depression Scale. Socio-demographic and social characteristics, and co-morbidity variables were available. RESULTS: Patients in the rehabilitation group had a lower level of overall health-related quality of life (63.8 vs 47.6, p = 0.000) and a higher prevalence of anxiety (34.6% vs 13.6%, p = 0.007) than the outpatients. The outpatients received more psychological support from spouse/partner than patients in the rehabilitation group (70.5% vs 49.1%, p = 0.012). There were no differences in disease severity and co-morbidity. CONCLUSION: The decision to choose rehabilitation may be determined by impaired health-related quality of life, psychological distress and lack of psychological support from a significant other. Our findings suggest that patients with chronic obstructive pulmonary disease are conscious of their overall health status and the necessary treatment to maintain or improve it. | Male | Humans | Patient Participation | Questionnaires | OK |
| Gianmaria | Gioga |  |  |  |  | Child & Family Social Work | 2013-09-10 septembre 10, 2013 | Abstract This paper reports research carried out in Italy designed to investigate the usability of Child Well‐Being Scale (CWBS) for the outcome evaluation of home‐care interventions for vulnerable families and children in need. Using a pre‐ and post‐test design, the study traces the changes in 18 vulnerable families and 23 children in need included in a programme of home‐care intervention over a period of 11 months. All the families and children were assessed twice: at intake and at the end of the intervention (after 6 months). Furthermore, 10 families and 11 children had a longer intervention and were assessed three times. Moreover, two focus groups involving 13 home‐care workers and 11 face‐to‐face interviews were used to collect practitioners' points of views on CWBS. The results generally support the idea that families' and children's situation improved over time, as shown by an improvement in almost all of the considered dimensions after 6 months and after 11 months. Specifically, the families improved more on household adequacy in the long term while children on the child performance dimension improved in the short term. Practitioners reported that CWBS was an aid to multi‐professional decision‐making, as the systematic evaluation of the subscales was a practical base upon which to activate shared decision‐making during the casework. (PsycINFO Database Record (c) 2014 APA, all rights reserved). (journal abstract) | No terms assigned | children in need | home‐care intervention | needs assessment | OK |
|  |  |  |  |  |  | Journal of Medical Ethics | 2010-04-00 Apr 2010 | BACKGROUND: Central to the involvement of children in health research is the notion of risk. In this paper we present one of the factors, a matter of trust, that shaped Canadian parents' and children's perceptions and assessments of risk in child health research. PARTICIPANTS AND METHODS: Part of a larger qualitative research study, 82 parents took part an in-depth qualitative interview, with 51 parents having children who had participated in health research and 31 having children with no research history. 51 children ranging from 6 to 19 years of age were also interviewed, with 28 having a history of participation in child health research and 23 having no history. Children also took part in 3 focus groups interviews. Themes emerged through a grounded theory analysis of coded interview transcripts. FINDINGS: The presence or absence of trust was not only perceived by parents and children as a contributing factor to involving children in health research, but also shaped their perceptions and assessments of risk. Three interrelated subthemes identified were: (1) relationships of trust; (2) placing trust in symbols of authority; and (3) the continuum of trust. CONCLUSIONS: Our study reinforces that trust is an important factor when parents assess risk in child health research and shows that children use the language of trust in relation to risk. More discussion regarding trust in training researchers is warranted given the trust in researchers and institutions evident in this study. We also recommend further study of the continuum of trust in child health research. | Humans | Patient Participation | Physician-Patient Relations | Questionnaires | OK |
| Jennifer | McAleer |  |  |  |  | Australian Occupational Therapy Journal | 2010-04-00 Apr 2010 | AIMS: This study aimed to describe participation at home, school and in the community of Australian children who had sustained an acquired brain injury (ABI). Parent ratings regarding the impact of cognitive, motor and behavioural impairments on participation were obtained. In addition, the influence of environmental factors on participation was investigated. METHODOLOGY: This study used a cross-sectional design with convenience sampling to recruit 20 children who attended a rehabilitation review clinic between September 2006 and September 2007. Participants completed the Child and Family Follow-up Survey (Bedell, 2004) to describe the participation of their children in home, school and community settings. The CFFS was developed based on the International Classification of Function, and uses parent report to measure the impact of impairments and environmental factors on children's participation in home, school and community life. RESULTS: The children were reported to have the greatest participation restrictions for structured events in the community, and social, play or leisure activities with peers either at school or in the community. Children were least restricted moving about in and around their own homes. CONCLUSIONS: This research describes difficulties encountered by Australian children with ABI in participating in community-based activities with their same aged peers. This study adds to the current literature describing patterns of participation of United States children who have sustained brain injuries, and provides useful information for Australian therapists to consider when addressing children's return to school and engagement with their peers following brain injury. | Male | Humans | Patient Participation | Questionnaires | OK |
| Martine M. | Versluijs | Harm J. | Smeets | Job | Kievit | Medical Decision Making: An International Journal of the Society for Medical Decision Making | 2010-10-00 2010 Sep-Oct | OBJECTIVE: To assess whether patients use information on quality of care when choosing a hospital for surgery compared with more general hospital information. METHODS: In this cross-sectional study in 3 Dutch hospitals, questionnaires were sent to 2122 patients who underwent 1 of 6 elective surgical procedures in 2005-2006 (aorta reconstruction [for treatment of aneurysm], cholecystectomy, colon resection, inguinal hernia repair, esophageal resection, thyroid surgery). Patients were asked which information they had used to choose this hospital and which information they intended to use if they would need similar surgical treatment in the future. RESULTS: In total, 1329 questionnaires were available for analysis (response rate 62.6%). Most patients indicated having used the hospital's good reputation (69.1%) and friendly hospital atmosphere (63.3%) to choose a hospital. For future choices, most patients intended to use the fact that they were already treated in that hospital (79.3%) and the hospital's good reputation (74.1%). Regarding quality-of-care information, patients preferred a summary measure (% patients with ''textbook outcome'') over separate more detailed measures (52.1% v. 38.0%, χ2 = 291, P < 0.01). For future choices, patients intend to use more information items than in 2005-2006, both in absolute terms (9 v. 4 items, t = 38.3, P < 0.01) as relative to the total number of available items (41.3% [40.1%-42.5%] v. 29.2% [28.1%-30.2%]). CONCLUSION: Patients intended to use more information for future choices than they used for past choices. For future choices, most patients prefer a summary measure on quality of care over more detailed measures but seem to value that they were already treated in that hospital or a hospital's good reputation even more. | Male | Humans | Patient Participation | Questionnaires | OK |
| J. | Dijs-Elsinga | H. J. | Smeets | J. | Kievit | Medical Decision Making: An International Journal of the Society for Medical Decision Making | 2012-12-00 2012 Nov-Dec | OBJECTIVE: Publicly available information on hospital performance is increasing, with the aim to support consumers when choosing a hospital. Besides general hospital information and information on outcomes of care, there is increasing availability of systematically collected information on experiences of other patients. The aim of this study was to assess the influence of previous patients' experiences relative to other information when choosing a hospital for surgical treatment. METHODS: Three hundred thirty-seven patient volunteers and 280 healthy volunteers (response rate of 52.4% and 93.3%, respectively) filled out an Internet-based questionnaire that included an adaptive choice-based conjoint analysis. They were asked to select hospital characteristics they would use for future hospital choice, compare hospitals, and choose the overall best hospital. Based on the respondents' choices, the relative importance (RI) of each hospital characteristic for each respondent was estimated using hierarchical Bayes estimation. RESULTS: Information based on previous patients' experience was considered at least as important as information provided by hospitals. "Report card regarding physician's expertise" had the highest RI (16.83 [15.37-18.30]) followed by "waiting time for outpatient clinic appointment" (14.88 [13.42-16.34]) and "waiting time for surgery" (7.95 [7.12-8.78]). Patient and healthy volunteers considered the same hospital attributes to be important, except that patient volunteers assigned greater importance to "positive judgment about physician communication" (7.65 v. 5.80, P < 0.05) and lower importance to "complications" (2.56 v. 4.22, P < 0.05). CONCLUSION: Consumers consider patient experience-based information at least as important as hospital-based information. They rely most on information regarding physicians' expertise, waiting time, and physicians' communication when choosing a hospital. | Humans | Patient Participation | Questionnaires | Internet | OK |
| Mark | Robbins | David | Daikh | Ashley | Beall | Arthritis Care & Research | 2013-03-00 Mar 2013 | OBJECTIVE: We sought to develop a list of 5 tests, treatments, or services commonly used in rheumatology practice whose necessity or value should be questioned and discussed by physicians and patients. METHODS: We used a multistage process combining consensus methodology and literature reviews to arrive at the American College of Rheumatology's (ACR) Top 5 list. Rheumatologists from diverse practice settings generated items using the Delphi method. Items with high content agreement and perceived high prevalence advanced to a survey of ACR members, who comprise >90% of the US rheumatology workforce. To increase the response rate, a nested random sample of 390 rheumatologists received more intensive survey followup. The samples were combined and weighting procedures were applied to ensure generalizability. Items with high ratings underwent literature review. Final items were then selected and formulated by the task force. RESULTS: One hundred five unique items were proposed and narrowed down to 22 items during the Delphi rounds. A total of 1,052 rheumatologists (17% of those contacted) participated in the member-wide survey, whereas 33% of those in the nested random sample participated; respondent characteristics were similar in both samples. Based on survey results and available scientific evidence, 5 items (relating to antinuclear antibodies, Lyme disease, magnetic resonance imaging, bone absorptiometry, and biologic therapy for rheumatoid arthritis) were selected for inclusion. CONCLUSION: The ACR Top 5 list is intended to promote discussions between physicians and patients about health care practices in rheumatology whose use should be questioned and to assist rheumatologists in providing high-value care. | Humans | Patient Participation | physicians | United States | OK |
| Ronald M. | Andersen | James | McGuire | Lisa | Rubenstein | Medical Care | 2013-03-00 Mar 2013 | BACKGROUND: Although vulnerable populations may benefit from in-home health information technologies (HIT) that promote disease self-management, there is a "digital divide" in which these groups are often unlikely to use such programs. We describe the early phases of applying and testing an existing Veterans Affairs (VA) HIT-care management program, Care Coordination Home Telehealth (CCHT), to recently homeless Veterans in the US Department of Housing and Urban Development-VA Supportive Housing (HUD-VASH) program. Peers were used to support patient participation. METHODS: CCHT uses in-home messaging devices to provide health education and daily questions about clinical indicators from chronic illness care guidelines, with patient responses reviewed by VHA nurses. Patients could also receive adjunctive peer support. We used medical record review, Veteran interviews, and staff surveys to "diagnose" barriers to CCHT use, assess program acceptability, explore the role of peer support, and inform future quality improvement. SUBJECTS: Fourteen eligible Veterans in HUD-VASH agreed to CCHT participation. Ten of these Veterans opted to have adjunctive peer support and the other 4 enrolled in CCHT usual care. RESULTS: Although barriers to enrollment/engagement must be addressed, this subset of Veterans in HUD-VASH was satisfied with CCHT. Most Veterans did not require support from peers to engage in CCHT but valued peer social assistance amidst the isolation felt in their scattered-site homes. CONCLUSIONS: HIT tools hold promise for in-home care management for recently housed Veterans. Patient-level barriers to enrollment must be addressed in the next steps of quality improvement, testing and evaluating peer-driven CCHT recruitment. | Male | Humans | Questionnaires | Aged | OK |
|  |  |  |  |  |  | Social Work | 2011-01-00 Jan 2011 | "Client participation" is a popular ideal and object of rhetorical commitment in social work service. But the much-touted potential of this concept requires careful and critical scrutiny. This article reports on a study of client-participation initiatives in the Hong Kong welfare sector. The study identified significant differences in the institutional structure ofclient-participation initiatives and their social dynamics between service units targeting elderly people or people with disabilities and those targeting a clientele with supposed moral or psychosocial failures. The findings suggest that client-participation mandates allowing sustained interaction with service users through regular membership in a structure for discussion are more effective than ad hoc measures with unstable participation, assuming that the goal is mutuality and trust in cooperative inquiry with service users. The findings also suggest that both a service provider's genuine belief in the primacy of the users' voice and a user's legitimate claim of experiential knowledge are imperative to realizing the potential of client participation. | Decision Making | Humans | Patient Participation | Patient Satisfaction | OK |
| Samereh | Abdoli | Mohammad Bagher | Saberizafarghandi | |  | Journal of Interprofessional Care | 2012-01-00 Jan 2012 | Interprofessional collaboration (IPC) has long been identified as a way of providing optimal mental health services (MHS). It is important, therefore, to identify and facilitate factors driving IPC. This paper presents health professionals' experiences and perceptions regarding the clients' role as a driving factor for IPC in MHS in an Iranian context. Health professionals included nurses, physicians (general physicians and medical specialists), psychologists and consultants. Qualitative methods were employed in the form of in-depth individual interviews and focus groups to collect data from 24 professionals and 4 clients. Data analysis generated four key themes: "Clients as axis of IPC", "Seamless services as a common incentive for IPC", "Clients as a common interprofessional linkage" and "Clients as a driving force to achieve IPC". From the professionals' perspective, it was found that clients had a mediating role in their collaborative relationships and practices. These findings are discussed in relation to the interprofessional literature. This paper goes on to argue that professionals need to be aware of and be trained about how to manage, as well as benefit from, the clients' fundamental role in IPC. | Male | Humans | Patient Participation | Questionnaires | OK |
| Stan D. | Musgrave | Shweta | Malhotra | Amanda J. | Lee | BMJ (Clinical research ed.) | 2012-00-00 2012 | OBJECTIVE: To determine whether mobile phone based monitoring improves asthma control compared with standard paper based monitoring strategies. DESIGN: Multicentre randomised controlled trial with cost effectiveness analysis. SETTING: UK primary care. PARTICIPANTS: 288 adolescents and adults with poorly controlled asthma (asthma control questionnaire (ACQ) score ≥ 1.5) from 32 practices. INTERVENTION: Participants were centrally randomised to twice daily recording and mobile phone based transmission of symptoms, drug use, and peak flow with immediate feedback prompting action according to an agreed plan or paper based monitoring. MAIN OUTCOME MEASURES: Changes in scores on asthma control questionnaire and self efficacy (knowledge, attitude, and self efficacy asthma questionnaire (KASE-AQ)) at six months after randomisation. Assessment of outcomes was blinded. Analysis was on an intention to treat basis. RESULTS: There was no significant difference in the change in asthma control or self efficacy between the two groups (ACQ: mean change 0.75 in mobile group v 0.73 in paper group, mean difference in change -0.02 (95% confidence interval -0.23 to 0.19); KASE-AQ score: mean change -4.4 v -2.4, mean difference 2.0 (-0.3 to 4.2)). The numbers of patients who had acute exacerbations, steroid courses, and unscheduled consultations were similar in both groups, with similar healthcare costs. Overall, the mobile phone service was more expensive because of the expenses of telemonitoring. CONCLUSIONS: Mobile technology does not improve asthma control or increase self efficacy compared with paper based monitoring when both groups received clinical care to guidelines standards. The mobile technology was not cost effective. TRIAL REGISTRATION: Clinical Trials NCT00512837. | Male | Humans | Patient Participation | Questionnaires | OK |
| Paul L. | Nguyen | Jeanette Y. | Ziegenfuss | Leona C. | Han | The Journal of Urology | 2013-06-00 Jun 2013 | PURPOSE: Although clinical guidelines recommend assessing quality of life, cancer aggressiveness and life expectancy for making localized prostate cancer treatment decisions, it is unknown whether instruments that objectively measure such outcomes have disseminated into clinical practice. In this context we determined whether quality of life and prediction instruments for prostate cancer have been adopted by radiation oncologists and urologists in the United States. MATERIALS AND METHODS: Using a nationally representative mail survey of 1,422 prostate cancer specialists in the United States, we queried about self-reported clinical implementation of quality of life instruments, prostate cancer nomograms and life expectancy prediction tools in late 2011. The Pearson chi-square test and multivariate logistic regression were used to determine differences in the use of each instrument by physician characteristics. RESULTS: A total of 313 radiation oncologists and 328 urologists completed the survey for a 45% response rate. Although 55% of respondents reported using prostate cancer nomograms, only 27% and 23% reported using quality of life and life expectancy prediction instruments, respectively. On multivariate analysis urologists were less likely to use quality of life instruments than radiation oncologists (OR 0.40, p <0.001). Physicians who spent 30 minutes or more counseling patients were consistently more likely to use quality of life instruments (OR 2.57, p <0.001), prostate cancer nomograms (OR 1.83, p = 0.009) and life expectancy prediction tools (OR 1.85, p = 0.02) than those who spent less than 15 minutes. CONCLUSIONS: Although prostate cancer nomograms have been implemented into clinical practice to some degree, the use of quality of life and life expectancy tools has been more limited. Increased attention to implementing validated instruments into clinical practice may facilitate shared decision making for patients with prostate cancer. | Male | Humans | Questionnaires | United States | OK |
|  |  |  |  |  |  | Ugeskrift for Laeger | 2010-03-08 Mar 8, 2010 | The idea of user involvement in the field of health care has evolved over the past 30 years. Over time the perception has changed to increasingly expect health care providers to involve users as active partners in treatment and in the development of services. Clinical microsystems is a strategy for quality improvement that has user involvement as its key element. The Region of Southern Denmark has worked systematically to introduce this approach at regional hospitals. Experience shows that clinical microsystems has the potential to improve user involvement and bring more quality into health care. | Humans | Patient Participation | Patient Satisfaction | Questionnaires | OK |
| Daniel | Dohan | E. | Shelley Hwang | Michelle | Melisko | Cancer causes & control: CCC | 2013-05-00 May 2013 | PURPOSE: Patient participation in cancer clinical trials is imperative to the advancement of medical science. Physicians play an important role in recruitment by discussing clinical trials with their cancer patients. Patient-physician discussion is influenced by many factors relating to the physician, the patient, and the healthcare system. METHODS: Physicians selected from the 2008-2009 American Medical Association Physician Masterfile who practiced in California, Florida, Illinois, or New York and specialized in medical oncology, surgery, or radiation oncology were surveyed about their attitudes and practices with respect to breast cancer clinical trials. Practice types were categorized according to the classifications provided by the American College of Surgeons, and clinical trial and practice addresses were geocoded. RESULTS: Surveys were completed by 706 of 1,534 eligible physicians (46 %). Medical oncologists were more likely than surgical or radiation oncologists to discuss the possibility, benefits, and risks of clinical trial enrollment with their breast cancer patients. Physicians who spent the most time in patient care were least likely to discuss clinical trials with their patients. Distance from a physician's practice to the nearest clinical trial site was inversely associated with referral and recruitment. Perceived barriers to clinical trial participation were associated with greater referral activity suggesting that physicians who were more involved in trials were also more likely to understand barriers to participation. CONCLUSIONS: Multilevel interventions may be successful at increasing participation of women in clinical trials. | Male | Humans | Physician-Patient Relations | Questionnaires | OK |
| Kjetil | Retterstøl | Leiv | Ose |  |  | Tidsskrift for Den Norske Lægeforening: Tidsskrift for Praktisk Medicin, Ny Række | 2010-08-26 Aug 26, 2010 | BACKGROUND: In relation to the large number of clinical trials performed, little is written about how the study subjects have experienced their participation. The article presents the results from a questionnaire survey among participants in 18 clinical trials undertaken at the Lipid Clinic, Rikshospitalet, Norway in 2005 and 2006. MATERIAL AND METHODS: A study-specific questionnaire was developed and comprised 17 questions about study participation. Response was anonymous. Results are given as percentage distribution of answers. RESULTS: 56 % (287 persons) completed the questionnaire. Motivation for study participation was twofold, both personal health gains and the desire to contribute to research and development are important motives. 28 % [corrected] thought the new drug could be beneficial for them. Study participants responded the following: 90 % meant that the risk of adverse events and harmful effects of the study drug was small, 73 % that follow-up was good, 85 % that they had received sufficient information about the study and 77 % that study participation had no disadvantages. 73 % thought it had a positive health impact for them to be involved in the study. INTERPRETATION: Participants in our clinical trials are concerned about their own health, but they also want to contribute to research for the well-being of others. Study participants seem to overestimate the health benefits associated with use of the study drug. Frequent medical follow-up and guidance in diet and lifestyle are benefits associated with participation in clinical studies, but the effects are difficult to measure. | Male | Humans | Patient Participation | Questionnaires | OK |
| Alex | Rybkin | Kiren | Leeds | Dominick L. | Frosch | BMJ quality & safety | 2014-11-00 Nov 2014 | BACKGROUND AND OBJECTIVES: Increased computer tomography (CT) scan use has contributed to a rise in medically-associated radiation exposure. The extent to which clinicians consider radiation exposure when ordering imaging tests is unknown. We examined (1) outpatient clinician attitudes towards considering radiation exposure when ordering CT scans; and (2) clinician reactions to displaying radiation exposure information for CT scans at clinician electronic order entry. METHODS: We conducted nine focus groups with primary care clinicians and subspecialty physicians (nephrology, pulmonary and neurology) (n=50) who deliver outpatient care across 12 hospital-based clinics and community health centres in an urban safety-net health system, which use a common electronic order entry system. We analysed focus group transcripts using an inductive framework to identify emergent themes and illustrative quotations. FINDINGS: Clinicians felt they had limited knowledge of the clinical implications of radiation exposure. Many believed clinically relevant information such as the increased risk of malignancy from CT scans would be useful to inform decision-making and patient-clinician discussions. Clinicians noted that patient vulnerability and long wait times for tests with less radiation exposure (such as MRI or ultrasound) often acted as barriers to minimise patient radiation exposure from CT scans. Clinicians suggested providing patients' cumulative radiation exposure or formal decision aids to improve the usefulness of the radiation exposure information. CONCLUSIONS: Displaying clinically relevant radiation exposure information at order entry may improve clinician knowledge and inform patient-clinician discussions regarding risks and benefits of imaging. However, limited access to tests with lower radiation exposure in safety-net settings may trump efforts to minimise patient radiation exposure. | | | | | OK |
| I.-hsuan | Shen | I.-Shu | Liu | Lin-ju | Kang | Research in Developmental Disabilities | 2013-05-00 May 2013 | This study examines the criterion-related validity and clinimetric properties of the Assessment of Preschool Children's Participation (APCP) for children with cerebral palsy (CP). Eighty-two children with CP (age range, two to five years and 11 months) and their caregivers participated in this study. The APCP consists of diversity and intensity scores in the areas of play (PA), skill development (SD), active physical recreation (AP), social activities (SA), and total areas. Tests were administered at baseline and at six-month follow-up. Concurrent and predictive validities were identified by assessing the strength of correlations between APCP scores and criterion-related measures--the 66-item Gross Motor Function Measure (GMFM-66) and Functional Independence Measure for Children (WeeFIM). Responsiveness was measured by standardized response mean (SRM). Minimal detectable change (MDC) at the 95% confidence level (MDC95) and minimal clinically important difference (MCID) were analyzed. The APCP with GMFM-66 and WeeFIM had fair to excellent concurrent validity (r=0.39-0.85) and predictive validity (r=0.46-0.82). The SRM values of the APCP diversity and intensity scales in all areas were 0.8-1.3. The MDC95 and MCID ranges for all areas (i.e., PA, SD, AP, SA, and total areas) were 0.1-0.7 and 0.4-1.2 points for intensity scores, respectively, and 4-17% and 10-19% for diversity scores, respectively. Therefore, the APCP scale was markedly responsive to change. Clinicians and researchers can use these clinimetric APCP data to determine whether a change score represents a "true" or clinically meaningful effect at post-treatment and follow-up. | Male | Humans | Patient Participation | Female | OK |
| Kasavan | Naidoo | Nonhlanhla | Yende | Tanuja | Gengiah | Contemporary Clinical Trials | 2011-05-00 May 2011 | BACKGROUND: In settings where multiple HIV prevention trials are conducted in close proximity, trial participants may attempt to enroll in more than one trial simultaneously. Co-enrollment impacts on participant's safety and validity of trial results. We describe our experience, remedial action taken, inter-organizational collaboration and lessons learnt following the identification of co-enrolled participants. EXPERIENCES: Between February and April 2008, we identified 185 of the 398 enrolled participants as ineligible. In violation of the study protocol exclusion criteria, there was simultaneous enrollment in another HIV prevention trial (ineligible co-enrolled, n=135), and enrollment of women who had participated in a microbicide trial within the past 12 months (ineligible not co-enrolled, n=50). Following a complete audit of all enrolled participants, ineligible participants were discontinued via study exit visits from trial follow-up. Custom-designed education program on co-enrollment impacting on participants' safety and validity of the trial results was implemented. Shared electronic database between research units was established to enable verification of each volunteer's trial participation and to prevent future co-enrollments. LESSONS LEARNT: Interviews with ineligible enrolled women revealed that high-quality care, financial incentives, altruistic motives, preference for sex with gel, wanting to increase their likelihood of receiving active gel, perceived low risk of discovery and peer pressure are the reasons for their enrollment in the CAPRISA 004 trial. CONCLUSION: Instituting education programs based on the reasons reported by women for seeking enrollment in more than one trial and using a shared central database system to identify co-enrollments have effectively prevented further co-enrollments. | Humans | Patient Participation | Young Adult | Female | OK |
| Amy E. | Kupper | Bradley S. | Galer | Arnold R. | Gammaitoni | The Clinical Journal of Pain | 2013-05-00 May 2013 | OBJECTIVE: To revise the Pain Quality Assessment Scale (PQAS) using feedback from patients to further increase its validity. METHODS: This project involved 3 cognitive interviewing studies. In Study 1, a group of patients with chronic pain (N=20) were asked a series of questions regarding the PQAS's understandability, and invited to make suggestions regarding how the measure could be improved. In Study 2, a second group of patients (N=21) responded to questions about a modified version of the PQAS. The PQAS was further modified on the basis of the findings of Study 2, and in Study 3 the participants were asked to indicate whether the changes made improved the understandability of the PQAS further. RESULTS: The participants in Studies 1 and 2 identified portions of the PQAS instructions and some of the PQAS items that could be modified to increase their understandability. Modifications resulted in a revised PQAS that was deemed by patients with chronic pain to be more understandable than the original PQAS by the majority of participants. DISCUSSION: testing can be used to improve the understandability of pain measures. The results of cognitive testing with the PQAS indicated that much of the content of the original instructions and items were understandable as written, but that minor changes could be made to make them even clearer to patients with chronic pain. The changes made resulted in a revised PQAS that is more understandable and may therefore be even more useful than with the original PQAS. | Male | Humans | Patient Participation | Questionnaires | OK |
| Gustavo Sátollo | Rolim | Antônio Bento Alves de | Moraes |  |  | Ciência & Saúde Coletiva | 2011-10-00 Oct 2011 | Situations of non-cooperation/participation of the patient are detected in healthcare,. In many cases, it is impossible to complete the assessment procedures, and procedures of intervention may be prejudiced. This problem has been researched in some health areas, however there are few studies involving activities in Speech Therapy. The scope of this study was to conduct a survey of knowledge and strategies of Speech Therapists when faced with situations considered difficult in clinical practice with children. Semi-structured interviews were staged with students and teachers of an undergraduate course in Speech Therapy at a Brazilian public university, addressing problems encountered in caring for children 4 to 12-year-olds, as well as proposed solutions. Analysis of content of the data involved the construction of a system of categories of reported and proposed strategies. The categories were compared with those obtained in an analogous survey (Pediatric Dental care) and the analysis highlighted specifics of care of each of the professions. The results contributed to the systematization of knowledge relating to collaboration/participation, and revealed the concern of the participants with the humanization of care. | Humans | Patient Participation | Questionnaires | Cooperative Behavior | OK |
| John | Kirwan | Marie | Urban | Sarah | Hewlett | Arthritis Care & Research | 2010-11-00 Nov 2010 | OBJECTIVE: Patient-reported outcome measures (PROMs) need to include concepts and language relevant to patients and be easily understood. These studies aimed to develop draft PROMs to measure rheumatoid arthritis (RA) fatigue and its impact by collaborating with patients to identify language and experiences, create draft PROM items, and test them for comprehension, with decisions supported throughout by a patient research partner. METHODS: In study 1, interview transcripts of RA patients describing their fatigue (n = 15) were subjected to content and inductive thematic analysis to identify fatigue language and experiences. In study 2, 3 focus groups of RA patients (n = 17) explored these and developed the wording for visual analog scales (VAS) and identical numerical rating scales (NRS), then a draft multi-item questionnaire was developed with the patient research partner. Study 3 comprised 15 RA patients who completed the PROMs during cognitive interviewing to explore understanding. RESULTS: Studies 1 and 2 identified key patient terminology (fatigue, exhaustion) and 12 potential fatigue concepts (Cognition, Coping, Duration, Emotion, Energy, Frequency, Impact, Planning, Quality of Life, Relationships, Sleep, and Social Life). Patients' proposals were clarified into draft screening VAS/NRS for fatigue severity, effect, and coping, plus a draft 45-item questionnaire. Study 3 showed that 14 questions required clarification or revision of response options. CONCLUSION: Collaboration with patients enabled development of draft RA fatigue PROMs grounded in the patient data, strengthening face and content validity and ensuring comprehension. The draft conceptual framework that emerged has resulted in draft PROMS ready for item reduction, and testing of construct and criterion validity and reliability. | Male | Humans | Patient Participation | Questionnaires | OK |
| Bauke | Koekkoek | Ad | Kerkhof | Aartjan | Beekman | BMC psychiatry | 2011-00-00 2011 | BACKGROUND: Structured psychotherapy is recommended as the preferred treatment of personality disorders. A substantial group of patients, however, has no access to these therapies or does not benefit. For those patients who have no (longer) access to psychotherapy a Collaborative Care Program (CCP) is developed. Collaborative Care originated in somatic health care to increase shared decision making and to enhance self management skills of chronic patients. Nurses have a prominent position in CCP's as they are responsible for optimal continuity and coordination of care. The aim of the CCP is to improve quality of life and self management skills, and reduce destructive behaviour and other manifestations of the personality disorder. METHODS/DESIGN: Quantitative and qualitative data are combined in a comparative multiple case study. This makes it possible to test the feasibility of the CCP, and also provides insight into the preliminary outcomes of CCP. Two treatment conditions will be compared, one in which the CCP is provided, the other in which Care as Usual is offered. In both conditions 16 patients will be included. The perspectives of patients, their informal carers and nurses are integrated in this study. Data (questionnaires, documents, and interviews) will be collected among these three groups of participants. The process of treatment and care within both research conditions is described with qualitative research methods. Additional quantitative data provide insight in the preliminary results of the CCP compared to CAU. With a stepped analysis plan the 'black box' of the application of the program will be revealed in order to understand which characteristics and influencing factors are indicative for positive or negative outcomes. DISCUSSION: The present study is, as to the best of our knowledge, the first to examine Collaborative Care for patients with severe personality disorders receiving outpatient mental health care. With the chosen design we want to examine how and which elements of the CC Program could contribute to a better quality of life for the patients. TRIAL REGISTRATION: Netherlands Trial Register (NTR): NTR2763. | Humans | Patient Participation | Questionnaires | Caregivers | OK |
| Magdi | Shehata | Mohamed | Shams | Mervat | Elshabrawy | Journal of Medical Ethics | 2010-09-00 Sep 2010 | OBJECTIVE: To determine the attitudes of Egyptian patients regarding their participation in research and with the collection, storage and future use of blood samples for research purposes. DESIGN: Cross-sectional survey. STUDY POPULATION: Adult Egyptian patients (n=600) at rural and urban hospitals and clinics. RESULTS: Less than half of the study population (44.3%) felt that informed consent forms should provide research participants the option to have their blood samples stored for future research. Of these participants, 39.9% thought that consent forms should include the option that future research be restricted to the illness being studied. A slight majority (66.2%) would donate their samples for future genetic research. Respondents were more favourable towards having their blood samples exported to other Arab countries (62.0%) compared with countries in Europe (41.8%, p<0.001) and to the USA (37.2%, p<0.001). CONCLUSIONS: This study shows that many individuals do not favour the donation of a blood sample for future research. Of those who do approve of such future research, many favour a consent model that includes an option restricting the future research to the illness being studied. Also, many Egyptians were hesitant to have their blood samples donated for genetic research or exported out of the Arab region to the USA and European countries. Further qualitative research should be performed to determine the underlying reasons for many of our results. | Male | Humans | Patient Participation | Female | OK |
| Stacy M. | Carter | Glenn P. | Salkeld | James A. | Gillespie | BMC gastroenterology | 2012-00-00 2012 | BACKGROUND: There are many test options available for colorectal cancer screening. The choice of test relates to the objectives of those offering or considering screening. DISCUSSION: While all screening programs aim to detect disease early in order to improve the length and/or quality of life for the individual, some organizations and individuals prefer screening tests that offer the opportunity for cancer prevention. Others favor maximizing participation or the opportunity for shared decision-making, including discussion of information on test quality and availability. We propose three additional objectives for screening: minimizing harms, optimizing economic efficiency and maximizing equity of access to screening. SUMMARY: Applying these objectives to colorectal cancer screening, we advocate the use of immunochemical FOBTs as the preferred screening strategy, as it satisfies all three of these important objectives. | Humans | Health Care Costs | Colorectal Neoplasms | Health Services Accessibility | OK |
| Julie | Aldridge | Ilona | Juraskova | Karin | Ribi | Psycho-Oncology | 2012-12-00 Dec 2012 | BACKGROUND: The International Breast Cancer Study Group conducted a phase III trial in Australian/New Zealand (ANZ) and Swiss/German/Austrian (SGA) centres on training doctors in clear and ethical information delivery about treatment options and strategies to encourage shared decision making. METHODS: Medical, surgical, gynaecological and radiation oncologists, and their patients for whom adjuvant breast cancer therapy was indicated, were eligible. Doctors were randomised to participate in a workshop with standardised teaching material and role playing. Patients were recruited in the experimental and control groups before and after the workshop. RESULTS: In ANZ centres, 21 eligible doctors recruited a total of 304 assessable patients. In SGA centres, 41 doctors recruited 390 patients. The training was well accepted. There was no overall effect on patient decisional conflict (primary endpoint) 2 weeks after the consultation. Overall, patients were satisfied with their treatment decision, their consultation and their doctors' consultation skills. Considerable variation was observed in patient outcomes between SGA and ANZ centres; the effect sizes of the intervention were marginal (<0.2). CONCLUSIONS: Shared decision making remains a challenge. A sustained training effect may require more intensive training tailored to the local setting. Cross-cultural differences need attention in conducting trials on communication interventions. | Male | Decision Making | Humans | Patient Participation | OK |
| C. | Dayoub | C. | Wakefield | J. | Homewood | Transplantation Proceedings | 2013-02-00 2013 Jan-Feb | BACKGROUND: Organ transplantation is a significant medical advancement. However, number of individuals registering as donors and next-of-kin's refusal to grant consent are notable limitations on the availability of organs for transplantation. Therefore, investigation into factors that influence willingness to donate and consent decisions is warranted. We examined the relationship between attitudes and family communication in relation to consent decisions for self and family member organ donation (OD). Data were collected from students at an Australian university and individuals from the wider community (N = 267). METHOD: Participants completed an on-line survey composed of questions relating to demographic information, questionnaires from previous research, and several single items relating to family communication and consent created specifically for the study. The main outcome measure was participants' willingness to consent to OD for themselves or for a family member. RESULTS: Attitudes and prior discussion of OD were predictive of registration and willingness to consent. Positive attitudes were also related to previous conversations regarding OD. A more open level of communication within families was associated with an increased tendency to discuss OD, but was not directly related to consent decisions. CONCLUSION: Findings reiterate the importance of promoting positive attitudes within the community and specific, informed discussion within families. The positive influence these factors exert on next-of-kin decisions may be vital to maximize donation rates in opt-in systems (such as Australia). | Male | Decision Making | Humans | Patient Participation | OK |
| Catherine | Slota | Dennis | Williams | Gail | Tudor | Pediatrics | 2012-10-00 Oct 2012 | OBJECTIVE: Our objectives were to examine how certain aspects of provider-patient communication recommended by national asthma guidelines (ie, provider asking for child and caregiver input into the asthma treatment plan) were associated with child asthma medication adherence 1 month after an audio-taped medical visit. METHODS: Children ages 8 through 16 with mild, moderate, or severe persistent asthma and their caregivers were recruited at 5 pediatric practices in nonurban areas of North Carolina. All medical visits were audio-tape recorded. Children were interviewed 1 month after their medical visits, and both children and caregivers reported the child's control medication adherence. Generalized estimating equations were used to determine if communication during the medical visit was associated with medication adherence 1 month later. RESULTS: Children (n = 259) completed a home visit interview ∼1 month after their audio-taped visit, and 216 of these children were taking an asthma control medication at the time of the home visit. Children reported an average control medication adherence for the past week of 72%, whereas caregivers reported the child's average control medication adherence for the past week was 85%. Child asthma management self-efficacy was significantly associated with both child- and caregiver-reported control medication adherence. When providers asked for caregiver input into the asthma treatment plan, caregivers reported significantly higher child medication adherence 1 month later. CONCLUSIONS: Providers should ask for caregiver input into their child's asthma treatment plan because it may lead to better control medication adherence. | Male | Humans | Patient Participation | Physician-Patient Relations | OK |
| E. | Farin |  |  |  |  | Schmerz (Berlin, Germany) | 2012-02-00 Feb 2012 | BACKGROUND: Successful treatment of chronic pain patients can be dependent on successful patient-provider communication. Patient-physician communication for patients with chronic back pain (CBP) has rarely been explored. The current study examines two questions: what is important for CBP patients concerning communication with the healthcare provider and are there interindividual differences in communication preferences dependent on sociodemographic characteristics? METHODS: Patient preferences were measured using the KOPRA questionnaire (32 items and 4 scales). The mean age of the 701 participating rehabilitants was 51 years (SD ±11.1 years) of which 60% were women. RESULTS: An effective and open communication with the healthcare physician was rated as the most important preference for CBP patients. Gender (depending on the scale: p = 0.01-0.82) and age (p = 0.01-0.12) seemed to influence some preferences in patient-provider communication. CONCLUSION: By taking into account individual communication preferences of CBP patients, healthcare providers could purposefully be responsive to these, possibly contributing to a more successful treatment. | Male | Humans | Patient Participation | Physician-Patient Relations | OK |
| Vin Shen | Ban | Simon J. | Crick | Rohitashwa | Sinha | British Journal of Neurosurgery | 2013-10-00 Oct 2013 | INTRODUCTION: Chronic subdural haematoma (CSDH) is one of the most common neurosurgical disorders and is especially prevalent in old age. The subdural evacuating port system (SEPS) has emerged in the last few years as a minimally invasive alternative to the standard procedure of burr-hole evacuation. NHS practice is evidence-driven and evidence from high-quality clinical studies is required prior to implementation of any changes. In the UK, the National Research Ethics Service (NRES) advises community consultation prior to starting a clinical trial, where the patient is unlikely to have capacity to consent to enrolment in the trial. To prepare for a trial comparing minimally invasive (SEPS) versus burr-hole evacuation for evacuation of a CSDH, we have designed and undertaken a pre-protocol community consultation survey to examine potential patient participation. MATERIAL(S) AND METHODS: The study population consisted of patients, family members and carers/friends in neurosurgical clinic waiting rooms and wards at Addenbrooke's Hospital, Cambridge, who individually completed a questionnaire (n = 215). RESULTS: Most respondents were willing to participate in the proposed randomised clinical trial (77%; 165/215). Moreover, 80% (171/215) and 74% (159/215) were willing to allow their next of kin and an independent consultant neurosurgeon to give surrogate consent, respectively. CONCLUSION: The results of our pre-protocol community consultation showed that not only would most respondents be willing to participate in the proposed trial, but also would be happy for either next of kin or an independent consultant neurosurgeon to give surrogate consent if they lacked capacity to consent themselves. The advantages of this type of survey are twofold: they increase patient and public involvement in the research process and allow researchers to design study protocols that are acceptable to the community. | Male | Humans | Questionnaires | Young Adult | OK |
| Matt G. | Mutchler | Brian | Risley | Bryce W. | McDavitt | AIDS education and prevention: official publication of the International Society for AIDS Education | 2012-02-00 Feb 2012 | Treatment advocacy (TA) programs, based in AIDS service organizations and clinics, aim to engage clients into care and support antiretroviral treatment (ART) adherence through client-centered counseling; advocate for patients with providers; and provide social service referrals. Systematic evaluations of TA are lacking. We conducted a non-randomized evaluation examining relationships of TA participation to adherence, care engagement, social services utilization, unmet needs, patient self-advocacy, and adherence self-efficacy among 121 HIV-positive clients (36 in TA, 85 not in TA; 87% male, 34% African American, 31% White, 19% Latino). In multivariate models, TA participants (vs. non-TA participants) showed higher electronically monitored [85.3% vs. 70.7% of doses taken; b(SE) = 13.16(5.55), p < .05] and self-reported [91.1% vs. 75.0%; b(SE) = 11.60(5.65), p < .05] adherence; utilized more social service programs [Ms = 5.2 vs. 3.4; b(SE) = 1.97(0.48), p < .0001]; and had fewer unmet social-service needs [Ms = 1.8 vs. 2.7; b(SE) = -1.06(0.48), p < .05]. Findings suggest the need for a randomized controlled trial of TA. | Male | Humans | Patient Participation | Questionnaires | OK |
| Alison Tytell | Brenner | Trisha Melinda | Crutchfield | Sarah Tropman | Hawley | JAMA internal medicine | 2013-03-11 Mar 11, 2013 | IMPORTANCE: To make good decisions about prostate-specific antigen (PSA) screening, men must consider how they value the different potential outcomes. OBJECTIVE: To determine the effects of different methods of helping men consider such values. DESIGN AND SETTING: Randomized trial from October 12 to 27, 2011, in the general community. PARTICIPANTS: A total of 911 men aged 50 to 70 years from the United States and Australia who had average risk. Participants were drawn from online panels from a survey research firm in each country and were randomized by the survey firm to 1 of 3 values clarification methods: a balance sheet (n = 302), a rating and ranking task (n = 307), or a discrete choice experiment (n = 302). INTERVENTION: Participants underwent a values clarification task and then chose the most important attribute. MAIN OUTCOME MEASURES: The main outcome was the difference among groups in the most important attribute. Secondary outcomes were differences in unlabeled test preference and intent to undergo screening with PSA. RESULTS: The mean age was 59.8 years; most participants were white and more than one-third had graduated from college. More than 40% reported a PSA test within 12 months. The participants who received the rating and ranking task were more likely to report reducing the chance of death from prostate cancer as being most important (54.4%) compared with those who received the balance sheet (35.1%) or the discrete choice experiment (32.5%) (P < .001). Those receiving the balance sheet were more likely (43.7%) to prefer the unlabeled PSA-like option (as opposed to the "no screening"-like option) compared with those who received rating and ranking (34.2%) or the discrete choice experiment (20.2%). However, the proportion who intended to undergo PSA testing was high and did not differ between groups (balance sheet, 77.1%; rating and ranking, 76.8%; and discrete choice experiment, 73.5%; P = .73). CONCLUSIONS AND RELEVANCE: Different values clarification methods produce different patterns of attribute importance and different preferences for screening when presented with an unlabeled choice. Further studies with more distal outcome measures are needed to determine the best method of values clarification, if any, for decisions such as whether to undergo screening with PSA. | Male | Decision Making | Humans | Patient Participation | OK |
| Marco | Silvani | Bruno | Andreoni | Luigi | Bisanti | The American Journal of Gastroenterology | 2010-01-00 Jan 2010 | OBJECTIVES: The objective of this study was to study predictors of patients' participation in colorectal cancer (CRC) screening. METHODS: Men and women, aged 55-64 years, were randomized to the following: (i) biennial fecal occult blood test (FOBT) delivered by mail (n=2,266); (ii) FOBT delivered by a general practitioner (GP)/screening facility (n=5,893); (iii) "once-only" sigmoidoscopy (FS) (n=3,650); (iv) FS followed by FOBT for screenees with negative FS (n=10,867); and (v) patient's choice between FS and FOBT (n=3,579). A stratified (by screening arm) random sample of attenders and nonattenders was contacted by trained interviewers 4 months after the initial invitation. Subjects giving their consent were administered a questionnaire (available online) investigating perceptions of individual CRC risk, attitudes toward prevention, adoption of health protective behaviors, and reasons for attendance/nonattendance. Adjusted prevalence odds ratios (ORs) were computed by multivariable logistic regression. RESULTS: The response rate was 71.9% (701 of 975) among nonattenders and 88.9% (773 of 870) among attenders. Adjusting for screening arm, center, gender, age, and education, participation was significantly higher among people who consulted their GP before undergoing screening (OR: 4.24; 95% confidence interval (CI): 3.11-5.78), who mentioned one first-degree relative with CRC (OR: 3.62; 95% CI: 2.02-6.49), who reported regular physical activity (OR: 1.85; 95% CI: 1.33-2.55), and who read the mailed information (letter only: OR: 1.85; 95% CI: 1.23-2.78; letter+leaflet: OR: 3.18; 95% CI: 2.12-4.76). People who considered screening to be ineffective (OR: 0.12; 95% CI: 0.08-0.19), those who considered it to be effective but reported even moderate levels of anxiety (OR: 0.32; 95% CI: 0.23-0.45), and those who mentioned previous knowledge of CRC screening tests were less likely to accept the invitation (OR: 0.49; 95% CI: 0.34-0.70). CONCLUSIONS: Adoption of health protective behaviors is associated with a higher attendance rate, whereas anxiety represents a strong barrier, even among people who deemed screening to be effective. Increasing the proportion of people who consult their GP when making a decision regarding screening might enhance participation. | Male | Humans | Patient Participation | Questionnaires | OK |
| Silvia | Minozzi |  |  |  |  | Preventive Medicine | 2012-08-00 Aug 2012 | BACKGROUND: Biennial screening with faecal occult blood tests (FOBts) has been found to reduce colorectal cancer mortality. Faecal immunochemical tests (FITs) are superior to guaiac faecal occult blood tests (G-FOBts) due to their improved sensitivity and specificity. However the effectiveness of a screening programme depends highly on participation rates. The aim of this study was to review studies comparing guaiac faecal occult blood tests and faecal immunochemical tests, in terms of participation rates. METHODS: We searched PubMed and the Cochrane Library (2000-September 2011) to identify randomised control trials comparing guaiac faecal occult blood test with faecal immunochemical test participation rates. One author screened the titles and abstracts, and performed data extraction which was then checked by the other authors. Risk of bias in the included studies was also assessed. RESULTS: Seven studies met the eligibility criteria and were entered into a meta-analysis. Participation rates were significantly higher for individuals offered faecal immunochemical tests compared to those offered a guaiac faecal occult blood test (RR 1.21; 95% CI 1.09-1.33). Potential factors that could have influenced participation were discussed. CONCLUSIONS: Colorectal cancer screening programmes currently using guaiac faecal occult blood tests could improve participation rates by converting to faecal immunochemical tests. More research examining the acceptability of faecal immunochemical tests, from a patient perspective, is warranted. | Humans | Patient Participation | Colorectal Neoplasms | Mass Screening | OK |
| Joann N. | Bodurtha | Donna | McClish |  |  | Journal of Women's Health (2002) | 2011-08-00 Aug 2011 | BACKGROUND: Studies show that daughters of mothers with breast cancer may be at increased risk for developing the disease. However, daughters' adherence to health behavior and screening recommendations, compared to the general population, is unknown. METHODS: Telephone interviews explored characteristics of adult daughters (n=147), including primary and secondary preventive behaviors, body mass index (BMI, kg/m(2)), physical activity, fruit/vegetable intake, alcohol intake, smoking, and mammography. Daughters of mothers with breast cancer were recruited from the community and were compared with Virginia women (n=2528) from the 2005 Behavioral Risk Factor Surveillance System (BRFSS) survey. Differences were examined using logistic regression, adjusting for demographic covariates. RESULTS: Daughters were younger (p<0.001), more highly educated (p<0.001), and more likely to never have been married (p<0.001) than BRFSS participants, but groups were similar by race. In adjusted analyses, daughters were significantly more likely to have ever had a mammogram (p<0.001) and to have had one recently (p=0.001). Daughters also were significantly less likely to consume>3 fruit/vegetable servings daily (p=0.032) compared to BRFSS results. There were no differences in BMI, smoking rates, alcohol consumption, or level of physical activity at work. CONCLUSIONS: Daughters with familial breast cancer risk were more likely to receive mammography screening than BRFSS participants, but they were no different in BMI, physical activity at work, exercise, or smoking than BRFSS participants and were less likely to consume more fruits and vegetables. More research is needed to explore group differences in screening practices compared to modifiable health behaviors in daughters of mothers with breast cancer. | Humans | Patient Participation | Female | Middle Aged | OK |
| Jörg | Dirmaier | Martin | Härter |  |  | Health Expectations: An International Journal of Public Participation in Health Care and Health Policy | 2012-11-26 Nov 26, 2012 | BACKGROUND: While there has been a clear move towards shared decision-making (SDM) in the last few years, the measurement of SDM-related constructs remains challenging. There has been a call for further psychometric testing of known scales, especially regarding validity aspects. OBJECTIVE: To test convergent validity of the nine-item Shared Decision-Making Questionnaire (SDM-Q-9) by comparing it to the OPTION Scale. DESIGN: Cross-sectional study. SETTING AND PARTICIPANTS: Data were collected in outpatient care practices. Patients suffering from chronic diseases and facing a medical decision were included in the study. METHODS: Consultations were evaluated using the OPTION Scale. Patients completed the SDM-Q-9 after the consultation. First, the internal consistency of both scales and the inter-rater reliability of the OPTION Scale were calculated. To analyse the convergent validity of the SDM-Q-9, correlation between the patient (SDM-Q-9) and expert ratings (OPTION Scale) was calculated. RESULTS: A total of 21 physicians provided analysable data of consultations with 63 patients. Analyses revealed good internal consistency of the SDM-Q-9 and limited internal consistency of the OPTION Scale. Inter-rater reliability of the latter was less than optimal. Association between the total scores of both instruments was weak with a Spearman correlation of r = 0.19 and did not reach statistical significance. DISCUSSION: By the use of the OPTION Scale convergent validity of the SDM-Q-9 could not be established. Several possible explanations for this result are discussed. CONCLUSION: This study shows that the measurement of SDM remains challenging. | | | | | OK |
| Claire | O'Regan | Yumiko | Kamiya | Brendan J. | Whelan | Age and Ageing | 2011-01-00 Jan 2011 | BACKGROUND: some cohort studies of ageing and health supplement questionnaire-based surveys with in-home measurements of biological parameters and others have required respondents to attend assessment centres. Centre-based assessments facilitate detailed measurements and novel technologies, but may differentially influence participation. The aim of this paper is to compare the characteristics of participants who attended a centre with those who chose a home assessment and those who did not have a health assessment. METHODS: trained field workers administered a computer-assisted personal interview (CAPI) to a random sample of community-dwelling people aged 50 and over in the participants' homes. All questionnaire respondents were invited to attend an assessment centre for a comprehensive physical assessment. Participants who refused or were unable to attend a centre were offered a home assessment. RESULTS: of the 291 participants who completed the CAPI, 176 had a health assessment: 138 in an assessment centre and 38 in their own home. The centre, home and no visit respondents differed in demographic characteristics, behavioural factors, physical functioning and health. Lower socio-economic status, physical inactivity and current smoking were the most robust predictors of non-participation in the health assessment. Home respondents had the highest levels of physical disability and were much weaker (grip strength) and slower (walking speed) than centre respondents. CONCLUSION: home and centre physical assessments are required to avoid systematically over-representing healthier and wealthier respondents. | Male | Humans | Patient Participation | Female | OK |
| Shay | Bess | Behrooz A. | Akbarnia | Oheneba | Boachie-Adjei | Spine | 2013-04-20 Apr 20, 2013 | STUDY DESIGN: Survey based on complication scenarios. OBJECTIVE: To assess and compare perceived potential impacts of various perioperative adverse events by both surgeons and patients. SUMMARY OF BACKGROUND DATA: Incidence of adverse events after adult spinal deformity surgery remains substantial. Patient-centered outcomes tools measuring the impact of these events have not been developed. An important first step is to assess the perceptions of surgeons and patients regarding the impact of these events on surgical outcome and quality of life. METHODS: Descriptions of 22 potential adverse events of surgery (heart attack, stroke, spinal cord injury, nerve root injury, cauda equina injury, blindness, dural tear, blood transfusion, deep vein thrombosis, pulmonary embolism, superficial infection, deep infection, lung failure, urinary tract infection, nonunion, adjacent segment disease, persistent deformity, implant failure, death, renal failure, gastrointestinal complications, and sexual dysfunction) were presented to 14 spinal surgeons and 16 adult patients with spinal deformity. Impact scores were assigned to each complication on the basis of perceptions of overall severity, satisfaction with surgery, and effect on quality of life. Impact scores were compared between surgeons and patients with a Wilcoxon/Kruskal-Wallis test. RESULTS: Mean impact scores varied from 0.9 (blood transfusion) to 10.0 (death) among surgeons and 2.3 (urinary tract infection) to 9.2 (stroke) among patients. Patients' scores were consistently higher (P < 0.05) than surgeons in all 3 categories for 6 potential adverse events: stroke, lung failure, heart attack, pulmonary embolism, dural tear, and blood transfusion. Three additional complications (renal failure, non-union, and deep vein thrombosis) were rated higher in 1 or 2 categories by patients. CONCLUSION: There was substantial variation in how both surgeons and patients perceived impacts of various adverse events after spine surgery. Patients generally perceived the impact of adverse events to be greater than surgeons. Patient-centered descriptions of adverse events would provide a more complete description of surgical outcomes. | Male | Humans | Patient Participation | Physician-Patient Relations | OK |
| Manori | Malawaraarachchi | Karin | Leder |  |  | BMC medical research methodology | 2012-00-00 2012 | BACKGROUND: Epidemiological research often requires collection of data from a representative sample of the community or recruitment of specific groups through broad community approaches. The population coverage of traditional survey methods such as mail-outs to residential addresses, and telephone contact via public directories or random-digit-dialing is declining and survey response rates are falling. There is a need to explore new sampling frames and consider multiple response modes including those offered by changes in telecommunications and internet technology. METHODS: We evaluated response rates and cost-effectiveness for three modes of survey administration (postal invitation/postal survey, postal invitation/internet survey and postal invitation/telephone survey) and two styles of contact approach (personalised and generic) in a community survey of greywater use. Potential respondents were contacted only once, with no follow up of non-responders. RESULTS: The telephone survey produced the highest adjusted response rate (30.2%), followed by the personalised postal survey (10.5%), generic postal survey (7.5%) and then the internet survey (4.7% for the personalised approach and 2.2% for the generic approach). There were some differences in household characteristics and greywater use rates between respondents to different survey modes, and between respondents to personalised and generic approaches. These may be attributable to the differing levels of motivations needed for a response, and varying levels of interest in the survey topic among greywater users and non-users. The generic postal survey had the lowest costs per valid survey received (Australian $22.93), followed by the personalised postal survey ($24.75). CONCLUSIONS: Our findings suggest that postal surveys currently remain the most economic option for population-based studies, with similar costs for personalised and generic approaches. Internet surveys may be effective for specialised groups where email lists are available for initial contact, but barriers other than household internet access still exist for community-based surveys. Given the increasing recruitment challenges facing community-based studies, there is an imperative to gather contemporary comparative data on different survey modes and recruitment approaches in order to determine their strengths, limitations and costs. Researchers also need to document and report on the potential biases in the target and respondent populations and how this may affect the data collected. | Humans | Patient Participation | Internet | Patient Selection | OK |
| John D. | Corrigan | Jin-Shei | Lai | Marcel P. | Dijkers | Archives of Physical Medicine and Rehabilitation | 2011-04-00 Apr 2011 | OBJECTIVE: To develop and compare 2 scoring algorithms for a measure of participation, the Participation Assessment with Recombined Tools-Objective (PART-O) based on the assumption that more participation is better versus an alternative that reflects balance in domains of participation. DESIGN: Survey. SETTING: Community settings. PARTICIPANTS: Three groups of participants under the age of 65 years were included: (1) persons with spinal cord injury, traumatic brain injury, stroke, and other disorders who are commonly treated in acute rehabilitation settings (n=220), and (2) participants from the general population who did (n=366) or (3) did not (n=284) self-report limitations indicative of a disability who participated in the 2006 Colorado Behavioral Risk Factor Surveillance System (N=870). INTERVENTIONS: Not applicable. MAIN OUTCOME MEASURE: PART-O. RESULTS: We developed PART-O subscores using a consensus process and then evaluated them empirically. We combined subscores using 2 contrasting algorithms, one using average scores and the other reflecting the amount of participation and variation in participation across 3 domains. The algorithms for the total scores yielded normal distributions in all 3 samples and were equally sensitive to the impact of disability. While strongly correlated, about 30% of the variance of the 2 total scores was not shared. CONCLUSIONS: Two scoring algorithms for the PART-O illustrate contrasting perspectives of the construct of participation. The 2 algorithms may be used in future studies to expand our understanding of the construct of participation. | Male | Humans | Patient Participation | Questionnaires | OK |
| G. | Wassmer | J. | Dumit | A. | Rolfs | International Journal of Dental Hygiene | 2012-11-00 Nov 2012 | OBJECTIVE: Adherence of young adults to preventive programmes is low. The following study compares three different educational concepts to increase toothbrushing adherence in young adults. METHODS: Nine vocational school classes (157 young adults) were randomly assigned to three different 60-min approaches: (I) Education by a dentist, (II) Peer-teaching and (III) 'Adherence triangle concept' uniting dentists, teachers and participants as equal partners in intervention planning combined with peer teaching. Follow-up was 1 week for approaches I and II, and 1 week, 3 and 9 months for approach III. Adherence was defined as reported change from the childhood toothbrushing technique to adult technique. Adherence was evaluated using anonymous questionnaires and by diary analysis. RESULTS: After instruction, 90% of participants (approaches I-III) showed the desired behaviour in practice and theory. Reported adherence after 1 week with approach I was 28.5%, with approach II 39% and with approach III 95%. Prolonged follow-up of approach III yielded 76% after 3 months and 68% after 9 months. Adherence using approach III was significantly higher (P ≤ 0.001) than using approach I and II after 1 week. Adherence rates with approach III after 9 months were still higher than those of approaches I and II after 1 week. CONCLUSIONS: The 'adherence triangle' concept enhanced reported adherence significantly in comparison with previous studies and the one-dimensional concepts of approaches I and II. The tools of the adherence triangle concept seem worthwhile to be considered when planning new preventive action. | Male | Humans | Patient Participation | Young Adult | OK |
| Michael S. | Yi | Joel | Tsevat |  |  | The Journal of Asthma: Official Journal of the Association for the Care of Asthma | 2011-06-00 Jun 2011 | BACKGROUND/PURPOSE: Up to 80% of adolescents with asthma have used complementary and alternative medicine (CAM) for symptom management. However, little is known about patient characteristics associated with CAM factors other than use. Previous studies recommend provider-patient discussion of CAM use, although few adolescents with asthma disclose their CAM use to their providers. To inform clinical interactions, this study examined prevalence and predictors of CAM use, consideration of use, disclosure of use, and perceived efficacy of use, in urban adolescents with asthma. METHODS: Adolescents with asthma (N = 151) recruited from a children's hospital completed questionnaires addressing demographic and clinical variables and 10 CAM modalities. Response frequencies to four questions assessing CAM use, consideration of use, disclosure, and perceived efficacy were calculated for each modality. Multivariable logistic regression analyses examined characteristics associated with responses to each question for the two most commonly used CAM modalities. RESULTS: Participants' mean age was 15.8 (SD = 1.8), 60% were female and 85% were African-American. Seventy-one percent reported using CAM for symptom management in the past month. Relaxation (64%) and prayer (61%) were the most frequently reported modalities and were perceived to be the most efficacious. Adolescents most commonly reported considering using relaxation (85%) and prayer (80%) for future symptom management. Participants were most likely to disclose their use of yoga (59%) and diet (57%), and least likely to disclose prayer (33%) and guided imagery (36%) to providers. In multivariable analyses, older adolescents (OR = 1.27, p < .05) and African-Americans (OR = 2.76, p < .05) were more likely to use relaxation. Adolescents with more frequent asthma symptoms (OR = 0.98, p < .05) were more likely to use prayer. African-Americans were more likely to report using prayer (OR = 3.47, p < .05) and consider using prayer (OR = 7.98, p < .01) in the future for symptom management. CONCLUSIONS: Many urban adolescents used and would consider using CAM, specifically relaxation and prayer, for asthma symptom management. African-Americans, older adolescents, and those with more frequent symptoms were more likely to use and/or consider using CAM. Providers caring for urban adolescents with asthma should discuss CAM with patients, particularly those identified as likely to use CAM. Future studies should examine relationships between CAM use and health outcomes. | Male | Humans | Patient Participation | Questionnaires | OK |
| Christoph | Heesen | Sascha | Köpke | Friedemann | Geiger | Zeitschrift Für Evidenz, Fortbildung Und Qualität Im Gesundheitswesen | 2012-00-00 2012 | OBJECTIVE: Shared decision making is based on the idea of cooperation and partnership between patients and doctors. In this concept both parties may initiate and perform specific decision-making steps. However, the common observation-based instruments focus solely on doctors' behaviour. Content and quality of information provided to involve patients in medical decisions are hardly considered in evaluation of SDM. This study investigates the advantages of a revised observer inventory taking into account these aspects. METHODS: Based on the OPTION scale, a more comprehensive observation-based inventory was developed, additionally considering both the patient-sided indicators for patient involvement and the criteria of evidence-based patient information. The inventory comprises three scales (doctor, patient, doctor-patient dyad) and 15 indicators each. Rater training and re-analyses of 76 consultations previously analysed using the OPTION scale were conducted. Convergent validities were calculated between the observer-based scales and the patients' ratings on the Shared Decision Making Questionnaire, the Decisional Conflict Scale and the Control Preference Scale. RESULTS: Interrater reliabilities of the revised scales were high (r=.87 to .74) and even higher when only the dyadic perspective was coded (.86). The revised inventory provided additional information on the involvement taking place. No substantive correlations were found between observation-based and patients' subjective judgments. CONCLUSION: The observers' perspective on patient involvement needs to consider patient activities. Inconsistencies of patients' and observers' judgements concerning patient participation need further investigation. | Decision Making | Humans | Patient Participation | Physician-Patient Relations | OK |
| Jutta | Bleidorn | Eva | Hummers-Pradier | Marie-Luise | Dierks | Patient Education and Counseling | 2013-01-00 Jan 2013 | OBJECTIVE: To examine to what extent general practitioners in consultations after a geriatric assessment set shared health priorities with older patients experiencing multimorbidity and to what extent this was facilitated through patient-centered behavior. METHODS: Observation of consultations embedded in a cluster randomized controlled trial,(1) in which 317 patients from 41 general practices received the STEP assessment followed by a care planning consultation with their GPs. GPs in the intervention group used a structured procedure for setting health (care) priorities in contrast to control GPs. A sample of 43 consultations (24 intervention; 19 control) were recorded, transcribed and analyzed with regard to priority setting and patient-centeredness. RESULTS: Patient-centeredness was only moderately apparent in consultations dealing with complex care plans for older patients with multimorbidity. The shared determination of health priorities seemed unusual for both doctors and patients and was rarely practiced, albeit more frequently in intervention consultations. CONCLUSION: Setting health care priorities with patients experiencing multimorbidity is ethically desirable and medically appropriate. Yet a short structured guide for doctors cannot easily achieve this. PRACTICE IMPLICATIONS: More research is needed in regard to handling complex health needs of older patients. It requires a professional approach and training in patient-centered holistic care planning. | Male | Decision Making | Humans | Patient Participation | OK |
|  |  |  |  |  |  | Medical Decision Making: An International Journal of the Society for Medical Decision Making | 2010-10-00 2010 Sep-Oct | | Humans | Patient Participation | Questionnaires | Choice Behavior | OK |
| Raquel | Gabriel | Daniela | Figueiredo |  |  | Disability and Rehabilitation | 2013-09-00 Sep 2013 | PURPOSE: This study aimed to validate the Activities and Participation component of the Comprehensive International Classification of Functioning, Disability and Health (ICF) Core Set for Obstructive Pulmonary Diseases (OPD) from the patient's perspective. METHODS: A cross-sectional qualitative study was conducted with a convenience sample of outpatients with Chronic Obstructive Pulmonary Disease (COPD). Individual interviews were performed and analysed according to the meaning condensation procedure. RESULTS: Fifty-one participants (70.6% male) with a mean age of 69.5 ± 10.8 years old were included. Twenty-one of the 24 categories contained in the Activities and Participation component of the Comprehensive ICF Core Set for OPD were identified by the participants. Additionally, seven second-level categories that are not covered by the Core Set were reported: complex interpersonal interactions, informal social relationships, family relationships, conversation, maintaining a body position, eating and preparing meals. CONCLUSIONS: The activities and participation component of the ICF Core Set for OPD was largely supported by the patient's perspective. The categories included in the ICF Core Set that were not confirmed by the participants and the additional categories that were raised need to be further investigated in order to develop an instrument according to the patient's perspective. This will promote a more patient-centred assessments and rehabilitation interventions. Implications for Rehabilitation The Activities and Participation component of the Comprehensive ICF Core Set for OPD is largely supported by the perspective of patients with COPD and therefore could be used in the assessment of patients' individual and social life. The information collected through the Activities and Participation component of the Comprehensive ICF Core Set for OPD could be used to plan and assess rehabilitation interventions for patients with COPD. | Male | Humans | Patient Participation | Female | OK |
| Robert D. | Annett | David G. | Scherer | Charles W. | Turner | Journal of Health Psychology | 2012-07-00 Jul 2012 | 'Research literacy' is proposed as a key concept for advancing societal health. To examine whether improvements in research literacy would affect knowledge of and ethical participation in research, parents of young children received a brief educational intervention designed to enhance their understanding of child research. Results demonstrated that the intervention improved research-related knowledge and increased parents' comfort with their research participation decisions. Moreover, enhanced understanding of child volition increased parents' willingness to enrol their children in research. The proposed research literacy model identifies methods to enhance population knowledge and appreciation of research, strengthening links between scientific advancement and health. | Male | Decision Making | Humans | Patient Participation | OK |
| Ivan | Castellvi | Hector | Corominas | William J. | Taylor | Journal of Clinical Rheumatology: Practical Reports on Rheumatic & Musculoskeletal Diseases | 2014-04-00 Apr 2014 | BACKGROUND: Appropriate evaluation of disease or treatment outcomes requires a good understanding of the aspects of the disease and its consequences that actually matter to patients. The OMERACT process has identified several domains of outcome measurement for chronic gout, but patient involvement has only been briefly described. OBJECTIVES: To make the results of patient involvement more explicit, the current study reports on an exercise carried out in the early part of the OMERACT domain identification process. METHODS: Patients with gout were asked to identify areas of life affected by gout. These areas were rated (0-10) and ranked for importance by a second group of patients with gout recruited from both primary care and a rheumatology clinic. RESULTS: Thirty-one patients with gout performed the initial generation of areas of importance. These areas were combined to form 14 items, which were ranked and scored by 107 patients. Four areas were ranked first to third most important by at least 20% of participants. These were pain (median rating, 10), loss of joint motion (median rating, 9), work loss (median rating, 7), and joint inflammation/swelling (median rating, 8). Three additional areas were ranked highly by at least 10% of patients: difficulty with wearing shoes, having to undertake a restrictive diet, and sleep. CONCLUSIONS: These data largely confirmed the relevance of the gout OMERACT domains while identifying additional issues for patients that require further investigation. | Male | Humans | Patient Participation | Female | OK |
| Régis | Lassalle | Fanny | Depont | Philip | Robinson | Pharmacoepidemiology and Drug Safety | 2010-05-00 May 2010 | PURPOSE: Various data sources may be used in pharmacoepidemiological studies. When they cannot be obtained from valid databases, medical data must be obtained from physicians or patients. In the CADEUS study, both patients and their prescribers reported medical data allowing investigation of the concordance between these sources. METHODS: CADEUS is a French national cohort study of traditional non-steroidal anti-inflammatory drug (NSAID) and coxib users conducted between September 2003 and August 2004 in France that employed self-administered questionnaires to obtain medical data from patients and their prescribers. The Kappa statistic (kappa) was used to measure concordance between patients and prescribers in 18 530 pairs with regard to previous medical history and index NSAID indication. RESULTS: For previous medical history, the proportion of agreement ranged from 70.7 to 99.2% and concordance was: substantial (kappa = 0.61-0.80) for hypertension, myocardial infarction, stroke and diabetes; moderate (kappa = 0.41-0.60) for angina pectoris, peripheral arterial disease and hypercholesterolaemia; fair (kappa = 0.21-0.40) for unstable angina, cardiac insufficiency, dyspepsia, gastroesophageal reflux and gastric ulcer; slight (kappa < 0.21) for upper gastrointestinal haemorrhage. For index NSAID indication, the proportion of agreement ranged from 84.3 to 99.4% and concordance was almost perfect (kappa = 0.81-1.00) for inflammatory rheumatism, flu-like symptoms, dysmenorrhoea and dental pain; substantial for arthritis, back pain and headache; moderate for osteoarticular pain. CONCLUSIONS: Concordance was better for specific or serious conditions both regarding previous medical history and indication. Prescriber or patient perception and understanding may reduce concordance for certain items. | Male | Humans | Patient Participation | Questionnaires | OK |
| Andreas | Loh | Claudia | Spies | Fueloep | Scheibler | Health Expectations: An International Journal of Public Participation in Health Care and Health Policy | 2010-09-00 Sep 2010 | OBJECTIVE: Validation of the German version of the Autonomy-Preference-Index (API), a measure of patients' preferences for decision making and information seeking. METHODS: Stepwise confirmatory factor analysis was conducted on a sample of patients (n = 1592) treated in primary care for depression (n = 186), surgical and internal medicine inpatients (n = 811) and patients with minor trauma treated in an emergency department (n = 595). An initial test of the model was done on calculation and validation halves of the sample. Both local and global indexes-of-fit suggested modifications to the scale. The scale was modified and re-tested in the calculation sample and confirmed in the validation sample. Subgroup analyses for age, gender and type of treatment setting were also performed. RESULTS: The confirmatory analysis led to a modified version of the API with better local and global indexes-of-fit for samples of German-speaking patients. Two items of the sub-scale, 'preference for decision-making', and one item of the sub-scale, 'preference for information seeking', showed very low reliability scores and were deleted. Thus, several global indexes-of-fit clearly improved significantly. The modified scale was confirmed on the validation sample with acceptable to good indices of fit. Results of subgroup analyses indicated that no adaptations were necessary. DISCUSSION AND CONCLUSIONS: This first confirmatory analysis for a German-speaking population showed that the API was improved by the removal of several items. There were theoretically plausible explanations for this improvement suggesting that the modifications might also be appropriate in English and other language versions. | Male | Decision Making | Humans | Patient Participation | OK |
| Philip | Cotton |  |  |  |  | Education for primary care: an official publication of the Association of Course Organisers, National Association of GP Tutors, World Organisation of Family Doctors | 2012-11-00 Nov 2012 | | Humans | Patient Participation | Patient Satisfaction | Health Personnel | OK |
| K. | Smyth |  |  |  |  | Eye (London, England) | 2012-05-00 May 2012 | AIMS: To evaluate current trainers' attitudes and practices for informing patients about the trainee participation in cataract surgery within the United Kingdom. METHODS: An anonymous online survey was distributed to current cataract surgery trainers via all Royal College of Ophthalmologists' tutors within the United Kingdom. Trainers were asked specific questions about their current consent practice regarding trainee participation in the cataract surgery. Questions also targeted experiences of patient complaints about training. RESULTS: One hundred and twenty-three trainers completed the survey. Ninety-three percent (n=114) of responders were consultants and 7% (n=8) were non-consultant career-grade doctors or other grades. A total of 34% (n=42) of responders stated that consent was usually taken by themselves or the trainee assigned to the list, whereas 26% (n=32) always took consent themselves. Sixty percent of responders (n=74) stated that consent is taken on the day of surgery; 59% (n=73) indicated consent is taken where listing takes place. Thirty-three percent (n=41) of trainers indicated that they had experienced patient dissatisfaction or complaints. Surgical complications, length of surgery, and discussions during surgery were the leading causes of complaints. Thirty-nine percent (n=48) would operate themselves if patients requested no trainee participation. CONCLUSIONS: There is a wide variety in the current practice of disclosure and level of information given regarding trainee participation in surgery. This will influence patients' expectations, experiences, and satisfaction. | Humans | Patient Participation | Patient Satisfaction | Questionnaires | OK |
| J. C. | Carroll | B. | Wilson | J. | Little | Public Health Genomics | 2010-00-00 2010 | BACKGROUND: As newborn screening (NBS) expands to meet a broader definition of benefit, the scope of parental consent warrants reconsideration. METHODS: We conducted a mixed methods study of health care provider attitudes toward consent for NBS, including a survey (n = 1,615) and semi-structured interviews (n = 36). RESULTS: Consent practices and attitudes varied by provider but the majority supported mandatory screening (63.4%) and only 36.6% supported some form of parental discretion. Few health care providers (18.6%) supported seeking explicit consent for screening condition-by-condition, but a larger minority (39.6%) supported seeking consent for the disclosure of incidentally generated sickle cell carrier results. Qualitative findings illuminate these preferences: respondents who favored consent emphasized its ease while dissenters saw consent as highly complex. CONCLUSION: Few providers supported explicit consent for NBS. Further, those who supported consent viewed it as a simple process. Arguably, these attitudes reflect the public health emergency NBS once was, rather than the public health service it has become. The complexity of NBS panels may have to be aligned with providers' capacity to implement screening appropriately, or providers will need sufficient resources to engage in a more nuanced approach to consent for expanded NBS. | Humans | Patient Participation | Questionnaires | Internet | OK |
| Sara | O'Donnell | Dawn | Elsbree | Joy | Hardin | Patient Education and Counseling | 2010-07-00 Jul 2010 | OBJECTIVE: This study asked: (1) What do rural women with breast cancer need to make the most out of their major medical appointments? and (2) What can the community resource centers do to best support those needs? METHODS: We interviewed 12 doctors, 12 breast cancer survivors and 10 community agency staff, including those who provide services to Latinos and Native Americans. Interviews generated success factors and barriers related to meeting patient information needs. Examples were categorized into themes. RESULTS: Success factors included making sure patients review high quality educational materials before the visit; and that someone is available to take notes. Doctors felt that a patient list of questions was productive, but some survivors felt doctors did not always respond productively to the list. Respondents did not mention audiorecording unless prompted. Most then endorsed it. CONCLUSION: Educational materials, question lists, and note-takers can help rural women with breast cancer and their doctors achieve their goals during treatment discussions. Audiorecording may be an implicit but not explicit need. PRACTICE IMPLICATIONS: Other cancer resource centers and support agencies should consider offering information, question-listing, and note-taking services. They should assess whether audiorecording is an implicit need in their settings. | Male | Humans | Patient Participation | Physician-Patient Relations | OK |
| Nicola | Carey |  |  |  |  | International Journal of Nursing Studies | 2011-01-00 Jan 2011 | BACKGROUND: There is a drive to improve the quality of service provision for patients with diabetes and to enable better self-management of this condition. The adoption of prescribing by nurses is increasing worldwide and can potentially enhance service provision. Evidence suggests that patients prefer services where their lifestyle factors and opinions are considered by healthcare professionals within a partnership approach. Few studies have explored patients' views about their consultations with a nurse prescriber. OBJECTIVE: To explore the views patients with diabetes have about their consultations with nurse prescribers and any impact this may have on their medications management. DESIGN: A qualitative study involving semi-structured interviews and thematic analysis. SETTING: Six primary care sites in which nurses prescribed medications for patients with diabetes in England. Data was collected in 2009. PARTICIPANTS: Interviews took place with 41 patients with diabetes from the case loads of 7 nurse prescribers. RESULTS: Findings are reported under three themes; the nurse consultation style, benefits of the nurse prescriber consultation and views on involvement and decision-making. Key aspects of the nurse consultation style were a non-hurried approach, care and rapport, approachability, continuity, and providing clear information based on specialist knowledge. Many benefits were described, including improved access to appropriate advice and medication, greater understanding and ability to self-manage, ability to address problems and improved confidence, trust and wellbeing. While patients were happy with the amount of information received and involvement they had decisions about their treatment, there was some controversy over the consistency of information provided on side-effects of treatment. CONCLUSIONS: The study provides new knowledge about what patients with diabetes value and benefit from in respect to care provided by nurse prescribers. Continuity of relationship, flexibility over consultation length, nurses' interpersonal skills and specialist diabetes knowledge were identified as crucial to good quality care. Patients require that nurse prescribers are skilled in providing a person-centred approach and have access to specialist training. The level of information and involvement offered to patients should reflect patients' requirements. | Male | Humans | Patient Participation | Questionnaires | OK |
| Jed | Hand | Gerald E. | Denehy | Deborah S. | Cobb | Journal of Dental Education | 2012-06-00 Jun 2012 | This study aimed to better understand how and why people choose dental school faculty members as their oral health care providers. Increasing financial constraints in U.S. dental schools have led their administrators to seek alternative funding sources, one of which can be revenues from dental school faculty practice. To effectively promote faculty practice, it is necessary to understand how and why one chooses a dental school faculty member as his or her oral health care provider. A survey of 1,150 dental school faculty practice patients who recently chose their dentist was conducted, and 221 responded. The information sources these respondents said they used and rated highly were other dentists, friends, family members, clinic website, the Internet, and the insurance directory. Dentist-related attributes that were perceived to be important were quality of care, professional competence of dentist, and explanation of treatment/patient participation in the treatment decision. Dental practice-related attributes perceived to be important were the ability to get appointments at convenient times, reasonable waiting time to get appointments, and attitude/helpfulness of staff. This study found that traditionally popular (family, friends) and newly emerging information sources (the Internet, clinic website, and insurance directory) were both used and perceived to be important by patients of the dental school faculty practice. Dental schools and dentists can use this study's findings to select appropriate communication channels to promote their practices and to focus on attributes that dental consumers value the most. | Male | Decision Making | Humans | Questionnaires | OK |
|  |  |  |  |  |  | Social Work in Health Care | 2014-00-00 2014 | The escalating complexity in health-related decisions that people face have important implications for social work interventions. This article explores the nature of these implications within the context of decisional conflict, shared decision making, and the use of decision aids. In addition, the authors present the findings of a content analysis of 29 contemporary health-related decision aids. Emergent categories from this analysis are presented as a resource for social workers as they encounter, adapt, and create decision aids in their work to help address the health-related needs of their clients. | | | | | OK |
| Frank Y. | Chang | Anisha R. | Illa | Angela | Benoit | Journal of the American Medical Informatics Association: JAMIA | 2014-06-00 2014 May-Jun | BACKGROUND: Maintaining continuity of care (CoC) in the inpatient setting is dependent on aligning goals and tasks with the plan of care (POC) during multidisciplinary rounds (MDRs). A number of locally developed rounding tools exist, yet there is a lack of standard content and functional specifications for electronic tools to support MDRs within and across settings. OBJECTIVE: To identify content and functional requirements for an MDR tool to support CoC. MATERIALS AND METHODS: We collected discrete clinical data elements (CDEs) discussed during rounds for 128 acute and critical care patients. To capture CDEs, we developed and validated an iPad-based observational tool based on informatics CoC standards. We observed 19 days of rounds and conducted eight group and individual interviews. Descriptive and bivariate statistics and network visualization were conducted to understand associations between CDEs discussed during rounds with a particular focus on the POC. Qualitative data were thematically analyzed. All analyses were triangulated. RESULTS: We identified the need for universal and configurable MDR tool views across settings and users and the provision of messaging capability. Eleven empirically derived universal CDEs were identified, including four POC CDEs: problems, plan, goals, and short-term concerns. Configurable POC CDEs were: rationale, tasks/'to dos', pending results and procedures, discharge planning, patient preferences, need for urgent review, prognosis, and advice/guidance. DISCUSSION: Some requirements differed between settings; yet, there was overlap between POC CDEs. CONCLUSIONS: We recommend an initial list of 11 universal CDEs for continuity in MDRs across settings and 27 CDEs that can be configured to meet setting-specific needs. | Humans | Patient Participation | intensive care units | Data Collection | Doublon |
| Frank Y. | Chang | Anisha R. | Illa | Angela | Benoit | Journal of the American Medical Informatics Association: JAMIA | 2014-06-00 2014 May-Jun | BACKGROUND: Maintaining continuity of care (CoC) in the inpatient setting is dependent on aligning goals and tasks with the plan of care (POC) during multidisciplinary rounds (MDRs). A number of locally developed rounding tools exist, yet there is a lack of standard content and functional specifications for electronic tools to support MDRs within and across settings. OBJECTIVE: To identify content and functional requirements for an MDR tool to support CoC. MATERIALS AND METHODS: We collected discrete clinical data elements (CDEs) discussed during rounds for 128 acute and critical care patients. To capture CDEs, we developed and validated an iPad-based observational tool based on informatics CoC standards. We observed 19 days of rounds and conducted eight group and individual interviews. Descriptive and bivariate statistics and network visualization were conducted to understand associations between CDEs discussed during rounds with a particular focus on the POC. Qualitative data were thematically analyzed. All analyses were triangulated. RESULTS: We identified the need for universal and configurable MDR tool views across settings and users and the provision of messaging capability. Eleven empirically derived universal CDEs were identified, including four POC CDEs: problems, plan, goals, and short-term concerns. Configurable POC CDEs were: rationale, tasks/'to dos', pending results and procedures, discharge planning, patient preferences, need for urgent review, prognosis, and advice/guidance. DISCUSSION: Some requirements differed between settings; yet, there was overlap between POC CDEs. CONCLUSIONS: We recommend an initial list of 11 universal CDEs for continuity in MDRs across settings and 27 CDEs that can be configured to meet setting-specific needs. | Humans | Patient Participation | intensive care units | Data Collection | OK |
| Jade | Brelsford | Neil | Parsons | Heather | Skirton | BMC medical informatics and decision making | 2012-00-00 2012 | BACKGROUND: Better use of e-health services by patients could improve outcomes and reduce costs but there are concerns about inequalities of access. Previous research in outpatients suggested that anonymous personal email support may help patients with long term conditions to use e-health, but recruiting earlier in their 'journey' may benefit patients more. This pilot study explored the feasibility and cost of recruiting patients for an e-health intervention in one primary care trust. METHODS: The sample comprised 46 practices with total patient population of 250,000. We approached all practices using various methods, seeking collaboration to recruit patients via methods agreed with each practice. A detailed research diary was kept of time spent recruiting practices and patients. Researcher time was used to estimate costs. Patients who consented to participate were offered email support for their use of the Internet for health. RESULTS: Eighteen practices agreed to take part; we recruited 27 patients, most (23/27) from five practices. Practices agreed to recruit patients for an e-health intervention via waiting room leaflets (16), posters (16), practice nurses (15), doctors giving patients leaflets (5), a study website link (7), inclusion in planned mailshots (2), and a special mailshot to patients selected from practice computers (1). After low recruitment response we also recruited directly in five practices through research assistants giving leaflets to patients in waiting rooms. Ten practices recruited no patients. Those practices that were more difficult to recruit were less likely to recruit patients. Leaving leaflets for practice staff to distribute and placing posters in the practice were not effective in recruiting patients. Leaflets handed out by practice nurses and website links were more successful. The practice with lowest costs per patient recruited (£70) used a special mailshot to selected patients. CONCLUSION: Recruitment via general practice was not successful and was therefore expensive. Direct to consumer methods and recruitment of patients in outpatients to offer email support may be more cost effective. If recruitment in general practice is required, contacting practices by letter and email, not following up non-responding practices, and recruiting patients with selected conditions by special mailshot may be the most cost-effective approach. | Time Factors | Humans | Patient Participation | Questionnaires | OK |
| B. | Orgaz | M. A. | Verdugo |  |  | Child: Care, Health and Development | 2014-03-00 Mar 2014 | BACKGROUND: Despite growing interest in the topic of participation, the construct has not yet been assessed in children and adolescents with and without cerebral palsy (CP) in Spain. As there are no available instruments to measure participation in leisure activities which have been adapted in this country, the goal of this study was to validate a Spanish version of the Children's Assessment of Participation and Enjoyment (CAPE). METHOD: The sample comprised 199 children and adolescents with CP and 199 without CP, between 8 and 18 years of age, from seven regions in Spain. The adaptation of the original version of CAPE was carried out through translation and backward translation, and the validity of the instrument was analysed. Construct validity was assessed through the correlation of the diverse CAPE domains and the quality of life domains (KIDSCREEN questionnaire). Discriminant validity was established by comparing children and adolescents with CP and typically developing children and adolescents. For test-retest reliability, the children and adolescents with and without CP completed the CAPE questionnaire twice within 4 weeks. RESULTS: The correlations found between the CAPE domains and the quality of life domains show that the CAPE presents construct validity. The CAPE discriminated children and adolescents with CP from those without any disability in the results of participation. According to most CAPE domains, typically developing children and adolescents engage in a greater number of activities than children and adolescents with CP. Test-retest reliability for the Spanish version of CAPE was adequate. CONCLUSION: The study provides a valid instrument to assess the participation of children and adolescents with and without CP who live in Spain. | Male | Humans | Patient Participation | Questionnaires | OK |
|  |  |  |  |  |  | Journal of Hand Therapy: Official Journal of the American Society of Hand Therapists | 2014-03-00 2014 Jan-Mar | STUDY DESIGN: Cross-sectional descriptive survey INTRODUCTION: Best practice for conservative clinical care pathways is not well outlined in the literature for patients with thumb carpometacarpal joint (CMCJ) pain. This self-report survey investigated the current practice patterns of assessments and conservative interventions for the painful thumb CMCJ among hand therapists. METHODS: An online survey was distributed to members of the American Society of Hand Therapists (ASHT). Questions were included about evaluation measures and intervention techniques used for this population. A descriptive analysis was completed of the results. RESULTS: A total of 23.5% of the ASHT membership responded to the survey. Results were categorized using the International Classification of Functioning and Disability domains as a framework. The survey results report varying use of evaluation measures, therapeutic interventions, including orthotic fabrication, joint protection and patient education all therapeutic interventional techniques, and modalities. CONCLUSION: Therapists use a comprehensive array of evaluation measures and interventions for body functions and structures in the care of thumb CMC pain. In contrast, more consistent use is needed of psychometrically-sound functional outcome measures that show change in activities and participation. This survey highlights areas to employ current evidence, as well as, future research should address environmental factors and personal factors for this population LEVEL OF EVIDENCE: Not applicable. | Male | Humans | Patient Participation | Questionnaires | OK |
| Hoo-Yeon | Lee | Yeonju | Kim | Jae Kwan | Jun | Asian Pacific journal of cancer prevention: APJCP | 2011-00-00 2011 | OBJECTIVE: Since 2000, thyroid cancer has been increasing most rapidly in Korea. Although the cause of the increase is not clear, thyroid cancer screening could be identified as one of its causes. The purpose of this study was to examine the screening rate of thyroid cancer and its related factors using nationwide data. METHODS: The study population was derived from the 2009 Korea National Cancer Screening Survey (KNCSS), an annual cross-sectional survey that uses a nationally representative random sampling to investigate cancer screening rates. A total of 2,000 Korean adults participated. The screening method of thyroid cancer was restricted to thyroid ultrasonography. Logistic regression was used to identify factors associated with undergoing thyroid cancer screening. RESULTS: Of all participants, 13.2% (8.4% men and 16.4% women) underwent thyroid ultrasonography. On multiple analyses, age, residence, belief in cancer screening, regular health check-ups, smoking, alcohol drinking, and exercise were associated with thyroid cancer screening. Subjects who underwent other cancer screening, such as gastric, colorectal, breast, or cervical, were more likely to have had a thyroid ultrasonogram that those who did not get screened. CONCLUSIONS: We presented the number and characteristics of examinees utilized ultrasonography as a thyroid screening tool in Korea. Although these results revealed that cancer screening might play a major role in the increase of thyroid cancer incidence, further research is needed to determine causes of the rapidly increasing incidence of thyroid cancer in Korea. | Male | Humans | Patient Participation | Female | OK |
| J. | Kilsmark | H. | Voss | J. | Søgaard | Scandinavian Journal of Public Health | 2012-03-00 Mar 2012 | AIM: This study investigated the proportion of Danish patients in cardiac therapy for ischaemic heart disease who participated in cardiac rehabilitation. The study examined differences in patients' participation in and experience and satisfaction with cardiac rehabilitation. METHODS: Data were obtained from a postal questionnaire in a representative sample of patients in therapy for an ischaemic heart disease. Response data were tabulated and analysed by logistic regression. RESULTS: 3% of the patients participated in a complete rehabilitation programme according to Danish standards, 47% of the patients participated in a partial rehabilitation programme, and additionally 32% of the patients participated in a very limited rehabilitation programme. The number of patients participating in a complete rehabilitation programme was low due to the fact that only few patients (and their relatives) received psychological support. The factors living alone and low education are associated with low participation and in particular with receiving psychological support. Elderly (≥50 years) and male patients showed higher satisfaction scores. Patients receiving psychosocial elements in their rehabilitation programme reported higher satisfaction with their rehabilitation. CONCLUSIONS: Few Danish patients with ischaemic heart disease participate in a complete rehabilitation programme mainly due to psychosocial elements not yet being an integral part of cardiac rehabilitation in Denmark. There is a need to differentiate and tailor cardiac rehabilitation to different patient segments, e.g. by gender and age. | Male | Humans | Patient Participation | Patient Satisfaction | OK |
| Huib A. M. | Kerstjens | Steven M. | Uil | Judith M. | Vonk | International Journal of Chronic Obstructive Pulmonary Disease | 2013-00-00 2013 | BACKGROUND: Exacerbations of chronic obstructive pulmonary disease (COPD) are a major burden to patients and to society. Little is known about the possible role of day-to-day patient-reported outcomes during an exacerbation. This study aims to describe the day-to-day course of patient-reported health status during exacerbations of COPD and to assess its value in predicting clinical outcomes. METHODS: Data from two randomized controlled COPD exacerbation trials (n = 210 and n = 45 patients) were used to describe both the feasibility of daily collection of and the day-to-day course of patient-reported outcomes during outpatient treatment or admission to hospital. In addition to clinical parameters, the BORG dyspnea score, the Clinical COPD Questionnaire (CCQ), and the St George's Respiratory Questionnaire were used in Cox regression models to predict treatment failure, time to next exacerbation, and mortality in the hospital study. RESULTS: All patient-reported outcomes showed a distinct pattern of improvement. In the multivariate models, absence of improvement in CCQ symptom score and impaired lung function were independent predictors of treatment failure. Health status and gender predicted time to next exacerbation. Five-year mortality was predicted by age, forced expiratory flow in one second % predicted, smoking status, and CCQ score. In outpatient management of exacerbations, health status was found to be less impaired than in hospitalized patients, while the rate and pattern of recovery was remarkably similar. CONCLUSION: Daily health status measurements were found to predict treatment failure, which could help decision-making for patients hospitalized due to an exacerbation of COPD. | Male | Humans | Patient Participation | Female | OK |
|  | EHRA Scientific Initiatives Committee | | |  |  | Europace: European Pacing, Arrhythmias, and Cardiac Electrophysiology: Journal of the Working Groups on Cardiac Pacing, Arrhythmias, and Cardiac Cellular Electrophysiology of the European Society of Cardiology | 2010-08-00 Aug 2010 | This survey assesses the current opinion on and practice of the management of terminally ill patients with implanted cardioverter-defibrillators (ICDs) in 47 large European centres. The principal findings of this survey were that most physicians (62%) from European centres who responded to this survey would consider deactivating ICDs at the patient's end of life. In these circumstances, multiple appropriate ICD shocks may be an indication to deactivate an ICD (83% positive answers). Remote deactivation by a remote monitoring system is not considered appropriate by 68%. Practices of deactivating procedure differ and approach to standardized clinical scenarios is inhomogeneous. Patients are provided with surprisingly little information on the possibility of deactivation of ICDs since this subject is only actively discussed in 4% of centres. | Male | Humans | Patient Participation | Questionnaires | OK |
|  |  |  |  |  |  | Journal of Gerontological Social Work | 2011-04-00 Apr 2011 | Increasing use in the past decade has created pressure for hospital emergency rooms (ERs). Healthcare provided through an ER is expensive and is not designed to meet the complex needs of an older, chronically-ill population. ER visits are presented as the outcome of a decision-making process. Thirty veterans who had visited the ER in the previous year were asked about their decisions to use the ER. Their responses reflected four distinct approaches to ER use, which are characterized by frequency (frequent/infrequent) and risk for social isolation (low/high). Appropriate interventions by social work personnel might reduce inappropriate use of the ER and enhance the care of this vulnerable population. | Male | Humans | Patient Participation | Female | OK |
| Geoffrey | Dervin | Peter | Tugwell | Laura | Boland | BMC musculoskeletal disorders | 2014-00-00 2014 | BACKGROUND: To evaluate feasibility and potential effectiveness of a patient decision aid (PtDA) for patients and a preference report for surgeons to reduce wait times and improve decision quality in patients with osteoarthritis considering total knee replacement. METHODS: A prospective two-arm pilot randomized controlled trial. Patients with osteoarthritis were eligible if they understood English and were referred for surgical consultation about an initial total knee arthroplasty at a Canadian orthopaedic joint assessment clinic. Patients were randomized to the PtDA intervention or usual education. The intervention was an osteoarthritis PtDA for patients and a one-page preference report summarizing patients' clinical and decisional data for their surgeon. The main feasibility outcomes were rates of recruitment and questionnaire completion; the preliminary effectiveness outcomes were wait times and decision quality. RESULTS: Of 180 patients eligible for surgical consultation, 142 (79%) were recruited and randomized to the PtDA intervention (n = 71) or usual education (n = 71). Data collection yielded a 93% questionnaire completion rate with less than 1% missing items. After one year, 13% of patients remained on the surgical wait list. The median time from referral to being off the wait list (censored using survival analysis techniques) was 33.4 weeks for the PtDA group (n = 69, 95% CI: 26.0, 41.4) and 33.0 weeks for usual education (n = 71, 95% CI: 26.1, 39.9). Patients exposed to the PtDA had higher decision quality based on knowledge (71% versus 47%; p < 0.0001) and quality decision being an informed choice that is consistent with their values for option outcomes (56.4% versus 25.0%; p < 0.001). CONCLUSIONS: Recruitment of patients with osteoarthritis considering surgery and data collection were feasible. As some patients remained on the surgical waiting list after one year, follow-up should be extended to two years. Patients exposed to the PtDA achieved higher decision quality compared to those receiving usual education but there was no difference in wait for surgery. TRIALS REGISTRATION: ClinicalTrials.Gov NCT00743951. | Male | Decision Support Techniques | Humans | Patient Participation | OK |
| Ananda | Sen | Sarah T. | Hawley | Mack T. | Ruffin | Trials | 2013-00-00 2013 | BACKGROUND: Clinicians face challenges in promoting colorectal cancer screening due to multiple competing demands. A decision aid that clarifies patient preferences and improves decision quality can aid shared decision making and be effective at increasing colorectal cancer screening rates. However, exactly how such an intervention improves shared decision making is unclear. This study, funded by the National Cancer Institute, seeks to provide detailed understanding of how an interactive decision aid that elicits patient's risks and preferences impacts patient-clinician communication and shared decision making, and ultimately colorectal cancer screening adherence. METHODS/DESIGN: This is a two-armed single-blinded randomized controlled trial with the target of 300 patients per arm. The setting is eleven community and three academic primary care practices in Metro Detroit. Patients are men and women aged between 50 and 75 years who are not up to date on colorectal cancer screening. ColoDATES Web (intervention arm), a decision aid that incorporates interactive personal risk assessment and preference clarification tools, is compared to a non-interactive website that matches ColoDATES Web in content but does not contain interactive tools (control arm). Primary outcomes are patient uptake of colorectal cancer screening; patient decision quality (knowledge, preference clarification, intent); clinician's degree of shared decision making; and patient-clinician concordance in the screening test chosen. Secondary outcome incorporates a Structural Equation Modeling approach to understand the mechanism of the causal pathway and test the validity of the proposed conceptual model based on Theory of Planned Behavior. Clinicians and those performing the analysis are blinded to arms. DISCUSSION: The central hypothesis is that ColoDATES Web will improve colorectal cancer screening adherence through improvement in patient behavioral factors, shared decision making between the patient and the clinician, and concordance between the patient's and clinician's preferred colorectal cancer screening test. The results of this study will be among the first to examine the effect of a real-time preference assessment exercise on colorectal cancer screening and mediators, and, in doing so, will shed light on the patient-clinician communication and shared decision making 'black box' that currently exists between the delivery of decision aids to patients and subsequent patient behavior. TRIAL REGISTRATION: ClinicalTrials.gov ID NCT01514786. | Male | Decision Support Techniques | Humans | Physician-Patient Relations | OK |
| Eric | Dubé | Maggy | Wassef | Emmanuel | Bujold | Social Science & Medicine | 2012-06-00 juin 2012 | Support for a model of shared medical decision making, where women and their care providers discuss risks and benefits of their different options, reveal their preferences, and jointly make a decision, is a growing expectation in obstetric care. The objective of this study was to conduct a systematic review and meta-analysis of randomized controlled trials evaluating the efficacy of different decision aid tools compared to regular care for women facing several options in the specific field of obstetric care. We included published studies about interventions designed to aid mothers’ decision making and provide information about obstetrical treatment or screening options. Following a search of electronic databases for articles published in English and French from 1994 to 2010, we found ten studies that met the inclusion criteria. In this systematic review and meta-analysis we found that all decision aid tools, except for Decision Trees, facilitated significant increases in knowledge. The Computer-based Information Tool, the Decision Analysis Tools, Individual Counseling and Group Counseling intervention presented significant results in reducing anxiety levels. The Decision Analysis Tools and the Computer-based Information tool were associated with a reduction in levels of decisional conflict. The Decision Analysis Tool was the only tool that presented evidence of an impact on the final choice and final outcome. Decision aid tools can assist health professionals to provide information and counseling about choices during pregnancy and support women in shared decision making. The choice of a specific tool should depend on resources available to support their use as well as the specific decisions being faced by women, their health care setting and providers. (PsycINFO Database Record (c) 2012 APA, all rights reserved). (journal abstract) | Decision Making | decision aid tools | support women | pregnancy | Doublon |
| Eric | Dubé | Maggy | Wassef | Emmanuel | Bujold | Social Science & Medicine (1982) | 2012-06-00 Jun 2012 | Support for a model of shared medical decision making, where women and their care providers discuss risks and benefits of their different options, reveal their preferences, and jointly make a decision, is a growing expectation in obstetric care. The objective of this study was to conduct a systematic review and meta-analysis of randomized controlled trials evaluating the efficacy of different decision aid tools compared to regular care for women facing several options in the specific field of obstetric care. We included published studies about interventions designed to aid mothers' decision making and provide information about obstetrical treatment or screening options. Following a search of electronic databases for articles published in English and French from 1994 to 2010, we found ten studies that met the inclusion criteria. In this systematic review and meta-analysis we found that all decision aid tools, except for Decision Trees, facilitated significant increases in knowledge. The Computer-based Information Tool, the Decision Analysis Tools, Individual Counseling and Group Counseling intervention presented significant results in reducing anxiety levels. The Decision Analysis Tools and the Computer-based Information tool were associated with a reduction in levels of decisional conflict. The Decision Analysis Tool was the only tool that presented evidence of an impact on the final choice and final outcome. Decision aid tools can assist health professionals to provide information and counseling about choices during pregnancy and support women in shared decision making. The choice of a specific tool should depend on resources available to support their use as well as the specific decisions being faced by women, their health care setting and providers. | Decision Support Techniques | Humans | Patient Participation | Female | OK |
| Roland | Grad | Pierre | Pluye | R. Brian | Haynes | Implementation science: IS | 2012-00-00 2012 | BACKGROUND: This project engages patients and physicians in the development of Decision Boxes, short clinical topic summaries covering medical questions that have no single best answer. Decision Boxes aim to prepare the clinician to communicate the risks and benefits of the available options to the patient so they can make an informed decision together. METHODS: Seven researchers (including four practicing family physicians) selected 10 clinical topics relevant to primary care practice through a Delphi survey. We then developed two one-page prototypes on two of these topics: prostate cancer screening with the prostate-specific antigen test, and prenatal screening for trisomy 21 with the serum integrated test. We presented the prototypes to purposeful samples of family physicians distributed in two focus groups, and patients distributed in four focus groups. We used the User Experience Honeycomb to explore barriers and facilitators to the communication design used in Decision Boxes. All discussions were transcribed, and three researchers proceeded to thematic content analysis of the transcriptions. The coding scheme was first developed from the Honeycomb's seven themes (valuable, usable, credible, useful, desirable, accessible, and findable), and included new themes suggested by the data. Prototypes were modified in light of our findings. RESULTS: Three rounds were necessary for a majority of researchers to select 10 clinical topics. Fifteen physicians and 33 patients participated in the focus groups. Following analyses, three sections were added to the Decision Boxes: introduction, patient counseling, and references. The information was spread to two pages to try to make the Decision Boxes less busy and improve users' first impression. To try to improve credibility, we gave more visibility to the research institutions involved in development. A statement on the boxes' purpose and a flow chart representing the shared decision-making process were added with the intent of clarifying the tool's purpose. Information about the risks and benefits according to risk levels was added to the Decision Boxes, to try to ease the adaptation of the information to individual patients. CONCLUSION: Results will guide the development of the eight remaining Decision Boxes. A future study will evaluate the effect of Decision Boxes on the integration of evidence-based and shared decision making principles in clinical practice. | Male | Decision Support Techniques | Humans | Patient Participation | OK |
| Simon M. | Gilbody | Carl | Thompson |  |  | Psychological Assessment | 2013-09-00 septembre 2013 | For a test to be considered useful for making treatment decisions, it is necessary that making treatment decisions based on the results of the test be a preferable strategy to making treatment decisions without the test. Decision curve analysis is a framework for assessing when a test would be expected to be useful, which integrates evidence of a test’s performance characteristics (sensitivity and specificity), condition prevalence among at-risk patients, and patient preferences for treatment. We describe decision curve analysis generally and illustrate its potential through an application to tests for prodromal psychosis. Clinical psychosis is often preceded by a prodromal phase, but not all those with prodromal symptoms proceed to develop full psychosis. Patients identified as at risk for developing psychosis may be considered for proactive treatment to mitigate development of clinically defined psychosis. Tests exist to help identify those at-risk patients most likely to develop psychosis, but it is uncertain when these tests would be considered useful for making proactive treatment decisions. We apply decision curve analysis to results from a systematic review of studies investigating clinical tests for predicting the development of psychosis in at-risk populations, and present resulting decision curves that illustrate when the tests may be expected to be useful for making proactive treatment decisions. (PsycINFO Database Record (c) 2013 APA, all rights reserved). (journal abstract) | Shared decision making | Decision Making | Treatment | At Risk Populations | OK |
| Keith M. | Drake | Karen R. | Sepucha |  |  | Joint Commission Journal on Quality and Patient Safety / Joint Commission Resources | 2013-03-00 Mar 2013 | BACKGROUND: Good decision making has been increasingly cited as a core component of good medical care, and shared decision making is one means of achieving high decision quality. If it is to be a standard, good measures and protocols are needed for assessing the quality of decisions. Consistency with patient goals and concerns is one defining characteristic of a good decision. A new method for evaluating decision quality for major surgical decisions was examined, and a methodology for collecting the needed data was developed. METHODS: For a national probability sample of fee-for-service Medicare beneficiaries who had a coronary artery bypass graft (CABG), a lumpectomy or a mastectomy for breast cancer, or surgery for prostate cancer during the last half of 2008, a mail-survey of selected patients was carried out about one year after the procedures. Patients' goals and concerns, knowledge, key aspects of interactions with clinicians, and feelings about the decisions were assessed. A decision dissonance score was created that measured the extent to which patient ratings of goals ran counter to the treatment received. The construct and predictive validity of the decision dissonance score was then assessed. RESULTS: When data were averaged across all four procedures, patients with more knowledge and those who reported more involvement reported significantly lower Decision Dissonance Scores. Patients with lower Decision Dissonance Scores also reported more confidence in their decisions and feeling more positively about how the treatment turned out, and they were more likely to say that they would make the same decision again. CONCLUSIONS: Surveying discharged surgery patients is a feasible way to evaluate decision making, and Decision Dissonance appears to be a promising approach to validly measuring decision quality. | Male | Decision Making | Humans | Patient Satisfaction | OK |
| Nesochi | Adimorah | Jeff K. | Belkora | Beverly | Moy | Journal of the American College of Surgeons | 2012-01-00 Jan 2012 | BACKGROUND: Practice variation in breast cancer surgery has raised concerns about the quality of treatment decisions. We sought to evaluate the quality of decisions about surgery for early-stage breast cancer by measuring patient knowledge, concordance between goals and treatments, and involvement in decisions. STUDY DESIGN: A mailed survey of stage I/II breast cancer survivors was conducted at 4 sites. The Decision Quality Instrument measured knowledge, goals, and involvement in decisions. A multivariable logistic regression model of treatment was developed. The model-predicted probability of mastectomy was compared with treatment received for each patient. Concordance was defined as having mastectomy and predicted probability >0.5 or partial mastectomy and predicted probability <0.5. Frequency of discussion about partial mastectomy was compared with discussion about mastectomy using chi-square tests. RESULTS: Four hundred and forty patients participated (59% response rate). Mean overall knowledge was 52.7%; 45.9% knew that local recurrence risk is higher after breast conservation and 55.7% knew that survival is equivalent for the 2 options. Most participants (89.0%) had treatment concordant with their goals. Participants preferring mastectomy had lower concordance (80.5%) than those preferring partial mastectomy (92.6%; p = 0.001). Participants reported more frequent discussion of partial mastectomy and its advantages than of mastectomy, and 48.6% reported being asked their preference. CONCLUSIONS: Breast cancer survivors had major knowledge deficits, and those preferring mastectomy were less likely to have treatment concordant with goals. Patients perceived that discussions focused on partial mastectomy, and many were not asked their preference. Improvements in the quality of decisions about breast cancer surgery are needed. | Decision Making | Humans | Patient Participation | Questionnaires | OK |
| Jamie L. | Studts |  |  |  |  | Journal of Psychosocial Oncology | 2012-00-00 2012 | Follicular lymphoma (FL) is an indolent lymphoma that generally responds well to treatment. However, individuals with FL commonly face multiple complex treatment decision-making (TDM) experiences because it frequently follows a relapsing and remitting course. This study explored TDM and distress among individuals with FL (N = 32). Results indicated that most participants reported little decisional conflict or regret and wanted to be actively involved in TDM. However, more than 25% of participants reported clinically-relevant cancer-specific distress, and 60% indicated moderate or higher anxiety symptoms. Research is needed to clarify the cause and course of the psychological distress revealed in this study. | Male | Decision Making | Emotions | Humans | OK |
| David H. | Birnie |  |  |  |  | JAMA internal medicine | 2014-09-00 Sep 2014 | | Male | Decision Making | Humans | Patient Participation | OK |
| David C. | Grossman | Marie-Claire | Rosenberg | David P. | Stevens | The Laryngoscope | 2010-12-00 Dec 2010 | OBJECTIVES/HYPOTHESIS: To describe patterns of patient involvement in head and neck cancer decision making. STUDY DESIGN: Prospective longitudinal ethnography of otolaryngology patients making treatment decisions. METHODS: Grounded theory analysis of verbatim transcripts and original voice recordings from: 1) participant-driven diaries, 2) participants' office visits with their physicians, and 3) semistructured interviews completed after a treatment decision had been made. RESULTS: Patients with serious illness and experiencing considerable pain, discomfort, or alteration in the ability to perform activities of daily living, and who fear for their life, do not make decisions in a way that adheres to the conventional model of decision making, which presumes a sequential, office-based interaction with clear patient autonomy. These patients have the ability to interpret information they receive during office visits, but they describe making a treatment decision as "deciding to do something" not choosing a specific treatment. This group also describes "trust" or "confidence" in the physician as the most important factor in making a decision, not the type or amount of information received. They move through providers toward treatment in a linear fashion, from one physician specialty to the next, usually without doubling back to revisit previous decisions or discussions. CONCLUSIONS: Decision making in serious illness unfolds differently than in less serious problems. The conventional model does not fit this patient population, and reliance on trust of the physician figures prominently. Decision support should be aimed at physician decision making, promoting explicit incorporation of patient-specific data into the process. | Male | Decision Making | Humans | Patient Participation | OK |
| Barbara | Halpenny | Fangxin | Hong |  |  | Patient Education and Counseling | 2012-08-00 Aug 2012 | OBJECTIVE: To describe relationships between use of the Personal Patient Profile-Prostate (P3P) decision support system and patient characteristics, and perceived preparation for decision making (PrepDM), satisfaction and decisional regret in the context of prostate cancer treatment choice. METHODS: 494 men with localized prostate cancer (LPC) were randomized to receive the P3P intervention or usual care and completed pre-treatment, 1-month and 6-month outcome measures. Multivariable linear regression models were fit for each outcome. RESULTS: Physician consult visits prior to enrollment, race/ethnicity, and use of clinic-provided books were significant predictors of perceived PrepDM at 1 month. Prior Internet use and PrepDM significantly predicted 6-month decision satisfaction. Decisional regret was significantly predicted by demographics, anxiety, PrepDM score, and EPIC bowel domain score at 6 months. Use of P3P did not predict any outcome. CONCLUSION: While the P3P intervention did not significantly affect the outcomes, pre-enrollment information and preparation were strong predictors of the 1- and 6-month outcomes. Decision regret was significantly influenced by personal characteristics and post-treatment symptoms/side effects. PRACTICE IMPLICATIONS: Information received and used between biopsy and the treatment options consult visit is likely to make a difference in decision satisfaction. | Male | Decision Making | Emotions | Humans | OK |
|  |  |  |  |  |  | Danish Medical Journal | 2012-01-00 Jan 2012 | INTRODUCTION: Do-not-resuscitate (DNR) decisions are frequently made without informing the patients. We attempt to determine whether patients and physicians wish to discuss the DNR decision, who they think, should be the final decision maker and whether they agree on the indication for cardiopulmonary resuscitation (CPR) in case of cardiac arrest. MATERIAL AND METHODS: We carried out a questionnaire survey among 112 haemodialysis patients and 17 physicians at department of nephrology, Herlev Hospital. The patients were interviewed orally, the physicians responded to written questionnaires. RESULTS: The majority of patients (86%) and physicians (88%) answered, that patients ought to be involved in the DNR decision. However they both wanted to be the final decision maker. Most patients (69%) desired CPR in case of cardiac arrest. Physicians would attempt to resuscitate 88% of the patients. In 30% of the cases, the patient and the physician disagreed on whether or not to attempt resuscitation. CONCLUSION: Both patients and physicians think they ought to make the final DNR decision. In practice, patients are often not involved. Since the patient and the physician disagree regarding the indication for CPR in one third of the cases, we must assume that many patients are resuscitated against their wishes. National guidelines are required. FUNDING: not relevant. TRIAL REGISTRATION: not relevant. | Male | Decision Making | Humans | Patient Participation | OK |
| Peter | Charpentier | Blair | Olsen | Lanette | Errante | Arthritis Care & Research | 2012-07-00 Jul 2012 | OBJECTIVE: Despite the importance of achieving tight control, many patients with rheumatoid arthritis (RA) are not effectively treated with disease-modifying antirheumatic drugs. The objective of this study was to develop a decision support tool to inform RA patients with ongoing active disease about the risks and benefits related to biologic therapy. METHODS: We developed a balanced, web-based, decision support tool. Options, values, and probabilistic information were described using theoretically supported formulations. We conducted a pre-/posttest study to assess preliminary evidence of the tool's efficacy in improving knowledge related to biologics, clarity of values, willingness to take a biologic, and informed choice. RESULTS: We interviewed 104 subjects (mean age 62 years, 84% women, 87% white, and median duration of RA 8 years). Knowledge (coded on a 0-20 scale) and willingness to take a biologic (coded on a 0-10 scale) significantly increased after viewing the tool (mean differences 2.3 and 1.4, respectively; P < 0.0001 for both). Perceived knowledge and values clarity (coded on 0-100 scales) also significantly improved (mean differences 20.4 and 20.8, respectively; P < 0.0001 for both). The proportion of subjects making an informed value-concordant choice increased substantially from 35% to 64%. CONCLUSION: A tool designed to effectively communicate the risks and benefits associated with biologic therapy increased knowledge, patient willingness to escalate care, and the likelihood of making an informed choice. The results of this study support the need for a clinical trial to examine the impact of the tool in clinical practice. | Male | Humans | Patient Participation | Physician-Patient Relations | OK |
| Jessica | Ford | John | Cahill | Rajasekhar | Nekkanti | Pacing and clinical electrophysiology: PACE | 2014-01-00 Jan 2014 | INTRODUCTION: Sudden cardiac death is a well-documented public health problem and the implantable cardioverter defibrillator (ICD) has demonstrated benefit in reducing mortality. Prospective patients must identify and evaluate the ICD's pros and cons and produce a personal decision. The purpose of this study was to create and evaluate a measure of patient-evaluated pros and cons of the ICD, and its relationship to patient decision regarding ICD implantation. METHODS AND RESULTS: The ICD-decision analysis scale (ICD-DAS) was created and tested in prospective ICD recipients (N = 104). Factor analysis was performed to evaluate interitem relationships, and subsequently, identified subscales; additional psychosocial measures were used to predict the ICD decision. A two-factor measure for ICD decision making was established with two subscales: ICD Pros and ICD Cons. The subscales have high internal consistency and were strong predictors of intent to choose an ICD. Other psychosocial measures were not significantly predictive of ICD Choice, yet simultaneous entry of ICD Pros and Cons subscales resulted in a significant increase in R(2) , F(2, 59) = 19.36, P < 0.001. The full model was significantly greater than zero, F(11, 70) = 5.017, P < 0.001, R(2)  = 0.48. CONCLUSION: The ICD-DAS provides the first empirically tested and clinically useful approach to understanding the specific pros and cons for prospective ICD patients. The measure can assist clinicians with patient-centered discussions regarding sudden cardiac arrest treatments. The ICD-DAS will allow for the provision of tailored education or counseling and may be used to predict postdecision outcomes. | Male | Decision Making | Decision Support Techniques | Humans | OK |
| Robin | Tan | Jorge | Lujan-Zilberman | Patricia | Emmanuel | AIDS patient care and STDs | 2014-08-00 Aug 2014 | We assessed the decisional capacity (DC) of 72 youth with HIV, ages 13-24, using the MacArthur Competence Tool for Treatment, a structured interview that assesses DC along the following dimensions: understanding, appreciation, reasoning, and the ability to express a choice. Using previously established cutoff scores, results suggested 100% of youth were competent in the area of appreciation, but only 62% and 60% were competent in the areas of understanding and reasoning, respectively. Additional descriptive analyses reveal more detailed information regarding specific strengths and weaknesses within each of the dimensions of decisional capacity. These findings have important implications for health literacy initiatives, medical education, and treatment for youth with HIV, and support the need for adherence and secondary prevention interventions that include a decisional capacity component. | Male | Decision Making | Humans | Patient Participation | OK |
| Iffat | Javed | Saba | Pario | Shereen Zulfiqar | Bhutta | JPMA. The Journal of the Pakistan Medical Association | 2012-10-00 Oct 2012 | OBJECTIVE: To determine the attitude and factors leading to decision regarding the mode of delivery in women with previous experience of C-section. METHODS: The cross-sectional study was conducted at Maternity Unit of Jinnah Postgraduate Medical Centre, Karachi, from January to August 2008, and involved 150 women with one previous C-section without any recurrent cause. All women had a parity of two or more. Women with more than one caesarean section and women who lost contact were excluded. The women were recruited from antenatal clinic and were briefed by medical professionals before the questionnaire was filled out. SPSS version 10 and Chi square test were used for statistical analysis. RESULTS: Of the total 29 (19.3%) preferred a repeat caesarean section, and 121 (80.7%) opted for the trial of scar. No significant difference was seen in the mode of delivery when the decision was taken by women alone or when it was taken jointly by doctors and women. When the decision was taken by doctors, there was significant difference in the mode of deliveries. The patients expressed satisfaction towards the information received. Previous associated factors, like negative birth experience, had an influence on decision-making regarding the mode of delivery. CONCLUSION: Decision by women is extremely important regarding their mode of delivery and should be respected. Psychological support during pregnancy may provide an alternative to caesarean section for women with previous negative experience. | Decision Making | Humans | Patient Participation | Questionnaires | OK |
| Monica | Kleinefeld | Paolo | Confalonieri | Alessandra | Lugaresi | PloS One | 2013-00-00 2013 | OBJECTIVE: To assess decision-making in multiple sclerosis (MS) from third observer and patient perspectives. METHOD: Audio recordings of first-ever consultations with a participating physician (88 outpatients, 10 physicians) at four tertiary MS care clinics in Italy, were rated by a third observer using the Observing Patient Involvement in Shared Decision Making (OPTION) and by patients using the Perceived Involvement in Care Scale (PICS). RESULTS: Mean patient age was 37.5, 66% were women, 72% had MS, and 28% had possible MS or other disease. Mean PICS subscale scores (range 0 poor, 100 best possible) were 71.9 (SD 24.3) for "physician facilitation" (PICS-F); 74.6 (SD 22.9) for "patient information exchange" (PICS-I); and only 22.5 (SD 16.2) for "patient decision making" (PICS-DM). Mean OPTION total score (0 poor, 100 best possible) was 29.6 (SD 10.3). Poorest OPTION scores were found for items assessing "preferred patient approach to receiving information" and "preferred patient level of involvement." Highest scores were for "clinician drawing attention to identified problem", "indicating need for decision making," and "need to review the decision." Consultation time, woman physician, patient-physician gender concordance and PICS-F were associated with higher OPTION total score; older physician and second opinion consultation were associated with lower OPTION score. CONCLUSIONS: In line with findings in other settings, our third observer findings indicated limited patient involvement abilities of MS physicians during first consultations. Patient perceptions of physician skills were better than third observers', although they correlated. Consultations with women physicians, and younger physicians, were associated with higher third observer and patient-based scores. Our findings reveal a need to empower Italian MS physicians with better communication and shared decision-making skills, and show in particular that attention to MS patient preferences for reception of information and involvement in health decisions, need to be improved. | Male | Decision Making | Humans | Patient Participation | OK |
| Philip G. | Jones | Yan | Li | John A. | Spertus | JAMA internal medicine | 2013-07-08 Jul 8, 2013 | | Male | Decision Making | Humans | Patient Participation | OK |
| Michael P. | Pignone | Mick P. | Couper | Michael J. | Barry | Medical Decision Making | 2010-09-00 septembre 2010 | Background: Patients should understand the risks and benefits of cancer screening in order to make informed screening decisions. Objectives: To evaluate the extent of informed decision making in patient-provider discussions for colorectal (CRC), breast (BrCa), and prostate (PCa) cancer screening. Setting: National sample of US adults identified by random-digit dialing. Design: Cross-sectional survey conducted between November 2006 and May 2007. Participants: English-speaking US adults aged 50 y and older who had discussed cancer screening with a health care provider within the previous 2 y. Measurements: Cancer screening survey modules that asked about demographic characteristics, cancer knowledge, the importance of various sources of information, and self-reported cancer screening decision-making processes. Results: Overall, 1082 participants completed 1 or more of the 3 cancer modules. Although participants generally considered themselves well informed about screening tests, half or more could not correctly answer even 1 open-ended knowledge question for any given module. Participants consistently overestimated risks for being diagnosed with and dying from each cancer and overestimated the positive predictive values of prostate-specific antigen tests and mammography. Providers were the most highly rated information source, usually initiated screening discussions (64%-84%), and often recommended screening (73%-90%). However, participants reported that providers elicited their screening preferences in only 31% (CRC women) to 57% (PCa) of discussions. Although more than 90% of the discussions addressed the pros of screening, only 19% (BrCa) to 30% (PCa) addressed the cons of screening. Limitations: Recall bias is possible because screening process reports were not independently validated. Conclusions: Cancer screening decisions reported by patients who discussed screening with their health care providers consistently failed to meet criteria for being informed. Given the high ratings for provider information and frequent recommendations for screening, providers have important opportunities to ensure that informed decision making occurs for cancer screening decisions. (PsycINFO Database Record (c) 2012 APA, all rights reserved). (journal abstract) | Decision Making | Therapeutic Processes | Cancer Screening | medical decision making | OK |
| Eleanor | Singer | Peter A. | Ubel | Sonja | Ziniel | Medical Decision Making: An International Journal of the Society for Medical Decision Making | 2010-10-00 2010 Sep-Oct | BACKGROUND: Although many researchers have examined patient involvement and patient-provider interactions within specific clinical environments, no nationally representative data exist to characterize patient perceptions of decision making and patient-provider communications across multiple common medical decisions. OBJECTIVE: To identify deficits and variations in the patient experience of making common medical decisions about initiation of prescription medications for hypertension, hypercholesterolemia, or depression; screening tests for colorectal, breast, or prostate cancer; and surgeries for knee or hip replacement, cataracts, or lower back pain, as well as to identify factors associated with patient confidence in the decisions. SETTING: National sample of US adults identified by random-digit dialing. DESIGN: Cross-sectional survey conducted from November 2006 to May 2007. PARTICIPANTS: Included 2473 English-speaking adults age 40 and older who reported undertaking 1 or more of the above 9 medical actions or discussing doing so with a health care provider within the past 2 years. MEASUREMENTS: Patients reported who initiated discussions and made the final decisions, how much discussion of pros and cons occurred, whether they were asked about their preferences, and their confidence that the decision "was the right one." RESULTS: The proportion of patient-driven decisions varied significantly across decisions (range: blood pressure: 16% to knee/hip replacement: 48%). Most patients (78%-85%) reported that providers made a recommendation, and such recommendations generally favored taking medical action. Fewer patients reported that providers asked them about their preferences (range: colon cancer screening: 34% to knee/hip replacement: 80%) or discussed reasons not to take action (range: breast cancer screening: 20% to lower back surgery: 80%). Decision confidence was higher among patients who reported primarily making the decision themselves (odds ratio [OR] = 14.6, P < 0.001) or having been asked for their preference (OR = 1.32, P < 0.01) and was lower among patients whose patient-provider discussions included cons (OR = 0.74, P = 0.008). LIMITATIONS: Recall biases may affect patients' memories of their decision-making processes. CONCLUSIONS: DECISIONS participants reported wide variations in the proportion of discussions that included a conversation about reasons not to take action or a conversation about patients' preferences about what they would like to do. These factors appear directly related to patients' confidence that the decision was "right." | Male | Humans | Patient Participation | Patient Satisfaction | OK |
| Mark | Friedberg | Meredith B. | Rosenthal | Lucian | Leape | Medical care research and review: MCRR | 2011-02-00 Feb 2011 | Integration of care is emerging as a central challenge of health care delivery, particularly for patients with multiple, complex chronic conditions. The authors argue that the concept of "integrated patient care" would benefit from further clarification regarding (a) the object of integration and (b) its essential components, particularly when constructing measures.To address these issues, the authors propose a definition of integrated patient care that distinguishes it from integrated delivery organizations, acknowledging that integrated organizational structures and processes may fail to produce integrated patient care. The definition emphasizes patients' central role as active participants in managing their own health by including patient centeredness as a key element of integrated patient care. Measures based on the proposed definition will enable empirical assessment of the potential relationships between the integration of organizations, the integration of patient care, and patient outcomes, providing valuable guidance to health systems reformers. | Humans | Patient Participation | health promotion | Patient-Centered Care | OK |
| Katell | Mignotte | Jean-Pierre | Lebeau | Michel | Bismuth | The European Journal of General Practice | 2013-09-00 Sep 2013 | BACKGROUND: Colorectal cancer (CRC) is the third most common cancer worldwide. In France, mass screening has been established with FOBT since 2008. The participation rate remains too low. Previous studies were conducted to explore doctors' and patients' perspectives. OBJECTIVE: This study was conducted to explore GPs' performance during consultations in which patients ask for FOBT, focusing on two different aspects: the core content of the consultation and the communication style used between GPs and patients. METHODS: Nine purposively sampled GPs were asked to audiotape specific consultations. Content analysis was performed using Nvivo 9 software. Communication between doctors and patients was explored using RIAS coding. RESULTS: GPs audio taped specific parts of 35 different consultations when they discussed and delivered the FOBT. The core content included primarily biomedical statements with a large portion dedicated to technical aspects. The communication style was not patient-centred. CONCLUSION: While the participation rate of mass screening in France is still low, the analysis of recorded consultations by French GPs confirms that the way of delivering FOBT can be improved. | Male | Decision Making | Humans | Patient Participation | Doublon |
| Katell | Mignotte | Jean-Pierre | Lebeau | Michel | Bismuth | The European Journal of General Practice | 2013-09-00 Sep 2013 | BACKGROUND: Colorectal cancer (CRC) is the third most common cancer worldwide. In France, mass screening has been established with FOBT since 2008. The participation rate remains too low. Previous studies were conducted to explore doctors' and patients' perspectives. OBJECTIVE: This study was conducted to explore GPs' performance during consultations in which patients ask for FOBT, focusing on two different aspects: the core content of the consultation and the communication style used between GPs and patients. METHODS: Nine purposively sampled GPs were asked to audiotape specific consultations. Content analysis was performed using Nvivo 9 software. Communication between doctors and patients was explored using RIAS coding. RESULTS: GPs audio taped specific parts of 35 different consultations when they discussed and delivered the FOBT. The core content included primarily biomedical statements with a large portion dedicated to technical aspects. The communication style was not patient-centred. CONCLUSION: While the participation rate of mass screening in France is still low, the analysis of recorded consultations by French GPs confirms that the way of delivering FOBT can be improved. | Male | Decision Making | Humans | Patient Participation | OK |
| Peter John | Connelly |  |  |  |  | Dementia (London, England) | 2013-01-00 Jan 2013 | Scotland's National Dementia Strategy calls for people with dementia and their carers to give voice to what they see as the priorities for dementia research. We sent questionnaires on dementia research priorities, locus and type of research, desired outcome measures and willingness to volunteer, to two groups of dementia research stakeholders: (1) people with dementia and their carers who may or may not be participating in research and (2) those who are directly participating in research. We also made the questionnaire available on a national dementia research website. Five hundred and fourteen responses were received. The top four topics rated by importance were identical across all three groups of respondents: early detection (38.1%), drug trials (14.2%), studies on people living at home (9.7%) and study of carers (6.0%). The data can help shape the dementia research agenda, but more information needs to be made available to the public about other potential research areas. | Humans | Patient Participation | Questionnaires | Outcome Assessment (Health Care) | OK |
| Amy | Duncan | Deborah | Turnbull | Stephen R. | Cole | BMC public health | 2011-00-00 2011 | BACKGROUND: Previous research points to differences between predictors of intention to screen for colorectal cancer (CRC) and screening behavior, and suggests social ecological factors may influence screening behavior. The aim of this study was to compare the social cognitive and social ecological predictors of intention to screen with predictors of participation. METHODS: People aged 50 to 74 years recruited from the electoral roll completed a baseline survey (n = 376) and were subsequently invited to complete an immunochemical faecal occult blood test (iFOBT). RESULTS: Multivariate analyses revealed five predictors of intention to screen and two predictors of participation. Perceived barriers to CRC screening and perceived benefits of CRC screening were the only predictor of both outcomes. There was little support for social ecological factors, but measurement problems may have impacted this finding. CONCLUSIONS: This study has confirmed that the predictors of intention to screen for CRC and screening behaviour, although overlapping, are not the same. Research should focus predominantly on those factors shown to predict participation. Perceptions about the barriers to screening and benefits of screening are key predictors of participation, and provide a focus for intervention programs. | Male | Humans | Patient Participation | Questionnaires | OK |
| Atsushi | Ohyama | Yoshinori | Takaesu |  |  | The Bulletin of Tokyo Dental College | 2012-00-00 2012 | In economics, the concept of utility refers to the strength of customer preference. In health care assessment, the visual analogue scale (VAS), the standard gamble, and the time trade-off are used to measure health state utilities. These utility measurements play a key role in promoting shared decision-making in dental care. Individual preference, however, is complex and dynamic. The purpose of this study was to investigate the relationship between patient preference and educational intervention in the field of dental health. The data were collected by distributing questionnaires to employees of two companies in Japan. Participants were aged 18-65 years and consisted of 111 males and 93 females (204 in total). One company (Group A) had a dental program of annual check-ups and health education in the workplace, while the other company (Group B) had no such program. Statistical analyses were performed with the t-test and Chi-square test. The questionnaire items were designed to determine: (1) oral health-related quality of life, (2) dental health state utilities (using VAS), and (3) time trade-off for regular dental check-ups. The percentage of respondents in both groups who were satisfied with chewing function, appearance of teeth, and social function ranged from 23.1 to 42.4%. There were no significant differences between groups A and B in the VAS of decayed, filled, and missing teeth. The VAS of gum bleeding was 42.8 in Group A and 51.3 in Group B (p<0.05). The percentage of persons having a regular dental check-up every three months was 34.1 and 31.3% in Groups A and B respectively. These results suggest that low preference results from lack of opportunity or utilization of dental care in the worksite. Ascertaining the factors involved in patient preference may have significant potential benefits in shared decision-making. | Male | Decision Making | Humans | Questionnaires | OK |
| Naureen | Attiullah | Michael | Friedman | Cristina | Toba | Annals of Clinical Psychiatry: Official Journal of the American Academy of Clinical Psychiatrists | 2011-08-00 Aug 2011 | BACKGROUND: Current operational definitions of remission, at their root, are exclusively symptom-based and therefore limited in scope. In this report from the Rhode Island Methods to Improve Diagnostic Assessment and Services (MIDAS) project we examined patient acceptability of a new measure, the Remission from Depression Questionnaire (RDQ), a scale designed to capture a broader array of domains patients consider relevant to the construct of remission. Patient acceptance of the RDQ was compared with that of the Quick Inventory of Depressive Symptomatology (QIDS), the instrument used to measure outcome in the Sequenced Treatment Alternatives to Relieve Depression study. METHODS: One hundred and two depressed outpatients in ongoing treatment completed the RDQ, QIDS, and a 9-item measure of patient preference. RESULTS: Patients indicated the RDQ was a better indicator of their overall state and treatment goals. Patients judged the RDQ to be a more accurate and preferred measure to determine treatment outcome, and a more accurate indicator of remission. CONCLUSIONS: Patients considered the multifactorial RDQ a more accurate indicator of their treatment goals than a purely symptom measure such as the QIDS. | Male | Humans | Patient Participation | Questionnaires | OK |
| Ranak | Trivedi | Diana | Parrish | Connie | Standiford | The American Journal of Managed Care | 2013-11-00 Nov 2013 | OBJECTIVE: To understand patient participation in interactive voice response (IVR) depression monitoring and self-management support calls and estimate the workload from clinical alerts based on patients' IVR reports. STUDY DESIGN: Observational study from program implementation in 13 community- and university-based primary care practices. METHODS: Patients with depression were identified using electronic records and enrolled by telephone. Patients were asked to complete IVR assessments weekly; those with significantly improved symptoms had the option of completing assessments monthly. Patients could enroll with an informal caregiver who received automated feedback based on patients' IVR reports. Clinicians received alerts regarding significant changes in the scores on the Patient Health Questionnaire 9-item depression measure, antidepressant adherence problems, or suicidal ideation. RESULTS: A total of 387 patients were followed for 12,042 weeks. More than half (59%) opted to participate with a caregiver. Patients completed 68% of 7912 attempted IVR assessments. Assessment completion was unrelated to patients' depressive symptoms and was higher among those who participated with a caregiver, were married, had more comorbidities, or reported missing a prior appointment. Assessment completion was lower when patients received monthly versus weekly assessment attempts. Clinical alerts were generated during 4.9% of follow-up weeks; most represented medication adherence problems (2.8%). Alerts indicating suicidal ideation were rare (0.2% of patient-weeks). CONCLUSIONS: IVR support calls represent a viable strategy for increasing access to depression monitoring and self-management assistance in primary care. These programs generate a manageable number of alerts, most of which can be triaged with limited physician involvement. | | | | | OK |
| Elizabeth B. | Habermann | Erik P. | Hess | Robert R. | Cima | Journal of the American College of Surgeons | 2014-02-00 Feb 2014 | BACKGROUND: Surgical patients and their physicians currently have tools to provide individualized prognostication for morbidity and mortality. For improved shared decision making, formal prediction of patient-centered outcomes is necessary. We derived and validated a simple, interview-based method to predict discharge home after surgery. STUDY DESIGN: We used the American College of Surgeons National Surgical Quality Improvement Program (ACS NSQIP) Patient User File for 2011. Derivation in general and vascular surgery patients undergoing inpatient surgery was completed using serial multiple logistic regression. Validation was performed within multiple surgical specialties. RESULTS: The derivation cohort included 88,068 patients, of whom 11,771 (13.4%) were not discharged home. The derived Home Calculator had excellent discrimination (c-statistic = 0.864) using 5 [corrected] variables: age, American Society of Anesthesiologists' performance status, elective surgery, and preadmission residence. Validation cohorts had varying rates of home discharge as follows: general (63,826 of 71,591, 89.2%), vascular (12,319 of 16,102, 76.5%), gynecologic (16,603 of 17,005, 97.6%), urologic (13,662 of 14,435, 94.6%), orthopaedic (12,000 of 19,514, 61.5%), thoracic (4,467 of 5,092, 87.7%). The Home Calculator provided good to excellent discrimination in validation cohorts: general (c = 0.866), vascular (c = 0.800), gynecologic (c = 0.793), urologic (c = 0.814), orthopaedic (c = 0.876), and thoracic (c = 0.800). Comparable discrimination was demonstrated in sensitivity analyses in surgical patients admitted exclusively from home. CONCLUSIONS: We derived and validated a simple Home Calculator that reliably predicts discharge to home after surgery and may be useful when counseling patients about postoperative course. Patient-centered tools such as this may allow physicians to better prepare patients and families for surgery and the recovery process. | Male | Humans | United States | Young Adult | OK |
| Harbinder | Sandhu | Frances | Griffiths | Jill | Thistlewaite | Arthritis Care & Research | 2014-06-00 Jun 2014 | OBJECTIVE: To develop a decision support package for people with low back pain (LBP) referred for physiotherapy. METHODS: We used a program of exploratory work, including literature reviews, a Delphi study, a nominal group with physiotherapists, focus groups with patients, and secondary analysis of existing interview data. RESULTS: We developed an information booklet describing the evidence-based treatment modalities available in a physiotherapy department. This includes data on likely benefits and risks and how the intervention is delivered. The booklet specifically addresses questions identified as important in our exploratory work. Space is provided for patients to note down the pros and cons of each treatment and what matters to them when choosing treatments. The patient is subsequently directed to a section that explores any gaps in knowledge, values, support, and choice before finally clarifying if a treatment decision is possible. At this stage they are encouraged to note down any questions or concerns they have to be discussed at the first physiotherapy consultation. This overall package includes patient material in the form of a booklet posted prior to their consultation, plus the enhanced consultation with the specially trained physiotherapist. Patients then receive their chosen treatment. In addition we developed a training package for physiotherapists that explains the content of the booklet and supports them in using informed, shared decision making in their consultation. CONCLUSION: This package has the potential to improve effectiveness of treatments and patient satisfaction for LBP by facilitating patient choice and therefore matching patients more effectively to different treatments. | Decision Support Techniques | Humans | Evidence-based medicine | Choice Behavior | OK |
|  |  |  |  |  |  | Circulation. Cardiovascular Quality and Outcomes | 2014-05-00 May 2014 | |  |  |  |  | OK |
| Jacky | Boivin | Glyn | Elwyn |  |  | Health Expectations: An International Journal of Public Participation in Health Care and Health Policy | 2012-03-00 Mar 2012 | BACKGROUND: Evidence suggests that in decision contexts characterized by uncertainty and time constraints (e.g. health-care decisions), fast and frugal decision-making strategies (heuristics) may perform better than complex rules of reasoning. OBJECTIVE: To examine whether it is possible to design deliberation components in decision support interventions using simple models (fast and frugal heuristics). DESIGN: The 'Take The Best' heuristic (i.e. selection of a 'most important reason') and 'The Tallying' integration algorithm (i.e. unitary weighing of pros and cons) were used to develop two deliberation components embedded in a Web-based decision support intervention for women facing amniocentesis testing. Ten researchers (recruited from 15), nine health-care providers (recruited from 28) and ten pregnant women (recruited from 14) who had recently been offered amniocentesis testing appraised evolving versions of 'your most important reason' (Take The Best) and 'weighing it up' (Tallying). RESULTS: Most researchers found the tools useful in facilitating decision making although emphasized the need for simple instructions and clear layouts. Health-care providers however expressed concerns regarding the usability and clarity of the tools. By contrast, 7 out of 10 pregnant women found the tools useful in weighing up the pros and cons of each option, helpful in structuring and clarifying their thoughts and visualizing their decision efforts. Several pregnant women felt that 'weighing it up' and 'your most important reason' were not appropriate when facing such a difficult and emotional decision. CONCLUSION: Theoretical approaches based on fast and frugal heuristics can be used to develop deliberation tools that provide helpful support to patients facing real-world decisions about amniocentesis. | Decision Support Techniques | Humans | Patient Participation | Questionnaires | OK |
| Agathe I. | Charvet-Bérard | Thomas V. | Perneger |  |  | Patient Education and Counseling | 2011-04-00 avril 2011 | Objective: To examine factors associated with desire for autonomy in health care decisions in the general population. Methods: Mailed survey of 2348 residents of Geneva, Switzerland. Participants answered questions on a scale measuring their desire for autonomy in health care decisions. The scale was scored between 0 (lowest desire for autonomy) and 100 (highest desire for autonomy). Results: On average the respondents favoured shared or active involvement in medical decisions (mean score 62.0, SD 20.9), but attitudes varied considerably. In the multivariate model, factors associated with a higher desire for autonomy included female gender, younger age, higher education, living alone, reporting an excellent global health and—a new observation compared to previous studies—having made several medical decisions in the past 6 months. Conclusions: The attitudes of the general public appear to be consistent with the model of shared decision making. However, people vary considerably in their desire for autonomy. Practice implications: An explicit assessment of each individual's desire for autonomy may improve the decision-making process. Such an assessment should be repeated regularly, as familiarity with medical decisions may increase the desire for autonomy. (PsycINFO Database Record (c) 2012 APA, all rights reserved). (journal abstract) | Decision Making | Primary Health Care | health care decisions | Independence (Personality) | Doublon |
| Agathe I. | Charvet-Bérard | Thomas V. | Perneger |  |  | Patient Education and Counseling | 2011-04-00 Apr 2011 | OBJECTIVE: To examine factors associated with desire for autonomy in health care decisions in the general population. METHODS: Mailed survey of 2348 residents of Geneva, Switzerland. Participants answered questions on a scale measuring their desire for autonomy in health care decisions. The scale was scored between 0 (lowest desire for autonomy) and 100 (highest desire for autonomy). RESULTS: On average the respondents favoured shared or active involvement in medical decisions (mean score 62.0, SD 20.9), but attitudes varied considerably. In the multivariate model, factors associated with a higher desire for autonomy included female gender, younger age, higher education, living alone, reporting an excellent global health and - a new observation compared to previous studies - having made several medical decisions in the past 6 months. CONCLUSIONS: The attitudes of the general public appear to be consistent with the model of shared decision making. However, people vary considerably in their desire for autonomy. PRACTICE IMPLICATIONS: An explicit assessment of each individual's desire for autonomy may improve the decision-making process. Such an assessment should be repeated regularly, as familiarity with medical decisions may increase the desire for autonomy. | Male | Decision Making | Humans | Patient Participation | OK |
| Anant R. C. | Patel | Richa | Singh | Beverly | Kowlessar | The European Respiratory Journal | 2014-03-00 Mar 2014 | Uncertainty exists over the ability of the exacerbations of chronic pulmonary disease tool (EXACT) patient-reported outcome diary to quantify exacerbation severity and frequency. To clarify this, we investigated the ability of the EXACT to assess severity of exacerbations and examined the relationship between exacerbations diagnosed using London chronic obstructive pulmonary disease (COPD) cohort diary cards, physician review and symptom-defined events using the EXACT. 58 patients enrolled in the London Chronic Obstructive Pulmonary Disease (COPD) cohort prospectively completed the EXACT during 128 cohort diary card-defined exacerbations between January 2010 and April 2012. Mean ± sd EXACT scores increased from 42.6 ± 8.6 at baseline to 48.0 ± 8.6 at exacerbation onset (p<0.001), and rose further to a maximum score of 54.1 ± 8.9. Maximum EXACT scores were significantly higher in treated than untreated events. Time taken for EXACT scores to return to baseline was significantly related to symptom recovery time as judged by London COPD cohort diary cards, and to peak expiratory flow rate recovery. ∼50% of both diary card-defined and healthcare utilisation exacerbations crossed the EXACT event threshold. However, only 27.9% of diary card-defined and 34.6% of healthcare utilisation exacerbations fully met the criteria for an EXACT event. Patients exhibited smaller rises in the EXACT score at exacerbation as baseline disease severity increased. The EXACT is an effective method of evaluating chronic obstructive pulmonary disease exacerbation severity. However, concerns remain about the ability of the EXACT to accurately detect exacerbations. | Time Factors | Male | Humans | Patient Participation | Doublon |
| Anant R. C. | Patel | Richa | Singh | Beverly | Kowlessar | The European Respiratory Journal | 2014-03-00 Mar 2014 | Uncertainty exists over the ability of the exacerbations of chronic pulmonary disease tool (EXACT) patient-reported outcome diary to quantify exacerbation severity and frequency. To clarify this, we investigated the ability of the EXACT to assess severity of exacerbations and examined the relationship between exacerbations diagnosed using London chronic obstructive pulmonary disease (COPD) cohort diary cards, physician review and symptom-defined events using the EXACT. 58 patients enrolled in the London Chronic Obstructive Pulmonary Disease (COPD) cohort prospectively completed the EXACT during 128 cohort diary card-defined exacerbations between January 2010 and April 2012. Mean ± sd EXACT scores increased from 42.6 ± 8.6 at baseline to 48.0 ± 8.6 at exacerbation onset (p<0.001), and rose further to a maximum score of 54.1 ± 8.9. Maximum EXACT scores were significantly higher in treated than untreated events. Time taken for EXACT scores to return to baseline was significantly related to symptom recovery time as judged by London COPD cohort diary cards, and to peak expiratory flow rate recovery. ∼50% of both diary card-defined and healthcare utilisation exacerbations crossed the EXACT event threshold. However, only 27.9% of diary card-defined and 34.6% of healthcare utilisation exacerbations fully met the criteria for an EXACT event. Patients exhibited smaller rises in the EXACT score at exacerbation as baseline disease severity increased. The EXACT is an effective method of evaluating chronic obstructive pulmonary disease exacerbation severity. However, concerns remain about the ability of the EXACT to accurately detect exacerbations. | Time Factors | Male | Humans | Patient Participation | OK |
| Peggy Pei-Chia | Chiang | Ecosse L. | Lamoureux | Gwyneth | Rees | Canadian Journal of Ophthalmology. Journal Canadien D'ophtalmologie | 2012-04-00 Apr 2012 | OBJECTIVE: To identify the determinants of patients' decision-making for participation in a cataract surgery clinical trial in a tertiary care hospital. DESIGN: Prospective observational study. PARTICIPANTS: We interviewed by questionnaire 40 patients scheduled to have cataract surgery. METHODS: The data collected from patients included attitudes about informed consent, evaluation of the provision of information, and sociodemographic variables. RESULTS: Overall, 20 (50%) patients consented to enter the trial. All patients (n = 20;100%) in the consenting group expected positive implications compared to 6 (30%) in the nonconsenting group, who expected negative implications (p = 0.008). The majority of patients (90%) in the nonconsenting group felt that the degree of risk incurred by undergoing trial treatment was moderate to high. The perceived burden imposed on daily life by participating was seen by the nonconsenting group to be moderately higher in 11 patients (55%) compared to 3 patients (15%) in the consenting group (p = 0.026). Of the nonconsenting patients, 50% were dissatisfied with the attitudes of medical experiments as compared to 2% of those who did consent (p = 0.023). CONCLUSION: The major reasons for participation in a cataract surgery trial were (i) expected positive implications resulting from involvement; and (ii) satisfaction with the attitudes of medical experiments. Patients who refused to enter the trial felt that the degree of risk as well as the burden imposed on daily life by participating were high. Demographic variables did not have any impact on a patients' decisions to accept or decline entry into the trial. | Male | Decision Making | Humans | Patient Participation | OK |
| Steven T. | Johnson | Peggy | Dunbar | Marie-France | Langlois | BMC public health | 2013-00-00 2013 | BACKGROUND: Lifestyle behavior modification is an essential component of self-management of type 2 diabetes. We evaluated the prevalence of engagement in lifestyle behaviors for management of the disease, as well as the impact of healthcare professional support on these behaviors. METHODS: Self-reported data were available from 2682 adult respondents, age 20 years or older, to the 2011 Survey on Living with Chronic Diseases in Canada's diabetes component. Associations with never engaging in and not sustaining self-management behaviors (of dietary change, weight control, exercise, and smoking cessation) were evaluated using binomial regression models. RESULTS: The prevalence of reported dietary change, weight control/loss, increased exercise and smoking cessation (among those who smoked since being diagnosed) were 89.7%, 72.1%, 69.5%, and 30.6%, respectively. Those who reported not receiving health professional advice in the previous 12 months were more likely to report never engaging in dietary change (RR = 2.7, 95% CI 1.8 - 4.2), exercise (RR = 1.7, 95% CI 1.3 - 2.1), or weight control/loss (RR = 2.2, 95% CI 1.3 - 3.6), but not smoking cessation (RR = 1.0; 95% CI: 0.7 - 1.5). Also, living with diabetes for more than six years was associated with not sustaining dietary change, weight loss and smoking cessation. CONCLUSION: Health professional advice for lifestyle behaviors for type 2 diabetes self-management may support individual actions. Patients living with the disease for more than 6 years may require additional support in sustaining recommended behaviors. | Male | Humans | Patient Participation | Young Adult | OK |
| Norbert | Schmitz | Lucyna | Lach | Mary | Law | Research in Developmental Disabilities | 2013-09-00 Sep 2013 | Studies have identified restrictions in engagement in leisure activities for adolescents with disabilities. Participation is a complex construct and likely influenced by a variety of factors. These potential determinants have not yet been sufficiently explored in the population of adolescents with cerebral palsy (CP). The objective of this study is to estimate the potential influence of adolescent characteristics and environmental factors as determinants of participation in leisure activities for adolescents with CP. A cross-sectional design was used. Participants were adolescents (12-19 years old) with cerebral palsy. Participants were assessed with the Vineland Adaptive Behavior Scale - II, Gross Motor Function Measure, Gross Motor Function Classification System, Manual Ability Classification System and completed the Self-Perception Profile for Adolescents, Dimensions of Mastery Questionnaire, Strengths and Difficulties Questionnaire, Family Environment Scale, the European Child Environment Questionnaire and the Preferences for Activities of Children. The main outcome measure was the Children's Assessment of Participation and Enjoyment. 187 adolescents (age M=15.4; SD=2.2) completed the study. Multivariate models of participation in leisure revealed associations with factors related to the adolescents' functional characteristics and attitudes, the family environment, socioeconomic status, and contextual factors such as school type, and collectively explained from 28% (diversity of skill-based activities) up to 48% (intensity and diversity of self-improvement activities) of the variance in intensity and diversity in five leisure participation domains (diversity: r(2)=.33 recreational; r(2)=.39 active-physical; r(2)=.33 social activities). Adolescent's mastery motivation, self-perception and behavior were individually associated with participation in different activity domains, but did not strongly predict participation within multivariate models, while preferences for activities were strong predictors of participation in all domains, except for skill-based activities. Engagement in different types of leisure activities is important for adolescents' development and well-being. Health care professionals should consider adolescents' and families' characteristics to promote participation in leisure activities. | Male | Humans | Patient Participation | Questionnaires | OK |
| Reinier P. | Akkermans | J. H. A. | Vollebergh | Richard P. T. M. | Grol | Fertility and Sterility | 2010-09-00 Sep 2010 | OBJECTIVE: To assess determinants of patients' experiences and satisfaction with fertility care. DESIGN: Cross-sectional questionnaire study. SETTING: Sixteen fertility clinics in The Netherlands. PATIENT(S): A total of 1,499 infertile women in The Netherlands who visited a participating clinic in April-June 2005 for diagnostics or treatment. MAIN OUTCOME MEASURES: Patients experiences and satisfaction with several aspects of fertility care, and the patient and clinic characteristics that are determinants of those two concepts. RESULT(S): In general, patients' satisfaction with care was high (94%). Waiting times, information provision and emotional support were experienced the least positive aspects of care. Determinants of all care aspects were found to be significant at four different domains: three at patient level, i.e., demographic characteristics, type of received treatment and both general and mental health status, and one at clinic level, i.e., organization of care. CONCLUSION(S): This study provides an increased understanding of the determinants of patients' experiences and satisfaction with fertility care. This enables professionals to tailor their care to specific subgroups of patients and adjust their organization of fertility care where needed. Moreover, the study underlines the need to investigate whether case-mix correction is necessary whenever interpreting patient-surveys on care experiences, because both the patient's and the clinic's characteristics can influence the way that health care delivery is experienced. Demographic background of this regional patient sample was rather homogeneous, which should be taken into account when interpreting results. | Male | Humans | Patient Participation | Patient Satisfaction | OK |
| Sara J. | MacLennan | Craig R. | Ramsay | Marion K. | Campbell | Clinical Trials (London, England) | 2013-00-00 2013 | BACKGROUND: The use of decision-support interventions in the context of decisions about trial participation is an emergent field. There is a lack of evidence about what information is deemed important to support decisions about informed consent for clinical trials, and whether different groups agree on the information for inclusion. PURPOSE: The overall objective was to determine the items which different stakeholder groups viewed to be important for inclusion in a decision-support tool when making decisions about clinical trial participation, with a view to use these as a framework for developing decision-support tools in this context. This is the first study to have addressed this issue. METHODS: A modified Delphi method was used to determine agreement on importance of items. The 'stakeholder' panel was made up of 49 individuals from 5 groups: 11 trialists, 6 research nurses, 7 ethics committee chairs, 9 decision-support experts, and 16 patients (9 trial experienced and 7 trial non-experienced). Two rounds of rating were completed. Items with a median of 7-10 with ≥65% of any one group (from aggregate ratings) in agreement were considered important for inclusion. RESULTS: The stakeholder panel achieved consensus on the majority of items included (60/66), agreeing that these were important for inclusion in a decision-support tool for trial participation. These included items covering information about trial participation and standard care, information on the likelihood of receiving different treatments, information to help patients determine what matters most to them, ensuring that the information is balanced, guidance on how to make a decision, disclosure of any conflicts of interest, using plain language in the tool, and guidance on the decision-support development process. Some areas of divergence among the panel were also identified relating to the use of patient stories. LIMITATIONS: Selection bias may be a limitation in this study due to the manner in which the participants were invited to take part, and therefore, the representativeness, and reproducibility with another group of stakeholders, may differ. CONCLUSIONS: Agreement was obtained on a number of items, which we recommend should be used as a framework to develop useful tools to support decision-making about participation in clinical trials. | Male | Decision Making | Decision Support Techniques | Humans | OK |
| Niek S. | Klazinga | Rudolf B. | Kool | Robbert | Huijsman | BMC medical research methodology | 2010-00-00 2010 | BACKGROUND: The CQ Index for the elderly, a quality-of-care questionnaire administered by conducting interviews, is used to assess clients' experiences in Dutch nursing homes and homes for the elderly. This article describes whether inter-interviewer differences influence the perceived quality of healthcare services reported by residents, the size of this interviewer effect and the influence of the interviewer characteristics on CQ Index dimensions for public reporting. METHODS: Data from 4345 questionnaires was used. Correlations were calculated, reliability analyses were performed, and a multilevel analysis was used to calculate the degree of correlation between two interviewers within one health care institution. Five models were constructed and the Intra Class Correlation (ICC) was calculated. Healthcare institutions were given 1-5 stars on every quality dimensions (1 = worst and 5 = best), adjusted for resident and interviewer characteristics. The effect of these characteristics on the assignment of the stars was investigated. RESULTS: In a multilevel approach, the ICC showed a significant amount of variance on five quality dimensions. Of the interviewer characteristics, only previous interviewing experience, the reason of interviewing and general knowledge of health care had a significant effect on the quality dimensions. Adjusting for interviewer characteristics did not affect the overall star assignment to the institutions regarding 7 of 12 quality dimensions. For the other five dimensions (Shared decision-making, Meals, Professional competency, Autonomy, and Availability of personnel) a minor effect was found. CONCLUSIONS: We have shown that training, the use of experienced interviewers, written instructions, supervision and educational meetings do not automatically prevent interviewer effects. While the results of this study can be used to improve the quality of services provided by these institutions, several CQ index dimensions should be interpreted with caution for external purposes (accountability and transparency). | Male | Humans | Questionnaires | Female | OK |
| Nita | Mazurat |  |  |  |  | Journal of Dental Education | 2012-10-00 Oct 2012 | A shortcoming identified in the dental education literature is the scarcity of patient assessment of the quality of communication between student clinicians and patients. This study, the second in a series, attempts to address this scarcity by testing the communication components deemed critical to patients identified in the first article. Two instruments were tested: the Patient Communication Assessment Instrument (PCAI) and the Student Communication Assessment Instrument (SCAI). Item-to-total correlations and Cronbach's alpha were used to determine internal consistency reliability. Construct validity was examined through principal components factor analysis with varimax rotation using a total of 820 participants (410 patients and 410 students), who completed communication skills questionnaires collected in the 2006-07 school year as part of dental and dental hygiene clinical courses. Each component in the assessment instruments demonstrated internal consistency (alpha range=0.779-0.960). Based on a principal components analysis, six new factors were found to be significantly associated with communication skills: being caring and respectful, sharing information, interacting with team members, tending to comfort, professional relationship-building, and appointment preparation/follow-up. Correlational analysis demonstrated a core of critical instrument items to be considered for future assessment of the quality of communication between student clinicians and patients. Adequate estimates of reliability and validity for the PCAI and SCAI were demonstrated. Further research is needed in other countries and cultures to test and confirm the constructs. | Decision Making | Humans | Patient Participation | Patient Satisfaction | OK |
| Marie-Anne | Durand | France | Légaré | Glyn | Elwyn | Patient Education and Counseling | 2010-02-00 février 2010 | Objective: Our aim was to develop a measurement which enables research into the interdependent nature of clinical encounters. The prime objective was to develop an instrument capable of assessing the extent to which patients have been involved in (shared) decision making from two viewpoints—that of the patient and the clinician. Methods: To develop an initial ‘dyadic OPTION’ instrument, the twelve original third-person items were drafted in passive, first person plural forms. Using this version initially, three rounds of cognitive debriefing interviews were held. These were audio-recorded and analyzed at the end of each round and the results used to revise the dyadic OPTION scale. Results: It was possible to modify the observer OPTION instrument into an instrument for completion by both clinicians and patients after a dyadic interaction. Cognitive debriefing revealed five areas of interpretative difficulty. Each item of the observer OPTION scale underwent modification in order to develop a dyadic version of the scale. Conclusions: The dyadic OPTION scale is acceptable and comprehensible by both clinicians and public respondents. Cognitive debriefing adapted and refined an existing scale and provided confidence that the core constructs of the scale (perceived involvement in decisions making) were understood. Practice implications: Further validation of the dyadic OPTION scale is required prior to its use in research settings. (PsycINFO Database Record (c) 2012 APA, all rights reserved). (journal abstract) | Decision Making | Test Construction | test validity | patients | Doublon |
| Marie-Anne | Durand | France | Légaré | Glyn | Elwyn | Patient Education and Counseling | 2010-02-00 Feb 2010 | OBJECTIVE: Our aim was to develop a measurement which enables research into the interdependent nature of clinical encounters. The prime objective was to develop an instrument capable of assessing the extent to which patients have been involved in (shared) decision making from two viewpoints-that of the patient and the clinician. METHODS: To develop an initial 'dyadic OPTION' instrument, the twelve original third-person items were drafted in passive, first person plural forms. Using this version initially, three rounds of cognitive debriefing interviews were held. These were audio-recorded and analysed at the end of each round and the results used to revise the dyadic OPTION scale. RESULTS: It was possible to modify the observer OPTION instrument into an instrument for completion by both clinicians and patients after a dyadic interaction. Cognitive debriefing revealed five areas of interpretative difficulty. Each item of the observer OPTION scale underwent modification in order to develop a dyadic version of the scale. CONCLUSIONS: The dyadic OPTION scale is acceptable and comprehensible by both clinicians and public respondents. Cognitive debriefing adapted and refined an existing scale and provided confidence that the core constructs of the scale (perceived involvement in decisions making) were understood. PRACTICE IMPLICATIONS: Further validation of the dyadic OPTION scale is required prior to its use in research settings. | Male | Decision Making | Humans | Patient Participation | OK |
| Ikhlaq | Din | Rosemary R. C. | McEachan |  |  | BMJ quality & safety | 2013-07-00 Jul 2013 | BACKGROUND: Tools that proactively identify factors that contribute to accidents have been developed within high-risk industries. Although patients provide feedback on their experience of care in hospitals, there is no existing measure which asks patients to comment on the factors that contribute to patient safety incidents. The aim of the current study was to determine those contributory factors from the Yorkshire Contributory Factors Framework (YCFF) that patients are able to identify in a hospital setting and to use this information to develop a patient measure of safety (PMOS). METHODS: Thirty-three qualitative interviews with a representative sample of patients from six units in a teaching hospital in the north of England were carried out. Patients were asked either to describe their most recent/current hospital experience (unstructured) or were asked to describe their experience in relation to specific contributory factors (structured). Responses were coded using the YCFF. Face validity of the PMOS was tested with 12 patients and 12 health professionals, using a 'think aloud' approach, and appropriate revisions made. The research was supported by two patient representatives. RESULTS: Patients were able to comment on/identify 13 of the 20 contributory factors contained within the YCFF domains. They identified contributory factors relating to communication and individual factors more frequently, and contributory factors relating to team factors, and support from central functions less frequently. In addition, they identified one theme not included in the YCFF: dignity and respect. The draft PMOS showed acceptable face validity. DISCUSSION: Patients are able to identify factors which contribute to the safety of their care. The PMOS provides a way of systematically assessing these and has the potential to help health professionals and healthcare organisations understand and identify, safety concerns from the patients' perspective, and, in doing so, make appropriate service improvements. | Male | Humans | Patient Participation | Patient Satisfaction | OK |
| Lena | Ring | Karin | Kjellgren |  |  | Blood Pressure | 2014-10-00 Oct 2014 | Low adherence remains a struggle in hypertension management, despite improvement efforts. Presuming that increased patient participation is a possible approach, we collaborated with patients and healthcare professionals to design a self-report system to support self-management. The study aimed to explore and describe relevant aspects of hypertension and hypertension treatment, for use in the development of an interactive mobile phone self-report system. It further aimed to suggest which clinical measures, lifestyle measures, symptoms and side-effects of treatment would be meaningful to include in such a system. Five focus group interviews were performed with 15 patients and 12 healthcare professionals, and data was analysed using thematic analysis. Patients suggested trust, a good relationship with caregivers, and well-being as important aspects of hypertension self-management. Furthermore, they regarded blood pressure, dizziness, stress, headache and tiredness as important outcomes to include. Patients sought to understand interconnections between symptoms and variations in blood pressure, whilst healthcare professionals doubted patients' ability to do so. Healthcare professionals emphasized accessibility, clear and consistent counselling, complication prevention and educational efforts. The study presents aspects of importance for follow-up to understand the interplay between blood pressure and daily life experiences for patients with hypertension. | | | | | OK |
| Stuart W. | Grande | Rachel | Thompson | Thom | Walsh | Patient Education and Counseling | 2013-10-00 octobre 2013 | Objective: Measuring the process of shared decision making is a challenge, which constitutes a barrier to research and implementation. The aim of the study was to report the development of CollaboRATE, brief patient-reported measure of shared decision making. Methods: We used the following stages: (1) item formulation; (2) cognitive interviews; (3) item refinement; and (4) pilot testing of final items. Participants were over 18 years old, recruited from the public areas of the Dartmouth-Hitchcock Medical Center. Results: The key finding of this study is that developing a brief patient-reported measure of shared decision making requires a move away from terms such as ‘decisions’, ‘options’ and ‘preferences’. Although technically correct, these terms act as barriers. They are often unfamiliar, and they also implicitly assume that patients are willing to take active roles in decision making; whereas patients are often unaware that decisions are required, or have taken place, never mind feel that they could or should have participated in them. Conclusion: These methods have allowed us to develop a brief, patient-reported measure of shared decision making that is highly accessible to intended users. Practice implications: The potential strength of the CollaboRATE will be the ability for completion in less than 30 s, and across a range of routine settings. (PsycINFO Database Record (c) 2014 APA, all rights reserved). (journal abstract) | Shared decision making | Decision Making | Psychometrics | Test Construction | Doublon |
| Stuart W. | Grande | Rachel | Thompson | Thom | Walsh | Patient Education and Counseling | 2013-10-00 Oct 2013 | OBJECTIVE: Measuring the process of shared decision making is a challenge, which constitutes a barrier to research and implementation. The aim of the study was to report the development of CollaboRATE, brief patient-reported measure of shared decision making. METHODS: We used the following stages: (1) item formulation; (2) cognitive interviews; (3) item refinement; and (4) pilot testing of final items. Participants were over 18 years old, recruited from the public areas of the Dartmouth-Hitchcock Medical Center. RESULTS: The key finding of this study is that developing a brief patient-reported measure of shared decision making requires a move away from terms such as 'decisions', 'options' and 'preferences'. Although technically correct, these terms act as barriers. They are often unfamiliar, and they also implicitly assume that patients are willing to take active roles in decision making; whereas patients are often unaware that decisions are required, or have taken place, never mind feel that they could or should have participated in them. CONCLUSION: These methods have allowed us to develop a brief, patient-reported measure of shared decision making that is highly accessible to intended users. PRACTICE IMPLICATIONS: The potential strength of the CollaboRATE will be the ability for completion in less than 30s, and across a range of routine settings. | Time Factors | Male | Decision Making | Decision Support Techniques | OK |
| Nita | Mazurat |  |  |  |  | Journal of Dental Education | 2011-12-00 Dec 2011 | Effectively using patients as teachers to provide authentic feedback is an underused strategy in dental education, but it has potential for integrating the teaching of therapeutic communication skills within the dental clinic setting. This study focuses on the absence of patient input into the design of instruments used to assess students' clinical communication skills and demonstrates how a holistic approach, with input from key stakeholders including patients, was used to produce two such instruments. The development of complementary communication assessment instruments, one for patient use and one for student use, took place in three phases. In Phase I the authors reviewed a sample of existing patient satisfaction surveys; in Phase II they captured input from stakeholders; and Phase III resulted in the generation of the patient communication assessment instrument and the student communication self-assessment instrument. This article highlights communication skill issues relevant to the education of oral health professionals and describes the rationale and process for the development of the first iteration of the patient assessment and student self-assessment clinical communication instruments. | Male | Humans | Patient Participation | Patient Satisfaction | OK |
|  |  |  |  |  |  | International journal of STD & AIDS | 2011-10-00 Oct 2011 | Local service quality indicators (SQIs) for both genitourinary (GU) medicine and HIV were drawn up using patient and public involvement (PPI). Our clinic's performance was audited against these SQIs. All measures were achieved, almost achieved or probably achieved. SQIs need to be challenging yet achievable and it is difficult to judge precisely where standards should be set. This paper provides a first estimate for our service after PPI. We hope other services may wish to audit performance against these SQIs. If such data were to be pooled, there would be valuable evidence for setting 'SMART' (specific, measurable, attainable, relevant and time-based) SQIs that are challenging yet achievable for the majority of clinics. If the bar were to be set correctly, the actual performance of clinics ought generally to lie just above or just below the set standard. | Male | Humans | Patient Participation | Young Adult | OK |
|  |  |  |  |  |  | Health and Quality of Life Outcomes | 2013-00-00 2013 | BACKGROUND: Amblyopia is a visual condition that occurs in childhood. Screening programmes exist within the United Kingdom (UK) to detect amblyopia, and once detected treatment is given.Existing patient reported outcome (PRO) measures for amblyopia do not meet current recommendations for the methods adopted during their development, or the way in which the instruments are administered. The overall aim of this study was to produce a self-complete PRO instrument for amblyopia for children aged 4-7 years that uses children's responses in the development phase. The study comprised a number of stages. This paper reports on the refinement of the descriptive system for the draft instrument (the Child Amblyopia Treatment Questionnaire, CAT-QoL) using qualitative and quantitative methods. METHODS: The study consisted of three components. Children were asked to read, and complete the draft questionnaire as independently as possible. They were then asked about the questionnaire, and its format, in a cognitive debriefing exercise. Observations were made as to the child's ability to read the questionnaire, particular attention was made as to which individual words participants struggled to read. Children were also asked their opinion on the design layout of the questionnaire. Finally, some children were asked to complete a ranking task to help determine the order of the levels of the items as judged by the children. Mid-rank scoring and statistical level of agreement were calculated for the ranking exercise. RESULTS: Thirty-two (n=32) participants completed a draft questionnaire; each of these underwent a cognitive de-briefing interview. Twenty-two (n=22) children completed the ranking exercise. Ten children did not understand the concept of ranking. The results of the qualitative phase (cognitive de-briefing interview) were used to modify the wording of items and layout of the instrument. Results of the ranking exercise were used to inform the order of the response levels for the items. CONCLUSION: Responses of young children can be used in the development of PRO instruments. They are able to help inform the content, wording, and format of an instrument, ensuring good content and face validity. The results have been used to further refine the CAT-QoL, however further research is required to assess the psychometric properties of the instrument. | Male | Humans | Patient Participation | Questionnaires | OK |
| William | Haslett | Rajesh | Munusamy |  |  | Psychiatric Rehabilitation Journal | 2010-00-00 Sum 2010 | Topic: This article discusses the development of Web-based online decision support tools intended for researchers examining the issue of shared decision making for the population of individuals with serious mental illnesses. Purpose: The authors describe the background and use of decision support tools to facilitate shared decision-making between individuals diagnosed with a psychiatric condition and clinicians and describes a novel software platform that allows researchers and other system designers to build decision support systems. Conclusions: In supporting ongoing research efforts, an online decision support tool appears to be useful for individuals facing preference-sensitive decisions and an online designer tool allows for rapid deployment of these research sites to support ongoing research efforts in shared decision making. (PsycINFO Database Record (c) 2013 APA, all rights reserved). (journal abstract) | Shared decision making | Decision Making | Therapeutic Processes | Mental Disorders | Doublon |
| William | Haslett | Rajesh | Munusamy |  |  | Psychiatric Rehabilitation Journal | 2010-00-00 2010 | TOPIC: This article discusses the development of Web-based online decision support tools intended for researchers examining the issue of shared decision making for the population of individuals with serious mental illnesses. PURPOSE: The authors describe the background and use of decision support tools to facilitate shared decision-making between individuals diagnosed with a psychiatric condition and clinicians and describes a novel software platform that allows researchers and other system designers to build decision support systems. CONCLUSIONS: In supporting ongoing research efforts, an online decision support tool appears to be useful for individuals facing preference-sensitive decisions and an online designer tool allows for rapid deployment of these research sites to support ongoing research efforts in shared decision making. | Decision Making | Decision Support Techniques | Humans | Patient Participation | OK |
|  |  |  |  |  |  |  | 2014-00-00 2014 | Patient decision support refers to the provision of appropriate information to a patient about his or her health conditions to aid in the goal of informed medical decision-making. Medical decisions in which there are multiple valid options in which the risks and benefits differ, deemed "preference-sensitive" decisions, necessitate shared decision-making and patient decision support. Tailoring and targeting can be used to adapt the content or the amount of information delivered to the patient. Tailored or targeted health information for patient decision support has long been shown to be more effective than generic information at modifying health behaviors. To increase adoption in clinical care, one goal in developing targeting frameworks is to choose theory constructs and personal characteristics measured by a few data items that are simple to collect. Thus, in developing parsimonious algorithms, it is important to understand the relationships between socio-demographic characteristics and theories of health behavior. Finally, to ensure acceptance of algorithms in patient decision support, it is critical to understand patient perceptions of such algorithms, particularly what additional factors are important in decision-making. This dissertation addresses these needs in two parts: Part 1) development of algorithms to guide content and intensity of patient decision support in the context of colorectal cancer screening, and Part 2) assessment of patient perceptions of the use of algorithms to guide patient decision support in the context of heart disease prevention. In the Part 1 of this dissertation, I conducted two studies that consider health beliefs, demographics, and patient behavior in the context of colorectal cancer screening behavior. The first study sought to understand the relationship between socio-demographic characteristics and constructs of the health belief model in the context of colorectal cancer screening behavior in a racially, ethnically, and linguistically diverse population. The second study sought to develop a practical regression model to predict the probability of completing colorectal cancer screening and, from this model, a framework for targeting patient decision support materials based on level of probability of completing CRC screening. In Part 2 of this dissertation, I conducted one study to assess the use of an algorithm in patient decision support for heart disease prevention therapy. This study was a qualitative analysis of interviews conducted after a discrete choice experiment (DCE) for heart disease prevention. I sought to understand new factors that were influencing heart disease prevention therapy choices and perception of DCE-based "values concordant" choice results. In the first part of the dissertation, I observed several key differences across racial/ethnic and language groups in terms of health beliefs about CRC and CRC screening. These differences were largely dependent upon primary spoken language, which may approximate level of acculturation. Non-English speaking Hispanics typically reported lower perceived susceptibility to CRC than non-Hispanic Whites, and higher perception of several barriers to CRC screening (prior testing experience, preparation for the test, need for sedation). Non-English speaking Asians also reported lower perceived susceptibility than non-Hispanic Whites, but lower perception of several barriers to CRC screening (need for additional testing, fear of results of the test, concern about complications from the test, need for sedation, anxiety about the procedure). These results may suggest topic areas that could be highlighted in CRC screening promotion interventions that are targeted at specific racial/ethnic and language groups. In the second study, I developed a simple model for predicting CRC screening completion. From that model I developed an intervention framework that may be useful for targeting the amount… (PsycINFO Database Record (c) 2014 APA, all rights reserved) | Decision Making | Health Behavior | Cancer Screening | Medical decisions | OK |
| Hou Heng | Su | Dai | Lie | Janet | Dinnella | Journal of Clinical Rheumatology: Practical Reports on Rheumatic & Musculoskeletal Diseases | 2011-08-00 Aug 2011 | OBJECTIVE: The objective of the study was to develop and test a survey of gout patients regarding their level of disease-related knowledge, to identify potential targets for patient education. METHODS: A 10-item questionnaire with readability at a Flesch-Kincaid grade level of 4.6 and Flesch reading ease of 83.9% was designed to address parameters considered important for patient participation in the management of gout. The questionnaire was primarily evaluated at the Veterans Affairs (VA) Medical Center in Philadelphia, but was also secondarily performed at 2 Chinese hospitals, the Sun Yat-sen Memorial Hospital of Zhong Shan University, Guangdong Province (GZ), and the Qingdao Municipal Hospital, Qingdao City, Shandong Province (QD). Demographic and questionnaire data by institution were evaluated using descriptive statistics, and significant differences were identified by χ and Fisher exact tests. Patient responses were displayed by each individual question and by the distribution of total scores. Kruskal-Wallis tests of significance were used for nonparametric or skewed data. Intraclass correlations (ICCs) were performed within the VA population to determine internal consistency of the individual questions. A high score was defined as greater than 7 (the median value). Multivariate regression models using demographic and clinical characteristics attempted to identify factors associated with correct answers to each question. RESULTS: Total correct score for individual patients varied widely at each institution with a mean (SD) and median (interquartile range [IQR]) scores in all 3 hospitals of 6.15 (2.25) and 7 (5-8), respectively. The average numbers of correct responses for each institution were 4.38 (SD, 3.04) (median, 4 [IQR, 2-7]) at GZ; 7.05 (SD, 1.37) (median, 8 [IQR, 6-8]) at QD; 6.21 (SD, 1.74) (median, 7 [IQR, 6-7]) at VA; P = 0.0010. Two questions (Q4 and Q10) were identified as difficult to understand by patients and showed poor ICC (ICC = 0.0000, P > 0.5) at the VA. Questions that were more difficult to answer were (1) Q3: What inside the joint causes attacks of gout? (GZ, 28.6%; QD, 7.7%; VA, 72.4%; P = 0.000); (2) Q8: How long should patients continue with serum uric acid-lowering drugs? (GZ, 19.1%; QD, 10.3%; VA, 82.7%; P = 0.000); (3) Q6: The ideal serum uric acid to aim at during treatment? (GZ, 42.8%; QD, 89.7%; and VA, 17.2%; P = 0.000); and (4) Q5: Which drugs can lower serum uric acid? (GZ, 61.9%; QD, 89.7%; VA, 51.7%; P = 0.002). CONCLUSIONS: This study describes an easy-to-read 10-item questionnaire that can identify important knowledge gaps in patients with gout. This can be the first step in designing educational interventions to improve patient understanding and improve clinical care. | Male | Humans | Patient Participation | Questionnaires | OK |
|  |  |  |  |  |  | Journal of Korean Academy of Nursing | 2012-08-00 Aug 2012 | PURPOSE: The study was done to develop a shared decision-making scale for end-of-life patients in Korea. METHODS: The process included construction of a conceptual framework, generation of initial items, verification of content validity, selection of secondary items, preliminary study, and extraction of final items. The participants were 388 adults who lived in one of 3 Korean metropolitan cities: Seoul, Daegu, or Busan. Item analysis, factor analysis, criterion related validity, and internal consistency were used to analyze the data. Data collection was done from July to October 2011. RESULTS: Thirty-four items were selected for the final scale, and categorized into 7 factors explaining 61.9% of the total variance. The factors were labeled as sharing information (9 items), constructing system (7 items), explanation as a duty (5 items), autonomy (4 items), capturing time (3 items), participation of family (3 items), and human respect (3 items). The scores for the scale were significantly correlated among shared decision-making scale, terminating life support scale, and dignified dying scale. Cronbach's alpha coefficient for the 34 items was .94. CONCLUSION: The above findings indicate that the shared decision-making scale has a good validity and reliability when used for end-of-life patients in Korea. | Male | Decision Making | Humans | Female | OK |
| Jennifer L. | Paruch | Lynn | Zhou | Thomas E. | Kmiecik | Journal of the American College of Surgeons | 2013-11-00 Nov 2013 | BACKGROUND: Accurately estimating surgical risks is critical for shared decision making and informed consent. The Centers for Medicare and Medicaid Services may soon put forth a measure requiring surgeons to provide patients with patient-specific, empirically derived estimates of postoperative complications. Our objectives were to develop a universal surgical risk estimation tool, to compare performance of the universal vs previous procedure-specific surgical risk calculators, and to allow surgeons to empirically adjust the estimates of risk. STUDY DESIGN: Using standardized clinical data from 393 ACS NSQIP hospitals, a web-based tool was developed to allow surgeons to easily enter 21 preoperative factors (demographics, comorbidities, procedure). Regression models were developed to predict 8 outcomes based on the preoperative risk factors. The universal model was compared with procedure-specific models. To incorporate surgeon input, a subjective surgeon adjustment score, allowing risk estimates to vary within the estimate's confidence interval, was introduced and tested with 80 surgeons using 10 case scenarios. RESULTS: Based on 1,414,006 patients encompassing 1,557 unique CPT codes, a universal surgical risk calculator model was developed that had excellent performance for mortality (c-statistic = 0.944; Brier score = 0.011 [where scores approaching 0 are better]), morbidity (c-statistic = 0.816, Brier score = 0.069), and 6 additional complications (c-statistics > 0.8). Predictions were similarly robust for the universal calculator vs procedure-specific calculators (eg, colorectal). Surgeons demonstrated considerable agreement on the case scenario scoring (80% to 100% agreement), suggesting reliable score assignment between surgeons. CONCLUSIONS: The ACS NSQIP surgical risk calculator is a decision-support tool based on reliable multi-institutional clinical data, which can be used to estimate the risks of most operations. The ACS NSQIP surgical risk calculator will allow clinicians and patients to make decisions using empirically derived, patient-specific postoperative risks. | Male | Decision Support Techniques | Humans | United States | Doublon |
| Jennifer L. | Paruch | Lynn | Zhou | Thomas E. | Kmiecik | Journal of the American College of Surgeons | 2013-11-00 Nov 2013 | BACKGROUND: Accurately estimating surgical risks is critical for shared decision making and informed consent. The Centers for Medicare and Medicaid Services may soon put forth a measure requiring surgeons to provide patients with patient-specific, empirically derived estimates of postoperative complications. Our objectives were to develop a universal surgical risk estimation tool, to compare performance of the universal vs previous procedure-specific surgical risk calculators, and to allow surgeons to empirically adjust the estimates of risk. STUDY DESIGN: Using standardized clinical data from 393 ACS NSQIP hospitals, a web-based tool was developed to allow surgeons to easily enter 21 preoperative factors (demographics, comorbidities, procedure). Regression models were developed to predict 8 outcomes based on the preoperative risk factors. The universal model was compared with procedure-specific models. To incorporate surgeon input, a subjective surgeon adjustment score, allowing risk estimates to vary within the estimate's confidence interval, was introduced and tested with 80 surgeons using 10 case scenarios. RESULTS: Based on 1,414,006 patients encompassing 1,557 unique CPT codes, a universal surgical risk calculator model was developed that had excellent performance for mortality (c-statistic = 0.944; Brier score = 0.011 [where scores approaching 0 are better]), morbidity (c-statistic = 0.816, Brier score = 0.069), and 6 additional complications (c-statistics > 0.8). Predictions were similarly robust for the universal calculator vs procedure-specific calculators (eg, colorectal). Surgeons demonstrated considerable agreement on the case scenario scoring (80% to 100% agreement), suggesting reliable score assignment between surgeons. CONCLUSIONS: The ACS NSQIP surgical risk calculator is a decision-support tool based on reliable multi-institutional clinical data, which can be used to estimate the risks of most operations. The ACS NSQIP surgical risk calculator will allow clinicians and patients to make decisions using empirically derived, patient-specific postoperative risks. | Male | Decision Support Techniques | Humans | United States | OK |
| M. | Miyakubo | Y. | Sekine | Y. | Tamura | Prostate Cancer and Prostatic Diseases | 2012-06-00 Jun 2012 | BACKGROUND: We previously reported the diagnostic efficacy of the age- and prostate volume-adjusted prostate biopsy method (the adjusted biopsy method). Here, we developed a new nomogram for predicting cancer probability at initial biopsy using the adjusted method. METHODS: Between 2002 and 2010, 1059 Japanese men with PSA levels between 1.1 and 40 ng ml(-1) and biopsied for the first time using the adjusted method at Gunma University Hospital were enrolled. All subjects underwent digital rectal examination (DRE) and transrectal ultrasonography (TRUS). Data from the initial 849 subjects were used for development of the nomogram and those from the final 210 subjects were used for internal validation. External validation was conducted using data from two affiliated hospitals where the same adjusted biopsy method was used. The nomogram was developed through logistic regression analysis, and predictive accuracy and performance characteristics were assessed using the area under the curve (AUC) of the receiver operating characteristics and calibration plots. Furthermore, we compared the predictive accuracy of the newly developed nomogram with the 'Prostate Risk Indicator' using the development data set, as well as the two external data sets. RESULTS: The AUC of the logistic regression-based nomogram was significantly higher than those of any single clinical parameter. External validation showed significant correlations with the present model. The AUC-receiver operating characteristic of the 'Prostate Risk Indicator' was the second largest following the new nomogram using the development data set and one external data set and almost equal to the new nomogram using the other external data set. CONCLUSIONS: Nevertheless the present model does not include somewhat subjective findings on TRUS abnormality, which is necessary for the estimation by 'Prostate Risk Indicator', the predictive accuracy of the present simple nomogram could be excellent enough to contribute to accurate shared decision-making between doctors and men who are candidates for the adjusted biopsy scheme. | Male | Humans | Aged | Middle Aged | OK |
| Dawn | Stacey | Jeff S. | Healey | Gina | Browne | Trials | 2013-00-00 2013 | BACKGROUND: Patients, identified to be at risk for but who have never experienced a potentially lethal cardiac arrhythmia, have the option of receiving an implantable cardioverter defibrillator (ICD) as prophylaxis against sudden cardiac death - a primary prevention indication. In Canada, there is no clear framework to support patients' decision-making for these devices. Decision support, using a decision aid, could moderate treatment-related uncertainty and prepare patients to make well-informed decisions. Patient decision aids provide information on treatment options, risks, and benefits, to help patients clarify their values for outcomes of treatment options. The objectives of this research are: 1) develop a decision aid, 2) evaluate the decision aid, and 3) determine the feasibility of conducting a trial. METHODS/DESIGN: A development panel comprised of the core investigative team, health service researchers, decision science experts, cardiovascular healthcare practitioners, and ICD patient representatives will collaborate to provide input on the content and format of the aid. To generate probabilities to include in the aid, we will synthesize primary prevention ICD evidence. To obtain anonymous input about the facts and content, we will employ a modified Delphi process. To evaluate the draft decision aid will invite ICD patients and their families (n = 30) to rate its acceptability. After we evaluate the aid, to determine the feasibility, we will conduct a feasibility pilot randomized controlled trial (RCT) in new ICD candidates (n = 80). Participants will be randomized to receive a decision aid prior to specialist consultation versus usual care. Results from the pilot RCT will determine the feasibility of research processes; inform sample size calculation, measure decision quality (knowledge, values, decision conflict) and the influence of health related quality of life on decision-making. DISCUSSION: Our study seeks to develop a decision aid, for patients offered their first ICD for prophylaxis against sudden cardiac death. This paper outlines the background and methods of a pilot randomized trial which will inform a larger multicenter trial. Ultimately, decision support prior to specialist consultation could enhance the decision-making process between patients, physicians, and families, associated with life-prolonging medical devices like the ICD. TRIAL REGISTRATION: ClinicalTrials.gov: NCT01876173. | Decision Support Techniques | Humans | Patient Participation | Physician-Patient Relations | OK |
| Ann J. | Russ |  |  |  |  | BMC medical informatics and decision making | 2013-00-00 2013 | BACKGROUND: For many healthcare decisions, multiple alternatives are available with different combinations of advantages and disadvantages across several important dimensions. The complexity of current healthcare decisions thus presents a significant barrier to informed decision making, a key element of patient-centered care.Interactive decision dashboards were developed to facilitate decision making in Management, a field marked by similarly complicated choices. These dashboards utilize data visualization techniques to reduce the cognitive effort needed to evaluate decision alternatives and a non-linear flow of information that enables users to review information in a self-directed fashion. Theoretically, both of these features should facilitate informed decision making by increasing user engagement with and understanding of the decision at hand. We sought to determine if the interactive decision dashboard format can be successfully adapted to create a clinically realistic prototype patient decision aid suitable for further evaluation and refinement. METHODS: We created a computerized, interactive clinical decision dashboard and performed a pilot test of its clinical feasibility and acceptability using a multi-method analysis. The dashboard summarized information about the effectiveness, risks of side effects and drug-drug interactions, out-of-pocket costs, and ease of use of nine analgesic treatment options for knee osteoarthritis. Outcome evaluations included observations of how study participants utilized the dashboard, questionnaires to assess usability, acceptability, and decisional conflict, and an open-ended qualitative analysis. RESULTS: The study sample consisted of 25 volunteers - 7 men and 18 women - with an average age of 51 years. The mean time spent interacting with the dashboard was 4.6 minutes. Mean evaluation scores on scales ranging from 1 (low) to 7 (high) were: mechanical ease of use 6.1, cognitive ease of use 6.2, emotional difficulty 2.7, decision-aiding effectiveness 5.9, clarification of values 6.5, reduction in decisional uncertainty 6.1, and provision of decision-related information 6.0. Qualitative findings were similarly positive. CONCLUSIONS: Interactive decision dashboards can be adapted for clinical use and have the potential to foster informed decision making. Additional research is warranted to more rigorously test the effectiveness and efficiency of patient decision dashboards for supporting informed decision making and other aspects of patient-centered care, including shared decision making. | Time Factors | Male | Decision Support Techniques | Humans | OK |
| Tal | Jarus | Orit | Bart |  |  | Research in Developmental Disabilities | 2010-12-00 2010 Nov-Dec | The purpose of this manuscript was to develop and test the psychometric properties of the Environmental Restriction Questionnaire (ERQ) a parent-reported questionnaire for measuring perceived environmental restrictions for young children participation. Reliability and homogeneity were tested by Cronbach's alpha and inter-item correlations. Construct validity was computed by factor analysis and known group differences analysis. Convergent and divergent validities were calculated by correlation with the Children Participation Questionnaire (CPQ). Participants were 290 children and their parent. Seventy-five children who were referred to occupational therapy evaluation as consequence of moderate developmental disabilities and 215 children without any disability (mean age ± standard deviation for total sample, 5 y, 3 mo ± .65 y; range, 3 y, 11 mo to 6 y, 10 mo). The ERQ has good internal reliability. Cronbach's alpha for the ERQ measures ranged between .75 and .91, indicating adequate homogeneity. Factor analysis yielded three factors that explained almost 48% of the total variance. Significant differences were found between known groups. Convergent and divergent validity were supported by various correlations with the Children Participation Questionnaire (CPQ). The ERQ has demonstrated good psychometric properties and can be used as a reliable and valid measure to assess perceived environmental restriction at the age of 4-6 y. | Male | Humans | Patient Participation | Questionnaires | OK |
| P. | Yates |  |  |  |  | European Journal of Cancer Care | 2012-07-00 Jul 2012 | The objective of this research was to develop a question prompt list aimed at increasing question asking and reducing the unmet information needs of adults with primary brain tumours, and to pilot the question prompt list to determine its suitability for the intended population. Thematic analysis of existing resources was used to create a draft which was refined via interviews with 12 brain tumour patients and six relatives, readability testing and review by health professionals. A non-randomised before-after pilot study with 20 brain tumour patients was used to assess the acceptability and usefulness of the question prompt list, compared with a 'standard brochure', and the feasibility of evaluation strategies. The question prompt list developed covered seven main topics (diagnosis, prognosis, symptoms and changes, treatment, support, after treatment finishes and the health professional team). Pilot study participants provided with the question prompt list agreed that it was helpful (7/7), contained questions that were useful to them (7/7) and prompted them to ask their medical oncologist questions (5/7). The question prompt list is acceptable to patients and contains questions relevant to them. Research is now needed to assess its effectiveness in increasing question asking and reducing unmet information needs. | Male | Humans | Patient Participation | Patient Satisfaction | OK |
| Miranda C. M. | Chan | Amy Y. M. | Or | Ava | Kwong | Health Expectations: An International Journal of Public Participation in Health Care and Health Policy | 2011-12-00 Dec 2011 | BACKGROUND: Women choosing breast cancer surgery encounter treatment decision-making (TDM) difficulties, which can cause psychological distress. Decision Aids (DAs) may facilitate TDM, but there are no DAs designed for Chinese populations. We developed a DA for Chinese women newly diagnosed with breast cancer, for use during the initial surgical consultation. AIMS: Conduct a pilot study to assess the DA acceptability and utility among Chinese women diagnosed with breast cancer. METHODS: Women preferred the DA in booklet format. A booklet was developed and revised and evaluated in two consecutive pilot studies (P1 and P2). On concluding their initial diagnostic consultation, 95 and 38 Chinese women newly diagnosed with breast cancer received the draft and revised draft DA booklet, respectively. Four-day post-consultation, women had questionnaires read out to them and to which they responded assessing attitudes towards the DA and their understanding of treatment options. RESULTS: The original DA was read/partially read by 66/22% (n = 84) of women, whilst the revised version was read/partially read by 74/16% (n = 35), including subliterate women (χ(2) = 0.76, P = 0.679). Knowledge scores varied with the extent the booklet was read (P1: F = 12.68, d.f. 2, P < 0.001; P2: F = 3.744, d.f. 2, P = 0.034). The revised, shorter version was graphically rich and resulted in improved perceived utility, [except for the 'treatment options' (χ(2) = 5.50, P = 0.019) and 'TDM guidance' (χ(2) = 8.19, P = 0.004) sections] without increasing anxiety (F = 0.689, P = 0.408; F = 3.45, P = 0.073). CONCLUSION: The DA was perceived as acceptable and useful for most women. The DA effectiveness is currently being evaluated using a randomized controlled trial. | Decision Making | Humans | Patient Participation | Questionnaires | OK |
| Michael T. | French | Karen L. | Dugosh | David S. | Festinger | Journal of Substance Abuse Treatment | 2012-07-00 Jul 2012 | Financial incentives are often used in research, yet no measure exists to determine whether they lead to perceptions of coercion in subjects. We present a preliminary evaluation of a recently developed Financial Incentive Coercion Assessment (FICA) questionnaire. FICA measures perceived coercion specifically related to payment for participation in a research study. Two hundred sixty-six subjects were recruited from a large randomized controlled trial; 152 returned for a 6-month follow-up and completed the FICA. Approximately 30% of participants reported the major reason for participating was "for the money," but less than 5% felt that the financial incentives were coercive. FICA results are consistent with levels of perceived coercion using an alternative measure. Initial assessment of responses on the FICA suggests that it may provide a novel approach to measuring perceived coercion from financial incentives in research. Future work will refine the FICA and analyze its psychometric properties. | Male | Humans | Patient Participation | Questionnaires | OK |
| Harriet | Jordan | Mike | Slade | Andrea | Fiorillo | BMC psychiatry | 2013-00-00 2013 | BACKGROUND: The aim of this study was to develop and evaluate psychometric properties of the Clinical Decision Making Style (CDMS) scale which measures general preferences for decision making as well as preferences regarding the provision of information to the patient from the perspectives of people with severe mental illness and staff. METHODS: A participatory approach was chosen for instrument development which followed 10 sequential steps proposed in a current guideline of good practice for the translation and cultural adaptation of measures. Following item analysis, reliability, validity, and long-term stability of the CDMS were examined using Spearman correlations in a sample of 588 people with severe mental illness and 213 mental health professionals in 6 European countries (Germany, UK, Italy, Denmark, Hungary, and Switzerland). RESULTS: In both patient and staff versions, the two CDMS subscales "Participation in Decision Making" and "Information" reliably measure distinct characteristics of decision making. Validity could be demonstrated to some extent, but needs further investigation. CONCLUSIONS: Together with two other five-language patient- and staff-rated measures developed in the CEDAR study (ISRCTN75841675) - "Clinical Decision Making in Routine Care" and "Clinical Decision Making Involvement and Satisfaction" - the CDMS allows empirical investigation of the complex relation between clinical decision making and outcome in the treatment of people with severe mental illness across Europe. | Male | Decision Making | Humans | Patient Participation | OK |
|  |  |  |  |  |  | BMC health services research | 2013-00-00 2013 | BACKGROUND: Effective patient-centred health care requires internal participation, which is defined as interprofessional patient-centred teamwork. Many scales are designed for measuring teamwork from the perspective of one type of health care professional (e.g. physician or nurse), rather than for the use for all health care professionals as well as patients. Hence, this paper's purpose is to develop a scale for measuring internal participation from all relevant perspectives and to check its psychometric properties. METHODS: In a multicentre cross-sectional study, a 6-item Internal Participation Scale (IPS) was developed and administered to 661 health care professionals (staff) and 1419 patients in 15 rehabilitation clinics to test item characteristics, acceptance, reliability (internal consistency) and construct validity. Additionally, we performed an exploratory factor analysis (EFA) to determine the factorial structure and explained variance. Confirmatory factor analysis (CFA) was used to verify the theoretically assumed one-dimensional factorial structure. RESULTS: A total of 275 health care professionals and 662 patients participated, and the complete data sets of 272 staff members and 536 patients were included in the final analysis. The discrimination index was above .4 for all items in both samples. Internal consistency was very good, with Cronbach's alpha equalling .87 for the staff and .88 for the patient sample. EFA supported a one-dimensional structure of the instrument (explained variance: 61.1% (staff) and 62.3% (patients)). CFA verified the factorial structure, with the factor loadings exceeding .4 for five of six items in both samples. Global goodness-of-fit indices indicated a good model fit, with a Tucker-Lewis index (TLI) of .974 (staff) and .976 (patients) and a comparative fit index (CFI) of .988 (staff) and .989 (patients). The root mean square error of approximation (RMSEA) amounted to .068 for the patient sample and .069 for the staff sample. There is evidence of construct validity for both populations. CONCLUSIONS: The analysis of the scale's psychometric properties resulted in good values. The scale is a promising instrument to assess internal participation from the perspective of both patients and staff. Further research should investigate the scale's psychometric properties in other interprofessional health care settings to examine its generalizability as well as its sensitivity to change. | Male | Decision Making | Humans | Patient Participation | OK |
| Jörg | Dirmaier | Angela | Buchholz | Martin | Härter | Patient Education and Counseling | 2012-08-00 Aug 2012 | OBJECTIVE: To develop and psychometrically test a brief instrument for assessing the physician's perspective of the shared decision-making process in clinical encounters. METHODS: We adapted the 9-item Shared Decision Making Questionnaire (SDM-Q-9) for patients to generate a new version for physicians (SDM-Q-Doc). The physician version was tested in clinical encounters between 29 physicians and 324 patients in German outpatient care contexts. Analyses of the extent to which the instrument was accepted, the reliability of the instrument, and the factorial structure of the scale were performed. RESULTS: Physicians showed a high level of acceptance toward the SDM-Q-Doc. Item discrimination parameters were above .4 for all but one item. An analysis of internal consistency yielded a Cronbach's α of .88. Factor analysis confirmed a one-dimensional structure. CONCLUSION: The results of this study suggest that the SDM-Q-Doc is a well-accepted and reliable instrument for assessing the physician's perspective during SDM processes in clinical encounters. To our knowledge, the SDM-Q-Doc is the first psychometrically tested scale available for assessing the physician's perspective. PRACTICE IMPLICATIONS: The SDM-Q-Doc can be used in studies that analyze the effectiveness of the implementation of SDM and as a quality indicator in quality assurance programs and health service assessments. | Male | Decision Making | Humans | Patient Participation | OK |
| Jörg | Dirmaier | Angela | Buchholz | Martin | Härter | Patient Education and Counseling | 2012-08-00 août 2012 | Objective: To develop and psychometrically test a brief instrument for assessing the physician’s perspective of the shared decision-making process in clinical encounters. Methods: We adapted the 9-item Shared Decision Making Questionnaire (SDM-Q-9) for patients to generate a new version for physicians (SDM-Q-Doc). The physician version was tested in clinical encounters between 29 physicians and 324 patients in German outpatient care contexts. Analyses of the extent to which the instrument was accepted, the reliability of the instrument, and the factorial structure of the scale were performed. Results: Physicians showed a high level of acceptance toward the SDM-Q-Doc. Item discrimination parameters were above .4 for all but one item. An analysis of internal consistency yielded a Cronbach’s a of .88. Factor analysis confirmed a one-dimensional structure. Conclusion: The results of this study suggest that the SDM-Q-Doc is a well-accepted and reliable instrument for assessing the physician’s perspective during SDM processes in clinical encounters. To our knowledge, the SDM-Q-Doc is the first psychometrically tested scale available for assessing the physician’s perspective. Practice implications: The SDM-Q-Doc can be used in studies that analyze the effectiveness of the implementation of SDM and as a quality indicator in quality assurance programs and health service assessments. (PsycINFO Database Record (c) 2013 APA, all rights reserved). (journal abstract) | Shared decision making | Decision Making | Questionnaires | physicians | Doublon |
|  |  |  |  |  |  | Midwifery | 2012-06-00 Jun 2012 | OBJECTIVE: to develop a scale to measure maternal satisfaction with birth to evaluate women's experiences in labour and the early postpartum period. DESIGN: development and psychometric assessment of a multidimensional maternal satisfaction questionnaire. SETTING: maternity unit of a university hospital in Istanbul. PARTICIPANTS: 500 healthy postpartum women. METHODS: five steps were taken in development of the scale: literature review, generating item pool, content validity testing, administration of draft scale and psychometric testing. Two versions of the scale were developed: the Scale for Measuring Maternal Satisfaction-normal birth and the Scale for Measuring Maternal Satisfaction-caesarean birth. Content validity was evaluated by experts. The appropriate draft scale and the Newcastle Satisfaction with Nursing Scale were administered to postpartum women before hospital discharge. FINDINGS: content validity index scores for the vaginal and caesarean birth scales were 0.91 and 0.89, respectively. Item-total and subscale-total scores correlated significantly for each scale. Evaluation of construct validity through factor analysis yielded 10 subscales: 'perception of health professionals', 'nursing/midwifery care in labour (in caesarean version: preparation for caesarean)', 'comforting', 'information and involvement in decision making', 'meeting baby', 'postpartum care', 'hospital room', 'hospital facilities', 'respect for privacy' and 'meeting expectations'. Both scales had good internal reliability, with Cronbach's α coefficients of 0.91. The scales established their convergent validity with significant correlations with the Newcastle Satisfaction with Nursing Scale. CONCLUSION: the scales are valid and reliable tools for evaluating Turkish women's experiences in labour and the early postpartum period. IMPLICATIONS FOR PRACTICE: the scales can contribute to the assessment of women's satisfaction with different aspects of care, the quality of care and developments in maternity services. | Humans | Patient Participation | Patient Satisfaction | Questionnaires | OK |
| S. M. T. | Nardi | C. H. | van der Zee | N. | Banstola | Disability and Rehabilitation | 2012-00-00 2012 | PURPOSE: To validate a shortened version of the Participation Scale (P-scale) that will be quicker to use and to describe the factor structure found in the P-scale data in various study samples. METHODS: A large multi-country and multi-cultural database was compiled consisting of 5125 respondents. Item analysis, explanatory factor analysis and confirmatory factor analysis were applied to identify items for deletion and investigate the factor structure of the P-scale. RESULTS: The multi-country database included 11 databases from six different countries. Respondents were affected by a range of health conditions, including leprosy, HIV/AIDS, dermatological conditions and various disabilities. Of the respondents included 57% were male. The P-scale Short (PSS) contains 13 items. A two-factor structure, with factors named "work-related participation" (three items) and "general participation" (10 items), showed the best model fit (Comparative Fit Index = 0.983, Tucker Lewis Index = 0.979, Rooted Mean Square Error of Approximation = 0.061). The Cronbach's alphas were very good for both the whole scale and the subscales, 0.91, 0.83 and 0.90, respectively. Correlation between the two factors was high (r = 0.75) indicating that interpreting the P-scale as measuring an overall factor "participation" is still valid. A very high correlation (r = 0.99) was found between the full P-scale and the PSS. CONCLUSIONS: The findings suggest good validity of the P-scale across a range of languages and cultures. However, field testing needs to confirm the validity of the PSS to measure the level of social participation restrictions across cultures and health conditions. | Male | Humans | Patient Participation | Questionnaires | OK |
| Marion | Bizé | Barbara | Koloshuk | Joël | Ladner | Population Health Management | 2013-04-00 avril 2013 | The objective was to develop and validate a multilateral index to determine patient ability to pay for medication in low- and middle-income countries. Primary data were collected in 2009 from 117 cancer patients in China, India, Thailand, and Malaysia. The initial tool included income, expenditures, and assets-based items using ad hoc determined brackets. Principal components analysis was performed to determine final weights. Agreement (Kappa) was measured between results from the final tool and from an Impact Survey (IS) conducted after beginning drug therapy to quantify a patient’s actual ability to pay in terms of number of drug cycles per year. The authors present the step-by-step methodology employed to develop the tool on a country-by-country basis. Overall Cronbach value was 0.84. Agreement between the Patient Financial Eligibility Tool (PFET) and IS was perfect (equal number of drug cycles) for 58.1% of patients, fair (1 cycle difference) for 29.1%, and poor ( &gt; 1 cycle) for 12.8%. Overall Kappa was 0.76 (P &lt; 0.0001). The PFET is an effective tool for determining an individual’s ability to pay for medication. Combined with tiered models for patient participation in the cost of medication, it could help to increase access to high-priced products in developing countries. (PsycINFO Database Record (c) 2014 APA, all rights reserved). (journal abstract) | Test Construction | test validity | Drug Therapy | Client Characteristics | Doublon |
| Marion | Bizé | Barbara | Koloshuk | Joël | Ladner | Population Health Management | 2013-04-00 Apr 2013 | The objective was to develop and validate a multilateral index to determine patient ability to pay for medication in low- and middle-income countries. Primary data were collected in 2009 from 117 cancer patients in China, India, Thailand, and Malaysia. The initial tool included income, expenditures, and assets-based items using ad hoc determined brackets. Principal components analysis was performed to determine final weights. Agreement (Kappa) was measured between results from the final tool and from an Impact Survey (IS) conducted after beginning drug therapy to quantify a patient's actual ability to pay in terms of number of drug cycles per year. The authors present the step-by-step methodology employed to develop the tool on a country-by-country basis. Overall Cronbach value was 0.84. Agreement between the Patient Financial Eligibility Tool (PFET) and IS was perfect (equal number of drug cycles) for 58.1% of patients, fair (1 cycle difference) for 29.1%, and poor (>1 cycle) for 12.8%. Overall Kappa was 0.76 (P<0.0001). The PFET is an effective tool for determining an individual's ability to pay for medication. Combined with tiered models for patient participation in the cost of medication, it could help to increase access to high-priced products in developing countries. | Humans | Questionnaires | developing countries | Social Class | OK |
| Jan A. | Hazelzet | Johannes B. | van Goudoever | |  | Pediatric Critical Care Medicine: A Journal of the Society of Critical Care Medicine and the World Federation of Pediatric Intensive and Critical Care Societies | 2012-09-00 Sep 2012 | OBJECTIVE: To develop and test the psychometric properties of the EMPATHIC-N (EMpowerment of PArents in THe Intensive Care-Neonatology) questionnaire measuring parent satisfaction. DESIGN: A psychometric study testing the reliability and validity of a parent satisfaction questionnaire by applying confirmatory factor analysis including standardized factor loadings and subsequently Cronbach's α reliability estimates across time, congruent validity, and nondifferential validity testing. SETTING: A 30-bed neonatal intensive care unit in a university hospital. PATIENTS: Two cohorts with a total of 441 parents whose child was admitted to the neonatal intensive care unit, January to December 2009. INTERVENTIONS: None. MEASUREMENTS AND MAIN RESULTS: In the first cohort, 220 of 339 (65%) parents responded; in the second cohort, 59 of 102 (58%) parents responded. Structural equation modeling and confirmatory factor analysis resulted in a sufficient model fit of 57 statements within five domains: Information, Care & Treatment, Organization, Parental Participation, and Professional Attitude. Standardized factor loading of these statements were between 0.58 and 0.91. Reliability measures, Cronbach's α, of the domains ranged from 0.82 to 0.95. Reliability across time showed no evidence of statistically significant differences between the domains. Congruent validity was confirmed by a good correlation (p = .01) between the domains and four general satisfaction questions. Nondifferential validity showed no significant effect sizes between the infants' characteristics and the domains, except between ventilated infants and parent participation statements and infants ≥30 wks gestational age and organizational statements. CONCLUSIONS: The EMPATHIC-N questionnaire is a valid quality performance indicator to measure the delivered care as perceived by parents. | Male | Humans | Patient Participation | Patient Satisfaction | OK |
| Takuhiro | Yamaguchi | Hiroshi | Soeda | Yusuke | Sekine | BMC medical informatics and decision making | 2013-00-00 2013 | BACKGROUND: The information provided in patient-centered care and shared decision-making influences patients' concerns and adherence to treatment. In the decision-making process, patients experience decisional conflict. The Decisional Conflict Scale (DCS) is a 16-item, self-administered questionnaire consisting of 5 subscales developed to assess patients' decisional conflict. This study aimed to develop the Japanese version of the DCS and to clarify the influence of the information provided by pharmacists' on decisional conflict among patients with cancer. METHODS: We developed the Japanese version of the DCS by using the forward-backward translation method. One hundred patients who were recommended a new chemotherapy regimen were recruited. The psychometric properties of the Japanese DCS, including internal consistency, convergent validity, discriminant validity, and construct validity, were examined. We assessed the decisional conflict of patients before and after the pharmacists' provision of information. RESULTS: Ninety-four patients, predominately female, with an average age of 58.1 years were sampled. The scores on the 5 subscales of the DCS showed high internal consistency (Cronbach's alpha = 0.84-0.96). Multi-trait scaling analysis and cluster analysis showed strong validity. The mean total DCS score decreased significantly from 40.2 to 31.7 after patients received information from the pharmacists (p < 0.001, paired t-test). Scores on all 5 subscales, namely, uncertainty, informed, values clarity, support, and effective decision, also significantly improved (p < 0.001 for all categories, paired t-test). CONCLUSIONS: The psychometric properties of the Japanese version of the DCS are considered appropriate for it to be administered to patients with cancer. Pharmacists' provision of information was able to decrease decisional conflict among patients with cancer who were recommended a new chemotherapy regimen. | | | | | OK |
| Hakmook | Kang | Jose L. | Moreno | David B. | Abrams | Journal of Medical Internet Research | 2011-00-00 2011 | BACKGROUND: Social networks play an important role in smoking. Provision of social support during cessation is a cornerstone of treatment. Online social networks for cessation are ubiquitous and represent a promising modality for smokers to receive and provide the support necessary for cessation. There are no existing measures specific to online social support for smoking cessation. OBJECTIVE: The objective was to develop a measure of social support to be used in online smoking cessation treatment research. METHODS: Initial items for the Online Social Support for Smokers Scale (OS4) were based on existing theory and scales delineated in various taxonomies. Preliminary field analysis (N = 73) was conducted on 23 initial items to optimize the scale. Further development was conducted on a refined 15-item scale in the context of a large randomized trial of Internet and telephone cessation treatment with follow-ups at 3, 6, 12, and 18 months. In all, 1326 participants were randomized to an enhanced Internet arm that included a large online social network; psychometric analyses employed 3-month follow-up data from those reporting use of the enhanced Internet intervention at least once (n = 873). Items were subjected to a factor analysis, and the internal consistency reliability of the scale was examined along with construct and criterion validity. Other measures used in the study included demographics, nicotine dependence, partner support for cessation, general social support, social integration, stress, depression, health status, online community use, Internet use behaviors, intervention satisfaction, and 30-day point prevalence abstinence. RESULTS: The final 12-item OS4 scale demonstrated high internal consistency reliability (Cronbach alphas .86-.89) across demographic and smoking strata of interest. The OS4 also demonstrated good construct and criterion validity, with the directionality of the observed associations providing support for most a priori hypotheses. Significant Pearson correlations were observed between the OS4 and the Partner Interaction Questionnaire (PIQ) Positive subscale (ρ = .24, P < .001). As hypothesized, participants with the highest OS4 scores were more likely to have actively participated in the enhanced Internet community and to have high levels of satisfaction with the enhanced Internet intervention. In logistic regression analyses, the OS4 was highly predictive of 30-day point-prevalence abstinence at 6, 12, and 18 months (all P values <.001). The odds of abstinence at 6 months rose by 48% for each standard unit increase in online social support (95% confidence interval [CI] 1.17-1.71), dropping only slightly to 37% at 12 and 18 months (95% CI 1.17-1.59). CONCLUSIONS: The OS4 is a brief, reliable, and valid instrument for measuring online social support for smoking cessation. Results should be replicated and extended, but this study suggests the OS4 can be used to advance theory, understand mechanisms, and potentially help to improve the tailoring of Internet-based smoking cessation treatments. It can also inspire development of similar measures for other online health-related intervention research. TRIAL REGISTRATION: Clinicaltrials.gov #NCT00282009; http://clinicaltrials.gov/ct2/show/NCT00282009 (Archived by WebCite at http://www.webcitation.org/60XNj3xM6). | Male | Humans | Patient Participation | Questionnaires | OK |
|  |  |  |  |  |  | Journal of Holistic Nursing: Official Journal of the American Holistic Nurses' Association | 2014-12-23 Dec 23, 2014 | Holistic care has long been a defining attribute of nursing practice. From the earliest years of its formal history, nursing has favored a holistic approach in the care of patients, and such an approach has become more important over time. The expansion of nursing's responsibility in delivering comprehensive primary care, the recognition of the importance of relationship-centered care, and the need for evidence-based legitimation of holistic nursing care and practices to insurance companies, policy-makers, health care providers, and patients highlight the need to examine the holistic properties of nursing care. The Holistic Caring Inventory is a theoretically sound, valid, and reliable tool; however, it does not comprehensively address attributes that have come to define holistic nursing care, necessitating the development of a more current instrument to measure the elements of a holistic perspective in nursing care. The development of a current and more comprehensive measure of holistic nursing care may be critical in demonstrating the importance of a holistic approach to patient care that reflects the principles of relationship-based care, shared decision-making, authentic presence, and pattern recognition. | | | | | OK |
| Louis A. | Penner | Lorna | Mabunda | Janella | Hudson | Journal of Cancer Education: The Official Journal of the American Association for Cancer Education | 2013-06-00 Jun 2013 | Racial disparities have been found in the use of chemotherapy as cancer treatment. These disparities may be, in part, due to well-documented differences in the quality of communication during clinical interactions with oncologists and Black versus White patients. In this study using a community-based participatory research approach, academic researchers, community members, and oncologists formed a partnership to develop a communication intervention to address racial disparities in cancer care. Partners developed a question prompt list (QPL), a simple tool that can be used to improve communication, and thus treatment, during clinical interactions in which oncologists and Black patients discuss chemotherapy. Partners endorsed the use of a QPL, provided specific suggestions for content and format, conducted and analyzed qualitative interviews with Black patients receiving chemotherapy, and approved the final version. The feasibility and effectiveness of the QPL that resulted from this research process are currently under evaluation in a separate study. | Male | Humans | Patient Participation | Patient Satisfaction | OK |
| Desiree | Kosiol |  |  |  |  | Patient Education and Counseling | 2011-01-00 janvier 2011 | Objective: The objectives of the study are to develop a patient-oriented and theory-based questionnaire on the communication preferences of chronically ill patients (KOPRA questionnaire) and to carry out psychometric testing of the instrument. Methods: Following two preliminary studies (focus groups, cognitive interviews), a total of 472 patients with chronic back pain or chronic ischemic heart disease were surveyed. In the main sample (N = 333), communication preferences regarding the physician were assessed; for N = 89 (or N = 50) patients, preferences regarding nursing staff (or therapists) were analyzed. Psychometric testing was done with respect to unidimensionality, fit to an item response theory (IRT) model, and for reliability. The questionnaire was developed and validated in German. Results: In the physician version with a total of 32 items, there are four scales ("Patient participation and patient orientation", "Effective and open communication", "Emotionally supportive communication", and "Communication about personal circumstances") that are unidimensional, fulfill the demands for a 1-parameter IRT model, and are reliable (Cronbach's alpha between .80 and .92). The psychometric properties with respect to nursing staff and therapists are slightly worse. Conclusion: The KOPRA questionnaire has good psychometric properties. Practice implications: Clinical use of the questionnaire appears useful to determine patients' communication preferences. (PsycINFO Database Record (c) 2013 APA, all rights reserved). (journal abstract) | Questionnaires | Psychometrics | communication | patients | Doublon |
| Desiree | Kosiol |  |  |  |  | Patient Education and Counseling | 2011-01-00 Jan 2011 | OBJECTIVE: the objectives of the study are to develop a patient-oriented and theory-based questionnaire on the communication preferences of chronically ill patients (KOPRA questionnaire) and to carry out psychometric testing of the instrument. METHODS: following two preliminary studies (focus groups, cognitive interviews), a total of 472 patients with chronic back pain or chronic ischemic heart disease were surveyed. In the main sample (N=333), communication preferences regarding the physician were assessed; for N=89 (or N=50) patients, preferences regarding nursing staff (or therapists) were analyzed. Psychometric testing was done with respect to unidimensionality, fit to an item response theory (IRT) model, and for reliability. The questionnaire was developed and validated in German. RESULTS: In the physician version with a total of 32 items, there are four scales ("Patient participation and patient orientation", "Effective and open communication", "Emotionally supportive communication", and "Communication about personal circumstances") that are unidimensional, fulfill the demands for a 1-parameter IRT model, and are reliable (Cronbach's alpha between .80 and .92). The psychometric properties with respect to nursing staff and therapists are slightly worse. CONCLUSION: the KOPRA questionnaire has good psychometric properties. PRACTICE IMPLICATIONS: clinical use of the questionnaire appears useful to determine patients' communication preferences. | Male | Humans | Patient Participation | Physician-Patient Relations | OK |
| Maya M. | Harper | Danielle G. | Koby | Adam R. | Williams | Patient Education and Counseling | 2012-09-00 Sep 2012 | OBJECTIVES: To describe the development and refinement of a scheme, detail of essential elements and participants in shared decision making (DEEP-SDM), for coding shared decision making (SDM) while reporting on the characteristics of decisions in a sample of patients with metastatic breast cancer. METHODS: The evidence-based patient choice instrument was modified to reflect Makoul and Clayman's integrative model of SDM. Coding was conducted on video recordings of 20 women at the first visit with their medical oncologists after suspicion of disease progression. Noldus Observer XT v.8, a video coding software platform, was used for coding. RESULTS: The sample contained 80 decisions (range: 1-11), divided into 150 decision making segments. Most decisions were physician-led, although patients and physicians initiated similar numbers of decision-making conversations. CONCLUSION: DEEP-SDM facilitates content analysis of encounters between women with metastatic breast cancer and their medical oncologists. Despite the fractured nature of decision making, it is possible to identify decision points and to code each of the essential elements of shared decision making. Further work should include application of DEEP-SDM to non-cancer encounters. PRACTICE IMPLICATIONS: A better understanding of how decisions unfold in the medical encounter can help inform the relationship of SDM to patient-reported outcomes. | Decision Making | Decision Support Techniques | Humans | Patient Participation | OK |
| Terri R. | Fried |  |  |  |  | BMC medical informatics and decision making | 2011-00-00 2011 | BACKGROUND: Decision-making about appropriate therapy to reduce the stroke risk associated with non-valvular atrial fibrillation (NVAF) involves the consideration of trade-offs among the benefits, risks, and inconveniences of different treatment options. The objective of this paper is to describe the development of a decision support tool for NVAF based on the provision of individualized risk estimates for stroke and bleeding and on preparing patients to communicate with their physicians about their values and potential treatment options. METHODS: We developed a tool based on the principles of the International Patient Decision Aids Standards. The tool focuses on the patient-physician dyad as the decision-making unit and emphasizes improving the interaction between the two. It is built on the recognition that the application of patient values to a specific treatment decision is complex and that the final treatment choice is best made through a process of patient-clinician communication. RESULTS: The tool provides education incorporating patients ' illness perceptions to explain the relationship between NVAF and stroke, and then presents individualized risk estimates, derived using separate risk calculators for stroke and bleeding over a clinically meaningful time period (5 years) associated with no treatment, aspirin, and warfarin. Sequelae of both stroke and bleeding outcomes are also described. Patients are encouraged to verbalize how they value the incremental risks and benefits associated with each option and write down specific concerns to address with their physician. A physician prompt to encourage patients to discuss their opinions is included as part of the decision support tool. In pilot testing with 11 participants (mean age 78 ± 9 years, 64% with ≤ high-school education), 8 (72%) rated ease of completion as "very easy," and 9 (81%) rated amount of information as "just right." CONCLUSIONS: The risks and benefits of different treatment options for reduction of stroke in NVAF vary widely according to patients' comorbidities. This tool facilitates the provision of individualized outcome data and encourages patients to communicate with their physicians about these risks and benefits. Future studies will examine whether use of the tool is associated with improved quality of decision making. | Decision Support Techniques | Humans | Patient Participation | Physician-Patient Relations | OK |
| Cayce C. | Hughes | Elena | Debartolo | Carina | Behrens | Academic Pediatrics | 2012-10-00 2012 Sep-Oct | OBJECTIVES: To describe the development and validation of an instrument to measure parents' attention deficit-hyperactivity disorder (ADHD) treatment preferences and goals. METHODS: Parents of children 6 to 12 years of age diagnosed with ADHD in the past 18 months were recruited from 8 primary care sites and an ADHD treatment center (autism excluded). A 16-item medication, 15-item behavior therapy preference scale and a 23-item goal scale, were developed after a review of the literature, 90 parent and clinician semistructured interviews, and input from parent advocates and professional experts were administered to parents. Parent cognitive interviews confirmed item readability, clarity, content, and response range. We conducted an exploratory factor analysis and assessed internal consistency and test-retest reliability and construct and concurrent validity. RESULTS: We recruited 237 parents (mean child age 8.1 years, 51% black, 59% from primary care, 61% of children medication naive). Factor analyses identified 4 medication preference subscales (treatment acceptability, feasibility, stigma, and adverse effects, Cronbach's α 0.74-0.87); 3 behavior therapy subscales (treatment acceptability, feasibility, and adverse effects, α 0.76-0.83); and 3 goal subscales (academic achievement, behavioral compliance, and interpersonal relationships, α 0.83-0.86). The most strongly endorsed goal was academic achievement. The scales demonstrated construct validity, concurrent validity (r = 0.3-0.6) compared with the Treatment Acceptability Questionnaire and Impairment Rating Scale and moderate to excellent test-retest reliability (intraclass coefficient = 0.7-0.9). CONCLUSIONS: We developed a valid and reliable instrument for measuring preferences and goals for ADHD treatment, which may help clinicians more easily comply with new national treatment guidelines for ADHD that emphasize shared decision making. | Male | Decision Making | Humans | Patient Participation | OK |
| Aart | Schene | Giel | Hutschemaekers | |  | BMC health services research | 2010-00-00 2010 | BACKGROUND: Health clinicians perceive certain patients as 'difficult' across all settings, including mental health care. In this area, patients with non-psychotic disorders that become long-term care users may be perceived as obstructing their own recovery or seeking secondary gain. This negative perception of patients results in ineffective responses and low-quality care by health clinicians. Using the concept of illness behaviour, this paper describes the development, implementation, and planned evaluation of a structured intervention aimed at prevention and management of ineffective behaviours by long-term non-psychotic patients and their treating clinicians. METHODS: The principles of Intervention Mapping were applied to guide the development, implementation, and planned evaluation of the intervention. Qualitative (individual and group interviews), quantitative (survey), and mixed methods (Delphi-procedure) research was used to gain a broad perspective of the problem. Empirical findings, theoretical models, and existing evidence were combined to construct a program tailored to the needs of the target groups. RESULTS: A structured program to increase effective illness behaviour in long-term non-psychotic patients and effective professional behaviour in their treating clinicians was developed, consisting of three subsequent stages and four substantial components, that is described in detail. Implementation took place and evaluation of the intervention is being carried out. CONCLUSIONS: Intervention Mapping proved to be a suitable method to develop a structured intervention for a multi-faceted problem in mental health care. | Decision Making | Humans | Patient Participation | Adult | OK |
| Lin | Ding | Stephanie | Fry | Patricia | Gallagher | Medical Care | 2012-11-00 novembre 2012 | Objective: To develop and evaluate survey questions that assess processes of care relevant to Patient-Centered Medical Homes (PCMHs). Research Design: We convened expert panels, reviewed evidence on effective care practices and existing surveys, elicited broad public input, and conducted cognitive interviews and a field test to develop items relevant to PCMHs that could be added to the Consumer Assessment of Healthcare Providers and Systems (CAHPS®) Clinician & Group (CG-CAHPS) 1.0 Survey. Surveys were tested using a 2-contact mail protocol in 10 adults and 33 pediatric practices (both private and community health centers) in Massachusetts. A total of 4875 completed surveys were received (overall response rate of 25%). Analyses: We calculated the rate of valid responses for each item. We conducted exploratory factor analyses and estimated item-to-total correlations, individual and site-level reliability, and correlations among proposed multi-item composites. Results: Ten items in 4 new domains (Comprehensiveness, Information, Self-Management Support, and Shared Decision-Making) and 4 items in 2 existing domains (Access and Coordination of Care) were selected to be supplemental items to be used in conjunction with the adult CG-CAHPS 1.0 Survey. For the child version, 4 items in each of 2 new domains (Information and Self-Management Support) and 5 items in existing domains (Access, Comprehensiveness-Prevention, Coordination of Care) were selected. Conclusions: This study provides support for the reliability and validity of new items to supplement the CG-CAHPS 1.0 Survey to assess aspects of primary care that are important attributes of PCMHs. (PsycINFO Database Record (c) 2013 APA, all rights reserved). (journal abstract) | Measurement | Psychometrics | Test Construction | test validity | Doublon |
| Lin | Ding | Stephanie | Fry | Patricia | Gallagher | Medical Care | 2012-11-00 Nov 2012 | OBJECTIVE: To develop and evaluate survey questions that assess processes of care relevant to Patient-Centered Medical Homes (PCMHs). RESEARCH DESIGN: We convened expert panels, reviewed evidence on effective care practices and existing surveys, elicited broad public input, and conducted cognitive interviews and a field test to develop items relevant to PCMHs that could be added to the Consumer Assessment of Healthcare Providers and Systems (CAHPS®) Clinician & Group (CG-CAHPS) 1.0 Survey. Surveys were tested using a 2-contact mail protocol in 10 adults and 33 pediatric practices (both private and community health centers) in Massachusetts. A total of 4875 completed surveys were received (overall response rate of 25%). ANALYSES: We calculated the rate of valid responses for each item. We conducted exploratory factor analyses and estimated item-to-total correlations, individual and site-level reliability, and correlations among proposed multi-item composites. RESULTS: Ten items in 4 new domains (Comprehensiveness, Information, Self-Management Support, and Shared Decision-Making) and 4 items in 2 existing domains (Access and Coordination of Care) were selected to be supplemental items to be used in conjunction with the adult CG-CAHPS 1.0 Survey. For the child version, 4 items in each of 2 new domains (Information and Self-Management Support) and 5 items in existing domains (Access, Comprehensiveness-Prevention, Coordination of Care) were selected. CONCLUSIONS: This study provides support for the reliability and validity of new items to supplement the CG-CAHPS 1.0 Survey to assess aspects of primary care that are important attributes of PCMHs. | Male | Humans | Patient Satisfaction | Questionnaires | OK |
| Cox | Terhorst | David M. | Markusic |  |  | Molecular Therapy. Methods & Clinical Development | 2014-04-30 Apr 30, 2014 | Gene replacement therapies, like organ and cell transplantation are likely to introduce neo-antigens that elicit rejection via humoral and/or effector T cell immune responses. Nonetheless, thanks to an ever growing body of pre-clinical studies it is now well accepted that gene transfer protocols can be specifically designed and optimized for induction of antigen-specific immune tolerance. One approach is to specifically express a gene in a tissue with a tolerogenic microenvironment such as the liver or thymus. Another strategy is to transfer a particular gene into hematopoietic stem cells or immunological precursor cells thus educating the immune system to recognize the therapeutic protein as "self". In addition, expression of the therapeutic protein in pro-tolerogenic antigen presenting cells such as immature dendritic cells and B cells has proven to be promising. All three approaches have successfully prevented unwanted immune responses in pre-clinical studies aimed at the treatment of inherited protein deficiencies, e.g. lysosomal storage disorders and hemophilia, and of type I diabetes and multiple sclerosis. In this review we focus on current gene transfer protocols that induce tolerance, including gene delivery vehicles and target tissues, and discuss successes and obstacles in different disease models. | | | | | OK |
| Carrie A. | Levin | Michael J. | Barry | Carol | Cosenza | Health Expectations: An International Journal of Public Participation in Health Care and Health Policy | 2010-09-00 Sep 2010 | BACKGROUND: Women with early-stage breast cancer face a multitude of decisions. The quality of a decision can be measured by the extent to which the treatment reflects what is most important to an informed patient. Reliable and valid measures of patients' knowledge and their goals and concerns related to breast cancer treatments are needed to assess the decision quality. OBJECTIVE: To identify a set of key facts and goals relevant to each of three breast cancer treatment decisions (surgery, reconstruction and adjuvant chemotherapy and hormone therapy) and to evaluate the validity of the methods used to identify them. METHODS: Candidate facts and goals were chosen based on evidence review and qualitative studies with breast cancer patients and providers. Cross-sectional surveys of patients and providers were conducted for each decision. The accuracy, importance and completeness of the items were examined. RESULTS: Thirty-eight facts (11-14 per decision) and 27 goals (8-10 per decision) were identified. An average of 17 patients and 21 providers responded to each survey. The sets of facts were accurate and complete for all three decisions. The sets of goals and concerns were important for surgery and reconstruction, but not chemotherapy/hormone therapy. Patients and providers disagreed about the relative importance of several key facts and goals. CONCLUSIONS: Overall, breast cancer patients and providers found the sets of facts and goals accurate, important and complete for three treatment decisions. Because patients' and providers' perspectives are different, it is vital that instrument development should include items reflecting both views. | Decision Making | Humans | Patient Participation | Questionnaires | OK |
| Jan | Angus | Larry | Paszat | Kelly | Metcalfe | International Journal of Radiation Oncology, Biology, Physics | 2012-09-01 Sep 1, 2012 | PURPOSE: To develop a patient decision aid (PtDA) for older women with Stage I, pathologically node negative, estrogen receptor-positive progesterone receptor-positive breast cancer who are considering adjuvant radiotherapy after lumpectomy and to examine its impact on patients' decision making. METHODS AND MATERIALS: A PtDA was developed and evaluated in three steps according to the Ottawa Decision Support Framework: (1) needs assessment (n = 16); (2) Pilot I to examine PtDA acceptability (n = 12); and (3) Pilot II, a pretest posttest (n = 38) with older women with estrogen receptor-positive progesterone receptor-positive breast cancer after lumpectomy who were receiving adjuvant radiation therapy. Measures included patients' satisfaction with the PtDA, self-reported decisional conflict, level of distress, treatment-related knowledge, and choice predisposition. RESULTS: The PtDA is a booklet that details each adjuvant treatment option's benefits, risks, and side effects tailored to the patient's clinical profile; includes a values clarification exercise; and includes steps to guide patients towards their decision. On the basis of qualitative comments and satisfaction ratings, all women thought that the PtDA was helpful and informative. In comparison with their baseline scores, patients had a statistically significant (p < 0.05) reduction in decisional conflict (adjusted mean difference [AMD], -7.18; 95% confidence interval [CI], -13.50 to 12.59); increased clarity of the benefits and risks (AMD, -10.86; CI, -20.33 to 21.49); and improved general treatment knowledge (AMD, 8.99; CI, 2.88-10.28) after using the PtDA. General trends were also reported in the patients' choice predisposition scores that suggested potential differences in treatment decision after PtDA use. CONCLUSIONS: This study provides evidence that this PtDA may be a helpful educational tool for this group of women. The quality of care for older breast cancer patients may be enhanced by the use of a tailored PtDA to help patients be better informed about their treatment options. | Decision Support Techniques | Humans | Patient Participation | Questionnaires | OK |
| Merlin | Njoya | Robert | Thivierge | France | Légaré | Patient Education and Counseling | 2012-08-00 Aug 2012 | OBJECTIVE: To develop and evaluate items for inclusion in PRIDe (Preferred Role in Decision Making), a new tool to assess changes of role preference among professionals exposed to training in shared decision making (SDM). METHODS: This study was part of a pilot trial to evaluate the effectiveness of SDM training on the doctors' prescription of antibiotics for acute respiratory infections. Thirty-nine family physicians were randomized to immediate exposure to training or to delayed exposure. Potential items for PRIDe and a questionnaire about physicians' intention to engage in SDM were administered at baseline and at follow-up. RESULTS: Following analysis, we retained five items that captured a change in physicians' preference. The items' scores were pooled and the resulting tool showed limited internal consistency (Cronbach's alpha = 0.41) but significant test-retest reliability (immediate group: P = 0.03; delayed group: P = 0.008) and acceptable discriminant validity, with patients involved in decision making more actively after training than before (Fisher's test, P = .02). CONCLUSION: This initial step to develop an evaluation tool to assess changes in doctors' preference of role in decision making following SDM training shows promising results. The next step is to develop more clinical vignettes followed by questions inspired from this analysis. PRACTICE IMPLICATIONS: The PRIDe instrument can be used in the assessment of health professionals' attitude towards shared decision making after training in shared decision making. Additional research is needed to evaluate its validity before it can be recommended for use. | Male | Decision Making | Humans | Patient Participation | OK |
| Merlin | Njoya | Robert | Thivierge | France | Légaré | Patient Education and Counseling | 2012-08-00 août 2012 | Objective: To develop and evaluate items for inclusion in PRIDe (Preferred Role in Decision Making), a new tool to assess changes of role preference among professionals exposed to training in shared decision making (SDM). Methods: This study was part of a pilot trial to evaluate the effectiveness of SDM training on the doctors’ prescription of antibiotics for acute respiratory infections. Thirty-nine family physicians were randomized to immediate exposure to training or to delayed exposure. Potential items for PRIDe and a questionnaire about physicians’ intention to engage in SDM were administered at baseline and at follow-up. Results: Following analysis, we retained five items that captured a change in physicians’ preference. The items’ scores were pooled and the resulting tool showed limited internal consistency (Cronbach’s alpha = 0.41) but significant test–retest reliability (immediate group: P = 0.03; delayed group: P = 0.008) and acceptable discriminant validity, with patients involved in decision making more actively after training than before (Fisher’s test, P = 0.02). Conclusion: This initial step to develop an evaluation tool to assess changes in doctors’ preference of role in decision making following SDM training shows promising results. The next step is to develop more clinical vignettes followed by questions inspired from this analysis. Practice implications: The PRIDe instrument can be used in the assessment of health professionals’ attitude towards shared decision making after training in shared decision making. Additional research is needed to evaluate its validity before it can be recommended for use. (PsycINFO Database Record (c) 2012 APA, all rights reserved). (journal abstract) | Measurement | Decision Making | Test Construction | clinical decision making | Doublon |
| Thomas | Cerny | Florian | Strasser |  |  | Critical Reviews in Oncology/Hematology | 2010-01-00 Jan 2010 | Assessment of individual patients' distress is a cornerstone of clinical care for advanced cancer. Patients' ability to fill out lengthy questionnaires is compromised by many factors. Computer-adaptive tests (CAT) offer a promising approach to developing tailored instruments, that administer only items relevant to the individual patient. A systematic review of the literature about CATs in medical databases was conducted. Based on the results, a method for developing a CAT was designed that requires nine steps: (1) build an item pool; (2) administer the items to a predefined sample in a calibration study; (3) eliminate inappropriate items; (4) examine whether all items are influenced by a single dominant trait; (5) calibrate the items to the best-fitting item response theory (IRT) model; (6) evaluate items' parameter equivalence across subgroups; (7) build an item bank with the calibrated items; (8) develop the CAT; and (9) pilot test the developed CAT. CAT offers the chance to extend the usefulness of patient-reported outcome (PRO) measurements from clinical studies to daily clinical practice. | Humans | Patient Participation | Outcome Assessment (Health Care) | Software | OK |
| J. | Brodersen |  |  |  |  | Scandinavian Journal of Medicine & Science in Sports | 2013-10-00 Oct 2013 | Patient-related outcome measures (PROMs) are commonly used to gauge treatment effects in patients with anterior cruciate ligament (ACL) deficiency. Valid measures of specific conditions depend on relevant item content. While item content can be derived either from clinicians (face validity) or from patients, item relevance and comprehensiveness can only be confirmed by the patient (content validity). Focus group and single interviews were conducted with patients' pre- and post-ACL reconstruction in order to construct a condition-specific PROM for the target patients. One hundred fifty-seven items from a previously conducted literature search were used as a basis for content genesis. Content saturation was achieved after three focus groups and seven single interviews. Thirty-eight items from the literature search were directly endorsed, and five modified items were endorsed. Twelve new item themes with verbatim content emerged. Thus, a 55-item pilot PROM consisting of six conceptual domains was assembled. We have constructed a condition-specific PROM for measurement of surgical and non-surgical treatment effects on ACL deficiency. The instrument possesses face and content validity. A pilot survey to assess the psychometric properties will be performed using Rasch measurement theory. | Male | Humans | Patient Participation | Young Adult | OK |
| Allen W. | Heinemann | Jennifer A. | Bogner | Tamara | Bushnik | Archives of Physical Medicine and Rehabilitation | 2011-04-00 Apr 2011 | OBJECTIVE: To develop a new measure, the Participation Assessment With Recombined Tools-Objective (PART-O), based on items from 3 participation instruments. DESIGN: Instrument development based on survey research. SETTING: Community. PARTICIPANTS: Adults (N=400) with traumatic brain injury (TBI) 1 to 15 years postinjury, recruited from 8 TBI Model Systems (TBIMS). INTERVENTIONS: None. MAIN OUTCOME MEASURE: Community Integration Questionnaire version 2; Participation Objective, Participation Subjective; Craig Handicap Assessment and Reporting Technique; PART-O. RESULTS: Using Rasch rating scale analysis to evaluate the psychometric properties of participation items drawn from 3 instruments, a set of 24 items was developed that covered a broad range of participation content and formed a measure with person separation of 2.47, person reliability of .86, item spread of 4.25 logits, item separation of 11.36, and item reliability of .99. Items were well targeted on the sample with only 1 item misfitting. The PART-O showed expected relationships with measures of impairment, activity limitations, and subjective well-being. CONCLUSIONS: The 24-item PART-O is an acceptable measure of objective participation for persons with moderate and severe TBI. It has been adopted as the measure of participation in the TBIMS. | Male | Humans | Patient Participation | Questionnaires | OK |
| Yuko | Shimizu | Yuji | Kono | Akihiro | Hirashiki | American Journal of Physical Medicine & Rehabilitation / Association of Academic Physiatrists | 2012-06-00 Jun 2012 | OBJECTIVE: We report on the development of a participation measure for patients with congestive heart failure (CHF)-the Participation Scale for Patients with CHF (PS-CHF)-and the determination of its unidimensionality, reliability, and validity. DESIGN: This study included two rounds of data collections: 62 patients enrolled in the first round for the purpose of item reduction, and 130 CHF patients and 96 healthy controls participated in the second round for questionnaire validation. RESULTS: The PS-CHF was organized into five items designed to ask about limitations to participation. The PS-CHF score ranges from 5 points (lowest level of participation) to 20 points (highest level of participation). On Rasch analysis, the item calibrations ranged from +0.51 logit for the most difficult item to -0.56 logit for the least difficult item. The range of mean square statistics values was 1.20-0.81 for infit and 1.34-0.72 for outfit. The intraclass correlation coefficient between scores from two successive administrations of the PS-CHF, separated by 2 wks, was 0.94. The total PS-CHF score in CHF patients was significantly lower than in healthy controls. CONCLUSIONS: The PS-CHF was developed to determine a disease-specific participation measure for patients with CHF. The PS-CHF may serve as a clinically meaningful outcome measure for CHF patients. | Male | Humans | Patient Participation | Questionnaires | OK |
| Benjamin M. | Craig |  |  |  |  | BMC health services research | 2012-00-00 2012 | BACKGROUND: This study examines the psychometric properties of 9 items on the Patient Activation component of the Medicare Current Beneficiary Survey (MCBS) that assess how patients approach and communicate with their physicians. The MCBS is a nationally representative, cross-sectional survey of Medicare beneficiaries. METHODS: We analyzed MCBS data collected in 2002 and 2005 from 15,165 adults aged 65 and older. Exploratory factor analysis was conducted using maximum likelihood to estimate a polychoric correlation matrix on the 2002 data, and confirmatory factor analysis was performed using the 2005 data. RESULTS: Exploratory factor analysis of the 2002 data showed a 2-factor solution: approach to interactions (5 items) and views about physician's healthcare communication (6 items). Findings were confirmed using the 2005 data. Items were combined to form the Patient Approach and Views toward Healthcare Communication (PAV-COM) scale (range 1 to 100; Cronbach's alpha of 0.75, and item-rest correlations between 0.33 and 0.54). Higher PAV-COM scores were associated with greater fulfillment of preventive health behaviors such as vaccinations and cancer screenings. CONCLUSIONS: The PAV-COM measure is a valid tool for assessing patient approaches and views toward communication with physicians. This measure can be used to evaluate interventions to improve patient participation during healthcare encounters. | Male | Humans | Patient Participation | Physician-Patient Relations | OK |
| Shannon M. | Canfield | Julie A. | Stuppy | David R. | Mehr | BMC family practice | 2014-00-00 2014 | BACKGROUND: Technology-based aids for lifestyle change are becoming more prevalent for chronic conditions. Important "digital divides" remain, as well as concerns about privacy, data security, and lack of motivation. Researchers need a way to characterize participants' readiness to use health technologies. To address this need, we created an instrument to measure patient readiness to engage with health technologies among adult patients with chronic conditions. METHODS: Initial focus groups to determine domains, followed by item development and refinement, and exploratory factor analysis to determine final items and factor structure. The development sample included 200 patients with chronic conditions from 6 family medicine clinics. From 98 potential items, 53 best candidate items were examined using exploratory factor analysis. Pearson's Correlation for Test/Retest reliability at 3 months. RESULTS: The final instrument had 28 items that sorted into 8 factors with associated Cronbach's alpha: 1) Health Information Need (0.84), 2) Computer/Internet Experience (0.87), 3) Computer Anxiety (0.82), 4) Preferred Mode of Interaction (0.73), 5) Relationship with Doctor (0.65), 6) Cell Phone Expertise (0.75), 7) Internet Privacy (0.71), and 8) No News is Good News (0.57). Test-retest reliability for the 8 subscales ranged from (0.60 to 0.85). CONCLUSION: The Patient Readiness to Engage in Health Internet Technology (PRE-HIT) instrument has good psychometric properties and will be an aid to researchers investigating technology-based health interventions. Future work will examine predictive validity. | Male | Humans | Patient Participation | Questionnaires | OK |
| Artur K. | Krupa | Richard F. | Hinds | John M. | Litell | BMC anesthesiology | 2014-00-00 2014 | BACKGROUND: Shared-decision-making about resuscitation goals of care for intensive care unit (ICU) patients depends on a basic understanding of cardiopulmonary resuscitation (CPR). Our objective was to develop and validate a survey to assess comprehension of CPR among ICU patients and surrogate decision-makers. METHODS: We developed a 12-item verbally-administered survey incorporating input from patients, clinicians, and expert focus groups. RESULTS: We administered the survey to 32 ICU patients and 37 surrogates, as well as to 20 resident physicians to test discriminative validity. Median (interquartile range) total knowledge scores were 7 (5-10) for patients, 9 (7-12) for surrogates, and 14.5 (14-15) for physicians (p <.001). Forty-four percent of patients and 24% of surrogates could not explain the purpose of CPR. Eighty-eight percent of patients and 73% of surrogates could not name chest compressions and breathing assistance as two components of CPR in the hospital. Forty-one percent of patients and 24% of surrogates could not name a single possible complication of CPR. Forty-three percent of participants could not specify that CPR would be performed with a full code order and 25% of participants could not specify that CPR would not be performed with a do-not-resuscitate order. Internal consistency (Cronbach's alpha = 0.97) and test-retest reliability (Pearson correlation = 0.96, p < .001) were high. CONCLUSIONS: This easily administered survey, developed to measure knowledge of CPR and resuscitation preference options among ICU patients and surrogates, showed strong face validity, content validity, internal consistency, test-retest reliability, and discriminative validity. A substantial proportion of ICU patients and surrogates decision-makers have poor knowledge of CPR and basic resuscitation options. | | | | | OK |
| Chung-Ping | Cheng | Chi-Chun | Chin | Joel | Stocker | Journal of Advanced Nursing | 2011-01-00 Jan 2011 | AIM: This paper is a report of the development and psychometric testing of the Chinese version of the Diabetes Empowerment Process Scale. BACKGROUND: In people living with diabetes, empowerment can improve metabolic and psychosocial outcomes. A scale for measuring empowerment processes can also help healthcare professionals to optimize their empowering actions and would improve their interactions with people living with diabetes. METHOD: Based on literature reviews and interviews with people living with diabetes, a 27-item Chinese Diabetes Empowerment Process Scale was developed. Fifteen items were produced after item analysis and content validity testing. To test construct validity, concurrent validity and internal consistency, 211 outpatients living with diabetes completed the Chinese Diabetes Empowerment Process Scale, a Self-care Behaviour Scale and a Diabetes Empowerment Scale. Test-retest reliability was also analysed with 30 patients. The study was conducted in 2008 and 2009 in Taiwan. RESULTS: Confirmatory factor analysis indicated that a second-order factorial model with four subscales and 15 items best fitted the data. The four subscales were Mutual participation, Raising awareness, Providing necessary information and Open communication. Scores on the Chinese Diabetes Empowerment Process Scale correlated statistically significantly with those on the Self-care Behaviour Scale (r=0.21-0.40; P<0.01) and Diabetes Empowerment Scale (r=0.35-0.65; P<0.01). Reliability was supported by acceptable Cronbach's α (range: 0.73-0.91) and test-retest reliability scores (range: 0.75-0.83). CONCLUSION: The Chinese Diabetes Empowerment Process Scale has satisfactory validity and reliability for measuring the empowerment process of health professionals. Further studies are needed to test the applicability of the scale to other populations. | Male | Humans | Patient Participation | Questionnaires | OK |
| L. | Weissbach |  |  |  |  | Der Urologe. Ausg. A | 2012-02-00 Feb 2012 | BACKGROUND: It is challenging to decide in which way we should care for localized prostate cancer. The current guideline can be accepted as an appropriate foundation. However, there are doubts if guideline recommendations are implemented. RESULTS: The presented opinion survey of prostate cancer support groups shows that we have too many prostatectomies in senior patients. It reveals deficiencies in the guidance of the patients. They are not sufficiently embedded in the process of decision-making. More attention should be paid to initiatives of patient support groups. | Male | Decision Making | Humans | Patient Participation | OK |
| Hans | Geinitz | Anca L. | Grosu |  |  | Medical Science Monitor: International Medical Journal of Experimental and Clinical Research | 2012-07-00 Jul 2012 | BACKGROUND: Assessment of cancer- and host-related prognostic factors has a long tradition in patients with brain metastases. In continuation of large-scale studies performed by the Radiation Therapy Oncology Group (RTOG) in the United States, the 4-tiered diagnosis-specific graded prognostic assessment (DS-GPA) score has been developed. It stratifies patients with common primary tumours metastasizing to the brain (malignant melanoma, lung, breast, kidney and gastrointestinal cancers) into subgroups with different prognoses. However, many patients in the DS-GPA study were treated with surgical resection or radiosurgery (SRS). The present multi-institutional analysis examined for the first time whether DS-GPA is a valid score in European patients managed in routine clinical practice. MATERIAL/METHODS: This was a retrospective analysis of 412 patients with primary malignant melanoma, lung, breast, kidney or gastrointestinal cancers. Survival was evaluated in uni- and multivariate tests. RESULTS: DS-GPA significantly predicted survival and outperformed initial GPA, a score that is not diagnosis-specific. Median survival by DS-GPA strata (all 412 patients) was 2.7, 3.6, 7.0 and 11.3 months in the 4 groups with 0-1, 1.5-2, 2.5-3 and 3.5-4 points, respectively. The previously published survival data (median 7.2 months for all patients) could not be replicated in this cohort (median 3.6 months). CONCLUSIONS: DS-GPA is a valid prognostic score that might improve shared decision making as well as patient stratification in prospective clinical trials. | Male | Humans | Young Adult | Female | OK |
| Marc | Evans | Cynthia F. | Corbett | Sue | Nicholson Butkus | Ecology of Food and Nutrition | 2012-00-00 2012 | Diabetes education programs need improved measures of goal setting for dietary control of diabetes. Additionally, measures of patient experiences with dietary goal setting are needed to better prepare patients for diabetes self-management. Measures of dietary goals and strategies were investigated via survey of 100 Mexican Americans and non-Hispanic whites with type 2 diabetes at a community clinic. Analyses tested novel goal measures as stages of change and goal attainment with a food plan compared to a traditional measure of food plan adherence. Ethnic groups varied in some reported experiences with goal setting education and goal attainment, but did not differ in most clinical characteristics of diabetes. Results indicated that different measures of goal setting vary in their psychosocial predictors, suggesting changes in how health care providers use and monitor goal setting for patients. At the time this research was conducted, Dr. Briggs Early was a doctoral candidate in the Department of Food Science and Human Nutrition at Washington State University. She is currently an Assistant Professor of Biochemistry and Nutrition at Pacific Northwest University of Health Sciences - College of Osteopathic Medicine, and a certified diabetes educator, and insulin pump trainer in Yakima, Washington. | Male | Humans | Patient Participation | Patient Satisfaction | OK |
| Anca | Romantan | Bridget J. | Kelly | Angela | DeMichele | Patient Education and Counseling | 2010-12-00 Dec 2010 | OBJECTIVE: There is much research describing cancer patients' information needs and their use of the Internet, print media, and other sources to fulfill these needs. Yet little is known about whether patients with different types of cancer vary in their information needs and seeking behaviors. This study used population-based data to address this question. METHODS: A sample was randomly drawn from the list of patients with breast, prostate, or colorectal cancer reported to the Pennsylvania Cancer Registry in 2005. Patients completed a mail survey (N=2010); respective response rates were 68%, 64%, and 61%. RESULTS: Colorectal cancer patients reported consistently less information seeking than breast and prostate cancer patients. Multivariate analyses revealed that differences by cancer type were not explained by sex or other demographics, disease stage, or treatment received. These differences were most pronounced among patients with early stage cancer. CONCLUSION: Cancer patients have myriad information needs and use a range of sources to satisfy these needs, but there appear to be important differences in information engagement by cancer type. PRACTICE IMPLICATIONS: Understanding differences in information seeking among disease-specific populations may help guide future patient education and decision making across the care continuum. | Male | Humans | Patient Participation | Female | OK |
| Jorge | Girotti | Angela | Bixby | Luis | Rivera | Journal of Immigrant and Minority Health / Center for Minority Public Health | 2013-06-00 Jun 2013 | Patient-reported experiences of care are an important focus in health disparities research. This study explored the association of patient-reported experiences of care with race and acculturation status in a primary care setting. 881 adult patients (African-American 34%; Hispanic--classified as unacculturated or biculturated--31%; Caucasian 33%; missing race 2%), in outpatient Family Medicine clinics, completed a written survey in Spanish or English. Consumer Assessment of Healthcare Providers and Systems (CAHPS) Clinician & Group (CAG) Survey Adult Primary Care instrument was used for experiences of care and Short Form-12 survey for health status. Controlling for other variables, race and acculturation were significantly associated with several CAG subscales. Hispanic patients gave significantly higher ratings for care experiences and expressed greater interest in shared decision making. Selected patient-reported measures of care are associated with patients' race and acculturation status (for Hispanic patients). We discuss implications for both provision and measurement of quality care. | Male | Humans | United States | Young Adult | OK |
| C. | Meller |  |  |  |  | European Journal of Dental Education: Official Journal of the Association for Dental Education in Europe | 2010-11-00 Nov 2010 | Whilst preparing undergraduate students for a clinical course in paediatric dentistry, four consecutive classes (n = 107) were divided into two groups. Seven behaviour-modifying techniques were introduced: systematic desensitization, operant conditioning, modelling, Tell, Show, Do-principle, substitution, change of roles and the active involvement of the patient. The behaviour-modifying techniques that had been taught to group one (n = 57) through lecturing were taught to group two (n = 50) through video sequences and vice versa in the following semester. Immediately after the presentations, students were asked by means of a questionnaire about their perceptions of ease of using the different techniques and their intention for clinical application of each technique. After completion of the clinical course, they were asked about which behaviour-modifying techniques they had actually used when dealing with patients. Concerning the perception of ease of using the different techniques, there were considerable differences for six of the seven techniques (P < 0.05). Whilst some techniques seemed more difficult to apply clinically after lecturing, others seemed more difficult after video-based teaching. Concerning the intention for clinical application and the actual clinical application, there were higher percentages for all techniques taught after video-based teaching. However, the differences were significant only for two techniques in each case (P < 0.05). It is concluded that the use of video based teaching enhances the intention for application and the actual clinical application only for a limited number of behaviour-modifying techniques. | Male | Humans | Patient Participation | Questionnaires | OK |
| Xun | Zhang | Helen | Magdalinos |  |  | Injury Prevention: Journal of the International Society for Child and Adolescent Injury Prevention | 2011-06-00 Jun 2011 | BACKGROUND: Many injury studies use samples of hospital patients. If a study requires further contact, consent is usually required but the sample may be biased if many fail to consent. OBJECTIVE: To determine whether wording requiring 'active' consent resulted in more refusals than wording that was 'passive'--that is, where consent is implied in the absence of direct refusal. METHODS: Subjects were injured children seen in the emergency departments in five hospitals where the Canadian Hospital Injury Reporting and Prevention Program (CHIRPP) operates. For CHIRPP, parents or older children complete a one-page questionnaire to describe the injury; one question seeks consent to follow-up for research. Three of the hospitals use passive wording for this question and two use active wording. All cases where CHIRPP coordinators completed forms using the medical record were treated as refusals. It was hypothesised that there would be a significant association between the form of wording (active or passive) and the rate of consent, and that this association would be affected by socio-demographic and clinical variables. RESULTS: On average, 64.5% of parents gave consent for follow-up at hospitals using passive consent wording versus 42% where active consent wording was used. Passive wording consistently yielded a higher percentage of consents for all variables. The differences were greater than 5% for families living in census tracts with low median household incomes and greater than 10% for those age 15-19 years. For parent completed forms the adjusted OR for active wording was 0.48 (95% CI 0.43 to 0.54). CONCLUSION: Caution is needed when interpreting results from studies using hospital samples, especially when wording of consent for follow-up requires direct affirmation. | Male | Humans | Patient Participation | Questionnaires | OK |
| S. | Kreiner | J. | Brodersen |  |  | Scandinavian Journal of Medicine & Science in Sports | 2013-10-00 Oct 2013 | The benefit of anterior cruciate ligament (ACL) reconstruction has been questioned based on patient-reported outcome measures (PROMs). Valid interpretation of such results requires confirmation of the psychometric properties of the PROM. Rasch analysis is the gold standard for validation of PROMs, yet PROMs used for ACL reconstruction have not been validated using Rasch analysis. We used Rasch analysis to investigate the psychometric properties of the Knee Numeric-Entity Evaluation Score (KNEES-ACL), a newly developed PROM for patients treated for ACL deficiency. Two-hundred forty-two patients pre- and post-ACL reconstruction completed the pilot PROM. Rasch models were used to assess the psychometric properties (e.g., unidimensionality, local response dependency, and differential item functioning). Forty-one items distributed across seven unidimensional constructs measuring impairment, functional limitations, and psychosocial consequences were confirmed to fit Rasch models. Fourteen items were removed because of statistical lack of fit and inadequate face validity. Local response dependency and differential item functioning were identified and adjusted. The KNEES-ACL is the first Rasch-validated condition-specific PROM constructed for patients with ACL deficiency and patients with ACL reconstruction. Thus, this instrument can be used for within- and between-group comparisons. | Male | Humans | Patient Participation | Questionnaires | OK |
| Marcello | Anti | Diego | Baiocchi | Alessandra | Barca | Journal of Medical Screening | 2011-00-00 2011 | BACKGROUND: Sending faecal occult blood tests (FOBT) by mail has been proposed both as a method to increase participation and a way to reduce staff costs in colorectal cancer screening. METHODS: Two multicentre randomized controlled trials (ISRCTN10351276) were performed: one randomly assigned 3196 individuals who had previously participated in colorectal screening to receive a FOBT kit at home or a standard invitation; in the second, 4219 people aged 50-69 years who did not respond to a screening invitation were either sent a FOBT or a standard recall letter. The cost per returned kit was calculated in each arm. RESULTS: Participation was higher with direct FOBT mailing in both trials: relative risk 1.11 (95% CI 1.06-1.17) and 1.36 (95% CI 1.16-1.60) for previous responders and non-responders, respectively. The cost per returned kit for previous responders ranged from 4.24€ to 16.10€, and from 3.29€ to 7.36€ with FOBT mailing and standard invitation, respectively, not including staff costs; for non-responders it ranged from 17.13€ to 46.80€, and from 7.36€ to 18.30€ with FOBT mailing and standard recall, respectively. CONCLUSIONS: The FOBT mailing strategy modestly increased participation. This method can be used on a population of previous responders to reduce personnel costs and workload. When used as a reminder to non-responders, this method increases costs. | Male | Humans | Patient Participation | Female | OK |
| Carmelo | Aguirre | Montserrat | García | Lucía | Ordóñez | Farmacia Hospitalaria: Órgano Oficial De Expresión Científica De La Sociedad Española De Farmacia Hospitalaria | 2013-02-00 2013 Jan-Feb | The Spanish Pharmacovigilance System for Medicinal Products for Human Use, integrated by regional centers of pharmacovigilance coordinated by the Spanish Agency for Medicines and Health Products, is responsible for developing the Program of Spontaneous Reporting of Suspected Adverse Drug Reactions in our country. Although, until now, reports were only requesting to health professionals, the current understanding of the role of patients in the clinical setting and the experience gained in other countries of our environment, have demonstrated the convenience of developing active participation systems to patients in the reporting of suspected adverse drug reactions. In addition, this is taking into account in the new European legislation on pharmacovigilance. | Humans | Patient Participation | Internet | Europe | OK |
| Paul B. | Greenberg |  |  |  |  | Archives of Ophthalmology | 2012-07-00 Jul 2012 | | Humans | Patient Participation | Questionnaires | United States | OK |
| Jonathan | Samuels | George | Luta | Theodore | Pincus | Arthritis Care & Research | 2014-06-00 Jun 2014 | OBJECTIVE: To analyze discordance between global estimates by patients (PATGL) and their physicians (DOCGL) according to demographic and self-report variables on a Multidimensional Health Assessment Questionnaire (MDHAQ) in patients with many rheumatic diseases seen in usual care. METHODS: Each patient completed an MDHAQ at each visit, which includes scores for physical function, pain, and PATGL, each found on the traditional Health Assessment Questionnaire (HAQ), and scores for sleep quality, anxiety, depression, self-report joint count, and fatigue, which are not found on the HAQ. A random visit of 980 patients with any rheumatic diagnosis was analyzed in 3 categories: PATGL=DOCGL (within 2 of 10 units), PATGL>DOCGL (by ≥2 of 10 units), and DOCGL>PATGL (by ≥2 of 10 units), using descriptive statistics and multinomial logistic regression models. RESULTS: Patients included 145 with rheumatoid arthritis, 57 with systemic lupus erythematosus, 173 with osteoarthritis, 348 with other inflammatory diseases, and 257 with other noninflammatory diseases. Overall, PATGL=DOCGL in 509 (52%), PATGL>DOCGL in 371 (38%), and DOCGL>PATGL in 100 (10%). PATGL>DOCGL was associated significantly with older age, female sex, low formal education, Hispanic ethnicity, not working, high MDHAQ physical function and pain scores, and high scores for fatigue, poor sleep, anxiety, depression, and self-report joint count, which are not available on the HAQ. Pain and fatigue were significant in a final multinomial logistic regression; the other variables may raise awareness of discordance to clinicians. CONCLUSION: Global estimates of patients indicated significantly poorer status than estimates of their physicians in 38% of 980 patients with rheumatic conditions, and were associated with demographic and MDHAQ scores, 5 of which are not available on the HAQ. | Male | Humans | Patient Participation | Questionnaires | OK |
| Thomas | Almdal | Ingrid | Willaing |  |  | Patient Education and Counseling | 2012-05-00 May 2012 | OBJECTIVE: To determine preferences among patients with type 2 diabetes for content and format of patient education. METHODS: Using discrete choice methods, we surveyed patients about their preferences for patient education. We investigated preferred content and format regarding education on living well with diabetes, preventing complications, healthy eating, exercising, and psychosocial issues related to diabetes. RESULTS: We obtained usable responses from 2187 patients with type 2 diabetes. Acquiring competencies to live a fulfilling life with diabetes, adjust diet and exercise habits, and prevent complications was significantly more highly valued than was simply being informed about these topics. Patients preferred to be involved in the planning of their diabetes care and valued individually tailored content higher than prescheduled content. Women and younger patients found diet and exercise significantly more important than did men, and patients with poorly controlled diabetes valued all education and support more highly than did patients in better control. CONCLUSION: Patients with type 2 diabetes prefer to be actively involved in educational activities, to develop competencies to prevent and manage complications, and to involve their social network in supporting them. PRACTICE IMPLICATIONS: Future patient education should enhance participation and competence development and include relatives. | Male | Humans | Patient Participation | Questionnaires | OK |
| Richard | Barron | Jason | Legg | Xiaoyan | Li | Value in Health: The Journal of the International Society for Pharmacoeconomics and Outcomes Research | 2014-06-00 Jun 2014 | OBJECTIVES: Rising out-of-pocket costs for cancer patients have increased shared decision making. Clinical guidelines recommend prophylactic granulocyte colony-stimulating factor (G-CSF) for patients receiving chemotherapy with a 20% or greater risk of febrile neutropenia. A discrete choice experiment was conducted to explore breast cancer patients' preferences and willingness to pay (WTP) for prophylactic G-CSF to decrease the risk of chemotherapy-induced febrile neutropenia. METHODS: An online discrete choice experiment questionnaire survey of a national US convenience sample of self-reported breast cancer patients with prior chemotherapy treatment was conducted. Sixteen paired G-CSF treatment scenarios, each with four attributes (risk of disruption to chemotherapy schedule due to low white blood cell counts, risk of developing an infection requiring hospitalization, frequency of administration, and total out-of-pocket cost) were presented with a follow-up "no treatment" option. Participant preferences and WTP out of pocket were estimated by logistic regression. RESULTS: Participants (n = 296) preferred G-CSF regimens with lower out-of-pocket costs, lower risk of chemotherapy disruption, lower risk of infection, and greater convenience (one G-CSF injection per chemotherapy cycle). Participants' WTP was $1076 out of pocket per cycle to reduce the risk (high to low) of disrupting their chemotherapy schedule, $884 per cycle to reduce the risk (24% [high] to 7% [low]) of infection, and $851 per cycle to decrease the number of G-CSF injections (11 to 1) per cycle. CONCLUSIONS: Participants highly valued specific features of prophylactic G-CSF treatment including maintaining their chemotherapy schedule, lowering their risk of infection, and reducing the number of injections. Physicians should consider patient preferences to inform the best treatment choices for individual patients. | Decision Making | Humans | Questionnaires | Female | OK |
| Deborah | Schrag | John Z. | Ayanian | Steven B. | Clauser | Journal of the National Cancer Institute | 2014-10-00 Oct 2014 | BACKGROUND: Clinical trials are essential to establish the effectiveness of new cancer therapies, but less than 5% of adults with cancer enroll in trials. In addition to ineligibility or lack of available trials, barriers to enrollment may include limited patient awareness about the option of participation. METHODS: We surveyed a multiregional cohort of patients with lung or colorectal cancer (or their surrogates) three to six months after diagnosis. We assessed whether respondents reported learning that clinical trial participation might be an option, and, if so, with whom they discussed trials. We used logistic regression to assess the association of patient characteristics with discussing trial participation and enrolling in trials. All statistical tests were two-sided. RESULTS: Of 7887 respondents, 1114 (14.1%) reported discussing the possibility of clinical trial participation; most learned about trials from their physicians, and 287 patients (3.6% of all patients, 25.8% of trial discussants) enrolled. Among 2173 patients who received chemotherapy for advanced (stage III/IV lung or stage IV colorectal) cancer, 25.7% discussed trials, and 7.6% (29.5% of trial discussants) enrolled. Discussions were less frequent among older patients, African American or Asian vs white patients, and those with lower incomes and more comorbidity. Enrollment was higher among patients reporting shared vs physician-driven decisions (all P < .05). CONCLUSIONS: In this population-based cohort, only 14% of patients discussed participation in clinical trials. Discussions were more frequent among advanced cancer patients but were still reported by a minority of patients. Strategies to expand access to trials and facilitate patient-provider communication about participation may accelerate development of better cancer therapeutics. | Male | Decision Making | Humans | Patient Participation | OK |
| Angela | Fagerlin |  |  |  |  | Patient Education and Counseling | 2012-05-00 mai 2012 | Objective: To identify patient- and decision-type predictors of two key aspects of informed decision making: discussing the cons (not just the pros) of medical interventions and asking patients what they want to do. Methods: Using data from 2473 members of the DECISIONS survey, a nationally representative sample of U.S. adults age 40+, we used logistic regression analysis to identify which patient characteristics predicted patient reports of healthcare providers discussing cons or eliciting preferences about one of 9 common medical decisions. Results: Multiple demographic characteristics predicted both discussions of cons and elicitations of preferences, although the specific characteristics varied between decision contexts. In particular, African-American respondents reported being more likely to receive a discussion of the cons of cancer screening (OR=1.69, p &lt;0.05) yet less likely to have been asked their opinion about either getting a cancer screening test (OR=0.56, p &lt;0.05) or initiating medications (OR=0.53, p &lt;0.05). Significant cross-decision variations remained even after controlling for patient characteristics. Conclusions: Important disparities in patient communication and involvement appear to exist both between different types of medical decisions and between different types of patients. Practice implications: Providers must make sure to consistently discuss the cons of treatment and to solicit input from all patients, especially African-Americans. (PsycINFO Database Record (c) 2012 APA, all rights reserved). (journal abstract) | Decision Making | patients | Intervention | health disparities | Doublon |
| Angela | Fagerlin |  |  |  |  | Patient Education and Counseling | 2012-05-00 May 2012 | OBJECTIVE: To identify patient- and decision-type predictors of two key aspects of informed decision making: discussing the cons (not just the pros) of medical interventions and asking patients what they want to do. METHODS: Using data from 2473 members of the DECISIONS survey, a nationally representative sample of U.S. adults age 40+, we used logistic regression analysis to identify which patient characteristics predicted patient reports of healthcare providers discussing cons or eliciting preferences about one of 9 common medical decisions. RESULTS: Multiple demographic characteristics predicted both discussions of cons and elicitations of preferences, although the specific characteristics varied between decision contexts. In particular, African-American respondents reported being more likely to receive a discussion of the cons of cancer screening (OR=1.69, p<0.05) yet less likely to have been asked their opinion about either getting a cancer screening test (OR=0.56, p<0.05) or initiating medications (OR=0.53, p<0.05). Significant cross-decision variations remained even after controlling for patient characteristics. CONCLUSIONS: Important disparities in patient communication and involvement appear to exist both between different types of medical decisions and between different types of patients. PRACTICE IMPLICATIONS: Providers must make sure to consistently discuss the cons of treatment and to solicit input from all patients, especially African-Americans. | Male | Decision Making | Humans | Patient Participation | OK |
| Richard L. | Kravitz |  |  |  |  | Patient Education and Counseling | 2010-11-00 Nov 2010 | OBJECTIVE: To examine how online depression support group members respond to direct-to-consumer (DTC) antidepressant advertising. METHODS: Survey of 148 depression forum members, administered via an online questionnaire. RESULTS: Chronicity was high, as 79.1% had received a diagnosis of depression 3 or more years earlier. Respondents reported seeing advertisements for an average of 4.3 of seven brands investigated. A majority rated the information quality of these advertisements as "poor" or "fair." Attitudes toward antidepressant advertisements were neutral (mean: 2.96 on a five-point scale). More than half (52.4%) visited official websites provided in these advertisements, 39.9% had talked with a doctor after seeing an advertisement, 20.3% made an advertisement-induced prescription request, and 25.7% said these advertisements reminded them to take their antidepressants. Amount of attention given to these advertisements correlated positively with belief in the brain chemical imbalance causal model, but belief in this model did not predict prescription requests. CONCLUSION: Awareness of DTC antidepressant advertisements is high among individuals with depression, but so is skepticism. PRACTICE IMPLICATIONS: Among members of an online support group, these advertisements encourage patient-doctor dialogue, prescription requests, and adherence, but might also reduce the acceptability of psychotherapy and encourage doctor switching in a small number of patients. | Male | Humans | Patient Participation | Physician-Patient Relations | OK |
| Monique J. | Roobol | Fritz H. | Schröder | Chris H. | Bangma | The Journal of Urology | 2010-05-00 May 2010 | PURPOSE: Anxiety and distress may be present in patients with low risk prostate cancer who are on active surveillance. This may be a reason to discontinue active surveillance. MATERIALS AND METHODS: A total of 150 Dutch patients with prostate cancer on active surveillance in a prospective active surveillance study received questionnaires at study inclusion and 9 months after diagnosis. We assessed changes in scores on decisional conflict with the decisional conflict scale, depression with the Center for Epidemiologic Studies Depression Scale, generic anxiety with the State Trait Anxiety Inventory, prostate cancer specific anxiety with the Memorial Anxiety Scale for Prostate Cancer and the self-estimated risk of progression. We explored scores 9 months after diagnosis vs those at study inclusion for physical health (SF-12 physical component summary), personality (Eysenck Personality Questionnaire), shared decision making, prostate cancer knowledge, demographics, medical parameters and prostate specific antigen doubling time during followup. RESULTS: Questionnaires at study inclusion and 9 months after diagnosis were completed by 129 of 150 (86%) and 108 of 120 participants (90%) a median of 2.4 and 9.2 months after diagnosis, respectively. Anxiety and distress at study inclusion were previously found to be generally favorable. Significant but clinically irrelevant decreases were seen in mean scores of the State Trait Anxiety Inventory (p = 0.016), Memorial Anxiety Scale for Prostate Cancer fear of progression subscale (p = 0.005) and the self-estimated risk of progression (p = 0.049). Anxiety and distress 9 months after diagnosis were mainly predicted by scores at study inclusion. Higher Eysenck Personality Questionnaire neuroticism score and an important role of the physician in the treatment decision had additionally unfavorable effects. Good physical health, palpable disease and older age had favorable effects. No association was seen for prostate specific antigen doubling time. Nine men discontinued active surveillance, including 2 due to nonmedical reasons. CONCLUSIONS: Anxiety and distress generally remain favorably low during the first 9 months of surveillance. | Male | Humans | Questionnaires | Middle Aged | OK |
| Renata | Axler | Pascale | Lehoux | Martin | French | Academic Medicine: Journal of the Association of American Medical Colleges | 2013-04-00 Apr 2013 | PURPOSE: To quantify and compare the preferences of researchers and laypeople in Canada regarding the outcomes of basic biomedical research. METHOD: In autumn 2010, the authors conducted a cross-sectional, national survey of basic biomedical researchers funded by Canada's national health research agency and a representative sample of Canadian citizens to assess preferences for research outcomes across five attributes using a discrete choice experiment. Attributes included advancing scientific knowledge (assessed by published papers); building research capacity (assessed by trainees); informing decisions in the health products industry (assessed by patents); targeting economic, health, or scientific priorities; and cost. The authors reduced a fractional factorial design (18 pairwise choices plus an opt-out option) to three blocks of six. They also computed part worth utilities, differences in predicted probabilities, and willingness-to-pay values using nested logit models. RESULTS: Of 3,260 potential researchers, 1,749 (53.65% response rate) completed the questionnaire, along with 1,002 citizens. Researchers and citizens prioritized high-quality scientific outcomes (papers, trainees) over other attributes. Both groups disvalued research targeted at economic priorities relative to health priorities. Researchers granted a premium to proposals targeting scientific priorities. CONCLUSIONS: Citizens and researchers fundamentally prioritized the same outcomes for basic biomedical research. Notably, they prioritized traditional scientific outcomes and disvalued the pursuit of economic returns. These findings have implications for how academic medicine assigns incentives and value to basic health research and how biomedical researchers and the public may jointly contribute to setting the future research agenda. | Male | Humans | Patient Participation | Questionnaires | OK |
| David E. | Edelman | Patricia H. | Hartwell | Eugene Z. | Oddone | Telemedicine Journal and E-Health: The Official Journal of the American Telemedicine Association | 2010-06-00 Jun 2010 | OBJECTIVE: The Veterans Health Administration has developed My HealtheVet (MHV), a Web-based portal that links veterans to their care in the veteran affairs (VA) system. The objective of this study was to measure diabetic veterans' access to and use of the Internet, and their interest in using MHV to help manage their diabetes. MATERIALS AND METHODS: Cross-sectional mailed survey of 201 patients with type 2 diabetes and hemoglobin A(1c) > 8.0% receiving primary care at any of five primary care clinic sites affiliated with a VA tertiary care facility. Main measures included Internet usage, access, and attitudes; computer skills; interest in using the Internet; awareness of and attitudes toward MHV; demographics; and socioeconomic status. RESULTS: A majority of respondents reported having access to the Internet at home. Nearly half of all respondents had searched online for information about diabetes, including some who did not have home Internet access. More than a third obtained "some" or "a lot" of their health-related information online. Forty-one percent reported being "very interested" in using MHV to help track their home blood glucose readings, a third of whom did not have home Internet access. Factors associated with being "very interested" were as follows: having access to the Internet at home (p < 0.001), "a lot/some" trust in the Internet as a source of health information (p = 0.002), lower age (p = 0.03), and some college (p = 0.04). Neither race (p = 0.44) nor income (p = 0.25) was significantly associated with interest in MHV. CONCLUSIONS: This study found that a diverse sample of older VA patients with sub-optimally controlled diabetes had a level of familiarity with and access to the Internet comparable to an age-matched national sample. In addition, there was a high degree of interest in using the Internet to help manage their diabetes. | Male | Humans | Patient Participation | Questionnaires | OK |
| J. | Ameen | W. | Daly |  |  | European Journal of Cancer Care | 2011-03-00 Mar 2011 | Traditionally healthcare providers have determined how healthcare resources should be allocated. However, in recent years, healthcare users have increasingly been invited to identify those priorities important to them. Yet, healthcare users' priorities do not always match the priorities set by healthcare providers. In Greece, there has not been any research to measure the extent of agreement between healthcare providers and users on cancer care priorities. This project aimed to identify the priorities of healthcare users and providers with regards to the areas of cancer care that needed to be developed or improved in Greece and to examine the extent of agreement between the two groups. Two parallel Delphi surveys were conducted to identify the priorities and arrive at a consensus. Thirty participants formed the panel of healthcare providers and 30 participants the panel of healthcare users. The participants identified 18 key cancer care areas that require development or improvement; however, there were significant differences on the prioritisation between the two panels. It is encouraging that 18 key areas were shared by both panels. These areas can form an agenda for further exploration. Healthcare users can participate in developing priorities for cancer care; however, education is required so that they are better able to make informed choices. | Male | Humans | Patient Participation | Questionnaires | OK |
| Jörg | Haasenritter | Stefan | Bösner | Erika | Baum | BMC family practice | 2011-00-00 2011 | BACKGROUND: Most guidelines concentrate on investigations, treatment, and monitoring instead of patient history and clinical examination. We developed a guideline that dealt with the different aetiologies of chest pain by emphasizing the patient's history and physical signs. The objective of this study was to evaluate the guideline's acceptance and feasibility in the context of a practice test. METHODS: The evaluation study was nested in a diagnostic cross-sectional study with 56 General Practitioners (GPs) and 862 consecutively recruited patients with chest pain. The evaluation of the guideline was conducted in a mixed method design on a sub-sample of 17 GPs and 282 patients. Physicians' evaluation of the guideline was assessed via standardized questionnaires and case record forms. Additionally, practice nursing staff and selected patients were asked for their evaluation of specific guideline modules. Quantitative data was analyzed descriptively for frequencies, means, and standard deviations. In addition, two focus groups with a total of 10 GPs were held to gain further insights in the guideline implementation process. The data analysis and interpretation followed the standards of the qualitative content analysis. RESULTS: The overall evaluation of the GPs participating in the evaluation study regarding the recommendations made in the chest pain guideline was positive. A total of 14 GPs were convinced that there was a need for this kind of guideline and perceived the guideline recommendations as useful. While the long version was partially criticized for a perceived lack of clarity, the short version of the chest pain guideline and the heart score were especially appreciated by the GPs. However, change of clinical behaviour as consequence of the guideline was inconsistent. While on a concrete patient related level, GPs indicated to have behaved as the guideline recommended, the feedback on a more general level was heterogeneous. Several suggestions to improve guideline implementation were made by participating physicians. Due to the small number of practice nursing staff evaluating the flowchart and patients remembering the patient leaflet, no valid results regarding the flowchart and patient leaflet modules could be reported. CONCLUSIONS: Overall, the participating GPs perceived the guideline recommendations as useful to increase awareness and to reflect on diagnostic issues. Although behaviour change in consequence of the guideline was not reported on a general level, guidelines on history taking and the clinical examination may serve an important conservative and practical function in a technology driven environment. Further research to increase the implementation success of the guideline should be undertaken. | Male | Humans | Patient Participation | Questionnaires | OK |
| Desmond | Wright |  |  |  |  | Age and Ageing | 2014-05-00 May 2014 | BACKGROUND: patient experience is now a key parameter in health care. Yet, very little is known about the possible impact of dentist-patient relationships on patient-centred outcomes including older peoples' oral health-related quality of life (OHRQoL). OBJECTIVE: this study assessed the relationship between OHRQoL and dentist-patient relationships related to perceived unmet dental needs; shared decision-making; time spent discussing oral health problems; respect and confidence and trust. PARTICIPANTS: older people aged 65 years and over living in East London, U.K. in 2011. METHODS: a cross-sectional study using stratified random sampling recruited a representative sample of older people (n = 772). PARTICIPANTS completed an oral examination and a structured questionnaire including the Oral Health Impact Profile-14 (OHIP-14) measuring OHRQoL and five dentist-patient relationship questions taken from the U.K. 2009 Adult Dental Health Survey. Multivariate Poisson regressions modelled the association between OHRQoL and dentist-patient factors adjusting for socio-demographic factors, clinical oral indicators, and dental attendance. RESULTS: having a perceived unmet need for dental treatment (PRR = 1.84; 95% CI: 1.32, 2.56) and expressing a lack of trust and confidence in one's dentist (PRR = 1.74; 95% CI: 1.01, 2.98) were significant predictors of poor OHRQoL among older people. CONCLUSIONS: these findings suggest that older people with unmet dental needs and those who expressed a lack of trust and confidence in their dentist were more likely to experience poor OHRQoL reinforcing the importance of the dental patient experience in healthy ageing and well-being. | Male | Humans | Questionnaires | Female | OK |
|  |  |  |  |  |  | Health Psychology: Official Journal of the Division of Health Psychology, American Psychological Association | 2011-05-00 May 2011 | OBJECTIVES: Doctors have been increasingly encouraged to involve patients in decision making rather than pursuing the paternalistic model in which they make the decisions for their patients. But do patients want to participate in making decisions about their health? Is there a relationship between their preferences for shared decision making and numeracy skills? Are those preferences different in countries with different medical systems, and for different age groups? Extant studies cannot answer these questions because most are based on nonprobabilistic, highly selective patient samples that prevent generalizations to a broader population. DESIGN: In a survey on probabilistic national samples in the United States and Germany, we interviewed participants with low numeracy skills (Germany: n = 127, mean numeracy = 37; United States: n = 117, mean numeracy = 36) and high numeracy skills (Germany: n = 133, mean numeracy = 96; U.S.: n = 121, mean numeracy = 91). MAIN OUTCOME MEASURES: Usual and preferred role in medical decision making. RESULTS: A significant number of people with low numeracy in both the United States (35%, SE = 8.2) and Germany (30%, SE = 6.1) preferred to be more passive than they currently were. High-numeracy people, in contrast, were mostly satisfied with their current role. On average, Americans were more active than Germans. Middle-aged participants preferred to be more active compared to both younger and older ones. CONCLUSIONS: Shared decision-making preferences are related to numeracy skills, country, and age. Education efforts to increase numeracy, as well as using nonquantitative communication formats, may foster involvement of low-numeracy patients in decisions about their health. | Male | Decision Making | Humans | Patient Participation | OK |
| Uwe | Koch | Sylke | Andreas |  |  | Clinical Rehabilitation | 2011-07-00 Jul 2011 | BACKGROUND: Besides the measurement of symptoms, information on functioning and disability in daily routines expands the description of impairments in patients with anxiety disorders. Therefore, the measurement of activities and participation, as theoretically included in the International Classification of Functioning, Disability and Health (ICF) is of increasing importance. The objective of this study is to give an overview over the implementation of the concept 'activities and participation' in outcome measurements of patients with anxiety disorders. METHODS: Based on a literature review in PubMed and PsycInfo databases and including 200 papers, this study analyses 65 outcome instruments used in randomized controlled trials in anxiety disorders according to their content related to the ICF domain of 'Activities and participation'. RESULTS: Instruments used in trials evaluating outcome in patients with anxiety disorder only partially address activities and participation. Over all 65 instruments, the chapters on 'Interpersonal interactions and relationships' and 'Mobility' were most frequently included in measurements. CONCLUSIONS: The identification of relevant ICF categories (core sets) according to activities and participation and especially interpersonal interactions and relationships as well as mobility, can be a basis for describing functioning and evaluating treatment in anxiety disorders, helping to improve outcome assessment in clinical practice. | Male | Humans | Patient Participation | Female | OK |
| Peter J. | Schulz |  |  |  |  | Patient Education and Counseling | 2013-08-00 Aug 2013 | OBJECTIVES: To evaluate the effectiveness of an Internet-based patient education intervention, which was designed upon principles of personalization and participatory design. METHODS: Fifteen months after the first release of the website, 209 fibromyalgia patients recruited through health professionals completed an online questionnaire to assess patients' use of the website, health knowledge, self-management behavior, and health outcomes. These constructs were combined into an a-priory model that was tested using a structural equation modeling approach. RESULTS: Results show that the usage of certain tools of the website - designed and personalized involving the end users - impacts patients' health knowledge, which in turn impacts self-management. Improvements in self-management ultimately lower the impact of Fibromyalgia Syndrome leading to better health outcomes. CONCLUSION: This study empirically confirmed that the adoption of a participatory approach to the design of eHealth interventions and the use of personalized contents enhance the overall effectiveness of systems. PRACTICE IMPLICATIONS: More time and effort should be invested in involving patients in the preliminary phases of the development of Internet-based patient education interventions and in the definition of models that can guide the systems' evaluation beyond technology-related variables such as usability, accessibility or adoption. | Male | Humans | Patient Participation | Questionnaires | OK |
| Elizabeth A. | Jacobs | Sara | Zerbel | Martha E. | Gaines | Infection Control and Hospital Epidemiology: The Official Journal of the Society of Hospital Epidemiologists of America | 2012-12-00 Dec 2012 | | Male | Humans | Patient Participation | Physician-Patient Relations | OK |
| Jos W. R. | Twisk | Marie-Paule | Dain | Frank J. | Snoek | Patient Education and Counseling | 2011-11-00 Nov 2011 | OBJECTIVE: To explore across countries the extent to which physicians understand Type 2 diabetes patients' perceptions of seriousness, worries about complications, emotional distress, and needs for care improvement. METHODS: Cross-sectional data were collected in a multinational survey (SHARED). Type 2 diabetes patients (n=1609), general practitioners (n=818) and diabetes specialists (n=697) from eight countries were included. Data were gathered online and via telephone interviews. Responses from patients and professionals were compared using descriptive statistics and multilevel analyses. RESULTS: Patients generally perceived diabetes as a serious condition and reported moderate distress. Physicians tended to underestimate patients' perceived seriousness, while overestimating their level of distress. Physicians had difficulty estimating which diabetes complications concerned patients most, and what they needed to feel more confident about their diabetes. Patients did not wish for more consultation time, but rather active involvement, information and easy access to their physician. CONCLUSION: Results of this large survey highlight the importance of patient involvement and shared decision making. PRACTICE IMPLICATIONS: Further improvement of patient-provider communication as a basis for shared responsibilities and achieving optimal treatment outcomes is needed. With the growing numbers of diabetes patients worldwide, task delegation should be considered, in the framework of a multidisciplinary diabetes care model. | Male | Emotions | Humans | Physician-Patient Relations | OK |
| Jos W.R. | Twisk | Marie-Paule | Dain | Frank J. | Snoek | Patient Education and Counseling | 2011-11-00 novembre 2011 | Objective: To explore across countries the extent to which physicians understand Type 2 diabetes patients’ perceptions of seriousness, worries about complications, emotional distress, and needs for care improvement. Methods: Cross-sectional data were collected in a multinational survey (SHARED). Type 2 diabetes patients (n = 1609), general practitioners (n = 818) and diabetes specialists (n = 697) from eight countries were included. Data were gathered online and via telephone interviews. Responses from patients and professionals were compared using descriptive statistics and multilevel analyses. Results: Patients generally perceived diabetes as a serious condition and reported moderate distress. Physicians tended to underestimate patients’ perceived seriousness, while overestimating their level of distress. Physicians had difficulty estimating which diabetes complications concerned patients most, and what they needed to feel more confident about their diabetes. Patients did not wish for more consultation time, but rather active involvement, information and easy access to their physician. Conclusion: Results of this large survey highlight the importance of patient involvement and shared decision making. Practice implications: Further improvement of patient–provider communication as a basis for shared responsibilities and achieving optimal treatment outcomes is needed. With the growing numbers of diabetes patients worldwide, task delegation should be considered, in the framework of a multidisciplinary diabetes care model. (PsycINFO Database Record (c) 2012 APA, all rights reserved). (journal abstract) | physicians | Client Attitudes | type 2 diabetes | patients perceptions | Doublon |
| Fernando | Herrera |  |  |  |  | International Journal of Telemedicine and Applications | 2011-00-00 2011 | The purpose of our study was to determine the factors that influence the use of telemedicine consultation by primary care physicians (PCPs) in the management of patients with problem wounds. A short questionnaire was administered to thirty-six PCPs who referred to our Wound Care Program within one year. Participants were asked to rate the importance of specific concerns and benefits regarding the role of wound care surgical specialist (WCSS) and the use of telemedicine consults prior to possible face-to-face consultation. Sixty percent of respondents felt comfortable with telemedicine consultation based on recommendations alone. The total number of patients referred for telemedicine consult was 230, and face-to-face consultation with a WCSS was offered and arranged for 30% of patients. The perception of shared decision making, streamlining patient care, and an opportunity for followup were all highly ranked benefits. The majority of PCPs (93%) agreed that telemedicine wound care consult is a useful tool in their practice and would continue to use the telemedicine consult service. | | | | | OK |
| Sally | Jennings | Elizabeth A. | Maylor |  |  | Patient Education and Counseling | 2012-08-00 Aug 2012 | OBJECTIVE: To investigate whether general practitioners (GPs) and patients agree on what constitutes the best evidence for the effectiveness of treatments. METHODS: GPs and members of the public aged 18-83 read five scenarios describing comparisons between hypothetical treatments for common ailments. Each scenario reported that one treatment was the more effective, as determined by randomised controlled trial (RCT), audit of treatment outcomes from many doctors' patients, a single doctor's clinical experience, a friend's experience, or a web-based sales site. Participants rated how confident they would be that the treatment reported to be more effective would work for them. RESULTS: All participants had least confidence in the web-based sales site, more confidence in a friend's experience and more still in one doctor's experience. For doctor's experience, audit and RCT, amongst the public there were some differences by age but, importantly, only GPs had most confidence in evidence from an RCT. CONCLUSION: GPs may treat evidence from RCTs as the gold standard while members of the public (their patients) may not afford it that same respect. PRACTICE IMPLICATIONS: GPs engaged in shared decision-making should be alert to possible differences from their patients in the weight given to different types of evidence. | Male | Decision Making | Humans | Questionnaires | OK |
| Chris L. | Paul | Rob W. | Sanson-Fisher | Catherine A. | D'Este | Psycho-Oncology | 2013-12-00 Dec 2013 | OBJECTIVE: A patient-centred approach to discussing life expectancy with cancer patients is recommended in Western countries. However, this approach to eliciting and meeting patient preferences can be challenging for clinicians. The aims of this study were the following: (i) to examine cancer patients' preferences for life expectancy disclosure; and (ii) to explore agreement between cancer patients' preferences for, and perceived experiences of, life expectancy disclosure. METHODS: Cancer patients undergoing radiotherapy treatment in metropolitan Australia completed a cross-sectional touchscreen computer survey including optional questions about their life expectancy disclosure preferences and experiences. RESULTS: Of the 208 respondents, 178 (86%) indicated that they would prefer their clinician to ask them before discussing life expectancy, and 30 (14%) indicated that they would prefer others (i.e. clinicians, family) to decide whether they were given life expectancy information. Of the 175 respondents who were classified as having a self- determined or other-determined disclosure experience, 105 (60%) reported an experience of life expectancy disclosure that was in accordance with their preferences. Cohen's κ was -0.04 (95% CI, -0.17, 0.08), indicating very poor agreement between patients' preferences for and perceived experiences of life expectancy disclosure (p = 0.74). CONCLUSIONS: In light of patient-centred prognosis disclosure guidelines, our findings of a majority preference for, and experience of, a self-determined approach to life expectancy disclosure amongst radiation oncology patients are encouraging. However, poor agreement between preferences and experiences highlights that additional effort from clinicians is required in order to achieve a truly patient-centred approach to life expectancy disclosure. | Male | Humans | Patient Participation | Physician-Patient Relations | OK |
| Claudine | Burton-Jeangros | Bernice S. | Elger |  |  | Journal of Medical Ethics | 2014-05-00 May 2014 | OBJECTIVE: Routine prenatal screening for Down syndrome challenges professional non-directiveness and patient autonomy in daily clinical practices. This paper aims to describe how professionals negotiate their role when a pregnant woman asks them to become involved in the decision-making process implied by screening. METHODS: Forty-one semi-structured interviews were conducted with gynaecologists-obstetricians (n=26) and midwives (n=15) in a large Swiss city. RESULTS: Three professional profiles were constructed along a continuum that defines the relative distance or proximity towards patients' demands for professional involvement in the decision-making process. The first profile insists on enforcing patient responsibility, wherein the healthcare provider avoids any form of professional participation. A second profile defends the idea of a shared decision making between patients and professionals. The third highlights the intervening factors that justify professionals' involvement in decisions. CONCLUSIONS: These results illustrate various applications of the principle of autonomy and highlight the complexity of the doctor-patient relationship amidst medical decisions today. | Decision Making | Humans | Physician-Patient Relations | Questionnaires | OK |
| Hardyanto | Soebono | Jan | Van Dalen | Cees | Van der Vleuten | Advances in Health Sciences Education: Theory and Practice | 2011-03-00 Mar 2011 | Doctor-patient communication has been extensively studied in non-Western contexts and in relation to patients' cultural and education backgrounds. This study explores the perceived ideal communication style for doctor-patient consultations and the reality of actual practice in a Southeast Asian context. We conducted the study in a teaching hospital in Indonesia, using a qualitative and a quantitative design. In-depth interviews were conducted with ten internal medicine specialists, ten internal medicine residents, 16 patients in two groups based on education level and ten most senior medical students. The contributions of doctors and patients to the communication during consultations were observed and rated quantitatively by thirty internal medicine residents, 393 patients with different educational backgrounds and ten senior medical students. The 'informed and shared decision making' is the central observation in this quantitative study. The results of the interviews showed that Southeast Asian stakeholders are in favor of a partnership style of communication and revealed barriers to achieving this: doctors and patients are not prepared for a participatory style and high patient load due to an inefficient health care system does not allow sufficient time for this type of communication. The results of the quantitative study showed a sharp contrast between observed and ideal communication styles. A paternalistic style seems to prevail, irrespective of patients' educational background. We found a sharp conflict between ideal and reality concerning doctor-patient communication in a Southeast Asian context. Further studies should examine ways to change the prevailing communication style in the desired direction. | Male | Humans | Patient Participation | Physician-Patient Relations | OK |
| Cees Pm | Van Der Vleuten | |  |  |  | Patient Education and Counseling | 2011-12-00 Dec 2011 | OBJECTIVE: To explore the relationship between the style of doctor-patient communication and patients' educational background in a Southeast Asian teaching hospital setting using the Roter Interaction Analysis System (RIAS). METHODS: We analyzed a total of 245 audio-taped consultations involving 30 internal medicine residents with 7-10 patients each in the internal medicine outpatient clinics. The patients were categorized into a group with a high and a group with a low educational level. We ranked the data into 41 RIAS utterances and RIAS-based composite categories in order of observed frequency during consultations. RESULTS: The residents invariantly used a paternalistic style irrespective of patients' educational background. The RIAS utterances and the composite categories show no significant relationship between communication style and patients' educational level. CONCLUSION: Doctors in a Southeast Asian country use a paternalistic communication style during consultations, regardless of patients' educational background. PRACTICE IMPLICATION: To approach a more partnership doctor-patient communication, culture and clinical environment concern of Southeast Asian should be further investigated. | Male | Humans | Patient Participation | Physician-Patient Relations | OK |
| Michael R. | Lowe | Antonia P. | O'Planick | Doug W. | Bunnell | Eating Behaviors | 2010-01-00 Jan 2010 | Attrition is a pervasive problem in eating disorders research. This study examined whether those who do and do not respond to follow-up assessments differ before or during treatment. Participants (N=268) receiving residential eating disorders treatment were categorized according to those who did ("responders," n=152) and did not ("non-responders," n=116) complete a one-month follow-up assessment. Among participants diagnosed with bulimia nervosa (n=136), responders exhibited significantly higher scores than non-responders at intake on restraint, weight concern, eating concerns, body dissatisfaction, drive for thinness, and depressive symptoms, and had significantly less improvement in eating concerns during treatment. Among participants with anorexia nervosa (n=132), there were no significant differences between responders and non-responders at intake or in treatment improvement. Research on bulimia nervosa treatment based on responders to follow-up assessments may underestimate the amount of improvement that patients experience. | Humans | Patient Participation | Questionnaires | Female | OK |
| Dan | Greenberg | Joshua T. | Cohen | Gregory | Rossi | Expert Review of Pharmacoeconomics & Outcomes Research | 2013-08-00 Aug 2013 | AIMS: To investigate the relationship between the framing of survival gains and the perceived value of cancer care. METHODS: Through a population-based survey of 2040 US adults, respondents were randomized to one of the two sets of hypothetical scenarios, each of which described the survival benefit for a new treatment as either an increase in median survival time (median survival), or an increase in the probability of survival for a given length of time (landmark survival). Each respondent was presented with two randomly selected scenarios with different prognosis and survival improvements, and asked about their willingness to pay (WTP) for the new treatments. RESULTS: Predicted WTP increased with survival benefits and respondents' income, regardless of how survival benefits were described. Framing therapeutic benefits as improvements in landmark rather than median time survival increased the proportion of the population willing to pay for that gain by 11-35%, and the mean WTP amount by 42-72% in the scenarios we compared. CONCLUSION: How survival benefits are described may influence the value people place on cancer care. | Time Factors | Male | Humans | Patient Participation | Doublon |
| Dan | Greenberg | Joshua T. | Cohen | Gregory | Rossi | Expert Review of Pharmacoeconomics & Outcomes Research | 2013-08-00 Aug 2013 | AIMS: To investigate the relationship between the framing of survival gains and the perceived value of cancer care. METHODS: Through a population-based survey of 2040 US adults, respondents were randomized to one of the two sets of hypothetical scenarios, each of which described the survival benefit for a new treatment as either an increase in median survival time (median survival), or an increase in the probability of survival for a given length of time (landmark survival). Each respondent was presented with two randomly selected scenarios with different prognosis and survival improvements, and asked about their willingness to pay (WTP) for the new treatments. RESULTS: Predicted WTP increased with survival benefits and respondents' income, regardless of how survival benefits were described. Framing therapeutic benefits as improvements in landmark rather than median time survival increased the proportion of the population willing to pay for that gain by 11-35%, and the mean WTP amount by 42-72% in the scenarios we compared. CONCLUSION: How survival benefits are described may influence the value people place on cancer care. | Time Factors | Male | Humans | Patient Participation | OK |
| Jon D. | Lurie | Wenyan | Zhao | Anna | Tosteson | Spine | 2010-02-15 Feb 15, 2010 | STUDY DESIGN: A subanalysis study. OBJECTIVE: The purpose of this study was to determine the impact of multilevel lumbar stenosis with or without degenerative spondylolisthesis compared to single level disease on patients' baseline symptoms and clinical outcomes over time. SUMMARY OF BACKGROUND DATA: Previous studies have demonstrated better clinical outcomes with surgery than nonoperative treatment in patients with spinal stenosis with or without degenerative spondylolisthesis. However, the impact of multilevel stenosis has not been studied in these patients. METHODS: The results from a multicenter randomized and observational study, the Spine Patient Outcomes Research Trial (SPORT) comparing surgical versus nonoperative treatment for spinal stenosis with or without spondylolisthesis, were analyzed. The primary outcomes measures were the Bodily Pain and Physical Function scales of the Medical Outcomes Study 36-item Short-Form General Health Survey (SF-36) and the modified Oswestry Disability Index at 6 weeks, 3 months, 6 months, 1 year, and 2 years. Secondary outcome measures included the stenosis bothersomeness index, leg pain bothersomeness, low back pain bothersomeness, and patient satisfaction. RESULTS: In this subanalysis of SPORT data, multilevel spinal stenosis did not demonstrate worse baseline symptoms or worse treatment outcomes in isolated spinal stenosis; however, if concomitant degenerative spondylolisthesis existed, patients with only single level stenosis tended to improve more than those with multilevel stenosis, particularly after surgery. CONCLUSION: Patients with spinal stenosis without associated degenerative spondylolisthesis or scoliosis can be managed nonoperatively irrespective of the number of levels involved. If surgery is performed, the number of levels treated does not predict outcome. In contrast, patients with concomitant degenerative spondylolisthesis and single level stenosis do better surgically than those with additional levels of stenosis. This study emphasizes the importance of shared decision-making between the physician and patient when considering treatment for spinal stenosis. | Time Factors | Male | Humans | Patient Satisfaction | OK |
| Ana J. | Tebar-Martínez | Ángel | Asúnsolo-Del Barco | |  | Clinical Orthopaedics and Related Research | 2014-08-00 Aug 2014 | BACKGROUND: Preoperative psychologic distress is considered to be a risk factor for clinical dissatisfaction stemming from persistent pain and physical limitations after elective orthopaedic procedures such as lower-extremity arthroplasty. However, the degree to which psychologic distress, specifically in the form of anxiety and depression, influences surgical results has been poorly characterized. QUESTIONS/PURPOSES: We analyzed the effect of preoperative psychologic distress on changes in pain, function, and quality of life 1 year after elective TKA. METHODS: In this prospective cohort study, we assessed patients who underwent TKAs in 2009 and 2010. Before surgery, patients completed the Folstein Mini Mental Test, the Hospital Anxiety and Depression Scale (HAD), The Knee Society Score(©), the WOMAC quality-of-life questionnaire, and the VAS for pain. The patients were divided into two groups based on the degree of psychologic distress on the HAD Scale, and the groups were compared in terms of the above-listed clinical outcomes tools 1 year after surgery using multivariate linear models. Two hundred sixty-three patients met the inclusion criteria, and 202 (77%) completed the study protocol. RESULTS: The presence of preoperative psychologic distress did not influence 1-year postoperative pain assessment (average reduction in pain, 40.33; 95% CI, 36.9-43.8; p = 0.18). The only factor influencing change in pain experienced by patients was the preoperative pain recorded (R(2) = 0.31; β = -0.82; p < 0.001). The patients experiencing preoperative psychologic distress obtained poorer outcomes in function (R(2) = 0.16; β = -5.62; p = 0.001) and quality of life (R(2) = 0.09; β = -0.46; p < 0.001) 1 year after receiving TKA. CONCLUSIONS: The presence of preoperative psychologic distress is associated with worse 1-year outcomes for function and quality of life in patients undergoing TKA. Interventions designed to reduce psychologic distress may be indicated for patients to undergo this type of surgery, and incorporation of these data into discussions with patients may facilitate informed and shared decision making regarding the surgical treatment of knee osteoarthritis. LEVEL OF EVIDENCE: Level II, therapeutic study. See the Instructions for Authors for a complete description of levels of evidence. | Time Factors | Male | Humans | Questionnaires | OK |
| H. | Stützer | S. H. M. | Derman | N. V. | Lichtenstein | European Journal of Dental Education: Official Journal of the Association for Dental Education in Europe | 2013-02-00 Feb 2013 | OBJECTIVES: Aim of this RCT was to evaluate whether the added use of a decision board (DB) during shared decision-making improves patients' knowledge as for different treatment options and overall satisfaction with the consultation. METHODS: Forty-nine undergraduate students were trained in shared decision-making (SDM) and evaluated by an Objective Structured Clinical Examination (OSCE). According to their test results, all participants were randomly allocated to either the test- (DB) or the control-group (Non-DB). Both groups performed SDM with patients showing a defect in a posterior tooth (Class-II defect). Prior to the interview, patients of the DB group were given the decision aid for review. In the Non-DB group, patients were consulted without additional aids. After treatment decision, a questionnaire was completed by all patients to measure knowledge (costs, survival rate, characteristics and treatment time) and overall satisfaction with the consultation. Fifty DB patients and 31 Non-DB patients completed the questionnaire. RESULTS: DB patients (n = 50) demonstrated a statistically significant increase in knowledge compared to the Non-DB group (n = 31) (Mann-Whitney U-test; DB group = 10.04; Non-DB group = 4.16; P = 0.004). There was no significant difference between groups regarding satisfaction with the consultation (t-test; P > 0.05). CONCLUSIONS: During the shared decision-making process, the use of a decision board yielding information about Class-II treatment options leads to a significantly higher patient knowledge compared to knowledge gained through consultation alone. It is therefore desirable to provide DBs for dental diagnoses with several treatment options to increase transparency for the patient. | Male | Decision Making | Decision Support Techniques | Humans | OK |
| Elizabeth | Broadbent | Craig | MacKenzie | Karl | Cole | Journal of Primary Health Care | 2011-03-00 Mar 2011 | INTRODUCTION: Explaining what cardiovascular disease (CVD) risk means and engaging in shared decision-making regarding risk factor modification is challenging. An electronic CVD risk visualisation tool containing multiple risk communication strategies (Your Heart Forecast) was designed in 2009. AIM: To assess whether this tool facilitated explaining CVD risk to primary care patients. METHODS: Health professionals who accessed a Primary Health Organisation website or who attended educational peer groups over a three-month period were invited to complete questionnaires before and after viewing a four-minute video about the tool. Respondents were asked to make an informed guess of the CVD risk of a 35-year-old patient (actual CVD risk 5%) and rate the following sentence as being true or false: 'If there were 100 people like Mr Andrews, five would go on to have a cardiac event in the next five years.' They also were asked to rank their understanding of CVD risk and confidence in explaining the concept to patients. RESULTS: Fifty health professionals (37 GPs, 12 practice nurses, one other) completed before and after questionnaires. Respondents' CVD risk estimates pre-video ranged from <5% to 25% and nine rated the sentence as being false. After the video, all respondents answered these questions correctly. Personal rankings from zero to 10 about understanding CVD risk and confidence in explaining risk reduced in range and shifted towards greater efficacy. DISCUSSION: Whether this tool facilitates discussions of CVD risk with patients and improves patient understanding and lifestyle behaviour needs to be evaluated in a randomised trial. | Male | Humans | Physician-Patient Relations | Questionnaires | OK |
| D. | Farewell | J. | Darmanin | J. | Wagstaff | British Journal of Cancer | 2013-04-16 Apr 16, 2013 | BACKGROUND: Recruitment of patients into randomised clinical trials (RCTs) is essential for treatment evaluation. Appreciation of the barriers and drivers towards participation is important for trial design, communication and information provision. METHOD: As part of an intervention to facilitate effective multidisciplinary team communication about RCTs, cancer patients completed two study-specific questionnaires following trial discussions. One questionnaire examined reasons why patients accepted or declined trial entry, the other perceptions about their health-care professionals' (HCPs) information giving. RESULTS: Questionnaires were completed by 74% (358/486) of patients approached; of these 81% (291/358) had joined an RCT, 16% (56/358) had declined and 3% (11/358) were undecided. Trial participation status of the 128 patients not returning questionnaires is unknown. Trial acceptance was not dependent on disease stage, tumour type, sex or age. Satisfaction with trial information and HCPs' communication was generally very good, irrespective of participation decisions. The primary reason given for trial acceptance was altruism (40%; 110/275), and for declining, trust in the doctor (28%; 12/43). Decliners preferred doctors to choose their treatment rather than be randomised (54% vs 39%; P<0.027). Acceptors were more likely to perceive doctors as wanting them to join trials (54% vs 30%; P<0.001). Trial type, that is, standard treatment vs novel or different durations of treatment, also influenced acceptance rates. CONCLUSION: The drivers and barriers to trial participation are partly related to trial design. Unease about randomisation and impact of duration on treatment efficacy are barriers for some. Altruism and HCPs' perceived attitudes are powerful influencing factors. | Male | Humans | Patient Participation | Questionnaires | OK |
| Marie-Anne | Durand | Robert | Newcombe | France | Légaré | Patient Education and Counseling | 2011-04-00 avril 2011 | Background: Current models of the medical consultation emphasize shared decision-making (SDM), whereby the expertise of both the doctor and the patient are recognized and seen to equally contribute to the consultation. The evidence regarding the desirability and effectiveness of the SDM approach is often conflicting. It is proposed that the conflicts are due to the nature of assessment, with current assessments from the perspective of an outside observer. Aims: To empirically assess perceived involvement in the medical consultation using the dyadic OPTION instrument. Method: 36 simulated medical consultations were organized between general practitioners and standardized- patients, using the observer OPTION and the newly developed dyadic OPTION instruments. Results: SDM behaviors observed in the consultations were seen to depend on both members of the doctor and patient dyad, rather than each in isolation. Thus a dyadic approach to measurement is supported. Conclusions: This current study highlights the necessity for a dyadic approach to assessment and introduces a novel research instrument: the dyadic OPTION instrument. (PsycINFO Database Record (c) 2013 APA, all rights reserved). (journal abstract) | Shared decision making | Decision Making | clinical practice | practice | Doublon |
| Marie-Anne | Durand | Robert | Newcombe | France | Légaré | Patient Education and Counseling | 2011-04-00 Apr 2011 | BACKGROUND: Current models of the medical consultation emphasize shared decision-making (SDM), whereby the expertise of both the doctor and the patient are recognised and seen to equally contribute to the consultation. The evidence regarding the desirability and effectiveness of the SDM approach is often conflicting. It is proposed that the conflicts are due to the nature of assessment, with current assessments from the perspective of an outside observer. AIMS: To empirically assess perceived involvement in the medical consultation using the dyadic OPTION instrument. METHOD: 36 simulated medical consultations were organised between general practitioners and standardized- patients, using the observer OPTION and the newly developed dyadic OPTION instruments. RESULTS: SDM behaviours observed in the consultations were seen to depend on both members of the doctor and patient dyad, rather than each in isolation. Thus a dyadic approach to measurement is supported. CONCLUSIONS: This current study highlights the necessity for a dyadic approach to assessment and introduces a novel research instrument: the dyadic OPTION instrument. | Male | Decision Making | Humans | Patient Participation | OK |
| Carol | Harvey | Vera A. | Morgan | Anna | Waterreus | The Australian and New Zealand Journal of Psychiatry | 2012-08-00 Aug 2012 | OBJECTIVE: Participation in mainstream education and employment facilitates both the recovery and the social inclusion of people with psychotic disorders. As part of the second Australian survey of psychosis, we assessed labour force activity and participation in formal education among working age adults with psychotic disorders. METHOD: Data were drawn from a large national community prevalence survey of adults with psychotic disorders. Known as the Survey of High Impact Psychosis (SHIP), it was conducted in seven Australian catchment areas during March to December 2010. Current and past year labour force activity, current employment, past year participation in formal education and vocational training, and key clinical and demographic characteristics were examined in a sample of 1825 participants. RESULTS: Only 22.4% of people with psychotic disorders were found to be employed (either full-time or part-time) in the month prior to the survey. In the previous 12 months, 32.7% were employed at some time. Of those in competitive employment, the majority worked part-time (63.9%), while a quarter worked 38 or more hours per week (23.4%). In terms of educational attainment, 18.4% reported difficulties with reading or writing, while 31.9% completed high school, which represents 12 years of formal education. CONCLUSIONS: The proportion currently employed has remained stable at 22% since the last national survey in 1997. Policy makers and service providers could do more to ensure people with psychotic disorders obtain access to more effective forms of assistance with respect to both their continuing education and employment. More effective vocational and educational interventions for people with psychotic disorders appear to be urgently needed. | Male | Humans | Patient Participation | Female | OK |
|  |  |  |  |  |  | Health Expectations: An International Journal of Public Participation in Health Care and Health Policy | 2012-12-00 Dec 2012 | | Decision Making | Humans | Patient Participation | Questionnaires | OK |
| Jessica | Nijman | Dolf | de Boer |  |  | BMC health services research | 2012-00-00 2012 | BACKGROUND: Educational attainment is strongly related to specific health outcomes. The pathway in which individual patient-provider interactions contribute to (re)producing these inequalities has yet to be studied. In this article, the focus is on differences between less and more highly educated patients in their preferences for and experiences with patient-centred care., e.g. shared decision making, receiving understandable explanations and being able to ask questions. METHODS: Data are derived from several Consumer Quality-index (CQ-index) studies. The CQ-index is a family of standardized instruments which are used in the Netherlands to measure quality of care from the patient's perspective. RESULTS: The educational level of patients is directly related to the degree of importance patients attribute to specific aspects of patient-centred care. It has a minor influence on the experienced level of shared decision making, but not on experiences regarding other aspects of patient-centred care. CONCLUSIONS: All patients regard patient-centred care as important and report positive experiences. However, there is a discrepancy between patient preferences for patient-centred care on one hand and the care received on the other. Less educated patients might receive 'too much', and more highly educated patients 'too little' in the domains of communication, information and shared decision making. | Male | Decision Making | Humans | Physician-Patient Relations | OK |
| Mariscal | Susana | Lori J. | Davidson | Karen | Cook | Psychiatric Rehabilitation Journal | 2011-00-00 2011 | OBJECTIVE: Self-management of psychiatric illness is a central tenet of consumer-directed mental health treatment. While several manualized self-management programs have been developed in recent years, the most widely disseminated is the Wellness Recovery Action Plan (WRAP). This study examined the effects of WRAP participation on psychiatric symptoms, hope, and recovery outcomes for people with severe and persistent mental illness. METHODS: A quasi-experimental study, with an experimental (n=58) and a comparison (n=56) group was conducted. WRAP sessions (8-12 week) were facilitated by one staff person and one peer worker at five community mental health centers in a Midwestern state. The Modified Colorado Symptom Index, the State Hope Scale, and the Recovery Markers Questionnaire (RMQ) were employed at the first and last WRAP sessions, as well as six months following the intervention. Repeated measures analysis of covariance and planned comparisons before and after the intervention were conducted. RESULTS: Findings revealed statistically significant group intervention effects for symptoms and hope, but not for RMQ. Planned comparisons showed statistically significant improvements for the experimental group in psychiatric symptoms and hope after the intervention, while non-significant changes occurred in the comparison group. CONCLUSIONS AND IMPLICATIONS FOR PRACTICE: The study results offer promising evidence that WRAP participation has a positive effect on psychiatric symptoms and feelings of hopefulness. If recovery is the guiding vision for mental health system reform, the study results provide evidence that WRAP programming may warrant a place in the current array of services offered through the publicly funded mental health system. | Male | Humans | Patient Participation | Questionnaires | OK |
| Marit | Kirkevold | Torsten | Lauritzen |  |  | Scandinavian Journal of Public Health | 2011-05-00 May 2011 | AIM: To investigate the reach of the ''Ready to Act'' programme and the 1-year effects on psychological determinants of healthy behaviour: motivation, perceived competence, and activation level. METHODS: A total of 509 adults with dysglycaemia were recruited from general practioners (GPs) in the intensive arm of the Danish Anglo-Danish-Dutch Study of Intensive Treatment in People with Screen-Detected Diabetes in Primary Care (ADDITION) study, a type 2 diabetes screening programme. The participants were randomised to the ''Ready to Act'' programme added on top of GP care (n = 322) or to GP care (n = 187). The core components of the programme were motivation, action experience, informed decision-making, and social involvement conducted in two one-to-one sessions and eight group-meetings (18 hours). The reach of the programme was measured by the proportion of people who signed up. Outcomes were changes in treatment motivation (Treatment Self-Regulation Questionnaire, TSRQ), perceived competence (Perceived Competence Scale, PCS), and activation in chronic care (Patient Activation Measure, PAM). Effect size was the difference between 1-year changes in the randomisation groups analysed by intention-to-treat. RESULTS: A total of 142 (44%) of 322 signed up and 123 (87%) of these completed. At 1 year, the difference in autonomous motivation for behavioural treatment (TSRQ) between the randomisation groups was 1.0 (95% CI 0.1 to 2.0), and the difference in perceived competence changes in healthy diet (PCS-d) was 1.5 (95% CI 0.2 to 2.7). No differences were observed for activation (PAM) between the groups. Subgroup analysis revealed men to benefit more from the intervention than women. CONCLUSIONS: The programme is a promising health-promoting component in prevention and care for people with screen-detected dysglycaemia, as it attracted four of 10 people and had effects on motivation and perceived competence. | Male | Humans | Patient Participation | Questionnaires | OK |
| Timothy | Skinner | Marian E. | Carey | Kathryn | Realf | BMJ (Clinical research ed.) | 2012-00-00 2012 | OBJECTIVE: To measure whether the benefits of a single education and self management structured programme for people with newly diagnosed type 2 diabetes mellitus are sustained at three years. DESIGN: Three year follow-up of a multicentre cluster randomised controlled trial in primary care, with randomisation at practice level. SETTING: 207 general practices in 13 primary care sites in the United Kingdom. PARTICIPANTS: 731 of the 824 participants included in the original trial were eligible for follow-up. Biomedical data were collected on 604 (82.6%) and questionnaire data on 513 (70.1%) participants. INTERVENTION: A structured group education programme for six hours delivered in the community by two trained healthcare professional educators compared with usual care. MAIN OUTCOME MEASURES: The primary outcome was glycated haemoglobin (HbA(1c)) levels. The secondary outcomes were blood pressure, weight, blood lipid levels, smoking status, physical activity, quality of life, beliefs about illness, depression, emotional impact of diabetes, and drug use at three years. RESULTS: HbA(1c) levels at three years had decreased in both groups. After adjusting for baseline and cluster the difference was not significant (difference -0.02, 95% confidence interval -0.22 to 0.17). The groups did not differ for the other biomedical and lifestyle outcomes and drug use. The significant benefits in the intervention group across four out of five health beliefs seen at 12 months were sustained at three years (P<0.01). Depression scores and quality of life did not differ at three years. CONCLUSION: A single programme for people with newly diagnosed type 2 diabetes mellitus showed no difference in biomedical or lifestyle outcomes at three years although there were sustained improvements in some illness beliefs. TRIAL REGISTRATION: Current Controlled Trials ISRCTN17844016. | Male | Humans | Patient Participation | Questionnaires | OK |
| Bart J. M. | Melis-Dankers | Marcel W. M. | Post | Theo P. B. M. | Suurmeijer | Disability and Rehabilitation | 2012-00-00 2012 | PURPOSE: To pilot test the newly developed multidisciplinary group rehabilitation programme Visually Impaired elderly Persons Participating (VIPP). METHOD: A single group pretest-posttest design pilot study included 29 visually impaired persons (≥ 55 years). The intervention (20 weekly meetings) consisted of four components (practical training; education, social interaction, counselling and training of problem-solving skills; individual and group goal setting; home-based exercise programme). Participation was assessed with the Utrecht Scale for Evaluation of Rehabilitation-Participation (USER-P) and the Impact on Participation and Autonomy (IPA) questionnaire at baseline, immediately and 6 months after the intervention. RESULTS: At scale level, no statistical significant changes over time were found whereas the effect size indicated small effects for restrictions and satisfaction with participation, and a medium effect for autonomy outdoors. At item level, improvements tended to occur in frequency of housekeeping, in restrictions in housekeeping and outdoor activities and in satisfaction with the partner relationship. Satisfaction with leisure indoors and autonomy regarding using leisure time tended to increase as well. CONCLUSIONS: The tentative conclusion of this small-scale pilot study is that the VIPP programme modestly benefits perceived restrictions in participation, satisfaction with participation and autonomy outdoors of the visually impaired elderly. These preliminary findings warrant further investigation. | Male | Humans | Patient Participation | Questionnaires | OK |
| Marcel A. L. M. | van Assen | Chijs | van Nieuwenhuizen | |  | Psychiatric Services (Washington, D.C.) | 2012-01-00 Jan 2012 | OBJECTIVE: Research on the effectiveness of peer-run services on the recovery of people with major psychiatric problems has been limited and poorly controlled. This study evaluated the effects of a 12-week peer-run course on recovery, "Recovery Is Up to You." METHODS: Recruitment of people with major psychiatric problems took place in the Netherlands between 2006 and 2008, and the effects of the peer-run intervention were evaluated in a randomized controlled trial. A total of 333 people were randomly assigned to the experimental (N=168) or control (N=165) condition. Self-report instruments used were the Herth Hope Index, the Manchester Short Assessment of Quality of Life, the Mental Health Confidence Scale, the Dutch Empowerment Scale, and the Loneliness Scale. Assessments took place at baseline, after three months (at the end of the course), and after six months. Data were analyzed by using linear mixed modeling. RESULTS: The intervention had a significant and positive effect on empowerment, hope, and self-efficacy beliefs but not on quality of life and loneliness. The effects of the intervention persisted three months after participants completed the course. CONCLUSIONS: The results suggest that the peer-run course contributed to improvement in important domains of recovery. Peer-run services, such as "Recovery Is Up to You," add value to recovery-oriented mental health care because they offer participants an opportunity to make an active start on their recovery. | Male | Humans | Patient Participation | Young Adult | OK |
| Paraskevi | Iosifidou | Rosmarie | Mendel | Rudolf | Cohen | The International Journal of Social Psychiatry | 2014-05-00 May 2014 | BACKGROUND: A deeper engagement into medical decision-making is demanded by treatment guidelines for patients with affective disorders. There is to date little evidence on what facilitates active behaviour of patients with depression. In general medicine 'question prompt sheets' (QPSs) have been shown to change patients' behaviour in the consultation and improve treatment satisfaction but there is no evidence for such interventions for mental health settings. AIMS: To study the effects of a QPS on active patient behaviour in the consultation. METHODS: Randomized controlled trial (involving N = 100 outpatients with depression) evaluating the effects of a QPS on patients' behaviour in the consultation. RESULTS: The QPS showed no influence on the number of topics raised by patients (p = .13) nor on the external rater's perception of 'Who made the decisions in today's consultation?' (p = .50). CONCLUSIONS: A QPS did not change depressed patients' behaviour in the consultation. More complex interventions might be needed to change depressed patients' behaviour within an established doctor-patient dyad. Patient seminars addressing behavioural aspects have been shown to be effective in other settings and may also be feasible for outpatients with affective disorders. | Male | Humans | Patient Participation | Patient Satisfaction | OK |
| Ute | Buschmann | Reto | Babst |  |  | Journal of Evaluation in Clinical Practice | 2013-04-00 Apr 2013 | Rationale, aims and objectives  The study aims to investigate the effects of a patient safety advisory on patients' risk perceptions, perceived behavioural control, performance of safety behaviours and experience of adverse incidents. Method  Quasi-experimental intervention study with non-equivalent group comparison was used. Patients admitted to the surgical department of a Swiss large non-university hospital were included. Patients in the intervention group received a safety advisory at their first clinical encounter. Outcomes were assessed using a questionnaire at discharge. Odds ratios for control versus intervention group were calculated. Regression analysis was used to model the effects of the intervention and safety behaviours on the experience of safety incidents. Results  Two hundred eighteen patients in the control and 202 in the intervention group completed the survey (75 and 77% response rates, respectively). Patients in the intervention group were less likely to feel poorly informed about medical errors (OR = 0.55, P = 0.043). There were 73.1% in the intervention and 84.3% in the control group who underestimated the risk for infection (OR = 0.51, CI 0.31-0.84, P = 0.009). Perceived behavioural control was lower in the control group (meanCon  = 3.2, meanInt  = 3.5, P = 0.010). Performance of safety-related behaviours was unaffected by the intervention. Patients in the intervention group were less likely to experience any safety-related incident or unsafe situation (OR for intervention group = 0.57, CI 0.38-0.87, P = 0.009). There were no differences in concerns for errors during hospitalization. There were 96% of patients (intervention) who would recommend other patients to read the advisory. Conclusions  The results suggest that the safety advisory decreases experiences of adverse events and unsafe situations. It renders awareness and perceived behavioural control without increasing concerns for safety and can thus serve as a useful instrument for communication about safety between health care workers and patients. | Male | Humans | Patient Participation | Questionnaires | OK |
| Hilde | Eide | Cornelia M. | Ruland |  |  | Psycho-Oncology | 2013-01-00 Jan 2013 | BACKGROUND: To test the effect of Choice, an interactive tailored patient assessment (ITPA) tool on the number and types of symptoms addressed during consultations with cancer patients, cancer patients' active participation during consultation with clinicians, and clinicians' responses. METHOD: A total of 193 consultations were audio taped and coded with the task-oriented part of Roter Interactive Assessment System. In addition, we recorded the initiator of each coded utterance (clinician or patient) as defined by Verona Coding Definitions of Emotional Sequences and indicated the symptoms addressed in a list of all symptoms within the Choice ITPA. Of the 193 consultations, 99 were standard consultations that served as a control group. In the 94 intervention group consultations, patients utilized the Choice ITPA prior to the consultation, and the assessment summary was available to both patients and clinicians. We compared the two groups in both multilevel and multivariate analyses. RESULTS: We found significantly more symptoms addressed in the intervention group as compared with the control group. We also found that the patients asked more questions in the intervention group, indicating that they were more active participants when utilizing Choice. The clinicians also provided more information in the intervention group. CONCLUSION: The Choice ITPA was successful in making cancer patients more active participants in the consultation with their clinician. | Male | Emotions | Humans | Patient Participation | OK |
| Hilde | Eide | Cornelia M. | Ruland |  |  | Psycho-Oncology | 2013-01-00 janvier 2013 | Background: To test the effect of Choice, an interactive tailored patient assessment (ITPA) tool on the number and types of symptoms addressed during consultations with cancer patients, cancer patients’ active participation during consultation with clinicians, and clinicians’ responses. Method: A total of 193 consultations were audio taped and coded with the task-oriented part of Roter Interactive Assessment System. In addition, we recorded the initiator of each coded utterance (clinician or patient) as defined by Verona Coding Definitions of Emotional Sequences and indicated the symptoms addressed in a list of all symptoms within the Choice ITPA. Of the 193 consultations, 99 were standard consultations that served as a control group. In the 94 intervention group consultations, patients utilized the Choice ITPA prior to the consultation, and the assessment summary was available to both patients and clinicians. We compared the two groups in both multilevel and multivariate analyses. Results: We found significantly more symptoms addressed in the intervention group as compared with the control group. We also found that the patients asked more questions in the intervention group, indicating that they were more active participants when utilizing Choice. The clinicians also provided more information in the intervention group. Conclusion: The Choice ITPA was successful in making cancer patients more active participants in the consultation with their clinician. (PsycINFO Database Record (c) 2013 APA, all rights reserved). (journal abstract) | Professional Consultation | consultation | Neoplasms | cancer care | Doublon |
| Arnstein | Finset |  |  |  |  | Patient Education and Counseling | 2012-02-00 février 2012 | Objective: To test the effect of Choice, an interactive tailored patient assessment tool (ITPA), on cancer patients’ expressed cues and concerns (C&Cs), and clinicians’ responses to these C&Cs. Methods: 97 experimental group consultations, where patients used the Choice ITPA to report their symptoms and problems in preparation to their consultation, were compared to 99 control group consultations. All consultations were audio-taped and coded using the Verona Coding Definitions of Emotional Sequences (VR-CoDES). Results: We identified 473 cues and 109 concerns with a mean number of 3.0 (SD = 3.2). The most frequent utterance was cue B (45.2%), indicating expression of uncertainty or hope. We found more C&Cs in consultations with the Choice ITPA compared to the control group (p &lt; 0.01), and in consultations with nurses compared to physicians (p &lt; 0.001). No differences in clinicians’ response types in the two groups were found. However, significant differences in response type between nurses and physicians were found. Conclusion: The Choice ITPA was an effective tool to disclose cancer patients’ cues and concerns. Practice implications: The Choice ITPA proved to be an effective intervention for cancer patients to express more C&Cs, but should be accompanied with communication skills training to potentially produce more patient-centered responses from the clinicians. (PsycINFO Database Record (c) 2012 APA, all rights reserved). (journal abstract) | Measurement | physicians | Professional Consultation | cancer | Doublon |
| Virginia | Towle | John R. | O'Leary | Lynne | Iannone | Archives of Internal Medicine | 2011-05-23 May 23, 2011 | BACKGROUND: Quality-assurance initiatives encourage adherence to evidenced-based guidelines based on a consideration of treatment benefit. We examined older persons' willingness to take medication for primary cardiovascular disease prevention according to benefits and harms. METHODS: In-person interviews were performed with 356 community-living older persons. Participants were asked about their willingness to take medication for primary prevention of myocardial infarction (MI) with varying benefits in terms of absolute 5-year risk reduction and varying harms in terms of type and severity of adverse effects. RESULTS: Most (88%) would take medication, providing an absolute benefit of 6 fewer persons with MI out of 100, approximating the average risk reduction of currently available medications. Of participants who would not take it, 17% changed their preference if the absolute benefit was increased to 10 fewer persons with MI, and, of participants who would take it, 82% remained willing if the absolute benefit was decreased to 3 fewer persons with MI. In contrast, large proportions (48%-69%) were unwilling or uncertain about taking medication with average benefit causing mild fatigue, nausea, or fuzzy thinking, and only 3% would take medication with adverse effects severe enough to affect functioning. CONCLUSIONS: Older persons' willingness to take medication for primary cardiovascular disease prevention is relatively insensitive to its benefit but highly sensitive to its adverse effects. These results suggest that clinical guidelines and decisions about prescribing these medications to older persons need to place emphasis on both benefits and harms. | Male | Humans | Patient Participation | Questionnaires | OK |
| Heinz-Harald | Abholz | Karl | Wegscheider | Michael | Pentzek | Trials | 2013-00-00 2013 | BACKGROUND: In order to give informed consent for mammography screening, women need to be told the relevant facts; however, screening information often remains vague because of the worry that detailed information might deter women from participating in recommended screening programs. Since September 2010, German women aged 50 to 69 invited for mammography screening have received a new, comprehensive information brochure that frankly discusses the potential benefit and harm of mammography screening. In contrast, the brochure that was in use before September 2010 contained little relevant information.The aim of this study is to compare the impact of the two different brochures on the intention of women to undergo mammography screening, and to broaden our understanding of the effect that factual information has on the women's decision-making. METHODS: This is a controlled questionnaire study comparing knowledge, views and hypothetical preferences of women aged 48-49 years after receiving the old versus the new information brochure. German GP's in the region of North Rhine-Westfalia will be asked by mail and telephone to participate in the study. Eligible women will be recruited via their general practitioners (GPs) and randomized to groups A ('new brochure') and B ('old brochure'), with an intended recruitment of 173 participants per group. The study is powered to detect a 15% higher or lower intention to undergo mammography screening in women informed by the new brochure. DISCUSSION: This study will contribute to our understanding of the decision-making of women invited to mammography screening. From both ethical and public health perspectives, it is important to know whether frank, factual information leads to a change in the intention of women to participate in a recommended breast cancer screening program. TRIAL REGISTRATION: DRKS00004271. | Humans | Patient Participation | Questionnaires | Female | OK |
| Karen H. | Costenbader | Christina | Chambers | Kaleb | Michaud | Arthritis Care & Research | 2012-05-00 May 2012 | OBJECTIVE: Women with rheumatoid arthritis (RA) and systemic lupus erythematosus (SLE) have fewer children on average than other women. We sought to determine the roles of infertility, pregnancy loss, and personal choice on family size in women with these diseases. METHODS: A reproductive history questionnaire was completed by women with RA and SLE participating in a longitudinal observational study. Within each disease cohort, participants were divided into 3 groups: those interested in having children at symptom onset who had either fewer children than planned (group A) or the same number as planned (group B), and those no longer interested in having children at diagnosis (group C). RESULTS: Of the 578 RA and 114 SLE women surveyed, >60% were in group C. Of those interested in having children, 55% with RA and 64% with SLE had fewer children than originally planned. Among women with RA, group A had 1 less pregnancy, 1 less live birth, and an infertility rate 1.5 times higher than group B; the miscarriage rate was similar in both groups. Compared to SLE group B, SLE group A had a similar number of pregnancies, but a 3-fold higher rate of miscarriage and 1 less live birth. Concerns about child health and personal welfare were associated with a lower pregnancy rate. CONCLUSION: In this population, more than one-half of young women with RA or SLE had fewer biologic children than desired. While patient choice plays a role, infertility in RA patients and miscarriage in SLE patients are also important. | Humans | Patient Participation | Questionnaires | Young Adult | OK |
| Naoya | Sugiyama | Hana | Hasegawa | Toshie | Noda | BMC psychiatry | 2014-00-00 2014 | BACKGROUND: Shared decision making is a promising model for patient-centred medicine, resulting in better clinical outcomes overall. In the mental health field, interventions that consider the patient-centred perspective--such as patient quality of life, involvement in the treatment, treatment satisfaction, and working alliance--have increased and better clinical outcomes discovered for patients with schizophrenia. However, few studies have examined the efficacy of shared decision making for schizophrenia treatment. The objective of this study is to evaluate the effect of a shared decision making intervention compared to treatment as usual on patient satisfaction at discharge for first-admission patients with schizophrenia. METHODS/DESIGN: This is a randomised, parallel-group, two-arm, open-label, single-centre study currently being conducted in an acute psychiatric ward of Numazu Chuo Hospital, Japan. We are recruiting patients between 16 and 65 years old who are admitted to the ward with a diagnosis of schizophrenia without prior experience of psychiatric admission. Fifty-eight participants are being randomised into a shared decision making intervention group or a treatment as usual control group in a 1:1 ratio. The intervention program was developed based on a shared decision making model and is presented as a weekly course lasting the duration of the patients' acute psychiatric ward stay. The primary outcome measure is patient satisfaction at discharge as assessed by the Client Satisfaction Questionnaire. Due to the study's nature, neither the patient nor staff can be blinded. DISCUSSION: This is the first randomised controlled trial to evaluate the efficacy of shared decision making for patients with early-treatment-stage schizophrenia. The intervention program in this study is innovative in that it includes both of the patient and staff who are involved in the treatment. TRIAL REGISTRATION: The study has been registered with ClinicalTrials.gov as NCT01869660. | Male | Decision Making | Humans | Patient Satisfaction | OK |
| Simon | Paternotte | Catherine | Beauvais | Philippe | Gaudin | Clinical and Experimental Rheumatology | 2012-08-00 2012 Jul-Aug | OBJECTIVES: Rheumatoid arthritis (RA) may have consequences on sexual life. The objective was to develop and validate a questionnaire assessing the impact of RA on sexuality. METHODS: First, 6 patients (5 women, 1 man) with RA, 2 rheumatologists and 1 sexologist elaborated during a one-day focus-group type meeting an exhaustive list of issues relating to impact of RA on sexuality. The list was reduced by merging similar issues, then according to the relative importance for patients of each issue. A questionnaire was developed with input from these patients, with particular attention on phrasing. Psychometric properties (missing data, correlations with other disease aspects, reliability) were assessed in a multi-centre study. RESULTS: The list of 33 issues related to impact of RA on sexuality included psychological issues (9), couple/relationship issues (9), physical issues (7), and general aspects (5). A 10-question numeric rating scale questionnaire was constructed. Preliminary validation was obtained on 53 patients (44 women, mean age 50.7 years; mean disease duration 14.4 years). The mean score was 3.3±2.5, missing data were acceptable (13%). Qualisex results were correlated with disease activity and symptoms (r=0.50-0.65, p< 0.001); but not with demographics, depression or coping. Qualisex was reliable in 40 patients: the intra-class correlation coefficient was 0.83 (95% CI: 0.70-0.91). CONCLUSIONS: A simple (10 questions) and valid tool investigating impact of RA on sexuality has been developed with the involvement of patients. This tool can be useful to assess this important aspect of quality of life. | Male | Humans | Questionnaires | Female | OK |
| Fredrik | Sjöstrand | Birgitta Wireklint | Sundström |  |  | International Journal of Qualitative Studies on Health and Well-Being | 2013-00-00 2013 | As organizational changes in the healthcare system are in progress, to enhance care quality and reduce costs, it is important to investigate how these changes affect elderly patients' experiences and their rights to participate in the choice of healthcare. The aim of this study is to describe elderly patients' lived experience of participating in the choice of healthcare when being offered an alternative care pathway by the emergency medical services, when the individual patient's medical needs made this choice possible. This study was carried out from the perspective of caring science, and a phenomenological approach was applied, where data were analysed for meaning. Data consist of 11 semi-structured interviews with elderly patients who chose a healthcare pathway to a community-based hospital when they were offered an alternative level of healthcare. The findings show that the essence of the phenomenon is described as "There was a ray of hope about a caring encounter and about being treated like a unique human being". Five meaningful constituents emerged in the descriptions: endurable waiting, speedy transference, a concerned encounter, trust in competence, and a choice based on memories of suffering from care. The conclusion is that patient participation in the choice of a healthcare alternative instead of the emergency department is an opportunity of avoiding suffering from care and being objectified. | Male | Humans | Patient Participation | Patient Satisfaction | OK |
|  |  |  |  |  |  | The American Journal of Geriatric Pharmacotherapy | 2011-02-00 Feb 2011 | BACKGROUND: It is generally believed that elderly persons know little about the purpose of their medications. However, researchers have reported contradictory results. Moreover, little is known about which patient characteristics are associated with knowledge of a drug's purpose. OBJECTIVES: To assess whether community-dwelling elderly persons could correctly identify the general purpose of their prescription and nonprescription drugs and to examine the link between participant and drug characteristics and this knowledge. METHODS: During an in-home interview, a random sample of pharmacy clients aged ≥65 years were asked to report all the drugs they had taken over the previous 30 days and to state the reasons for their use. Drugs were classified according to the anatomical therapeutic chemical (ATC) classification system. We looked for any information in the stated reasons that concerned the anatomical system, organs and diseases. We assumed that the person knew the general purpose of the drug if the reason stated referred to the anatomical system targeted by the drug. A multivariate logistic regression model was used to identify participant characteristics that were associated with knowledge. RESULTS: We interviewed 193 seniors who reported using 1645 drugs that could be classified according to the ATC classification system. Of those drugs, 1364 (82.9%) were self reported as physician prescribed. Among participants, 134 (69.4%) knew the general purpose of all drugs they reported. Characteristics associated with knowledge of the purpose were female gender (adjusted odds ratios [ORs], 3.3, 95% confidence intervals [CI] 1.5-7.1), normal cognition (adjusted OR, 5.0; 95% CI, 2.0-12.8), and average to superior socioeconomic status (adjusted OR, 2.7, 95% CI, 1.2-6.3). Knowledge was slightly better for nonprescribed drugs than for prescribed drugs (overall proportion of appropriate reasons: 96.1% vs 91.1%; P < 0.01). CONCLUSIONS: Community-dwelling elderly persons have a good knowledge of the general purpose of their prescription and nonprescription drugs. | Humans | Patient Participation | Questionnaires | Female | OK |
| David | Spitzer | Steven | Martin | Ian | Forde | BMC family practice | 2011-00-00 2011 | BACKGROUND: To investigate general practitioners' (GPs') attitudes to guidelines for elective surgical referral in England. To understand their use of guidelines, and attitudes to shared decision making in the referral decision. METHODS: A questionnaire was developed which investigated attitudes to and use of guidelines. It was given to a stratified random sample 30% (n = 310) drawn from GP lists of 10 English health districts (primary care trusts (PCTs)). GPs were invited to respond online, by telephone, fax or post. Data were analysed using descriptive statistics and backwards stepwise logistic regression. RESULTS: Responses were representative of GPs in England, but (despite up to 6 contacts per non-responder) the overall response rate was 41.6% (n = 129; with the range across PCTs of 25-61%). Most responding GPs indicated support for referral guidelines but 18% reported that they had never used them. Less than three per cent reported use for most or all referral decisions. The odds of using guidelines decreased with increasing age, with a ten year increase in age associated with halving odds of use (OR = 0.53, 95%CI = 0.29-0.90). Over 50% of GPs wanted good access to electronic guidelines with expert information and advice on guideline availability. Almost all (>89%) GPs agreed with sharing referral decisions with patients. Female doctors (OR = 5.2, 95%CI: 1.02-26.3) were more likely to agree with this than male GPs as were those working in larger compared to small or single handed practices (OR = 5.3, 95%CI: 1.4-19.9). CONCLUSIONS: This group of responding GPs was supportive of guidelines but used them in different ways. Referral guidelines should have an educational component for background reading; include key messages for internalisation and application; and incorporate mechanisms to facilitate accessibility and appropriate shared decision making with patients. | Male | Decision Making | Humans | Questionnaires | OK |
| Decha | Pinkaew | John R. | Petersen | Amornrat | Phongdara | BBA clinical | 2014-12-01 Dec 1, 2014 | BACKGROUND: Billions of cells undergo apoptosis each day in the average normal adult. The ability to readily assess the degree of apoptosis in human diseases is hampered by the lack of sensitive and specific serum biomarkers of apoptosis. Fortilin is a novel prosurvival molecule that protects cells against various noxious stimuli. While fortilin is secreted into the extracellular space under certain conditions, the relationship between the serum concentration of fortilin and the presence and extent of apoptosis in vivo remains unknown. METHODS & RESULTS: Using a newly developed fortilin ELISA system, we show here that fortilin exists in the normal human and mouse circulation. We further demonstrate that fortilin serum levels are significantly elevated in patients with solid cancer, in response to anti-cancer chemo- or radiation therapy. The elevation of fortilin serum levels is more robust and sensitive than that of such previously-reported serum biomarkers of apoptosis as fragmented cytokeratin-18, cytochrome c, and nucleosomal DNA. In addition, targeted apoptotic liver damage induced by Jo2 anti-Fas (CD95) antibody consistently and significantly increased serum fortilin levels in C57BL/6J mice. Finally, when challenged by anti-human-Fas IgM antibody, Jurkat leukemic T cells apoptosed and released fortilin into the medium before plasma membrane integrity was compromised. CONCLUSIONS: Taken together, these data suggest that serum fortilin levels reflect the degree and extent of apoptosis occurring in vivo. GENERAL SIGNIFICANCE: Fortilin is a viable serum biomarker of in vivo apoptosis and can be utilized to noninvasively assess the status of in vivo apoptosis in humans. | | | | | OK |
|  |  |  |  |  |  | Journal of Palliative Medicine | 2010-03-00 Mar 2010 | This case report reviews some of the clinical aspects of epidermal growth factor receptor (EGFR) inhibitor-induced rash, and, more importantly, it describes a specific case in which the diagnosis of such a rash occurred by means of a patient-initiated photographic electronic communication. The fact that this electronic communication was patient-initiated suggests that health care providers will likely be asked to make clinical decisions based on this type of communication soon and that this type of seemingly low-cost approach will likely become an important aspect of cancer symptom control and palliative care in the future. | Male | Humans | Patient Participation | Middle Aged | OK |
|  |  |  |  |  |  | Journal of the American Academy of Nurse Practitioners | 2010-11-00 Nov 2010 | PURPOSE: Despite the recognized importance of patient involvement in primary care interactions, little information describing women's needs and expectations for these interactions is available. This participatory action study was based in Critical Action Theory and designed to describe any emancipatory interests that surfaced when eight ethnically diverse women examined their interactions with primary care nurse practitioners (PCNPs) over the course of five successive focus group meetings. DATA SOURCES: Focus group meeting transcripts, field notes, interaction notations, seating maps, and first impression summaries. CONCLUSIONS: Participants wanted to learn how to "stand up" for themselves in primary care interactions. They believed this could be accomplished by developing a positive sense of self-esteem. Ultimately, they identified the right way to "talk back" to clinicians and created a method for regaining control of their own health care and maintaining equality in interactions with primary care clinicians. IMPLICATIONS FOR PRACTICE: Nurse practitioners working in the primary setting are especially well situated to support self-management and foster patient participation by women as they live with chronic disease, engage in health promotion activities, and deal with common symptomatic problems for themselves and their families. | Humans | Patient Participation | Questionnaires | Female | OK |
| H. R. | Pasman | Lieve | Van den Block | Gé A. | Donker | Journal of Pain and Symptom Management | 2014-03-00 Mar 2014 | CONTEXT: Effective communication is central to high-quality end-of-life care. OBJECTIVES: This study examined the prevalence of general practitioner (GP)-patient discussion of end-of-life topics (according to the GP) in Italy, Spain, Belgium, and The Netherlands and associated patient and care characteristics. METHODS: This cross-sectional, retrospective survey was conducted with representative GP networks. Using a standardized form, GPs recorded the health and care characteristics in the last three months of life, and the discussion of 10 end-of-life topics, of all patients who died under their care. The mean number of topics discussed, the prevalence of discussion of each topic, and patient and care characteristics associated with discussions were estimated per country. RESULTS: In total, 4396 nonsudden deaths were included. On average, more topics were discussed in The Netherlands (mean=6.37), followed by Belgium (4.45), Spain (3.32), and Italy (3.19). The topics most frequently discussed in all countries were "physical complaints" and the "primary diagnosis," whereas "spiritual and existential issues" were the least frequently discussed. Discussions were most prevalent in The Netherlands, followed by Belgium. The GPs from all countries tended to discuss fewer topics with older patients, noncancer patients, patients with dementia, patients for whom palliative care was not an important treatment aim, and patients for whom their GP had not provided palliative care. CONCLUSION: The prevalence of end-of-life discussions varied across the four countries. In all countries, training priorities should include the identification and discussion of spiritual and social problems and early end-of-life discussions with older patients, those with cognitive decline if possible, and those with non-malignant diseases. | | | | | OK |
| Siebe | Swart | Carin | van der Rijt | Paul J. | van der Maas | Journal of Pain and Symptom Management | 2010-01-00 Jan 2010 | Differences in the general focus of care among hospitals, nursing homes, and homes may affect the adequacy of end-of-life decision making for the dying. We studied end-of-life decision-making practices for cancer patients who died in each of these settings and assessed the impact of the Liverpool Care Pathway for the Dying Patient (LCP), a template for care in the dying phase. Physicians and relatives of 311 deceased cancer patients completed questionnaires. The LCP was introduced halfway through the study period. During the last three months of life, patients who died in hospital received anticancer therapy and medication to relieve symptoms more often than those in both other settings. During the last three days of life, patients who died in the hospital or nursing home received more medication than those who died at home. The LCP reduced the extent to which physicians used medication that might have hastened death. Relatives of patients who died in the hospital tended to be least positive about the patient's and their own participation in the decision making. We conclude that cancer patients who die in the hospital are more intensively treated during the last phase of life than those who die elsewhere. The LCP has an impact on the use of potentially life-shortening medication during the dying phase. Communication about medical decision making tends to be better in the nursing home and at home. | Decision Making | Humans | Patient Participation | Physician-Patient Relations | OK |
| Tomás | Vega Alonso | Lieve | Van den Block | Guido | Miccinesi | PloS One | 2013-00-00 2013 | BACKGROUND: Making treatment decisions in anticipation of possible future incapacity is an important part of patient participation in end-of-life decision-making. This study estimates and compares the prevalence of GP-patient end-of-life treatment discussions and patients' appointment of surrogate decision-makers in Italy, Spain, Belgium and the Netherlands and examines associated factors. METHODS: A cross-sectional, retrospective survey was conducted with representative GP networks in four countries. GPs recorded the health and care characteristics in the last three months of life of 4,396 patients who died non-suddenly. Prevalences were estimated and logistic regressions were used to examine between country differences and country-specific associated patient and care factors. RESULTS: GP-patient discussion of treatment preferences occurred for 10%, 7%, 25% and 47% of Italian, Spanish, Belgian and of Dutch patients respectively. Furthermore, 6%, 5%, 16% and 29% of Italian, Spanish, Belgian and Dutch patients had a surrogate decision-maker. Despite some country-specific differences, previous GP-patient discussion of primary diagnosis, more frequent GP contact, GP provision of palliative care, the importance of palliative care as a treatment aim and place of death were positively associated with preference discussions or surrogate appointments. A diagnosis of dementia was negatively associated with preference discussions and surrogate appointments. CONCLUSIONS: The study revealed a higher prevalence of treatment preference discussions and surrogate appointments in the two northern compared to the two southern European countries. Factors associated with preference discussions and surrogate appointments suggest that delaying diagnosis discussions impedes anticipatory planning, whereas early preference discussions, particularly for dementia patients, and the provision of palliative care encourage participation. | Male | Decision Making | Humans | Patient Participation | OK |
| C. | Brunelli | C. | Borreani |  |  | European Journal of Cancer Care | 2012-09-00 Sep 2012 | The aim of the present study is to describe end-of-life preferences of advanced cancer patients willing to talk about death issues. Eighty-eight advanced cancer patients were interviewed through End of Life Preferences Interview (ELPI), a 23-item interview covering a wide range of end-of-life care issues. Most interviewed subjects were home care patients and their median survival after ELPI administration was 69 days. In total, 100% of responders expressed the will to receive some kind of information on the disease process and/or the treatments proposed. Approximately 77% declared to be willing to talk about what it is important at the end of life in case of worsening of their conditions and 31% prefer to be left alone in difficult moments. Approximately 67% choose home as the preferred place of death and 63% think it is preferable to die in a state of unconsciousness induced by drugs. About half of responders declare to believe in any kind of life after death and 40% consider very important to find any meaning at the end of life. ELPI can be a useful instrument to adapt the model of care to the specific needs and values of each patient. | Male | Decision Making | Humans | Patient Participation | OK |
| Marit | Jordhøy |  |  |  |  | Scandinavian Journal of Urology and Nephrology | 2011-09-00 Sep 2011 | OBJECTIVE: Recruitment is one of the most serious challenges in randomized clinical trials (RCTs), especially in the old and frail population. A Norwegian multicentre RCT targeting end-stage renal disease (ESRD) patients older than 70 years was initiated to compare the impact on quality of life of early or late start of dialysis. Owing to poor inclusion the RCT was closed. The aim of the present study was to explore possible reasons for the recruitment failure. MATERIAL AND METHODS: A questionnaire was distributed to all Norwegian nephrologists. The questionnaire presented 11 statements which cited possible reasons for not including elderly ESRD patients in the RCT in question. RESULTS: The highest rated reasons for non-inclusion were the physician's wish to decide the timing of dialysis individually and the patient's wish to postpone the start of treatment. High mean scores were also found for reasons related to workload and capacity at the dialysis unit, whereas the influence of the doctor-patient relationship and competing studies were judged not to be important. CONCLUSIONS: The results indicate that confidence in individually decided treatment and fear of losing professional autonomy make Norwegian nephrologists reluctant to include patients in RCTs. To succeed in recruitment, there seems to be a need for cultural changes as well as increased resources to meet practical challenges. | Humans | Patient Participation | Questionnaires | Aged | OK |
| Dirk | vom Lehn | William | Gibson |  |  | Symbolic Interaction | 2013-05-00 mai 2013 | Many of the procedures undertaken within healthcare require specialized forms of participation that may be unfamiliar, even disturbing, to patients or clients. The practitioner has to encourage and enable participation in the investigation in an appropriate fashion while preserving the structure and integrity of the procedure. In this article, we consider optometry and the deployment of a vision test, known as subject refraction, that provides data to help determine the characteristics of any corrective lens that may be required by clients. The procedure’s ability to establish robust and reliable data relies upon the optometrist’s ability to encourage the client to respond to a series of stimuli without consideration or reflection. That is, the client is required to produce an unwitting response—conduct that might be considered nonsymbolic rather than symbolic. In this article, we also consider the optometrist’s talk and bodily comportment during subjective refraction and how it serves to shape and determine the quality of the client’s response and participation, and in turn to produce reliable test results. (PsycINFO Database Record (c) 2013 APA, all rights reserved). (journal abstract) | Client Participation | Professional Consultation | professional gesture | optometry consultations | OK |
|  |  |  |  |  |  | Psychiatry Research | 2010-11-30 Nov 30, 2010 | Interventions aimed at enhancing psychiatric patients' outpatient treatment adherence frequently include informal coercion such as inducements, and threats of negative sanctions. Little is known about patients' subjective perspective concerning these practices. This study examined the perceived coercion, and also the appraisal of the fairness and effectiveness of the treatment. A total of 187 psychiatric patients with different diagnoses were interviewed using a structured questionnaire that included socio-demographic and clinical data, insight into illness (ITAQ), psychopathology (BPRS), functioning (GAF) and a modified version of the MacArthur Admission Experience Survey (AES) as a measure of perceived coercion, fairness and effectiveness. Bivariate correlations and logistic regression analyses were used to identify the influencing factors on the outcome variables. Perceived coercion was associated with experience with informal coercive treatment pressures (OR 2.5-2.9; P<0.05), and a main diagnosis of a schizophrenic disorder (OR 3.8; P<0.001). Experience with informal coercion was inversely associated with fairness (OR 0.3-0.4; P<0.05), but not with effectiveness. Patients with more insight into their illness indicated higher fairness and effectiveness concerning the procedures for enhancing their treatment adherence (OR 3.1; P<0.01). Insight into illness was not associated with perceived coercion. This might indicate that enhancing insight using psycho-educational approaches and high transparency when applying informal coercive practices could improve patients' appreciation for these procedures. They could probably cope better with perceived coercion when understanding its purpose, and when they perceive they are being treated fairly. | Male | Humans | Patient Participation | Questionnaires | OK |
| Mark | Ashworth |  |  |  |  | BMC Psychiatry | 2013-12-16 décembre 16, 2013 | Background: Involving patients in treatment is becoming increasingly popular in mental health [Sales & Alves: Personalized evaluation of psychological treatments: A review of tools and research designs, submitted]. However, in substance misuse treatment settings, the patient perspective about treatment tends to be overlooked. This has been cited as a key priority by Orford et al. [Addiction, 103: 875-885, 2008] who included patient feedback about treatment as one of ten areas requiring an urgent paradigm shift in addiction research and practice. This project will apply an innovative method to involve substance misuse patients in psychological therapies, by asking them to suggest topics to evaluate their treatment. These topics suggested by patients can be written as a list of personalised items, so-called as patient-generated outcome measures (PGOM). Despite its patient-friendly features, PGOM’s have never been used in this population, which is what this project aims to overcome. Methods/design: This project is part of an International Exchange Platform on Personalising Addiction Treatment. Data will be collected in two phases (pre-post study and focus groups with patients) to explore the following: 1). How reliable and sensitive to change are PGOM’s and standardised measures in substance misuse treatment? 2). Do PGOM’s add relevant information to standardised measures? 3). What are the views of substance misuse patients about personalised outcome assessment? 4). Development of guidelines on using PGOM’s in this population. Discussion: This research will potentially demonstrate the diversity of personal problems among patients seeking substance misuse treatment, suggesting the relevance of PGOM as a method to personalise outcome measurement and, ultimately, guiding treatment provision. It is expected that, as in previous studies, PGOM’s will be perceived as helpful and patient-friendly tools, where patients may express their own concerns in a semi-structured setting. Similarly to other populations, we also expect PGOM’s to be reliable, valid and sensitive to clinical changes in substance misuse treatment, as well as more content informative than their standardised counterparts. If these results are achieved, we might hypothesize that PGOM’s are a potentially valid supplement to traditional standardised scales, by providing a closer insight to what motivates patients to participate in substance misuse treatment programmes. (PsycINFO Database Record (c) 2014 APA, all rights reserved). (journal abstract) | treatment outcomes | substance misuse | patient generated outcome measures | addiction treatment | Doublon |
| Mark | Ashworth |  |  |  |  | BMC psychiatry | 2013-00-00 2013 | BACKGROUND: Involving patients in treatment is becoming increasingly popular in mental health [Sales & Alves: Personalized evaluation of psychological treatments: A review of tools and research designs, submitted]. However, in substance misuse treatment settings, the patient perspective about treatment tends to be overlooked. This has been cited as a key priority by Orford et al. [Addiction, 103: 875-885, 2008] who included patient feedback about treatment as one of ten areas requiring an urgent paradigm shift in addiction research and practice.This project will apply an innovative method to involve substance misuse patients in psychological therapies, by asking them to suggest topics to evaluate their treatment. These topics suggested by patients can be written as a list of personalised items, so-called as patient-generated outcome measures (PGOM). Despite its patient-friendly features, PGOM's have never been used in this population, which is what this project aims to overcome. METHODS/DESIGN: This project is part of an International Exchange Platform on Personalising Addiction Treatment. Data will be collected in two phases (pre-post study and focus groups with patients) to explore the following: (1). How reliable and sensitive to change are PGOM's and standardised measures in substance misuse treatment? (2). Do PGOM's add relevant information to standardised measures? (3). What are the views of substance misuse patients about personalised outcome assessment? (4). Development of guidelines on using PGOM's in this population DISCUSSION: This research will potentially demonstrate the diversity of personal problems among patients seeking substance misuse treatment, suggesting the relevance of PGOM as a method to personalise outcome measurement and, ultimately, guiding treatment provision. It is expected that, as in previous studies, PGOM's will be perceived as helpful and patient-friendly tools, where patients may express their own concerns in a semi-structured setting. Similarly to other populations, we also expect PGOM's to be reliable, valid and sensitive to clinical changes in substance misuse treatment, as well as more content informative than their standardised counterparts. If these results are achieved, we might hypothesize that PGOM's are a potentially valid supplement to traditional standardised scales, by providing a closer insight to what motivates patients to participate in substance misuse treatment programmes. | Male | Humans | Patient Participation | Female | OK |
| Joanne | Lally | Carrie A. | Levin | Daniel D. | Matlock | BMC medical informatics and decision making | 2013-00-00 2013 | BACKGROUND: Establishing the effectiveness of patient decision aids (PtDA) requires evidence that PtDAs improve the quality of the decision-making process and the quality of the choice made, or decision quality. The aim of this paper is to review the theoretical and empirical evidence for PtDA effectiveness and discuss emerging practical and research issues in the measurement of effectiveness. METHODS: This updated overview incorporates: a) an examination of the instruments used to measure five key decision-making process constructs (i.e., recognize decision, feel informed about options and outcomes, feel clear about goals and preferences, discuss goals and preferences with health care provider, and be involved in decisions) and decision quality constructs (i.e., knowledge, realistic expectations, values-choice agreement) within the 86 trials in the Cochrane review; and b) a summary of the 2011 Cochrane Collaboration's review of PtDAs for these key constructs. Data on the constructs and instruments used were extracted independently by two authors from the 86 trials and any disagreements were resolved by discussion, with adjudication by a third party where required. RESULTS: The 86 studies provide considerable evidence that PtDAs improve the decision-making process and decision quality. A majority of the studies (76/86; 88%) measured at least one of the key decision-making process or decision quality constructs. Seventeen different measurement instruments were used to measure decision-making process constructs, but no single instrument covered all five constructs. The Decisional Conflict Scale was most commonly used (n = 47), followed by the Control Preference Scale (n = 9). Many studies reported one or more constructs of decision quality, including knowledge (n = 59), realistic expectation of risks and benefits (n = 21), and values-choice agreement (n = 13). There was considerable variability in how values-choice agreement was defined and determined. No study reported on all key decision-making process and decision quality constructs. CONCLUSIONS: Evidence of PtDA effectiveness in improving the quality of the decision-making process and decision quality is strong and growing. There is not, however, consensus or standardization of measurement for either the decision-making process or decision quality. Additional work is needed to develop and evaluate measurement instruments and further explore theoretical issues to advance future research on PtDA effectiveness. | Decision Making | Decision Support Techniques | Humans | Patient Participation | OK |
|  |  |  |  |  |  | International psychogeriatrics / IPA | 2012-03-00 Mar 2012 | BACKGROUND: Problems with out-of-home mobility are among the more common behavioral disturbances in dementia. Today people with dementia can be aided by easily accessible assistive technologies, such as tracking devices using Global Positioning Systems (GPS). Attitudes toward these technologies are still inconclusive and their use with people with dementia raises ethical concerns. The lack of ethical consensus on the use of GPS for people with dementia underlines the need for clearer policies and practical guidelines. METHODS: Here we summarize qualitative and quantitative findings from a larger research project on the ethical aspects of using GPS for tracking people with dementia. RESULTS: The findings are formulated in a list of recommendations for policy-makers as well as for professional and family caregivers. Among other points, the recommendations indicate that the preferences and best interests of the people with dementia should be central to the difficult decisions required in dementia care. Further, no-one should be coerced into using tracking technology and, where possible, people with dementia must be involved in the decision-making and their consent sought. CONCLUSIONS: The decision whether, when and how to use GPS for tracking people with dementia should be made at the time of diagnosis jointly by the person with dementia, his/her family and professional caregivers. This decision should be made in formal structured meetings facilitated by a professional team. | Male | Humans | Patient Participation | Questionnaires | OK |
|  |  |  |  |  |  | Current Opinion in Obstetrics & Gynecology | 2010-04-00 Apr 2010 | PURPOSE OF REVIEW: First-trimester risk assessment has now become sophisticated and of increasing relevance and applicability to decision-making by pregnant woman about invasive diagnosis. Ethics is an essential dimension of understanding this relevance and applicability. This paper addresses the ethical dimensions of first-trimester risk assessment for trisomy 21. RECENT FINDINGS: It is now well established in the ethics and law of the informed consent process that physicians are obligated to offer to patients all medically reasonable alternatives for managing the patient's condition. This disclosure should be guided by the reasonable person standard: the physician should provide clinically important information about the patient's condition or diagnosis, the medically reasonable alternatives for managing it, and the clinical benefits and risks of each such alternative. SUMMARY: On the basis of the ethics of informed consent, we argue that routinely offering first-trimester risk assessment in centers qualified to provide it is ethically obligatory. We describe how pregnant women can be expected to respond to this offer. We then argue that routinely withholding the results of first-trimester risk assessment is ethically unjustified. The ethics of informed consent is an essential dimension of first-trimester risk assessment for trisomy 21. | Humans | Patient Participation | Physician-Patient Relations | Female | OK |
|  |  |  |  |  |  | The Journal of Clinical Psychiatry | 2013-07-00 Jul 2013 | Even with many treatment options available for major depressive disorder, many patients fail to achieve remission and return to their presymptomatic levels of functioning at work, in leisure activities, and in relationships. Throughout treatment, clinicians should implement measurement-based care by systematically monitoring patients' response using self-rated scales, such as the PHQ-9, QIDS-SR, or BDI. By tracking depressive symptoms, as well as suicidality, treatment adherence, and side effects, clinicians can adjust treatment to help patients achieve the best outcomes. Measurement-based care enables clinicians to make informed decisions at critical points throughout the treatment process and to involve patients in making those decisions. | Humans | Patient Participation | medication adherence | depression | OK |
| Michael H. | Kanter | Mildred | Hugh | Rachel R. | Oglesby | Clinical Therapeutics | 2014-05-00 May 2014 | OBJECTIVE: The goal of this study was to evaluate the reliability and validity of the Consumer Assessment of Healthcare Providers and Systems (CAHPS) Patient-Centered Medical Home (PCMH) survey. METHODS: We conducted a field test of the CAHPS PCMH survey with 2740 adults. We collected information by mail (n = 1746), telephone (n = 672), and from the Web (n = 322) from 6 sites of care affiliated with a West Coast staff model health maintenance organization. RESULTS: An overall response rate of 37% was obtained. Internal consistency reliability estimates for 7 multi-item scales were as follows: access to care, 5 items, α = 0.79; communication with providers, 6 items, α = 0.93; office staff courtesy and respect, 2 items, α = 0.80; shared decision making about medicines, 3 items, α = 0.67; self-management support, 2 items, α = 0.61; attention to mental health issues, 3 items, α = 0.80; and care coordination, 4 items, α = 0.58. The number of responses needed to get reliable information at the site of care level for the composites was generally acceptable (<300 for 0.70 reliability-level) except for self-management support and shared decision making about medicines. Item-scale correlations provided support for distinct composites except for access to care and shared decision making about medicines, which overlapped with the communication with providers scale. Shared decision making and self-management support were significantly, uniquely associated with the global rating of the provider (dependent variable), along with access and communication in a multiple regression model. CONCLUSIONS: This study provides further support for the reliability and validity of the CAHPS PCMH survey, but refinement of the self-management support and shared decision-making scales is needed. The survey can be used to provide information about the performance of different health plans on multiple domains of health care, but future efforts to improve some of the survey items is needed. | | | | | OK |
|  |  |  |  |  |  | Journal of Wound Care | 2014-01-00 Jan 2014 | Although healing rates have improved with the use of compression therapy, venous leg ulceration recurrence rates remain very high. There is evidence that certain self-care activities may reduce the risk of recurrence, however, many patients do not adhere to performing these self-care activities. The reasons for this may be multifactorial. Furthermore, the literature indicates that some patients appear to have a poor understanding of their condition despite having received information from health professionals. Since a concordant relationship is dependent on shared decision-making, this apparent knowledge deficit may have major implications in changing health behaviours and achieving adherence with self-care. This article is in two parts - part one will briefly discuss health behaviour change models underpinned by social cognitive theory. It introduces the VeLUSET, a newly developed and validated tool which provides an objective measure of nursing interventions designed to increase self-efficacy. | Humans | Questionnaires | self efficacy | Social Support | OK |
| Mei Sheng | Duh | Howard G. | Birnbaum | Alice Kate | Cummings | The Patient | 2013-00-00 2013 | BACKGROUND: While there are validated patient-reported outcomes (PRO) instruments for use in specific cancer populations, no validated general instruments exist for use in conditions common to multiple cancers, such as muscle wasting and consequent physical disability. The Medicare Current Beneficiary Survey (MCBS), a survey in a nationally representative sample of Medicare beneficiaries, includes items from three well known scales with general applicability to cancer patients: Katz activities of daily living (ADL), Rosow-Breslau instrumental ADL (IADL), and a subset of physical performance items from the Nagi scale. OBJECTIVE: This study evaluated properties of the Katz ADL, Rosow-Breslau IADL, and a subset of the Nagi scale in patients with pancreatic cancer, lung cancer, and myeloproliferative neoplasms (MPN) using data from MCBS linked with Medicare claims in order to understand the potential utility of the three scales in these populations; understanding patient-perceived significance was not in scope. METHODS: The study cohorts included Medicare beneficiaries aged ≥65 years as of 1 January of the year of their first cancer diagnosis with one or more health assessments in a community setting in the MCBS Access to Care data from 1991 to 2009. Beneficiaries had at least two diagnoses in de-identified Medicare claims data linked to the MCBS for one of the following cancers: pancreatic, lung, or MPN. The Katz ADL, Rosow-Breslau IADL, and Nagi scales were calculated to assess physical functioning over time from cancer diagnosis. Psychometric properties for each scale in each cohort were evaluated by testing for internal consistency, test-retest reliability, and responsiveness by comparing differences in mean scale scores over time as cancer progresses, and differences in mean scale scores before and after hospitalization (for lung cancer cohort). RESULTS: The study cohorts included 90 patients with pancreatic cancer, 863 with lung cancer, and 135 with MPN. Among each cancer cohort, the Katz ADL, Rosow-Breslau IADL, and Nagi scales had acceptable internal consistency (Cronbach's alpha generally between 0.70 and 0.90) and test-retest reliability for consecutive surveys before diagnosis and consecutive surveys after diagnosis (when patients' functioning was more stable). Compared with mean scale scores at the survey 1-2 years before cancer diagnosis (baseline), mean scale scores at the first survey after cancer diagnosis were significantly higher (P < 0.05), indicating worsening, for Katz ADL, Rosow-Breslau IADL, and Nagi scales (items scored 0-1) (0.54 vs. 1.45, 1.15 vs. 2.20, and 2.29 vs. 3.08, respectively, for pancreatic cancer; 0.73 vs. 1.24, 1.29 vs. 2.01, and 2.41 vs. 2.85 for lung cancer; and 0.44 vs. 0.86, 0.87 vs. 1.36, and 1.87 vs. 2.32 for MPN). Among lung cancer patients, scale scores increased significantly following a hospitalization, suggesting a worsening of functional status. CONCLUSIONS: The Katz ADL, Rosow-Breslau IADL, and Nagi scales collected in the MCBS demonstrate acceptable internal consistency and test-retest reliability among patients with pancreatic cancer, lung cancer, and MPN, and are consistent with clinical worsening following diagnosis or hospitalization. These results suggest that using retrospective data may allow researchers to conduct preliminary assessments of existing PRO instruments in new populations of interest and generate useful exploratory disease information before embarking on de novo PRO development. | Male | Humans | Patient Participation | United States | OK |
| Samya Z. | Nasr |  |  |  |  | Pediatric Pulmonology | 2013-07-00 Jul 2013 | PURPOSE: As the cystic fibrosis (CF) patient population median survival increase, the need for transitioning their care to adult care centers increase as well. We have a structured transition program since the early 1980s. The purpose of this study is to evaluate the experiences and opinions of patients in our adult CF center who went through a formal transition versus those who did not, in an attempt to evaluate the overall process and to identify means for improvement. METHODS: A questionnaire was given to adult CF patients at the University of Michigan during a clinic visit or inpatient hospitalization, after consent was obtained. Inclusion criteria included diagnosis of CF in childhood, previous care in a pediatric center, and current participation in our adult CF clinic. RESULTS: A total of 91 patients completed the questionnaire. 44 went through our structured transition program. On average, patients who participated in a formal transition process had higher satisfaction with both CF programs, perceived health status, and independence but no differences in their level of anxiety about transferring to the adult program. Patient opinions regarding when to transfer care were considered more often in patients who participated in a transition program (88% vs. 62% and P = 0.02). CONCLUSION: Although structured transitions programs do not appear to decrease patient anxiety during this stressful period, it does appear to improve patient satisfaction, perceived health status, and independence. These findings suggest that establishing a transition program could be important in optimizing patient health, continuity of care and improving adherence. | Male | Humans | Patient Participation | Patient Satisfaction | OK |
| Christine | Rocca | Patrick | Boyle |  |  | Cochlear Implants International | 2010-06-00 Jun 2010 | | Male | Humans | Patient Participation | Questionnaires | OK |
| Nicolette | Spera | Andrea | Whelan | Alison | Nightingale | Systematic Reviews | 2012-00-00 2012 | BACKGROUND: In April 2005, researchers based at the Medical Research Council Clinical Trials Unit, set out to involve women affected by cervical cancer in a systematic review and meta-analysis of individual patient data to evaluate treatments for this disease. Each of the women had previously been treated for cervical cancer. Following completion of the meta-analysis, we aimed to evaluate the process of involvement from the researcher and research partner perspective. METHODS: An advisory group was first established to give advice on recruiting, supporting and involving women and led to efforts to recruit women to take part in the systematic review using different approaches. Evaluation of the process and outcomes of the partnership between the systematic reviewers and the patients, in respect to what the partnership achieved; what worked well and what were the difficulties; what was learned and the resource requirements, took place during the conduct of the meta-analysis and again after completion of the project. RESULTS: Six women, each of whom had received treatments for cervical cancer, were recruited as Patient Research Partners and five of these women subsequently took part in a variety of activities around the systematic review. They attended progress meetings and all but one attended a meeting at which the first results of the review were presented to all collaborators and gave feedback. Three of the women also became involved in a further related research project which led to an editorial publication from the patient perspective and also participated, along with two lead researchers, in the evaluation of the process and outcomes. While they were generally positive about the experience, one Patient Research Partner questioned the extent of the impact patients could make to the systematic review process. CONCLUSIONS: In general, researchers and patient research partners felt that they had learned a lot from the process and considered it to have been a positive experience. The researchers felt that because of resource implications, patient involvement in future systematic reviews would probably have to be prioritized to those in which the greatest impacts could be achieved. | Humans | Patient Participation | Questionnaires | Female | OK |
| Todd G. | Morrison | Brian E. | McGuire |  |  | Brain Injury | 2013-00-00 2013 | OBJECTIVES: Quality-of-life (QoL) measures may be useful in quantifying the personal impact of an acquired brain injury and as an indicator of the effectiveness of service provision. This study investigated the validity of the patient generated index (PGI) as a measure of QoL with a sample of adults who had sustained a severe traumatic brain injury. METHOD: Fifty-five participants with an acquired brain injury were selected from four regional rehabilitation centres. Each participant completed validated questionnaires measuring subjective satisfaction with life, community integration, emotional distress, socially desirable responding and the patient generated index. RESULTS: The criterion-related validity of the PGI was demonstrated through a statistically significant positive correlation with life satisfaction. Evidence of construct validity was observed through significant correlations with measures of emotional distress and community integration. Depression had a strong negative association with QoL. Social desirability correlated significantly with both indices of QoL, suggesting that this variable may be treated as a covariate. CONCLUSION: The results offer some preliminary evidence for the validity of the PGI as a measure of QoL in people with severe brain injury. | Male | Humans | Patient Participation | Questionnaires | OK |
| Nicolaas C. | Schaper | Maartje H. G. | Schoonus-Spit | Monique A. J. | Janssen | Patient Education and Counseling | 2012-02-00 Feb 2012 | OBJECTIVE: The objective in this study was to evaluate a web-based type 2 diabetes self-management education programme aimed at improving knowledge, encouraging active patient participation and providing supportive self-management tools. METHODS: (1) An effect evaluation was conducted using a randomized controlled trial with a pre-test and post-test design (n=99) and a knowledge questionnaire. (2) A user evaluation was conducted using an online questionnaire (n=564) and one-on-one interviews (n=11) to examine the perceived quality, use of functionalities and use of the programme as a supportive tool in education. RESULTS: The effect evaluation showed a significant intervention effect (p<0.01) on knowledge. The user evaluation showed high satisfaction with the programme's content, credibility and user-friendliness. However, functionalities and self-management tools were used by less than half of the participants. CONCLUSION: The programme can improve knowledge, but it is not fully used as intended. A more optimal use of the programme is necessary for higher efficacy. PRACTICE IMPLICATIONS: The use of mostly spoken text instead of written text was highly appreciated and could be used more often for educational websites. Furthermore, health care practitioners need support in implementing new educational programmes during consultations. | Male | Humans | Patient Satisfaction | Questionnaires | OK |
| S. M. | Cox | L. C. | Li |  |  | Chronic Illness | 2010-09-00 Sep 2010 | BACKGROUND: Sociological understandings of chronic illness have revealed tensions and complexities around help-seeking. Although ethics underpins healthcare, its application in the area of chronic illness is limited. Here we apply an ethical framework to interview accounts and identify ethical challenges in the early rheumatoid arthritis (RA) experience. METHODS: In-depth interviews were conducted with eight participants who had been diagnosed with RA in the 12 months prior to recruitment. Applying the concepts of autonomous decision-making and procedural justice highlighted ethical concerns which arose throughout the help-seeking process. Analysis was based on the constant-comparison approach. RESULTS: Individuals described decision-making, illness actions and the medical encounter. The process was complicated by inadequate knowledge about symptoms, common-sense understandings about the GP appointment, difficulties concerning access to specialists, and patient-practitioner interactions. Autonomous decision-making and procedural justice were compromised. The accounts revealed contradictions between the policy ideals of active self-management, patient-centred care and shared decision-making, and the everyday experiences of individuals. CONCLUSIONS: For ethical healthcare there is a need for: public knowledge about early RA symptoms; more effective patient-practitioner communication; and increased support during the wait between primary and secondary care. Healthcare facilities and the government may consider different models to deliver services to people requiring rheumatology consults. | Male | Decision Making | Humans | Questionnaires | OK |
| Mary | Ferguson-Pare | Diane | Doran |  |  | Canadian Journal of Neuroscience Nursing | 2010-00-00 2010 | PURPOSE: to examine the extent to which staff nurses provided patient-centred care (PCC), as perceived by staff nurses and patients, and to explore the relationships between implementation of PCC and patient outcomes. DESIGN: A descriptive correlational design with repeated measures was used. Descriptive statistics, correlations and t-tests were calculated. METHODS: Data were collected from 63 nurses and 44 patients admitted to cardiology, neurology/neurosurgery and orthopedic units. Nurses' and patients' perception of implementation of dimensions of PCC, and patient outcomes were measured with validated instruments. FINDINGS: Overall, nurses and patients reported implementation of PCC to a moderate extent. Provision of different aspects of PCC was associated with high levels of patient self-care. CONCLUSIONS: Implementation of PCC is expected to improve patient outcome by increasing patient self-care ability and improving satisfaction with care and quality of life. CLINICAL RELEVANCE: The findings will guide further improvement in the implementation of PCC to continuously enhance quality of nursing care, the patients' hospital experience and readiness for discharge. | Male | Humans | Patient Participation | Patient Satisfaction | OK |
| K. D. | Anderson |  |  |  |  | Spinal Cord | 2013-01-00 Jan 2013 | STUDY DESIGN: Pass-code protected web survey. OBJECTIVES: Defining exercise participation barrier prevalence and association with exercise participation status in adults with spinal cord injury (SCI). SETTING: World-wide web. METHODS: Individuals ≥18 years with ShCI in the United States completed a pass-code protected website survey (N=180). Odds ratios (OR) and OR 95% confidence interval (95% CI) assessed association between barrier presence and exercise participation. RESULTS: No differences existed between exercisers and non-exercisers with respect to age, gender, injury level, injury duration, education level, or employment status. A larger percentage of non-exercisers reported household annual incomes <$7,500. The five most prevalent barriers were not associated with participation status (all OR 95% CI included 1). Low prevalence (≤13%) characterized four of the five barriers most strongly related to being a non-exerciser. Identifying too lazy, too difficult, or no interest as a barrier decreased odds of being an exerciser by 86%, 83%, and 71%, respectively. Not liking exercise decreased the odds of being an exerciser by 90%. CONCLUSION: Highly prevalent barriers were not associated with exercise participation status, whereas low prevalence barriers were strongly related to being a non-exerciser. Internal barriers had the strongest association with exercise participation status. The possible association between socioeconomic factors and exercise participation may be underappreciated. The most effective interventions to increase exercise participation may be multifocal approaches to enhance internal perceptions about and motivation to exercise, increase knowledge of how and where to exercise, while also reducing program and transportation financial costs. | Male | Humans | Patient Participation | Questionnaires | OK |
|  |  |  |  |  |  | Scandinavian Journal of Public Health | 2010-08-00 Aug 2010 | AIMS: The paper presents a recent Danish programme using exercise to alter the behaviour and body image of drug addicts. METHODS: 38 participants (23 male and 15 female) took part in groups three times per week for a minimum of two to a maximum of six months. Self-reported data combined with the European Addiction Severity Index (EuropASI) collected at initial admission and in follow-up interviews included information on drug use, body image, self-confidence and motivation to change behaviour. RESULTS: The completion rate of the participants during the training period was on average 52%, which is considered as a success in treatments with drug abusers, usually characterized by a low compliance and commitment. The results of the participants who completed the programme (n = 20) showed an increased oxygen uptake of an average of 10%, improved self-reported quality of life and a higher energy level for the majority of the participants. The addicts obtained a better body image, became more sensitive to physical pain and disorders and reduced their drug intake during the training period. The long-term effect showed that five of the 20 abusers interviewed reported that they still had not taken drugs, 10 had downgraded their intake, four experienced no change at all and one died through an overdose. CONCLUSIONS: The results show that physical exercise can provide important support in the treatment of drug abuse and that the main problem is maintaining change in behaviour and peer group influence to ensure long-term change. | Time Factors | Male | Humans | Patient Participation | OK |
| M. | Reece | B. | Van Der Pol |  |  | International journal of STD & AIDS | 2013-04-00 Apr 2013 | Routine screening is a key component of sexually transmitted infection (STI) prevention and control; however, traditional programmes often fail to effectively reach men and women in hidden communities. To reduce prevalence, we must understand the programmatic features that would encourage utilization of services among asymptomatic individuals. Using incentivized snowball sampling, 44 women and men recently engaging in transactional sex were recruited (24 women, 20 men); median age 37 years. Respondents were offered the opportunity to collect genital, oropharyngeal and rectal samples for STI testing and completed a face-to-face interview about their experience with self-obtained sampling. Interviews were analysed using qualitative methods. Participants were unaware of potential risk for STI, but found self-sampling in non-clinical settings to be acceptable and preferable to clinic-based testing. All participants collected genital specimens; 96% and 4% collected oropharyngeal and rectal specimens, respectively. The burden of disease in this population was high: 38% tested positive for at least one STI. We detected multiple concomitant infections. Incorporating field collection of self-obtained samples into STI control programmes may increase utilization among high-risk populations unlikely to access clinic-based services. High infection rates indicate that individuals engaging in transactional sex would benefit from, and be responsive to, community-based self-sampling for STI screening. | Male | Humans | Patient Participation | Questionnaires | OK |
| V. | Díaz Díaz | S. | Estalella Mendoza | L. | Díaz Gómez | Revista De Calidad Asistencial: Organo De La Sociedad Española De Calidad Asistencial | 2010-08-00 2010 Jul-Aug | BACKGROUND: Adjuvant! Online estimates 10-year recurrence and mortality outcomes for breast cancer patients and predicts the effect of each type of treatment. Our purpose was to test the applicability by only analysing mortality estimations. METHOD: We present estimations of 66 women with definitive surgery and axillary staging for unilateral, unicentric, invasive adenocarcinoma, without metastatic or residual disease. Age, co-morbidity, estrogen receptor status, histological grade, tumor size, number of positive nodes, and hormone therapy or chemotherapy option, were the variables required. RESULTS: Median of survival estimations was 77%, cancer mortality 18% and mortality for other reasons 5%. The average of absolute risk reduction (ARR) with hormone therapy was 4%, with chemotherapy 4.5% and with combined treatment 7%. All the patients with some benefit decided to receive hormone therapy. Forty-three patients (65%) decided to receive chemotherapy and 23 (35%) did not. The average risk reduction with chemotherapy was 2% in those who decided not to receive chemotherapy and 8% in those who decided to receive it. There was an association between a chemotherapy decision and the estimation of the risk of breast cancer mortality (P=0.0001), risk of mortality for other reasons (P=0.038), and the ARR (P=0.0001). There were 6% of the patients with an ARR of 1%, 50% between 2-5% and 61.8% between 6-10%, who chose chemotherapy. CONCLUSIONS: All women opted for hormone therapy regardless of benefit. The reasons for choosing chemotherapy were the prognosis itself and the magnitude of benefit. Some patients decided to choose chemotherapy even when the benefit was minimal. | Decision Making | Humans | Patient Participation | Female | OK |
| Linda | Wilson |  |  |  |  | International Journal of Speech-Language Pathology | 2010-04-00 Apr 2010 | This paper investigates the continuum of care experienced by adults and their significant others following a moderate to severe traumatic brain injury (TBI) in Victoria, Australia. Clinical care guidelines exist defining best practice in TBI rehabilitation, but little is currently known about the experiences of those who sustain a TBI, their needs and the barriers they encounter when attempting to access services. This paper presents the journeys of 202 Victorians admitted for acute care following a moderate to severe TBI over 4 years. Results of this study indicate very few participants report receiving services in line with recommendations made in clinical care guidelines. In addition reported access to services was noted to vary according to allocated healthcare funding (i.e., compensable vs. private vs. public). Clinicians' consideration of healthcare consumers' experiences are essential if services provided are to match consumers' needs. | Time Factors | Male | Humans | Patient Participation | OK |
| Pamela L. | Wolters | Brigitte C. | Widemann |  |  | Contemporary Clinical Trials | 2011-01-00 Jan 2011 | BACKGROUND: With increasing knowledge of the molecular pathways contributing to the progression of neurofibromatosis type 1 (NF1)-related plexiform neurofibroma (PN), the number of clinical trials for PNs has increased. However, little is known about the experiences of families with children with NF1 participating in clinical trials. METHOD: A 47-item anonymous survey, designed to assess experiences in research, was sent to parents with a child with NF1 and PNs who was evaluated at the National Cancer Institute (NCI) for a clinical trial. RESULTS: Sixty-four (85%) parents completed the paper or online questionnaire. Fifty-nine percent of the children were male; 44% were 11 years or older upon enrollment. Most parents reported understanding the purpose of the study, possible side effects, and their right to withdraw. Of respondents whose child had participated in a placebo-controlled trial, 100% said they understood the reason for the placebo at least somewhat. Seventy-four percent felt that study participation helped their child; most would "definitely" or "probably" participate in a future study, including a placebo-controlled trial, and particularly those assessing cognitive functioning or pain. Overall satisfaction with participation was high and correlated with fewer transportation problems, fewer study-related financial difficulties, and fewer school problems for the child (ps<.05). CONCLUSIONS: Most parents of children with NF1 who participated in research at the NCI reported a positive impact and would participate in future studies. Respondents identified several areas for improvement. This report may help plan future studies to optimize experiences of children and their families enrolled in clinical trials. | Male | Humans | Patient Participation | Patient Satisfaction | OK |
| V. | Radhakrishnan | J. | McCarthy |  |  | Journal of intellectual disability research: JIDR | 2012-09-00 Sep 2012 | BACKGROUND: Patient experience of those accessing mental health services has been found to be different between ethnic groups. Although the needs of people with intellectual disabilities (ID) from different ethnic communities are being increasingly recognised, little has been published about their experiences of mental health services. The aim of this study was to establish whether there are any differences in the experiences of people with ID and mental health problems from two ethnic communities in South London. METHOD: A two-round Delphi process was utilised. White British and Black or Black British service users from a specialist community-based mental health service for adults with ID completed a specially compiled questionnaire. Statements on participants' experiences, including satisfaction with care, staff members' attitudes, cultural awareness and level of support, were rated using a Likert scale. RESULTS: Twenty-four out of 32 participants (75%) completed both rounds of the Delphi consultation. Consensus (≥80% agreement with the group median) was reached for 20 items in the White group and five items in the Black group. All responses that reached consensus were positive about the services that were being received. The Black group were less positive about a range of their experiences, including the use of medication. CONCLUSIONS: People with ID from two ethnic groups were able to successfully complete a Delphi consultation regarding their experiences of mental health services. Broad consensus on positive experiences of services was reached in the White group but not for the Black participants. | Male | Humans | Patient Participation | Patient Satisfaction | OK |
| Shanthi | Pal | Sten | Olsson | Kees | van Grootheest | Drug Safety | 2012-01-01 Jan 1, 2012 | BACKGROUND: Patients are important stakeholders in pharmacovigilance; however, little formal evaluation has been undertaken of existing patient reporting schemes within and outside Europe. If patient reporting is to be recognized as beneficial for pharmacovigilance and further optimized, methodology and best practice must be internationally shared and promoted. OBJECTIVE: This study aims to review the methods of patient reporting of adverse drug reactions (ADRs) in 11 countries worldwide and to compare different aspects of their experiences. METHODS: A survey based on telephone interviews, e-mail discussions and field visits of existing practices in consumer and patient reporting of ADRs was performed in the second half of 2010. RESULTS: The start dates of the patient reporting schemes vary from 1964 (Australia) to 2010 (Norway). The number of patient reports per country varies widely. Most countries would ideally spend more resources on making the public aware of the possibility that patients can report ADRs. Most countries have three different ways for patients to report ADRs - a paper form, an electronic form on a website or by telephone. The level of sophistication of the electronic forms varies. The route of handling of patients' and healthcare professionals' ADR reports is the same for most countries. Personalized feedback on the reported association for each report is only given by four organizations. All countries have guidelines for the identification of patients in the database and most undertake checks for duplicate reports. In all countries, with the exception of Norway, it is possible to ask patients for follow-up. None of the organizations seek medical confirmation for each patient report. There is a difference between countries that do a causality assessment for each incoming ADR report and countries that only do a causality assessment of reports when they are looking at a potential signal. All countries assess the seriousness of reports, mostly by using the criteria of the CIOMS committee. In all countries, patient reports are used for signal detection purposes and in publications about ADRs. The Netherlands and the UK are actively evaluating their patient reporting schemes. None of the organizations hired additional staff when they started with patient reporting. CONCLUSIONS: This study provides a comprehensive review on the methods used in patient reporting of ADRs. Although there are some differences in the way various countries handle patient reports of ADRs, the importance of giving the public the opportunity to report and the additional scientific value of the collected data is widely recognized by the countries who participated in this survey. | Humans | Patient Participation | Health Surveys | Databases, Factual | OK |
|  |  |  |  |  |  | Nurse Education Today | 2012-02-00 Feb 2012 | Assessment tools were designed to provide health and social care students with multi-sourced, interprofessional feedback in practice. This includes feedback from service users. Third year medical students at the University of Leeds were given accesses to 4 assessment tools whilst in practice. Completed assessments were then sent to the university where service users and carers worked with university tutors to give further feedback and comment on the overall development of students. Three service users then took part in a focus group and one provided written feedback. Four key themes were identified from the focus group: • Preparation and support • The design of the tools • The process of using the tools in practice • Feedback. We found that the project provided both challenges and rewards for all involved. The service user educators involved were able to bring a different and valuable perspective to formative feedback. The combination of their personal and professional experiences, along with the preparation they had received, helped bridge the gap between service users in practice and university based tutors. The findings from this study went on to inform a review of the assessment tools and revised versions are now being used. | Humans | Patient Participation | Interprofessional Relations | Great Britain | OK |
| Sarah-Maude | Deschênes | Marie-Pierre | Gagnon | France | Légaré | Implementation science: IS | 2011-00-00 2011 | BACKGROUND: Shared decision making (SDM), a process by which health professionals and patients go through the decision-making process together to agree on treatment, is a promising strategy for promoting diet-related decisions that are informed and value based and to which patients adhere well. The objective of the present study was to identify dietitians' salient beliefs regarding their exercise of two behaviors during the clinical encounter, both of which have been deemed essential for SDM to take place: (1) presenting patients with all dietary treatment options for a given health condition and (2) helping patients clarify their values and preferences regarding the options. METHODS: Twenty-one dietitians were allocated to four focus groups. Facilitators conducted the focus groups using a semistructured interview guide based on the Theory of Planned Behavior. Discussions were audiotaped, transcribed verbatim, coded, and analyzed with NVivo8 (QSR International, Cambridge, MA) software. RESULTS: Most participants stated that better patient adherence to treatment was an advantage of adopting the two SDM behaviors. Dietitians identified patients, physicians, and the multidisciplinary team as normative referents who would approve or disapprove of their adoption of the SDM behaviors. The most often reported barriers and facilitators for the behaviors concerned patients' characteristics, patients' clinical situation, and time. CONCLUSIONS: The implementation of SDM in nutrition clinical practice can be guided by addressing dietitians' salient beliefs. Identifying these beliefs also provides the theoretical framework needed for developing a quantitative survey questionnaire to further study the determinants of dietitians' adoption of SDM behaviors. | Decision Making | Humans | Questionnaires | Female | OK |
| Lindsay A. | Thompson | Darren A. | DeWalt | Dennis A. | Revicki | Respiratory Research | 2013-00-00 2013 | BACKGROUND: Little is known about factors contributing to children's asthma control status and health-related quality of life (HRQoL). The study objectives were to assess the relationship between asthma control and asthma-specific HRQoL in asthmatic children, and to examine the extent to which parental health literacy, perceived self-efficacy with patient-physician interaction, and satisfaction with shared decision-making (SDM) contribute to children's asthma control and asthma-specific HRQoL. METHODS: This cross-sectional study utilized data collected from a sample of asthmatic children (n = 160) aged 8-17 years and their parents (n = 160) who visited a university medical center. Asthma-specific HRQoL was self-reported by children using the National Institutes of Health's Patient-Reported Outcomes Measurement Information System (PROMIS) Pediatric Asthma Impact Scale. Satisfaction with SDM, perceived self-efficacy with patient-physician interaction, parental health literacy, and asthma control were reported by parents using standardized measures. Structural equation modeling (SEM) was performed to test the hypothesized pathways. RESULTS: Path analysis revealed that children with better asthma control reported higher asthma-specific HRQoL (β = 0.4, P < 0.001). Parents with higher health literacy and greater perceived self-efficacy with patient-physician interactions were associated with higher satisfaction with SDM (β = 0.38, P < 0.05; β = 0.58, P < 0.001, respectively). Greater satisfaction with SDM was in turn associated with better asthma control (β = -0.26, P < 0.01). CONCLUSION: Children's asthma control status influenced their asthma-specific HRQoL. However, parental factors such as perceived self-efficacy with patient-physician interaction and satisfaction with shared decision-making indirectly influenced children's asthma control status and asthma-specific HRQoL. | Male | Decision Making | Humans | Patient Satisfaction | OK |
|  |  |  |  |  |  | BMC public health | 2012-00-00 2012 | BACKGROUND: The aim of this study was to develop an instrument to measure laypeople's beliefs about the nature of medical knowledge and knowing (the EBAM). Such beliefs should be a target of increased research interest because they influence how people handle medical information, for example in shared decision making. METHODS: An online survey was completed by 284 participants. Items assessed different aspects of laypeople's epistemic beliefs about medicine and explicitly focused on the appearance of medical knowledge in everyday life and the evaluation of different sources as a way to justify knowledge. RESULTS: Factor analysis yielded a five-factor solution for the instrument. Dimensions covered by the instrument are certainty of medical knowledge, credibility of medical textbooks, credibility of medical information on the Internet, justification of medical knowledge, and preliminarity of medical knowledge. CONCLUSIONS: Results indicate that laypeople have meaningful beliefs about the nature of medical knowledge and the trustworthiness of different sources. The instrument developed seems promising for measuring laypeople's epistemic beliefs about medicine, which may help to increase patients' compliance in medical decision making. | Male | Humans | Questionnaires | Young Adult | OK |
| Robert | Feldman | Le | Zhou |  |  | Health Expectations: An International Journal of Public Participation in Health Care & Health Policy | 2014-12-00 décembre 2014 | Context: Existing measurements of patient preferences cover only a limited range of health information and participation in decision making. A broader approach is necessary to understand the breadth and variations in patient preferences. Objective: To explore the breadth and variances in patient preferences for health information and participation in decision making and to understand the relationship between age and each type of preference. Design: The Health Information Wants Questionnaire (HIWQ) was administered during May–December 2010 to gather data about the information and corresponding decision‐making autonomy participants would want in seven areas: diagnosis, treatment, laboratory tests, self‐care, complementary and alternative medicine (CAM), psychosocial factors and health‐care providers. Setting: A large state university, public libraries and senior centres in Maryland, USA. Participants: A convenience sample of 438 individuals, including 226 undergraduates (mean age = 20; SD = 2.15) and 212 community‐dwelling older adults (mean age = 72; SD = 9.00). Main Outcome Measures Ratings on the information and decision‐making items of the HIWQ. Results: Participants expressed higher levels of preference for information than for participation in decision making on six of seven subscales. On the psychosocial subscale, they expressed stronger desire for participation in decision making than for information. Age had no predictive effect on the overall preferences or specific preferences for information and participation in decision making about standard treatments and CAM. The predictive effect of age on the other types of preferences varied significantly. Conclusions: Physicians should take into account the breadth and variations in patient preferences. The predictive effect of age on patient preferences varied depending on the specific area of preferences. (PsycINFO Database Record (c) 2014 APA, all rights reserved). (journal abstract) | Shared decision making | Decision Making | Questionnaires | Psychometrics | Doublon |
| Robert | Feldman | Le | Zhou |  |  | Health Expectations: An International Journal of Public Participation in Health Care and Health Policy | 2014-12-00 Dec 2014 | CONTEXT: Existing measurements of patient preferences cover only a limited range of health information and participation in decision making. A broader approach is necessary to understand the breadth and variations in patient preferences. OBJECTIVE: To explore the breadth and variances in patient preferences for health information and participation in decision making and to understand the relationship between age and each type of preference. DESIGN: The Health Information Wants Questionnaire (HIWQ) was administered during May-December 2010 to gather data about the information and corresponding decision-making autonomy participants would want in seven areas: diagnosis, treatment, laboratory tests, self-care, complementary and alternative medicine (CAM), psychosocial factors and health-care providers. SETTING: A large state university, public libraries and senior centres in Maryland, USA. PARTICIPANTS: A convenience sample of 438 individuals, including 226 undergraduates (mean age = 20; SD = 2.15) and 212 community-dwelling older adults (mean age = 72; SD = 9.00). MAIN OUTCOME MEASURES: Ratings on the information and decision-making items of the HIWQ. RESULTS: Participants expressed higher levels of preference for information than for participation in decision making on six of seven subscales. On the psychosocial subscale, they expressed stronger desire for participation in decision making than for information. Age had no predictive effect on the overall preferences or specific preferences for information and participation in decision making about standard treatments and CAM. The predictive effect of age on the other types of preferences varied significantly. CONCLUSIONS: Physicians should take into account the breadth and variations in patient preferences. The predictive effect of age on patient preferences varied depending on the specific area of preferences. | | | | | OK |
| Marc A. | Childress |  |  |  |  | Health Education & Behavior: The Official Publication of the Society for Public Health Education | 2013-06-00 Jun 2013 | OBJECTIVE: To further conceptualize and operationalize patient activation (PA), using measures from patient, physician, and researcher perspectives. DATA SOURCE/STUDY SETTING: Multimethod observation in 2010 within a family medicine clinic. STUDY DESIGN: Part of an intervention with 130 patients with type 2 diabetes, this observational study further looked at PA in 19 physician-patient dyads. Data Collection. Observations occurred in a teaching hospital, which served as recruiting and study site. PRINCIPAL FINDINGS: PA correlated with knowledge, self-efficacy, promotion orientation, and exercise intent. Patient-reported PA did not correlate with researcher-observed or physician-reported PA behavior. Researcher-observed PA correlated with physician-observation items. CONCLUSIONS: Results provide evidence for measuring different perspectives in studies of PA. When patients report they are activated in self-management, behavior does not indicate they are active in clinical communication, a critical component of collaborative decision making. | Male | Humans | Patient Participation | Physician-Patient Relations | OK |
| Karen | Cox | Eleanor | Wilson | Penny | Howard | Health (London, England: 1997) | 2011-03-00 Mar 2011 | Patient information is widely regarded both as a resource and an entitlement: a means of 'empowering' patients to behave as 'consumers' of health care. Patient 'satisfaction' has come to be regarded as an important outcome of care. This article presents qualitative interview data regarding the experience of patient information provision and the results of a self-completed Information Satisfaction Questionnaire (ISQ) among patients and relatives affected by cancer. It considers the implications of the differences between these for service evaluation and current policy implementation promoting patients as informed and expert consumers of health care. The study findings contribute to growing evidence that the high rate of patients' expressed satisfaction with different aspects of service provision as indicated by structured questionnaire responses is largely an artefact of the method of data collection. Accounts of negative experiences were common, but did not translate into expressed criticism or overt dissatisfaction. It is important that the limitations of such surveys are contextualized in relation to qualitative findings such as those of the present study. Especially in the face of serious and life-threatening illness, professional constructs such as 'information delivery', 'satisfaction' and 'shared decision making' have little resonance for many patients, who prefer to trust in professional expertise and to eschew the acquisition of specialist knowledge and active involvement in decisions about health care. | Male | Humans | Patient Participation | Patient Satisfaction | OK |
| Karen | Cox | Eleanor | Wilson | Penny | Howard | Health: An Interdisciplinary Journal for the Social Study of Health, Illness and Medicine | 2011-03-00 mars 2011 | Patient information is widely regarded both as a resource and an entitlement: a means of ‘empowering’ patients to behave as ‘consumers’ of health care. Patient ‘satisfaction’ has come to be regarded as an important outcome of care. This article presents qualitative interview data regarding the experience of patient information provision and the results of a self-completed Information Satisfaction Questionnaire (ISQ) among patients and relatives affected by cancer. It considers the implications of the differences between these for service evaluation and current policy implementation promoting patients as informed and expert consumers of health care. The study findings contribute to growing evidence that the high rate of patients’ expressed satisfaction with different aspects of service provision as indicated by structured questionnaire responses is largely an artefact of the method of data collection. Accounts of negative experiences were common, but did not translate into expressed criticism or overt dissatisfaction. It is important that the limitations of such surveys are contextualized in relation to qualitative findings such as those of the present study. Especially in the face of serious and life-threatening illness, professional constructs such as ‘information delivery’, ‘satisfaction’ and ‘shared decision making’ have little resonance for many patients, who prefer to trust in professional expertise and to eschew the acquisition of specialist knowledge and active involvement in decisions about health care. (PsycINFO Database Record (c) 2012 APA, all rights reserved). (journal abstract) | Patient Satisfaction | Questionnaires | Therapeutic Processes | Client Satisfaction | OK |
|  |  |  |  |  |  | Pharmacy world & science: PWS | 2010-10-00 Oct 2010 | OBJECTIVE: To characterize the communication ritual in the pharmacist-customer dyad during a blood pressure measurement and counselling episode. SETTING: A Portuguese urban community pharmacy. METHOD: An exploratory, cross-sectional design was used. Participants were purposively selected and data collected via audio recording, as well as demographics and clinical information via questionnaire. Encounters' verbal content was transcribed verbatim, utterances identified, time stamped, and classified according to a coding scheme of fifteen categories. All data was statistically analyzed using SPSSv17. MAIN OUTCOME MEASURES: Four dialogue structures: speaker turn, interactivity, turn density and turn duration measurements. RESULTS: From a total of 51 participants, 72.5% were female with a median age of 66 years. The average systolic blood pressure was 140 mmHg, while the diastolic was 78 mmHg. The blood pressure measurement episode lasted for 5:35 min, with an average of 81 utterances. From all utterances registered, 55.3% were produced by the customer. Visits averaged 38 speaker turns, with an interactivity rate of approximately 7 turns per episode minute. For pharmacists, turn duration averaged 7.0 s and turn density 2.1 utterances. The customers' turns comprised a mean of 8.0 s, with 2.4 utterances. Longer episodes were related to more speaker turns and greater customer turn density and duration, but lower dialogue interactivity. The interactivity rate was also lower when the customers' utterances increased. Pharmacists asked more questions (essentially closed ones), while the customers gave more information. No significant associations were observed between elderly/non-elderly and gender in relation to all communication variables. However, an increased number of speaker turns and closed-questions were associated to a higher systolic pressure. CONCLUSION: It seems that pharmacists tend to control the content of the dialogue, while customers have more influence on the visit duration and interactivity. Specific hypertensive episodes induce a higher information search. Since the closed questioning format was prevalent, it seems that open information exchange was limited, mainly serving confirmation purposes rather than having a true exploratory nature. Although talk dominance is balanced, further analysis is required to better inform these results, which would confirm the low interactivity and the reduced information-seeking behaviour showed in the counselling episodes. | Male | Humans | Patient Participation | Questionnaires | OK |
| Martin | Smalbrugge | Dirk L. | Knol | Henriëtte E. | van der Horst | BMC geriatrics | 2013-00-00 2013 | BACKGROUND: In clinical settings, it is important for health care providers to measure different aspects of functioning in older adults with joint pain and comorbidity. Besides the use of distinct measures, it could also be attractive to have one general measure of functioning that incorporates several distinct measures, but provides one summary score to quantify overall level of functioning, for example for the identification of older adults at risk of poor functional outcome. Therefore, we selected four measures of functioning: Physical Functioning (PF), Activities of Daily Living (ADL), Instrumental Activities of Daily Living (IADL) and participation, and tested the possibility to aggregate these measures into one general measure of functioning. METHODS: A prospective cohort study of older adults (≥65 years) with joint pain and comorbidity provided baseline data (n = 407) consisting of PF (PF subscale, RAND-36; 10 items), ADL (KATZ index; 6 items), IADL (Lawton index; 7 items) and participation (KAP; 6 items). We tested two models with confirmatory factor analysis: first, a bifactor model with all four measures and second, a bifactor model with PF, ADL and IADL and a correlated but distinct subgroup factor for participation. Several model fit indexes and reliability coefficients, such as explained common variance (ECV) and omegas were computed for both models. RESULTS: The first model fitted the data well, but the reliability analysis indicated multidimensionality and unique information in the subgroup factor participation. The second model showed similar model fits, but better reliability; ECV = 0.67, omega-t = 0.94, low omega-s = 0.18-0.22 on the subgroup factors and high omega of 0.82 on participation, which all were in favour of the second model. CONCLUSIONS: The results indicate that PF, ADL and IADL could be aggregated into one general measure of functioning, whereas participation should be considered as a distinct measure. | Male | Humans | Patient Participation | Questionnaires | OK |
|  |  |  |  |  |  | Psycho-Oncology | 2010-10-00 Oct 2010 | OBJECTIVE: To evaluate the correlation and concordance between patients' and physicians' estimations of prognoses before initiation of the conditioning regimen for allogeneic haematopoietic stem-cell transplantation. METHODS: A total of 123 patients and their attending physicians were asked to estimate a prognosis on a six-point scale. The patients were also asked to fill out questionnaires addressing their psychological state and coping. RESULTS: The mean prognostic estimations differed by 1.17 points (p<0.001), with the patients being more optimistic than the physicians. With respect to concordance: Pearson correlation r=0.024 (ns); unweighted kappa and kappa with linear weighting are 0.115 and 0.068, respectively. The prognostic estimates of the patients correlated with their psychological state, but not with the objective disease- or treatment-related variables, whereas the physicians' estimates were partially based on such objective factors. CONCLUSIONS: A clear significant association between actual survival and the physicians' estimates, but not the patients' estimates, was observed. If agreement regarding the prognosis exists, the relationship between physicians' and patients' estimates is probably non-linear. Assessing one's chances of being cured is a highly emotional task, and psychological processes such as denial or repression most likely play a decisive role. Moreover, collusion between the patient and physician may be inevitable in this situation. Whether it is desirable to gain concordance and who will benefit from such efforts must be discussed and empirically studied. | Male | Decision Making | Humans | Patient Participation | OK |
| Anil | Coumar | Larry S. | Dean | Frederick A. | Matsen | IEEE transactions on information technology in biomedicine: a publication of the IEEE Engineering in Medicine and Biology Society | 2011-03-00 Mar 2011 | With the growing availability of health information on the Web, people are becoming more knowledgeable on their health conditions and treatment options, and more patients seek specialists by themselves. To aid patients in requesting self-referrals, we have developed and evaluated a web-based self-referral system in three specialty clinics at the University of Washington. Two clinics adopted the system for routine clinical use, while the third clinic decided not to. A major difference between these two groups was in how fast online requests from patients were handled, which significantly influenced patients' satisfaction. Clinic's preparedness for handling the temporarily increased workload due to the introduction of a new health information system played a role as well. Also, we noticed that the physician leadership/championship made a difference in the acceptance of our system. | Humans | Patient Participation | Patient Satisfaction | Internet | OK |
| Abdullah A. | Al-Sayyari | Hani | Tamim | Yaser | Babgi | Saudi Journal of Kidney Diseases and Transplantation: An Official Publication of the Saudi Center for Organ Transplantation, Saudi Arabia | 2010-05-00 May 2010 | The current cross sectional study is based on a questionnaire database on patients with end-stage renal disease (ESRD) to determine their preferences about end-of-life care and differences of certainty regarding the application of cardiopulmonary resuscitation and life sustaining measures in case of cardiac arrest. The study was performed on 100 patients on hemodialysis for at least 2 years and not on the transplant list in two tertiary hospitals in Saudi Arabia; King Fahad National Guard in Riyadh and King Faisal Specialist Hospital in Jeddah in March 2007. More than two thirds of the surveyed patients were willing to make decisive decisions. Having more than 5 children was the only factor significantly associated with the ability to make decisive decisions; there was an insignificant association with factors such as marital status or non-Saudi nationality. Factors such as self-perception or disease curability, previous admissions to hospital or intensive care units, prior knowledge of mechanical ventilation, or cardiopulmonary resuscitation did not have any influence on making certain decisions on end-of-life care. There was a significant lack of knowledge in our study patients of cardiopulmonary resuscitation, mechanical ventilation, and disease outcome. | Time Factors | Male | Decision Making | Humans | OK |
| Su-Jin | Kim |  |  |  |  | International Journal of Colorectal Disease | 2012-08-00 Aug 2012 | PURPOSE: Despite the Government's National Cancer Screening Program for colorectal cancer (CRC), the number of individuals participating in screening in Korea is low. Therefore, the aim of this study was to identify associations between relevant risk factors and the uptake of screening in Korea. METHODS: The Health Interview Survey sub-dataset derived from the Fourth Korean National Health and Nutrition Examination Survey (KNHANES IV) was used to evaluate participation in CRC screening and factors associated with attendance in individuals aged ≥50. Those that completed the questionnaire and not previously diagnosed with CRC were enrolled (8,042 subjects). Multi-dimensional covariates were considered as potential predictors for CRC screening in multivariate analyses. RESULTS: A total of 33.2% complied with the CRC screening recommendations. The following were associated with participation: age (aged 70 or older [ref], aged 70 or over; odds ratio (OR) 1.81, 95% confidence interval (CI) 1.54-2.14), marital status (OR 1.43, 95%CI 1.23-1.66), urban-dwelling (OR 1.16, 95%CI 1.02-1.32), education level (elementary school or less [ref], high school (OR 1.29, 95%CI 1.09-1.53), university or higher (OR 1.53, 95%CI 1.23-1.91)), household income (fourth quartile [ref], first quartile (OR 1.29, 95%CI 1.07-1.56)), private health insurance (OR 1.38, 95%CI 1.21-1.58), smoking (OR 1.35, 95%CI 1.43-1.60), self-reported depression (OR 0.79, 95%CI 0.68-0.92), and number of chronic diseases (0-3 [ref], ≥4 (OR 1.41, 95%CI 1.22-1.62)). CONCLUSION: To improve participation in CRC screening, appropriate strategies must be directed toward vulnerable populations, such as those with low socioeconomic status. | Male | Humans | Patient Participation | Female | OK |
| Laura A. | Siminoff | Susan A. | Flocke | Jennifer Elston | Lafata | Patient Education and Counseling | 2012-10-00 Oct 2012 | OBJECTIVE: To evaluate the patient, physician, and visit-related factors associated with patient ratings of positive physician relational communication. METHODS: Pre- and post-visit surveys were conducted with 485 patients attending a routine periodic health exam with one of 64 participating physicians. The audio-recorded visits were coded for elements of patient-physician communication including assertive responses, partnership building, question asking, supportive talk, and expressions of concern. RESULTS: Patient reports of positive physician relational communication were associated with patient perceptions of how well the physician understood the patient's health care preferences and values, a patient-physician interaction outside of the exam room, and physician-prompted patient expressions of concern. CONCLUSION: In addition to a patient's perception of their relationship with their physician going into the visit, relatively simple acts like extending the interaction beyond the exam room and ensuring that patients feel invited to express concerns they may have during the visit may influence patient perceptions of physician relational communication. PRACTICE IMPLICATIONS: This study offers preliminary support for the idea that relational communication and its associated benefits may be fostered through simple physician-driven acts such as interacting with patients outside of the exam room and encouraging patients to express concerns within the visit. | Male | Humans | Patient Participation | Patient Satisfaction | OK |
| Michelle R. | Simpson | Sheila | Reynolds |  |  | American Journal of Alzheimer's Disease and Other Dementias | 2010-06-00 Jun 2010 | This study describes new problems emerging over 6 weeks for nursing home residents with advanced dementia and factors associated with time to identify the problems. The sample of 65 developed 149 new acute problems or exacerbations of existing conditions over the 6 weeks of data collection. The majority of these problems involved uncontrolled pain, new infections, and severe psychoses. Nurse assessment skill was associated with a shorter time to identify the new problem and more time spent on the problem. A higher ratio of new to existing interventions was also associated with a shorter time to identify the problem. Other patient characteristics associated with time to identify problems included nonspecific vocalizations, physical signs, cognitive status, and length of stay. While future research is warranted, findings from this study highlight the frequency of problems requiring treatment and suggest that improved assessment of residents may decrease the time to identify new problems. | Male | Humans | Patient Participation | Questionnaires | OK |
| Manuel | García Ramírez | Carmen | Fernández Garrido | |  | Gaceta Sanitaria / S.E.S.P.A.S | 2011-12-00 2011 Nov-Dec | OBJECTIVE: To examine which contextual factors explain the implementation of the Patient Autonomy Law (LAP) by professionals involved in the surgical process. METHODS: A descriptive cross sectional study survey. It was conducted at the Hospital Virgen Macarena in Seville with the surgical staff. Dependent variable: the application of LAP. INDEPENDENT VARIABLES: the perception of socio-structural organizational characteristics according to Kanter's model (organizational culture, sociopolitical support, access to information, and access to resources), the perception of interprofessional collaboration, and knowledge of the LAP. We performed exploratory analysis, bivariate with parametric and nonparametric tests depending on the nature of the distributions; simple regression to determine the weight of each explanatory variable on the application of the LAP and, multiples introducing in the models those variables that had obtained significant regression coefficients. The analysis performed with a confidence level of 95%. RESULTS: PARTICIPANTS: 170 professionals (response rate: 60.28%). Behaviour according to the LAP is present in a medium-high level among physicians and nurses. Significant differences were observed in the application of the LAP according to the different sociolaboral variables and considering medical staff, the working environment and the specialty. Multiple regression analyses revealed the relationship between the application of the LAP and interprofessional collaboration, in physicians. CONCLUSIONS: Kanter's model and the scale of interprofessional collaboration, may contribute to the explanation of the influence of the hospital context in the implementation of the LAP and the results may guide us when designing strategies to implement the law. It would be necessary to overcome the traditional structures and create decentralized and flexible conditions that encourage teamwork and shared decision making. | Questionnaires | physicians | Cooperative Behavior | Informed Consent | OK |
| Annica | Öhrn | Hans | Rutberg | Mikael | Rahmqvist | BMC health services research | 2013-00-00 2013 | BACKGROUND: National, regional and local activities to improve patient safety in Sweden have increased over the last decade. There are high ambitions for improved patient safety in Sweden. This study surveyed health care professionals who held key positions in their county council's patient safety work to investigate their perceptions of the conditions for this work, factors they believe have been most important in reaching the current level of patient safety and factors they believe would be most important for achieving improved patient safety in the future. METHODS: The study population consisted of 218 health care professionals holding strategic positions in patient safety work in Swedish county councils. Using a questionnaire, the following topics were analysed in this study: profession/occupation; number of years involved in a designated task on patient safety issues; knowledge/overview of the county council's patient safety work; ability to influence this work; conditions for this work; and the importance of various factors for current and future levels of patient safety. RESULTS: The response rate to the questionnaire was 79%. The conditions that had the highest number of responses in complete agreement were "patients' involvement is important for patient safety" and "patient safety work has good support from the county council's management". Factors that were considered most important for achieving the current level of patient safety were root cause and risk analyses, incident reporting and the Swedish Patient Safety Law. An organizational culture that encourages reporting and avoids blame was considered most important for improved patient safety in the future, closely followed by improved communication between health care practitioners and patients. CONCLUSION: Health care professionals with important positions in the Swedish county councils' patient safety work believe that conditions for this work are somewhat constrained. They attribute the current levels of patient safety to a broad range of factors and believe that many different solutions can contribute to enhanced patient safety in the future, suggesting that this work must be multifactorial. | Humans | Patient Participation | Questionnaires | physicians | OK |
| S. | McCann | A. C. | Milne | G. | Maclennan | The Journal of Nutrition, Health & Aging | 2010-01-00 Jan 2010 | BACKGROUND: Older people are less likely to be included in clinical trials. Little is known about factors influencing older people's decisions about participating in clinical trials. OBJECTIVES: To examine the views of older people about participating in clinical trials. METHODS: Postal questionnaire to 801 participants who had completed the MAVIS nutrition trial, aged 65 yrs and older. Closed and open questions sought participants' views about factors important to them when deciding to take part in a trial, features of the MAVIS trial they liked and disliked and changes they would suggest. RESULTS: 540 (59% of MAVIS trial participants) returned the questionnaire. The most important reasons reported for taking part in the trial were helping the research team and medical knowledge, and helping other older people. Participants valued good communication with the trial staff and good organisation. Participants reported concerns about swallowing pills and taking a placebo. Participants reported that future participation in trials could be influenced by poor health status. LIMITATIONS: This questionnaire surveyed older participants who had taken part in a randomised controlled trial. It did not elicit the views of people who had withdrawn or never decided to take part in the trial. CONCLUSIONS: Older people report altruistic reasons for taking part in trials. Simple trial designs, which minimise demands on participants and maintain good communications should be preferred. Explaining the need for older people, despite poor health, to participate in trials may help the generalisability of clinical trials. | Male | Humans | Patient Participation | Questionnaires | OK |
| Winson Y. | Cheung |  |  |  |  | Medical Oncology (Northwood, London, England) | 2013-00-00 2013 | The human papillomavirus (HPV) vaccine helps to prevent cervical cancer. However, research indicates that public acceptance of the vaccine is suboptimal. Our aims were to evaluate the willingness of US women to use the HPV vaccine in their daughters, examine their current understanding of HPV, and determine the impact of HPV knowledge and other socio-demographic factors on their willingness to get their daughters vaccinated. Women aged ≥ 18 years were identified from the US Health Information National Trends Survey. We developed a 6-point composite scoring system based on individual responses to HPV-related questions to characterize personal understanding about HPV. Logistic regression models were constructed to explore the influence of the women's HPV knowledge level and additional socio-demographic factors on the willingness to use HPV in their daughters. There were 804 female respondents: mean age was 44.9 (SD = 2.53) years and 73 % were White. In total, 75 % of women indicated they would vaccinate their daughters against HPV. Mean knowledge score was 4.6 (SD = 0.80). While White race was associated with higher willingness to use the vaccine in their daughters (OR = 1.86, p = 0.04), HPV knowledge level was not (OR = 0.47, p = 0.22). Among US women, HPV knowledge level was high, but it was not associated with the willingness to vaccinate their daughters against HPV. Interventions focused on alleviating racial disparities might better modify the use of the HPV vaccine. | Humans | Patient Participation | Questionnaires | United States | OK |
|  |  |  |  |  |  | Patient Education and Counseling | 2012-06-00 Jun 2012 | OBJECTIVE: To assess information and decision making preferences of patients on active surveillance (AS), and the factors influencing their decision. METHODS: A cross-sectional sample of 180 patients on AS for <10 years completed a survey exploring the role men assumed with their physician in treatment decision making (TDM), factors influencing their decision to go on AS, and information preferences. RESULTS: Thirty-five percent of patients reported assuming an active role in TDM, 38% a collaborative role and 27% a passive role. Results suggest that patients<60 years prefer to play an active role in TDM whereas, men>70 years prefer to play a passive role. Available treatment options, eating a 'prostate friendly' diet, and non-traditional therapies were identified as the top three information preferences. Patients with higher levels of anxiety wanted access to more information compared to those with lower levels of anxiety. The urologists' recommendation was rated the most important factor influencing patients' decisions to go on AS. CONCLUSION: The urologist's recommendation for treatment continues to have the most influence on the decision to go on AS. Our results suggest that age has an impact on the role patients wish to assume in TDM. PRACTICE IMPLICATIONS: Assessments of patients' information and decision preferences, and levels of anxiety are suggested for all prostate cancer patients considering AS. | Male | Decision Making | Humans | Patient Participation | OK |
| Eucharia C. | Iwuanyanwu | Bahareh | Tavana | Gerald S. | Falchook | Journal of Cancer Research and Clinical Oncology | 2013-06-00 Jun 2013 | PURPOSE: Tumor biopsies are critical for delineating pharmacodynamic effects of drugs and for optimal patient selection during oncology clinical trials of molecular targeted therapies. The purpose of this study was to identify factors related to patients' willingness to provide study-related tumor biopsies in phase 1 clinical trials of molecularly targeted therapy. METHODS: An investigator-designed survey, that assessed biopsy willingness, demographic and clinical factors, was completed anonymously by patients with advanced cancer in a phase 1 clinic for targeted therapy. Data were analyzed using multivariate logistic regression models with odds ratios (OR) and 95 % confidence intervals (CI). RESULTS: Three hundred and sixty-two patients with advanced cancer (50 % male, 56 % aged ≤ 60 years) participated. In univariate analyses, willingness to provide study-related biopsy was associated with male gender, white race, higher income, using the Internet for cancer-related information, and having had a biopsy previously (p < 0.05). In multivariate analyses, male gender (OR 2.41, 95 % CI 1.54, 3.78) and having had a biopsy (OR 3.71, 95 % CI 1.68, 8.15) were associated with willingness to have one biopsy; male gender (OR 1.97, 95 % CI 1.30, 3.00) and relying on the Internet as a source of information (OR 1.87, 95 % CI 1.21, 2.89) were associated with willingness to have more than one biopsy. CONCLUSIONS: The results suggest that male gender is associated with greater stated willingness to undergo biopsy. Also, the Internet is an important source of information for patients with cancer and may strongly influence their decisions about whether to consent to biopsies in early clinical trials. | Male | Humans | Patient Participation | Questionnaires | OK |
| José Francisco | García-Gutiérrez | Jaime | Jiménez-Pernett | Fernando | Vázquez-Alonso | Supportive Care in Cancer: Official Journal of the Multinational Association of Supportive Care in Cancer | 2012-12-00 Dec 2012 | AIMS: The aims of this study were to describe the profile of urological cancer patients who look for health information on the Internet and to analyse the factors related to use of the Internet as a source of health information. METHODOLOGY: A cross-sectional descriptive study using individual, semi-structured, questionnaire-based interviews was carried out in oncology clinics in a hospital in Granada (Spain) in a sample group of 169 patients with prostate, bladder and kidney cancer. The dependent variable was use of the Internet as a source of health information. The independent variables were sociodemographic variables, health status, relationship with healthcare services, patient's role in decision-making process, satisfaction with healthcare, Internet use, Internet skills and attitude. Data analyses include descriptive, bivariate and multivariate analyses. RESULTS: Of the patients in the sample group, 72.2 % had prostate cancer, 19.4 % had bladder cancer and 8.3 % had kidney cancer. Only 11.2 % of patients in the group used the Internet as a source of health information. These patients were typically men of an average age of 62 years, who live in urban areas, who have completed secondary or university education, with a high income and who usually share the role of decision maker with their doctor. Patients who use the Internet as a source of health information usually look for support from psychological support groups, have family members who also look for information on the Internet and prefer sources of information other than those provided by the health services. CONCLUSIONS: The study outlines the profile of urological cancer patients who use the Internet as a source of health information. Internet use is related to a patient's attitude towards decision making, level of education and whether or not they look for information from sources other than the health system itself. | Male | Humans | Patient Participation | Patient Satisfaction | OK |
| Gary | Whittle | Janet | Robinson | Hilary | Whitehead | Primary dental care: journal of the Faculty of General Dental Practitioners (UK) | 2011-01-00 Jan 2011 | AIMS: To investigate the factors that dentists use to decide whether or not to make a patient edentulous. METHOD: A previous qualitative investigation identified factors that dentists would consider when making a patient edentulous. Using this information, a questionnaire was created and sent to all dentists practising in East Lancashire, asking them whether these factors would make them more or less likely to extract all remaining teeth for a patient. RESULTS: 123 usable questionnaires were returned from 187 dentists, a response rate of 67%. Dentists felt that clinical factors such as poor periodontal health and active decay were more likely to make them extract all remaining teeth. They were also concerned about retention and were keen to retain strategic teeth. Apart from their acknowledgment of the need to comply with the patient's wish to keep his or her teeth, the dentists had a neutral opinion of other factors such as poor health or their personal attitude to treatment. On average, respondents were rendering just over three patients per year edentulous. CONCLUSION: In this group of dentists, the key clinical factors that were considered when they decided whether or not to render a patient edentulous were periodontal disease, caries, and the attitude of the patient to tooth loss. Relatively few patients were rendered edentulous each year and if this pattern is common elsewhere in the United Kingdom, it may lead to a lack of skills within the dental workforce in managing patients' transition from dentate to edentulous. | Male | Decision Making | Humans | Patient Participation | OK |
| Karin D. | Hekkert | Karin | Hekkert | Marie-Christine | van Dongen | Health Policy (Amsterdam, Netherlands) | 2012-10-00 Oct 2012 | INTRODUCTION: To facilitate empowerment, the government encourages patient associations to participate in policy making discussions. To play a crucial role as one of the partners for the government in formulating policy on healthcare, information was needed about the activities and aims of Dutch patient associations. This article describes the development of the monitor in 2005 and 2006 and the most important outcomes and trends for 2007, 2008 and 2009. METHODS: Seven years ago, a yearly monitor of patient associations was started to quantify the activities of the patient and consumer movement in the Netherlands. We analyze individual Dutch patient associations focusing on empowerment and advocacy for their own members. RESULTS: Different types of associations pursue different goals to provide a 'voice' for their members. There was a very slight decline in individual members when comparing 2007 and 2009. More than a third of all associations have professional, paid employees. Organizations for disabled or mental disorders have the most volunteers. Peer support meetings for their own members remain the most popular activities. There are many small organizations and a few big ones. Advocacy remains important although the motives differ between patient associations. CONCLUSION: Dutch patient organizations reported activities they are expected to perform. They try to reduce information asymmetry by informing patients better through several media. They also provide peer support groups to their members. They reach the general public through their social media activities. Their primary focus is providing services to their members. | Humans | Patient Participation | Questionnaires | Health Care Reform | OK |
| Cécile | Barbaret | Anne | Chirac | Celine | Font-Truchet | Journal of Pain and Symptom Management | 2014-12-00 Dec 2014 | CONTEXT: Family conferences are conducted to assist with end-of-life discussions and discharge planning. OBJECTIVES: This study describes the current practices of family conferences in palliative care units (PCUs) in France. METHODS: A cross-sectional descriptive survey was sent to each PCU in France (n = 113). Members of the interdisciplinary health care team (palliative care physician, nurse, psychologist, and social worker) who were active in each PCU at the time of the survey were asked to respond. RESULTS: Two hundred seventy-six of 452 responses (61%) were obtained from members of the health care team in 91 units (81%). Two hundred seventy-two of 276 health care providers (HCPs) (99%) reported conducting family conferences in their clinical practice. Only 13 participants (5%) reported that they followed a structured protocol. Most respondents completed the questionnaire: palliative care physicians (n = 225; 82%), nurses (n = 219; 79%), and psychologists (n = 181; 66%). The three primary goals of family conferences were to allow family members to express their feelings (n = 240; 87%), identify family caregivers (n = 233; 84%), and discuss the patient's plan of care (n = 219; 79%). The primary reasons for conducting a family conference were: the patient's illness was terminal (n = 216; 78%), family caregivers requested a conference (n = 208; 75%), or terminal sedation was required (n = 189; 69%). One hundred six of 452 HCPs (38%) reported that patients were not invited to participate. The primary indications and goals for a family conference were significantly different among the four health care disciplines. CONCLUSION: Most HCPs in our study conducted family conferences. However, most of the family conferences had no structured protocol, half of the participants preferred no patient participation, and a significant variation was noted in the primary indications and goals among disciplines. | | | | | OK |
| Diana | Denboba | Bonnie | Strickland |  |  | Maternal and Child Health Journal | 2014-08-00 Aug 2014 | The Maternal and Child Health Bureau recently revised its measure of family-provider shared decision-making (SDM) to better align with parents' views and the intent of SDM. We sought to assess achievements in meeting the revised measure; examine socio-demographic/health correlates; and determine the relationships between SDM and access to quality health care. We analyzed data for 40,242 children with special health care needs (CSHCN) from the 2009-2010 National Survey of CSHCN and assessed the prevalence of SDM and association with other US CSHCN socio-demographic/health characteristics using bivariate and multivariate methods. Logistic regression was used to determine associations between SDM and having a medical home and preventive medical/dental visits. Approximately 70% of families of CSHCN perceived themselves as shared decision-makers in their child's care. Families of CSHCN with greater functional limitations had twice the odds of lacking SDM than those never affected. Disparities in attainment rates were noted for families with low versus high income (61 vs. 77%), less versus more than high school education (59 vs. 73%), privately insured versus uninsured (76 vs. 57%), and minority versus white race (63 vs. 74%). CSHCN with medical homes had 6 times greater odds of perceived SDM and as much as one and a half times the odds of receiving preventive care than CSHCN without a medical home. Major differences in family SDM perceptions are associated with having a medical home, particularly when characterized by family-centered care. Populations of concern are those with more functionally limited children and increased socio-economic challenges. | | | | | Doublon |
| Diana | Denboba | Bonnie | Strickland |  |  | Maternal and Child Health Journal | 2014-08-00 Aug 2014 | The Maternal and Child Health Bureau recently revised its measure of family-provider shared decision-making (SDM) to better align with parents' views and the intent of SDM. We sought to assess achievements in meeting the revised measure; examine socio-demographic/health correlates; and determine the relationships between SDM and access to quality health care. We analyzed data for 40,242 children with special health care needs (CSHCN) from the 2009-2010 National Survey of CSHCN and assessed the prevalence of SDM and association with other US CSHCN socio-demographic/health characteristics using bivariate and multivariate methods. Logistic regression was used to determine associations between SDM and having a medical home and preventive medical/dental visits. Approximately 70% of families of CSHCN perceived themselves as shared decision-makers in their child's care. Families of CSHCN with greater functional limitations had twice the odds of lacking SDM than those never affected. Disparities in attainment rates were noted for families with low versus high income (61 vs. 77%), less versus more than high school education (59 vs. 73%), privately insured versus uninsured (76 vs. 57%), and minority versus white race (63 vs. 74%). CSHCN with medical homes had 6 times greater odds of perceived SDM and as much as one and a half times the odds of receiving preventive care than CSHCN without a medical home. Major differences in family SDM perceptions are associated with having a medical home, particularly when characterized by family-centered care. Populations of concern are those with more functionally limited children and increased socio-economic challenges. | | | | | OK |
| Diana | Denboba | Bonnie | Strickland |  |  | Maternal and Child Health Journal | 2014-08-00 août 2014 | The Maternal and Child Health Bureau recently revised its measure of family-provider shared decision-making (SDM) to better align with parents’ views and the intent of SDM. We sought to assess achievements in meeting the revised measure; examine socio-demographic/health correlates; and determine the relationships between SDM and access to quality health care. We analyzed data for 40,242 children with special health care needs (CSHCN) from the 2009–2010 National Survey of CSHCN and assessed the prevalence of SDM and association with other US CSHCN socio-demographic/health characteristics using bivariate and multivariate methods. Logistic regression was used to determine associations between SDM and having a medical home and preventive medical/dental visits. Approximately 70 % of families of CSHCN perceived themselves as shared decision-makers in their child’s care. Families of CSHCN with greater functional limitations had twice the odds of lacking SDM than those never affected. Disparities in attainment rates were noted for families with low versus high income (61 vs. 77 %), less versus more than high school education (59 vs. 73 %), privately insured versus uninsured (76 vs. 57 %), and minority versus white race (63 vs. 74 %). CSHCN with medical homes had 6 times greater odds of perceived SDM and as much as one and a half times the odds of receiving preventive care than CSHCN without a medical home. Major differences in family SDM perceptions are associated with having a medical home, particularly when characterized by family-centered care. Populations of concern are those with more functionally limited children and increased socio-economic challenges. (PsycINFO Database Record (c) 2014 APA, all rights reserved). (journal abstract) | Decision Making | Health Care Services | Children with special health care needs | Family-centered care | Doublon |
| Vera | Nierkens | Mary | Nicolaou | Barend J. C. | Middelkoop | BMC public health | 2012-00-00 2012 | BACKGROUND: South Asian migrants are at particularly high risk of type 2 diabetes. Previous studies have shown that intensive lifestyle interventions may prevent the onset of diabetes. Such interventions have not been culturally adapted and evaluated among South Asians in industrialized countries. Therefore, we have set up a randomized controlled trial to study the effectiveness of a targeted lifestyle intervention for the risk of type 2 diabetes and cardiovascular risk factors among 18 to 60-year-old Hindustani Surinamese (South Asians) in The Hague, the Netherlands. Here we present the study design and describe the characteristics of those recruited. METHODS: Between May 18, 2009 and October 11, 2010, we screened 2307 Hindustani Surinamese (18-60 years old) living in The Hague. We sent invitations to participate to those who had an impaired fasting glucose of 5.6-6.9 mmol/l, an impaired glucose tolerance of 7.8-11.0 mmol/L, a glycated hemoglobin level of 6.0% or more and/or a value of 2.39 or more for the homeostasis model assessment of estimated insulin resistance. In total, 536 people (56.1% of those eligible) participated. People with a higher level of education and a family history of type 2 diabetes were more likely to participate. The control and intervention groups were similar with regard to important background characteristics. The intervention group will receive a culturally targeted intervention consisting of dietary counseling using motivational interviewing and a supervised physical activity program. The control group will receive generic lifestyle advice. To determine the effectiveness, a physical examination (anthropometrics, cardiorespiratory test, lipid profile, and measures of oral glucose tolerance, glycated hemoglobin, and insulin) and interview (physical activity, diet, quality of life, and intermediate outcomes) were carried out at baseline and will be repeated at 1 year and 2 years. The process and the costs will be evaluated. DISCUSSION: This trial will provide insight into the feasibility and effectiveness of a targeted, intensive, lifestyle intervention for the risk of type 2 diabetes and cardiovascular risk factors among 18 to 60-year-old South Asians. TRIAL REGISTRATION: Dutch Trial Register: NTR1499. | Male | Humans | Patient Participation | Questionnaires | OK |
| Antonio C. | Wolff | Michael A. | Carducci | Joseph M. | Herman | Psycho-Oncology | 2013-04-00 Apr 2013 | BACKGROUND: The PatientViewpoint website collects patient-reported outcomes and links them with the electronic health record to aid patient management. This pilot test evaluated PatientViewpoint's use, usefulness, and acceptability to patients and clinicians. METHODS: This was a single-arm prospective study that enrolled breast and prostate cancer patients undergoing treatment and the clinicians who managed them. Patients completed patient-reported outcomes every 2 weeks, and clinicians could access the results for patient visits. Scores that were poor relative to norms or substantially worse than the previous assessment were highlighted. After three on-study visits, we assessed patient and clinician perspectives on PatientViewpoint using close-ended and open-ended questions. RESULTS: Eleven out of 12 eligible clinicians (92%) and 52/76 eligible patients (68%) enrolled. Patients completed a median of 71% of assigned questionnaires; clinicians reported using the information for 79% of patients, most commonly as a source of additional information (51%). At the median, score reports identified three potential issues, of which 1 was discussed during the visit. Patients reported the system was easy to use (92%), useful (70%), aided recall of symptoms/side effects (72%), helped them feel more in control of their care (60%), improved discussions with their provider (49%), and improved care quality (39%). Patients and clinicians desired more information on score interpretation and minor adjustments to site navigation. CONCLUSIONS: These results support the feasibility and value of PatientViewpoint. An ongoing study is using a continuous quality improvement approach to further refine PatientViewpoint. Future studies will evaluate its impact on patient care and outcomes. | Male | Humans | Patient Participation | Patient Satisfaction | OK |
| Jose M. L. | Knijnenburg | Jako S. | Burgers | Rosella P. M. G. | Hermens | Journal of Medical Internet Research | 2012-09-00 septembre 2012 | Background: Patient participation is essential in developing high-quality guidelines but faces practical challenges. Evidence on timing, methods, evaluations, and outcomes of methodologies for patient participation in guideline development is lacking. Objective: To assess the feasibility of a wiki as a participatory tool for patients in the development of a guideline on infertility determined by (1) use of the wiki (number of page views and visitors), (2) benefits of the wiki (ie, number, content, and eligibility of the recommendations to be integrated into the guideline), and (3) patients’ facilitators of and barriers to adoption, and the potential challenges to be overcome in improving this wiki. Methods: To obtain initial content for the wiki, we conducted in-depth interviews (n = 12) with infertile patients. Transcripts from the interviews were translated into 90 draft recommendations. These were presented on a wiki. Over 7 months, infertile patients were invited through advertisements or mailings to formulate new or modify existing recommendations. After modifying the recommendations, we asked patients to select their top 5 or top 3 recommendations for each of 5 sections on fertility care. Finally, the guideline development group assessed the eligibility of the final set of recommendations within the scope of the guideline. We used a multimethod evaluation strategy to assess the feasibility of the wiki as a participatory tool for patients in guideline development. Results: The wiki attracted 298 unique visitors, yielding 289 recommendations. We assessed the 21 recommendations ranked as the top 5 or top 3 for their eligibility for being integrated into the clinical practice guideline. The evaluation identified some challenges needed to be met to improve the wiki tool, concerning its ease of use, website content and layout, and characteristics of the wiki tool. Conclusions: The wiki is a promising and feasible participatory tool for patients in guideline development. A modified version of this tool including new modalities (eg, automatically limiting the number and length of recommendations, using a fixed format for recommendations, including a motivation page, and adding a continuous prioritization system) should be developed and evaluated in a patient-centered design. (PsycINFO Database Record (c) 2013 APA, all rights reserved). (journal abstract) | Client Participation | Internet | wiki feasibility | participatory tool | Doublon |
| Jose M. L. | Knijnenburg | Jako S. | Burgers | Rosella P. M. G. | Hermens | Journal of Medical Internet Research | 2012-00-00 2012 | BACKGROUND: Patient participation is essential in developing high-quality guidelines but faces practical challenges. Evidence on timing, methods, evaluations, and outcomes of methodologies for patient participation in guideline development is lacking. OBJECTIVE: To assess the feasibility of a wiki as a participatory tool for patients in the development of a guideline on infertility determined by (1) use of the wiki (number of page views and visitors), (2) benefits of the wiki (ie, number, content, and eligibility of the recommendations to be integrated into the guideline), and (3) patients' facilitators of and barriers to adoption, and the potential challenges to be overcome in improving this wiki. METHODS: To obtain initial content for the wiki, we conducted in-depth interviews (n = 12) with infertile patients. Transcripts from the interviews were translated into 90 draft recommendations. These were presented on a wiki. Over 7 months, infertile patients were invited through advertisements or mailings to formulate new or modify existing recommendations. After modifying the recommendations, we asked patients to select their top 5 or top 3 recommendations for each of 5 sections on fertility care. Finally, the guideline development group assessed the eligibility of the final set of recommendations within the scope of the guideline. We used a multimethod evaluation strategy to assess the feasibility of the wiki as a participatory tool for patients in guideline development. RESULTS: The wiki attracted 298 unique visitors, yielding 289 recommendations. We assessed the 21 recommendations ranked as the top 5 or top 3 for their eligibility for being integrated into the clinical practice guideline. The evaluation identified some challenges needed to be met to improve the wiki tool, concerning its ease of use, website content and layout, and characteristics of the wiki tool. CONCLUSIONS: The wiki is a promising and feasible participatory tool for patients in guideline development. A modified version of this tool including new modalities (eg, automatically limiting the number and length of recommendations, using a fixed format for recommendations, including a motivation page, and adding a continuous prioritization system) should be developed and evaluated in a patient-centered design. | Male | Humans | Patient Participation | Female | OK |
| Gerbert J. | Renzenbrink | Govert J. | Snoek | Evelien | Dijkstra | Journal of Telemedicine and Telecare | 2010-00-00 2010 | Decision aids (DAs) may be helpful in improving patients' participation in medical decision-making. We investigated the potential for web-based DAs in a rehabilitation population. Two self-administered DAs focused on the treatment of acquired ankle-foot impairment in stroke and the treatment of arm-hand function in cervical spinal cord injury (SCI). Data collection comprised a telephone interview and a self-reported paper questionnaire. Of the patients who agreed to participate, 39 stroke patients (44%) and 38 patients with SCI (78%) returned a questionnaire. More than 75% of patients expressed a need for more information about the treatment of disease-related impairment. The DAs were highly appreciated by both patient groups. Nearly all patients expressed a positive attitude towards the use of the web-based DAs in general practice. The DAs had a positive effect on the knowledge about the treatment alternatives in the stroke patients (P = 0.001), although not in the patients with SCI. The DAs reduced patients' conflict about treatment (P < 0.05). The effect of the DAs on patients' desired role in decision-making was limited. Web-based aids are feasible in the rehabilitation population with access to a computer and can improve the knowledge gaps in patients. | Male | Decision Making | Decision Support Techniques | Humans | OK |
| Karen E. | Thomas | Eleanor K. L. | Mitchell |  |  | Journal of Cardiopulmonary Rehabilitation and Prevention | 2014-02-00 2014 Jan-Feb | PURPOSE: In 2010, the Healthy Heart (HH) community-based cardiac rehabilitation program was offered at Latrobe Community Health Service in rural Victoria, Australia. The 8-week program, based on National Heart Foundation guidelines, consisted of exercise sessions; health education on diet, stress, and smoking cessation; and behavioral change strategies. Participants were also informed about local community exercise opportunities. A program evaluation was conducted in 2011 to assess whether the content of the program was meeting the needs of participants and to identify what suggestions they had for improvement. METHODS: Eighteen patients had completed the HH program in 2010. Eight of these participants, 7 men and 1 woman, volunteered to take part in a focus group. Conventional content analysis was used to identify and group the common themes that emerged from the focus group discussions. RESULTS: Three themes were identified that reflected the participant experiences of attending the HH program. The first, "recovering confidence," described participant responses to the content of the sessions. The second, "putting it into practice," referred to their comments about taking responsibility for making lifestyle changes. The third, "feeling abandoned," emerged from the reported difficulty participants expressed about maintaining motivation for change after program completion. CONCLUSION: Participants rated the HH program as very successful by objective measures. However, they reported struggling to maintain self-management strategies postprogram. There is clearly a need to develop strategies that support cardiac rehabilitation participants over the longer-term. | Male | Emotions | Humans | Patient Participation | Doublon |
| Karen E. | Thomas | Eleanor K. L. | Mitchell |  |  | Journal of Cardiopulmonary Rehabilitation and Prevention | 2014-02-00 2014 Jan-Feb | PURPOSE: In 2010, the Healthy Heart (HH) community-based cardiac rehabilitation program was offered at Latrobe Community Health Service in rural Victoria, Australia. The 8-week program, based on National Heart Foundation guidelines, consisted of exercise sessions; health education on diet, stress, and smoking cessation; and behavioral change strategies. Participants were also informed about local community exercise opportunities. A program evaluation was conducted in 2011 to assess whether the content of the program was meeting the needs of participants and to identify what suggestions they had for improvement. METHODS: Eighteen patients had completed the HH program in 2010. Eight of these participants, 7 men and 1 woman, volunteered to take part in a focus group. Conventional content analysis was used to identify and group the common themes that emerged from the focus group discussions. RESULTS: Three themes were identified that reflected the participant experiences of attending the HH program. The first, "recovering confidence," described participant responses to the content of the sessions. The second, "putting it into practice," referred to their comments about taking responsibility for making lifestyle changes. The third, "feeling abandoned," emerged from the reported difficulty participants expressed about maintaining motivation for change after program completion. CONCLUSION: Participants rated the HH program as very successful by objective measures. However, they reported struggling to maintain self-management strategies postprogram. There is clearly a need to develop strategies that support cardiac rehabilitation participants over the longer-term. | Male | Emotions | Humans | Patient Participation | OK |
| G. J. | Aanerud | A. | Balanescu | D. T. | Boumpas | Annals of the Rheumatic Diseases | 2011-06-00 Jun 2011 | OBJECTIVE: A patient-derived composite measure of the impact of rheumatoid arthritis (RA), the rheumatoid arthritis impact of disease (RAID) score, takes into account pain, functional capacity, fatigue, physical and emotional wellbeing, quality of sleep and coping. The objectives were to finalise the RAID and examine its psychometric properties. METHODS: An international multicentre cross-sectional and longitudinal study of consecutive RA patients from 12 European countries was conducted to examine the psychometric properties of the different combinations of instruments that might be included within the RAID combinations scale (numeric rating scales (NRS) or various questionnaires). Construct validity was assessed cross-sectionally by Spearman correlation, reliability by intraclass correlation coefficient (ICC) in 50 stable patients, and sensitivity to change by standardised response means (SRM) in 88 patients whose treatment was intensified. RESULTS: 570 patients (79% women, mean ± SD age 56 ± 13 years, disease duration 12.5 ± 10.3 years, disease activity score (DAS28) 4.1 ± 1.6) participated in the validation study. NRS questions performed as well as longer combinations of questionnaires: the final RAID score is composed of seven NRS questions. The final RAID correlated strongly with patient global (R=0.76) and significantly also with other outcomes (DAS28 R=0.69, short form 36 physical -0.59 and mental -0.55, p<0.0001 for all). Reliability was high (ICC 0.90; 95% CI 0.84 to 0.94) and sensitivity to change was good (SRM 0.98 (0.96 to 1.00) compared with DAS28 SRM 1.06 (1.01 to 1.11)). CONCLUSION: The RAID score is a patient-derived composite score assessing the seven most important domains of impact of RA. This score is now validated; sensitivity to change should be further examined in larger studies. | Male | Humans | Patient Participation | Female | OK |
| R. P. | van der Heijden | K. M. | van Asselt | W. A. | Bemelman | European Journal of Surgical Oncology: The Journal of the European Society of Surgical Oncology and the British Association of Surgical Oncology | 2013-08-00 Aug 2013 | INTRODUCTION: Follow-up to detect recurrence is an important feature of care after colon cancer treatment. Currently, follow-up visits are surgeon-led with focus on recurrence. To date, there is increasing interest for general practitioners (GPs) providing this care, as GPs might provide more holistic care. The present study assessed how surgeons, GPs, and patients evaluate current surgeon-led colon cancer follow-up and to list their views on possible future GP-led follow-up. METHODS: The study consists of a cross-sectional survey including colorectal surgeons, patients who participate or recently finished a follow-up programme, and GPs in the Netherlands. RESULTS: Eighty-seven out of 191 GPs, 113 out of 238 surgeons, and 186 out of 243 patients responded. Patients are satisfied about current surgeon-led follow-up, especially about recurrence detection and identification of physical problems (94% and 85% respectively). However, only 56% and 49% of the patients were satisfied about the identification of psychological and social problems respectively. Only 16% of the patients evaluated future GP-led follow-up positively. Regarding healthcare providers, surgeons were more positive compared to GPs; 49% of the surgeons, and only 30% of the GPs evaluated future GP-led follow-up positively (P = 0.002). Furthermore, several reservations and principle requirements for GP-led follow-up were identified. DISCUSSION: The results suggest an unfavourable view among patients and healthcare providers, especially GPs, regarding a central role for GPs in colon cancer follow-up. However, low satisfaction on psychosocial aspects in current follow-up points out a lack in care. Therefore, the results provide a justification to explore future GP-led care further. | Time Factors | Male | Humans | Patient Participation | OK |
| Chiungfang | Chang | Hyunsoo | Hong | Izumi | Kobayashi | Journal of Human Genetics | 2011-05-00 May 2011 | This paper introduces methods used to communicate with participants in the 'Biobank Japan Project (BBJP)', which is a disease-focused biobanking project. The methods and their implications are discussed in the context of the ethical conduct of the biobanking project. Informed consent, which ensures the autonomous decisions of participants, is believed to be practically impossible for the biobanking project in general. Consequently, the concept of 'trust', which is 'judgement and action in conditions of less than perfect information', has been suggested to compensate for this limitation. As a means to maintain the trust participants feel for the project, this paper proposes communication with participants after receiving their consent. After describing the limitations of informed consent within the BBJP, based on a survey we conducted, we introduce our attempts to communicate with participants, discussing their implications as a means to compensate for the limitations of informed consent at the biobanking project. | Male | Humans | Patient Participation | Questionnaires | OK |
|  |  |  |  |  |  | The Journal of Ambulatory Care Management | 2010-09-00 2010 Jul-Sep | The Laboratory for Medical Research and Consumer Involvement was established in 2005 at Mario Negri Institute, a nonprofit institute for pharmacological research, as a consequence of the increasing interest in boosting citizens' and patients' involvement in the health care debate. It has developed several projects with patients' associations, researchers, and clinicians. Its objectives are to foster a strategic alliance among health care professionals, patients, and their organizations, developing activities with different levels of involvement. Among the laboratory' s activities, the PartecipaSalute project has organized training courses for consumers, published a Web site disseminating evidence-based information and critical appraisal tools, and collected research priorities set by patients. Two consensus conferences have been organized, one dealing with brain injury patients' assistance and the other with hormone therapy and menopause. The quality of health information covered by different sources (press articles, Web sites, and brochures) has also been assessed. Seventy consumers attended the training courses from 2006 to 2008, and between January 2008 and June 2009 the PartecipaSalute Web site registered a mean of 30 500 single visits monthly. At the consensus conference Informing women on hormone replacement therapy, 7 members of the 14-member panel defining the final recommendations were lay people. Other data from the laboratory's main activities are given in this article. The criteria for selecting patients and their organizations, the methods of involvement, and evaluation of the impact of the activities are still open questions. We are now developing ways of evaluating our activities, and trying to boost citizens' and patients' participation in decisional settings, concerning health care assistance and research studies. | Male | Humans | Patient Participation | Questionnaires | OK |
| Marie-José | Durand | Marc | Corbière | Dawn | Stacey | Implementation science: IS | 2011-00-00 2011 | BACKGROUND: From many empirical and theoretical points of view, the implementation of shared decision making (SDM) in work rehabilitation for pain due to a musculoskeletal disorder (MSD) is justified but typically the SDM model applies to a one on one encounter between a healthcare provider and a patient and not to an interdisciplinary team. OBJECTIVES: To adapt and implement an SDM program adapted to the realities of work rehabilitation for pain associated with a MSD. More specific objectives are to adapt an SDM program applicable to existing rehabilitation programs, and to evaluate the extent of implementation of the SDM program in four rehabilitation centres. METHOD: For objective one, we will use a mixed perspective combining a theory-based development program/intervention and a user-based perspective. The users are the occupational therapists (OTs) and clinical coordinators. The strategies for developing an SDM program will include consulting the scientific literature and group consensus with clinicians-experts. A sample of convenience of eight OTs, four clinical coordinators and four psychologists all of whom have been working full-time in MSD rehabilitation for more than two years will be recruited from four collaborating rehabilitation centres. For objective two, using the same criteria as for objective one, we will first train eight OTs in SDM. Second, using a descriptive design, the extent to which the SDM program has been implemented will be assessed through observations of the SDM process. The observation data will be triangulated with the dyadic working alliance questionnaire, and findings from a final individual interview with each OT. A total of five patients per trained OT will be recruited, for a total of 40 patients. Patients will be eligible if they have a work-related disability for more than 12 weeks due to musculoskeletal pain and plan to start their work rehabilitation programs. DISCUSSION: This study will be the first evaluation of the program and it is expected that improvements will be made prior to a broader-scale implementation. The ultimate aim is to improve the quality of decision making, patients' quality of life, and reduce the duration of their work-related disability by improving the services offered during the rehabilitation process. | Male | Decision Making | Humans | Questionnaires | OK |
| Margaret I. | Fitch |  |  |  |  | Journal of Cancer Education: The Official Journal of the American Association for Cancer Education | 2013-06-00 Jun 2013 | Participatory health research involves a wide spectrum of participation from the population of study. We describe the participatory research processes of a large mixed method study on the psychosocial impact of dragon boating in individuals with breast cancer. In particular, we discuss the involvement of a Community Advisory Group (consisting of five breast cancer patients/survivors) in the development of the research study, data collection and analysis, and dissemination of the study results. We also outline the elements of a research workshop, in which 13 breast cancer patients/survivors were involved in the development of a provincial survey for the study. The purpose of this article is to share our experience of engaging cancer patients/survivors in a participatory research study. We discuss the value-based elements of participatory research (power sharing, voice and respect, reciprocity, and mutual benefit), and provide a case-based example of how these participatory elements were employed to potentially increase the validity of the survey instrument, to enhance the ethics of working with a cancer population, and ultimately contributed to a high survey response rate. | Humans | Patient Participation | Patient Satisfaction | Questionnaires | OK |
|  |  |  |  |  |  | Bulletin Du Cancer | 2013-03-00 Mar 2013 | | Decision Making | Humans | Patient Participation | Patient Satisfaction | OK |
| Steven J. | Pearlman |  |  |  |  | Archives of Facial Plastic Surgery | 2010-10-00 2010 Sep-Oct | OBJECTIVES: To assess the subjective aesthetic and functional concerns of patients seeking revision rhinoplasty and to compare them with objective deformities found on evaluation by the surgeon. METHODS: This prospective study used a questionnaire to systematically target the aesthetic and functional concerns of 104 consecutive patients seeking revision rhinoplasty. Analysis of the subjective data revealed the frequency of each concern, which was then compared with objective deformities found on evaluation by the surgeon. RESULTS: The most common patient and surgeon aesthetic concerns were (1) tip asymmetry, (2) crooked middle third of the nose, and (3) upper third irregularity. A mean of 79% of patient concerns were also reported by the surgeon. Of the 64 patients describing subjective nasal obstruction, 60 (94%) had objective physical findings related to obstruction. The 3 most troublesome patient concerns were (1) tip asymmetry, (2) difficulty breathing or nasal blockage, and (3) crooked middle third of the nose. CONCLUSIONS: Tip asymmetry was the most common deformity noted. Symptomatic nasal obstruction was the second most frequent reason for seeking revision rhinoplasty. Differences in patient and surgeon findings were largely due to differences in assessment skills and should be addressed by thorough explanation of nasal aesthetics. | Male | Humans | Patient Participation | Patient Satisfaction | OK |
| Elizabeth J. | Campagna | L. Miriam | Dickinson | Jennifer | Bruny | Academic Pediatrics | 2012-12-00 2012 Nov-Dec | OBJECTIVE: Almost one-half of all pediatric gastrostomy tube insertions are accompanied by a fundoplication, yet little is understood about the surgical decision-making for these procedures. The objective of this study was to examine the decision-making process of surgeons about whether to perform a fundoplication in children already scheduled to have a gastrostomy tube placed. METHODS: A written questionnaire of all pediatric surgeons at a major children's hospital was completed for each planned gastrostomy procedure over the course of 1 year; the questionnaire asked about various influences on the fundoplication decision: primary care and subspecialty physicians' opinions, patient characteristics, and parent opinions. Patient demographics and clinical characteristics from the medical record, as well as questionnaire responses, were summarized for each gastrostomy occurrence. We modeled the association of questionnaire responses and patient characteristics with the outcome of having a fundoplication. RESULTS: We received questionnaires on 161 of 169 eligible patients (95%). A total of 52% of patients had fundoplication. Primary care physicians were involved in 44% of decisions, and when involved had "a lot" of influence on the fundoplication decision only 28% of time, compared with neonatologists (61%), hospitalists (44%), pediatric pulmonologists (42%), and pediatric gastroenterologists (40%). A total of 86% of patients had a subspecialist involved, and 28% had >1 subspecialist. A pH probe was performed in 7.5% of cases, and failed pharmacotherapy was noted by the surgeons in only 26.5% of the fundoplications performed. CONCLUSIONS: The decision to do a fundoplication was rarely based on definitive testing or failed medical treatment. From the surgeon's perspective, subspecialists were more influential than primary care physicians, which is at odds with current concepts of the medical home. | Male | Decision Making | Humans | Patient Participation | OK |
| Benedict C. | Nwomeh |  |  |  |  | Patient Education and Counseling | 2012-10-00 Oct 2012 | OBJECTIVE: Previous research in primary care has shown that physicians are more informative and/or patient-centered when they interact with high participation patients (e.g., those who ask questions, express preferences and concerns). This study contributes to this literature by examining parents' participation and physicians' discourse in a pediatric surgery context. METHODS: The audiotapes of 7 pediatric surgeons and 68 of their patients' parents/guardians were coded and examined for physicians' information exchange and support utterances as they interacted with parents/guardians with varying degrees of participation. RESULTS: The results of a multilevel regression analysis showed, consistent with related research, that the same physicians were more informative overall and provided more information in response to parents' questions when interacting with high participation parents. However, participation was not associated with physicians' volunteered information, general explanations, or support utterances. DISCUSSION AND CONCLUSION: The results are discussed with respect to the impact of participation on physicians' information provision and implications for future research, particularly with respect to general explanations, volunteered information, and medical context. PRACTICE IMPLICATIONS: This study suggests that surgeons may provide more detailed information to patients who are active participants in the preoperative interview, thereby improving the adequacy and validity of informed consent for surgical procedures. | Male | Humans | Patient Participation | Physician-Patient Relations | OK |
|  |  |  |  |  |  | South Dakota Medicine: The Journal of the South Dakota State Medical Association | 2014-01-00 Jan 2014 | | Humans | Patient Participation | United States | Quality Assurance, Health Care | OK |
|  |  |  |  |  |  | Healthcare Informatics: The Business Magazine for Information and Communication Systems | 2010-08-00 Aug 2010 | As text messaging becomes more established in everyday routines by millions of users, healthcare organizations are embracing the technology as a powerful tool to allow patients to better manage their health. Two health provider networks, Kaiser Permanente and Riverview Health System, have launched successful pilots and plan to build on those initiatives. | Humans | Patient Participation | United States | communication | OK |
| Erik | Taal | Mart A. F. J. | van de Laar |  |  | Rheumatology International | 2013-09-00 Sep 2013 | Technology enables patients home access to their electronic medical record (EMR), via a patient portal. This study aims to analyse (dis)advantages, preconditions and suitable content for this service, according to rheumatology health professionals. A two-phase policy Delphi study was conducted. First, interviews were performed with nurses/nurse practitioners (n = 9) and rheumatologists (n = 13). Subsequently, collected responses were quantified, using a questionnaire among the interviewees. The following advantages of patient home access to the EMR were reported: (1) enhancement of patient participation in treatment, (2) increased knowledge and self-management, (3) improved patient-provider interaction, (4) increased patient safety, and (5) better communication with others. Foreseen disadvantages of the service included: (1) problems with interpretation of data, (2) extra workload, (3) a change in consultation content, and (4) disturbing the patient-provider interaction. Also, the following preconditions emerged from the data: (1) optimal security, (2) no extra record, but a patient-accessible section, (3) no access to clinical notes, and (4) a lag time on the release of lab data. Most respondents reported that data on diagnosis, medication, treatment plan and consultations could be released to patients. On releasing more complex data, such as bodily examinations, lab results and radiological images the opinions differed considerably. Providing patients home access to their medical record might be a valuable next step into patient empowerment and in service towards the patient, provided that security is optimal and content and presentation of data are carefully considered. | Male | Humans | Patient Participation | Questionnaires | OK |
| Alan L. | Robin | Amy | Hennessy | Elizabeth D. | Sharpe | Ophthalmology | 2010-02-00 Feb 2010 | | Male | Humans | Patient Participation | Questionnaires | OK |
| F. | Pauly | S. | Godon | M. | Chevignard | Annals of Physical and Rehabilitation Medicine | 2013-04-00 Apr 2013 | Goal Attainment Scaling (GAS) is a method for quantifying progress on personal goals. Turner-Stokes's guide to GAS is a method for quantifying progress towards personal goals. Turner-Stokes's guide and the use of Kiresuk's T-score are the most widely used GAS-based approaches in rehabilitation. However, the literature describes a number of other approaches and emphasizes the need for caution when using the T-score. This article presents the literature debates on GAS, variations of GAS (in terms of the score level assigned to the patient's initial status and description of the scale's different levels), the precautions to be taken to produce valid GAS scales and the various ways of analyzing GAS results. Our objective is to (i) provide clinical teams with a critical view of GAS (the application of which is not limited to a single research group's practices) and (ii) present the most useful resources and guidelines on writing GAS scales. According to the literature, it appears to be preferable to set the patient's initial level to -2 (even when worsening is a possible outcome) and to describe all five GAS levels in detail. The use of medians and rank tests appears to be appropriate, given the ordinal nature of GAS. | Humans | Patient Participation | Psychometrics | goals | OK |
| M. | Gustke | S. | Dibbelt | B. | Greitemann | Die Rehabilitation | 2011-10-00 Oct 2011 | OBJECTIVE: Goal setting is regarded as a key element in the rehabilitation process. Information about current goal setting practice is limited. However, there is potential for further improvement, e. g. as far as patient participation in the goal setting process is concerned. The study reports results of a survey on the current practice of goal setting in medical rehabilitation, focusing on development potential. The study examines acceptance and requirements for goal setting as well as patients' and professionals' experiences regarding goal setting during the rehabilitation process, with an emphasis on patient participation. METHODS: A total of n=40 rehabilitation professionals and n=210 inpatients with a diagnosis of chronic back pain, diabetes mellitus type 2 or coronary heart disease were surveyed at six rehabilitation centres using a questionnaire. Questionnaires contained predominantly closed-ended items regarding the current practice of goal setting. Several items were identical for both groups. In addition to the quantitative evaluation by means of frequency analyses free text data were evaluated. RESULTS: Rehabilitation professionals saw various benefits in goal setting with patients. Many professionals experienced patients to have personal goals for their rehabilitation, and most of them believed that patient participation in goal setting is possible. Also, barriers and requirements for goal setting were identified. A consistent concept for goal setting, involvement of and exchange between the different occupational groups and an elaborate handling of information in the context of goal setting seem to be realised at least partially. Professionals and patients reported similar frequencies of talks on goal setting at different times during the rehabilitation course. Patient participation seems to be realised to some extent but not comprehensively. The results show slight discrepancies between patients' and professionals' statements. DISCUSSION: Current practice of goal setting in inpatient rehabilitation of patients with the diagnoses named above is altogether on a relatively advanced level in the rehabilitation centres taking part in the study. The study identifies potential for development concerning a comprehensive implementation of goal setting, the realisation of patient participation, goal documentation and their integration into rehabilitation. Moreover, deeper involvement of the rehabilitation team and a stronger conceptual integration of the subject within the centres seem desirable. This can be taken up within the scope of interventions. | Male | Humans | Patient Participation | Questionnaires | OK |
| Zachary D. | Erekson | Lauris C. | Kaldjian |  |  | The American Journal of Hospice & Palliative Care | 2011-08-00 Aug 2011 | Our objective was to validate 6 literature-derived goals of care by analyzing open-ended and closed-ended responses about goals of care from a previous study of hospitalized patients. Eight clinicians categorized patients' open-ended articulations of their goals of care using a literature-derived framework and then compared those categorizations to patients' own closed-ended selections of their most important goal of care. Clinicians successfully categorized patients' open-ended responses using the literature-derived framework 83.5% of the time, and their categorizations matched patients' closed-ended most important goal of care 87.8% of the time. Goals that did not fit within the literature-derived framework all pertained to the goal of understanding a patient's diagnosis or prognosis; this seventh potential goal can be added to the literature-derived framework of 6 goals of care. | Humans | Patient Participation | Patient Satisfaction | Physician-Patient Relations | OK |
| Tinna | Hlidarsdottir | Mads Ronald | Dahl | Jan | Mainz | Danish Medical Journal | 2013-05-00 May 2013 | INTRODUCTION: The objective was to evaluate the long-term results of outpatient tonsillectomy in Danish ear, nose and throat (ENT) practices in terms of satisfaction, quality of life (QoL) and relief of symptoms. MATERIAL AND METHODS: A telephone interview was carried out at least three years after tonsillectomy. Alternatively, a questionnaire was filled in by the patients. The answers were compared to a pre-operative questionnaire. RESULTS: The response rate was 63% (386/614). No differences were found between the responders and the non-responders except that the proportion of smokers was higher in the non-responder group. QoL improved significantly and 93% were satisfied with having chosen tonsillectomy. 95% experienced complete or partial relief from their former throat-related symptoms. Eight to 11% had developed new symptoms from the throat including sleep-disordered breathing. CONCLUSION: With regard to QoL, satisfaction and relief of symptoms, the long-term results of outpatient tonsillectomy in Danish ENT practices are comparable to those obtained among hospitalized patients and they are in conformity with international standards. However, as approximately 10% develop new symptoms, it is important that physicians are aware of the indication as well as of any information received during shared decision-making with the patients/parents to adjust expectations. FUNDING: none. TRIAL REGISTRATION: none. | Time Factors | Male | Humans | Patient Satisfaction | OK |
| Vicky Veitch | Wolfe | Elizabeth | Phoenix | Sharon | Brisebois | Behavior Therapy | 2011-06-00 Jun 2011 | The current study examined the relationship between child involvement and treatment outcome in a group cognitive-behavioral treatment (GCBT) program for children with anxiety, using an adapted version of the Child Involvement Rating Scale (CIRS). Thirty-four children with a primary diagnosis of an anxiety disorder, referred for outpatient treatment at a hospital, and their parents participated in a manualized 12-session GCBT program. The Multidimensional Anxiety Scale for Children (MASC), administered at pretreatment, posttreatment, and at 4-month follow-up, served as the outcome measure. The psychometric properties of the adapted CIRS were good. Early involvement (EI) was calculated as the average score from Sessions 1 to 6 and late involvement (LI), the average score from Sessions 7 to 12. EI predicted lower MASC scores at posttreatment, after controlling for pretreatment MASC scores and for LI, B=-1.05, t(3)=-2.34, p<.05. EI did not predict outcomes at follow-up. Results were replicated using the original CIRS measure. Exploratory analyses suggested that the relationship between LI and treatment outcomes was moderated by medication. Involvement in therapy prior to exposures might be an important predictor of posttreatment outcomes in GCBT in a community setting. | Male | Humans | Patient Participation | Female | OK |
| Sandra | van Dijk | Diana C. | Grootendorst | Friedo W. | Dekker | Nephrology, Dialysis, Transplantation: Official Publication of the European Dialysis and Transplant Association - European Renal Association | 2014-07-00 Jul 2014 | BACKGROUND: In predialysis patients, the optimal treatment choices for controlling haemoglobin (Hb) are unknown, because targeting high Hb levels has negative effects--poorer survival--but possible positive effects as well--better health-related quality of life (HRQOL). Moreover, these effects may be different in specific subgroups (e.g. young versus elderly). METHODS: In the PREPARE-2 follow-up study, incident predialysis patients were included (2004-2011) when referred to 1 of the 25 participating Dutch outpatient clinics. HRQOL was assessed at 6-month intervals with the short form-36 (SF-36) questionnaire [physical/mental summary measure and eight subscales (range 0-100)]. A linear mixed model was used to associate Hb [<11, ≥ 11 to <12 (reference), ≥ 12 to <13 and ≥ 13 g/dL] with HRQOL, stratified by prescription of anaemia medication (erythropoietin-stimulating agent (ESA)/iron) and age (young: <65 years and elderly: ≥ 65 years). RESULTS: Only elderly patients (n = 214) not prescribed ESA/iron and with a high Hb (≥ 13 versus ≥ 11 to <12 g/dL) had a statistically significant (P < 0.05) and/or clinically relevant (>3-5 points) higher physical [11.9, 95% confidence interval (CI) 1.7, 22.2] and mental (6.4, 95% CI -1.7, 14.6) summary score. High Hb was not associated with a higher HRQOL in elderly patients who were prescribed ESA/iron. However, only young patients (n = 157) prescribed ESA/iron and with a high Hb (≥ 13 versus ≥ 11 to <12 g/dL) had a higher physical (8.9, 95% CI 2.1, 15.8) and mental (6.2, 95% CI -0.4, 12.8) summary score. CONCLUSIONS: The association of Hb levels with HRQOL differs by age and use of ESA/iron medication on predialysis care. Therefore, medical care should aim for shared decision-making regarding the appropriate Hb target leading to more individualized care. | Male | Humans | Questionnaires | Female | OK |
| Jui-Kuang | Chen | Hung-Chin | Tsai | Ching-Hsien | Li | American Journal of Infection Control | 2013-04-00 Apr 2013 | BACKGROUND: Patient participation has been proven to increase hand hygiene compliance of health care workers. The objective of the study is to better understand patients' attitudes and perceptions toward hand hygiene, and to identify patients with the highest motivation to participate in hand hygiene. DESIGN: A 2-week, cross-sectional survey of hospitalized patients and their family members was conducted using an anonymous, self-reporting questionnaire in a large teaching hospital in Taiwan. RESULTS: Of the 859 respondents, 89.8% considered hand hygiene important, and 75.9% would take hand hygiene practices into consideration when they choose a hospital. Most respondents (78.4%) would like more information on hand hygiene, particularly persons who have had experience with health care-associated infection (odds ratio, 2.48; 95% confidence interval, 1.57-3.89; P < .001). Respondents would be more willing to ask a doctor or nurse to wash his or her hands if they knew that the doctor or nurse would appreciate the reminder (doctor: from 48.9% to 74.6% [P < .001]; nurse: from 50.8% to 76.3% [P < .001]). CONCLUSIONS: Hand hygiene is considered important by most patients and family members and plays an influential role in their choice of a hospital or doctor. Persons with experience with health care-associated infections have the greatest motivation to participate in hand hygiene. | Male | Humans | Questionnaires | Female | OK |
|  |  |  |  |  |  | Social Science & Medicine (1982) | 2010-08-00 Aug 2010 | Patient safety is a central issue in healthcare. In the United Kingdom, where there is more accurate information on National Health Service (NHS) hospitals than on primary care or the private sector, the evidence on adverse incidents shows that avoidable medical harm is a major concern. This paper looks at the occurrence of medical harm and argues that in the construction of patient safety reforms, it is important to be aware of alternative narratives about issues of power and accountability from harmed patients and self-help groups, that challenge dominant perspectives on the issues. The paper draws upon evidence from two sources. First, the paper draws on experiences of self-help groups set up as a result of medical harm and part of a campaigning network, where evidence was gathered from 14 groups over more than 2 years. In addition, data were obtained from 21 individuals affected by harm that attended a residential workshop called the Break Through Programme; 18 questionnaires were completed from participants and a written narrative account of their experiences and observational data were gathered from a range of workshop sessions. Looking at the issues from harmed patients' perspectives, the research illustrates that a model of medical harm focussing predominantly upon the clinical markers and individual agency associated with a medical model operates to obscure a range of social processes. These social processes, connected to the power and dominance of the medical profession and the activities of a wider state, are seen to be a major part of the construction of harm that impacts upon patients, which is further compounded by its concealment. Understanding the experiences of harmed patients is therefore seen as an important way of generating knowledge about the medical and social processes involved in harm, that can lead to a broader framework for addressing patient safety. | Humans | Patient Participation | Physician-Patient Relations | Questionnaires | OK |
| Stefanie | Joos |  |  |  |  | Zeitschrift Für Evidenz, Fortbildung Und Qualität Im Gesundheitswesen | 2011-00-00 2011 | Patients with little knowledge of the German language have a special need for information. Due to language barriers they behave more passively in medical encounter, have a poorer knowledge about their disease and are generally less satisfied with their medical care. Foreign language patient information material could bridge the gap between medical consultation and the patients' self-responsibility and involve patients more actively in the treatment process. Based on extensive research in several areas (internet, clinic and practice leaflets, drug patient information leaflets) the present article illustrates that in all these areas foreign language patient information is very poorly available compared to German material. The reinforcement of the development of such material could lead to a higher involvement of immigrant patients' in the decision-making process, higher rates of patient safety and satisfaction and, overall, to a better quality of care for all patient groups in Germany. | Humans | Patient Participation | Internet | Hospitalization | OK |
|  |  |  |  |  |  | Journal of the American Pharmacists Association: JAPhA | 2014-10-00 2014 Sep-Oct | |  |  |  |  | OK |
| Diana | Castillo | Neena S. | Abraham |  |  | Patient Education and Counseling | 2011-12-00 Dec 2011 | OBJECTIVES: To evaluate the effect of functional health literacy (FHL) on preferences for decision-making; and among those initially preferring a passive decision-making role, to explore how preferences change if their physician actively encourages their involvement. METHODS: Consecutive older adults with cardiovascular disease receiving complex antithrombotic therapy completed a comprehensive assessment including measures of FHL and preferences for shared decision making. RESULTS: Half of all participants had inadequate or marginal FHL. Those with inadequate FHL were more likely (P=0.01) to prefer passive rather than active decision making styles even after controlling for age, education, and numeracy. However, 40% of patients preferring passive styles had adequate FHL and these patients were significantly more likely to change their preference to more active styles (odds ratio=7.17, P<.01) if their physician "was more supportive or encouraged participation". CONCLUSIONS: Screening FHL can provide insight into patients' preferences for active participation in decision making. Clinicians' encouragement of participation can increase engagement by patients with adequate FHL. PRACTICE IMPLICATIONS: We propose an algorithm for screening FHL and preferences for participating in decisions about complex medication regimens. | Male | Decision Making | Humans | Patient Participation | OK |
|  |  |  |  |  |  | Lancet | 2013-10-12 Oct 12, 2013 | In this Review, we discuss the UK's Health Technology Assessment programme, which is 20 years old in 2013. We situate the programme in the context of the UK landscape for evidence-based medicine, including in relation to the National Institute for Health and Care Excellence and as guidance to the National Health Service. We identify features that might be of value to other health systems as they confront the challenges of rapid innovation and rising costs. We use examples of recent studies to show the strengths and weaknesses of the programme. | Humans | Patient Participation | Mental health | Evidence-based medicine | OK |
| Máirín | Ryan | Michael | Barry |  |  | Expert Review of Pharmacoeconomics & Outcomes Research | 2010-10-00 Oct 2010 | A record number of delegates from industry, academia and health policy convened at the 7th Annual Meeting of Health Technology Assessment International (HTAi) which was held in Dublin, Ireland in June 2010. The theme of this year's meeting was 'Maximizing the Value of HTA'. The scientific program covered a broad range of topics from coverage with evidence development to timeliness of conducting HTAs, early engagement with stakeholders, value of information analysis, patient involvement and international collaboration. There was also a lively social program with the conference dinner held at the Guinness Storehouse. | Time Factors | Humans | Patient Participation | International Cooperation | OK |
| Mercedes | Guerra | Juan Antonio | Blasco | Elena | Andradas | International Journal of Technology Assessment in Health Care | 2011-10-00 Oct 2011 | OBJECTIVES: The aim of this study was to develop a breast cancer Patient Decision Aid (PDA), using a Health Technology Assessment (HTA) process, to assist patients in their choice of therapeutic options, and to promote shared decision making among patients, healthcare professionals, and other interested parties. METHODS: A systematic review (SR) was conducted of existing breast cancer patient Decision Aids encountered in the main scientific journal databases and on institutional Web sites that create PDAs, together with a Qualitative Research (QR) study, using semi-structured interviews and focus group with stakeholders (patients, family members, and health professionals), with the aim of developing a PDA for breast cancer. RESULTS: The SR shows that PDAs in breast cancer not only increase patient knowledge of the illness, leading to more realistic expectations of treatment outcomes, but also reduce passivity in the decision-making process and facilitate the appropriate choice of treatment options in accordance with patient medical and personal preferences. The analysis of QR shows that both breast cancer patients and healthcare professionals agree that surgery, adjuvant treatments, and breast reconstruction represent the most important decisions to be made. Worry, anxiety, optimism, and trust in healthcare professionals were determined as factors that most affected patients subjective experiences of the illness. This HTA was used as the basis for developing a PDA software program. CONCLUSIONS: The SR and QR used in the development of this PDA for breast cancer allowed patients to access information, gain additional knowledge of their illness, make shared treatment decisions, and gave healthcare professionals a deeper insight into patient experiences of the disease. | Decision Support Techniques | Humans | Patient Participation | Young Adult | OK |
| Mbaraka | Amuri | Eveline | Geubbels | Fred | Lwilla | BMC health services research | 2013-00-00 2013 | BACKGROUND: Patient Centred Tuberculosis Treatment (PCT) is a promising treatment delivery strategy for Mycobacterium tuberculosis (TB). It aims to improve adherence to treatment by giving patients the choice of having drug intake supervised at the health facility by a medical professional or at home by a supporter of their choice. METHODS: A cross-sectional survey was undertaken in three districts of Tanzania during October 2007, one year after PCT was rolled out nationally. Semi-structured questionnaires were used to assess whether key elements of the PCT approach were being implemented, to evaluate supporters' knowledge, to capture opinions on factors contributing to treatment completion, and to assess how treatment completion was measured. Transcripts from open-ended responses were analysed using framework analysis. RESULTS: Interviews were conducted with 127 TB patients, 107 treatment supporters and 70 health workers. In total, 25.2% of TB patients were not given a choice about the place of treatment by health workers, and only 13.7% of those given a choice reported that they were given adequate time to make their decision. Only 24.3% of treatment supporters confirmed that they were instructed how to complete patients' treatment cards. Proper health education was the factor most frequently reported by health workers as favouring successful completion of TB treatment (45.7%). The majority of health workers (68.6%) said they checked returned blister packs to verify whether patients had taken their treatment, but only 20.0% checked patients' treatment cards. CONCLUSIONS: The provision of choice of treatment location, information on treatment, and guidance for treatment supporters need to be improved. There is a requirement for regular re-training of health workers with effective supportive supervision if successful implementation of the PCT approach is to be sustained. | Male | Humans | Patient Participation | Questionnaires | OK |
| Michael | Buitron | W. Scott | Comulada | Li-Jung | Liang | Rehabilitation Psychology | 2012-11-00 Nov 2012 | OBJECTIVE: New treatments introduced in the mid-1990s led many people with HIV/AIDS who previously had been disabled by their disease to contemplate workforce reentry; many remain unemployed, and little is known concerning interventions that might help them return to work. We report the results of a randomized clinical trial of an intervention designed to help people with HIV/AIDS reenter the workforce. DESIGN: We tested a mixed (group-individual) modality intervention that incorporated elements of Motivational Interviewing (Miller & Rollnick, 2002), skills building from Dialectical Behavior Therapy (Linehan, 1993), and job-related skills (Price & Vinokur, 1995). A total of 174 individuals participated in either the intervention or in standard of care and were followed for 24 months. RESULTS: Compared with individuals referred for standard of care, participants in the intervention engaged in more workforce-reentry activities over time and, once employed, were more likely to remain employed. Dose-response analyses revealed that among intervention participants, participants who attended more than 1 individual session engaged in more workforce-reentry activities than individuals who attended 1 or fewer individual sessions, whereas frequency of group session participation did not effect a difference between participants who attended more than 6 group sessions and participants who attended 6 or fewer group sessions. CONCLUSION: Theoretically based workforce-reentry assistance programs can assist disabled people with HIV/AIDS in their return-to-work efforts. | Male | Humans | Patient Participation | Questionnaires | OK |
|  |  |  |  |  |  | Harvard Men's Health Watch | 2012-08-00 Aug 2012 | | Male | Humans | Patient Participation | Arrhythmias, Cardiac | OK |
| Alice | Maruelli | Elena | Bandieri | Stefania | Boldini | Tumori | 2012-06-00 2012 May-Jun | AIMS AND BACKGROUND: Although hope is a widely used term, the experience of hope in patients with chronic or even life-threatening diseases is often disregarded due to the scarcity of carefully designed and validated assessment tools. The aim of this study was to validate the Hope Herth Index (HHI) questionnaire in the Italian population of patients with solid or hematological malignancies during active cancer treatment. METHODS: After the translation procedures, the psychometric properties of the Italian version of HHI were evaluated in 266 patients with non-advanced cancer cared for in four different settings. Summative scores ranged from 12-48, with a higher score denoting greater hope. Confirmative factorial analysis was performed to assess dimensionality. The test-retest reliability was assessed by means of the Lin concordance coefficient (two weeks' interval, 80 patients). Concurrent validity was assessed through the following questionnaires: Hospital Anxiety and Depression Scale (HADS), Functional Assessment of Chronic Illness Therapy-Spiritual Well-Being (FACIT-Sp), Edmonton Symptom Assessment Scale (ESAS), and System Belief Inventory (SBI-15R). RESULTS: A total of 266 patients were enrolled. Confirmative factor analysis did not confirm the original three-factor solution, whereas a one-factor solution did perform well. Cronbach's alpha was 0.84 and the test-retest reliability was 0.64 (95% CI 0.51; 0.76). Large convergence was found with spiritual well-being as measured by the FACIT-Sp (0.69) and with anxiety-depression as measured by the HADS (inverse correlation: -0.51). Physical symptoms and religiousness were only slightly correlated, as expected. CONCLUSIONS: The Italian version of HHI is a valid and reliable assessment tool - useful to initiate conversation with someone who is troubled but finds it difficult to talk - in patients with either solid or hematological malignancies on active cancer treatment during the non-advanced stages of the disease. | Decision Making | Humans | Patient Participation | Questionnaires | OK |
| Glyn | Elwyn | Eva | Wartner | Elisabeth | Hörlein | European Journal of Public Health | 2012-08-00 Aug 2012 | BACKGROUND: Increasing emphasis is being placed on involving patients in decisions concerning their health. This shift towards more patient engagement by health professionals and towards more desire by patients for participation may be partly based on socio-political factors. METHODS: To compare the preferences for shared decision making of patients from eastern and western Germany we analysed five patient samples (n = 2318) (general practice patients and schizophrenia patients from eastern and western Germany). Patients' role preferences for shared decisions were measured using the decision-making subscale of the Autonomy Preference Index. RESULTS: Patients resident in eastern Germany expressed lower preferences for shared decision making than patients in western Germany. This was true after controlling for socio-demographic variables and for patient group. CONCLUSION: The cultural imprint (e.g. western vs. former communist society) seems to have a significant influence on patients' expectations and behaviour in the medical encounter. Health services providers need to be aware that health attitudes within the same health system might vary for historical and cultural reasons. The engagement of patients in medical decisions might not be susceptible to a 'one size fits all' approach; doctors should instead aim to accommodate the individual patient's desire for autonomy. | Male | Decision Making | Humans | Patient Participation | OK |
| Jennifer | Nicolai | Mechthild | Hartmann | Wolfgang | Eich | Journal of Clinical Psychology in Medical Settings | 2010-06-00 Jun 2010 | The quality of physician-patient interaction is increasingly being recognized as an essential component of effective treatment. The present article reports on the development and validation of a brief patient self-report questionnaire (QQPPI) that assesses the quality of physician-patient interactions. Data were gathered from 147 patients and 19 physicians immediately after consultations in a tertiary care outpatient setting. The QQPPI displayed good psychometric properties, with high internal consistency and good item characteristics. The QQPPI total score showed variability between different physicians and was independent of patients' gender, age, and education. The QQPPI featured high correlations with other quality-related measures and was not influenced by social desirability, or patients' clinical characteristics. The QQPPI is a brief patient self-report questionnaire that allows assessment of the quality of physician-patient interactions during routine ambulatory care. It can also be used to evaluate physician communication training programs or for educational purposes. | Male | Humans | Patient Participation | Patient Satisfaction | OK |
| Kerry A. | Ryan | Paul S. | Appelbaum | David S. | Knopman | PloS One | 2013-00-00 2013 | BACKGROUND: There is a longstanding concern about the accuracy of surrogate consent in representing the health care and research preferences of those who lose their ability to decide for themselves. We sought informed, deliberative views of the older general public (≥50 years old) regarding their willingness to participate in dementia research and to grant leeway to future surrogates to choose an option contrary to their stated wishes. METHODOLOGY/PRINCIPAL FINDINGS: 503 persons aged 50+ recruited by random digit dialing were randomly assigned to one of three groups: deliberation, education, or control. The deliberation group attended an all-day education/peer deliberation session; the education group received written information only. Participants were surveyed at baseline, after the deliberation session (or equivalent time), and one month after the session, regarding their willingness to participate in dementia research and to give leeway to surrogates, regarding studies of varying risk-benefit profiles (a lumbar puncture study, a drug randomized controlled trial, a vaccine randomized controlled trial, and an early phase gene transfer trial). At baseline, 48% (gene transfer scenario) to 92% (drug RCT) were willing to participate in future dementia research. A majority of respondents (57-71% depending on scenario) were willing to give leeway to future surrogate decision-makers. Democratic deliberation increased willingness to participate in all scenarios, to grant leeway in 3 of 4 scenarios (lumbar puncture, vaccine, and gene transfer), and to enroll loved ones in research in all scenarios. On average, respondents were more willing to volunteer themselves for research than to enroll their loved ones. CONCLUSIONS/SIGNIFICANCE: Most people were willing to grant leeway to their surrogates, and this willingness was either sustained or increased after democratic deliberation, suggesting that the attitude toward leeway is a reliable opinion. Eliciting a person's current preferences about future research participation should also involve eliciting his or her leeway preferences. | Male | Decision Making | Humans | Patient Participation | OK |
|  |  |  |  |  |  | Patient Education and Counseling | 2011-12-00 Dec 2011 | OBJECTIVE: To examine the impact of trust on patient outcomes (satisfaction, HbA(1C), physical and mental health-related quality of life (HRQoL)) and to investigate the role of decision-making preferences in the trust-outcome relationship. METHODS: We conducted a one-year longitudinal analysis of 614 type 2 diabetic patients (mean age: 59.3 years; mean disease duration: 6.7 years). Patients' self-administered questionnaires and medical record were used for the research. Multiple regression analyses were conducted to investigate the relationship among variables during a 12-month follow-up. Further, we used latent growth modeling (LGM) to assess changes in health outcomes and to examine how these changes were related to trust. RESULTS: Regression analyses revealed that trust was positively related to glycemic control, physical HRQoL, and satisfaction at 12 months. Patients with higher decision-making preferences experienced a greater increase in subsequent satisfaction. The results of LGM showed that higher levels of trust were associated with greater increases in physical HRQoL. CONCLUSION: Trust contributes to improvements in health outcomes. The relationship between trust and satisfaction may be stronger among patients with higher decision-making preferences. PRACTICE IMPLICATIONS: For healthcare providers, efforts should be made to cultivate patients' trust and enhance their decision-making preferences to maximize satisfaction and improve outcomes. | Male | Decision Making | Humans | Patient Participation | OK |
| Michael J. | Barry |  |  |  |  | JAMA internal medicine | 2013-07-08 Jul 8, 2013 | IMPORTANCE: Informing and involving patients in their medical decisions is increasingly becoming a standard for good medical care, particularly for primary care physicians. OBJECTIVE: To learn how patients describe the decision-making process for 10 common medical decisions, including 6 that are most often made in primary care. DESIGN: A survey of a national sample of adults 40 years or older who in the preceding 2 years had either experienced or discussed with a health care provider 1 or more of 10 decisions: medication for hypertension, elevated cholesterol, or depression; screening for breast, prostate, or colon cancer; knee or hip replacement for osteoarthritis, or surgery for cataract or low back pain. SETTING: Adults living in households in the United States in 2011. PARTICIPANTS: A national sample of adults drawn from a probability sample-based web panel developed by Knowledge Networks. MAIN OUTCOMES AND MEASURES: Patients' perceptions of the extent to which the pros and cons were discussed with their health care providers, whether the patients were told they had a choice, and whether the patients were asked for their input. RESULTS: Responses were obtained from 2718 patients, with a response rate of 58.3%. Respondents reported much more discussion of the pros than the cons of all tests or treatments; discussions about the surgical procedures tended to be more balanced than those about medications to reduce cardiac risks and cancer screening. Most patients (60%-78%) said they were asked for input for all but 3 decisions: medications for hypertension and elevated cholesterol and having mammograms (37.3%-42.7%). Overall, the reported decision-making processes were most patient centered for back or knee replacement surgery and least for breast and prostate cancer screening. CONCLUSIONS AND RELEVANCE: Discussions about these common tests, medications, and procedures as reported by patients do not reflect a high level of shared decision making, particularly for 5 decisions most often made in primary care. | Male | Decision Making | Humans | Patient Participation | OK |
| Rob | Sanson-Fisher | Marita | Lynagh | Chris | Paul | Patient Education and Counseling | 2012-07-00 Jul 2012 | OBJECTIVE: This study aimed to examine preferred and perceived roles of haematological cancer survivors in treatment decision making. METHODS: Participants were within three years of diagnosis and registered with a cancer registry in one Australian state. 732 eligible survivors were invited to complete a paper and pencil survey. RESULTS: 268 survivors completed the survey (37% response rate). The majority of participants (46%) preferred a passive role in decision making. Thirty percent of participants preferred to make the decision in collaboration with the doctor, whereas 26% preferred an active role in decision making. Just over half of respondents (56%) reported an exact match between their preferred and perceived roles. Where discordance between preferred and perceived roles were identified, survivors were more likely to report having been more passively than actively involved compared to their preferred roles. CONCLUSION: There is considerable variation in haematological cancer survivors' preferred and perceived roles in treatment decision making. PRACTICE IMPLICATIONS: This study highlights the need to improve clinical communication to ensure that patient experiences align with their preferences for involvement in treatment decision making. | Male | Decision Making | Humans | Patient Participation | OK |
|  |  |  |  |  |  | Journal of General Internal Medicine | 2011-01-00 Jan 2011 | BACKGROUND: An important feature of patient-centered care is physician understanding of their patients' health beliefs and values. OBJECTIVE: Determine physicians' awareness of patients' health beliefs as well as communication, relationship, and demographic factors associated with better physician understanding of patients' illness perspectives. DESIGN: Cross-sectional, observational study. RESEARCH PARTICIPANTS: A convenience sample of 207 patients and 29 primary care physicians from 10 outpatient clinics. APPROACH AND MEASURES: After their consultation, patients and physicians independently completed the CONNECT instrument, a measure that assesses beliefs about the degree to which the patient's condition has a biological cause, is the patient's fault, is one the patient can control, has meaning for the patient, can be treated with natural remedies, and patient preferences for a partnership with the physician. Physicians completed the measure again on how they thought the patient responded. Active patient participation (frequency of questions, concerns, acts of assertiveness) was coded from audio-recordings of the consultations. Physicians' answers for how they thought the patient responded to the health belief measure were compared to their patients' actual responses. Degree of physician understanding of patients' health beliefs was computed as the absolute difference between patients' health beliefs and physicians' perception of patients' health beliefs. KEY RESULTS: Physicians' perceptions of their patients' health beliefs differed significantly (P<0.001) from patients' actual beliefs. Physicians also thought patients' beliefs were more aligned with their own. Physicians had a better understanding of the degree to which patients believed their health conditions had personal meaning (p=0.001), would benefit from natural remedies (p=0.049), were conditions the patient could control (p=0.001), and wanted a partnership with the doctor (p=0.014) when patients more often asked questions, expressed concerns, and stated their opinions. Physicians were poorer judges of patients' beliefs when patients were African-American (desire for partnership) (p=0.013), Hispanic (meaning) (p=0.075), or of a different race (sense of control) (p=0.024). CONCLUSIONS: Physicians were not good judges of patient's health beliefs, but had a substantially better understanding when patients more actively participated in the consultation. Strategies for increasing physicians' awareness of patients' health beliefs include preconsultation assessment of patients' beliefs, implementing culturally appropriate patient activation programs, and greater use of partnership-building to encourage active patient participation. | Male | Humans | Patient Participation | Patient Satisfaction | OK |
| Mark | Fenton | Lester | Firkins | Jenny | Versnel | Journal of Evaluation in Clinical Practice | 2010-06-00 Jun 2010 | BACKGROUND: To arrive at an agreed, prioritized ranking of treatment uncertainties in asthma that need further research, by developing a collaboration of patients, carers and clinicians, facilitated by the James Lind Alliance Working Partnership between Asthma UK and the British Thoracic Society. METHODS: A four-step procedure: (1) establish a collaborative Working Partnership; (2) identify and collect treatment uncertainties by using a patient survey and analysing existing systematic reviews, clinical guidelines and query-answering services; (3) categorize uncertainties; and (4) convene a workshop using a nominal group process to establish a ranked prioritization of treatment uncertainties in asthma. FINDINGS: Agreement and rankings were reached for 10 treatment uncertainties. The highest was given to the uncertainty surrounding the adverse effects of inhaled and oral steroids. The top three priorities dealt with clinical management issues, where uncertainties still exist, namely concerns about the side effects of inhaled and oral steroids, how to manage asthma when other illnesses exist or how to rely on personal decisions in an ever-changing illness (self-management). INTERPRETATION: The key outcome is the generation of a prioritized list of treatment uncertainties in asthma, agreed by a collaboration of patients and health professionals, to inform the commissioning of new research. Such a large number of patient-identified treatment uncertainties had not previously been identified in the literature, an indication perhaps that asthma self-management is a neglected research area. Whether the results have an influence of research funding decisions is not yet known. | Humans | Patient Participation | Questionnaires | Cooperative Behavior | OK |
| Phyllis | Butow | Shawna | Edgerson | Martin H. N. | Tattersall | Patient Education and Counseling | 2011-07-00 Jul 2011 | OBJECTIVE: Question Prompt Lists (QPLs) have demonstrated significant promise in facilitating communication in general, surgical and palliative oncology settings yet have not been tested in the oncology clinical trial setting. We aimed to develop a targeted QPL for Clinical Trials (QPL-CT). METHODS: Breast, lung, and genitourinary cancer patients who either had (trial experienced) or had not (trial naive) participated in a clinical trial joined focus groups to help develop and explore the acceptability of a QPL-CT. Clinicians also participated in separated focus groups. Focus groups were audio-recorded and transcribed. We explored patients' trial information needs and views about the utility of the QPL-CT. RESULTS: Patients focused on four trial information needs: understanding foundational information, conflict of interest issues and financial implications of trial participation. Of note, experienced patients need to personalize foundational information. CONCLUSION: Patient information needs varied considerably and were influenced by trial experience. Patients valued the QPL-CT as an aid to gathering new trial information and ensuring that their information needs were met. PRACTICE IMPLICATIONS: Physicians need to carefully elicit patient information needs and tailor trial information to meet these needs. The QPL-CT, may be of great benefit during clinical trial discussions. | Male | Humans | Patient Participation | Physician-Patient Relations | OK |
| Paul | Little | Rona | Moss-Morris | Nadine E. | Foster | The British Journal of General Practice: The Journal of the Royal College of General Practitioners | 2013-07-00 Jul 2013 | BACKGROUND: Current evidence-based guidelines for low back pain (LBP) recommend multiple diverse approaches to treatment and suggest considering patient preferences when formulating a treatment plan. AIM: To explore patient preferences and to identify patients' beliefs about LBP treatments. DESIGN AND SETTING: Qualitative study using focus groups in primary care in South-West England. METHOD: Thirteen focus groups were organised with a purposive sample of 75 adults with LBP. Group discussions of LBP treatments were facilitated, audiorecorded, and the verbatim transcripts thematically analysed. RESULTS: Eight themes were identified, four related to treatment beliefs and four to seeking treatment. Treatment beliefs comprised participants' expectations and appraisals of specific treatments, which were underpinned by four distinct dimensions: credibility, effectiveness, concerns and individual fit. Treatment beliefs were expressed in the broader context of treatment seeking: participants' primary concern was to obtain a clear explanation of their LBP which went beyond a diagnostic label and provided an understanding of the cause(s) of their LBP. They described engaging in self-management activities and claimed they were willing to try anything if it might help them. Participants wanted an empathic and expert practitioner who could deliver a suitable treatment (or refer them on to someone else) and help them to negotiate the challenges of the healthcare system. CONCLUSION: These findings highlight the importance of helping patients develop coherent illness representations about their LBP before trying to engage them in treatment-decisions, uptake, or adherence. Addressing patients' illness and treatment perceptions in clinical practice could improve shared decision making and patient outcomes. | Male | Decision Making | Humans | Patient Satisfaction | OK |
| Kimberly J. | Reid | Shannon M. | Dunlay | Paul S. | Chan | Circulation. Cardiovascular Quality and Outcomes | 2011-07-00 Jul 2011 | BACKGROUND: Communicating prognosis to enable shared decision-making is strongly endorsed by heart failure (HF) guidelines. Patients are concerned with both their quantity and quality of life (QoL). To facilitate the recognition of patients at high risk for unfavorable future QoL or death, we created a simple prognostic tool to estimate this combined outcome. METHODS AND RESULTS: We identified factors associated with 6-month mortality or persistently unfavorable QoL, defined by Kansas City Cardiomyopathy Questionnaire (KCCQ) scores <45 at 1 and 24 weeks after hospital discharge, among 1458 patients from the Efficacy of Vasopressin Antagonism in HF Outcome Study with Tolvaptan (EVEREST). Within 24 weeks of discharge, 478 (32.8%) patients had died and 192 (13.2%) patients had serial KCCQ scores <45. After adjusting for 23 predischarge covariates, independent predictors of the combined end point included low admission KCCQ score, high B-type natriuretic peptide, hyponatremia, tachycardia, hypotension, absence of β-blocker therapy, and history of diabetes mellitus and arrhythmia. A simplified predischarge HF score for subsequent death or unfavorable QoL had moderate discrimination (c-statistic 0.72). Predischarge clinical covariates were substantially different in predicting the QoL end point as compared with traditional death or rehospitalization end points. CONCLUSIONS: At the time of hospital discharge, readily available clinical characteristics are associated with HF patients at high risk for persistently unfavorable QoL or death over the next 6 months. Such information can target patients for whom aggressive treatment options (eg, devices or transplantation) and/or end-of-life discussions should be strongly considered before hospital discharge. | Male | Humans | Questionnaires | Female | OK |
| Constantine | Daskalakis | Michelle | Shwarz | Barry W. | Rovner | Journal of Clinical Psychopharmacology | 2012-12-00 Dec 2012 | OBJECTIVE: Methods to evaluate adverse effects of medications are significantly underdeveloped compared to those for efficacy. In this pilot proof-of-concept study, we preliminarily compared a novel approach-the Symptom Assessment Tool (SAT)-to a systematic and detailed assessment by a physician for identifying symptoms that were potentially adverse effects (sensitivity) and excluding symptoms that were unlikely to be adverse effects (specificity). METHODS: A symptom inventory and rating of symptom severity were completed before starting a psychotropic medication (or increasing its dose), and again 2 weeks later. Each symptom was systematically assessed by the patient-rated SAT and by a physician and was classified as either a potential or unlikely adverse effect. The primary analysis compared the classification of symptoms by the SAT to that by the physician. Potential adverse effects were also subcategorized as possible or probable adverse effects. RESULTS: A sample of 193 symptoms from 15 adults was evaluated, only 37.3% of which were considered potential adverse effects by the physician. Sensitivity of the SAT compared to physician's assessment was 90.3% for potential adverse effects and 97.5% for the subgroup of probable adverse effects. The SAT correctly identified 63.6% of the symptoms as unlikely adverse effects (specificity), and its negative predictive value was 91.7%. CONCLUSIONS: The SAT, appropriate for its intended use as a screening tool, had high sensitivity and moderate specificity and could present physicians with a limited number of potential adverse effects for further assessment and intervention. Further evaluation and refinement of this approach is warranted. | Male | Humans | Patient Participation | Questionnaires | OK |
| Lanae | Miner | Larry | Mauksch | Kelly | Edwards | Patient Education and Counseling | 2011-04-00 Apr 2011 | OBJECTIVE: To categorize physician communication demonstrating understanding of what patients want to know and skill in conveying that information. Physicians underestimate how much information patients want and patients rarely seek information during clinic visits. Transparent communication is advocated to facilitate patient understanding and support autonomy, informed decision-making and relationship development. METHODS: Analysis and coding of 263 audiotaped interactions between 33 primary care physicians and their patients in eight community-based, primary care clinics in Washington State, USA. RESULTS: Physicians proactively used five types of process transparency to preview speech and actions. Four types of content transparency were used to explicate diagnosis and treatment, demystify medical language and concepts, and interpret biomedical information. Physicians spent the greatest proportion of clinic time explicating medical content. CONCLUSION: The primacy of information exchange over process-oriented, relational communication was demonstrated. Proactive transparency appears promising to increase understanding and collaboration. PRACTICE IMPLICATIONS: In patient-centered care where collaboration is the ideal, transparency in its various forms is a critical ingredient. Without much communicative effort, physicians who proactively communicated that an examination was over, that they were leaving the exam room briefly so patients could dress provided information that appeared to address patient uncertainty and demonstrated empathy and respect. | Male | Decision Making | Humans | Patient Participation | OK |
| Jesus | Zarate | Claudine | Ward |  |  | Journal of Medical Internet Research | 2013-00-00 2013 | BACKGROUND: Personal health records (PHRs) are an important tool for empowering patients and stimulating health action. To date, the volitional adoption of publicly available PHRs by consumers has been low. This may be partly due to patient concerns about issues such as data security, accuracy of the clinical information stored in the PHR, and challenges with keeping the information updated. One potential solution to mitigate concerns about security, accuracy, and updating of information that may accelerate technology adoption is the provision of PHRs by employers where the PHR is pre-populated with patients' health data. Increasingly, employers and payers are offering this technology to employees as a mechanism for greater patient engagement in health and well-being. OBJECTIVE: Little is known about the antecedents of PHR acceptance in the context of an employer sponsored PHR system. Using social cognitive theory as a lens, we theorized and empirically tested how individual factors (patient activation and provider satisfaction) and two environment factors (technology and organization) influence patient intentions to use a PHR among early adopters of the technology. In technology factors, we studied tool empowerment potential and value of tool functionality. In organization factors, we focused on communication tactics deployed by the organization during PHR rollout. METHODS: We conducted cross-sectional analysis of field data collected during the first 3 months post go-live of the deployment of a PHR with secure messaging implemented by the Air Force Medical Service at Elmendorf Air Force Base in Alaska in December 2010. A questionnaire with validated measures was designed and completed by 283 participants. The research model was estimated using moderated multiple regression. RESULTS: Provider satisfaction, interactions between environmental factors (communication tactics and value of the tool functionality), and interactions between patient activation and tool empowerment potential were significantly (P<.05) associated with behavioral intentions to use the PHR tool. The independent variables collectively explained 42% of the variance in behavioral intentions. CONCLUSIONS: The study demonstrated that individual and environmental factors influence intentions to use the PHR. Patients who were more satisfied with their provider had higher use intentions. For patients who perceived the health care process management support features of the tool to be of significant value, communication tactics served to increase their use intentions. Finally, patients who believed the tool to be empowering demonstrated higher intentions to use, which were further enhanced for highly activated patients. The findings highlight the importance of communication tactics and technology characteristics and have implications for the management of PHR implementations. | Male | Humans | Patient Participation | Patient Satisfaction | OK |
| Catherine | Nadeau | Caroline | Rhéaume | Stéphane | Turcotte | Implementation science: IS | 2013-00-00 2013 | BACKGROUND: DECISION + 2, a training program for physicians, is designed to implement shared decision making (SDM) in the context of antibiotics use for acute respiratory tract infections (ARTIs). We evaluated the impact of DECISION + 2 on SDM implementation as assessed by patients and physicians, and on physicians' intention to engage in SDM. METHODS: From 2010 to 2011, a multi-center, two-arm, parallel randomized clustered trial appraised the effects of DECISION + 2 on the decision to use antibiotics for patients consulting for ARTIs. We randomized 12 family practice teaching units (FPTUs) to either DECISION + 2 or usual care. After the consultation, both physicians and patients independently completed questionnaires based on the D-Option scale regarding SDM behaviors during the consultation. Patients also answered items assessing the role they assumed during the consultation (active/collaborative/passive). Before and after the intervention, physicians completed a questionnaire based on the Theory of Planned Behavior to measure their intention to engage in SDM. To account for the cluster design, we used generalized estimating equations and generalized linear mixed models to assess the impact of DECISION + 2 on the outcomes of interest. RESULTS: A total of 270 physicians (66% women) participated in the study. After DECISION + 2, patients' D-Option scores were 80.1 ± 1.1 out of 100 in the intervention group and 74.9 ± 1.1 in the control group (p = 0.001). Physicians' D-Option scores were 79.7 ± 1.8 in the intervention group and 76.3 ± 1.9 in the control group (p = 0.2). However, subgroup analyses showed that teacher physicians D-Option scores were 79.7 ± 1.5 and 73.0 ± 1.4 respectively (p = 0.001). More patients reported assuming an active or collaborative role in the intervention group (67.1%), than in the control group (49.2%) (p = 0.04). There was a significant relation between patients' and physicians' D-Option scores (p < 0.01) and also between patient-reported assumed roles and both D-Option scores (as assessed by patients, p < 0.01; and physicians, p = 0.01). DECISION + 2 had no impact on the intention of physicians to engage in SDM. CONCLUSION: DECISION + 2 positively influenced SDM behaviors as assessed by patients and teacher physicians. Physicians' intention to engage in SDM was not affected by DECISION + 2. TRIAL REGISTRATION: ClinicalTrials.gov trials register no. NCT01116076. | Male | Decision Making | Humans | Patient Participation | OK |
| Jørgen | Nexøe | Henrik | Støvring | Dorte | Gyrd-Hansen | BMC health services research | 2013-00-00 2013 | BACKGROUND: Risk communication is an integral part of shared decision-making in health care. In the context of interventions for chronic diseases it represents a particular challenge for all health practitioners. By using two different quantitative formats to communicate risk level and effectiveness of a cholesterol-lowering drug, we posed the research question: how does the format of risk information influence patients' decisions concerning therapy, patients' satisfaction with the communication as well as confidence in the decision. We hypothesise that patients are less prone to accept therapy when the benefits of long-term intervention are presented in terms of prolongation of life (POL) in months compared to the absolute risk reduction (ARR). We hypothesise that patients presented with POL will be more satisfied with the communication and confident in their decision, suggesting understanding of the time-related term. METHODS/DESIGN: In 2009 a sample of 328 general practitioners (GPs) in the Region of Southern Denmark was invited to participate in a primary care-based clinical trial among patients making real-life clinical decisions together with their GP. Interested GPs were cluster-randomised to inform patients about cardiovascular disease (CVD) risk and the effectiveness of statin therapy using either POL or ARR. The GPs attended a training session before informing their patients. Before training and after the trial period they received a questionnaire about their attitudes to risk communication and the use of numerical information. Patients' redemptions of statin prescriptions will be registered in a regional prescription database to evaluate a possible association between redemption rates and effectiveness format. The Combined Outcome Measure for Risk Communication And Treatment Decision Making Effectiveness (COMRADE) questionnaire will be used to measure patients' confidence and satisfaction with the risk communication immediately after the conversation with their GPs. DISCUSSION: This randomised clinical trial compares the impact of two effectiveness formats on real-life risk communication between patients and GPs, including affective patient outcomes and actual choices about acceptance of therapy. Though we found difficulties in recruiting GPs, according to the study protocol we have succeeded in engaging sufficient GPs for the trial, enabling us to perform the planned analyses. | Humans | Patient Participation | Patient Satisfaction | Physician-Patient Relations | OK |
|  |  |  |  |  |  | Cancer Nursing | 2012-12-00 2012 Nov-Dec | BACKGROUND: Prostate cancer (PC) patients continue to have unmet information needs at the time of diagnosis and are often unable to communicate their preferences to physicians at the time of the treatment consultation. OBJECTIVE: The objective of the study was to determine the impact of health information-seeking behavior (HISB) and personal factors on patients' preferred role in treatment decision making (TDM). METHODS: Participants consisted of 150 men with newly diagnosed PC seen at 2 urology clinics in western Canada. A survey questionnaire was used to gather information on HISB, personal factors influencing treatment choice, and decision control. RESULTS: More than 90% of the participants reported a preference to play either an active or collaborative role in TDM and having either an "intense" or "complementary" HISB. No significant association was found between HISB and preferred role in TDM. Impact of treatment on survival and urinary function and the urologist's recommendation were identified as the 3 main factors influencing the treatment decision. CONCLUSIONS: At the time of diagnosis, the majority of men want to be involved in TDM and have access to information. Our findings suggest that the type and amount of information men want to access are dependent on HISB. Assessing factors having an impact on TDM may prove useful to guide patient-clinician treatment discussions. IMPLICATIONS FOR PRACTICE: This survey provides clinicians with a method to assess information and decision preferences of men with newly diagnosed PC and factors having an influence on treatment choice. | Male | Decision Making | Humans | Patient Participation | OK |
| Anthony C. | Leonard | Estrelita | Dixon | Christine | Burrows | Patient Education and Counseling | 2012-05-00 May 2012 | OBJECTIVE: Study impact of health literacy on educational intervention for patients "Living with Coronary Artery Disease." METHODS: 187 patients were randomized to: VHS/DVD plus printed booklet; or booklet alone prior to scheduled visit. Main outcome measures included CAD knowledge assessment, clinical outcomes (weigh and blood pressure) and health behaviors (diet, exercise, and smoking); while functional health literacy was assessed as a possible predictor variable. RESULTS: Knowledge scores and health behaviors improved following both interventions. Those receiving the booklet and video also had a significant improvement in exercise, and weight loss. There was a trend (p=0.07) towards greater improvement in test scores among those receiving the booklet plus video. Patients with lower health literacy benefited as much as higher literacy patients. CONCLUSIONS: Incorporation of an educational program into clinical visits for patients with chronic disease improved disease-specific knowledge and prompted patients to become activated and involved in their care, improving health behaviors and outcomes. Lower health literacy was not a barrier to this beneficial effect. PRACTICE IMPLICATIONS: Patients with lower health literacy may also benefit from educational, shared decision-making interventions. | Male | Humans | Questionnaires | Female | OK |
| Erik | Taal | K. Wiepke | Drossaers-Bakker | Harald E. | Vonkeman | BMC musculoskeletal disorders | 2014-00-00 2014 | BACKGROUND: To measure the use, satisfaction and impact of a web portal which provides patients with rheumatoid arthritis home access to their electronic medical records (EMR). METHODS: A pretest-posttest study was conducted among 360 patients. Questionnaires assessed socio-demographics, health literacy, Internet use, disease characteristics, patient-provider relationship and empowerment before and after launching a hospital-based patient web portal. To measure the impact of the portal, patients' satisfaction with care, trust in their rheumatologist, self-efficacy in patient-provider communication, illness perceptions, and medication adherence were assessed. The post-test included questions on portal use, satisfaction, and self-perceived impact due to portal use. RESULTS: 54% of respondents with Internet access had viewed their EMR. Respondents were positive about the ease of use and usefulness of the portal and reported very few problems. Age (P = .03), amount of Internet use (P = .01) and self-perceived Internet skills (P = .03) significantly predicted portal use. Of the respondents who had logged in, 44% reported feeling more involved in their treatment and 37% felt they had more knowledge about their treatment. Significant differences over time were not found on the empowerment-related instruments. CONCLUSIONS: The current portal succeeded in offering patients access to their EMR in a usable and understandable way. While its true impact is difficult to grasp, a relevant portion of the patients felt more involved in their treatment due to the web portal. Offering patients home EMR access, therefore, appears to be a valuable addition to the care process. | Male | Humans | Patient Participation | Patient Satisfaction | OK |
| Fatma | Abbou | Estelle | Henry | Emmanuel | Grimprel | Vaccine | 2012-08-10 Aug 10, 2012 | Parent-to-infant transmission of pertussis remains an issue in France. Although adult booster vaccination was introduced in 2004 as part of a cocooning strategy targeted primarily to parents of young infants, vaccination coverage in this population has remained low. The aim of this study was to evaluate the impact on vaccination coverage, over two consecutive years, of a protocol in which information about the pertussis booster and a prescription for pertussis vaccine were given to parents upon discharge from a French university maternity hospital. A questionnaire was administered to mothers two months after delivery, during two 3-month periods in 2008 and 2009. Participation rates were 67% (first period) and 76.3% (second period). Information about pertussis was delivered mainly by paediatricians and midwives and was considered clear and pertinent in more than 95% of cases. In 2009, 69% of mothers and 63% of fathers who received a prescription for pertussis vaccine before discharge from the maternity declared being vaccinated, with no difference as compared to 2008. Vaccination was done by a general practitioner (95.9%) and mostly in the first month after birth (81%). Postpartum information about pertussis was successfully implemented and well understood by parents in the maternity hospital and should contribute towards increasing pertussis vaccination coverage in parents of young children. | Male | Humans | Patient Participation | Questionnaires | OK |
| David | Reeves | Tom | Blakeman | Robert | Bowen | BMJ (Clinical research ed.) | 2013-00-00 2013 | OBJECTIVE: To determine the effectiveness of an intervention to enhance self management support for patients with chronic conditions in UK primary care. DESIGN: Pragmatic, two arm, cluster randomised controlled trial. SETTING: General practices, serving a population in northwest England with high levels of deprivation. PARTICIPANTS: 5599 patients with a diagnosis of diabetes (n=2546), chronic obstructive pulmonary disease (n=1634), and irritable bowel syndrome (n=1419) from 43 practices (19 intervention and 22 control practices). INTERVENTION: Practice level training in a whole systems approach to self management support. Practices were trained to use a range of resources: a tool to assess the support needs of patients, guidebooks on self management, and a web based directory of local self management resources. Training facilitators were employed by the health management organisation. MAIN OUTCOME MEASURES: Primary outcomes were shared decision making, self efficacy, and generic health related quality of life measured at 12 months. Secondary outcomes were general health, social or role limitations, energy and vitality, psychological wellbeing, self care activity, and enablement. RESULTS: We randomised 44 practices and recruited 5599 patients, representing 43% of the eligible population on the practice lists. 4533 patients (81.0%) completed the six month follow-up and 4076 (72.8%) the 12 month follow-up. No statistically significant differences were found between patients attending trained practices and those attending control practices on any of the primary or secondary outcomes. All effect size estimates were well below the prespecified threshold of clinically important difference. CONCLUSIONS: An intervention to enhance self management support in routine primary care did not add noticeable value to existing care for long term conditions. The active components required for effective self management support need to be better understood, both within primary care and in patients' everyday lives. TRIAL REGISTRATION: Current Controlled Trials ISRCTN90940049. | Male | Decision Making | Humans | Questionnaires | OK |
| J. | Kasper | T. | Heidegger |  |  | Anaesthesia | 2013-07-00 Jul 2013 | There is a lack of data about the implementation of shared decision making in anaesthesia. To assess patients' preference to be involved in medical decision making and its influence on patient satisfaction, we studied 197 matched pairs (patients and anaesthetists) using two previously validated questionnaires. Before surgery, patients had to decide between general vs regional anaesthesia and, where appropriate, between conventional postoperative pain therapy vs catheter techniques. One hundred and eighty-six patients (94%) wished to be involved in shared decision making. One hundred and twenty-two patients (62%) experienced the exact amount of shared decision making that they wanted; 44 (22%) were slightly more involved and 20 (10%) slightly less involved in shared decision making than they desired. Preferences regarding involvement in shared decision making were similar between patients and anaesthetists with mean (SD) points of 54.1 (16.2) vs 56.4 (27.6) (p=0.244), respectively on a 0-100 scale; however, patients were found to have a stronger preference for a totally balanced shared decision-making process (65% vs 32%). Overall patient satisfaction was high: 88% were very satisfied and 12% satisfied with a mean (SD) value of 96.1 (10.6) on a 0-100 scale. Shared decision making is important for providing high levels of patient satisfaction. | Male | Humans | Patient Satisfaction | Physician-Patient Relations | OK |
| Sandy J. | Rutherford | Nicola M. | Kayes | Kathryn M. | McPherson | Disability and Rehabilitation | 2012-00-00 2012 | PURPOSE: Client-centred practice is widely considered a key element of rehabilitation. However, there is limited discussion of how it should be implemented. This study explored how client-centred practice was operationalized during a clinical trial of innovative goal-setting techniques. METHOD: This study drew on principles of co-autoethnography. The personal experiences of three clinical researchers were explored to identify insights into client-centred practice, and seek understanding of this within the broader socio-cultural context. Data were collected through group discussions and written reflections. Thematic analysis and coding were used to identify the dominant themes from the data. RESULTS: The primary way that client-centred practice was operationalized was through listening in order to get to know, to uncover and to understand what was meaningful. Four strategies were identified: utilizing mindful listening, allowing time, supporting clients to prioritize what is meaningful and viewing the therapists' role differently. CONCLUSION: While technical competence in rehabilitation is important, our study suggested a starting point of 'being with' rather than 'doing to' may be beneficial for engaging people in their rehabilitation. We have highlighted a number of practical strategies that can be used to facilitate more client-centred practice. These approaches are consistent with what clients report they want and need from rehabilitation services. | Humans | Patient Participation | self report | communication | OK |
| Chuan-Fen | Liu | Elizabeth M. | Yano | Cory | Bolkan | Implementation science: IS | 2011-00-00 2011 | BACKGROUND: Meta-analyses show collaborative care models (CCMs) with nurse care management are effective for improving primary care for depression. This study aimed to develop CCM approaches that could be sustained and spread within Veterans Affairs (VA). Evidence-based quality improvement (EBQI) uses QI approaches within a research/clinical partnership to redesign care. The study used EBQI methods for CCM redesign, tested the effectiveness of the locally adapted model as implemented, and assessed the contextual factors shaping intervention effectiveness. METHODS: The study intervention is EBQI as applied to CCM implementation. The study uses a cluster randomized design as a formative evaluation tool to test and improve the effectiveness of the redesign process, with seven intervention and three non-intervention VA primary care practices in five different states. The primary study outcome is patient antidepressant use. The context evaluation is descriptive and uses subgroup analysis. The primary context evaluation measure is naturalistic primary care clinician (PCC) predilection to adopt CCM.For the randomized evaluation, trained telephone research interviewers enrolled consecutive primary care patients with major depression in the evaluation, referred enrolled patients in intervention practices to the implemented CCM, and re-surveyed at seven months. RESULTS: Interviewers enrolled 288 CCM site and 258 non-CCM site patients. Enrolled intervention site patients were more likely to receive appropriate antidepressant care (66% versus 43%, p = 0.01), but showed no significant difference in symptom improvement compared to usual care. In terms of context, only 40% of enrolled patients received complete care management per protocol. PCC predilection to adopt CCM had substantial effects on patient participation, with patients belonging to early adopter clinicians completing adequate care manager follow-up significantly more often than patients of clinicians with low predilection to adopt CCM (74% versus 48%%, p = 0.003). CONCLUSIONS: Depression CCM designed and implemented by primary care practices using EBQI improved antidepressant initiation. Combining QI methods with a randomized evaluation proved challenging, but enabled new insights into the process of translating research-based CCM into practice. Future research on the effects of PCC attitudes and skills on CCM results, as well as on enhancing the link between improved antidepressant use and symptom outcomes, is needed. TRIAL REGISTRATION: ClinicalTrials.gov: NCT00105820. | Male | Humans | United States | Female | OK |
| Jodi | Lynch | Elizabeth | Hovey | Meera | Agar | Patient Education and Counseling | 2012-02-00 Feb 2012 | OBJECTIVE: To examine the feasibility and acceptability of routine provision of patient question prompt lists (QPLs) to promote patient participation and patient-clinician communication in medical consultations. METHODS: Four cancer centres across NSW, Australia (two rural, two urban) were invited to participate, involving distribution of QPLs to patients seeing a medical or radiation oncologist, or palliative care clinician. Patients rated their satisfaction after their next consultation. Cancer specialists provided their views at the end of the study. RESULTS: Sixty-four percent (389/606) of patients attending consultations received a QPL. Of patients offered a QPL (426), 91% accepted. Of 139 patients surveyed post-consultation, 89% reported reading the QPL and, of these, 44% referred to the QPL during the consultation at least once. All of 10 cancer specialists providing their views post-implementation reported that QPL implementation in routine practice was feasible and did not strain resources. CONCLUSIONS: Cancer patients and cancer specialists showed support for routine dissemination of the QPL. PRACTICE IMPLICATIONS: For successful implementation of evidence-based tools we recommend promotion by local clinical champions, negotiation with clinic staff about dissemination methods, raised patient awareness through on-site project facilitators, media, consumer and support groups, and availability of resources in hard copy and via online sources. | Male | Humans | Patient Participation | Physician-Patient Relations | OK |
| Hamid | Benalia | Julia | Downing | Faith N. | Mwangi-Powell | Health and Quality of Life Outcomes | 2011-00-00 2011 | BACKGROUND: Patient-reported outcome measurement (PROM) plays an increasingly important role in palliative care. A variety of measures exists and is used in clinical care, audit and research. However, little is known about professionals' views using these measures. The aim of this study is to describe the use and experiences of palliative care professionals with outcome measures. METHODS: A web-based online survey was conducted in Europe and Africa. Professionals working in clinical care, audit and research in palliative care were invited to the survey via national palliative care associations and various databases. Invitation e-mails were sent with a link to the questionnaire. RESULTS: Overall participation rate 42% (663/1592), overall completion rate 59% (392/663). The majority of respondents were female (63.4%), mean age 46 years (SD 9). 68.1% respondents from Europe and 73.3% from Africa had experiences with outcome measures in palliative care. Non-users reported time constraints, burden, lack of training and guidance as main reasons. In clinical care/audit, assessment of patients' situation, monitoring changes and evaluation of services were main reasons for use. Choice of OMs for research was influenced by validity of the instrument in palliative care and comparability with international literature. Main problems were related to patient characteristics, staff, and outcome measures. Participants expressed the need for more guidance and training in the use of PROMs. CONCLUSIONS: Professionals need more support for the use and implementation of PROMs in clinical practice and research through training and guidance in order to improve patient care. | Male | Humans | Patient Participation | Patient Satisfaction | OK |
| Deborah | Debono | Jeffrey | Braithwaite |  |  | BMC health services research | 2014-00-00 2014 | BACKGROUND: There is growing recognition of the importance of the active involvement of consumers and community members in health care. Despite the long history of consumer and community engagement (CCE) research and practice, there is no consensus on the best strategies for CCE. In this paper, we identify various dimensions of CCE-related strategies and offer a practical model to assist policy-makers, practitioners and researchers. METHODS: We undertook a large-scale, scoping meta-review and searched six databases using a list of nine medical subject headings (MeSH) and a comprehensive list of 47 phrases. We identified and examined a total of 90 relevant systematic reviews. RESULTS: Identified reviews show that although there is a significant body of research on CCE, the development of the field is hindered by a lack of evidence relating to specific elements of CCE. They also indicate a diverse and growing enterprise, drawing on a wide range of disciplinary, political and philosophical perspectives and a mix of definitions, targets, approaches, strategies and mechanisms. CCE interventions and strategies aim to involve consumers, community members and the public in general, as well as specific sub-groups, including children and people from culturally and linguistically diverse backgrounds. Strategies for CCE vary in terms of their aim and type of proposed activity, as do the methods and tools which have been developed to support them. Methods and tools include shared decision making, use of decision aids, consumer representation, application of electronic and internet-based facilities, and peer support. The success of CCE is dependent on both the approach taken and contextual factors, including structural facilitators such as governmental support, as well as barriers such as costs, organisational culture and population-specific limitations. CONCLUSIONS: The diversity of the field indicates the need to measure each component of CCE. This meta-review provides the basis for development of a new eight stage model of consumer and community engagement. This model emphasises the importance of clarity and focus, as well as an extensive evaluation of contextual factors within specific settings, before the implementation of CCE strategies, enabling those involved in CCE to determine potential facilitators and barriers to the process. | | | | | OK |
| Robert | Bowen | Victoria | Lee | Tom | Blakeman | International Journal of Nursing Studies | 2014-08-00 août 2014 | Background: An implementation gap exists between policy aspirations for provision and the delivery of self-management support in primary care. An evidence based training and support package using a whole systems approach implemented as part of a randomised controlled trial was delivered to general practice staff. The trial found no effect of the intervention on patient outcomes. This paper explores why self-management support failed to become part of normal practice. We focussed on implementation of tools which capture two key aspects of self-management support—education (guidebooks for patients) and forming collaborative partnerships (a shared decision-making tool). Objectives: To evaluate the implementation and embedding of self-management support in a United Kingdom primary care setting. Design: Qualitative semi-structured interviews with primary care professionals. Settings: 12 General Practices in the Northwest of England located within a deprived inner city area. Participants: Practices were approached 3–6 months after undergoing training in a self-management support approach. A pragmatic sample of 37 members of staff—General Practitioners, nurses, and practice support staff from 12 practices agreed to take part. The analysis is based on interviews with 11 practice nurses and one assistant practitioner; all were female with between 2 and 21 years’ experience of working in general practice. Methods: A qualitative design involving face-to-face, semi-structured interviews audio-recorded and transcribed. Normalisation Process Theory framework allowed a systematic evaluation of the factors influencing the work required to implement the tools. Findings: The guidebooks were embedded in daily practice but the shared decision-making tools were not. Guidebooks were considered to enhance patient-centredness and were minimally disruptive. Practice nurses were reluctant to engage with behaviour change discussions. Self-management support was not formulated as a practice priority and there was minimal support for this activity within the practice: it was not auditable; was insufficiently differentiated from existing content and processes of work to value in its own right, and considered too disruptive and time-consuming. Conclusion: Supporting self-management through the encouragement of lifestyle change was problematic to realise with limited evidence of the development of the needed collaborative partnerships between patients and practitioners required by the ethos of self-management support. (PsycINFO Database Record (c) 2014 APA, all rights reserved). (journal abstract) | Decision Making | Primary Health Care | long term conditions | normalization process theory | Doublon |
| Robert | Bowen | Victoria | Lee | Tom | Blakeman | International Journal of Nursing Studies | 2014-08-00 Aug 2014 | BACKGROUND: An implementation gap exists between policy aspirations for provision and the delivery of self-management support in primary care. An evidence based training and support package using a whole systems approach implemented as part of a randomised controlled trial was delivered to general practice staff. The trial found no effect of the intervention on patient outcomes. This paper explores why self-management support failed to become part of normal practice. We focussed on implementation of tools which capture two key aspects of self-management support - education (guidebooks for patients) and forming collaborative partnerships (a shared decision-making tool). OBJECTIVES: To evaluate the implementation and embedding of self-management support in a United Kingdom primary care setting. DESIGN: Qualitative semi-structured interviews with primary care professionals. SETTINGS: 12 General Practices in the Northwest of England located within a deprived inner city area. PARTICIPANTS: Practices were approached 3-6 months after undergoing training in a self-management support approach. A pragmatic sample of 37 members of staff - General Practitioners, nurses, and practice support staff from 12 practices agreed to take part. The analysis is based on interviews with 11 practice nurses and one assistant practitioner; all were female with between 2 and 21 years' experience of working in general practice. METHODS: A qualitative design involving face-to-face, semi-structured interviews audio-recorded and transcribed. Normalisation Process Theory framework allowed a systematic evaluation of the factors influencing the work required to implement the tools. FINDINGS: The guidebooks were embedded in daily practice but the shared decision-making tools were not. Guidebooks were considered to enhance patient-centredness and were minimally disruptive. Practice nurses were reluctant to engage with behaviour change discussions. Self-management support was not formulated as a practice priority and there was minimal support for this activity within the practice: it was not auditable; was insufficiently differentiated from existing content and processes of work to value in its own right, and considered too disruptive and time-consuming. CONCLUSION: Supporting self-management through the encouragement of lifestyle change was problematic to realise with limited evidence of the development of the needed collaborative partnerships between patients and practitioners required by the ethos of self-management support. | | | | | OK |
| Lucinda | Colon | Patricia | Vega | Alina | Alonso | Journal of Women's Health (2002) | 2013-12-00 Dec 2013 | BACKGROUND: This study aimed to examine the effectiveness of the CenteringPregnancy group prenatal care model in improving maternal and child health outcomes, satisfaction with prenatal care, and engagement in prenatal care. METHODS: Pregnant Hispanic women ≤20 weeks gestation initiating prenatal care at two Palm Beach County, Florida, public health clinics selected CenteringPregnancy or traditional prenatal care. Data on postpartum visit compliance, establishment of a medical home, child emergency room visits, satisfaction with prenatal care, and engagement in prenatal care were obtained through surveys conducted 3 months post-delivery (n=176; 129 CenteringPregnancy, 47 traditional). Data on prenatal care visits were obtained through abstraction of Palm Beach County Health Department medical records (n=214; 144 CenteringPregnancy, 70 traditional). RESULTS: Statistically significant differences were found in satisfaction with prenatal care (84.3 Centering vs. 64.9 traditional, p<.001) and engagement in (39.7 vs. 28.1, p<.001). Centering participants had higher expected prenatal care ratios than traditional care participants (101.9 vs. 83.1, p<.001) and higher percentages receiving adequate prenatal care (90% vs. 63%, p<.001). Centering recipients were more likely to establish a medical home for their child (77% vs. 53%, p<.01) and have a postpartum checkup within six weeks of delivery (99% vs. 94%, p=.04). CONCLUSIONS: CenteringPregnancy improves engagement in prenatal care and healthcare utilization outcomes for low-income Latinas. Given disparities that exist between perinatal Latinas and women from other racial/ethnic groups, CenteringPregnancy should be considered as an alternative to traditional 1-on-1 prenatal care for this population. | Humans | Patient Participation | Patient Satisfaction | Questionnaires | OK |
| Didy E. | Jacobsen | Jan W. A. | Smit | Menko-Jan | de Boer | Nederlands Tijdschrift Voor Geneeskunde | 2013-00-00 2013 | Hospital care is highly disease-focused, tending towards further specialisation, and largely driven by technological innovations. Frail elderly, however, are an important and rapidly growing group of patients that is not best cared for in such a clinical environment. Case histories and the description of outcomes of an innovative programme called 'CareWell Primary Care' reveal that triage based on frailty and a global geriatric assessment of frail patients which is linked to goal-oriented hospital care and shared decision-making are the cornerstones of much needed improvement in delivering effective, safe, and sustainable hospital care to our ageing population. | Male | Humans | Female | Hospitalization | OK |
| P. | Reitmeir | W. | Rathmann | C. | Meisinger | Bundesgesundheitsblatt, Gesundheitsforschung, Gesundheitsschutz | 2011-11-00 Nov 2011 | To evaluate the quality of diabetes care, processes and outcomes of health care for type 2 diabetes were compared across three population-based surveys in Germany with cross-sectional and longitudinal perspectives. The surveys were conducted in the Augsburg region, southern Germany, in 1999-2001, 2003-2005, and 2006-2008 and included physical examinations, an interview, self-administered diabetes questionnaires, and laboratory tests. Quality indicators derived from guidelines for type 2 diabetes managed care programs in Germany served as the evaluation framework. Multiple regression models were used for analysis, adjusting for age, sex, education, diabetes duration, and cardiovascular comorbidity. Results show that medical examinations of eyes (61-71%) and feet (38-55%) and the use of antihypertensives, antiplatelet drugs, and lipid-lowering medications were reported more frequently over time. There was no increase in patient self-care behaviors or diabetes education. Blood pressure and cholesterol outcome targets were achieved more frequently over time. In conclusion, medical care and drug therapy of type 2 diabetes have improved; however clinical practice has failed to intensify patient participation and health behavior. | Male | Humans | Patient Participation | Female | OK |
| Oliver | Frank | Sue | Williams | Philip | Ryan | BMC family practice | 2012-00-00 2012 | BACKGROUND: Preventive health care is an important part of general practice however uptake of activities by patients is variable. Monetary incentives for doctors have been used in the UK and Australia to improve rates of screening and immunisation. Few studies have focussed on incentives for patients to attend preventive health care examinations. Our objective was to investigate the use of a monetary incentive to increase patient attendance with their general practitioner for a cardiovascular risk assessment (CVRA). METHODS: A pragmatic RCT was conducted in two Australian general practices. Participating GPs underwent academic detailing for cardiovascular risk assessment. 301 patients aged 40-74, who did not have cardiovascular disease, were independently randomised to receive a letter inviting them to a no cost cardiovascular risk assessment with their GP, or the same letter plus an offer of a $25 shopping voucher if they attended. An audit of patient medical records was also undertaken and a patient questionnaire administered to a sub sample of participants. Our main outcome measure was attendance for cardiovascular risk assessment. RESULTS: In the RCT, 56/301(18.6%) patients attended for cardiovascular risk assessment, 29/182 (15.9%) in the control group and 27/119 (22.7%) in the intervention group. The estimated difference of 6.8% (95% CI: -2.5% to 16.0%) was not statistically significant, P = 0.15. The audit showed that GPs may underestimate patients' absolute cardiovascular risk and the questionnaire that mailed invitations from GPs for a CVRA may encourage patients to attend. CONCLUSIONS: A small monetary incentive does not improve attendance for cardiovascular risk assessment. Further research should be undertaken to determine if there are other incentives that may increase attendance for preventive activities in the general practice setting. CLINICAL TRIALS REGISTRATION: ACTRN12608000183381. | Male | Humans | Patient Participation | Questionnaires | OK |
| C. | Bonner | M. L. | Bell | A. B. | Smith | British Journal of Cancer | 2014-07-08 Jul 8, 2014 | BACKGROUND: Decision aids may improve informed consent in clinical trial recruitment, but have not been evaluated in this context. This study investigated whether decision aids (DAs) can reduce decisional difficulties among women considering participation in the International Breast Cancer Intervention Study-II (IBIS-II) trial. METHODS: The IBIS-II trial investigated breast cancer prevention with anastrazole in two cohorts: women with increased risk (Prevention), and women treated for ductal carcinoma in situ (DCIS). Australia, New Zealand and United Kingdom participants were randomised to receive a DA (DA group) or standard trial consent materials (control group). Questionnaires were completed after deciding about participation in IBIS-II (post decision) and 3 months later (follow-up). RESULTS: Data from 112 Prevention and 34 DCIS participants were analysed post decision (73 DA; 73 control); 95 Prevention and 24 DCIS participants were analysed at follow-up (58 DA; 61 control). There was no effect on the primary outcome of decisional conflict. The DCIS-DA group had higher knowledge post decision, and the Prevention-DA group had lower decisional regret at follow-up. CONCLUSIONS: This was the first study to evaluate a DA in the clinical trial setting. The results suggest DAs can potentially increase knowledge and reduce decisional regret about clinical trial participation. | Decision Making | Decision Support Techniques | Humans | Patient Participation | OK |
| Suzanne | Leveille | Clementina | Dimonda | Jacob Y. | Shin | International journal for quality in health care: journal of the International Society for Quality in Health Care / ISQua | 2012-08-00 Aug 2012 | QUALITY PROBLEM: Patients often do not fully understand medical information discussed during office visits. This can result in lack of adherence to recommended treatment plans and poorer health outcomes. CHOICE OF SOLUTION: We developed and implemented a program utilizing an encounter form, which provides structure to the medical interaction and facilitates bidirectional communication and informed decision-making. IMPLEMENTATION: We conducted a prospective quality improvement intervention at a large tertiary-care academic medical center utilizing the encounter form and studied the effect on patient satisfaction, understanding and confidence in communicating with physicians. The intervention included 108 patients seen by seven physicians in five sub-specialties. EVALUATION: Ninety-eight percent of patients were extremely satisfied (77%) or somewhat satisfied (21%) with the program. Ninety-six percent of patients reported being involved in decisions about their care and treatments as well as high levels of understanding of medical information that was discussed during visit. Sixty-nine percent of patients reported that they shared the encounter form with their families and friends. Patients' self-confidence in communicating with their doctors increased from a score of 8.1 to 8.7 post-intervention (P-value = 0.0018). When comparing pre- and post-intervention experiences, only 38% of patients felt that their problems and questions were adequately addressed by other physicians' pre-intervention, compared with 94% post-intervention. LESSONS LEARNED: We introduced a program to enhance physician-patient communication and found that patients were highly satisfied, more informed and more actively involved in their care. This approach may be an easily generalizable approach to improving physician-patient communication at outpatient visits. | Male | Decision Making | Humans | Patient Participation | OK |
| Bernie | Mullin | Andrew | Old |  |  | The New Zealand Medical Journal | 2012-06-29 Jun 29, 2012 | AIM: This paper describes how co-design methods can be used to improve patient experiences and services within healthcare organisations. Using the Patient Co-design of Breast Service Project as an example, we describe how patient experiences were captured and understood, the improvements made and implications for future work. METHOD: We used a six-step process: engage, plan, explore, develop, decide and change. Tools and techniques employed were based on service design approaches. These included patient journey mapping, experience-based surveys and co-design workshops. RESULTS: Information, communication, navigation and co-ordination, and environment emerged as key themes for the Breast Service. And as a result, a suite of improvements were made. Key methodological learnings included using co-design alongside traditional quality improvement methodologies, engaging with patients early, the importance of staff buy-in and the necessity of trying things outside one's comfort zone. CONCLUSION: Use of co-design within the Breast Service has resulted in tangible improvements and has demonstrated the value of engaging patients and focussing on their experiences. It is recommended that: evaluation phases are factored into future co-design work, further research is conducted on sustainability and funding and support is given to allow co-design to become more widespread throughout New Zealand. | Humans | Patient Participation | Questionnaires | Female | OK |
| Hans J. | Kreder | Warren J. | McIsaac | Peter C. | Coyte | Spine | 2010-01-01 Jan 1, 2010 | STUDY DESIGN: Survey to all orthopedic and neurosurgeons, a random sample of family physicians (FPs) and patients in Ontario, Canada. OBJECTIVE: To identify the dominant clinical factors influencing patient and physician preferences for lumbar spinal surgery. SUMMARY OF BACKGROUND DATA: Surgery on the degenerative lumbar spine offers significant benefit for patients with moderate-severe symptoms failing nonoperative treatment. Referring FPs have little appreciation of factors that identify the ideal surgical candidate. Differences in preferences may lead to wide variation in referrals and impedes the shared decision-making process. METHODS: We used conjoint analysis, a rigorous method for eliciting preferences, to determine the importance that respondents place on decisions for lumbar spinal surgery. We identified 6 clinical factors (walking tolerance, pain duration, severity, neurologic symptoms, typical onset, and dominant location of pain) and presented hypothetical vignettes to participants who rated their preference for surgery. Data were analyzed using random-effects ordered probit regression models and the importance of each clinical factor relative to the others was determined. RESULTS: We obtained responses from 131 surgeons, 202 FPs, and 164 patients. We found that FPs had the highest overall preferences for surgery and surgeons had the lowest. Surgeons placed the highest importance on the location of pain. FPs considered neurologic symptoms, walking tolerance, and severity to be of similar importance. Pain severity, walking tolerance, and duration of pain were the most important factors for patients in deciding for surgery. Orthopedic (over neurosurgical) surgeons had a lower preference for surgery (P < 0.05). Older patients (P < 0.03) and previous surgical consultation (P < 0.03) had greater patient preferences for surgery. CONCLUSION: Different preferences for surgery exist between surgeons, FPs, and patients. FPs may reduce over- and under-referrals by appreciating surgeons' importance on location of pain (leg vs. back). Surgeons and FPs may improve the shared decision-making process by understanding that patients place high importance on quality of life symptoms. | Male | Humans | Physician-Patient Relations | Questionnaires | OK |
|  |  |  |  |  |  | Child: Care, Health and Development | 2011-07-00 Jul 2011 | BACKGROUND: With growing awareness of the need to involve children in their own health-related decisions, attention has primarily focused on the concept of assent, or a minor's participation in a research trial or experimental treatment. This study attempts to broaden that focus by examining the perceptions and practices of healthcare providers with respect to the role of children in more routine healthcare decisions and treatments. METHODS: In total, 103 nurses and 40 physicians who work in a hospital in Israel completed self-administered perceptions and practices questionnaires. RESULTS: Many participants agreed that children should be included in decision making. Factors that respondents felt would influence their approach to a particular child included child behaviour (80%), child communication (66%), experience of child, parent and healthcare provider (90%) and type of medical intervention (60%). Responses differed between physicians and nurses. In response to the question 'How often do you suggest the following methods to achieve child participation in treatment?' most respondents reported that they provide an explanation (98%) and recruit the parents (90%). The use of play was reported by only 63% of the professionals. CONCLUSION: This study demonstrated that many healthcare providers recognize the need to include children in routine health-related practices and outlined factors healthcare providers use in deciding when to include children in medical decisions. Involving children in even the minute aspects of everyday decisions and treatments can allow children to feel part of the process, improve their co-operation, increase their sense of control and affect future healthcare encounters. | Male | Decision Making | Humans | Patient Participation | OK |
| Arnaud | Perrier | Noelle Junod | Perron | Stéphane | Cochet | Journal of Medical Ethics | 2013-03-00 Mar 2013 | BACKGROUND: Decisions regarding Cardio-Pulmonary Resuscitation (CPR) and Do Not Attempt Resuscitation (DNAR) orders remain demanding, as does including patients in the process. OBJECTIVES: To explore physicians' justification for CPR/DNAR orders and decisions regarding patient inclusion, as well as their reports of how they initiated discussions with patients. METHODS: We administered a face-to-face survey to residents in charge of 206 patients including DNAR and CPR orders, with or without patient inclusion. RESULTS: Justifications were provided for 59% of DNAR orders and included severe comorbidity, patients and families' resuscitation preferences, patients' age, or poor prognosis or quality of life. Reasons to include patients in CPR/DNAR decisions were provided in 96% and 84% of cases, and were based on respect for autonomy, clinical assessment of the situation as not too severe, and the view that such inclusion was required. Reasons for not including patients were offered in 84% of cases for CPR and in 70% for DNAR. They included absent decision-making capacity, a clinical situation viewed as good (CPR) or offering little hope of recovery (DNAR), barriers to communication, or concern that discussions could be emotionally difficult or superfluous. Decisions made earlier in the patient's management were infrequently viewed as requiring revision. Residents reported a variety of introductions to discussions with patients. CONCLUSIONS: These results provide better understanding of reasons for CPR/DNAR decisions, reasons for patient inclusion or lack thereof, and ways in which such inclusion is initiated. They also point to potential side-effects of implementing CPR/DNAR recommendations without in-depth and practical training. This should be part of a regular audit and follow-up process for such recommendations. | Decision Making | Humans | Patient Participation | Questionnaires | OK |
| Allyson K. | Thomson | Lin | Fritschi | Jen | Girschik | Journal of Clinical Epidemiology | 2012-06-00 Jun 2012 | OBJECTIVE: The Breast Cancer Environment and Employment Study (BCEES) is a case--control study that began in 2009. The study experienced a lower than expected response fraction, a trend that appears to be occurring internationally. A 32-page questionnaire was included in the initial invitation to participate, and previous research suggests that long questionnaires decrease response fractions. The aim of this study was to test whether removal of the questionnaire from the invitation package increased participation. STUDY DESIGN AND SETTING: A randomized controlled trial was undertaken among the BCEES controls from June to August 2010. One group of 250 received the questionnaire in the initial invitation package, and the other group of 250 received only the invitation package and was sent the questionnaire after their consent was received. The proportion of responses for the two groups was compared using contingency tables and chi-square statistics. RESULTS: Those who received the questionnaire with the invitation package were more likely to consent to participate than those who did not (40.8% and 33.2%, respectively). However, this difference was not statistically significant (P=0.078). CONCLUSION: To improve response fractions and reduce time in following up nonresponses, questionnaires should be included in the invitation package. | Humans | Patient Participation | Questionnaires | Female | OK |
| George | Divine | Susan | Flocke | Nancy | Oja-Tebbe | Patient Education and Counseling | 2010-09-00 Sep 2010 | OBJECTIVE: To compare patient-reported and observer-rated shared decision making (SDM) use for colorectal cancer (CRC) screening and evaluate patient, physician and patient-reported relational communication factors associated with patient-reported use of shared CRC screening decisions. METHODS: Study physicians are salaried primary care providers. Patients are insured, aged 50-80 and due for CRC screening. Audio-recordings from 363 primary care visits were observer-coded for elements of SDM. A post-visit patient survey assessed patient-reported decision-making processes and relational communication during visit. Association of patient-reported SDM with observer-rated elements of SDM, as well as patient, physician and relational communication factors were evaluated using generalized estimating equations. RESULTS: 70% of patients preferred SDM for preventive health decisions, 47% of patients reported use of a SDM process, and only one of the screening discussions included all four elements of SDM per observer ratings. Patient report of SDM use was not associated with observer-rated elements of SDM, but was significantly associated with female physician gender and patient-reported relational communication. CONCLUSION: Inconsistencies exist between patient reports and observer ratings of SDM for CRC screening. PRACTICE IMPLICATIONS: Future studies are needed to understand whether SDM that is patient-reported, observer-rated or both are associated with informed and value-concordant CRC screening decisions. | Male | Decision Making | Humans | Patient Participation | OK |
| Antony S. R. | Manstead | Malcolm W. R. | Reed | Lisa | Caldon | Patient Education and Counseling | 2012-08-00 Aug 2012 | OBJECTIVES: To undertake a quantitative evaluation of a theory-based, interactive online decision aid (BresDex) to support women choosing surgery for early breast cancer (Stage I and II), based on observations of its use in practice. METHODS: Observational cohort study. Website log-files collected data on the use of BresDex. Online questionnaires assessed knowledge about breast cancer and treatment options, degree to which women were deliberating about their options, and surgery intentions, pre- and post-BresDex. RESULTS: Readiness to make a decision significantly increased after using BresDex (p<.001), although there was no significant improvement in knowledge. Participants that were 'less ready' to make a decision before using BresDex, spent a longer time using BresDex (p<.05). Significant associations between surgery intentions and choices were observed (p<.001), with the majority of participants going on to have BCS. Greater length of time spent on BresDex was associated with stronger intentions to have BCS (p<.05). CONCLUSION: The use of BresDex appears to facilitate readiness to make a decision for surgery, helping to strengthen surgery intentions. PRACTICE IMPLICATIONS: BresDex may prove a useful adjunct to the support provided by the clinical team for women facing surgery for early breast cancer. | Decision Making | Decision Support Techniques | Humans | Patient Participation | OK |
| Irene | Korstjens | Luc | Budé | Toine L. M. | Lagro-Janssen | Midwifery | 2013-11-00 Nov 2013 | OBJECTIVE: to explore whether choices in birthing positions contributes to women's sense of control during birth. DESIGN: survey using a self-report questionnaire. Multiple regression analyses were used to investigate which factors associated with choices in birthing positions affected women's sense of control. SETTING: midwifery practices in the Netherlands. PARTICIPANTS: 1030 women with a physiological pregnancy and birth from 54 midwifery practices. FINDINGS: in the total group of women (n=1030) significant predictors for sense of control were: influence on birthing positions (self or self together with others), attendance of antenatal classes, feelings towards birth in pregnancy and pain in second stage of labour. For women who preferred other than supine birthing positions (n=204) significant predictors were: influence on birthing positions (self or self together with others), feelings towards birth in pregnancy, pain in second stage of labour and having a home birth. For these women, influence on birthing positions in combination with others had a greater effect on their sense of control than having an influence on their birthing positions just by themselves. KEY CONCLUSIONS: women felt more in control during birth if they experienced an influence on birthing positions. For women preferring other than supine positions, home birth and shared decision-making had added value. IMPLICATIONS FOR PRACTICE: midwives can play an important role in supporting women in their use of different birthing positions and help them find the positions they feel most comfortable in. Thus, contributing to women's positive experience of birth. | Decision Making | Humans | Questionnaires | Female | OK |
| Marie-Annick | Le Pogam | Sophie | Figon | Laurent | Letrilliart | Santé Publique (Vandoeuvre-Lès-Nancy, France) | 2013-04-00 2013 Mar-Apr | AIM: Antihypertensive drugs are thought to be responsible for adverse drug events in 25% of patients, with severe consequences in 13% of cases. The purpose of this study was to develop an interactive booklet designed to inform and involve patients with a view to preventing adverse drug events. METHODS: The development of the booklet involved several stages, including a literature review, a Delphi survey, a readability assessment, a qualitative study in primary care, a revision process, and graphic design work. 27 experts (patients, general practitioners, public health practitioners, cardiologists, geriatricians, psychologists, economists, pharmacists, nurses and ethicists) participated in the Delphi survey, while the qualitative study was based on a sample that included 7 doctors, 13 patients and 2 healthcare assistants. RESULTS: We developed an interactive booklet containing information items on the benefits and risks of antihypertensive drugs, a care plan to be completed by the patient, and a form for reporting adverse drug events. Ambiguous sentences and incomprehensible medical terms were rephrased. The time required to present the booklet and ease of use were key acceptability criteria for caregivers. Among the patients, the study found that interest in the booklet required clear evidence of an expected benefit. CONCLUSION: An understandable and acceptable interactive booklet was developed using a systematic process to prevent severe adverse drug events in primary care. | Male | Humans | Patient Participation | Female | OK |
| Fritz A. | Muthny |  |  |  |  | Psychiatrische Praxis | 2011-07-00 Jul 2011 | OBJECTIVE: Main goal was to assess the wish for participation of patients in psychosomatic rehabilitation and their perception of medical information and own treatment involvement. Further, the influence of these constructs on patient satisfaction was investigated. METHODS: 276 patients were included in a cross-sectional survey at the end of their inpatient stay. RESULTS: Patients showed a strong need for participation in this sample. Socio-demographic and disease-related parameters had no or only weak associations to the shared decision making variables. Strong correlations to patient satisfaction were found for doctor facilitation of patient involvement (r = 0.57) and transfer of treatment relevant information (r = 0.52). CONCLUSIONS: The doctor's behaviour concerning information transfer and involvement of patients in their treatment proved to be an important factor for patient satisfaction. This underlines the importance of active and need-oriented information in the sense of "empowerment". | Male | Humans | Patient Participation | Patient Satisfaction | OK |
| Marcela | González | Luis | Villarroel | Henrique Afonseca | Parsons | Revista Médica De Chile | 2014-01-00 Jan 2014 | Information disclosure and decision making process are important steps in advanced cancer patients management; however, there is no research done in this area in Chile. AIMS: To know the preferences of patients with advanced cancer related to information disclosure and style of decision making process. METHODS: Prospective observational study with patients in the Palliative Care Unit of Sótero del Río Hospital, in Santiago, Chile. The preferences were evaluated with a Disclosure Information and a Decision Making Preferences Questionnaire. RESULTS: 100 patients were recruited, 52% males, average age 63 years; 90% wanted to receive complete information about diagnosis and 89% complete information about prognosis. The preferences related to decision making process style were: 60% shared, 27% passive and 13% active. The expressed satisfaction with the information received was 89% and 87% with the way decisions were actually made. CONCLUSIONS: A majority of patients preferred to receive complete information about diagnosis and prognosis and to make shared decisions. The satisfaction with information disclosure and decision making process was very high. The data of this study supports the need of an adequate information disclosure and of exploring the individual preferences of our patients, with the goal of promoting an informed decision making process that respects the preferences of our patients. | Male | Decision Making | Humans | Patient Participation | OK |
| Mihaela | Mates | Christine | Tong | Eva | Grunfeld | Breast (Edinburgh, Scotland) | 2013-10-00 Oct 2013 | PURPOSE: To identify the information that post-menopausal women with hormone-receptor positive, early-stage breast cancer want, to help them decide among six treatment options for adjuvant-endocrine therapy. METHODS: We surveyed women with early-stage breast cancer who were eligible for adjuvant endocrine-therapy 3-18 months earlier. Participants rated the importance of getting each of 95 questions answered before this decision is made (options: essential/desired/not important/avoid). For questions rated essential or desired, participants identified the purpose(s) for having the question answered: to help them understand, make the decision, plan, or other. Participants indicated the role they played in their actual decision and the role they would prefer if the decision was made today. They also indicated whether they felt they had had a choice of endocrine therapy treatments. RESULTS: 188 of 343 questionnaires were returned (response rate 55%). Mean age was 67 yr (range 38-88 yr); 76% were married, and 39% had secondary school education or less. On average, respondents rated 18 questions (range 0-94) essential for decision making. Each question was rated essential for decision making by ≥ 7% of participants but only 1 question by >50%. Regarding roles, 89% of respondents had participated in their actual decision and would want to again; an additional 9% had not participated in their actual decision but would want to at the time of the survey. The percentage of respondents who felt they had no choice of endocrine therapy treatments varied between centres, 25% vs 41% and 49%. CONCLUSIONS: Most patients want to participate in the decision but they vary widely in the amount and which specific details they want to help them make the decision. IMPLICATION: The wide variation in questions considered important means the support should be tailored to the needs of the individual patient. | Decision Making | Humans | Patient Participation | Questionnaires | OK |
| Margaret | Rees | Tim | Jack |  |  | Psychology & Health | 2013-00-00 2013 | OBJECTIVES: The aim of this study is to improve our understanding of the ways in which a medical consultation style relates to satisfaction and adherence. DESIGN: Participants completed questionnaires about preferred and perceived consultation styles; psychological variables such as satisfaction, anxiety and depression; and questions about taking medication. Questionnaires were completed prior to the consultation, immediately after, three months later and one year later. Doctors also completed a scale on completion of the consultation. The decisions in question related to women seeking advice about Hormone Replacement Therapy (HRT) and decisions about pain management. RESULTS: Patients expressed preferences for information about the treatment options and for active involvement in the decision process. The extent to which patients perceived themselves as having received information about the treatment options was a particularly consistent predictor of satisfaction. The extent to which patients perceived themselves as having been prepared for the side effects of HRT was a consistent predictor of anxiety. CONCLUSIONS: Perceptions of the amount of information received about medical options, including information preparing individuals for potential side effects of medication, appears to be more important to satisfaction and anxiety, than actual involvement in the decision. | Humans | Patient Participation | Patient Satisfaction | Questionnaires | OK |
| A. | Hanish | V. A. | Johnson | L. L. | Shah | Clinical Genetics | 2013-07-00 Jul 2013 | The potential for genomic incidental findings is increasing with the use of genome-based testing. At the same time approaches to clinical decision making are shifting to shared decision-making models involving both the healthcare community and the public. The public's voice has been nearly absent in discussions on managing incidental findings. We conducted nine focus groups and nine interviews (n = 63) with a broad cross-section of lay public groups to elucidate public viewpoints on incidental findings that could occur as a result of genome-based testing in clinical and research situations. Data were analyzed using qualitative content analysis. Participants wanted incidental findings disclosed to them whether or not these were clinical or research findings. Participants used different terms to define and describe incidental findings; they wanted to know that incidental findings are possible and be given a choice to learn about them. Personal utility was an important reason for disclosure, and participants believed that managing information is a shared responsibility between professionals and themselves. Broad public input is needed in order to understand and incorporate the public's perspective on management of incidental findings as disclosure guidelines, and policies are developed in clinical and research settings. | Male | Decision Making | Humans | Questionnaires | OK |
| Susanne | Fischer | Deb | Feldman-Stewart | Robin | Ruszat | Supportive Care in Cancer: Official Journal of the Multinational Association of Supportive Care in Cancer | 2014-04-00 Apr 2014 | PURPOSE: The aims of this study were to analyze agreement on information needs within a group of early-state prostate cancer patients and to compare information preferences of patients with the view of health-care professionals about patients' needs. METHODS: Sample consists of patients (n = 128) and six subgroups of health-care professionals (urologists, n = 32; nurses, n = 95; radiotherapy technologists (RTTs), n = 36; medical oncologists, n = 19; radiation oncologists, n = 12; general practitioners (GPs), n = 10). Information needs have been assessed with 92 questions concerning prostate cancer and its treatment. Respondents judged the importance of addressing each question. Within- and between-group agreements of patients and health-care professional groups were estimated with raw agreement indices as well as chance-corrected Kappa and Gwet's AC1 measures. Finally, group-specific core items rated with high importance as well as high agreement were defined. RESULTS: Patients rated on average (median) half, i.e., 51 out 92 items as essential (interquartile range (IQR) = 36-66), 26 items as desired (IQR = 14-38), and 10 items as avoidable (IQR = 2-22). Within-group agreement on the presented information topics is modest for any participating group (AC1(patients)= 0.319; AC1(professionals) = 0.295-0.398). Agreement between patients and professionals is low too (AC1 = 0.282-0.329). Defining group-specific core sets of information topics results in 51 items being part of at least one core set. Concordance of the item core sets of patients and professionals is moderate with κ = 0.38-0.66, sensitivity of professionals' core sets for patients' preferences varies between 56 and 74%. CONCLUSIONS: Results emphasize the need for dialogue between doctor/professional and patient in identifying the information needed by individual patients and support the importance of shared decision making. | Male | Decision Making | Humans | Questionnaires | OK |
| R. | Gügler | S. | Fischer | H.-P. | Schmid | Der Urologe. Ausg. A | 2011-09-00 Sep 2011 | BACKGROUND: In the context of shared decision-making it is important to know more about information needs of prostate cancer patients. Therefore, this study investigates content and extent of these information needs. MATERIAL AND METHODS: We surveyed 330 patients of 4 urological Clinics in Switzerland with a well-established written questionnaire between 3 and 24 months after their diagnosis of an early-stage prostate cancer. A total of 179 patients responded and 128 (39%) questionnaires were entered into final statistical analysis. RESULTS: Patients expressed broad information needs and pronounced differences between individuals were observed. Each of the 92 questions presented was rated as "essential" by at least 18% of the patients. On average 50 questions were rated as 'essential'. One patient rated only four questions as 'essential' whereas another participant reported all 92 questions as being 'essential' to him. CONCLUSIONS: Concerning patient education it is important to identify the individual information needs of each prostate cancer patient separately. | Male | Decision Making | Humans | Questionnaires | OK |
| Mihaela | Mates | Christine | Tong | Eva | Grunfeld | Patient Education and Counseling | 2013-10-00 Oct 2013 | OBJECTIVE: To identify questions that post-menopausal women with receptor-positive early-stage breast cancer want answered before their adjuvant-endocrine-therapy decision is made. METHODS: We surveyed patients eligible for adjuvant-endocrine therapy in the previous 3-18 months. Participants rated the importance of getting each of 95 questions answered before the decision is made (options: essential/desired/not important or no opinion/avoid). For each question rated "essential"/"desired", the participant also identified the purpose(s) for the answer: to help her understand, decide, plan, or other reason(s). RESULTS: The response rate was 55% (188/343). Participants rated a mean of 57 (range: 1-95) questions "essential", 80 (range: 1-95) "essential" or "desired", and 2 (range: 0-27) "avoid". Every question was "essential" to ≥31% of participants, and "essential"/"desired" to ≥63%. All but eleven questions were rated as "avoid" by ≥1 participant. The most frequent purposes for "essential" questions were to: understand their situations (mean 45, range: 0-95), decide (mean 18, range: 0-94), and plan (mean 13, range: 0-95). CONCLUSION: Many patients want a lot of information before this decision is made but there is wide variation within the group in both the number and in which questions they want answered. PRACTICE IMPLICATIONS: Patient education in this setting needs to be tailored to the needs of the individual patient. | Decision Making | Humans | Patient Participation | Questionnaires | Doublon |
| S. | Volz | H. S. | Rugo |  |  | Journal of Cancer Education: The Official Journal of the American Association for Cancer Education | 2014-03-00 Mar 2014 | In the setting of breast oncology consultations, we sought to understand communication patterns between patients with advanced breast cancer and their oncologists during visits with Decision Support Services. This is a descriptive study analyzing themes and their frequencies of premeditated question lists of patients with metastatic breast cancer. We identified topics physicians most commonly discussed among themes previously found, documenting questions patients with metastatic breast cancer prepare for physician consultations and oncologists' response. Inclusion criteria were as follows: diagnosis of metastatic breast cancer, completion of a question list before meeting with an oncologist, and receipt of a summary of the consultation. We identified 59 women with metastatic breast cancer who received both documents. We reviewed the question lists and consultation summaries of these patients. Of the 59 patients whose documents we reviewed, patients most often asked about prognosis (38), symptom management (31), clinical trials (43), and quality of life (38). Physicians answered questions about prognosis infrequently (37% of the time); other questions that were answered more than commonly are the following: symptom management (81%), clinical trials (79%), and quality of life (66%). Breast cancer patients have many questions regarding their disease, its treatment, and symptoms, which were facilitated in this setting by Decision Support Services. Question lists may be insufficient to bridge the divide between physicians and patient information needs in the setting of metastatic breast cancer, particularly regarding prognosis. Patients may need additional assistance defining question lists, and physicians may benefit from training in communication, particularly regarding discussions of prognosis and end of life. | Male | Decision Making | Humans | Patient Participation | OK |
| David | Satin | Lindsay | Schuster |  |  | Community Dentistry and Oral Epidemiology | 2011-02-00 Feb 2011 | OBJECTIVES: When it comes to their own health care, adult patients traditionally demonstrate strong information-seeking desire but a somewhat lower desire to make their own treatment decisions in nonelective situations. Little is known about these desires in patients facing elective health care situations. We used the well-tested Autonomy Preferences Index (API) as a base to construct and test our elective Autonomy Preferences Index (eAPI) for both information-seeking and decision-making and analyzed demographic variables on both. METHODS: The eAPI was constructed to mirror the API but uses elective scenarios rather than the API's nonelective scenarios. It was validated using cognitive interviews to determine item intent and comprehension and by Cronbach's alpha. Both the API and eAPI were distributed to 188 active-treatment patients at the Division of Orthodontics, University of Minnesota. API and eAPI items were scored using a 1 (low) to 5 (high) Likert scale of desire. RESULTS: Mean information-seeking desire was universally high (>4, P < 0.001) for both API and eAPI instruments. Mean decision-making (DM) desire was universally low to moderate: API-DM = 2.84 and eAPI-DM = 2.6. Decision-making preferences for nonelective items (API-DM) decreased as the condition severity presented in the vignettes increased: mild = 2.88, moderate = 2.67 and severe = 2.21. Conversely, elective decision-making preferences (eAPI-DM) increased with increasing condition severity: mild = 2.51, moderate = 2.79 and severe = 3.18 (P < 0.001). CONCLUSIONS: Adult patients have universally high information-seeking preferences and moderate to low decision-making preferences regardless of the elective or nonelective nature of their condition. However, as vignette condition severity increases, patients facing nonelective scenarios display progressively less desire for decision-making, whereas patients facing elective scenarios show progressively more decision-making desire. | Male | Decision Making | Humans | Patient Participation | OK |
| Leigh | Jackson | Heather | Skirton |  |  | Journal of Advanced Nursing | 2013-09-00 Sep 2013 | AIM: This article is a report of a study of informed consent in people with a learning disability. The aims of the study were to explore the information needs of people with mild-to-moderate learning disabilities with respect to consent for blood tests and to identify ways of facilitating informed consent. BACKGROUND: The recent political agenda for social change in the UK has emphasized the right of people with a learning disability to have more autonomy and make their own decisions. As in other countries, there has also been a shift towards shared decision-making in healthcare practice. DESIGN: Qualitative study using an ethnographic approach. METHODS: An ethnographic approach was used for this qualitative study. Phase 1 involved observation of six participants with a learning disability having a routine blood test in general practice, followed by semi-structured interviews with 14 participants with a learning disability in Phase 2. Data were collected between February 2009-February 2010. FINDINGS: The data showed that consent procedures were often inadequate and provision of information to patients prior to a blood test was variable. People with a learning disability expressed clearly their information requirements when having a routine blood test; this included not wanting any information in some cases. CONCLUSIONS: Healthcare practitioners and people with a learning disability need to be familiar with current consent law in their own country to facilitate valid consent in the healthcare context. This study demonstrated the value of qualitative research in exploring the knowledge and attitudes of people with learning disability. | Humans | Informed Consent | Hematologic Tests | Learning Disorders | OK |
| Paul | Legutko |  |  |  |  | Journal of Cataract and Refractive Surgery | 2012-01-00 Jan 2012 | PURPOSE: To determine the effectiveness of verbal, written, and videotaped descriptions of cataract surgery on patients' understanding of the risks, benefits, and treatment alternatives. SETTING: Veterans Affairs Boston Healthcare System, Boston, Massachusetts, USA. DESIGN: Randomized prospective study. METHODS: Patients eligible for cataract surgery were randomized into 1 of 4 arms of the informed consent process as follows: Group 1, conventional verbal information; Group 2, conventional verbal information plus second-grade reading level brochure; Group 3, conventional verbal information plus eighth-grade reading level brochure; Group 4, conventional verbal information plus American Academy of Ophthalmology DVD Understanding Cataract Surgery: Patient Education DVD Featuring an Aid to Informed Consent. After the informed consent process, patients in each group completed a 12-question questionnaire discussing the surgical procedure, its benefits, its foreseeable and unforeseeable risks, and the alternatives to cataract surgery. Scores in each group were calculated as the number of correct responses out of 12 questions. RESULTS: Patients in Group 2 (mean score 10.8 ± 1.29 [SD]) and Group 4 (mean score 10.56 ± 1.44) scored significantly higher than patients in Group 1 (mean score 7.68 ± 2.80) or Group 3 (mean score 9.08 ± 1.60). Previous cataract surgery and education level had no significant influence on recall of the informed-consent process. CONCLUSIONS: Concise informed consent information sheets at lower reading grade levels and videotape presentation optimized patient understanding of the risks, benefits, and treatment alternatives to cataract surgery. The cost-benefit of these results is important because better patient understanding has the potential to decrease the risk for indemnity payments awarded because of inadequate informed consent. | Male | Humans | Patient Participation | Questionnaires | OK |
| Megan | Whitehead | Benjamin H. | Levi | Michael J. | Green | Supportive Care in Cancer: Official Journal of the Multinational Association of Supportive Care in Cancer | 2013-02-00 Feb 2013 | PURPOSE: This study aimed to determine the extent to which patients with advanced cancer agree with their physicians regarding their cancer diagnoses prior to engaging in advance care planning (ACP) and whether variables such as age and level of education correlate with the degree of patient-physician concordance. METHODS: One hundred and fifty patients with a diagnosis of cancer and an estimated life expectancy of 18 months or less completed questionnaires about their cancer diagnoses prior to creating an advance directive. A review of the patients' medical records was performed and the physician-designated diagnosis was identified for each patient. Patient-physician agreement on diagnosis was coded based on predetermined study criteria. Concordance rates were expressed in percentages. RESULTS: The majority of patients (62.2 %) were in exact agreement with their physicians; 24.3 % were in partial agreement with the patient missing part of the diagnosis, and 9.5 % were in partial agreement with the physician missing part of the diagnosis; 4.1 % did not agree with their physicians on diagnosis. Age and education level did not correlate with patient-physician concordance rates. CONCLUSIONS: The majority of patients with advanced cancer correctly identified their cancer diagnosis. However, almost 40 % were not in full agreement with their physicians regarding diagnosis, a situation that has bearing on efforts to engage in meaningful shared decision making as well as advance care planning. | Decision Making | Humans | Physician-Patient Relations | Aged | OK |
| Christine | Edwards | Mike | Lucock | Stephen | Miller | BMC health services research | 2012-00-00 2012 | BACKGROUND: Supporting self-care is being explored across health care systems internationally as an approach to improving care for long term conditions in the context of ageing populations and economic constraint. UK health policy advocates a range of approaches to supporting self-care, including the application of generic self-management type programmes across conditions. Within mental health, the scope of self-care remains poorly conceptualised and the existing evidence base for supporting self-care is correspondingly disparate. This paper aims to inform the development of support for self-care in mental health by considering how generic self-care policy guidance is implemented in the context of services supporting people with severe, long term mental health problems. METHODS: A mixed method study was undertaken comprising standardised psychosocial measures, questionnaires about health service use and qualitative interviews with 120 new referrals to three contrasting community based initiatives supporting self-care for severe, long term mental health problems, repeated nine months later. A framework approach was taken to qualitative analysis, an exploratory statistical analysis sought to identify possible associations between a range of independent variables and self-care outcomes, and a narrative synthesis brought these analyses together. RESULTS: Participants reported improvement in self-care outcomes (e.g. greater empowerment; less use of Accident and Emergency services). These changes were not associated with level of engagement with self-care support. Level of engagement was associated with positive collaboration with support staff. Qualitative data described the value of different models of supporting self-care and considered challenges. Synthesis of analyses suggested that timing support for self-care, giving service users control over when and how they accessed support, quality of service user-staff relationships and decision making around medication are important issues in supporting self-care in mental health. CONCLUSIONS: Service delivery components - e.g. peer support groups, personal planning - advocated in generic self-care policy have value when implemented in a mental health context. Support for self-care in mental health should focus on core, mental health specific qualities; issues of control, enabling staff-service user relationships and shared decision making. The broad empirical basis of our research indicates the wider relevance of our findings across mental health settings. | Male | Humans | Questionnaires | Female | OK |
|  |  |  |  |  |  | Asian Pacific journal of cancer prevention: APJCP | 2011-00-00 2011 | AIM: The aim of the present study is to investigate the perceived inhibiting and facilitating factors concerning breast cancer early diagnosis behavior in women over age 40. METHOD: A qualitative focus group interview method was applied with 43 participating women, in the period between March-April 2010, using a semi-structured interview questionnaire based on the Health Belief Model and the Health Development Model. Content analysis was used to analyse study data. RESULTS: Inhibiting factors such as women' s lack of knowledge regarding breast cancer and early detection behaviors, lack of health insurance and transportation facilities, financial difficulties, difficulty to make an appointment, scarcity of female doctors, feeling of embarrassment, lack of awareness-negligence, forgetting, feeling of fear, and a fatalistic approach were frequently discussed. Among facilitating factors, informed level, concerned and tolerant health care personnel, free health services, free transportation to hospital, improved appointment system, telephone reminders were included. CONCLUSION AND RECOMMENDATIONS: Focus group interviews were found to be effective in determining inhibiting and facilitating factors concerning breast cancer early diagnosis behavior. National and regional training programs configured in accordance with the data obtained in the study may be effective in the implementation and maintenance of early diagnosis. | Humans | Patient Participation | Questionnaires | Female | OK |
| Leslie | Swartz |  |  |  |  | AIDS care | 2010-04-00 Apr 2010 | A psychometric scale assessing inhibitors and facilitators of willingness to participate (WTP) in an HIV vaccine trial has not yet been developed. This study aimed to construct and derive the exploratory factor structure of such a scale. The 35-item Inhibitors and Facilitators of Willingness to Participate Scale (WPS) was developed and administered to a convenience sample of 264 Black females between the ages of 16 and 49 years living in an urban-informal settlement near Cape Town. The subscales of the WPS demonstrated good internal consistency with Cronbach's alpha coefficients ranging between 0.69 and 0.82. A principal components exploratory factor analysis revealed the presence of five latent factors. The factors, which accounted for 45.93% of the variance in WTP, were (1) personal costs, (2) safety and convenience, (3) stigmatisation, (4) personal gains and (5) social approval and trust. Against the backdrop of the study limitations, these results provide initial support for the reliability and construct validity of the WPS among the most eligible trial participants in the Western Cape of South Africa. | Humans | Patient Participation | Questionnaires | Young Adult | OK |
| O. | Rixe | M. | Batt | A. L. | Brocvielle | Bulletin Du Cancer | 2010-10-00 Oct 2010 | During the initial phase of management, the caregivers' role is particularly difficult. These two consecutive surveys have been conducted to cover three main aspects: 1) How the initial management took place; 2) What the perceived deficits were; 3) What improvements could be made. A self administered and anonymous questionnaire was given to the patients by physicians. Surveys were conducted in numerous institutions representative of all kinds of practice except for Anticancer Centres. Two thousand five hundred and eighty three adult patients have completed the questionnaire (1366 and 1217 respectively in the first and subsequent survey): women (55%), age under 70 years (76%), breast cancer (32%). Results were rather encouraging. About sixty per cent of the patients are entirely satisfied by the given information and 95% are confident with the department of care. The mean level of global aid is 8.2/10 in the first survey and 8.6/10 in the second one. However, improvements remain needed, particularly for the 8% dissatisfied patients. In spite of the classical bias for these studies, this work gives several concrete responses for improving initial management, particularly for the first consultation in the centre, which has a major impact on the patient satisfaction. | Male | Humans | Patient Participation | Patient Satisfaction | OK |
|  |  |  |  |  |  | Expert Review of Molecular Diagnostics | 2011-01-00 Jan 2011 | | Humans | Patient Participation | Genome-Wide Association Study | Genetic Testing | OK |
|  |  |  |  |  |  | Computers in Biology and Medicine | 2013-05-00 May 2013 | The purpose of this research is to explore people's intention to use telecare and evaluate the degree of influence that behavioral factors on individuals' acceptance of telecare. The subjects are residents of Taiwan. The Structural Equation Modeling (SEM) technique was used to analyze the forecasting model applied to telecare. The results show that this model has good explanatory power for the behavioral intentions of using telecare. Among factors influencing the behavioral intentions of using telecare, the strongest factor is attitude, followed by the perceived usefulness, the perceived ease of use, subjective norms, and personal innovativeness, respectively. The findings of this study may serve as a reference to future studies. | Male | Humans | Patient Participation | Questionnaires | OK |
| Knud | Knudsen |  |  |  |  | The International Journal of Social Psychiatry | 2011-11-00 Nov 2011 | BACKGROUND: For two decades, mental health services in Norway have focused on service user involvement. Despite this, there is little knowledge about how inpatient mental health services have promoted user involvement. AIM: To examine service providers' reports of service user involvement at the individual and departmental levels in Norwegian community mental health centres (CMHCs). METHODS: One hundred and eighty six (186) inpatient service providers in five CMHCs filled out a questionnaire. RESULTS: Confirmatory factor analysis showed that service provider perceptions and awareness of service user involvement can be measured by three subscales: organizational user involvement; patient collaboration; and assisted patient involvement. Little user involvement was reported at the departmental level. Providers more often reported user involvement at the individual level. Providers in two of the CMHCs reported a somewhat higher degree of departmental-level user involvement. There were no significant differences across gender, age, leader position, profession and job tenure, though evening/night shift workers reported a lesser degree of user involvement. CONCLUSION: The results suggest that user involvement at the departmental level needs to be addressed in future efforts to promote user involvement in CMHCs. | Male | Humans | Patient Participation | Questionnaires | OK |
|  |  |  |  |  |  | Circulation. Heart Failure | 2011-11-00 Nov 2011 | | Humans | Patient Participation | Heart Failure | self report | OK |
| Kirsten J. | McCaffery |  |  |  |  | Journal of Health Psychology | 2013-08-00 août 2013 | This article explores the concept and measurement of health literacy in the context of shared health decision-making. It draws upon a series of qualitative and quantitative studies undertaken in the development and evaluation of a bowel cancer screening decision aid for low literacy populations. The findings indicate that different types of health literacy (functional, interactive and critical) are required in decision-making and present a set of instruments to assess and discriminate between higher level health literacy skills required for engagement in decision-making. It concludes that greater sophistication in both the definition and measurement of health literacy in research is needed. (PsycINFO Database Record (c) 2013 APA, all rights reserved). (journal abstract) | Measurement | Decision Making | health literacy concept & measurement | low literacy populations | Doublon |
| Kirsten J. | McCaffery |  |  |  |  | Journal of Health Psychology | 2013-08-00 Aug 2013 | This article explores the concept and measurement of health literacy in the context of shared health decision-making. It draws upon a series of qualitative and quantitative studies undertaken in the development and evaluation of a bowel cancer screening decision aid for low literacy populations. The findings indicate that different types of health literacy (functional, interactive and critical) are required in decision-making and present a set of instruments to assess and discriminate between higher level health literacy skills required for engagement in decision-making. It concludes that greater sophistication in both the definition and measurement of health literacy in research is needed. | Decision Making | Humans | Health Literacy | Early Detection of Cancer | OK |
| J. | Geissler | C. H. | Gleiter | F. J. | Prott | European Journal of Cancer Care | 2014-03-00 mars 2014 | Patient-reported outcomes are an important tool in clinical research. In the setting of cancer treatments, benefit of therapy is essentially characterised by improvement of survival as well as quality of life (QoL). A standardised instrument to assess QoL is the standardised QoL questionnaire of the European Organisation for Research and Treatment (EORTC QLQ-C30 questionnaire). QoL instruments provide data on different aspects (domains) of the framework of QoL. Using these questionnaires in studies provides data on how a treatment affects QoL in a group of patients. The goal of our concept is to individualise QoL and to use validated instruments in order to integrate patients’ perspectives and aims into treatment assessment, planning and control. We propose to use the domains of the EORTC QLQ-C30 and to ask the patient to determine which objectives besides survival are relevant for him and should be achieved by treatment. These individual goals can be used in a process of shared decision-making to choose and monitor treatment. In clinical studies, this approach would allow to recruit more patients who would most probably benefit from the therapy. In addition, supportive data could be gathered in correlation to treatment goals and actual benefits. (PsycINFO Database Record (c) 2014 APA, all rights reserved). (journal abstract) | Decision Making | Questionnaires | Psychometrics | Treatment | OK |
|  |  |  |  |  |  | Expert Review of Pharmacoeconomics & Outcomes Research | 2010-08-00 Aug 2010 | | Humans | Patient Participation | self report | health policy | OK |
| Fabian | Volz | Jeannette G. | van Manen | Beate | Wiegard | International Journal of Technology Assessment in Health Care | 2011-10-00 Oct 2011 | BACKGROUND: Patient involvement is widely acknowledged to be a valuable component in health technology assessment (HTA) and healthcare decision making. However, quantitative approaches to ascertain patients' preferences for treatment endpoints are not yet established. The objective of this study is to introduce the analytic hierarchy process (AHP) as a preference elicitation method in HTA. Based on a systematic literature review on the use of AHP in health care in 2009, the German Institute for Quality and Efficiency in Health Care (IQWiG) initiated an AHP study related to its HTA work in 2010. METHODS: The AHP study included two AHP workshops, one with twelve patients and one with seven healthcare professionals. In these workshops, both patients and professionals rated their preferences with respect to the importance of different endpoints of antidepressant treatment by a pairwise comparison of individual endpoints. These comparisons were performed and evaluated by the AHP method and relative weights were generated for each endpoint. RESULTS: The AHP study indicates that AHP is a well-structured technique whose cognitive demands were well handled by patients and professionals. The two groups rated some of the included endpoints of antidepressant treatment differently. For both groups, however, the same six of the eleven endpoints analyzed accounted for more than 80 percent of the total weight. CONCLUSIONS: AHP can be used in HTA to give a quantitative dimension to patients' preferences for treatment endpoints. Preference elicitation could provide important information at various stages of HTA and challenge opinions on the importance of endpoints. | Decision Support Techniques | Humans | Patient Participation | quality of life | OK |
| Shari L. | Hutchison | Nancy | Parrotta | James M. | Schuster | Psychiatric Rehabilitation Journal | 2013-12-00 Dec 2013 | OBJECTIVE: Illness management and recovery strategies are considered evidence-based practices. The article describes how a web-based application, CommonGround, has been used to support implementation of such strategies in outpatient mental health services and assess its impact. The specific focus of this article is Personal Medicine, self-management strategies that are a salient component of the CommonGround intervention. METHOD: With support from counties and a not-for-profit managed care organization, CommonGround has been introduced in 10 medication clinics, one Assertive Community Treatment (ACT) team, and one peer support center across Pennsylvania. Methods include analysis of data from the application's database and evaluation of health functioning, symptoms, and progress toward recovery. RESULTS: Health functioning improved over time and use of self-management strategies was associated with fewer concerns about medication side effects, fewer concerns about the impact of mental health medicine on physical health, more reports that mental health medicines were helping, and greater progress in individuals' recovery. CONCLUSIONS AND IMPLICATIONS FOR PRACTICE: Using Personal Medicine empowers individuals to work with their prescribers to find a "right balance" between what they do to be well and what they take to be well. This program helps individuals and their service team focus on individual strengths and resilient self-care strategies. More research is needed to assess factors that may predict changes in outcomes and how a web-based tool focused on self-management strategies may moderate those factors. | Male | Humans | Patient Participation | Computer-Assisted Instruction | OK |
| Sergio | Flores-Hernández | Claudia | Infante | Ricardo | Pérez-Cuevas | BMC women's health | 2011-00-00 2011 | BACKGROUND: Climacteric stage women experience significant biological, psychological and social changes. With demographic changes being observed in the growing number of climacteric stage women in Mexico, it is important to improve their knowledge about the climacteric stage and its potential associated problems, encourage their participation in screening programs, and promote the acquisition of healthy lifestyles.At Mexican health care institutions the predominant health care model for climacteric stage women has a biomedical perspective. Medical doctors provide mostly curative services and have limited support from other health professionals. This study aims to design an integrative health care model (IHCM: bio-psycho-social, multidisciplinary and women-centered) applicable in primary care services aimed at climacteric stage women. METHODS/DESIGN: We present the design, inclusion criteria and detailed description of an IHCM. The IHCM consists of collaborative and coordinated provision of services by a health team, which is involves a family doctor, nurse, psychologist, and the woman herself. The health team promotes the empowerment of women through individual and group counseling on the climacteric stage and health related self-care. The intervention lasts three months followed by a three-month follow-up period to evaluate the effectiveness of the model. The effectiveness of the model will be evaluated through the following aspects: health-related quality of life (HR-QoL), empowerment, self-efficacy and knowledge regarding the climacteric stage and health-related self-care activities, use of screening services, and improvement in lifestyles (regular leisure time physical activity and healthy diet). DISCUSSION: Participation in preventive activities should be encouraged among women in Mexico. Designing and evaluating the effectiveness of an integrative health care model for women at the climacteric stage, based on the empowerment approach and focus on health-related self-care to improve their HR-QoL is pertinent for current health conditions of this age group. TRIAL REGISTRATION: The study is registered at the ClinicalTrials.gov (NCT01272115). | Humans | Patient Participation | Questionnaires | Female | OK |
| Jen-Shi | Chen | Pei-Kwei | Tsay |  |  | Cancer Nursing | 2011-04-00 2011 Mar-Apr | BACKGROUND: Clinical trials provide information for developing new treatment strategies, enhancing cancer knowledge, and improving trial effectiveness. However, factors related to patients' participation in clinical trials have not been explored in Taiwan. OBJECTIVES: This study explored cancer patients' willingness to participate in clinical trials and motivational factors, barriers, needs, expectations, and other factors influencing their decision making. METHODS: In this cross-sectional study, information was collected using structured questionnaires with cancer patients from an academic medical center. RESULTS: Among 184 cancer patients sampled, most (87.5%) were willing to participate in a clinical trial. Their attitude toward participating in clinical trials was positive (average motivational score = 59.09; highest scoring item was "chance of cure"). Participants had a slight barrier level for participating in clinical trials (average barrier score = 21.51; highest scoring item was "concern over adverse effects of drugs used in clinical trials"). They had strong needs for clinical trial participation (average need factors score = 59.24; highest scoring need item was "supply of clinical trial-related information by medical personnel, including study purpose, adverse effects, effectiveness, and notices"). Participants had expectations of clinical trial participation (average expectations score = 66.58; highest scoring item was "nonrecurrence of tumor"). Factors predicting participation in clinical trials included medical issues and subjective viewpoint. CONCLUSIONS: These study results can help clinical researchers understand cancer patients' willingness to participate in clinical trials and other influencing factors. IMPLICATIONS FOR PRACTICE: Clinical research nurses can use this information in discussions with cancer patients and important stakeholders to help eligible patients better understand clinical trials to make informed decisions about participation. | Male | Humans | Patient Participation | Questionnaires | OK |
| Elizabeth | Spellman | Beth N. | Peshkin | Heiddis | Valdimarsdottir | Journal of Surgical Oncology | 2013-06-00 Jun 2013 | BACKGROUND: Recent trends suggest that bilateral mastectomy (BM) is on the rise among women diagnosed with unilateral breast cancer. Few studies have investigated the factors associated with the decision to have more aggressive surgery among young, high risk patients. METHODS: As part of a larger study, 284 women aged 50 and under completed an initial survey within 6 weeks of a breast cancer diagnosis. We assessed sociodemographics, medical and family history variables, treatment recommendations, preferences and concerns, distress, perceived risk, knowledge, and neuroticism. We used multiple regression with backward entry to assess the relationship between these variables and our outcomes of decisional conflict and intentions for BM. RESULTS: Higher decisional conflict was associated with being less educated, unmarried, more anxious and less likely to have received a surgical recommendation. Preference for BM was associated with higher neuroticism, perceived risk for contralateral breast cancer, pre-testing risk of carrying a BRCA1/2 mutation, having received either a surgical recommendation (vs. no recommendation), and lower preference for lumpectomy. CONCLUSIONS: For younger women, a surgical recommendation is associated with lower decisional conflict and stronger intention for BM. Results highlight the importance of effective risk communication and decision support between a woman and her surgeon. | Decision Making | Humans | Patient Participation | Questionnaires | OK |
| May Solveig | Fagermoen |  |  |  |  | Pain Management Nursing: Official Journal of the American Society of Pain Management Nurses | 2010-06-00 Jun 2010 | Women report more postoperative pain and problems performing domestic activities than men in the first month of recovery after cardiac surgery. The purpose of this article is to describe how women rate and describe pain interference with daily life after early discharge from cardiac surgery. A qualitative study was conducted in 2004-2005 with ten women recruited from a large Norwegian university hospital before discharge from their first elective cardiac surgery. Various aspects of the women's postoperative experiences were collected with qualitative interviews in the women's homes 8-14 days after discharge: a self-developed pain diary measuring pain intensity, types and amount of pain medication taken every day after returning home from hospital; and the Brief Pain Inventory-Short Form immediately before the interview. Qualitative content analysis was used to identify recurring themes from the interviews. Data from the questionnaires provided more nuances to the experiences of pain, pain management, and interference of postoperative pain. Postoperative pain interfered most with sleep, general activity, and the ability to perform housework during the first 2 weeks after discharge. Despite being advised at the hospital to take pain medication regularly, few women consumed the maximum amount of analgesics. Early hospital discharge after open cardiac surgery implies increased patient participation in pain management. Women undergoing this surgery need more information in hospital on why postoperative pain management beyond simple pain relief is important. | Humans | Questionnaires | Female | Aged | OK |
| Ingrid | Thyberg | Mikael | Thyberg |  |  | Journal of Rehabilitation Medicine | 2012-06-07 Jun 7, 2012 | OBJECTIVE: To explore internal consistency and correlations between perceived ability, performance and perceived importance in a preliminary selection of self-reported items representing the activity/participation component of the International Classification of Functioning, Disability and Health (ICF). DESIGN: Structured interview study. SUBJECTS: Fifty-five Swedish adolescents and adults with a mild intellectual disability. METHODS: Questions about perceived ability, performance and perceived importance were asked on the basis of a 3-grade Likert-scale regarding each of 68 items representing the 9 ICF domains of activity/participation. RESULTS: Internal consistency for perceived ability (Cronbach's alpha for all 68 items): 0.95 (values for each domain varied between 0.57 and 0.85), for performance: 0.86 (between 0.27 and 0.66), for perceived importance: 0.84 (between 0.27 and 0.68). Seventy-two percent of the items showed correlations >0.5 (mean=0.59) for performance vs perceived importance, 41% >0.5 (mean=0.47) for perceived ability vs performance and 12% >0.5 (mean=0.28) for perceived ability vs perceived importance. CONCLUSION: Measures of performance and perceived importance may have to be based primarily on their estimated clinical relevance for describing aspects of the ICF participation concept. With a clinimetric approach, parts of the studied items and domains may be used to investigate factors related to different patterns and levels of participation, and outcomes of rehabilitation. | Male | Humans | Patient Participation | Young Adult | OK |
|  |  |  |  |  |  | The Journal of Ambulatory Care Management | 2012-06-00 2012 Apr-Jun | We analyzed 2011 survey data of patients with complex health care needs in 11 countries (Australia, Canada, France, Germany, the Netherlands, New Zealand, Norway, Sweden, Switzerland, the United Kingdom, and the United States) on their care experiences. Wide country variation was reported in how well patients were engaged by their clinicians; UK and Swiss patients reported the most positive experiences, but gaps were reported in all countries. Disparities by income were found in several countries, with the widest gaps reported in the United States. Across countries, engaged patients reported receiving higher-quality care, fewer errors, and more positive views of the health system. | Male | Decision Making | Humans | Patient Participation | OK |
| Joel | Scanlan |  |  |  |  | Studies in Health Technology and Informatics | 2010-00-00 2010 | Exposure to phobic stimuli in subjects with specific phobia typically results in increased anxiety, ranging from mild to severe, followed by gradual habituation. The Internet is a candidate medium for the delivery of phobic stimuli to phobic subjects, such as pictures, video clips or computer animations. Delivery of such images in home settings warrants careful attention to the range and time course of anxiety responses elicited, and to tailoring of progression through hierarchies of images. The agency of the user is paramount, they need to have the final say at all stages of exposure as to whether to proceed or not. We have incorporated solutions to these requirements in the design of an internet-based exposure program (FEARDROP). This employs a database repository of pictures and videos of phobic stimuli. Images are called up by the user engaging a tracking circle with their mouse and following it around the screen. The image fades out if the circle is not followed, a form of 'dead man's brake'. Anxiety responses are measured at intervals on a visual analogue scale and graphed for the user. Initial results show substantial habituation to spider pictures within minutes, with a controlled comparison to video images in progress. | Animals | Humans | Patient Participation | Questionnaires | OK |
| Robert | Feldman | Le | Zhou |  |  | Journal of Medical Internet Research | 2013-00-00 2013 | BACKGROUND: The Internet is bringing fundamental changes to medical practice through improved access to health information and participation in decision making. However, patient preferences for participation in health care vary greatly. Promoting patient-centered health care requires an understanding of the relationship between Internet use and a broader range of preferences for participation than previously measured. OBJECTIVE: To explore (1) whether there is a significant relationship between Internet use frequency and patients' overall preferences for obtaining health information and decision-making autonomy, and (2) whether the relationships between Internet use frequency and information and decision-making preferences differ with respect to different aspects of health conditions. METHODS: The Health Information Wants Questionnaire (HIWQ) was administered to gather data about patients' preferences for the (1) amount of information desired about different aspects of a health condition, and (2) level of decision-making autonomy desired across those same aspects. RESULTS: The study sample included 438 individuals: 226 undergraduates (mean age 20; SD 2.15) and 212 community-dwelling older adults (mean age 72; SD 9.00). A significant difference was found between the younger and older age groups' Internet use frequencies, with the younger age group having significantly more frequent Internet use than the older age group (younger age group mean 5.98, SD 0.33; older age group mean 3.50, SD 2.00; t436=17.42, P<.01). Internet use frequency was positively related to the overall preference rating (γ=.15, P<.05), suggesting that frequent Internet users preferred significantly more information and decision making than infrequent Internet users. The relationships between Internet use frequency and different types of preferences varied: compared with infrequent Internet users, frequent Internet users preferred more information but less decision making for diagnosis (γ=.57, P<.01); more information and more decision-making autonomy for laboratory test (γ=.15, P<.05), complementary and alternative medicine (γ=.32, P<.01), and self-care (γ=.15, P<.05); and less information but more decision-making autonomy for the psychosocial (γ=-.51, P<.01) and health care provider (γ=-.27, P<.05) aspects. No significant difference was found between frequent and infrequent Internet users in their preferences for treatment information and decision making. CONCLUSIONS: Internet use frequency has a positive relationship with the overall preferences for obtaining health information and decision-making autonomy, but its relationship with different types of preferences varies. These findings have important implications for medical practice. | Male | Humans | Patient Participation | Questionnaires | OK |
| Nehama | Lewis |  |  |  |  | Patient Education and Counseling | 2010-12-00 Dec 2010 | OBJECTIVE: This study examines whether cancer patients' Internet use leads them to prefer a more active role in medical decision making and whether the effects of Internet use on active participation preferences vary according to patients' education levels. METHODS: Randomly drawn sample (N=2013) from the Pennsylvania Cancer Registry, comprised of breast, prostate, and colon cancer patients, completed mail surveys in the fall of 2006 (overall response rate=64%). Of 2013 baseline respondents, 85% agreed to participate in follow-up survey (N=1703). Of those who agreed, 76% (N=1293) completed follow-up surveys in the fall of 2007. RESULTS: Cancer patients' Internet use for health information at wave one led them to want to be more active participants in medical decision making at wave two (β=.06, p<.05). This applied to all cancer patients regardless of their education levels. CONCLUSION: Higher levels of Internet use among cancer patients may lead patients to want to be more actively involved in medical decision making. PRACTICE IMPLICATIONS: Considering the beneficial effects of patients' active participation in medical decision making, it will be worthwhile for health educators to recommend Internet use to cancer patients. | Male | Decision Making | Humans | Patient Participation | OK |
| Christine | Beaulieu | Fatima | Bouharaoui | Jean-Frédéric | Lévesque | Healthcare Policy = Politiques De Santé | 2011-12-00 Dec 2011 | The operational definition of interpersonal communication is "the ability of the provider to elicit and understand patient concerns, to explain healthcare issues and to engage in shared decision-making if desired." OBJECTIVE: To examine how well interpersonal communication is captured in validated instruments that evaluate primary healthcare from the patient's perspective. METHOD: 645 adults with at least one healthcare contact in the previous 12 months responded to instruments that evaluate primary healthcare. Eight subscales measure interpersonal communication: the Primary Care Assessment Survey (PCAS, two subscales); the Components of Primary Care Index (CPCI, one subscale); the first version of the EUROPEP (EUROPEP-I); and the Interpersonal Processes of Care Survey, version II (IPC-II, four subscales). Scores were normalized for descriptive comparison. Exploratory and confirmatory (structural equation) factor analysis examined fit to operational definition, and item response theory analysis examined item performance. RESULTS: Items not pertaining to interpersonal communication were removed from the EUROPEP-I. Most subscales are skewed positively. Normalized mean scores are similar across subscales except for IPC-II Patient-Centred Decision-Making and IPC-II Hurried Communication. All subscales load reasonably well on a single factor, presumed to be interpersonal communication. The best model has three underlying factors corresponding to eliciting (eigenvalue = 26.56), explaining (eigenvalue = 2.45) and decision-making (eigenvalue = 1.34). Both the PCAS Communication and the EUROPEP-I Clinical Behaviour subscales capture all three dimensions. Individual subscales within IPC-II measure each sub-dimension. CONCLUSION: The operational definition is well reflected in the available measures, although shared decision-making is poorly represented. These subscales can be used with confidence in the Canadian context to measure this crucial aspect of patient-centred care. | | | | | OK |
| Anne-Kathrin | Steger | Jürgen | Bengel |  |  | Patient Education and Counseling | 2012-10-00 Oct 2012 | OBJECTIVE: The aim of the study was to evaluate the interprofessional SDM training program "Fit for SDM" in medical rehabilitation, which was implemented in two steps: (1) university staff trained providers in executive positions as trainers and (2) the providers trained their staff. METHODS: For the evaluation of the first step a questionnaire for shared decision-making (SDM) skills and satisfaction with the training was completed by the providers in executive positions. A staff survey was used in a cluster-randomized controlled study to determine the overall impact of the train-the-trainer program on internal and external participation in the team. RESULTS: The providers in the six clinics evaluated their SDM competences and satisfaction very positively after training (step 1). External participation was enhanced by application of the training content, with significant changes recorded for females and nurses in particular. However, it had no direct influence on internal participation. CONCLUSIONS: This is the first interprofessional SDM train-the-trainer program in Germany to bridge interprofessionalism (internal participation) and SDM (external participation); it was implemented successfully and evaluated positively. PRACTICE IMPLICATIONS: Establishing interprofessional SDM training programs should be encouraged for all health care professionals. Implementation in the interprofessional setting should consider interprofessional team factors. | Male | Decision Making | Humans | Patient Participation | OK |
| Johanne | Gagnon | Michèle | St‐Pierre | François‐Pierre | Gauvin | Health Expectations: An International Journal of Public Participation in Health Care & Health Policy | 2014-12-00 décembre 2014 | Background: The recent establishment of health technology assessment (HTA) units in University hospitals in the Province of Quebec (Canada) provides a unique opportunity to foster increased participation of patients in decisions regarding health technologies and interventions at the local level. However, little is known about factors that influence whether the patient's perspective is taken into consideration when such decisions are made. Objective: To explore the practices, perceptions and views of the various HTA stakeholders concerning patient involvement in HTA at the local level. Method: Data were collected using semi‐structured interviews with 24 HTA producers and hospital managers and two focus groups with a total of 13 patient representatives. Results: Patient representatives generally showed considerable interest in being involved in HTA. Our findings support the hypothesis that the patient perspective contributes to a more accurate and contextualized assessment of health technologies and produces HTA reports that are more useful for decision makers. They also suggest that participation throughout the assessment process could empower patients and improve their knowledge. Barriers to patient involvement in HTA at the local level are also discussed as well as potential strategies to overcome them. Discussion and conclusion: This study contributes to knowledge that could guide interventions in favour of patient participation in HTA activities at the local level. Experimenting with different patient involvement strategies and assessing their impact is needed to provide evidence that will inform future interventions of this kind. (PsycINFO Database Record (c) 2014 APA, all rights reserved). (journal abstract) | Decision Making | Patient Participation | Client Participation | Intervention | Doublon |
| Johanne | Gagnon | Michèle | St-Pierre | François-Pierre | Gauvin | Health Expectations: An International Journal of Public Participation in Health Care and Health Policy | 2014-12-00 Dec 2014 | BACKGROUND: The recent establishment of health technology assessment (HTA) units in University hospitals in the Province of Quebec (Canada) provides a unique opportunity to foster increased participation of patients in decisions regarding health technologies and interventions at the local level. However, little is known about factors that influence whether the patient's perspective is taken into consideration when such decisions are made. OBJECTIVE: To explore the practices, perceptions and views of the various HTA stakeholders concerning patient involvement in HTA at the local level. METHOD: Data were collected using semi-structured interviews with 24 HTA producers and hospital managers and two focus groups with a total of 13 patient representatives. RESULTS: Patient representatives generally showed considerable interest in being involved in HTA. Our findings support the hypothesis that the patient perspective contributes to a more accurate and contextualized assessment of health technologies and produces HTA reports that are more useful for decision makers. They also suggest that participation throughout the assessment process could empower patients and improve their knowledge. Barriers to patient involvement in HTA at the local level are also discussed as well as potential strategies to overcome them. DISCUSSION AND CONCLUSION: This study contributes to knowledge that could guide interventions in favour of patient participation in HTA activities at the local level. Experimenting with different patient involvement strategies and assessing their impact is needed to provide evidence that will inform future interventions of this kind. | | | | | OK |
| Janet E. | Hiller | Anne | Gardner |  |  | Journal of Advanced Nursing | 2014-05-00 May 2014 | AIM: To investigate characteristics of collaboration between nurse practitioners and medical practitioners in the primary healthcare setting in Australia. BACKGROUND: Recent definitions of collaboration in the literature describe it as being based on communication, shared decision-making and the respect and equality of team members. However, research demonstrates a tension between this theoretical ideal and how collaboration between nurse practitioners and medical practitioners occurs in practice. Different socialization processes of the two professions and legislative requirements influence collaborative practice. The way these two professions overcome traditional boundaries and realize collaborative practice in the primary healthcare setting needs to be examined. DESIGN: Mixed methods multiple case study including up to six sites with a minimum of six and a maximum of 20 participants in total. METHODS: Data on collaborative practice between nurse practitioners and medical practitioners in primary health care will be collected in three phases: (1) two-week direct observation in the practice setting to capture actual behaviour and context; (2) questionnaire to measure dimensions of collaboration; and (3) one-to-one semi-structured interviews with nurse practitioners, medical practitioners and practice managers to record experiences, perceptions and understanding of collaboration. DISCUSSION: Triangulation of findings will generate a comprehensive understanding of how collaboration between nurse practitioners and medical practitioners in Australia occurs in the primary care setting. The results of this study will inform nurse practitioners, medical practitioners practice managers and policy makers on successful models of collaboration. | Questionnaires | Primary Health Care | medical staff | Interpersonal Relations | OK |
| Rugayah | Bakri | Alun | Cameron | Britta | Göhlen | International Journal of Technology Assessment in Health Care | 2013-01-00 Jan 2013 | OBJECTIVES: To obtain further information from members of the International Network of Agencies for Health Technology Assessment (INAHTA) on the involvement of consumers in their programs. METHODS: A questionnaire for a survey was developed and sent to member agencies in November 2010. Survey responses were compared with those from an earlier survey conducted in 2005. RESULTS: Of the thirty-three agencies that provided responses, 67 percent involve consumers in some aspects of their health technology assessment (HTA) programs, compared with 57 percent in 2005. As in the earlier survey, most agencies reporting involvement have contact with consumer or patient organizations and a large minority also involve individual consumers. Summaries of HTA reports that are intended to be easily understood by consumers are prepared by 84 percent of the agencies, and 42 percent involve consumers in dissemination of HTA material. In both areas, there was some increase from the levels previously reported. CONCLUSIONS: The survey results suggest that there is a trend to increased involvement of consumers by the INAHTA agencies in their programs but that the level of involvement remains relatively limited. The manner of consumer participation varies between agencies. | Humans | Patient Participation | Questionnaires | Consumer Participation | OK |
| Tom | Defloor | Sofie T. L. | Verhaeghe |  |  | Nurse Education Today | 2011-02-00 Feb 2011 | Even though the central position of the client has been recognized in psychiatric nursing education, the client is seldom formally involved in the feedback provided to students during practical training. This research paper focuses on three questions: (1) What conditions support the gathering of meaningful client feedback to enhance the student's learning process and client's wellbeing? (2) Does the use of the practical model for client feedback lead to positive experiences, and if so, under what conditions? (3) To what extent is a client's feedback on the student's work performance, consistent with feedback from the mentor (nurse from the ward), the teacher and the student? Based on a literature review, participatory observation and contacts with experts, a practical model was developed to elicit client feedback. Using this model in two psychiatric inpatient services, clients were actively and formally involved in providing feedback to four, final year psychiatric nursing students. Clients, nurses, teachers and students were interviewed and data were analysed using a qualitative explorative research approach. Analyses revealed that client feedback becomes meaningful in a safe environment created by the psychiatric nurse. Client feedback generates a learning effect for the student and supports the student's recognition of the value and vulnerability of the psychiatric client. | Humans | Patient Participation | Patient Satisfaction | mental health services | OK |
| Pauline S. C. | Kouwenhoven | Cristiano | Vezzoni | Ghislaine J. M. W. | van Thiel | Journal of Palliative Medicine | 2013-09-00 Sep 2013 | BACKGROUND AND OBJECTIVE: Advance care planning (ACP) is a process of communication among patients, health care providers, and relatives regarding end-of-life care. The aim of our study was to determine if a representative sample of the Dutch general public is currently involved or is inclined to be engaged in ACP, and to delineate the factors associated with greater engagement. METHODS: An online questionnaire was completed by a representative sample of the Dutch general public, derived from an established Internet panel (CentERdata, University of Tilburg, The Netherlands). RESULTS AND CONCLUSIONS: Although the majority of the Dutch population seems open to discussions about end-of-life care, our study revealed that discussions with physicians are exceedingly rare. To improve ACP as a joint process among patients, relatives, and physicians, the general public's awareness of the importance of end-of-life discussions with their physicians needs to be increased and physicians need to play a more active role by initiating conversations and dialogue regarding end-of-life care. | Male | Decision Making | Humans | Patient Participation | OK |
| Marie | Desmartis | Johanne | Gagnon | Daniel | La Roche | BMC health services research | 2014-00-00 2014 | BACKGROUND: Public and patient involvement in the different stages of the health technology assessment (HTA) process is increasingly encouraged. The selection of topics for assessment, which includes identifying and prioritizing HTA questions, is a constant challenge for HTA agencies because the number of technologies requiring an assessment exceeds the resources available. Public and patient involvement in these early stages of HTA could make assessments more relevant and acceptable to them. Involving them in the development of the assessment plan is also crucial to optimize their influence and impact on HTA research. The project objectives are: 1) setting up interventions to promote patient participation in three stages of the HTA process: identification of HTA topics, prioritization, and development of the assessment plan of the topic prioritized; and 2) assessing the impact of patient participation on the relevance of the topics suggested, the prioritization process, and the assessment plan from the point of view of patients and other groups involved in HTA. METHODS: Patients and their representatives living in the catchment area of the HTA Roundtable of Université Laval's Integrated University Health Network (covering six health regions of the Province of Quebec, Canada) will be involved in the following HTA activities: 1) identification of potential HTA topics in the field of cancer; 2) revision of vignettes developed to inform the prioritization of topics; 3) participation in deliberation sessions for prioritizing HTA topics; and 4) development of the assessment plan of the topic prioritized. The research team will coordinate the implementation of these activities and will evaluate the process and outcomes of patient involvement through semi-structured interviews with representatives of the different stakeholder groups, structured observations, and document analysis, mainly involving the comparison of votes and topics suggested by various stakeholder groups. DISCUSSION: This project is designed as an integrated approach to knowledge translation and will be conducted through a close collaboration between researchers and knowledge users at all stages of the project. In response to the needs expressed by HTA producers, the knowledge produced will be directly useful in guiding practices regarding patient involvement in the early phases of HTA. | | | | | OK |
| Geoffrey | Henning | Perla J. | Marang-van de Mheen | Eloy | Espin | The Patient | 2014-00-00 2014 | CONTEXT: High-quality cancer care should be accessible for patients and healthcare professionals. Involvement of patients as partners in guideline formation and consensus processes is still rarely found. EURECCA, short for European Registration of Cancer Care, is the platform to improve outcomes of cancer care by reducing variation in the diagnostic and treatment process. EURECCA acknowledges the important role of patients in implementation of consensus information in clinical practice. OBJECTIVE: The aim of this article is to describe the process of involving patients in the consensus process and in developing the patient summary of the consensus for colon and rectal cancer care. METHODS: The Delphi method for achieving consensus was used. Three online voting rounds and one tele-voting round were offered to an expert panel of oncology professionals and patient representatives. At four different stages, patients and/or patient representatives were involved in the process: (1) during the consensus process, (2) lecturing about the role of the patient, (3) development of the patient summary, and (4) testing the patient summary. RESULTS: Representatives were invited to the voting and commenting rounds of this process and given an equal vote. Although patients were not consulted during the planning stages of this process, patient involvement increased following the panel's discussion of the implementation of the consensus among the patient population. After the consensus meeting, the patient summary was written by patient representatives, oncologists and nurses. A selection of proactive patients reviewed the draft patient summary; responses were positive and several patient-reported outcomes were added. Questionnaires to evaluate the use and implementation of the patient summary in daily practice are currently being developed and tested. Patient consultation will be needed in future planning for selection of topics. DISCUSSION: The present study may function as a model for future consensus processes to involve patients at different stages and to implement both patient and healthcare professional versions in daily practice. | Decision Making | Patient Participation | Europe | Colorectal Neoplasms | OK |
| Anja | Klingenberg | Katharina | Glassen | Friederike | Heiss | International Journal for Quality in Health Care | 2014-06-00 juin 2014 | Objective: The purpose of this study was to develop and validate a generic questionnaire to evaluate experiences and reported outcomes in patients who receive treatment across a range of healthcare sectors. Design: Mixed-methods design including focus groups, pretests and field test. Setting: The patient questionnaire was developed in the context of a nationwide program in Germany aimed at quality improvements across the healthcare sectors. Participants: For the field test, 589 questionnaires were distributed to patients via 47 general practices. Main Measurements: Descriptive item analyzes non-responder analysis and factor analysis (PCA). Retest coefficients (r) calculated by correlation of sum scores of PCA factors. Quality gaps were assessed by the proportion of responders choosing a response category defined as indicating shortcomings in quality of care. Results: The conceptual phase showed good content validity. Four hundred and seventy-four patients who received a range of treatment across a range of sectors were included (response rate: 80.5%). Data analysis confirmed the construct, oriented to the patient care journey with a focus on transitions between healthcare sectors. Quality gaps were assessed for the topics ‘Indication’, including shared-decision-making (6 items, 24.5–62.9%) and ‘Discharge and Transition’ (10 items; 20.7–48.2%). Retest coefficients ranged from r = 0.671 until r = 0.855 and indicated good reliability. Low ratios of item-non-response (0.8–9.3%) confirmed a high acceptance by patients. Conclusions: The number of patients with complex healthcare needs is increasing. Initiatives to expand quality assurance across organizational borders and healthcare sectors are therefore urgently needed. A validated questionnaire (called PEACS 1.0) is available to measure patients’ experiences across healthcare sectors with a focus on quality improvement. (PsycINFO Database Record (c) 2014 APA, all rights reserved). (journal abstract) | Patient involvement | Questionnaires | Psychometrics | test validity | Doublon |
| Anja | Klingenberg | Katharina | Glassen | Friederike | Heiss | International journal for quality in health care: journal of the International Society for Quality in Health Care / ISQua | 2014-06-00 Jun 2014 | OBJECTIVE: The purpose of this study was to develop and validate a generic questionnaire to evaluate experiences and reported outcomes in patients who receive treatment across a range of healthcare sectors. DESIGN: Mixed-methods design including focus groups, pretests and field test. SETTING: The patient questionnaire was developed in the context of a nationwide program in Germany aimed at quality improvements across the healthcare sectors. PARTICIPANTS: For the field test, 589 questionnaires were distributed to patients via 47 general practices. MAIN MEASUREMENTS: Descriptive item analyzes non-responder analysis and factor analysis (PCA). Retest coefficients (r) calculated by correlation of sum scores of PCA factors. Quality gaps were assessed by the proportion of responders choosing a response category defined as indicating shortcomings in quality of care. RESULTS: The conceptual phase showed good content validity. Four hundred and seventy-four patients who received a range of treatment across a range of sectors were included (response rate: 80.5%). Data analysis confirmed the construct, oriented to the patient care journey with a focus on transitions between healthcare sectors. Quality gaps were assessed for the topics 'Indication', including shared-decision-making (6 items, 24.5-62.9%) and 'Discharge and Transition' (10 items; 20.7-48.2%). Retest coefficients ranged from r = 0.671 until r = 0.855 and indicated good reliability. Low ratios of item-non-response (0.8-9.3%) confirmed a high acceptance by patients. CONCLUSIONS: The number of patients with complex healthcare needs is increasing. Initiatives to expand quality assurance across organizational borders and healthcare sectors are therefore urgently needed. A validated questionnaire (called PEACS 1.0) is available to measure patients' experiences across healthcare sectors with a focus on quality improvement. | | | | | OK |
| Hazel | Walters |  |  |  |  | Cochlear Implants International | 2013-11-00 Nov 2013 | BACKGROUND: The National Institute for Health and Clinical Excellence guidelines recommended young people who currently have one cochlear implant be offered assessment for a second, sequential implant, due to the reported improvements in sound localization and speech perception in noise. The possibility and benefits of group information and counselling assessments were considered. Previous research has shown advantages of group sessions involving young people and their families and such groups which also allow young people opportunity to discuss their concerns separately to their parents/guardians are found to be 'hugely important'. Such research highlights the importance of involving children in decision-making processes. METHODS: Families considering a sequential cochlear implant were invited to a group information/counselling session, which included time for parents and children to meet separately. Fourteen groups were held with approximately four to five families in each session, totalling 62 patients. The sessions were facilitated by the multi-disciplinary team, with a particular psychological focus in the young people's session. RESULTS: Feedback from families has demonstrated positive support for this format. Questionnaire feedback, to which nine families responded, indicated that seven preferred the group session to an individual session and all approved of separate groups for the child and parents/guardians. CONCLUSION/DISCUSSION: Overall the group format and psychological focus were well received in this typically surgical setting and emphasized the importance of involving the young person in the decision-making process. This positive feedback also opens up the opportunity to use a group format in other assessment processes. | Male | Decision Making | Humans | Patient Participation | OK |
| Steen | Terpling | Klaus M. | Pedersen | Peter | Gustenhoff | Maturitas | 2012-01-00 Jan 2012 | OBJECTIVE: To assess thyroid autoimmunity among elderly people living in an area with low iodine intake compared to the sustained recommended iodine intake from a natural source, and to estimate the importance of migration. DESIGN AND SETTING: Iodine content of drinking water is highly different in the Danish towns Randers and Skagen. We collected blood and spot urine samples from 430 long-term Randers and Skagen dwellers aged 75-80 years, who filled in a questionnaire. We measured thyroid peroxidase antibody (TPOAb) and thyroglobulin antibody (TGAb) in serum and iodine and creatinine in urine. RESULTS: Participation rate was 47% (n=212 (men/women 82/130) in Randers; 218 (84/134) in Skagen). Iodine deficiency prevailed in Randers while Skagen dwellers were iodine replete (median urinary iodine 74 μg/24h vs. 184 μg/24h, p<0.001). Thyroid antibodies were more frequent in Randers than in Skagen residents (42% vs. 32%; p=0.006) and more likely with iodine excretion <50 μg/24h (OR, 95%CI: 1.9, 1.1-3.4). Differences between towns increased with longer duration of residence as trends in the occurrence of TGAb and TPOAb were opposite (p<0.001; p=0.007). CONCLUSIONS: Thyroid autoantibodies were common in old age, influenced by the iodine intake level, and the lowest frequency was found at the recommended iodine intake level. | Male | Humans | Patient Participation | Questionnaires | OK |
| Stephen | Leeder | Kerryn | Phelps |  |  | Journal of Evaluation in Clinical Practice | 2013-10-00 Oct 2013 | RATIONALE: A multidisciplinary primary care clinic in Sydney, Australia, was planning to use electronic questionnaires to measure patient-reported outcomes. METHODS: Semi-structured interviews with 20 patients were undertaken to explore, among other things, practical issues regarding different questionnaire formats. The response rates and costs of email versus postal invitations were also evaluated. RESULTS: Compared with postal invitations, email invitations offered a cost-effective and practical alternative, with a greater proportion of patients volunteering for an interview. Assuming the interface is well-designed and user-friendly, many patients were happy to use the Internet to answer questionnaires. Most patients thought alternate formats should also be offered. Patients discussed advantages and disadvantages of the Internet format. Although more younger patients and females had given the clinic an email address; both sexes, and young and old patients, expressed strong preferences for either wanting or not wanting to use the Internet. CONCLUSION: Researchers should consider using email invitations as a cost-effective first-line strategy to recruit patients to participate in health services research. Internet questionnaires are potentially cheaper than paper questionnaires, and the format is acceptable to many patients. However, for the time being, concurrent alternate formats need to be offered to ensure wider acceptability and to maximize response rates. | Male | Humans | Patient Participation | Questionnaires | OK |
| Anna T. | Höglund | Bertil | Lindahl | Kalle | Spångberg | Health Expectations: An International Journal of Public Participation in Health Care and Health Policy | 2010-09-00 Sep 2010 | OBJECTIVE: To investigate whether patient involvement during hospitalization for acute myocardial infarction (MI) was associated with health and behavioural outcomes 6-10 weeks after hospital discharge. BACKGROUND: Patient involvement has been associated with improved health outcomes in chronic disease, but less research has focused on the effects of patient involvement in acute conditions, such as MI. DESIGN: Self-administered questionnaire study. Questionnaire results were run against medical outcome data in a national database of cardiac patients. SETTING AND PARTICIPANTS: Cardiac patients (n = 591) on their first follow-up visit after hospitalization for MI at 11 Swedish hospitals. MAIN OUTCOME MEASURES: Patient ratings of three questionnaire scales related to involvement; cardiovascular symptoms, medication compliance, participation in cardiac rehabilitation, and achievement of secondary preventive goals. RESULTS: More positive patient ratings of involvement were significantly associated with fewer cardiovascular symptoms 6-10 weeks after hospital discharge. In contrast, patients who attended cardiac rehabilitation and achieved the goals for smoking cessation and systolic blood pressure were significantly less satisfied with their involvement. No association was found between involvement ratings and medication compliance. CONCLUSION: This study represents a first attempt to examine associations between patient involvement in the acute phase of illness and short-term health outcomes. Some significant associations between involvement and health and behavioural outcomes after acute MI were found. However, higher involvement ratings were not consistently associated with more desirable outcomes, and involvement during hospitalization was not associated with MI patient health and behaviour 6-10 weeks after hospital discharge to the extent hypothesized. | Male | Humans | Patient Participation | Questionnaires | OK |
| Reidar | Pedersen |  |  |  |  | Tidsskrift for Den Norske Lægeforening: Tidsskrift for Praktisk Medicin, Ny Række | 2011-01-21 Jan 21, 2011 | BACKGROUND: All health enterprises in Norway today have at least one clinical ethics committee (CEC). One of the aims is to give advice and to counsel the hospital staff on ethical issues. As part of the quality assurance of this work, we wanted to find out if clinicians have benefited from these committees in individual cases. MATERIAL AND METHODOLOGY: The local committees were requested to distribute a questionnaire to all clinicians who had submitted a case to the committee during the previous 18 months. The survey was anonymous. Out of the 86 questionnaires that were distributed, 43 (50%) were returned to the Centre for Medical Ethics. RESULTS: The majority of clinicians had a number of reasons for contacting the committee. The most usual reason was the desire to have a broad consultation on a case (70%), which was regarded as useful. The most common issue discussed was limiting the treatment of a seriously ill patient (56%), the will/wishes of the next-of-kin (40%) and patient autonomy (37%). The committee gave advice in 50% of the cases. Thirty-eight percent of the consultations resulted in practical consequences, including the discontinuation of treatment in six cases. INTERPRETATION: Because of the low response percentage, the results must be interpreted with caution. The work of the committees is generally evaluated as useful, and the consultations can have practical consequences. However, it is challenging to make this work better known among clinicians and to conduct quality assurance. | Humans | Patient Participation | Questionnaires | communication | OK |
| Monika | Sztankay | Verena | Meraner | Beate | Beer | Breast Cancer Research and Treatment | 2011-07-00 Jul 2011 | Adjuvant endocrine treatment-related adverse effects have a strong impact on patients' quality of life and thereby limit therapy's risk benefit ratio resulting in morbidity and treatment discontinuation. Still, many AI adverse effects remain untreated given that they are unrecognized by conservative methods (e.g., proxy ratings). The ability of complementary patient-reported outcomes (PROs) to provide a more comprehensive assessment of side-effects is to be explored. A cross-sectional study sample of 280 postmenopausal, early stage breast cancer patients was subjected to a comprehensive PRO assessment (FACT-B/+ES) at their after-care appointment. Prevalence and severity of patient-reported physical side-effects and psychosocial burden related to adjuvant AI therapy were compared with prevalences derived from pivotal phase IV trials (ATAC 2005, BIG1-98 2005). Across all symptom categories, highest prevalence rates were found for joint pain (59.6%), hot flushes (52%), lost interest in sexual intercourse (51.4%), and lack of energy (40.3%). Overall, PROs resulted in significantly higher prevalence rates as compared to physician ratings for all symptoms published in pivotal clinical trials except vaginal bleeding and nausea. The treatment duration exerted no significant impact on symptom frequency (P > 0.05). Established prevalence rates of endocrine treatment-related toxicity seem to be underestimated. The incorporation of PRO data should be mandatory or at least highly recommended in clinical treatment planning to arrive at a more accurate assessment of a patient's actual symptom burden enabling improved individualized management of side-effects and mediating the preservation of treatment adherence. | Humans | Patient Participation | Female | Aged | OK |
|  |  |  |  |  |  | Archives of Physical Medicine and Rehabilitation | 2010-09-00 Sep 2010 | The ever-growing number of participation measures without consensus on which is best makes it difficult to determine which measure to use in rehabilitation research and clinical trials. In an effort to address issues affecting the selection of a participation measure for a specific research purpose, this article (1) outlines the types and characteristics of participation measures, (2) enumerates various uses of participation measures in disability and rehabilitation research, (3) discusses appropriate matching of the type of participation measure with the research task, and (4) offers recommendations for future participation research. Participation instruments vary in terms of their degree of participation specificity, the conceptual model that underlies their development, whether they include multiple domains or take a more global approach, the extent to which they are objective versus subjective, whether they use general population norms, who is the respondent, the method of item and scale development, and their psychometric properties. Participation measures are used in individual and population assessments, observational research, and interventional research. Selection of a participation measure for use in a specific study requires an understanding of the characteristics of available tools and the nature of the research design, but most importantly, it requires matching the instrument to the specific research question or hypothesis. Instruments assessing participation are currently appropriate as secondary outcomes in trials evaluating interventions targeting activity limitation, and they will become appropriate as primary outcomes when interventions are tested that target participation directly. It will be easier to apply participation measures appropriately to their many research uses once substantial progress is made in obtaining better participation measurements and consensus is reached about the best tools. | Humans | Patient Participation | Questionnaires | Rehabilitation | OK |
| Marcel | Dijkers | Margaret | Brown | Susan | Magasi | Archives of Physical Medicine and Rehabilitation | 2010-09-00 Sep 2010 | This article summarizes the recommendations made by participants at the International Symposium on Measurement of Participation in Rehabilitation Research. We draw on presentations by researchers, clinicians, and consumers; a respondent panel; and small group discussions that included symposium faculty and participants. Breakout groups discussion questions focused on several key issues: What do we know about defining and measuring participation? What don't we know? What are the research barriers to defining and measuring participation? What are the research priorities to resolve these obstacles and develop better instruments? What are the organizations and opportunities to continue the work of this symposium? What are the major aspects of participation that should be measured? With what kinds of participation constructs do item response theory methods and clinimetric and other psychometric approaches fit? Do people form "types" of "participants" that can be categorized? Which person characteristics should be considered when evaluating differential item functioning? What cultural and physical features should be considered in measuring participation? What legacy measures could be used as an item pool to tap "participation?" Can a crosswalk unite "legacy" participation measures? Reporters summarized the subgroups' discussions, and then the larger group focused on shared concerns, which are summarized here. | Humans | Patient Participation | Psychometrics | quality of life | OK |
|  |  |  |  |  |  | Archives of Physical Medicine and Rehabilitation | 2010-09-00 Sep 2010 | While participation is increasingly defined as the key outcome of rehabilitation, disagreements on and shortcomings in the definition, operationalization, and measurement of this concept abound and interfere with the progress of clinical services and research. This article explores a number of the major issues related to the quantification of participation and makes suggestions for new directions, using the following orienting questions: What is the definition of participation? Where is the border between Participation and Activity? Is there more to participation than performance? What domains should be included in a participation measure? What are the appropriate metrics in quantifying participation? How do we define adequate participation? How should participation be operationalized? What is the proper measurement model for participation instruments? How should we collect data on participation? How do we evaluate the quality of a participation instrument? | Humans | Patient Participation | Patient Satisfaction | Questionnaires | OK |
| Galina | Velikova | Brenda | Roe | Kieran | Horgan | Tumori | 2014-10-00 2014 Sep-Oct | AIMS AND BACKGROUND: In breast cancer (BC) there are different therapies available with different side effects affecting the health-related quality of life (HRQOL) of patients. Here we report a novel tool, the BC-specific Patient Concerns Inventory (PCI). This work includes a survey that is part of the validation process to allow a larger cohort and comparisons with clinical characteristics. We report the concerns that BC patients would like to discuss in the outpatient clinic - using the PCI - and also their choice of multidisciplinary team members they would like to see. METHODS AND STUDY DESIGN: We carried out a cross-sectional survey - using the BC-specific PCI, the EORTC QLQ-C30 and the EORTC BC QLQ-BR23 - of patients who had completed their initial treatment and were attending a review outpatient clinic. 249 patients were recruited from February to July 2012. RESULTS: Survey responses were obtained from 80% (200/249). The three most frequent items were fear of cancer coming back (62%, 124), breast sensitivity/pain (46%, 92), and fatigue/tiredness and low energy levels overall (46%, 92). The most frequently selected members of the multidisciplinary team that patients wished to see were the breast care nurse (46%, 92), the medical oncologist (28%, 55) and the psychologist (20%, 40). CONCLUSIONS: The PCI provides the opportunity for multiprofessional engagement across a range of issues specific to BC. It can identify issues relating to physical, psychological, sexual and social functioning, as well as issues relating to body image and lifestyle. | Male | Humans | Patient Participation | Questionnaires | OK |
| Rosemary | Chesson |  |  |  |  | Patient Education and Counseling | 2010-02-00 Feb 2010 | OBJECTIVES: Medicines management plays a key role in schizophrenia treatment. Objectives were to investigate the feasibility of undertaking in-depth research involving in-patients with a diagnosis of schizophrenia and to determine patients' views and experiences of medicines management. METHODS: Research was carried out in the forensic ward of a psychiatric hospital and two rehabilitation settings in north-east Scotland. A qualitative methodology was adopted incorporating semi-structured interviews. Full text transcripts were produced and analysed. RESULTS: Fifteen of 16 patients approached were willing to participate. Ages ranged from 27 to 70 years and they had been prescribed antipsychotics from six months to 27 years. Nearly everyone was aware of their medication regimen. Most relied on staff for information, but this was supplemented by learning from other patients' experiences. Most were happy to leave decisions to staff and few sought any role in shared decision making. CONCLUSION: The research demonstrated the feasibility of undertaking qualitative work with patients with schizophrenia. Findings indicated that it should not be assumed that all patients want to take responsibility for their medications. PRACTICE IMPLICATIONS: Patients' reluctance to assume responsibility needs to be explored and patients' wishes taken into account when planning future medicine related education. | Male | Humans | Patient Participation | Questionnaires | OK |
| Traudel | Weber-Reich | Alfred | Simon |  |  | Journal of Medical Ethics | 2014-05-00 May 2014 | This study explores the attitude of German psychiatrists in leading positions towards joint crisis plans and psychiatric advance directives. This topic was examined by contacting 473 medical directors of German psychiatric hospitals and departments. They were asked to complete a questionnaire developed by us. That form contained questions about the incidence and acceptance of joint crisis plans and psychiatric advance directives and previous experiences with them. 108 medical directors of psychiatric hospitals and departments responded (response rate: 22.8%). Their answers demonstrate that in their hospitals these documents are rarely used. Among the respondents, joint crisis plans are more accepted than psychiatric advance directives. There is a certain uncertainty when dealing with these instruments. Our main conclusion is that German psychiatry needs an intensified discussion on the use of instruments for patients to constitute procedures for future critical psychiatric events. For this purpose it will be helpful to collect more empirical data. Furthermore, the proposal of joint crisis plans in psychiatric hospitals and departments should be discussed as well as the possibility of consulting an expert during the preparation of a psychiatric advance directive. | Male | Decision Making | Humans | Patient Participation | OK |
| Ingrid V. F. | van den Broek | Jan E. A. M. | van Bergen | Elfi E. H. G. | Brouwers | BMC public health | 2012-00-00 2012 | BACKGROUND: Effectiveness of Chlamydia screening programs is determined by an adequate level of participation and the capturing of high-risk groups. This study aimed to evaluate the contribution of automated reminders by letter, email and short message service (SMS) on package request and sample return in an Internet-based Chlamydia screening among people aged 16 to 29 years in the Netherlands. METHODS: Individuals not responding to the invitation letter received a reminder letter after 1 month. Email- and SMS-reminders were sent to persons who did not return their sample. It was examined to what extent reminders enhanced the response rate (% of package requests) and participation rate (% of sample return). Sociodemographic and behavioural correlates of providing a cell phone number and participation after the reminder(s) were studied by logistic regression models. RESULTS: Of all respondents (screening round 1: 52,628, round 2: 41,729), 99% provided an email address and 72% a cell phone number. Forty-two percent of all package requests were made after the reminder letter. The proportion of invitees returning a sample increased significantly from 10% to 14% after email/SMS reminders (round 2: from 7% to 10%). Determinants of providing a cell-phone number were younger age (OR in 25-29 year olds versus 16-19 year olds = 0.8, 95%CI 0.8-0.9), non-Dutch (OR in Surinam/Antillean versus Dutch = 1.3, 95%CI 1.2-1.4, Turkish/Moroccan: 1.1, 95%CI 1.0-1.2, Sub Sahara African: 1.5, 95%CI 1.3-1.8, non-Western other 1.1, 95%CI 1.1-1.2), lower educational level (OR in high educational level versus low level = 0.8, 95%CI 0.7-0.9), no condom use during the last contact with a casual partner (OR no condom use versus condom use 1.2, 95%CI 1.1-1.3), younger age at first sexual contact (OR 19 years or older versus younger than 16: 0.7, 95%CI 0.6-0.8). Determinants for requesting a test-package after the reminder letter were male gender (OR female versus male 0.9 95%CI 0.8-0.9), non-Dutch (OR in Surinam/Antillean versus Dutch 1.3, 95%CI 1.2-1.4, Turkish/Moroccan: 1.4, 95%CI 1.3-1.5, Sub Sahara African: 1.4, 95%CI 1.2-1.5, non-Western other: 1.2, 95%CI 1.1-1.2), having a long-term steady partnership (long-term versus short-term.1.2 95%CI 1.1-1.3). Email/SMS reminders seem to have resulted in more men and people aged 25-29 years returning a sample. CONCLUSIONS: Nearly all respondents (99.5%) were reachable by modern communication media. Response and participation rates increased significantly after the reminders. The reminder letters also seemed to result in reaching more people at risk. Incorporation of automated reminders in Internet-based (Chlamydia) screening programs is strongly recommended. | Male | Humans | Patient Participation | Questionnaires | OK |
| Nancy | Booker | Mary | Kizito | Kyalo | wa Ngula | Patient Education and Counseling | 2011-02-00 Feb 2011 | OBJECTIVE: This study explored Kenyan patients' perspectives on the role of ethnicity in the doctor-patient relationship. METHODS: 221 participants completed questionnaires on ethnicity in doctor-patient relationships; eight focus groups were held with low- and middle-income urban and rural women. RESULTS: About half of participants expressed no preference for doctor ethnicity. Participants rated demographic factors as less important than factors related to the doctor's qualifications, communication skills, and cost of service. Those who did indicate a preference were more likely to prefer Indian doctors for eye problems and Europeans for major surgery, cancer, and heart problems. With less severe medical issues participants were more likely to prefer a doctor who was ethnically concordant with them. Reasons for this centered around communication issues. In contrast, several focus group participants did not want to be treated by doctors from their own ethnic group because of concerns about confidentiality. CONCLUSION: Additional research is needed on negative implications of patient-provider concordance. PRACTICE IMPLICATIONS: Medical service providers must be aware of concerns about ethnic concordance. Alternatively medical centers that deal with sensitive medical information need to consider hiring staff who are not of the majority ethnic group in their region. | Male | Humans | Patient Participation | Physician-Patient Relations | OK |
| Linda | Robertson | Stacy | Eckstein | Camille | Ragin | Journal of Cancer Education: The Official Journal of the American Association for Cancer Education | 2014-03-00 Mar 2014 | Disparities exist in breast cancer knowledge and education, which tend to influence symptom interpretation and decision to seek screening/care. The present project describes a cohort of women's experiences, knowledge, and health behavior prior to and after a diagnosis of breast cancer. It also studies how knowledge and demographic factors are associated with level of involvement participants had in the treatment of their breast cancer. Women >18 years who have been diagnosed and treated for breast cancer within 10 years were recruited in Pittsburgh, PA, through the Healthy People Cohort Registry, a database of volunteers from the community, and Brooklyn, NY, through the American Cancer Society breast cancer survivor database. Subsequent to institutional ethics approval, a questionnaire was administered by mail and through an electronic interactive format. The study included 124 breast cancer survivors, one-quarter of whom were of African ancestry. Roughly half of the women indicated that their overall knowledge of breast cancer was limited before diagnosis; no significant association between overall knowledge before diagnosis and stage at diagnosis or an active role of the patient in treatment choices was observed. Two-third of the women reported using personal research on internet, books, and other media to increase knowledge on breast cancer after diagnosis; the improvement of knowledge was associated with an active role in therapy choice. White women's self report of breast cancer knowledge prior to diagnosis was higher than that of women of African origin (p = 0.03); the latter experienced more delays in getting results about the diagnosis (p = 0.002), in starting treatment (p = 0.03), and in having treatment available at local facilities (p = 0.007) than white women. White women were more likely to improve their knowledge through their own research (p = 0.08) and through the contribution of their physician (p = 0.06) than women of African origin.There is still a need for addressing breast cancer knowledge among black women, and improvement in physician emotional support and in their contribution to the patient's knowledge is necessary. These efforts may have a positive impact on breast cancer knowledge among black women in the US. | Decision Making | Humans | Patient Participation | Questionnaires | Doublon |
| Linda | Robertson | Stacy | Eckstein | Camille | Ragin | Journal of Cancer Education: The Official Journal of the American Association for Cancer Education | 2014-03-00 Mar 2014 | Disparities exist in breast cancer knowledge and education, which tend to influence symptom interpretation and decision to seek screening/care. The present project describes a cohort of women's experiences, knowledge, and health behavior prior to and after a diagnosis of breast cancer. It also studies how knowledge and demographic factors are associated with level of involvement participants had in the treatment of their breast cancer. Women >18 years who have been diagnosed and treated for breast cancer within 10 years were recruited in Pittsburgh, PA, through the Healthy People Cohort Registry, a database of volunteers from the community, and Brooklyn, NY, through the American Cancer Society breast cancer survivor database. Subsequent to institutional ethics approval, a questionnaire was administered by mail and through an electronic interactive format. The study included 124 breast cancer survivors, one-quarter of whom were of African ancestry. Roughly half of the women indicated that their overall knowledge of breast cancer was limited before diagnosis; no significant association between overall knowledge before diagnosis and stage at diagnosis or an active role of the patient in treatment choices was observed. Two-third of the women reported using personal research on internet, books, and other media to increase knowledge on breast cancer after diagnosis; the improvement of knowledge was associated with an active role in therapy choice. White women's self report of breast cancer knowledge prior to diagnosis was higher than that of women of African origin (p = 0.03); the latter experienced more delays in getting results about the diagnosis (p = 0.002), in starting treatment (p = 0.03), and in having treatment available at local facilities (p = 0.007) than white women. White women were more likely to improve their knowledge through their own research (p = 0.08) and through the contribution of their physician (p = 0.06) than women of African origin.There is still a need for addressing breast cancer knowledge among black women, and improvement in physician emotional support and in their contribution to the patient's knowledge is necessary. These efforts may have a positive impact on breast cancer knowledge among black women in the US. | Decision Making | Humans | Patient Participation | Questionnaires | OK |
| Jessica | Ching | Khay-Guan | Yeoh | Deng-Chyang | Wu | Gastrointestinal Endoscopy | 2012-07-00 Jul 2012 | BACKGROUND: The rapid increase in the incidence of colorectal cancer (CRC) in the Asia-Pacific region in the past decade has resulted in recommendations to implement mass CRC screening programs. However, the knowledge of screening and population screening behaviors between countries is largely lacking. OBJECTIVE: This multicenter, international study investigated the association of screening test participation with knowledge of, attitudes toward, and barriers to CRC and screening tests in different cultural and sociopolitical contexts. METHODS: Person-to-person interviews by using a standardized survey instrument were conducted with subjects from 14 Asia-Pacific countries/regions to assess the prevailing screening participation rates, knowledge of and attitudes toward and barriers to CRC and screening tests, intent to participate, and cues to action. Independent predictors of the primary endpoint, screening participation was determined from subanalyses performed for high-, medium-, and low-participation countries. RESULTS: A total of 7915 subjects (49% male, 37.8% aged 50 years and older) were recruited. Of the respondents aged 50 years and older, 809 (27%) had undergone previous CRC testing; the Philippines (69%), Australia (48%), and Japan (38%) had the highest participation rates, whereas India (1.5%), Malaysia (3%), Indonesia (3%), Pakistan (7.5%), and Brunei (13.7%) had the lowest rates. Physician recommendation and knowledge of screening tests were significant predictors of CRC test uptake. In countries with low-test participation, lower perceived access barriers and higher perceived severity were independent predictors of participation. Respondents from low-participation countries had the least knowledge of symptoms, risk factors, and tests and reported the lowest physician recommendation rates. "Intent to undergo screening" and "perceived need for screening" was positively correlated in most countries; however, this was offset by financial and access barriers. LIMITATIONS: Ethnic heterogeneity may exist in each country that was not addressed. In addition, the participation tests and physician recommendation recalls were self-reported. CONCLUSIONS: In the Asia-Pacific region, considerable differences were evident in the participation of CRC tests, physician recommendations, and knowledge of, attitudes toward, and barriers to CRC screening. Physician recommendation was the uniform predictor of screening behavior in all countries. Before implementing mass screening programs, improving awareness of CRC and promoting the physicians' role are necessary to increase the screening participation rates. | Male | Humans | Patient Participation | Young Adult | OK |
| Jeanne F. | Duffy |  |  |  |  | Sleep Medicine | 2012-06-00 Jun 2012 | OBJECTIVE: To determine whether an accurate circadian phase assessment could be obtained from saliva samples collected by patients in their home. METHODS: Twenty-four individuals with a complaint of sleep initiation or sleep maintenance difficulty were studied for two evenings. Each participant received instructions for collecting eight hourly saliva samples in dim light at home. On the following evening they spent 9h in a laboratory room with controlled dim (<20 lux) light where hourly saliva samples were collected. Circadian phase of dim light melatonin onset (DLMO) was determined using both an absolute threshold (3 pg ml(-1)) and a relative threshold (two standard deviations above the mean of three baseline values). RESULTS: Neither threshold method worked well for one participant who was a "low-secretor". In four cases the participants' in-lab melatonin levels rose much earlier or were much higher than their at-home levels, and one participant appeared to take the at home samples out of order. Overall, the at-home and in-lab DLMO values were significantly correlated using both methods, and differed on average by 37 (± 19)min using the absolute threshold and by 54 (± 36)min using the relative threshold. CONCLUSIONS: The at-home assessment procedure was able to determine an accurate DLMO using an absolute threshold in 62.5% of the participants. Thus, an at-home procedure for assessing circadian phase could be practical for evaluating patients for circadian rhythm sleep disorders. | Male | Humans | Patient Participation | Young Adult | OK |
| Kathleen M. | Fairfield | Bethany S. | Gerstein | Carrie A. | Levin | American Journal of Preventive Medicine | 2014-09-00 Sep 2014 | BACKGROUND: Clinicians are encouraged to support patients in achieving shared decision making (SDM) for cancer screening. PURPOSE: To describe decision making processes and outcomes for cancer screening discussions. METHODS: A 2011 national Internet survey of adults aged ≥50 years who made cancer screening decisions (breast, BrCa; colorectal, CRC; prostate, PCa) within the previous 2 years was conducted. Participants were asked about their perceived cancer risk; how informed they felt about cancer tests; whether their healthcare provider addressed pros/cons of testing, presented the option of no testing, and elicited their input; whether they were tested; and their confidence in the screening decision. Data were analyzed in 2013-2014 with descriptive statistics and logistic regression. RESULTS: Overall, 1,134 participants (477 men, 657 women) aged ≥50 years made cancer screening decisions, and 1,098 (354, BrCa; 598, CRC; 146, PCa) decisions were discussed with a healthcare provider. Most discussions (51%-67%) addressed pros of screening some or a lot, but few (7%-14%) similarly addressed cons. For all cancer screening decisions, providers usually (63%-71%) explained that testing was optional, but less often asked women (43%-57%) than men (70%-71%) whether they wanted testing. Only 27%-38% of participants reported SDM, 69%-93% underwent screening, and 55%-76% would definitely make the same decision again. Perceived high/average cancer risk and feeling highly informed were associated with confidence in the screening decision. CONCLUSIONS: Discussions often failed to provide balanced information and meet SDM criteria. Supporting SDM could potentially improve the quality of cancer screening decisions. | | | | | OK |
|  |  |  |  |  |  | Santé Publique (Vandoeuvre-Lès-Nancy, France) | 2012-02-00 2012 Jan-Feb | In the health sector, lay expertise refers to two distinct but related phenomena: experiential expertise, i.e. expertise based on the experience of a specific condition, and medical-scientific expertise. A significant part of the activities of patient organizations are devoted to developing both forms of expertise: on the one hand, they collect, shape, analyze their members' testimonies, conduct surveys and produce statistics; on the other hand, they provide a scientific watch, synthesize the academic literature, publish documents for the public or organize conferences. This two-fold expertise is mobilized in actions directed both at empowering of the individual patient as well as at shaping health policies: therefore it contributes to health democracy, understood in the double sense used in the March 4th 2002 Act, i.e. as participation of individuals to decisions regarding their own health and as participation of patients' and users' representatives to the governance of health. | Humans | Patient Participation | Evidence-based medicine | Health Knowledge, Attitudes, Practice | OK |
| Gert P. | Westert | Diana Mj | Delnoij |  |  | BMC health services research | 2011-00-00 2011 | BACKGROUND: Legislation demands the establishment of client councils in Dutch nursing homes and residential care facilities. The members of those councils are residents or their representatives. Client councils have the right to participate in the strategic management of long-term care facilities. More specifically, they need to be consulted regarding organisational issues and a right to consent on issues regarding daily living of residents, including CQ-index research. CQ-index research concerns a method that measures, analyses and report clients' experiences about the quality of care. Research questions were: 'Do client councils exercise their rights to be consulted and to give their consent?' and 'What is the role of client councils in the process of measuring clients' experiences with the CQ-index and what is their opinion about the CQ-index?' METHODS: Postal questionnaires were sent to members of 1,540 client councils of Dutch nursing homes and residential care facilities. The questionnaire focussed on background information and client councils' involvement in decision-making and strategic management. RESULTS: The response rate was 34% (n = 524). Most councils consisted of seven members (range: 5 to 12 members). One out of four members participating in the client councils were clients themselves. Although councils have a legal right to be consulted for organisational issues like finance, vision, annual report, and accommodation, less than half the councils (31-46%) reported that they exercised this right. The legal right to consent was perceived by 18 to 36% of the councils regarding client care issues like food and drink, complaints registration, respectful treatment, and activities. For CQ-index research, only 18% of the client councils perceived a right to consent. Their rights to choose an approved contractor -who performs CQ-index research- and indicating improvement priorities, were hardly used. CONCLUSIONS: Client councils play a rather passive role in determining the policy on quality of long-term care. Therefore, specific attention and actions are needed to create a more proactive attitude in councils towards exercising their rights, which are already supported by legislation. | Male | Humans | Patient Participation | Patient Satisfaction | OK |
|  |  |  |  |  |  | Clinical Trials (London, England) | 2013-08-00 Aug 2013 | BACKGROUND: Completion of a randomised controlled trial is one way by which the resident research requirement can be met in Canadian obstetrics and gynaecology programmes. However, little is known about the specific challenges of performing clinical trials within the specialty, let alone as a resident project. PURPOSE: A resident-led randomised controlled trial comparing two methods of labour induction at term was halted due to insufficient patient enrolment. A structured review of the study design and recruitment process was conducted to identify factors contributing to poor recruitment. METHODS: In addition to completing a literature review and internal review by the research team, we surveyed obstetricians and residents regarding recruitment efforts and barriers to participation. We solicited feedback on trial design and the expectations of clinicians with respect to participation in research studies. RESULTS: Eight obstetricians (67%) and 13 residents (93%) responded to the survey. All were able to identify eligible patients, though only 60% had invited one or more patients to participate during the recruitment period. Failure to consider trial participation and excessive clinical workload were the most commonly cited barriers for clinicians. Resistance to the test intervention was the major barrier to patient participation. Several residents cited a lack of personal incentive to recruit patients as a significant barrier. LIMITATIONS: The research team was unable to contact patients directly, thus limiting the scope of our review to our internal methods and feedback from clinicians. CONCLUSIONS: Poor recruitment in a resident-led clinical trial in obstetrics resulted from multiple coexisting factors. A structured review provided valuable insight for the research team. Academic clinicians and trainees in all specialties should be encouraged to share their experiences in the hope of improving the likelihood of success in future research endeavours. | Humans | Questionnaires | Female | pregnancy | OK |
| Marthe R. | Gold |  |  |  |  | Journal of Health Care for the Poor and Underserved | 2010-08-00 Aug 2010 | Patient activation refers to people's ability to engage in self-management of their health and health care. We assessed the performance of the Patient Activation Measure (PAM) for patients attending three inner-city health centers and compared resultant scores with those of the general U.S. adult population. We approached 801 patients and 527 (65.8%) participated; the majority were Latino(a) or African American/Black. No differences in activation were seen according to age. Males and more educated patients were more activated (p<.05) and patients with better self-rated health and adequate health literacy were more activated than their counterparts (p<.001). Patterns of scores resembled those of the U.S. general population for educational attainment and self-rated health but not for gender and age. Compared with the general population, more patients were characterized as level 1 (least activated). Developing strategies that enhance patient activation is critical to improving health outcomes, particularly in less advantaged populations. | Male | Humans | Patient Participation | Questionnaires | OK |
| Signe A. | Flottorp | Per S. | Stensland |  |  | Family Practice | 2011-04-00 avril 2011 | Background: GPs consider individual lifestyle counselling as part of their obligation. There is a lack of knowledge about how such counselling is done. Objective: To investigate what tools GPs utilize in individual consultations concerning lifestyle change. Methods: Qualitative analysis of six focus groups with 50 GPs sharing and commenting each other’s case stories. Results: To enhance change of lifestyle, GPs adjusted the organization of their practice and utilized visualization tools. They established doctor–patient relationships based on shared decision making and trust, and gave their patients advice and tips to accomplish change, but also used paternalistic approaches and rhetoric manipulation. Conclusions: GPs use a variety of tools in consultations about lifestyle change. A patient-centred approach is shown, but GPs also deliberately use distressing communication tools. (PsycINFO Database Record (c) 2013 APA, all rights reserved). (journal abstract) | physicians | general practitioners | Professional Consultation | lifestyle consultation | OK |
| Signe A. | Flottorp | Per S. | Stensland |  |  | Family Practice | 2011-04-00 Apr 2011 | BACKGROUND: GPs consider individual lifestyle counselling as part of their obligation. There is a lack of knowledge about how such counselling is done. OBJECTIVE: To investigate what tools GPs utilize in individual consultations concerning lifestyle change. METHODS: Qualitative analysis of six focus groups with 50 GPs sharing and commenting each other's case stories. RESULTS: To enhance change of lifestyle, GPs adjusted the organization of their practice and utilized visualization tools. They established doctor-patient relationships based on shared decision making and trust, and gave their patients advice and tips to accomplish change, but also used paternalistic approaches and rhetoric manipulation. CONCLUSIONS: GPs use a variety of tools in consultations about lifestyle change. A patient-centred approach is shown, but GPs also deliberately use distressing communication tools. | Male | Humans | Questionnaires | general practitioners | OK |
| Aung Soe | Tin | Saskia | van der Erf | Jeremy Fung Yen | Lim | BMC nephrology | 2012-00-00 2012 | BACKGROUND: Kidney disease is the 9th leading cause of death in Singapore. While preventive effects have focused on early detection and education, little is known about the knowledge level of chronic kidney disease (CKD) locally. We seek to evaluate the knowledge of CKD among primary care patients. METHODS: We conducted a cross-sectional survey of a convenience sample of 1520 patients from 3 primary care centers. Those with existing CKD or on dialysis were excluded. Knowledge was assessed based on 7 questions on CKD in the self-administered questionnaire. One point was given for each correct answer with a maximum of 7 points. RESULTS: 1435 completed all 7 questions on CKD. Mean age was 48.9 ± 5.0 (SD) years. 50.9% were male. 62.3% had a secondary and below education and 52.4% had a monthly household income of ≤ $2000. 43.7% had chronic diseases. Mean score was 3.44 ± 1.53 (out of a maximum of 7). Median score was 4. In multivariate logistic regression, being older {>60 years [Odds Ratio (OR) 0.50, 95% Confidence Interval (CI) 0.32-0.79]; 40-60 years (OR 0.62, 95% CI 0.43,0.89)}, less educated [up to primary education (OR 0.33, 95% CI 0.22-0.49)], having a lower monthly household income [<S$2000 (OR 0.41, 95% CI 0.26-0.66); S$2000-4999 (OR 0.53, 95%CI, 0.33-0.83)], and being non-professionals [OR 0.66, 95% CI 0.43-0.99] (all p < 0.05)] were likely to score less. CONCLUSION: This suggests that CKD education should be targeted at older patients with lower education and lower socioeconomic status. | Male | Humans | Patient Participation | Female | OK |
| Mesach | Mubiru | Joseph | Konde-Lule | Frank | Cobelens | PloS One | 2010-00-00 2010 | BACKGROUND: Early detection and treatment of tuberculosis cases are the hallmark of successful tuberculosis control. We conducted a cross-sectional study at public primary health facilities in Kampala city, Uganda to quantify diagnostic delay among pulmonary tuberculosis (PTB) patients, assess associated factors, and describe trajectories of patients' health care seeking. METHODOLOGY/PRINCIPAL FINDINGS: Semi-structured interviews with new smear-positive PTB patients (≥ 15 years) registered for treatment. Between April 2007 and April 2008, 253 patients were studied. The median total delay was 8 weeks (IQR 4-12), median patient delay was 4 weeks (inter-quartile range [IQR] 1-8) and median health service delay was 4 weeks (IQR 2-8). Long total delay (>14 weeks) was observed for 61/253 (24.1%) of patients, long health service delay (>6 weeks) for 71/242 (29.3%) and long patient delay (>8 weeks) for 47/242 (19.4%). Patients who knew that TB was curable were less likely to have long total delay (adjusted Odds Ratio [aOR] 0.28; 95%CI 0.11-0.73) and long patient delay (aOR 0.36; 95%CI 0.13-0.97). Being female (aOR 1.98; 95%CI 1.06-3.71), staying for more than 5 years at current residence (aOR 2.24 95%CI 1.18-4.27) and having been tested for HIV before (aOR 3.72; 95%CI 1.42-9.75) was associated with long health service delay. Health service delay contributed 50% of the total delay. Ninety-one percent (231) of patients had visited one or more health care providers before they were diagnosed, for an average (median) of 4 visits (range 1-30). All but four patients had systemic symptoms by the time the diagnosis of TB was made. CONCLUSIONS/SIGNIFICANCE: Diagnostic delay among tuberculosis patients in Kampala is common and long. This reflects patients waiting too long before seeking care and health services waiting until systemic symptoms are present before examining sputum smears; this results in missed opportunities for diagnosis. | Time Factors | Male | Humans | Patient Participation | OK |
|  |  |  |  |  |  | The Health Service Journal | 2014-03-21 Mar 21, 2014 | | Decision Making | Humans | Patient Participation | Great Britain | OK |
| Hermien | Hartog | Aebele Mink | van der Molen | Corry | van der Sluis | Disability and Rehabilitation | 2013-08-00 Aug 2013 | PURPOSE: To evaluate all functional aspects of patients with longitudinal radial dysplasia and to clarify the relationship between body functions on the one hand and limitations in activity and participation on the other hand. METHODS: Thirty-one arms of seventeen adult patients with longitudinal radial dysplasia were analysed. Body function was assessed by measuring grip and pinch strength and active range of motion (ROM) of the hand. Activities were measured using the "Sequential Occupational Dexterity Assessment ", to measure perceived restrictions in participation the "Impact on Participation and Autonomy questionnaire" was used. Relationships between severity of dysplasia, body function, participation and activity were determined. RESULTS: Patients with a severe type scored significantly lower in body function scores than patients with a mild form. Patients with limited active finger joint motion performed worse on activities. We found no significant differences in activity and participation between mild or severe types and found no correlation in participation scores. CONCLUSION: Although considerable restrictions in joint mobility and strength were revealed, little or no limitations on the activity and participation level were found. Limitations in body functions hardly influenced capacity on activity level and did not influence participation in societal roles. | Male | Humans | Patient Participation | Questionnaires | OK |
| J. | Kasper | F. | Ufer | W. G. | Elias | Multiple Sclerosis (Houndmills, Basingstoke, England) | 2013-06-00 Jun 2013 | BACKGROUND: Balancing treatment benefits and risks is part of a shared decision-making process before initiating any treatment in multiple sclerosis (MS). Patients understand, appreciate and profit from evidence-based patient information (EBPI). While these processes are well known, long-term risk awareness and risk processing of patients has not been studied. Mitoxantrone treatment in MS is associated with long-term major potential harms - leukaemia (LK) and cardiotoxicity (CT). The risk knowledge and perception among patients currently or previously treated with mitoxantrone is unknown. OBJECTIVES: The objective of this article is to conduct a retrospective cohort study in greater Hamburg, Germany, to estimate risk awareness and perception in MS patients treated with mitoxantrone. METHODS: MS patients with at least one dose of mitoxantrone between 1991 and 2010 from six major MS centres in greater Hamburg received a questionnaire assessing risk awareness and perception as well as a written EBPI about mitoxantrone-associated LK and CT. RESULTS: Fifty-one per cent in the cohort of n = 575 patients returned the questionnaire. Forty per cent correctly estimated the risk of LK (CT 16%); 56% underestimated the risk (CT 82%). Reading the information increased the accuracy of LK risk estimation, and patients did not report an increase of worries. The EBPI was appreciated and recommended by 85%. CONCLUSION: Risk awareness of mitoxantrone-treated patients is insufficient, but can be increased by EBPI without increasing worries. Continued patient information during and after treatment should be implemented in management algorithms. | Male | Humans | Questionnaires | Female | OK |
| Dirk | Knol | Ger | van Rens |  |  | Optometry and Vision Science: Official Publication of the American Academy of Optometry | 2012-02-00 Feb 2012 | PURPOSE: This qualitative study investigates possible differences in identified rehabilitation needs indicated by the usual intake procedures at a Multidisciplinary Rehabilitation Center (MRC) for visually impaired persons compared with those indicated by the use of a structured Dutch version [based on the International Classification of the Disability, Functioning and Health (ICF)] of the Activity Inventory (D-AI). METHODS: Twenty patients who enrolled at the MRC received a D-AI assessment by telephone, in addition to the usual intake. All patients received usual care, based on rehabilitation needs identified by the usual intake procedure at the MRC. Rehabilitation needs identified at the MRC were obtained from patient files retrospectively and were compared with rehabilitation needs identified by the D-AI. RESULTS: The mean number of rehabilitation needs reported in the patient files was 6.9 (± 5.1) vs. 24.0 (± 11.2) using the D-AI. Only 22.6% (± 14.3) of the rehabilitation needs identified by the D-AI were present in the patient files; 79.3% (± 28.2) of the rehabilitation needs reported in the patient files were identified by the D-AI. Overall agreement corrected for chance between both intake methods revealed a fair Cohen kappa of 0.27. CONCLUSIONS: At the MRC, more needs were revealed using the D-AI compared with the usual intake procedure. The systematic character of the D-AI prevents important topics being overlooked. In the usual intake, it was not clear whether needs were investigated from the patient's perspective. This may hamper (medical) communication and shared decision making about the rehabilitation program that needs to be followed. Moreover, using the unstructured information from the patient files makes it difficult to evaluate rehabilitation outcomes. With the D-AI, although an extensive overview of rehabilitation needs is produced, it remains difficult to focus on the most relevant needs. However, after assessment with the D-AI, all aspects of the ICF, Disability, and Health scheme can be discussed in a process of shared decision making, which leads to the final determination of rehabilitation goals. | Male | Humans | Questionnaires | Female | OK |
| Fiona M. | Foley | Paul B. | Fitzgerald | Steve | Ellen | Australian Family Physician | 2010-12-00 Dec 2010 | BACKGROUND: Patients are not always fully aware of the details of their assessment and management plan detailed in the letter sent from the specialist to the general practitioner following referral. One approach to solving this problem is for the specialist to copy the GP reply letter to the patient. OBJECTIVE: To determine whether receiving a copy of the GP reply letter improves outcomes in patients referred by their GP for a psychiatric assessment. METHODS: A single blinded randomised control trial comparing outcomes following a one-off consultation for a depressive and/or anxiety disorder in patients who received the GP reply letter sent from a community mental health service, to patients who did not. RESULTS: Data was collected for 21 letter recipients and 18 control participants. A significant group by time interaction found total DASS-21 scores improved to a greater extent for the letter recipient group, no significant difference in adherence was found. | Male | Humans | Patient Participation | Female | OK |
| Sandra | Schwaner | Abdullah | Al-Osaimi | Saher | Sabri | Journal of Clinical Imaging Science | 2014-00-00 2014 | OBJECTIVES: Endoscopic experience in the management of duodenal varices (DVs) is limited and challenging given the anatomic constraints and limited experience. The endovascular management of DVs is not yet established and the controversy of whether to manage them by decompression with a transjugular intrahepatic portosystemic shunt (TIPS) or by transvenous obliteration is unresolved. In the literature, the 6-12 month rebleeding rate of DVs after TIPS is 21-37% and after transvenous obliteration is 13%. The purpose of the study is to evaluate the clinical outcome of combined TIPS decompression and transvenous obliteration/sclerosis. MATERIALS AND METHODS: This is a retrospective study (case series) of two institutions, evaluating patients who underwent TIPS and/or transvenous obliteration/sclerosis for bleeding DVs (from January 2009 to June 2013). TIPS was performed according to a standard procedure using covered stents. Transvenous obliteration (variceal sclerosis) from the systemic and/or portal venous circulation was performed utilizing 3% sodium tetradecyl sulfate foam. Transvenous obliteration was commonly augmented with coils and/or vascular plugs. Technical (technical success of establishing TIPS and completely obliterating the DVs) and clinical outcomes (rebleeding rate and survival) were evaluated. RESULTS: Five patients with liver cirrhosis presenting with bleeding DVs were included in the study with all eventually (and coincidentally) receiving TIPS and transvenous obliteration. Two of the five patients underwent concomitant TIPS and transvenous obliteration in the same procedural setting. However, three patients underwent transvenous obliteration due to bleeding despite a patent TIPS that had been previously placed. The average time from TIPS placement to transvenous obliteration was 125 days (range: 3-324 days). After having both procedures, there was no rebleeding in the patients during a mean follow-up period of 22 months (6-50 months). Coils and/or metallic vascular plugs were used to augment the sclerosant obliteration in four of five patients. CONCLUSION: The combination of TIPS decompression and foam sclerosant transvenous obliteration appears to be effective in preventing rebleeding in this limited case series and compares favorably with the existing evidence for either approach [TIPS or balloon-occluded retrograde transvenous obliteration (BRTO)] alone. | | | | | OK |
| Wigdan | Al-Sukhni | Heidi | Rothenmund | David | Urbach | Canadian Journal of Surgery. Journal Canadien De Chirurgie | 2011-02-00 Feb 2011 | BACKGROUND: Pancreatic adenocarcinoma (PA) is largely incurable, although recent progress has been made in the safety of surgery for PA and in adjuvant and palliative chemotherapy. The purpose of this study was to describe the management of PA in Ontario, Canada. METHODS: The Pathology Information Management System (PIMS), which uses electronic pathology reporting (E-path), was used to rapidly identify and recruit patients based on a pathologic diagnosis of PA between 2003 and 2006. Patients were mailed questionnaires for additional data. RESULTS: The patient participation rate was 26% (351 of 1325). Nonresponders were more likely to be older than 70 years (43% v. 28%, p < 0.001) and to have received treatment in nonacademic centres (53% v. 34%, p < 0.001). Fifty-four percent of responders underwent a potentially curative operation, and most (77%) were 70 years or younger (p = 0.03). Completed resections were documented in 83% of patients who underwent exploratory surgery with curative intent; 17% of patients had unresectable and/or metastatic disease at laparotomy. Of the completed resections, 24% were performed in nonacademic centres with a 32% positive margin rate; 76% were performed in academic centres with a 29% positive margin rate (p = 0.84). Resections with curative intent were less frequently aborted in academic centres (10% v. 33%, p < 0.001). Of the patients who responded to our questionnaire, 43% received chemotherapy and 7% participated in clinical trials. CONCLUSION: Despite using PIMS and E-path, the response rate for this study was low (< 30%). Nonresponders were older and more commonly treated in nonacademic centres. Patients undergoing surgery in academic centres had higher resection rates. The rate of adjuvant and palliative chemotherapy was stage-dependent and low. | Male | Humans | Questionnaires | Female | OK |
| Sally | Taylor | Naheed | Hanif | Emma | Podmore | Psycho-Oncology | 2012-01-00 Jan 2012 | BACKGROUND: Implementation of guidance on assessment and management of psychosocial and supportive-care problems or needs will be successful only if consideration is given to existing skills, experience and expectations of staff and patients. This study examines the roles and responsibilities of staff, patients and families in relation to management of social difficulties and proposes a pathway for response. METHODS: A qualitative study was performed using staff and patient interviews. Seventeen doctors and 16 nurses were interviewed using patient scenarios and a support service questionnaire. Patients (n = 41) completed a screening questionnaire (the Social Difficulties Inventory) and were interviewed. Interviews were audio-recorded, transcribed and subjected to a Framework analysis. Analysis examined (1) actions taken by staff and patients in response to social difficulties, (2) reasons given for action taken and (3) perceptions of staff and patients of who was responsible for taking action. RESULTS: Staff were confident concerning clinically related issues (i.e. mobility) but more hesitant concerning difficulties related to money, work and family concerns. Patients liked to cope with problems on their own where possible, would have liked information or support from staff but were uncertain how to access this. Results led to development of a hierarchy of interventions in response to detected social difficulties. DISCUSSION: For routine assessment of social difficulties, patients, nurses and doctors will have to work collaboratively, with nurses taking a lead in discussion. For specific clinically related problems doctors would play a more primary role. | Male | Humans | Patient Participation | Questionnaires | OK |
| Christa J. | Caruso |  |  |  |  | Nursing Research | 2013-12-00 2013 Nov-Dec | BACKGROUND: The desire to retain personal control over self and life circumstances continues into old age; it exists in tension with late-life vulnerabilities. OBJECTIVES: This article investigates how older adults respond to threats against control in light of changes surrounding health and identity. METHODS: Community-dwelling African American (n = 10) and European-American older adults (n = 10), aged 70 years and older, with varied self-reported health statuses were qualitatively interviewed. Open-ended interviews explored older adults' perceptions of control and threats to control in older age. RESULTS: Three themes linked older adults' responses to threats to control. Older adults (a) proactively monitored physical and mental health, (b) maintained roles that shaped important aspects of identity, and (c) fostered personal growth and development by generative practices. Responses of participants who had difficulty countering threats to control are also offered. DISCUSSION: This study shows that the construct of control is not abstract; it is interpreted and applied by older adults in the contexts of everyday life. Respondents used personal resources honed throughout the life course to respond to threats to control. Older adults viewed control as a cultural construct with nuanced meanings that recalled past roles and current changes that occur with age. Suggestions are offered for how health professionals can assist older adults with the cognitive and emotional tasks required to deal with threats to personal control surrounding health and identity. | Male | Humans | Patient Participation | Female | OK |
| Christoph | Heesen | Sascha | Köpke | Friedemann | Geiger | PLoS ONE | 2012-04-13 avril 13, 2012 | Background: The wide scale permeation of health care by the shared decision making concept (SDM) reflects its relevance and advanced stage of development. An increasing number of studies evaluating the efficacy of SDM use instruments based on various sub-constructs administered from different viewpoints. However, as the concept has never been captured in operable core definition it is quite difficult to link these parts of evidence. This study aims at investigating interrelations of SDM indicators administered from different perspectives. Method: A comprehensive inventory was developed mapping judgements from different perspectives (observer, doctor, patient) and constructs (behavior, perception) referring to three units (doctor, patient, doctor-patient-dyad) and an identical set of SDM-indicators. The inventory adopted the existing approaches, but added additional observer foci (patient and doctor-patient-dyad) and relevant indicators hitherto neglected by existing instruments. The complete inventory comprising a doctor-patient-questionnaire and an observer-instrument was applied to 40 decision consultations from 10 physicians from different medical fields. Convergent validities were calculated on the basis of Pearson correlation coefficients. Results: Reliabilities for all scales were high to excellent. No correlations were found between observer and patients or physicians neither for means nor for single items. Judgements of doctors and patients were moderately related. Correlations between the observer scales and within the subjective perspectives were high. Inter-perspective agreement was not related to SDM performance or patient activity. Conclusion: The study demonstrates the contribution to involvement made by each of the relevant perspectives and emphasizes the need for an inter-subjective approach regarding SDM measurement. (PsycINFO Database Record (c) 2014 APA, all rights reserved). (journal abstract) | Shared decision making | Decision Making | health care | Psychometrics | OK |
|  |  |  |  |  |  | JAMA | 2012-06-06 Jun 6, 2012 | | Decision Making | Humans | Patient Participation | Physician-Patient Relations | OK |
| A. | Guttmann | A. R. | Willan | P. | Rosenbaum | Child: Care, Health and Development | 2014-03-00 Mar 2014 | BACKGROUND: Neonatal Follow-Up (NFU) programmes provide health services for families of infants at high risk of developmental problems following difficult or extremely premature birth: yet, up to 30% of families do not attend these programmes with their infants. METHODS: The study objective was to determine maternal and infant factors that predicted attendance at NFU programmes. Utilizing Andersen's Behavioural Model of Health Services Use, a prospective two-phase multi-site descriptive cohort study was conducted in three Canadian Neonatal Intensive Care Units (NICU) that refer to two affiliated NFU programmes. In Phase 1, 357 mothers completed standardized questionnaires that addressed maternal and infant factors, prior to their infants' NICU discharge. In Phase 2, attendance at NFU was followed at three time points over a 12-month period. Factors of interest included predisposing factors (e.g. demographic characteristics and social context); enabling factors (e.g. social support, travel distance, and income); and infant illness severity (i.e. needs factors). Multivariate logistic regression was used to estimate the odds ratio for each independent factor. RESULTS: Mothers parenting alone, experiencing higher levels of worry about maternal alcohol or drug use, or at greater distances from NFU were less likely to attend. Mothers experiencing higher maternal stress at the time of the infant's NICU hospitalization were more likely to attend NFU. No infant factors were predictive of NFU attendance. CONCLUSIONS: Mothers at risk of not attending NFU programmes with their infants require better identification, triage, referral and additional support to promote engagement with NFU programmes and improved quality of life for their high-risk infants. | Male | Humans | Patient Participation | Questionnaires | OK |
|  |  |  |  |  |  | The Journal of Legal Medicine | 2013-00-00 2013 | | Decision Making | Humans | Patient Participation | United States | OK |
| Christa A. | Bruce | Walter F. | Stewart |  |  | American Journal of Preventive Medicine | 2011-05-00 May 2011 | Quantitative risk (QR) formulas have been developed for multiple conditions but are not routinely used in clinical practice. Tests were made of the feasibility of an automated clinical care process for using QR in routine primary care. Several modifications were made to the Framingham Risk Score (FRS) and it was applied to routine care in three areas: (1) for risk-stratification, (2) patient education about care options, and (3) guidance on optimizing choice of care options. Evidence-based methods were used to convert the smoking status variable from a binary- to a continuous-scale format and to add variables for alcohol use and HbA1c. An automated protocol tested in 2008-2010 was successful for all three applications. At-risk patients (defined according to criteria from the Joint National Committee on Prevention, Detection, Evaluation, and Treatment of High Blood Pressure [JNC]-7 or the National Cholesterol Education Program Expert Panel on Detection, Evaluation, and Treatment of High Blood Cholesterol in Adults [adult treatment panel/ATP-III]) were automatically identified during routine encounters. Patient-reported data were obtained (n = 1826) by touchscreen questionnaire and automatically used with electronic health record (EHR) data to calculate risks on 1068 patients who had complete data. Patients were risk-stratified. Higher-risk patients viewed an interactive web-based tool and chose options to modify risk factors. Feasibility was successful for use of the FRS in the interactive web tool. | Male | Decision Making | Humans | Patient Participation | OK |
| L. Ebony | Boulware | Claire F. | Snyder |  |  | Journal of Clinical Epidemiology | 2013-08-00 Aug 2013 | OBJECTIVE: This article presents the current state of patient-reported outcome measures and explains new opportunities for leveraging the recent adoption of electronic health records to expand the application of patient-reported outcomes in both clinical care and comparative effectiveness research. STUDY DESIGN AND SETTING: Historic developments of patient-reported outcome, electronic health record, and comparative effectiveness research are analyzed in two dimensions: patient centeredness and digitization. We pose the question, "What needs to be standardized around the collection of patient-reported outcomes in electronic health records for comparative effectiveness research?" RESULTS: We identified three converging trends: the progression of patient-reported outcomes toward greater patient centeredness and electronic adaptation; the evolution of electronic health records into personalized and fully digitized solutions; and the shift toward patient-oriented comparative effectiveness research. Related to this convergence, we propose an architecture for patient-reported outcome standardization that could serve as a first step toward a more comprehensive integration of patient-reported outcomes with electronic health record for both practice and research. CONCLUSION: The science of patient-reported outcome measurement has matured sufficiently to be integrated routinely into electronic health records and other electronic health solutions to collect data on an ongoing basis for clinical care and comparative effectiveness research. Further efforts and ideally coordinated efforts from various stakeholders are needed to refine the details of the proposed framework for standardization. | Humans | Patient Participation | Patient Satisfaction | United States | OK |
| W. | Fries | P. | Reuther |  |  | Fortschritte Der Neurologie-Psychiatrie | 2013-10-00 Oct 2013 | OBJECTIVE: To date there is a lack of tools to measure participation and the already existing measures are not properly used as yet. In 2005 the IMET (Index zur Messung von Einschränkungen der Teilhabe) was developed and is able to measure the ICF associated construct participation as a generic instrument in chronic diseases. IMET and numerous instruments were applied in our own study and results were compared with results of an unpublished study. In addition, to test IMET for its use in neurorehabilitation the effects of outpatient neurorehabilitation were investigated and compared with results obtained in an inpatient setting. METHOD: In a multicentric observational study, consecutively treated patients of 6 outpatient neurorehabilitation centres were asked to fill in a questionnaire at three time points (admission and discharge in the course of rehabilitation and at 4 months follow-up). Additionally, clinical experts were asked to rate the patients' status at admission and discharge. The data were compared with results of a sample of inpatients of an unpublished study. RESULTS: The IMET seems to be the to date best instrument to measure participation in a global, ICF-defined and economic way. Especially participation, general health status and capacity in leisure time and daily routine show the biggest improvements. In comparison, the outpatients show improvements in their participation status. Participation-oriented outpatient neurorehabilitation seems to have a considerable impact on participation status in neurological patients through the course of rehabilitation. | Humans | Patient Participation | Questionnaires | Aged | Doublon |
| W. | Fries | P. | Reuther |  |  | Fortschritte Der Neurologie-Psychiatrie | 2013-10-00 Oct 2013 | OBJECTIVE: To date there is a lack of tools to measure participation and the already existing measures are not properly used as yet. In 2005 the IMET (Index zur Messung von Einschränkungen der Teilhabe) was developed and is able to measure the ICF associated construct participation as a generic instrument in chronic diseases. IMET and numerous instruments were applied in our own study and results were compared with results of an unpublished study. In addition, to test IMET for its use in neurorehabilitation the effects of outpatient neurorehabilitation were investigated and compared with results obtained in an inpatient setting. METHOD: In a multicentric observational study, consecutively treated patients of 6 outpatient neurorehabilitation centres were asked to fill in a questionnaire at three time points (admission and discharge in the course of rehabilitation and at 4 months follow-up). Additionally, clinical experts were asked to rate the patients' status at admission and discharge. The data were compared with results of a sample of inpatients of an unpublished study. RESULTS: The IMET seems to be the to date best instrument to measure participation in a global, ICF-defined and economic way. Especially participation, general health status and capacity in leisure time and daily routine show the biggest improvements. In comparison, the outpatients show improvements in their participation status. Participation-oriented outpatient neurorehabilitation seems to have a considerable impact on participation status in neurological patients through the course of rehabilitation. | Humans | Patient Participation | Questionnaires | Aged | OK |
|  |  |  |  |  |  | Archives of Physical Medicine and Rehabilitation | 2010-09-00 Sep 2010 | The Rehabilitation Research and Training Center on Measuring Rehabilitation Outcomes and Effectiveness sponsored a 2-day International Symposium on Measurement of Participation in Rehabilitation Research in October 2008 as a preconference to the annual meeting of the American Congress of Rehabilitation Medicine and the American Society of Neurorehabilitation. The aims of the symposium were to highlight the state of the art in the measurement of participation and discuss its utility as an outcome measure for persons with disabilities by examining the construct of participation and its measurement. Plenary presentations and group discussions were organized around 4 key issues: (1) conceptualization, (2) operationalization, (3) environmental influences, and (4) personal characteristics. The full set of symposium articles, including recommendations for future research, appears in this supplement to Archives of Physical Medicine and Rehabilitation. | Humans | Patient Participation | Psychometrics | Rehabilitation | OK |
|  |  |  |  |  |  | Archives of Physical Medicine and Rehabilitation | 2010-09-00 Sep 2010 | The goals of this article are to describe participation as a transaction and issues involved in measuring and intervening using this transactional approach; describe ecologic and systems-based theoretic approaches for conceptualizing person-task-environment transactions; and illustrate examples of an exploratory strategy, radar plots, as a clinical tool for rehabilitation professionals to show this interaction and use it to inform participation-focused interventions with people with disabilities in rehabilitation settings. Participation necessarily occurs at the intersection of what the person can do, wants to do, has the opportunity to do, and is not prevented from doing. It is a transaction that occurs at the nexus of the person-task-environment. Measurement of participation should capture this transactive nature. Radar plots are part of a group of graphic displays frequently referred to as exploratory data analysis. In situations in which theory is not well developed, exploratory techniques such as radar plots may hold promise as ways to explore better the relationship among variables. This article describes strengths and limitations of radar plots and presents an example with data from the Community Participation Database. | Humans | Patient Participation | Rehabilitation | Outcome Assessment (Health Care) | OK |
|  |  |  |  |  |  | Zeitschrift Für Evidenz, Fortbildung Und Qualität Im Gesundheitswesen | 2012-00-00 2012 | The article comments on Shared Decision Making (SDM) research from a different perspective, i.e. psychotherapy research. Psychotherapy research had to face similar challenges, both conceptual as well as methodological. Meanwhile, the generic model of psychotherapy has helped researchers to put single results into a perspective that also might be helpful to the SDM field. Since SDM research increasingly focuses upon process perspectives directed towards the doctor-patient dyad, we propose measures and constructs from interpersonal theory as useful tools to be introduced into SDM research. A selection of these measures and their potential within SDM research is described. | Decision Making | Humans | Patient Participation | Physician-Patient Relations | OK |
| Karen | Sepucha | Glyn | Elwyn | France | Légaré | Zeitschrift Für Evidenz, Fortbildung Und Qualität Im Gesundheitswesen | 2011-00-00 2011 | The last years have seen a clear move towards shared decision making (SDM) and increased patient involvement in many countries. However, as the field of SDM research is still relatively young, new instruments for the measurement of (shared) decision making (process, outcome and surrounding elements) are constantly being developed. Thus, the aims of this structured review were to give an update on current developments regarding the measurement in the field of SDM, as well as to give a short overview of published and unpublished instruments. We conducted an electronic literature search in PubMed and the Web of Science database, performed hand searches of relevant journals and contacted key authors in the field. We found eight scales that have been subjected to further psychometric testing, eleven new and psychometrically tested instruments and nine developments that are still in the publishing process. The results show that there is a trend towards measuring SDM processes from a dyadic approach (assessing both the patient's and the clinician's perspective). More and more scales have been developed and tested in languages other than English, which indicates the growing research efforts in various countries. While reliability of most scales is good, they differ in their extent of validation. Further psychometric testing is needed, as well as the development of a theoretical measurement framework in order to improve consistency of measured constructs across research groups. | Humans | Patient Participation | Psychometrics | health policy | OK |
| Barbara E. | Gibson |  |  |  |  | Disability and Rehabilitation: Assistive Technology | 2013-01-00 janvier 2013 | Purpose: This article proposes a conceptual underpinning for and examines the validity of the Youth Evaluation of Products (YEP) scale when used to measure the AT device attitudes of youth. Method: Consumer socialization is promoted to improve the utility of an AT device selection framework for children and youth and inform the development of the YEP scale. A descriptive, qualitative mixed-methods design explored the validity of the scale when used by six manual wheelchair users, aged 11–14 years, to evaluate a new pushrim-activated, power-assisted wheelchair. Three youth participated in a subsequent focus group to share how they assessed wheelchair products. Results: The items and dimensions of the YEP scale corresponded well to participants’ views about wheelchairs. Conclusions: Consumer socialization provides a new way to understand the developing role of youth as AT consumers and the YEP scale is emerging as a way to measure their product attitudes. When integrated with contemporary thinking about the assessment and selection of AT devices, providers and parents may be better able to incorporate the product perspectives of youth during this shared decision-making process. (PsycINFO Database Record (c) 2013 APA, all rights reserved). (journal abstract) | Psychometrics | test validity | test reliability | Product Design | Doublon |
| Barbara E. | Gibson |  |  |  |  | Disability and Rehabilitation. Assistive Technology | 2013-01-00 Jan 2013 | PURPOSE: This article proposes a conceptual underpinning for and examines the validity of the Youth Evaluation of Products (YEP) scale when used to measure the AT device attitudes of youth. METHOD: Consumer socialization is promoted to improve the utility of an AT device selection framework for children and youth and inform the development of the YEP scale. A descriptive, qualitative mixed-methods design explored the validity of the scale when used by six manual wheelchair users, aged 11-14 years, to evaluate a new pushrim-activated, power-assisted wheelchair. Three youth participated in a subsequent focus group to share how they assessed wheelchair products. RESULTS: The items and dimensions of the YEP scale corresponded well to participants' views about wheelchairs. CONCLUSIONS: Consumer socialization provides a new way to understand the developing role of youth as AT consumers and the YEP scale is emerging as a way to measure their product attitudes. When integrated with contemporary thinking about the assessment and selection of AT devices, providers and parents may be better able to incorporate the product perspectives of youth during this shared decision-making process. [Box: see text]. | Male | Humans | Patient Satisfaction | Questionnaires | OK |
| Dionne S. | Kringos | Evelyne | De Ryck | Stefan | Greß | Quality in Primary Care | 2013-00-00 2013 | BACKGROUND: The Quality and Costs of Primary Care in Europe (QUALICOPC) study aims to analyse and compare how primary health care systems in 35 countries perform in terms of quality, costs and equity. This article answers the question 'How can the organisation and delivery of primary health care and its outcomes be measured through surveys of general practitioners (GPs) and patients?' It will also deal with the process of pooling questions and the subsequent development and application of exclusion criteria to arrive at a set of appropriate questions for a broad international comparative study. METHODS: The development of the questionnaires consisted of four phases: a search for existing validated questionnaires, the classification and selection of relevant questions, shortening of the questionnaires in three consensus rounds and the pilot survey. Consensus was reached on the basis of exclusion criteria (e.g. the applicability for international comparison). Based on the pilot survey, comprehensibility increased and the number of questions was further restricted, as the questionnaires were too long. RESULTS: Four questionnaires were developed: one for GPs, one for patients about their experiences with their GP, another for patients about what they consider important, and a practice questionnaire. The GP questionnaire mainly focused on the structural aspects (e.g. economic conditions) and care processes (e.g. comprehensiveness of services of primary care). The patient experiences questionnaire focused on the care processes and outcomes (e.g. how do patients experience access to care?). The questionnaire about what patients consider important was complementary to the experiences questionnaire, as it enabled weighing the answers from the latter. Finally, the practice questionnaire included questions on practice characteristics. DISCUSSION: The QUALICOPC researchers have developed four questionnaires to characterise the organisation and delivery of primary health care and to compare and analyse the outcomes. Data collected with these instruments will allow us not only to show in detail the variation in process and outcomes of primary health care, but also to explain the differences from features of the (primary) health care system. | Humans | Patient Participation | Patient Satisfaction | Questionnaires | OK |
| Sara | Muller | Elaine | Nicholls | Emma L. | Healey | Arthritis Care & Research | 2011-11-00 Nov 2011 | | Humans | Patient Participation | patient care | Social Perception | OK |
| Robert L. | Comis | Sandi W. | Smith | Kami J. | Silk | Health Communication | 2011-01-00 Jan 2011 | Researchers, practitioners, and participants in cancer clinical trials must have a clear understanding of clinical trials if participation in them is to be solicited ethically and effectively. A valid and reliable measure of cancer clinical trial understanding did not exist prior to a 2005 study conducted for the Coalition of Cooperative Cancer Groups. This report outlines a measure derived from that study, discusses the rationale for its component items, examines its psychometric properties, and demonstrates the relationship of this measure to the enrollment decision. Data from national samples of cancer survivors and the general public demonstrate the measure's validity and reliability. Results are discussed as they relate to patient understanding of clinical trials, informed decision making, and health communication processes. | Male | Decision Making | Humans | Patient Participation | OK |
|  |  |  |  |  |  | Journal of Pediatric Psychology | 2012-04-00 Apr 2012 | OBJECTIVES: To develop a measure of decision-making involvement in children and adolescents with cystic fibrosis, diabetes, and asthma. METHODS: Parent-child dyads completed the Decision-Making Involvement Scale (DMIS) and measures of locus of control and family communication. DMIS items were subjected to exploratory and confirmatory factor analysis (CFA). Temporal stability and construct validity were assessed. RESULTS: The parent form was reduced to 20 items representing five factors. CFA showed that the five factors were an acceptable fit to the parent- and child-report data. Internal consistency values ranged from 0.71 to 0.91. Temporal stability was supported by moderate-substantial intraclass correlation coefficients. DMIS subscales were associated with child age, child locus of control, and family communication. CONCLUSIONS: The DMIS can be used to inform our understanding of the transition to greater independence for illness management. Additional research is needed to examine outcomes of decision-making involvement, including treatment adherence and responsibility. | Male | Decision Making | Humans | Patient Participation | OK |
| Rudolf B. | Kool | Therese A. | Wiegers |  |  | BMC health services research | 2010-00-00 2010 | BACKGROUND: This study aims to describe the development, testing and optimization of a new standard instrument, the Consumer Quality Index (CQ-index) Long-term Care, for measuring client experiences with long-term care in the Netherlands. METHODS: Three versions of the CQ-index questionnaires and protocols for study sampling and data collection were developed, designed for interviews with residents of nursing or residential care homes and postal surveys among representatives of psychogeriatric residents and homecare clients. From July to November 2006 a pilot study was conducted among 2,697 clients of 68 nursing or residential care homes, 2,164 representatives of clients in 57 psychogeriatric care institutions, and 1,462 clients of 19 homecare organizations. We performed psychometric analyses and descriptive analyses, and evaluated the pilot study. RESULTS: The pilot study showed the feasibility and usability of the instruments, supported the multidimensionality of the questionnaires and showed first findings on client experiences and possibilities for quality improvement. Nine scales applied to all care settings: shared decision making, attitude and courtesy, information, body care, competence and safety of care, activities, autonomy, mental well-being, and availability of personnel. The pilot resulted in three optimized questionnaires and recommendations for nationwide implementation. CONCLUSIONS: The CQ-index Long-term Care provides a good basis to investigate the quality of nursing homes, residential care homes and homecare from the clients' perspective. This standardized instrument enables a nationwide comparison of the quality of long-term care for the purpose of transparency and quality assurance. | Humans | Patient Satisfaction | Questionnaires | Aged | OK |
| H. P. G. | Mulders | S. | Brouwer |  |  | Journal of Occupational Rehabilitation | 2010-12-00 Dec 2010 | BACKGROUND: The Perceived Functioning & Health (PFH) questionnaire was developed to collect, in a standardized manner, which work activities are limited due to health conditions according to the perception of the client. In this study the questionnaire's reliability and validity are investigated. METHODS: The PFH questionnaire is comprised of 147 questions, distributed over 33 scales, pertaining to the client's psychosocial and physical work limitations. The PFH data of 800 respondents were analyzed: 254 healthy employees, 408 workers on sick leave and 138 recipients of a disability pension. Internal consistency (Cronbach's α) for the scales was established. The test-retest reliability was examined for the data of 52 recipients of a disability pension who filled out the PFH twice within an interval of 1 month. Validation was established by taking the nature of the limitations as a criterion: mental limitations, physical limitations or a mix of both. To this end, the respondents were divided into groups distinguished on the basis of self-classification, as well as classification on the basis of disease codes given by insurance and occupational health physicians: a "healthy" group, subjects with only physical ("physical" group) or mental limitations ("mental" group) or mixed limitations ("mixed" group). The scale scores of these groups were compared and tested using analyses-of-variance and discriminant analyses. RESULTS: The scales were found to have sufficient to good internal consistency (mean Cronbach's-α = 0.79) and test-retest reliability (mean correlation r = 0.76). Analyses-of-variance demonstrated significant differences between the scores of the mental, physical and healthy groups on most of the expected scales. These results were found both in groups defined by self-classification as well as in groups based on disease codes. Moreover, discriminant analyses revealed that the a priori classification of the respondents into three groups (mental, physical, healthy) for more than 75% of them corresponded with the classification on the basis of scale scores obtained from the questionnaire. Furthermore, limitations due to specific types of complaints (low back pain, fatigue, concentration problems) or diagnosed disorders (musculoskeletal disorders, reactive disorders, endogenous disorders) were clearly reflected in the scores of the related scales of the PFH. CONCLUSION: The psychometric properties of the PFH with respect to reliability and validity were satisfactory. The PFH would appear to be an appropriate instrument for systematically measuring functional limitations in subjects on sick leave and in those receiving disability pensions, and could be used as a starting point in a disability claim procedure. | Humans | Patient Participation | Questionnaires | Psychometrics | OK |
| Zara | Cooper | Darin J. | Correll | David L. | Hepner | Patient Education and Counseling | 2014-03-00 mars 2014 | Objective: Identifying patient factors correlated with specific needs in preoperative decision making is of clinical and ethical importance. We examined patterns and predictors of deficiencies in informed surgical consent and shared decision-making in preoperative patients. Methods: Validated measures were used to survey 1034 preoperative patients in the preoperative clinic after signed informed consent. Principal component analysis defined correlated groupings of factors. Multivariable analysis assessed patient factors associated with resultant groupings. Results: 13% of patients exhibited deficits in their informed consent process; 33% exhibited other types of deficits. Informed consent deficits included not knowing the procedure being performed or risks and benefits. Other deficits included not having addressed patient values, preferences and goals. Non-English language and lower educational level were factors correlated with higher risk for deficits. Conclusion: Deficits exist in over a third of patients undergoing preoperative decision-making. Sociodemographic factors such as language and educational level identified particularly vulnerable groups at risk for having an incomplete, and possibly ineffective, decision-making process. Practice implications: Interventions to identify vulnerable groups and address patient centered surgical decision making in the pre-operative setting are needed. Focused interventions to address the needs of at-risk patients have potential to improve the surgical decision-making process and reduce disparities. (PsycINFO Database Record (c) 2014 APA, all rights reserved). (journal abstract) | Shared decision making | Decision Making | Therapeutic Processes | health disparities | Doublon |
| Zara | Cooper | Darin J. | Correll | David L. | Hepner | Patient Education and Counseling | 2014-03-00 Mar 2014 | OBJECTIVE: Identifying patient factors correlated with specific needs in preoperative decision making is of clinical and ethical importance. We examined patterns and predictors of deficiencies in informed surgical consent and shared decision-making in preoperative patients. METHODS: Validated measures were used to survey 1034 preoperative patients in the preoperative clinic after signed informed consent. Principal component analysis defined correlated groupings of factors. Multivariable analysis assessed patient factors associated with resultant groupings. RESULTS: 13% of patients exhibited deficits in their informed consent process; 33% exhibited other types of deficits. Informed consent deficits included not knowing the procedure being performed or risks and benefits. Other deficits included not having addressed patient values, preferences and goals. Non-English language and lower educational level were factors correlated with higher risk for deficits. CONCLUSION: Deficits exist in over a third of patients undergoing preoperative decision-making. Sociodemographic factors such as language and educational level identified particularly vulnerable groups at risk for having an incomplete, and possibly ineffective, decision-making process. PRACTICE IMPLICATIONS: Interventions to identify vulnerable groups and address patient centered surgical decision making in the pre-operative setting are needed. Focused interventions to address the needs of at-risk patients have potential to improve the surgical decision-making process and reduce disparities. | | | | | OK |
| Donna L. | Berry |  |  |  |  | Journal of Psychosocial Oncology | 2011-10-00 octobre 2011 | The Control Preferences Scale is widely used in decision research to measure patient preferences for participation in treatment decision making with health care providers. Following anecdotal reports of confusion with the scale the authors conducted an exploratory interview study to examine perceptions of the meaning and applicability of the Control Preferences Scale for men with localized prostate cancer seeking treatment in a multidisciplinary urology clinic. The preliminary data suggest potential validity challenges when the Control Preferences Scale is used in a multidisciplinary prostate cancer care setting, including the clinical context of localized prostate cancer and the meaning of shared decision making. (PsycINFO Database Record (c) 2012 APA, all rights reserved). (journal abstract) | Decision Making | Treatment | Preferences | treatment decision making | Doublon |
| Donna L. | Berry |  |  |  |  | Journal of Psychosocial Oncology | 2011-00-00 2011 | The Control Preferences Scale is widely used in decision research to measure patient preferences for participation in treatment decision making with health care providers. Following anecdotal reports of confusion with the scale the authors conducted an exploratory interview study to examine perceptions of the meaning and applicability of the Control Preferences Scale for men with localized prostate cancer seeking treatment in a multidisciplinary urology clinic. The preliminary data suggest potential validity challenges when the Control Preferences Scale is used in a multidisciplinary prostate cancer care setting, including the clinical context of localized prostate cancer and the meaning of shared decision making. | Male | Decision Making | Humans | Physician-Patient Relations | OK |
| Clarence H. | Braddock | Elisabeth J. S. | Kunkel | James R. | Cocroft | Medical Decision Making: An International Journal of the Society for Medical Decision Making | 2012-04-00 2012 Mar-Apr | PURPOSE: To measure the extent of informed decision making (IDM) about prostate cancer screening in physician-patient encounters, describe the coding process, and assess the reliability of the IDM measure. METHODS: Audiorecoded encounters of 146 older adult men and their primary care physicians were obtained in a randomized controlled trial of mediated decision support related to prostate cancer screening. Each encounter was dual coded for the presence or absence of 9 elements that reflect several important dimensions of IDM, such as information sharing, patient empowerment, and engaging patients in preference clarification. An IDM-9 score (range = 0-9) was determined for each encounter by summing the number of elements that were coded as present. Estimates of coding reliability and internal consistency were calculated. RESULTS: Male patients tended to be white (59%), married (70%), and between the ages of 50 and 59 (70%). Physicians tended to be white (90%), male (74%), and have more than 10 years of practice experience (74%). IDM-9 scores ranged from 0 to 7.5 (mean [SD], 2.7 [2.1]). Reliability (0.90) and internal consistency (0.81) of the IDM-9 were both high. The IDM dimension observed most frequently was information sharing (74%), whereas the dimension least frequently observed was engagement in preference clarification (3.4%). CONCLUSIONS: In physician-patient encounters, the level of IDM concerning prostate cancer screening was low. The use of a dual-coding approach with audiorecorded encounters produced a measure of IDM that was reliable and internally consistent. | Male | Decision Making | Humans | Patient Participation | OK |
| Paula | Nelson-Marten | Jacqueline | Jones | Jean S. | Kutner | The American Journal of Hospice & Palliative Care | 2014-11-00 Nov 2014 | BACKGROUND: Understanding why some patients with terminal illness are reluctant to take sufficient medication to control pain is critical to effective pain management. OBJECTIVE: As a first step toward exploring the pain medication-taking behavior of palliative care patients, this pilot study tested a survey regarding pain medication adherence, medication beliefs, and quality of life (QoL). DESIGN: Convenience sample; survey. SETTING/SUBJECTS: Six patients receiving inpatient Palliative Care consultations at an academic medical center answered questions about their outpatient pain medication-taking behavior. MEASUREMENTS: Medication Adherence Report Scale (MARS), Beliefs about Medications Questionnaire (BMQ), Brief Pain Inventory (BPI), closed-response items from a pain medication adherence study in terminally ill patients, the McGill Quality of Life Questionnaire (McGill), and demographic items. RESULTS: The battery of questionnaires took approximately 53 minutes; five of six participants were able to complete all items. Respondents reported moderate to severe pain (mean 4.3/10 for pain on average; 7/10 for worst pain in past 24 hours), and excellent medication adherence. When asked how much relief was provided by pain therapies, respondents reported a mean 73% (range 50-100%) relief. They expressed little concern about addiction, but more concern about medication-induced nausea and constipation. Overall QoL was good (mean 6.8/10, range 5-10, higher score better), with notably high scores in existential and support domains. CONCLUSIONS: Inpatients receiving palliative care consultation were able to complete interviewer-administered questionnaires regarding their pain perceptions, medications, and QoL. Further studies using these instruments are feasible and could inform shared decision making about pain management. | | | | | Doublon |
| Paula | Nelson-Marten | Jacqueline | Jones | Jean S. | Kutner | American Journal of Hospice & Palliative Medicine | 2014-11-00 novembre 2014 | Background: Understanding why some patients with terminal illness are reluctant to take sufficient medication to control pain is critical to effective pain management. Objective: As a first step toward exploring the pain medication-taking behavior of palliative care patients, this pilot study tested a survey regarding pain medication adherence, medication beliefs, and quality of life (QoL). Design: Convenience sample; survey. Setting/Subjects: Six patients receiving inpatient Palliative Care consultations at an academic medical center answered questions about their outpatient pain medication-taking behavior. Measurements: Medication Adherence Report Scale (MARS), Beliefs about Medications Questionnaire (BMQ), Brief Pain Inventory (BPI), closed-response items from a pain medication adherence study in terminally ill patients, the McGill Quality of Life Questionnaire (McGill), and demographic items. Results: The battery of questionnaires took approximately 53 minutes; five of six participants were able to complete all items. Respondents reported moderate to severe pain (mean 4.3/10 for pain on average; 7/10 for worst pain in past 24 hours), and excellent medication adherence. When asked how much relief was provided by pain therapies, respondents reported a mean 73% (range 50-100%) relief. They expressed little concern about addiction, but more concern about medication-induced nausea and constipation. Overall QoL was good (mean 6.8/10, range 5-10, higher score better), with notably high scores in existential and support domains. Conclusions: Inpatients receiving palliative care consultation were able to complete interviewer-administered questionnaires regarding their pain perceptions, medications, and QoL. Further studies using these instruments are feasible and could inform shared decision making about pain management. (PsycINFO Database Record (c) 2014 APA, all rights reserved). (journal abstract) | medication adherence | Drug Therapy | Treatment Compliance | pain perceptions | Doublon |
| Paula | Nelson-Marten | Jacqueline | Jones | Jean S. | Kutner | The American Journal of Hospice & Palliative Care | 2014-11-00 Nov 2014 | BACKGROUND: Understanding why some patients with terminal illness are reluctant to take sufficient medication to control pain is critical to effective pain management. OBJECTIVE: As a first step toward exploring the pain medication-taking behavior of palliative care patients, this pilot study tested a survey regarding pain medication adherence, medication beliefs, and quality of life (QoL). DESIGN: Convenience sample; survey. SETTING/SUBJECTS: Six patients receiving inpatient Palliative Care consultations at an academic medical center answered questions about their outpatient pain medication-taking behavior. MEASUREMENTS: Medication Adherence Report Scale (MARS), Beliefs about Medications Questionnaire (BMQ), Brief Pain Inventory (BPI), closed-response items from a pain medication adherence study in terminally ill patients, the McGill Quality of Life Questionnaire (McGill), and demographic items. RESULTS: The battery of questionnaires took approximately 53 minutes; five of six participants were able to complete all items. Respondents reported moderate to severe pain (mean 4.3/10 for pain on average; 7/10 for worst pain in past 24 hours), and excellent medication adherence. When asked how much relief was provided by pain therapies, respondents reported a mean 73% (range 50-100%) relief. They expressed little concern about addiction, but more concern about medication-induced nausea and constipation. Overall QoL was good (mean 6.8/10, range 5-10, higher score better), with notably high scores in existential and support domains. CONCLUSIONS: Inpatients receiving palliative care consultation were able to complete interviewer-administered questionnaires regarding their pain perceptions, medications, and QoL. Further studies using these instruments are feasible and could inform shared decision making about pain management. | | | | | OK |
| Susan | Magasi | Joy | Hammel | John D. | Corrigan | Archives of Physical Medicine and Rehabilitation | 2011-04-00 Apr 2011 | OBJECTIVE: To reflect the perspectives of rehabilitation stakeholders in a measure of participation enfranchisement that can be used by people with and without disabilities. DESIGN: Survey. SETTING: Community settings. PARTICIPANTS: We pilot-tested a draft instrument with 326 adults who had sustained stroke, spinal cord injury, traumatic brain injury, or other disabling condition, as well as a general population sample. We administered a revised version of the instrument to a statewide sample drawn from the 2006 Colorado Behavioral Risk Factors Surveillance System that included persons with (N=461) and without (N=451) self-identified activity limitations. INTERVENTIONS: None. MAIN OUTCOME MEASURE: Participation enfranchisement. RESULTS: We used multidimensional scaling, exploratory factor analysis (EFA) and confirmatory factor analysis (CFA), followed by rating scale analysis to evaluate the psychometric properties of the instrument. EFA identified 3 participation enfranchisement factors that describe perceived choice and control, contributing to one's community, and feeling valued; the factors were supported marginally by CFA. Rating scale analysis revealed marginal person separation and no misfitting items. CONCLUSIONS: Participation enfranchisement constitutes a new, previously unmeasured aspect of participation-one that addresses subjective perceptions rather than objective performance-with items that are clearly distinct from more generalized satisfaction with participation. The 19 enfranchisement items describe aspects of participation that may prove useful in characterizing longer-term rehabilitation outcomes. | Male | Humans | Patient Participation | Questionnaires | OK |
|  |  |  |  |  |  | Disability and Rehabilitation | 2011-00-00 2011 | PURPOSE: Our aims were to: (1) describe participation in people with Parkinson disease (PD), (2) evaluate the relationship between quality of life and participation and (3) determine the mobility measures which are predictive of participation. METHODS: Participants with idiopathic PD (n = 62) were tested off medication for participation (Activity Card Sort), quality of life (PDQ-39), disease severity (MDS-UPDRS) and mobility (Berg Balance Scale, Five Time Sit to Stand (FTSTS), Six Minute Walk, forward walking velocity, dual-task walking velocity and Freezing of Gait Questionnaire (FOGQ)). Relationships of all variables to participation were examined using Pearson correlations. Subsequent regression analysis was employed to determine the mobility measures which best predicted the participation. RESULTS: Participants with PD retained, on average, 78.3% (SD = 15.6%) of total activities. Participation was negatively correlated with all PDQ-39 domains (r range -0.36 to -0.78, all p < 0.005) with the mobility domain having the strongest correlation. All mobility measures were significantly correlated with participation, with the final regression model including only FTSTS and FOGQ which combined explained 37% of the variance in participation. CONCLUSIONS: Participation is highly related to mobility-related QOL and may be most impacted by ability to stand up from a chair and freezing of gait in those with PD. | Male | Humans | Patient Participation | Questionnaires | OK |
| Robert | DeVellis | David | Cella |  | Patient-Reported Outcomes Measurement Information System Social Domain Working Group | Archives of Physical Medicine and Rehabilitation | 2010-09-00 Sep 2010 | OBJECTIVES: To describe the lessons learned in the initial development of Patient-Reported Outcomes Measurement Information System social function item banks. DESIGN: Development and testing of 2 item pools within a general population to create item banks that measure ability to participate and satisfaction with participation in social activities. SETTING: Administration via the Internet. PARTICIPANTS: General population members (N=956) of a national polling organization registry participated; data for 768 and 778 participants were used in the analysis. INTERVENTIONS: Not applicable. MAIN OUTCOME MEASURES: Measures of ability to participate and satisfaction with participation in social activities. RESULTS: Fifty-six items measuring the ability to participate were essentially unidimensional but did not fit an item response theory model. As a result, item banks were not developed for these items. Of the 56 items measuring satisfaction with participation, 14 items measuring social roles and 12 items measuring discretionary activities were unidimensional and met item response theory model assumptions. Two 7-item short forms were also developed. CONCLUSIONS: Four lessons, mostly concerning item content, were learned in the development of banks measuring social function. These lessons led to item revisions that are being tested in subsequent studies. | Male | Humans | Patient Participation | Patient Satisfaction | OK |
|  |  |  |  |  |  | Injury | 2011-03-00 Mar 2011 | When designing a study, it is important to select appropriate instruments to measure health outcomes. An investigator must have a comprehensive understanding of the disease and its effects on patient health to inform instrument selection. We provide a brief introduction of different ways that health is defined and the properties of a good measurement tool (reliability, validity, sensitivity to change). We describe patient-reported outcomes (PROs) and methods to improve their interpretability, and we include strategies to reduce bias in health measurement and considerations that may improve the feasibility of measuring outcomes in a research study. | Male | Humans | Patient Participation | Female | OK |
| Torvald | Øgaard | Kjell | Hausken |  |  | Journal of Clinical Nursing | 2014-02-00 Feb 2014 | AIMS AND OBJECTIVES: To develop, empirical test, and validate an instrument measuring patient participation in surgical treatment decision-making from healthcare professionals' perspective. BACKGROUND: Since the advent of New Public Management in many Western countries, patient participation in healthcare decision-making has been considered to be a best practice. A common notion is that well-educated and well-informed public want to choose their own treatments and providers and want to ask questions about the quality of their health services. DESIGN: Survey. METHODS: A self-report-measuring instrument was designed and administered to 620 healthcare professionals. Items were developed, validated and tested by 451 nurses and physicians working in six surgical wards in a University Hospital in Norway. RESULTS: A 16-item scale with the following four dimensions was developed: information dissemination, formulation of options, integration of information and control. Factor analysis procedures and reliability testing were performed. A one-way, between-groups analysis of variance was conducted to compare doctors' and nurses' opinions on four dimensions of patient participation in surgical treatment decision-making. CONCLUSIONS: This article shows that patient participation in surgical treatment decision-making can be measured by a 16-item scale and four distinct dimensions. The analysis demonstrated a reasonable level of construct validity and reliability. Nurses and physicians have a positive attitude towards patient participation overall, but the two groups differ in the extent to which they accept the idea of patient participation in treatment decision-making. RELEVANCE TO CLINICAL PRACTICE: The instrument can be a tool for managers and healthcare professionals in the implementation of patient participation in clinical practice. Data from the instrument can be useful to identify health services being provided and what areas that could strengthen patient participation. | | | | | OK |
| Torvald | Øgaard | Kjell | Hausken |  |  | Journal of Clinical Nursing | 2014-02-00 février 2014 | Aims and objectives: To develop, empirical test, and validate an instrument measuring patient participation in surgical treatment decision‐making from healthcare professionals' perspective. Background: Since the advent of New Public Management in many Western countries, patient participation in healthcare decision‐making has been considered to be a best practice. A common notion is that well‐educated and well‐informed public want to choose their own treatments and providers and want to ask questions about the quality of their health services. Design: Survey. Methods: A self‐report‐measuring instrument was designed and administered to 620 healthcare professionals. Items were developed, validated and tested by 451 nurses and physicians working in six surgical wards in a University Hospital in Norway. Results: A 16‐item scale with the following four dimensions was developed: information dissemination, formulation of options, integration of information and control. Factor analysis procedures and reliability testing were performed. A one‐way, between‐groups analysis of variance was conducted to compare doctors' and nurses' opinions on four dimensions of patient participation in surgical treatment decision‐making. Conclusions: This article shows that patient participation in surgical treatment decision‐making can be measured by a 16‐item scale and four distinct dimensions. The analysis demonstrated a reasonable level of construct validity and reliability. Nurses and physicians have a positive attitude towards patient participation overall, but the two groups differ in the extent to which they accept the idea of patient participation in treatment decision‐making. Relevance to clinical practice: The instrument can be a tool for managers and healthcare professionals in the implementation of patient participation in clinical practice. Data from the instrument can be useful to identify health services being provided and what areas that could strengthen patient participation. (PsycINFO Database Record (c) 2014 APA, all rights reserved). (journal abstract) | Decision Making | Client Participation | Health Personnel Attitudes | surgical treatment | Doublon |
| Rosalina | Butao | Victoria | McCue | Fatima | Garcia | Journal of Research in Nursing | 2014-02-00 février 2014 | Shared governance is considered an innovative management process model that includes shared decision-making between all members of the healthcare workforce and focuses on nurses’ control over their practice and accountability of care. One specific tool that measures shared governance perceptions of nurses is the RN-focused Index of Professional Governance (IPNG). The objective of this study was to assess reliability and add to the validity of the IPNG by further establishing the construct validity and correlating the scale scores to measures of satisfaction obtained from the National Database of Nursing Quality Indicators (or NDNQI) survey. There were 76 respondents representing six units in one hospital. We reported high reliability for each one of the six subscale scores as well as for the total score (Cronbach alphas of 0.94 and higher). Construct validity was supported by the invariance of the scores across age groups along with schooling and experience levels. Concurrent validity was supported by a correlation of the IPNG score with job enjoyment (r = 0.437, p = 0.002) and the desire to recommend the hospital as a place of employment (r = 0.442, p = 0.001). The IPNG should also be tested in other healthcare professionals as its scoring ability can compare management, units and departments. (PsycINFO Database Record (c) 2014 APA, all rights reserved). (journal abstract) | Psychometrics | test validity | test reliability | clinical practice | OK |
| M. | Carey |  |  |  |  | European Journal of Cancer Care | 2013-05-00 May 2013 | In this review, we explore professionally-driven and consumer-driven paradigms in measuring psychosocial outcomes for cancer care. Early measures of psychosocial well-being focussed on clinically-derived concepts of dysfunction. Recent literature reflects a paradigm shift toward a consumer-driven approach to the conceptualisation and measurement of psychosocial well-being. The key distinction between the two approaches rests on whether the professional or consumer retains judgement authority and raises the question of whether it is necessary to include both perspectives in research and practice. Research is proposed to clarify our interpretation of these approaches with a view to devising novel interventions to benefit patient well-being. | Humans | Patient Participation | Neoplasms | Survivors | OK |
|  |  |  |  |  |  | Circulation. Cardiovascular Quality and Outcomes | 2014-07-00 Jul 2014 | |  |  |  |  | OK |
|  |  |  |  |  |  | Scandinavian Journal of Caring Sciences | 2010-12-00 Dec 2010 | Measuring patients' experiences has been a major task for health care organisations during the past decade. The discharge process is identified as a vulnerable component of health care in need of assessment, especially when it concerns elderly persons. There are no published reviews or systematic assessment of the existing instruments developed to capture patients' perspective on the discharge process. This study gives a review of existing survey instruments designed to assess patients' perspectives on the discharge process. We used systematic searches for potentially relevant instruments in MEDLINE, PubMed, CINAHL, and the Cochrane Database of Systematic Reviews for English language studies published between 1998 and 2009 was considered to evaluate the patients' perspective on the discharge process. Ten studies were included and assessed according to the established criteria, and the studies presented a total of 47 items related to participation. The review identified only one instrument designed specifically to capture participation in the discharge process. The main focus is on the information flow from the professional to the patient and never vice versa. Few of the instruments studied/analysed to what degree the patients were invited to share their knowledge, and none of the instruments inquired whether, in the patients' experiences, their perspective was taken into account. The major finding of the review is that none of the existing instruments capture the full range of participation, nor do they cover those areas of the discharge process identified by elderly patients themselves as the most essential. | Humans | Patient Participation | Aged | Patient Discharge | OK |
| Lucie | Nadeau | Suzanne | Deshaies | Toby | Measham | International Journal of Integrated Care | 2012-03-00 2012 Jan-Mar | OBJECTIVE: This pilot study examines the potential utility of the Perception of Interprofessional Collaboration Model and the shared decision-making scales in evaluating the quality of partnership in child mental health collaborative care. METHODS: Ninety-six primary care professionals working with children and youth responded to an internet survey which included the Perception of Interprofessional Collaboration Model scale (PINCOM-Q) and an adapted version of a shared decision-making scale (Échelle de confort décisionnel, partenaire-ECD-P). The perceptions of child mental health professionals were compared with those of other professionals working with children. RESULTS: The PINCOM-Q and the ECD-P scales had an excellent internal consistency and they were moderately correlated. Child mental health professionals' Individual Interprofessional Collaboration scores from the PINCOM-Q individual aspects subscale were better than that of other child professionals. CONCLUSION: These scales may be interesting instruments to measure the quality of partnership in child mental health collaborative care settings. Research needs to replicate these findings and to determine whether the quality of collaboration is a predictor of mental health outcome. | | | | | OK |
| Yuchiao | Chang | Sarah | Hewitt | Argyrios | Ziogas | Health Expectations: An International Journal of Public Participation in Health Care & Health Policy | 2014-05-12 mai 12, 2014 | Abstract Background A high‐quality decision for breast cancer surgery requires that patients are well informed, meaningfully involved in decision making, and receive treatments that match their goals. There is little in the existing literature that examines a comprehensive measure of decision quality for Latina breast cancer patients. Objective To examine the quality of surgical decisions among Latina breast cancer survivors and explore factors associated with decision quality and decision regret. Design Cross‐sectional mailed survey. Main outcome measures English and certified Spanish translations of Breast Cancer Surgery Decision Quality Instrument (BCS‐DQI), Short Acculturation Scale for Hispanics (SASH) and decision regret. Participants and setting Ninety‐seven breast cancer survivors of Hispanic or Spanish descent identified through the cancer registry from Orange or San Diego Counties in California. Results The 97 respondents were on average 55.7 years old, 39.1% had high school diploma or more education, and 62.9% had low acculturation (SASH scores &lt; 2.99). The average knowledge score was 48.2%, the average decision process score was 67.5%, and many (77.3%) received treatments that matched their goals. In multivariable models, there were no significant associations with education, age, acculturation and any aspect of decision quality or decision regret in this sample. Respondents who had higher decision process scores, indicating more involvement in decision making, had significantly lower decision regret. Conclusions The BCS‐DQI may require some adaptation for Latina populations to improve acceptability. The different aspects of decision quality, including knowledge, decision process and concordance, did not vary by level of acculturation. (PsycINFO Database Record (c) 2014 APA, all rights reserved). (journal abstract) | Shared decision making | No terms assigned | decision quality | | Doublon |
| Yuchiao | Chang | Sarah | Hewitt | Argyrios | Ziogas | Health Expectations: An International Journal of Public Participation in Health Care and Health Policy | 2014-05-12 May 12, 2014 | BACKGROUND: A high-quality decision for breast cancer surgery requires that patients are well informed, meaningfully involved in decision making, and receive treatments that match their goals. There is little in the existing literature that examines a comprehensive measure of decision quality for Latina breast cancer patients. OBJECTIVE: To examine the quality of surgical decisions among Latina breast cancer survivors and explore factors associated with decision quality and decision regret. DESIGN: Cross-sectional mailed survey. MAIN OUTCOME MEASURES: English and certified Spanish translations of Breast Cancer Surgery Decision Quality Instrument (BCS-DQI), Short Acculturation Scale for Hispanics (SASH) and decision regret. PARTICIPANTS AND SETTING: Ninety-seven breast cancer survivors of Hispanic or Spanish descent identified through the cancer registry from Orange or San Diego Counties in California. RESULTS: The 97 respondents were on average 55.7 years old, 39.1% had high school diploma or more education, and 62.9% had low acculturation (SASH scores < 2.99). The average knowledge score was 48.2%, the average decision process score was 67.5%, and many (77.3%) received treatments that matched their goals. In multivariable models, there were no significant associations with education, age, acculturation and any aspect of decision quality or decision regret in this sample. Respondents who had higher decision process scores, indicating more involvement in decision making, had significantly lower decision regret. CONCLUSIONS: The BCS-DQI may require some adaptation for Latina populations to improve acceptability. The different aspects of decision quality, including knowledge, decision process and concordance, did not vary by level of acculturation. | | | | | OK |
| Angela L. | Rollins | John H. | McGrew | Lia J. | Hicks | Journal of the American Psychiatric Nurses Association | 2013-06-00 2013 May-Jun | BACKGROUND: Approaches to measuring recovery orientation are needed, particularly for programs that may struggle with implementing recovery-oriented treatment. OBJECTIVE: A mixed-methods comparative study was conducted to explore effective approaches to measuring recovery orientation of assertive community treatment (ACT) teams. DESIGN: Two ACT teams exhibiting high and low recovery orientation were compared using surveys, treatment plan ratings, diaries of treatment visits, and team leader-reported treatment control mechanisms. RESULTS: The recovery-oriented team differed on one survey measure (higher expectations for consumer recovery), treatment planning (greater consumer involvement and goal-directed content), and use of control mechanisms (less use of representative payee, agency-held lease, daily medication delivery, and family involvement). Staff and consumer diaries showed the most consistent differences (e.g., conveying hope and choice) and were the least susceptible to observer bias but had the lowest response rates. CONCLUSIONS: Several practices differentiate recovery orientation on ACT teams, and a mixed-methods assessment approach is feasible. | Humans | Patient Participation | United States | Cooperative Behavior | OK |
| Ellen F. | Binder | Brian | Carpenter | Kenneth E. | Freedland | American Journal of Physical Medicine & Rehabilitation / Association of Academic Physiatrists | 2012-08-00 Aug 2012 | Attaining and demonstrating treatment fidelity is critical in the development and testing of evidence-based interventions. Treatment fidelity refers to the extent to which an intervention was implemented in clinical testing as it was conceptualized and is clearly differentiable from control or standard-of-care interventions. In clinical research, treatment fidelity is typically attained through intensive training and supervision techniques and demonstrated by measuring therapist adherence and competence to the protocol using external raters. However, in occupational and physical therapy outcomes research, treatment fidelity methods have not been used, which, in our view, is a serious gap that impedes novel treatment development and testing in these rehabilitation fields. In this article, we describe the development of methods to train and supervise therapists to attain adequate treatment fidelity in a treatment development project involving a novel occupational and physical therapy-based intervention. We also present a data-driven model for demonstrating therapist adherence and competence in the new treatment and its differentiation from standard of care. In doing so, we provide an approach that rehabilitation researchers can use to address treatment fidelity in occupational and physical therapy-based interventions. We recommend that all treatment researchers in rehabilitation disciplines use these or similar methods as a vital step in the development and testing of evidence-based rehabilitation interventions. | Humans | Patient Participation | Middle Aged | Adult | OK |
| Joachim | Cohen | Kenneth | Chambaere | Luc | Deliens | Archives of Pediatrics & Adolescent Medicine | 2010-06-00 Jun 2010 | OBJECTIVES: To estimate the prevalence of end-of-life decisions and to describe their characteristics and the preceding decision-making process in minors in Belgium. DESIGN: Population-based postmortem anonymous physician survey. SETTING: Flanders, Belgium. PARTICIPANTS: All physicians signing the death certificates of all patients (N = 250) aged 1 to 17 years who died between June 2007 and November 2008 in Flanders, Belgium. OUTCOME MEASURES: Prevalence and characteristics of end-of-life decisions and the preceding decision-making process. RESULTS: For 165 of the 250 deaths, a physician questionnaire was returned (70.5%). In 36.4%, death was preceded by an end-of-life decision. Drugs were administered to alleviate pain and symptoms with a possible life-shortening effect in 18.2% of all deaths, nontreatment decisions were made in 10.3%, and lethal drugs without the patient's explicit request were used in 7.9%. No cases of euthanasia, ie, the use of drugs with the explicit intention to hasten death at the patient's explicit request, were reported. Poor clinical prospects (84.6%) and low quality of life expectations (61.5%) were important reasons for the physicians to engage in end-of-life decisions. Parents were involved in decision making in 85.2% of these decisions, patients in 15.4%. CONCLUSIONS: Medical end-of-life decisions are frequent in minors in Flanders, Belgium. Whereas parents were involved in most end-of-life decisions, the patients themselves were involved much less frequently, even when the ending of their lives was intended. At the time of decision making, patients were often comatose or the physicians deemed them incompetent or too young to be involved. | Male | Decision Making | Humans | Patient Participation | OK |
| Megan A. | Moreno | Roger L. | Brown | Ted D. | Sigrest | Academic Medicine: Journal of the Association of American Medical Colleges | 2012-06-00 Jun 2012 | PURPOSE: Factors that support self-efficacy in family-centered care (FCC) must be understood in order to foster FCC in trainees. Using social cognitive theory, the authors examined (1) how three supportive experiences (observing role models, practicing for mastery, and receiving feedback) influence self-efficacy with FCC during rounds and (2) whether the influence of these supportive experiences was mediated by self-efficacy with three key FCC tasks (relationship building, information exchange, and decision making). METHOD: Researchers surveyed third-year students during pediatric clerkship rotations during the 2008-2011 academic years. Surveys assessed supportive experiences and students' self-efficacy both with FCC during rounds and with key FCC tasks. Researchers constructed measurement models via exploratory and confirmatory factor analyses. Composite indicator structural equation models evaluated whether supportive experiences influenced self-efficacy with FCC during rounds and whether self-efficacy with key FCC tasks mediated any such influences. RESULTS: Of 184 eligible students, 172 (93%) completed preclerkship surveys. Observing role models and practicing for mastery supported self-efficacy with FCC during rounds (each P < .01), whereas receiving feedback did not. Self-efficacy with two specific FCC tasks-relationship building and decision making (each P < .05)-mediated the effects of these two supportive experiences on self-efficacy with FCC during rounds. CONCLUSIONS: Both observing role models and practicing for mastery foster students' self-efficacy with FCC during rounds, operating through self-efficacy with key FCC tasks. Results suggest the importance both of helping students gain self-efficacy in key FCC tasks before rounds and of helping educators implement supportive experiences during rounds. | Decision Making | Humans | Patient Participation | Physician-Patient Relations | OK |
| Justin | Teng |  |  |  |  | Emergency medicine Australasia: EMA | 2011-06-00 Jun 2011 | OBJECTIVES: To determine the proportion of patients in a teaching hospital ED who are available to medical students; identify barriers to student access to patients; and determine whether patients are more likely to be accessible if the term student doctor is used rather than medical student. METHODS: Repeated cross-sectional study of the ED of a tertiary teaching hospital. Interviews were attempted with all patients in the ED during six 4 h periods. Outcome measures included: number of patients present and accessible to students; present but inaccessible, absent or unfit to be seen for clinical reasons; number of patients consenting to history, physical examination and certain procedures; and difference in patient consent between the terms 'medical student' and 'student doctor'. RESULTS: Overall, 180 of 450 (40.0%) patients completed the interview, 72 (16.0%) were able to be observed only, and 198 (44.0%) were not suitable for interview or observation. The common reasons for patient unsuitability were: physically not available (60%), being assessed by a health professional or undergoing a procedure (13.0%) altered mental status (7.4%), unstable or terminally ill (5.2%); refusal to participate in the study (4.8%), or dangerous or under arrest (4.1%). No significant differences were found in patient willingness to undergo clinical skills from 'student doctors' compared with 'medical students'. CONCLUSION: A minimum 40% of patients in a tertiary ED are accessible for student learning, with high proportions of patients accepting of students practising supervised history-taking, physical examination, and most less-invasive procedural skills. | Male | Humans | Patient Participation | Physician-Patient Relations | OK |
|  |  |  |  |  |  | The Clinical Teacher | 2011-06-00 Jun 2011 | BACKGROUND: Obtaining consent before engaging patients in teaching activities is essential. The presence of medical students as observers in the operating theatre is sometimes overlooked as a form of teaching activity, in which patients could have become unwitting or unwilling participants. PURPOSE: To investigate patients' attitudes towards student observers in theatre. METHOD: A cross-sectional, voluntary, guided questionnaire survey on 225 general surgical patients at a teaching hospital. RESULTS: Over two-thirds of patients would accept student observers, and regard a prior consent process as essential. Gender, level of education, subjective state of health and the perceived risk of surgery were not found to influence their decisions. Younger patients were more likely to refuse observers. Close to 10 percent of patients who were happy to participate in bedside teaching would not accept theatre observers, whereas 25 percent who were not happy to participate in bedside teaching would accept theatre observers. CONCLUSION: An explicit consent process is essential for theatre-based teaching, even when students are simply acting as observers. Patients who are willing to participate in ward-based teaching should not be presumed to accept theatre observers. | Male | Humans | Patient Participation | Patient Satisfaction | OK |
| Dorte Ejg | Jarbøl | Jørgen | Nexøe | Dorte | Gyrd-Hansen | BMC medical informatics and decision making | 2012-00-00 2012 | BACKGROUND: Shared decision-making and patients' choice of interventions are areas of increasing importance, not least seen in the light of the fact that chronic conditions are increasing, interventions considered important for public health, and still non-acceptance of especially risk-reducing treatments of cardiovascular diseases (CVD) is prevalent. A better understanding of patients' medication-taking behavior is needed and may be reached by studying the reasons why people accept or decline medication recommendations. The aim of this paper was to identify factors that may influence people's decisions and reasoning for accepting or declining a cardiovascular preventive medication offer. METHODS: From a random sample of 4,000 people aged 40-59 years in a Danish population, 1,169 participants were asked to imagine being at increased risk of cardiovascular disease and being offered a preventive medication. After receiving 'complete' information about effectiveness of the medication they were asked whether they would accept medication. Finally, they were asked about reasons for the decision. RESULTS: A total of 725 (67%) of 1,082 participants accepted the medication offer. Even quite large effects of medication (up to 8 percentage points absolute risk reduction) had a smaller impact on acceptance to medication than personal experience with cardiovascular disease. Furthermore, increasing age of the participant and living with a partner were significantly associated with acceptance. Some 45% of the respondents accepting justified their choice as being for health reasons, and they were more likely to be women, live alone, have higher income and higher education levels. Among those who did not accept the medication offer, 56% indicated that they would rather prefer to change lifestyle. CONCLUSIONS: Medication effectiveness seems to have a moderate influence on people's decisions to accept preventive medication, while factors such as personal experience with cardiovascular disease may have an equally strong or stronger influence, indicating that practitioners could do well to carefully identify the reasons for their patients' treatment decisions. | Male | Decision Making | Humans | Questionnaires | OK |
| Maureen | Wilson-Genderson | Juerg | Bernhard | Karin | Ribi | Journal of Clinical Oncology: Official Journal of the American Society of Clinical Oncology | 2012-03-10 Mar 10, 2012 | PURPOSE: To investigate how involvement preferences of patients with breast cancer change during the treatment decision-making process and determine the impact of meeting patients' expectations on decision-making outcomes. PATIENTS AND METHODS: Participants were 683 patients with breast cancer from 62 oncologists in five different countries recruited to an International Breast Cancer Study Group (IBCSG 33-03) project. Questionnaires elicited patients' pre- and postconsultation preferences for involvement in treatment decision making and whether these were met or not. Decision-related outcomes were assessed postconsultation. RESULTS: Before the consultation, most patients preferred shared or patient-directed treatment decision making. After the consultation, 43% of patients' preferences changed, and most shifted toward patient-directed decisions. The actual postconsultation decision was more likely to be made according to postconsultation rather than preconsultation preferences. Compared with patients who were less involved than they had hoped to be, patients who were as involved as they had hoped to be or were even more involved in decision making had significantly better decision-related outcomes. This was true regardless of whether preference change occurred. CONCLUSION: Many patients with early-stage breast cancer have treatment options and approach treatment decisions with a desire for decisional control, which may increase after their consultation. Patients' ultimate involvement preferences were more likely to be consistent with the way the decision was actually made, suggesting that patients need to feel concordance between their preference and the actual decision. Patients who directed decisions, even if more than they hoped for, fared better on all decision-related outcomes. These results emphasize the need for oncologists to endorse and facilitate patient participation in treatment decision making. | Male | Decision Making | Humans | Patient Participation | OK |
|  |  |  |  |  |  | Australian Family Physician | 2011-05-00 May 2011 | BACKGROUND: The presentation of a woman in midlife can be an opportunity for both the woman and her doctor to consider a wide range of issues that may be impacting on quality of life or that present a risk to her future health. OBJECTIVE: This article considers the assessment of a woman in the menopausal transition. DISCUSSION: The aim of assessment is to manage acute menopausal symptoms (eg. hot flushes); the complications of menopause (eg. osteoporosis); to avoid risk factors for complications (eg. fracture, thromboembolism); and to ensure a preventive healthcare plan is in place. | Humans | Patient Participation | Physician-Patient Relations | Female | OK |
| Lilisbeth | Perestelo-Perez | Jeanette | Perez-Ramos | Marien | Gonzalez-Lorenzo | Pharmacoepidemiology and Drug Safety | 2012-02-00 Feb 2012 | PURPOSE: To explore psychiatrists' attitudes toward concordance by validating the Leeds Attitude to Concordance Scale II (LATCon II) in a Spanish sample. METHODS: This was a cross-sectional survey. An opportunistic sample of 125 psychiatrist and 100 psychiatry registrars attending a national conference completed the LATCon II questionnaire and sociodemographic and professional data. The principal component analysis of the LATCon II items was performed. Associations with sociodemographic and mental health professional variables were calculated. RESULTS: Principal component analysis yielded three components labeled "communication/empathy," "shared control," and "eventual paternalistic style." Women obtained significantly lower scores than men on the second component. Mental health professional variables were not related to attitude to concordance. CONCLUSIONS: Psychiatrists show a favorable attitude to involve patients in a process of reciprocal communication, where patients' preferences, values, and expectations are considered, but they are more cautious in their attitude to sharing decisions with patients. There is scope for the different kinds of research in this area: studying sex-based differences in psychiatrists' attitudes to concordance and also exploring the gap in mental health care between patients' and professionals' views of shared decision making. Only in this way can the real partnership for shared decision making be fully understood. | Male | Decision Making | Humans | Patient Participation | OK |
|  |  |  |  |  |  | Archives of Internal Medicine | 2010-03-08 Mar 8, 2010 | | Decision Support Techniques | Humans | Patient Participation | Controlled Clinical Trials as Topic | OK |
| Evelien R. | Spelten | Janneke T. | Gitsels-van der Wal | Sandra | van Dulmen | Midwifery | 2014-06-00 Jun 2014 | OBJECTIVE: this study aims to provide insight into: (a) midwives' views on appropriate antenatal counselling for congenital anomaly tests, and (b) whether these views match clients' preferences regarding antenatal counselling. DESIGN: a comparative (midwives versus clients) questionnaire survey. Cognitive interviews (n=8) were used to validate the internal validity of the midwifery questionnaire results. PARTICIPANTS AND SETTING: 1416 Dutch midwives (response 62%) completed a questionnaire measuring their views on appropriate antenatal counselling for congenital anomaly tests. MEASUREMENTS: we used the 58-item midwives' version of the QUOTE (prenatal), an instrument to assess clients' counselling preferences. Descriptive statistics were used to explore midwives' views on appropriate counselling and how these relate to client preferences as measured previously with the clients' version of the QUOTE (prenatal). FINDINGS: almost all midwives consider the client-midwife relation (100%) and health education (95%) to be (very) important for appropriate antenatal counselling for congenital anomaly tests. Almost half of the midwives consider decision-making support (47%) to be (very) important. These findings are practically congruent with client preferences. Still, clinically relevant differences were found regarding 13 individual items, e.g. more clients than midwives value 'medical information about congenital anomalies' and 'getting advice whether to take prenatal tests or not'. KEY CONCLUSION: like clients, most midwives value a good client-midwife relation and health education as (very) important for antenatal counselling for congenital anomaly tests. Less than half of them value decision-making support. These findings are in contrast with the literature in which decision-making support is seen as the most important part of antenatal counselling for congenital anomaly tests. IMPLICATION FOR PRACTICE: preferably, antenatal counselling for congenital anomaly tests should be consistent with the three-function model of antenatal counselling i.e. maintaining a client-midwife relation, providing health education as well as decision-making support, and tailored to clients' individual preferences. As not all midwives subscribe to these functions, reflection on their views is important. Furthermore, midwives need to bridge their views on appropriate antenatal counselling and client preferences. To do so, midwives may benefit from the Shared Decision Making approach. | | | | | OK |
| Rhonda | Burke | Susan | Hales | Geraldine | Gillies | Journal of Cardiac Failure | 2013-09-00 Sep 2013 | OBJECTIVE: Cognitive impairments are prevalent in heart failure (HF) patients, worsening outcomes but often undetected.The aim of this study was to screen HF outpatients for mild cognitive impairment (MCI), determine the areas of cognition affected, patient awareness of cognitive change, and associated factors. METHOD AND RESULTS: HF patients (n = 128) newly registered for the Management of Cardiac Function program, free from neurocognitive disorder, and with sufficient visual acuity were assessed with the use of the Montreal Cognitive Assessment tool (MoCA). MCI was classified as MoCA score ≤22. The sample was elderly (mean, 80.65 years; SD, 11.52). Mean MoCA score was 24.58 (SD 3.45), 22% were classified as impaired, 45% had noticed a change in cognition, and 15% reported that they were affected in their daily lives. Patients noticing this impact had lower MoCA scores (22.74, SD 3.0) than those who did not (25.17, SD 2.96; P ≤ .02). Most impairments occurred for delayed recall, visuospatial/executive function, and abstraction. The odds of impairment increased by the presence of ischemic heart disease (odds ratio, 4.18; 95% confidence interval, 1.15-15.69). CONCLUSIONS: In HF outpatients without a dementia diagnosis, MCI is prevalent. Screening for MCI and incorporation of compensatory strategies are essential. | Male | Humans | Patient Participation | Female | Doublon |
| Rhonda | Burke | Susan | Hales | Geraldine | Gillies | Journal of Cardiac Failure | 2013-09-00 Sep 2013 | OBJECTIVE: Cognitive impairments are prevalent in heart failure (HF) patients, worsening outcomes but often undetected.The aim of this study was to screen HF outpatients for mild cognitive impairment (MCI), determine the areas of cognition affected, patient awareness of cognitive change, and associated factors. METHOD AND RESULTS: HF patients (n = 128) newly registered for the Management of Cardiac Function program, free from neurocognitive disorder, and with sufficient visual acuity were assessed with the use of the Montreal Cognitive Assessment tool (MoCA). MCI was classified as MoCA score ≤22. The sample was elderly (mean, 80.65 years; SD, 11.52). Mean MoCA score was 24.58 (SD 3.45), 22% were classified as impaired, 45% had noticed a change in cognition, and 15% reported that they were affected in their daily lives. Patients noticing this impact had lower MoCA scores (22.74, SD 3.0) than those who did not (25.17, SD 2.96; P ≤ .02). Most impairments occurred for delayed recall, visuospatial/executive function, and abstraction. The odds of impairment increased by the presence of ischemic heart disease (odds ratio, 4.18; 95% confidence interval, 1.15-15.69). CONCLUSIONS: In HF outpatients without a dementia diagnosis, MCI is prevalent. Screening for MCI and incorporation of compensatory strategies are essential. | Male | Humans | Patient Participation | Female | OK |
| Wen Hui | Tan | Jeffrey | Ketchersid | Ryan P. | Calfee | The Journal of Hand Surgery | 2013-04-00 Apr 2013 | PURPOSE: Patient-rated instruments are increasingly used to measure orthopedic outcomes. However, the clinical relevance of modest score changes on such instruments is often unclear. This study was designed to define the minimal clinically important differences (MCIDs) of the Disabilities of the Arm, Shoulder, and Hand (DASH), QuickDASH (subset of DASH), and Patient-Rated Wrist Evaluation (PRWE) questionnaires for atraumatic conditions of the hand, wrist, and forearm. METHODS: We prospectively analyzed 102 patients undergoing nonoperative treatment for isolated tendinitis, arthritis, or nerve compression syndromes from the forearm to the hand. By phone, patients completed the DASH, QuickDASH, and PRWE at enrollment and at 2 weeks (n = 78 used in the analysis) and 4 weeks (n = 24 used in the analysis) after initiating treatment. Patients reporting clinical improvement each contributed a single data point categorized as no change (n = 41), minimal improvement (n = 30), or marked improvement (n = 31) via a validated anchor-based approach. We calculated the MCID as the mean change score for each outcome measure in the minimal improvement group. RESULTS: The MCID (95% confidence interval) for the DASH was 10 (5-15). The MCID for the QuickDASH was 14 (9-20). The MCID was 14 (8-20) for the PRWE. The MCID values were significantly different from changes in these outcome measures at times of either no change or marked improvement. The MCID values positively correlated with baseline outcome measure scores to a greater degree than final outcome measure scores. CONCLUSIONS: Longitudinal changes on the DASH of 10 points, on the QuickDASH of 14 points, and on the PRWE of 14 points represent minimal clinically important changes. We recommend application of these MCID values for group-level analysis when conducting research and interpreting data examining groups of patients as opposed to assessing individual patients. These MCID values may provide a basis for sample size calculations for future investigation using these common patient-rated outcome measures. TYPE OF STUDY/LEVEL OF EVIDENCE: Diagnostic III. | Male | Humans | Patient Participation | Questionnaires | OK |
| Susanne | Iwarsson |  |  |  |  | Disability and Rehabilitation. Assistive Technology | 2010-00-00 2010 | PURPOSE: The aim of this study was to investigate the constructs of mobility-related participation and user satisfaction, two important outcome dimensions within praxis and research on mobility device interventions. METHOD: To fulfill this aim, validity and reliability of a 12-item scale on mobility-related participation and a 10-item scale on user satisfaction were examined in the context of older people's powered wheelchair use (n = 111). Rasch analysis and correlation analysis were applied. RESULTS: Construct validity of both scales was confirmed. The reliability of the user satisfaction scale was good, while the mobility-related participation scale was not optimal in discriminating between persons with a high degree of mobility-related participation. It was demonstrated that mobility-related participation and user satisfaction are separate, not related constructs. CONCLUSIONS: It can be concluded that the investigated mobility-related participation and user satisfaction constructs appear to be valid. Since the two constructs are not related and both yield important information, both dimensions should be evaluated in outcomes research and praxis targeting powered wheelchair interventions. Reliability problems of the mobility-related participation scale indicate the complexity of this construct. The results have been instrumental in the development of a new scale for measuring mobility-related participation 'The NOMO 1.0'. | Male | Humans | Patient Participation | Patient Satisfaction | OK |
| Michael J. | Levy |  |  |  |  | Seminars in Reproductive Medicine | 2013-05-00 May 2013 | As fertility rates among women of advanced reproductive age have steadily increased, so has the utilization of fertility services. National health policies provide infertility treatment coverage in several developed countries; however, in the United States infertility treatment is largely privately funded, resulting in limited access to care. In response to the lack of insurance coverage, many practices offer fertility treatment on a risk-sharing or contingency fee basis. The ethical delivery of care under the auspices of these programs requires adherence to core principles including transparency, patient autonomy, and the delivery of appropriate medical care. Moreover, concerns regarding patient understanding and decision making have also been of foremost concern. Patients must be able to fully appreciate the financial and clinical implications of contingency fee programs. To further explore patient comprehension and satisfaction, we surveyed participants in our shared risk assisted reproductive technology program. The overwhelming majority of respondents felt adequately informed of and fairly charged for their treatment. Our results demonstrate that shared risk programs can receive strong endorsement from participants, which may lead to improved utilization of and perseverance with fertility treatment. | Male | Humans | Patient Participation | Patient Satisfaction | OK |
| Leif | Jörgensen | Leif | Lohm |  |  | Läkartidningen | 2011-02-02 2011 Feb 2-8 | | Male | Humans | Patient Participation | Patient Satisfaction | OK |
| Deborah | Macmillan | Alexis | McCullough | Victoria L. | Handa | American Journal of Perinatology | 2011-05-00 May 2011 | We sought to describe maternal satisfaction with childbirth among women planning either cesarean or vaginal birth. We enrolled primiparous women planning cesarean birth and a comparison group planning vaginal birth. After delivery, the maternal subjective experience was assessed with a visual analogue satisfaction scale and the Salmon questionnaire, with scale scores for these aspects of the maternal experience of birth: fulfillment, distress, and difficulty. The sample included 160 women planning vaginal birth and 44 women planning cesarean. Eight weeks postpartum, women planning cesarean reported higher satisfaction ratings ( P = 0.023), higher scores for fulfillment ( P = 0.017), lower scores for distress ( P = 0.010), and lower scores for difficulty ( P < 0.001). The least favorable scores were associated with unplanned cesarean ( N = 48). Women planning cesarean reported a more favorable birth experience than women planning vaginal birth, due in part to low satisfaction associated with unplanned cesarean. Maternal satisfaction with childbirth may be improved by efforts to reduce unplanned cesarean, but also by support for maternal-choice cesarean. | Humans | Patient Participation | Patient Satisfaction | Questionnaires | OK |
|  |  |  |  |  |  | Clinics (São Paulo, Brazil) | 2012-00-00 2012 | OBJECTIVE: The participation of humans in clinical cardiology trials remains essential, but little is known regarding participant perceptions of such studies. We examined the factors that motivated participation in such studies, as well as those that led to participant frustration. METHODS: Patients who had participated in hypertension and coronary arterial disease (phases II, III, and IV) clinical trials were invited to answer a questionnaire. They were divided into two groups: Group I, which included participants in placebo-controlled clinical trials after randomization, and Group II, which included participants in clinical trials in which the tested treatment was compared to another drug after randomization and in which a placebo was used in the washout period. RESULTS: Eighty patients (47 patients in Group I and 33 patients in Group II) with different socio-demographic characteristics were interviewed. Approximately 60% of the patients were motivated to participate in the trial with the expectation of personal benefit. Nine participants (11.2%) expressed the desire to withdraw, which was due to their perception of risk during the testing in the clinical trial (Group I) and to the necessity of repeated returns to the institution (Group II). However, the patients did not withdraw due to fear of termination of hospital treatment. CONCLUSIONS: Although this study had a small patient sample, the possibility of receiving a benefit from the new tested treatment was consistently reported as a motivation to participate in the trials. | Male | Humans | Patient Participation | Questionnaires | OK |
|  |  |  |  |  |  | JACC. Heart failure | 2013-08-00 Aug 2013 | Nearly 700,000 emergency department (ED) visits were due to acute heart failure (AHF) in 2009. Most visits result in a hospital admission and account for the largest proportion of a projected $70 billion to be spent on heart failure care by 2030. ED-based risk prediction tools in AHF rarely impact disposition decision making. This is a major factor contributing to the 80% admission rate for ED patients with AHF, which has remained unchanged over the last several years. Self-care behaviors such as symptom monitoring, medication taking, dietary adherence, and exercise have been associated with decreased hospital readmissions, yet self-care remains largely unaddressed in ED patients with AHF and thus represents a significant lost opportunity to improve patient care and decrease ED visits and hospitalizations. Furthermore, shared decision making encourages collaborative interaction between patients, caregivers, and providers to drive a care path based on mutual agreement. The observation that “difficult decisions now will simplify difficult decisions later” has particular relevance to the ED, given this is the venue for many such issues. We hypothesize patients as complex and heterogeneous as ED patients with AHF may need both an objective evaluation of physiologic risk as well as an evaluation of barriers to ideal self-care, along with strategies to overcome these barriers. Combining physician gestalt, physiologic risk prediction instruments, an evaluation of self-care, and an information exchange between patient and provider using shared decision making may provide the critical inertia necessary to discharge patients home after a brief ED evaluation. | Decision Making | Humans | Heart Failure | risk assessment | OK |
| Bertrand | Delclaux | Yan | Martinat | Jean-Michel | Marcos | BMC pulmonary medicine | 2012-00-00 2012 | BACKGROUND: In some situations, practice guidelines do not provide firm evidence-based guidance regarding COPD treatment choices, especially when large trials have failed to identify subgroups of particularly good or poor responders to available medications. METHODS: This observational cross-sectional study explored the yield of four types of multidimensional analyses to assess the associations between the clinical characteristics of COPD patients and pharmacological and non-pharmacological treatments prescribed by lung specialists in a real-life context. RESULTS: Altogether, 2494 patients were recruited by 515 respiratory physicians. Multiple correspondence analysis and hierarchical clustering identified 6 clinical subtypes and 6 treatment subgroups. Strong bi-directional associations were found between clinical subtypes and treatment subgroups in multivariate logistic regression. However, although the overall frequency of prescriptions varied from one clinical subtype to the other for all types of pharmacological treatments, clinical subtypes were not associated with specific prescription profiles. When canonical analysis of redundancy was used, the proportion of variation in pharmacological treatments that was explained by clinical characteristics remained modest: 6.23%. This proportion was greater (14.29%) for non-pharmacological components of care. CONCLUSION: This study shows that, although pharmacological treatments of COPD are quantitatively very well related to patients' clinical characteristics, there is no particular patient profile that could be qualitatively associated to prescriptions. This underlines uncertainties perceived by physicians for differentiating the respective effects of available pharmacological treatments. The methodology applied here is useful to identify areas of uncertainty requiring further research and/or guideline clarification. | Male | Humans | Patient Participation | physicians | OK |
| Nicole | Pélicier | Philippe | Rosset | Etienne | Lemarié | Presse Médicale (Paris, France: 1983) | 2012-03-00 Mar 2012 | CONTEXT: The setting of multidisciplinary meeting (MDM) by the French Cancer Plan has introduced new decisional elements in the patient-physician relationship in oncology. METHODS: To assess the potential impact of MDM on this relationship, a study was conducted at the Tours Hospital: 145 questionnaires were collected from patients whose files have been discussed in MDM, 40 questionnaires were collected from physicians attending these meetings and an analysis of 324 files was performed. RESULTS: Patients recognize the decisional process of MDM as reassuring for 80% of them. However, a majority (73%) expressed that the most important for them is the relationship with the referring physician, almost all (96%) having a total or great confidence in him. The results emphasize that trust appears to be related to the quality of communication, open dialogue and the competence of the doctor in particular in the choice of treatment. A review of files shows that in 91% of cases, the opinion of the RCP is applied and that, in 69% of cases, the referring doctor delivers the information to the patient after MDM. From the physicians' perspective, 33/40 report that the MDM do not alter their relationship with the patient. We note that 35/40 express that the consultation after MDM facilitates the presentation of the decision and 37/40 that the decision is always or often applied in accordance with the opinion of the MDM. CONCLUSION: MDM appears in most cases in this study not to modify the patient-physician relationship. Due to the patient confidence into the referring physician, the role of this one is essential in integrating the decisional multidisciplinary opinion of MDM and it is important to ensure from his/her disengagement in the decisional process. | Decision Making | Humans | Patient Participation | Patient Satisfaction | OK |
| Michael | Poch | Brady T. | West | Paula M. | Lantz | The Canadian Journal of Urology | 2010-10-00 Oct 2010 | OBJECTIVE: Patients turn to their physicians for information and guidance when making a prostate cancer treatment decision. The objectives of this study were to determine the likelihood of men consulting with and receiving treatment recommendations from different providers (urologists, radiation oncologists, and primary care physicians), the content of these recommendations, the perceived influence of recommendations and which recommendations, if any, were associated with prostate cancer treatment decisions. METHODS: One hundred and fifty-eight participants with localized prostate cancer completed a survey regarding their treatment decision-making process. Associations between treatment choice and urologist recommendations, consultations with radiation oncologists and primary care physicians, potential side effects and other factors were examined using regression analysis. RESULTS: Among men consulting multiple providers, more than half received at least one treatment recommendation. Most men chose a treatment recommended by at least one provider. The likelihood of choosing a treatment increased when the urologist recommended it. Consulting a radiation oncologist decreased and increased likelihood of choosing a radical prostatectomy and radiation, respectively. CONCLUSION: Most men consulted multiple providers and received multiple treatment recommendations. Recommendations appear to play a significant role in prostate cancer treatment decision-making. | Male | Decision Making | Humans | Patient Participation | OK |
|  |  |  |  |  |  | The Johns Hopkins Medical Letter Health After 50 | 2010-04-00 Apr 2010 | | Male | Decision Making | Humans | Patient Participation | OK |
| Marcus A. | Doel | Aled | Jones | Matteo | Cella | Social Psychiatry and Psychiatric Epidemiology | 2010-03-00 Mar 2010 | BACKGROUND: Integration of patient views in mental health service planning is in its infancy despite service provision being clearly dominated by narratives from professional consultations and medical records. We wished to clarify perceptions of uncertainty about mental health conditions from a range of provider and user perspectives (patients, carers, parents, mental health service providers) and understand the role of narratives in mental health research. AIMS: (1) To explore the utility of qualitative research methods, particularly narrative content analysis in mental health research, and (2) identify aspects of uncertainty in mental health service users and providers. MATERIAL: Four hundred and six free text responses were considered as one element of an existing questionnaire about uncertainties about mental health treatments, collected from mental healthcare users and providers through charities, the Mental Health Research Network in Wales, health professionals and websites. Free text responses were analysed using narrative content analysis, an elaborate and rigorous research technique that involves groups of analysts working independently and together over extended group sessions. FINDINGS: Three main themes emerged across respondent groups: "medication and treatment options", "objectification and marginalisation of patient" and "integrity of service delivery". Within these, patients embraced the opportunity to write about their illness at length, whilst carers' and parents' main concerns were about how patients were dealing with their illnesses, the services they were getting and the side effects of treatments. Carers and patients' parents perceived themselves to be the 'go-between', carrying messages between patients and professionals, in order to enable services to function. Mental health service providers and professionals considered uncertainties surrounding medication and treatment from an 'evidence-base' perspective, concentrating on medication choices and the adoption of new approaches to care rather than patient need and expectation. Patients wanted to know what alternatives were available to the drug regimes they were on and felt their opinions were rarely listened to. As a consequence patients felt marginalised by the health systems there to support them and by society as a whole. CONCLUSIONS: Narrative content analysis can help distil large amounts of free text data and enable their successful interpretation. Listening to patients' voices should become an integral part of routine service evaluation and may help bring patient expectation more in line with service organisation and delivery towards an optimal delivery of care. | Humans | Patient Participation | Questionnaires | community mental health services | OK |
| A. | Mohr | M. | Schrader |  |  | Der Urologe. Ausg. A | 2013-09-00 Sep 2013 | BACKGROUND: The national second opinion project of the German Testicular Cancer Study Group (GTCSG) has served to improve the quality of care provided to testicular cancer patients since 2006. AIM: A recent online survey was carried out to characterize the users of the second opinion offer and clarify their motivation for participating in the project. Furthermore, the aim was to identify weaknesses of the project which could be improved. A total of 440 users of the second opinion project were contacted of whom 192 participated in the survey. RESULTS: In summary, the data collected showed a high degree of satisfaction among the participants who appreciated the second opinions received. Some issues with a need for improvement, predominantly in the structural organisational area, were disclosed. These served as a basis for a recently completed revision of the project immanent internet-based communication platform with a new data mask facilitating the introduction of patients with relapsed tumors. Interestingly, a high proportion of survey participants expressed the desire for establishment of a similar second opinion project for patients with penile cancer (77.1% of the participants). | Male | Humans | Patient Participation | Patient Satisfaction | OK |
| Paulette F. | Egberts | Sophie C. E. | van Blijswijk | Willem J. J. | Assendelft | Annals of Family Medicine | 2013-10-00 2013 Sep-Oct | PURPOSE: Because cutaneous warts resolve spontaneously and available treatments often fail, family physicians and patients may consider a wait-and-see policy. We examined the natural course of cutaneous warts and treatment decisions in a prospective observational cohort of primary schoolchildren. METHODS: We inspected the hands and feet of children aged 4 to 12 years from 3 Dutch primary schools for the presence of warts at baseline and after a mean follow-up of 15 months. Parental questionnaires at follow-up provided information on inconvenience caused by warts and any treatments used. RESULTS: Of the 1,134 eligible children, 1,099 (97%) participated, of whom 366 (33%) had cutaneous warts at baseline. Among these children with warts, loss to follow-up was 9% and the response rate to the parental questionnaires was 83%. The complete resolution rate was 52 per 100 person-years at risk (95% CI, 44-60). Younger age (hazard ratio = 1.1 per year decrease; 95% CI, 1.0-1.2) and non-Caucasian skin type (hazard ratio = 2.0; 95% CI, 1.3-2.9) increased the likelihood of resolution. During follow-up, 38% of children with warts at baseline treated their warts: 18% used over-the-counter treatment only, 15% used a family physician-provided treatment only, and 5% used both. Children were more likely to initiate treatment if the warts measured at least 1 cm in diameter (odds ratio = 3.2; 95% CI, 1.9-5.3) and especially if parents reported that the warts caused inconvenience (odds ratio = 38; 95% CI, 16-90). CONCLUSIONS: One-half of primary schoolchildren with warts will be free of warts within 1 year. Young age and non-Caucasian skin type enhance resolution. Children with large or inconvenient warts are more likely to start treatment. These findings will be useful in the process of shared decision making with parents and children. | Male | Decision Making | Humans | Patient Participation | Doublon |
| Paulette F. | Egberts | Sophie C. E. | van Blijswijk | Willem J. J. | Assendelft | Annals of Family Medicine | 2013-10-00 2013 Sep-Oct | PURPOSE: Because cutaneous warts resolve spontaneously and available treatments often fail, family physicians and patients may consider a wait-and-see policy. We examined the natural course of cutaneous warts and treatment decisions in a prospective observational cohort of primary schoolchildren. METHODS: We inspected the hands and feet of children aged 4 to 12 years from 3 Dutch primary schools for the presence of warts at baseline and after a mean follow-up of 15 months. Parental questionnaires at follow-up provided information on inconvenience caused by warts and any treatments used. RESULTS: Of the 1,134 eligible children, 1,099 (97%) participated, of whom 366 (33%) had cutaneous warts at baseline. Among these children with warts, loss to follow-up was 9% and the response rate to the parental questionnaires was 83%. The complete resolution rate was 52 per 100 person-years at risk (95% CI, 44-60). Younger age (hazard ratio = 1.1 per year decrease; 95% CI, 1.0-1.2) and non-Caucasian skin type (hazard ratio = 2.0; 95% CI, 1.3-2.9) increased the likelihood of resolution. During follow-up, 38% of children with warts at baseline treated their warts: 18% used over-the-counter treatment only, 15% used a family physician-provided treatment only, and 5% used both. Children were more likely to initiate treatment if the warts measured at least 1 cm in diameter (odds ratio = 3.2; 95% CI, 1.9-5.3) and especially if parents reported that the warts caused inconvenience (odds ratio = 38; 95% CI, 16-90). CONCLUSIONS: One-half of primary schoolchildren with warts will be free of warts within 1 year. Young age and non-Caucasian skin type enhance resolution. Children with large or inconvenient warts are more likely to start treatment. These findings will be useful in the process of shared decision making with parents and children. | Male | Decision Making | Humans | Patient Participation | OK |
| Susan C. | Scott | Lois E. | Finch | Sydney | Miller | Quality of Life Research: An International Journal of Quality of Life Aspects of Treatment, Care and Rehabilitation | 2014-02-00 Feb 2014 | INTRODUCTION: Participation, a construct within the disability/functioning framework, is evaluated on a person's involvement in life situations including family, community, work, social, and civic life. In the context of recovering from a major health event, participation is a treatment goal and it is known to correlate with the quality of life. OBJECTIVE: The purpose of this study is to track the dynamics of participation post-stroke in relationship to the dynamics of walking capacity, social support, and mood. METHODS: An inception cohort was followed over the first post-stroke year. Group-based trajectory analysis, a form of latent class analysis, was used to identify distinctive groups of individuals with similar trajectories. Dual trajectories were used to estimate concordance between participation trajectory and trajectories for each of the three constructs under study. RESULTS: From the sample of 102 persons (mean age 70), four trajectories of participation were identified, two of which were qualified as excellent and very good, and two qualified as fair and poor. All those with excellent walking showed excellent participation. However, people with excellent (and very good) community participation had a range of walking capacities. Most (82%) people with normal mood showed excellent participation. People with good mood but not meeting norms for age showed the complete range of participation trajectories from excellent to poor. The higher proportion of people with excellent or good social support (57%) showed excellent participation. CONCLUSION: Two treatable component causes of participation, walking capacity and mood, were identified; of these, only excellent walking capacity could be considered a sufficient cause. | Male | Humans | Patient Participation | Questionnaires | OK |
|  |  |  |  |  |  | Journal of Cancer Survivorship: Research and Practice | 2011-09-00 Sep 2011 | INTRODUCTION: The purpose of this study was to examine the relationship between needs, expectations, and attendance patterns among users of a cancer wellness centre to better access future programming and improve attendance. METHODS: We interviewed staff from 18 cancer wellness centers in Canada and the United States to assess if our attendance expectations were realistic. Then, from a total sample of non-users in 2008 (n  =  67) and infrequent users (n  =  67) at the Hope & Cope Wellness Centre in Montreal, we randomly selected individuals from each group who registered and never returned (n  =  16), and those who registered and returned 1-3 times (n  =  17). Telephone interviews were conducted using a brief questionnaire. We used a phenomenological approach to assess the meaning of attendance for the registrants. RESULTS: Both groups of participants described similar reasons for not attending such as health complications, scheduling conflicts, child care, language issues, and parking restrictions. However, non-users focused specifically on functional aspects such as administrative issues and scheduling, and distance from the centre, while infrequent users expressed more emotional disappointments such as feeling more depressed and inability to engage with others. DISCUSSION: Strategies are suggested to optimize service delivery for short-term attendees, including home-exercise programming, virtual support, outreach systems, and caregiving support. Information from other wellness centers in Canada and the United States revealed similar trends in attendance but also revealed a wide range of programs and systems of data recording. IMPLICATIONS: The results of this study have implications for cancer survivors, for health care providers, for the development of strategies to address participation barriers, and for future research on understanding optimal use of wellness centers. | Male | Emotions | Humans | Patient Participation | OK |
| Vivien | Yadegar | Derjung M. | Tarn |  |  | Patient Education and Counseling | 2012-12-00 Dec 2012 | OBJECTIVE: To empirically investigate the ways in which patients and providers discuss Complementary and Alternative Medicine (CAM) treatment in primary care visits. METHODS: Audio recordings from visits between 256 adult patients aged 50 years and older and 28 primary care physicians were transcribed and analyzed using discourse analysis, an empirical sociolinguistic methodology focusing on how language is used to negotiate meaning. RESULTS: Discussion about CAM occurred 128 times in 82 of 256 visits (32.0%). The most frequently discussed CAM modalities were non-vitamin, non-mineral supplements and massage. Three physician-patient interactions were analyzed turn-by-turn to demonstrate negotiations about CAM use. Patients raised CAM discussions to seek physician expertise about treatments, and physicians adopted a range of responses along a continuum that included encouragement, neutrality, and discouragement. Despite differential knowledge about CAM treatments, physicians helped patients assess the risks and benefits of CAM treatments and made recommendations based on patient preferences for treatment. CONCLUSION: Regardless of a physician's stance or knowledge about CAM, she or he can help patients negotiate CAM treatment decisions. PRACTICE IMPLICATIONS: Providers do not have to possess extensive knowledge about specific CAM treatments to have meaningful discussions with patients and to give patients a framework for evaluating CAM treatment use. | Male | Decision Making | Humans | Patient Participation | OK |
| Marit | Svisdahl | Marius | Veseth |  |  | Disability and Rehabilitation | 2012-00-00 2012 | PURPOSE: Traditionally, the voices of service users have been silent in research into mental health issues. A Norwegian research network, however, recognizes the importance of involving service users as coresearchers and initiated a training program in research methodology and design intended to empower them as active participants in research projects. In this article, we explore how these coresearchers with a mental health service user background experience their participation in projects as well as in attending the training: What is it like being a service user coresearcher in collaborative studies on issues in mental health? How do coresearchers negotiate their roles and mandate? METHOD: We used focus groups as our data collection method, transcribed the group discussions verbatim, and analyzed the transcriptions using qualitative methodology. We then took the preliminary analyses back to the participants for discussion, auditing, and reanalysis. RESULTS: We identified themes that represent important social processes around which the participants developed a consensual understanding: self-definition, constructive differentiation and negotiations. CONCLUSION: Our findings generate hypotheses on how participatory research into mental health issues can be fruitfully organized, in a way that empowers service users to active and constructive participation. | Humans | Patient Participation | Questionnaires | Cooperative Behavior | OK |
| Franziska | Kämpf | Peter | Joraschky | Andreas | Maercker | Zeitschrift Für Psychosomatische Medizin Und Psychotherapie | 2010-00-00 2010 | OBJECTIVES: Adjustment disorders (AD) represent a frequently diagnosed type of disorder for which scientific interest has been limited because of insufficient diagnostic criteria. This study presents a concept comprising cognitive, behavioural, and emotional criteria (ADNM) which investigates differences in symptom severity, willingness to change, and motivation for psychotherapy in subjects with adjustment disorders. METHOD: 331 outpatients were examined with a number of tools (ADNM, SCL-90-R, FPTM-23, FEVER). The ADNM is evaluated on the basis of theoretical assumptions and divides the sample into subjects with elevated and normal scores. RESULTS: The sample comprised patients with elevated scores (61%) and normal scores (39%).Subjects with AD scored higher on depression, anxiety, and impulsivity as well as exhibiting elevated scores on the SCL-90-R, for contemplation/action, and for psychotherapy motivation. CONCLUSION: The ADNM identifies patients experiencing stress. Further longitudinal studies should examine whether patients more frequently enter psychotherapy or not. | Male | Humans | Patient Participation | Young Adult | OK |
| Alma | Kuby | Wendy | Levinson | Clarence Henry | Braddock | Patient Education and Counseling | 2012-03-00 Mar 2012 | OBJECTIVE: To explore older adults' views of existing informed decision making (IDM) elements and investigate the need for additional elements. METHODS: We recruited persons 65 and older to participate in six focus groups. Participants completed questionnaires about IDM preferences, and discussed videotapes of idealized patient-physician interactions in light of seven IDM elements: (1) discussion of the patient's role in decision making; (2) discussion of the clinical issue; (3) discussion of alternatives; (4) discussion of benefits/risks; (5) discussion of uncertainties; (6) assessment of patient understanding; and (7) exploration of patient preference. We used a modified grounded theory approach to assess agreement with existing IDM elements and identify new elements. RESULTS: In questionnaires, 97-100% of 59 participants rated each IDM element as "somewhat" or "very" important. Qualitative analysis supported existing elements and suggested two more: opportunity for input from trusted others, and discussion of decisions' impacts on patients' daily lives. Elements overlapped with global communication themes. CONCLUSION: Focus groups affirmed existing IDM elements and suggested two more with particular relevance for older patients. PRACTICE IMPLICATIONS: Incorporation of additional IDM elements into clinical practice can enhance informed participation of older adults in decision making. | Male | Decision Making | Humans | Patient Participation | OK |
| Mark D. | Schwartz | Eric S. | Holmboe | Tavinder K. | Ark | Academic Medicine: Journal of the Association of American Medical Colleges | 2010-05-00 May 2010 | Researchers lack the rich evidence base and benchmark patient outcomes needed to evaluate the effectiveness of medical education practice and guide policy. The authors offer a framework for medical education research that focuses on physician-influenced patient outcomes that are potentially sensitive to medical education. Adapting the concept of ambulatory care sensitive conditions, which provided traction to health services research by defining benchmark patient outcomes to measure health system performance, the authors introduce the concept and propose the adoption of educationally sensitive patient outcomes and suggest two measures: patient activation and clinical microsystem activation. They assert that the ultimate goal of medical education is to ensure that measurement of future physicians' competence and skills is based not only on biomedical knowledge and critical clinical skills but also on the ability to translate these competencies into effective patient- and systems-level outcomes. The authors consider methodological approaches and challenges to measuring such outcomes and argue for large, multiinstitutional, prospective cohort studies and the development of a national Database for Research in Education in Academic Medicine to provide the needed infrastructure. They advocate taking the next steps to establish an educational evidence base to guide the academic medical centers of the 21st century in aligning medical education practice with health care delivery that meets the needs of individuals and populations. | Humans | Patient Participation | Professional Competence | Outcome Assessment (Health Care) | OK |
| A. | Gautier | J. | Warszawski | B. | Riandey | Revue D'épidémiologie Et De Santé Publique | 2011-04-00 Apr 2011 | BACKGROUND: Survey non-response rates are important quality indicators. Refusal rates can induce non-response bias in health survey estimates. However, comparisons across surveys highlight inconsistencies in the use of survey outcome categories and in the calculation of response rates. In this paper we discuss the relevance of these indicators and suggest other survey quality indicators. METHODS: Outcome rates from two French random-digit dialing (RDD) telephone surveys are compared : the Nicolle survey on infectious diseases of 4112 individuals conducted in 2006, and the HIV knowledge, attitude, belief and practices (KABP) survey of 5071 individuals in 2004. Based on the same protocol, we describe in details the way the two RDD samples were drawn and how non-response rates were estimated. RESULTS: Non-response rates were different: 36% in Nicolle survey and 18% in KABP survey. However, the quantity of telephone numbers required to obtain one interview was higher in the KABP survey: 2.8 telephone numbers versus 2.1 in the Nicolle survey. The participation rates, aggregating together refusals, break-off and non-reachable numbers, were equivalent for the two surveys. This result occurred because of a greater proportion of unreached calls in the KABP surveys, which is not integrated into the non-response rates commonly used. CONCLUSION: Survey non-response rate is insufficient to estimate the quality of a survey. The need for other indicators has been previously stressed in the literature, notably with the adoption and utilization of the American Association for Public Opinion Research (AAPOR) standard definitions of four indicators. But these indicators are quite complex for evaluating non-response bias between surveys. In addition to the classical refusal rate, two other indicators are proposed in this paper: participation rate (number of complete interviews divided by the number of eligible and of unknown eligibility units) and a liking contact rate (number of unreachable units because of a long absence, break-off or non-answer divided by the number of eligible and of unknown eligibility units). The sum of these three indicators is equal to 100% and thus easier to manipulate when comparing surveys. | Humans | Patient Participation | Questionnaires | Aged | OK |
| France | Légaré | Jürgen | Kasper |  |  | Zeitschrift Für Evidenz, Fortbildung Und Qualität Im Gesundheitswesen | 2012-00-00 2012 | | Decision Making | Patient Participation | Evidence-based medicine | Health Services Needs and Demand | OK |
| Nathe | François | Julie | Bordeleau | Sonia | Lupien | BMC medical informatics and decision making | 2013-00-00 2013 | BACKGROUND: Despite the increasing pervasiveness of mobile computational technologies, knowledge about psychiatric patients' preferences regarding the design and utility of mobile applications is very poor. This paper reports on a pilot-study that involved 120 psychiatric patients in the development of a mobile application (app) that is being used for data entry into the Signature Project data bank at the Institut universitaire en santé mentale de Montréal (IUSMM), Canada. Participants were invited to comment on the 'look and feel' of the Signature App. Their input also extended the procedures for data collection. These suggestions may contribute to increased mental health literacy and empowerment of persons with mental illness receiving services at the IUSMM. METHODS: Participants were recruited to fill out a questionnaire on a tablet computer while waiting at the Emergency Room (ER, n = 40), Psychotic Disorders outpatient clinic (n = 40) or Anxiety and Mood Disorders outpatient clinic (n = 40) of IUSMM. Nine patients from each of these sub-groups participated in a focus group to review the results and to discuss how the design and use of the Signature App could be improved to better meet the needs of patients. RESULTS: This study (n = 120) indicated that psychiatric patients are clearly capable of using a tablet computer to fill out questionnaires for quantitative data entry, and that they enjoyed this experience. Results from the focus groups (n = 27) highlight that the app could also be used by patients to communicate some personal and contextual qualitative information. This would support a holistic and person-centered approach, especially at the ER where people acutely need to describe their recent history and receive emotional support. CONCLUSIONS: This pilot-study has confirmed the necessity of involving patients not only in the testing of a new mobile application, but also as active contributors in the entire research and development process of a person-centered information and communication technology infrastructure. The input of participants was essential in designing the Signature Project computational procedure and making use of the app a positive and empowering experience. Participants also gave critical feedback remarks that went beyond the initial scope of the pilot-study, for example they suggested the addition of a client-clinician component. | Humans | Patient Participation | Questionnaires | Video Recording | OK |
| Asgeir R. | Helgason | Peter | Allebeck |  |  | Substance Abuse Treatment, Prevention, and Policy | 2013-00-00 2013 | BACKGROUND: This study examines whether alcohol-related hospitalization predicts survey non-response, and evaluates whether this missing data result in biased estimates of the prevalence of hazardous alcohol use and abstinence. METHODS: Registry data on alcohol-related hospitalizations during the preceding ten years were linked to two representative surveys. Population data corresponding to the surveys were derived from the Stockholm County registry. The alcohol-related hospitalization rates for survey responders were compared with the population data, and corresponding rates for non-responders were based on the differences between the two estimates. The proportions with hazardous alcohol use and abstinence were calculated separately for previously hospitalized and non-hospitalized responders, and non-responders were assumed to be similar to responders in this respect. RESULTS: Persons with previous alcohol-related admissions were more likely currently to abstain from alcohol (RR=1.58, p<.001) or to have hazardous alcohol use (RR=2.06, p<.001). Alternatively, they were more than twice as likely to have become non-responders. Adjusting for this skewed non-response, i.e., the underrepresentation of hazardous users and abstainers among the hospitalized, made little difference to the estimated rates of hazardous use and abstinence in total. During the ten-year period 1.7% of the population were hospitalized. CONCLUSIONS: Few people receive alcohol-related hospital care and it remains unclear whether this group's underrepresentation in surveys is generalizable to other groups, such as hazardous users. While people with severe alcohol problems--i.e. a history of alcohol-related hospitalizations--are less likely to respond to population surveys, this particular bias is not likely to alter prevalence estimates of hazardous use. | Male | Humans | Patient Participation | Female | OK |
| D. | Singer | S. J. | Clark | A. | Butchart | Public Health Genomics | 2010-00-00 2010 | BACKGROUND: State newborn screening (NBS) programs are considering the storage and use of NBS blood samples for research. However, no systematic assessment of parents' attitudes exists. METHODS: We conducted an Internet-based survey of a nationally representative parent sample. We examined parents' willingness (1) to permit use of their children's NBS samples for research with/without their permission and (2) to allow NBS sample storage. Using bivariate and multinomial logistic regression, we examined the association of parent and child characteristics with parents' willingness to permit NBS sample storage and use for research, respectively. RESULTS: The response rate was 49.5%. If permission is obtained, 76.2% of parents were 'very or somewhat willing' to permit use of the NBS sample for research. If permission is not obtained, only 28.2% of parents were 'very or somewhat willing'. Of parents surveyed, 78% would permit storage of their children's NBS sample. Parents who refused NBS sample storage were also less willing to permit use of the NBS sample for research. CONCLUSIONS: Three-quarters of parents would permit use of their children's NBS samples for research - if their permission is obtained. Parents not in favor of storing NBS samples often opposed the use of NBS samples for research. | Male | Humans | Patient Participation | Questionnaires | OK |
| Steve | Holmes | Peter | Kardos | Roger | Escamilla | BMC medical research methodology | 2013-00-00 2013 | The quality of a consultation provided by a physician can have a profound impact on the quality of care and patient engagement in treatment decisions. When the COPD Assessment Test (CAT) was developed, one of its aims was to aid the communication between physician and patient about the impact of COPD. We developed a novel study design to assess this in a primary care consultation. Primary care physicians across five countries in Europe conducted videoed consultations with six standardised COPD patients (played by trained actors) which had patient-specific issues that the physician needed to identify through questioning. Half the physicians saw the patients with the completed CAT, and half without. Independent assessors scored the physicians on their ability to identify and address the patient-specific issues, review standard COPD aspects, their understanding of the case and their overall performance. This novel study design presented many challenges which needed to be addressed to achieve an acceptable level of robustness to assess the utility of the CAT. This paper discusses these challenges and the measures adopted to eliminate or minimise their impact on the study results. | Humans | Patient Participation | Physician-Patient Relations | Video Recording | OK |
| Anneli | Ozanne | Hanna | Falk | Kristin | Falk | Nurse Education in Practice | 2014-03-00 Mar 2014 | BACKGROUND: Nursing education can positively affect nurses' attitudes toward nursing research, resulting in better patient outcomes. Experiential learning theory was the basis for this study. OBJECTIVES: To explore nursing students' experiences of involvement in clinical research, their approach to learning and their interest in nursing research. DESIGN: Cross-sectional. METHODS: One hundred and twenty-six nursing students were invited to be involved as data collectors in a research project as part of their training in research methodology. The students completed an evaluation form and the Revised Study Process Questionnaire. The questionnaires were analyzed quantitatively and one open-ended question was analyzed qualitatively. RESULTS: On the whole, the students were happy to be involved in the data collection although a minority felt uncertain and exposed. Students with a deeper approach to learning felt that their involvement had increased their interest in nursing research and they stated that data collection should be a regular feature of the course. CONCLUSIONS: Participation as data collectors in research has the potential to increase interest in nursing research among students with higher levels of deep learning. Further studies are needed to examine ways to increase interest in research among students with lower levels of deep learning. | Male | Humans | Patient Participation | Questionnaires | OK |
|  |  |  |  |  |  | Arerugī = [Allergy] | 2012-01-00 Jan 2012 | | Humans | Patient Participation | Severity of Illness Index | Biological Markers | OK |
|  |  |  |  |  |  | Zeitschrift Für Evidenz, Fortbildung Und Qualität Im Gesundheitswesen | 2012-00-00 2012 | OBJECTIVE: Shared decision making (SDM) between patient and physician is an interpersonal process. Most SDM measures use the view of one party (patient, physician or observer) as a proxy to capture this process although these views typically diverge. This study suggests the compound measure SDM(MASS) (SDM Meeting its concept's ASSumptions) integrating these three perspectives in one single index. METHODS: SDM(MASS) was derived theoretically and compared empirically to unilateral perspectives of patients, physicians and observers by application to a data set of 10 physicians (40 consultations) receiving an SDM training. RESULTS: The constituting parts of SDM(MASS) were highly reliable (Cronbach's alpha .94; interrater reliability .74-.87). Unilateral appraisal of training effects was divergent. SDM(MASS) revealed no effect. CONCLUSION: SDM(MASS) combines noteworthy information about SDM processes from different viewpoints and thereby delivers plausible assessments. It could overcome immanent shortcomings of unilateral approaches. However, it is a complex measure needing further validation. | Male | Decision Making | Humans | Physician-Patient Relations | OK |
| SangNam | Ahn | Terry L. | Bazzarre | Barbara | Resnick | The Gerontologist | 2011-12-00 décembre 2011 | Purpose of the Study: The Exercise Assessment Screening for You (EASY) tool was developed to encourage older adults at every functional level to be more physically active. The purposes of this study were to examine characteristics of older adults who participated in an evidence-based falls prevention program by their entry to EASY tool scores, associations between EASY tool responses and class completion, and influences of EASY scores and items on improvements in days limited from usual activity and unhealthy physical health days. Methods and Design: The program consisted of eight sessions conducted statewide by trained lay leaders in Texas. Assessments were collected at the beginning of session 1 and conclusion of session 8. Data included responses of 2,235 older adults. Logistic regression analyses were performed to assess the influence of individual EASY items and cumulative scores on health outcomes from baseline to postintervention. Results: Neither the composite EASY scores nor the individual items significantly influenced program attendance. Higher cumulative EASY scores and individual items were associated with improvements in both health outcomes. Implications: The EASY tool enabled seniors with major health problems to enroll in falls prevention classes, without the additional burden of requiring medical consent for participation. (PsycINFO Database Record (c) 2013 APA, all rights reserved). (journal abstract) | Client Participation | treatment outcomes | older adults | falls prevention exercise program | Doublon |
| SangNam | Ahn | Terry L. | Bazzarre | Barbara | Resnick | The Gerontologist | 2011-12-00 Dec 2011 | PURPOSE OF THE STUDY: The Exercise Assessment Screening for You (EASY) tool was developed to encourage older adults at every functional level to be more physically active. The purposes of this study were to examine characteristics of older adults who participated in an evidence-based falls prevention program by their entry to EASY tool scores, associations between EASY tool responses and class completion, and influences of EASY scores and items on improvements in days limited from usual activity and unhealthy physical health days. METHODS AND DESIGN: The program consisted of eight sessions conducted statewide by trained lay leaders in Texas. Assessments were collected at the beginning of session 1 and conclusion of session 8. Data included responses of 2,235 older adults. Logistic regression analyses were performed to assess the influence of individual EASY items and cumulative scores on health outcomes from baseline to postintervention. RESULTS: Neither the composite EASY scores nor the individual items significantly influenced program attendance. Higher cumulative EASY scores and individual items were associated with improvements in both health outcomes. IMPLICATIONS: The EASY tool enabled seniors with major health problems to enroll in falls prevention classes, without the additional burden of requiring medical consent for participation. | Male | Humans | Patient Participation | Female | OK |
| Ross | Larsen |  |  |  |  | Women's Health Issues: Official Publication of the Jacobs Institute of Women's Health | 2010-12-00 2010 Nov-Dec | BACKGROUND: Older women who are vulnerable to falls and their negative consequences have been shown, in controlled randomized clinical trials, to benefit from fall prevention programs. The purpose of this study was to identify personal characteristics of female participants enrolled in a falls prevention program, the effectiveness of the program for female participants, and whether personal characteristics indicate which women might benefit most from programs delivered in real-world settings. METHODS: Data were collected from seniors enrolled in A Matter of Balance/Voluntary Lay Leader (AMOB/VLL) program sessions conducted in Texas over the 2-year period from 2007 to 2009. Baseline and postintervention data from 1,101 female participants were drawn from a larger, state-wide dataset and analyzed using structural equation modeling to identify relationships between variables of interest. FINDINGS: Analyses revealed that women who attended AMOB/VLL significantly increased falls efficacy from baseline to postintervention (t = 1.680; p < .05; d = 0.143) and reduced the number of times fallen (t = 3.790; p < .01; d = 0.313). Further, participants reported decreases in days of physical (t = 3.810; p < .01; d = 0.323) and mental health (t = 1.850; p < .05; d = 0.156) reported as not good. CONCLUSION: Findings from this study support the effectiveness of evidence-based programs for reducing falls-related risks in older women. Identifying the characteristics of female participants enrolled in AMOB/VLL can enable public health professionals to better target and meet the health demands of the aging female population. Such translational research can help to guide the dissemination of additional state-wide health promotion programs for older women. | Humans | Patient Participation | Questionnaires | Female | OK |
|  |  |  |  |  |  | Oncology Nursing Forum | 2010-05-00 May 2010 | PURPOSE/OBJECTIVES: To examine how oncology nurses define palliative care, views about who should and should not receive palliative care, and beliefs about palliative care decision making, including who should be involved and how decisions should be managed. DESIGN: Qualitative interviews and analysis. SETTING: Preferred location of each respondent. SAMPLE: 12 nurses representing different aspects of oncology nursing. METHODS: An interview guide was employed to ensure that specific topics were covered. Interviews were transcribed verbatim. Qualitative analysis consisted of independent, multiple reviews of the transcripts to share initial findings and identify, refine, and reach consensus on major themes and subthemes. MAIN RESEARCH VARIABLES: Nurses' definitions of palliative care, views about who should and should not receive palliative care, and beliefs about palliative care decision making. FINDINGS: Nurses' perceptions of palliative care focused on symptom management. Most did not distinguish between palliative care and hospice and believed that only patients who were near the end of life should receive palliative care. They viewed their role in decisions regarding palliative care to be limited and indirect. CONCLUSIONS: Although oncology nurses should be at the cutting edge with regard to palliative care, these nurses' personal understandings could serve to limit care for many patients with cancer who could benefit from it. IMPLICATIONS FOR NURSING: Education and clinical experience embedded in a continuous quality-improvement model are needed to ensure sustained change that will overcome the multiple, interwoven barriers to providing appropriate palliative care. | Male | Decision Making | Humans | Patient Participation | OK |
| S. | Salloch | W. | Uhl | J. | Vollmann | Annals of oncology: official journal of the European Society for Medical Oncology / ESMO | 2013-09-00 Sep 2013 | BACKGROUND: Information about diagnosis, treatment options and prognosis has been emphasized as a key to empower cancer patients to make treatment decisions reflecting their values. However, surveys indicate that patients' preferences regarding information and treatment decision-making differ. In this qualitative interview study, we explored pancreatic cancer patients' perceptions and preferences on information and treatment decision-making. PATIENTS AND METHODS: Qualitative in-depth interviews with patients with pancreatic cancer. Purposive sampling and qualitative analysis were carried out. RESULTS: We identified two stages of information and treatment decision-making. Patients initially emphasize trust in their physician and indicate rather limited interest in details about surgical and medical treatment. In the latter stage of disease, patients perceive themselves more active regarding information seeking and treatment decision-making. All patients discuss their poor prognosis. Reflecting on their own situation, all patients interviewed pointed out that hope was an important driver to undergo further treatment also in advanced stages of the disease. Interviewees unanimously emphasized the difficulty of anticipating the time at which stopping cancer treatment would be the right decision. CONCLUSIONS: The findings can serve as starting point for reflection on professional decision-making in pancreatic cancer and larger representative surveys on ethical issues in treatment decision-making in pancreatic cancer. | Male | Decision Making | Humans | Patient Participation | OK |
|  |  |  |  |  |  | Journal of the American Society of Echocardiography: Official Publication of the American Society of Echocardiography | 2014-02-00 Feb 2014 | | Humans | Patient Participation | United States | Primary Prevention | OK |
| Loren | Brener | L. Clair | Jackson | Priscilla | Johnson | Ethnicity & Health | 2013-00-00 2013 | OBJECTIVES: Cancer is the second biggest killer of Aboriginal Australians. For some cancers, the mortality rate is more than three times higher in Aboriginal people than for non-Aboriginal people. The Aboriginal Patterns of Cancer Care Study explored barriers to and facilitators of cancer diagnosis and treatment among Aboriginal and Torres Strait Islander people in New South Wales. DESIGN: Our team--which includes both Aboriginal and non-Aboriginal researchers--conducted in-depth interviews between 2009 and 2010 with Aboriginal people with cancer, their carers and health professionals who care for them. In this paper, we identify recurrent patterns of 'discursive framing' in the 16 interviews with health care professionals. We are particularly interested in how these frames assisted participants in constructing a professional position on what 'cultural difference' means for the design and delivery of cancer care services to Aboriginal people. RESULTS: Despite geographical, organisational, disciplinary and cultural diversity, these interview participants consistently drew upon six discursive frames, which we have interpreted as either eliding a discussion of difference ('everyone is the same' and 'everyone is different') or facilitating that discussion ('different priorities,' 'different practices' and 'making difference safe'). An additional strategy appeared to actively resist either of these positions but then tended to ultimately prioritise the eliding frames. CONCLUSIONS: While none of our participants were dismissive of the idea that cultural identity might matter to Aboriginal people, their reliance upon familiar narratives about what that means for cancer care services has the potential to both symbolically and practically exclude the voices of a group of people who may already feel disenfranchised from the mainstream health care system. Critically unpacking the 'taken for granted' assumptions behind how health care professionals make sense of cultural difference can enrich our understanding of and response to the care needs of indigenous people affected by cancer. | Male | Humans | Patient Participation | Questionnaires | OK |
| Kathrin | Sommerhalder | Peter J. | Schulz |  |  | Qualitative Health Research | 2010-08-00 Aug 2010 | A growing number of patients search for medical information on the Internet. Understanding how they use the Internet is important, as this might impact their health, patient-practitioner roles, and general health care provision. In this article, we illustrate the motives of online health information seeking in the context of the doctor-patient relationship in Switzerland. We conducted semistructured interviews with patients who searched for health information online before or after a medical consultation. Findings suggest that patients searched for health information online to achieve the goals of preparing for the consultation, complementing it, validating it, and/or challenging its outcome. The initial motivations for online health information seeking are identified in the needs for acknowledgment, reduction of uncertainty, and perspective. Searching health information online was also encouraged by personal and contextual factors, that is, a person's sense of self-responsibility and the opportunity to use the Internet. Based on these results, we argue that online health information seeking is less concerned with what happens during the consultation than with what happens before or after it, in the sociocultural context. | Male | Humans | Patient Participation | Patient Satisfaction | OK |
| Peter | Rust | Elizabeth A. | Schackmann | Michael | Smith | Journal of Clinical Oncology: Official Journal of the American Society of Clinical Oncology | 2012-02-10 Feb 10, 2012 | PURPOSE: Women with BRCA1 or BRCA2 (BRCA1/2) mutations must choose between prophylactic surgeries and screening to manage their high risks of breast and ovarian cancer, comparing options in terms of cancer incidence, survival, and quality of life. A clinical decision tool could guide these complex choices. METHODS: We built a Monte Carlo model for BRCA1/2 mutation carriers, simulating breast screening with annual mammography plus magnetic resonance imaging (MRI) from ages 25 to 69 years and prophylactic mastectomy (PM) and/or prophylactic oophorectomy (PO) at various ages. Modeled outcomes were cancer incidence, tumor features that shape treatment recommendations, overall survival, and cause-specific mortality. We adapted the model into an online tool to support shared decision making. RESULTS: We compared strategies on cancer incidence and survival to age 70 years; for example, PO plus PM at age 25 years optimizes both outcomes (incidence, 4% to 11%; survival, 80% to 83%), whereas PO at age 40 years plus MRI screening offers less effective prevention, yet similar survival (incidence, 36% to 57%; survival, 74% to 80%). To characterize patients' treatment and survivorship experiences, we reported the tumor features and treatments associated with risk-reducing interventions; for example, in most BRCA2 mutation carriers (81%), MRI screening diagnoses stage I, hormone receptor-positive breast cancers, which may not require chemotherapy. CONCLUSION: Cancer risk-reducing options for BRCA1/2 mutation carriers vary in their impact on cancer incidence, recommended treatments, quality of life, and survival. To guide decisions informed by multiple health outcomes, we provide an online tool for joint use by patients with their physicians (http://brcatool.stanford.edu). | Decision Support Techniques | Humans | Female | quality of life | OK |
| Enrique | Gavilán-Moral | Cesar | Hueso-Montoro | Dolores | Cano-Caballero Gálvez | Atencion Primaria / Sociedad Española De Medicina De Familia Y Comunitaria | 2012-07-00 Jul 2012 | OBJECTIVE: To determine what family doctors think about various aspects of patient involvement in clinical decision making in Primary Care. DESIGN: Qualitative study using focus groups. LOCATION: Primary Care. PARTICIPANTS: Family physicians with and without expertise in clinical communication. METHODS: Three focus groups were developed, involving 6-8 professionals per group, and took part in two meetings. The conversations were recorded and transcribed verbatim. The discussion was analysed using literature-based categories and other emerging from the text, encoding the information and making an inductive interpretation. RESULTS: Family physicians refer mainly to involving the patient in decisions by proposing a plan tailored to the knowledge of patient problems and then verifying their approval or rejection. However, some professionals ponder whether this could be classified as patient involvement, questioning the real role that both players would take at the time of deciding. CONCLUSIONS: The explanation of how family physicians would involve the patient in decisions clashes with the most widespread theories on the subject and, also opposes the view of patients who would like to be involved more actively. Taking into account discordant reflections on the relevance of considering this process as real patient involvement, it is necessary to describe a realistic theoretical model that allows further development of strategies to improve the attitude and training of professionals to patient involvement in clinical decisions. | Decision Making | Humans | Patient Participation | Questionnaires | OK |
| Rob R. | de Leeuw | Jozien M. | Bensing | Guy E. H. M. | Rutten | Patient Education and Counseling | 2011-07-00 Jul 2011 | OBJECTIVE: To assess opinions and their determinants of patients with type 2 diabetes about responsibility for managing their diabetes, setting treatment targets and willingness taking medication. METHODS: Questionnaire survey carried out in general practices and outpatient clinics across the Netherlands. OUTCOMES: opinions about responsibility, targets and medication. Multinomial logistic regression analysis. RESULTS: Data of 994 consecutive persons were analysed (mean age 65 years; 54% males). Of these 62% agreed to take responsibility for their diabetes. In the opinion of 89% the setting of targets should be by or in cooperation with their physician or nurse and 40% were willing to take tablets until all targets were attained. Patients who perceived dysfunction by barriers to activity did not agree to take responsibility (OR 3.68; 1.65-8.19). Patients with complications preferred to set targets in cooperation with their physician or nurse (OR 1.98; 1.03-3.80). Males were more willing to take tablets until all targets were attained (OR 1.62; 1.17-2.25). CONCLUSION: Not all patients want to take responsibility for their diabetes or taking all necessary tablets, especially those with barriers to activity or complications. PRACTICE IMPLICATIONS: Doctors and nurses should ask for patients' opinions about responsibility and treatment goals before starting education. | Male | Humans | Patient Participation | Questionnaires | OK |
| W. | Hameeteman | R. J. | de Ridder | A. A. | Masclee | Digestion | 2011-00-00 2011 | BACKGROUND: Participation in and tolerability of primary colonoscopy screening are presumed to be relatively low. The present study aimed to test its feasibility in a well-informed population of hospital staff using an intensive information campaign, and to identify factors associated with screening colonoscopy rated as uncomfortable. METHODS: Data were collected using standardized forms. RESULTS: Out of 1,090 invited employees (50-65 years), 447 (41.0%) participated. Bowel preparation and colonoscopy were rated as 'somewhat to very uncomfortable' by 79.5 and 21.9%, respectively. 96.3% of participants were willing to repeat colonoscopy in the future. Participants rating colonoscopy as uncomfortable were more likely unwilling to repeat the procedure (OR 8.026, CI 2.667-24.154). Multivariate analysis (age- and gender-adjusted) showed an association of colonoscopy rated as uncomfortable with: abdominal pain during colonoscopy (OR 3.185, CI 1.642-6.178), other pain (OR 2.428, CI 1.335-4.416), flatulence (OR 2.175, CI 1.219-3.881), embarrassment (OR 2.843, CI 1.350-5.989), abdominal pain after colonoscopy (OR 1.976, CI 1.041-3.751), and a prolonged procedure time (OR 1.000, CI 1.000-1.001). CONCLUSIONS: Acceptance of primary colonoscopy screening for colorectal neoplasia was high, although participants with symptoms during and after colonoscopy were more likely to rate colonoscopy as uncomfortable. This type of opportunistic screening procedure is suitable for the introduction of screening programs and may be useful in areas that have no access to population-based screening. | Time Factors | Male | Humans | Patient Participation | OK |
| Peter | De Paepe | Walter | Buylaert | Paul | Van Royen | BMC family practice | 2010-00-00 2010 | BACKGROUND: Overuse of emergency departments (ED) is of concern in Western society and it is often referred to as 'inappropriate' use. This phenomenon may compromise efficient use of health care personnel, infrastructure and financial resources of the ED. To redirect patients, an extensive knowledge of the experiences and attitudes of patients and their choice behaviour is necessary. The aim of this study is to quantify the patients and socio-economical determinants for choosing the general practitioner (GP) on call or the ED. METHODS: Data collection was conducted simultaneously in 4 large cities in Belgium. All patients who visited EDs or used the services of the GP on call during two weekends in January 2005 were enrolled in the study in a prospective manner. We used semi-structured questionnaires to interview patients from both services. RESULTS: 1611 patient contacts were suitable for further analysis. 640 patients visited the GP and 971 went to the ED. Determinants that associated with the choice of the ED are: being male, having visited the ED during the past 12 months at least once, speaking another language than Dutch or French, being of African (sub-Saharan as well as North African) nationality and no medical insurance. We also found that young men are more likely to seek help at the ED for minor trauma, compared to women. CONCLUSIONS: Patients tend to seek help at the service they are acquainted with. Two populations that distinctively seek help at the ED for minor medical problems are people of foreign origin and men suffering minor trauma. Aiming at a redirection of patients, special attention should go to these patients. Informing them about the health services' specific tasks and the needlessness of technical examinations for minor trauma, might be a useful intervention. | Male | Humans | Patient Participation | Questionnaires | OK |
| Joseph | Bauer | Katie | Dahlquist | Larissa | Tis | Journal of Cancer Education | 2012-03-00 mars 2012 | The American Cancer Society (ACS) and Coalition of Cancer Cooperative Groups (CCCG) provide a clinical trial (CT) information/matching/eligibility service (Clinical Trials Matching Service [CTMS]). Patients' demographic and clinical data, enrollment status, and self-reported barriers to CT participation were analyzed to assess enrollment rates and determinants of enrollment. During 3 years beginning October 1, 2007, the CTMS served 6,903 patients via the ACS call center. Among the 1,987 patients with follow-up information on enrollment, 219 (11.0%) enrolled in a CT; 48 of these 219 enrollees chose a CT they found via the CTMS. Patients were less likely to enroll if they had poor ECOG performance status (P = 0.032); were African American (P = 0.0003), were uninsured or had Medicaid coverage (P = 0.024), or had lower stage disease (P = 0.018). Enrollment varied by trial type/cancer site/system (P = .026). Several barriers significantly predicted nonenrollment. Broader availability of a CTMS might help improve patient participation in cancer clinical trials. (PsycINFO Database Record (c) 2014 APA, all rights reserved). (journal abstract) | self report | Medicaid | Health Care Services | cancer | OK |
| Graham A. | Tyson | Virginia | Mitsch | Margaret | Alston | Brain Injury | 2011-00-00 2011 | PRIMARY OBJECTIVE: To investigate the participation of adults living with traumatic brain injury in regional and rural NSW as measured by the Participation Objective Participation Subjective (POPS) measure. RESEARCH DESIGN: A quantitative survey of a single cohort as the first of a two phase mixed methods study. METHODS AND PROCEDURES: One hundred and thirty-one adults with a TBI were recruited through eight rural brain injury rehabilitation programmes in NSW. These adults completed a range of survey instruments, including the POPS. MAIN OUTCOMES AND RESULTS: The POPS measure supported findings from other research that identified varying impacts of TBI. However, in contrast to other studies, there was no correlation between participation scores and age, gender, time since injury and severity of injury. CONCLUSIONS: The findings from this study suggest that the issue of participation of adults with TBI living in non-metropolitan areas needs to be further researched. | Male | Humans | Patient Participation | Questionnaires | OK |
| Paul H. | Lysaker | Kelly D. | Buck | Lars | Hansson | BMC Psychiatry | 2014-02-03 février 3, 2014 | Background: Recruitment is one of the most serious challenges in performing randomized controlled trials. Often clinical trials with participants diagnosed with schizophrenia are terminated prematurely because of recruitment challenges resulting in a considerable waste of resources in the form of time, funding, and the participants’ efforts. Dropout rates in schizophrenia trials are also high. Recruitment challenges are often due to patients not wanting to participate in research but can also be due to clinicians’ concerns regarding individuals diagnosed with schizophrenia as participants in research. This paper reports how overcoming recruitment challenges not related to patients revealed high readiness to take part and low dropout rates in a one year long randomized controlled trial testing Guided Self-Determination (GSD) among outpatients with schizophrenia receiving treatment in Assertive Outreach Teams in the northern part of Denmark. Methods: GSD is a shared decision-making and mutual problem-solving method using reflection sheets, which was developed in diabetes care and adjusted for this study and utilized by patients with schizophrenia. Descriptive data on strategies to overcome recruitment challenges were derived from notes and observations made during the randomized controlled trial testing of GSD in six outpatient teams. Results: Three types of recruitment challenges not related to patients were identified and met during the trial: 1) organizational challenges, 2) challenges with finding eligible participants and 3) challenges with having professionals invite patients to participate. These challenges were overcome through: 1) extension of time, 2) expansion of the clinical recruitment area and 3) encouragement of professionals to invite patients to the study. Through overcoming these challenges, we identified a remarkably high patient-readiness to take part (101 of 120 asked accepted) and a low dropout rate (8%). Conclusion: Distinction between recruitment challenges was important in discovering the readiness among patients with schizophrenia to take part in and complete a trial with the GSD-intervention. (PsycINFO Database Record (c) 2014 APA, all rights reserved). (journal abstract) | Intervention | schizophrenia | recruitment barriers | low dropout rate | OK |
|  |  |  |  |  |  | Birth (Berkeley, Calif.) | 2012-09-00 Sep 2012 | BACKGROUND: Enabling women to make informed decisions is a crucial component of consumer-focused maternity care. Current evidence suggests that health care practitioners' communication of care options may not facilitate patient involvement in decision-making. The aim of this study was to investigate the effect of specific variations in health caregiver communication on women's preferences for induction of labor for prolonged pregnancy. METHODS: A convenience sample of 595 female participants read a hypothetical scenario in which an obstetrician discusses induction of labor with a pregnant woman. Information provided on induction and the degree of encouragement for the woman's involvement in decision-making was manipulated to create four experimental conditions. Participants indicated preference with respect to induction, their perceptions of the quality of information received, and other potential moderating factors. RESULTS: Participants who received information that was directive in favor of medical intervention were significantly more likely to prefer induction than those given nondirective information. No effect of level of involvement in decision-making was found. Participants' general trust in doctors moderated the relationship between health caregiver communication and preferences for induction, such that the influence of information provided on preferences for induction differed across levels of involvement in decision-making for women with a low trust in doctors, but not for those with high trust. Many women were not aware of the level of information required to make an informed decision. CONCLUSIONS: Our findings highlight the potential value of strategies such as patient decision aids and health care professional education to improve the quality of information available to women and their capacity for informed decision-making during pregnancy and birth. | Decision Making | Humans | Patient Participation | Questionnaires | OK |
| Tijn | Brinkkemper | Lia | van Zuylen | Wijnanda A. | van Burg-Verhage | Nederlands Tijdschrift Voor Geneeskunde | 2011-00-00 2011 | OBJECTIVE: To evaluate the practice of continuous palliative sedation after the introduction of a national guideline. DESIGN: Investigation by questionnaire. METHOD: In 2008, 1580 physicians were asked to fill out a questionnaire regarding the last patient for whom they had prescribed continuous sedation until death. RESULTS: The response was 38% (n = 606). Eighty-two percent of the respondents were aware of the national guideline. Dyspnoea, pain and physical exhaustion were most often mentioned as the decisive indications for continuous sedation. The decision to use sedation was discussed with all mentally competent patients; in 18% of these cases, the patients had only been informed of the decision. Life expectancy at the start of continuous sedation was estimated to be less than 2 weeks in 97% of the cases. In 14% of the cases, the physicians felt pressured to start the sedation, predominantly by patients and relatives. Physicians were present at the start of sedation in 81% of the cases. Midazolam was used to induce sedation in 92%, and 41% of the physicians estimated that continuous sedation had hastened death to some extent. Most physicians believed that their patients' symptoms had been adequately relieved by continuous sedation, that the relatives were satisfied and that the quality of dying had been good. CONCLUSION: Continuous sedation practice in the Netherlands largely reflects the recommendations of the national guideline. Issues needing further attention are the pressure felt by physicians to start continuous sedation, as well as the possible life-shortening effect of continuous sedation as perceived by some of the physicians. | Time Factors | Male | Decision Making | Humans | OK |
| A. N. | Kolomenskaia | A. D. | Siniakina |  |  | Gigiena I Sanitariia | 2012-02-00 2012 Jan-Feb | The fact that parents are found to be inadequately aware of the prevention and correction of disorders and disorders of the musculoskeletal system (MSS) negatively affects its state in their children. Work increasing the parents'awareness has become an integral part of the first developed medicoeducational module which is the basis of a conceptual model to prevent and correct MSS disorders and diseases in schoolchildren. The introduction of the medicoeducational module into the work of comprehensive educational schools with the active participation of parents could reduce the prevalence of functional impairments and early diseases of MSS in schoolchildren by 50% during 3 years. | Male | Humans | Patient Participation | Questionnaires | OK |
| J. W. | Gorter | M. | Ketelaar | M. J. | Jongmans | Disability and Rehabilitation | 2011-00-00 2011 | PURPOSE: This study provides information on the psychometric properties of a newly developed Dutch-language instrument for measuring parental perceptions of participation of preschool children (aged 2+ to 5+ years): the PART. METHOD: The PART was administered to parents of preschool children with (n = 51) and without (n = 285) physical developmental disabilities. In the group with disabilities, children with no or relatively minor mobility limitations (capability scores in mobility domain higher than one standard deviation below population mean) were excluded from all analyses. A subgroup of parents of children without disabilities (n = 56) filled out the PART a second time, after a five-week interval. Hypotheses regarding group differences in PART scores and correlations of PART scores with different, but related ICF-constructs were tested. Test-retest reliability of the PART scales was examined. RESULTS: Expectations about group differences were confirmed. PART scores were associated in predicted ways with related constructs. PART scales showed good test-retest reliability (ICCs from 0.76 to 0.87). CONCLUSIONS: These findings indicate that the Dutch-language PART is a reliable and valid instrument for measuring parental perceptions of participation of preschool children with and without mobility limitations. The PART can prove useful for both clinical and research purposes. | Male | Humans | Patient Participation | Psychometrics | OK |
| Kumanan | Wilson |  |  |  |  | PloS One | 2011-00-00 2011 | BACKGROUND: Parents who choose to selectively vaccinate or avoid vaccination for their children may do so at risk of compromising relations with their family physician or pediatrician. Groups that are associated with reduced rates of pedicatic vaccination, such as parents who access naturopathic care, may be particularly vulnerable to this issue. METHODOLOGY/PRINCIPAL FINDINGS: In March through September 2010, we administered a 26-item cross-sectional survey to 129 adult patients, all of whom were parents with children ≤ 16 years of age, presenting for naturopathic care in Ontario, Canada. Ninety-five parents completed the survey (response rate 74%), and only 50.5% (48 of 95) reported that their children had received all recommended vaccines. Most parents (50.5%; 48 of 95) reported feeling pressure to vaccinate from their allopathic physician and, of those who discussed vaccination with their physician, 25.9% (21 of 81) were less comfortable continuing care as a result. Five percent (4 of 81) of respondents were advised by their physician that their children would be refused care if they decided against vaccination. In our adjusted generalized linear model, feeling pressure to vaccinate (odds ratio [OR] = 3.07; 95% confidence interval [CI] = 1.14 to 8.26) or endorsing a naturopathic physician as their most trusted source of information regarding vaccination (OR = 3.57; 95% CI = 1.22 to 10.44) were associated with greater odds of having a partially vaccinated or unvaccinated child. The majority (69.6%; 32 of 46) of parent's with partially vaccinated or unvaccinated children reported a willingness to re-consider this decision. CONCLUSIONS/SIGNIFICANCE: Use of naturopathic care should be explored among parents in order to identify this high-risk group and engage them in discussion regarding pediatric vaccination to encourage evidence-based, shared decision making. Physicians should ensure that discussions regarding vaccination are respectful, even if parents are determined not to vaccinate their children. | Humans | Questionnaires | Adolescent | Adult | OK |
| Julia | Addington-Hall | |  |  |  | BMC medical research methodology | 2013-00-00 2013 | BACKGROUND: Although in health services survey research we strive for a high response rate, this must be balanced against the need to recruit participants ethically and considerately, particularly in surveys with a sensitive nature. In survey research there are no established recommendations to guide recruitment approach and an 'opt-in' system that requires potential participants to request a copy of the questionnaire by returning a reply slip is frequently adopted. However, in observational research the risk to participants is lower than in clinical research and so some surveys have used an 'opt-out' system. The effect of this approach on response and distress is unknown. We sought to investigate this in a survey of end of life care completed by bereaved relatives. METHODS: Out of a sample of 1422 bereaved relatives we assigned potential participants to one of two study groups: an 'opt in' group (n=711) where a letter of invitation was issued with a reply slip to request a copy of the questionnaire; or an 'opt out' group (n=711) where the survey questionnaire was provided alongside the invitation letter. We assessed response and distress between groups. RESULTS: From a sample of 1422, 473 participants returned questionnaires. Response was higher in the 'opt out' group than in the 'opt in' group (40% compared to 26.4%: χ(2) =29.79, p-value<.01), there were no differences in distress or complaints about the survey between groups, and assignment to the 'opt out' group was an independent predictor of response (OR=1.84, 95% CI: 1.45-2.34). Moreover, the 'opt in' group were more likely to decline to participate (χ(2)=28.60, p-value<.01) and there was a difference in the pattern of questionnaire responses between study groups. CONCLUSION: Given that the 'opt out' method of recruitment is associated with a higher response than the 'opt in' method, seems to have no impact on complaints or distress about the survey, and there are differences in the patterns of responses between groups, the 'opt out' method could be recommended as the most efficient way to recruit into surveys, even in those with a sensitive nature. | Male | Humans | Patient Participation | Questionnaires | OK |
| Bonnie | Spring | Sattar | Hadi | Frances M. | Weaver | Preventing Chronic Disease | 2012-00-00 2012 | INTRODUCTION: Participant retention is a frequent concern in structured weight-management programs. Although research has explored participant characteristics influencing retention, little attention has been given to the influence of program characteristics. The objective of this study was to examine how program characteristics relate to participant retention in the Veterans Health Administration's weight-management program, MOVE! METHODS: We conducted semistructured interviews with coordinators of 12 MOVE! programs located throughout the United States, 5 with high participant retention rates and 7 with low rates. We transcribed and descriptively coded interviews and compared responses from high- and low-retention programs. RESULTS: Characteristics related to retention were provider knowledge of and referral to the program, reputation of the program within the medical facility, the MOVE! meeting schedule, inclusion of physical activity in group meetings, and involvement of the MOVE! physician champion. MOVE! introductory sessions, frequency of group meetings, and meeting topics were not related to retention. Coordinators described efforts to improve retention, including participant contracts and team competitions. Coordinators at 5 high-retention facilities and 1 low-retention facility discussed efforts to improve retention. CONCLUSION: Coordinators identified important program characteristics that could guide improvements to retention in group-based weight-management programs. Training for providers is needed to assist with referral decisions, and program planners should consider incorporating physical activity in group meetings. | Humans | Patient Participation | Questionnaires | United States | OK |
| C. | Becker | M. | Zimmermann |  |  | Die Rehabilitation | 2011-12-00 Dec 2011 | Because of the diversity of institutions funding rehabilitative measures and the lack of a shared data base, little is known so far about how many people in all and what kind of people make use of medical rehabilitation. Starting out from this dilemma, the rehabilitant population of 2004 was described in greater detail with the help of the population-representative and not institution-oriented German Socio-Economic Panel (SOEP) and by means of health variables (subjective health, severe disability, periods of disability, health care utilisation) as well as of socio-demographic characteristics (sex, age, social status, region). In addition, the influence of these variables on the chances of taking part in rehabilitation was evaluated. In 2004, 4% of the respondents took part in rehabilitative measures, with women having a lower chance of participating as men have. A bivariate distinct age gradient leveled under multifactorial analysis. The strongest predictors, in contrast, were found to be subjective health, amount of visits to doctors' offices as well as hospitalizations. Contrary to our expectations, an influence of the social stratum could not be found. The analysis in addition shows a higher utilization of rehabilitative procedures in eastern Germany. | Humans | Patient Participation | Female | Aged | OK |
|  |  |  |  |  |  | Disability and Rehabilitation | 2012-00-00 2012 | PURPOSE: The aim was to examine stroke patients', carers' and volunteer supporters' experiences of peer support groups during hospital rehabilitation. METHODS: Semi-structured interviews and questionnaires were analysed by inductive thematic analysis. Participants also answered a standardised Therapeutic Factors Inventory (TFI). RESULTS: Five superordinate themes emerged for patients, carers and volunteer supporters. Three themes related to group processes; 'practical issues' (five subthemes), 'staff presence', 'similarity-difference', and comparison with other group members. 'Value of peers' (five subthemes) described beneficial outcomes. The 'similarity-difference' theme and four subthemes under 'value of peers' were related to items from the TFI which drew agreement from most participants. The supporters had some unique themes; two were concerned with group organisation, one was the experience of 'being helpful to others' and one described the experiences of training. As well as its links with themes, agreement with TFI items revealed experiences that did not emerge as themes; feeling secure, expressing emotions and increased independence. CONCLUSIONS: Participation in the group was experienced as beneficial by participants. Benefits included helpful information, advice, making new connections and increased awareness of stroke. Participants identified important group processes such as upward and downward comparison. Responses to the TFI suggested that attendance brought therapeutic gains. | Male | Humans | Patient Participation | Questionnaires | OK |
| Ilse | Van Vlaenderen | Omer | Saka | Paul | Dendale | European Journal of Preventive Cardiology | 2013-04-00 Apr 2013 | BACKGROUND: International research indicates that attendance of patients to a proposed cardiac rehabilitation (CR) programme varies between 21% and 75%. Addressing the reasons why cardiac patients are not participating will improve accessibility to CR. The objective of this study was to investigate patient compliance with cardiac rehabilitation and the reasons of refusing or abandoning the programme. METHODS: Twenty hospital centres were recruited to participate. Each centre was asked to recruit patients from three patient groups, namely: percutaneous coronary intervention patients, patients that underwent major cardiac surgery, and patients being admitted because of an acute myocardial infarction and not belonging to the other two groups. Patients were asked to fill out a questionnaire during a follow-up outpatient consultation after the cardiac intervention. RESULTS: In total, 226 patients participated in the survey. Most patients were proposed (86%) and accepted (81% out of proposed) to attend a CR programme. Of those who accepted, 77% completed the programme. The main reasons that led to patients' refusal to participate in a CR programme were distance to the CR centre, patients' belief they could handle their own problems, and lack of time. The main three reasons for not completing an initiated CR programme were other physical problems, patients' belief they could handle their own problems, and the cost of rehabilitation. CONCLUSION: Our findings demonstrate the importance of raising patients' awareness of the benefits of CR. Addressing potential barriers to attend a CR programme should be investigated with patients individually in order to ensure compliance. | Time Factors | Male | Humans | Patient Participation | OK |
| Norhayati Mohd | Noor | Amry Abdul | Rahim |  |  | Asian Pacific journal of cancer prevention: APJCP | 2012-00-00 2012 | In Malaysia, colorectal cancer is the most common cancer in males and the third most common in females. Mortality due to colorectal cancer can be effectively reduced with early diagnosis. This study was designed to look into colorectal cancer screening participation and its barriers among average risk individuals in Malaysia. A cross sectional study was conducted from August 2009 till April 2010 involving average risk individuals from 44 primary care clinics in West Malaysia. Each individual was asked whether they have performed any of the colorectal cancer screening methods in the past five years. The barrier questions had three domains: patient factors, test factors and health care provider factors. Descriptive analysis was achieved using Statistical Program for Social Sciences (SPSS) version 12.0. A total of 1,905 average risk individuals responded making a response rate of 93.8%. Only 13 (0.7%) respondents had undergone any of the colorectal cancer screening methods in the past five years. The main patient and test factors for not participating were embarrassment (35.2%) and feeling uncomfortable (30.0%), respectively. There were 11.2% of respondents who never received any advice to do screening. The main reason for them to undergo screening was being advised by health care providers (84.6%). The study showed that participation in colorectal cancer screening in Malaysia is extremely low and multiple factors contribute to this situation. Given the importance of the disease, efforts should be made to increase colorectal cancer screening activities in Malaysia. | Male | Humans | Patient Participation | Questionnaires | OK |
| Suji | Shin | Jun Chul | Park | Sung Kwan | Shin | Journal of Surgical Oncology | 2012-07-01 Jul 1, 2012 | BACKGROUND: This study was to evaluate the participation role and conflict of patients during the decision-making process for endoscopic or surgical treatment for early gastric cancer (EGC). METHODS: In this prospective observational study, the sequential survey for patients under consideration for treatment of differentiated EGC was performed at the tertiary referral center. RESULTS: Among the 82 responders, 63.4% preferred endoscopic resection. The total decisional conflict scores were high and significantly different between groups that preferred endoscopic resection or surgical treatment (44.8 vs. 51.6, P = 0.016). Values assigned to the two treatment attributes "preservation of stomach (OR = 0.51)" and "bother in case of incomplete resection (OR = 2.13)" clearly discriminated between patients reaching a final decision of surgical gastrectomy or endoscopic resection. Regarding the participation role, a shared role was more frequent in the group with preference for endoscopic treatment before consultation (42.3% vs. 24.0%, P = 0.045). However, at the time of therapeutic decision, the passive role was most remarkable, both for the endoscopic (53.2%) and surgical (71.4%) groups. CONCLUSION: Despite the high overall decisional conflict, patients with preference for endoscopic treatment tended to be more autonomous. After consultation, all patients exhibited a passive participation role in the decision-making process. | Male | Decision Making | Humans | Patient Participation | OK |
|  |  |  |  |  |  | Fortschritte Der Neurologie-Psychiatrie | 2013-10-00 Oct 2013 | | Humans | Patient Participation | Stroke | Nervous System Diseases | Doublon |
|  |  |  |  |  |  | Fortschritte Der Neurologie-Psychiatrie | 2013-10-00 Oct 2013 | | Humans | Patient Participation | Stroke | Nervous System Diseases | OK |
|  |  |  |  |  |  | Bundesgesundheitsblatt, Gesundheitsforschung, Gesundheitsschutz | 2013-05-00 May 2013 | A growing number of people in Germany participate in the cancer screening services offered by statutory health insurance. Using data from the first wave of the German Health Interview and Examination Survey for Adults (DEGS1), current levels of participation in cancer screening services were determined. DEGS1 (2008-2011) permits representative cross-sectional analyses to be performed. In DEGS1, persons who were entitled to different cancer screening services were interviewed on their awareness, participation and regular utilisation of cancer screening for different types of cancer. Overall, 67.2% of women and 40.0% of men participate regularly. Participation rates fluctuate to a great extent for individual types of cancer screening. Women participate in cancer screening more frequently than men do. For women, a better socioeconomic status was associated with higher participation rates. Participation rates improve with increasing age, meaning that the difference in participation rates between women and men becomes smaller. The current analyses present information on specifically targeted population groups to promote informed decision-making about cancer screening, so that participation rates can be improved further. The analyses thus provide an important basis for health policy measures. An English full-text version of this article is available at SpringerLink as supplemental. | Male | Humans | Patient Participation | Young Adult | OK |
| Punita | Lal |  |  |  |  | Journal of Cancer Research and Therapeutics | 2012-06-00 2012 Apr-Jun | INTRODUCTION: Breast conserving surgery followed by radiotherapy is an established modality of treatment in early breast cancer patients since three decades, but yet it has not been adopted worldwide. PURPOSE: The aim of this study is to investigate the factors affecting decision making regarding type of surgery and satisfaction with type of surgery in North Indian women with early breast cancer. MATERIALS AND METHODS: A questionnaire was prepared to assess the factors responsible for decision making regarding type of surgery (breast conserving surgery (BCS) versus modified radical mastectomy (MRM) and to evaluate involvement of patient in decision making regarding the type of surgery. 47 women with early breast cancer on radiotherapy or on follow-up were interviewed by the resident doctors. RESULTS: Out of 47 patients, 28 underwent BCS and 19 MRM. Women undergoing BCS were younger, more literate than in those undergoing MRM. In the two arms (BCS versus MRM), decision for surgery was made by surgeon alone in 53% versus 73%, along with patient in 42% versus 6%, and only 10% women participated in decision making in each arm. Only 50% versus 30% patients had a clear understanding of the risks and benefits of both procedures in the two arms. CONCLUSION: North Indian women do not independently take decision regarding any type of surgery. The reason for opting for either kind of surgery was based on surgeon's recommendation or concern about recurrence. Body image was not an issue amongst majority. | Decision Making | Humans | Patient Participation | Patient Satisfaction | OK |
|  |  |  |  |  |  | Bundesgesundheitsblatt, Gesundheitsforschung, Gesundheitsschutz | 2013-05-00 May 2013 | Health behaviour change programmes to promote healthy behaviours are aimed at, among other things, counteracting the emergence of widespread non-communicable diseases. Which population groups use these programmes? This analysis is based on data from DEGS1, which was conducted from 2008-2011. People aged 18-79 years were asked about their participation in programmes in the last 12 months in the fields of nutrition, physical activity and relaxation (n = 7,807). The analysis was stratified by sex, age, socioeconomic status (SES), and type of statutory health insurance fund. A total of 16.6 % of respondents participate in at least one programme for behaviour change, with women using these programmes significantly more frequently, indeed twice as often, as men (22.1 % versus 11 %). The older population participates more often than younger age groups. Women and men with low SES use the programmes significantly less frequently than those with middle or high SES. Women who are insured by the AOK health insurance group have a significantly lower rate of participation than women insured by any other statutory health insurance fund. Overall participation has almost doubled since the "German National Health Interview and Examination Survey 1998" (9.1 %). Further efforts are necessary to reach population groups with low participation rates. An English full-text version of this article is available at SpringerLink as supplemental. | Male | Humans | Patient Participation | Young Adult | OK |
| Emanuel | Tirosh |  |  |  |  | Research in Developmental Disabilities | 2010-12-00 2010 Nov-Dec | ADHD is a neural developmental disorder expressed in various life settings. Yet, previous studies have focused mainly on children's function in school and academic achievement. The purpose of the present study was, therefore, to examine participation patterns in outside formal school activities among boys with ADHD compared to typical boys. Participants included 25 boys aged 8-11 years with ADHD and 25 age-matched typical boys. All participants completed the Children's Assessment of Participation and Enjoyment (CAPE). Several aspects of participation were examined: diversity, intensity, enjoyment, place, and partners in 49 extra curricular activities. The findings indicate that boys with ADHD reported significant lower intensity rates of participation in most activity domains. Furthermore, boys with ADHD also reported higher diversity scores and lower enjoyment in 'formal' activities. Yet, no significant differences were found with regard to activity place and partners. These findings enhance the importance of providing therapy that refers to after school activities. Accordingly, CAPE can be useful for assessing boys with ADHD and planning appropriate intervention programs. | Male | Humans | Patient Participation | Questionnaires | OK |
| Louise | Gustafsson | Janelle | Griffin | Ann Maree | Collier | Brain Injury | 2011-00-00 2011 | PRIMARY OBJECTIVE: To describe and compare pre- and post-injury leisure activities of individuals receiving brain injury rehabilitation and explore levels of leisure participation and satisfaction. RESEARCH DESIGN: Cross-sectional descriptive study incorporating a survey of current and past leisure activities. METHODS AND PROCEDURES: Questionnaires were completed by 40 individuals with an acquired brain injury receiving inpatient or outpatient rehabilitation. MEASURES: Shortened Version of the Nottingham Leisure Questionnaire and Changes in Leisure Questionnaire (developed for this study). RESULTS: Leisure participation declined following injury, particularly in social leisure activities. Pre-injury activities with high rates of discontinued or decreased participation were driving, going to pubs and parties, do-it-yourself activities and attending sports events. Inpatient participants generally attributed decreased participation to the hospital environment, whereas outpatient participants reported this predominantly as a result of disability. Post-injury levels of perceived leisure satisfaction were significantly lower for the inpatient group compared to pre-injury, but not for the outpatient group. Uptake of some new leisure activities was reported post-injury, however not at the rate to which participation declined. CONCLUSIONS: Leisure participation decreases during brain injury rehabilitation compared to pre-injury levels. Re-engagement in relevant, age-appropriate leisure activities needs to be addressed during rehabilitation to improve participation in this domain. | Male | Humans | Patient Participation | Patient Satisfaction | OK |
| S. | Rosenblum |  |  |  |  | Research in Developmental Disabilities | 2014-01-00 Jan 2014 | The aim of the current study was to compare varied dimensions of participation in leisure activities among school-aged children ages 10-16 with and without disabilities. The Children Leisure Activity Scale (CLASS) was administrated to 294 children, 81 with and 213 without physical disability. Two-way MANCOVA revealed significant differences between the frequency of participation in leisure activities of the study groups: an effect of disability F(4,265=239.57; p<0.001, η(2)=0.78); an effect of gender F(4,265=3.35; p<0.01, η(2)=0.05); and an interaction effect between gender and disability F(4,265=5.23; p<0.001, η(2)=0.64). Children with disabilities, and mostly girls, were found at risk to participate in a narrower variety of activities that involved fewer social interactions. Using linear regressions for each group two different models were identified. Using the CLASS the study contributes evidence-based data regarding children at risk for leisure participation. In addition, the research further established the discriminate validity of the CLASS. | Male | Humans | Patient Participation | Questionnaires | OK |
| Ashley L. | Doherty | Eric | Garshick | Ross | Zafonte | American Journal of Physical Medicine & Rehabilitation / Association of Academic Physiatrists | 2013-05-00 May 2013 | OBJECTIVE: The aim of this study was to determine the association between participation in organized sports programs and employment in adults with chronic spinal cord injury. DESIGN: This is a cross-sectional study of 149 adults with chronic spinal cord injury. Motor level and completeness of injury were confirmed by physical examination. Information related to demographics, employment, level of education, body mass index, duration of injury, participation in individually planned exercise, and participation in organized sports was obtained using a standardized questionnaire. Multivariable logistic regression analyses were used to assess factors associated with employment. RESULTS: In univariate analyses, employment was associated with younger age (P = 0.001) and a higher level of education (P = 0.01), whereas obesity decreased the likelihood of employment (P = 0.04). Participation in organized sports approached significance (P = 0.06). In the multivariable analysis and after adjusting for age, education, and body mass index, participation in organized sports was significantly associated with employment (odds ratio, 2.4; P = 0.04). Sex, duration of injury, wheelchair use, and participation in individually planned exercise were not significantly associated with employment (P = 0.16-0.94). CONCLUSIONS: In the adults with chronic spinal cord injury, participation in organized sports was positively associated with employment. Further studies are necessary to determine the causative nature of this association and how various factors related to sports participation may contribute. | Male | Humans | Patient Participation | Questionnaires | OK |
| Eline | Lindeman | L. | Jaap Kappelle | Marcel W. M. | Post | Topics in Stroke Rehabilitation | 2013-02-00 2013 Jan-Feb | BACKGROUND: Participation is a multidimensional concept, consisting of an objective and a subjective dimension. Many studies have focused on determinants of only 1 dimension of participation post stroke. OBJECTIVE: To describe participation (both objective and subjective) and to determine how physical and cognitive independence and subjective complaints (pain, fatigue, and mood) influence participation in community-dwelling stroke survivors in the Netherlands. METHODS: The Utrecht Scale for Evaluation of Rehabilitation (USER) measures physical and cognitive independence and subjective complaints. USER-Participation measures 3 dimensions of participation: frequency (objective perspective), restrictions (subjective perspective), and satisfaction (subjective perspective). Spearman correlations and backward linear regression analyses were used to analyze associations between the 3 USER-Participation scores with demographics, stroke characteristics, physical and cognitive independence, and subjective complaints. RESULTS: Of the 111 participants, 48.5% returned to work post stroke, but mostly for only 1 to 16 hours a week. Experienced participation restrictions were most prevalent in physical exercise, chores in/around the house, housekeeping, and outdoor activities. On average, participants were relatively satisfied with their participation, but dissatisfaction occurred in cognition, activities outdoors, and work/housekeeping. Regression analysis revealed that objective participation was determined by physical and cognitive independence, age, and education, whereas subjective participation was determined by physical and cognitive independence, fatigue, and mood. CONCLUSIONS: Most participants experienced participation problems, despite relatively good physical recovery. In addition to physical and cognitive factors, subjective complaints of persons with stroke should be addressed in the rehabilitation program. | Male | Humans | Patient Participation | Female | OK |
| Luciano | Santana-Cabrera | Manuel | Sánchez-Palacios | |  | Medicina Clínica | 2010-03-20 Mar 20, 2010 | | Male | Decision Making | Humans | Patient Participation | OK |
| Christine | Imms | Monique | Black | Clare | Ardern | Research in Developmental Disabilities | 2013-05-00 May 2013 | We compared participation in out-of-school activities between children with intellectual disability and children with typical development using the Children's Assessment of Participation and Enjoyment and Preferences for Activities of Children questionnaires. Thirty-eight pairs of children were matched for age (mean age 12.3 ± 2.7 years), sex (17 female, 21 male), location (32 metropolitan, 6 regional) and socioeconomic background (mean SEIFA score 1021 ± 70 and 1024 ± 66). When compared to their typically developing peers, children with intellectual disability participated in fewer Active-Physical and Skill-Based activities and in more Recreational activities. Children with intellectual disability participated less frequently in Skilled-Based activities, had a higher preference for Recreational and Self-Improvement activities, enjoyed Self-Improvement activities more, and participated in a higher proportion of Social activities at home and in a lower proportion of Recreational, Active-Physical, Skill-Based, and Self-Improvement activities alone. These differences may be due to reduced physical, cognitive and social skills in children with intellectual disability, or a lack of supportive environments. | Male | Humans | Patient Participation | Questionnaires | OK |
| Sally | Tamsett | Mark | McMahon |  |  | Australian Occupational Therapy Journal | 2012-06-00 Jun 2012 | INTRODUCTION: A call for active inclusion of consumers in research; recognising and valuing the knowledge that arises through lived experience, is made in the literature. However, genuine inclusion of consumers as members of the research team remains rare. Opponents present two barriers: lack of required knowledge and skills and lack of capacity to conduct rigorous research. METHODS: This study involved a collaborative process to develop training materials for consumer researchers. Reliability of self-report outcome data collected by five trained consumers was then examined. RESULTS: Quantitative results suggest that trained consumers can facilitate self-report interviews to the high standard expected by the general research community. CONCLUSIONS: This study provides both methods and resources to extend occupational therapy consumer-centred practice into the realm of research. | Male | Humans | Patient Participation | Young Adult | OK |
| S. | Tabolli |  |  |  |  | Clinical and Experimental Dermatology | 2011-12-00 Dec 2011 | There have been few studies about the participation in health care and the satisfaction of patients with psoriasis and with psoriatic arthritis. The objective of this study was to examine the attitudes of and satisfaction with decision-making of patients with psoriasis. The study was based on a self-completed questionnaire, given after a routine medical visit to a consecutive sample of 240 patients with psoriasis [207 with cutaneous psoriasis (CP) and 33 with psoriatic arthritis (PA)]. Of the patients with CP and PA, only about a third (27.3% and 28.1%, respectively) preferred to leave decisions entirely to their doctor, whereas the majority (72.7% and 71.9%, respectively) wanted to be involved in decision-making. However, the level of knowledge about the disease was not as high, with 17.0% and 21.4% of patient with CP and PA, respectively, having a good level of knowledge about psoriasis treatment. Participation, knowledge and overall satisfaction were not significantly different between patients with CP and those with PA. Information on treatment side-effects (OR = 5.11; 95% CI 2.5-15.0; P < 0.001) and information on treatment options (OR = 3.15; 95% CI 1.4-7.1; P = 0.006) were significantly associated with overall patient satisfaction. | Male | Decision Making | Humans | Patient Participation | OK |
| Desleigh | de Jonge |  |  |  |  | Disability and Rehabilitation | 2012-00-00 2012 | PURPOSE: The purpose of this study was to describe the preliminary development and validation of a potential measure for assessing the accessibility of the built environment in Zambia. It was designed to identify environmental features that present barriers to participation for people with mobility limitations (PWML) using mobility devices such as wheelchairs or crutches. METHOD: The Participation-Based Environment Accessibility Assessment Tool (P-BEAAT) was developed through focus group discussions and personal interviews with 88 PWML from five provinces of Zambia regarding the accessibility of their built environment. The content validity of the P-BEAAT checklist was accomplished through three phases of development with data gathered from 11 focus groups and nine personal interviews. RESULTS: Participants described accessibility barriers which affect their participation in daily life. This information generated the P-BEAAT with 66 items describing eight environmental features with potential for identifying environmental barriers. The P-BEAAT has shown good homogeneity with Cronbach's α score of 0.91. CONCLUSION: The P-BEAAT was constructed grounded in the reality of people's experiences in Zambia for use in assessing environmental features important in the participation of daily life of PWML pertinent to developing countries. Further clinimetric testing of the properties of the P-BEAAT to establish reliability should be conducted next. | Male | Humans | Patient Participation | Questionnaires | OK |
| T. | Froböse | A. | Gsottschneider | Z. | Keller | Psychotherapeut | 2012-07-00 juillet 2012 | Background: The effectiveness of psychoeducation groups for people with schizophrenia is well documented; however, there are no studies which examined patient participation behavior although this behavior might be critical for accomplishment of the therapeutic goals. This article presents newly developed 4-point Likert scale, the participation quality rating scale (PQRS), together with initial results on practicability, validity, change sensitivity, sociodemographic and clinical correlates and on the question whether participation quality might be a significant predictor of the short and long-term outcome of psychoeducation. Patient and methods: Within the framework of the Munich Study “Cognitive Determinants of Psychoeducation and Information in Schizophrenic Psychoses” (COGPIP) the individual participation behavior of 97 patients with schizophrenia was examined after each psychoeducational group session (4 weeks). Individual mean PQRS scores were correlated with sociodemographic, anamnestic and clinical variables. In addition to change sensitivity the ability of the scale to predict the acquisition of illness knowledge during psychoeducation was examined as well as readmission during a 9-month follow-up period and the psychopathological endpoint (positive and negative syndrome scale, PANSS). Results: Practicability and change sensitivity of the PQRS were found to be sufficient. Significant correlations with independently rated illness insight and medication compliance indicated a construct validity of the scale. Higher age, female sex and less pronounced psychopathological symptoms (PANSS) were associated with better participation behavior. Even patients with clearly impaired participation behavior profited from psychoeducation in that they were able to catch up with the delay with regard to illness knowledge. However, the PQRS was not a predictor of readmission during the 9-month follow-up period or of psychopathological endpoints. Conclusions: The PQRS is qualified as a new tool for the standardized assessment of participation behavior in people with schizophrenia attending psychoeducation groups. The scale can be applied in research as well as clinical contexts in order to further improve understanding of how psychoeducation works and how this kind of supplementary therapy could become even more effective. (PsycINFO Database Record (c) 2012 APA, all rights reserved). (journal abstract) | Client Participation | Test Construction | schizophrenia | treatment outcomes | OK |
|  |  |  |  |  |  | BJU international | 2011-12-00 Dec 2011 | What's known on the subject? and What does the study add? Active surveillance is a management strategy that offers patients the hope of avoiding the side effects associated with unnecessary treatment. This study identifies the resources required by men who choose to be on active surveillance to support them in their treatment decision. OBJECTIVES: •  To examine the decision-making processes of men on active surveillance (AS). •  To identify the resources that men want to access to make, support and sustain them while on AS. PATIENTS AND METHODS: •  Three-part survey developed for this study based on a qualitative study of 25 men on AS. •  Survey items explored: role men assumed with their physician in treatment decision-making, factors influencing decision to go on AS, and resources required while on AS. •  Surveys mailed out to cross-sectional sample of men on AS for less than 10 years. RESULTS: •  27% of the 73 men reported assuming an active role in treatment decision-making with their urologist, 41% a shared role and 32% a passive role. •  82% of men reported being comfortable and 90% being satisfied with their decision to be on AS. •  55% reported not being anxious about the cancer progressing while on AS. •  Urologist's opinion, current age, and impact of treatment on urinary function were main factors influencing treatment decision. •  Information on future treatment options, non-traditional treatments and diet considered most important resources. CONCLUSIONS: •  Results suggest that once men make a decision to go on AS, they are satisfied with their decision and few report being anxious about progression of cancer. •  Men are strongly influenced by the treating specialist in taking up AS. •  Additional information and psychological support resources are required for men on AS. | Male | Decision Making | Humans | Patient Participation | OK |
| Anne | Brabers | Judith | de Jong | Jany | Rademakers | Journal of Health Communication | 2014-00-00 2014 | In demand-led health care systems, consumers are expected to play an informed, active role in health care decisions by making use of health information. The ability to seek and use this information depends on specific knowledge, skills, and self-confidence. In this study, the authors validated a translated instrument to measure patient activation (Dutch PAM-13) in a general sample of Dutch health care consumers. Furthermore, the authors examined the relative contribution of patient activation and functional health literacy to the seeking and use of health information in The Netherlands. The mean patient activation score in the Dutch sample was higher for younger health care consumers and for those with a higher education, higher income, and better self-reported general and mental health status. More activated consumers were more likely to seek and use health information. Patient activation proved to be a stronger predictor for seeking and using health information than functional health literacy. | Male | Humans | Patient Participation | Questionnaires | OK |
| Charlyn | Black |  |  |  |  | Medical Care | 2011-05-00 May 2011 | BACKGROUND AND OBJECTIVE: Current policy directions place increasing expectations on patients to actively engage in their care, especially in chronic disease management. We examined relationships between patient activation and multiple dimensions of primary healthcare (PHC), including access, utilization, responsiveness, interpersonal communication, and satisfaction for patients with and without chronic illness. RESEARCH DESIGN: Cross-sectional, random digit dial survey conducted in British Columbia (BC), Canada. SUBJECTS: Stratified sample of adults (n=504), aged 19 to 90 years, who had visited their regular provider within the past 24 months. All data were weighted to represent residents living in BC. MEASURES: Patient activation and PHC experiences include accessibility, continuity, whole-person care, interpersonal communication, responsiveness, chronic disease management, and satisfaction. RESULTS: The multivariate models provide evidence that both quantity of time and quality of interactions with one's regular provider are associated with higher patient activation. Those with no chronic illness had higher activation scores when they spent more time talking with their regular provider, experienced less hurried communication, or if their test results were explained. The more time people with chronic illness are able to spend with their physician, the more activated they were. Chronic illness respondents also had higher activation scores if they reported higher whole-person care or if they were more satisfied. CONCLUSIONS: Positive interactions between the patient and the provider can influence the patient's abilities to engage in and be confident in maintaining/improving his/her health. Supporting patients in becoming actively engaged, in ways that work for them, is essential to providing high quality care, especially among those with a chronic condition. | Male | Humans | Patient Participation | Patient Satisfaction | OK |
| Dan | Moore | Shelley | Volz | Karen | Sepucha | Patient Education and Counseling | 2012-07-00 Jul 2012 | OBJECTIVE: Explore the validity of using patient reports to measure shared decision making (SDM). METHODS: 178 patients diagnosed with breast cancer obtained SDM assistance in a university-based clinic. Trained observers rated physician SDM behaviors and surveyed patients. We calculated the frequency with which patients and observers reported maximum SDM behaviors for each survey item. We also calculated agreement frequency between patients and observers. RESULTS: Over 90% of patients rated doctors as reflecting SDM competencies. Patients reported doctors making recommendations more than soliciting their preferred choice (90% vs. 69%, p<0.001). Patients heard benefits discussed "a lot" more often than they heard risks and side effects discussed "a lot" (81% vs. 58%, p<0.001). Agreement between patients and observers was 75%. In cases of disagreement, patients more frequently perceived SDM behaviors than did observers (15% vs. 9%, p=0.002), suggesting a possible agreement bias. CONCLUSIONS: High agreement supports further investigation into using patients as efficient and effective raters of SDM. Patient ratings may be inflated by agreement bias. PRACTICE IMPLICATIONS: Doctors presentations may be biased toward discussing benefits more than risks. Policy makers can solicit patient ratings of SDM as long as they are aware of possibly inflated ratings due to agreement bias. | Decision Making | Humans | Patient Participation | Physician-Patient Relations | OK |
| Craig | Wood | Walter F. | Stewart |  |  | The Diabetes Educator | 2012-06-00 2012 May-Jun | PURPOSE: The purpose of this study was to examine the influence of patient and physician psychosocial, sociodemographic, and disease-related factors on diabetes medication adherence. These factors were also examined as effect modifiers of the association between quality of the patient-physician relationship and medication adherence. METHODS: Data were collected from 41 Geisinger Clinic primary care physicians and 608 of their patients with type 2 diabetes. Adherence to oral hypoglycemic medications was calculated using a medication possession ratio based on physician orders in electronic health records (MPR(EHR)). MPR(EHR) was defined as the proportion of total time in the 2 years prior to study enrollment that the patient was in possession of oral hypoglycemic medications. Linear regression was used to examine the influence of patient- and physician-level factors on adherence. Effect modification of the patient-physician relationship-adherence association was evaluated by adding the main effects of the individual-level factors and their cross-products to the models. RESULTS: In adjusted analyses, satisfaction with the physician's patient education skills, patient beliefs about the need for their medications, and lower diabetes-related knowledge were associated with better adherence to oral hypoglycemic medications. Shorter duration of time with diabetes and taking only oral hypoglycemic medications were also associated with better adherence. Finally, the association between shared decision making and medication adherence was significantly modified by patients' level of social support. CONCLUSIONS: This study identified several patient-, physician-, and disease-related factors that should be targeted to maximize the potential for developing tailored adherence-enhancing interventions within the context of a collaborative patient-physician relationship. | Male | Decision Making | Humans | Physician-Patient Relations | OK |
| Jennifer M. | Creasman | Waldo | Sepulveda | Juan E. | Vargas | International Journal of Gynaecology and Obstetrics: The Official Organ of the International Federation of Gynaecology and Obstetrics | 2011-12-00 Dec 2011 | OBJECTIVE: To examine patient and provider attitudes toward first trimester nuchal translucency (NT) screening for Down syndrome and to assess how patients consent to screening in a country where abortion is illegal. METHODS: Patients presenting for first trimester ultrasound including NT screening in two obstetric units in Chile completed a questionnaire about their attitudes toward NT screening and perspectives on the consent process. A follow-up questionnaire assessed satisfaction with the test. Prenatal care providers also completed a questionnaire ascertaining their perspectives on NT screening. RESULTS: A total of 107 patients completed the initial questionnaire and 78 completed the follow-up questionnaire. Although 98 (94%) patients desired NT screening only 38 (38%) indicated that they would undergo diagnostic testing if they received screen positive results. Only 3 patients screened positive; however, 15 (20%) participants experienced increased anxiety after the test. Almost all of the 36 providers surveyed indicated that they counsel their patients thoroughly, but 38 (39%) patients reported that they received adequate information. CONCLUSION: NT screening is often performed without patients' full understanding of the implications of potential results and may cause anxiety. Providers should elicit patients' preferences regarding prenatal testing and engage them in shared decision making about whether to undergo screening, particularly when abortion is not an option. | Decision Making | Humans | Patient Participation | Patient Satisfaction | OK |
| James W. | Ferguson | James M. | Neuberger |  |  | Transplantation | 2014-02-27 Feb 27, 2014 | | Humans | Patient Participation | Questionnaires | Internet | OK |
| Jo | Brett | Liz | Tutton |  |  | The Patient | 2012-00-00 2012 | This paper considers the potential for collaborative patient and public involvement in the development, application, evaluation, and interpretation of patient-reported outcome measures (PROMs). The development of PROMs has followed a well trodden methodological path, with patients contributing as research subjects to the content of many PROMs. This paper argues that the development of PROMs should embrace more collaborative forms of patient and public involvement with patients as research partners in the research process, not just as those individuals who are consulted or as subjects, from whom data are sourced, to ensure the acceptability, relevance, and quality of research. We consider the potential for patients to be involved in a much wider range of methodological activities in PROM development working in partnership with researchers, which we hope will promote paradigmal evolution rather than revolution. | Humans | Patient Participation | Cooperative Behavior | self report | OK |
| Karen | Tse | Peter | Rakaric | Bernard | Cummings | Journal of Cancer Education: The Official Journal of the American Association for Cancer Education | 2013-09-00 Sep 2013 | The purpose of this study was to understand and compare patient and staff perceptions of a video-based preparatory education tool for head and neck radiotherapy. Patients and staff completed a questionnaire assessing their perceptions of whether the education tool was relevant, clear, complete and reassuring. Staff rated the video's accuracy and anticipated impact on future patient information needs. Demographic information was collected. Open-ended questions were used to elicit additional feedback. Quantitative responses from 50 patients and 48 staff were very positive and not significantly different between the two groups. Content analysis of the qualitative data provided insight into the information and approaches valued by patients and staff and how these differed. Staff members were more critical of the production quality and completeness of information related to procedures and treatment side effects. Patients valued seeing procedures acted out and desired more information about what these experiences would feel like and how to engage in self-care. Although staff-driven development may be an effective method of designing the content and approach of a preparatory education video, care should be taken to consider differences between patient and staff perceptions of information needs. | Male | Humans | Patient Participation | Female | OK |
|  |  |  |  |  |  | Wiener Klinische Wochenschrift | 2012-06-00 Jun 2012 | The majority of therapeutic decision-making takes place between therapists and elderly people. The present study sought to determine the individual preferences of elderly people in the therapist-patient interaction. From October 2010 to January 2011, data from elderly students (n = 297, mean age  > 60) were collected at three different university sites in Germany (Halle-Wittenberg, Cologne and Munich). The study used a structured questionnaire. Younger students (n = 182, mean age = 20.5) at the University of Halle-Wittenberg were questioned as well, in order to show age-specific differences. None of the participants was hospitalised. At the University of Munich, elderly students (n = 42) were given interaction-skill training for patients prior to taking part in the study. The effect of the intervention was measured in the study. Elderly participants and especially those with higher morbidity and a poorer state of information about their state of health refused to get involved in the process of medical decision-making. A group of elderly participants who received special interaction-skill training for patients afterwards preferred an equal therapist-patient relationship as well as shared decision-making. Younger participants favored an informative-participative interaction. The guidelines of therapeutic decision-making that are applied in practise do not always meet the individual needs of the elderly. Interaction-skill training for patients has great potential to both: encourage patients to get involved in the process of medical decision-making and to assume more autonomy. Hence, the aim should be to compound autonomy-empowerment and interaction-skill training for elderly people. | Humans | Patient Participation | Physician-Patient Relations | Questionnaires | OK |
| A. | Solari | F. | Geiger | J. | Kasper | Journal of the Neurological Sciences | 2013-08-15 Aug 15, 2013 | Patient autonomy has been increasingly acknowledged as prerequisite for successful medical decision making in Western countries. In medical decisions with a need to involve a health professional, patient autonomy becomes apparent in the extent of patients' participation in the communication as described in the concept of shared decision making. Patient autonomy can be derived from different perspectives or goals and the focus of evaluation approaches may vary accordingly. Multiple sclerosis (MS) is a paradigmatic disease to study patient autonomy mainly because MS patients are highly disease competent and due to ambiguous evidence on many aspects of disease-related medical decision making. This review gives an overview on measurement issues in studying decision making in MS, categorized according to prerequisites, process measures and outcomes of patient autonomy. As relevant prerequisites role preferences, risk attribution, risk tolerance, and risk knowledge are discussed. Regarding processes, we distinguish intra-psychic and interpersonal aspects. Intra-psychic processes are elucidated using the theory of planned behavior, which guided development of a 30-item scale to capture decisions about immunotherapy. Moreover, a theory of uncertainty management has been created resulting in the development of a corresponding measurement concept. Interpersonal processes evolving between physician and patient can be thoroughly analyzed from different perspectives by use of the newly developed comprehensive MAPPIN'SDM inventory. Concerning outcomes, besides health related outcomes, we discuss match of preferred roles during the decision encounters (preference match), decisional conflict as well as an application of the multidimensional measure of informed choice to decisions of MS patients. These approaches provide an overview on patient-inherent and interpersonal factors and processes modulating medical decision making and health behavior in MS and beyond. | Decision Making | Humans | Patient Participation | Physician-Patient Relations | OK |
| A. | Solari | F. | Geiger | J. | Kasper | Journal of the Neurological Sciences | 2013-08-15 août 15, 2013 | Patient autonomy has been increasingly acknowledged as prerequisite for successful medical decision making in Western countries. In medical decisions with a need to involve a health professional, patient autonomy becomes apparent in the extent of patients' participation in the communication as described in the concept of shared decision making. Patient autonomy can be derived from different perspectives or goals and the focus of evaluation approaches may vary accordingly. Multiple sclerosis (MS) is a paradigmatic disease to study patient autonomy mainly because MS patients are highly disease competent and due to ambiguous evidence on many aspects of disease-related medical decision making. This review gives an overview on measurement issues in studying decision making in MS, categorized according to prerequisites, process measures and outcomes of patient autonomy. As relevant prerequisites role preferences, risk attribution, risk tolerance, and risk knowledge are discussed. Regarding processes, we distinguish intra-psychic and interpersonal aspects. Intra-psychic processes are elucidated using the theory of planned behavior, which guided development of a 30-item scale to capture decisions about immunotherapy. Moreover, a theory of uncertainty management has been created resulting in the development of a corresponding measurement concept. Interpersonal processes evolving between physician and patient can be thoroughly analyzed from different perspectives by use of the newly developed comprehensive MAPPIN'SDM inventory. Concerning outcomes, besides health related outcomes, we discuss match of preferred roles during the decision encounters (preference match), decisional conflict as well as an application of the multidimensional measure of informed choice to decisions of MS patients. These approaches provide an overview on patient-inherent and interpersonal factors and processes modulating medical decision making and health behavior in MS and beyond. (PsycINFO Database Record (c) 2013 APA, all rights reserved). (journal abstract) | Decision Making | medical decision making | patient autonomy | multiple sclerosis | Doublon |
|  |  |  |  |  |  | International journal for quality in health care: journal of the International Society for Quality in Health Care / ISQua | 2011-10-00 Oct 2011 | QUALITY PROBLEM: Patient-centred care is increasingly being acknowledged as an integral part of evaluating health care. Yet, from a quality improvement perspective the rationale, measurement and implementation of strategies to improve patient-centred care is often subject to debate. OBJECTIVE: The aim of this paper is to review why quality improvement efforts should embrace patient-centredness, to examine some of the measurement issues and to assess conceptual underpinnings that should inform both measurement and actions to improve patient-centred care. LESSONS: The causal pathway through which quality improvement affects and/or is associated with patient centredness is complex and goes beyond patients' rights and assessing patient views. Interventions to improve patient-centred care should reflect on key rationale, measurement strategy and underlying theory. | Humans | Patient Participation | patient preference | Hospitals | OK |
| Hua | He | Zarina | Ali | Thomas M. | Richardson | Emergency medicine journal: EMJ | 2010-06-00 Jun 2010 | BACKGROUND: Patient satisfaction is related to the perception of care. Some patients prefer, and are more satisfied with, providers of the same gender, race or religious faith. This study examined emergency medical provider attitudes towards, as well as patient and provider characteristics that are associated with, accommodating such requests. METHODS: A survey administered to a convenience sample of participants at the 2007 American College of Emergency Physicians Scientific Assembly. The nine-question survey ascertained Likert-type responses to the likelihood of accommodating patient requests for specific provider types. Statistical analyses used Wilcoxon rank-sum, Wilcoxon signed-rank and Cochran's Q tests. RESULTS: The 176 respondents were predominantly white (83%) and male (74%), with a mean age of 42 y. Nearly a third of providers felt that patients perceive better care from providers of shared demographics with racial matching perceived as more important than gender or religion (p=0.02). Female providers supported patient requests for same gender providers more so than males (p<0.01). Provider race, practice location, type and duration did not significantly affect the level of accommodation. When requesting like providers, female patients had higher accommodation scores than male patients (p<0.001), non-whites than whites (p<0.05), with Muslim patients (male or female) most likely to be accommodated (p<0.01). CONCLUSION: Accommodating patient requests for providers of specific demographics within the emergency department may be related to provider characteristics. When patients ask for same gender providers, female providers are more likely to accommodate such a request than male providers. Female, non-white and Muslim patients may be more likely to have their requests honoured for matched providers. | Male | Humans | Patient Participation | Patient Satisfaction | OK |
| Shawn J. | Pelletier | Brian J. | Zikmund-Fisher | Anna S. F. | Lok | Liver Transplantation: Official Publication of the American Association for the Study of Liver Diseases and the International Liver Transplantation Society | 2011-12-00 Dec 2011 | It is challenging to discuss the use of high-risk organs with patients, in part because of the lack of information about how patients view this topic. This study was designed to determine how patients think about organ quality and to test formats for risk communication. Semistructured interviews of 10 patients on the waiting list revealed limited understanding about the spectrum of organ quality and a reluctance to consider anything but the best organs. A computerized quantitative survey was then conducted with an interactive graph to elicit the risk of graft failure that patients would accept. Fifty-eight percent of the 95 wait-listed patients who completed the survey would accept only organs with a risk of graft failure of 25% or less at 3 years, whereas 18% would accept only organs with the lowest risk possible (19% at 3 years). Risk tolerance was increased when the organ quality was presented relative to average organs rather than the best organs and when feedback was provided about the implications for organ availability. More than three-quarters of the patients reported that they wanted an equal or dominant role in organ acceptance decisions. Men tended to prefer lower risk organs (mean acceptable risk = 29%) in comparison with women (mean acceptable risk = 35%, P = 0.04), but risk tolerance was not associated with other demographic or clinical characteristics (eg, the severity of liver disease). In summary, patients want to be involved in decisions about organ quality. Patients' risk tolerance varies widely, and their acceptance of high-risk organs can be facilitated if we present the risks of graft failure with respect to average organs and provide feedback about the implications for organ availability. | Time Factors | Male | Humans | Patient Participation | OK |
| Jianbo | Li | Manoj | Monga |  |  | Urology | 2013-02-00 Feb 2013 | OBJECTIVE: To evaluate which variables affect patients' decisions on the management of asymptomatic renal calculi. MATERIALS AND METHODS: We hypothesized that patients who had experienced greater pain, passed larger or more stones, or passed stones more recently would be more likely to choose surgical intervention (ureteroscopy [URS] or shock wave lithotripsy [ESWL]) instead of observation (OBS). The survey was distributed to 101 patients in our stone clinic. The patients were given a hypothetical scenario of an asymptomatic 8-mm lower pole stone and descriptions for OBS (annual radiography, 40% chance of growth >10 mm within 4 years, 20% chance of passage), ESWL (65% success rate), and URS (90% success rate, with stent placement for 1 week). The patients were also asked whether they would rather defer the decision to their physician. RESULTS: Of the patients, 22.8% chose OBS, 29.7% chose URS, and 47.5% chose ESWL. The patients who had passed larger stones (P = .029) were less likely to choose OBS over surgery. Of the 78 patients who chose intervention, 61.5% preferred ESWL. The patients were more likely to choose URS if they had previously undergone URS (P = .0064) and stent placement (P = .048), and the patients were more likely to choose ESWL if they had a previously undergone ESWL (P = .019). Surgical choice was not affect by size of the largest stone passed (P = .46), date of last passage (P = .080), previous pain intensity (P = .11), previous percutaneous nephrolithotomy (P = .73), or number of stones passed (P = .51). Finally, 56.4% of patients deferred the decision of the treatment approach to the physician. CONCLUSION: Previous stone experience and treatment significantly affected treatment choice. Patients rely on their physician to educate them on the alternatives and play an active role in selecting the treatment approach. | Decision Making | Humans | Patient Participation | Questionnaires | OK |
|  |  |  |  |  |  | Journal of Clinical Rheumatology: Practical Reports on Rheumatic & Musculoskeletal Diseases | 2011-08-00 Aug 2011 | | Male | Decision Making | Humans | Patient Participation | OK |
| I.-Chen | Hung | Yu-Jiun | Lin | Ya-Ling | Yang | American Journal of Infection Control | 2013-11-00 Nov 2013 | BACKGROUND: "Patient empowerment" is an important component of World Health Organization hand hygiene program, but little is known about the intentions and attitude of patients/families and health care workers (HCWs) regarding this. METHODS: A cross-sectional survey using questionnaires was conducted in a tertiary teaching hospital in Taiwan to assess hand hygiene knowledge and the attitudes and intentions regarding patient empowerment among patients/families and HCWs. RESULTS: Among patients/families, 95.4% (329/345) had positive attitudes regarding patient empowerment; however, only 67.2% (232/345) had the positive intention to remind HCWs about hand hygiene (P < .001). Risk factors for negative intention were being female (odds ratio [OR], 1.82; 95% confidence interval [CI]: 1.08-3.03), illiteracy (OR, 3.18; 95% CI: 0.86-11.7), and being patients/families in the pediatric department (OR, 1.86; 95% CI: 0.93-3.64). Among HCWs, the difference between positive attitude (81.1%; 714/880) and positive intention regarding being reminded about hand hygiene (62.8%; 553/880) was significant (P < .001). Risk factors for negative intention were age > 25 years (OR, 3.20; 95% CI: 1.51-6.81) and a negative attitude toward patient empowerment (OR, 10.00; 95% CI: 5.88-16.67). CONCLUSION: There were significant gaps between attitude and intention regarding patient empowerment both among patients/families and HCWs. Special strategies targeting women, the pediatric population, or illiterate people may help improve patient/family participation. Additionally, hand hygiene education should be incorporated into early-stage medical/nursing education to create a facilitating environment. Patients/families and HCWs cooperation is needed to promote the hand hygiene program further. | Male | Humans | Patient Participation | Questionnaires | OK |
| Juan | de Dios Luna | Lorena | Saletti Cuesta | Natalia | Gil Garrido | Gaceta Sanitaria / S.E.S.P.A.S | 2010-02-00 2010 Jan-Feb | OBJECTIVES: To identify patient expectations of clinical decision-making at consultations with their general practitioners for distinct health problems and to determine the patient and general practitioner characteristics related to these expectations, with special focus on gender. METHODS: We performed a multicenter cross-sectional study in 360 patients who were interviewed at home. Data on patients' sociodemographic, clinical characteristics and satisfaction were gathered. General practitioners supplied information on their gender and postgraduate training in family medicine. A questionnaire was used to collect data on patients' expectations that their general practitioner <listen, explain, and take account of their opinion and on expectations of clinical decision making> at consultations with their general practitioner for five problems or hypothetical clinical scenarios (strong chest pain/cold with fever/abnormal discharge/depression or sadness/severe family problem). Patients were asked to indicate their preference that decisions on diagnosis and treatment be taken by: a) the general practitioner alone; b) the general practitioner, taking account of the patient's opinion; c) the patient, taking account of the general practitioner's opinion and d) the patient alone. A logistic regression was performed for clinical decision-making. RESULTS: The response rate was 90%. The mean age was 47.3 + or - 16.5 years and 51% were female. Patients' expectations that their general practitioner listen, explain and take account of their opinions were higher than their expectations of participating in decision-making, depending on the problem in question: 32% wished to participate in chest pain and 49% in family problems. Women had lower expectations of participating in depression and family problems. Patients with female general practitioners had higher expectations of participating in family problems and colds. CONCLUSIONS: Most patients wished to be listened to, informed and taken into account by their general practitioners and, to a lesser extent, wished to take decisions autonomously, especially for biomedical problems. | Male | Decision Making | Humans | Patient Participation | OK |
| Annie W. L. | Cheung | Carrie H. K. | Yam | E. K. | Yeoh | Hong Kong Medical Journal = Xianggang Yi Xue Za Zhi / Hong Kong Academy of Medicine | 2012-10-00 Oct 2012 | OBJECTIVE. To measure patient satisfaction in relation to in-patient experience in public hospitals. DESIGN. Cross-sectional study. SETTING. Twenty-five selected Hospital Authority acute and convalescence hospitals in Hong Kong. PARTICIPANTS. Eligible patients discharged between 15 June and 27 September 2010 from the selected Hospital Authority public hospitals. MAIN OUTCOME MEASURES. A total of 54 items were used to measure patient experience on aspects of hospital care. They included the process of admission to hospital, staying in the hospital and ward (environment, food and facilities; hospital staff; patient care and treatment), the process of leaving hospital, and the overall impression of hospital care. Free-text comments from respondents were also recorded. RESULTS. A total of 5030 patients were successfully interviewed, amounting to a response rate of 52%. The findings showed that 80% (confidence interval, 79-81%) of patients rated the care they received in hospital as good or better. However, there were a few areas where performance was relatively low, including waiting time for a ward bed for accident and emergency cases, food quality, infection control, information provided about their condition/treatment, seeking patient input about their opinions and quality of care, and patient engagement in the decisions about their treatment and care, as well as the discharge process. CONCLUSIONS. This patient experience survey used a validated instrument (Hong Kong Inpatient Experience Questionnaire) to provide important insights to executives and health care professionals on their care to patients and to identify areas for improvement in public hospitals. Further surveys should be carried out to monitor changes in patient experience and satisfaction on a regular basis. Such surveys could facilitate improvements through analysis of results on patient satisfaction. | Time Factors | Male | Humans | Patient Participation | OK |
|  |  |  |  |  |  | Soins; La Revue De Référence Infirmière | 2013-12-00 Dec 2013 | | Humans | Patient Participation | Hospitalization | Patient Education as Topic | OK |
| Kathy S. | Albain | Carol M. | Moinpour | Judith A. | Petersen | Journal of Clinical Oncology: Official Journal of the American Society of Clinical Oncology | 2013-02-10 Feb 10, 2013 | PURPOSE: Studies have shown an association between socioeconomic status (SES) and quality of oncology care, but less is known about the impact of patient SES on clinical trial participation. PATIENTS AND METHODS: We assessed clinical trial participation patterns according to important SES (income, education) and demographic factors in a large sample of patients surveyed via an Internet-based treatment decision tool. Logistic regression, conditioning on type of cancer, was used. Attitudes toward clinical trials were assessed using prespecified items about treatment, treatment tolerability, convenience, and cost. RESULTS: From 2007 to 2011, 5,499 patients were successfully surveyed. Forty percent discussed clinical trials with their physician, 45% of discussions led to physician offers of clinical trial participation, and 51% of offers led to clinical trial participation. The overall clinical trial participation rate was 9%. In univariate models, older patients (P = .002) and patients with lower income (P = .001) and education (P = .02) were less likely to participate in clinical trials. In a multivariable model, income remained a statistically significant predictor of clinical trial participation (odds ratio, 0.73; 95% CI, 0.57 to 0.94; P = .01). Even in patients age ≥ 65 years, who have universal access to Medicare, lower income predicted lower trial participation. Cost concerns were much more evident among lower-income patients (P < .001). CONCLUSION: Lower-income patients were less likely to participate in clinical trials, even when considering age group. A better understanding of why income is a barrier may help identify ways to make clinical trials better available to all patients and would increase the generalizability of clinical trial results across all income levels. | Male | Decision Support Techniques | Humans | Patient Participation | OK |
|  |  |  |  |  |  | Der Unfallchirurg | 2011-01-00 Jan 2011 | BACKGROUND: In order to be able to make a differentiated choice between healthcare providers, people require information regarding quality. An understanding of patient needs and preferences is crucial in providing helpful information regarding hospital quality. METHODS: This study investigated patient involvement in, and preferences for, information on hospital quality using a choice-based experiment based on best-worst scaling in a questionnaire. A total of 276 respondents participated including hospital patients and healthy persons (response rate 71%). The work was conducted in 2008. RESULTS: The analysis showed a high involvement in information regarding hospital quality. In the measurement of information preferences (35 items/quality indicators), patients rated indicators of structure quality as the most important attributes. Information about process quality was moderately relevant from the patients' point of view. Objective results of outcome quality were more important for patients than subjective quality indicators. CONCLUSION: Both the assessment of patient involvement in hospital quality information and the measurement of patient preferences in order to rank patient perception provide important insights into information needs of patients. The findings can subsequently be used for tailoring patient information services regarding hospital quality. | Humans | Patient Participation | Questionnaires | Hospitalization | OK |
| Pawel R. | Nowak | Wieslawa | Szumilo-Grzesik | |  | Health Expectations: An International Journal of Public Participation in Health Care & Health Policy | 2010-03-00 mars 2010 | Background: Promoting a more patient-responsive service has been the focus of policy initiatives in newer EU states. One measure of success should be the patient's assessment of their consultation with their doctor. Objectives: To measure consultation quality in Polish primary care using patient enablement (a patient-driven instrument developed in the UK) and to test its theoretical framework. To compare the patient enablement outcome of different types of doctor delivering primary care in Poland following reform. Design: Cross-sectional quantitative questionnaire survey. Setting: Random sample of primary care doctors practicing within a 60-km radius of Gdansk, Poland. Subjects and outcome measures: Patient Enablement Instrument and correlates were measured in 7924 consecutive adult consultations of 48 doctors, stratified according to training: family medicine specialists (diploma holders), non-diplomats and general medicine doctors (polyclinic internists). Results: Completion was high (78%). The mean patient enablement score in Poland was 4.0 (SD 3.3) and mean consultation length was 10.3 min (SD 5.4 min). Consultation length and knowing the doctor are independently related to patient enablement in the Polish context. Variation between doctors is significant, but earlier differences in enablement between alternative providers have largely been ameliorated in practice. Conclusion: It is feasible to use patient enablement on a large scale at routine consultation in primary care in Poland: acceptability was good in diverse environments. The internal consistency of enablement and its relationships broadly mirror those found in the UK. The effect of patient expectations shaped by social and cultural issues influencing enablement outcome requires further investigation. (PsycINFO Database Record (c) 2012 APA, all rights reserved). (journal abstract) | patients | Primary Health Care | Professional Consultation | primary care | Doublon |
| Pawel R. | Nowak | Wieslawa | Szumilo-Grzesik | |  | Health Expectations: An International Journal of Public Participation in Health Care and Health Policy | 2010-03-00 Mar 2010 | BACKGROUND: Promoting a more patient-responsive service has been the focus of policy initiatives in newer EU states. One measure of success should be the patient's assessment of their consultation with their doctor. OBJECTIVES: To measure consultation quality in Polish primary care using patient enablement (a patient-driven instrument developed in the UK) and to test its theoretical framework. To compare the patient enablement outcome of different types of doctor delivering primary care in Poland following reform. DESIGN: Cross-sectional quantitative questionnaire survey. SETTING: Random sample of primary care doctors practising within a 60-km radius of Gdansk, Poland. SUBJECTS AND OUTCOME MEASURES: Patient Enablement Instrument and correlates were measured in 7924 consecutive adult consultations of 48 doctors, stratified according to training: family medicine specialists (diploma holders), non-diplomates and general medicine doctors (polyclinic internists). RESULTS: Completion was high (78%). The mean patient enablement score in Poland was 4.0 (SD 3.3) and mean consultation length was 10.3 min (SD 5.4 min). Consultation length and knowing the doctor are independently related to patient enablement in the Polish context. Variation between doctors is significant, but earlier differences in enablement between alternative providers have largely been ameliorated in practice. CONCLUSION: It is feasible to use patient enablement on a large scale at routine consultation in primary care in Poland: acceptability was good in diverse environments. The internal consistency of enablement and its relationships broadly mirror those found in the UK. The effect of patient expectations shaped by social and cultural issues influencing enablement outcome requires further investigation. | Male | Humans | Patient Participation | Patient Satisfaction | OK |
| Luis Angel | Pérula de Torres | Enrique | Gavilán Moral | José Ramón | Loayssa Lara | Atencion Primaria / Sociedad Española De Medicina De Familia Y Comunitaria | 2010-05-00 May 2010 | OBJECTIVES: This work aims to explore to what extent Spanish primary care providers involve patients in decisions and describe the development of a suggested tool for assessing patient involvement in these settings. DESIGN: Cross-sectional, development of a measurement tool. SETTING: Primary care clinics. PARTICIPANTS: Family doctors and residents. INTERVENTIONS: Based on a review of the literature and the opinions of primary care doctors, a selection was made of items from a previous scale used to measure general communication skills (CICAA-Patient Centred) and new specific items were added to this to measure involvement. MAIN MEASUREMENTS: The involvement of patients in decision-making was evaluated initially with this tool in 31 different clinical visits and the scale was then reformulated. A pool of 161 interviews was used to complete the process. Some psychometric properties (reliability and internal consistency) were estimated for the different samples and stages of the process. RESULTS: Some degree of patient involvement was found in just 31 visits. Despite this, only in 18 of these (58%) was there some involvement in a discussion about more than one treatment option. The Cohen's kappa values of the CICAA-Decision scale were between 0.48 and 0.94. Cronbach's alpha was 0.60/0.51. The global Intra-class correlation coefficient was 0.96. CONCLUSIONS: The levels of patient involvement were lower than expected. A simple question, such as that defined by one item in particular, and the CICAA-D scale, in general, could be useful to assess patient involvement in decision making in primary care. | Humans | Patient Participation | Questionnaires | Primary Health Care | OK |
| Mina O. | Rakoski | Sherry | Fu | Grace L. | Su | Journal of Clinical Gastroenterology | 2011-09-00 Sep 2011 | BACKGROUND: Surveillance for hepatocellular carcinoma (HCC) is recommended in patients with cirrhosis, but earlier studies suggest that it is used less than one-third of the time. Patient factors associated with surveillance rates are incompletely understood. GOALS: The aims of our study were to determine HCC surveillance rates in a tertiary-care center and to identify patient predictors of receiving surveillance. STUDY: Patients with Child A or B cirrhosis seen in the University of Michigan liver clinics between October 2008 and March 2009 were enrolled to complete a self-administered survey. Surveillance rates and clinical data were extracted from the patient electronic medical record. RESULTS: Of the 160 patients enrolled, 74.4% had HCC surveillance performed in the past year. On multivariate analysis, predictors of receiving surveillance included male sex (odds ratio 7.1, 95% confidence interval, 1.2-43.2) and patient involvement in their care (odds ratio 3.4, 95% confidence interval, 1.5-7.9). Patients expressed high levels of concern regarding HCC, desired more information from their physicians, and wanted to be more involved in their care. CONCLUSIONS: HCC surveillance rates in a tertiary-care center were significantly higher than earlier reported rates. Direct patient involvement in decisions regarding HCC surveillance may help to improve surveillance rates. | Time Factors | Male | Humans | Patient Participation | OK |
| Mel | Brooke | Willemina | Campbell | Laura C. | Coates | Current Rheumatology Reports | 2014-05-00 May 2014 | Psoriatic arthritis (PsA) is a heterogeneous inflammatory arthritis with a varied clinical phenotype. There has been considerable international collaboration over recent years to develop and prioritise appropriate disease domains and outcome measures to capture all aspects of this complex disease. It has been recognised that patient-reported measures and physician assessments are complementary and, when used together, allow an improved reflection of disease burden. Taking this concept one step further, the experience in rheumatoid arthritis has demonstrated benefits of incorporating the patient perspective in the development of outcome measures. We report a systematic review demonstrating (1) that there has been little incorporation of the patient perspective in the development of outcome measures and domains in PsA, (2) the proceedings from the preliminary patient involvement in outcome measures for PsA (PIOMPSA) meetings, and (3) a proposed roadmap for improving patient involvement. | Humans | Patient Participation | Severity of Illness Index | Treatment Outcome | OK |
| Rebecca | Lawton | Gerry | Armitage | Ian | Watt | BMC health services research | 2011-00-00 2011 | BACKGROUND: Patients have the potential to provide a rich source of information on both organisational aspects of safety and patient safety incidents. This project aims to develop two patient safety interventions to promote organisational learning about safety - a patient measure of organisational safety (PMOS), and a patient incident reporting tool (PIRT) - to help the NHS prevent patient safety incidents by learning more about when and why they occur. METHODS: To develop the PMOS 1) literature will be reviewed to identify similar measures and key contributory factors to error; 2) four patient focus groups will ascertain practicality and feasibility; 3) 25 patient interviews will elicit approximately 60 items across 10 domains; 4) 10 patient and clinician interviews will test acceptability and understanding. Qualitative data will be analysed using thematic content analysis.To develop the PIRT 1) individual and then combined patient and clinician focus groups will provide guidance for the development of three potential reporting tools; 2) nine wards across three hospital directorates will pilot each of the tools for three months. The best performing tool will be identified from the frequency, volume and quality of reports. The validity of both measures will be tested. 300 patients will be asked to complete the PMOS and PIRT during their stay in hospital. A sub-sample (N = 50) will complete the PMOS again one week later. Health professionals in participating wards will also be asked to complete the AHRQ safety culture questionnaire. Case notes for all patients will be reviewed. The psychometric properties of the PMOS will be assessed and a final valid and reliable version developed. Concurrent validity for the PIRT will be assessed by comparing reported incidents with those identified from case note review and the existing staff reporting scheme. In a subsequent study these tools will be used to provide information to wards/units about their priorities for patient safety. A patient panel will provide steering to the research. DISCUSSION: The PMOS and PIRT aim to provide a reliable means of eliciting patient views about patient safety. Both interventions are likely to have relevance and practical utility for all NHS hospital trusts. | Humans | Patient Participation | Questionnaires | United States | OK |
| Anssi | Peräkylä |  |  |  |  | Communication & Medicine | 2010-00-00 2010 | This article reports a conversation analytic study of primary care physicians' orientations to different types of patients' problem presentation. Four types of problem presentation are examined: 1. symptoms only; 2. candidate diagnosis; 3. diagnosis implicative symptom description; and 4. candidate diagnosis as background information. The analysis shows that both in receiving the problem presentation at the beginning of the visit and in delivering a diagnosis later on, doctors address the patients' presentations which involved or implied a candidate diagnosis. In contrast, following a symptoms-only type of problem presentation such references predominantly are not made. The study suggests that patients' problem presentation have a crucial role in shaping the doctor's communication patterns also in the phases of consultation in which the patient's active participation is of lesser significance, such as the diagnostic phase. The findings are discussed in relation to the question of patient participation in the medical consultation. The data consist of 86 video-recorded Finnish primary care consultations for upper respiratory tract infection including both child and adult patients. | Male | Humans | Patient Participation | Physician-Patient Relations | OK |
| Ülla | Linnamägi |  |  |  |  | European Spine Journal: Official Publication of the European Spine Society, the European Spinal Deformity Society, and the European Section of the Cervical Spine Research Society | 2014-04-00 Apr 2014 | PURPOSE: To evaluate involvement of patients in surgical treatment decision making (STDM) in relations to satisfaction with the results of lumbar discectomy. METHODS: We evaluated prospectively 150 surgically treated patients with radicular pain due to lumbar disc herniation (LDH). The patients completed self-reported questionnaires about preferences for involvement and actual involvement in STDM. Global satisfaction with the results of the treatment was assessed at 1 year after the operation. RESULTS: Most of the patients (129 patients, 86%) stated that they had been sufficiently informed about LDH to be involved in the treatment decisions, almost half of the patients (47%) preferred active or collaborative involvement and 58% of the patients reported higher actual involvement in STDM. Congruence between preferred and actual roles in decision making was 64%. Most of the patients (77%) were satisfied with the results of the operation, but satisfaction was not associated with involvement of patients in STDM. CONCLUSION: A significant proportion of patients with LDH prefer to be actively involved in treatment decisions and experience an STDM process that matches their preferences for participation. However, individual differences in preferences for involvement in STDM are common and global satisfaction with the treatment results is not significantly related to the activity of involvement in STDM. | Male | Humans | Patient Participation | Patient Satisfaction | OK |
| Aws | Al-Hity | Naomi D. | Quinton | Trudie E. | Roberts | Medical Education | 2010-04-00 avril 2010 | Objectives: There are recognised difficulties in teaching and assessing intimate examination skills that relate to the sensitive nature of the various examinations and the anxiety faced by novice learners. This systematic review provides a summary of the evidence for the involvement of real patients (RPs) and simulated patients (SPs) in the training of health care professionals in intimate examination skills. Methods: For the review, ‘intimate examinations’ included pelvic, breast, testicular and rectal examinations. Major databases were searched from the start of the database to December 2008. The synthesis of findings is integrated by narrative structured to address the main research questions, which sought to establish: the objectives of programmes involving RPs and SPs as teachers of intimate examination skills; reasons why SPs have been involved in this training; the evidence for the effectiveness of such training programmes; the evidence for measures of anxiety in students learning how to perform intimate examinations; how well issues of sexuality are addressed in the literature; any reported negative effects of involvement in teaching on the patients, and suggestions for practical strategies for involving patients in the teaching of intimate examination skills. Results: A total of 65 articles were included in the review. Involving patients in teaching intimate examination skills offers advantages over traditional methods of teaching. Objective evidence for the effectiveness of this method is demonstrated through improved clinical performance, reduced anxiety and positive evaluation of programmes. Practical strategies for implementing such programmes are also reported. Conclusions: There is evidence of a short-term positive impact of patient involvement in the teaching and assessment of intimate examination skills; however, evidence of longer-term impact is still limited. The influences of sexuality and anxiety related to such examinations are explored to some extent, but the psychological impact on learners and patients is not well addressed. (PsycINFO Database Record (c) 2012 APA, all rights reserved). (journal abstract) | Measurement | Patient involvement | patients | Health Education | Doublon |
| Aws | Al-Hity | Naomi D. | Quinton | Trudie E. | Roberts | Medical Education | 2010-04-00 Apr 2010 | OBJECTIVES: There are recognised difficulties in teaching and assessing intimate examination skills that relate to the sensitive nature of the various examinations and the anxiety faced by novice learners. This systematic review provides a summary of the evidence for the involvement of real patients (RPs) and simulated patients (SPs) in the training of health care professionals in intimate examination skills. METHODS: For the review, 'intimate examinations' included pelvic, breast, testicular and rectal examinations. Major databases were searched from the start of the database to December 2008. The synthesis of findings is integrated by narrative structured to address the main research questions, which sought to establish: the objectives of programmes involving RPs and SPs as teachers of intimate examination skills; reasons why SPs have been involved in this training; the evidence for the effectiveness of such training programmes; the evidence for measures of anxiety in students learning how to perform intimate examinations; how well issues of sexuality are addressed in the literature; any reported negative effects of involvement in teaching on the patients, and suggestions for practical strategies for involving patients in the teaching of intimate examination skills. RESULTS: A total of 65 articles were included in the review. Involving patients in teaching intimate examination skills offers advantages over traditional methods of teaching. Objective evidence for the effectiveness of this method is demonstrated through improved clinical performance, reduced anxiety and positive evaluation of programmes. Practical strategies for implementing such programmes are also reported. CONCLUSIONS: There is evidence of a short-term positive impact of patient involvement in the teaching and assessment of intimate examination skills; however, evidence of longer-term impact is still limited. The influences of sexuality and anxiety related to such examinations are explored to some extent, but the psychological impact on learners and patients is not well addressed. | Male | Humans | Patient Participation | Female | OK |
| Helen E. | Smith |  |  |  |  | The Journal of Allergy and Clinical Immunology | 2013-12-00 Dec 2013 | | Humans | self report | quality of life | Patient Outcome Assessment | Doublon |
| Helen E. | Smith |  |  |  |  | The Journal of Allergy and Clinical Immunology | 2013-12-00 Dec 2013 | | Humans | self report | quality of life | Patient Outcome Assessment | OK |
| Claudia | Appel |  |  |  |  | Psychotherapie, Psychosomatik, Medizinische Psychologie | 2010-05-00 May 2010 | OBJECTIVES: Patient involvement (PI) and shared decision making (SDM) have increasingly come into the focus of (inter-)national research. SDM, however, is not a standard component of standard care so far. Therefore it is of importance to take into consideration all parties concerned. This article points out the barriers physicians see; one group has not been interrogated on this topic often. METHOD: 25 physicians at a clinical centre were interviewed about decision-making processes within their environment, their concerns as well as their ideas towards PI. The consultations were realized with the help of a guideline-oriented questionnaire. The outcome was then interpreted by means of the thematic content analysis. RESULTS: A system of categories was constructed containing 28 categories on the micro-, meso-, and macro-level. From a physician's perspective, changes are necessary on all levels to achieve patient involvement and SDM. Essential points of approach for this are: sufficient time and personnel, sufficient communicative abilities in physicians, and a solid physician-patient relationship as well as changes in healthcare politics. CONCLUSIONS: To consistently improve patient involvement and SDM, physicians' concerns must be taken seriously. Here SDM can only be properly applied by taking into account the meso- and macro-level. Furthermore, it would be helpful to clear up the conceptually vague aspects of SDM, which would also help to communicate the concept more clearly. | Male | Decision Making | Humans | Patient Participation | OK |
| Adelaida | Castillo | Cheryl | Koopman |  |  | International Clinical Psychopharmacology | 2010-01-00 janvier 2010 | The motivation for clinical trials inventory-depression (MCTI-D) was developed and evaluated for assessing motivations to participate in clinical trials on depression. Sixty-four participants completed the MCTI-D: 40 individuals expressing interest in participating in a randomized clinical trial (RCT); and 24 clinic patients receiving traditional care for depression. Content validity was supported by feedback derived from a panel of experts in depression research and respondents completing the measure. The motivation most frequently endorsed for participating in an RCT was the desire to help others and/or to further science. The potential stigma associated with seeing a psychiatrist was reported to have the least influence. Patients expressed a greater likelihood to participate in RCTs that involved psychotherapy than in experimental medication or placebo-controlled trials. Data from the MCTI-D may provide useful information for depression researchers to consider as possible influences on patients' decisions about whether or not they will participate. (PsycINFO Database Record (c) 2012 APA, all rights reserved). (journal abstract) | Patient Participation | Psychometrics | test validity | test reliability | OK |
| Luis Ángel | Pérula de Torres | Jorge | Olloqui Mundet | Teresa | Carrión de la Fuente | Atencion Primaria / Sociedad Española De Medicina De Familia Y Comunitaria | 2012-01-00 Jan 2012 | OBJECTIVES: To find out the views of the patient on their participation in decision making (DM) when visiting their family physician. DESIGN: A cross-sectional, qualitative and quantitative study. SETTING AND PARTICIPANTS: Patients attending their family doctors in diverse geographical Health Centres. MEASUREMENTS: Personal interviewing using different kinds of questions (close-ended, close-ended with options and open-ended questions). RESULTS: Patients participation: 658 (52 ± 17.4 years, 62% females, consulting with 97 doctors (from urban centres: 36 (458); rural centres: 22 (200). Most patients (94%; 620) declared to be satisfied with DM and up to 41% (266) thought that DM should be taken only by the doctor. Nevertheless, after the consultation 60% of patients (360) confirmed that they would have liked the physician to have asked them for their opinion, but the doctor did not encourage them to do this. Furthermore, patients considered information, discussion about options, ways to make decisions, medical advice, active listening and empathy as key aspects to encourage them to participate. CONCLUSIONS: After a medical consultation, most patients wanted to give their opinion about the proposals of treatment. Nevertheless, they felt that their doctors offered them these opportunities on very few occasions. Some types of questions are better than others in detecting of these kinds of needs, and are more useful to design strategies for involving patients in the DM process. | Male | Decision Making | Humans | Patient Participation | OK |
| Roland | Friele |  |  |  |  | BMC health services research | 2010-00-00 2010 | BACKGROUND: Little is known about the role European patient organisations play in the process of deciding on reimbursement for medicines. Therefore we explore the current role of patient organisations in the process of reimbursement for medicines in Western Europe. We focus in particular on collaboration between patient organisations and the pharmaceutical industry in this respect. METHODS: Sixty-eight patient organisations representing seven medical conditions, from ten Western European countries, were asked to participate in the study. The participating organisations reported their experiences in a web-based questionnaire. RESULTS: Twenty-one patient organisations completed the questionnaire (response rate: 31%), of which ten (47.6%) demanded reimbursement for medicines. Organisations demanding reimbursement were larger than those not demanding reimbursement. The main aim of these organisations was to create better accessibility of medicines for patients. Most organisations limited themselves to single actions. Only two engaged in multiple actions. Almost all organisations had general policies on cooperation with the pharmaceutical industry, with autonomy as the key feature. The patient organisations said they were reasonably successful and almost always satisfied with their own role in the reimbursement process. CONCLUSION: Our study has found that the role of European patient organisations in the reimbursement process still seems limited, especially for small patient organisations. | Humans | Patient Participation | Questionnaires | health policy | OK |
| Walter | Villani | Silvio | Garattini |  |  | PloS One | 2012-00-00 2012 | BACKGROUND: Many patients' and consumers' organizations accept drug industry funding to support their activities. As drug companies and patient groups move closer, disclosure become essential for transparency, and the internet could be a useful means of making sponsorship information accessible to the public. This survey aims to assess the transparency of a large group of Italian patient and consumer groups and a group of pharmaceutical companies, focusing on their websites. METHODOLOGY/PRINCIPAL FINDINGS: Patient and consumer groups were selected from those stated to be sponsored by a group of pharmaceutical companies on their websites. The websites were examined using two forms with principal (name of drug companies providing funds, amount of funding) and secondary indicators of transparency (section where sponsors are disclosed, update of sponsorship). Principal indicators were applied independently by two reviewers to the patient and consumer groups' websites. Discordances were solved by discussion. One hundred fifty-seven Italian patient and consumer groups and 17 drug companies were considered. Thirteen drug companies (76%) named at least one group funded, on their Italian websites. Of these, four (31%) indicated the activities sponsored and two (15%) the amount of funding. Of the 157 patient and consumer groups, 46 (29%) named at least one pharmaceutical company as providing funds. Three (6%) reported the amount of funding, 25 (54%) the activities funded, none the proportion of income derived from drug companies. Among the groups naming pharmaceutical company sponsors, 15 (33%) declared them in a dedicated section, five (11%) on the home page, the others in the financial report or other sections. CONCLUSIONS/SIGNIFICANCE: Disclosure of funds is scarce on Italian patient and consumer groups' websites. The levels of transparency need to be improved. Disclosure of patient and consumer groups provided with funds is frequent on Italian pharmaceutical companies' websites, but information are often not complete. | Humans | Patient Participation | Patient Satisfaction | Internet | OK |
| C. | Taft | L.-O. | Person |  |  | Spinal Cord | 2013-11-00 Nov 2013 | STUDY DESIGN: Qualitative method, semi-structured interviews. OBJECTIVES: The aim of the study was to explore the meaning of patient participation in care and rehabilitation from the perspective of patients with spinal cord injury (SCI). SETTING: Post discharge community setting. METHODS: Semi-structured interviews were performed with 10 persons with SCI representing different ages, gender and levels of injury. All interviews were conducted individually and lasted 40-120 min. The interviews were verbally transcribed and the data were analyzed by means of content analysis. RESULTS: All informants stressed the importance of patient participation as a necessary prerequisite for successful care and rehabilitation, but emphasized that participation must be tailored to each patient's own preferences, capacities and needs. They also underscored that the staff should be sensitive and responsive to the fact that desired levels and kinds of participation may vary from patient to patient, as well as for the same patient during the course of the rehabilitation. Five themes reflecting central aspects of participation emerged: respect and integrity, planning and decision-making, information and knowledge, motivation and encouragement, and involvement of family. CONCLUSIONS: Patient participation is a critical component of successful SCI rehabilitation and must be facilitated, promoted and tailored to each patient by the staff. Based on the finding from this study a questionnaire has been developed for assessing patient experiences of five domains of participation in rehabilitation to serve as a tool to help in evaluating provided care and in identifying patients' preferences for participation. | Male | Decision Making | Humans | Patient Participation | Doublon |
| C. | Taft | L.-O. | Person |  |  | Spinal Cord | 2013-11-00 Nov 2013 | STUDY DESIGN: Qualitative method, semi-structured interviews. OBJECTIVES: The aim of the study was to explore the meaning of patient participation in care and rehabilitation from the perspective of patients with spinal cord injury (SCI). SETTING: Post discharge community setting. METHODS: Semi-structured interviews were performed with 10 persons with SCI representing different ages, gender and levels of injury. All interviews were conducted individually and lasted 40-120 min. The interviews were verbally transcribed and the data were analyzed by means of content analysis. RESULTS: All informants stressed the importance of patient participation as a necessary prerequisite for successful care and rehabilitation, but emphasized that participation must be tailored to each patient's own preferences, capacities and needs. They also underscored that the staff should be sensitive and responsive to the fact that desired levels and kinds of participation may vary from patient to patient, as well as for the same patient during the course of the rehabilitation. Five themes reflecting central aspects of participation emerged: respect and integrity, planning and decision-making, information and knowledge, motivation and encouragement, and involvement of family. CONCLUSIONS: Patient participation is a critical component of successful SCI rehabilitation and must be facilitated, promoted and tailored to each patient by the staff. Based on the finding from this study a questionnaire has been developed for assessing patient experiences of five domains of participation in rehabilitation to serve as a tool to help in evaluating provided care and in identifying patients' preferences for participation. | Male | Decision Making | Humans | Patient Participation | OK |
| Sarah | Jorgenson | John | Coffey | Nandita | Anand | Health Expectations: An International Journal of Public Participation in Health Care and Health Policy | 2014-03-11 Mar 11, 2014 | BACKGROUND: There is evidence suggesting that active participation of patients in their health care can improve the quality of care and decrease health-care costs. Further, patient reports of their health-care experience are increasingly used to monitor health-care quality. OBJECTIVE: This paper describes a systematic review of peer-reviewed studies to identify measures of patients' active participation in their encounters with health-care providers. METHODS: A systematic literature review was conducted for publications indexed from 1975 to 2011. Of interest were self-reported measures of patient participation that were not limited to a specific health concern. All abstracts were reviewed independently by two authors, and the full paper was considered for those meeting inclusion criteria. MAIN RESULTS: From a review of 4528 citations, ten measures were identified. The approaches to development of the measures varied considerably, as did their study samples and their psychometric quality. DISCUSSION: These measures represented three conceptual frameworks: empowerment and self-efficacy, therapeutic alliance, and consumerism/satisfaction. They provide a more comprehensive perspective of patients' experiences of their provider encounters, and a better understanding patient behaviour enhanced the quality of health-care delivery or improved health outcomes. These measures underscore the continuing challenge of defining patient participation and the multiple theoretical approaches that underlie this form of patient behaviour. CONCLUSIONS: Current interest in quality-related physician report cards gives significant weight to patients' self-reported experiences as one dimension of physician performance. It is critical to identify the specific focus and quality of measures selected for this and research purposes. | | | | | OK |
| Erik | Taal | Harald E. | Vonkeman | Mart A. F. J. | van de Laar | BMC musculoskeletal disorders | 2014-00-00 2014 | BACKGROUND: Involvement of patients in decision-making about medication is currently being advocated. This study examined (the concordance between) inflammatory arthritis patients' preferred and perceived involvement in decision-making in general, and in four specific decisions about Disease-Modifying Anti-Rheumatic Drugs (DMARDs). Furthermore, this study examined how patients' involvement is related to satisfaction about decision-making and which factors are related to preferred roles, perceived roles and concordance. METHODS: Using a cross-sectional survey, 894 patients diagnosed with Rheumatoid Arthritis, Psoriatic Arthritis or Ankylosing Spondylitis were sent a questionnaire which focused on medical decisions in general and on four specific decisions: (a) starting with a traditional DMARD; (b) starting to inject methotrexate; (c) starting a biological DMARD; and (d) decreasing or stopping a DMARD. For each decision preferred and perceived involvement in decision-making was assessed using the Control Preference Scale. Concordance was calculated by subtracting the scores for perceived role from scores for the preferred role. Furthermore, satisfaction with the decision process and socio-demographic, health-related, patient-related and physician-related variables were assessed. RESULTS: The response rate was 58%. For all decisions, most patients (59%-63%) preferred Shared Decision-Making (SDM). SDM was perceived frequently (26%-55%) and patients' preferences were met in 54% of the respondents. Yet, in some specific decisions, 26% to 54% of patients would have liked more participation. Perceiving less participation then preferred was associated with less satisfaction with the decision-process, but perceiving more participation than preferred was not. Our results did not reveal any meaningful models to predict preferred or perceived participation in decision-making in general or with reference to specific decisions about DMARDs. CONCLUSIONS: Most arthritis patients prefer to be involved in decisions about their medication and SDM is perceived frequently. Yet, in some specific decisions patient participation can be further improved. Patients especially prefer more participation in decision-making regarding starting a first traditional DMARD, which occurs most commonly in newly diagnosed patients. Whereas perceiving too little participation was associated with decreased satisfaction, perceiving too much participation was not. Therefore, rheumatologists should urge patients to participate in every medical decision. | | | | | OK |
|  |  |  |  |  |  | Tidsskrift for Den Norske Lægeforening: Tidsskrift for Praktisk Medicin, Ny Række | 2011-01-21 Jan 21, 2011 | BACKGROUND: Users of the Norwegian health services have a legal right to influence the services through user committees, and also to assess the treatment they are being subject to on an individual basis. The aim of this study was to investigate the experience health personnel have with patient participation in hospital wards. MATERIAL AND METHODS: Qualitative interview study with 18 health workers from 12 hospital wards in Central Norway Regional Health Authority. RESULTS: Health personnel stated that they took the initiative to patient participation in the hospital ward. User representatives were either appointed by the user committee or chosen because of personal characteristics that suited the ward's needs. Health personnel were positively surprised by the user representatives' qualifications. It was still a challenge to achieve equality between health personnel and user representatives. Health personnel limited patient participation, especially regarding medical issues. INTERPRETATION: Patient participation in hospital wards seems to be regulated by health personnel, who initiate and limit such participation. | Decision Making | Humans | Patient Participation | Questionnaires | OK |
| L.-O. | Person | C. | Taft |  |  | Spinal Cord | 2013-11-00 Nov 2013 | STUDY DESIGN: A cross-sectional postal questionnaire study. OBJECTIVES: The aim of the study was to evaluate selected psychometric properties of a draft version of the Patient Participation in Rehabilitation Questionnaire (PPRQ) measuring patients' experiences of participation in care and rehabilitation. SETTING: Sweden. METHODS: On the basis of previous qualitative analyses of patient interview data, a 32-item questionnaire covering five domains of participation was developed and sent to 268 persons with spinal cord injury, aged 18-80 years and injured 1-12 years previously. In total, 141 (51%) evaluable questionnaires were returned. Multi-trait analysis was used to assess scaling assumptions by testing item convergent and discriminant validity and internal consistency reliability (Cronbach's α) associated with the hypothesized item-scale structure of the questionnaire. RESULT: Nine items failed to meet scaling assumptions and were omitted. Scaling assumptions were thereafter substantiated for the scales: 'respect and integrity' (6 items); 'planning and decision-making' (4 items); 'information and knowledge' (4 items); 'motivation and encouragement' (5 items); and 'involvement of family' (4 items). Item-scale correlations ranged from 0.67 to 0.85 and most items correlated higher or significantly higher with their hypothesized scale than with other scales. Cronbach's α was 0.89 for all scales. CONCLUSION: The PPRQ appears to adequately assess central aspects of participation in care and rehabilitation from the perspective of patients with spinal cord injury. Further studies using larger samples will be undertaken to confirm the scale structure as well as the sensitivity and responsiveness of the questionnaire. | Male | Humans | Patient Participation | Questionnaires | Doublon |
| L.-O. | Person | C. | Taft |  |  | Spinal Cord | 2013-11-00 Nov 2013 | STUDY DESIGN: A cross-sectional postal questionnaire study. OBJECTIVES: The aim of the study was to evaluate selected psychometric properties of a draft version of the Patient Participation in Rehabilitation Questionnaire (PPRQ) measuring patients' experiences of participation in care and rehabilitation. SETTING: Sweden. METHODS: On the basis of previous qualitative analyses of patient interview data, a 32-item questionnaire covering five domains of participation was developed and sent to 268 persons with spinal cord injury, aged 18-80 years and injured 1-12 years previously. In total, 141 (51%) evaluable questionnaires were returned. Multi-trait analysis was used to assess scaling assumptions by testing item convergent and discriminant validity and internal consistency reliability (Cronbach's α) associated with the hypothesized item-scale structure of the questionnaire. RESULT: Nine items failed to meet scaling assumptions and were omitted. Scaling assumptions were thereafter substantiated for the scales: 'respect and integrity' (6 items); 'planning and decision-making' (4 items); 'information and knowledge' (4 items); 'motivation and encouragement' (5 items); and 'involvement of family' (4 items). Item-scale correlations ranged from 0.67 to 0.85 and most items correlated higher or significantly higher with their hypothesized scale than with other scales. Cronbach's α was 0.89 for all scales. CONCLUSION: The PPRQ appears to adequately assess central aspects of participation in care and rehabilitation from the perspective of patients with spinal cord injury. Further studies using larger samples will be undertaken to confirm the scale structure as well as the sensitivity and responsiveness of the questionnaire. | Male | Humans | Patient Participation | Questionnaires | OK |
| Michael | Walsh | Nirmal C. | Tejwani |  |  | Journal of Patient Safety | 2010-12-00 Dec 2010 | INTRODUCTION: Wrong-site surgery is defined as an operation conducted at the wrong site, on the wrong person, or resulting in the wrong procedure. Since 1993, more than 2200 wrong-site surgeries have been reported by the National Practitioner Data Base. A 2005 survey reported, 5.6% of replying academy fellows encountered a wrong-site surgery incident. Multiple interventions have been since suggested for prevention of these occurrences by the American Academy of Orthopaedic Surgeons and Joint Commission on Accreditation of Healthcare Organizations. MATERIALS AND METHODS: This institutional review board-approved study was conducted to investigate patient compliance and reliability in marking the surgical site. Two hundred patients undergoing orthopedic surgery who agreed to participate were enrolled at the time of preoperative testing and clearance for surgery. The patients received instructions to mark the surgical site with a "YES." Patient data collected included age, sex, procedure type and location, history of previous orthopedic surgeries, workmen's compensation status or motor vehicle collision involvement, employment status, primary language, level of education, presence of depression or toxic habits, and the time between enrollment and day of surgery. RESULTS: We achieved an overall compliance rate of 68.2%. There was no difference with respect to sex, tobacco use or history of depression, level of education or occupation, workmen's compensation, or government insurance status. The mean age of compliant patients was 48.6 years versus 53.3 years for those who did not comply (P = 0.05). About 72% of those who spoke English as a primary language complied, as opposed to 49% in others (P = 0.009). The time between enrollment and surgery was 10.4 days in compliant patients versus 23.1 days in noncompliant patients (P = 0.05). No statistically significant difference was noted with all other variables recorded. In no instance did patients mark the wrong side or make any marks likely to contribute to the wrong operation. CONCLUSIONS: Patient's ability to be involved in this preventative measure is independent of most demographic variables previously thought to be significant. However, in view of the 68.2% compliance, patient involvement in surgical site marking is unreliable and may not help in decreasing the chances of wrong-site surgery. | Male | Humans | Patient Participation | Physician-Patient Relations | OK |
| Aslaug | Mikkelsen | Kjell | Hausken |  |  | Nursing Research and Practice | 2012-00-00 2012 | The aim of this paper is to describe the development of a new, brief, easy-to-administer self-reported instrument designed to assess patient participation in decision making in surgical treatment. We describe item generation, psychometric testing, and validity of the instrument. The final scale consisted of four factors: information dissemination (5 items), formulation of options (4 items), integration of information (4 items), and control (3 items). The analysis demonstrated a reasonable level of construct validity and reliability. The instrument applies to patients in surgical wards and can be used to identify the health services that are being provided and the areas that could strengthen patient participation. | | | | | OK |
|  |  |  |  |  |  | Evidence-Based Nursing | 2015-01-00 Jan 2015 | |  |  |  |  | OK |
| Marie | Desmartis | France | Legare | Florence | Piron | Santé Publique (Vandoeuvre-Lès-Nancy, France) | 2014-04-00 2014 Mar-Apr | AIM: To explore the perceptions of members of a working group of caregivers, health technology assessment (HTA) units members, healthcare facilities managers and patients representatives regarding the participation of the latter in the assessment of alternatives to restraint and seclusion among adults in short-term psychiatric wards and in long-term care facilities for the elderly, in Quebec. METHODS: A descriptive design was used for this research based on fifteen semi-structured interviews with caregivers, healthcare managers, patient representatives and HTA unit members. The direct observation of two meetings of the working group and informal discussions with participants at these meetings was also performed. Data analysis was based on a framework developed to assess patients' involvement in HTA. RESULTS: Information resources and human resources were mobilized to support the participation of patients' representatives. Nevertheless, this participation was uneven between patients' representatives due to their personal characteristics, the understanding of their role in this working group and expectations of the committee. The presence of patient representatives led caregivers and managers to focus the assessment on patients' concerns and to adjust the language used during the meeting. Moreover, organizational and political context has been identified as a facilitator for this participation. CONCLUSION: Involving patients' representatives in HTA activities is thus possible. However, some resources must be mobilized to equip patients' representatives who participate in these activities and personal characteristics should be considered when selecting them to favour their involvement | Humans | Patient Participation | Attitude of Health Personnel | Patient Isolation | OK |
| Amir | Baigi | Margareta | Asp |  |  | Journal of Advanced Nursing | 2011-04-00 avril 2011 | Aim: This study aimed at evaluating patient participation from the perspective of patients who received care in emergency departments, with a separate examination of the relationship between participation and age, gender, education and priority level. Background: International and national guidelines encourage patient participation. High patient participation is required to ensure a high quality of care. No studies evaluating patient participation at an emergency department have been published. Methods: An evaluating study, with the Patient Participation Emergency Department questionnaire, was conducted at emergency departments in Sweden. A consecutive sample of 356 patients participated. Data were collected in 2008: participants were 49% women and with an average age of 56 years. The statistical methods used were Student’s t-test, one-way ANOVA and Spearman correlation. Results: The results revealed that patients experienced good requirement for participation such as time and information. Mutual participation demonstrated a reasonable level, but patient participation is low in two dimensions (Fight for participation, Participation in getting basic needs satisfied). Young and well-educated patients fought more to participate in their care and gained less attention for basic needs than older and less well-educated patients. Conclusions: Patient participation in a mutual care situation between patients and healthcare professionals requires further improvement to ensure that patients are satisfied and do not have to struggle and fight to participate in their care. (PsycINFO Database Record (c) 2012 APA, all rights reserved). (journal abstract) | Patient Participation | Client Participation | emergency departments | hospital programs | Doublon |
| Amir | Baigi | Margareta | Asp |  |  | Journal of Advanced Nursing | 2011-04-00 Apr 2011 | AIM: This study aimed at evaluating patient participation from the perspective of patients who received care in emergency departments, with a separate examination of the relationship between participation and age, gender, education and priority level. BACKGROUND: International and national guidelines encourage patient participation. High patient participation is required to ensure a high quality of care. No studies evaluating patient participation at an emergency department have been published. METHODS: An evaluating study, with the Patient Participation Emergency Department questionnaire, was conducted at emergency departments in Sweden. A consecutive sample of 356 patients participated. Data were collected in 2008: participants were 49% women and with an average age of 56 years. The statistical methods used were Student's t-test, one-way ANOVA and Spearman correlation. RESULTS: The results revealed that patients experienced good requirement for participation such as time and information. Mutual participation demonstrated a reasonable level, but patient participation is low in two dimensions (Fight for participation, Participation in getting basic needs satisfied). Young and well-educated patients fought more to participate in their care and gained less attention for basic needs than older and less well-educated patients. CONCLUSIONS: Patient participation in a mutual care situation between patients and healthcare professionals requires further improvement to ensure that patients are satisfied and do not have to struggle and fight to participate in their care. | Male | Humans | Patient Participation | Questionnaires | OK |
| Zhongze | Li | Mark | Hegel | Tim A. | Ahles | Palliative Medicine | 2013-04-00 Apr 2013 | BACKGROUND: ENABLE (Educate, Nurture, Advise Before Life Ends) II was one of the first randomized controlled trials (RCTs) examining the effects of a concurrent oncology palliative care intervention on quality of life, mood, and symptom control for advanced cancer patients and their caregivers. However, little is known about how participants experience early palliative care and the benefits and burdens of participating in a palliative care clinical trial. AIM: To gain a deeper understanding of participants' perspectives of the intervention and palliative care trial participation. DESIGN: A qualitative descriptive study using thematic analysis to determine benefits and burdens of a new palliative care intervention and trial participation. SETTING/PARTICIPANTS: Of the 72 participants who were alive when the study commenced, 53 agreed to complete an in-depth, semi-structured interview regarding the ENABLE II intervention and clinical trial participation. RESULTS: Participants' perceptions of intervention benefits were represented by four themes: enhanced problem-solving skills, better coping, feeling empowered, and feeling supported or reassured. Three themes related to trial participation: helping future patients and contributing to science, gaining insight through completion of questionnaires, and trial/intervention aspects to improve. CONCLUSIONS: The benefits of the intervention and the positive aspects of trial participation outweighed trial "burdens". This study raises additional important questions relevant to future trial design and intervention development: when should a palliative care intervention be initiated and what aspects of self-care and healthy living should be offered in addition to palliative content for advanced cancer patients when they are feeling well? | Male | Humans | Patient Participation | Questionnaires | OK |
| Martin | Härter |  |  |  |  | BMC health services research | 2013-00-00 2013 | BACKGROUND: A comprehensive model of the relationships among different shared decision-making related constructs and their effects on patient-relevant outcomes is largely missing. Objective of our study was the development of a model linking decision-making in medical encounters to an intermediate and a long-term endpoint. The following hypotheses were tested: physicians are more likely to involve patients who have a preference for participation and are willing to take responsibility in the medical decision-making process, increased patient involvement decreases decisional conflict, and lower decisional conflict favourably influences patient satisfaction with the physician. METHODS: This model was tested in a German primary care sample (N = 1,913). Psychometrically tested instruments were administered to assess the following: patients' preference for being involved in medical decision-making, patients' experienced involvement in medical decision-making, decisional conflict, and satisfaction with the primary care provider. Structural equation modelling was used to explore multiple associations. The model was tested and adjusted in a development sub-sample and cross-validated in a confirmatory sample. Demographic and clinical characteristics were accounted for as possible confounders. RESULTS: Local and global indexes suggested an acceptable fit between the theoretical model and the data. Increased patient involvement was strongly associated with decreased decisional conflict (standardised regression coefficient Β = -.73). Both high experienced involvement (Β = .34) and low decisional conflict (B = -.28) predicted higher satisfaction with the physician. Patients' preference for involvement was negatively associated with the experienced involvement (B = -.24). CONCLUSION: Altogether, our model could be largely corroborated by the collected empirical data except the unexpected negative association between preference for involvement and experienced involvement. Future research on the associations among different SDM-related constructs should incorporate longitudinal studies in order to strengthen the hypothesis of causal associations. | Male | Decision Making | Humans | Patient Participation | OK |
| Gale | Harding | Seema | Haider | Margaret | Tawadrous | Health and Quality of Life Outcomes | 2013-00-00 2013 | BACKGROUND: Patient-reported outcomes (PROs) may provide valuable information to clinicians and patients when choosing initial antiretroviral therapy. OBJECTIVE: To identify and classify PRO instruments used to measure treatment effects in clinical trials evaluating NNRTIs. METHODS: We conducted a structured literature review using PubMed to identify NNRTI trials published from March 2003 to February 2013. Studies identified--based on disease, instrument, PRO, and NNRTI medication terms were reviewed--to identify PRO instruments. Domains measured within each instrument were recorded to understand key areas of interest in NNRTIs. RESULTS: Of 189 articles reviewed, 27 validated instruments were administered in 26 unique trials, with a mean of 1.9 instruments (median: 1; range: 1-7) per trial. The Medical Outcomes Study HIV Health Survey (MOS-HIV) was the most commonly used instrument (n = 8 trials). Seventeen trials (65%) included at least one multidimensional health-related quality of life (HRQL) instrument (HIV-targeted, n = 11; general, n = 8). Other validated instruments measured sleep (n = 5), depression (n = 5), anxiety (n = 4), psychiatric symptoms (n = 2), beliefs about HIV medications (n = 2), HIV symptoms (n = 1), and stress (n = 1). CONCLUSIONS: Although review of recent NNRTI trials suggests a lack of consensus on the optimal PRO instruments, a typical battery is comprised of a multidimensional HRQL measure coupled with one or more symptom measures. Further work is needed to clarify advantages and disadvantages of using specific PRO instruments to measure relevant constructs and to identify the most useful batteries of instruments for NNRTI trials. | Male | Humans | Patient Participation | Questionnaires | OK |
|  |  |  |  |  |  | Reumatología Clinica | 2012-06-00 2012 May-Jun | | Humans | Patient Participation | Physician-Patient Relations | Patient-Centered Care | OK |
| E. | Hohmann | F. | Verheyen | C. | Kranich | Gesundheitswesen (Bundesverband Der Ärzte Des Öffentlichen Gesundheitsdienstes (Germany)) | 2014-11-00 Nov 2014 | During the last decades patient and public involvement has gained more and more political approval. An important milestone regarding collective patient interests was the Statutory Health Insurance Modernisation Act in 2004 (§ 140 f SGB V). No systematic research has been conducted regarding the situation of patient representatives at the federal state level as yet. The aim of the following study is to close this gap, focusing on educational needs of patient representatives.After screening the literature we identified and discussed relevant aspects of patient participation in a focus group of 7 patient representatives. Based on these findings a standardised questionnaire was developed in coopera-tion with scientists and patient representatives. We sent the written questionnaire to 504 patient representatives at the federal state level. The survey was targeted at patient representatives who currently participate or have in the past participated in a committee at federal state level according to § 140 f SGB V.A total of 184 completed questionnaires was returned for an overall response rate of 36.5%. Most respondents felt poorly prepared for their role and regard a compulsory prepara-tion course as necessary. Patient representatives want to be an active part in the decision-making process of the committee meetings. They report educational needs on different topics and they are willing to close these gaps. For the future, patient representatives expect increasing significance of collective participation.The results can be used to develop educational programmes for patient representatives in order to qualify them for the best possible performance of their tasks. Furthermore the results can provide a scientific basis for political discussions and for reasoning capacity building of patient representatives. | | | | | OK |
| Johannes | van Delden |  |  |  |  | British Journal of Clinical Pharmacology | 2014-12-00 Dec 2014 | In the European Medicines Agency (EMA), the involvement of patients has been increasingly recognized as valuable and necessary. Specifically in scientific committees, patients through patient representatives are actively involved in deliberations and decision making processes. These scientific committees are meant to ensure that licensed medicines have a positive benefit-risk ratio in favour of the patients and users. To investigate what the contributions are of patient representatives in benefit-risk assessment, we interviewed 15 scientific committee members, 10 of whom are/were EU-state regulatory representatives and five are/were patient representatives. We asked the participants questions related to the benefit-risk assessment tasks of their committees, the connection between patient representatives and the patient perspective, and the contribution of patient representatives in the various benefit-risk assessments tasks. We found that the contribution of patient representatives benefit-risk assessment may be a variable of the benefits and the risks involved in the drug such that the necessity of their contribution is strongly felt when both benefits and risks are high, when benefits are almost equal or are equal to risks and when both benefits and risks are low. In terms of the various benefit-risk tasks, patient representatives contribute to benefit-risk analysis by providing criteria that help define the benefit-risk picture. In benefit-risk evaluation, patient representatives aid in providing a specific basis for the values and weights given to specific benefits and risks and in decision making, they provide what may be a crucial patient perspective in terms of the acceptability of risks. Hence, patient representatives provide a specific expertise in these scientific committees. | | | | | OK |
| Núria | Siñol | Juan | del Pozo | Maria J. | Portella | Drug and Alcohol Dependence | 2012-06-01 Jun 1, 2012 | BACKGROUND: Patients' satisfaction with methadone maintenance treatment (MMT) is a key measure of treatment quality. The main objective of the present study is to identify independent factors that contribute significantly to satisfaction with MMT. METHOD: Participants were a representative sample of methadone-maintained patients (n=123) from the region of La Rioja. Satisfaction with MMT was assessed with the Verona Service Satisfaction Scale for Methadone Treatment (VSSS-MT), and mental health status with the General Health Questionnaire-28 (GHQ-28). Multivariate linear- and logistic-regression analyses were performed to identify variables independently associated with satisfaction with MMT. RESULTS: Multiple linear regression analysis revealed that the variables independently associated with VSSS-MT total score were number of hours per week that the centre dispensed methadone (β=0.193), number of patients per centre (β=0.233), perceived frequency of receiving information about methadone dose changes (β=0.246), perceived influence on these changes (β=0.194), and Social Dysfunction subscale of GHQ-28 (β=-0.179). Multivariate binary logistic regression showed that the variables independently associated with the likelihood of being satisfied with MMT were number of years of education completed (OR=0.835), number of patients per centre (OR=1.009), perceived frequency of receiving information about methadone dose changes (OR=1.571), and Social Dysfunction subscale of GHQ-28 (OR=0.748). CONCLUSIONS: Patients from larger centres, who perceive themselves as participating to some extent in treatment decisions, and showing lower deterioration in social functioning are more likely to be satisfied with MMT. | Male | Humans | Patient Participation | Patient Satisfaction | OK |
| Amanda J. | Lee | Margaret C. | Watson | Philip C. | Hannaford | Pharmacoepidemiology and Drug Safety | 2011-05-00 May 2011 | PURPOSE: To describe the characteristics of patient reporters to the UK's Yellow Card Scheme (YCS) and the suspect drugs reported, and to determine patient views and experiences of making a Yellow Card report. METHODS: A questionnaire was developed for distribution by the Medicines and Healthcare products Regulatory Agency (MHRA) to all patients reporting through the YCS between March 2008 and January 2009. Associations between reporting method (online, postal and telephone) and questionnaire responses were examined using Pearson's Chi-squared test. RESULTS: Evaluable questionnaires were returned by 1362 out of 2008 reporters (68%). Respondents' median (IQR) age was 56.5 (43.0, 67.0) years, 910 (66.8%) were female, 1274 (93.5%) were white and 923 (67.8%) had at least a further education qualification. The most frequent reporting method was postal (59.8%), followed by online (32.8%) and telephone (6.3%). Online reporters were younger with a higher education level than those using other reporting methods. Most respondents, 1274 (93.6%), thought that the report was fairly or very easy to complete, although many identified the need for improvements to the system. One third (n = 448; 32.9%) expected feedback from the MHRA and 828 (60.8%) would have liked feedback. Almost all respondents (n = 1302; 95.6%) would report again. CONCLUSIONS: The majority of patients found the current methods of reporting suspected ADRs easy to use and would recommend them to others. Different methods of reporting were used by different demographic subgroups of reporters. Improvements to the system, including the provision of feedback to reporters, could be made. | Male | Humans | Patient Participation | Questionnaires | OK |
| Anna | Prenestini |  |  |  |  | Studies in Health Technology and Informatics | 2010-00-00 2010 | The arrival of the Internet contributes to the growth of new areas for patient empowerment. In the presence of a challenge such as this, we nevertheless note that the adequacy and characteristics of the web strategies of healthcare providers have, up to the present, not been subjected to thoroughgoing critical analysis. The aim of this paper is to: (a) provide an analysis of the key factors of an efficient web strategy for healthcare organizations with regard to the issue of patient empowerment (b) build a concise indicator for measuring the degree of empowerment potential of healthcare providers' web sites (Patient Web Empowerment Index -PWEI-). PWEI was calculated in order to assess the web sites of 340 Italian National Health Service healthcare organizations, the aim being the appraisal of the current degree of maturity of their web strategies in relation to potentials for an effective increase in patient empowerment. | Humans | Patient Participation | Internet | Health Education | OK |
| Arpana R. | Vidyarthi | Mary A. | Blegen | Robert M. | Wachter | Journal of Hospital Medicine: An Official Publication of the Society of Hospital Medicine | 2010-04-00 Apr 2010 | BACKGROUND: Patient whiteboards can serve as a communication tool between hospital providers and as a mechanism to engage patients in their care, but little is known about their current use or best practices. METHODS: We surveyed bedside nurses, internal medicine housestaff, and hospitalists from the medical service at the University of California, San Francisco. A brief survey about self-reported whiteboard practices and their impact on patient care was administered via paper and a commercial online survey tool. RESULTS: Surveys were collected from 104 nurse respondents (81% response rate), 118 internal medicine housestaff (74% response rate), and 31 hospitalists (86% response rate). Nurses were far more likely to use and read whiteboards than physicians. While all respondents highly valued the utility of family contact information on whiteboards, nurses valued the importance of a "goal for the day" and an "anticipated discharge date" more than physicians. Most respondents believed that nurses should be responsible for accurate and updated information on whiteboards, that goals for the day should be created by a nurse and physician together, and that unavailability of pens was the greatest barrier to use. DISCUSSION: Despite differences in practice patterns of nurses and physicians in using whiteboards, our findings suggest that all providers value their potential as a tool to improve teamwork, communication, and patient care. Successful adoption of whiteboard use may be enhanced through strategies that emphasize a patient-centered focus while also addressing important barriers to use. | Humans | Patient Participation | Health Care Surveys | Physician's Practice Patterns | OK |
| Michela | Moraldo | Judith | Finegold | Khalil | Hassanally | Circulation | 2014-06-17 Jun 17, 2014 | BACKGROUND: Primary prevention guidelines focus on risk, often assuming negligible aversion to medication, yet most patients discontinue primary prevention statins within 3 years. We quantify real-world distribution of medication disutility and separately calculate the average utilities for a range of risk strata. METHOD AND RESULTS: We randomly sampled 360 members of the general public in London. Medication aversion was quantified as the gain in lifespan required by each individual to offset the inconvenience (disutility) of taking an idealized daily preventative tablet. In parallel, we constructed tables of expected gain in lifespan (utility) from initiating statin therapy for each age group, sex, and cardiovascular risk profile in the population. This allowed comparison of the widths of the distributions of medication disutility and of group-average expectation of longevity gain. Observed medication disutility ranged from 1 day to >10 years of life being required by subjects (median, 6 months; interquartile range, 1-36 months) to make daily preventative therapy worthwhile. Average expected longevity benefit from statins at ages ≥50 years ranges from 3.6 months (low-risk women) to 24.3 months (high-risk men). CONCLUSION: We can no longer assume that medication disutility is almost zero. Over one-quarter of subjects had disutility exceeding the group-average longevity gain from statins expected even for the highest-risk (ie, highest-gain) group. Future primary prevention studies might explore medication disutility in larger populations. Patients may differ more in disutility than in prospectively definable utility (which provides only group-average estimates). Consultations could be enriched by assessing disutility and exploring its reasons. | Male | Decision Making | Humans | Patient Participation | OK |
| Barbara | Basberg | Swaytha | Yalamanchi | Kimberly | Baker-Genaw | Quality Management in Health Care | 2011-06-00 2011 Apr-Jun | PURPOSE: In developing our Patient-Centered Team Care (PCTC) program, we designed a Plan-of-Care (POC) tool to facilitate the physician-patient discussion for setting health goals. This study aimed to determine the effectiveness of the POC tool in improving clinical outcomes. METHODS: We compared baseline and 6-month or greater follow-up values for hemoglobin A1c (HbA1c), low-density lipoprotein (LDL), systolic blood pressure (SP), diastolic blood pressure (DP), and weight for PCTC patients (intervention group) and non-PCTC patients (control group). For the intervention group, we also compared POC tool completeness scores (1, low and 8, high). RESULTS: Of 1366 patients, 1110 (593 intervention, 517 control) had baseline and follow-up clinical measures for analysis. After adjusting for demographics, significant effects occurred in the intervention group for HbA1c (P = .0067), LDL (P = .012), and DP (P = .091). For completeness of POC, a significant association occurred between more fully completed forms (scores, 5-8) and change in HbA1c (P < .001) and SP (P = .011). CONCLUSION: Patients receiving a POC showed significant improvement in 3 of 5 clinical outcomes compared with those without the tool, and those with more fully completed forms had significant improvement in 2 of 5 clinical outcomes compared with those with partially completed forms. | Male | Animals | Humans | Patient Participation | OK |
| Ilme | Aro | Ly | Kalam-Salminen | Pirkko | Routasalo | International Journal of Older People Nursing | 2012-12-00 Dec 2012 | AIM: The aim of this study was to describe assessments of older people about patient-centeredness in the long-term care of Estonian hospitals, and to determine correlations between patients' assessments and their socio-demographic characters. BACKGROUND: Patient-centeredness increases patients' satisfaction and enhances their recovery. Still, patients' opinions are not always taken into account. METHOD: A cross-sectional study included 111 older people in long-term care of 14 Estonian hospitals. Data were collected in 2008 by means of structured interviews. RESULTS: What patients agreed most was that they were given enough opportunity to carry out activities they were capable of performing themselves. Two thirds of participants had not been sufficiently consulted on who would be providing their care. Half of the participants were not given sufficient opportunity to decide what kind of care was needed and how they would receive it. The more assistance patients needed in daily living activities, the less they considered the care as patient-centred. CONCLUSIONS: From the perspective of older people, the patient-centeredness in Estonian long-term care is above average. Providing patient-centred long-term care in Estonian hospitals deserves more attention. RELEVANCE TO CLINICAL PRACTICE: Older people should be more involved in decision-making, especially those who need more assistance in daily living activities. | Male | Humans | Patient Participation | Patient Satisfaction | OK |
| Haoling H. | Weng | Yun | Chon | Xing-Yue | Huang | The Journal of Rheumatology | 2010-05-00 May 2010 | OBJECTIVE: To investigate the correlation between the Disease Activity Score using a 28-joint count (DAS28) based on physician-derived joint counts and the DAS28 based on patient-derived joint counts (Pt-DAS28) in rheumatoid arthritis (RA). METHODS: Data from a multicenter, open-label study investigating the immunogenicity of etanercept (ETN) were analyzed. ETN-naive patients with active RA received ETN 50 mg once weekly alone or with methotrexate (MTX). Joint counts were performed at baseline, Week 12, and Week 24 by the physician and patient independently. Patients received instruction in performing joint assessments. RESULTS: Of 447 patients enrolled (ETN, n = 218; ETN + MTX, n = 229), most were women (79%) and the mean age was 54.5 years. Correlation coefficients between DAS28 and Pt-DAS28 were > or = 0.57 at baseline, Week 12, and Week 24. At Week 24, 48%, 39%, and 12% of patients could be classified as having low, moderate, or high disease activity, respectively, using DAS28. Using Pt-DAS28, 43%, 39%, and 18% were similarly classified. Agreement in the category of disease activity classification occurred in 72% of patients (kappa = 0.55). At Week 24, 78% of patients using DAS28 and 72% of patients using Pt-DAS28 were classified as moderate or good European League Against Rheumatism responders. CONCLUSION: These results support the possible use of patient-derived tender and swollen joint counts to aid in the assessment of disease activity and clinical response in patients with RA. | Male | Humans | Patient Participation | Patient Satisfaction | OK |
| C. | Fleischer | B. | Greitemann |  |  | Die Rehabilitation | 2010-10-00 Oct 2010 | OBJECTIVES: A body of evidence suggests that good interaction is crucial for high-quality medical practice and that it has a considerable impact on treatment outcomes. Less is known about the role and significance of doctor-patient interaction in rehabilitation. The study's aim was to capture perceived quality of doctor-patient interaction in rehabilitation by a rating instrument (P.A.INT-Questionnaire) and to examine the relationship between perceived quality of interaction and long-term treatment outcomes. METHODS: Referring to the approach of Bensing (1990) and Rogers (1972) we defined "quality of interaction" in terms of 3 dimensions: (1) affective behaviour, i. e., empathy, positive regard and coherence; (2) instrumental behaviour: providing and collecting information, structuring and reinforcement; (3) participation and involvement of patients. 2 parallel versions of the Questionnaire were developed for patients and physicians. 7 rehabilitation clinics in north-western Germany participated in the multi-centre study. 61 doctors and their 470 patients evaluated both their shared dialogues upon admission, discharge and at least one ward round. Furthermore, patients rated their health status on admission (t0), discharge (t1) and 6 months after discharge (t2) using the IRES-3 (Indicators of Rehabilitation Status Questionnaire Version 3). RESULTS: (1) Comparisons of patient and physician evaluations on admission revealed the following: Affective quality of contact (empathy and coherence) was rated positively and without discrepancies by both patients and physicians. On the other hand, instrumental behaviour (information and structuring) was rated less positively by patients than by physicians. (2) Patients who rated the dialogue on admission more positively showed stronger treatment effects with respect to pain, to anxiety and depression 6 months after discharge. Also disability days decreased about 40% stronger in the group of positive raters. (3) Patient ratings of quality of interaction showed low but significant correlations with patient-related aspects like coping with disease, health-related knowledge and former positive experiences with physicians. A stepwise regression analysis revealed that interaction quality seems to contribute to enhanced treatment results independently of patients' competences. DISCUSSION: Our results suggest a positive relationship between perceived interaction quality as defined by the P.A.INT-Questionnaire and treatment effects 6 months after discharge. Comparisons of patient and physician evaluations showed that physicians seem to be successful in building relationships on the affective level but less successful on the instrumental level (i. e., information, structuring and reinforcement). Our data underline the importance of interaction quality for the success of rehabilitation and thus the importance of specific skills such as providing and collecting information, recognizing patients' concerns and goals as well as reinforcement of health-related action. This is especially important when knowledge of disease and coping with disease on patients' side is poor. Interaction quality seems to contribute to better treatment results independently of patients' competences. | Male | Humans | Patient Participation | Patient Satisfaction | OK |
| Morten | Fenger-Grøn | Peter | Vedsted |  |  | BMC family practice | 2013-00-00 2013 | BACKGROUND: People living with chronic disease currently account for the majority of the total healthcare costs. The Central Denmark Region implemented a disease management programme (DMP) for chronic obstructive pulmonary disease (COPD) in 2008. This presented an opportunity to examine the effect of an evidence-based, planned and proactive implementation of a DMP compared to the usual implementation strategy. METHODS: We performed a block- and cluster-randomised controlled trial with two groups and an extra external control group. The primary outcome was patients' assessment of their care after using an active implementation model for a DMP for COPD measured with the Patient-Assessment-of-Chronic-Illness-Care (PACIC) instrument. At baseline, questionnaires were sent to 2,895 patients identified by an algorithm based on health registry data on lung-related contacts to the healthcare system. Patients were asked to confirm or refute their diagnosis of COPD. Of those who responded, 1,445 (72.8%) confirmed their diagnosis. PACIC data were collected at baseline and at a 12-month follow-up for 744 (51.1%) patients. RESULTS: Comparing the three groups after the implementation of the DMP, we found a statistically significantly higher change in the PACIC score in the intervention group than in the control groups. No statistically significant differences were found between the control and the external control groups in any of the dimensions. CONCLUSIONS: Reinforcing the role of general practice as coordinator for care-and self-management-support with an active implementation of a DMP for COPD made patients score higher on the PACIC instrument, which indicates a better experience of the received healthcare. TRIAL REGISTRATION: NCT01228708. | Male | Humans | Patient Participation | Patient Satisfaction | OK |
| Beth | Elverdam | Bengt | Owall |  |  | The International Journal of Prosthodontics | 2010-10-00 2010 Sep-Oct | PURPOSE: The effect of impaired oral functions is best described by the patient, and a shift toward a patient-oriented decision-making process in oral rehabilitation is evident. The Oral Health Impact Profile-49 (OHIP-49) questionnaire has been the most commonly used method to measure oral health-related quality of life (OHRQoL) in dentistry. An individualized method, the Schedule for the Evaluation of Individual Quality of Life-Direct Weighing (SEIQoL-DW), has proven to fulfill most of the criteria for a method to assist in the decision-making process. The purpose of this study was to compare the ability of the OHIP-49 questionnaire and the SEIQoL-DW method in measuring OHRQoL and generating useful information for decision making in oral rehabilitation. MATERIALS AND METHODS: Sixty participants in need of oral rehabilitation were enrolled in the study. Patients received a clinical examination, were interviewed using the SEIQoL-DW, filled out the OHIP-49 questionnaire, and answered two global oral health-rating questions. RESULTS: The SEIQoL-DW generated additional information compared to the OHIP-49. The information was more oral- and treatment-specific, including consultation issues and the patient-practitioner relationship. The overall SEIQoL-DW and OHIP-49 scores were significantly correlated. The OHIP-49 and SEIQoL-DW scores were related to oral health subjectively. CONCLUSIONS: The SEIQoL-DW method proved a useful aid in clinical decision making for oral rehabilitation. The SEIQoL-DW was more appropriate for generating information useful for decision making than measuring OHRQoL; the OHIP-49 was more appropriate for measuring OHRQoL than generating information. | Male | Decision Support Techniques | Humans | Patient Participation | OK |
| Claudia | Appel |  |  |  |  | PPmP: Psychotherapie Psychosomatik Medizinische Psychologie | 2010-05-00 mai 2010 | Objectives: Patient involvement (PI) and shared decision making (SDM) have increasingly come into the focus of (inter-)national research. SDM, however, is not a standard component of standard care so far. Therefore it is of importance to take into consideration all parties concerned. This article points out the barriers physicians see; one group has not been interrogated on this topic often. Method: 25 physicians at a clinical centre were interviewed about decision-making processes within their environment, their concerns as well as their ideas towards PI. The consultations were realized with the help of a guideline-oriented questionnaire. The outcome was then interpreted by means of the thematic content analysis. Results: A system of categories was constructed containing 28 categories on the micro-, meso-, and macro-level. From a physician's perspective, changes are necessary on all levels to achieve patient involvement and SDM. Essential points of approach for this are: sufficient time and personnel, sufficient communicative abilities in physicians, and a solid physician-patient relationship as well as changes in healthcare politics. Conclusions: To consistently improve patient involvement and SDM, physicians' concerns must be taken seriously. Here SDM can only be properly applied by taking into account the meso- and macro-level. Furthermore, it would be helpful to clear up the conceptually vague aspects of SDM, which would also help to communicate the concept more clearly. (PsycINFO Database Record (c) 2013 APA, all rights reserved). (journal abstract) | Shared decision making | Patient involvement | Decision Making | physicians | Doublon |
| Pamela Jo | Johnson | Carol | Sakala |  |  | Health Services Research | 2014-09-23 Sep 23, 2014 | OBJECTIVE: To determine whether patient-perceived pressure from clinicians for labor induction or cesarean delivery is significantly associated with having these procedures. DATA SOURCES/STUDY SETTING: Listening to Mothers III, a nationally representative survey of women 18-45 years who delivered a singleton infant in a U.S. hospital July 2011-June 2012 (N = 2,400). STUDY DESIGN: Multivariate logistic regression analysis of factors associated with perceived pressure and estimation of odds of induction and cesarean given perceived pressure. PRINCIPAL FINDINGS: Overall, 14.8 percent of respondents perceived pressure from a clinician for labor induction and 13.3 percent for cesarean delivery. Women who perceived pressure for labor induction had higher odds of induction overall (adjusted odds ratio [aOR]: 3.51; 95 percent confidence interval [CI]: 2.5-5.0) and without medical reason (aOR: 2.13; 95 percent CI: 1.3-3.4) compared with women who did not perceive pressure. Those perceiving pressure for cesarean delivery had higher odds of cesarean overall (aOR: 5.17; 95 percent CI: 3.2-8.4), without medical reason (aOR: 6.13; 95 percent CI: 3.4-11.1), and unplanned cesarean (aOR: 6.70; 95 percent CI: 4.0-11.3). CONCLUSIONS: Patient-perceived pressure from clinicians significantly predicts labor induction and cesarean delivery. Efforts to reduce provider-patient miscommunication and minimize potentially unnecessary procedures may be warranted. | | | | | OK |
|  |  |  |  |  |  | Stroke; a Journal of Cerebral Circulation | 2013-04-00 Apr 2013 | BACKGROUND AND PURPOSE: Patient-reported outcome measures have been found useful in many disciplines but have received limited evaluation after stroke. The current study investigated the relationship that patient-reported measures have with standard impairment and disability scales after stroke. METHODS: Patients with motor deficits after stroke were scored on standard assessments including the National Institutes of Health Stroke Scale, modified Rankin Scale, and Fugl-Meyer motor scale, and on 2 patient-reported measures, the hand function domain of the Stroke Impact Scale, which documents difficulty of hand motor usage, and the amount of use portion of the Motor Activity Log, which records amount of arm motor usage. RESULTS: The 43 participants had mild disability (median modified Rankin Scale=2), moderate motor deficits (Fugl-Meyer motor scale=46 ± 22), and mild cognitive/language deficits. The 2 patient-reported outcome measures, Stroke Impact Scale and Motor Activity Log, were sensitive to the presence of arm motor deficits. Of 21 patients classified as having minimal or no impairment or disability by the National Institutes of Health Stroke Scale or modified Rankin Scale (score of 0-1), 15 (71%) reported difficulty with hand movements by the Stroke Impact Scale score or reduced arm use by the Motor Activity Log score. Furthermore, of 14 patients with a normal examination, 10 (71%) reported difficulty with hand movements or reduction in arm use. CONCLUSIONS: Patient-reported measures were a unique source of insight into clinical status in the current population. Motor deficits were revealed in a majority of patients classified by standard scales as having minimal or no disability, and in a majority of patients classified as having no deficits. | Male | Humans | Patient Participation | Female | OK |
| Philip | Seo | Peter A. | Merkel |  |  | Arthritis Care & Research | 2010-11-00 Nov 2010 | OBJECTIVE: To assess patients' self-estimates of the burden of disease in vasculitis and to compare data across patient populations from the US, Germany, and the UK. Outcome assessment in vasculitis primarily focuses on physician evaluations of disease activity and damage. Little is known about the patients' perspectives regarding the burden of disease. METHODS: Patients ranked (scale 0-5) a list of vasculitis-related items to estimate a combination of pain, interference with daily function, discomfort and/or annoyance, anxiety/psychological impact, and medical importance. Patients were also asked to list the 5 most troublesome aspects of the disease. RESULTS: Data from 264 patients with vasculitis from 3 countries were collected. Wegener's granulomatosis was the predominant disease, comprising 63% of the cohort. Diagnoses were confirmed by physicians in 98% of cases. The most common item from the free-text sections was fatigue (75%), followed by pain (31%), musculoskeletal symptoms (24%), difficulty breathing (19%), financial aspects (13%), nasal discharge/crusting (14%), and weight gain (10%). Rankings of individual items varied substantially: fatigue (3.5), loss of energy (3.4), weight gain (3.1), joint pain (3.0), and sinusitis (3.0) were the highest-ranked symptoms among those manifestations experienced by at least 50% of patients. Several severe manifestations (e.g., dialysis, seizures, oxygen dependency) were ranked lower (<3.0). CONCLUSION: Fatigue and reduced energy level are considered the most important disease burdens by patients with vasculitis; manifestations associated with organ damage were rated lower. The patients' perspectives of the impact of vasculitis differ from the physicians' ratings. Future outcome assessment in vasculitis should include the patients' perspectives. | Male | Humans | Patient Participation | Questionnaires | OK |
| R. | Roberts | D. | Howe | D. | Mort | Clinical Oncology (Royal College of Radiologists (Great Britain)) | 2010-05-00 May 2010 | | Humans | Patient Participation | Female | Breast Neoplasms | OK |
| I. Ralph | Edwards | Paul | Wicks | Meredith Y. | Smith | Drug Safety | 2013-12-00 Dec 2013 | The Patient-Reported Outcomes Safety Event Reporting (PROSPER) Consortium was convened to improve safety reporting by better incorporating the perspective of the patient. PROSPER comprises industry, regulatory authority, academic, private sector and patient representatives who are interested in the area of patient-reported outcomes of adverse events (PRO-AEs). It has developed guidance on PRO-AE data, including the benefits of wider use and approaches for data capture and analysis. Patient-reported outcomes (PROs) encompass the full range of self-reporting, rather than only patient reports collected by clinicians using validated instruments. In recent years, PROs have become increasingly important across the spectrum of healthcare and life sciences. Patient-centred models of care are integrating shared decision making and PROs at the point of care; comparative effectiveness research seeks to include patients as participatory stakeholders; and industry is expanding its involvement with patients and patient groups as part of the drug development process and safety monitoring. Additionally, recent pharmacovigilance legislation from regulatory authorities in the EU and the USA calls for the inclusion of patient-reported information in benefit-risk assessment of pharmaceutical products. For patients, technological advancements have made it easier to be an active participant in one's healthcare. Simplified internet search capabilities, electronic and personal health records, digital mobile devices, and PRO-enabled patient online communities are just a few examples of tools that allow patients to gain increased knowledge about conditions, symptoms, treatment options and side effects. Despite these changes and increased attention on the perceived value of PROs, their full potential has yet to be realised in pharmacovigilance. Current safety reporting and risk assessment processes remain heavily dependent on healthcare professionals, though there are known limitations such as under-reporting and discordant perspectives between patient reports and clinician perceptions of adverse outcomes. PROSPER seeks to support the wider use of PRO-AEs. The scope of this guidance document, which was completed between July 2011 and March 2013, considered a host of domains related to PRO-AEs, including definitions and suitable taxonomies, the range of datasets that could be used, data collection mechanisms, and suitable analytical methodologies. PROSPER offers an innovative framework to differentiate patient populations. This framework considers populations that are prespecified (such as those in clinical trials, prospective observational studies and some registries) and non-prespecified populations (such as those in claims databases, PRO-enabled online patient networks, and social websites in general). While the main focus of this guidance is on post-approval PRO-AEs from both prespecified and non-prespecified population groups, PROSPER has also considered pre-approval, prespecified populations. The ultimate aim of this guidance is to ensure that the patient 'voice' and perspective feed appropriately into collection of safety data. The guidance also covers a minimum core dataset for use by industry or regulators to structure PRO-AEs (accessible in the online appendix) and how data, once collected, might be evaluated to better inform on the safe and effective use of medicinal products. Structured collection of such patient data can be considered both a means to an end (improving patient safety) as well as an end in itself (expressing the patient viewpoint). The members of the PROSPER Consortium therefore direct this PRO-AE guidance to multiple stakeholders in drug safety, including industry, regulators, prescribers and patients. The use of this document across the entirety of the drug development life cycle will help to better define the benefit-risk profile of new and existing medicines. Because of the clinical relevance of 'real-world' data, PROs have the potential to contribute important new knowledge about the benefits and risks of medicinal products, communicated through the voice of the patient. | Humans | Data Collection | risk assessment | Outcome Assessment (Health Care) | OK |
| N. | Nnamoko | A. | Whiteman | J. | Ring | Journal of the American Medical Informatics Association: JAMIA | 2014-08-00 2014 Jul-Aug | An innovative web-based system was developed to allow patient-reported outcome measures (PROMs) to be easily administered. Stakeholders guided the design and implementation. The software gives patients access to their current and previous scores. This pilot study focused on patients undergoing arthroscopic subacromial decompression, evaluated using the Oxford shoulder score (OSS). Patients showing good improvement in their OSS were offered the choice to return for routine follow-up clinic appointments, or continue rehabilitation, reassured by their improved score. Thirty-six of 117 patients were eligible. Thirty of these (83%) were opted to avoid further clinics. PROMs 2.0 can be used for any medical intervention with a validated PROM. Evolution and refinement is ongoing. Funding has been granted for 12 primary and secondary healthcare trusts to implement PROMs 2.0. Further work is needed to assess economic impact, patient views and satisfaction with the process. | Humans | Patient Participation | Severity of Illness Index | Patient Outcome Assessment | Doublon |
| N. | Nnamoko | A. | Whiteman | J. | Ring | Journal of the American Medical Informatics Association: JAMIA | 2014-08-00 2014 Jul-Aug | An innovative web-based system was developed to allow patient-reported outcome measures (PROMs) to be easily administered. Stakeholders guided the design and implementation. The software gives patients access to their current and previous scores. This pilot study focused on patients undergoing arthroscopic subacromial decompression, evaluated using the Oxford shoulder score (OSS). Patients showing good improvement in their OSS were offered the choice to return for routine follow-up clinic appointments, or continue rehabilitation, reassured by their improved score. Thirty-six of 117 patients were eligible. Thirty of these (83%) were opted to avoid further clinics. PROMs 2.0 can be used for any medical intervention with a validated PROM. Evolution and refinement is ongoing. Funding has been granted for 12 primary and secondary healthcare trusts to implement PROMs 2.0. Further work is needed to assess economic impact, patient views and satisfaction with the process. | Humans | Patient Participation | Severity of Illness Index | Patient Outcome Assessment | OK |
| Nicolaas | de Jonge | Kadir | Caliskan | Jennifer | Kealy | Circulation. Heart Failure | 2011-11-00 Nov 2011 | BACKGROUND: Technological advancements of left ventricular assist devices (LVAD) have created today's potential for extending the lives of patients with end-stage heart failure. Few studies have examined the effect of LVAD therapy on patient-reported outcomes (PROs), such as health status, quality of life, and anxiety/depression, despite poor PROs predicting mortality and rehospitalization in patients with heart failure. In this systematic review, we provide an overview of available evidence on the impact of LVAD therapy on PROs and discuss recommendations for clinical research and practice. METHODS AND RESULTS: A systematic literature search identified 16 quantitative studies with a sample size ≥10 (mean±SD age=50.1±12.6 years) that examined the impact of LVAD therapy on PROs using a quantitative approach. Initial evidence suggests an improvement in health status, anxiety, and depression in the first few months after LVAD implantation. However, PRO scores of patients receiving LVAD therapy are still lower for physical, social, and emotional functioning compared with transplant recipients. These studies had several methodological shortcomings, including the use of relatively small sample sizes, and only a paucity of studies focused on anxiety and depression. CONCLUSIONS: There is a paucity of studies on the patient perspective of LVAD therapy. To advance the field of LVAD research and to optimize the care of an increasingly growing population of patients receiving LVAD therapy, more well-designed large-scale studies are needed to further elucidate the impact of LVAD therapy on PROs. | Humans | Patient Participation | Aged | Middle Aged | OK |
| A. | Nordin | R. | Liston | M. | Burnell | British Journal of Cancer | 2013-08-06 Aug 6, 2013 | BACKGROUND: Most studies use hospital data to calculate postoperative complication rates (PCRs). We report on improving PCR estimates through use of patient-reporting. METHODS: A prospective cohort study of major surgery performed at 10 UK gynaecological cancer centres was undertaken. Hospitals entered the data contemporaneously into an online database. Patients were sent follow-up letters to capture postoperative complications. Grade II-V (Clavien-Dindo classification) patient-reported postoperative complications were verified from hospital records. Postoperative complication rate was defined as the proportion of surgeries with a Grade II-V postoperative complication. RESULTS: Patient replies were received for 1462 (68%) of 2152 surgeries undertaken between April 2010 and February 2012. Overall, 452 Grade II-V (402 II, 50 III-V) complications were reported in 379 of the 1462 surgeries. This included 172 surgeries with 200 hospital-reported complications and 231 with 280 patient-reported complications. All (100% concordance) 36 Grade III-V and 158 of 280 (56.4% concordance) Grade II patient-reported complications were verified on hospital case-note review. The PCR using hospital-reported data was 11.8% (172 out of 1462; 95% CI 11-14), patient-reported was 15.8% (231 out of 1462; 95% CI 14-17.8), hospital and verified patient-reported was 19.4% (283 out of 1462; 95% CI 17.4-21.4) and all data were 25.9% (379 out of 1462; 95% CI 24-28). After excluding Grade II complications, the hospital and patient verified Grade III-V PCR was 3.3% (48 out of 1462; 95% CI 2.5-4.3). CONCLUSION: This is the first prospective study of postoperative complications we are aware of in gynaecological oncology to include the patient-reported data. Patient-reporting is invaluable for obtaining complete information on postoperative complications. Primary care case-note review is likely to improve verification rates of patient-reported Grade II complications. | Humans | Patient Participation | Female | Aged | OK |
| Nick | Sevdalis | Charles | Vincent | Rachel | Massey | Journal of Evaluation in Clinical Practice | 2012-08-00 Aug 2012 | RATIONALE, AIMS AND OBJECTIVES: Patients can play an important role in reducing health care harm. Finding strategies to encourage patients to take on an active role in issues related to the quality and safety of their care is therefore essential. The aim of this study was to examine patients' and health care professionals' attitudes towards a video aimed at promoting patient involvement in safety-related behaviours. METHOD: A within-subjects design was used where participants were required to complete a questionnaire pre and post screening of a patient safety video. Participants are 201 patients aged 19-103 years (mean 52) and 95 health care professionals aged 23-48 years (mean 32). Main outcome measures include (i) patients' willingness to participate and perceived importance in participating in safety-related behaviours; and (ii) health care professionals' willingness to support patient involvement. RESULTS: After watching the video patients elicited more positive attitudes towards asking doctors and nurses if they had washed their hands and notifying them about issues to do with personal hygiene. No significant effects were observed in relation to patients notifying staff if they have not received their medication or if they were in pain or feeling unwell. In relation to health care professionals, doctors and nurses were more willing to support patient involvement in asking about hand hygiene after they had watched the video. CONCLUSION: Video may be effective at changing patients' and health care professionals' attitudes towards patient involvement in some, but not all safety-related behaviours. Our findings suggest video may be most effective at encouraging involvement in behaviours patients are less inclined to participate in and health care professionals are less willing to support. | Male | Humans | Patient Participation | Questionnaires | OK |
| Sascha | Köpke | Gary | Fulcher | Friedemann | Geiger | PloS One | 2011-00-00 2011 | OBJECTIVE: Patient involvement into medical decisions as conceived in the shared decision making method (SDM) is essential in evidence based medicine. However, it is not conclusively evident how best to define, realize and evaluate involvement to enable patients making informed choices. We aimed at investigating the ability of four measures to indicate patient involvement. While use and reporting of these instruments might imply wide overlap regarding the addressed constructs this assumption seems questionable with respect to the diversity of the perspectives from which the assessments are administered. METHODS: The study investigated a nested cohort (N = 79) of a randomized trial evaluating a patient decision aid on immunotherapy for multiple sclerosis. Convergent validities were calculated between observer ratings of videotaped physician-patient consultations (OPTION) and patients' perceptions of the communication (Shared Decision Making Questionnaire, Control Preference Scale & Decisional Conflict Scale). RESULTS: OPTION reliability was high to excellent. Communication performance was low according to OPTION and high according to the three patient administered measures. No correlations were found between observer and patient judges, neither for means nor for single items. Patient report measures showed some moderate correlations. CONCLUSION: Existing SDM measures do not refer to a single construct. A gold standard is missing to decide whether any of these measures has the potential to indicate patient involvement. PRACTICE IMPLICATIONS: Pronounced heterogeneity of the underpinning constructs implies difficulties regarding the interpretation of existing evidence on the efficacy of SDM. Consideration of communication theory and basic definitions of SDM would recommend an inter-subjective focus of measurement. TRIAL REGISTRATION: Controlled-Trials.com ISRCTN25267500. | Decision Making | Decision Support Techniques | Humans | Patient Participation | OK |
| Jim | Stewart | Andrew | Letters |  |  | Nurse Education Today | 2012-02-00 Feb 2012 | The care of patients with inflammatory bowel disease (IBD) frequently falls short of the highest standards. This is noted in several publications, including national standards, despite nursing students being taught the importance of listening to and understanding patients. Teaching staff at the University of Glasgow primarily responsible for teaching third year undergraduate nursing students undertook a radical rethink of the planning, delivery and assessment of lectures on IBD. The subject had previously been delivered in a modified lecture format. Although the topic could be included in the end-of-year exams, there was little evidence to show whether this traditional teaching method had any effect on students' clinical practice. In a novel approach to learning and assessment, students were invited to research and produce an information leaflet for newly diagnosed patients with IBD. The leaflets were then assessed and grades awarded by an expert panel of patients and carers. Such enquiry based learning (EBL) intended to demonstrate in practice, the key role patients can play in both undergraduate nurse education and in service planning and delivery in the National Health Service (NHS). The panel found the exercise both interesting and insightful, while the students reported being invigorated and felt the expert assessment meant they were forced to achieve a higher level of work. | Humans | Patient Participation | Great Britain | Students, Nursing | OK |
| Upali W. | Jayasinghe | Bettina | Christl | Judy | Proudfoot | Journal of Evaluation in Clinical Practice | 2011-04-00 Apr 2011 | AIMS: To validate the Patients Assessment of Chronic Illness Care (PACIC) among patients with chronic disease in the Australian context and to examine the relationship between patient-assessed quality of care and patient and practice characteristics. METHODS: Cross-sectional analysis of baseline data in two independent health service intervention studies that involved patients with type 2 diabetes, ischaemic heart disease and/or hypertension in general practice. The first study involved 2552 patients from 60 urban and rural general practices. The second involved 989 patients from 26 practices in Sydney. Patients were mailed a questionnaire, which included the PACIC and Short Form Health Survey. Factor analysis was performed and the factor scores and total PACIC were analysed using multi-level regression models against practice and patient characteristics. RESULTS: Factor analysis revealed a two-factor solution with similar loading of PACIC items in both studies: one for shared decision making and self-management and the other for planned care. Practice characteristics were not related to PACIC scores. Scores were related to patient characteristics - education, retirement, type and number and duration of conditions. CONCLUSIONS: The two-factor structure of the PACIC found in these Australian studies is different from the five-factor structure found in the US and the European studies. This may be related to differences in the way patients interact with the health system especially the use of Team Care plans. The association of total scores with patient characteristics was consistent with those found in other studies including a lack of association with gender, age and ethnicity. These findings should be taken into consideration when comparing patient-assessed quality of care between countries using this tool. | Male | Humans | Patient Satisfaction | Questionnaires | OK |
|  |  |  |  |  |  | BMC medical education | 2014-00-00 2014 | BACKGROUND: Professionalism and communication skills constitute important components of the integral formation of physicians which has repercussion on the quality of health care and medical education. The objective of this study was to assess medical graduates' professionalism and communication skills from the patients' perspective and to examine its association with patients' socio-demographic variables. METHODS: This is a hospital based cross-sectional study. It involved 315 patients and 105 medical graduates selected by convenient sampling method. A modified and validated version of the American Board of Internal Medicine's (ABIM) Patient Assessment survey questionnaire was used for data collection through a face to face interview. Data processing and analysis were performed using the Statistical Package for Social Science (SPSS) 16.0. Mean, frequency distribution, and percentage of the variables were calculated. A non-parametric Kruskal Wallis test was applied to verify whether the patients' assessment was influenced by variables such as age, gender, education, at a level of significance, p ≤ 0.05. RESULTS: Female patients constituted 46% of the sample, whereas males constituted 54%. The mean age was 36 ± 16. Patients' scoring of the graduate's skills ranged from 3.29 to 3.83 with a mean of 3.64 on a five-point Likert scale. Items assessing the "patient involvement in decision-making" were assigned the minimum mean values, while items dealing with "establishing adequate communication with patient" assigned the maximum mean values. Patients, who were older than 45 years, gave higher scores than younger ones (p < 0.001). Patients with higher education reported much lower scores than those with lower education (p = 0.003). Patients' gender did not show any statistically significant influence on the rating level. CONCLUSION: Generally patients rated the medical graduates' professionalism and communication skills at a good level. Patients' age and educational level were significantly associated with the rating level. | Male | Humans | Patient Participation | Patient Satisfaction | OK |
|  |  |  |  |  |  | Journal of Mental Health (Abingdon, England) | 2010-08-00 Aug 2010 | BACKGROUND: A dichotomous ICD-10 or DSM-IV diagnosis of depression does not fully capture its natural phenotype: a dimensional continuum of environmentally reactive fluctuations in mood. Therefore, ICD and DSM labels of depression, although useful from a service perspective, fail to help patients directly. This paper will show, using the author's experiences as an example, that patients with mood disorders can "diagnose" the natural phenotype, in the sense that systematic recording of painful fluctuations in environmentally reactive moods by the patient him- or herself may: (i) not only provide an accurate assessment of depression, but (ii) result in a process of conscious awareness of aberrant responses to the environment - a first step on the way to recovery. METHOD: The author developed a rudimentary but useable method to diagnose his dynamic mood fluctuations over the years. The continuous assessment method allowed him to systematically score his mood and activities every day, enabling him to study and analyse in detail the course and context of mood symptoms over time. RESULTS: With time, this initial strategy evolved into a new, more informed, more empowered diagnosis of his problems and ultimately contributed to a more resilient mind set with regard to mood changes and ways of active rather than avoidant coping with them. CONCLUSIONS: Many people with mood problems receive a DSM label but are not taught to engage in diagnosing what depression is: a dynamic pattern of environmentally reactive mood responses. Continuous diagnostic self-assessment strategies may help patients to learn, adjust and cope with their emotional vulnerabilities expressed as dimensional and reactive variation in mood. Patients can be empowered to diagnose experiences as a means to understand the sources and consequences of continuous mood variation over time. | Humans | Patient Participation | Social environment | Risk Factors | OK |
| Laura | Thomas | John | Campbell | Glyn | Lewis | Family Practice | 2013-12-00 Dec 2013 | BACKGROUND: Adequate recruitment and retention rates are vital to achieving a successful randomized controlled trial. Historically this has been particularly challenging in mental health research. Few researchers have explored patients' reasons for taking part and remaining in a depression trial. OBJECTIVE: To explore patients' reasons for taking part and remaining in a trial that aimed to assess the effectiveness of cognitive behavioural therapy (CBT) as an adjunct to usual care for primary care patients with treatment resistant depression. METHOD: (i) DESIGN: Patients completed a short exit questionnaire about their experiences of taking part in the CoBalT trial. In addition, 40 semi-structured telephone interviews were conducted with a purposive sample of these patients to further explore their experiences. (ii) SETTING: General practices, England and Scotland. RESULTS: Of 469 patients randomized into the trial, 302 (64.4%) completed an exit questionnaire. The most frequently rated reason for taking part in the study were 'I was willing to try anything that might help me feel better' (66%). Patients indicated in interviews why they preferred follow-up data to be collected on a face-to-face basis rather than over the telephone. Some patients reported that taking part in the trial gave them a sense of self-worth and accomplishment. CONCLUSION: Patients felt they benefited from being in the trial because it enabled them to reflect on their feelings. For some, taking part increased their feelings of self-worth. These findings may be applicable to trials where feelings of inclusion and being valued are likely to promote continued participation. | Male | Humans | Patient Participation | Questionnaires | OK |
| Anne | Rogers | Martin | Eden | Caroline | Gardner | Medical Decision Making: An International Journal of the Society for Medical Decision Making | 2013-01-00 Jan 2013 | BACKGROUND: Shared decision making (SDM) and patient self-management support are key components of US and UK policy for chronic disease management, whereby SDM is seen as enhancing physician-patient negotiation around self-management. The WISE trial is implementing training in self-management support for primary care physicians in one UK region. This article describes preintervention levels of patient-reported SDM and explores how this varies with patient and practice characteristics. METHODS: We analyzed baseline data from a cluster randomized controlled trial for 2965 patients with diabetes, chronic obstructive pulmonary disease, and irritable bowel syndrome (IBS) from 29 family practices. Patient-level measures included self-report of chronic conditions, SDM (Health Care Climate Questionnaire [HCCQ]), health status, and demographic characteristics. Area and practice characteristics included chronic disease workload and socioeconomic deprivation. RESULTS: The mean SDM score was 75 (out of 100), but the range was wide. The mean score was lower for IBS patients but did not vary with other disease conditions. Younger patients and those with poorer health status reported lower degrees of SDM. No associations were found with practice characteristics. LIMITATIONS: The study was restricted to one socioeconomically deprived region, and hence results may not be nationally representative of the United Kingdom. Ceiling effects on SDM scores may limit the utility of the HCCQ. CONCLUSIONS: Lower ratings from some patient groups may reflect differences in expectations rather than differences in physician behavior. Overall levels of SDM were high, and no patient or practice characteristic represented a serious barrier to SDM. However, we cannot say to what extent SDM in this chronic population addressed self-management issues rather than clinical care. More nuanced measures of SDM are required that distinguish between different forms of care. | Male | Decision Making | Humans | Questionnaires | OK |
| S. | Lorenzo | C. | Royuela | V. | Pérez-Jover | Anales Del Sistema Sanitario De Navarra | 2012-04-00 2012 Jan-Apr | BACKGROUND: Diabetes and kidney disease are risk factors for adverse events (AE). There are no other studies on the perception of risk in these patients. This study analyzes the frequency of adverse event triggers reported by diabetic and renal patients and their perception of the risk. MATERIAL AND METHODS: Descriptive study based on interviews with randomly selected patients. Field study conducted between February and May 2010 in three health centers and two hospitals in Alicante and Madrid. RESULTS: A total of 199 patients answered, 98 diabetic patients and 101 renal patients. Renal patients accumulated more AE triggers (21.8% referred to an AE trigger, 17.8% two AE triggers and 3% referred to > 3 AE triggers) than diabetic patients (16.3% referred to one AE trigger, 7.1% to two AE triggers and 7.1% referred to > 3 AE triggers). During the last year 6/98 diabetic patients and 10/101 renal patients required additional treatment due to a clinical error. The probability of the patient being the victim of a clinical error with serious consequences was 1:10. Women with renal illness believed themselves to have a greater probability of suffering an error (Chi2=12.7, p=0.002). Errors were attributed to a lack of time to attend to all patients and a lack of means and resources, without statistically significant differences between the subsamples. Interviewed patients considered that the risks of suffering a traffic accident or robbery were similar to the risk of an error with serious consequences. CONCLUSION: Information provided by patients can help improve safety procedures. | Male | Humans | Patient Participation | Female | OK |
| Mick P. | Couper | Carrie A. | Levin | Eleanor | Singer | Medical Decision Making: An International Journal of the Society for Medical Decision Making | 2010-10-00 2010 Sep-Oct | BACKGROUND: To make informed decisions, patients must have adequate knowledge of key decision-relevant facts. OBJECTIVE: To determine adults' knowledge about information relevant to common types of medication, screening, or surgery decisions they recently made. SETTING: National sample of US adults identified by random-digit dialing. DESIGN: Cross-sectional survey conducted between November 2006 and May 2007. PARTICIPANTS: A total of 2575 English-speaking adults aged 40 y or older who reported having discussed the following medical decisions with a health care provider within the previous 2 y: prescription medications for hypertension, hypercholesterolemia, or depression; screening tests for colorectal, breast, or prostate cancer; or surgeries for knee/hip replacement, cataracts, or lower back pain. MEASUREMENTS: Participants answered knowledge questions and rated the importance of their health care provider, family/friends, and the media as sources of information. RESULTS: Accuracy rates varied widely across questions and decision contexts. For example, patients considering cataract surgery were more likely to correctly estimate recovery time than those patients considering lower back pain or knee/hip replacement (78% v. 29% and 39%, P < 0.001). Similarly, participants were more knowledgeable of facts about colorectal cancer screening than those who were asked about breast or prostate cancer. Finally, respondents were consistently more knowledgeable on comparable questions about blood pressure medication than cholesterol medication or antidepressants. The impact of demographic characteristics and sources of information also varied substantially. For example, blacks had lower knowledge than whites about cancer screening decisions (odds ratio [OR] = 0.57; 95% confidence interval [CI] = 0.43, 0.75; P = 0.001) and medication (OR = 0.77; 95% CI = 0.60, 0.97; P = 0.03) even after we controlled for other demographic factors. The same was not true for surgical decisions. LIMITATIONS: The questions did not measure all knowledge relevant to informed decision making, were subject to recall biases, and may have assessed numeracy more than knowledge. CONCLUSIONS: Patient knowledge of key facts relevant to recently made medical decisions is often poor and varies systematically by decision type and patient characteristics. Improving patient knowledge about risks, benefits, and characteristics of medical procedures is essential to support informed decision making. | Male | Decision Making | Humans | Patient Participation | OK |
| Nassim | Kamar | Lionel | Rostaing | Vincent | Donckier | Clinical Transplantation | 2013-10-00 2013 Sep-Oct | BACKGROUND: The goal of this health-psychology study was to investigate the range of motives that make someone consent or refuse participation in a clinical transplant trial. METHODS: The study involved (i) preparatory interviews with five transplant patients who took part in clinical trials and five persons from the general public; (ii) we created a questionnaire with scaled responses; (iii) we selected 468 patients, divided into two groups: patients waiting for a transplant and patients who already had a transplant; (iv) we obtained patient consents, sent out the questionnaire, and recorded responses; (v) data were analysed using descriptive statistics, exploratory factorial analyses, correlations and regressions. RESULTS: Two hundred and ten patients answered the questionnaire. Motives were classified into the following: (i) Motives to consent participating in a clinical trial (pride in participating, hope for better quality of life, sufficient and clear information, discussion with others participating in a trial, altruism); or (ii) Motives to refuse participating (no information on medical teams, lack of explanation, fear of additional expenses, being considered a "guinea pig"). CONCLUSIONS: This study contributes to our understanding of the motivations of patients who accepted or refused participation in a clinical transplantation trial. | Male | Humans | Patient Participation | Questionnaires | Doublon |
| Nassim | Kamar | Lionel | Rostaing | Vincent | Donckier | Clinical Transplantation | 2013-10-00 2013 Sep-Oct | BACKGROUND: The goal of this health-psychology study was to investigate the range of motives that make someone consent or refuse participation in a clinical transplant trial. METHODS: The study involved (i) preparatory interviews with five transplant patients who took part in clinical trials and five persons from the general public; (ii) we created a questionnaire with scaled responses; (iii) we selected 468 patients, divided into two groups: patients waiting for a transplant and patients who already had a transplant; (iv) we obtained patient consents, sent out the questionnaire, and recorded responses; (v) data were analysed using descriptive statistics, exploratory factorial analyses, correlations and regressions. RESULTS: Two hundred and ten patients answered the questionnaire. Motives were classified into the following: (i) Motives to consent participating in a clinical trial (pride in participating, hope for better quality of life, sufficient and clear information, discussion with others participating in a trial, altruism); or (ii) Motives to refuse participating (no information on medical teams, lack of explanation, fear of additional expenses, being considered a "guinea pig"). CONCLUSIONS: This study contributes to our understanding of the motivations of patients who accepted or refused participation in a clinical transplantation trial. | Male | Humans | Patient Participation | Questionnaires | OK |
| K. | Brodersen | M. | Ringdal |  |  | Acta Anaesthesiologica Scandinavica | 2011-08-00 Aug 2011 | BACKGROUND: Patients' difficulties following critical illness and the willingness of intensive care units (ICU) to take an expanded responsibility during the recovery period have led to the development of different follow-up programs. The aim of this study was to explore and describe patients' participation in and evaluation of a follow-up program at a nurse-led clinic (NLC). METHODS: Patients with a length of stay ≥72 h, discharged from the ICU, participated in a follow-up program based on three contacts, as a visit to the NLC, telephone contact, ward visit or as an indirect contact, during a 6-month period. A specially developed database recorded information regarding patients' participation and questionnaires were used to obtain patients' views of the follow-up program. RESULTS: Of 96 study patients, 51% visited the NLC once or twice. These patients were younger (P<0.001) and had lower Acute Physiology and Chronic Health Evaluation II (P=0.017) compared with those who did not visit the clinic. The most common reason for not visiting the clinic was not enough strength, chiefly physical. In the evaluation, patients answered that they received advice and information, an opportunity to talk, increased knowledge and re-evaluated memories and experiences from the ICU stay. Patients appreciated the follow-up and expressed gratitude to the competent and obliging staff. CONCLUSION: The current follow-up program, adjusted to individual patients' conditions and needs in terms of different types of contacts and continuity, was found to be of great value. Effects of the program other than the patient perspective are also relevant to evaluate. | Male | Humans | Patient Participation | Questionnaires | OK |
| Yussuf | Al-Jawarneh | Hala | Amer | Muhammad B. | Hammami | BMC medical ethics | 2014-00-00 2014 | BACKGROUND: Although informed consent is an integral part of clinical practice, its current doctrine remains mostly a matter of law and mainstream ethics rather than empirical research. There are scarce empirical data on patients' perceived purpose of informed consent, which may include administrative routine/courtesy gesture, simple honest permission, informed permission, patient-clinician shared decision-making, and enabling patient's self decision-making. Different purposes require different processes. METHODS: We surveyed 488 adults who were planning to undergo or had recently undergone written informed consent-requiring procedures. Perceptions of informed consent purpose (from norm and current practice perspectives) were explored by asking respondents to rank (1 = most reflective) 10 randomly-presented statements: "meaningless routine", "courtesy gesture" "litigation protection", "take away compensation rights", "inform patient', "make sure patient understand", "document patient's decision", "discover patient's preferences", "have shared decision", and "help patient decide". RESULTS: Respondents' mean (SD) age was 38.3 (12.5); 50.4% were males, 56.8% had ≥ college education, and 37.3% had undergone a procedure. From the norm perspective, the least reflective statement was "meaningless routine" (ranked 1-3 by 2.6% of respondents) and the most reflective statements were "help patient decide", "make sure patient understand", and "inform patient" (ranked 1-3 by 65%, 60%, and 48% of respondents with median [25%,75%] ranking scores of 2 [1,5], 3 [2,4], and 4 [2,5], respectively). Compared to their counterparts, males and pre-procedure respondents ranked "help patient decide" better, whereas females and post-procedure respondents ranked "inform patient" better (p = 0.007 to p < 0.001). Age was associated with better ranking of "help patient decide" and "make sure patient understand" statements (p < 0.001 and p = 0.002, respectively), which were ranked 1-3 by only 46% and 42% of respondents from the current practice perspective (median ranking score 4 [2,6], p < 0.001 vs. norm perspective for both). CONCLUSIONS: 1) the informed consent process is important to patients, however, patients vary in their views of its purpose with the dominant view being enabling patients' self decision-making, 2) males, pre-procedure, and older patients more favor a self decision-making purpose, whereas females and post-procedure patients more favor an information disclosure purpose, and 3) more self decision-making and more effective information disclosure than is currently practiced are desired. An informed consent process consistent with Mill's individual autonomy model may be suitable for most patients. | Male | Decision Making | Humans | Physician-Patient Relations | OK |
| Asao | Ogawa | Yu | Yamada | Yutaka | Nishiwaki | Psycho-Oncology | 2012-07-00 Jul 2012 | OBJECTIVE: The objective of this study was to evaluate the patients' perception of the usefulness of a question prompt sheet (QPS) in facilitating the involvement of advanced cancer patients during consultation. METHODS: Advanced cancer patients attending their first consultation after diagnosis were randomly assigned to the intervention group (received QPS and a hospital introduction sheet (HIS)) or the control group (received HIS only). Analysis was conducted on an intention-to-treat basis. The primary outcome measure was patient rating of the usefulness of the material(s) (numerical rating scale of 0-10). RESULTS: Sixty-three advanced cancer patients (72.4% response rate) were enrolled and analyzed. Nearly three-quarters of patients in both groups read the material(s) before consultation. The rated usefulness of the material(s) for asking questions of physicians was significantly higher in the intervention group than in controls (4.4 ± 3.6 and 2.7 ± 2.8, respectively; p = 0.033). The mean score of the usefulness of the material(s) for understanding the treatment plan tended to be higher in the intervention group than in the controls (4.9 ± 3.6 and 3.3 ± 2.8; p = 0.051). The mean score of willingness to use the material(s) in the future was significantly higher in the intervention group than in the controls (5.3 ± 3.8 and 2.8 ± 2.8; p = 0.006). There were no significant differences between the groups in the average total number of questions asked by patients (median, 1.0; interquartile range in both groups, 2.0). CONCLUSIONS: QPS provided before oncology consultation may be useful for advanced cancer patients, on the other hand, it did not directly promote patient confidence to ask questions. | Male | Decision Making | Humans | Patient Participation | OK |
| Tim | Newton |  |  |  |  | BMC oral health | 2012-00-00 2012 | BACKGROUND: Oral cancer is increasing in incidence in the UK and indeed worldwide. Delay in diagnosis is common; up to half of patients are diagnosed with advanced lesions. Thus it is essential to develop methods to aid early detection. This study aimed to assess dental patients' experiences and awareness of oral cancer and screening within general dental practice. METHODS: A cross-sectional questionnaire survey of 184 English-speaking adults, with no previous history of oral cancer was conducted. The questionnaire collected data on participant's knowledge of oral cancer, experience of 'screening', attitudes and feelings towards having a screening, anticipated help-seeking behaviours, health-related behaviours (particularly risk factors) and sociodemographics. RESULTS: Twenty percent of respondents had never heard of oral cancer; 77% knew little or nothing about it and 72% did not know that their Dentist routinely screens for oral cancer. Overall, attitudes to screening were positive. Ninety two percent of respondents would like their Dentist to tell them if they were being screened for signs of oral cancer and 97% would like help from their Dentists to reduce their risk. CONCLUSION: Patients seem generally unaware of oral cancer screening by their dentist but are happy to take part in screening, would like to be informed, and welcome the support of their Dentist to reduce their risk of developing oral cancer. | Male | Humans | Patient Participation | Questionnaires | OK |
| Javier | Gracia | Helle Ploug | Hansen | Alessandra | Lo Scalzo | International Journal of Technology Assessment in Health Care | 2010-07-00 Jul 2010 | There is increasing emphasis on providing patient-focused health care and ensuring patient involvement in the design of health services. As health technology assessment (HTA) is meant to be a multidisciplinary, wide-ranging policy analysis that informs decision making, it would be expected that patients' views should be incorporated into the assessment. However, HTA is still driven by collection of quantitative evidence to determine the clinical and cost effectiveness of a health technology. Patients' perspectives about their illness and the technology are rarely included, perhaps because they are seen as anecdotal, biased views. There are two distinct but complementary ways in which HTAs can be strengthened by: (i) gathering robust evidence about the patients' perspectives, and (ii) ensuring effective engagement of patients in the HTA process from scoping, through evidence gathering, assessment of value, development of recommendations and dissemination of findings. Robust evidence eliciting patients' perspectives can be obtained through social science research that is well conducted, critically appraised and carefully reported, either through meta-synthesis of existing studies or new primary research. Engagement with patients can occur at several levels and we propose that HTA should seek to support effective patient participation to create a fair deliberative process. This should allow two-way flow of information, so that the views of patients are obtained in a supportive way and fed into decision-making processes in a transparent manner. | Humans | Patient Satisfaction | Patient-Centered Care | Health Knowledge, Attitudes, Practice | OK |
| Fariba | Asghari |  |  |  |  | Journal of Medical Ethics | 2011-09-00 Sep 2011 | INTRODUCTION: Substitute decision making on behalf of incapable patients is based on the ethical principle of 'respect for autonomy'. This study was conducted to assess patients' wishes and preferences in terms of a substitute decision maker and determinants of such preferences. METHODS: The authors conducted a cross-sectional study and selected samples randomly from patients presenting at Farabi Eye Hospital clinics who were 18&emsp14;years of age or older. Questionnaires were completed through interviews. RESULTS: 200 patients between the ages of 18 and 83&emsp14;years were interviewed. About 52% (N=105) were men and 73% (N=77) were married. Among married patients, the spouse was chosen as the substitute decision maker in only 51% of cases. Single men preferred their father first in 36% (N=9) of cases, while single girls chose their father in 5.6% (N=1) of cases and their most prevalent choice was other unmentioned people (33.3%, N=6). Most patients (93.5%) wished to be asked about their substitute decision maker when hospitalised. CONCLUSION: The results of this study show that the people we usually consult for decisions concerning patient treatment are significantly different from the patients' preferred substitute decision makers. The authors suggest patients be allowed to choose their substitute decision maker while conscious. | Male | Decision Making | Humans | Patient Participation | OK |
| Anke | Rentsch | Beate | Hornemann | Ulrich S. | Schuler | BMC cancer | 2013-00-00 2013 | BACKGROUND: A better understanding of patients' views on the benefit and burden obtained from palliative chemotherapy would facilitate shared decision making. We evaluated palliative cancer patients' reported outcomes (PROs) for toxicity and investigated the survival threshold for which they would repeat chemotherapy (CTx). METHODS: Patients who had received a minimum of three months of palliative CTx for advanced colorectal (CRC) or non-colorectal (non-CRC: upper gastrointestinal, lung and head-and-neck) cancer were assessed by questionnaire. Patients were questioned about PROs for toxicity, subjective burden from side effects, and were asked for the survival threshold necessary for them to repeat CTx. Expected survival (sum of indicated survival threshold and median survival time with best supportive care) was compared to the patients' actual survival. RESULTS: One hundred and thirty-four patients (CRC: 58; non-CRC: 76) were surveyed. The most frequent PRO- grade 3/4 toxicities were acne (12.8%), fatigue (9.0%), and diarrhea (8.5%). The symptom causing the highest subjective burden was fatigue and was worse than expected in 29.9% of the patients. The median survival threshold for which patients would repeat CTx was significantly longer in CRC than in non-CRC patients (p=0.01). Median expected survival was significantly longer than actual median survival (CRC: 44.0 months [22.0-65.9] compared with 30.0 months of actual survival [20.9-39.1]; non-CRC: 22.0 months [15.3-28.6] compared with 19.0 months of actual survival [15.1-22.9], p=0.03). CONCLUSION: Fatigue deserves more attention when toxicity of treatment and symptoms of disease are explained to patients. Patients' survival expectations from palliative chemotherapy are higher than previously described, exceed the median survival time known from phase III trials, and are significantly longer than their actual survival. | Male | Humans | Female | Aged | OK |
| Stephen | Cha | Amit | Sood |  |  | Alternative Therapies in Health and Medicine | 2011-12-00 2011 Nov-Dec | CONTEXT: Patients' unwillingness to participate in clinical research is one of the main challenges that researchers face today. In Complementary and Alternative Medicine (CAM) research, a pertinent yet understudied question is the attitude of potential participants toward clinical trials that test CAM interventions. OBJECTIVES: To assess patients' attitudes and preferences regarding participation in clinical trials which test CAM rather than conventional treatments. DESIGN: A 78-question, pencil-and-paper survey was conducted in collaboration with the Survey Research Center located in the Division of Biomedical Statistics and Informatics in the Department of Health Sciences Research at Mayo Clinic in Rochester, Minnesota. SETTING: The study took place at Mayo Clinic, an academic medical center for tertiary care in Rochester, Minnesota, USA. PATIENTS OR OTHER PARTICIPANTS: Participants were patients undergoing a general medical evaluation at the Division of General Internal Medicine in the Department of Internal Medicine at Mayo Clinic. INTERVENTION: The researchers designed a cross-sectional, point-of-care survey. The study coordinator approached patients and invited them to participate. OUTCOME MEASURES: The study summarized the data for categorical variables, identifying numbers of participants and percentages and comparing two proportions using the Fisher exact test. RESULTS: Out of 485 patients asked to participate, 400 (82%) completed the survey. Respondents were equally as interested in participating in clinical trials testing CAM as they were in trials testing conventional treatments (79% vs 69%, P = .09). For all participants, 107 (27%) were in favor of participating in CAM clinical trials and 118 (30%) were in favor of participating in conventional clinical trials. CONCLUSION: Respondents were interested equally in participating in clinical trials testing CAM vs conventional treatments. | Male | Humans | Patient Participation | Patient Satisfaction | OK |
| Jesper T. | Andreasen |  |  |  |  | Patient Education and Counseling | 2013-11-00 Nov 2013 | OBJECTIVES: To investigate patients' preferences for patient-centered communication (PCC) in the encounter with healthcare professionals in an outpatient department in rural Sierra Leone. METHODS: A survey was conducted using an adapted version of the Patient-Practitioner Orientation Scale (PPOS) as a structured interview guide. The study population was drawn from the population of all adults attending for treatment or treatment for their children. RESULTS: 144 patients were included in the analysis. Factors, such as doctor's friendly approach, the interpersonal relationship and information-sharing were all scored high (patient-centered) on the PPOS. Factors associated with shared-decision making had a lower (doctor-centered) score. A high educational level was associated with a more patient-centered scoring, an association that was most pronounced in the female population. CONCLUSION: The results provide an insight into the patients' preferences for PCC. Patients expressed a patient-centered attitude toward certain areas of PCC, while other areas were less expressed. More research is needed in order to fully qualify the applicability of PCC in resource-poor settings. PRACTICE IMPLICATIONS: Stakeholders and healthcare professionals should aim to strengthen healthcare practice by focusing on PCC in the medical encounter while taking into considerations the patients' awareness and preferences for PCC. | Male | Decision Making | Humans | Questionnaires | OK |
| Joseph | Lim | Guadalupe | Garcia-Tsao |  |  | Medical Decision Making: An International Journal of the Society for Medical Decision Making | 2010-02-00 2010 Jan-Feb | BACKGROUND: The objective of this study was to ascertain patient preferences for treatment of hepatitis C virus (HCV). METHODS: The authors recruited consecutive patients eligible for treatment of HCV and used adaptive conjoint analysis (ACA), a hybrid approach of conjoint analysis that uses both self-explicated ratings and pair-wise comparisons, to elicit preferences for pegylated-interferon and ribavirin. They examined the association between patient characteristics and treatment preferences using the Mann-Whitney U test and chi(2) statistic for continuous and categorical variables, respectively, and subsequently calculated adjusted odds ratios and 95% confidence intervals using logistic regression. RESULTS: A total of 140 subjects completed the ACA task. The mean (+/-SD) age of the sample was 51+/-8 y; 85% were male, and 59% were white. When described as being associated with mild side effects, 67% (n = 94) of subjects preferred treatment for HCV. The percentage of subjects preferring therapy decreased to 51% (n = 72) when it was described as being associated with severe side effects. Preferences for treatment of HCV were stronger among subjects with a higher perceived risk of developing cirrhosis, more severe underlying liver disease, and worse HCV-related quality of life. Subjects having more severe disease placed greater weight on the importance of expected benefits and less on the risk of toxicity compared with those with mild or no fibrosis. CONCLUSIONS: Whether to choose treatment for HCV is a difficult decision for many patients. Treatment is usually recommended for those with moderate to severe liver disease, and these results demonstrate that most patients' preferences are concordant with this practice. | Male | Humans | Patient Participation | Questionnaires | OK |
| Alend | Saadi | Valentin | Rousson | Nicolas | Demartines | World Journal of Surgery | 2013-09-00 Sep 2013 | BACKGROUND: The relationship between physicians and patients has undergone important changes, and the current emancipation of patients has led to a real partnership in medical decision making. The present study aimed to assess patients' preferences on different aspects of decision making during treatment and potential complications, as well as the amount and type of preoperative information wanted before visceral surgery. METHODS: This was a prospective non-randomized study based on a questionnaire given to 253 consecutive patients scheduled for elective gastrointestinal surgery. RESULTS: In considering surgical complications or treatment in the intensive care unit, 64 % of patients wished to take an active role in any medical decisions. The respective figures for cardiac resuscitation and treatment limitations were 89 and 60 %. As for information, 73, 77, and 47 % of patients wish detailed information, information on a potential ICU hospitalization, and knowledge of cardiac resuscitation, respectively. Elderly and low-educated patients were significantly less interested in shared medical decision making (p = 0.003 and 0.015), and in receiving information (p = 0.03 and 0.05). Similarly, involvement of the family in decision making was significantly less important to elderly and male patients (p = 0.05 and 0.03, respectively). Neither the type of operation (minor or major) nor the severity of disease (malignancies versus non-malignancies) was a significant factor for shared decision making, information, or family involvement. CONCLUSIONS: The vast majority of surgical patients clearly want to get adequate preoperative information about their disease and the planned treatment. They also consider it crucial to be involved in any kind of decision making for treatment and complications. For most patients, the family role is limited to supporting the treating physicians if the patient is unable to participate in decision making. | Male | Humans | Patient Participation | Physician-Patient Relations | OK |
| Ernst J. | Kuipers | C. Janneke | van der Woude | |  | Digestion | 2010-00-00 2010 | BACKGROUND: Shared decision-making is gaining favor in clinical practice, although the extent to which patients want to be involved in choosing their treatment varies substantially. Because data are lacking on the preferences of patients with chronic diseases such as inflammatory bowel disease (IBD), we wanted to assess IBD patients' preferences about being involved in such decisions. METHODS: Adult IBD patients were asked to anonymously complete an online survey on their preferences. Non-parametric tests (chi(2)) were used to determine the relationship between responses and respondents. RESULTS: The questionnaire was completed by 1,067 patients, 617 with Crohn's disease and 450 with ulcerative colitis. Patients' mean age was 43 (SD 13.7) years; the majority were female (66%). In total, 866 patients (81%) reported it as 'very important' to be actively involved in the decision-making process, and another 177 (17%) rated it as 'quite important'. When asked how their treatment could be improved, 537 patients (50%) wanted close, equitable collaboration with their physician. This preference was significantly associated with a disease duration of <or=8 years (p = 0.03). Gender and type of IBD were not significantly associated with patients' preferences. CONCLUSIONS: This study demonstrates IBD patients' desire to be actively involved in the decision-making process. Further research is needed on physicians' perspectives on shared decision-making, and on finding predictive factors for developing a model for shared decision-making in IBD. | Male | Decision Making | Humans | Patient Participation | OK |
| Florence | Dalenc | Jean-Marc | Ferrero | Pierre | Kerbrat | Journal of Clinical Epidemiology | 2012-06-00 Jun 2012 | OBJECTIVE: To identify the factors associated with long-term regrets expressed a posteriori by randomized controlled trial (RCT) participants questioned about their decision to participate in an RCT. STUDY DESIGN AND SETTING: Participants were questioned 6 years on average after their inclusion in a breast cancer adjuvant therapy RCT. Among 115 women from 21 centers, 93 (81%) answered a self-administered questionnaire based on the Decision Regret Scale (DRS). RESULTS: Mean DRS score was 16.8 (standard deviation=15.9); 43.0% of participants expressed mild regret, and 25.8% expressed moderate to strong regret. A quarter of the women (25.6%) said that the decision was taken by the doctor alone, and 13.5% said it was not consistent with their own wishes. In the multivariate ordinal regression analysis, an involuntarily passive role in decision making was found to be associated with greater regret (cumulative proportional odds ratio=7.3, 95% confidence interval=2.0-27.6), regardless of age and being allotted or not to the standard treatment in the RCT. CONCLUSION: Whether patients' regret depended on their level of participation in the decision making or vice versa could not be determined in this cross-sectional survey, but efforts should be made to ensure that patients' participation in trials is always based on an active personal decision. | Decision Making | Emotions | Humans | Patient Participation | OK |
|  |  |  |  |  |  | Nederlands Tijdschrift Voor Geneeskunde | 2010-00-00 2010 | Various generic and disease-specific instruments have been developed to measure quality of life. In a recent study the results of 3 standardized quality of life questionnaires were compared with qualitative research consisting of open interviews among 343 patients with inoperable lung cancer. The questionnaires did not fully cover all the factors that caused distress in lung cancer patients. For example, about a quarter of patients reported an overall form of distress instead of specific problems at some time point. In addition, 27% reported some element of their contact with the health care system as distressing. These aspects are not covered in quality of life questionnaires. Patients should always be involved in the development of questionnaires measuring quality of life or quality of care from the patients' perspective. | Humans | Patient Participation | Questionnaires | Psychometrics | OK |
| Jesper T. | Andreasen |  |  |  |  | Patient Education and Counseling | 2013-11-00 novembre 2013 | Objectives: To investigate patients’ preferences for patient-centered communication (PCC) in the encounter with healthcare professionals in an outpatient department in rural Sierra Leone. Methods: A survey was conducted using an adapted version of the Patient-Practitioner Orientation Scale (PPOS) as a structured interview guide. The study population was drawn from the population of all adults attending for treatment or treatment for their children. Results: 144 patients were included in the analysis. Factors, such as doctor's friendly approach, the interpersonal relationship and information-sharing were all scored high (patient-centered) on the PPOS. Factors associated with shared-decision making had a lower (doctor-centered) score. A high educational level was associated with a more patient-centered scoring, an association that was most pronounced in the female population. Conclusion: The results provide an insight into the patients’ preferences for PCC. Patients expressed a patient-centered attitude toward certain areas of PCC, while other areas were less expressed. More research is needed in order to fully qualify the applicability of PCC in resource-poor settings. Practice implications: Stakeholders and healthcare professionals should aim to strengthen healthcare practice by focusing on PCC in the medical encounter while taking into considerations the patients’ awareness and preferences for PCC. (PsycINFO Database Record (c) 2014 APA, all rights reserved). (journal abstract) | Shared decision making | Decision Making | Client Attitudes | stakeholders | Doublon |
|  |  |  |  |  |  | Health Affairs (Project Hope) | 2012-09-00 Sep 2012 | Hospitals frequently exhibit wide variation in their prices, and employers and insurers are now experimenting with the use of incentives to encourage employees to make price-conscious choices. This article examines two major new benefit design instruments being tested. In reference pricing, an employer or insurer makes a defined contribution toward covering the cost of a particular service and the patient pays the remainder. Through centers of excellence, employers or insurers limit coverage or strongly encourage patients to use particular hospitals for such procedures as orthopedic joint replacement, interventional cardiology, and cardiac surgery. We compare these two types of benefit designs with respect to consumer choice and how they balance price and quality. The article then examines their potential role in the policy debate over appropriate coverage and cost-sharing requirements. | Humans | Patient Participation | Choice Behavior | Quality of Health Care | OK |
| Hyojee | Joung | John P. | Allegrante |  |  | Journal of Health Communication | 2010-00-00 2010 | Patient-provider communication is an important element in cancer treatment and prevention. We examined the degree of concordance perceived to exist between the patient's preferences for inclusion in decision-making processes and their actual experiences among two population-based cohorts of U.S. adults with and without cancer histories who were surveyed in 2003 and 2007. Associations were examined between selected sociodemographic characteristics of respondents and the extent to which respondents perceived their health providers "always" involved them in decisions about their health to the extent desired. Data came from the Health Information National Trends Survey (HINTS), and SPSS and SAS-Callable SUDAAN statistical packages were used to analyze the data. Results showed a decrement in the proportion of favorable responses between 2003 and 2007. While there was no gender effect on the reported perceptions in either year, there was a significant effect of ethnicity (p = .001) in both years. Age, income, and employment were also independently associated (p = .001) in 2007. In contrast to 2003, higher education was significantly associated with communication satisfaction, as was having a cancer diagnosis, in 2007. There was a significant relationship between several sociodemographic variables and respondent perceptions about the consistency with which providers included patients as desired in decision-making. We conclude that communication between patient and provider remains suboptimal in cancer prevention and treatment. | Male | Decision Making | Humans | Patient Participation | OK |
| Sheila | Doss |  |  |  |  | Nephrology News & Issues | 2010-09-00 Sep 2010 | Usage of renal replacement modalities available for the treatment of end stage renal disease varies widely between countries, indicating that non-medical factors contribute to the choice of therapy. In the United States, 93% of the patients are treated with in-center hemodialysis (HD), about 7% undergo peritoneal dialysis (PD) and less than 1% are on home HD. In comparison, a Northern California-based nonprofit dialysis provider with home dialysis centers throughout the United States has achieved a home therapy penetration of 22%, the highest proportion of home therapies among U.S. dialysis providers. To better understand the perceptions about the various modality choices among caretakers (nephrologists and nurses) in this organization a short questionnaire was used. We examined the hypothetical setting of the caretakers being in the patient role. More than 90% of the nephrology professionals chose a home therapy as initial treatment option with close to equal distribution between PD and home HD. This pattern persisted for maintenance therapy with home HD being the preferred modality. Nephrologists' and nurses' perception of who makes modality decisions varied profoundly. | Decision Making | Humans | Patient Participation | Questionnaires | OK |
| Debra B. | Stulberg | Goutham | Rao |  |  | Patient Education and Counseling | 2013-12-00 décembre 2013 | Objective: Physicians face barriers to incorporating recommended contraceptive and preconception health services, including reproductive life plans (RLPs), into primary care. With promising findings from early studies of RLPs, we examined the impact of a novel reproductive health self-assessment tool (RHSAT) on reproductive health counseling. Methods: We created the RH-SAT for an urban community health center population and trained providers on preconception and contraceptive guidelines. Semi-structured interviews were conducted to assess perceptions of the tool with 22 patients and with all 15 providers at the clinic. Transcripts were thematically analyzed using a grounded theoretical approach. Results: Patients and providers reported the RH-SAT presented new and thought-provoking material that promoted patient participation and facilitated counseling. Conclusion: This RH-SAT is acceptable and useful to patients and providers in an underserved urban health center. In accordance with Medical Communication Alignment Theory (MCAT), increased patient participation in reproductive health discussions may alert providers to patient interest in these topics. Practice implications: This study provides preliminary evidence that the RH-SAT can help overcome barriers to reproductive health counseling in primary care. Providers may wish to incorporate tools into their practice to improve communication with patients about their reproductive health goals. (PsycINFO Database Record (c) 2014 APA, all rights reserved). (journal abstract) | physicians | Psychometrics | Test Construction | Reproductive Health Self-Assessment Tool | Doublon |
| Debra B. | Stulberg | Goutham | Rao |  |  | Patient Education and Counseling | 2013-12-00 Dec 2013 | OBJECTIVE: Physicians face barriers to incorporating recommended contraceptive and preconception health services, including reproductive life plans (RLPs), into primary care. With promising findings from early studies of RLPs, we examined the impact of a novel reproductive health self-assessment tool (RH-SAT) on reproductive health counseling. METHODS: We created the RH-SAT for an urban community health center population and trained providers on preconception and contraceptive guidelines. Semi-structured interviews were conducted to assess perceptions of the tool with 22 patients and with all 15 providers at the clinic. Transcripts were thematically analyzed using a grounded theoretical approach. RESULTS: Patients and providers reported the RH-SAT presented new and thought-provoking material that promoted patient participation and facilitated counseling. CONCLUSION: This RH-SAT is acceptable and useful to patients and providers in an underserved urban health center. In accordance with Medical Communication Alignment Theory (MCAT), increased patient participation in reproductive health discussions may alert providers to patient interest in these topics. PRACTICE IMPLICATIONS: This study provides preliminary evidence that the RH-SAT can help overcome barriers to reproductive health counseling in primary care. Providers may wish to incorporate tools into their practice to improve communication with patients about their reproductive health goals. | | | | | OK |
| Alan F. | List | David P. | Steensma | Andrew | Artz | The Oncologist | 2011-00-00 2011 | PURPOSE: Myelodysplastic syndromes (MDSs) are a heterogenous group of clonal hematopoietic disorders affecting approximately 60,000 people in the U.S. Little information is available regarding how aware MDS patients are of their disease severity, prognosis, and treatment outcomes. METHODS: This Internet-based survey assessed patient perceptions regarding these factors, determined differences between patients with higher- and lower-risk disease and between those receiving active treatment and supportive care, and assessed patient-reported outcomes. RESULTS: Among 358 patients (median age, 65 years), the median time since MDS diagnosis was 3 years and time from initial hematologic abnormality detection was 6 years. Many patients (55%) did not know their International Prognostic Scoring System score, 42% were unaware of their blast percentage, and 28% were unaware of their cytogenetics. Patients were unlikely to recall having their MDS described as cancer (7%), 37% felt their treatment would improve survival, and 16% felt treatment would be curative. Patients receiving active treatment were more likely to believe their therapy would prolong survival than those receiving supportive care (52% versus 31%; p < .001) or be curative (23% versus 14%; p = .03). Patients with higher-risk disease were more likely to think their therapy would be curative than those with lower-risk disease (26% versus 11%; p = .01). Patients with MDS reported poor physical or mental health on two to three times more days per month than population norms. CONCLUSION: Patients with MDS have a limited understanding of their disease characteristics, prognosis, and treatment goals. These results may help improve physician-patient communication and identify factors to consider when making treatment decisions. | Male | Humans | Patient Participation | Questionnaires | OK |
| Dean J. | Kereiakes | Jeffrey | Moses | John | Lasala | JACC. Cardiovascular interventions | 2012-02-00 Feb 2012 | Appropriate utilization of percutaneous coronary intervention (PCI) and medical therapy is deservedly a national healthcare policy priority for the United States. Because PCI is both common and costly, appraisal of appropriateness is warranted. The initial appropriate use criteria (AUC) have been developed for coronary revascularization procedures and investigators recently reported the appropriateness for the approximately 500,000 PCI procedures performed at centers participating in the National Cardiovascular Data Registry. The AUC have broad implications for both healthcare providers and our patients and will be used as the basis for indications, referral patterns, treatment options, physician education, shared decision-making, and reimbursement for years to come. While we acknowledge the importance of thoughtfully assessing appropriateness for all medical procedures including PCI, there are a number of concerns with the current AUC and methods used to report appropriateness that warrant expanded commentary. | Time Factors | Decision Making | Humans | United States | OK |
|  |  |  |  |  |  | Clinical Trials (London, England) | 2011-02-00 Feb 2011 | BACKGROUND: Participation in clinical trials is critical for the success of evidence-based healthcare. Much previous research suggests that people take part in clinical trials mostly for altruistic reasons, and that deriving personal benefit is a secondary consideration. PURPOSE: To investigate patients' reasons for deciding whether to take part in clinical trials and the extent to which personal benefit may be a motivating factor. METHODS: A qualitative study utilizing in-depth semi-structured interviews. Participants were 42 men and women who had been asked to participate in clinical trials testing a range of interventions in different conditions, including some who did not participate or withdrew. Trials were based in either primary or secondary care. Interviews were conducted at home. RESULTS: Reasons for taking part were complex and a wide variety of personal considerations were evident. Gaining some personal benefit emerged as an important primary motivation in this group of respondents, whereas altruistic considerations appeared to be largely subsidiary. People who decided not to take part were also mainly concerned about the balance of personal risk and benefit. LIMITATIONS: Public dissemination of the results may have influenced participants' responses. CONCLUSIONS: Achieving target sample sizes for clinical trials may be enhanced by an understanding of what is likely to motivate patients to take part. In this study, respondents identified a range of attractive benefits, such as care in a specialist team with expert staff, active engagement in their health monitoring or care, more frequent or intensive monitoring, and information about the latest research into their condition. | Male | Decision Making | Humans | Patient Participation | OK |
|  |  |  |  |  |  | Health and Quality of Life Outcomes | 2010-00-00 2010 | BACKGROUND: Numerous studies document the influence of psychosocial variables on the course of coronary heart disease. This study examines the influence of personality traits (trait anger, cynicism) and aspects of the physician-patient relationship (promoting patient participation by the physician, active communication behavior of the patient, trust in the physician) on the health related quality of life (HRQOL) of cardiac patients after rehabilitation. METHODS: N = 331 patients with chronic ischemic heart disease were surveyed using questionnaires at two time points (beginning and end of 3-weeks inpatient rehabilitation). In addition, characteristics of the disease and cardiac risk factors were provided by the physician. HRQOL was measured using a total of six scales and three instruments: SF-12, MacNew questionnaire, and SAQ. Hierarchical regression analyses were carried out to predict HRQOL after rehabilitation, in which the baseline values of HRQOL, sociodemographic variables, characteristics of the disease and risk factors, personality traits, and finally the aspects of the physician-patient relationship were included stepwise. As a number of variables were used for the regression models, multiple imputation was conducted. RESULTS: The baseline values explain most of the variance (42%-60%). After controlling the baseline values, the sociodemographic variables explain up to 5% incremental variance of HRQOL, with income being the most important predictor. The characteristics of the disease and cardiac risk factors explain between 0.4% and 3.8% incremental variance, however, variance increase is often not significant. The personality traits added in the fourth step explain up to 2% additional variance; trait anger is a significant predictor of HRQOL in three of the six scales. The features of the physician-patient relationship included in the last step lead to a significant increase in explained variance (between 1.3% and 3.9%) for all six scales. In particular, the physician's promotion of patient participation has a significant influence. The overall explanation of variance for HRQOL is between 50% and 64%. CONCLUSIONS: Low income, a high level of trait anger, and low patient participation are significant risk factors, even if a number of potential confounders are adjusted. Research is needed that shows which causal pathway low income functions on and what therapies in rehabilitation can mitigate the disadvantage of persons with a high level of trait anger. The providers should implement measures to actively integrate rehabilitation patients in treatment (e.g. encourage them to ask questions). | Male | Humans | Physician-Patient Relations | Questionnaires | OK |
| Wim J. | Dekkers | Glyn | Elwyn | Kris C. | Vissers | Palliative Medicine | 2013-01-00 Jan 2013 | BACKGROUND: Euthanasia has been legally performed in the Netherlands since 2002. Respect for patient's autonomy is the underpinning ethical principal. However, patients have no right to euthanasia, and physicians have no obligation to provide it. Although over 3000 cases are conducted per year in the Netherlands, there is little known about how decision-making occurs and no guidance to support this difficult aspect of clinical practice. AIM: To explore the decision-making process in cases where patients request euthanasia and understand the different themes relevant to optimise this decision-making process. DESIGN: A qualitative thematic analysis of interviews with patients making explicit requests for euthanasia, most-involved relative(s) and treating physician. PARTICIPANTS/SETTING: Thirty-two cases, 31 relatives and 28 treating physicians. Settings were patients' and relatives' homes and physicians' offices. RESULTS: Five main themes emerged: (1) initiation of sharing views and values about euthanasia, (2) building relationships as part of the negotiation, (3) fulfilling legal requirements, (4) detailed work of preparing and performing euthanasia and (5) aftercare and closing. CONCLUSIONS: A patient's request for euthanasia entails a complex process that demands emotional work by all participants. It is characterised by an intensive period of sharing information, relationship building and negotiation in order to reach agreement. We hypothesise that making decisions about euthanasia demands a proactive approach towards participants' preferences and values regarding end of life, towards the needs of relatives, towards the burden placed on physicians and a careful attention to shared decision-making. Future research should address the communicational skills professionals require for such complex decision-making. | Male | Decision Making | Humans | Physician-Patient Relations | OK |
| Susan T. | Nedorost |  |  |  |  | Dermatitis: Contact, Atopic, Occupational, Drug | 2010-10-00 2010 Sep-Oct | BACKGROUND: The efficacy of patch testing may be enhanced by data allowing the physician to estimate the likelihood that results of a patch-test reading are relevant to a patient's dermatitis. OBJECTIVE: The goal of this study was to compare the rates of agreement between the physician's assessment of relevance at the time of final reading and the patients' report 3 months to 3 years later in regard to whether avoidance of an allergen was needed to remain free of dermatitis. We hypothesize that the agreement rates between the physician and patient relevance assessments will vary based on properties both intrinsic and extrinsic to the allergen in question. METHODS: We mailed 407 Institutional Review Board-approved questionnaires to patients and analyzed results for the 92 patients reporting greater than 80% improvement of their dermatitis. Cross-reacting allergens tested on the same patient were combined for analysis. Percent agreement was used to assess interrater concordance. RESULTS: Percent agreement regarding relevance for each allergen or group of allergens was as follows: formaldehyde and formaldehyde-releasing preservatives, 88%; neomycin sulfate, 78%; nickel sulfate hexahydrate, 71%; fragrance mix and related products, 65%; and gold sodium thiosulfate, 56%. CONCLUSION: Relevance varies between allergens. Physician assessment of relevance at the time of final reading is not the ideal method for determining allergen relevance. This has implications for when best to determine the relevance of certain allergens. For allergens with lower agreement, in particular, extended follow-up is recommended to accurately determine an allergen's contribution to a patient's allergic contact dermatitis, especially in those circumstances in which a patient's condition has not improved. | Humans | Patient Participation | Questionnaires | Observer Variation | OK |
| Heather MacLeod | Williams | Alison J. | Sum | Patti-Jean | Naylor | British Journal of Sports Medicine | 2012-03-00 Mar 2012 | BACKGROUND: Healthcare providers are a primary source of information on preventive health issues for patients. Further research related to physical activity (PA) promotion in the primary care setting is needed. OBJECTIVE: To explore the feasibility of a physician and community PA intervention using a pedometer for: increasing PA levels among inactive patients; enhancing relationships between physicians and community PA partners; and influencing the PA promotion habits of physicians. METHODS: A 6-week PA intervention was delivered to 152 inactive patients who were recruited in physician waiting rooms, counselled by their physicians, provided with a pedometer and referred to a community action site (CAS) at their local recreation centre for further support. PA was measured at baseline and follow-up using the International Physical Activity Questionnaire. Follow-up interviews were conducted with physicians and recreation representatives on the challenges and benefits associated with the intervention. RESULTS: Study patients significantly increased their PA (103% change in MET min/week). Physicians and recreation professionals were highly satisfied with the intervention and partnership. There were challenges to ongoing communication. Physicians reported increased awareness of the pedometer and community resources as supports for PA. CONCLUSION: A pedometer-based PA intervention delivered by physicians in partnership with a community PA stakeholder is feasible and warrants further research. Long-term maintenance of this intervention would require resources for pedometers, and ongoing contact and communication between the physicians and CAS to ensure availability of community supports. | Male | Humans | Patient Participation | Questionnaires | OK |
| Jonathan D. | Fuchs | Mercé | Meroño | Ernesto | Sierra | AIDS and behavior | 2010-06-00 Jun 2010 | The purpose of this study was to assess HIV risk and willingness to participate in HIV vaccine trials in three high risk populations in Spain. Eight hundred and forty-four participants, comprising female sex workers, injection and non-injection drug users (IDUs and NIDUs, respectively), and men who have sex with men were tested for HIV and surveyed for risk and willingness to participate in future preventive HIV vaccine trials. HIV seroprevalence was 3.8% (95% CI: 2-11). HIV infection was associated with transgender identification, IDU in the past year, and sex with an IDU or other drug-using partner. The majority (82%) expressed their willingness to participate in HIV vaccine trials. Substantial sexual and parenteral risk in all groups and concomitant willingness to participate in vaccine trials was found, particularly among women and IDUs. Additional longitudinal cohort studies in Spain are needed to plan future vaccine efficacy trials. | Male | Humans | Patient Participation | Questionnaires | OK |
| Carolyn | Goldberg | Richard | Gusberg | Liana | Fraenkel | Journal of Vascular Surgery | 2011-02-00 Feb 2011 | OBJECTIVE: Patients with abdominal aortic aneurysms (AAAs) who are surgical candidates have as many as three options: open surgery, endovascular surgery, or no surgery. As with all treatment decisions, informed patient preferences are critical. Decision support tools have the potential to better inform patients about the risks and benefits associated with each treatment option and to empower patients to participate meaningfully in the decision-making process. The objective of this study was to develop and pilot test a decision support tool for patients with AAAs. METHODS: We developed a personalized, interactive, computer-based decision support tool reflecting the most current outcomes data and input from surgeons and patients. We piloted the tool with AAA repair candidates who used the tool prior to meeting with their surgeon. Patients were recruited from a university-based vascular surgery clinic and affiliated VA hospital clinic. To determine feasibility and acceptability, the following outcomes were measured: (1) percent of patients who agreed to participate, (2) length of time required to use the tool, (3) the amount of assistance required to use the tool, and (4) patients' opinions on the acceptability of the tool. To assess effectiveness of the tool, we measured change in knowledge and decisional conflict pre- and post-tool using the paired t-test. RESULTS: One hundred percent of patients who were approached (n = 12) agreed to participate in the study. The tool was administered in a median time of 35 minutes (range, 25-45 minutes), and all patients were able to navigate the program with minor technical assistance. Mean knowledge scores increased from 56% to 90% (P = .005), and decisional conflict scores decreased from 29% to 8% (P = .04). Overall, patients reported that the program content was balanced across treatment options, presented information clearly and concisely, helped them to organize their thoughts about the decision, and prepared them to talk to their surgeon about what mattered most to them. CONCLUSIONS: Preliminary evidence suggests that use of an evidence-based AAA decision support tool is feasible and acceptable to patients, increases knowledge, and decreases decisional conflict. Widespread use of such a tool might improve the content and quality of informed consent for this difficult treatment decision. | Time Factors | Male | Humans | Patient Participation | OK |
| Beata | Wożakowska-Kapłon | Grzegorz | Opolski | Piotr | Ponikowski | Kardiologia Polska | 2013-00-00 2013 | BACKGROUND: Heart failure (HF) is currently one of the main causes of cardiovascular mortality. In order to collect current epidemiological data on patients with HF, the Heart Failure Pilot Survey (ESC-HF Pilot) registry was initiated. AIM: Primary objective of the study was to compare clinical epidemiology of outpatients and inpatients with HF and investigate currently used diagnostic and therapeutic modalities in Poland and 11 other European countries. METHODS: The ESC-HF Pilot Survey study was a prospective multicentre observational registry conducted in 2009-2011 in 136 cardiology centres in 12 European countries selected to represent different health systems and care attitudes across Europe. All outpatients with HF and patients admitted due to acute decompensated HF were included into the registry during the enrolment period (1 day per week for 8 consecutive months). Researchers completed detailed medical data questionnaires for all HF patients recruited to the study. RESULTS: In all participating centres across Europe, 6108 patients were recruited, including 1159 patients from Poland (19% of the survey population). The majority of Polish participants were admitted due to acute HF (73%), while ambulatory chronic HF patients predominated in the remaining European centres (69%). Polish patients develop HF at a younger age compared to other European countries (proportion of patients above 65 years: 54 vs. 65%, respectively) and they are more severely ill (NYHA class III: 44 vs. 34%, respectively; NYHA class IV: 18 vs. 11%; mean BNP level 910 vs. 773 pg/mL). Angiographically documented coronary artery disease was the major aetiology of HF in Poland (39 vs. 33%) which explains a higher rate of invasive revascularisation procedures in the Polish population (13 vs. 7%). In Poland, therapy with implantable cardioverter- -defibrillators was used more frequently during the initial hospitalisation (7 vs. 4%), but the rate of cardiac resynchronisation therapy device implantation was smaller than in other European countries (4 vs. 7%). Drug therapy used in our country was comparable to the rest of Europe, except for more frequent use of aldosterone antagonists. Despite significant differences in the clinical characteristics seen between Polish and other European patients participating in the ESC-HF Pilot study, mortality at 3 months did not differ between Polish and other European centres (2.5 vs. 3%). CONCLUSIONS: The ESC-HF Pilot Survey findings indicate a very high standard of inpatient HF treatment but at the same time unsatisfactory current ambulatory HF therapy in Poland. | Male | Humans | Patient Participation | Female | OK |
| Bertrand | Berger |  |  |  |  | Community Mental Health Journal | 2013-12-00 Dec 2013 | Disengagement from outpatient care following psychiatric hospitalization is common in high-utilizing psychiatric patients and contributes to intensive care utilization. To investigate variables related to treatment attrition, a range of demographic, diagnostic, cognitive, social, and behavioral variables were collected from 233 veterans receiving inpatient psychiatric services who were then monitored over the following 2 years. During the follow-up period, 88.0 % (n = 202) of patients disengaged from post-inpatient care. Attrition was associated with male gender, younger age, increased expectations of stigma, less short-term participation in group therapy, and poorer medication adherence. Of those who left care, earlier attrition was predicted by fewer prior-year inpatient psychiatric days, fewer lifetime psychiatric hospitalizations, increased perceived treatment support from family, and less short-term attendance at psychiatrist appointments. Survival analyses were used to analyze the rate of attrition of the entire sample as well as the sample split by short-term group therapy attendance. Implications are discussed. | Male | Humans | Patient Participation | Patient Satisfaction | OK |
| Mark I. | van Berge Henegouwen | Hanneke C. J. M. | de Haes | Mirjam A. G. | Sprangers | Patient Education and Counseling | 2012-07-00 Jul 2012 | OBJECTIVE: Given the poor prognosis of esophageal cancer and the impact of surgery on health-related quality of life (HRQL), addressing patients' postoperative information needs is important. This study aimed to examine (1) the content and type of patients' information needs and (2) patient perceived facilitators and barriers to patient participation. METHODS: Interviews were conducted with 20 purposefully selected esophageal cancer patients. Open and structured questions were alternated. The transcribed interviews were analysed inductively and deductively, using MAXqda. RESULTS: Patients' post-operative information needs concerned HRQL, medical care and prognosis, covering several sub-domains. Different types of needs were identified, e.g., requests for information about cause, course and self-management. Barriers to patient participation mostly reflected beliefs and skills, and could be categorized into agenda and communication barriers. Facilitators of patient participation reflected physician, patient and interaction characteristics, companion support and pre-visit preparation. Many patients saw merit in pre-visit preparation interventions; few endorsed skill-building interventions. CONCLUSION: This study listed the postoperative information needs of esophageal cancer patients. Moreover, it gained insight into patient-perceived barriers and facilitators of patient participation. PRACTICE IMPLICATIONS: The findings demonstrate what information physicians should have available and informs interventions to support patients in meeting their information needs. | Male | Humans | Patient Participation | Physician-Patient Relations | OK |
| Ricardo A. | Cruciani |  |  |  |  | Journal of Opioid Management | 2014-02-00 2014 Jan-Feb | OBJECTIVES: This survey highlights the perspective of patients with chronic pain when confronted with the possibility of abrupt opioid discontinuation. DESIGN: Anonymous and self-administered survey. SETTING: Pain Clinic in an urban academic hospital setting. PARTICIPANTS: Patients with chronic pain of at least 6 months, and age 18 years or older. MAIN OUTCOME MEASURES: The perceived impact of abrupt opioid discontinuation on mood, daily functioning, and potential for opioid misuse. RESULTS: When patients were asked how they would feel if told their opioids would be discontinued, the responses were neglected (scale mean, 7.1), angry (6.3), helpless (6.7), and upset (7.0) on a scale of 0-10. The majority predicted loss of independence in activities of daily living (scale mean, 7.5), inability to enjoy their lives (7.4), and inability to work (7.8). A group of 19 patients (11.6 percent) reported that they would obtain opioids from friends or family, and 7 (4.4 percent) would obtain them illegally. These patients reported higher scores of neglect, anger, suspicion, helplessness, and upset (p = 0.000-0.019) and were more likely to have previously obtained opioids illegally (p = 0.008-0.023). The most common nonopioid strategies tried by patients were lumbar epidural (71.5 percent), physical therapy (78.9 percent), and exercise (83.5 percent). The strategies considered to be effective as opioid replacement therapy were lumbar epidural (42.3 percent), exercise (43.2 percent), and massage (42.0 percent). CONCLUSIONS: These data suggest that opioid therapy discontinuation is an emotionally distressing experience for most patients, and the transition to nonopioid treatments is a complex process that will require patient participation to achieve optimal care. | Male | Humans | Questionnaires | Female | OK |
| Marcos | Ballester | Emile | Daraï |  |  | BMC urology | 2012-00-00 2012 | BACKGROUND: To evaluate prolapse-related symptoms, quality of life and sexuality of patients with validated questionnaires before and after surgery for genital prolapse and assess relevance of such an evaluation to select women for surgery. METHODS: From November 2009 to April 2010, 16 patients operated on for genital prolapse of grade greater than or equal to 2 (POP-Q classification) were evaluated prospectively by three questionnaires of quality of life Pelvic Floor Distress Inventory (PFDI-20), Pelvic Floor Impact Questionnaire (PFIQ-7) and Pelvic Organ Prolaps/Urinary Incontinence Sexual Questionnaire (PISQ-12). Data were collected the day before surgery and 6 weeks postoperatively. RESULTS: Eleven patients had laparoscopic surgery and five vaginal surgery. There was a significant decrease in pelvic heaviness, vaginal discomfort and urinary symptoms after surgery. The score of symptoms of prolapse, the PFDI-20 score was 98.5 preoperatively and 31.8 postoperatively (p < 0.0001). The score for quality of life, the PFIQ-7 score was 54.5 preoperatively and 7.4 postoperatively (p = 0.001). The score of sexuality, the PISQ-12 score was 35.3 preoperatively and 37.5 postoperatively (p = 0.1). Two of the 3 patients with a PFIQ 7 under or equal to 20 were not improved while all the women with a preoperative PFIQ-7 over 20 were improved after surgery. CONCLUSIONS: This study suggests that surgery improves quality of life of patients with genital prolapse. Quality of life questionnaires could help select good candidates for surgery. Further studies are required to determine threshold to standardize indications of surgery. | Humans | Patient Participation | Questionnaires | Female | OK |
| Javier F. | Magrina |  |  |  |  | American Journal of Obstetrics and Gynecology | 2015-01-00 Jan 2015 | Power morcellation has come under scrutiny because of a highly publicized case of disseminated leiomyosarcoma following a laparoscopic hysterectomy. A recent Federal and Drug Administration safety communication discouraging use of power morcellators on presumed uterine leiomyoma further highlights the need for reexamination of uterine tissue extraction. This clinical opinion aims to summarize current approaches to uterine/fibroid tissue extraction including the associated immediate and long-term potential risks of open power morcellation. The known data about risk of uterine sarcoma is reviewed followed by a discussion of acceptable risk and informed consent in the context of shared-decision making. | | | | | OK |
| L. | Rossignol | T. | Blanchon | S. | Kernéis | Médecine Et Maladies Infectieuses | 2012-09-00 Sep 2012 | OBJECTIVES: The authors assessed the knowledge and practices of French family physicians concerning the application of the new 2007 varicella vaccination guidelines for non-immune teenagers, 12 to 18 years of age. They also estimated the vaccination coverage in this population. METHOD: A questionnaire link was sent by to 1008 family physicians of the French Inserm Sentinel network. Each family physician had to include the last teenager aged 12 to 18 years seen in consultation, with no or uncertain history of clinical varicella. RESULTS: One hundred and forty-one family physicians agreed to participate and included one patient (participation rate=14%) between 4th November 2010 and 4th January 2011. One hundred and thirty-three questionnaires out of 141 (94%) were analyzed. Three patients were vaccinated and 127 were not, giving a weak vaccination coverage in the investigated population at 2%. Eighty-nine family physicians (70%) did not know about the recommendation, and 90 (71%) declared that they had no intention to vaccinate their patient against varicella. CONCLUSION: Guidelines on varicella vaccination of non-immune teenagers are poorly followed and accepted by family physicians. Vaccination coverage is very low, and efforts should be made to improve application of recommendations. | Male | Humans | Questionnaires | Female | OK |
| M. R. | Partridge |  |  |  |  | Clinical Medicine (London, England) | 2012-04-00 Apr 2012 | Patients referred to secondary care for specialist respiratory review frequently undergo multiple hospital attendances for investigations and consultations. This study evaluated the potential of a preclinic telephone consultation and subsequent coordination of tests and face-to-face consultations to reduce hospital visits. Total hospital attendances were recorded for three cohorts (participants, non-participants and comparators) for 6 months from first specialist contact. Patients completed the medical interview satisfaction scale-21 (MISS-21). The study showed that a preclinic telephone consultation can significantly reduce hospital visits over a fixed period without reducing patient satisfaction. In total, 20.8% of the participant group had three or more hospital attendances compared with 42.9% of the non-participant group (p = 0.001) and 44.7% of the comparator group (p = 0.002). Participants had fewer follow up visits and lower rates of non-attendance/late rearrangement of appointments. This service reduces unnecessary hospital visits, seems to improve patient compliance and may save costs associated with non-attendance and follow up consultations. | Male | Humans | Patient Participation | Patient Satisfaction | OK |
| Kristi M. | Logue | Karen | Glanz | Boadie W. | Dunlop | Journal of Clinical Epidemiology | 2011-08-00 Aug 2011 | OBJECTIVE: To determine whether preconsent education about research processes and protections affects the willingness of African Americans to participate. STUDY DESIGN AND SETTING: This study examined the willingness of 192 African American outpatients (stratified by age, gender, and education) to participate in a hypothetical clinical study under varying consent conditions: phase I participants underwent a typical informed consent process and were asked to indicate whether they would be willing to participate in the hypothetical clinical study and the reasons for their decision; their responses were used to develop a preconsent educational digital video disk (DVD). Phase II participants viewed the DVD before the consent process. We compared the proportion of those who stated they were willing to participate in the clinical study using Fisher's exact tests and used qualitative methods to analyze open-ended responses. RESULTS: When the consent process included education about research processes and protections, significantly more patients reported willingness to participate in the hypothetical clinical study (43% vs. 27%; P=0.002). Patients receiving preconsent education were significantly less likely to cite mistrust, fear of side effects, lack of perceived benefits, and privacy as reasons for not participating. CONCLUSION: Preconsent education may improve the willingness of African Americans to participate in clinical research and may address important concerns about research participation. | Male | Humans | Patient Participation | Questionnaires | OK |
| Mieke | Rijken | Monique | Heijmans | Ad A. | Kaptein | BMC nephrology | 2010-00-00 2010 | BACKGROUND: Compared to healthy people, patients with chronic kidney disease (CKD) participate less in paid jobs and social activities. The aim of the study was to examine a) the perceived autonomy, self-esteem and labor participation of patients in the pre-dialysis phase, b) pre-dialysis patients' illness perceptions and treatment perceptions, and c) the association of these perceptions with autonomy, self-esteem and labor participation. METHODS: Patients (N = 109) completed questionnaires at home. Data were analysed using bivariate and multivariate analyses. RESULTS: The results showed that the average autonomy levels were not very high, but the average level of self-esteem was rather high, and that drop out of the labor market already occurs during the pre-dialysis phase. Positive illness and treatment beliefs were associated with higher autonomy and self-esteem levels, but not with employment. Multiple regression analyses revealed that illness and treatment perceptions explained a substantial amount of variance in autonomy (17%) and self-esteem (26%). The perception of less treatment disruption was an important predictor. CONCLUSIONS: Patient education on possibilities to combine CKD and its treatment with activities, including paid work, might stimulate positive (realistic) beliefs and prevent or challenge negative beliefs. Interventions focusing on these aspects may assist patients to adjust to CKD, and ultimately prevent unnecessary drop out of the labor market. | Male | Humans | Patient Participation | Questionnaires | OK |
| L. V. | Gabis | I. | Morag |  |  | Research in Developmental Disabilities | 2011-12-00 2011 Nov-Dec | The aim of the study was to prospectively assess the differences in participation and sensory modulation between late preterm infants (LPI) and term babies, and to predict it by LPI characteristics. The study population includes 124 late preterm infants at gestational age between 34 and 35 6/7 weeks who were born at the same medical center. The control group comprised of 33 term babies (18 boys, 15 girls), born during the same period and location (mean age 12.47, SD = 0.73). Sensory modulation was assessed by the test of sensory functions in infants and the infant/toddler sensory profile and for assessment of participation and parents' satisfaction we used questionnaires. Term infants had better sensory modulation than LPI. Approximately 10% of the sensory modulation of participants in the study was explained by gestational age and head circumference. LPI participation and parental satisfaction decreased in the LPI group. Among all the explanatory variables only multiple gestations and head circumference contributed to the explained variance of participation (16%), and parents' satisfaction (13%). At age of 1 year, children born as late preterm are at increased risk of developing sensory modulation disorder, showing less participation, and resulting in less parental satisfaction. | Male | Humans | Patient Participation | Questionnaires | OK |
| Eun-Jung | Shim | Myung-Il | Hahm | Jong-Hyock | Park | Supportive Care in Cancer: Official Journal of the Multinational Association of Supportive Care in Cancer | 2012-09-00 Sep 2012 | BACKGROUND: Shared decision making and an integrative approach is expected to result in better outcomes, but might require more time. While ensuring that sufficient consultation time is essential to quality cancer care, it is not clear whether cancer patients feel that the amount of consultation time with their oncologists is sufficient. METHODS: Data were collected from 2,556 cancer patients on their perceived and preferred consultation time, and included potential predictors, such as socio-demographics, clinical, behavioral, and quality-of-life factors, as well as potential outcomes, including unmet patient needs, trust in doctor, and satisfaction. The feeling of insufficient consultation time was defined as the perception that consultation time is less than the preferred consultation time; multivariate analyses were used for identification of predictors and comparison of outcomes. RESULTS: Overall, 37.1% felt that consultation time was less than preferred. Younger age, female sex, higher education level, having national health insurance, having been diagnosed with less common cancers, and having anxiety/depression were significantly associated with feelings of insufficient consultation time. Subjects with a feeling of insufficient consultation time reported higher current needs for information, physical symptoms, and psychological problems. They also reported less trust in their physician, lower overall satisfaction, and lower intention to continue treatment at the current cancer center. CONCLUSION: This study illustrated that high-quality cancer care, characterized by shared decision making and an integrative approach, seems to be related to sufficient consultation time that meets the individual's subjective needs, and measures should be taken to ensure sufficient consultation time. | Time Factors | Male | Decision Making | Humans | OK |
| L. R. | Young | J. T. | Hagaman | N. | Oprescu | Respiratory Medicine | 2010-04-00 Apr 2010 | BACKGROUND: Lymphangioleiomyomatosis (LAM) is a rare, progressive and frequently lethal cystic lung disease that almost exclusively affects women and has no proven therapies. An improved understanding of the pathogenesis has identified promising molecular targets for clinical trials. Although barriers, modifiers, and benefits for clinical trial participation in common diseases such as cancer have been studied, we are unaware of such evaluations concerning rare diseases. METHODS: We performed a survey of a population-based registry of 780 LAM subjects in North America to identify predictors of trial participation. Logistic regression analysis evaluated the association of demographic and clinical features with trial participation. RESULTS: 41 of 263 (16%) LAM patient respondents in North America had participated in a clinical trial. Age, disease duration, lack of any college education, use of oxygen therapy, and presentation without chest pain were associated with trial participation in unadjusted analyses. Multivariate analyses indicate that patient age was the strongest independent predictor for trial participation (OR=2.07, p=0.004 per decade greater of patient age). Common reasons reported against trial participation included not meeting enrollment criteria (44%), drug toxicity (25%), and stable disease (20%). The most frequent reason reported for trial participation was to help future patients (85%). CONCLUSIONS: Study entry criteria, drug toxicity, and stability of disease are barriers to trial enrollment among subjects with LAM. Older LAM patients and those with more advanced disease are more likely to have participated in clinical trials. Altruism is commonly a motivating factor. | Humans | Patient Participation | Questionnaires | Female | OK |
| Christian | Blickem | Anne | Kennedy | David | Reeves | BMC family practice | 2014-11-30 Nov 30, 2014 | BackgroundImproving the quality of care for patients with vascular disease is a priority. Clinical guidance has emphasised the importance of early identification and active management of chronic kidney disease (CKD) in primary care in order to maintain vascular health. However, awareness of stage 3 CKD amongst patients remains limited. We aimed to identify predictors of patient self-report of CKD to inform tailoring of conversations around CKD in primary care for diverse patient populations.MethodsWe conducted a cross-sectional analysis of baseline data from 436 patients with stage 3 CKD from 24 GP practices taking part in a randomised controlled trial (RCT) evaluating a complex self-management intervention, which aimed to support the maintenance of vascular health in patients with stage 3 CKD. Potential predictors of patient self-report of CKD included demographics, stage of CKD, cardiovascular risk, self-reported co-morbidities, health status, self-management ability, and health service utilisation.ResultsAround half (52%, n =227) of patients did not self-report CKD. Self-report rates did not appreciably differ by practice. Multivariate analysis revealed that female patients (p¿=¿0.003), and patients with stage 3b CKD (p¿<¿0.001), and with higher anxiety levels (p¿<¿0.001), were more likely to self-report CKD.ConclusionsSelf-report of kidney problems by patients on CKD registers was variable and patterned by sociodemographic factors. Although it cannot be assumed that failure to self-report indicates a lack of awareness of CKD, our data do suggest the need for greater consistency in discussions around kidney health, with meaningful and relevant clinical dialogue that is aligned with existing clinical encounters to enable shared decision making and minimise anxiety. | | | | | OK |
| Wenyan | Zhao | Tor | Tosteson | Alan S. | Hilibrand | Spine | 2012-09-01 Sep 1, 2012 | STUDY DESIGN: A retrospective cohort study. OBJECTIVE: In this article, we examined the Spine Patient Outcomes Research Trial lumbar stenosis observational cohort to determine baseline patient characteristics that are predictive of the treatment patients chose. We also evaluated cutoff points on validated patient questionnaires that differentiate patients who chose surgery from those who chose nonsurgical management. SUMMARY OF BACKGROUND DATA: Although the evidence from current studies suggests that surgical intervention is effective for lumbar spinal stenosis, the same studies show that nonoperative patients also improve. Thus, the reasons for patients choosing surgery versus nonoperative care are of continuing interest. METHODS: Baseline patient and clinical characteristics between those who received operative intervention and those who received nonoperative care were compared to determine baseline predictors of lumbar spinal stenosis management. Also, an evaluation of responses to the 36-Item Short Form Health Survey Bodily Pain (BP), 36-Item Short Form Health Survey Physical Function (PF), and the modified Oswestry Disability Index (ODI) questionnaires was performed to determine the percentage of patients choosing surgical versus nonoperative care relative to their initial questionnaire values. RESULTS: This analysis looked at the 356 patients in the observational spinal stenosis cohort of Spine Patient Outcomes Research Trial who completed at least 1 follow-up visit. Patients choosing surgery were younger (P = 0.022), had worse BP (P < 0.001), worse PF (P < 0.001), worse ODI (P < 0.001), worse Stenosis Bothersomeness Index (P < 0.001), were dissatisfied with their symptoms (P = 0.001), and had a worse self-assessed health trend (P < 0.001). Patients tended to choose surgery if they had lateral recess stenosis (P = 0.022). Kaplan-Meier curves demonstrate that patients with a BP score of 32 or less, PF score of 30 or less, and ODI greater than 29 chose surgery 75% of the time. CONCLUSION: A greater understanding of baseline characteristics that influence patient choices in the treatment of lumbar spinal stenosis can aid the patient and the surgeon during the shared decision-making process. | Male | Decision Making | Humans | Questionnaires | OK |
| Robert L. | Grant | Clive | Seale | Richard | Ashcroft | BMC medical research methodology | 2012-00-00 2012 | BACKGROUND: Longitudinal studies are crucial providers of information about the needs of an ageing population, but their external validity is affected if partipants drop out. Previous research has identified older age, impaired cognitive function, lower educational level, living alone, fewer social activities, and lower socio-economic status as predictors of attrition. METHODS: This project examined attrition in participants of the Whitehall II study aged between 51-71 years, using data from questionnaires participants have completed biennially since 1985 when the study began. We examine the possibility of two distinct forms of attrition--non-response and formally requesting to withdraw--and whether they have different predictors. Potential predictors were age, gender, marital status, occupational grade, retirement, home ownership, presence of longstanding illness, SF-36 quality of life scores, social participation and educational level comparing participants and those who had withdrawn from the study. RESULTS: The two forms of attrition share many predictors and are associated but remain distinct. Being older, male, having a lower job grade, not being a home owner, not having a long standing illness, having higher levels of education, and not having retired, were all associated with a greater probability of non-response; being married was associated with higher probability in women and lower in men. Being older, male, having a lower job grade, not being a home owner, having lower SF-36 scores, taking part in fewer social activities, and not having a long standing illness, were all associated with greater probability of withdrawal. CONCLUSIONS: The results suggest a strong gender effect on both routes not previously considered in analyses of attrition. Investigators of longitudinal studies should take measures to retain older participants and lower level socio-economic participants, who are more likely to cease participating. Recognition should be given to the tendency for people with health problems to be more diligent participants in studies with a medical screening aspect, and for those with lower socio-economic status (including home ownership), quality of life and social participation, to be more likely to request withdrawal. Without taking these features into account, bias and loss of power could affect statistical analyses. | Male | Humans | Patient Participation | Questionnaires | OK |
| Stephanie | Wagner | Thomas C. | Havighurst | Jane A. | McElroy | Clinical Medicine & Research | 2013-09-00 Sep 2013 | OBJECTIVE: We conducted a pilot survey to evaluate breast cancer patients' willingness to participate in a preoperative chemoprevention (ie, window-of-opportunity) study. Design A 27-question written survey was developed and administered to participants. Setting A breast cancer specialty clinic at the University of Wisconsin Hospital and Clinics. Participants 30 adult patients with newly diagnosed operable breast cancer participated after signing informed consent. METHODS: A convenience sample of 30 participants was recruited from July 2005 through January 2006. Participants were administered the survey in clinic. Univariate ordinal logistic regression models were used to identify predictors of willingness to participate in window-of-opportunity trials. RESULTS: Overall, 26.7% of respondents were willing to participate in a research trial between the time of breast cancer diagnosis and surgery. Univariate ordinal logistic regression models identified that women with a prior history of breast cancer (P=0.060), prior research participation (P=0.006), more education (P=0.034), and self-reported breast cancer knowledge (P=0.043) were more willing to participate. On average, women preferred to have surgery 7 days (range 1-14) after their diagnosis, but the actual average wait time between diagnostic biopsy and surgery was 37.5 days (standard deviation = 23.4 days). CONCLUSION: There is ample time before breast surgery to conduct preoperative window-of-opportunity trials. Interventions aimed at expanding patients' breast cancer knowledge may improve accrual to window-of-opportunity studies. | Humans | Patient Participation | Questionnaires | Female | OK |
| H. | Raspe |  |  |  |  | Zeitschrift Für Gastroenterologie | 2012-04-00 Apr 2012 | BACKGROUND: Evidence-based and consented pathways for patients with inflammatory bowel diseases (IBD, Crohn's disease, ulcerative colitis) call for tailored education programmes to foster shared decision-making and patient self-management. Their preferences should be taken into account. METHODS: In 2005 a cross-sectional postal questionnaire survey was conducted in different regions of Germany. Adult patients with ulcerative colitis (UC) or Crohn's disease (CD) were recruited from specialised gastroenterological practices, university outpatient clinics and the member registry of the relevant patient organisation DCCV. They returned a questionnaire including (inter)nationally established scales (e. g., HADS) as well as questions on their information needs, preferred information sources and their role in decision-making. RESULTS: Data of 1056 responders could be analysed (65 % female; CD: 58 %; DCCV member: 71 %). The mean age of the patients was 42 (SD 12,8) years. Almost all patients wanted more information on "treatment alternatives" (83 %), "causes of disease" (80 %) and "what can I (still) do by myself" (79 %). 27 % of the patients asked for more information on 15 or more of overall 19 topics. The high information need was significantly associated with HADS potential depressive disorder (score > 8) and HADS probable anxiety disorder (score > 10). Most IBD patients (70 %) obviously regard their physicians as the most desirable source of information, 67 % prefer an active involvement in clinical decision-making. CONCLUSIONS: The data demonstrate high information needs of IBD patients and may serve in the planning of future educational programmes. | Male | Decision Making | Humans | Patient Participation | OK |
| Mieke | Rijken |  |  |  |  | Patient Education and Counseling | 2011-07-00 juillet 2011 | Objective: (1) To describe the importance chronically ill and disabled patients attach to involvement in decision-making when various care types are considered, and (2) to analyse the degree to which these patients are involved in shared decision-making (SDM) regarding these care types, and whether their involvement reflects the importance they attach to SDM. Methods: The study sample consisted of 812 chronically ill and disabled patients who experienced a situation of decision-making during the last year. Data were collected by a self-report survey in 2006 and were analysed by multilevel linear regression analyses. Results: Participants attached most importance to SDM when occupational healthcare issues were at stake, but perceived their actual involvement in these decisions as relatively low. Patients dealing with decision-making regarding medical care or home care experienced higher levels of involvement. The importance attached to SDM corresponds moderately with the actual role patients experience in the decision-making process. Conclusion: The type of care to decide upon impacts on the importance patients attach to SDM as well as on their actual involvement in decision-making. Practice implications: We suggest healthcare practitioners to pay attention to the preferred level of patient involvement each time a new care issue has to be decided upon. (PsycINFO Database Record (c) 2012 APA, all rights reserved). (journal abstract) | Decision Making | self report | chronically ill | occupational health care | OK |
| Marguerite | Ennis | Navjot | Brar | Alexandria R. | Maida | BMC cancer | 2010-00-00 2010 | BACKGROUND: Intrinsic to "Patient-Centered Care" is being respectful and responsive to individual patient preferences, expressed needs, and personal values. Establishing a patient's preferences for active and aggressive intervention is imperative and foundational to the development of advance care planning. With the increasing awareness and acceptance of palliative philosophies of care, patients with advanced cancer are increasingly transitioning from active and aggressive medical management (AAMM) to conservative palliative management (CPM). METHODS: A cross-sectional study based on a prospective and sequential case series of patients referred to a regional palliative medicine consultative program was assembled between May 1, 2005 and June 30, 2006. Patients and/or their substitute decision makers (SDM) completed a questionnaire, at baseline, that assessed their preferences for AAMM en route to their eventual deaths. Seven common interventions constituting AAMM were surveyed: cardiopulmonary resuscitation (CPR) & mechanical ventilation (MV), chemotherapy, antibiotics, anticoagulants, blood transfusions, feeding tubes, and artificial hydration. Multivariable analyses were conducted on the seven interventions individually as well as on the composite score that summed preferences for the seven interventions. RESULTS: 380 patients with advanced cancer agreed to participate in the study. A trend to desire a mostly conservative palliative approach was noted as 42% of patients desired one or fewer interventions. At baseline, most patients and their SDM's were relatively secure about decisions pertaining to the seven interventions as the rates of being "undecided" ranged from a high of 23.4% for chemotherapy to a low of 3.9% for feeding tubes. Multivariable modeling showed that more AAMM was preferred by younger patients (P < 0.0001), non-Caucasians (P = 0.042), patients with higher baseline Palliative Performance Scale scores (P = 0.0002) and where a SDM was involved in the decision process (p = 0.027). Non-statistically significant trends to prefer more AAMM was observed with male gender (p = 0.077) and higher levels of the Charlson Comorbidity index (p = 0.059). There was no association between treatment preferences and cancer class. CONCLUSIONS: Although the majority of patients with advanced cancer in this study expressed preferences for CPM, younger age, higher baseline PPSv2, and involvement of SDMs in the decision process were significantly associated with preferences for AAMM. | Male | Decision Making | Humans | Patient Participation | OK |
| Claire | Julian-Reynier | Didier | Bechlian | Norbert | Vey | Journal of the American Geriatrics Society | 2014-06-00 Jun 2014 | | Male | Humans | Patient Participation | Questionnaires | OK |
| Robert Vander | Stichele | Jan L. | Bernheim | Freddy | Mortier | Journal of Palliative Medicine | 2010-10-00 Oct 2010 | OBJECTIVE: To explore the preferences of competent patients with advanced lung cancer regarding involvement of family and/or others in their medical decision-making, and their future preferences in case of loss of competence. METHODS: Over 1 year, physicians in 13 hospitals in Flanders, Belgium, recruited patients with initial non-small–cell lung cancer, stage IIIb or IV. The patients were interviewed with a structured questionnaire every 2 months until the fourth interview and every 4 months until the sixth interview. RESULTS: At inclusion, 128 patients were interviewed at least once; 13 were interviewed 6 consecutive times. Sixty-nine percent of patients wanted family members to be involved in medical decision-making and this percentage did not change significantly over time. One third of these patients did not achieve this preference. Ninety-four percent of patients wanted family involvement if they lost competence, 23% of these preferring primary physician control over decision-making, 41% shared physician and family control, and 36% primary family control. This degree of preferred family involvement expressed when competent did not change significantly over time at population level, but did at individual level; almost half the patients changed their minds either way at some point during the observation period. CONCLUSIONS: The majority of patients with lung cancer wanted family involvement in decision-making, and almost all did so in case of future loss of competence. However, as half of the patients changed their minds over time about the degree of family involvement they wanted if they lost competence, physicians should regularly rediscuss a patient's preferences. | Male | Decision Making | Humans | Patient Participation | OK |
| Caridad | Dios Guerra | Celia | Jiménez García | Valeria | González Neubauer | Enfermería Clínica | 2011-06-00 2011 May-Jun | AIMS: To assess patient preferences their satisfaction level and their participation in decision making with nurses. METHODS: Cross-sectional and mixed quantitative-qualitative study carried out in people attending the nursing services of 9 Health Centres in Andalusia. Patients were interviewed immediately after receiving nursing treatment using two different questionnaires for assessing their opinions, satisfaction and perception of involvement in the decisional process. A descriptive analysis using the χ(2) test (P<.05) was performed to assess the differences among close-ended and open-ended questions. Qualitative analysis: Open-ended questions were grouped into categories by a process involving three researchers independently. RESULTS: A total of 235 patients took part, of whom 59% (138) preferred a collaborative role with the nurse when making decisions. In the closed questions, 96.2% (228) of the surveyed patients declared to be satisfied or very satisfied with the decision making process; nevertheless 17.4% (41) made specific suggestions for improving this process. For them the main improvement areas were related to: general communication skills or a more specific one such as: strategies for helping them make decisions, reaching common ground or giving advice. CONCLUSIONS: Nurses should be aware that most patients wish to be involved in decision making and in clinical practice this participation can be improved by obtaining specific communicational skills. Surveys that include open-ended questions are more useful to assess the quality of care. | Male | Decision Making | Humans | Patient Participation | OK |
| Pamela J. | Atherton | Tenbroeck | Smith | Thomas F. | Hack | The American Journal of Managed Care | 2010-09-00 Sep 2010 | OBJECTIVES: To collect normative data, assess differences between demographic groups, and indirectly compare US and Canadian medical systems relative to patient expectations of involvement in cancer treatment decision making. STUDY DESIGN: Meta-analysis. METHODS: Individual patient data were compiled across 6 clinical studies among 3491 patients with cancer who completed the 2-item Control Preferences Scale indicating the roles they preferred versus actually experienced in treatment decision making. RESULTS: The roles in treatment decision making that patients preferred were 26% active, 49% collaborative, and 25% passive. The roles that patients reported actually experiencing were 30% active, 34% collaborative, and 36% passive. Roughly 61% of patients reported having their preferred role; only 6% experienced extreme discordance between their preferred versus actual roles. More men than women (66% vs 60%, P = .001) and more US patients than Canadian patients (84% vs 54%, P <.001) reported concordance between their preferred versus actual roles. More Canadian patients than US patients preferred and actually experienced (42% vs 18%, P <.001) passive roles. More women than men reported taking a passive role (40% vs 24%, P <.001). Older patients preferred and were more likely than younger patients to assume a passive role. CONCLUSIONS: Roughly half of the studied patients with cancer indicated that they preferred to have a collaborative relationship with physicians. Although most patients had the decision-making role they preferred, about 40% experienced discordance. This highlights the need for incorporation of individualized patient communication styles into treatment plans. | Male | Decision Making | Humans | Patient Participation | OK |
| Lena | Marions | Susanne Georgsson | Ohman |  |  | Sexual & Reproductive Healthcare: Official Journal of the Swedish Association of Midwives | 2013-06-00 Jun 2013 | OBJECTIVES: The aims of the study were to explore factors influencing the decision to continue or terminate pregnancy due to detection of fetal malformation following ultrasound examination, to elucidate the need for more information or other routines to facilitate the decision-making process and to assess satisfaction with the decision made. DESIGN: Descriptive study. SETTING: Four fetal care referral centres in Stockholm, Sweden. POPULATION: Pregnant women with a detected fetal malformation. METHODS: Data was collected by questionnaires. 134 women participated, 99 completing the questionnaire. Descriptive statistical analysis was performed. RESULTS: Both women who continued and those who terminated pregnancy based their decision on the severity of the malformation. Other reasons for terminating the pregnancy were aspects including socioeconomic considerations. None stated religious factors. The doctor at the fetal care unit also had an influence on the decision-making. The timeframe receiving information was regarded as long enough in duration but not the number of occasions. In both groups the women made the decision by themselves or together with their partners. The majority experienced that they had made the right decision. Women who terminated their pregnancy had a significant higher rate (51.2%) (p < or = 0.004) of previous abortions than those in the continuing group (23.2%). CONCLUSION: The decision to continue or terminate the pregnancy was to a great extent based on the severity of the malformation. Religious aspects did not seem to influence the decision. Many women expressed a need for additional occasion of information. The vast majority of women were satisfied with their decision. | Humans | Patient Participation | Patient Satisfaction | Questionnaires | OK |
| Susie | Gagnon | Merlin | Njoya | Michel | Brisson | Prenatal Diagnosis | 2011-04-00 Apr 2011 | OBJECTIVE: To assess the willingness of women and their family physicians (FPs) to engage in shared decision-making (SDM) as regards prenatal Down-syndrome screening and the factors that might influence their willingness to do so. METHODS: We conducted a survey of participants in Québec City, Canada, using the theory of planned behavior. We used a general linear model and multilevel approach that took the fact that some women consulted the same FP into account. RESULTS: This study comprised 109 pregnant women and 41 FPs. On a scale of - 3 to + 3, the pregnant women's and FPs' response scores were, respectively, 2.11 ± 1.38 and 2.66 ± 0.40. In women, attitude, significant others, self-efficacy, perceived moral correctness, and their FP's attitude influenced their willingness to engage in SDM. However, women without a post-secondary education were less likely to engage in SDM than women with a post-secondary education, mostly because the former lacked a sense of self-efficacy. In FPs, only attitude and significant others influenced their willingness to engage in SDM. CONCLUSION: Overall, the women and their FPs wished to engage in SDM as regards prenatal Down-syndrome screening. Only a few factors influenced this desire which therefore may be modifiable. | Decision Making | Humans | Patient Participation | Physician-Patient Relations | OK |
| B. A. | Bonsing | A. C. | de Vries | R. a. E. M. | Tollenaar | Colorectal Disease: The Official Journal of the Association of Coloproctology of Great Britain and Ireland | 2014-02-00 Feb 2014 | AIM: Surgery for rectal and sigmoid cancer is a model setting for investigating preoperative information provision and shared decision making (SDM), as the decision consists of a trade-off between the pros and cons of different treatment options. The aim of this study was to explore surgeons' opinion on the preoperative information that should be given to rectal and sigmoid cancer patients and to evaluate what is actually communicated. In addition, we assessed surgeons' attitudes towards SDM and compared these with patient involvement. METHOD: A questionnaire was sent to Dutch surgeons with an interest in gastroenterology. Preoperative consultations were recorded. A checklist was used to code the information that surgeons communicated to the patients. The OPTION-scale was used to measure patient involvement. RESULTS: Questionnaires were sent to 240 surgeons, and 103 (43%) responded. They stated that information on anastomotic leakage and its consequences, the benefits and risks of a defunctioning stoma and the impact of a stoma on quality of life were necessary preoperative information. In practice, patients were inconsistently informed of these items. Most participants agreed to using SDM in their consultations. However, in practice, most patients were offered only one treatment option and little SDM was seen. The mean OPTION-score was low (7/100). CONCLUSION: Insufficient information is given to patients with rectal and sigmoid cancer to guide them on their preferred surgical option. Information should be given on all treatment options, together with their complications and outcome, before any decision is made. | Male | Decision Making | Humans | Patient Participation | OK |
| Siebe J. | Swart | Wouter W. A. | Zuurmond | Agnes | van der Heide | BMC family practice | 2012-00-00 2012 | BACKGROUND: Little is known about pressure from patients or relatives on physician's decision making of continuous palliative sedation. We aim to describe experienced pressure by general practitioners (GPs) in cases of continuous sedation after the introduction of the Dutch practice guideline, using a questionnaire survey. METHODS: A sample of 918 Dutch GPs were invited to fill out a questionnaire about their last patient under continuous sedation. Cases in which GPs experienced pressure from the patient, relatives or other persons were compared to those without pressure. RESULTS: 399 of 918 invite GPs (43%) returned the questionnaire and 250 provided detailed information about their most recent case of continuous sedation. Forty-one GPs (16%) indicated to have experienced pressure from the patient, relatives or colleagues. In GPs younger than 50, guideline knowledge was not related to experienced pressure, whereas in older GPs, 15% with and 36% without guideline knowledge reported pressure. GPs experienced pressure more often when patients had psychological symptoms (compared to physical symptoms only) and when patients had a longer estimated life expectancy. A euthanasia request of the patient coincided with a higher prevalence of pressure for GPs without, but not for GPs with previous experience with euthanasia. GPs who experienced pressure had consulted a palliative consultation team more often than GPs who did not experience pressure. CONCLUSION: One in six GPs felt pressure from patients or relatives to start sedation. This pressure was related to guideline knowledge, especially in older GPs, longer life expectancy and the presence of a euthanasia request, especially for GPs without previous experience of euthanasia. | Male | Decision Making | Humans | Patient Participation | OK |
| Judith K. | Sluiter |  |  |  |  | International Journal of Adolescent Medicine and Health | 2012-00-00 2012 | OBJECTIVE: The purpose of the study was to assess common mental disorders and the related use and need for mental health care among clinically not yet active and clinically active medical students. METHODS: All medical students (n=2266) at one Dutch medical university were approached. Students from study years 1-4 were defined as clinically not yet active and students from study years 5 and 6 as clinically active. An electronic survey was used to detect common mental disorders depression (BSI-DEP), anxiety (BSI-ANG), stress (4DSQ) and post-traumatic stress disorder (IES). The use of mental health services in the past 3 months and the need for mental health services were asked for. The prevalence of common mental disorders, the use and need for mental health services and differences between groups were calculated. RESULTS: The response rate was 52%: 814 clinically not yet active and 316 clinically active students. The prevalence of common mental disorders among clinically not yet active and clinically active students was 54% and 48%, respectively. The use of mental health services was 14% in clinically not yet active and 12% in clinically active students with common mental disorders (n.s.). The need for mental health services by clinically not yet active and clinically active students was 52% and 46%, respectively (n.s.). CONCLUSIONS: The prevalence of probable common mental disorders are higher among clinically not yet active than among clinically active students. The need of mental health services exceeds use, but is the same in the two groups of students. | Male | Humans | Patient Participation | Questionnaires | OK |
| Ingela | Lundgren |  |  |  |  | Journal of obstetric, gynecologic, and neonatal nursing: JOGNN / NAACOG | 2010-06-00 2010 May-Jun | OBJECTIVE: To describe the meaning of previous experiences of childbirth in pregnant women who have exhibited intense fear of childbirth such that it has an impact on their daily lives. DESIGN: A descriptive phenomenological study. SETTING: A maternity clinic for women with fear of childbirth in the western part of Sweden. PARTICIPANTS: Nine women with intense fear of childbirth who were pregnant with their second child and considered their previous birth experiences negative. METHODS: Interviews that were transcribed verbatim and analyzed with a reflective life-world approach. RESULTS: The essential meanings that emerged were a sense of not being present in the delivery room and an incomplete childbirth experience. The women felt as if they had no place there, that they were unable to take their place, and that even if the midwife was present, she did not provide support. The experience remained etched in the women's minds and gave rise to feelings of fear, loneliness, and lack of faith in their ability to give birth and diminished trust in maternity care. These experiences contrasted with brief moments that made sense. CONCLUSIONS: Previous childbirth experiences for pregnant women with intense fear of childbirth have a deep influence and can be related to suffering and birth trauma. The implication is to provide maternity care where the nurse/midwife is present and supports women during birth in a way that enables them to be present and take their place during birth. | Humans | Patient Participation | Questionnaires | Female | OK |
| Thomas N. | Wise |  |  |  |  | Advances in Psychosomatic Medicine | 2012-00-00 2012 | There is increasing awareness of the limitations of disease as the primary focus of medical care. It is not that certain disorders lack an organic explanation, but that our assessment is inadequate in most clinical encounters. The primary goal of psychosomatic medicine is to correct this inadequacy by incorporation of its operational strategies into clinical practice. At present, the research evidence which has accumulated in psychosomatic medicine offers unprecedented opportunities for the identification and treatment of medical problems. Taking full advantage of clinimetric methods (such as with the use of Emmelkamp's two levels of functional analysis and the Diagnostic Criteria for Psychosomatic Research) may greatly improve the clinical process, including shared-decision making and self-management. Endorsement of the psychosomatic perspective may better clarify the pathophysiological links and mechanisms underlying symptom presentation. Pointing to individually targeted methods may improve final outcomes and quality of life. | Humans | Female | Middle Aged | Adult | OK |
| Sophie | Lanzkron | Neda | Ratanawongsa | Shawn M. | Bediako | Journal of Health Care for the Poor and Underserved | 2010-11-00 Nov 2010 | BACKGROUND: Adults with sickle cell disease (SCD) have often reported difficulties obtaining care during vaso-occlusive crisis (VOC) in qualitative studies. METHODS: We measured the experiences of 45 SCD patients who received in-hospital care for VOC using the Picker Patient Experience Questionnaire (PPE-15), and used the one sample binomial test to compare with national norms. RESULTS: Most SCD patients reported that they were insufficiently involved in decisions (86%), staff gave conflicting information (64%), it wasn't easy to find someone to discuss concerns (61%), doctors' answers to questions were not clear (58%), nurses' answers to questions were not clear (56%), doctors did not always discuss fears and anxieties (53%), and nurses did not always discuss fears and anxieties (52%). A greater percentage of SCD patients than the U.S. sample in 9 of 12 areas reported problems. CONCLUSIONS: Further research is needed to determine the consequences of and potential interventions to improve these poor experiences. | Male | Humans | Patient Participation | Patient Satisfaction | OK |
| Jonathan J. | Paly | Scott D. | Halpern | Deborah W. | Bruner | International Journal of Radiation Oncology, Biology, Physics | 2012-05-01 May 1, 2012 | PURPOSE: To investigate patients' willingness to participate (WTP) in a randomized controlled trial (RCT) comparing intensity-modulated radiotherapy (IMRT) with proton beam therapy (PBT) for prostate cancer (PCa). METHODS AND MATERIALS: We undertook a qualitative research study in which we prospectively enrolled patients with clinically localized PCa. We used purposive sampling to ensure a diverse sample based on age, race, travel distance, and physician. Patients participated in a semi-structured interview in which they reviewed a description of a hypothetical RCT, were asked open-ended and focused follow-up questions regarding their motivations for and concerns about enrollment, and completed a questionnaire assessing characteristics such as demographics and prior knowledge of IMRT or PBT. Patients' stated WTP was assessed using a 6-point Likert scale. RESULTS: Forty-six eligible patients (33 white, 13 black) were enrolled from the practices of eight physicians. We identified 21 factors that impacted patients' WTP, which largely centered on five major themes: altruism/desire to compare treatments, randomization, deference to physician opinion, financial incentives, and time demands/scheduling. Most patients (27 of 46, 59%) stated they would either "definitely" or "probably" participate. Seventeen percent (8 of 46) stated they would "definitely not" or "probably not" enroll, most of whom (6 of 8) preferred PBT before their physician visit. CONCLUSIONS: A substantial proportion of patients indicated high WTP in a RCT comparing IMRT and PBT for PCa. | Time Factors | Male | Humans | Patient Participation | OK |
| Misa | Komatsu | Chizuko | Suishu | Keiko | Osuka | Journal of Gerontological Nursing | 2010-03-00 Mar 2010 | To date, no elder care facilities in Japan have formally introduced the Eden Alternative philosophy of care. The purpose of this cross-sectional descriptive study was to identify the perceptions of care workers and nurses regarding the lives of the older adults in care facilities to consider the prospects for introducing the Eden Alternative to Japan. The participants included 139 care workers and 41 nurses who responded to a survey questionnaire based on Eden Alternative principles developed by the researchers for this study. More than half of the participants indicated that they sometimes thought the older adults experienced feelings of helplessness, loneliness, and boredom and hoped for changes in the manner of care to improve the lives of residents. Participants were also in favor of the residents having plants and visits from children, but opinions about having animals on site were split. The fact that the survey respondents noticed the problems indicated by the Eden Alternative suggests there is great potential for introducing the Eden Alternative to Japan. | Male | Humans | Patient Participation | Questionnaires | OK |
| Sze Lin | Yoong | Grant | Russell | Daniel | Barker | BMC family practice | 2013-00-00 2013 | BACKGROUND: Despite controversy about the benefits of routine prostate specific antigen (PSA) testing, rates of participation continue to rise. It is important to ensure that men are fully informed about the potential risks associated with this test. Little is known about the processes of shared decision making for PSA testing in the family practice setting. This study aimed to explore men's experiences of PSA testing participation and risk disclosure for PSA testing. METHODS: A cross-sectional survey of male family practice attendees aged 40 years or older, with no previous history of prostate cancer, between June 2010 and November 2011. Questions related to whether participants had undertaken PSA testing or discussed this with their doctor over the past 5 years, whether the patient or doctor had initiated the discussion, reasons for undergoing testing, and whether their doctor had discussed particular risks associated with PSA testing. RESULTS: Sixty-seven percent (215/320) of men recalled having a PSA test in the past five years. Of the respondents who reported not having a test, 14% had discussed it with their doctor. The main reasons for having a PSA test were doctor recommendation and wanting to keep up to date with health tests. Thirty-eight percent or fewer respondents reported being advised of each potential risk. CONCLUSIONS: Despite debate over the benefits of routine PSA testing, a high proportion of male family practice attendees report undertaking this test. Risks associated with testing appear to be poorly disclosed by general practitioners. These results suggest the need to improve the quality of informed consent for PSA testing in the family practice setting. | Male | Decision Making | Humans | Patient Participation | OK |
|  |  |  |  |  |  | Patient Education and Counseling | 2011-11-00 Nov 2011 | OBJECTIVE: To determine the effect size of provider communication about self-care and the provider's following treatment guidelines on the self-care behaviors of people with Type 2 diabetes (T2DM). METHODS: Data were from the TRIAD telephone surveys of Michigan patients in managed care with T2DM (n=1438). The survey asked about the patients' self-care and provider communication about blood glucose monitoring, exercise, foot care, flu vaccination and annual retina screening. RESULTS: After controlling for patient socio-demographics and disease severity, remembering having received provider information on these activities more than doubled the odds of performing blood glucose monitoring, and performing foot examinations. It also significantly increased the amount of exercise the patient performed. Provider modeling had a strong association with foot care. Counseling by a diabetes educator was positively and significantly associated with having an annual retina examination and receiving an influenza vaccination. CONCLUSIONS: Remembering self-care information is associated with increased home based self-care by patients and their families. Consulting with a diabetes educator can substantially improve patient self-care. PRACTICE IMPLICATIONS: Health care providers should encourage patient education. However, physician time is costly and most nurses do not specialize in patient education; one solution is to refer patients to a diabetes educator. | Male | Humans | Patient Participation | Questionnaires | OK |
| Susan J. | Blalock | Robyn | Sayner | Delesha M. | Carpenter | Optometry and Vision Science: Official Publication of the American Academy of Optometry | 2014-05-00 May 2014 | PURPOSE: The purpose of this preliminary study was to describe the extent to which providers used collaborative goal setting and individualized assessment with patients who were newly prescribed glaucoma medications. METHODS: English-speaking glaucoma suspect patients from six ophthalmology clinics who were newly prescribed glaucoma medications had their medical visits videotaped and were interviewed after the visits. The videotapes were transcribed and coded to examine provider use of collaborative goal setting and individualized assessment. RESULTS: Fifty-one patients seeing 12 ophthalmologists participated. Providers gave patients glaucoma treatment options during 37% of the visits; only five providers gave patients treatment options Providers asked for patient treatment preferences in less than 20% of the visits; only two providers asked for patient treatment preferences. Providers were significantly more likely to ask African American patients for their preferences or ideas concerning treatment than non-African American patients (Pearson χ² = 4.1, p = 0.04). Providers were also significantly more likely to ask African American patients about their confidence in using glaucoma medication regularly than non-African American patients (Pearson χ² = 8.2, p = 0.004). Providers asked about patient views about glaucoma in less than 20% of the visits; five providers asked patients their views on glaucoma and its treatment. Providers were significantly more likely to ask African American patients about their views on glaucoma than non-African American patients (Pearson χ² = 5.62, p = 0.02). CONCLUSIONS: Eye care providers often did not use collaborative goal setting or conduct individualized assessments of patient views on glaucoma when prescribing treatment for the first time. | Male | Humans | Patient Participation | Questionnaires | OK |
| Lilisbeth | Perestelo-Perez | Marien | Gonzalez | Jeanette | Perez | Patient Education and Counseling | 2011-12-00 Dec 2011 | OBJECTIVES: To assess psychiatric outpatients' attitudes towards concordance and shared decision making in a psychiatric setting and to evaluate the role that self-perceived knowledge and beliefs about psychiatric medicines play in those attitudes. METHODS: The Leeds Attitude to Concordance Scale (LATCon) was tested on a sample of 435 psychiatric outpatients. Principal Component Analysis was used to assess the structure of LATCon items. Regression analysis on LATCon scores was performed with sociodemographics, Belief about Medicines Questionnaire (BMQ) subscales, self-perceived knowledge, perceived psychiatrist behaviour and current medications as predictor variables. RESULTS: The LATCon scale showed a good factorial validity, with a monofactorial structure and high internal consistency. Psychiatric outpatients tended to be in agreement with the concept of concordance, but they did not share some relevant aspects of the construct. Cognitive representations of psychiatric medications, assessed by the BMQ subscales, significantly predicted scores on the LATCon scale. CONCLUSION: Psychiatric outpatients show a considerable desire to participate in decision making about their treatment. The Spanish version of the LATCon Scale seems to be a valid instrument. PRACTICE IMPLICATIONS: Psychiatrists must consider their patients' desire to participate in treatment decisions and explore how patients' views about psychiatric medications influence their attitudes towards concordance. | Male | Decision Making | Humans | Patient Participation | OK |
| Marcel W. M. | Post | Christel M. C. | van Leeuwen | Christina S. | Werner | Archives of Physical Medicine and Rehabilitation | 2014-09-00 Sep 2014 | OBJECTIVE: To test the Spinal Cord Injury Adjustment Model and gain a better understanding about whether and how the psychological resources general self-efficacy (SE), purpose in life (PIL), appraisals, and coping influence participation in persons with spinal cord injury (SCI). DESIGN: Cross-sectional data collection within the Swiss Spinal Cord Injury Cohort. SETTING: Community setting. PARTICIPANTS: Persons with SCI (N=516) who are ≥ 16 years old and living in the community in Switzerland. INTERVENTIONS: Not applicable. MAIN OUTCOME MEASURES: Participation was measured with the restrictions subscale of the Utrecht Scale for Evaluation of Rehabilitation-Participation, General SE with the General Self-Efficacy Scale, PIL with the Purpose in Life Test-Short Form, appraisals with the Appraisal of Life Events Scale, and coping with the Brief COPE. RESULTS: General SE (r=.32) and PIL (r=.23) were associated with less participation restrictions. The initial model yielded a poor model fit. The modified final model had an acceptable fit (χ(2)11=36.2; P<.01; root mean square error of approximation=.067 [90% confidence interval: .045-.09]; comparative fit index=.98). A total of 15% of the variance of participation was explained. In the final model, general SE had a moderate direct effect (β=.24) and mediated effects via threat appraisal and challenge appraisal and humor on participation, indicating a partial mediation effect. The association between PIL and participation was indirect: challenge appraisal and humor acted as mediators. CONCLUSIONS: The results only partly support the double-mediating effect as suggested in the SCI adjustment model because both direct and indirect effects on participation were observed. Individuals with higher general SE and PIL perceive less participation restrictions. General SE seems an appropriate target to enhance participation. Longitudinal studies are needed to support our findings. | Male | Humans | Patient Participation | Questionnaires | OK |
| Candace | Hayden | Rhonda | Dzierzon | Jose | Morales | Quality of Life Research: An International Journal of Quality of Life Aspects of Treatment, Care and Rehabilitation | 2013-04-00 Apr 2013 | PURPOSE: The patient activation measure short form (PAM-13) assesses patients' self-reported health management skills, knowledge, confidence, and motivation. We used item response theory to evaluate the psychometric properties of the PAM-13 utilized in rural settings. METHODS: A Rasch partial credit model analysis was conducted on the PAM-13 instrument using a sample of 812 rural patients recruited by providers and our research staff. Specially, we examined dimensionality, item fit, and quality of measures, category response curves, and item differential functioning. Convergent and divergent validities were also examined. FINDINGS: The PAM-13 instrument has excellent convergent and divergent validities. It is fairly unidimensional, and all items fit the Rasch model well. It has relatively high person and item reliability indices. Majority of the items were free of item differential functioning. There were, however, some issues with ceiling effects. Additionally, there was a lack of responses for category one across all items. CONCLUSIONS: Patient activation measure short form (PAM-13) performs well in some areas, but not all. In general, more items need to be added to cover the upper end of the trait. The four response categories of PAM-13 should be collapsed into three. | Male | Humans | Patient Participation | Questionnaires | OK |
| Michelle P. | Turner | John | Lu | Nicola | Wright | The Oncologist | 2010-00-00 2010 | BACKGROUND: Patient-reported outcomes (PROs) are essential for evaluating treatment effects on health-related quality of life and symptoms from the patient's perspective. This study sought to evaluate the psychometric properties of the nine-item Functional Assessment of Cancer Therapy/National Comprehensive Cancer Network Colorectal Cancer Symptom Index (FCSI-9) in a metastatic colorectal cancer (mCRC) population. METHODS: The FCSI-9 and EQ-5D were administered every 2-4 weeks to mCRC subjects in a phase III clinical trial. Three hundred ninety-one mCRC subjects completed the questionnaires at baseline and at least one follow-up assessment. Internal consistency reliability, test-retest reliability, construct validity, known groups validity, responsiveness, and the minimum important difference (MID) of the FCSI-9 were evaluated. RESULTS: The internal consistency and test-retest reliability of the FCSI-9 were acceptable (0.81 and 0.76, respectively). Construct validity was supported based on moderate correlations with the EQ-5D. Known groups validity was evaluated by examining the FCSI-9 scores of subjects categorized by their Eastern Cooperative Oncology Group performance status (PS) score. Subjects with better PS scores reported significantly higher FCSI-9 scores than those with lower PS scores at both baseline and week 8. Responsiveness, as measured by Guyatt's statistic, was 0.77 from baseline to week 8 and 0.60 from week 4 to week 12. Considering all data together, the MID of the FCSI-9 is estimated to be in the range of 1.5-3.0 points. CONCLUSION: Results provide preliminary evidence of the reliability, validity, and responsiveness of the FCSI-9. | Male | Humans | Patient Participation | Patient Satisfaction | OK |
|  |  |  |  |  |  | Western Journal of Nursing Research | 2013-02-00 Feb 2013 | The purpose of this study was to evaluate the psychometric properties of the Shared Decision-Making Inventory-Revised (SDMI-R) to measure four constructs (knowledge, attitudes, self-efficacy, and intent) theoretically defined as vital in discussing the human papillomavirus (HPV) disease and vaccine with clients. The SDMI-R was distributed to a sample (N = 1,525) of school nurses. Correlational matrixes denoted moderate to strong correlations, indicating adequate internal reliability. Reliability for the total instrument was satisfactory (α = .874) along with Attitude, Self-Efficacy and Intent subscales .828, .917, .891, respectively. Exploratory factor analysis revealed five components that explained 75.96% of the variance. | Decision Making | Humans | Female | Psychometrics | OK |
|  |  |  |  |  |  | Western Journal of Nursing Research | 2013-02-00 février 2013 | The purpose of this study was to evaluate the psychometric properties of the Shared Decision-Making Inventory–Revised (SDMI-R) to measure four constructs (knowledge, attitudes, self-efficacy, and intent) theoretically defined as vital in discussing the human papillomavirus (HPV) disease and vaccine with clients. The SDMI-R was distributed to a sample (N = 1,525) of school nurses. Correlational matrixes denoted moderate to strong correlations, indicating adequate internal reliability. Reliability for the total instrument was satisfactory (α = .874) along with Attitude, Self-Efficacy and Intent subscales .828, .917, .891, respectively. Exploratory factor analysis revealed five components that explained 75.96% of the variance. (PsycINFO Database Record (c) 2014 APA, all rights reserved). (journal abstract) | Decision Making | Psychometrics | Inventories | test validity | Doublon |
| Norhayati Mohd | Noor | Norsa’adah | Bachok | Siti Nor Ismalina | Isa | Family Practice | 2014-04-00 avril 2014 | Background: Patient satisfaction influences the outcomes of the patient-physician encounter. Objective: The objective of this study was to validate the Malay version patient satisfaction (MISS-21) questionnaire using a confirmatory validity approach. Methods: A cross-sectional study was conducted involving 252 patients attending primary health clinic, Hospital Universiti Sains Malaysia. Construct validity (convergent and discriminant) using confirmatory factor analysis and internal consistency were performed after the translation, content validity and face validity processes. Criterion validity was assessed using Pearson correlations with the scale of shared decision making 9-item questionnaire (SDMQ-9). The data was analysed using Analysis of Moment Structure version 19. Results: A total of 252 (100%) outpatients responded to this study. The final model that consists of three domains with 11 items had a good fit; (χ²(df) = 65.805 (32), P &lt; 0.001, Tucker–Lewis indices = 0.902, comparative fit index = 0.927, root mean square error of approximation = 0.061, standardized root mean square residual = 0.058). Composite reliability and average variance extracted of the domains ranged from 0.541 to 0.760 and 0.240 to 0.522, respectively. The SDMQ-9 had a moderate correlation with the total score of the final construct (r = 0.406, P &lt;0.001). Conclusion: The study suggested that the three-factor model with 11 items of the Malay version MISS-21 could be used to assess patient satisfaction on patient-physician interaction in primary health care setting because it is acceptably valid, reliable and simple. The validated Malay version questionnaire was called as ‘Skala Kepuasan Interaksi Perubatan-11’. (PsycINFO Database Record (c) 2014 APA, all rights reserved). (journal abstract) | Decision Making | physicians | Psychometrics | test validity | OK |
|  |  |  |  |  |  | Issues in Mental Health Nursing | 2011-00-00 2011 | In the context of patient-centered care and the increasing number of evidence-based substance abuse treatments, outpatient substance abuse treatment programs are poised to provide patients with a menu of options. Not all patients will be ready for such an open choice field in substance abuse treatment and they will undoubtedly differ on how they weigh the risks and benefits of having autonomy to choose their own treatment. Given the lack of an existing valid measure to assess this decision making process, this study sought to establish a measure to assess the relative weight that patients give to the pros and cons of choosing their own substance abuse treatment. Construct validity of the Decisional Balance for Patient Choice in Substance Abuse Treatment was assessed in a sample of 231 outpatients using confirmatory factor analysis. As another validity aspect, the use of decisional balance across a continuum of choice options also was investigated. The model fit was acceptable (CFI = 0.904). Internal consistency of the measure was established. The final 22-item measure revealed sound psychometric properties, but further testing is warranted. | Male | Decision Making | Humans | Patient Participation | OK |
| Ida | Løchting | Ingvild | Kjeken | Kåre Birger | Hagen | Rheumatology (Oxford, England) | 2013-05-00 May 2013 | OBJECTIVE: In rehabilitation, treatment is individually tailored to each patient's goals. Individualized instruments allow patients to choose domains that they consider important, which may make them particularly appropriate as evaluative tools in this setting. We aimed to evaluate the psychometric properties of the Norwegian version of the patient generated index (PGI) in patients with rheumatic diseases participating in inpatient rehabilitation or self-management programmes. METHODS: Patients completed the PGI together with other outcome measures at arrival and 5 and 52 weeks after arrival. The PGI was assessed for data quality by completion rates, reliability by the intraclass correlation coefficient (ICC), agreement by standard error of measurement (SEM) and smallest detectable change (SDC). Construct validity was assessed by testing a priori hypotheses regarding correlation between PGI scores and other outcome measures. Responsiveness was assessed by an a priori hypothesis regarding the correlation of different change scores and standardized response means (SRMs). RESULTS: A total of 145 patients participated and 118 (81%) completed the PGI correctly. The ICC was 0.87, SEM 7.25 and SDC 20.10. Ninety-three per cent of the hypotheses of correlation were confirmed in tests for construct validity. Responsiveness was confirmed in 53% and 71% of hypotheses tested at 5 and 52 weeks. SRMs were 0.2 and 0.4, respectively. CONCLUSION: The results support the validity, reliability and responsiveness of the Norwegian version of the PGI in patients with rheumatic diseases and its application as an outcome measure in rehabilitation or self-management programmes. Further research is needed to improve completion rates for the PGI. | Male | Humans | Patient Participation | Questionnaires | OK |
| Daniel | Scharfstein | Chad | Boult | Lisa | Reider | Health Services Research | 2011-04-00 Apr 2011 | OBJECTIVES: The Patient Activation Measure (PAM) quantifies the extent to which people are informed about and involved in their health care. Objectives were to determine the psychometric properties of PAM among multimorbid older adults and evaluate a theoretical, four-stage model of patient activation. Methods. A cross-sectional analysis was used to assess the psychometric properties of PAM. Internal consistency was assessed using Cronbach α. Construct validity was evaluated using general linear modeling to compute associations between PAM scores and health-related behaviors, functional status, and health care quality. Latent class analysis was used to evaluate the theoretical four-stage structure of patient activation. STUDY SETTING: Participants in a randomized trial of Guided Care (N = 855), a model of comprehensive health care for older adults with chronic conditions that put them at risk of using health services heavily during the coming year. PRINCIPAL FINDINGS: Higher PAM activation scores and stage were positively associated with higher functional status, health care quality, and adherence to some health behaviors. Latent class analysis supported the multistage theory of patient activation. CONCLUSIONS: The PAM is a reliable, valid, and potentially clinically useful measure of patient activation for multimorbid older adults. | Male | Humans | Patient Participation | Questionnaires | OK |
| Norhayati | Mohd Noor | Norsa'adah | Bachok | Siti | Nor Ismalina Isa | Family Practice | 2014-04-00 Apr 2014 | BACKGROUND: Patient satisfaction influences the outcomes of the patient-physician encounter. OBJECTIVE: The objective of this study was to validate the Malay version patient satisfaction (MISS-21) questionnaire using a confirmatory validity approach. METHODS: A cross-sectional study was conducted involving 252 patients attending primary health clinic, Hospital Universiti Sains Malaysia. Construct validity (convergent and discriminant) using confirmatory factor analysis and internal consistency were performed after the translation, content validity and face validity processes. Criterion validity was assessed using Pearson correlations with the scale of shared decision making 9-item questionnaire (SDMQ-9). The data was analysed using Analysis of Moment Structure version 19. RESULTS: A total of 252 (100%) outpatients responded to this study. The final model that consists of three domains with 11 items had a good fit; (χ (2) (df) = 65.805 (32), P < 0.001, Tucker-Lewis indices = 0.902, comparative fit index = 0.927, root mean square error of approximation = 0.061, standardized root mean square residual = 0.058). Composite reliability and average variance extracted of the domains ranged from 0.541 to 0.760 and 0.240 to 0.522, respectively. The SDMQ-9 had a moderate correlation with the total score of the final construct (r = 0.406, P <0.001). CONCLUSION: The study suggested that the three-factor model with 11 items of the Malay version MISS-21 could be used to assess patient satisfaction on patient-physician interaction in primary health care setting because it is acceptably valid, reliable and simple. The validated Malay version questionnaire was called as 'Skala Kepuasan Interaksi Perubatan-11'. | Male | Humans | Patient Satisfaction | Physician-Patient Relations | OK |
| Paul | Oh | Donna E. | Stewart | Stephanie J. | Brister | Clinical Rehabilitation | 2012-02-00 Feb 2012 | OBJECTIVE: The purpose of this study was to investigate the factor structure and psychometric properties of the Cardiac Rehabilitation Barriers Scale (CRBS). DESIGN, SETTING, AND PARTICIPANTS: In total, 2636 cardiac inpatients from 11 hospitals completed a survey. One year later, participants completed a follow-up survey, which included the CRBS. A subsample of patients also completed a third survey which included the CRBS, the Cardiac Rehabilitation Enrolment Obstacles scale, and the Beliefs About Cardiac Rehabilitation scale three weeks later. The CRBS asked participants to rate 21 cardiac rehabilitation barriers on a five-point Likert scale regardless of cardiac rehabilitation referral or enrolment. RESULTS: Maximum likelihood factor analysis with oblique rotation resulted in a four-factor solution: perceived need/healthcare factors (eigenvalue = 6.13, Cronbach's α = .89), logistical factors (eigenvalue = 5.83, Cronbach's α = .88), work/time conflicts (eigenvalue = 3.78, Cronbach's α = .71), and comorbidities/functional status (eigenvalue = 4.85, Cronbach's α = .83). Mean total perceived barriers were significantly greater among non-enrollees than cardiac rehabilitation enrollees (P < .001). Convergent validity with the Beliefs About Cardiac Rehabilitation and Cardiac Rehabilitation Enrolment Obstacles scales was also demonstrated. Test-retest reliability of the CRBS was acceptable (intraclass correlation coefficient = .64). CONCLUSION: The CRBS consists of four subscales and has sound psychometric properties. The extent to which identified barriers can be addressed to facilitate greater cardiac rehabilitation utilization warrants future study. | Male | Humans | Patient Participation | Questionnaires | OK |
|  |  |  |  |  |  | Women & Health | 2013-00-00 2013 | Psychopharmaceutical use by pregnant and postpartum women is complicated by the complexity of prescribing as well as the sociocultural context in which medication-related decisions are made. This study sought to advance understanding of decision-making processes and communication experiences regarding use of psychopharmaceuticals during pregnancy by considering both provider and consumer perspectives. An electronic survey was conducted with health care providers (N = 88) and women consumers (N = 83) from July 2010 through October 2011 regarding the perceived costs and benefits of taking mental health medication during and around the time of pregnancy. Descriptive analysis compared and contrasted experiences between the two groups regarding consumer-provider communication, critical incidents and triggers in decision-making, and response to case scenarios crafted around hypothetical client experiences. Both similarities and differences were evident among health care provider and women consumer responses regarding costs, benefits, communication experiences, and case scenario responses. Both quantitative and qualitative survey results indicated the need for more accurate, unbiased, and complete information exchange around mental health and medication. Study results suggested the centrality of the client-provider milieu to guide decision-making and emphasized the expressed need within both groups to create a shared decision-making practice environment characterized by authenticity, non-judgmental decision-making, compassion, humaneness, and reciprocity. | Decision Making | Humans | Questionnaires | Young Adult | OK |
| France | Légaré | Annie | Lapointe | Stéphane | Turcotte | PloS One | 2013-00-00 2013 | OBJECTIVES: While shared decision making (SDM) promotes health-related decisions that are informed, value-based and adhered to, few studies report on theory-based approaches to SDM adoption by healthcare professionals. We aimed to identify the factors influencing dietitians' intentions to adopt two SDM behaviours: 1) present dietary treatment options to patients and 2) help patients clarify their values and preferences. METHODS: We conducted a cross-sectional postal survey based on the Theory of Planned Behaviour among 428 randomly selected dietitians working in clinical practice across the Province of Quebec, Canada. We performed descriptive analyses and multiple regression analyses to determine the variables that explained the variance in intention to perform the behaviours. RESULTS: A total of 203 dietitians completed the questionnaire. Their ages were from 23 to 66 and they had been practising dietetics for 15.4±11.1 years (mean ± SD). On a scale from 1 to 7 (from strongly disagree to strongly agree), dietitians' intentions to present dietary treatment options and to clarify their patients' values and preferences were 5.00±1.14 and 5.68±0.74, respectively. Perceived behavioural control (β = 0.56, ρ<0.0001), subjective norm (β = 0.16, ρ<0.05), and moral norm (β = 0.22, ρ<0.0001), were the factors significantly predicting the intention to present dietary treatment options, while perceived behavioural control (β = 0.60, ρ<0.0001), attitude (β = 0.20, ρ<0.05), and professional norm (β = 0.22, ρ<0.001), significantly predicted the intention to help patients' clarify their values and preferences. CONCLUSION: Our results showed that dietitians intend to adopt the two SDM behaviours studied. Factors influencing intention were different for each behaviour, except for perceived behavioural control which was common to both behaviours. Thus, perceived behavioural control could be a key factor in interventions aiming to encourage implementation of SDM by dietitians. | Male | Decision Making | Humans | Questionnaires | OK |
| Alessandro | Coppo | Giuseppe | Tibaldi | Massimo | Di Giannantonio | PLoS ONE | 2013-05-20 mai 20, 2013 | Background: Previous studies have shown that attitudes towards depression may be influenced by country-specific social and cultural factors. A survey was carried out to collect beliefs on and attitudes toward depression in Italy, which has an established community-based mental health system. Methods: A telephone survey was carried out in a probabilistic sample aged ≥15 years. A 20-item questionnaire was administered to explore knowledge of depression, stigma, causal beliefs, treatment preference, and help-seeking attitudes. Results: Of the 1001 participants, 98% were aware of depression, and 62% had experienced it, either directly or indirectly. A widespread belief (75%) was that people suffering from depression should avoid talking about their problem. A minority of the sample viewed depression as a condition that should be managed without recourse to external help or a "socially dangerous" illness. Among perceived causes of depression, most respondents mentioned life stressors or physical strains. Psychologists were often indicated as an adequate source of professional help. Half of the sample believed that depression should be pharmacologically treated, but drugs were often seen as addictive. Referring to a primary care physician (PCP) was considered embarrassing; furthermore, many people thought that PCPs are too busy to treat patients suffering from depression. Conclusions: Our findings indicate that depression is seen as a reaction to significant life events that should be overcome with the support of significant others or the help of health professionals (mainly psychologists). However, there are still barriers to the disclosure of depressive symptoms to PCPs, and concerns about the addictive effect of antidepressants. In the presence of a gap between people’s beliefs and what health professionals consider appropriate for the treatment of depression, a "shared decision making" approach to treatment selection should be adopted taking into account the patients’ preference for psychological interventions to ensure active compliance with effective treatments. (PsycINFO Database Record (c) 2014 APA, all rights reserved). (journal abstract) | Mental health | public beliefs | depression | social factors | Doublon |
| Alessandro | Coppo | Giuseppe | Tibaldi | Massimo | Di Giannantonio | PloS One | 2013-00-00 2013 | BACKGROUND: Previous studies have shown that attitudes towards depression may be influenced by country-specific social and cultural factors. A survey was carried out to collect beliefs on and attitudes toward depression in Italy, which has an established community-based mental health system. METHODS: A telephone survey was carried out in a probabilistic sample aged ≥15 years. A 20-item questionnaire was administered to explore knowledge of depression, stigma, causal beliefs, treatment preference, and help-seeking attitudes. RESULTS: Of the 1001 participants, 98% were aware of depression, and 62% had experienced it, either directly or indirectly. A widespread belief (75%) was that people suffering from depression should avoid talking about their problem. A minority of the sample viewed depression as a condition that should be managed without recourse to external help or a "socially dangerous" illness. Among perceived causes of depression, most respondents mentioned life stressors or physical strains. Psychologists were often indicated as an adequate source of professional help. Half of the sample believed that depression should be pharmacologically treated, but drugs were often seen as addictive. Referring to a primary care physician (PCP) was considered embarrassing; furthermore, many people thought that PCPs are too busy to treat patients suffering from depression. CONCLUSIONS: Our findings indicate that depression is seen as a reaction to significant life events that should be overcome with the support of significant others or the help of health professionals (mainly psychologists). However, there are still barriers to the disclosure of depressive symptoms to PCPs, and concerns about the addictive effect of antidepressants. In the presence of a gap between people's beliefs and what health professionals consider appropriate for the treatment of depression, a "shared decision making" approach to treatment selection should be adopted taking into account the patients' preference for psychological interventions to ensure active compliance with effective treatments. | Male | Humans | Questionnaires | Female | OK |
| Kristine C. | Jordan | John F. | Hurdle |  |  | AMIA ... Annual Symposium proceedings / AMIA Symposium. AMIA Symposium | 2013-00-00 2013 | The United States, indeed the world, struggles with a serious obesity epidemic. The costs of this epidemic in terms of healthcare dollar expenditures and human morbidity/mortality are staggering. Surprisingly, clinicians are ill-equipped in general to advise patients on effective, longitudinal weight loss strategies. We argue that one factor hindering clinicians and patients in effective shared decision-making about weight loss is the absence of a metric that can be reasoned about and monitored over time, as clinicians do routinely with, say, serum lipid levels or HgA1C. We propose that a dietary quality measure championed by the USDA and NCI, the HEI-2005/2010, is an ideal metric for this purpose. We describe a new tool, the quality Dietary Information Extraction Tool (qDIET), which is a step toward an automated, self-sustaining process that can link retail grocery purchase data to the appropriate USDA databases to permit the calculation of the HEI-2005/2010. | Humans | Questionnaires | United States | Adult | OK |
| Hugo J. | Duivenvoorden | Jan | Passchier |  |  | Headache | 2010-04-00 Apr 2010 | OBJECTIVES: To evaluate the qualities of lay trainers with migraine and to quantify their self-management results. BACKGROUND: Little is known about the qualities of lay trainers with chronic diseases and the benefits for their own health. METHODS: Thirteen lay trainers (12 F, 1 M) completed a 3-step program that consisted of self-experience of a behavioral training (BT), providing BT to one fellow patient, and subsequently to a small group at home. Successful mastery of own migraine attacks was required for participation, and lay trainers received intensive guidance. Evaluation of the qualities of trainers took place post-BT by means of a specially constructed questionnaire. Their self-management was measured before self-experience of BT, post-BT, and at 6-month follow-up by a headache diary and questionnaires. RESULTS: The qualities of the 13 trainers were positively evaluated by 95 trainees, particularly their warmth, expertise, organization, explanation of BT, active control, and advice and guidance. Higher active control of lay trainers during the group sessions was significantly related to improvements on migraine frequency and internal locus of control in their trainees post-BT. Advice and guidance increased the likelihood of less attacks at follow-up and supportive encouragement promoted a higher internal locus of control. However, humor slightly increased the likelihood of more attacks post-BT, while fellowship and individualization negatively influenced internal locus of control. Lay trainers showed significantly more improvement in migraine frequency than their trainees at follow-up, as well as enhanced internal locus of control and quality of life. CONCLUSIONS: Participation in a stepwise training program can produce capable trainers and may positively influence their own health. Lay trainers may be more motivated to enhance their self-management skills as they have to present the benefits to their trainees. | Humans | Patient Participation | Questionnaires | Health Personnel | OK |
| Richard | Thomson | Stephen | Lord |  |  | Patient Education and Counseling | 2012-04-00 Apr 2012 | OBJECTIVE: Clinical consultations with patients should be informed by the evidence-based and involve shared decision making (SDM). We aimed to determine the delivery of SDM by clinicians with patients referred for invasive treatment of cardiac electrical disease and to establish whether decisions made corresponded with patient and referring physician expectations. METHODS: Forty-nine outpatient consultations with two consultant cardiologists in one large tertiary centre were audio-recorded. Demographic data, diagnosis, reasons for referral and decision reached were compared directly with patient and referring physician expectations. The OPTION instrument was used to measure SDM. Patient expectations and satisfaction were elicited. RESULTS: Quality of SDM was good (mean OPTION score 49%) and there was broad patient satisfaction. While all patients were suitable for invasive treatment, and the majority (80%, n=39) had been explicitly referred for it, only 59% (n=29) opted to proceed. Consultation quality with respect to SDM was significantly greater for patients choosing a less invasive option. CONCLUSION: These consultations often change expected management. Where decision making in the consultation is of higher quality, patients were more likely to change to a less invasive option. PRACTICE IMPLICATIONS: Clinicians performing invasive cardiac treatment should be able to demonstrate high quality decision making. | Male | Decision Making | Humans | Patient Participation | OK |
| Olivia | Pérez Corral | Sergio Minué | Lorenzo | Alina | Danet | The Health Care Manager | 2010-06-00 2010 Apr-Jun | A study was undertaken for the purpose of describing internal communication and the professional-patient relationship and to establish a descriptive model of the interaction between these 2 variables. A nationwide survey was carried out in primary care and specialist care centers in Spain. A simple random sampling method was used with 1183 health care professionals. The data collection instrument was a Likert questionnaire that recorded information on the perceived quality of internal communication (0-100 scale), professional-patient relationships (0-100 scale), and sociodemographic variables. The results were analyzed using SPSS 15.0, performing mean comparisons and a suitable linear regression model.The total average of the quality of internal communication was 53.79 points, and that of the professional-patient relationships was 74.17 points. Sex made no statistically significant difference. Age shows that the older the participant, the better his/her opinion of internal communication and professional-patient relationships. Nursing staff had the highest opinion of internal communication and professional-patient relationships. The association between internal communication and professional-patient relationship was positive (R = 0.45).It was concluded that continuous exchange of information among health care professionals, together with learning and shared decision making or a positive emotional climate, is an element that will consolidate good professional-patient relationships and ensure patient satisfaction. | Male | Humans | Patient Satisfaction | Questionnaires | OK |
| Elizabeth | Nabiwemba | Christopher G. | Orach |  |  | BMC pregnancy and childbirth | 2013-00-00 2013 | BACKGROUND: Quality of intrapartum care is an important intervention towards increasing clients' utilization of skilled attendance at birth and accelerating improvements in newborn's and maternal survival and wellbeing. Ensuring quality of care is one of the key challenges facing maternal and neonatal services in Uganda. The study assessed quality of intrapartum care services in the general labor ward of the Mulago national referral and teaching hospital in Uganda from clients' perspective. METHODS: A cross sectional study was conducted using face to face interviews at discharge with 384 systematically selected clients, who delivered in general labor ward at Mulago hospital during May, 2012. Data analysis was done using STATA Version (10) software. Means and median general index scores for quality of intrapartum care services were calculated. Linear regression models were used to determine factors associated with quality of care. RESULTS: Overall, quality of intrapartum care mean index score was 49.4 (standard deviation (sd) 15.46, and the median (interquartile range (IQR)) was 49.1 (37.5-58.9). Median index scores (IQR) per selected quality of care indicators were; dignity and respect 75 (50-87.5); relief of pain and suffering 71.4 (42.8-85.7); information 42.1 (31.6-55.3); privacy and confidentiality 33.3 (1-66.7); and involvement in decision making 16.7 (1-33.3). On average, higher educational level (college/university) (β: 6.81, 95% CI: 0.85-15.46) and rural residence of clients (β: 5.67, 95% CI: 0.95-10.3) were statistically associated with higher quality scores. CONCLUSION: This study has revealed that quality of intrapartum care services from clients' perspective was low. Improvements should be focused on involving clients in decision making, provision of information about their conditions and care, and provision of privacy and confidentiality. There is also need to improve the number and availability of health care providers in the labor ward. | Humans | Patient Participation | Patient Satisfaction | Questionnaires | OK |
| Bengt | Fridlund | Amir | Baigi |  |  | Journal of Advanced Nursing | 2011-03-00 mars 2011 | Aim: The aim of the study was to develop and test the psychometric properties of a patient participation questionnaire in emergency departments. Background: Patient participation is an important indicator of the quality of healthcare. International and national healthcare policy guidelines promote patient participation. While patients cared for in emergency departments generally express dissatisfaction with their care, a review of the literature fails to reveal any scientifically tested instruments for assessing patient participation from the perspective of patients. Methods: A methodological study was conducted involving a convenience sample of 356 patients recently cared for in emergency departments in Sweden. Data were collected in 2008 and analysed for construct and criterion validity, also homogeneity and stability reliability. Results: A 17-item questionnaire was developed. Two separate factor analyses revealed a distinct 4-factor solution which was labelled: Fight for participation, Requirement for participation, Mutual participation and Participating in getting basic needs satisfied. Criterion validity testing showed 9 out of 20 correlations above 0·30 and, of these, three were moderate correlations of 0·62, 0·63 and 0·70. Cronbach’s alpha coefficient ranged from 0·63 to 0·84 and test–retest varied between 0·59 and 0·93. Conclusion: The results signify evidence of acceptable validity and reliability, and the questionnaire makes it possible to evaluate patient participation in emergency department caring situations. In addition, it produces data which are usable by a diverse range of healthcare professionals. (PsycINFO Database Record (c) 2014 APA, all rights reserved). (journal abstract) | Psychometrics | Test Construction | test development | emergency departments | Doublon |
| Bengt | Fridlund | Amir | Baigi |  |  | Journal of Advanced Nursing | 2011-03-00 Mar 2011 | AIM: The aim of the study was to develop and test the psychometric properties of a patient participation questionnaire in emergency departments. BACKGROUND: Patient participation is an important indicator of the quality of healthcare. International and national healthcare policy guidelines promote patient participation. While patients cared for in emergency departments generally express dissatisfaction with their care, a review of the literature fails to reveal any scientifically tested instruments for assessing patient participation from the perspective of patients. METHODS: A methodological study was conducted involving a convenience sample of 356 patients recently cared for in emergency departments in Sweden. Data were collected in 2008 and analysed for construct and criterion validity, also homogeneity and stability reliability. RESULTS: A 17-item questionnaire was developed. Two separate factor analyses revealed a distinct 4-factor solution which was labelled: Fight for participation, Requirement for participation, Mutual participation and Participating in getting basic needs satisfied. Criterion validity testing showed 9 out of 20 correlations above 0.30 and, of these, three were moderate correlations of 0.62, 0.63 and 0.70. Cronbach's alpha coefficient ranged from 0.63 to 0.84 and test-retest varied between 0.59 and 0.93. CONCLUSION: The results signify evidence of acceptable validity and reliability, and the questionnaire makes it possible to evaluate patient participation in emergency department caring situations. In addition, it produces data which are usable by a diverse range of healthcare professionals. | Male | Humans | Patient Participation | Patient Satisfaction | Doublon |
| Mick P. | Couper | John | Van Hoewyk | Neil R. | Powe | Medical Decision Making | 2010-09-00 septembre 2010 | Background: Racial/ethnic differences in shared decision making about cardiovascular risk-reduction therapy could affect health disparities. Objective: To investigate whether patient race/ethnicity is associated with experiences discussing cardiovascular risk-reduction therapy with health care providers. Setting: National sample of US adults identified by random-digit dialing. Design: Cross-sectional survey conducted in November 2006 to May 2007. Participants: Among participants in the National Survey of Medical Decisions (DECISIONS), a nationally representative sample of English-speaking US adults aged 40 and older, the authors analyzed respondents who reported discussing hyperlipidemia or hypertension medications with a health care provider in the previous 2 years. Measurements: In multivariate linear and logistic regressions adjusting for age, gender, income, insurance status, perceived health, and current therapy, they assessed the relation between race/ethnicity (black/Hispanic v. white) and decision making: knowledge, discussion of pros and cons of therapy, discussion of patient preference, who made the final decision, preferred involvement, and confidence in the decision. Results: Of respondents who discussed high cholesterol (N = 738) or hypertension (N = 745) medications, 88% were white, 9% black, and 4% Hispanic. Minorities had lower knowledge scores than whites for hyperlipidemia (42% v. 52%, difference-10% [95% confidence interval (CI): 15, -5], P &lt; 0.001), but not for hypertension. For hyperlipidemia, minorities were more likely than whites to report that the health care provider made the final decision for treatment (31.7% v. 12.3% whites, difference 19.4% [95% CI: 6.9, 33.1%], P &lt; 0.01); this was not true for hypertension. Limitations: Possible limitations include the small percentage of minorities in the sample and potential recall bias. Conclusions: Minorities considering hyperlipidemia therapy may be less informed about and less involved in the final decision-making process. (PsycINFO Database Record (c) 2012 APA, all rights reserved). (journal abstract) | Shared decision making | Decision Making | Client Participation | Therapeutic Processes | Doublon |
| Mick P. | Couper | John | Van Hoewyk | Neil R. | Powe | Medical Decision Making: An International Journal of the Society for Medical Decision Making | 2010-10-00 2010 Sep-Oct | BACKGROUND: Racial/ethnic differences in shared decision making about cardiovascular risk-reduction therapy could affect health disparities. OBJECTIVE: To investigate whether patient race/ethnicity is associated with experiences discussing cardiovascular risk-reduction therapy with health care providers. SETTING: National sample of US adults identified by random-digit dialing. DESIGN: Cross-sectional survey conducted in November 2006 to May 2007. PARTICIPANTS: Among participants in the National Survey of Medical Decisions (DECISIONS), a nationally representative sample of English-speaking US adults aged 40 and older, the authors analyzed respondents who reported discussing hyperlipidemia or hypertension medications with a health care provider in the previous 2 years. MEASUREMENTS: In multivariate linear and logistic regressions adjusting for age, gender, income, insurance status, perceived health, and current therapy, they assessed the relation between race/ethnicity (black/Hispanic v. white) and decision making: knowledge, discussion of pros and cons of therapy, discussion of patient preference, who made the final decision, preferred involvement, and confidence in the decision. RESULTS: Of respondents who discussed high cholesterol (N = 738) or hypertension (N = 745) medications, 88% were white, 9% black, and 4% Hispanic. Minorities had lower knowledge scores than whites for hyperlipidemia (42% v. 52%, difference -10% [95% confidence interval (CI): 15, -5], P < 0.001), but not for hypertension. For hyperlipidemia, minorities were more likely than whites to report that the health care provider made the final decision for treatment (31.7% v. 12.3% whites, difference 19.4% [95% CI: 6.9, 33.1%], P < 0.01); this was not true for hypertension. LIMITATIONS: Possible limitations include the small percentage of minorities in the sample and potential recall bias. CONCLUSIONS: Minorities considering hyperlipidemia therapy may be less informed about and less involved in the final decision-making process. | Male | Decision Making | Humans | Patient Participation | OK |
| Christopher R. | Friese | John J. | Graff | Kendra | Schwartz | Oncology Nursing Forum | 2014-01-01 Jan 1, 2014 | PURPOSE/OBJECTIVES: To characterize the perspectives of partners (husbands or significant others) of patients with breast cancer in the treatment decision-making process and to evaluate racial and ethnic differences in decision outcomes. DESIGN: A cross-sectional survey. SETTING: Los Angeles, CA, and Detroit, MI. SAMPLE: 517 partners of a population-based sample of patients with breast cancer four years post-treatment. METHODS: A self-administered mailed questionnaire. Chi-square tests and logistic regression were used to assess associations between race and ethnicity and decision outcomes. MAIN RESEARCH VARIABLES: Decision regret and three elements of the decision process: information received, actual involvement, and desired involvement. FINDINGS: Most partners reported receiving sufficient information (77%), being involved in treatment decisions (74%), and having sufficient involvement (73%). Less-acculturated Hispanic partners were more likely than their Caucasian counterparts to report high decision regret (45% versus 14%, p<0.001). Factors significantly associated (p<0.05) with high decision regret were insufficient receipt of treatment information, low involvement in decision making, and a desire for more involvement. CONCLUSIONS: Partners were generally positive regarding their perspectives about participating in the breast cancer treatment decision-making process. However, less acculturated Hispanic partners were most vulnerable to decision regret. In addition, high decision regret was associated with modifiable elements of the decision-making process. IMPLICATIONS FOR NURSING: Attention should be paid to ensuring racial and ethnic minority partners are sufficiently involved in breast cancer treatment decisions and receive decision support. | Male | Decision Making | Emotions | Humans | OK |
| Somnath | Saha | Elizabeth A. | Jacobs |  |  | Journal of General Internal Medicine | 2014-01-00 Jan 2014 | BACKGROUND: Prior research has documented a high prevalence of conspiracy beliefs about the origin of the human immunodeficiency virus (HIV) and the role of the government in the acquired immunodeficiency syndrome (AIDS) epidemic, particularly among racial and ethnic minorities in the United States. Whether such beliefs are a barrier to participation in HIV prevention research is not known. OBJECTIVE: To understand the prevalence of HIV conspiracy beliefs and their relationship to willingness to participate in HIV vaccine research among three racial/ethnic groups. DESIGN: Cross-sectional survey. PARTICIPANTS: Six hundred and one community-recruited volunteers (33.0 % White, 32.5 % Mexican American, and 34.5 % African American). MAIN MEASURES: We evaluated the level of agreement with six previously described HIV conspiracy beliefs, trust in medical research, and willingness to participate in HIV vaccine research. Multivariate models were used to compare these parameters among the three racial/ethnic groups while controlling for the potential confounding effects of socioeconomic status, access to health care, and other demographic factors. RESULTS: African Americans, Mexican Americans, and whites had similar levels of distrust in medical research. African and Mexican Americans were more likely to endorse one or more of six HIV conspiracy beliefs than whites (59.0 % and 58.6 % versus 38.9 %, respectively, P < 0.001), but were significantly more willing to participate in HIV vaccine research (ORs 1.58, CI 1.10-2.25 and 2.53, CI 1.75-3.66, respectively). Among respondents of all racial/ethnic groups, endorsing HIV conspiracy beliefs was not associated with willingness to participate in research. CONCLUSIONS: HIV conspiracy beliefs, while common among all racial and ethnic groups in the United States, do not preclude willingness to participate in HIV prevention research. | Male | Humans | Patient Participation | Female | Doublon |
| Somnath | Saha | Elizabeth A. | Jacobs |  |  | Journal of General Internal Medicine | 2014-01-00 Jan 2014 | BACKGROUND: Prior research has documented a high prevalence of conspiracy beliefs about the origin of the human immunodeficiency virus (HIV) and the role of the government in the acquired immunodeficiency syndrome (AIDS) epidemic, particularly among racial and ethnic minorities in the United States. Whether such beliefs are a barrier to participation in HIV prevention research is not known. OBJECTIVE: To understand the prevalence of HIV conspiracy beliefs and their relationship to willingness to participate in HIV vaccine research among three racial/ethnic groups. DESIGN: Cross-sectional survey. PARTICIPANTS: Six hundred and one community-recruited volunteers (33.0 % White, 32.5 % Mexican American, and 34.5 % African American). MAIN MEASURES: We evaluated the level of agreement with six previously described HIV conspiracy beliefs, trust in medical research, and willingness to participate in HIV vaccine research. Multivariate models were used to compare these parameters among the three racial/ethnic groups while controlling for the potential confounding effects of socioeconomic status, access to health care, and other demographic factors. RESULTS: African Americans, Mexican Americans, and whites had similar levels of distrust in medical research. African and Mexican Americans were more likely to endorse one or more of six HIV conspiracy beliefs than whites (59.0 % and 58.6 % versus 38.9 %, respectively, P < 0.001), but were significantly more willing to participate in HIV vaccine research (ORs 1.58, CI 1.10-2.25 and 2.53, CI 1.75-3.66, respectively). Among respondents of all racial/ethnic groups, endorsing HIV conspiracy beliefs was not associated with willingness to participate in research. CONCLUSIONS: HIV conspiracy beliefs, while common among all racial and ethnic groups in the United States, do not preclude willingness to participate in HIV prevention research. | Male | Humans | Patient Participation | Female | OK |
| Alison | Avenell | Marie | Johnston | Suzanne | McDonald | PloS One | 2011-00-00 2011 | BACKGROUND: Interventions for dietary and physical activity changes in obese adults may be less effective for participants with additional obesity-related risk factors and co-morbidities than for otherwise healthy individuals. This study aimed to test the feasibility and acceptability of the recruitment, allocation, measurement, retention and intervention procedures of a randomised controlled trial of an intervention to improve physical activity and dietary practices amongst obese adults with additional obesity related risk factors. METHOD: Pilot single centre open-labelled outcome assessor-blinded randomised controlled trial of obese (Body Mass Index (BMI)≥30 kg/m2) adults (age≥18 y) with obesity related co-morbidities such as type 2 diabetes, impaired glucose tolerance or hypertension. Participants were randomly allocated to a manual-based group intervention or a leaflet control condition in accordance to a 2∶1 allocation ratio. Primary outcome was acceptability and feasibility of trial procedures, secondary outcomes included measures of body composition, physical activity, food intake and psychological process measures. RESULTS: Out of 806 potentially eligible individuals identified through list searches in two primary care general medical practices N = 81 participants (63% female; mean-age = 56.56(11.44); mean-BMI = 36.73(6.06)) with 2.35(1.47) co-morbidities were randomised. Scottish Index of Multiple Deprivation (SIMD) was the only significant predictor of providing consent to take part in the study (higher chances of consent for invitees with lower levels of deprivation). Participant flowcharts, qualitative and quantitative feedback suggested good acceptance and feasibility of intervention procedures but 34.6% of randomised participants were lost to follow-up due to overly high measurement burden and sub-optimal retention procedures. Participants in the intervention group showed positive trends for most psychological, behavioural and body composition outcomes. CONCLUSIONS: The intervention procedures were found to be acceptable and feasible. Attrition rates were unacceptably high and areas for improvements of trial procedures were identified. TRIAL REGISTRATION: Controlled-Trials.com ISRCTN90101501. | Male | Humans | Patient Participation | Questionnaires | OK |
| Randal J. | Thomas | Ray W. | Squires |  |  | Journal of Cardiopulmonary Rehabilitation and Prevention | 2011-08-00 2011 Jul-Aug | OBJECTIVE: Outpatient cardiac rehabilitation (CR) services are underutilized. Fewer women participate in CR than men. It is unclear whether women referred to CR enroll at the same rate as men. The purpose of this study was to compare enrollment rates of men and women who were referred to CR. METHODS: A retrospective medical record review was performed for selected variables for all adult patients who resided in Olmsted County, Minnesota, were hospitalized from January 2005 through July 2007 and were referred to a CR program. Enrollment in CR was defined as attendance at 1 or more CR sessions. RESULTS: A total of 450 patients referred to CR were identified. Of them, 286 patients (64%) enrolled in CR. Rates of enrollment for men and women were similar (65% vs 62%, P = .54). Patients of either gender who did not enroll were older, less educated, less often married, more likely to smoke, more likely to be diabetic, and less likely to have had cardiac surgery. Enrollment rates of women with or without comorbidities were similar (62% vs 61%, P = .87), while enrollment rate of men with comorbidities was lower than for men without comorbidities (52% vs 75%, P < .01). CONCLUSION: In our CR program, enrollment rates for men and women, once referred, are similar. Enrollment rates are lower for older patients of both genders and for men with comorbidities. Lower utilization of CR by women than by men does not appear to be because of a gender difference in enrollment rates after a referral is made. | Male | Humans | Patient Participation | Patient Satisfaction | OK |
| Soraya | Zabihi | David L. | Brown |  |  | Medical Decision Making: An International Journal of the Society for Medical Decision Making | 2013-05-00 May 2013 | BACKGROUND: The Institute of Medicine has called for a new health care paradigm that integrates patient values into discussions of the risks and benefits of treatment. Although cardiovascular disease (CVD) affects one-third of Americans, little is known about how adults regard the potential harms or complications of treatment. OBJECTIVE: We sought to determine the preferences of community-dwelling adults for 15 potential harms or complications resulting from treatment of CVD. METHODS: In a telephone survey, adults older than 18 years residing on Long Island, New York, were asked to score the preferences for 15 potential harms or complications of treatment of CVD on a scale from 0 to 100. All statistical analyses were based on nonparametric methods. Multivariable general linear model analyses were performed to identify demographic factors associated with the score assigned for each adverse outcome. RESULTS: The 807 individuals surveyed generated 723 unique sequences of scores for the 15 outcomes. The ranking of scores from least to most acceptable was stroke, major myocardial infarction (MI), cognitive dysfunction, renal failure, death, prolonged ventilator support, heart failure, angina, sternal wound infection, major bleeding, reoperation, prolonged recovery in a nursing home, cardiac readmission, minor MI, and percutaneous coronary intervention. Demographic factors accounted for less than 7% of the observed variation in the score attributed to each outcome. CONCLUSIONS: Individual community-dwelling adults living on Long Island, New York, assign unique values to their preferences for potential harms encountered following treatment of CVD. Thus, risk-benefit discussions and treatment decisions regarding CVD should be harmonized to the value system of each individual. | Male | Humans | Young Adult | Female | OK |
| D. | Hergert-Lüder | A. | Katalinic | A. | Waldmann | Deutsche Medizinische Wochenschrift (1946) | 2013-11-00 Nov 2013 | BACKGROUND: In 2007, an organized population-based mammography screening program in Schleswig-Holstein has been implemented. According the European guideline a participation rate of 70% should be reached as a measurement of quality. In Schleswig-Holstein participation rate reached only 45% and is one of the lowest rates in Germany. The reasons are unknown. METHOD: To 20,000 women, aged 50-69, who did not participate in mammography screening after an invitation letter, a questionnaire was sent to assess reasons of non-participation and knowledge about the program. We calculated the proportion of women answering to given aspects in the questionnaire. RESULTS: The response rate was 14.8% (n = 2591). Women reported multiple reasons for non-participation. Medical reasons and personal attitude were the most frequent ones. Organizational reasons were of minor importance. 75% of women get primarily informed about the program by the invitation letter. 25% of women talked with their practitioner about the program. CONCLUSION: The low participation rate of mammography screening in Schleswig-Holstein is not based on worse organization but on medical reasons or personal attitudes of the women. It seems, that information about the program and benefits and harms of mammography screening has not reached the general population. So general practitioner or gynecologists should advice women more intensively. Leaflets and pamphlets should be more disseminated. | Humans | Patient Participation | Female | Aged | OK |
| Ashish Mark | Ingle | Peter C. | Adamson | Susan M. | Blaney | Pediatric Blood & Cancer | 2010-07-15 Jul 15, 2010 | PURPOSE: Pharmacokinetic (PK) studies provide critical information about the disposition of anticancer drugs in children. In the Children's Oncology Group (COG) Phase 1 Consortium, pharmacokinetic studies are usually optional. We surveyed the attitudes towards PK studies among subjects in phase 1 trials at COG institutions. METHODS: Subjects were eligible if they participated in a phase 1 anticancer drug study with optional PK studies within the 4 weeks, regardless of whether they agreed to participate in the PK studies. Staff provided demographics; subjects/parents completed a questionnaire. RESULTS: Fifty eligible subjects enrolled. Thirty-six (72%) of the 50 eligible subjects consented to participate in PK studies; 14 (25%) declined. The most common reasons for participating were "the results might help researchers learn more about the drug" and "results from the pharmacokinetic studies might help other children." The most common reasons for not participating were "having the samples drawn would mean spending extra time in the hospital," and "my child might have needed a separate IV catheter in order to participate." CONCLUSIONS: The majority of subjects identified altruistic motives for participation in PK studies. Subjects who did not participate in PK studies identified extra time and need for an extra IV as important concerns. Simple interventions like sending staff to the subjects' home to draw PK samples or drawing samples from existing catheters could increase the number of subjects who are willing to participate in PK studies. | Male | Humans | Patient Participation | Questionnaires | OK |
| Margaret | Mordin | Sheri | Fehnel | Catherine | Copley-Merriman | Value in Health: The Journal of the International Society for Pharmacoeconomics and Outcomes Research | 2012-05-00 May 2012 | OBJECTIVES: Previous analyses of patient-reported outcome (PRO) label claims concentrated only on successful label claims. The goal of this research was to explore the reasons why PRO label claims were denied and to compile regulatory feedback regarding the use of PROs in clinical trials. METHODS: By using the Food and Drug Administration's Drug Approval Report Web page, all new molecular entities and biologic license applications approved between January 2006 and December 2010 were identified. For identified drug products, medical review sections from publicly available drug approval packages were reviewed to identify PRO end-point status and any Study Endpoints and Label Development team comments. RESULTS: Of the 116 new molecular entities and biologic license applications with accompanying drug approval packages identified and reviewed, 44.8% of the products included PROs as part of the pivotal studies; however, only 24.1% received PRO label claims. Primary reasons for denial included issues of fit for purpose, issues of study design, data quality or interpretation, statistical issues, administrative issues, and lack of demonstrated treatment benefit. CONCLUSIONS: Based on drug approval packages, nearly half (45%) of new molecular entitity/biologic license application products in the years 2006 to 2010 included PROs in the clinical trials supporting their approval, yet this rate is not reflected by claims granted. Understanding the nature of PRO claims granted under the current regulatory guidance is important. In addition, a clear understanding of denied claims yields valuable insight into where sponsors may improve implementation of PROs in clinical trials and submission of PRO evidence to increase the likelihood of obtaining PRO label claims. | Humans | Patient Participation | United States | self report | OK |
| L. | Fallowfield | D. C. | Talbot | V. | Jenkins | European Journal of Cancer (Oxford, England: 1990) | 2011-07-00 Jul 2011 | BACKGROUND: Communication with patients contemplating Phase 1 cancer trial participation can be challenging. Controversy exists as to whether they are provided with sufficient information to give genuinely informed consent. We present data examining the reasons patients gave for trial entry. METHOD: Following discussions with oncologists about Phase 1 trials, participants completed a 19-item study specific 'accept or decline measure' exploring hope, expectations of benefit, altruism, concerns, and general perceptions of the trial information. They also completed 2 standardised questionnaires measuring psychological morbidity and predisposition towards optimism. RESULTS: Forty patients completed the study questionnaires. Patients were generally optimistic with few concerns about the experimental nature of Phase 1 trials. Most 36/40 (90%) consented to trial entry. Fifty-one percent thought the trial was the only treatment option available. The four main reasons for trial entry were: expectation of some medical benefit (21%); trial the best available option (21%); to maintain hope (15%) and to help with research (13%). Only one patient gave altruism as their main reason for trial participation. CONCLUSION: Patients considering Phase 1 trials may be a self-selected group with optimistic expectations of personal benefit driving trial entry rather than altruism. Achieving genuinely informed consent and avoidance of therapeutic misconceptions in such patients may be difficult. | Male | Humans | Patient Participation | Questionnaires | OK |
| Rosa | Baier | Kristen | Butterfield | Gary | Epstein-Lubow | Journal of Hospital Medicine: An Official Publication of the Society of Hospital Medicine | 2013-04-00 Apr 2013 | BACKGROUND: Randomized controlled trials provide strong evidence for guidelines and interventions. Yet, much of the eligible population declines to be studied. OBJECTIVE: To identify differences between participants and eligible nonparticipants in (1) perceived stress, (2) self-efficacy, (3) recovery expectations, (4) discussing advance directives, and (5) understanding a standard prescription label (health literacy). DESIGN: Quasi-experimental prospective cohort study in 5 acute-care hospitals. METHODS: We approached 295 hospital inpatients as they were being recruited for a behavioral intervention and asked them to answer 5 screening questions. We matched respondents' answers to their acceptance of the behavioral intervention and to Medicare claims and enrollment data. We used multivariate logistic regression to compare consent rates based on screening-question responses. SETTING/PATIENTS: Hospitalized fee-for-service Medicare patients. RESULTS: Patients were less likely to consent to the behavioral intervention when they reported feeling unable to control important things in their lives (odds ratio [OR]: 0.35, 95% confidence interval [CI]: 0.14-0.92), had low recovery expectations (OR: 0.17, 95% CI: 0.06-0.45), or were confused by any question (OR: 0.11, 95% CI: 0.05-0.24). Conversely, individuals who answered the medication question incorrectly were more likely to consent to the behavioral intervention (OR: 3.82, 95% CI: 1.12-13.03). There were no significant differences in consent for patients who reported feeling overwhelmed or reported discussing advance care planning with family members or doctors. CONCLUSIONS: Hospitalized eligible nonparticipants differ in constructs related to perceived stress, recovery expectation, and health literacy. Recognizing such characteristics may inform strategies to improve intervention recruitment in the hospital and representation in clinical trials. | Male | Humans | Patient Participation | Questionnaires | OK |
| Richard | Novak | Edith | Swann | David | Metzger | AIDS and behavior | 2013-02-00 Feb 2013 | Enrollment of US women with sufficient risk of HIV infection into HIV vaccine efficacy trials has proved challenging. A cohort of 799 HIV-negative women, aged 18-45, recruited from three US cities was enrolled to assess recruitment strategies based on geographic risk pockets, social and sexual networks and occurrence of sexual concurrency and to assess HIV seroincidence during follow-up (to be reported later). Among enrolled women, 90 % lived or engaged in risk behaviors within a local risk pocket, 64 % had a male partner who had concurrent partners and 50 % had a male partner who had been recently incarcerated. Nearly half (46 %) were recruited through peer referral. At enrollment, 86 % of women said they were willing to participate in a vaccine efficacy trial. Results indicate that participant and partner risk behaviors combined with a peer referral recruitment strategy may best identify an at-risk cohort willing to participate in future trials. | Humans | Patient Participation | Questionnaires | Female | OK |
| Sara | Zerbel | Alyssa | Bernardo | Ajay K. | Sethi | American Journal of Infection Control | 2013-11-00 Nov 2013 | BACKGROUND: Multidrug-resistant organisms (MDROs) are common in health care-associated infection (HAI). The aim of this study was to examine patient's willingness to learn about MDROs and HAIs and their preferred ways of education about MDRO HAIs. METHODS: Interviewer-administered questionnaire at a tertiary care academic medical center. We recruited a sample of 100 adult patients who had HAI and were placed in contact precautions. RESULTS: Ninety-six percent of patients had heard of MDROs. Ninety-eight percent of patients thought that their involvement in learning about MDROs was very important or important. Most of the patients thought that receiving information about MDROs would probably or definitely help them to make choices that would improve their health care. Patients expressed interest in more than 1 form of educational material: 70% chose written material, 57% verbal information, 53% an informational video, and 50% Internet-based material. Patients with a higher level of education were more likely to prefer written material. Patients emphasized simple language and incorporation of actual patient stories. CONCLUSION: Hospitalized patients with MDROs in our study believed that involvement on their part was important. Individuals' level of education affected their preference regarding choice of educational material, but most patients prefered receiving written or verbal information. Patient preferences must be incorporated into education to increase engagement for prevention of MDROs and HAIs. | Male | Humans | Patient Participation | Questionnaires | OK |
| Robert L. | Askew | Judy Lynn | Palmer | Merrick I. | Ross | Melanoma Research | 2012-04-00 Apr 2012 | Respondent burden has been defined as the cumulative demand placed on study participants related to the use of questionnaires or measurement instruments. The aim of this study was to reduce respondent burden associated with the Functional Assessment of Cancer Therapy-Melanoma (FACT-M), a melanoma-specific quality-of-life questionnaire, through item reduction using multiple psychometric approaches. Data for this study were pooled from three institutional review board-approved protocols. Poorly performing items were identified through distributional and correlation analyses, confirmatory factor analysis, reliability estimation, and Rasch-based approaches in a developmental dataset, and the reduced scale was assessed in a separate testing cohort. Validity, reliability, goodness of fit, and Rasch-based testing were carried out for both the full and the reduced scales. The clinical characteristics of the development (n=198) and testing (n=204) cohorts were similar. Three items identified through classical psychometric approaches and three items identified by Rasch-based analyses were excluded from the FACT-M subscale. Two additional items were identified for potential reduction but were ultimately maintained due to the adverse consequences to the psychometric integrity of the reduced instrument. The reduced FACT-M module contains 18 items. In addition to psychometric assessment, expert consultation was essential when examining areas of content redundancy and was critical when considering specific items for removal. This methodological approach reduced respondent burden by 25% while maintaining the psychometric integrity of the FACT-M. | Humans | Patient Participation | Patient Satisfaction | Questionnaires | OK |
| Malcolm | Cox | Lesley | Foulkes |  |  | British Journal of Neurosurgery | 2011-06-00 Jun 2011 | BACKGROUND: Subarachnoid haemorrhage (SAH) is neurological catastrophe, creating major disruption for patient and family, hence the importance of considering Patient-Related-Outcome-Measures (PROM). This study uses the National Study of SAH (2006) to explore any fiscal benefits to patients and NHS if they had an enhanced Neuro-Vascular-Specialist-Nurse (NVSN) service compared to Treatment-as-Usual (TAU). METHOD: Ensuring total confidentiality, clinical data from the National Study (n=2397) were matched with regional clinical data of a TAU (n=137) and prospective NVSN service (n=184) patients. The TAU and NVSN fiscal outcomes were projected onto the National Study patients to provide estimates of the potential benefits that could accrue nationally from a NVSN service based upon length of stay and earlier return to work of patients and carers. RESULTS: There were substantial benefits for NVSN cohort related to shorter time in hospital, reduced family disruption, earlier return to work and fiscal benefits to family and the NHS. NVSN patients and carers potential savings were estimated at £ 8.097 million and £ 2.492 million to the service, £ 10.497 million overall. PRACTICE IMPLICATIONS: This PROM approach allows the 'patient's voice' to be heard, which facilitates speedier patient and family recovery, showing that an integrated treatment approach in 'high tech' neuro-surgery is cost-effective. | Male | Humans | Patient Participation | Patient Satisfaction | OK |
| Richard | Madden |  |  |  |  | Disability and Rehabilitation: An International, Multidisciplinary Journal | 2013-06-00 juin 2013 | Purpose: This study describes a case formulation approach applying a prospective ICF derived clinical tool to assess rehabilitation needs for a community dwelling stroke survivor with care from an outpatient rehabilitation medicine clinic. Method: Case history data on the person were assessed for rehabilitation management planning using a prospective tool to interlink current with projected future functional status in everyday settings. Implicit assessment with reflective action informed decision points at each stage of the rehabilitation process. Results: As a result of reflective action using the prospective tool, rehabilitation management led to significant changes in client participation after limitations to mobility and self care were mapped to the living conditions of the stroke survivor. The context sensitive rehabilitative plan resulted in higher subjective health-related quality of life in the stroke survivor and significant other and enhanced their capacity for participation. Conclusions: Reflective action informed assessment applying ICF concepts to clinical problem solving resulted in positive gains in health-related quality of life in a stroke survivor. (PsycINFO Database Record (c) 2014 APA, all rights reserved). (journal abstract) | Client Participation | Clinical Trials | needs assessment | action assessment | OK |
| E. S. | Lebedeva | A. A. | Lysenko | M. M. | Shmarov | Acta Naturae | 2014-10-00 Oct 2014 | Replication-defective adenoviral vectors are effective molecular tools for both gene therapy and gene vaccination. Using such vectors one can deliver and express target genes in different epithelial, liver, hematopoietic and immune system cells of animal and human origin. The success of gene therapy and gene vaccination depends on the production intensity of the target protein encoded by the transgene. In this work, we studied influence of Toll-like receptors (TLR) agonists on transduction and expression efficacy of adenoviral vectors in animal and human antigen-presenting cells. We found that agonists of TLR2, 4, 5, 7, 8 and 9 significantly enhance a production of the target protein in cells transduced with adenoviral vector having the target gene insert. The enhancement was observed in dendritic cells and macrophages expressing cytoplasmic (GFP), membrane (HA) or secretory (SEAP) proteins encoded by the respective rAd-vectors. Experiments in mice showed that enhancement of the transgene expression can be achieved in the organism of animals using a pharmaceutical-grade TLR4-agonist. In contrast to other TLR-agonists, the agonist of TLR3 substantially suppressed the expression of transgene in cells transduced with adenoviral vectors having insert of GFP or SEAP target genes. We propose that the enhancement of transgene expression is linked to the activation of MyD88→ NF-kB, while the inhibition of transgene expression depends on TRIF→ IRF signaling pathways. Both of these pathways jointly exploited by TLR4-agonists lead to the enhancement of transgene expression due to the dominant role of the MyD88→ NF-kB signaling. | | | | | OK |
|  |  |  |  |  |  | Die Rehabilitation | 2014-02-00 Feb 2014 | BACKGROUND: [corrected] The goal orientation of rehabilitation has increasingly found its way in research and practice. Though, shortcomings were ascertained for the implementation into the clinical routine furthermore. An empirical analysis of goal setting in clinical practice should complement the available evidence. METHODS: The study is based on a cross-sectional survey of all endometriosis patients, who had been treated at a rehabilitation center in 2008 or 2009. Data stemmed from the uniform medical rehabilitation discharge reports, and a patient questionnaire, which was sent to the registered women by the clinic routinely before the beginning of the rehabilitation. Data about rehabilita­tion goals were available as free text information for both the discharge reports as well as the pa­tient questionnaires. A category system oriented at the ICF system (International Classification of Functioning, Disability and Health), was developed to structure the goals. RESULTS: 293 patients had provided information concerning their rehabilitation goals, while 69 patients didn’t reply to the question of their rehabilitation goals. The latter were excluded to the analysis. In the discharge reports, altogether, more goals were documented than in the patient questionnaires (5.98 compared to 4.97 goals per patient). In 35% of the numbers of rehabilitation goals clear congruence was found between the data in the discharge reports and the patient questionnaires. CONCLUSION: The results support the assump­tion that the included patients had difficulties in goal-setting. In addition it is obvious that the goals in the discharge reports had little relation to the goals framed by the patients, in language and content. A goal-setting process less oriented towards the medical and clinical needs but more towards the patients needs and expectation would be desirable particularly with regard to a more intensive patient orientation. The implementation of a prepared list of possible rehabilitation goals could serve for the shared goal-setting process in the context of anamnesis. | Humans | Patient Participation | Questionnaires | Young Adult | OK |
| Jens | Søndergaard | Bo | Christensen |  |  | Scandinavian Journal of Primary Health Care | 2011-12-00 Dec 2011 | OBJECTIVE: To describe the rehabilitation status three months after first-time myocardial infarction (MI) to identify focus areas for long-term cardiac rehabilitation (CR) in general practice. Design. Population-based cross-sectional study. SETTING AND SUBJECTS: Patients with first-time MI in 2009 from the Central Denmark Region. Data were obtained from patient questionnaires and from registers. RESULTS: Of the 1288 eligible patients, 908 (70.5%) responded. The mean (SD) age was 67.1 (11.7) years and 626 (68.9%) were men. Overall, 287 (31.6%) of the patients lived alone and 398 (45.4%) had less than 10 years of education. Upwards of half (58.5%) of the patients stated that they had participated in hospital-based rehabilitation shortly after admission. A total of 262 (29.2%) were identified with anxiety or depressive disorder or both, according to the Hospital Anxiety and Depression Scale. Of these, 78 (29.8%) reported that they had participated in psychosocial support, and 55 (21.0%) used antidepressants. One in five patients smoked three months after MI although nearly half of the smokers had stopped after the MI. Regarding cardioprotective drugs, 714 (78.6%) used aspirin, 694 (76.4%) clopidogrel, 756 (83.3%) statins, and 735 (81.0%) beta-blockers. CONCLUSION: After three months, there is a considerable potential for further rehabilitation of MI patients. In particular, the long-term CR should focus on mental health, smoking cessation, and cardioprotective drugs. | Time Factors | Male | Humans | Patient Participation | OK |
| Barbara J. | Bowers | Jean S. | Anthony | Bas | Tidjani | Annals of Family Medicine | 2010-06-00 2010 May-Jun | PURPOSE: Despite the sophisticated development of depression instruments during the past 4 decades, the critical topic of how primary care clinicians actually use those instruments in their day-to-day practice has not been investigated. We wanted to understand how primary care clinicians use depression instruments, for what purposes, and the conditions that influence their use. METHODS: Grounded theory method was used to guide data collection and analysis. We conducted 70 individual interviews and 3 focus groups (n = 24) with a purposeful sample of 70 primary care clinicians (family physicians, general internists, and nurse practitioners) from 52 offices. Investigators' field notes on office practice environments complemented individual interviews. RESULTS: The clinicians described occasional use of depression instruments but reported they did not routinely use them to aid depression diagnosis or management; the clinicians reportedly used them primarily to enhance patients' acceptance of the diagnosis when they anticipated or encountered resistance to the diagnosis. Three conditions promoted or reduced use of these instruments for different purposes: the extent of competing demands for the clinician's time, the lack of objective evidence of depression, and the clinician's familiarity with the patient. No differences among the 3 clinician groups were found for these 3 conditions. CONCLUSIONS: Depression instruments are reinvented by primary care clinicians in their real-world primary care practice. Although depression instruments were originally conceptualized for screening, diagnosing, or facilitating the management of depression, our study suggests that the real-world practice context influences their use to aid shared decision making-primarily to suggest, tell, or convince patients to accept the diagnosis of depression. | Decision Making | Humans | Questionnaires | Psychometrics | OK |
| Barbara J. | Bowers | Jean S. | Anthony | Bas | Tidjani | Annals of Family Medicine | 2010-05-00 mai 2010 | Purpose: Despite the sophisticated development of depression instruments during the past 4 decades, the critical topic of how primary care clinicians actually use those instruments in their day-to-day practice has not been investigated. We wanted to understand how primary care clinicians use depression instruments, for what purposes, and the conditions that influence their use. Methods: Grounded theory method was used to guide data collection and analysis. We conducted 70 individual interviews and 3 focus groups (n = 24) with a purposeful sample of 70 primary care clinicians (family physicians, general internists, and nurse practitioners) from 52 offices. Investigators’ field notes on office practice environments complemented individual interviews. Results: The clinicians described occasional use of depression instruments but reported they did not routinely use them to aid depression diagnosis or management; the clinicians reportedly used them primarily to enhance patients’ acceptance of the diagnosis when they anticipated or encountered resistance to the diagnosis. Three conditions promoted or reduced use of these instruments for different purposes: the extent of competing demands for the clinician’s time, the lack of objective evidence of depression, and the clinician’s familiarity with the patient. No differences among the 3 clinician groups were found for these 3 conditions. Conclusions: Depression instruments are reinvented by primary care clinicians in their real-world primary care practice. Although depression instruments were originally conceptualized for screening, diagnosing, or facilitating the management of depression, our study suggests that the real-world practice context influences their use to aid shared decision making—primarily to suggest, tell, or convince patients to accept the diagnosis of depression. (PsycINFO Database Record (c) 2012 APA, all rights reserved). (journal abstract) | Primary Health Care | primary care | diagnosis | depression | Doublon |
| Stephanie R. | Land | Thomas B. | Julian | Ann M. | Brown | The Journal of Supportive Oncology | 2013-03-00 Mar 2013 | BACKGROUND: The impact of arm morbidity following breast cancer surgery on patient-observed changes in daily functioning and health-related quality of life (HRQoL) has not been well-studied. OBJECTIVE: To examine the association of objective measures such as range of motion (ROM) and lymphedema, with patient-reported outcomes (PROs) in the arm and breast, upper extremity function, activities, and HRQoL. METHODS: The National Surgical Adjuvant Breast and Bowel Project Protocol B-32 was a randomized trial comparing sentinel node resection (SNR) with axillary dissection (AD) in women with node-negative breast cancer. ROM and arm volume were measured objectively. PROs included symptoms; arm function; limitations in social, recreational, occupational, and other regular activities; and a global index of HRQoL. Statistical methods included cross-tabulations and multivariable linear regression models. RESULTS: In all, 744 women provided at least 1 postsurgery assessment. About one-third of the patients experienced arm mobility restrictions. A similar number of patients avoided the use of the arm 6 months after surgery. Limitations in work and other regular activities were reported by about a quarter of the patients. In this multivariable analysis, arm mobility and sensory neuropathy were predictors of patient-reported arm function and overall HRQoL. Predictors for activity limitations also included side of surgery (dominant vs nondominant). Edema was not significant after adjustment for sensory neuropathy and ROM. LIMITATIONS: Arm mobility and edema were measured simultaneously only once during the follow-up (6 months). CONCLUSION: Clinical measures of sensory neuropathy and restrictions in arm mobility following breast cancer surgery are associated with self-reported limitations in activity and reductions in overall HRQoL. | Humans | Patient Participation | Female | Middle Aged | OK |
| Pamela | Semanik | Cheryl | Cox | Dorothy | Dunlop | Arthritis Care & Research | 2011-12-00 Dec 2011 | OBJECTIVE: To determine the relationship between beliefs, motivation, and worries about physical activity and physical activity participation in persons with rheumatoid arthritis (RA). METHODS: A cross-sectional study used baseline data from 185 adults with RA enrolled in a randomized clinical trial assessing the effectiveness of an intervention to promote physical activity. Data included patients' self-reported beliefs that physical activity can be beneficial for their disease, motivation for physical activity participation, worries about physical activity participation, and average daily accelerometer counts of activity over a week's time. Body mass index (BMI), sex, age, race, and disease activity were measured as potential statistical moderators of physical activity. RESULTS: Physical activity participation was greater for those with higher scores on scales measuring beliefs that physical activity is beneficial for their disease (P for trend = 0.032) and motivation for physical activity participation (P for trend = 0.007) when adjusted for age, sex, BMI, race, and disease activity. There was a positive but nonsignificant trend in physical activity participation in relation to worries. CONCLUSION: Stronger beliefs that physical activity can be helpful for managing disease and increased motivation to engage in physical activity are related to higher levels of physical activity participation. These data provide a preliminary empirical rationale for why interventions targeting these concepts should lead to improved physical activity participation in adults with RA. | Male | Emotions | Humans | Patient Participation | OK |
| Meike | Müller‐Engelmann | Monika Heinzel | Gutenbrunner | Tanja | Krones | Health Expectations: An International Journal of Public Participation in Health Care & Health Policy | 2012-12-00 décembre 2012 | Objective: To examine the psychometric properties of the German version of the observing patient involvement scale (OPTION) by analysing video recordings of primary care consultations dealing with counselling in cardiovascular prevention. Design: Cross-sectional assessment of physician–patient interaction by two rater pairs and two experts in shared decision making (SDM). Setting: Primary care. Participants: Fifteen general practitioners provided 40 videographed consultations. Measurements: Video ratings using the OPTION instrument. Results: Mean differences on item level between the four raters were quite large. Most items were skewed towards minimal levels of shared decision making. Measures of inter-rater association showed low to moderate associations on item level and high associations on total score level. Cronbach-α of the whole scale based on the data of all four raters is 0.90 and therefore on a high level. An oblique factor analysis revealed two factors, but both factors were highly correlated so we can confirm a one-dimensional structure of the instrument. ROC analyses between the rater total scores and dichotomized expert ratings (SDM yes/no) revealed a good discriminability of the OPTION total score. Physicians with more expertise in shared decision making received higher OPTION ratings. Conclusions: The German version of the OPTION scale is reliable at total score level. Some items need further revision in the direction of more concrete, observable behaviour. We were only able to perform a quasi-validation of the scale. Validity issues need further research efforts. (PsycINFO Database Record (c) 2013 APA, all rights reserved). (journal abstract) | Psychometrics | test validity | test reliability | Primary Health Care | Doublon |
| Meike | Müller-Engelmann | Monika Heinzel | Gutenbrunner | Tanja | Krones | Health Expectations: An International Journal of Public Participation in Health Care and Health Policy | 2012-12-00 Dec 2012 | OBJECTIVE: To examine the psychometric properties of the German version of the 'observing patient involvement' scale (OPTION) by analysing video recordings of primary care consultations dealing with counselling in cardiovascular prevention. DESIGN: Cross-sectional assessment of physician-patient interaction by two rater pairs and two experts in shared decision making (SDM). SETTING: Primary care. PARTICIPANTS: Fifteen general practitioners provided 40 videographed consultations. MEASUREMENTS: Video ratings using the OPTION instrument. RESULTS: Mean differences on item level between the four raters were quite large. Most items were skewed towards minimal levels of shared decision making. Measures of inter-rater association showed low to moderate associations on item level and high associations on total score level. Cronbach-α of the whole scale based on the data of all four raters is 0.90 and therefore on a high level. An oblique factor analysis revealed two factors, but both factors were highly correlated so we can confirm a one-dimensional structure of the instrument. ROC analyses between the rater total scores and dichotomized expert ratings (SDM yes/no) revealed a good discriminability of the OPTION total score. Physicians with more expertise in shared decision making received higher OPTION ratings. CONCLUSIONS: The German version of the OPTION scale is reliable at total score level. Some items need further revision in the direction of more concrete, observable behaviour. We were only able to perform a quasi-validation of the scale. Validity issues need further research efforts. | Male | Decision Making | Humans | Patient Participation | OK |
| Luikje | van der Dussen | Albert | Kap | Vera P. M. | Schepers | Journal of Rehabilitation Medicine | 2010-09-00 Sep 2010 | OBJECTIVE: To assess the reproducibility of 3 participation measures. DESIGN: Repeated administration of a postal questionnaire with a 2-week interval. PARTICIPANTS: Outpatients (n = 47) from 2 rehabilitation centres and a university hospital in The Netherlands. METHODS: Measures were the ICF Measure of Participation and Activities Screener (IMPACT-S), the Participation Scale, and the Utrecht Scale for Evaluation of Rehabilitation-Participation (USER-P). Test-retest reliability was analysed using Cohen's weighted kappa and the intraclass correlation coefficient (ICC). Agreement was expressed as the standard error of measurement and the smallest detectable change (SDC), substantiated as the ratio between the SDC and the standard deviation (SDC/SD). RESULTS: ICC values of the IMPACT-S were 0.54-0.90 for the scale scores, 0.92 and 0.74 for sub-total scores Activities and Participation, and 0.88 for the total score. The ICC of the Participation Scale was 0.82. The ICC of the USER-P was 0.65 for the Frequency scale, 0.85 for the Restrictions scale, and 0.84 for the Satisfaction scale. The SDC/SD ratios for all measures were small (0.11-0.28) at the group level, but large (0.78-1.91) at the individual level. Most participants found all measures relevant and easy to complete. CONCLUSION: All 3 measures showed generally satisfying reproducibility and were acceptable to the participants. | Male | Humans | Patient Participation | Questionnaires | OK |
| Anja M. C. | Horemans | Baziel G. M. | van Engelen |  |  | Disability and Rehabilitation | 2013-03-00 Mar 2013 | PURPOSE: This paper describes a study in which patients with neuromuscular disease (NMD) were engaged to list top-priorities for scientific research in order to complement the researchers' agenda. METHOD: A dialogic model for research agenda setting was used. Interviews, focus groups and expert meetings with patients were held to identify research topics. Research topics were prioritized via a questionnaire. Agendas were integrated in a dialogue meeting with professionals and patients. RESULTS: The research agenda of NMD patients is divided in four research domains, with a total of 24 research topics. These domains include (1) health; (2) quality of life; (3)quality of care and support, and (4) basic issues. Among the research domains highest priority was given to research on health, followed by research on quality of life. CONCLUSIONS: Both patients and professionals agreed a proper balance needs to be found between fundamental research and research on symptomatic treatment and quality of life. They concluded that more attention is required for research on the effective treatment of symptoms, quality of life and implementation of knowledge about NMD in regular care. | Male | Humans | Patient Participation | Questionnaires | OK |
| Beatrice Pui Yee | Lai | Lai Loi | Lee | Kwong Wai | Choy | International Urogynecology Journal | 2013-02-00 Feb 2013 | INTRODUCTION AND HYPOTHESIS: This study evaluated the responsiveness and minimal important differences (MID) of the Chinese Pelvic Floor Distress Inventory (PFDI) and Pelvic Floor Impact Questionnaire (PFIQ) in women undergoing urodynamic stress incontinence (USI) and/or pelvic organ prolapse (POP) treatment. METHODS: One hundred and fifty-six women were assessed using the PFDI and PFIQ before and after they received continence surgery and or pelvic floor repair (PFR) surgery, or vaginal pessary. Symptom severity was recorded using a visual analog scale (VAS) before and after treatment as was rating of their satisfaction with the treatment they received. Responsiveness of the PFDI and PFIQ were evaluated by effect sizes, standardized response mean, paired samples t test or Wilcoxon Signed Rank Test. MID in the PFDI and PFIQ for different treatments were determined by satisfaction rating, change in VAS scoring, and distribution-based methods. RESULTS: There were significant improvements in the respective subscales of PFDI and PFIQ, demonstrating moderate to great responsiveness after different treatments. The estimation of MID for the Urinary Distress Inventory (UDI) was -30 to -14 and the Urinary Impact Questionnaire (UIQ) was -28 to -14 for women who underwent continence surgery. The MID for the Pelvic Organ Prolapse Distress Inventory (POPDI) was -44 to -21, the Pelvic Organ Prolapse Impact Questionnaire (POPIQ) -40 to -27, the UDI -22 to -16, the UIQ -37 to -31, the Colo-Rectal-Anal Distress Inventory (CRADI) -37 to -14, and the Colo-Rectal-Anal Impact Questionnaire (CRAIQ) -34 to -6 for women who underwent PFR surgery; and estimation of MID for the POPDI was -16, the POPIQ -29, the UDI -28, the UIQ -17, the CRADI -25, and the CRAIQ -31 for women who received a vaginal pessary. The MID of the respective subscales were supported by the distribution-based methods. CONCLUSIONS: The Chinese PFDI and PFIQ instruments are responsive to change in women undergoing continence surgery, PFR surgery or vaginal pessary for USI or POP. | Humans | Patient Participation | Patient Satisfaction | Questionnaires | OK |
| Birgitta | Pålsson | Gunnel | Abjörnsson | Birgitta | Malmberg | BMC public health | 2010-00-00 2010 | BACKGROUND: In the present study the effect of a workplace-oriented intervention for persons on long-term sick leave for clinical burnout, aimed at facilitating return to work (RTW) by job-person match through patient-supervisor communication, was evaluated. We hypothesised that the intervention group would show a more successful RTW than a control group. METHODS: In a prospective controlled study, subjects were identified by the regional social insurance office 2-6 months after the first day on sick leave. The intervention group (n = 74) was compared to a control group who had declined participation, being matched by length of sick leave (n = 74). The RTW was followed up, using sick-listing register data, until 1.5 years after the time of intervention. RESULTS: There was a linear increase of RTW in the intervention group during the 1.5-year follow-up period, and 89% of subjects had returned to work to some extent at the end of the follow-up period. The increase in RTW in the control group came to a halt after six months, and only 73% had returned to work to some extent at the end of the 1.5-year follow-up. CONCLUSIONS: We conclude that the present study demonstrated an improvement of long-term RTW after a workplace-oriented intervention for patients on long-term sick leave due to burnout. TRIAL REGISTRATION: Current Controlled Trials NCT01039168. | Male | Humans | Patient Participation | Questionnaires | OK |
| Sheilah | Hogg-Johnson | Hyunmi | Lee | Niklas | Krause | Journal of Occupational Rehabilitation | 2011-06-00 Jun 2011 | INTRODUCTION We report on the development and validation of a 10-item scale assessing self-efficacy within the return-to-work context, the Return-to-Work Self-Efficacy (RTWSE) scale. METHODS Lost-time claimants completed a telephone survey 1 month (n = 632) and 6 months (n = 446) after a work-related musculoskeletal injury. Exploratory (Varimax and Promax rotation) and confirmatory factor analyses of self-efficacy items were conducted with two separate subsamples at both time points. Construct validity was examined by comparing scale measurements and theoretically derived constructs, and the phase specificity of RTWSE was studied by examining changes in strength of relationships between the RTWSE Subscales and the other constructs at both time measures. RESULTS Factor analyses supported three underlying factors: (1) Obtaining help from supervisor, (2) Coping with pain (3) Obtaining help from co-workers. Internal consistency (alpha) for the three subscales ranged from 0.66 to 0.93. The total variance explained was 68% at 1-month follow-up and 76% at 6-month follow-up. Confirmatory factor analyses had satisfactory fit indices to confirm the initial model. With regard to construct validity: relationships of RTWSE with depressive symptoms, fear-avoidance, pain, and general health, were generally in the hypothesized direction. However, the hypothesis that less advanced stages of change on the Readiness for RTW scale would be associated with lower RTWSE could not be completely confirmed: on all RTWSE subscales, RTWSE decreased significantly for a subset of participants who started working again. Moreover, only Pain RTWSE was significantly associated with RTW status and duration of work disability. With regard to the phase specificity, the strength of association between RTWSE and other constructs was stronger at 6 months post-injury compared to 1 month post-injury. CONCLUSIONS A final 10-item version of the RTWSE has adequate internal consistency and validity to assess the confidence of injured workers to obtain help from supervisor and co-workers and to cope with pain. With regard to phase specificity, stronger associations between RTWSE and other constructs at 6-month follow-up suggest that the association between these psychological constructs consolidates over time after the disruptive event of the injury. | Humans | Patient Participation | Questionnaires | self efficacy | OK |
| Melanie G. | Hagen |  |  |  |  | Patient Education and Counseling | 2012-09-00 Sep 2012 | OBJECTIVE: To assess physician needs for patient-centered communication training for medical consultations and to develop an updated patient training curriculum. METHODS: An online needs assessment was distributed through physician email listserves at the University of Florida College of Medicine. Frequency tabulation and content analyses were conducted to assess patient communication themes. RESULTS: Responses were received from 336 physicians. Physicians reported that patients are reluctant to ask questions when they do not understand information related to their medical condition, treatment plan, or medical advice. Furthermore, physicians reported that a lack of patient negotiation inhibits patient-centered communication and may negatively influence patient adherence and compliance. Based on these results the AGENDA model was created for patient training and consists of (1) agenda setting; (2) goals for health; (3) expressing concerns, questions, and negotiations; (4) navigating health literacy issues; (5) disclosing detailed information; and (6) active types of listening. CONCLUSION: This study supports revisiting patient communication training and tailoring future training interventions to specific communities. PRACTICE IMPLICATIONS: The AGENDA model can be used to train patients to enhance patient-centered communication with physicians. Additionally, patient communication training could help to address the barriers to care identified by the physicians in our study. | Male | Humans | Patient Participation | Physician-Patient Relations | OK |
| Z. | Khadr | H. F. | El-Sayed |  |  | Clinical and Experimental Obstetrics & Gynecology | 2014-00-00 2014 | PURPOSE OF INVESTIGATION: To investigate the effect of social class, education and antenatal care on cesarian section (CS) rate and women's preference for CS. MATERIALS AND METHODS: This is a descriptive study performed on 509 women attending postnatal clinics in three primary care units in Ismailia governorate, Egypt. The study performed via a structured questionnaire including questions related to background socio-demographic data, questions related to obstetrical history for the last pregnancy and delivery, and questions related to economic standards. RESULTS: The study included 266 participants (52.3%) that delivered by CS in their last delivery. CS rate was significantly higher in highly educated women and high economic standard. Only 29 (7.7%) reported that they had preferred CS as a method of delivery. Educational level and economic standards significantly affected the proportion of women preferring CS as a method of delivery (p = 0.02 and p = 0.01, respectively). In a stepwise logistic regression model, significant factors affecting CS rate were: the method of delivery preferred (p < 0.005) and educational level (p < 0.05). CONCLUSION: Educational level and economic standards significantly affected women's preference towards CS and CS rate. | Decision Making | Humans | Patient Participation | Questionnaires | OK |
| Bonnie J. | Sherman | Timothy C. | Heeren | Tracy A. | Battaglia | Health Expectations: An International Journal of Public Participation in Health Care and Health Policy | 2013-07-30 Jul 30, 2013 | BACKGROUND: Shared decision making (SDM) related to test preference has been advocated as a potentially effective strategy for increasing adherence to colorectal cancer (CRC) screening, yet primary care providers (PCPs) are often reluctant to comply with patient preferences if they differ from their own. Risk stratification advanced colorectal neoplasia (ACN) provides a rational strategy for reconciling these differences. OBJECTIVE: To assess the importance of risk stratification in PCP decision making related to test preference for average-risk patients and receptivity to use of an electronic risk assessment tool for ACN to facilitate SDM. DESIGN: Mixed methods, including qualitative key informant interviews and a cross-sectional survey. PARTICIPANTS: PCPs at an urban, academic safety-net institution. MAIN MEASURES: Screening preferences, factors influencing patient recommendations and receptivity to use of a risk stratification tool. KEY RESULTS: Nine PCPs participated in interviews and 57 completed the survey. Despite an overwhelming preference for colonoscopy by 95% of respondents, patient risk (67%) and patient preferences (63%) were more influential in their decision making than patient comorbidities (31%; P < 0.001). Age was the single most influential risk factor (excluding family history), with <20% of respondents choosing factors other than age. Most respondents reported that they would be likely to use a risk stratification tool in their practice either 'often' (43%) or sometimes (53%). CONCLUSIONS: Risk stratification was perceived to be important in clinical decision making, yet few providers considered risk factors other than age for average-risk patients. Providers were receptive to the use of a risk assessment tool for ACN when recommending an appropriate screening test for select patients. | | | | | OK |
| H. | Beintema | T. | Mulder | T. W. D. P. | van Os | The British Journal of Psychiatry | 2013-05-00 mai 2013 | Background: Forensic psychiatry aims to reduce recidivism and makes use of risk assessment tools to achieve this goal. Various studies have reported on the predictive qualities of these instruments, but it remains unclear whether their use is associated with actual prevention of recidivism in clinical care. Aims: To test whether an intervention combining risk assessment and shared care planning is associated with a reduction in violent and criminal behaviour. Method: A cluster randomised controlled trial (Netherlands Trial Register number NTR1042) was conducted in three outpatient forensic psychiatric clinics. The intervention comprised risk assessment with the Short Term Assessment of Risk and Treatability (START) and a shared care planning protocol formulated according to shared decision-making principles. The control group received usual care. The outcome consisted of the proportion of clients with violent or criminal incidents at follow-up. Results: In total 58 case managers and 632 of their clients were included. In the intervention group (n = 310), 65% received the intervention at least once. Findings showed a general treatment effect (22% of clients with an incident at baseline v. 15% at follow-up, P &lt; 0.01) but no significant difference between the two treatment conditions (odds ratio (OR) = 1.46, 95% CI 0.89–2.44, P = 0.15). Conclusions: Although risk assessment is common practice in forensic psychiatry, our results indicate that the primary goal of preventing recidivism was not reached through risk assessment embedded in shared decision-making. (PsycINFO Database Record (c) 2013 APA, all rights reserved). (journal abstract) | Decision Making | Clinical Trials | Outpatients | risk assessment | Doublon |
| H. | Beintema | T. | Mulder | T. W. D. P. | van Os | The British Journal of Psychiatry: The Journal of Mental Science | 2013-05-00 May 2013 | BACKGROUND: Forensic psychiatry aims to reduce recidivism and makes use of risk assessment tools to achieve this goal. Various studies have reported on the predictive qualities of these instruments, but it remains unclear whether their use is associated with actual prevention of recidivism in clinical care. AIMS: To test whether an intervention combining risk assessment and shared care planning is associated with a reduction in violent and criminal behaviour. METHOD: A cluster randomised controlled trial (Netherlands Trial Register number NTR1042) was conducted in three out-patient forensic psychiatric clinics. The intervention comprised risk assessment with the Short Term Assessment of Risk and Treatability (START) and a shared care planning protocol formulated according to shared decision-making principles. The control group received usual care. The outcome consisted of the proportion of clients with violent or criminal incidents at follow-up. RESULTS: In total 58 case managers and 632 of their clients were included. In the intervention group (n = 310), 65% received the intervention at least once. Findings showed a general treatment effect (22% of clients with an incident at baseline v. 15% at follow-up, P<0.01) but no significant difference between the two treatment conditions (odds ratio (OR) = 1.46, 95% CI 0.89-2.44, P = 0.15). CONCLUSIONS: Although risk assessment is common practice in forensic psychiatry, our results indicate that the primary goal of preventing recidivism was not reached through risk assessment embedded in shared decision-making. | Male | Decision Making | Humans | Young Adult | OK |
| V. | Mollo | E. | Lartigau |  |  | Bulletin Du Cancer | 2010-07-00 Jul 2010 | Radiation therapy was one of the first medical specialities to implement quality assurance (QA) programs in clinical practice. These QA programs have mainly focused on equipment performance neglecting human factors. Risk management is a relatively new approach in medical disciplines. Methods of evaluation and risk management must be identified, applied and adapted to the specificities of this domain. Based on the experience of industry, risk management will allow us to substantially improve safety and develop our own models of management. Recent experiences use these approaches to implement risk management in radiation therapy departments. | Humans | Patient Participation | risk assessment | Safety Management | OK |
| Julian | Thumboo | Cynthia | Goh | Ricardo | Pietrobon | BMC medical informatics and decision making | 2010-00-00 2010 | BACKGROUND: A Royal Statistical Society Working Party recently recommended that "Greater use should be made of numerical, as opposed to verbal, descriptions of risk" in first-in-man clinical trials. This echoed the view of many clinicians and psychologists about risk communication. As the clinical trial industry expands rapidly across the globe, it is important to understand risk communication in Asian countries. METHODS: We conducted a cognitive experiment about participation in a hypothetical clinical trial of a pain relief medication and a survey in cancer and arthritis patients in Singapore. In part 1 of the experiment, the patients received information about the risk of side effects in one of three formats (frequency, percentage and verbal descriptor) and in one of two sequences (from least to most severe and from most to least severe), and were asked about their willingness to participate. In part 2, the patients received information about the risk in all three formats, in the same sequence, and were again asked about their willingness to participate. A survey of preference for risk presentation methods and usage of verbal descriptors immediately followed. RESULTS: Willingness to participate and the likelihood of changing one's decision were not affected by the risk presentation methods. Most patients indicated a preference for the frequency format, but patients with primary school or no formal education were indifferent. While the patients used the verbal descriptors "very common", "common" and "very rare" in ways similar to the European Commission's Guidelines, their usage of the descriptors "uncommon" and "rare" was substantially different from the EU's. CONCLUSION: In this sample of Asian cancer and arthritis patients, risk presentation format had no impact on willingness to participate in a clinical trial. However, there is a clear preference for the frequency format. The lay use of verbal descriptors was substantially different from the EU's. | Male | Decision Making | Humans | Patient Participation | OK |
| Johannes | Blum | Reto | Nüesch | Christoph | Hatz | Journal of Travel Medicine | 2013-02-00 2013 Jan-Feb | BACKGROUND: This study assessed the risk perception ratings of travelers pre- and post-travel and in comparison to the ratings by travel health experts. While most surveys on travel health knowledge, attitudes, and practices focus on malaria and vaccine-preventable diseases, noninfectious travel risks were included in this study. METHODS: Pre- and post-travel perception of nine travel-associated health risks was recorded among 314 travelers to tropical and subtropical destinations. All travelers sought pre-travel health advice at the Travel Clinic of the Swiss Tropical and Public Health Institute in 2008 and 2009. In addition, 18 Swiss travel health experts provided an assessment of the respective risks. A validated visual psychometric measuring instrument was used [pictorial representation of illness and self measure (PRISM)]. RESULTS: Travelers and experts rated most risks similarly, except for accidents and sexually transmitted infections (STIs) which experts rated higher. Compared to other risks, accidents ranked highly in both groups and were the only risk perceived higher after travel. Pre- and post-travel perceptions of all other risks were similar with a tendency to be lower after travel. Travelers perceived mosquitoes to be the highest risk before travel and accidents after travel. CONCLUSION: Travelers' risk perception appears to be accurate for most risks stated in this study. However, travel health professionals should be aware that some perception patterns among travelers regarding travel-related health risks may be different from professional risk assessment. Important but insufficiently perceived health risks, such as sexual behavior/STIs and accidents, should be considered to be part of any pre-travel health advice package. | Time Factors | Male | Animals | Humans | OK |
| Sanjay | Mohanty | Jennifer L. | Paruch | Ravi | Rajaram | Journal of Surgical Oncology | 2014-10-00 Oct 2014 | Healthcare has increasingly focused on patient engagement and shared decision-making. Decision aids can promote engagement and shared decision making by providing patients and their providers with care options and outcomes. This article discusses decision aids for surgical oncology patients. Topics include: short-term risk prediction following surgery, long-term risk prediction of survival and recurrence, the combination of short- and long-term risk prediction to help guide treatment choice, and decision aid usability, transparency, and accessibility. | Decision Support Techniques | Humans | Health Literacy | Neoplasms | OK |
| Alethea | Paradis | Zachary | Ortbal |  |  | Pharmacoepidemiology and Drug Safety | 2011-03-00 Mar 2011 | PURPOSE: The present study was undertaken to assess the degree to which key informants' perceptions of relative abuse liability of opioids in their communities correspond to actual drug use in their patients. METHODS: Key informants (n = 49) and their patients (n = 738) were asked to complete anonymous, independent paper surveys about perceived or actual abuse of nine targeted opioid drugs: buprenorphine, fentanyl, heroin, hydrocodone, hydromorphone, methadone, morphine, oxycodone and tramadol. RESULTS: There was a strong correspondence (R = 0.98) between KI rankings of relative abuse of opioids in their patient population and the actual rank order calculated from their patients' selection of a primary euphorigenic opioid. The patient surveys not only validated the KI's conclusions, but also provided a great deal of important information about the nature of the abuse patterns. Notably, significant co-morbidity was found in the patient population: 55% had a history of psychiatric problems; chronic pain of moderate intensity was common; 67% were nicotine dependent; and 43% were diagnosed alcoholics. DISCUSSION: Our results demonstrate that KI's perceived rank order of drugs of choice correlated almost perfectly with their patients' choice of a primary drug. These data suggest that key informants, as they have for decades in epidemiological research, provide a reliable, high-level view of substance abuse in their catchment area. As such, KIs provide valid and reliable information on scope and patterns of abuse, but if more detail is required about the characteristics of abuse, such as that required for FDA-mandated Risk Evaluation and Mitigation Strategies, then direct interviews with the abusers themselves seem more appropriate. | Male | Humans | Patient Participation | Questionnaires | OK |
|  |  |  |  |  |  | Expert Review of Pharmacoeconomics & Outcomes Research | 2011-02-00 Feb 2011 | Health technology assessment (HTA) has become an integral part of decision-making on the coverage of new health technologies in most health systems in the developed world. In recent years, pressure to involve patients and members of the public in HTA has grown. In this article, we summarize findings from peer-reviewed and 'gray' literature, and discussions with key informants to determine potential roles for patients and the public in HTA and coverage decision-making. We also summarize existing roles for both groups in jurisdictions. Although there appears to be a general view that involvement of patients and the public is highly desirable, research offering insights into the effectiveness of different approaches to accomplish this is scarce. Nonetheless, many of the HTA agencies in developed countries have established some mechanism for seeking input from patients or the public in their processes. | Decision Making | Humans | Patient Participation | Consumer Participation | OK |
| I. | Van Herzeele | M. | Plessers | N. | Stroobant | The British Journal of Surgery | 2012-09-00 Sep 2012 | BACKGROUND: Recent advances in simulation science permit patient-specific rehearsal of endovascular stenting procedures. This study aimed to evaluate how effectively real interventions are replicated by patient-specific rehearsal technology, and to assess its value as a preparatory tool for the interventionalist and the operating team. METHODS: All patients deemed candidates for carotid artery stenting procedures with suitable computed tomography images were enrolled. Each team member rehearsed the virtual procedure in the laboratory, simulated operating theatre or angiography suite environment immediately before treating the real patient. Dexterity and qualitative metrics were recorded. Subjective questionnaires used a Likert scale from 1 (poor) to 5 (excellent). RESULTS: Of 18 patients, three were excluded. In 11 of 15 and 13 of 15 patients respectively endovascular tool use and fluoroscopy angles were identical during rehearsal and the real procedure. In a third of patients, the simulator did not adequately predict difficulties in cannulating the stenotic internal or common carotid arteries. The procedure realism, value in evaluating the case, increase in efficiency in tool use, and potential to increase communication, confidence and team performance were all rated highly (4 of 5). CONCLUSION: Patient-specific rehearsal was rated highly for both face and content validity. Access strategy, endovascular material use and angiographic imaging were all replicated effectively, although certain biomechanical vessel properties seemed to be replicated to a lesser degree. Patient-specific rehearsal constitutes a unique tool that may help tailor endovascular material choice, and optimize the preoperative preparation of the interventionalist and team. | Male | Humans | Patient Participation | Questionnaires | OK |
| Vikki | Knott |  |  |  |  | Asian Pacific journal of cancer prevention: APJCP | 2013-00-00 2013 | BACKGROUND: The study investigated the influence of culturally-based health beliefs on engagement in healthy lifestyle behaviour. Specifically, the study compared levels of engagement between Western and Chinese youth in Australia and assessed the extent to which culture-specific attributions about the causes of illness, and health beliefs, predict engagement in healthy lifestyle behaviour. MATERIALS AND METHODS: Ninety-four Western and 95 Chinese (N=189; Mean Age=20.8 years, SD=3 years) young adults completed an online questionnaire. Predictor variables were cultural health beliefs measured by the Chinese Cultural Views on Health and Illness scale (CCVH, Liang et al., 2008), and illness attributions beliefs measured by the Cause of Illness Questionnaire (CIQ, Armstrong and Swartzman, 1999). Outcomes variables were levels of engagement in healthy lifestyle behaviour. RESULTS: Results indicated that Chinese participants have a significantly lower exercising rate and healthy dietary habits compared to the Western sample. Moreover, Chinese participants were found to believe more strongly than Westerners that cancer was associated with factors measured by the Traditional-Chinese-Model (TCM). Finally, the observed relationship between cultural health beliefs and physical inactivity was mediated by attributions of illness, in particular to the supernatural subscale, with the Sobel Test showing a significant mediation (z=-2.63, p=0.004). CONCLUSIONS: Mainstream approaches to encourage healthy lifestyles are unlikely to be effective when educating Chinese youth. Instead, health promotion programs should attempt to address the illness attribution beliefs and educate Chinese youth about the role of diet and exercise in prevention of diseases such as cancer. | Male | Humans | Patient Participation | Questionnaires | OK |
| Hannele | Turunen |  |  |  |  | Research in Nursing & Health | 2014-06-00 juin 2014 | Abstract We examined patients' experiences of patient safety and participation in promoting safe care during their most recent care period. A survey of patients (N = 175) revealed that treatment, medication, and device safety were mostly experienced as very good or excellent, but responses varied by age and experience. Patients ages 66–75 were most critical of treatment and medication safety. Device safety was rated the worst aspect of safety. Twenty percent of respondents had experienced errors at some time during their care. Patients who had experienced errors and those who were treated at inpatient wards versus a day surgery unit were most critical towards patient participation. Open and transparent error management involving patients is needed to promote treatment, medication, and especially device safety. © 2014 Wiley Periodicals, Inc. (PsycINFO Database Record (c) 2014 APA, all rights reserved). (journal abstract) | Medical Patients | Drug Therapy | Client Attitudes | patient safety | Doublon |
| Hannele | Turunen |  |  |  |  | Research in Nursing & Health | 2014-06-00 Jun 2014 | We examined patients' experiences of patient safety and participation in promoting safe care during their most recent care period. A survey of patients (N = 175) revealed that treatment, medication, and device safety were mostly experienced as very good or excellent, but responses varied by age and experience. Patients ages 66-75 were most critical of treatment and medication safety. Device safety was rated the worst aspect of safety. Twenty percent of respondents had experienced errors at some time during their care. Patients who had experienced errors and those who were treated at inpatient wards versus a day surgery unit were most critical towards patient participation. Open and transparent error management involving patients is needed to promote treatment, medication, and especially device safety. | Male | Humans | Patient Participation | Questionnaires | OK |
| Christina | Albohn-Kühne | Tanja | Krones | Norbert | Donner-Banzhoff | Evaluation & the Health Professions | 2010-09-00 Sep 2010 | Satisfaction with treatment is regarded as an important outcome measure, but its suitability has not been thoroughly investigated in the context of shared decision making (SDM). The authors evaluated whether both patients' and physicians' satisfaction ratings differ between an intervention group and a control group within a structured tool for cardiovascular prevention (ARRIBA-Herz). In a pragmatic, cluster-randomized, controlled trial, 44 family physicians in the intervention group consecutively recruited 550 patients whereas 47 physicians in the control group included 582 patients. Main findings were high satisfaction ratings independent of group allocation in patients and physicians. Significant differences had only negligible effect sizes. Compared to global satisfaction ratings, the effects of the shared decision-making process are better measured by a more concrete approach representing different steps of this process. Further research should refine behaviorally oriented questionnaires that measure SDM and a version for physicians should also be created. | Male | Decision Making | Humans | Physician-Patient Relations | OK |
| M. | Petrucci | R. | Hussein | W. | Micheelis | Gesundheitswesen (Bundesverband Der Ärzte Des Öffentlichen Gesundheitsdienstes (Germany)) | 2013-06-00 Jun 2013 | BACKGROUND: Patient satisfaction is a central topic in quality management in outpatient dental care. The ZAP questionnaire was validated to explore patient satisfaction in general and specialist outpatient settings. This study aims at assessing the psychometric properties of the ZAP in dental care. METHODS: A minimally modified version of the ZAP consisting of 4 domains (office organisation, cooperation, interaction, information) was administered in personal interviews to a population-based sample. Descriptive, exploratory and confirmatory psychometric analyses were conducted with random subsets of the study sample. RESULTS: The study population comprised 1 773 subjects with at least one dental visit during their lifetime (mean age=50 years, female=51.6%). The exploratory factor analysis identified 3 subscales (office organisation, interaction, information). Based on these results, items of the subscale "cooperation" were excluded from further analyses. The remaining items had a medium difficulty of 0.75, all item-total-correlations were above 0.4. Missing values ranged between 2.3% and 28.7%. Cronbach's alpha ranged between 0.79 and 0.95. After introduction of 3 residual correlations, the confirmatory factor analyses reached a good model fit (TLI: 0.97; CFI: 0.97, RMSEA: 0.06). Partial standardised factor loadings ranged between 0.77 and 0.87. The 3 latent factors were highly correlated. There was a positive correlation between the 3 subscales and global patient satisfaction with the dentist. CONCLUSION: The psychometric assessment can be used in the 3 modified subscales (office organisation, interaction, and information) to assess patient satisfaction with dental care. To assess dentist's competence in relation to dental anxiety and pain as well as shared decision making new scales specific to dental care should be explored. | Humans | Patient Satisfaction | Questionnaires | Young Adult | OK |
| Núria | Siñol | Maria J. | Portella | Víctor | Pérez | Journal of Clinical Psychopharmacology | 2012-02-00 Feb 2012 | There is a manifest lack of psychometrically sound instruments designed for specific and multidimensional assessment of satisfaction with methadone as a medication within the context of methadone maintenance treatment. Therefore, it may be worthwhile to assess the pertinence and utility of using a generic and multidimensional medication satisfaction instrument that has not been specifically developed for use in methadone maintenance treatment.The aim of this study was thus to explore the psychometric properties of the Spanish version of the Treatment Satisfaction Questionnaire for Medication (TSQM version 1.4 [Health Qual Life Outcomes. 2004;2:12]) in a sample of methadone-maintained heroin-dependent patients.Two hundred three methadone-maintained patients filled out the TSQM and other several measures related to the construct of patient satisfaction (eg, Verona Service Satisfaction Scale for methadone treatment). Dimensionality of the TSQM was assessed by means of a confirmatory factor analysis. Internal consistency was examined using the ordinal coefficient α. Spearman correlations were used to explore the relationship between the TSQM and the measures conceptually related to patient satisfaction.Regarding the dimensionality of the TSQM, its original factor structure adequately fitted the data (Satorra-Bentler χ58, 72.14 [P = 0.100]; root-mean-square error of approximation, 0.045; comparative fit index, 0.978). All but 1 of the 4 TSQM subscales showed acceptable to good internal consistency values (0.78-0.89). The dimensions of the TSQM were differentially and congruently correlated with related measures.The results strongly suggest the TSQM value as a brief, generic, and psychometrically sound instrument to assess satisfaction with methadone as a medication in a multidimensional manner. Notwithstanding, more research is needed not only to assess the generalizability of these findings but also to provide pieces of evidence for other psychometric properties, especially the TSQM predictive validity. | Male | Humans | Patient Participation | Patient Satisfaction | OK |
| Sofie | Van Roosbroeck | |  |  |  | European Journal of Cancer (Oxford, England: 1990) | 2011-09-00 Sep 2011 | PURPOSE: In this paper we investigate the reasons for non-participation in a trial programme for colorectal cancer (CRC) screening in Flanders. Besides, the feasibility and possibilities of a full-blown screening programme in Flanders are examined, given the context of a low participation rate in breast cancer screening. METHODS: A trial programme for CRC screening was set up for all average-risk persons aged 50 to 74 years in three Flemish regions to obtain information about potential participation rates, and to compare two invitation strategies. Faecal samples were analysed for precursors of CRC using an immunochemical faecal occult blood test (iFOBT). A questionnaire was administered to participants and non-participants of the trial programme to find out whether and to what extent the taking of a sample of one's own stool is a taboo. This could be of great importance concerning the participation rate. RESULTS: In total, 19,542 persons were invited to participate in the trial programme for CRC screening, of whom 18,541 were found to be eligible. The overall participation rate was 44.3%. The three regions varied in participation rates: with 60.9% in the most rural region to 34.3% in the most urban region. Of 2,000 questionnaires sent to participants, 1,385 were returned (69.3%). The response in the non-participants was 43.2%i (691 questionnaires of 1,600 returned). CONCLUSIONS: A population-based screening programme for colorectal cancer by means of an iFOBT proves to be feasible, since adequate participation rates were obtained and because of the absence of a 'stool taboo' in Flanders. | Male | Humans | Patient Participation | Questionnaires | OK |
| Hiroto | Ito | Päivi | Soininen | Hanna | Putkonen | Psychiatry and Clinical Neurosciences | 2012-08-00 Aug 2012 | AIM: To develop a standardized self-reporting questionnaire to evaluate patients' perceptions of their overall treatment in specific relation to the use of seclusion and/or restraint (SR) measures as part of the treatment program. METHODS: A 17-item self-rating questionnaire was given to 56 patients with experience of SR-related treatment to develop a new scale, the Secluded/Restrained Patients' Perceptions of their Treatment (SR-PPT). Concurrent validity was examined against the Client Satisfaction Questionnaire-8 Japanese Version (CSQ-8J). In addition, Patient burden induced by answering the SR-PPT was evaluated. RESULTS: On factor analysis, two factors named as Cooperation with Staff (nine items) and Perceptions of SR (two items) were derived. Cronbach's coefficient alphas were 0.928 and 0.887, and correlation coefficients against the CSQ-8J were 0.838 and 0.609, respectively. Answering the SR-PPT was found to induce little burden on the patients. CONCLUSION: Adequate internal consistency and concurrent validity of the final version of the SR-PPT, which consists of 11 items, indicate that it is acceptable as a measurement scale. Use of this questionnaire will add the patient's view to the assessment of overall treatment involving SR. | Male | Humans | Patient Participation | Patient Satisfaction | OK |
| David S. | Metzger |  |  |  |  | Vaccine | 2011-08-18 Aug 18, 2011 | Efficacy studies of investigational HIV vaccines require enrollment of individuals at 'high risk' for HIV. This paper examines participation in HIV vaccine trials among women at 'high risk' for HIV acquisition. In-depth interviews were conducted with 17 African-American women who use crack cocaine and/or exchange sex for money/drugs to elicit attitudes toward medical research and motivators and deterrents to HIV vaccine trial participation. Interviews were digitally recorded and transcribed; data were coded and compiled into themes. Most women expressed favorable attitudes toward medical research in general. Motivators for trial participation included compensation; personal benefits including information, social services, and the possibility that the trial vaccine could prevent HIV; and altruism. Deterrents included: dislike of needles; distrust; concern about future consequences of participating. In addition, contingencies, care-giving responsibilities, and convenience issues constituted barriers which could impede participation. Respondents described varied, complex perspectives, and individual cases illustrate how these themes played out as women contemplated trial participation. Understanding factors which influence vaccine research participation among women at 'high risk' can aid sites to tailor recruitment procedures to local contexts. Concerns about future reactions can be addressed through sustained community education. Convenience barriers can be ameliorated by providing rides to study visits when necessary, and/or conducting study visits in accessible neighborhood locations. Women in this sample thought carefully about enrolling in HIV vaccine trials given the structural constraints within which they lived. Further research is needed regarding structural factors which influence personal agency and individuals' thinking about research participation. | Humans | Patient Participation | Questionnaires | Female | OK |
| Wei-Hsian | Yin | Ching-Hui | Cheng | Tsae-Jyy | Wang | European Journal of Cardiovascular Nursing: Journal of the Working Group on Cardiovascular Nursing of the European Society of Cardiology | 2012-06-00 Jun 2012 | OBJECTIVE: The aim of this study was to describe the self-care behaviors and associated demographic factors affecting such behaviors in the heart failure population in Taiwan. METHODS: A descriptive, cross-sectional, correlational design was used in this study and 86 heart failure patients were sampled by convenience sampling. Demographic questionnaire and Self-Care of Heart Failure Index, V. 6 were used to collect these data. Descriptive statistic, correlation coefficient and multiple regression were used to analyze the data. RESULTS: The result indicated a low level of self-care maintenance and management although self-care confidence was adequate. Patients who were married had better self-care maintenance and self-care confidence than those who were single. Self-care confidence was a determinant of self-care maintenance. CONCLUSION: This study provided detailed information on self-care behavior status in heart failure patients in Taiwan. | Male | Humans | Patient Participation | Questionnaires | OK |
| D. | Stirnimann | S. | Ehrat | F. | Michel | Spinal Cord | 2012-09-00 Sep 2012 | STUDY DESIGN: A multi-centre cross-sectional study. OBJECTIVE: To examine the relationship of self-efficacy and self-esteem with participation of persons with spinal cord injury (SCI) from a comprehensive bio-psycho-social perspective, based on the conceptual framework of the International Classification of Functioning, Disability and Health (ICF). SETTING: Community-dwelling participants, <5 years post discharge, recruited through three SCI rehabilitation centers in Switzerland. METHODS: Data were collected by means of standardized self-report questionnaires sent to the eligible participants by postal mail. The questionnaires covered the different components of the ICF's bio-psycho-social model, namely health conditions, body functions, participation, environmental and personal factors. Bivariate correlations and multivariate linear regression analyses with participation as the dependent variable have been conducted. RESULTS: In all, 102 persons with SCI answered the survey, response rate 25.9%. Self-esteem (r=0.61) and self-efficacy (r=0.54) correlated highly with participation and were the strongest correlates of participation. They were stronger correlates of participation than symptoms of anxiety, depressive symptoms, pain, health conditions, social support, coping styles or sense of coherence. Participation seemed to be independent of gender, age, level or completeness of injury. Self-efficacy and self-esteem explained together with time since discharge and years of education 48% of the variance in participation adjusting for health condition, depressive symptoms, pain interference and social support. CONCLUSION: Considering self-efficacy and self-esteem within the comprehensive framework of the ICF can contribute to a better understanding of functioning, disability and health in SCI, which in turn may facilitate the development of interventions to support the persons' adjustment and reintegration. | Male | Humans | Patient Participation | Questionnaires | OK |
|  |  |  |  |  |  | Gesundheitswesen (Bundesverband Der Ärzte Des Öffentlichen Gesundheitsdienstes (Germany)) | 2011-03-00 Mar 2011 | With rising costs in health care, especially in the area of pharmaceuticals, patients are expected to be more involved in managing their care. Concepts such as shared decision-making or self-management support in chronic illness have been suggested as a way to facilitate this process and to empower patients. It is unclear however, whether the self-responsible patient role outlined in these concepts aligns with the patient's needs. The aim of this paper is to examine this proposed self-responsible role with regard to the patient's medication management needs and to discuss whether self-responsibility and autonomy are suitable concepts when illness-related challenges become difficult or complex. Selected findings from a qualitative longitudinal study on medication management from the perspective of people with chronic conditions are presented. Results from interviews with 27 patients indicate that both the patient's view and the challenges people with chronic conditions face in everyday life remain a 'blind spot' in health care. Providing information to improve health literacy may not be enough; there is a need to systematically support patients in developing skills to adequately (self-)manage a chronic condition. | Humans | Patient Participation | Questionnaires | Attitude to Health | OK |
|  |  |  |  |  |  | Nurse Education Today | 2010-08-00 Aug 2010 | As part of a larger evaluation study, 20 members of staff in a Faculty of Health were interviewed about the impact of service user and carer involvement on learning and teaching. A qualitative approach was adopted and semi-structured interviews were used to explore current levels of involvement, barriers and solutions. The data generated was analysed using the principles of grounded theory. Findings suggest respondents recognised the requirement to involve service users and carers in their learning activities. Most wanted to develop this aspect of their educational provision but a number of barriers were described. Strategic and operational solutions were proposed to overcome these and respondents were positive about achieving meaningful involvement. | Humans | Patient Participation | Questionnaires | Cooperative Behavior | OK |
|  |  |  |  |  |  | Community Mental Health Journal | 2013-12-00 Dec 2013 | This study assessed differences between service users', family carers' and mental healthcare providers' perceptions on service user and family carer involvement in mental healthcare. We conducted questionnaires in care networks for persons with serious and persistent mental illness, among 111 service users, 73 family carers and 216 mental healthcare providers. Many aspects of service user and family carer involvement are achieved to satisfactory levels while other aspects are scarcely realized. Service users and mental healthcare providers perceive family carer involvement as less important and realized than service user involvement. Family carers hold more favorable views on the importance and realization of involvement than do mental healthcare providers. The implementation of stakeholders' involvement in healthcare is ongoing. Notwithstanding great efforts, service users' and family carers' involvement preferences are not yet fully considered. Developing procedures, training and monitoring cycles regarding involvement may enhance this situation. | Male | Humans | Patient Participation | Questionnaires | OK |
| M. | Watts | M. | Creaner | E. | McCann | Journal of Psychiatric and Mental Health Nursing | 2011-08-00 Aug 2011 | In recent years, there is an ever increasing call to involve people who use mental health services in the development, delivery and evaluation of education programmes. Within Ireland, there is very little evidence of the degree of service user involvement in the educational preparation of mental health practitioners. This paper presents the findings on service user involvement in the education and training of professionals working in mental health services in Ireland. Findings from this study indicate that in the vast majority of courses curricula are planned and delivered without consultation or input from service users. Currently the scope of service user involvement is on teaching, with little involvement in curriculum development, student assessment and student selection. However, there is evidence that this is changing, with many respondents indicating an eagerness to move this agenda forward. | Humans | Patient Participation | Questionnaires | Adult | OK |
| Brenda | Rush |  |  |  |  | Nurse Education Today | 2012-07-00 Jul 2012 | | Humans | Patient Participation | Educational Measurement | Great Britain | OK |
| Lillian Sofie | Eng |  |  |  |  | International Journal of Mental Health Systems | 2014-01-09 janvier 9, 2014 | Background: The involvement of persons with lived experiences of mental illness and service use is increasingly viewed as key to improving the relevance and utility of mental health research and service innovation. Guided by the principles of Community-Based Participatory Research we developed an online tool for assisted self-help in mental health. The resulting tool, PsyConnect, is ready for testing in two communities starting 2014. This case study reports from the design phase which entailed clarifying very basic questions: Who is the primary target group? What are the aims? What functions are priorities? Roles and responsibilities? What types of evidence can legitimize tool design decisions? Here we highlight the views of service users as a basis for discussing implications of user involvement for service design and research. Case description: PsyConnect has become a tool for those who expect to need assistance over long periods of time regardless of their specific condition(s). The aim is to support service users in gaining greater overview and control, legitimacy, and sense of continuity in relationships. It has a personalized “my control panel” which depicts status → process → goals. Functionality includes support for: mapping life domains; medication overview; crisis management; coping exercises; secure messaging; and social support. While the types of evidence that can legitimize design decisions are scattered and indirectly relevant, recent trends in recovery research will be used to guide further refinements. Discussion: PsyConnect has undoubtedly become something other than it would have been without careful attention to the views of service users. The tool invites a proactive approach that is likely to challenge treatment cultures that are reactive, disorder-focused and consultation-based. Service user representatives will need to play central roles in training peers and clinicians in order to increase the likelihood of tool usage in line with intentions. Similarly, their influence on tool design has implications for choice of methods for evaluation. Conclusions: Starting down the path of service user involvement in intervention design fosters commitment to follow through in the remaining implementation and research phases. While this can be time-consuming and less meriting for researchers, it is probably vital to increasing the likelihood of success of person-centered service innovations. (PsycINFO Database Record (c) 2014 APA, all rights reserved). (journal abstract) | Mental health | design process | service users | online tool | OK |
| Damon | Mitchell |  |  |  |  |  | 2014-00-00 2014 | Clinicians working within a forensic context have a front row seat to the unique characteristics, mannerisms, and thought processes of their clients. Simple observation of clients during clinical interactions reveals a vast array of useful information such as their physical characteristics (e.g., grooming, tattoos, physical disabilities, energy level), personal attributes (e.g., intellectual abilities, response style), cognitive orientation (e.g., degree of insight, interest in self-improvement, degree of dissimulation), and interpersonal skills (e.g., politeness, cooperativeness, respectfulness). The richness of this data is extraordinary valuable in the formulation of a clinical opinion. This may be particularly true within the context of conducting forensic evaluations regarding such issues as criminal responsibility, sentencing recommendations, custody and release decisions, and/or future criminal potential. These data can also be of considerable value in the context of a forensic treatment performance evaluation in which the objective is to determine the degree to which clients have benefited from therapeutic intervention. Unfortunately, surveys of clinicians and casual observation of the curricula of many standardized offender treatment programs indicate that this information is under-utilized in the evaluation of forensic client treatment performance. The purpose of the present chapter is to describe a multi-method approach of measuring treatment performance that uses dynamic client information obtained during the treatment process. In keeping with the orientation of this book, discussion of these procedures will be weighted more toward practical application than on theoretical/empirical considerations. (PsycINFO Database Record (c) 2014 APA, all rights reserved). (chapter) | Client Participation | Clients | clinicians | progress | OK |
| Karolina | Budych | Carsten | Schultz |  |  | Implementation science: IS | 2011-00-00 2011 | BACKGROUND: A rare disease is a pattern of symptoms that afflicts less than five in 10,000 patients. However, as about 6,000 different rare disease patterns exist, they still have significant epidemiological relevance. We focus on rare diseases that affect multiple organs and thus demand that multidisciplinary healthcare professionals (HCPs) work together. In this context, standardized healthcare processes and concepts are mainly lacking, and a deficit of knowledge induces uncertainty and ambiguity. As such, individualized solutions for each patient are needed. This necessitates an intensive level of innovative individual behavior and thus, adequate idea generation. The final implementation of new healthcare concepts requires the integration of the expertise of all healthcare team members, including that of the patients. Therefore, knowledge sharing between HCPs and shared decision making between HCPs and patients are important. The objective of this study is to assess the contribution of shared communication and decision-making processes in patient-centered healthcare teams to the generation of innovative concepts and consequently to improvements in patient satisfaction. METHODS: A theoretical framework covering interaction processes and explorative outcomes, and using patient satisfaction as a measure for operational performance, was developed based on healthcare management, innovation, and social science literature. This theoretical framework forms the basis for a three-phase, mixed-method study. Exploratory phase I will first involve collecting qualitative data to detect central interaction barriers within healthcare teams. The results are related back to theory, and testable hypotheses will be derived. Phase II then comprises the testing of hypotheses through a quantitative survey of patients and their HCPs in six different rare disease patterns. For each of the six diseases, the sample should comprise an average of 30 patients with six HCP per patient-centered healthcare team. Finally, in phase III, qualitative data will be generated via semi-structured telephone interviews with patients to gain a deeper understanding of the communication processes and initiatives that generate innovative solutions. DISCUSSION: The findings of this proposed study will help to elucidate the necessity of individualized innovative solutions for patients with rare diseases. Therefore, this study will pinpoint the primary interaction and communication processes in multidisciplinary teams, as well as the required interplay between exploratory outcomes and operational performance. Hence, this study will provide healthcare institutions and HCPs with results and information essential for elaborating and implementing individual care solutions through the establishment of appropriate interaction and communication structures and processes within patient-centered healthcare teams. | Decision Making | Humans | Patient Satisfaction | Questionnaires | OK |
|  |  |  |  |  |  | Gesundheitswesen (Bundesverband Der Ärzte Des Öffentlichen Gesundheitsdienstes (Germany)) | 2011-07-00 Jul 2011 | Shared decision making (SDM) has been investigated in numerous research projects. In 2001 the German Ministry of Health initiated a research program on patients as partners in medical decision making ("Der Patient als Partner im medizinischen Entscheidungsprozess"). In this context a large number of studies has been conducted on SDM. In this review the concept of SDM and the use of this term in scientific publications of German study groups is explored. A systematic literature review generated 147 publications on SDM by German researchers, which had been published between 2000 and 2009. The majority of publications identified in this study refer to clinical studies on patients with chronic diseases. Content analysis of the retrieved publications indicates that SDM as a term is used heterogeneously. There is only little research on the theoretical foundations and ethical implications of SDM. In addition to existent Anglo-Saxon definitions of SDM, new concepts of SDM such as involvement of relatives in the process of decision making were identified as part of this review of the German literature. There is only a limited number of empirical studies with a consistent approach towards a definition of SDM and the respective measurements. Against this background, the "SDM questionnaire", which had been developed as part of the German Ministry of Health's research programme, is an important contribution with respect to operationalising SDM and testing possible effects of SDM in clinical practice. | Decision Making | Humans | Physician-Patient Relations | Questionnaires | OK |
| Christopher | Holloman | Jacklyn | Olson | Catherine | Hechmer | Patient Education and Counseling | 2012-07-00 juillet 2012 | Objective: The purpose of this study was to examine the relationship between shared decision-making (SDM) and satisfaction with decision (SWD) within a larger survey of patient decision-making in healthcare consultations. Methods: A randomly selected age-proportionate national sample of adults (aged 21–70 years) stratified on race, ethnicity, and gender (N = 488) was recruited from a health research volunteer registry and completed an online survey with reference to a recent health consultation. Measures included the Shared Decision Making-9 questionnaire (SDM-Q-9), Satisfaction With Decision (SWD) scale, sociodemographic,health, and other standardized decision-making measures. Forward selection weighted multiple regression analysis was used to model correlates of SWD. Results: After controlling for sociodemographic variables, SDM-Q-9 total score was associated with SWD, adjusted R² = .368, p &lt; .001. Three of nine SDM-Q-9 items accounted for significant proportions of variance in SWD. Conclusion: SDM was positively associated with SWD and was strongest for three areas of SDM: patients being helped in a health care consultation with understanding information, with treatment preference elicitation, and with weighing options thoroughly. Practice implications: By identifying variables such as SDM that are associated with SWD, health care interventions can better target modifiable factors to enhance satisfaction and other outcomes. (PsycINFO Database Record (c) 2012 APA, all rights reserved). (journal abstract) | Shared decision making | Decision Making | United States | Cooperation | Doublon |
| Christopher | Holloman | Jacklyn | Olson | Catherine | Hechmer | Patient Education and Counseling | 2012-07-00 Jul 2012 | OBJECTIVE: The purpose of this study was to examine the relationship between shared decision-making (SDM) and satisfaction with decision (SWD) within a larger survey of patient decision-making in health care consultations. METHODS: A randomly selected age-proportionate national sample of adults (aged 21-70 years) stratified on race, ethnicity, and gender (N=488) was recruited from a health research volunteer registry and completed an online survey with reference to a recent health consultation. Measures included the shared decision making-9 questionnaire (SDM-Q-9), Satisfaction With Decision (SWD) scale, sociodemographic, health, and other standardized decision-making measures. Forward selection weighted multiple regression analysis was used to model correlates of SWD. RESULTS: After controlling for sociodemographic variables, SDM-Q-9 total score was associated with SWD, adjusted R(2)=.368, p<.001. Three of nine SDM-Q-9 items accounted for significant proportions of variance in SWD. CONCLUSION: SDM was positively associated with SWD and was strongest for three areas of SDM: patients being helped in a health care consultation with understanding information, with treatment preference elicitation, and with weighing options thoroughly. PRACTICE IMPLICATIONS: By identifying variables such as SDM that are associated with SWD, health care interventions can better target modifiable factors to enhance satisfaction and other outcomes. | Male | Decision Making | Humans | Patient Participation | OK |
| Sarah | Chang | Anna-Lena | Lopez | Richard | Brown | Patient Education and Counseling | 2010-02-00 Feb 2010 | OBJECTIVE: The current study aimed to evaluate three coding systems which have been used to assess shared decision making in oncology consultations (OPTION, Decision Support Analysis Tool (DSAT) and Decision Analysis System for Oncology (DAS-O)): (i) comparing their ability to identify competencies of shared decision making, and (ii) determining their ability to predict patient outcomes in a single data set. METHOD: Twenty oncologists from Australia and New Zealand participated in the IBCSG Trial 33-03. The consultations of 55 women with early stage breast cancer were audio-taped, transcribed and then coded using the OPTION, DAS-O and DSAT coding systems by three different raters. Women completed the questionnaires 2 weeks and 4 months after their consultation. RESULTS: DAS-O was strongly correlated with OPTION (r=0.73). DSAT was moderately correlated with DAS-O and OPTION (r<0.6). Decisional satisfaction and satisfaction with doctor SDM skills were significantly correlated with OPTION (r=0.39 and 0.42 respectively) and the latter variable was correlated with DAS-O (r=0.40). These relationships persisted in multiple linear regression analyses. CONCLUSIONS: OPTION may be the most efficient and sensitive coding system for research purposes; however, DSAT appeared to document behaviours reducing decisional conflict and both DSAT and DAS-O offer more detailed feedback to doctors. PRACTICE IMPLICATIONS: Optimal coding system will depend on research goals and training purposes. | Male | Decision Making | Decision Support Techniques | Humans | OK |
| Susan B. | Sorenson |  |  |  |  | Research on Social Work Practice | 2013-11-00 novembre 2013 | Objective: The goal of this study was to test the degree to which client clinical characteristics and environmental context and social workers’ practice values and experience influenced support for client’s autonomy and willingness to engage in shared decision making (SDM), and whether willingness to engage in SDM was mediated by support for autonomy. Method: A randomized factorial survey of social workers working with adults with severe mental illness was employed. Eighty-seven social workers responded yielding 435 vignettes. Results: Hypotheses were partially supported. Diagnosis, symptomology, threats of harm, treatment adherence, substance use, and social workers’ values and experience predicted support for autonomy and willingness to engage in SDM. Willingness to engage in SDM was modestly mediated by support for autonomy. Conclusion: Helping social workers avoid bias in decision making is critical to the goal of supporting clients’ autonomy, building their capacity, minimizing disempowerment, and promoting recovery. (PsycINFO Database Record (c) 2014 APA, all rights reserved). (journal abstract) | Decision Making | Social Workers | Mental Disorders | Professional Consultation | OK |
| Francesca | Dwamena | Katherine | Dontje | Rebecca C. | Henry | Patient Education and Counseling | 2011-11-00 Nov 2011 | OBJECTIVE: Develop a system of practice tools and procedures to prompt shared decision making in primary care. SDM-GRIP (Shared Decision Making Guidance Reminders in Practice) was developed for suspected stable coronary artery disease (CAD), prior to the percutaneous coronary intervention (PCI) decision. METHODS: Program evaluation of SDM-GRIP components: Grand Rounds, provider training (communication skills and clinical evidence), decision aid (DA), patient group visit, encounter decision guide (EDG), SDM provider visit. RESULTS: Participation-Physician training=73% (21/29); patient group visits=25% of patients with diagnosis of CAD contacted (43/168). SDM visits=16% (27/168). Among SDM visit pairs, 82% of responding providers reported using the EDG in SDM encounters. Patients valued the SDM-GRIP program, and wanted to discuss comparative effectiveness information with a cardiologist. SDM visits were routinely reimbursed. CONCLUSION: Program elements were well received and logistically feasible. However, recruitment to an extra educational group visit was low. Future implementation will move SDM-GRIP to the point of routine ordering of non-emergent stress tests to retain pre-decision timing of PCI and to improve coordination of care, with SDM tools available across primary care and cardiology. PRACTICE IMPLICATIONS: Guidance prompts and provider training appear feasible. Implementation at stress testing requires further investigation. | Decision Making | Decision Support Techniques | Humans | Patient Participation | OK |
| Michelle | Farnan | Tricia A. | Kelly | Eric R. | Manahan | Annals of Surgical Oncology | 2013-10-00 Oct 2013 | BACKGROUND: The purpose of this study was to assess national practices of surgeons who treat breast cancer in order to identify opportunities to improve patient education. METHODS: In June 2012, the membership of the American Society of Breast Surgeons (ASBrS) (n = 2,818) was surveyed via email questionnaire to evaluate their current practice of shared decision making and informed consent for breast cancer patients. RESULTS: A total of 737 members (26 %) responded, including 384 breast surgeons and 306 general surgeons, 13 midlevel providers, and 25 other specialists. It was found that 90 % of surgeons spent more than 30 min meeting with a new cancer patient, and of these, 30 % spent more than an hour. Surgeons who spent more than 1 h face-to-face with a new cancer patient reported higher levels of overall patient knowledge compared with those who spent less (mean = 3.80 vs. 3.64 of 5; p = 0.001). Also, 89 % of respondents reported using educational tools, of whom more than 90 % used written tools. In addition, 65 % of members stated an interest in a free online educational tool if available and indicated a preference for a flexible tool that could be used by the patient alone or with a nurse. CONCLUSIONS: While practice patterns may vary, our results reveal that one-third of surgeons spend at least 1 h in consultation with a new breast cancer patient. More time spent translated to a higher perceived patient understanding of their disease and treatment options. Although the majority of surgeons currently use written materials, there was clear support for a free online educational tool. | Decision Making | Humans | Physician-Patient Relations | Questionnaires | OK |
| Frank | Meiss | Jürgen | Bengel | Katrin | Reuter | Melanoma Research | 2014-02-00 Feb 2014 | Increasing importance is being conferred to the implementation of shared decision making (SDM) in clinical practice for medical, ethical, and sociological reasons. In Germany, SDM has recently been adopted as an explicit goal in the S3-melanoma treatment guideline. The aim of this study is to present data on how melanoma patients want to be involved in treatment decisions and second on the dynamic of these preferences for involvement. This was investigated in consecutively recruited melanoma patients (stages I-III) in two German Skin Cancer Centers as part of a longitudinal questionnaire study. The Control Preference Scale assessed patients' preferences at baseline (n=405) and was readministered 1 year later (n=314) to detect potential changes. In addition, the perceived realization of SDM in the adjuvant interferon-α treatment decision was investigated in a subgroup of patients (n=108) using the nine-item Shared Decision Making Questionnaire (SDM-Q-9). More than 80% of the patients want to play an active role (autonomous or collaborative) in treatment decisions and only 17% want to delegate their decision to the doctor. We found a significant preference shift within a year in 43% of the patients, predominantly toward more active involvement. The results of the SDM-Q-9 indicate a moderate degree of perceived participation, with differing perceived implementation of the individual the SDM process steps. With the majority of melanoma patients preferring an active role in treatment decisions and improvable implementation of the SDM process steps in clinical practice, our findings support the relevance of SDM in dermato-oncology. | Male | Decision Making | Decision Support Techniques | Humans | OK |
| Norbert | Donner-Banzhoff | Tanja | Krones |  |  | Patient Education and Counseling | 2011-02-00 Feb 2011 | OBJECTIVE: Although shared decision making (SDM) has become increasingly important in bioethical discussions and clinical practice, it is not clear in which treatment situations SDM is suitable. We address this question by investigating social norms on the appropriateness of SDM in different situations. METHODS: We conducted qualitative expert interviews with patients, general practitioners, and health administration and research professionals. RESULTS: SDM was considered to be most important in severe illness and chronic condition. Furthermore, SDM was indicated to be required if there is more than one therapeutic option, especially if it is not clear which option is best. Interviewees classified end-of-life decisions and decisions about prevention as those that primarily should be made by informed patients. On the other hand a paternalistic decision was considered most appropriate in emergency situations and when the patient does not want to participate in decision making. CONCLUSION: This study demonstrates that multiple situational factors and their interactions must be considered regarding the scope of SDM in medical consultation. PRACTICE IMPLICATIONS: Research addressing this question will help physicians adjust their consultation style and allow implementations of SDM and decision aids to be tailored more appropriately to complex treatment situations. | Male | Decision Making | Humans | Patient Participation | OK |
| Margaret | Charles | Martin H.N. | Tattersall |  |  | Health Expectations: An International Journal of Public Participation in Health Care & Health Policy | 2010-09-00 septembre 2010 | Introduction: Shared decision making (SDM) is now considered a desirable goal in health care, yet little is known about current practice in cancer care, and its impact on patient outcomes. This study aimed to develop an oncology-specific coding system for SDM, explore variations in SDM according to patient and disease characteristics, determine the relationship between SDM and patient satisfaction with the consultation, and explore the impact of SDM on patient anxiety. Methods: Sixty-three medical and radiation oncology consultations with patients with primary cancer involving consideration of adjuvant therapy after surgery were audio-taped, transcribed and coded. Intra and inter-rater reliability of the coding system was 95 and 90% respectively. Patients completed questionnaires before and after the consultation. Results: Construct validity of the SDM coding system was successfully conducted. Oncologists demonstrated on average under 11 of 18 SDM behaviours. Behaviours seeking patient preferences were particularly rare. SDM behaviours were more apparent in consultations involving female breast cancer patients. SDM behaviour scores in combination with patient involvement preference could predict achievement of patient involvement preference but not overall patient satisfaction. Although there was no overall relationship between patient anxiety and SDM scores, it did appear that physicians may change SDM behaviour according to patient factors including anxiety. Conclusion: Our findings reinforce the importance of the doctor in facilitating shared decision making in oncology consultations. (PsycINFO Database Record (c) 2012 APA, all rights reserved). (journal abstract) | Shared decision making | Decision Making | Patient Satisfaction | physicians | Doublon |
| Margaret | Charles | Martin H. N. | Tattersall |  |  | Health Expectations: An International Journal of Public Participation in Health Care and Health Policy | 2010-09-00 Sep 2010 | INTRODUCTION: Shared decision making (SDM) is now considered a desirable goal in health care, yet little is known about current practice in cancer care, and its impact on patient outcomes. This study aimed to develop an oncology-specific coding system for SDM, explore variations in SDM according to patient and disease characteristics, determine the relationship between SDM and patient satisfaction with the consultation, and explore the impact of SDM on patient anxiety. METHODS: Sixty-three medical and radiation oncology consultations with patients with primary cancer involving consideration of adjuvant therapy after surgery were audio-taped, transcribed and coded. Intra and inter-rater reliability of the coding system was 95 and 90% respectively. Patients completed questionnaires before and after the consultation. RESULTS: Construct validity of the SDM coding system was successfully conducted. Oncologists demonstrated on average under 11 of 18 SDM behaviours. Behaviours seeking patient preferences were particularly rare. SDM behaviours were more apparent in consultations involving female breast cancer patients. SDM behaviour scores in combination with patient involvement preference could predict achievement of patient involvement preference but not overall patient satisfaction. Although there was no overall relationship between patient anxiety and SDM scores, it did appear that physicians may change SDM behaviour according to patient factors including anxiety. CONCLUSION: Our findings reinforce the importance of the doctor in facilitating shared decision making in oncology consultations. | Male | Decision Making | Humans | Patient Participation | OK |
| Vanessa | Chan | Jiwon | Youm | Tianzan | Zhou | The Journal of Bone and Joint Surgery. American Volume | 2013-09-18 Sep 18, 2013 | BACKGROUND: Despite evidence that shared decision-making tools for treatment decisions improve decision quality and patient engagement, they are not commonly employed in orthopaedic practice. The purpose of this study was to evaluate the impact of decision and communication aids on patient knowledge, efficiency of decision making, treatment choice, and patient and surgeon experience in patients with osteoarthritis of the hip or knee. METHODS: One hundred and twenty-three patients who were considered medically appropriate for hip or knee replacement were randomized to either a shared decision-making intervention or usual care. Patients in the intervention group received a digital video disc and booklet describing the natural history and treatment alternatives for hip and knee osteoarthritis and developed a structured list of questions for their surgeon in consultation with a health coach. Patients in the control group received information about the surgeon's practice. Both groups reported their knowledge and stage in decision making and their treatment choice, satisfaction, and communication with their surgeon. Surgeons reported the appropriateness of patient questions and their satisfaction with the visit. The primary outcome measure tracked whether patients reached an informed decision during their first visit. Statistical analyses were performed to evaluate differences between groups. RESULTS: Significantly more patients in the intervention group (58%) reached an informed decision during the first visit compared with the control group (33%) (p = 0.005). The intervention group reported higher confidence in knowing what questions to ask their doctor (p = 0.0034). After the appointment, there was no significant difference between groups in the percentage of patients choosing surgery (p = 0.48). Surgeons rated the number and appropriateness of patient questions higher in the intervention group (p < 0.0001), reported higher satisfaction with the efficiency of the intervention group visits (p < 0.0001), and were more satisfied overall with the intervention group visits (p < 0.0001). CONCLUSIONS: Decision and communication aids used in orthopaedic practice had benefits for both patients and surgeons. These findings could be important in facilitating adoption of shared decision-making tools into routine orthopaedic practice. | Male | Decision Making | Humans | Patient Participation | Doublon |
| Vanessa | Chan | Jiwon | Youm | Tianzan | Zhou | The Journal of Bone and Joint Surgery. American Volume | 2013-09-18 Sep 18, 2013 | BACKGROUND: Despite evidence that shared decision-making tools for treatment decisions improve decision quality and patient engagement, they are not commonly employed in orthopaedic practice. The purpose of this study was to evaluate the impact of decision and communication aids on patient knowledge, efficiency of decision making, treatment choice, and patient and surgeon experience in patients with osteoarthritis of the hip or knee. METHODS: One hundred and twenty-three patients who were considered medically appropriate for hip or knee replacement were randomized to either a shared decision-making intervention or usual care. Patients in the intervention group received a digital video disc and booklet describing the natural history and treatment alternatives for hip and knee osteoarthritis and developed a structured list of questions for their surgeon in consultation with a health coach. Patients in the control group received information about the surgeon's practice. Both groups reported their knowledge and stage in decision making and their treatment choice, satisfaction, and communication with their surgeon. Surgeons reported the appropriateness of patient questions and their satisfaction with the visit. The primary outcome measure tracked whether patients reached an informed decision during their first visit. Statistical analyses were performed to evaluate differences between groups. RESULTS: Significantly more patients in the intervention group (58%) reached an informed decision during the first visit compared with the control group (33%) (p = 0.005). The intervention group reported higher confidence in knowing what questions to ask their doctor (p = 0.0034). After the appointment, there was no significant difference between groups in the percentage of patients choosing surgery (p = 0.48). Surgeons rated the number and appropriateness of patient questions higher in the intervention group (p < 0.0001), reported higher satisfaction with the efficiency of the intervention group visits (p < 0.0001), and were more satisfied overall with the intervention group visits (p < 0.0001). CONCLUSIONS: Decision and communication aids used in orthopaedic practice had benefits for both patients and surgeons. These findings could be important in facilitating adoption of shared decision-making tools into routine orthopaedic practice. | Male | Decision Making | Humans | Patient Participation | OK |
| Lilisbeth | Perestelo-Pérez | Pedro | Serrano-Aguilar | |  | Neuropsychiatric Disease and Treatment | 2013-07-31 juillet 31, 2013 | Background: To measure and compare the extent to which shared a decision making (SDM) process is implemented both in psychiatric outpatient clinical encounters and in the primary care setting from the patient’s perspective. Methods: A total of 1,477 patients recruited from the Canary Islands Health Service mental health and primary care departments were invited to complete the nine-item Shared Decision Making Questionnaire (SDM-Q-9) immediately after their consultation. MANCOVA, Student’s t-test, and Pearson correlations were used to assess the relationship and differences between SDM-Q-9 scores in patient samples. Results: No differences were found in SDM-Q-9 total scores between the two patient samples, but there were relevant differences when item by item analysis was applied; differences were observed according to the different steps of the SDM process. SDM is present to a very limited extent in the routine psychiatric setting compared to primary care. Patients’ age, education, type of appointment, and treatment decision all play a specific role in predicting SDM. Conclusion: The study provides evidence that SDM is a complex process that needs to be analyzed according to its different steps. SDM patterns were different in the primary care and psychiatric outpatient care settings and reflect quite a different perspective of the decision making process. (PsycINFO Database Record (c) 2013 APA, all rights reserved). (journal abstract) | Shared decision making | Decision Making | Questionnaires | Psychometrics | Doublon |
| Lilisbeth | Perestelo-Pérez | Pedro | Serrano-Aguilar | |  | Neuropsychiatric Disease and Treatment | 2013-00-00 2013 | BACKGROUND: To measure and compare the extent to which shared a decision making (SDM) process is implemented both in psychiatric outpatient clinical encounters and in the primary care setting from the patient's perspective. METHODS: A total of 1,477 patients recruited from the Canary Islands Health Service mental health and primary care departments were invited to complete the nine-item Shared Decision Making Questionnaire (SDM-Q-9) immediately after their consultation. MANCOVA, Student's t-test, and Pearson correlations were used to assess the relationship and differences between SDM-Q-9 scores in patient samples. RESULTS: No differences were found in SDM-Q-9 total scores between the two patient samples, but there were relevant differences when item by item analysis was applied; differences were observed according to the different steps of the SDM process. SDM is present to a very limited extent in the routine psychiatric setting compared to primary care. Patients' age, education, type of appointment, and treatment decision all play a specific role in predicting SDM. CONCLUSION: The study provides evidence that SDM is a complex process that needs to be analyzed according to its different steps. SDM patterns were different in the primary care and psychiatric outpatient care settings and reflect quite a different perspective of the decision making process. | | | | | OK |
| Sara | Poplau | Deborah H. | Boehm | Nilay D. | Shah | Journal of the American Board of Family Medicine: JABFM | 2014-04-00 2014 Mar-Apr | BACKGROUND: Shared decision making (SDM) is an interactive process between clinicians and patients in which both share information, deliberate together, and make clinical decisions. Clinics serving safety net patients face special challenges, including fewer resources and more challenging work environments. The use of SDM within safety net institutions has not been well studied. METHODS: We recruited a convenience sample of 15 safety net primary care clinicians (13 physicians, 2 nurse practitioners). Each answered a 9-item SDM questionnaire and participated in a semistructured interview. From the transcribed interviews and questionnaire data, we identified themes and suggestions for introducing SDM into a safety net environment. RESULTS: Clinicians reported only partially fulfilling the central components of SDM (sharing information, deliberating, and decision making). Most clinicians expressed interest in SDM by stating that they "selected a treatment option together" with patients (8 of 15 in strong or complete agreement), but only a minority (3 of 15) "thoroughly weighed the different treatment options" together with patients. Clinicians attributed this gap to many barriers, including time pressure, overwhelming visit content, patient preferences, and lack of available resources. All clinicians believed that lack of time made it difficult to practice SDM. CONCLUSIONS: To increase use of SDM in the safety net, efficient SDM interventions designed for this environment, team care, and patient engagement in SDM will need further development. Future studies should focus on adapting SDM to safety net settings and determine whether SDM can reduce health care disparities. | Decision Making | Humans | Patient Participation | Questionnaires | OK |
| Scott | Haldeman |  |  |  |  | Journal of Manipulative and Physiological Therapeutics | 2012-04-00 2012 Mar-Apr | OBJECTIVE: The purpose of this study was to propose questions that may be helpful to educate patients considering treatment approaches to manage low back pain (LBP) and to determine if the information currently presented in informed consent (IC) documents at chiropractic colleges is sufficient to help a patient considering chiropractic management of LBP make a fully informed decision. METHODS: Questions to inform decision making for a patient contemplating any intervention for LBP were developed by the authors based on their clinical and research experience. Answers to the questions were suggested based on findings from recent clinical practice guidelines and systematic reviews. Institutions that are members of the Association of Chiropractic Colleges (ACC) were surveyed and asked to provide a copy of the IC documents currently used in their outpatient educational clinics. The IC documents were analyzed to determine if they stated (or implied) information that may be helpful in addressing each of the proposed questions. RESULTS: The list of 20 questions included 4 questions on each of the following 5 topics: condition, proposed treatment, potential benefits, potential harms, and possible alternatives. A total of 21 ACC institutions were contacted, of which 20 responded. The number of questions that could potentially be answered with information provided in the IC documents ranged from 2 to 13, with a mean of 6.5, including a mean of 3.6 stated answers and 2.9 implied answers. CONCLUSIONS: Some information to help patients consider chiropractic management of LBP is currently included in the IC documents used in clinics of ACC institutions. However, many of the questions that could help achieve shared decision making are not included. Modifying IC documents may help patients understand the nature, benefits, harms, costs, and alternatives to LBP care. | Male | Decision Making | Humans | Questionnaires | OK |
|  |  |  |  |  |  | The Journal of the American Academy of Orthopaedic Surgeons | 2013-03-00 Mar 2013 | | Decision Making | Humans | needs assessment | Dentist-Patient Relations | OK |
| Hanneke C. J. M. | de Haes | Caro C. E. | Koning | Elisabeth D. | Geijsen | Patient Education and Counseling | 2011-12-00 Dec 2011 | OBJECTIVE: To assess clinicians' use of shared decision making (SDM) skills, enabling patient treatment evaluations (appraisals); and varieties of patient appraisals and clinicians' preceding and following utterances. METHODS: Two coders rated videotaped initial visits of 25 early-stage prostate cancer patients to their radiation oncologist. SDM skills were assessed using the Decision Analysis System for Oncology (DAS-O); appraisals and clinicians' utterances were labeled using qualitative methodology. RESULTS: Clinicians offered a treatment choice to 10 patients. They informed 15/25 about pros and 20/25 about cons of options. Patients expressed 67 appraisals (median/visit=2; range, 0-12). Half of appraisals were favorable and one-fourth was unfavorable toward treatment options. One-fifth referred to explicit tradeoffs. One-third of appraisals followed clinician requests; 58% followed clinician information. Clinicians approved almost half of appraisals. They contested, ignored or highlighted a minority. CONCLUSION: Clinicians infrequently offered patients a choice or explored appraisals. Most appraisals supported rather than challenged treatment options. Clinicians most often legitimized appraisals, thereby helping patients to feel good about the decision. Exploring appraisals may help patients in forming more stable preferences, thus benefiting patients in the long run. PRACTICE IMPLICATION: Clinicians should request patient appraisals and ascertain whether these seem well-informed before making treatment recommendations. | Male | Decision Making | Decision Support Techniques | Humans | OK |
| U. | Schwager |  |  |  |  | Praxis | 2010-12-01 Dec 1, 2010 | BACKGROUND: Although there is much talk about shared decision making and individualized goal setting, there is a lack of knowledge and knowhow in their realization in daily clinical practice. There is a lack in tools for easy applicable tools to ameliorate person-centred individualized goal setting processes. METHOD: In three selected psychiatric inpatients the semistructured, theory driven use of PRISM (Pictorial Representation of Illness and Self Measure) in patients with complex psychiatric problems is presented and discussed. RESULTS: PRISM sustains a person-centred individualized process of goal setting and treatment and reinforces the active participation of patients. The process of visualisation and synchronous documentation is validated positively by patients and clinicians. The visual goal setting requires 30 to 45 minutes. CONCLUSIONS: In patients with complex psychiatric illness PRISM was used successfully to ameliorate individual goal setting. Specific effects of PRISM-visualisation are actually evaluated in a randomized controlled trial. | Male | Decision Making | Humans | Patient Participation | OK |
| Victor M. | Montori | Bjorg | Thorsteinsdottir | Jason S. | Egginton | BMJ open | 2014-00-00 2014 | OBJECTIVE: To assess US physicians' attitudes towards using shared decision-making (SDM) to achieve cost containment. DESIGN: Cross-sectional mailed survey. SETTING: US medical practice. PARTICIPANTS: 3897 physicians were randomly selected from the AMA Physician Masterfile. Of these, 2556 completed the survey. MAIN OUTCOME MEASURES: Level of enthusiasm for "Promoting better conversations with patients as a means of lowering healthcare costs"; degree of agreement with "Decision support tools that show costs would be helpful in my practice" and agreement with "should promoting SDM be legislated to control overall healthcare costs". RESULTS: Of 2556 respondents (response rate (RR) 65%), two-thirds (67%) were 'very enthusiastic' about promoting SDM as a means of reducing healthcare costs. Most (70%) agreed decision support tools that show costs would be helpful in their practice, but only 24% agreed with legislating SDM to control costs. Compared with physicians with billing-only compensation, respondents with salary compensation were more likely to strongly agree that decision support tools showing costs would be helpful (OR 1.4; 95% CI 1.1 to 1.7). Primary care physicians (vs surgeons, OR 1.4; 95% CI 1.0 to 1.6) expressed more enthusiasm for SDM being legislated as a means to address healthcare costs. CONCLUSIONS: Most US physicians express enthusiasm about using SDM to help contain costs. They believe decision support tools that show costs would be useful. Few agree that SDM should be legislated as a means to control healthcare costs. | | | | | OK |
| Peter A. | Fasching | Christian R. | Löhberg | Mayada R. | Bani | Breast Cancer Research and Treatment | 2012-10-00 Oct 2012 | Several factors can influence individual perceptions of the expected benefit of recommended adjuvant treatment for breast cancer. This study investigated differences between patients and physicians with regard to the required efficacy of treatment and the factors influencing patients' and physicians' willingness to accept different therapeutic options. A total of 9,000 questionnaires were distributed to patients with breast cancer, and 6,938 questionnaires were distributed to physicians treating breast cancer patients. The patients were asked for personal information and about their medical history and experiences during treatment. The physicians were asked about personal information and their specialty and work environment. The treatment efficacy required by the two groups was assessed using six virtual cases of breast cancer and the treatment regimens proposed, with specific benefits and side effects. A total of 2,155 patients and 527 physicians responded to the questionnaire (return rates of 23.9 and 7.6 %). Significantly different ratings between patients and physicians with regard to the expected benefit of certain treatment options were observed. The differences were noted not only for chemotherapy but also for antihormonal and antibody treatments. Whereas physicians had a quite realistic view of the expected treatment benefits, the patients' expectations were varied. Approximately one-fifth of the patients were willing to accept treatment regimens even with marginal anticipated benefits, whereas one-third required unrealistic treatment benefits. Several influencing factors that were significantly associated with the quality rating of treatment regimens in the groups of breast cancer patients and physicians were also identified. In contrast to physicians, many breast cancer patients required treatment benefits beyond what was realistically possible, although a large group of patients were also satisfied with minimal benefits. Individual factors were also identified in both groups that significantly influence thresholds for accepting adjuvant treatment, independently of risk estimates and therapy guidelines. | Male | Decision Making | Humans | Patient Satisfaction | OK |
| Christian R. | Loehberg | Peter A. | Fasching | Michael G. | Schrauder | Breast Cancer Research and Treatment | 2013-06-00 Jun 2013 | Treatment decisions in oncology are based on a balance between the efficacy of therapy and its side effects. Patients with metastases and patients with a limited prognosis are a particular challenge, since communication about the disease situation and the expected therapeutic benefit is difficult not only for patients, but also for physicians. The aim of this study was therefore to compare the benefits expected of therapy by patients and physicians. Questionnaires were sent to 9,000 breast cancer patients and to 6,938 physicians. The questionnaires described 10 cases of breast cancer in the metastatic setting. The patients and physicians were asked to state the treatment benefit they would require to decide for the therapy options chemotherapy, endocrine therapy, antibody therapy, radiotherapy, and bisphosphonates. Additionally, the participants provided data on patient and physician characteristics. Expected treatment benefits were compared between patients and physicians, and influencing factors that modified the expected benefit were identified. Patients expected much greater benefits from the therapies offered than the physicians. For all treatment modalities, about 50 % or more of patients expected more than a 12-month increase in overall survival from all therapies. Among the doctors, this proportion ranged from 7 to 30 %. Among patients, previous experience of side effects and having young children in the family were the strongest influencing factors. Among the doctors, age and level of education had a strong influence on the expected prognostic improvement to indicate a therapy option. As expectations of treatment differ greatly between patients and doctors, a structured approach to solving this conflict is required. There appear to be some indicators that might help address the problem, such as the physicians' level of training and experience and the patients' specific social circumstances. | Male | Decision Making | Humans | Questionnaires | OK |
| Peter | Bailey | Stefan | Priebe |  |  | Patient Education and Counseling | 2013-06-00 Jun 2013 | OBJECTIVE: Research on patient involvement in decision-making in psychiatry has focused on first encounters. This study investigated what decisions are made, level of patient involvement and factors influencing patient involvement in ongoing outpatient visits. METHODS: 72 visits conducted by 20 psychiatrists were video recorded. Patients had a diagnosis of depression or schizophrenia. RESULTS: On average, there was one medication related and one other decision per visit. Some psychiatrists involved patients more in decisions, as did female psychiatrists. Involvement was lower when patients had more negative symptoms. CONCLUSION: Involvement in decision-making appears to be influenced by the individual psychiatrist and specific symptoms but not visit length. PRACTICE IMPLICATIONS: It is noteworthy that patient involvement is not influenced by length of the visit given that this would be a barrier in busy clinical practice. The next step would be to identify the communication patterns of psychiatrists who involve patients more in decision-making. | Male | Decision Making | Humans | Patient Participation | OK |
| Evaline A. | Alessandrini | David A. | Asch | James P. | Guevara | Pediatrics | 2010-08-00 Aug 2010 | OBJECTIVES: To identify patterns of shared decision-making (SDM) among a nationally representative sample of US children with attention-deficit/hyperactivity disorder (ADHD) or asthma and determine if demographics, health status, or access to care are associated with SDM. PATIENTS AND METHODS: We performed a cross-sectional study of the 2002-2006 Medical Expenditure Panel Survey, which represents 2 million children with ADHD and 4 million children with asthma. The outcome, high SDM, was defined by using latent class models based on 7 Medical Expenditure Panel Survey items addressing aspects of SDM. We entered factors potentially associated with SDM into logistic regression models with high SDM as the outcome. Marginal standardization then described the standardized proportion of children's households with high SDM for each factor. RESULTS: For both ADHD and asthma, 65% of children's households had high SDM. Those who reported poor general health for their children were 13% less likely to have high SDM for ADHD (64 vs 77%) and 8% less likely for asthma (62 vs 70%) when adjusting for other factors. Results for behavioral impairment were similar. Respondent demographic characteristics were not associated with SDM. Those with difficulty contacting their clinician by telephone were 26% (ADHD: 55 vs 81%) and 29% (asthma: 48 vs 77%) less likely to have high SDM than those without difficulty. CONCLUSIONS: These findings indicate that households of children who report greater impairment or difficulty contacting their clinician by telephone are less likely to fully participate in SDM. Future research should examine how strategies to foster ongoing communication between families and clinicians affect SDM. | Male | Humans | Questionnaires | United States | OK |
| So Hyun | Chun | Hyun-Ki | Lee | Young-Jin | Ko | Menopause (New York, N.Y.) | 2014-07-00 Jul 2014 | OBJECTIVE: This study aims to assess the current status of shared decision-making on instituting postmenopausal hormone therapy (HT). METHODS: Two cross-sectional nationwide surveys of postmenopausal women and primary care physicians in the Republic of Korea were conducted in 2012 via face-to-face interviews. A total of 685 women (aged 50-69 y) who with natural menopause and 250 primary care physicians were included. RESULTS: Only 56.8% of primary care physicians reported that they explain the benefits and risks of HT and leave the decision to postmenopausal women. The others usually recommended using or not using HT. Of those postmenopausal women who had discussed such therapy with physicians (147 of 685; 21.5%), not all were aware of breast cancer or cardiovascular risks (only 65.3% and 38.8% were informed, respectively). Although most physicians perceived HT as beneficial for menopausal symptom control (99.6%) and acknowledged the related risk of breast cancer (84.8%), nearly half had the impression that HT was preventive of cardiovascular diseases. The interviewed women were less informed of the benefits and risks of HT than were the physician respondents. The awareness levels of the treated and untreated women did not differ. CONCLUSIONS: Participation of postmenopausal women in deciding whether to use HT is not prevalent. Physician-woman information transfer is suboptimal, and treatment decisions often are not based on the best available evidence. The current status of shared decision-making in this setting is clearly in need of improvement. | | | | | OK |
| Gerdien H. | de Weert-van Oene | Tom | Sensky | Cees P. F. | van der Staak | Substance Use & Misuse | 2011-00-00 2011 | This study examines the effect of a shared decision-making intervention (SDMI) on patients' and clinicians' self-perceived interpersonal behavior. Clinicians (n = 34) in three addiction treatment centers in the Netherlands were randomly assigned to SDMI or treatment decision-making as usual. Patients receiving inpatient treatment in 2005-2006 were included (n = 212). Baseline characteristics were measured by the European Addiction Severity Index (EuropASI) and the Composite International Diagnostic Interview-Substance Abuse Module (CIDI-SAM). Treatment goals were assessed using the Goals of Treatment Questionnaire (GoT-Q) plus a Q-sort ranking procedure. Interpersonal behavior was measured by Interpersonal Checklist-Revised (ICL-R) at baseline, end of treatment, and 3-month follow-up. Repeated measures analyses of variance and multiple hierarchical linear regression analysis were used. The key finding of this study was that SDMI is associated with an increase of patient autonomy (independent behavior) and control behavior. The study limitations have been noted. | Decision Making | Humans | Patient Participation | Patient Satisfaction | OK |
| A. Sonia | Buist | Sarah B. | Knowles | Philip W. | Lavori | American Journal of Respiratory and Critical Care Medicine | 2010-03-15 Mar 15, 2010 | RATIONALE: Poor adherence to asthma controller medications results in poor treatment outcomes. OBJECTIVES: To compare controller medication adherence and clinical outcomes in 612 adults with poorly controlled asthma randomized to one of two different treatment decision-making models or to usual care. METHODS: In shared decision making (SDM), nonphysician clinicians and patients negotiated a treatment regimen that accommodated patient goals and preferences. In clinician decision making, treatment was prescribed without specifically eliciting patient goals/preferences. The otherwise identical intervention protocols both provided asthma education and involved two in-person and three brief phone encounters. MEASUREMENTS AND MAIN RESULTS: Refill adherence was measured using continuous medication acquisition (CMA) indices-the total days' supply acquired per year divided by 365 days. Cumulative controller medication dose was measured in beclomethasone canister equivalents. In follow-up Year 1, compared with usual care, SDM resulted in: significantly better controller adherence (CMA, 0.67 vs. 0.46; P < 0.0001) and long-acting beta-agonist adherence (CMA, 0.51 vs. 0.40; P = 0.0225); higher cumulative controller medication dose (canister equivalent, 10.9 vs. 5.2; P < 0.0001); significantly better clinical outcomes (asthma-related quality of life, health care use, rescue medication use, asthma control, and lung function). In Year 2, compared with usual care, SDM resulted in significantly lower rescue medication use, the sole clinical outcome available for that year. Compared with clinician decision making, SDM resulted in: significantly better controller adherence (CMA, 0.67 vs. 0.59; P = 0.03) and long-acting beta-agonist adherence (CMA, 0.51 vs. 0.41; P = 0.0143); higher cumulative controller dose (CMA, 10.9 vs. 9.1; P = 0.005); and quantitatively, but not significantly, better outcomes on all clinical measures. CONCLUSIONS: Negotiating patients' treatment decisions significantly improves adherence to asthma pharmacotherapy and clinical outcomes. Clinical trials registered with www.clinicaltrials.gov (NCT00217945 and NCT00149526). | Male | Decision Making | Humans | Patient Participation | OK |
| Ilona | Juraskova | Karin | Ribi | Daniela | Gerber | Health Expectations: An International Journal of Public Participation in Health Care & Health Policy | 2011-03-00 mars 2011 | Background: Shared Decision Making (SDM) is widely accepted as the preferred method for reaching treatment decisions in the oncology setting including those about clinical trial participation: however, there is some disagreement between researchers over the components of SDM. Specific standardized coding systems are needed to help overcome this difficulty. Objective: The first objective was to describe the development of an oncology specific SDM coding system, the DAS-O. The second objective was to provide reliability and validity data supporting the DAS-O. Setting and participants: Consultation data were available from tertiary cancer center out patient oncology clinics in: Australia, New Zealand (ANZ), Switzerland, Germany and Austria (SGA). Patients were women with a confirmed diagnosis of early stage breast cancer. Reliability data were from 18 randomly selected coded transcripts drawn from ANZ and SGA. Concurrent validity data are from 55 (ANZ) consultations. Measurement: Inter and Intra rater reliability data was evaluated using Kappa correlation statistics and correlation coefficients. Correlation coefficients were used to assess concurrent validity between the DAS-O and two other SDM coding systems, OPTION and DSAT. Results: Inter and Intra rater reliability for the system were high with average Kappas of 0.58 and 0.65 respectively. Correlation coefficients between DAS-O and OPTION was 0.73 and &gt; 0.5 for DSAT. (PsycINFO Database Record (c) 2012 APA, all rights reserved). (journal abstract) | Shared decision making | Decision Making | Psychometrics | Treatment | OK |
|  |  |  |  |  |  | Healthcare Informatics: The Business Magazine for Information and Communication Systems | 2013-09-00 Sep 2013 | | Humans | Patient Participation | Patient Satisfaction | Electronic Health Records | OK |
|  |  |  |  |  |  | Zeitschrift Für Evidenz, Fortbildung Und Qualität Im Gesundheitswesen | 2012-00-00 2012 | It is argued that attempts at measuring and modeling shared decision making (SDM) could benefit from trying harder to answer basic theoretical questions on the assumed nature of SDM first. (As supplied by publisher). | Decision Making | Humans | Physician-Patient Relations | Questionnaires | OK |
|  |  |  |  |  |  | International Journal of Obstetric Anesthesia | 2014-02-00 Feb 2014 | | Male | Humans | Patient Participation | Female | OK |
| Pervin | Anklesaria | Philip J. | Mease |  | 13G01 Study Team | The Journal of Rheumatology | 2010-05-00 May 2010 | OBJECTIVE: To assess the validity, responsiveness, and reliability of single-joint outcome measures for determining target joint (TJ) response in patients with inflammatory arthritis. METHODS: Patient-reported outcomes (PRO), consisting of responses to single questions about TJ global status on a 100-mm visual analog scale (VAS; TJ global score), function on a 100-mm VAS (TJ function score), and pain on a 5-point Likert scale (TJ pain score) were piloted in 66 inflammatory arthritis subjects in a phase 1/2 clinical study of an intraarticular gene transfer agent and compared to physical examination measures (TJ swelling, TJ tenderness) and validated function questionnaires (Disabilities of the Arm, Shoulder and Hand scale, Rheumatoid Arthritis Outcome Score, and the Health Assessment Questionnaire). Construct validity was assessed by evaluating the correlation between the single-joint outcome measures and validated function questionnaires using Spearman's rank correlation. Responsiveness or sensitivity to change was assessed through calculating effect size and standardized response means (SRM). Reliability of physical examination measures was assessed by determining interobserver agreement. RESULTS: The single-joint PRO were highly correlated with each other and correlated well with validated functional measures. The TJ global score exhibited modest effect size and modest SRM that correlated well with the patient's assessment of response on a 100-mm VAS. Physical examination measures exhibited high interrater reliability, but correlated less well with validated functional measures and the patient's assessment of response. CONCLUSION: Single-joint PRO, particularly the TJ global score, are simple to administer and demonstrate construct validity and responsiveness in patients with inflammatory arthritis. (ClinicalTrials.gov identifier NCT00126724). | Male | Humans | Patient Participation | Questionnaires | OK |
| Phillip J. | Newton | Peter J. S. | Cripps | Peter S. | MacDonald | Collegian (Royal College of Nursing, Australia) | 2014-00-00 2014 | BACKGROUND: The World Wide Web has changed the way in which people communicate and consume information. More importantly, this innovation has increased the speed and spread of information. There has been recent increase in the percentage of cardiovascular professionals, including journals and associations using Twitter to engage with others and exchange ideas. Evaluating the reach and impact in scientific meetings is important in promoting the use of social media. OBJECTIVE: This study evaluated Twitter use during the recent 61st Annual Scientific Meeting at the Cardiac Society of Australia and New Zealand. METHODS: During the Cardiac Society of Australia and New Zealand 2013 61st Annual Scientific Meeting Symplur was used to curate conversations that were publicly posted with the hashtag #CSANZ2013. The hashtag was monitored with analysis focused on the influencers, latest tweets, tweet statistics, activity comparisons, and tweet activity during the conference. Additionally, Radian6 social media listening software was used to collect data. A summary is provided. RESULTS: There were 669 total tweets sent from 107 unique Twitter accounts during 8th August 9 a.m. to 11th August 1 p.m. This averaged nine tweets per hour and six tweets per participant. This assisted in the sharing of ideas and disseminating the findings and conclusions from presenters at the conference with a total 1,432,573 potential impressions in Twitter users tweet streams. CONCLUSION: This analysis of Twitter conversations during a recent scientific meeting highlights the significance and place of social media within research dissemination and collaboration. Researchers and clinicians should consider using this technology to enhance timely communication of findings. The potential to engage with consumers and enhance shared decision-making should be explored further. | Humans | Internet | communication | Australia | OK |
|  |  |  |  |  |  | Behavioural and Cognitive Psychotherapy | 2011-03-00 Mar 2011 | BACKGROUND: Therapeutic alliance has been found to be a significant predictor of outcome in psychotherapy yet what constitutes therapeutic alliance remains unclear. Examining the common constructs of therapeutic alliance, it is possible that there may be a conceptual overlap between active components of therapeutic alliance and socialization to the treatment model. AIM: To investigate the relationship between socialization to the model and therapeutic alliance. METHOD: Participants (N = 43) were taken from the active treatment arm in a RCT for the treatment of chronic fatigue syndrome (CFS/ME). Therapeutic alliance was measured using a 5-item questionnaire (brief CALPAS) and socialization to the model was extracted from therapy tapes using a novel coding system. RESULTS: Key findings were that when patients and therapists agreed about goals of treatment, there were higher levels of concordance, less evidence of applying principles incongruent to the model, and less resistance during the treatment sessions. CONCLUSIONS: The outcome of this preliminary study contributes to the potential understanding of active components in the therapeutic alliance, and supports further research to achieve a more detailed picture of "non-specific" factors in therapy, including the active process of socialization in therapeutic alliance. | Humans | Patient Participation | Patient Satisfaction | Questionnaires | OK |
| Hubert | Robitaille | Moira | Stewart | Dominick | Frosch | Journal of Clinical Epidemiology | 2012-12-00 Dec 2012 | OBJECTIVE: To assess the psychometric properties of dyadic measures for shared decision making (SDM) research. STUDY DESIGN AND SETTING: We conducted an observational cross-sectional study in 17 primary care clinics with physician-patient dyads. We used seven subscales to measure six elements of SDM: (1) defining the problem, presenting options, and discussing pros and cons; (2) clarifying the patient's values and preferences; (3) discussing the patient's self-efficacy; (4) drawing on the doctor's knowledge; (5) verifying the patient's understanding; and (6) assessing the patient's uncertainty. We assessed the reliability and invariance of the factorial structure and considered a measure to be dyadic if the factorial structure of the patient version was similar to that of the physician version and if there was equality of loading (no significant chi-square). RESULTS: We analyzed data for 264 physicians and 269 patients. All measures except one showed adequate reliability (Cronbach alpha, 0.70-0.93) and factorial validity (root mean square error of approximation, 0.000-0.06). However, we found only four measures to be dyadic (P>0.05): the values clarification subscale, perceived behavioral subscale, information-verifying subscale, and uncertainty subscale. CONCLUSION: The subscales for values clarification, perceived behavioral control, information verifying, and uncertainty are appropriate dyadic measures for SDM research and can be used to derive dyadic indices. | Male | Decision Making | Humans | Patient Participation | OK |
| Pieter U. | Dijkstra | Jan H. B. | Geertzen | Rienk | Dekker | Prosthetics and Orthotics International | 2013-12-00 Dec 2013 | OBJECTIVE: To analyze sports participation of Dutch lower limb amputees and factors influencing sports participation. STUDY DESIGN: A cross-sectional survey was performed. Dutch lower limb amputees (N = 2039) were invited to participate in a postal survey addressing personal and amputation characteristics, physical limitations, sports participation, skin problems, and prosthesis use. Only data concerning personal and amputation characteristics together with the data concerning sports participation were used for this study. RESULTS: Of the 816 questionnaires received, 780 were suitable for statistical analysis. The mean age of the participants was 59.6 years (SD 14.8), 62% were men and 27% of the amputations was due to vascular diseases or diabetes. Only 15% of all respondents participate in sports at least 5 hours a month. Smoking (odds ratio: 0.55), an age older than 60 (odds ratio: 0.97 per year), and a vascular cause of amputation (odds ratio: 0.42) were negatively associated with sports participation. CONCLUSION: Less than 15% of the Dutch lower limb amputees participate in sports at least 5 hours per month. Older age, smoking and a vascular cause of amputation have a negative influence on the sports participation of these individuals. | Male | Humans | Patient Participation | Questionnaires | OK |
| Kari | Bø |  |  |  |  | Journal of Pregnancy | 2013-00-00 2013 | BACKGROUND: The transtheoretical model (TTM) has been successful in promoting health behavioral change in the general population. However, there is a scant knowledge about physical activity in relation to the TTM during pregnancy. Hence, the aims of the present study were (1) to assess readiness to become or stay physically active according to the TTM and (2) to compare background and health variables across the TTM. METHODS: Healthy pregnant women (n = 467) were allocated to the study from Oslo University Hospital, Norway. The participants filled in a validated self-administered questionnaire, physical activity pregnancy questionnaire (PAPQ) in gestation, weeks 32-36. The questionnaire contained 53 questions with one particular question addressing the TTM and the five stages: (1) precontemplation stage, (2) contemplation stage, (3) preparation stage, (4) action stage, and (5) maintenance stage. RESULTS: More than half of the participants (53%) were involved in regular exercise (stages 4-5); however, only six specified that they had recently started an exercise program (stage 4). About 33% reported engaging in some physical activity, but not regularly (stage 3). The results showed that receiving advice from health professionals to exercise during pregnancy increased the likeliness of being in stages 4-5, while higher age, multiparity, pregravid overweight, unhealthy eating habits, pelvic girdle pain, and urinary incontinence were more prevalent with low readiness to change exercise habits (stages 1-3). CONCLUSION: According to the TTM, more than half of the participants reported to be physically active. Moreover, most of the participants classified as inactive showed a high motivational readiness or intention to increase their physical activity level. Hence, pregnancy may be a window of opportunity for the establishment of long-term physical activity habits. | Humans | Patient Participation | Questionnaires | Female | OK |
| Bonnie | Spring | Rosemary M. | Caron | Joav | Merrick |  | 2012-00-00 2012 | Evidence-based practice is a cornerstone of effective health promotion for patients and populations. However, few tools have been available to train the diverse professional groups that need to collaborate to address behavioral and psychosocial problems. The Evidence Based Behavioral Practice (EBBP) project, sponsored by the National Institutes of Health, provides web-based tools that were designed as a learning resource to harmonize and upgrade the approach to evidence-based practice among a variety of stakeholders. Interactive online learning modules provide experiential training in the evidence-based practice process for individuals and communities, critical appraisal of evidence, and shared decision-making with affected parties in a health decision. A recent module, entitled "Stakeholder Dialogue about Evidence-Based Practice" was developed as a stimulus to spark discussion and perspective-taking among academics, practitioners, and community members. The module features clips of interviews with a variety of stakeholders as well as didactic commentary. Community clinicians offer candid descriptions about how trust, communication, and time impose challenges to conducting research, while conveying more favorable views about quality improvement research. The stakeholder module affords a valuable tool to introduce academic and community partners to each other's points of view, as a way of paving the way toward successful collaboration for research to practice translation. (PsycINFO Database Record (c) 2013 APA, all rights reserved). (chapter) | Decision Making | Stakeholder | evidence based practice | stakeholder dialogue | OK |
| A. | Kotwal | S. | Sanghi | A. K. | Verma | Indian Journal of Leprosy | 2010-09-00 2010 Jul-Sep | No disease has been more closely associated with stigma than leprosy such that it has become a metaphor for stigma. Stigma has been difficult to measure and little research has been done on this issue. Stigma reduction has not been an important component of anti-leprosy program. The study was undertaken to measure the stigma associated with leprosy by using P scale which is used for assessing participation restriction of those affected by the disease. This comparative questionnaire based study was carried out in two sets of patients. Two groups of 30 patients each were studied. First group belonged to a Government run Leprosarium and group two from a tertiary care skin and leprosy centre. The study used the Participation (P) scale and data was collected by interviewing the patients. Participation restriction was defined as any score equal to and more than 13. Participation restriction was observed in 27 (90%) cases of group 1while participation restriction was present in only 7 (23.3%) subjects of group 2. It was observed that mean score of participation restriction in group 1 was quite high at 31.9 while it was only 8.3 for group 2. The participation restriction was directly related to the duration of disease and the grade of disability. Longer the duration of disease, greater was the likelihood of restriction. The participation restriction was found to be negatively correlated with the education. Recommendation about prevention of disability would require program about early diagnosis of nerve damage and subsequent action at the patient-family-community level and health care providers. | Time Factors | Humans | Patient Participation | Questionnaires | OK |
| Rajendran | Chandrasekharan | |  |  |  | International Journal of Health Care Quality Assurance | 2014-00-00 2014 | PURPOSE: The purpose of this paper is to provide strategic recommendations to Indian hospital administrators for improving service quality by analysing performance dimensions and the importance attached to them by patients and attendants. DESIGN/METHODOLOGY/APPROACH: Patient and attendant perceptions were collected using a questionnaire. FINDINGS: Patients and attendants have different perceptions. Different customers have different needs of which providers need to be aware to better serve their consumers. RESEARCH LIMITATIONS/IMPLICATIONS: The study captured only 408 patient and attendants' perceptions--a 32 per cent response rate. PRACTICAL IMPLICATIONS: Results enable hospital administrators to develop appropriate strategies to improve their structure and function by analysing their strengths and weaknesses regarding their tangible and intangible assets. ORIGINALITY/VALUE: The study included attendants, specifically in an Indian healthcare context. | Male | Humans | Patient Participation | Patient Satisfaction | OK |
| Nan | Cochran | Anna N. A. | Tosteson | W. | Blair Brooks | Patient Education and Counseling | 2010-02-00 Feb 2010 | OBJECTIVE: Decision aids (DAs) have been shown to facilitate shared decision making about cancer screening. However, little data exist on optimal strategies for dissemination. Our objective was to compare different decision aid distribution models. METHODS: Eligible patients received video decision aids for prostate cancer (PSA) or colon cancer screening (CRC) through 4 distribution methods. Outcome measures included DA loans (N), % of eligible patients receiving DA, and patient and provider satisfaction. RESULTS: Automatically mailing DAs to all age/gender appropriate patients led to near universal receipt by screening-eligible patients, but also led to ineligible patients receiving DAs. Three different elective (non-automatic) strategies led to low rates of receipt. Clinician satisfaction was higher when patients viewed the DA before the visit, and this model facilitated implementation of the screening choice. Regardless of timing or distribution method, patient satisfaction was high. CONCLUSIONS: An automatic DA distribution method is more effective than relying on individual initiative. Enabling patients to view the DA before the visit is preferred. PRACTICE IMPLICATIONS: Systematically offering DAs to all eligible patients before their appointments is the ideal strategy, but may be challenging to implement. | Male | Decision Making | Decision Support Techniques | Humans | OK |
| R. D. | Friele |  |  |  |  | Social Science & Medicine | 2014-08-00 août 2014 | This article deals with the questions what the benefits and limitations are of the instruments and strategies that patient organisations use to influence quality of care. The advocacy of patients' interests has become more important for patient organisations in recent years, which is partly due to Dutch health care policy reform. Thirty state funded quality improvement projects run by patient organisations between 2009 and 2012 have been analysed. The quality improvement instruments developed and used in these projects are concerned with: standardization and standard setting (What is good care?); consultation, comparison and checking (What is the state of the care given?); and negotiating and advising (How can quality of care be improved?). The choice for these instruments is partly based on patient organisations' strategies of scientization, valuing institutionalized methods and valuing good relationships. We see that the development and use of these quality improvement instruments do strengthen patient organisation and therefore have internal identity and organisational effects. However, the external effects patient organisations can have by using these instruments and strategies is limited or at least insecure by lack of economic capital after the development phase and lack of negotiating power. The external effects of these instruments and strategies depend largely on a patient organisation's network and the willingness, degree of openness and policy of other stakeholders to cooperate. Therefore, these forms of patient participation remain vulnerable. (PsycINFO Database Record (c) 2014 APA, all rights reserved). (journal abstract) | Health Care Policy | patient organizations | quality of care | organizational effects | Doublon |
| R. D. | Friele |  |  |  |  | Social Science & Medicine (1982) | 2014-08-00 Aug 2014 | This article deals with the questions what the benefits and limitations are of the instruments and strategies that patient organisations use to influence quality of care. The advocacy of patients' interests has become more important for patient organisations in recent years, which is partly due to Dutch health care policy reform. Thirty state funded quality improvement projects run by patient organisations between 2009 and 2012 have been analysed. The quality improvement instruments developed and used in these projects are concerned with: standardization and standard setting (What is good care?); consultation, comparison and checking (What is the state of the care given?); and negotiating and advising (How can quality of care be improved?). The choice for these instruments is partly based on patient organisations' strategies of scientization, valuing institutionalized methods and valuing good relationships. We see that the development and use of these quality improvement instruments do strengthen patient organisation and therefore have internal identity and organisational effects. However, the external effects patient organisations can have by using these instruments and strategies is limited or at least insecure by lack of economic capital after the development phase and lack of negotiating power. The external effects of these instruments and strategies depend largely on a patient organisation's network and the willingness, degree of openness and policy of other stakeholders to cooperate. Therefore, these forms of patient participation remain vulnerable. | | | | | OK |
| Dominic | Beer | Janet | Parrott | Tom | Craig | Trials | 2013-00-00 2013 | BACKGROUND: Forensic mental health services have largely ignored examining patients' views on the nature of the services offered to them. A structured communication approach (DIALOG) has been developed with the aim of placing the patient's perspective on their care at the heart of the discussions between patients and clinicians. The effectiveness of the structured communication approach in community mental health services has been demonstrated, but no trial has taken place in a secure psychiatric setting. This pilot study is evaluating a 6-month intervention combining DIALOG with principles of solution-focused therapy on quality of life in medium-secure settings. METHODS AND DESIGN: A cluster randomized controlled trial design is being employed to conduct a 36-month pilot study. Participants are recruited from six medium-secure inpatient services, with 48 patients in the intervention group and 48 in the control group. The intervention uses a structured communication approach. It comprises six meetings between patient and nurse held monthly over a 6-month period. During each meeting, patients rate their satisfaction with a range of life and treatment domains with responses displayed on a tablet. The rating is followed by a discussion of how to improve the current situation in those domains identified by the patient. Assessments take place prior to the intervention (baseline), at 6 months (postintervention) and at 12 months (follow-up). The primary outcome is the patient's self-reported quality of life. DISCUSSION: This study aims to (1) establish the feasibility of the trial design as the basis for determining the viability of a large full-scale trial, (2) determine the variability of the outcomes of interest (quality of life, levels of satisfaction, disturbance, ward climate and engagement with services), (3) estimate the costs of the intervention and (4) refine the intervention following the outcome of the study based upon the experiences of the nurses and patients. The intervention allows patients to have a greater say in how they are treated and targets care in areas that patients identify as important to them. It is intended to establish systems that support meaningful patient and caregiver involvement and participation. TRIAL REGISTRATION: Current Controlled Trials, ISRCTN34145189. | Time Factors | Humans | Patient Participation | Patient Satisfaction | OK |
| Thomas | Rotthoff | André | Karger | Regine | Schmelzer | Patient Education and Counseling | 2014-06-00 Jun 2014 | OBJECTIVE: To evaluate the summative assessment (OSCE) of a communication training programme for dealing with challenging doctor-patient encounters in the 4th study year. METHODS: Our OSCE consists of 4 stations (breaking bad news, guilt and shame, aggressive patients, shared decision making), using a 4-item global rating (GR) instrument. We calculated reliability coefficients for different levels, discriminability of single items and interrater reliability. Validity was estimated by gender differences and accordance between GR and a checklist. RESULTS: In a pooled sample of 456 students in 3 OSCEs over 3 terms, total reliability was α=0.64, reliability coefficients for single stations were >0.80, and discriminability in 3 of 4 stations was within the range of 0.4-0.7. Except for one station, interrater reliability was moderate to strong. Reliability on item level was poor and pointed to some problems with the use of the GR. CONCLUSION: The application of the GR on regular undergraduate medical education shows moderate reliability in need of improvement and some traits of validity. Ongoing development and evaluation is needed with particular regard to the training of the examiners. PRACTICE IMPLICATIONS: Our CoMeD-OSCE proved suitable for the summative assessment of communication skills in challenging doctor-patient encounters. | | | | | OK |
| Thomas | Rotthoff | André | Karger | Regine | Schmelzer | Patient Education and Counseling | 2014-06-00 juin 2014 | Objective: To evaluate the summative assessment (OSCE) of a communication training programme for dealing with challenging doctor–patient encounters in the 4th study year. Methods: Our OSCE consists of 4 stations (breaking bad news, guilt and shame, aggressive patients, shared decision making), using a 4-item global rating (GR) instrument. We calculated reliability coefficients for different levels, discriminability of single items and interrater reliability. Validity was estimated by gender differences and accordance between GR and a checklist. Results: In a pooled sample of 456 students in 3 OSCEs over 3 terms, total reliability was α = 0.64, reliability coefficients for single stations were &gt; 0.80, and discriminability in 3 of 4 stations was within the range of 0.4–0.7. Except for one station, interrater reliability was moderate to strong. Reliability on item level was poor and pointed to some problems with the use of the GR. Conclusion: The application of the GR on regular undergraduate medical education shows moderate reliability in need of improvement and some traits of validity. Ongoing development and evaluation is needed with particular regard to the training of the examiners. Practice implications: Our CoMeD-OSCE proved suitable for the summative assessment of communication skills in challenging doctor–patient encounters. (PsycINFO Database Record (c) 2014 APA, all rights reserved). (journal abstract) | Professional Competence | Psychometrics | test validity | test reliability | Doublon |
| Benjamin | Barankin | Robert | Bissonnette | Wayne | Gulliver | Journal of Cutaneous Medicine and Surgery | 2011-08-00 2011 Jul-Aug | BACKGROUND: Little is known about the interaction between dermatologists and their patients in facilitating treatment decisions for psoriasis. PURPOSE: Our objective was to determine dermatologists' perceptions of the needs of psoriasis patients in treatment decisions. METHODS: Dermatologists were invited to complete an 18-item online survey on the treatment of psoriasis, including questions on decision-making roles, factors they considered important to patients in treatment decisions, and patients' needs for decision support. RESULTS: Seventy dermatologists completed the survey (15% response rate). The highest rated factors in decision making were access to physicians for discussion (86%) and information about the risks and benefits (80%); the latter was more frequently reported by those ≥ 50 years (p  =  .021). Treatment-specific factors of greatest importance were side-effect profile (87%) and cost (80%). Potential hindrances were patient misconceptions about disease, inadequate patient education materials, patient indecision, and inadequate physician time. CONCLUSION: Although dermatologists consider accessibility to dermatologists and information on treatment risk and benefits to be important in treatment decision making, they report time with patients and educational materials to be inadequate. LIMITATIONS: The small sample size may limit the generalizability of our findings. | Time Factors | Decision Making | Humans | Patient Participation | OK |
| Phyllis N. | Butow | Stephen J. | Clarke | Margaret | McJannett | Journal of Clinical Oncology: Official Journal of the American Society of Clinical Oncology | 2011-05-20 May 20, 2011 | PURPOSE: Decision making in advanced cancer is increasingly complex. We developed a decision aid (DA) for patients with advanced colorectal cancer who are considering first-line chemotherapy and reviewing treatment options, prognostic information, and toxicities. We examined its impact on patient understanding, treatment decisions, decisional conflict, decision making, consultation satisfaction, anxiety, and quality of life by using a randomized trial design. PATIENTS AND METHODS: In all, 207 patients with colorectal cancer who were considering first-line chemotherapy for metastatic disease were randomly assigned to receive a standard medical oncology consultation or a consultation in which the DA (take-home booklet with audio recording, reviewed by an oncologist) was used. Participants completed questionnaires postconsultation, postdecision, and 1 month later. RESULTS: In this study, 100 patients were randomly assigned to the control arm, and 107 received the DA. Median age of the sample was 62 years, 58% were male, 89% had a performance status of 0 or 1, and 36% had received prior adjuvant chemotherapy. Patients receiving the DA demonstrated a greater increase in understanding of prognosis, options, and benefits, with higher overall understanding (P < .001). Decisional conflict, treatment decisions, and achievement of involvement preferences were similar between the groups. Anxiety was similar across groups and decreased over time. Most patients were confident in a decision during the first consultation; 74% chose chemotherapy, 7% supportive care alone, and 10% observation. CONCLUSION: This randomized trial of a decision aid in advanced cancer showed that its use in advanced colorectal cancer improved patient understanding of prognosis, treatment options, risks, and benefits without increasing anxiety. DAs can improve informed consent and can be tested through randomized trials even in the advanced cancer setting. | Male | Decision Making | Decision Support Techniques | Humans | OK |
| Arden | Morris | John J. | Graff | Ann | Hamilton | Annals of Surgical Oncology | 2013-01-00 Jan 2013 | BACKGROUND AND PURPOSE: While variation in breast cancer quality indicators has been studied, to date there have been no studies examining the degree of surgeon-level variation in patient-reported outcomes. The purpose of this study is to examine surgeon-level variation in patient appraisals of their breast cancer care experiences. METHODS: Survey responses and clinical data from breast cancer patients reported to Detroit and Los Angeles Surveillance, Epidemiology and End Results registries from 6/2005 to 2/2007 were merged with attending surgeon surveys (1,780 patients, 291 surgeons). Primary outcomes were patient reports of access to care, care coordination, and decision satisfaction. Random-effects models examined variation due to individual surgeons for these three outcomes. RESULTS: Mean values on each patient-reported outcome scale were high. The amount of variation attributable to individual surgeons in the unconditional models was low to modest: 5.4% for access to care, 3.3% for care coordination, and 7.5% for decision satisfaction. Few factors were independently associated with patient reports of better access to or coordination of care, but less-acculturated Latina patients had lower decision satisfaction. CONCLUSIONS: Patients reported generally positive experiences with their breast cancer treatment, though we found disparities in decision satisfaction. Individual surgeons did not substantively explain the variation in any of the patient-reported outcomes. | Male | Humans | Patient Participation | Patient Satisfaction | OK |
| Maughan | Guymon | Lenora M. | Olson |  |  | Nursing Research | 2013-08-00 2013 Jul-Aug | BACKGROUND: Rural older adults are not often targeted for surveys, and little is known about survey response rates in this population. Because overall numbers of rural older adults are small, it is important to consider techniques to improve their survey response rates. OBJECTIVES: The purpose of this study was to work with community partners to determine whether rural older adults participating in a rural county home-delivered meals program were more likely to respond to an injury risk survey hand-delivered via the postal service or hand-delivered by the home-delivered meals drivers. METHODS: All home-delivered meals clients older than 65 years old were randomized to one of two groups. One group (n = 70) received the following via the postal service: a prenotice letter, a survey packet containing a description of the survey, the survey, a stamped and addressed return envelope, and a reminder/thank-you card. Older adults in the other group (n = 69) were personally handed the survey packet by the home-delivered meals driver. RESULTS: The overall survey response rate was 43.9%. Older adults who were handed the survey packets by the home-delivered meals drivers were older and significantly more likely to return the survey (57%) compared with those who received survey materials in the mail (31%). Only 27% of respondents agreed to be contacted regarding participation in future face-to-face interviews. When taking into account response rates, postage costs alone were over five times higher for the postal-delivered surveys compared with the hand-delivered surveys. DISCUSSION: By working with community partners, we were able to determine that older adults in a rural community were more likely to respond to surveys personally handed to them by someone they knew. | Humans | Patient Participation | Questionnaires | Aged | OK |
| Lindsey | Mitrani | Simon | Baarbe | Brett | Yarusi | Injury | 2014-09-00 Sep 2014 | OBJECTIVE: Assess factors that influence both the patient and the physician in the setting of minor head injury in adults and the decision-making process around CT utilization. METHODS: This is a convenience sample survey study of adult minor head injury patients (GCS 15) and their physicians regarding factors influencing the decision to use CT to evaluate for intra-cranial haemorrhage. Once a head CT was ordered and before the results were known, both the patient and physician were given a one-page survey asking questions about their concern for injury and rationale for CT use. CT results and surveys were then recorded in a centralized database and analyzed. RESULTS: 584 subjects were enrolled over the 27-month study period. The rate of any intra-cranial haemorrhage was 3.3%. Both the physicians (6% pre-test estimate) and the patients (22% pre-test estimate) over-estimated risk for haemorrhage. Clinical decision rules were not met in 46% of cases where CT was used. Physicians listed an average of 5 factors from a list of 9 that influenced their decision to order CT. Patients listed an average of 1.7 factors influencing their decision to present to the Emergency Department for evaluation. Many patients felt cost (45%) and low risk stratification (34%) should weigh heavily in the decision to use CT. If asked to limit CT utilization, physicians were able to identify a group with less than 2% risk of injury. CONCLUSIONS: Patients with low risk of intra-cranial injury continue to be evaluated by CT. Physician decision-making around the use of CT to evaluate minor head injury is multi-factorial. Shared decision-making between the patient and the physician in a low risk minor head injury encounter shows promise as a method to reduce CT utilization in this low risk cohort. | | | | | OK |
| Suzanne | Morton | Christine | Bechtel | Rodger | Kessler | Health Affairs (Project Hope) | 2013-02-00 Feb 2013 | Directly involving patients and families in care improvement increasingly is viewed as an important component of patient-centered care. To assess the extent to which practices actually involve patients, we surveyed 112 patient-centered medical home practices in twenty-two states. Nearly all of these practices sought patient feedback. However, only 29 percent involved patients and families as advisers and sought feedback through surveys, and only 32 percent involved patients in a continuing role in quality improvement. Interviews showed that practices that highly value patient involvement overcame barriers to ongoing patient participation. We argue that a cultural shift is needed in how practices view patients as partners, not just in areas such as personal responsibility and self-management, but also in quality improvement and governance. Practices must gain more experience and see more examples of the benefits of engaging patients, and they may need more incentives and support for engaging them. | Humans | Patient Participation | Patient Satisfaction | Self Care | OK |
| Scott | Haldeman |  |  |  |  | The Spine Journal: Official Journal of the North American Spine Society | 2010-06-00 Jun 2010 | BACKGROUND CONTEXT: Low back pain (LBP) is a prevalent, costly, and challenging condition to manage. Clinicians must choose among numerous assessment and management options. Several recent clinical practice guidelines (CPGs) on LBP have attempted to inform these decisions by evaluating and summarizing the best available supporting evidence. The quality and consistency of recommendations from these CPGs are currently unknown. PURPOSE: To conduct a systematic review of recent CPGs and synthesize their recommendations on assessing and managing LBP for clinicians. STUDY DESIGN/SETTING: Systematic review. METHODS: Literature search using MEDLINE, National Guidelines Clearinghouse, National Institute for Clinical Excellence, Internet search engines, and references of known articles. Only CPGs related to both assessment and management of LBP written in English were eligible; CPGs that summarized evidence from before the year 2000 were excluded. Data related to methods and recommendations for assessment and management of LBP were abstracted independently by two reviewers. Methodological quality was assessed using the Appraisal of Guidelines Research and Evaluation (AGREE) instrument by two reviewers. RESULTS: The search uncovered 669 citations, of which 95 were potentially relevant and 10 were included in the review; 6 discussed acute LBP, 6 chronic LBP, and 6 LBP with neurologic involvement. Methods used to develop CPGs varied, but the overall methodological quality was high as defined by AGREE scores. Recommendations for assessment of LBP emphasized the importance of ruling out potentially serious spinal pathology, specific causes of LBP, and neurologic involvement, as well as identifying risk factors for chronicity and measuring the severity of symptoms and functional limitations, through the history, physical, and neurologic examination. Recommendations for management of acute LBP emphasized patient education, with short-term use of acetaminophen, nonsteroidal anti-inflammatory drugs, or spinal manipulation therapy. For chronic LBP, the addition of back exercises, behavioral therapy, and short-term opioid analgesics was suggested. Management of LBP with neurologic involvement was similar, with additional consideration given to magnetic resonance imaging or computed tomography to identify appropriate candidates willing to undergo epidural steroid injections or decompression surgery if more conservative approaches are not successful. CONCLUSIONS: Recommendations from several recent CPGs regarding the assessment and management of LBP were similar. Clinicians who care for patients with LBP should endeavor to adopt these recommendations to improve patient care. Future CPGs may wish to invite coauthors from targeted clinician user groups, increase patient participation, update their literature searches before publication, conduct their own quality assessment of studies, and consider cost-effectiveness and other aspects in their recommendations more explicitly. | Humans | Evidence-based medicine | Practice Guidelines as Topic | Low Back Pain | OK |
| B. | Rosen | M. | Burnstein | A. L. | Halverson | Diseases of the Colon and Rectum | 2014-01-00 Jan 2014 | BACKGROUND: Patients diagnosed with colorectal cancer often seek information on the Internet to help them make treatment decisions. OBJECTIVE: The aim of this study is to evaluate the quality of Web-based patient information regarding surgery for colorectal cancer. DESIGN: This study is a cross-sectional survey of patient-directed Web sites. SETTINGS: The search engine Google (Mountain View, CA) and the search terms "colorectal cancer surgery," "colon cancer surgery," and "rectal cancer surgery" were used to identify Web sites. MAIN OUTCOME MEASURES: To assess quality, we used the DISCERN instrument, a validated questionnaire developed to analyze written consumer health information on treatment options to aid consumers in evaluating the quality of health-related information on treatment choices for a specific health problem. An additional colorectal cancer-specific questionnaire was used to evaluate Web site content for colorectal cancer surgical treatment. Two independent assessors reviewed each Web site. RESULTS: Searches revealed a total of 91 distinct Web sites, of which 37 met inclusion criteria. Web site affiliation was as follows: 32% open-access general information, 24% hospital/health care organization, and 19% professional medical society. Twelve (32.4%) Web sites had clear aims, 10 (27.0%) had identifiable references to their sources of information, and 9 (24.3%) noted the date of published information. Ten sites (27.0%) provided some description of the surgical procedure, 8 (21.6%) discussed either the risks or the benefits of surgery, and 4 (10.8%) addressed quality-of-life issues. Nineteen (51.4%) Web sites discussed postoperative complications, and 7 (18.9%) discussed stoma-related maintenance/care. LIMITATIONS: The small sample size and interrater reliability bias are limitations of this study. CONCLUSIONS: The quality of online patient information regarding colorectal cancer treatment is highly variable, often incomplete, and does not adequately convey the information necessary for patients to make well-informed medical decisions regarding treatment for colorectal cancer. An opportunity exists for professional medical societies to create more comprehensive online patient information materials that may serve as a resource to physicians and their patients (see Video, Supplemental Digital Content 1, http://links.lww.com/DCR/A122). | Decision Making | Humans | Patient Participation | Questionnaires | OK |
| Denis | Martin | Derek | Jones | Amanda | Clarke | BMC geriatrics | 2014-00-00 2014 | BACKGROUND: The Engaging with older adults in the development of strategies for the self management of chronic pain (EOPIC) study aims to design and develop self management strategies to enable older adults to manage their own pain. Involving older adults in research into chronic pain management will better enable the identification and development of strategies that are more appropriate for their use, but how can perspectives really be utilised to the best possible outcomes? METHOD: Seven older adults were recruited through a local advertising campaign to take part. We also invited participants from the local pain services, individuals who had been involved in earlier phase of the EOPIC study and a previous ESRC funded project. The group undertook library training and research skills training to facilitate searching of the literature and identified sources of material. A grading tool was developed using perceived essential criteria identified by the older adults and material was graded according to the criteria within this scale. RESULTS: Fifty-seven resources from over twenty-eight sources were identified. These materials were identified as being easily accessible, readable and relevant. Many of the web based materials were not always easy to find or readily available so they were excluded by the participants. All but one were UK based. Forty-four items were identified as meeting the key criteria for inclusion in the study. This included five key categories as follows; books, internet, magazines, leaflets, CD's/Tapes. CONCLUSION: This project was able to identify a number of exemplars of self management material along with some general rules regarding the categories identified. We must point out that the materials identified were not age specific, were often locally developed and would need to be adapted to older adults with chronic pain. For copyright issues we have not included them in this paper. The key message is really related to the format rather than the content. However, the group acknowledge that these may vary according to the requirements of each individual older adult and therefore recommend the development of a leaflet to help others in their search for resources. This leaflet has been developed as part of Phase IV of the EOPIC study. | Humans | Patient Participation | Aged | chronic pain | OK |
| Maurice P. | Paterson |  |  |  |  | Orthopedics | 2013-03-00 Mar 2013 | Tablets are becoming commonplace in the health care setting. Patients often request to view their radiographs after sustaining trauma. This can be challenging, especially if patients are immobile. The authors performed a prospective, questionnaire-based study to assess inpatient desire to view radiographs on tablets and whether viewing images affected patient-rated outcomes of understanding and satisfaction. Enabling trauma patients to view their images on a tablet is a worthwhile practice because it improves patient involvement in decision making, satisfaction, perceived understanding, and overall experience. | Male | Humans | Patient Participation | Physician-Patient Relations | OK |
| Pei-Fan | Mu |  |  |  |  | Midwifery | 2010-08-00 Aug 2010 | OBJECTIVE: to explore women's experiences in interaction with their midwives during their antenatal checks and during labour. DESIGN: a qualitative study using a phenomenological approach. Data were collected via tape-recorded interviews. All interviews were transcribed verbatim. Data were analysed using Colaizzi's method for data analysis. SETTING: the homes of the study participants in the district of a Taipei (Taiwan) teaching hospital. PARTICIPANTS: a purposive sample of 11 Taiwanese women, one primipara, and 10 multiparae, who were one to three months post-childbirth at the time of interview. FINDINGS: five major themes revealed the essence of women's experiences of their interaction with a midwife during pregnancy and childbirth: (1) being respected, (2) being accompanied, (3) trust, (4) being satisfied, and (5) professional competence. KEY CONCLUSIONS: the women recognised the service model of the midwife; they treasured their mutual relationships and the benefits that women derived from midwifery care during childbirth. In Taiwan, the government is mandated to offer midwifery models of care in hospitals, and to allow women to choose different types of care provider. IMPLICATIONS FOR PRACTICE: an awareness of women's experiences will help identify the caring behaviours as recognised by the women and may help health-care professionals provide better support and care for women during the pregnancy and childbirth periods. These findings can serve as references for future midwifery practice models and improvements in quality of care. | Humans | Patient Participation | Patient Satisfaction | Questionnaires | OK |
| Cornelia M. | Ruland |  |  |  |  | Cancer Nursing | 2013-03-00 mars 2013 | Background: Patients are experts of their own symptoms and worries, but tend not to express their concerns spontaneously in the consultation. Even when emotions are brought up, they are discussed briefly. Objective: The objective of this study was to examine the impact of an interactive tailored patient assessment (Choice) on communication of emotional cues and concerns expressed by cancer patients in terms of source of initiation of cues/concern, explicitness, timing during the consultation, and consultation type (inpatient/outpatient). Methods: We audiotaped and coded consultations between cancer patients and nurses or physicians in 1 control group (n = 99) with standard consultations and 1 intervention group (n = 97) where patients used Choice prior to the consultation. Direct and interaction effects were tested using multilevel analyses. Results: In the Choice intervention group, there were significantly more frequent and more explicit expressions of cues and concerns; more clinician-initiated concerns occurred during the first 10 minutes; and it was more likely for any cue or concern to be succeeded by a subsequent one. In consultations with many cues/concerns, these were on average more emotionally descriptive or explicit and occurred somewhat earlier in the consultation in the Choice group. Furthermore, more cues/concerns were expressed in inpatient consultations with nurses than in outpatient consultations with physicians. Conclusion: Cancer patients’ expressions of cues and concerns do not occur at random. More cues/concerns are expressed early in the consultation, in consultations with nurses, and in the Choice intervention group. Implication for Practice: Choice may be a useful clinical tool. Use of Choice might aid cancer patients in handling their emotions. (PsycINFO Database Record (c) 2014 APA, all rights reserved). (journal abstract) | Emotions | Therapeutic Processes | Professional Consultation | Anxiety | OK |
| Lena | Hartelius | Ulrika | Ferm |  |  | Disability and Rehabilitation. Assistive Technology | 2013-01-00 Jan 2013 | PURPOSE: To evaluate the function of Talking Mats (TM) in a discussion group for people with Huntington's disease (HD). METHOD: Four persons with HD and their group leader participated. The group was videotaped during four discussions, two with and two without TM. A mixed method quantitative and qualitative study was implemented by evaluating the conversations using the protocol Effectiveness Framework of Functional Communication by analyzing the number of follow-up questions and by post discussion interviews. RESULTS: The results showed improved effectiveness of communication for all participants and an increased number of follow-up questions, both from the group leader and from some of the participants. All participants and the leader were positive regarding the use of TM but there were also drawbacks. CONCLUSIONS: TM could be useful in discussion groups for people with HD. An increased number of follow-up questions when TM is used may lead to a deeper understanding within the group. This study is the first of its kind and more research in the area is needed. [Box: see text]. | Male | Humans | Patient Participation | Questionnaires | OK |
| Georgia | Scott-Brien | Anne | Macdonald | Juliet | MacArthur | Journal of Child Sexual Abuse | 2011-03-00 Mar 2011 | This study aimed to elicit perceptions and experiences of talking therapy services for CSA survivors and professionals utilizing qualitative interviews and analyzing transcripts using Interpretative Phenomenological Analysis. Participants included 13 adult survivors and 31 professionals in statutory and voluntary services in Scotland. Main themes were benefits from and challenges of the therapeutic process. Benefits included a trusting therapeutic relationship, feeling safe to disclose, breaking isolation, enhancing self-esteem and self-worth, contextualizing the abuse, and moving toward recovery. Challenges included trauma-focused work, supportive contact, continuity and consistency of services, accessibility during acute episodes, hearing and managing disclosures, child protection issues, and availability and accessibility of services. The findings support a greater emphasis on relational models, supervision, and training. | Male | Humans | Patient Participation | Physician-Patient Relations | OK |
|  |  |  |  |  |  | Neurogastroenterology and Motility: The Official Journal of the European Gastrointestinal Motility Society | 2014-02-00 Feb 2014 | BACKGROUND: Patient reported outcome (PRO) is an important healthcare concept that describes patient's participation in their care by self-evaluation, usually in the form of questionnaires. This report describes an unique computerized technique, electronic PRO (ePRO), for following the progress of patients with irritable bowel syndrome (IBS). METHODS: Patients first completed a series of questionnaires, including questions about their illness history, symptom severity, and, in this application, psychological and relationship issues. The symptom severity and psychological questionnaires were then completed at intervals by the patients on their own computers. The ePRO was constructed to allow scores to be automatically summed and placed on a time-line graph for review at the time of the next office visit. KEY RESULTS: Of the 32 patients who completed the initial set of questionnaires, 20 maintained participation in the program for a 6-month period. Of those 20 patients, median number of submissions was 7.0; median interval between questionnaire submissions was 3.0 weeks, whereas median interval between office visits was 5.9 weeks. On average, questionnaire completion took less than 5 min and was positively experienced by the patients. CONCLUSIONS & INFERENCES: The ePRO program proved to be technically feasible, clinically useful, and positively experienced by the patients. It provides a focus on a collaborative conversation between physician and patient. It has significant potential as a technique for evaluating outcome in response to various therapies. | Humans | Patient Participation | Questionnaires | Severity of Illness Index | OK |
|  |  |  |  |  |  | Cochlear Implants International | 2010-06-00 Jun 2010 | | Time Factors | Male | Humans | Patient Participation | OK |
| Elsa | Marziali | Anne | Hunt | Deirdre R. | Dawson | Brain Injury | 2013-00-00 2013 | PRIMARY OBJECTIVE: To investigate the feasibility of implementing the Cognitive Orientation to daily Occupational Performance approach (CO-OP) in a telerehabilitation format and to examine its impact on community integration and executive dysfunction for adults with traumatic brain injury (TBI). RESEARCH DESIGN: A pilot series of three case studies with 3-month follow-up was conducted. METHODS AND PROCEDURES: Three adults (all males, >10 years post-TBI) and their significant others were recruited. The CO-OP intervention, a meta-cognitive approach, was delivered through videoconferencing via Internet to train three of five participant-identified goals. Two goals were not trained to allow examination of transfer. Outcome measures included the Canadian Occupational Performance Measure, the Mayo-Portland Adaptability Inventory-4 Participation Index, and the Dysexecutive Questionnaire. Descriptive statistical analysis was used. MAIN OUTCOMES AND RESULTS: The CO-OP approach administered in a telerehabilitation format was found to be feasible. All participants indicated self-reported improvement in both trained and untrained goals. Trends toward fewer symptoms of executive dysfunction and greater community integration were demonstrated. All participants expressed satisfaction with the Internet delivery method. CONCLUSIONS: Telerehabilitation shows promise as a way to deliver the CO-OP approach and may help promote community integration of individuals living with TBI. Further study is warranted. | Male | Humans | Patient Participation | Patient Satisfaction | OK |
| Dawn | Stacey | Glyn | Elwyn |  |  | BMC medical informatics and decision making | 2013-00-00 2013 | In 2003, the International Patient Decision Aid Standards (IPDAS) Collaboration was established to enhance the quality and effectiveness of patient decision aids by establishing an evidence-informed framework for improving their content, development, implementation, and evaluation. Over this 10 year period, the Collaboration has established: a) the background document on 12 core dimensions to inform the original modified Delphi process to establish the IPDAS checklist (74 items); b) the valid and reliable IPDAS instrument (47 items); and c) the IPDAS qualifying (6 items), certifying (6 items + 4 items for screening), and quality criteria (28 items). The objective of this paper is to describe the evolution of the IPDAS Collaboration and discuss the standardized process used to update the background documents on the theoretical rationales, evidence and emerging issues underlying the 12 core dimensions for assessing the quality of patient decision aids. | Time Factors | Decision Support Techniques | Humans | Patient Participation | OK |
| Daniela | Barge | Roberto P. | Bakker |  |  | PloS One | 2014-00-00 2014 | BACKGROUND: Routine Outcome Monitoring (ROM) is used as a means to enrich the process of treatment with feedback on patient outcomes, facilitating patient involvement and shared decision making. While traditional ROM measures focus on retrospective accounts of symptoms, novel mHealth technology makes it possible to collect real life, in-the-moment ambulatory data that allow for an ecologically valid assessment of personalized and contextualized emotional and behavioural adjustment in the flow daily life (mROM). METHOD: In a sample of 34 patients with major depressive disorder, treated with antidepressants, the combined effect of treatment and natural course was examined over a period of 18 weeks with Ecological Momentary Assessment (EMA). EMA consisted of repeated, within-subject, mini-measurements of experience (eg positive affect, negative affect, medication side effects) and context (eg stressors, situations, activities) at 10 unselected semi-random moments per day, for a period of six days, repeated three times over the 18-week period (baseline, week 6 and week 18). RESULTS: EMA measures of emotional and behavioural adjustment were sensitive to the effects of treatment and natural course over the 18-week period, particularly EMA measures focussing on positive mood states and the ability to use natural rewards (impact of positive events on positive mood states), with standardized effect sizes of 0.4-0.5. EMA measures of activities, social interaction, stress-sensitivity and negative mood states were also sensitive to change over time. CONCLUSION: This study supports the use of mROM as a means to involve the patient in the process of needs assessment and treatment. EMA data are meaningful to the patient, as they reflect daily life circumstances. Assessment of treatment response with mROM data allows for an interpretation of the effect of treatment at the level of daily life emotional and social adjustment - as an index of health, obviating the need for an exclusive focus on traditional measures of 'sickness'. | | | | | OK |
| Susan | Eller | John A. | Vozenilek | Danielle M. | McCarthy | Patient Education and Counseling | 2014-09-00 Sep 2014 | OBJECTIVE: To explore patient observations of teamwork-related behaviors such as inter-team communication through a newly designed survey. METHODS: In this cross-sectional study, 101 patients (N=86) and caregivers (N=15) recruited from the emergency department (ED) of an urban, academic medical center (>85,000 visits/year) completed the 16-item Patients' Insights and Views Observing Teams (PIVOT) Survey. We evaluated validity evidence through descriptive statistics and analysis including a Many-facet Rasch model to determine associations between questionnaire items and sociodemographic characteristics. RESULTS: Participant responses provided evidence survey items performed well and reflected patients' awareness of team behaviors such as inter-team communication, coordination, and keeping teammates informed. Also, participants responded about the consistency of information from team members and knowing what people's jobs were on the team. Rasch analysis largely supported that the PIVOT items reflected the intended content area and adequacy of ratings scales supporting evidence of response processes. High internal consistency (Cronbach alpha, r=.87) supported evidence of internal structure. As expected, response patterns differed by ED visit acuity level and length of stay. CONCLUSIONS: The PIVOT survey offered a means to collect patient and caregiver observations of health care teams. PRACTICE IMPLICATIONS: PIVOT survey responses may contribute to evaluation of teamwork behaviors. | | | | | OK |
| Susan | Eller | John A. | Vozenilek | Danielle M. | McCarthy | Patient Education and Counseling | 2014-06-16 juin 16, 2014 | OBJECTIVE: To explore patient observations of teamwork-related behaviors such as inter-team communication through a newly designed survey. METHODS: In this cross-sectional study, 101 patients (N=86) and caregivers (N=15) recruited from the emergency department (ED) of an urban, academic medical center (&gt;85,000 visits/year) completed the 16-item Patients’ Insights and Views Observing Teams (PIVOT) Survey. We evaluated validity evidence through descriptive statistics and analysis including a Many-facet Rasch model to determine associations between questionnaire items and sociodemographic characteristics. RESULTS: Participant responses provided evidence survey items performed well and reflected patients’ awareness of team behaviors such as inter-team communication, coordination, and keeping teammates informed. Also, participants responded about the consistency of information from team members and knowing what people's jobs were on the team. Rasch analysis largely supported that the PIVOT items reflected the intended content area and adequacy of ratings scales supporting evidence of response processes. High internal consistency (Cronbach alpha, r=.87) supported evidence of internal structure. As expected, response patterns differed by ED visit acuity level and length of stay. CONCLUSIONS: The PIVOT survey offered a means to collect patient and caregiver observations of health care teams. PRACTICE IMPLICATIONS: PIVOT survey responses may contribute to evaluation of teamwork behaviors. (PsycINFO Database Record (c) 2014 APA, all rights reserved). (journal abstract) | Patient Participation | No terms assigned | Delivery of health care | Health care teams | Doublon |
| Robert L. | Stephens | Ye | Xu | Lawrence E. | Scholl | Journal of Health Communication | 2010-04-00 Apr 2010 | There is an ever-growing trend toward more patient involvement in making health care decisions. This trend has been accompanied by the development of "informed decision-making" interventions to help patients become more engaged and comfortable with making these decisions. We describe the effects of a prostate cancer screening decision aid on knowledge, beliefs about screening, risk perception, control preferences, decisional conflict, and decisional anxiety. Data were collected from 200 males aged 50-70 years in the general population who randomly were assigned to exposure to the decision aid or no exposure as a control condition. A Solomon four-group design was used to test for possible pretest sensitization effects and to assess the effects of exposure to the decision aid. No significant pretest sensitization effects were found. Analysis of the exposure effects found that knowledge increased significantly for those exposed to the decision aid compared with those unexposed. Exposure to the decision aid also had some influence on decreasing both decisional conflict and decisional anxiety. Decision aids can play an important role in increasing patients' knowledge and decreasing anxiety when asked to make health care decisions. | Male | Decision Making | Decision Support Techniques | Humans | OK |
|  |  |  |  |  |  |  | 2012-00-00 2012 | The Medical Education literature discusses the importance of training competent medical professionals. The public expects physicians to be sensitive and aware of multiple factors, not just medical ailments. The biopsychosocial approach is a guide used to assist doctors in the exploration of a patient's narrative and in diagnosing a patient's difficulty based on multiple aspects of a patient's life including biological, psychological and sociological. During the 1980s, a pedagogical shift occurred in medical education toward an emphasis on the importance of teaching communication skills and taking a biopsychosocial approach to medicine. However, presently there is not a known, validated biopsychosocial assessment instrument in the medical education literature. This study attempts to provide psychometric validity for a biopsychosocial evaluation instrument. This evaluation tool is currently utilized to assess third and fourth year medical students during a complex Integrated Standardized Patient Examination (ISPE). The ISPE format provides an opportunity for students to demonstrate competency of skills in an integrated fashion. A sample of 149 digital recordings of medical student ISPE encounters was used to test the validity and reliability of the biopsychosocial D-D ISPE (Diabetes, Depression, Integrated Standardized Patient Examination) scoring instrument. The instrument's psychometric properties were determined using a variety of statistical analyses. The analyses included a card sort and an exploratory factor analysis to test the instrument's dimensionality. Discriminant and convergent validity methods were conducted in order to compare the ISPE scoring instrument's constructs to the constructs of two other previously validated instruments. The D-D ISPE communication and shared decision making, indicate weaker convergent validity than hypothesized. The D-D-ISPE biological scale evidenced discriminant validity (i.e., non significant correlations) with the measures of communication and shared decision making. The coder's scores of the two comparison instruments demonstrated good inter-rater reliability. The initial validity test also determined the instrument is reliable and valid. Cronbach's alpha tests determined the instrument as a whole and its subscales demonstrate high internal consistency. The instrument dimensionality analyzed by the card sort and the factor analysis, demonstrated the instrument possess three distinctive biological, psychological and sociological/communication factors. The psychological, biological and social Communication subscales are all moderately to strongly correlate with the D-D ISPE total scores. These findings suggest that the instrument can assess biopsychosocial competency of medical students during an ISPE examine focused on diabetes and depression. Finally, strengths and limitations of the study are examined and its implications for future use in medical education and research are explored. (PsycINFO Database Record (c) 2012 APA, all rights reserved) | Professional Competence | test validity | test reliability | medical students | OK |
| Yuelin | Li | Shawna | Edgerson | Phyllis | Butow | Patient Education and Counseling | 2012-08-00 Aug 2012 | OBJECTIVE: A Question Prompt List (QPL) is a proven, simple intervention to aid patients to be active participants in consultations with their physicians by asking questions. We aimed to further develop and test the efficacy of a targeted QPL for clinical trials (QPL-CT). METHODS: Breast, Lung and Genitourinary cancer patients who were facing a discussion about a therapeutic clinical trial completed short pre- and post-consultation questionnaires and used the QPL-CT in their discussions with their oncologists. RESULTS: 30 participants were recruited from 6 oncologists. All QPL-CT questions were selected by at least one-third of participants. Participants mostly wanted and asked questions about personal trial benefit. Oncologists provided information about personal benefit to varying degrees, thus patients did not ask some questions. Patients were still left with some unasked and unanswered questions. CONCLUSION: The QPL-CT has potential as a simple, inexpensive intervention to aid such communication. Further investigation is needed to demonstrate the efficacy of the QPL-CT in improving cancer patient outcomes. PRACTICE IMPLICATIONS: These preliminary finding suggest that important areas of clinical trials are overlooked in clinical consultations. The QPL-CT may be an effective method to encourage oncologists to endorse patient question asking about clinical trials and prompt patient questions. | Male | Humans | Patient Participation | Patient Satisfaction | OK |
|  |  |  |  |  |  | International journal of STD & AIDS | 2013-05-00 May 2013 | Genitourinary medicine (GUM) departments need to be resource efficient to manage the increasing numbers of patients seeking to access services. At the Edinburgh GUM department, we wished to develop a new No-Talk Testing (NTT) clinic for asymptomatic, low-risk patients attending for routine sexually transmitted infection (STI) screening. We undertook a questionnaire feasibility study to determine patient acceptability and ability to self-screen for this service. A total of 267 respondents completed questionnaires regarding acceptability of a future NTT service; 227 agreed to comparison of their self-screen with clinician risk-assessment. Overall, patient acceptability for a future NTT service was high, with an average of 7.8/10 awarded for opinion. Seventy-three percent of patients agreed they would consider utilizing such a service in the future. Sixty-one percent of respondents suggested at least one benefit to a future NTT service; principally, prospects for increased speed, efficiency, capacity and reduced waiting times. Comparing STI risk self-assessment with clinician assessment, discrepancies were identified for 37% of individuals. However, a majority (70%) of the discrepancies identified were due to a risk being noted in the self-screen alone, and missed from clinician notes. In summary, the study demonstrated NTT as acceptable and feasible. Based on these results, we have now successfully introduced such a service within our department. | Male | Humans | Patient Participation | Questionnaires | OK |
|  |  |  |  |  |  | Journal of Nuclear Cardiology: Official Publication of the American Society of Nuclear Cardiology | 2012-06-00 Jun 2012 | | Humans | Patient Participation | United States | Cardiovascular Diseases | OK |
| H. | Raspe | T. | Meyer |  |  | Gesundheitswesen (Bundesverband Der Ärzte Des Öffentlichen Gesundheitsdienstes (Germany)) | 2013-07-00 Jul 2013 | OBJECTIVES: The Social Code Book Nine (SGB IX), which was implemented in 2001, contains the "Wunsch- und Wahlrecht" for medical rehabilitation services in para. 9. This describes a right to participate and choose in the process of rehabilitation. The aim of the study was to collect representative data about the implementation of the para. 9 SGB IX during application for medical rehabilitation services from the applicants' point of view. METHODS: A total of 2 000 applicants for rehabilitation services (all somatic and psychosomatic indications) of a federal pension fund and a compulsory health insurance fund were invited to take part in a postal survey about the "Wunsch- und Wahlrecht". RESULTS: The response rate was 66%. Most respondents reported that their application for rehabilitation services was approved immediately. People who supported respondents during application and gave information about the "Wunsch- und Wahlrecht" differed according to the kind of rehabilitation services the respondent applied for. Half of the respondents reported to have known about their "Wunsch- und Wahlrecht". The percentage was considerably higher for patients applying for post-hospital rehabilitation. Approximately 2/3 of the respondents had wishes concerning the choice of rehabilitation clinic. Half of those respondents reported their wishes to the insurer. Most wishes concerned the choice of a specific clinic, a place/region or the clinic's distance from the applicant's home. Wishes reported to the insurer were often considered (89%). Most important selection criteria for rehabilitation clinics were aspects of accommodation, clinic facilities and location, followed by aspects of treatment and the respectful treatment of rehabilitation patients. Concerning the mode of information about rehabilitation clinics, applicants preferred brochures (vs. internet), comparative and impartial information about the clinic and subjective accounts of individual rehabilitation patients. CONCLUSIONS: This study is an evaluation of how the "Wunsch- und Wahlrecht" has been translated to reality of rehabilitation services provided by a federal pension fund and a compulsory health insurance fund. The findings show that many respondents know about their "Wunsch- und Wahlrecht" and make use of it to a certain degree. At the moment wishes still relate mainly to the place or region of rehabilitation. This shows the necessity of informing applicants about their "Wunsch- und Wahlrecht" on a regular basis. At the same time they must be enlightened about potentially important criteria for choosing a rehabilitation clinic. | Male | Humans | Patient Participation | Female | OK |
| Lars | Hölzel | Daniela | Simon | Andreas | Loh | Patient Education and Counseling | 2010-07-00 juillet 2010 | Objective: To develop and psychometrically test a brief patient-report instrument for measuring Shared Decision Making (SDM) in clinical encounters. Methods: We revised an existing instrument (Shared Decision Making Questionnaire; SDM-Q), including the generation of new items and changing the response format. A 9-item version (SDM-Q-9) was developed and tested in a German primary care sample of 2351 patients via face validity ratings, investigation of acceptance, as well as factor and reliability analysis. Findings were cross-validated in a randomly selected subsample. Results: The SDM-Q-9 showed face validity and high acceptance. Factor analysis revealed a clearly one-dimensional nature of the underlying construct. Both item difficulties and discrimination indices proved to be appropriate. Internal consistency yielded a Cronbach’s α of 0.938 in the test sample. Conclusion: The SDM-Q-9 is a reliable and well accepted instrument. Generalizability of the findings is limited by the elderly sample living in rural areas of Germany. While the current results are promising, further testing of criterion validity and administration in other populations is necessary. Practice implications: The SDM-Q-9 can be used in studies investigating the effectiveness of interventions aimed at the implementation of SDM and as a quality indicator in health services assessments. (PsycINFO Database Record (c) 2013 APA, all rights reserved). (journal abstract) | Decision Making | Questionnaires | Psychometrics | Test Construction | Doublon |
| Lars | Hölzel | Daniela | Simon | Andreas | Loh | Patient Education and Counseling | 2010-07-00 Jul 2010 | OBJECTIVE: To develop and psychometrically test a brief patient-report instrument for measuring Shared Decision Making (SDM) in clinical encounters. METHODS: We revised an existing instrument (Shared Decision Making Questionnaire; SDM-Q), including the generation of new items and changing the response format. A 9-item version (SDM-Q-9) was developed and tested in a German primary care sample of 2351 patients via face validity ratings, investigation of acceptance, as well as factor and reliability analysis. Findings were cross-validated in a randomly selected subsample. RESULTS: The SDM-Q-9 showed face validity and high acceptance. Factor analysis revealed a clearly one-dimensional nature of the underlying construct. Both item difficulties and discrimination indices proved to be appropriate. Internal consistency yielded a Cronbach's alpha of 0.938 in the test sample. CONCLUSION: The SDM-Q-9 is a reliable and well accepted instrument. Generalizability of the findings is limited by the elderly sample living in rural areas of Germany. While the current results are promising, further testing of criterion validity and administration in other populations is necessary. PRACTICE IMPLICATIONS: The SDM-Q-9 can be used in studies investigating the effectiveness of interventions aimed at the implementation of SDM and as a quality indicator in health services assessments. | Male | Decision Making | Humans | Patient Participation | OK |
|  |  |  |  |  |  | Journal of intellectual disability research: JIDR | 2011-08-00 Aug 2011 | BACKGROUND: This study sought to describe the activities and participation of adolescents with autism spectrum disorders (ASD) in Singapore and to examine the suitability of the Activity and Participation component of the International Classification of Functioning, Disability and Health for achieving this purpose. This information may guide the development of intervention programmes for adolescents and adults as well as the provision of a means to document meaningful outcomes. METHODS: Parents of 20 adolescents with ASD attending special schools in Singapore were interviewed using the Vineland Adaptive Behavioural Scales - Second Edition and the Activities and Participation Rating Scale (APRS), which was developed for this study. RESULTS: The adolescents with ASD were rated to have more difficulties with participation than with the engagement of activities. Individual domain analyses indicate no difficulties with mobility and mild difficulties with self-care. The performance of general tasks and demands were rated as less problematic than domestic, major life areas, communication and interpersonal interactions. The adolescents with ASD were rated to have more difficulties in communication and community environments than in at home. In addition, analysis of associations between the APRS and Vineland Adaptive Behavioural Scales - Second Edition reveal a pattern of strong relationships between sub-tests. CONCLUSION: This study highlights the imperative for researchers and practitioners alike to develop a focus on strengths, generalisation and the quality of life of adolescents with ASD. The APRS also shows promise in helping document outcomes for adolescents with ASD in Asia and further development of this instrument is needed. | Male | Humans | Patient Participation | Female | OK |
| Jakob | de Vries | Peter C. | Baas | Robbert | Sanderman | Psychology & Health | 2010-11-00 Nov 2010 | This prospective study examines the cognitive and behavioural mediators of the relation between personal control and the initial response to a breast cancer diagnosis as well as subsequent psychological adjustment. A total of 143 patients participated immediately after diagnosis (T1), after surgery (T2) and 2 months after the end of treatment (T3), of whom 92 also completed a questionnaire pre-diagnosis (T0). The buffering effect of personal control on psychological distress shortly after diagnosis was mediated by cancer-specific cognitions, i.e. threat appraisal and coping self-efficacy. Moreover, a strong sense of personal control predicted lower levels of anxiety 2 months after the end of treatment, but was unrelated to distress at T3. The adaptive effect on anxiety was mediated by threat appraisal and active engagement in social life after surgery, but not by active patient participation or coping self-efficacy. These results confirm and explain the adaptive effect of control. Apparently, women with a low sense of control appraise cancer and their personal coping skills more negatively, which makes them vulnerable to distress in response to diagnosis. Furthermore, women with a strong sense of control might regulate anxiety by remaining engaged in social life. | Humans | Questionnaires | Female | Aged | OK |
| Sara | Urowitz | Emma | Apatu | Christine | Massey | BMC medical informatics and decision making | 2010-00-00 2010 | BACKGROUND: Personal health records (PHRs) provide patients with access to personal health information (PHI) and targeted education. The use of PHRs has the potential to improve a wide range of outcomes, including empowering patients to be more active participants in their care. There are a number of widespread barriers to adoption, including privacy and security considerations. In addition, there are clinical concerns that patients could become anxious or distressed when accessing complex medical information. This study assesses the implementation of a PHR, and its impact on anxiety levels and perceptions of self-efficacy in a sample of breast cancer patients. METHODS: A quasi-experimental pre-test/post-test design was used to collect data from participants to evaluate the use of the PHR. Study participants completed background and pre-assessment questionnaires and were then registered into the portal. By entering an activation key, participants were then able to review their lab results and diagnostic imaging reports. After six weeks, participants completed post-assessment questionnaires and usability heuristics. All data were collected using an online survey tool. Data were cleaned and analyzed using SAS v9.1. RESULTS: A total of 311 breast cancer patients completed demographic and pre-assessment questionnaires, 250 registered to use the online intervention, and 125 participants completed all required study elements. Matching the pre- and post-anxiety scores demonstrated a decrease in mean anxiety scores (-2.2, p = 0.03); the chemotherapy sub-group had a statistically insignificant mean increase (1.8, p = .14). There was no mean change in self-efficacy scores. CONCLUSIONS: Participants generally found the portal easy to use; however, the perceived value of improved participation was not detected in the self-efficacy scores. Having access to personal health information did not increase anxiety levels. While these results suggest that the use of this PHR may be of benefit for informing patients, further research is required to investigate the impact on the patients experiences, their participation in their care, their relationships with the health care team, and their health outcomes. | Humans | Patient Participation | Questionnaires | Female | OK |
|  |  |  |  |  |  | Journal of Gambling Studies / Co-Sponsored by the National Council on Problem Gambling and Institute for the Study of Gambling and Commercial Gaming | 2011-03-00 Mar 2011 | Increased recognition of the risks associated with gambling has resulted in a greater focus on empirical research to increase the understanding of gambling and design appropriate response strategies. Laboratory studies are a popular mode of research due to their relative ease and lower costs compared to field research; however such studies may be limited in the extent to which results can be generalized to real gambling scenarios. The current research investigated the validity of a laboratory research study using 127 university students (male = 97, mean age = 20.4) investigating the impact of harm-minimisation measures by replicating the study in gambling venues with 124 club patrons (male = 89, mean age = 44.1). The main results and effects of both studies were in the same direction, but fewer significant results were found in the venue study. Venue participants provided much less information in response to survey questions than student participants and were less likely to return follow-up questionnaires. It was concluded that both laboratory and field studies provide valuable contributions to the field, but caution should be taken in interpreting results, and where possible both methodologies should be used to verify conclusions. | Male | Humans | Patient Participation | Questionnaires | OK |
|  |  |  |  |  |  | Journal of Psychosomatic Obstetrics and Gynaecology | 2012-06-00 Jun 2012 | Patient satisfaction is frequently used to evaluate the quality of medical care and to guide the development of health care services. Improved satisfaction is a goal recommended by the Institute of Medicine and the government of the United Kingdom for health care reform. During the perinatal period, dynamic changes in physical and psychological state impose unique challenges in the assessment of satisfaction. This article reviews the measurement of satisfaction with care, together with factors that may influence satisfaction and its measurement during the perinatal period. Recommendations are also provided for further research and development of satisfaction instruments and potential interventions to improve satisfaction with perinatal care. | Humans | Patient Participation | Patient Satisfaction | Female | OK |
|  |  |  |  |  |  | Journal of Human Nutrition and Dietetics: The Official Journal of the British Dietetic Association | 2013-04-00 Apr 2013 | BACKGROUND: Dietetic students are exposed to bioethical dilemmas on clinical placements; however, the nature of these situations has not been investigated. The present study aimed to analyse students' reports of incidents and their associated personal development plans (PDP) to assess the type of ethical incidents faced and how they cope with them. METHODS: Final-year dietetic students were recruited via an announcement in class. The students' PDPs were analysed using thematic analysis to identify the type of ethical issues faced and how they felt about them. The 500-word PDPs were submitted after the final placement. RESULTS: Thirteen (13/26) students identified 'ethics' as a learning need and most of these incidents were related to enteral feeding (10/13). Ethical issues identified related to nonmaleficence; best interest; consent and capacity; truth telling and integrity; autonomy and substituted decision-making; futile treatment; beneficence and confidentially. The themes for how the students coped included being unsure; lack of confidence or knowledge with ethics and/or with communication; lack of experience; unease/concern about shared decision-making; and their own role in the situation. CONCLUSIONS: Student dietitians are exposed to a wide range of complex ethical clinical situations, with those related to enteral feeding being the most common. Students felt unsure of what to do and lacked confidence, which resulted in unease and concern; however, students appeared to have some ethical sensitivity. Communication problems with the multidisciplinary team or families were a common theme. Clinical educators need to support students in these situations and act as role models. | Humans | Young Adult | Adult | self report | OK |
| Anthony V. | D'Amico | Michael W. | Kattan | Martin G. | Sanda | BJU international | 2010-05-00 May 2010 | OBJECTIVE: To design a decision-support tool to facilitate evidence-based treatment decisions in clinically localized prostate cancer, as individualized risk assessment and shared decision-making can decrease distress and decisional regret in patients with prostate cancer, but current individual models vary or only predict one outcome of interest. METHODS: We searched Medline for previous reports and identified peer-reviewed articles providing pretreatment predictive models that estimated pathological stage and treatment outcomes in men with biopsy-confirmed, clinical T1-3 prostate cancer. Each model was entered into a spreadsheet to provide calculated estimates of extracapsular extension (ECE), seminal vesicle invasion (SVI), and lymph node involvement (LNI). Estimates of the prostate-specific antigen (PSA) outcome after radical prostatectomy (RP) or radiotherapy (RT), and clinical outcomes after RT, were also entered. The data are available at http://www.capcalculator.org. RESULTS: Entering a patient's 2002 clinical T stage, Gleason score and pretreatment PSA level, and details from core biopsy findings, into the CaP Calculator provides estimates from predictive models of pathological extent of disease, four models for ECE, four for SVI and eight for LNI. The 5-year estimates of PSA relapse-free survival after RT and 10-year estimates after RP were available. A printout can be generated with individualized results for clinicians to review with each patient. CONCLUSIONS: The CaP Calculator is a free, online 'clearing house' of several predictive models for prostate cancer, available in an accessible, user-friendly format. With further development and testing with patients, the CaP Calculator might be a useful decision-support tool to help doctors promote evidence-based shared decision-making in prostate cancer. | Male | Decision Support Techniques | Humans | Evidence-based medicine | OK |
| Matthias | Rose | Neil K. | Aaronson | John E. | Chaplin | Health and Quality of Life Outcomes | 2013-00-00 2013 | Patient-reported outcomes (PROs) play an increasingly important role in clinical practice and research. Modern psychometric methods such as item response theory (IRT) enable the creation of item banks that support fixed-length forms as well as computerized adaptive testing (CAT), often resulting in improved measurement precision and responsiveness. Here we describe and discuss the case for developing an international core set of PROs building from the US PROMIS® network.PROMIS is a U.S.-based cooperative group of research sites and centers of excellence convened to develop and standardize PRO measures across studies and settings. If extended to a global collaboration, PROMIS has the potential to transform PRO measurement by creating a shared, unifying terminology and metric for reporting of common symptoms and functional life domains. Extending a common set of standardized PRO measures to the international community offers great potential for improving patient-centered research, clinical trials reporting, population monitoring, and health care worldwide. Benefits of such standardization include the possibility of: international syntheses (such as meta-analyses) of research findings; international population monitoring and policy development; health services administrators and planners access to relevant information on the populations they serve; better assessment and monitoring of patients by providers; and improved shared decision making.The goal of the current PROMIS International initiative is to ensure that item banks are translated and culturally adapted for use in adults and children in as many countries as possible. The process includes 3 key steps: translation/cultural adaptation, calibration, and validation. A universal translation, an approach focusing on commonalities, rather than differences across versions developed in regions or countries speaking the same language, is proposed to ensure conceptual equivalence for all items. International item calibration using nationally representative samples of adults and children within countries is essential to demonstrate that all items possess expected strong measurement properties. Finally, it is important to demonstrate that the PROMIS measures are valid, reliable and responsive to change when used in an international context.IRT item banking will allow for tailoring within countries and facilitate growth and evolution of PROs through contributions from the international measurement community. A number of opportunities and challenges of international development of PROs item banks are discussed. | Humans | Psychometrics | quality of life | Patient Outcome Assessment | OK |
| Jeffrey A. | Kline | Nilay D. | Shah | Maggie | Breslin | Trials | 2010-00-00 2010 | BACKGROUND: Chest pain is a common presenting complaint in the emergency department (ED). Despite the frequency with which clinicians evaluate patients with chest pain, accurately determining the risk of acute coronary syndrome (ACS) and sharing risk information with patients is challenging. The aims of this study are (1) to develop a decision aid (CHEST PAIN CHOICE) that communicates the short-term risk of ACS and (2) to evaluate the impact of the decision aid on patient participation in decision-making and resource use. METHODS/DESIGN: This is a protocol for a parallel, 2-arm randomized trial to compare an intervention group receiving CHEST PAIN CHOICE to a control group receiving usual ED care. Adults presenting to the Saint Mary's Hospital ED in Rochester, MN USA with a primary complaint of chest pain who are being considered for admission for prolonged ED observation in a specialized unit and urgent cardiac stress testing will be eligible for enrollment. We will measure the effect of CHEST PAIN CHOICE on six outcomes: (1) patient knowledge regarding their short-term risk for ACS and the risks of radiation exposure; (2) quality of the decision making process; (3) patient and clinician acceptability and satisfaction with the decision aid; (4) the proportion of patients who decided to undergo observation unit admission and urgent cardiac stress testing; (5) economic costs and healthcare utilization; and (6) the rate of delayed or missed ACS. To capture these outcomes, we will administer patient and clinician surveys after each visit, obtain video recordings of the clinical encounters, and conduct 30-day phone follow-up. DISCUSSION: This pilot randomized trial will develop and evaluate a decision aid for use in ED chest pain patients at low risk for ACS and provide a preliminary estimate of its effect on patient participation in decision-making and resource use. TRIAL REGISTRATION: Clinical Trials.gov Identifier: NCT01077037. | Time Factors | Decision Support Techniques | Humans | Patient Satisfaction | OK |
| G. | Spitaleri | L. | Adamoli | C. | Noberasco | Psycho-Oncology | 2014-03-00 Mar 2014 | OBJECTIVE: In a previous study, we found that patients who were offered the possibility of participation in a clinical trial had unexpressed concerns and fears that prevented them from making free or fully knowledgeable choices about their trial participation. In a selected population of patients who were offered participation in a phase I trial, we prospectively investigated whether a face-to-face discussion about their unexpressed fears might lead to a more conscious decision about whether to accept/refuse participation in the trial. METHODS: After the presentation of the trial, a questionnaire was administered to assess the presence of specific fears. Before the patients decided whether to participate in the trial, they discussed any fears that they had; finally, the impact of the discussion on the patients' choice to participate was evaluated. RESULTS: The majority (86%) of the patients thought that physicians conduct clinical trials for scientific interest, 13% felt exploited as 'guinea pigs' and 20% believed they were offered participation because they had no further hope for improvement. These existing fears were not elicited during the trial interview because the patients were themselves unaware of having them (28%) and because of fear of the doctors (3%). The possibility of discussing these fears was felt as an opportunity and made patients feel more conscious (92%) and freer (97%) when making their choice. CONCLUSIONS: Recognising and discussing misconceptions and fears, often unexpressed, make patients freer and more aware when facing the choice of whether or not \to participate in a phase I clinical trial. | Male | Decision Making | Humans | Patient Participation | OK |
| Karin | Lange |  | APG-Lehrteam MHH | |  | GMS Zeitschrift für medizinische Ausbildung | 2011-00-00 2011 | In the model medical curriculum HannibaL at Hannover Medical School (MHH, Hannover, Germany), communication skills in taking case histories and disclosing diagnoses (breaking bad news) are assessed through an objective structured clinical examination (OSCE). This is part of the examinations which at the MHH represent the equivalent to the First Part of the Medical Examinations. The second year doctor-patient communication course preparing for these examinations was evaluated during the 2009/10 academic year.Using questionnaires specific to the learning objectives, learning needs were assessed, pre-post comparisons of self-assessed competencies were performed and key teaching methods were evaluated (5-point Likert scales, "5"=fully agree). At T0 (start of the course) 267 students participated (response rate: 93.7%), of which 180 filled out the T1 questionnaire during the last session of the course (67.4%). Within-subject analyses of variance and paired t-tests were conducted.The highest learning needs were found for the "to show how"-items regarding history taking and disclosing diagnoses (M=4.4). The T1-T0 comparisons showed the greatest improvements for history taking ("to know how": mean difference = +1.7, "to show how": +1.8, p<.0001 as with all tests) and the "to know how"-item regarding the disclosure of diagnoses (+1.6), followed by the "to show how"-items on disclosing a diagnosis (+1.4), shared decision making (+1.2), self-assessing one's own strengths/weaknesses (+1.0) and confidently approaching new patients (+0.7). Students with T0 values of 1 or 2 on the respective scales improved on average by 2.2 points across all items, students with the value of 3 by 1.1, and from 4 or 5 by 0.1. Methodically, the use of simulated patients was rated the most helpful (M=4.8, 87% with the scale value 5). This doctor-patient communication course is associated with substantial improvements regarding all key learning objectives. Regarding methods, the deployed simulated patients (2-4 per 10-student-course group in 3 of the 7 course sessions, respectively) were rated the most helpful. The present evaluation calls for both further development of the doctor-patient communication curriculum at the MHH and joint activities across medical schools, which are discussed at the end of the paper. | Male | Humans | Physician-Patient Relations | Questionnaires | OK |
| Peter | Knapp | Karl | Atkin |  |  | The British Journal of General Practice: The Journal of the Royal College of General Practitioners | 2012-04-00 Apr 2012 | BACKGROUND: UK policy expects health professionals to involve patients in decisions about their care (including medicines use) and, at the same time, to follow prescribing guidelines. The compatibility of these approaches is unclear. AIM: To explore the relationship between prescribing guidelines and patient-partnership by exploring the attitudes of patients, GPs and primary care trust (PCT) prescribing advisors. DESIGN AND SETTING: A mixed-methods study using qualitative, semi-structured interviews followed by a quantitative, questionnaire survey in primary care in Northern England. METHOD: Interviews were conducted with 14 patients taking a statin or a proton pump inhibitor, eight GPs and two prescribing advisors. A multi-variate sampling strategy was used. Qualitative findings were analysed using framework analysis. Questionnaires based on themes derived from the interviews were distributed to 533 patients and 305 GPs of whom 286 (54%) and 142 (43%) responded. RESULTS: Areas of tension between guidelines and patient partnership were identified, including potential damage to trust in the doctor and reduced patient choice, through the introduction of the policy maker as a third stakeholder in prescribing decisions. Other areas of tension related to applying single condition guidelines to patients with multiple illnesses, competition for doctors' time and the perception of cost containment. Many GPs coped with these tensions by adopting a flexible approach or prioritising the doctor-patient relationship over guidelines. CONCLUSION: Rigidly applied guidelines can limit patient choice and may damage the doctor-patient relationship. GPs need flexibility in order to optimise the implementation of prescribing guidelines, while responding to individuals' needs and preferences. | Decision Making | Humans | Patient Participation | Patient Satisfaction | OK |
| Erika | Schmidt |  |  |  |  | Journal of Cardiopulmonary Rehabilitation and Prevention | 2011-12-00 2011 Nov-Dec | BACKGROUND: The purpose of this study was to examine the communication preferences of patients with chronic ischemic heart disease (CIHD) and matching between the preferences and physician communication behavior. Prior to this, psychometric testing was performed on a questionnaire on the perceived communication behavior of the physician (KOVA Questionnaire). METHODS: Patients with CIHD undergoing rehabilitation (N = 342) in Germany were questioned. At the beginning of rehabilitation, patients answered questions about their communication preferences (KOPRA Questionnaire), and at the end of rehabilitation, they answered questions regarding the perceived communication behavior of the physician. Preference-matching values were determined by combining the KOPRA and KOVA items. RESULTS: The KOVA Questionnaire psychometric properties proved to be good. Patients with CIHD indicated clear and open communication and patient participation were especially important. This was followed by emotionally supportive communication and, finally, communication about personal things. Overall, the behavior of physicians corresponded quite closely with the patients' communication preferences. However, preference matching was low (the physicians demonstrated too little of the desired behavior) regarding the open communication of bad news and explanation of treatments. There was relatively high fulfillment of expectations on the part of patients in terms of seeking information from the physician, the physician's explanation of the diagnosis, and regarding aspects of shared decision making. We observed no gender differences, but did identify age-group differences. CONCLUSIONS: The instruments developed (KOPRA and KOVA questionnaires) can be used for communication studies of patients with chronic conditions. However, some patient communication needs seem to require greater consideration from physicians. | Male | Humans | Patient Participation | Physician-Patient Relations | OK |
| Beverly | Weidmer | Margarita | Hurtado | Quyen | Ngo-Metzger | Medical Care | 2012-09-00 Sep 2012 | BACKGROUND: There is a need for reliable and valid measures of cultural competence (CC) from the patient's perspective. OBJECTIVE: This paper evaluates the reliability and validity of the Consumer Assessments of Healthcare Providers and Systems (CAHPS) CC item set. RESEARCH DESIGN: Using 2008 survey data, we assessed the internal consistency of the CAHPS CC scales using the Cronbach α's and examined the validity of the measures using exploratory and confirmatory factor analysis, multitrait scaling analysis, and regression analysis. SUBJECTS: A random stratified sample (based on race/ethnicity and language) of 991 enrollees, younger than 65 years, from 2 Medicaid managed care plans in California and New York. MEASURES: CAHPS CC item set after excluding screener items and ratings. RESULTS: Confirmatory factor analysis (Comparative Fit Index=0.98, Tucker Lewis Index=0.98, and Root Mean Square Error or Approximation=0.06) provided support for a 7-factor structure: Doctor Communication--Positive Behaviors, Doctor Communication--Negative Behaviors, Doctor Communication--Health Promotion, Doctor Communication--Alternative Medicine, Shared Decision-Making, Equitable Treatment, and Trust. Item-total correlations (corrected for item overlap) for the 7 scales exceeded 0.40. Exploratory factor analysis showed support for 1 additional factor: Access to Interpreter Services. Internal consistency reliability estimates ranged from 0.58 (Alternative Medicine) to 0.92 (Positive Behaviors) and was 0.70 or higher for 4 of the 8 composites. All composites were positively and significantly associated with the overall doctor rating. CONCLUSIONS: The CAHPS CC 26-item set demonstrates adequate measurement properties and can be used as a supplemental item set to the CAHPS Clinician and Group Surveys in assessing culturally competent care from the patient's perspective. | Male | Humans | Patient Participation | Patient Satisfaction | OK |
| M. | Cartes |  |  |  |  | Der Unfallchirurg | 2011-09-00 Sep 2011 | The probability that an inpatient will be harmed by a medical procedure is at least 3% of all patients. As a consequence, hospital risk management has become a central management task in the health care sector. The critical incident reporting system (CIRS) as a voluntary instrument for reporting (near) incidents plays a key role in the implementation of a risk management system. The goal of the CIRS is to register system errors without assigning guilt or meting out punishment and at the same time increasing the number of voluntary reports. | Humans | Patient Participation | Cooperative Behavior | patient safety | OK |
| Joanne P. I. | Fokkema | Cees P. M. | Van Der Vleuten | Albert J. J. A. | Scherpbier | Medical Teacher | 2011-00-00 2011 | BACKGROUND: Medical curricula should focus on the future of health care. Contemporary competency frameworks for curriculum design such as Canadian Medical Education Directions for Specialists (CanMEDS), ACGME and Tomorrow's Doctors share this vision by stressing generic competencies. AIM: The objective of this study was to investigate how well a contemporary competency framework fits in with clinicians' perspectives on future health care. METHODS: Using a strategic planning approach, a semi-structured open-ended questionnaire on the future of their profession was sent to 102 Dutch gynecologists. Through inductive analysis, a future perspective and its needed competencies were identified and compared to the CanMEDS framework. RESULTS: The 62 responses showed content validity for the CanMEDS roles. Additionally, two roles were identified: advanced technology user and entrepreneur. Within the role Communicator, the focus will change through more active patient participation. The roles Collaborator and Manager are predicted to change in focus because of an increase of complex interdisciplinary teamwork and leadership roles. CONCLUSION: By studying the Dutch gynecologists' perspective of the future in a strategic planning approach, two additional roles and focus areas within a contemporary competency framework were identified. The perspective of clinicians on future health care provides valuable messages on how to design future-proof curricula. | Male | Humans | Physician-Patient Relations | Questionnaires | OK |
| Hubert | Robitaille | Stéphane | Turcotte |  |  | Zeitschrift Für Evidenz, Fortbildung Und Qualität Im Gesundheitswesen | 2012-00-00 2012 | Decisional conflict is a central determinant of decision making, particularly in the context of uncertainty. It is also one of the most frequently reported outcomes in studies on decision support interventions. Decisional conflict is defined as personal uncertainty about which option to choose. The Decisional Conflict Scale (DCS) is a self-administered questionnaire that was originally designed to assess decisional conflict in patients. The scale has since been adapted to and tested among health professionals, since decisional conflict as seen by doctors, nurses and other healthcare providers has proven useful in evaluating the quality of the shared decision making (SDM) process. In recent years, however, more and more researchers have found that evaluating the perspectives of the patient and the health professional as interdependent members of a dyad, rather than as two autonomous individuals, offers exciting avenues for developing interventions to improve decision making in the clinical setting. For that reason, the SDM community has increasingly turned its attention to a dyadic approach to SDM. In this paper, we briefly review the history of the Dyadic Decisional Conflict Scale (D-DCS), update its psychometrics based on published work, and propose a research agenda for refining it further. | Decision Making | Humans | Patient Satisfaction | Physician-Patient Relations | OK |
| Andreas | Schwarzer | Ali | Aldaoud | Dietger | Niederwieser | Psycho-Oncology | 2011-02-00 Feb 2011 | GOAL: The desire for shared decision making arises especially for frequently occurring cases of solid cancer. For hematological cancer conditions, there are no analogous results. This study compares the participation patients' desires concerning medical decisions dealing with their solid and hematological tumors. PATIENTS AND METHODS: The 533 inpatients with solid cancer (age<65: 61.0%; female: 39.6 %) and 177 patients with hematological cancer (inpatient: 62.1%, outpatient: 37.9%; age<65: 63.3%; female: 42.4%) were given a questionnaire after admission to a hospital or medical practice. The dependent variable was patient preference for control in decision making for eight different medical areas of decision. RESULTS: Descriptive results showed that patients with solid cancer had a stronger desire to participate in the decisions in six of a total of eight survey fields (p<0.01). When considering medical and socio-demographic control variables, the multivariate regression shows that the differences between the patient groups remain in all areas (p<0.01). Further predictor variables are educational background and age (p<0.05). No influence resulted from the factors of gender, medical or treatment characteristics. CONCLUSION: The results show differences between patients with hematological cancer and patients with solid tumors, and these differences concern the preference to participate in medical decisions. Hemato-oncological patients desire less active participation and prefer a more dominant role of the physician in the various areas requiring decisions. Physicians should respect this in the course of the treatment. | Male | Decision Making | Humans | Patient Participation | OK |
| Margareta | Ehnfors |  |  |  |  | Health Expectations: An International Journal of Public Participation in Health Care and Health Policy | 2014-06-17 Jun 17, 2014 | AIMS: To report on the development and initial testing of a clinical tool, The Patient Preferences for Patient Participation tool (The 4Ps), which will allow patients to depict, prioritize, and evaluate their participation in health care. BACKGROUND: While patient participation is vital for high quality health care, a common definition incorporating all stakeholders' experience is pending. In order to support participation in health care, a tool for determining patients' preferences on participation is proposed, including opportunities to evaluate participation while considering patient preferences. METHODS: Exploratory mixed methods studies informed the development of the tool, and descriptive design guided its initial testing. The 4Ps tool was tested with 21 Swedish researcher experts (REs) and patient experts (PEs) with experience of patient participation. Individual Think Aloud interviews were employed to capture experiences of content, response process, and acceptability. RESULTS: 'The 4Ps' included three sections for the patient to depict, prioritize, and evaluate participation using 12 items corresponding to 'Having Dialogue', 'Sharing Knowledge', 'Planning', and 'Managing Self-care'. The REs and PEs considered 'The 4Ps' comprehensible, and that all items corresponded to the concept of patient participation. The tool was perceived to facilitate patient participation whilst requiring amendments to content and layout. CONCLUSIONS: A tool like The 4Ps provides opportunities for patients to depict participation, and thus supports communication and collaboration. Further patient evaluation is needed to understand the conditions for patient participation. While The 4Ps is promising, revision and testing in clinical practice is required. | | | | | OK |
| Margareta | Ehnfors |  |  |  |  | Health Expectations: An International Journal of Public Participation in Health Care & Health Policy | 2014-06-17 juin 17, 2014 | Abstract Aims To report on the development and initial testing of a clinical tool, The Patient Preferences for Patient Participation tool (The 4Ps), which will allow patients to depict, prioritize, and evaluate their participation in health care. Background While patient participation is vital for high quality health care, a common definition incorporating all stakeholders' experience is pending. In order to support participation in health care, a tool for determining patients' preferences on participation is proposed, including opportunities to evaluate participation while considering patient preferences. Methods Exploratory mixed methods studies informed the development of the tool, and descriptive design guided its initial testing. The 4Ps tool was tested with 21 Swedish researcher experts (REs) and patient experts (PEs) with experience of patient participation. Individual Think Aloud interviews were employed to capture experiences of content, response process, and acceptability. Results ‘The 4Ps’ included three sections for the patient to depict, prioritize, and evaluate participation using 12 items corresponding to ‘Having Dialogue’, ‘Sharing Knowledge’, ‘Planning’, and ‘Managing Self‐care’. The REs and PEs considered ‘The 4Ps’ comprehensible, and that all items corresponded to the concept of patient participation. The tool was perceived to facilitate patient participation whilst requiring amendments to content and layout. Conclusions A tool like The 4Ps provides opportunities for patients to depict participation, and thus supports communication and collaboration. Further patient evaluation is needed to understand the conditions for patient participation. While The 4Ps is promising, revision and testing in clinical practice is required. (PsycINFO Database Record (c) 2014 APA, all rights reserved). (journal abstract) | Patient Participation | clinical tool | content validity | instrument development | Doublon |
| Kimberly H. | Davis | M. Chris | Runken | M. E. | Beach | Headache | 2012-04-00 Apr 2012 | OBJECTIVE: To assess the psychometric properties of a new patient-reported migraine instrument, the Completeness of Response Survey (CORS), which measures a comprehensive set of factors important to patients' decisions regarding the initiation and continuation of treatment. BACKGROUND: Traditionally, migraine treatments and the instruments used to demonstrate their efficacy have focused on the relief of headache pain. As new treatments emerge with the potential for more complete and consistent migraine relief, more comprehensive tools are needed to demonstrate these benefits. The CORS includes 2 modules, the static CORS, which comprehensively evaluates one treatment at one time point, and the comparative CORS, which provides a more global comparison between 2 treatments at one time point. Together, the 2 modules can measure unmet treatment needs and improvements over the course of a clinical study. METHODS: Data from an 8-site study comparing 147 patients' recent experiences with their current triptan therapy and 2 months of study treatment with a single-tablet formulation of sumatriptan/naproxen sodium were used to conduct a preliminary psychometric evaluation of the CORS. The study included both modules of the CORS, the Headache Impact Test, the revised Patient Perception of Migraine Questionnaire, and a migraine diary. RESULTS: The CORS response categories in both the static and comparative modules demonstrated limited floor or ceiling effects and few missing values (<3%). Inter-item correlations, principal components analysis (component loading range: 0.62 to 0.95), and high estimates of internal consistency (alpha range: 0.88 to 0.94) for each composite score supported the structure and proposed scoring algorithm for the static module. The pattern of correlations between the CORS static and comparative items and composites with the revised Patient Perception of Migraine Questionnaire items and subscales, as well as the relationships between responses to selected static CORS items and the migraine diary, supported the construct validity of the CORS. CONCLUSIONS: The CORS is capable of demonstrating advantages of more comprehensive migraine therapies over traditional therapies, which are primarily focused on the resolution of headache pain, by addressing the frequency and speed with which the most common migraine symptoms are resolved and patients' return to normal functioning. This research shows evidence for the value and utility for the CORS static and comparative items and components, and further evaluation is underway. | Male | Humans | Patient Participation | Questionnaires | OK |
|  |  |  |  |  |  | Die Rehabilitation | 2011-12-00 Dec 2011 | OBJECTIVE: In the context of an increasing patient orientation in health care, surveying patients on the success of treatment has become highly relevant. The objective of the study was to develop and test an instrument for surveying the patients' perceptions of the participation relevance of changes in health status after inpatient medical rehabilitation with respect to various areas of life for 3 diagnoses: breast cancer, chronic ischemic heart disease, and chronic back pain. METHODS: The instrument refers to certain disease-specific areas of health (e. g., mobility) with regard to which the patients are asked whether they perceived an improvement and if so, what improvement in participation had ensued as a result. A questionnaire study (measuring time points: end of rehab and 6 months after the end of rehab) was conducted in 3 samples (breast cancer: n=297, chronic ischemic heart disease: n=295, chronic back pain: n=186) to test the methodology. The share of missing values and undesirable ceiling/floor effects were assessed to analyze the distribution characteristics. In a partial sub-sample, a third measuring time point was introduced approx. 10 days after the second measuring time point, and the retest reliability of the assessment of participation relevance is determined using the ICC. We also present descriptive results relative to the participation relevance from the patient perspective. RESULTS: The instrument shows good distribution characteristics for the items. Regarding retest reliability, there were satisfactory values for all diagnoses in all areas of health. The descriptive analyses showed that patients who perceived a noticeable improvement in their health status at the end of rehabilitation or 6 months after inpatient rehab generally assessed the participation relevance of this improvement in health to be "moderate" to "strong". DISCUSSION: The concept of assessing the participation relevance from the patient perspective is a satisfactorily reliable construct. By including the subjective significance of treatment success it takes into account the concept of clinical significance from the patient's perspective, a concept which has thus far been considered only insufficiently. Results show that patients ascribe the health improvements achieved by rehabilitation a noticeable relevance for their participation. | Male | Humans | Patient Participation | Patient Satisfaction | OK |
| Marcel Olde | Rikkert | Inge | Cantegreil | Kevin | Charras | International psychogeriatrics / IPA | 2012-06-00 Jun 2012 | BACKGROUND: The evidence for the effectiveness of psychosocial interventions in dementia care is growing but the implementation of available evidence is not automatic. Our objective was to develop valid quality indicators (QIs) for psychosocial dementia care that facilitate the implementation process in various countries and settings. METHODS: A RAND-modified Delphi technique was used to develop a potential set of QIs. Two multidisciplinary, international expert panels were involved in achieving content and face validity. Consensus on the final set was reached after a conference meeting where a third panel of dementia experts discussed measurability and applicability of the potential set. A retrospective cohort study was conducted to study the feasibility of using the final set in day care centers, hospitals, and nursing homes in Spain and The Netherlands. RESULTS: A total of 104 recommendations were selected from guidelines and systematic reviews and appraised for their contribution to improving the quality of dementia care by 49 dementia experts. Twenty-five experts attended the conference meeting and reached consensus on a set of 12 QIs representing the key elements of effective psychosocial care, such as shared decision-making and interventions tailored to needs and preferences. Data from 153 patient records showed that all but one QI subitem were applicable to all three settings in both countries. CONCLUSION: Our multidisciplinary and multinational strategy resulted in a set of unique QIs that aims exclusively at assessing the quality of psychosocial dementia care. Following implementation, these QIs will assist dementia care professionals to individualize and tailor psychosocial interventions. | Humans | Aged | Delphi Technique | Feasibility Studies | OK |
| Benjamin | Gedrose | Falk | Hoffmann | Barbara | Russ-Thiel | BMC health services research | 2010-00-00 2010 | BACKGROUND: In summer 2003 a disease management program (DMP) for type 2 diabetes was introduced on a nationwide basis in Germany. Patient participation and continuity of care within the DMP are important factors to achieve long-term improvements in clinical endpoints. Therefore it is of interest, if patients experience any positive or negative effects of the DMP on their treatment that would support or hamper further participation. The main objective of the study was to find out if the German Disease Management Program (DMP) for type 2 diabetes improves process and outcome quality of medical care for patients in the light of their subjective experiences over a period of one year. METHODS: Cohort study with a baseline interview and a follow-up after 10.4 +/- 0.64 months. Data on process and outcome measures were collected by telephone interviews with 444 patients enrolled and 494 patients not enrolled in the German DMP for type 2 diabetes. Data were analyzed by multivariate logistic regression analyses. RESULTS: DMP enrolment was significantly associated with a higher process quality of care. At baseline enrolled patients more often reported that they had attended a diabetes education course (OR = 3.4), have > or = 4 contacts/year with the attending physician (OR = 3.3), have at least one annual foot examination (OR = 3.1) and one referral to an ophthalmologist (OR = 3.4) and possess a diabetes passport (OR = 2.4). Except for the annual referral to an ophthalmologist these parameters were also statistically significant at follow-up. In contrast, no differences between enrolled and not enrolled patients were found concerning outcome quality indicators, e.g. self-rated health, Glycated hemoglobin (GHb) and blood pressure. However, 16-36% of the DMP participants reported improvements of body weight and/or GHb and/or blood pressure values due to enrolment - unchanged within one year of follow-up. CONCLUSIONS: In the light of patient's experiences the DMP enhances the process quality of medical care for type 2 diabetes in Germany. The lack of significant differences in outcome quality between enrolled and not enrolled patients might be due to the short program duration. Our data suggest that the DMP for type 2 diabetes should not be withdrawn unless an evidently more promising approach is found. | Humans | Program Evaluation | Quality of Health Care | Follow-Up Studies | OK |
| Graeme | Tucker | Anh-Minh | Nguyen | David | Wilson | Patient Education and Counseling | 2012-01-00 Jan 2012 | OBJECTIVE: To examine the population distribution of different types of relationships between people with chronic conditions and their doctors that influence decisions being made from a shared-decision making perspective. METHODS: A survey questionnaire based on recurring themes about the doctor/patient relationship identified from qualitative in-depth interviews with people with chronic conditions and doctors was administered to a national population sample (n=999) of people with chronic conditions. RESULTS: Three factors explained the doctor/patient relationship. Factor 1 identified a positive partnership characteristic of involvement and shared decision-making; Factor 2 doctor-controlled relationship; Factor 3 relationship with negative dimensions. Cluster analysis identified four population groups. Cluster 1 doctor is in control (9.7% of the population); Cluster 2 ambivalent (27.6%); Cluster 3 positive long-term relationship (58.6%); Cluster 4 unhappy relationship (4.4%). The proportion of 18-34 year olds is significantly higher than expected in Cluster 4. The proportion of 65+ year olds is significantly higher than expected in Cluster 1, and significantly lower than expected in Cluster 4. CONCLUSION: This study adds to shared decision-making literature in that it shows in a representative sample of people with chronic illnesses how their perceptions of their experiences of the doctor-patient relationship are distributed across the population. PRACTICE IMPLICATIONS: Consideration needs to be given as to whether it is better to help doctors to alter their styles of interactions to suit the preferences of different patients or if it is feasible to match patients with doctors by style of decision-making and patient preference. | Male | Decision Making | Humans | Patient Satisfaction | OK |
| Kenneth R. | Fox |  |  |  |  | BMC public health | 2012-00-00 2012 | BACKGROUND: The beneficial effect of physical activity for the prevention of a range of chronic diseases is widely acknowledged. These conditions are most prevalent in low-income groups where physical activity levels are consistently lower. Social marketing is the government's recommended approach to promoting physical activity but evidence of its effectiveness is limited. The purpose of this study was to examine the effect of a social marketing campaign on the monthly recruitment, attendance and retention levels at a community-based physical activity programme in a low income area. METHODS: A six-month social marketing campaign was designed and delivered in a highly-deprived suburban neighbourhood. Analysis of variance was used to assess effects on recruitment and attendance. χ2 tests of independence were used to compare dropouts and adherers and effectiveness of recruitment mechanisms. Percentages were used to compare adherence rates at intervention, pre-existing sessions in the intervention area and control area sessions. RESULTS: Attendance data were collected weekly and presented and analysed monthly to provide a view of changing participation over the six month intervention period, as compared to attendance at pre-existing sessions in the intervention area and in a control area. Recruitment into intervention sessions was significantly greater than into pre-existing and control area sessions in Month 1 (18.13v1.04 p = .007, 18.13v.30 p=.005), Month 5 (3.45v.84 p=.007, 3.45v.30 p<.001) and Month 6 (5.60v.65 p<.001, 5.60v.25 p<.001). Attendance at intervention sessions was significantly greater in all six months than at pre-existing and control area sessions; Month 1 (38.83v7.17 p<.001, 38.83v4.67, p<.001), Month 2 (21.45v6.20 p<.001, 21.45v4.00, p<.001), Month 3 (9.57v6.15 p<.001, 9.57v3.77, p<.001), Month 4 (17.35v7.31 p<.001, 17.35v4.75, p<.001), Month 5 (20.33v8.81 p=.007, 20.33v4.54 p<.001) and Month 6 (28.72v8.28 p<.001, 28.72v.4.00 p<.001). Drop-out rates in the intervention area were similar to the control area (66.2%v69.9%), and considerably lower than in pre-existing sessions (83%). In months one and two, traditional marketing techniques (posters/outdoor banners/flyers) had the greatest influence on recruitment compared to word of mouth communication (84.5%v15.5%). In months five and six word of mouth influenced 57.5% of new recruits. CONCLUSIONS: Direct comparisons with other programmes were difficult due to a lack of standard definitions of recruitment and adherence and limited reporting of findings. However when compared to pre-existing sessions and sessions delivered in a control area, monthly attendance patterns indicated that a reasonably well funded social marketing campaign increased recruitment into exercise sessions, maintained good levels of attendance and reasonable levels of adherence. Good attendance levels support on-going campaign success by offering evidence of peer and social support for the activity and increasing opportunities for social interaction. They also increase the capacity and reach of the word of mouth communication channels, the most effective form of promotion. Further study into methods of improving exercise adherence is required. | Male | Humans | Patient Participation | Questionnaires | OK |
|  |  |  |  |  |  | Psychotherapy Research: Journal of the Society for Psychotherapy Research | 2010-01-00 Jan 2010 | Evidence regarding the effect of clients' choice of treatment on treatment outcome is inconsistent. This possible effect was examined by presenting participants with two treatments of test anxiety: advanced muscle relaxation and changing of internal dialogue. Clients (N=73) were allocated to three groups: choice (participants chose their preferred treatment), no choice (participants were assigned to their preferred treatment but were led to believe they couldn't choose the treatment they were assigned to), and wait-list control (participants received no treatment until the end of the study). There was a significant linear pattern, with the choice group performing better than the no-choice group with no feeling of control, which in turn performed better than the control group. Limitations and suggestions for future research are discussed. | Male | Humans | Patient Participation | Young Adult | OK |
| Jacob | Mohrs | Patrick Je | Bindels | Milo A. | Puhan | BMC medical research methodology | 2012-00-00 2012 | BACKGROUND: In research with long-term follow-up and repeated measurements, quick and complete response to questionnaires helps ensure a study's validity, precision and efficiency. Evidence on the effect of non-monetary incentives on response rates in observational longitudinal research is scarce. OBJECTIVES: To study the impact of two strategies to enhance completeness and efficiency in observational cohort studies with follow-up durations of around 2 years. METHOD AND INTERVENTION: In a factorial design, 771 children between 2 and 5 years old and their parents participating in a prospective cohort study were randomized to three intervention groups and a control group. Three types of lotteries were run: (i) daytrip tickets for the whole family to a popular amusement park if they returned all postal questionnaires, (ii) €12.50-worth gift vouchers for sending back the questionnaire on time after each questionnaire round and (iii) a combination of (i) and (ii). MAIN OUTCOME MEASURES: Primary outcome was the proportion of participants who returned all questionnaires without any reminder. Secondary outcomes were '100% returned with or without reminder', 'probability of 100% non-response', 'probability of withdrawal', 'proportion of returned questionnaires' and 'overall number of reminders sent'. STATISTICAL ANALYSIS: After testing for interaction between the two lottery interventions, the two trials were analysed separately. We calculated risk differences (RD) and numbers needed to "treat" and their 95% confidence intervals. RESULTS: Daytrip nor voucher intervention had an effect on the proportion of participants who returned all questionnaires (RD -0.01; 95% CI-0.07 - 0.06) and (RD 0.02; 95% CI-0.50 - 0.08), respectively. No effects were found on the secondary outcomes. CONCLUSION: Our findings do not support the idea that lottery-style incentives lead to more complete response to postal questionnaires in observational cohort studies with repeated data collection and follow-up durations of around 2 years. | Male | Humans | Patient Participation | Questionnaires | OK |
| Beth | Potter | Dawn | Stacey |  |  | BMC surgery | 2014-00-00 2014 | BACKGROUND: Colorectal cancer is common in North America. Two surgical options exist for rectal cancer patients: low anterior resection with re-establishment of bowel continuity, and abdominoperineal resection with a permanent stoma. A rectal cancer decision aid was developed using the International Patient Decision Aid Standards to facilitate patients being more actively involved in making this decision with the surgeon. The overall aim of this study is to evaluate this decision aid and explore barriers and facilitators to implementing in clinical practice. METHODS: First, a pre- and post- study will be guided by the Ottawa Decision Support Framework. Eligible patients from a colorectal cancer center include: 1) adult patients diagnosed with rectal cancer, 2) tumour at a maximum of 10 cm from anal verge, and 3) surgeon screened candidates eligible to consider both low anterior resection and abdominoperineal resection. Patients will be given a paper-version and online link to the decision aid to review at home. Using validated tools, the primary outcomes will be decisional conflict and knowledge of surgical options. Secondary outcomes will be patient's preference, values associated with options, readiness for decision-making, acceptability of the decision aid, and feasibility of its implementation in clinical practice. Proposed analysis includes paired t-test, Wilcoxon, and descriptive statistics. Second, a survey will be conducted to identify the barriers and facilitators of using the decision aid in clinical practice. Eligible participants include Canadian surgeons working with rectal cancer patients. Surgeons will be given a pre-notification, questionnaire, and three reminders. The survey package will include the patient decision aid and a facilitators and barriers survey previously validated among physicians and nurses. Principal component analysis will be performed to determine common themes, and logistic regression will be used to identify variables associated with the intention to use the decision aid. DISCUSSION: This study will evaluate the impact of the rectal cancer decision aid on patients and help with planning strategies to overcome barriers and facilitate implementation of the decision aid in routine clinical practice. To our knowledge this is the first study designed to evaluate a decision aid in the field of colorectal surgery. | Decision Making | Decision Support Techniques | Humans | Patient Participation | OK |
| Naynesh | Kamani |  |  |  |  | Pediatrics | 2010-04-00 Apr 2010 | BACKGROUND: Most children with cancer enroll in clinical research trials. Whenever possible, children must provide their assent before enrolling in research studies. We studied what children aged 7 to 18 with cancer understand about research, their research-related treatment, and their preferences for inclusion in decision-making. PROCEDURE: Thirty-seven face-to-face, audiorecorded interviews using a novel, semi-structured tool, the quality-of-assent instrument, were conducted. Exploratory univariate and bivariate analyses of the quantitative data elucidated patterns and trends of understanding and preferences. RESULTS: Nineteen of the 37 children (51%) did not know or recall that their treatment was considered research, and 19 of 22 (86%) did not understand their doctor when he or she discussed the trial. More children enrolled in trials to help future children with cancer (27 of 37 [73%]), than to get better personally (22 of 37 [60%]). Irrespective of age, children with Hodgkin's disease, germ-cell tumors, and leukemia had significantly greater research awareness and appreciation than children with other cancers (P = .019 and P < .001, respectively). Although all children wanted to be involved in decision-making, 18 of 37 (49%) did not have or recall having a role in deciding to enroll in their trial, and 14 of 37 (38%) did not feel free to dissent to trial enrollment. Only 4 of 37 children (11%) discussed increased decision-making roles with parents, and only 7 of 37 (19%) discussed them with their doctors. CONCLUSIONS: Most children have limited understanding of research despite physicians' explanations. Many children reported that they feel minimally involved in the decision to enroll in clinical trials. Tools to assist investigators ascertain that children understand what they are agreeing to when they assent to research and to determine their preferences for inclusion in research may help make assent more meaningful. | Male | Humans | Patient Participation | Questionnaires | OK |
| Lise | Goulet | William | Fraser |  |  | BMC women's health | 2011-00-00 2011 | BACKGROUND: Considering the fact that a significant proportion of high-risk pregnancies are currently referred to tertiary level hospitals; and that a large proportion of low obstetric risk women still seek care in these hospitals, it is important to explore the factors that influence the childbirth experience in these hospitals, particularly, the concept of humanized birth care.The aim of this study was to explore the organizational and cultural factors, which act as barriers or facilitators in the provision of humanized obstetrical care in a highly specialized, university-affiliated hospital in Quebec province, in Canada. METHODS: A single case study design was chosen. The study sample included 17 professionals and administrators from different disciplines, and 157 women who gave birth in the hospital during the study. The data was collected through semi-structured interviews, field notes, participant observations, a self-administered questionnaire, documents, and archives. Both descriptive and qualitative deductive content analyses were performed and ethical considerations were respected. RESULTS: Both external and internal dimensions of a highly specialized hospital can facilitate or be a barrier to the humanization of birth care practices in such institutions, whether independently, or altogether. The greatest facilitating factors found were: caring and family- centered model of care, professionals' and administrators' ambient for the provision of humanized birth care besides the medical interventional care which is tailored to improve safety, assurance, and comfort for women and their children, facilities to provide a pain-free birth, companionship and visiting rules, dealing with the patients' spiritual and religious beliefs. The most cited barriers were: the shortage of health care professionals, the lack of sufficient communication among the professionals, the stakeholders' desire for specialization rather than humanization, over estimation of medical performance, finally the training environment of the hospital leading to the presence of too many health care professionals, and consequently, a lack of privacy and continuity of care. CONCLUSION: The argument of medical intervention and technology at birth being an opposing factor to the humanization of birth was not seen to be an issue in the studied highly specialized university affiliated hospital. | Humans | Patient Participation | Questionnaires | Young Adult | OK |
|  |  |  |  |  |  | Quality of Life Research: An International Journal of Quality of Life Aspects of Treatment, Care and Rehabilitation | 2010-02-00 Feb 2010 | PURPOSE: The Functional Living Index-Cancer was developed to measure quality of life in cancer trials as an adjunct to the usual clinical outcomes. The scale is considered conceptually good, since it covers a broad range of relevant aspects of quality of life, but the main criticism has been that its reliability has never been properly investigated. In this paper, we investigate the reliability of the FLIC. METHODS: We apply a new methodology based on linear mixed models that allows estimating reliability from real clinical data. The reliability of the FLIC is estimated using data coming from a longitudinal study in breast cancer. With this new approach, we avoid the need for additional data collection on which classical reliability studies are based. RESULTS: The average reliability of the FLIC over the repeated measurements is satisfactory, even though the initial measurement in the study showed a somewhat lower value. Taking into account the longitudinal character of the measurements, we show that highly reliable information can be obtained with a relatively small number of measurements per patient. CONCLUSION: The FLIC provides reliable quality of life measurements in patients with breast cancer. Additional studies would be welcome to validate these results in other populations. | Humans | Patient Participation | Questionnaires | Female | OK |
| Brigitte | Erbslöh-Möller | Mechthild | Gesmann | Hedi | Kühn-Becker | BMC musculoskeletal disorders | 2012-00-00 2012 | BACKGROUND: Consumer surveys provide information on effectiveness and side effects of medical interventions in routine clinical care. A report of fibromyalgia syndrome (FMS) consumers has not been carried out in Europe. METHODS: The study was carried out from November 2010 to April 2011. Participants diagnosed with FMS rated the effectiveness and side effects of pharmacological and non-pharmacological FMS interventions on a 0 to 10 scale, with 10 being most efficacious (harmful). The questionnaire was distributed by the German League for people with Arthritis and Rheumatism and the German Fibromyalgia Association to their members and to all consecutive FMS patients of nine clinical centers of different levels of care. RESULTS: 1661 questionnaires (95% women, mean age 54 years, mean duration since FMS diagnosis 6.8 years) were analysed. The most frequently used therapies were self-management strategies, prescription pain medication and aerobic exercise. The highest average effectiveness was attributed to whole body and local warmth therapies, thermal bathes, FMS education and resting. The highest average side effects were attributed to strong opioids, local cold therapy, gamma-amino-butyric acid analogues (pregabalin and gabapentin), tramadol and opioid transdermal systems. CONCLUSION: The German fibromyalgia consumer reports highlight the importance of non-pharmcological therapies in the long-term management of FMS, and challenges the strong recommendations for drug therapies given by FMS-guidelines. | Male | Humans | Patient Participation | Questionnaires | OK |
| Stephanie | Pahlke | Levente | Kriston | Edward | Krupat | Patient Education and Counseling | 2014-02-00 février 2014 | Objective: To translate a measure of physicians’ communication skills, the Four Habits Coding Scheme (4HCS), into German, to examine its psychometric properties, and to analyze its association with the OPTION Scale, which assesses physicians’ shared decision making (SDM) behavior. Methods: We performed a secondary data analysis of 67 audio-recorded medical consultations. Reliability, internal consistency, and factorial validity of the translated 4HCS were analyzed. The association with the OPTION Scale was examined using correlation and linear regression. Results: Testing of reliability revealed intraclass correlation coefficients above .70. Results regarding internal consistency and factorial validity were inconclusive. The correlations between the OPTION score and the four dimensions of the 4HCS were .04 (p = .782), −.14 (p = .303), −.15 (p = .279) and .55 (p &lt; .001), respectively. In multiple regression the four dimensions of the 4HCS explained substantial amount of variation in the OPTION scores (R² = .42, P &lt; .001). Conclusion: The measure showed good observer reliability, however further testing is necessary. Due to the strong interrelation of both measures, SDM should be seen in the context of broader communication skills. Practice implications: The 4HCS can be used in research and medical education. Further studies are necessary that investigate SDM within the context of communication skills. (PsycINFO Database Record (c) 2014 APA, all rights reserved). (journal abstract) | Measurement | Shared decision making | Decision Making | physicians | OK |
| Jochen | Ernst |  |  |  |  | Patient Education and Counseling | 2012-07-00 Jul 2012 | OBJECTIVE: To explore differences between conventional medicine (COM) and complementary and alternative medicine (CAM) regarding the attitude toward and the perceived use of shared decision-making (SDM) from the health professional perspective. METHODS: Thirty guideline-based interviews with German GPs and nonmedical practitioners were conducted using qualitative analysis for interpretation. RESULTS: The health professional-patient-relationship in CAM differs from that in COM, as SDM is perceived more often. Reasons for this include external context variables (e.g., longer consultation time) and internal provider beliefs (e.g., attitude toward SDM). German health care policy was regarded as one of the most critical factors which affected the relationship between GPs and their patients and their practice of SDM. CONCLUSION: Differences between COM and CAM regarding the attitude toward and the perceived use of SDM are attributable to diverse concepts of medicine, practice context variables and internal provider factors. Therefore, the perceived feasibility of SDM depends on the complexity of different occupational socialization processes and thus, different value systems between COM and CAM. PRACTICE IMPLICATIONS: Implementation barriers such as insufficient communication skills, lacking SDM training or obedient patients should be reduced. Especially in COM, contextual variables such as political restrictions need to be eliminated to successfully implement SDM. | Male | Decision Making | Humans | Patient Participation | OK |
| Pål | Romundstad | Jon | Heggland | Jostein | Holmen | BMC medical research methodology | 2012-00-00 2012 | BACKGROUND: Population based studies are important for prevalence, incidence and association studies, but their external validity might be threatened by decreasing participation rates. The 50 807 participants in the third survey of the HUNT Study (HUNT3, 2006-08), represented 54% of the invited, necessitating a nonparticipation study. METHODS: Questionnaire data from HUNT3 were compared with data collected from several sources: a short questionnaire to nonparticipants, anonymous data on specific diagnoses and prescribed medication extracted from randomly selected general practices, registry data from Statistics Norway on socioeconomic factors and mortality, and from the Norwegian Prescription Database on drug consumption. RESULTS: Participation rates for HUNT3 depended on age, sex and type of symptoms and diseases, but only small changes were found in the overall prevalence estimates when including data from 6922 nonparticipants. Among nonparticipants, the prevalences of cardiovascular diseases, diabetes mellitus and psychiatric disorders were higher both in nonparticipant data and data extracted from general practice, compared to that reported by participants, whilst the opposite pattern was found, at least among persons younger than 80 years, for urine incontinence, musculoskeletal pain and headache. Registry data showed that the nonparticipants had lower socioeconomic status and a higher mortality than participants. CONCLUSION: Nonparticipants had lower socioeconomic status, higher mortality and showed higher prevalences of several chronic diseases, whilst opposite patterns were found for common problems like musculoskeletal pain, urine incontinence and headache. The impact on associations should be analyzed for each diagnosis, and data making such analyses possible are provided in the present paper. | Male | Humans | Patient Participation | Questionnaires | OK |
| Vera-Christina | Mertens | Jikke | van der Linden | Trudy | van der Weijden | BMC family practice | 2012-00-00 2012 | BACKGROUND: Chronically ill patients often experience psychosocial problems in everyday life. A biopsychosocial approach is considered to be essential in chronic care. In Dutch primary health care the current biomedically oriented clinical practice may conflict with the biopsychosocial approach. This study is aimed to explore the views of Dutch stakeholders on achieving a biopsychosocial approach to the care of patients with chronic diseases. METHODS: In a qualitative explorative study design, we held semi-structured interviews with stakeholders, face-to-face or by telephone. Data were analysed using content analysis. Thirty representatives of Dutch patients with chronic illnesses, primary care professionals, policy makers, health inspectorate, health insurers, educational institutes and researchers were interviewed. RESULTS: Stakeholders were aware that a systematic biopsychosocial care approach is lacking in current practice. Opportunities for effective change are multidimensional. Achieving a biopsychosocial approach to care relates to active patient participation, the training of professionals, high-quality guidelines, protocols and tools, integrated primary care, research and financial issues. CONCLUSIONS: Although the principles and importance of the biopsychosocial model have been recognized, the provision of care that starts from the medical, emotional or social needs of individual patients does not fit in easily with the current Dutch health care system. All parties involved need to make a commitment to realize the ideal of biopsychosocial chronic care. Together they need to equip health professionals with skills to understand patients' multifaceted needs and to reward integrated biopsychosocial care. Patients need to be empowered to be active partners in their own care. | Male | Humans | Patient Participation | Questionnaires | OK |
| Geert | Van Hove | Staffan | Josephsson | Ignaas | Devisch | Disability and Rehabilitation | 2012-00-00 2012 | PURPOSE: To open a discourse on the concept of autonomy as a precursor for participation in individuals with Spinal Cord Injury (SCI) by exploring the experiences about their perceived autonomy in their transition period from hospital to home. METHOD: Based on the 'grounded theory' approach; in-depth, semi structured interviews were conducted with 11 SCI-patients. A theoretical sampling strategy was used and the data was analysed according to the constant comparative method. RESULTS: To capture this complexity of autonomy, the results have been structured in themes with regard to the self of the patient and his independency. The analysis showed four different typologies of how autonomy is perceived; (1) the active agent, (2) the active follower, (3) the passive follower and (4) the passive victim. CONCLUSION: Rehabilitation professionals can help individuals in disconnecting their internalised ideal of independency to avoid the illusion of being autonomous and can gain insight in the patients' typology and empower patients to avoid the paradox of being autonomous. If the ultimate goal of rehabilitation is participation; empowering the patient to achieve a 'sense of agency' instead of autonomy is the central goal for rehabilitation professionals. Empowerment and agency are key topics for the patient to gain mastery over his own life. | Male | Humans | Patient Participation | Questionnaires | OK |
| Ellen | Peters | Julie T. | Glick | Patricia A. | Robinson | Medical Decision Making: An International Journal of the Society for Medical Decision Making | 2011-02-00 2011 Jan-Feb | BACKGROUND: Eliciting patients' preferences within a framework of shared decision making (SDM) has been advocated as a strategy for increasing colorectal cancer (CRC) screening adherence. Our objective was to assess the effectiveness of a novel decision aid on SDM in the primary care setting. METHODS: An interactive, computer-based decision aid for CRC screening was developed and evaluated within the context of a randomized controlled trial. A total of 665 average-risk patients (mean age, 57 years; 60% female; 63% black, 6% Hispanic) were allocated to 1 of 2 intervention arms (decision aid alone, decision aid plus personalized risk assessment) or a control arm. The interventions were delivered just prior to a scheduled primary care visit. Outcome measures (patient preferences, knowledge, satisfaction with the decision-making process [SDMP], concordance between patient preference and test ordered, and intentions) were evaluated using prestudy/poststudy visit questionnaires and electronic scheduling. RESULTS: Overall, 95% of patients in the intervention arms identified a preferred screening option based on values placed on individual test features. Mean cumulative knowledge, SDMP, and intention scores were significantly higher for both intervention groups compared with the control group. Concordance between patient preference and test ordered was 59%. Patients who preferred colonoscopy were more likely to have a test ordered than those who preferred an alternative option (83% v. 70%; P < 0.01). Intention scores were significantly higher when the test ordered reflected patient preferences. CONCLUSIONS: Our interactive computer-based decision aid facilitates SDM, but overall effectiveness is determined by the extent to which providers comply with patient preferences. | Male | Decision Support Techniques | Humans | Patient Satisfaction | OK |
| Jin Young | Choi | Jina | Kang | Dae Kyun | Kim | Psycho-Oncology | 2013-12-00 Dec 2013 | OBJECTS: We aimed to assess whether awareness of a terminal illness can affect care decision making processes and the achievement of a good death in advanced cancer patients receiving palliative care services. METHODS: Awareness of terminal illness at the time of palliative care service admission was assessed by the health care professionals during the routine initial comprehensive assessment process and was recorded in the national terminal cancer patient registry. A follow-up nationwide bereavement survey was conducted, which contained questions regarding decision making processes and the Korean version of the Good Death Inventory. RESULTS: Among the 345 patients included in the final analysis, the majority (68.4%) of the patients were aware of the terminal illness. Awareness of the terminal illness tended to reduce discordances in care decision making (adjusted odds ratio = 0.55; 95% CI: 0.29-1.07), and increased the patients' own decision making when there were discordances between patients and their families (adjusted odds ratio = 3.79; 95% CI: 1.31-10.94). The Good Death Inventory score was significantly higher among patients who were aware of their terminal illnesses compared with those who were not (5.04 vs. 4.80; p = 0.013) and especially in the domains of 'control over the future' (5.18 vs. 4.04; p < 0.001), 'maintaining hope and pleasure' (4.55 vs. 3.92; p = 0.002), and 'unawareness of death' (4.41 vs. 4.26; p = 0.024). CONCLUSION: Awareness of the terminal illness had beneficial effect on the harmonious decision making, patient autonomy, and patient's quality of death. Disclosure of terminal illness should be encouraged. | Male | Decision Making | Humans | Patient Participation | OK |
| James C. | Diggans | Lyssa | Friedman | Giulia C. | Kennedy | Thyroid: Official Journal of the American Thyroid Association | 2012-10-00 Oct 2012 | BACKGROUND: Seventy-five percent of thyroid nodules with indeterminate fine-needle aspiration (FNA) cytology are found to be benign postoperatively. A novel genomic test, the Afirma gene expression classifier (AGEC), has been available for clinical use in the United States, since late 2010. In 2010, two modest-sized validation studies showed that the AGEC could identify a benign gene expression signature in indeterminate cytology thyroid FNA samples with a negative predictive value >95%. The objective of this study was to evaluate how the AGEC impacted the joint decision of the endocrinologist and patient to operate when FNA cytology was indeterminate, but the AGEC reading of the nodule was benign. METHODS: In this cross-sectional cohort study, data were contributed retrospectively by 51 endocrinologists at 21 practice sites that had previously obtained ≥3 benign AGEC readings in ≥1 cm nodules with indeterminate FNA cytology readings. Information regarding demographic data, nodule size and location, decision to operate, surgery type (hemithyroidectomy [HT] or total thyroidectomy [TT]), and reason for recommending surgery was retrospectively collected. RESULTS: Compared to a 74% previous historical rate of surgery for cytologically indeterminate nodules, the operative rate fell to 7.6% during the period that AGEC were obtained in the clinical practices, a highly significant reduction in the decision to operate (p<0.001). The rate of surgery on cytologically indeterminate nodules that were benign by the AGEC reading did not differ from the historically reported rate of operation on cytologically benign nodules (p=0.41). The four primary reasons reported by the physicians for operating on nodules with a benign AGEC reading, in descending order: large nodule size (46.4%), symptomatic nodules (25.0%), rapidly growing nodules (10.7%), or a second suspicious or malignant nodule in the same patient (10.7%). These reasons are concordant with those typically given for operation on cytologically benign nodules. CONCLUSIONS: In a substantial group of medical practices, obtaining an AGEC test in patients with cytologically indeterminate nodules was associated with a striking reduction in the rate of diagnostic thyroidectomy. Approximately, one surgery was avoided for every two AGEC tests run on thyroid FNAs with indeterminate cytology. | Male | Humans | Patient Participation | Female | OK |
| Miranda Ritterman | Weintraub | Michael J. | Maranda | Suzanne | Elshafey | Patient Education and Counseling | 2012-10-00 Oct 2012 | OBJECTIVE: Decision aids are designed to assist patients in understanding their health care choices but lower SES populations are less activated and may not be prepared to benefit. Activating interventions may help prepare patients for using decision aids. METHODS: We evaluated the impact of a decision aid video (DA) and the Patient Activation Intervention (PAI) on patient's level of activation measured by the Patient Activation Measure (PAM) and their decision-making confidence measured by the decision self-efficacy (DSE) scale. Patients were randomized into control, PAI alone, DA alone, and DA+PAI groups. RESULTS: PAM and DSE scores increased significantly in all groups with repeated measures. Restricting analyses to those with pre-intervention PAM scores at stages 1 or 2, the change in PAM scores was significant only for the intervention groups. The change in DSE scores was significantly only in the DA group. CONCLUSION: These findings provide support for the utility of the DA, the PAI, and the DA+PAI in activating lower SES individuals. The DA alone changed DSE scores in the least activated patients while the PAI and DA both changed PAM scores. PRACTICE IMPLICATIONS: Interventions directed at increasing patient engagement in their care may be useful particularly for less activated patients from lower SES populations. | Male | Decision Making | Decision Support Techniques | Humans | OK |
| Mary Catherine | Beach | Debra L. | Roter | Lisa A. | Cooper | Journal of General Internal Medicine | 2013-11-00 Nov 2013 | BACKGROUND: Low health literacy (HL) is associated with poor healthcare outcomes; mechanisms for these associations remain unclear. OBJECTIVE: To elucidate how HL influences patients' interest in participating in healthcare, medical visit communication, and patient reported visit outcomes. DESIGN, SETTING, AND PATIENTS: Cross-sectional study of enrollment data from a randomized controlled trial of interventions to improve patient adherence to hypertension treatments. Participants were 41 primary care physicians and 275 of their patients. Prior to the enrollment visit, physicians received a minimal intervention or communication skills training and patients received a minimal intervention or a pre-visit coaching session. This resulted in four intervention groups (minimal patient/minimal physician; minimal patient/intensive physician; intensive patient/minimal physician; and intensive patient/intensive physician). MEASUREMENTS: Rapid Estimate of Adult Literacy in Medicine; patients' desire for involvement in decision making; communication behaviors; patient ratings of participatory decision making (PDM), trust, and satisfaction. RESULTS: A lower percentage of patients with low versus adequate literacy had controlled blood pressure. Both groups were similarly interested in participating in medical decision making. Communication behaviors did not differ based on HL except for medical question asking by patients, which was lower among low literacy patients. This was particularly true in the intensive patient /intensive physician group (3.85 vs. 6.42 questions; p = 0.002). Overall, ratings of care didn't differ based on HL; however, in analyses stratified by intervention assignment, patients with low literacy in minimal physician intervention groups reported significantly lower PDM scores than adequate literacy patients. CONCLUSIONS: Patients with low and adequate literacy were similarly interested in participating in medical decision making. However, low literacy patients were less likely to experience PDM in their visits. Low literacy patients in the intensive physician intervention groups asked fewer medical questions. Patients with low literacy may be less able to respond to physicians' use of patient-centered communication approaches than adequate literacy patients. | Male | Humans | Patient Participation | Patient Satisfaction | OK |
|  |  |  |  |  |  | CNS drugs | 2013-08-00 Aug 2013 | Over the past two decades there has been increasing interest in including patients' self-reports in the management of their illness. Among the many reasons for such recent interest has been a rising consumer movement over the past few decades, which has led patients, their caregivers and their families to press for more meaningful sharing with physicians in the clinical decision-making process, with the clear expectations of better therapies and improved outcomes. Patients as consumers of services, their views, attitudes towards healthcare, as well as their level of satisfaction with care, have become increasingly recognized. The recent interest by the US Food and Drug Administration (FDA), as well as other regulatory agencies, in patient-reported outcomes (PROs) in the process of developing and testing new antipsychotics, has also added more impetus. It is clear that including patients in the decision-making process about the management of their psychiatric conditions also broadens the concept of 'recovery', by empowering patients to be active participants and gives a clear message that successful treatment in schizophrenia is more than a symptomatic improvement, but also includes improved functional status. Additionally, the recent interest in personalized medicine puts the patient in the centre of such development. Since 2004, when we published our review about the impact of new antipsychotics on quality of life in CNS Drugs, a number of newer antipsychotics have been introduced and include ziprasidone, aripiprazole, paliperidone, asenapine, iloperidone and lurasidone. The current review is based on 31 selected publications that cover the years 2004-2012, and deals with the impact of such newer antipsychotics on specific domains of PROs, such as subjective tolerability, quality of life, medication preference, satisfaction and social functioning. Most of the available data deal with ziprasidone, aripiprazole and paliperidone. Though the great majority of the studies indicate the newer antipsychotics have favourably impacted on aspects of PROs, such a conclusion can only be considered a trend due to the many design and methodological limitations of many of these studies. It is interesting to note, as the field awaits more rigorous studies, that there seems to be a unifying core that exists among the various subjective outcomes and that tends to generalize from one subjective outcome to other subjective outcomes. The patient who experiences good subjective tolerability to medications tends generally to be more satisfied and has a strong medication preference. The identification of such a unifying core can prove helpful, not only in the development of appropriate scales, but also in informing and guiding the process of development of new antipsychotics. | Decision Making | Humans | Patient Participation | Patient Satisfaction | OK |
| Phyllis | Naragon | Brian | Forrest | Jason | Dees | Patient Education and Counseling | 2012-09-00 Sep 2012 | OBJECTIVE: The objective was to evaluate parallel patient and physician computer-mediated communication skill training on participants' report of skill use and patient satisfaction. METHODS: Separate patient and clinician web-tools comprised of over 500, 10-s video clips demonstrating patient-centered skills in various ways. Four clinician members of the American Academy of Family Physicians National Research Network participated by enrolling 194 patients into a randomized patient trial and 29 physicians into a non-randomized clinician trial of respective interventions. All participants completed baseline and follow-up self-report measures of visit communication and satisfaction. RESULTS: Intervention patients reported using more skills than controls in five of six skill areas, including identification of problems/concerns, information exchange, treatment adherence, shared decision-making and interpersonal rapport (all p<.05); post intervention, physicians reported using more skills in the same 5 areas (all p<.01). Intervention group patients reported higher levels of satisfaction than controls in five of six domains (all p<.05). CONCLUSION: Communication skill training delivered in a computer mediated format had a positive and parallel impact on both patient and clinician reported use of patient-centered communication and in patient satisfaction. PRACTICE IMPLICATIONS: Computer-mediated interventions are cost and time effective thereby increasing patient and clinician willingness to undertake training. | Male | Humans | Patient Satisfaction | Physician-Patient Relations | OK |
| Jennifer A. | Gueguen | Catherine | Diamond | Jennifer | Bianculli | Psycho-Oncology | 2010-06-00 juin 2010 | Objective: The objective of this paper is to report the implementation and assessment of the Comskil Training Curriculum at Memorial Sloan-Kettering Cancer Center. Method: Twenty-eight attending physicians and surgeons participated in communication skills training modules as part of a train-the-trainer program. Doctors were video recorded in clinical consultations with patients two times before training and two times after training, resulting in 112 video recordings for analysis. Recordings were coded using the Comskil Coding System. Results: Communication skills related to two of the six major skill sets, Establishing the Consultation Framework and Checking, increased following training. Limited changes emerged in three skill sets, while one skill set, Shared Decision Making, did not change. Doctors who attended more training modules had higher levels of change. Female participants demonstrated three skills more frequently than males post-training. Conclusions: The intervention produced significant communication skills uptake in a group of experienced attending clinicians, mediated by the amount of training. Future research should focus on the dose of training necessary to achieve skills uptake and the effect of skills training on patient outcomes. (PsycINFO Database Record (c) 2012 APA, all rights reserved). (journal abstract) | physicians | Oncology | communication skills | Neoplasms | Doublon |
| Jennifer A. | Gueguen | Catherine | Diamond | Jennifer | Bianculli | Psycho-Oncology | 2010-06-00 Jun 2010 | OBJECTIVE: The objective of this paper is to report the implementation and assessment of the Comskil Training Curriculum at Memorial Sloan-Kettering Cancer Center. METHOD: Twenty-eight attending physicians and surgeons participated in communication skills training modules as part of a train-the-trainer program. Doctors were video recorded in clinical consultations with patients two times before training and two times after training, resulting in 112 video recordings for analysis. Recordings were coded using the Comskil Coding System. RESULTS: Communication skills related to two of the six major skill sets, Establishing the Consultation Framework and Checking, increased following training. Limited changes emerged in three skill sets, while one skill set, Shared Decision Making, did not change. Doctors who attended more training modules had higher levels of change. Female participants demonstrated three skills more frequently than males post-training. CONCLUSIONS: The intervention produced significant communication skills uptake in a group of experienced attending clinicians, mediated by the amount of training. Future research should focus on the dose of training necessary to achieve skills uptake and the effect of skills training on patient outcomes. | Male | Humans | Physician-Patient Relations | Video Recording | OK |
| Glyn | Elwyn |  |  |  |  | Health Expectations: An International Journal of Public Participation in Health Care & Health Policy | 2011-03-00 mars 2011 | Background or context: Regret is a common consequence of decisions, including those decisions related to individuals' health. Several assessment instruments have been developed that attempt to measure decision regret. However, recent research has highlighted the complexity of regret. Given its relevance to shared decision making, it is important to understand its conceptualization and the instruments used to measure it. Objectives: To review current conceptions of regret. To systematically identify instruments used to measure decision regret and assess whether they capture recent conceptualizations of regret. Search strategy: Five electronic databases were searched in 2008. Search strategies used a combination of MeSH terms (or database equivalent) and free text searching under the following key headings: 'Decision' and 'regret'and 'measurement'. Follow-up manual searches were also performed. Inclusion criteria: Articles were included if they reported the development and psychometric testing of an instrument designed to measure decision regret, or the use of a previously developed and tested instrument. Main results: Thirty-two articles were included: 10 report the development and validation of an instrument that measures decision regret and 22 report the use of a previously developed and tested instrument. Content analysis found that existing instruments for the measurement of regret do not capture current conceptualizations of regret and they do not enable the construct of regret to be measured comprehensively. Conclusions: Existing instrumentation requires further development. There is also a need to clarify the purpose for using regret assessment instruments as this will, and should, focus their future application. (PsycINFO Database Record (c) 2014 APA, all rights reserved). (journal abstract) | Measurement | Decision Making | decision regret complexity | good decisions | Doublon |
| Glyn | Elwyn |  |  |  |  | Health Expectations: An International Journal of Public Participation in Health Care and Health Policy | 2011-03-00 Mar 2011 | BACKGROUND OR CONTEXT: Regret is a common consequence of decisions, including those decisions related to individuals' health. Several assessment instruments have been developed that attempt to measure decision regret. However, recent research has highlighted the complexity of regret. Given its relevance to shared decision making, it is important to understand its conceptualization and the instruments used to measure it. OBJECTIVES: To review current conceptions of regret. To systematically identify instruments used to measure decision regret and assess whether they capture recent conceptualizations of regret. SEARCH STRATEGY: Five electronic databases were searched in 2008. Search strategies used a combination of MeSH terms (or database equivalent) and free text searching under the following key headings: 'Decision' and 'regret' and 'measurement'. Follow-up manual searches were also performed. INCLUSION CRITERIA: Articles were included if they reported the development and psychometric testing of an instrument designed to measure decision regret, or the use of a previously developed and tested instrument. MAIN RESULTS: Thirty-two articles were included: 10 report the development and validation of an instrument that measures decision regret and 22 report the use of a previously developed and tested instrument. Content analysis found that existing instruments for the measurement of regret do not capture current conceptualizations of regret and they do not enable the construct of regret to be measured comprehensively. CONCLUSIONS: Existing instrumentation requires further development. There is also a need to clarify the purpose for using regret assessment instruments as this will, and should, focus their future application. | Decision Making | Emotions | Humans | Patient Participation | OK |
| Elizabeth | Murphy | Anthony | Avery |  | Yellow Card Study Collaboration | British Journal of Clinical Pharmacology | 2011-11-00 Nov 2011 | AIM: To explore the opinions of patient reporters to the UK Yellow Card Scheme (YCS) on the importance of the scheme. METHODS: Postal questionnaires were distributed on our behalf to all patient reporters submitting a Yellow Card to the Medicines and Healthcare Regulatory Agency (MHRA) between March and December 2008, with one follow-up reminder to non-responders. Qualitative analysis was undertaken of responses to an open question asking why respondents felt patient reporting was important. This was followed up by telephone interviews with a purposive sample of selected respondents. RESULTS: There were 1362 evaluable questionnaires returned from 2008 distributed (68%) and 1238 (91%) respondents provided a total of 1802 comments. Twenty-seven interviews were conducted, which supported and expanded the views expressed in the questionnaire. Four main themes emerged, indicating views that the YCS was of importance to pharmacovigilance in general, manufacturers and licensing authorities, patients and the public and health professionals. Reporters viewed the YCS as an important opportunity to describe their experiences for the benefit of others and to contribute to pharmacovigilance. The scheme's independence from health professionals was regarded as important, in part to provide the patient perspective to manufacturers and regulators, but also because of dismissive attitudes and under-reporting by health professionals. CONCLUSION: Direct patient reporting through the YCS is viewed as important by those who have used the scheme, in order to provide the patient experience for the benefit of pharmacovigilance, as an independent perspective from those of health professionals. | Male | Humans | Patient Participation | Questionnaires | OK |
| Maarten P. | de Wit |  |  |  |  | Annals of the Rheumatic Diseases | 2012-07-00 Jul 2012 | | Humans | Patient Satisfaction | Arthritis, Rheumatoid | Outcome Assessment (Health Care) | OK |
| Robert A. C. | Ruiter | Ingrid J. T. | Veldhuizen |  |  | Transfusion | 2013-02-00 Feb 2013 | BACKGROUND: This study investigated the effects of adverse events (i.e., needle reactions, fatigue, and vasovagal reactions) and feelings of distress and anxiety on retention of first-time blood donors. All effects were explored separately for men and women. STUDY DESIGN AND METHODS: First-time blood donors (n = 2438) received a questionnaire, asking them about their experience of adverse events, subjective distress, and anxiety at their first donation. Provision of a second donation was checked approximately 18 months later. After exclusion of nonresponders and donors who did not experience an adverse event, 1278 first-time donors were included in the logistic regression analyses. RESULTS: Nine percent of donors who experienced an adverse event at their first donation did not return for a second donation. Vasovagal reactions decreased retention in both males and females (men-odds ratio [OR], 0.45; 95% CI, 0.23-0.89; women-OR, 0.71; 95% CI, 0.51-0.98). Fatigue decreased retention in males only (OR, 0.62; 95% CI, 0.42-0.91), and subjective distress decreased retention in females only (OR, 0.77; 95% CI, 0.65-0.92). CONCLUSION: In addition to decreasing vasovagal reactions, retention interventions could productively target coping with fatigue and reducing subjective distress after adverse reactions. | Male | Humans | Patient Participation | Questionnaires | OK |
| Ma'rof | Redzuan | Zahid | Emby |  |  | Global Journal of Health Science | 2012-01-00 Jan 2012 | BACKGROUND: Although significant consideration has been devoted to women participation in breast cancer prevention programs, our understanding about the psychosocial factors which influence participation remains incomplete. METHOD: The study applied a quantitative approach based on the cross-sectional survey design and multistage cluster random sampling. A total of 400 women aged 35-69 years, were surveyed at 4 obstetric and gynecologic clinics affiliated to Tehran University of Medical Sciences in Tehran: the participation levels of 86 women who have had a mammogram were analyzed based on their self-efficacy, belief, social influence, and barriers concerning mammography utilization. RESULTS: Consistent with the study framework, in bivariate analysis, the higher level of women's participation in breast cancer prevention programs was significantly related to more positive belief about mammography (p< .05), greater social influence on mammography (p< .01) and fewer barriers to mammography (p< .01). Self efficacy (p= .114) was not significantly related to the higher level of participation. CONCLUSION: Results suggest that women's participation levels in breast cancer prevention programs might be associated with the specific psychosocial factors on breast cancer preventive behavior such as mammography screening. | Humans | Patient Participation | Questionnaires | Female | OK |
| Asmae | Khattabi | I. Ralph | Edwards | Rachida Soulaymani | Bencheikh | The International Journal of Risk & Safety in Medicine | 2011-00-00 2011 | OBJECTIVES: This study assesses the ability of Pharmacovigilance Centres (PVCs) to detect medication errors (ME) and to proceed to building Patient Safety (PS) via their information networks and to underline the limits for this challenge. METHODS: This was an exploratory study conducted in PVCs members of the World Health Organization International Drug Monitoring network. A questionnaire specifically designed for the needs of the study was sent to a network via a confidential email system. The questionnaire asked for information, progress and improvement made by PVCs in PS and ME. RESULTS: Among the 88 countries, 21 answered. Reporting of Adverse Drug Reactions (ADRs) by health care professionals (HCP) is mandatory for 42% of PVCs. 100% of countries receive reports from HCP, 66% from patients and 24% from PCCs. ADRs reports are received by all communications means. There is an heterogeneity between countries regarding PVCs and PS activities. Among them, 4 PVCs have the prime activity of PS organization. CONCLUSION: PVCs are able to detect and analyze ME. There is a need to coordinate efforts between countries to optimize ME detection, and its analysis. Bridges need to be built linking PVCs, PCCs and PS organizations in order to avoid duplication of workload. | Humans | Patient Participation | self report | Adverse Drug Reaction Reporting Systems | OK |
| Marsha | Davis | Kayshin | Chan | Takeia | Horton | Journal of Clinical Epidemiology | 2010-10-00 Oct 2010 | OBJECTIVE: We developed the Clinical Research Involvement Scales (CRIS) to assess the willingness to participate in a clinical trial. STUDY DESIGN AND SETTING: Diverse populations (N=919) aged 18 years or older from Atlanta, Georgia, were included in comprehensive testing of the 41-item CRIS instrument. The formative phase focused on item content for the new measures (n=54). Questionnaires from potential vaccine trial participants (n=865), collected at multiple time points, resulted in the evaluation of scale reliability and validity (i.e., attitudes, behavioral and normative beliefs, perceived social support for clinical research participation, social norm compliance, perceptions of the clinical research organization, and perceived relevance of the research endeavor). RESULTS: Qualitative testing revealed adequate comprehension and content validity of the initial item set. The subjective norms domain (n=3) initially exhibited poor internal consistency in pilot testing (Cronbach's alpha=0.525); yet, rewording of the items resulted in consistently stable measurement improvement (Cronbach's alpha=0.850). Each of the CRIS subscales demonstrated extremely high reliability, ranging from 0.734 to 0.918. Confirmatory factor analysis verified item-factor relationships and determined construct and convergent validity (root mean square error of approximation=0.068; comparative fit index=0.835). CONCLUSIONS: CRIS is a reliable instrument for measuring community attitudes toward participation in biomedical research studies. Results of this study support the use of these scales to recruit diverse populations to clinical trials. | Male | Humans | Patient Participation | Questionnaires | OK |
| Surachet | Roungprakhon | Riccardo | Scarpa |  |  | Vaccine | 2013-08-12 Aug 12, 2013 | This paper estimates the marginal willingness-to-pay for attributes of a hypothetical HIV vaccine using discrete choice modeling. We use primary data from 326 respondents from Bangkok and Chiang Mai, Thailand, in 2008-2009, selected using purposive, venue-based sampling across two strata. Participants completed a structured questionnaire and full rank discrete choice modeling task administered using computer-assisted personal interviewing. The choice experiment was used to rank eight hypothetical HIV vaccine scenarios, with each scenario comprising seven attributes (including cost) each of which had two levels. The data were analyzed in two alternative specifications: (1) best-worst; and (2) full-rank, using logit likelihood functions estimated with custom routines in Gauss matrix programming language. In the full-rank specification, all vaccine attributes are significant predictors of probability of vaccine choice. The biomedical attributes of the hypothetical HIV vaccine (efficacy, absence of VISP, absence of side effects, and duration of effect) are the most important attributes for HIV vaccine choice. On average respondents are more than twice as likely to accept a vaccine with 99% efficacy, than a vaccine with 50% efficacy. This translates to a willingness to pay US$383 more for a high efficacy vaccine compared with the low efficacy vaccine. Knowledge of the relative importance of determinants of HIV vaccine acceptability is important to ensure the success of future vaccination programs. Future acceptability studies of hypothetical HIV vaccines should use more finely grained biomedical attributes, and could also improve the external validity of results by including more levels of the cost attribute. | Male | Humans | Patient Participation | Questionnaires | OK |
| Krishan | Patel | M. | Briggs | Charles A. | Vincent | Journal of Evaluation in Clinical Practice | 2013-10-00 Oct 2013 | BACKGROUND: In recent years, factors that affect patients' willingness and ability to participate in safety-relevant behaviours have been investigated. However, how trained healthcare professionals or medical students would feel participating in safety-relevant behaviours as a patient in hospital remains largely unexplored. OBJECTIVES: To investigate medical students' willingness to participate in behaviours related to the quality and safety of their health care. DESIGN: A cross-sectional exploratory study using a survey that addressed willingness to participate in different behaviours recommended by current patient safety initiatives. Three types of interactional behaviours (asking factual or challenging questions, notifying doctors or nurses of errors/problems) and three non-interactional behaviours (choosing a hospital based on the safety record, bringing medicines and a list of allergies into hospital, and reporting an error to a national reporting system) were assessed. PARTICIPANTS: One hundred and seventy-nine medical students from an inner city London teaching hospital participated in the study. FINDINGS: Students' willingness to participate was affected (P < 0.05) by the action required by the patient and (for interactional behaviours) whether the patient was engaging in the specific action with a doctor or nurse. Students were least willing to ask 'challenging' questions to doctors and nurses and to report errors to a national reporting system. Doctors' and nurses' encouragement appeared to increase self-reported willingness to participate in behaviours where baseline willingness was low. CONCLUSION: Similar to research on lay patient populations; medical students do not view involvement in safety-related behaviours equally. Interventions should be tailored at encouraging students to participate in behaviours they are less inclined to take on an active role in. Future research is required to examine students' motivations for participation in this important but heavily under-researched area. | Male | Humans | Patient Participation | Questionnaires | OK |
| Staffan | Lindblad | Garey | Mazowita | Kelly | McQuillen | The Journal of Ambulatory Care Management | 2012-06-00 2012 Apr-Jun | With the phrase "the medium is the message", Marshall McLuhan argued that technologies are the messages themselves and not just the medium. Almost 50 years later, we understand that modern information and communication technologies expand our ability to perceive our world to an extent that would be impossible without the medium. In this article, we contend that information and communication technologies are becoming the dominant medium for patient engagement. Information and communication technologies will efficiently change patient-reported measurement into much more behaviorally sophisticated information that will create a very different interaction between patients and a new kind of health care workforce. | Decision Making | Humans | Patient Participation | Health Literacy | OK |
|  |  |  |  |  |  | The New England Journal of Medicine | 2010-03-11 Mar 11, 2010 | | Humans | Patient Participation | Questionnaires | United States | OK |
| Lena | Haglund | Renée R. | Taylor |  |  | Scandinavian Journal of Occupational Therapy | 2012-09-00 Sep 2012 | Participation is often the comprehensive objective of treatment but also an indication of the extent to which the process of occupational therapy is client-centred. The purpose of this study was to explore levels of participation during occupational therapy among clients in the area of mental health from the occupational therapists' perspectives. Additionally the authors sought to identify factors that might hinder client participation. Postal questionnaires were sent out to 670 Swedish occupational therapists working with persons with mental illness and learning disabilities. The questionnaire required therapists to rate clients' levels of participation during occupational therapy. Findings indicated that the most common level of participation for the clients was interdependent, meaning that problems, goals, and plans were identified jointly and collaboratively with the occupational therapist. However, more than 20% of the clients were described as being dependent. Almost 90% of the occupational therapists rated client participation in therapy to be very important and nearly 70% claimed that client participation in general needed to be increased. Occupational therapists rated the primary barriers to participation as being clients' inability to participate and organizational and financial problems. Implications of these findings for education in client-centred practice approaches are discussed. | Male | Humans | Patient Participation | Questionnaires | OK |
| Wolfgang | Eich | Christiane | Bieber |  |  | Zeitschrift Für Evidenz, Fortbildung Und Qualität Im Gesundheitswesen | 2012-00-00 2012 | BACKGROUND: Promoting patient involvement in medical decision making has become a desirable goal in medical consultations. Reliable and valid measures are necessary to evaluate interventions designed to promote shared decision making and to understand determinants and associations. The OPTION ("observing patient involvement") scale is the most prominent observation instrument for assessing the extent to which clinicians actively involve patients in decision making. OBJECTIVE: This paper discusses psychometric and methodological characteristics of the OPTION scale. RESULTS: There is little support for the purported unidimensional structure. Although reliabilities are acceptable, results are highly heterogeneous across studies. There is also little evidence concerning validity. In particular, studies mainly failed to support convergent validity. Additional issues pertain to lack of item independence, restriction of range, and failure to consider dyadic aspects. CONCLUSIONS: Given these findings, a number of methodological and conceptual issues still need to be addressed for the effective measurement of patient involvement. Directions for future research are discussed. | Decision Making | Humans | Patient Participation | Physician-Patient Relations | OK |
| Hector | Falcoff | Jean | Bouyer | Virginie | Ringa | Journal of Clinical Epidemiology | 2013-04-00 Apr 2013 | OBJECTIVE: To analyze the participation of the patients in a survey about prevention, according to their own medical (hypertension and inclusion during house call) and social (occupational class and birthplace) characteristics and characteristics related to the organization of their physicians' practice. STUDY DESIGN AND SETTING: Fifty-nine randomly recruited physicians from the Paris metropolitan area enrolled every consecutive patient seen during a 2-week period. Actual patient participation (responding to the telephonic questionnaire) was analyzed with a logistic mixed model separately for male and female patients. RESULTS: The participation rate among 4,106 eligible patients was 66.7% and varied among physicians (from 48.7% to 80.8% for the 10th and 90th percentiles of the distribution). Participation was better for higher occupational classes, patients included during office visits, men with hypertension, and women born in France. After controlling for all patient characteristics, participation was best if the physician saw at least some patients by appointment [odds ratio (OR), 2.12; 95% confidence interval (CI): 1.12, 4.01 for men and OR, 3.38; 95% CI: 1.72, 6.63 for women). This characteristic explained 14% of the variability between physicians for participation by men and 28% of that by women. CONCLUSION: Cluster studies should take the characteristics of the health care providers into account in their design, particularly their practice organization. | Male | Humans | Patient Participation | Questionnaires | OK |
| Francis | Rossignol | Susie | Bernier | Marie-Eve | Poitras | BMC family practice | 2011-00-00 2011 | BACKGROUND: Patient enablement can be defined as the extent to which a patient is capable of understanding and coping with his or her health issues. This concept is linked to a number of health outcomes such as self-management of chronic diseases and quality of life. The Patient Enablement Instrument (PEI) was designed to measure this concept after a medical consultation. The instrument, in its original form and its translations into several languages, has proven to be reliable and valid. The purpose of this study was to evaluate the reliability of the French version of the PEI (PEI-Fv) in a family practice setting. METHODS: One hundred and ten participants were recruited in a family medicine clinic in the Saguenay region of Quebec (Canada). The PEI-Fv was completed twice, immediately after consultation with a physician (T1) and 2 weeks after the consultation (T2). The internal consistency of the tool was assessed with Cronbach's α and test-retest reliability by intraclass correlation coefficient. RESULTS: The mean score for the PEI-Fv was 5.06 ± 3.97 (95% confidence interval [CI]: 4.30-5.81) at T1 and 4.63 ± 3.90 (95% CI: 3.82-5.44) at T2. Cronbach's α was high at T1 (α1 = 0.93; 95% CI: 0.91-0.95) and T2 (α2 = 0.93; 95% CI: 0.91-0.95). The intraclass correlation coefficient was 0.62 (95% CI: 0.48-0.74), indicating a moderate test-retest reliability. CONCLUSIONS: The internal consistency of the PEI-Fv is excellent. Test-retest reliability was moderate to good. Test-retest reliability should be examined in further studies at a less than 2-week interval to reduce maturation bias. This instrument can be used to measure enablement after consultation in a French-speaking family practice setting. | Male | Humans | Patient Participation | Questionnaires | OK |
|  |  |  |  |  |  | Quality of Life Research: An International Journal of Quality of Life Aspects of Treatment, Care and Rehabilitation | 2013-03-00 Mar 2013 | PURPOSE: The objective of this study was to examine whether aspects of the patient-physician relationship for breast cancer patients have an influence on the change in health-related quality of life (HRQOL) after inpatient rehabilitation. METHODS: N = 329 breast cancer patients undergoing inpatient rehabilitation in Germany were surveyed using questionnaires at the beginning of rehabilitation, end of rehabilitation, and 6 months after rehabilitation. Multiple imputations and multilevel models of change were used in the data analyses. RESULTS: Even after comprehensive adjustment for sociodemographic, medical, psychological variables, and center effects, aspects of the physician-patient relationship were statistically and clinically relevant predictors of HRQOL after rehabilitation. Satisfaction with physician's care appears to have a rather short-term effect, but the effect of promoting patient participation can still be partially determined 6 months after rehabilitation. Other important predictors of HRQOL improvement are optimism, higher level of education, higher income, living with a partner, and the ability to work. CONCLUSIONS: By taking into consideration the patient's communication and participation needs, physicians can contribute to an improved HRQOL after rehabilitation. The high predictive power of socioeconomic factors shows that rehabilitation care can be more effective if it accounts for the specific situation of socially disadvantaged individuals. | Humans | Patient Participation | Patient Satisfaction | Physician-Patient Relations | OK |
| Karina | Mill | Margo S. | Rowan | Clare | Liddy | BMC family practice | 2012-00-00 2012 | BACKGROUND: Given the potential value of self-management support programs for people with chronic diseases, it is vital to understand how they influence participants' health attitudes and behaviours. The Stanford Chronic Disease Self-Management Program (CDSMP), the most well-known and widely studied such program, is funded in many provinces and jurisdictions throughout Canada. However, there is little published evidence on its impact in the Canadian health-care system. We studied participants' reactions and perceived impacts of attending the Stanford program in one Ontario health region so we could assess its value to the health region. The study asked: What are participants' reactions and perceived impacts of attending the Stanford CDSMP? METHODS: This mixed methods exploratory study held four focus groups approximately one year after participants attended a Stanford program workshop. At the beginning of each session, participants filled out a survey on the type and frequency of community and health resources used for their self-management. During the sessions, a moderator guided the discussion, asking about such things as long-term impact of the program on their lives and barriers to self-management of their chronic conditions. RESULTS: Participants perceived diverse effects of the workshop: from having a profound impact on one area to affecting all aspects of their lives. A change in physical activity patterns was the most prominent behaviour change, noted by over half the participants. Other recurrent effects included an improved sense of social connection and better coping skills. Barriers to self-management were experienced by almost all participants with several dominant themes emerging including problems with the health system and patient-physician interaction. Participants reported a wide variety of resources used in their self-management, and in some cases, an increase in use was noted for some resources. CONCLUSIONS: Self-management support is, at its core, a complex and patient-centred concept, so a diversity of outcomes to match the diversity of participants should be expected. As these interventions move into different target populations and communities, it is essential that we continue to explore through multiple research methods, the effects, and their meaning to participants, ensuring the optimal investment of resources for the very individuals these interventions aim to serve. | Male | Humans | Patient Participation | Questionnaires | OK |
|  |  |  |  |  |  | Pediatric Physical Therapy: The Official Publication of the Section on Pediatrics of the American Physical Therapy Association | 2012-00-00 2012 | PURPOSE: The purposes of this article were to discuss participation relative to the International Classification of Functioning, Disability, and Health for Children and Youth (ICF-CY) model, evaluate 3 current pediatric participation measures that have been designed to assess participation, and review research findings relevant to participation in children and adolescents with disabilities. KEY POINTS: The concept of participation has been challenging to define and measure. Successful participation of children and adolescents with disabilities in daily life activities is influenced by individual, family, and environmental factors. Currently available tools designed to measure participation of children and adolescents have strengths and limitations, which should be considered when selecting tools for use in clinical practice. CONCLUSION: Pediatric physical therapists can increase participation of children and adolescents with disabilities by measuring and promoting meaningful outcomes in the individual, family, and environmental components of the ICF-CY. | Humans | Patient Participation | Adolescent | Child | OK |
|  |  |  |  |  |  | Scandinavian Journal of Occupational Therapy | 2012-11-00 Nov 2012 | OBJECTIVE: to evaluate the test-retest reliability of children's perceptions of their own competence in performing daily tasks and of their choice of goals for intervention using the Swedish version of the perceived efficacy and goal setting system (PEGS). A second aim was to evaluate agreement between children's and parents' perceptions of the child's competence and choices of intervention goals. METHODS: Forty-four children with disabilities and their parents completed the Swedish version of the PEGS. Thirty-six of the children completed a retest session allocated into one of two groups: (A) for evaluation of perceived competence and (B) for evaluation of choice of goals. Cohen's kappa, weighted kappa and absolute agreement were calculated. RESULTS: Test-retest reliability for children's perceived competence showed good agreement for the dichotomized scale of competent/non-competent performance; however, using the four-point scale the agreement varied. The children's own goals were relatively stable over time; 78% had an absolute agreement ranging from 50% to 100%. There was poor agreement between the children's and their parents' ratings. Goals identified by the children differed from those identified by their parents, with 48% of the children having no goals identical to those chosen by their parents. These results indicate that the Swedish version of the PEGS produces reliable outcomes comparable to the original version. | Male | Humans | Patient Participation | Questionnaires | OK |
| Gabriella | Calvi | Claudia | Goss |  |  | Patient Education and Counseling | 2013-04-00 Apr 2013 | OBJECTIVE: To test for differences between experts and lay people in assessment of the degree to which a doctor engaged in a shared decision making (SDM) with a patient using the OPTION scale and a movie clip as stimulus material. METHODS: A segment of the movie 'Wit', depicting the communication of the diagnosis and the therapy proposed of a cancer, was shown to (a) university students with no knowledge about doctor-patient communication; (b) nurses working in medicine departments; (c) advanced medical students; (d) hospital physicians. The participants were asked to complete the OPTION scale which measures the extent to which physicians involve patients in medical decisions. An analysis of variance was used to compare OPTION scores across the four groups and to compare males and females. RESULTS: Being female [F(1,190)=11.9; p<.001] and being familiar with medical issues [F(3,190)=11.09; p<.001] were both significantly associated with a negative evaluations of the doctor's ability to involve the patient in the SDM. CONCLUSION: Lay people and males (including male experts), are less demanding regarding SDM abilities. PRACTICE IMPLICATIONS: A more systematic use of videos and the OPTION scale as validated outcome measure could be helpful educational strategy for the teaching of SDM. | Male | Humans | Questionnaires | Choice Behavior | OK |
| Cynthia | Goh | Chung Pheng | Alethea Yee |  |  | Palliative & Supportive Care | 2013-02-00 Feb 2013 | BACKGROUND: The traditional relationship between patient and physician in East Asian society has often been described as "paternalistic." However, in an increasingly Westernized world, our knowledge of how patients perceive the role of the physician in their decision making regarding treatment is lacking. OBJECTIVE: This article is part of a larger pilot study exploring the patient-physician dynamic on decision making among Southeast Asian palliative cancer patients. We explore: (1) influence of physicians, (2) the effect of symptom control and quality of life, and (3) dynamics and communication of physicians. DESIGN: An interviewer-administered questionnaire was distributed, with 18 questions related to physician-patient interactions asked. Most questions followed a three point scale: "agree," "neutral," and "disagree," and spontaneous answers beyond this framework were recorded. SETTING/PARTICIPANTS: Thirty patients from the palliative care service were interviewed, including inpatients at Singapore General Hospital and those attending outpatient clinics at the National Cancer Centre. RESULTS: Patients said that they themselves and their physicians were the main influences (80% each), over family members (48.3%). Some patients (26.7%) felt that symptoms were not well controlled, and 42.9% identified low mood or anxiety. Some patients (44.8%) felt that their condition had an effect on decision making. Most patients (89.3%) had a good relationship with the staff, with >80% being comfortable with discussions held. However, 20.7% of patients felt dissatisfied with the information provided, and 62.1% of patients wanted full disclosure of information. SIGNIFICANCE OF RESULTS: Patients appeared to place highest regard in both autonomy and physician input in making decisions, accompanied by an increased desire for more information. These reflect deviation away from traditional thinking of paternalistic doctoring in East Asia. | Male | Decision Making | Humans | Patient Participation | OK |
| Jari | Haukka | Mark | Taylor | Maxine X. | Patel | Schizophrenia Research | 2011-09-00 septembre 2011 | Comments on an article by C. Leucht et al. (see record [rid]2011-01677-001[/rid]). The authors which found that antipsychotic depots, compared to oral antipsychotics, significantly reduced relapses. Methodological strengths include restricting the analysis to randomized controlled trials (RCTs) of ≥1 year in duration and excluding inpatient studies. Both factors reflect how depots are used in clinical practice and increase the generalizability of the results. Methodologically the weakest type of observational study is the mirror-image (‘before–after’) study. These usually calculate total inpatient days on depot versus total inpatient days during an equal duration period on oral medication, immediately preceding the commencement of depot, for each patient. In these cases potential differences in efficacy of the individual drugs, rather than the mode of administration, may affect the results, a problem acknowledged by the authors as applying to half the RCTs in their meta-analysis. The current evidence, from both RCTs and observational studies, is limited and subject to methodological weaknesses and further research is necessary before a definitive view on the comparative efficacy of depot and oral antipsychotics can be reached. It is important to emphasize that depots are unlikely to improve adherence and outcomes unless their use results from a shared decision making process involving the patient and clinician. (PsycINFO Database Record (c) 2012 APA, all rights reserved) | Treatment Compliance | schizophrenia | Clinical Trials | antipsychotic drugs | OK |
| R. Annemiek | Punter | Maarten J. | Ijzerman |  |  | Journal of Rehabilitation Medicine | 2010-06-00 Jun 2010 | OBJECTIVE: Shared decision-making and the use of decision aids are increasingly promoted in various healthcare settings. The extent of their current use and potential in rehabilitation medicine is unknown. The aim of the present study was to explore the barriers to and facilitators of shared decision-making and use of decision aids in daily practice, and to explore the perceptions of physical and rehabilitation medicine (PRM) physicians toward them. METHODS: A cross-sectional survey of 408 PRM physicians was performed (response rate 31%). RESULTS: PRM physicians expressed the highest levels of comfort with shared decision-making as opposed to paternalistic and informed decision-making. The majority reported that shared decision-making constituted their usual approach. The most important barriers to shared decision-making were cases in which the patient received conflicting recommendations and when the patient had difficulty accepting the disease. Key facilitators were the patient's trust in the PRM physician and the patient being knowledgeable about the disease and about treatment options. PRM physicians' attitudes towards the use of decision aids to inform patients were moderately positive. CONCLUSION: Shared decision-making appears to have great potential in the rehabilitation setting. Increasing the use of decision aids may contribute to the further implementation of shared decision-making. | Male | Decision Making | Decision Support Techniques | Humans | OK |
| Kristian | Pollock | Angie | Smith | Janet | Betinis | Nurse Education Today | 2010-01-00 Jan 2010 | This paper reports on a participatory action research project focusing upon service-user involvement in the assessment of student mental health nurses in practice. The principle of student nurses being assessed by service-users was found to be desirable in theory. However, its implementation proved to be fraught with difficulty especially with regard to the concerns about increased workload and feelings of vulnerability amongst students. It is recommended that the model of assessment should be adjusted to that of review and that a program of service-users reviewing student nurses on placement be implemented and evaluated. | Humans | Patient Participation | Mental Disorders | mental health services | OK |
| Thom | Walsh | Stuart W. | Grande | Elissa M. | Ozanne | Journal of Medical Internet Research | 2014-01-00 janvier 2014 | Background: Patient-centered health care is a central component of current health policy agendas. Shared decision making (SDM) is considered to be the pinnacle of patient engagement and methods to promote this are becoming commonplace. However, the measurement of SDM continues to prove challenging. Reviews have highlighted the need for a patient-reported measure of SDM that is practical, valid, and reliable to assist implementation efforts. In consultation with patients, we developed CollaboRATE, a 3-item measure of the SDM process. Objective: There is a need for scalable patient-reported measure of the SDM process. In the current project, we assessed the psychometric properties of CollaboRATE. Methods: A representative sample of the US population were recruited online and were randomly allocated to view 1 of 6 simulated doctor-patient encounters in January 2013. Three dimensions of SDM were manipulated in the encounters: (1) explanation of the health issue, (2) elicitation of patient preferences, and (3) integration of patient preferences. Participants then completed CollaboRATE (possible scores 0-100) in addition to 2 other patient-reported measures of SDM: the 9-item Shared Decision Decision Making Questionnaire (SDM-Q-9) and the Doctor Facilitation subscale of the Patient’s Perceived Involvement in Care Scale (PICS). A subsample of participants was resurveyed between 7 and 14 days after the initial survey. We assessed CollaboRATE’s discriminative, concurrent, and divergent validity, intrarater reliability, and sensitivity to change. Results: The final sample consisted of 1341 participants. CollaboRATE demonstrated discriminative validity, with a significant increase in CollaboRATE score as the number of core dimensions of SDM increased from zero (mean score: 46.0, 95% CI 42.4-49.6) to 3 (mean score 85.8, 95% CI 83.2-88.4). CollaboRATE also demonstrated concurrent validity with other measures of SDM, excellent intrarater reliability, and sensitivity to change; however, divergent validity was not demonstrated. Conclusions: The fast and frugal nature of CollaboRATE lends itself to routine clinical use. Further assessment of CollaboRATE in real-world settings is required. (PsycINFO Database Record (c) 2014 APA, all rights reserved). (journal abstract) | Decision Making | Psychometrics | CollaboRATE | psychometric properties | Doublon |
| Thom | Walsh | Stuart W. | Grande | Elissa M. | Ozanne | Journal of Medical Internet Research | 2014-00-00 2014 | BACKGROUND: Patient-centered health care is a central component of current health policy agendas. Shared decision making (SDM) is considered to be the pinnacle of patient engagement and methods to promote this are becoming commonplace. However, the measurement of SDM continues to prove challenging. Reviews have highlighted the need for a patient-reported measure of SDM that is practical, valid, and reliable to assist implementation efforts. In consultation with patients, we developed CollaboRATE, a 3-item measure of the SDM process. OBJECTIVE: There is a need for scalable patient-reported measure of the SDM process. In the current project, we assessed the psychometric properties of CollaboRATE. METHODS: A representative sample of the US population were recruited online and were randomly allocated to view 1 of 6 simulated doctor-patient encounters in January 2013. Three dimensions of SDM were manipulated in the encounters: (1) explanation of the health issue, (2) elicitation of patient preferences, and (3) integration of patient preferences. Participants then completed CollaboRATE (possible scores 0-100) in addition to 2 other patient-reported measures of SDM: the 9-item Shared Decision Decision Making Questionnaire (SDM-Q-9) and the Doctor Facilitation subscale of the Patient's Perceived Involvement in Care Scale (PICS). A subsample of participants was resurveyed between 7 and 14 days after the initial survey. We assessed CollaboRATE's discriminative, concurrent, and divergent validity, intrarater reliability, and sensitivity to change. RESULTS: The final sample consisted of 1341 participants. CollaboRATE demonstrated discriminative validity, with a significant increase in CollaboRATE score as the number of core dimensions of SDM increased from zero (mean score: 46.0, 95% CI 42.4-49.6) to 3 (mean score 85.8, 95% CI 83.2-88.4). CollaboRATE also demonstrated concurrent validity with other measures of SDM, excellent intrarater reliability, and sensitivity to change; however, divergent validity was not demonstrated. CONCLUSIONS: The fast and frugal nature of CollaboRATE lends itself to routine clinical use. Further assessment of CollaboRATE in real-world settings is required. | Male | Decision Making | Humans | Patient Participation | OK |
| Bożena | Mroczek | Anna | Hans-Wytrychowska | Aneta | Nitsch-Osuch | Advances in Clinical and Experimental Medicine: Official Organ Wroclaw Medical University | 2013-08-00 2013 Jul-Aug | BACKGROUND: Patients with at least one chronic disease requiring regular contact with their GP, additional tests and systematic use of medicines constitute one of the challenges for the future of primary medical care. To date, no studies have been published describing the most important factors in increasing the quality of care for the chronically ill. OBJECTIVES: To evaluate correlations between patients' characteristics and their assessments of the quality of health care and to identify the primary factors influencing the quality of care for chronically ill patients. MATERIAL AND METHODS: The sample for the current analysis included 232 chronically ill adult patients of primary care clinics in Poland. The patients' opinions regarding subjective and objective examinations, their participation in clinical decision-making processes, psychosocial factors, contact with the doctor in emergency situations and confidence in the doctor were investigated. RESULTS: The strongest correlations defining the quality of care for the chronically ill were identified as the occurrence of hypertension (0.248, p < 0.001), the patient's gender (0.271, p < 0.001), the patient's level of education (0.169, p < 0.01) and the patient's age (0.155, p = 0.018). The results of the ANOVA test were statistically significant (p < 0.05) for the occurrence of myocardial infarction, hypertension, type 2 diabetes, psoriasis, multiple sclerosis, the doctor's gender, the patient's gender, age and education. CONCLUSIONS: The areas that the participants gave the highest ratings to are subjective and objective examinations, kindness and willingness to help. The strongest correlations between high assessments of the quality of care provided for chronically ill patients at primary care clinics were observed in the following variables: gender, age, level of education, frequency of visits, therapy conducted by a doctor at a specialist clinic, arterial hypertension, COPD and/or myocardial infarction. | Male | Humans | Patient Participation | Patient Satisfaction | Doublon |
| Bożena | Mroczek | Anna | Hans-Wytrychowska | Aneta | Nitsch-Osuch | Advances in Clinical and Experimental Medicine: Official Organ Wroclaw Medical University | 2013-08-00 2013 Jul-Aug | BACKGROUND: Patients with at least one chronic disease requiring regular contact with their GP, additional tests and systematic use of medicines constitute one of the challenges for the future of primary medical care. To date, no studies have been published describing the most important factors in increasing the quality of care for the chronically ill. OBJECTIVES: To evaluate correlations between patients' characteristics and their assessments of the quality of health care and to identify the primary factors influencing the quality of care for chronically ill patients. MATERIAL AND METHODS: The sample for the current analysis included 232 chronically ill adult patients of primary care clinics in Poland. The patients' opinions regarding subjective and objective examinations, their participation in clinical decision-making processes, psychosocial factors, contact with the doctor in emergency situations and confidence in the doctor were investigated. RESULTS: The strongest correlations defining the quality of care for the chronically ill were identified as the occurrence of hypertension (0.248, p < 0.001), the patient's gender (0.271, p < 0.001), the patient's level of education (0.169, p < 0.01) and the patient's age (0.155, p = 0.018). The results of the ANOVA test were statistically significant (p < 0.05) for the occurrence of myocardial infarction, hypertension, type 2 diabetes, psoriasis, multiple sclerosis, the doctor's gender, the patient's gender, age and education. CONCLUSIONS: The areas that the participants gave the highest ratings to are subjective and objective examinations, kindness and willingness to help. The strongest correlations between high assessments of the quality of care provided for chronically ill patients at primary care clinics were observed in the following variables: gender, age, level of education, frequency of visits, therapy conducted by a doctor at a specialist clinic, arterial hypertension, COPD and/or myocardial infarction. | Male | Humans | Patient Participation | Patient Satisfaction | OK |
| Holger | Pfaff | Christian | Janssen |  |  | International Journal of Public Health | 2011-06-00 Jun 2011 | OBJECTIVES: Empirical studies have confirmed that a trusting physician-patient interaction promotes patient satisfaction, adherence to treatment and improved health outcomes. The objective of this analysis was to investigate the relationship between social support, shared decision-making and inpatient's trust in physicians in a hospital setting. METHODS: A written questionnaire was completed by 2,197 patients who were treated in the year 2000 in six hospitals in Germany. Logistic regression was performed with a dichotomized index for patient's trust in physicians. RESULTS: The logistic regression model identified significant relationships (p < 0.05) in terms of emotional support (standardized effect coefficient [sc], 3.65), informational support (sc, 1.70), shared decision-making (sc, 1.40), age (sc, 1.14), socioeconomic status (sc, 1.15) and gender (sc, 1.15). We found no significant relationship between 'tendency to excuse' and trust. The last regression model accounted for 49.1% of Nagelkerke's R-square. CONCLUSIONS: Insufficient physician communication skills can lead to extensive negative effects on the trust of patients in their physicians. Thus, it becomes clear that medical support requires not only biomedical, but also psychosocial skills. | Male | Decision Making | Humans | Patient Participation | OK |
|  |  |  |  |  |  | Patient Education and Counseling | 2011-09-00 Sep 2011 | OBJECTIVE: To examine whether patient participation in medical consultations have differing effects on self-efficacy and diabetes control by the level of patient communicative health literacy (CHL). METHODS: Participants were 143 outpatients with type 2 diabetes at a university-affiliated hospital. Patient CHL was measured using a newly developed self-rated scale of health literacy. Patient perceived participation in medical consultations and self-efficacy of diabetes self-care were assessed using the self-reported questionnaire. Patient clinical characteristics were obtained from electronic medical records. RESULTS: Both patient CHL and perceived participation were related to greater self-efficacy and decreased HbA1c at the 3-month follow-up. Patient CHL had a moderating effect on the relationship between perceived participation and self-efficacy. Patients with lower CHL reported greater self-efficacy when they actively participated in patient-physician communication, whereas this relationship was less evident among patients with higher CHL. CONCLUSIONS: The examination of patient CHL levels may provide a better understanding of the potential barriers to patients' self-management of disease. PRACTICE IMPLICATIONS: The benefit of active participation may be greater among patients with lower CHL who are likely to have greater difficulties in communicating with the physician, yet tend to rely on the physician as the sole source of health information. | Male | Humans | Patient Participation | Physician-Patient Relations | OK |
| David | Mellor | Weimin | Yang | Marita | McCabe | Social Science & Medicine | 2012-09-00 septembre 2012 | To explore and compare attitudes of consumers (patients and their family members) and medical staff toward clinical trials related to mental health in China, we developed two questionnaires for medical staff and patients and their family members. Approximately 66.2% of medical staff who had no research experience believed that patients could be persuaded to participate in clinical trials, but the percentage of consumers who believed so was just 12.5%. Both groups agreed that written informed consent was required; however, more medical staff than patients agreed that such consent could be provided by patients or their guardian (88.4% vs. 71.4%). Only 9.5% of medical staff thought that patient treatment would be compromised by refusal to participate; the proportion of consumers who thought the same was 29.4%. Great differences exist between medical staff and consumers’ attitudes and beliefs regarding clinical trials. Medical staff were more likely to have a favorable attitude toward their patients participating in clinical trials and considered that informed consent could be provided by guardians rather than the patient. (PsycINFO Database Record (c) 2012 APA, all rights reserved). (journal abstract) | Patient Participation | Client Participation | Mental health | psychiatric patients | Doublon |
| David | Mellor | Weimin | Yang | Marita | McCabe | Social Science & Medicine (1982) | 2012-09-00 Sep 2012 | To explore and compare attitudes of consumers (patients and their family members) and medical staff toward clinical trials related to mental health in China, we developed two questionnaires for medical staff and patients and their family members. Approximately 66.2% of medical staff who had no research experience believed that patients could be persuaded to participate in clinical trials, but the percentage of consumers who believed so was just 12.5%. Both groups agreed that written informed consent was required; however, more medical staff than patients agreed that such consent could be provided by patients or their guardian (88.4% vs. 71.4%). Only 9.5% of medical staff thought that patient treatment would be compromised by refusal to participate; the proportion of consumers who thought the same was 29.4%. Great differences exist between medical staff and consumers' attitudes and beliefs regarding clinical trials. Medical staff were more likely to have a favorable attitude toward their patients participating in clinical trials and considered that informed consent could be provided by guardians rather than the patient. | Humans | Patient Participation | Questionnaires | Mental Disorders | OK |
| Michael L. | Millenson |  |  |  |  | Israel Journal of Health Policy Research | 2013-00-00 2013 | Patient-centered care has become an increasing priority in the United States and plays a prominent role in recent healthcare reforms. One way the country has managed to advance patient-centered care is through establishment of a family of national patient experience surveys (the Consumer Assessment of Healthcare Providers and Systems Plans (CAHPS). CAHPS is publicly reported for several types of providers and was recently tied to hospital reimbursement. This is part of a trend over the last two decades that has shifted provider-patient relationships from a traditional paternal approach to customer service and then to clinical partnership. The health care system in Israel, however, is still struggling to overcome barriers to change in this area. While community based biannual patient experience surveys are conducted by the Myers-JDC-Brookdale Institute, there is no comprehensive national approach to measuring the patient experience across a broad range of settings. Only recently did the Israeli Ministry of Health take its first steps to include patient experience as a dimension of health care quality.In its current position, Israel should learn from the U.S. experience with policies promoting patient-centered care, and specifically the impact on clinical services of measuring the patient experience. Looking at what has happened in the United States, we suggest three main lessons. First, there is a need for a set of national patient experience surveys that would be publicly reported and eventually tied to provider reimbursement. Secondly, the national survey tools should be customized to the unique characteristics of Israeli society and draw from recent research on patient-centeredness to include new and important domains such as patient activation and shared decision-making. Finally, newer technological approaches should be explored with the aim of increasing response rates and the timeliness and usefulness of the surveys. | | | | | Doublon |
| Michael L. | Millenson |  |  |  |  | Israel Journal of Health Policy Research | 2013-00-00 2013 | Patient-centered care has become an increasing priority in the United States and plays a prominent role in recent healthcare reforms. One way the country has managed to advance patient-centered care is through establishment of a family of national patient experience surveys (the Consumer Assessment of Healthcare Providers and Systems Plans (CAHPS). CAHPS is publicly reported for several types of providers and was recently tied to hospital reimbursement. This is part of a trend over the last two decades that has shifted provider-patient relationships from a traditional paternal approach to customer service and then to clinical partnership. The health care system in Israel, however, is still struggling to overcome barriers to change in this area. While community based biannual patient experience surveys are conducted by the Myers-JDC-Brookdale Institute, there is no comprehensive national approach to measuring the patient experience across a broad range of settings. Only recently did the Israeli Ministry of Health take its first steps to include patient experience as a dimension of health care quality.In its current position, Israel should learn from the U.S. experience with policies promoting patient-centered care, and specifically the impact on clinical services of measuring the patient experience. Looking at what has happened in the United States, we suggest three main lessons. First, there is a need for a set of national patient experience surveys that would be publicly reported and eventually tied to provider reimbursement. Secondly, the national survey tools should be customized to the unique characteristics of Israeli society and draw from recent research on patient-centeredness to include new and important domains such as patient activation and shared decision-making. Finally, newer technological approaches should be explored with the aim of increasing response rates and the timeliness and usefulness of the surveys. | | | | | OK |
| Stefan | Hacker | Andrea | Schopper | Claus G. | Krenn | BMC medical ethics | 2010-00-00 2010 | BACKGROUND: Currently, intensive care medicine strives to define a generally accepted way of dealing with end-of-life decisions, therapy limitation and therapy discontinuation.In 2006 a new advance directive legislation was enacted in Austria. Patients may now document their personal views regarding extension of treatment. The aim of this survey was to explore Austrian intensive care physicians' experiences with and their acceptance of the new advance directive legislation two years after enactment (2008). METHODS: Under the aegis of the OEGARI (Austrian Society of Anaesthesiology, Resuscitation and Intensive Care) an anonymised questionnaire was sent to the medical directors of all intensive care units in Austria. The questions focused on the physicians' experiences regarding advance directives and their level of knowledge about the underlying legislation. RESULTS: There were 241 questionnaires sent and 139 were turned, which was a response rate of 58%. About one third of the responders reported having had no experience with advance directives and only 9 directors of intensive care units had dealt with more than 10 advance directives in the previous two years. Life-supporting measures, resuscitation, and mechanical ventilation were the predominantly refused therapies, wishes were mainly expressed concerning pain therapy. CONCLUSION: A response rate of almost 60% proves the great interest of intensive care professionals in making patient-oriented end-of-life decisions. However, as long as patients do not make use of their right of co-determination, the enactment of the new law can be considered only a first important step forward. | Decision Making | Humans | Patient Participation | Questionnaires | OK |
|  |  |  |  |  |  | Oncology Nursing Forum | 2011-01-00 Jan 2011 | PURPOSE/OBJECTIVES: to assess the role of education sources and objective risk status on knowledge and practice of lymphedema risk-minimization behaviors among women recently diagnosed with breast cancer. RESEARCH APPROACH: prospective survey. SETTING: a hospital in Sydney, Australia. PARTICIPANTS: 106 women recently diagnosed with breast cancer at increased risk for developing lymphedema following lymph node dissection. METHODOLOGIC APPROACH: a questionnaire administered at the time of surgery and three months after surgery measured demographics, lymphedema knowledge, lymphedema information sources used, and adherence to risk-minimization recommendations. MAIN RESEARCH VARIABLES: lymphedema knowledge, source of information used, objective lymphedema risk, and adherence to risk-minimization behaviors. FINDINGS: knowledge was high and increased over time. Lymphedema information from the clinic (e.g., brochures, nursing staff) was the most cited source. Adherence to recommendations was moderate; nonadherence was mostly for behaviors requiring regular enactment. Regression analysis revealed that only receipt of information from nursing staff and lymphedema knowledge three months after surgery were significant predictors of risk-minimization behaviors. CONCLUSIONS: exposing women to lymphedema risk information at the time of breast cancer diagnosis facilitates increased awareness and enactment of risk-minimization behaviors. Nursing staff play a key role in disseminating this information and in convincing women to perform the recommendations. INTERPRETATION: provision of lymphedema education by breast clinic staff is critical to ensure that women realize the importance of early detection and treatment. Reminder booster sessions by nursing staff may be beneficial particularly for longer-term knowledge retention and adherence to recommended behaviors. | Humans | Patient Participation | Questionnaires | Female | OK |
| Edel | Murray | Todd | Allen | Peter | Jones | BMC nursing | 2014-00-00 2014 | BACKGROUND: Pressure injury prevention (PIP) is an important area of patient safety. Encouraging patient participation in care is a growing trend in healthcare as it can increase adherence to treatment plans and improve outcomes. Patients in acute care settings may be able to take on an active role in PIP. However, there is limited information on patients' views of their perceived role in PIP. The aims of our study were to survey hospitalised patients' views on a) their perceived roles in PIP and, b) factors that enable or inhibit patient participation in PIP strategies. METHODS: Eligible participants were 18 years of age or older, from a neurology or orthopaedic ward and had been admitted to hospital at least 24 hours prior to enrolment in the study. A questionnaire comprising of fixed and open-ended responses was administered by researchers to participants. Numerical data was analysed descriptively and free-text comments were content-analysed and grouped into themes. RESULTS: The mean age of participants (n = 51) was 65 years (sd = 16.6); over half were female and three quarters were orthopaedic surgical patients. Eighty-six per cent of participants understood the concept of pressure injury and 80% agreed that patients have a role in PIP. Participants nominated the following PIP strategies that could be undertaken by patients: Keep skin healthy; Listen to your body and Looking after the inside. Strategies required for patient participation in PIP were represented by three themes: Manage pain and discomfort; Work together; Ongoing PI education. CONCLUSION: To ensure successful participation in PIP, patients require education throughout admission, management of pain and discomfort and a supportive and collaborative relationship with health care staff. Health professionals should identify patient ability and motivation to prevent pressure injury (PI), work in partnership with patients to adhere to PIP, and ensure that PIP actions are facilitated with appropriate pain relief. | | | | | OK |
| Fiona | Stevenson | Elizabeth | Murray |  |  | Journal of Medical Internet Research | 2013-00-00 2013 | BACKGROUND: Incidence of sexually transmitted infections (STIs) among young people in the United Kingdom is increasing. The Internet can be a suitable medium for delivery of sexual health information and sexual health promotion, given its high usage among young people, its potential for creating a sense of anonymity, and ease of access. Online randomized controlled trials (RCTs) are increasingly being used to evaluate online interventions, but while there are many advantages to online methodologies, they can be associated with a number of problems, including poor engagement with online interventions, poor trial retention, and concerns about the validity of data collected through self-report online. We conducted an online feasibility trial that tested the effects of the Sexunzipped website for sexual health compared to an information-only website. This study reports on a qualitative evaluation of the trial procedures, describing participants' experiences and views of the Sexunzipped online trial including methods of recruitment, incentives, methods of contact, and sexual health outcome measurement. OBJECTIVE: Our goal was to determine participants' views of the acceptability and validity of the online trial methodology used in the pilot RCT of the Sexunzipped intervention. METHODS: We used three qualitative data sources to assess the acceptability and validity of the online pilot RCT methodology: (1) individual interviews with 22 participants from the pilot RCT, (2) 133 emails received by the trial coordinator from trial participants, and (3) 217 free-text comments from the baseline and follow-up questionnaires. Interviews were audio-recorded and transcribed verbatim. An iterative, thematic analysis of all three data sources was conducted to identify common themes related to the acceptability and feasibility of the online trial methodology. RESULTS: Interview participants found the trial design, including online recruitment via Facebook, online registration, email communication with the researchers, and online completion of sexual health questionnaires to be highly acceptable and preferable to traditional methods. Incentives might assist in recruiting those who would not otherwise participate. Participants generally enjoyed taking part in sexual health research online and found the questionnaire itself thought-provoking. Completing the sexual health questionnaires online encouraged honesty in responding that might not be achieved with other methods. The majority of interview participants also thought that receiving and returning a urine sample for chlamydia testing via post was acceptable. CONCLUSIONS: These findings provide strong support for the use of online research methods for sexual health research, emphasizing the importance of careful planning and execution of all trial procedures including recruitment, respondent validation, trial related communication, and methods to maximize follow-up. Our findings suggest that sexual health outcome measurement might encourage reflection on current behavior, sometimes leading to behavior change. TRIAL REGISTRATION: International Standard Randomized Controlled Trial Number (ISRCTN): 55651027; http://www.controlled-trials.com/isrctn/pf/55651027 (Archived by WebCite at http://www.webcitation.org/6LbkxdPKf). | Male | Humans | Patient Participation | Questionnaires | OK |
| Hilde | Grimstad | Aslak | Steinsbekk |  |  | BMC health services research | 2012-00-00 2012 | BACKGROUND: The main aim was to investigate the effect of using two brief feedback scales in mental health out-patient treatment six weeks after starting treatment, compared to treatment as usual. Hypotheses were that use of feedback scales would improve treatment alliance and patient satisfaction. METHODS: An open parallel-group randomised controlled trial was conducted in an out-patient unit in a mental health hospital in Central Norway. Eight therapists trained in using the feedback scales in the Partners for Change Outcome Management System (PCOMS) treated the intervention group. Seventeen therapists treated the controls, providing treatment without using feedback scales. The main outcome measures were treatment alliance and patient satisfaction. RESULTS: Seventy-five patients participated. There were no differences between the groups in the intention to treat (ITT) analyses on alliance (mean difference = 0.08, 95% CI -0.44, 0.59, p = 0.760) or satisfaction (mean difference = 0.24, 95% CI -1.85, 2.32, p = 0.819), and no statistically significant differences between the groups in the per protocol (PP, n = 58) analyses on alliance (mean difference = 0.32, 95% CI -0.84, 3.16, p = 0.137) or satisfaction (mean difference = 1.16, 95% CI -0.84, 3.16, p = 0.248) six weeks after the treatment started. The effect size in favour of the PCOMS group increased from 0.07 for alliance and 0.06 for satisfaction in the intention to treat analysis to 0.40 on alliance and 0.31 for satisfaction in the per protocol analysis. Among the other outcomes, the PCOMS group had better motivation for treatment (estimated mean difference ITT: 0.29, 95% CI 0.00 to 0.57, p = 0.05, PP: 0.28, 95% CI 0.04, 0.52, p = 0.024). CONCLUSION: Six weeks after starting treatment, there were no effects on alliance and satisfaction from using two brief feedback scales. Since the per protocol analyses showed higher effect sizes, future investigations in a larger study with longer follow-up are warranted. | Male | Humans | Patient Participation | Patient Satisfaction | OK |
| Victor M. | Montori | Jacqueline | Arciniega | Thomas | McGinn | Patient Education and Counseling | 2010-07-00 Jul 2010 | OBJECTIVE: To assess the impact of a decision aid on perceived risk of heart attacks and medication adherence among urban primary care patients with diabetes. METHODS: We randomly allocated 150 patients with diabetes to participate in a usual primary care visit either with or without the Statin Choice tool. Participants completed a questionnaire at baseline and telephone follow-up at 3 and 6 months. RESULTS: Intervention patients were more likely to accurately perceive their underlying risk for a heart attack without taking a statin (OR: 1.9, CI: 1.0-3.8) and with taking a statin (OR: 1.4, CI: 0.7-2.8); a decline in risk overestimation among patients receiving the decision aid accounts for this finding. There was no difference in statin adherence at 3 or 6 months. CONCLUSION: A decision aid about using statins to reduce coronary risk among patients with diabetes improved risk communication, beliefs, and decisional conflict, but did not improve adherence to statins. PRACTICE IMPLICATIONS: Decision aid enhanced communication about the risks and benefits of statins improved patient risk perceptions but did not alter adherence among patients with diabetes. | Male | Decision Support Techniques | Humans | Patient Participation | OK |
| Patricia | Gooding | Judith | Johnson | Daniel | Pratt | The Journal of Nervous and Mental Disease | 2010-05-00 May 2010 | Mental health research may pose a risk to those who participate in it, especially for potentially vulnerable groups such as those diagnosed with schizophrenia. The current study aimed to investigate the subjective experience of research participation in this group. Seventy-nine individuals with diagnoses of schizophrenia spectrum disorders who had taken part in research looking at suicide were asked to provide feedback about their experiences. Responses were analyzed using qualitative and quantitative methods. Results indicate that negative feedback concerning participation was rare, occurring in 2.5% to 15.2% of responses. Positive feedback was more frequent, occurring in 45.6% to 60.8% of responses. Qualitative thematic analysis identified 5 key themes in participants' responses: Altruism, Value of being involved in research, Therapeutic effect, Enjoyable experience and Negative experiences. The results support the view that mental health research in this group can be ethically viable. | Male | Humans | Patient Participation | Patient Satisfaction | OK |
| Edward L. | Hannan | Turner M. | Osler | Dana B. | Mukamel | Annals of Surgery | 2012-04-00 Apr 2012 | OBJECTIVE: To develop a 30-day mortality risk index for noncardiac surgery that can be used to communicate risk information to patients and guide clinical management at the "point-of-care," and that can be used by surgeons and hospitals to internally audit their quality of care. BACKGROUND: Clinicians rely on the Revised Cardiac Risk Index to quantify the risk of cardiac complications in patients undergoing noncardiac surgery. Because mortality from noncardiac causes accounts for many perioperative deaths, there is also a need for a simple bedside risk index to predict 30-day all-cause mortality after noncardiac surgery. METHODS: Retrospective cohort study of 298,772 patients undergoing noncardiac surgery during 2005 to 2007 using the American College of Surgeons National Surgical Quality Improvement Program database. RESULTS: The 9-point S-MPM (Surgical Mortality Probability Model) 30-day mortality risk index was derived empirically and includes three risk factors: ASA (American Society of Anesthesiologists) physical status, emergency status, and surgery risk class. Patients with ASA physical status I, II, III, IV or V were assigned either 0, 2, 4, 5, or 6 points, respectively; intermediate- or high-risk procedures were assigned 1 or 2 points, respectively; and emergency procedures were assigned 1 point. Patients with risk scores less than 5 had a predicted risk of mortality less than 0.50%, whereas patients with a risk score of 5 to 6 had a risk of mortality between 1.5% and 4.0%. Patients with a risk score greater than 6 had risk of mortality more than 10%. S-MPM exhibited excellent discrimination (C statistic, 0.897) and acceptable calibration (Hosmer-Lemeshow statistic 13.0, P = 0.023) in the validation data set. CONCLUSIONS: Thirty-day mortality after noncardiac surgery can be accurately predicted using a simple and accurate risk score based on information readily available at the bedside. This risk index may play a useful role in facilitating shared decision making, developing and implementing risk-reduction strategies, and guiding quality improvement efforts. | Male | Decision Support Techniques | Humans | Female | OK |
| Margareta R. | Schmid |  |  |  |  | Health Expectations: An International Journal of Public Participation in Health Care & Health Policy | 2014-06-00 juin 2014 | Background: Growing recognition of the role of citizens and patients in health and health care has placed a spotlight on health literacy and patient education. Objective: To identify specific competencies for health in definitions of health literacy and patient‐centred concepts and empirically test their dimensionality in the general population. Methods: A thorough review of the literature on health literacy, self‐management, patient empowerment, patient education and shared decision making revealed considerable conceptual overlap as competencies for health and identified a corpus of 30 generic competencies for health. A questionnaire containing 127 items covering the 30 competencies was fielded as a telephone interview in German, French and Italian among 1255 respondents randomly selected from the resident population in Switzerland. Findings: Analyses with the software MPlus to model items with mixed response categories showed that the items do not load onto a single factor. Multifactorial models with good fit could be erected for each of five dimensions defined a priori and their corresponding competencies: information and knowledge (four competencies, 17 items), general cognitive skills (four competencies, 17 items), social roles (two competencies, seven items), medical management (four competencies, 27 items) and healthy lifestyle (two competencies, six items). Multiple indicators and multiple causes models identified problematic differential item functioning for only six items belonging to two competencies. Conclusions: The psychometric analyses of this instrument support broader conceptualization of health literacy not as a single competence but rather as a package of competencies for health. (PsycINFO Database Record (c) 2014 APA, all rights reserved). (journal abstract) | Psychometrics | Test Construction | Health Literacy | Swiss Health Literacy Survey | Doublon |
| Margareta R. | Schmid |  |  |  |  | Health Expectations: An International Journal of Public Participation in Health Care and Health Policy | 2014-06-00 Jun 2014 | BACKGROUND: Growing recognition of the role of citizens and patients in health and health care has placed a spotlight on health literacy and patient education. OBJECTIVE: To identify specific competencies for health in definitions of health literacy and patient-centred concepts and empirically test their dimensionality in the general population. METHODS: A thorough review of the literature on health literacy, self-management, patient empowerment, patient education and shared decision making revealed considerable conceptual overlap as competencies for health and identified a corpus of 30 generic competencies for health. A questionnaire containing 127 items covering the 30 competencies was fielded as a telephone interview in German, French and Italian among 1255 respondents randomly selected from the resident population in Switzerland. FINDINGS: Analyses with the software MPlus to model items with mixed response categories showed that the items do not load onto a single factor. Multifactorial models with good fit could be erected for each of five dimensions defined a priori and their corresponding competencies: information and knowledge (four competencies, 17 items), general cognitive skills (four competencies, 17 items), social roles (two competencies, seven items), medical management (four competencies, 27 items) and healthy lifestyle (two competencies, six items). Multiple indicators and multiple causes models identified problematic differential item functioning for only six items belonging to two competencies. CONCLUSIONS: The psychometric analyses of this instrument support broader conceptualization of health literacy not as a single competence but rather as a package of competencies for health. | Male | Humans | Questionnaires | Female | OK |
| Annette | Becker | Andreas | Sönnichsen | Erika | Baum | Patient Education and Counseling | 2010-02-00 Feb 2010 | OBJECTIVE: To assess the feasibility and outcome of measuring the theory of planned behaviour (TPB) in patients receiving routine counselling versus counselling with a decision aid (DA) during primary care consultation on cardiovascular risk prevention. METHODS: A DA was developed, based on models of shared decision-making (SDM) and the TPB. We evaluated the impact of the intervention in a randomized controlled trial. Main outcomes were previously reported. To assess the intermediate social cognitive processes and our theoretical framework, we evaluated the impact of the intervention on a TPB scale. RESULTS: The TPB scale showed satisfactory measurement properties. Factor analysis (main component analysis, confirmatory model) could mostly replicate the assumptions of the model. 44% of variance of the behavioural intention to adhere to the decision after counselling was explained in linear regression models. Of the TPB components, only attitude towards the decision and moral norm were significantly more positive in the intervention. No difference was found with regard to intention to adhere to the decision. High risk resulted in higher values of the TPB components in both groups. CONCLUSION: Most DAs are developed and tested without explicitly referring to a theoretical model of psychosocial processes. The TPB may serve as a useful theoretical framework. PRACTICE IMPLICATIONS: Trials on DAs demonstrate positive effects on psychological outcomes of patients without leading to better objective health results. Our study might contribute to an explanation: DAs might not cause stronger adherence to decisions even though one's attitude towards the decision becomes more positive. | Decision Making | Decision Support Techniques | Humans | Patient Participation | OK |
| V. | Cahill | J. | Appleton | E. | Willetts | The Journal of the Royal College of Physicians of Edinburgh | 2012-00-00 2012 | BACKGROUND: We believe that there is a need to increase awareness, particularly among foundation year doctors, of the importance of performing a full neurological examination, including ophthalmoscopy, in medical inpatients. Following a serious unexpected incident (missed papilloedema), we implemented a multifaceted intervention, including ensuring greater availability of equipment for neurological/ ophthalmological assessment, education and curriculum redesign in two large teaching hospitals in the UK. METHODS: Following the results of our initial intervention, we introduced a patient assessment scoring system to evaluate patient recollection of the completeness of neurological examination by medical staff in the two Trusts over a four-month period. RESULTS: Of the 93 patients referred to neurology during this period, 33% could not recollect being examined with a tendon hammer and 48% said they had not been examined with an ophthalmoscope. In contrast, the majority (95.7%) remembered the use of a stethoscope in their examination. The data were fed back to medical staff which resulted in greater awareness of the importance of a complete neurological examination. No further adverse incidents of missed papilloedema were reported in the following 12 months, although it would be premature to state that this situation has been resolved. CONCLUSIONS: A patient assessment score can be used by medical staff to raise awareness of the importance of a complete neurological examination from referring physicians. | Male | Humans | Patient Participation | physicians | OK |
| Esther | Park | Aileen | Ho | Jennifer | Shaffer | Journal of Burn Care & Research: Official Publication of the American Burn Association | 2010-10-00 2010 Sep-Oct | The objective of this study was to investigate perceptions of occupational therapists and physiotherapists on the use of Nintendo Wii™ (Nintendo of America Inc., Redmond, WA) in rehabilitation. Occupational therapists and physiotherapists in a rehabilitation hospital trialed four Wii games that addressed physical movement, balance, coordination, and cognitive performance. Then, they completed an opinion survey on the utility of Wii in rehabilitation. The results were compared between burn care therapists (BTs) and nonburn care therapists, using chi(2) with a P < .05 considered significant. The participation rate was 79% (63/80), and they agreed that Wii was easy to set up (71%), operate (68%), and safe to use (76%). Participants agreed that Wii would be beneficial in outpatient (76%) and inpatient (65%) settings and that it could improve treatment compliance (73%). Participants recommended 15 to 30-minute Wii intervention (59%) daily (81%) and twice per week (43%). Participants believed that neurologic (71%), trauma (68%), burn (59%), and musculoskeletal (49%) patients would benefit from Wii intervention but not cardiac (43%) or organ transplant patients (18%). Participants believed that outcomes using Wii could be measured reliably (49%), and skills learned while playing could be transferable to daily function (60%). The significant differences between BTs and nonburn care therapists' perceptions are that BT-treated younger patients (21-40 years vs >60 years, P < .05) and BT favored the therapeutic benefit of Wii in rehabilitation (93% vs 58%, P = .02), specifically in burn rehabilitation (85% vs 39%, P = .001). Occupational therapists and physiotherapists favored the use of Wii in rehabilitation as an adjunct to traditional therapy because it is therapeutic, engaging, and may increase patient participation in rehabilitation. | Male | Humans | Patient Participation | Questionnaires | OK |
| Claire E. | Wakefield |  |  |  |  | Patient Education and Counseling | 2013-02-00 Feb 2013 | OBJECTIVE: A key component of decision aids (DAs) are the Value Clarification Exercises (VCEs), however, rates of completion VCEs are variable. The aim of this paper is to propose explanations for these variations. METHODS: A review of the rates of completion and the reasons provided across eleven studies evaluating five different DAs was conducted. RESULTS: Retrospective evaluation does not appear to be an indication of prospective use. Differences in mode of administration are unclear. Gender does not appear to have a difference on completion rates. Complexity of the decision appears to reflect rates of VCE completion. The main reason provided for non-completion of VCEs was that patients had already made a decision. CONCLUSION: VCEs have shown a trend to producing better congruence between values and choices, but it remains unclear how VCEs impact on the quality of the decision. Value of VCEs in treatment decision-making has not been previously established and our data indicate information alone might be enough to clarify concepts. PRACTICE IMPLICATIONS: DAs are becoming more widely used in practice. It is important to understand the elements in a treatment decision-making so that the burden on the patient is at a minimum in practice. | Male | Decision Making | Decision Support Techniques | Humans | OK |
|  |  |  |  |  |  | Journal of Comparative Effectiveness Research | 2014-03-00 Mar 2014 | Two international movements, evidence-based medicine (EBM) and shared decision-making (SDM) have grappled for some time with issues related to defining the meaning, role and measurement of values/preferences in their respective models of treatment decision-making. In this article, we identify and describe unresolved problems in the way that each movement addresses these issues. The starting point for this discussion is that at least two essential ingredients are needed for treatment decision-making: research information about treatment options and their potential benefits and risks; and the values/preferences of participants in the decision-making process. Both the EBM and SDM movements have encountered difficulties in defining the meaning, role and measurement of values/preferences in treatment decision-making. In the EBM model of practice, there is no clear and consistent definition of patient values/preferences and no guidance is provided on how to integrate these into an EBM model of practice. Methods advocated to measure patient values are also problematic. Within the SDM movement, patient values/preferences tend to be defined and measured in a restrictive and reductionist way as patient preferences for treatment options or attributes of options, while broader underlying value structures are ignored. In both models of practice, the meaning and expected role of physician values in decision-making are unclear. Values clarification exercises embedded in patient decision aids are suggested by SDM advocates to identify and communicate patient values/preferences for different treatment outcomes. Such exercises have the potential to impose a particular decision-making theory and/or process onto patients, which can change the way they think about and process information, potentially impeding them from making decisions that are consistent with their true values. The tasks of clarifying the meaning, role and measurement of values/preferences in treatment decision-making models such as EBM and SDM, and determining whose values ought to count are complex and difficult tasks that will not be resolved quickly. Additional conceptual thinking and research are needed to explore and clarify these issues. To date, the values component of these models remains elusive and underdeveloped. | Decision Making | Humans | Patient Participation | Physician-Patient Relations | OK |
| Eun Jung | Kim | Min Soo | Park | Kyungsoo | Park | Contemporary Clinical Trials | 2012-07-00 Jul 2012 | BACKGROUND AND AIMS: This study was conducted to investigate the views of patients and healthy volunteers on participation in clinical trials. METHODS: A total of 291 clinical trial participants, including 140 patients and 151 healthy volunteers, were recruited from four university hospital-affiliated clinical trial centers among 15 Korean regional clinical trial centers in South Korea where the levels of information and care were sufficient to meet the global standard. Participants were recruited from phase I trials or bioequivalence tests, a short term hospitalization under close monitoring in the clinical trial centers, or from phase II, III or IV trials occurring in both wards and outpatient clinics. A structured questionnaire survey was performed to identify their perspectives on clinical trials. RESULTS: Participants who were patients were significantly influenced by medical personnels regarding the decision making processes for participation in clinical trials when compared to healthy volunteers. However, no difference was found between the two groups in the level of willingness to participate in and satisfaction with clinical trials. More than 50% of patient subjects misunderstood and thought that their physicians could persuade them to participate in clinical trials or that all the participants would receive a new drug or treatment during trials. CONCLUSIONS: Clinical researchers who are involved in clinical trials should make an extra effort to confirm the level of understanding of their patients regarding the clinical trial and to guarantee that each patient has sufficient time to make an informed decision before participating in a clinical trial. | Male | Humans | Patient Participation | Patient Satisfaction | OK |
|  |  |  |  |  |  |  | 2013-00-00 2013 | Objective. The purpose of this study was to determine the factors that influence social workers' support for client autonomy, and how support for client autonomy impacts the willingness to engage in shared decision making. Shared decision making creates a decisional partnership intended to promote client autonomy while also addressing concerns regarding client safety and decisional capacity. Methods. This study employed a randomized factorial survey of social workers employed working with adults with severe mental illness. The study was designed to test: (1) the degree to which the clinical characteristics such as diagnosis, symptomology, treatment adherence and environmental context such as housing and homeless history of individual clients, and the practice values and experience of social workers influence the social worker's support for a particular client's autonomy and, (2) the degree to which those same factors predict the willingness of a social worker to engage the client in shared decision making. The study also tested whether the willingness to engage in shared decision making was mediated by support for autonomy. In total, 87 social workers responded to the survey yielding 435 vignettes for analysis. Results. The hypotheses were partially supported by the data. Diagnosis, symptomology, threats of harm, treatment adherence, and drug and alcohol use all predicted support for autonomy, and also the willingness to engage in shared decision making. A diagnosis of schizophrenia, psychotic symptoms, or poor treatment adherence predicted reduced support for autonomy by social workers, and a diminished likelihood that social workers would utilize shared decision making. Social worker's values and experience also predicted support for autonomy and willingness to utilize shared decision making. The willingness to engage in shared decision making was mediated by support for autonomy, though the effect was modest. Implications. The results of this study indicate greater need for interventions targeting social work students designed to reduce stigma surrounding severe mental illness, and also the need for ongoing clinical supervision for early career social workers. Helping social workers avoid bias in their decision making is critical to the overall goal of supporting autonomy, building client capacity, minimizing client disempowerment, and promoting recovery oriented services. (PsycINFO Database Record (c) 2013 APA, all rights reserved) | Clients | Social Workers | Mental Disorders | clinical social workers | OK |
| A. | Gafni | S. | Meiyappan | D. | David | Supportive Care in Cancer: Official Journal of the Multinational Association of Supportive Care in Cancer | 2012-03-00 Mar 2012 | PURPOSE: We explored regret in thyroid cancer patients, relating to the decision to accept or reject adjuvant radioactive iodine treatment. METHODS: We studied patients with a recent diagnosis of early stage papillary thyroid carcinoma, in whom treatment decisions on adjuvant radioactive iodine had been finalized. Participants completed a Decision Regret Scale questionnaire. We asked the participants to identify who made the final decision about radioactive iodine treatment. We explored the relationship between decision regret and a) degree of patient involvement in decision-making and b) receipt of radioactive iodine treatment. RESULTS: We included 44 individuals, more than half of whom received adjuvant radioactive iodine treatment (26/44). Decision regret was generally low (mean 22.1, standard deviation [SD] 13.0). Participants reported that the final treatment decision was made by the following: patient and doctor (52.3%, 23/44), completely the patient (27.3%, 12/44), or completely the physician (20.5%, 9/44). Decision regret significantly differed according to who made the final decision: the patient (mean 19.0, SD 11.3), patient and doctor (mean 19.5, SD 7.4), and the doctor (mean 32.9, SD 20.37) (F = 4.569; degrees of freedom = 2, 41; p = 0.016). There was no significant difference in decision regret between patients who received radioactive iodine and those who did not (mean difference -2.5; 95% confidence interval -10.6, 5.6; p = 0.540). CONCLUSION: Thyroid cancer patients who reported being involved in the final treatment decision on adjuvant radioactive iodine had less regret than those who did not. | Male | Decision Making | Emotions | Humans | OK |
| Fliss E. M. | Murtagh | Natalia | Calanzani | Irene J. | Higginson | Palliative Medicine | 2013-05-00 May 2013 | BACKGROUND: The Council of Europe has recommended that member states of European Union encourage their citizens to make decisions about their healthcare before they lose capacity to do so. However, it is unclear whether the public wants to make such decisions beforehand. AIM: To examine public preferences for self-involvement in end-of-life care decision-making and identify associated factors. DESIGN: A population-based survey with 9344 adults in England, Belgium, Germany, Italy, the Netherlands, Portugal and Spain. RESULTS: Across countries, 74% preferred self-involvement when capable; 44% preferred self-involvement when incapable through, for example, a living will. Four factors were associated with a preference for self-involvement across capacity and incapacity scenarios, respectively: higher educational attainment ((odds ratio = 1.93-2.77), (odds ratio = 1.33-1.80)); female gender ((odds ratio = 1.27, 95% confidence interval = 1.14-1.41), (odds ratio = 1.30, 95% confidence interval = 1.20-1.42)); younger-middle age ((30-59 years: odds ratio = 1.24-1.40), (50-59 years: odds ratio = 1.23, 95% confidence interval = 1.04-1.46)) and valuing quality over quantity of life or valuing both equally ((odds ratio = 1.49-1.58), (odds ratio = 1.35-1.53)). Those with increased financial hardship (odds ratio = 0.64-0.83) and a preference to die in hospital (not a palliative care unit) (odds ratio = 0.73, 95% confidence interval = 0.60-0.88), a nursing home or residential care (odds ratio = 0.73, 95% confidence interval = 0.54-0.99) were less likely to prefer self-involvement when capable. For the incapacity scenario, single people were more likely to prefer self-involvement (odds ratio = 1.34, 95% confidence interval = 1.18-1.53). CONCLUSIONS: Self-involvement in decision-making is important to the European public. However, a large proportion of the public prefer to not make decisions about their care in advance of incapacity. Financial hardship, educational attainment, age, and preferences regarding quality and quantity of life require further examination; these factors should be considered in relation to policy. | Male | Decision Making | Humans | Patient Participation | OK |
| Claudius | King | Lukas | Radbruch | Raymond | Voltz | BMC medical ethics | 2014-00-00 2014 | BACKGROUND: Advance directives (ADs) are assumed to reflect the patients' preferences, even if these are not clearly expressed. Research into whether this assumption is correct has been lacking. This study explores to what extent ADs reflect the true wishes of the signatories. METHODS: Semi-structured interviews (INT), pretest. Transcribed INT and the contents of ADs were inductively categorised (Mayring) and triangulated. Software: MAXQDA 2007. PARTICIPANTS: Patients receiving palliative care (PPC), healthy (H) and chronically ill (CI) individuals with an AD completed ≥3 months prior to recruitment. RESULTS: Between 08/2008 and 07/2009, 53 individuals (20 H, 17 CI, 16 PPC) were interviewed (mean age 63.2 years (55-70 years)), 34% male). Most important (in)consistencies between preferences as expressed in INT compared to ADs included preconditions for termination/rejection of life-sustaining measures, refusal of/demand for medical interventions and the nomination of proxies. Standardized AD forms were rarely tailored to the individual. We found a high tendency to use set phrases, such as want to die with dignity or do not want to suffer/vegetate. Likely events in the course of an existing progressive disease were not covered, even in ADs of PPC close to death. CONCLUSIONS: Only some of the incongruities between verbally expressed preferences and the contents of the AD can be put down to use of standardized forms or lack of medical knowledge. Nevertheless, the non-involvement of a doctor in the process of making an AD must be seen as potentially problematic and seeking medical advice should be promoted by politics and physicians. Standardised forms should encourage amendments and present space for free text entries for all aspects covered. Set phrases need to be defined by the individual to enable them to be translated into a specific course of action. | Male | Humans | Patient Participation | Patient Satisfaction | OK |
|  |  |  |  |  |  | Yearbook of Medical Informatics | 2012-00-00 2012 | OBJECTIVE: This paper summarizes the recent trends and highlights the challenges and opportunities in decision support and knowledge management for patient-centered, personalized, and personal health care. METHODS: The discussions are based on a broad survey of related references, focusing on the most recent publications. Major advances are examined in the areas of i) shared decision making paradigms, ii) continuity of care infrastructures and architectures, iii) human factors and system design approaches, iv) knowledge management innovations, and v) practical deployment and change considerations. RESULTS: Many important initiatives, projects, and plans with promising results have been identified. The common themes focus on supporting the individual patients who are playing an increasing central role in their own care decision processes. New collaborative decision making paradigms and information infrastructures are required to ensure effective continuity of care. Human factors and usability are crucial for the successful development and deployment of the relevant systems, tools, and aids. Advances in personalized medicine can be achieved through integrating genomic, phenotypic and other biological, individual, and population level information, and gaining useful insights from building and analyzing biological and other models at multiple levels of abstraction. Therefore, new Information and Communication Technologies and evaluation approaches are needed to effectively manage the scale and complexity of biomedical and health information, and adapt to the changing nature of clinical decision support. CONCLUSION: Recent research in decision support and knowledge management combines heterogeneous information and personal data to provide cost-effective, calibrated, personalized support in shared decision making at the point of care. Current and emerging efforts concentrate on developing or extending conventional paradigms, techniques, systems, and architectures for the new predictive, preemptive, and participatory health care model for patient-centered, personalized medicine. There is also an increasing emphasis on managing complexity with changing care models, processes, and settings. | | | | | OK |
| Michel | Rousseau | Dawn | Stacey | Hubert | Robitaille | Academic Medicine: Journal of the Association of American Medical Colleges | 2011-08-00 Aug 2011 | PURPOSE: Do residents in family medicine practice share decision making with patients during consultations? This study used a validated scale to score family medicine residents' shared decision-making (SDM) skills in primary care consultations and to determine whether residents' demographic characteristics were correlated with their scores. METHOD: Between January 2009 and April 2010 at two Canadian academic health centers, the authors recruited unique dyads of patients consulting in primary care and family medicine residents. They recorded, transcribed, and assessed consultations using the Observing Patient Involvement in Decision Making (OPTION) scale, which measures 12 SDM-specific behaviors on a scale of 0% to 100% (high score = better SDM). They calculated descriptive and inferential statistics for the scores. RESULTS: From 212 eligible residents, the authors recruited 152 unique patient-resident dyads (participation rate = 75%): 68 dyads from 13 clinics in London, Ontario, and 84 from six family medicine units in Quebec City, Quebec. The mean global OPTION score was 24% ± 8%; the mean score for each of the 12 items ranged from 4% to 37%. Five of the 12 behaviors obtained a mean score below "a minimal attempt is made to exhibit the behavior" (i.e., <25%). There was a positive correlation between the score and the duration of the consultation (r = 0.24, P = .003), with longer consultations producing higher scores. CONCLUSIONS: Participating family medicine residents have not integrated SDM behaviors, which may also pertain to residencies elsewhere. Interventions are required to foster family medicine residents' practice of SDM. | Male | Humans | Patient Participation | Physician-Patient Relations | OK |
| Michel | Rousseau | Dawn | Stacey | Hubert | Robitaille | Academic Medicine | 2011-08-00 août 2011 | Purpose: Do residents in family medicine practice share decision making with patients during consultations? This study used a validated scale to score family medicine residents’ shared decision-making (SDM) skills in primary care consultations and to determine whether residents’ demographic characteristics were correlated with their scores. Method: Between January 2009 and April 2010 at two Canadian academic health centers, the authors recruited unique dyads of patients consulting in primary care and family medicine residents. They recorded, transcribed, and assessed consultations using the Observing Patient Involvement in Decision Making (OPTION) scale, which measures 12 SDM-specific behaviors on a scale of 0% to 100% (high score = better SDM). They calculated descriptive and inferential statistics for the scores. Results: From 212 eligible residents, the authors recruited 152 unique patient–resident dyads (participation rate = 75%): 68 dyads from 13 clinics in London, Ontario, and 84 from six family medicine units in Quebec City, Quebec. The mean global OPTION score was 24% ± 8%; the mean score for each of the 12 items ranged from 4% to 37%. Five of the 12 behaviors obtained a mean score below “a minimal attempt is made to exhibit the behavior” (i.e., &lt;25%). There was a positive correlation between the score and the duration of the consultation (r = 0.24, P = .003), with longer consultations producing higher scores. Conclusions: Participating family medicine residents have not integrated SDM behaviors, which may also pertain to residencies elsewhere. Interventions are required to foster family medicine residents’ practice of SDM. (PsycINFO Database Record (c) 2012 APA, all rights reserved). (journal abstract) | Shared decision making | Decision Making | family medicine residents | Observing Patient Involvement in Decision Making Scale | Doublon |
| Sally | Youssef | Deborah | Palmer |  |  | Joint, Bone, Spine: Revue Du Rhumatisme | 2010-12-00 Dec 2010 | OBJECTIVE: To assess the validity, reliability and responsiveness to change of a patient self-reported questionnaire that can assess construct outcome measures of patients with ankylosing spondylitis (AS)/spondyloarthritis. METHODS: The questionnaire was developed by integrating information obtained from patients suffering from AS/spondyloarthritis based on the Rasch model. The questionnaire includes assessment of functional disability, quality of life, VAS for spinal pain, joint pain, global status, fatigue, duration of morning stiffness, review of the systems, falls and cardiovascular risks, self-helplessness as well as self-reported joint and soft tissue pain. The questionnaire was completed by 267 consecutive patients with AS (124) and spondyloarthritis (143). RESULTS: The PROMs questionnaire was reliable as demonstrated by a high-standardized alpha. The questionnaire items correlated significantly (p<0.01) with clinical parameters of disease activity. Patient reported tender joints and enthesitis scores correlated significantly with the physician's scores (correlation coefficient 0.848 and 0.821 respectively). Changes in functional disability, quality of life as well as self-helplessness scores showed significant (p<0.01) variation with diseases activity status. The PROMs questionnaire showed also a high degree of comprehensibility (9.3). CONCLUSION: The developed PROMs questionnaire is a reliable and valid instrument for assessment of patients suffering from AS and spondyloarthritis. Being short, rapid and comprehensive, this adds more to its applicability. The data support the value of completion of the simple two pages patient questionnaire, which provides a quantitative written documented record by the patient, at each visit to the rheumatologist. | Male | Humans | Patient Participation | Questionnaires | OK |
| Kathryn | Church |  |  |  |  | Patient Education and Counseling | 2010-12-00 décembre 2010 | Objective: To describe development and testing of a counseling tool intended to inform family planning clients while helping the family planning provider facilitate the client's decision-making process; and to discuss challenges and requisites for shifting to shared decision-making from the extremes of decision-making dominated by the provider, on one hand, or unaided by the provider, on the other. Development of the tool: The WHO Decision-Making Tool for Family Planning Clients and Providers is derived from evidence-based principles of client-centered care and counseling. This article discusses how these principles are manifested in the Tool and how the Tool aids both provider and client in improving counseling. Methods: Development of the Tool involved formative workshops with providers in Indonesia, Mexico, South Africa and Trinidad and Tobago and observational evaluation research in Indonesia, Mexico, and Nicaragua. Analysis of videotaped counseling sessions quantitatively assessed client–provider communication and decision-making. Also, focus-group discussions, interviews, and a questionnaire collected qualitative data from providers and clients. Results: In general, use of the Tool improved providers’ counseling performance: they engaged clients more and gave more and better tailored information. For clients, the Tool increased their communication and involvement. Both the Nicaraguan and Mexican studies found marked shifts toward the client in the locus of decision-making after introduction of the Tool. Practice implications: Use of the tool improves the performance of both providers and clients in family planning counseling and decision-making. There are challenges, however, at the levels of both the provider and the organization in sustaining these changes and scaling up such initiatives in quality of care. (PsycINFO Database Record (c) 2012 APA, all rights reserved). (journal abstract) | Decision Making | Psychometrics | Test Construction | Clients | Doublon |
| Kathryn | Church |  |  |  |  | Patient Education and Counseling | 2010-12-00 Dec 2010 | OBJECTIVE: To describe development and testing of a counseling tool intended to inform family planning clients while helping the family planning provider facilitate the client's decision-making process; and to discuss challenges and requisites for shifting to shared decision-making from the extremes of decision-making dominated by the provider, on one hand, or unaided by the provider, on the other. DEVELOPMENT OF THE TOOL: The WHO Decision-Making Tool for Family Planning Clients and Providers is derived from evidence-based principles of client-centered care and counseling. This article discusses how these principles are manifested in the Tool and how the Tool aids both provider and client in improving counseling. METHODS: Development of the Tool involved formative workshops with providers in Indonesia, Mexico, South Africa and Trinidad and Tobago and observational evaluation research in Indonesia, Mexico, and Nicaragua. Analysis of videotaped counseling sessions quantitatively assessed client-provider communication and decision-making. Also, focus-group discussions, interviews, and a questionnaire collected qualitative data from providers and clients. RESULTS: In general, use of the Tool improved providers' counseling performance: they engaged clients more and gave more and better tailored information. For clients, the Tool increased their communication and involvement. Both the Nicaraguan and Mexican studies found marked shifts toward the client in the locus of decision-making after introduction of the Tool. PRACTICE IMPLICATIONS: Use of the tool improves the performance of both providers and clients in family planning counseling and decision-making. There are challenges, however, at the levels of both the provider and the organization in sustaining these changes and scaling up such initiatives in quality of care. | Male | Decision Making | Decision Support Techniques | Humans | OK |
| Renée | Drolet | Sophie | Desroches | Dawn | Stacey | Patient Education and Counseling | 2012-08-00 Aug 2012 | OBJECTIVE: To identify and analyze training programs in shared decision-making (SDM) for health professionals. METHODS: We conducted an environmental scan looking for programs that train health professionals in SDM. Pairs of reviewers independently analyzed the programs identified using a standardized data extraction sheet. The developers of the programs validated the data extracted. RESULTS: We identified 54 programs conducted between 1996 and 2011 in 14 countries and 10 languages. Thirty-four programs targeted licensed health professionals, 10 targeted pre-licensure health professionals, and 10 targeted both. Most targeted only the medical profession (n=32); six targeted more than one health profession. The five most frequently mentioned teaching methods were case-based discussion, small group educational session, role play, printed educational material, and audit and feedback. Thirty-six programs reported having evaluated their impacts but evaluation data was available only for 17. CONCLUSIONS: Health professional training programs in SDM vary widely in how and what they deliver, and evidence of their effectiveness is sparse. PRACTICE IMPLICATIONS: This study suggests there is a need for international consensus on ways to address the variability in SDM training programs. We need agreed criteria for certifying the programs and for determining the most effective types of training. | Decision Making | Humans | Patient Participation | Questionnaires | OK |
| Marissa | Dainton |  |  |  |  | Journal of Renal Care | 2013-09-00 Sep 2013 | BACKGROUND: Evidence supports the view that people with long term conditions who are encouraged to take a greater interest in their treatment can experience a range of health benefits. Traditionally centre-based haemodialysis patients have been passive recipients whilst nurses have been deliverers of care. The Shared Haemodialysis Care (SHC) programme changes this relationship. OBJECTIVE: Our aim was to initiate a programme of education in SHC across Yorkshire and Humber in North England, with its objective to support centre-based patients to take on aspects of their own treatment. DESIGN & PARTICIPANTS: A three tiered training model was designed to educate all grades of nursing staff. Central to this approach was the development of a four day competency based course aimed at training 25% of junior sisters/charge nurses, staff nurses and Level 3 health care assistants (unqualified nurses). MEASUREMENTS/APPROACH: We measured the number of staff trained and assessed staff knowledge using a pre and post course questionnaire. Individual course day evaluations and 'Light bulb moment 'sessions captured qualitative data and a census form relating to interest in and uptake of SHC captured patient engagement data. RESULTS: The pre and post delegate questionnaires from every cohort have demonstrated significant positive shifts in all the objectives of the training programme. The target of 25% of staff trained has been reached in all but one group of units. A third of all patients across Yorkshire and Humber are significantly engaged in their haemodialysis care. CONCLUSION: A nurse-led education programme can provide nurse participants with the tools to encourage and support patients to become more engaged in their haemodialysis treatment. | Humans | Patient Participation | Questionnaires | Patient Education as Topic | OK |
| Tommy A. | Brown | Scott R. | Steele | Matthew J. | Martin | Archives of Surgery (Chicago, Ill.: 1960) | 2012-01-00 Jan 2012 | OBJECTIVES: To examine patient perceptions and willingness to participate in resident education and to assess the effect on patient willingness and consent rates. DESIGN: Anonymous questionnaire designed to capture demographics, overall opinions of teaching programs, and willingness to consent to various scenarios of trainee participation. Descriptive and univariate analyses were performed. SETTING: Tertiary-level referral center. PATIENTS: Three hundred sixteen individuals scheduled for elective surgery. MAIN OUTCOME MEASURES: Consent rates for various scenarios. RESULTS: Of the 316 patients who completed the questionnaire, most expressed overall support of resident training: 91.2% opined that their care would be equivalent to or better than that of a private hospital, 68.3% believed they derived benefit from participation, and most consented to having an intern (85.0%) or a resident (94.0%) participate in their surgical procedure. However, when given specific, realistic scenarios involving trainee participation, major variations in the consent rate were observed. Affirmative consent rates decreased from 94.0% to 18.2% as the level of resident participation increased. Patients also were more willing to consent to the participation of a senior resident (83.1%) vs a junior resident (57.6%) or an intern (54.5%). Patients overwhelmingly opined that they should be informed of the level of resident participation and that this information could change their decision of whether to consent. CONCLUSIONS: Most patients expressed approval of teaching facilities and resident education. However, consent rates were significantly altered when more detailed information was provided and they declined with increasing levels of resident participation. Providing detailed informed consent is preferred by patients but it could adversely affect resident participation and training. | Male | Humans | Patient Participation | Questionnaires | OK |
| Fiona Y. Y. | Wong | Eliza L. Y. | Wong | Kwai Fun | Huen | The Journal of Adolescent Health: Official Publication of the Society for Adolescent Medicine | 2010-12-00 Dec 2010 | OBJECTIVES: To explore the attitudes of adolescents and parents toward transition care and to identify factors and barriers associated with transition decision. METHODS: The study was conducted at a pediatric out-patient clinic using a self-administered questionnaire to evaluate the perspective of adolescent chronic patients on transition care for common chronic diseases. Despite its importance as emphasized in medical literature, transition care for adolescent patients with chronic illnesses is under-developed in Hong Kong. RESULTS: A total of 137 adolescents aged 16-19 years, and 67 parents completed the survey; 85.3% of adolescents and 82.5% of parents were willing to transfer to adult care. "Adolescent's perception of his/her own responsibility towards chronic illness" was positively associated with willingness to transfer to adult care (OR = 3.84; 95% CI, 1.41-10.45; p = .008), whereas "Detailed explanation by doctors" encouraged transition decision for adolescents (OR = 12.20; 95% CI, 1.22-122.33; p = .033). "Do not want to change" was the only significant barrier for transition for both adolescents (OR = .08; 95% CI, .01-.50; p = .007) and parents (OR = .07; 95% CI, .02-.36; p = .001). However, less than 10% of subjects had ever received any transition information from doctors or other healthcare workers. CONCLUSIONS: This is the first study in Asia region evaluating attitudes of adolescents and parents on transition care. The majority of adolescents and parents accept transition care from pediatrics to adult service, but only a small proportion has ever received transition information from doctors. Our findings should be useful to healthcare providers in planning transition care programmes for adolescent patients with chronic illnesses. | Male | Humans | Patient Participation | Questionnaires | OK |
|  |  |  |  |  |  | ANS. Advances in nursing science | 2012-09-00 2012 Jul-Sep | Receiving a cancer diagnosis marks a life transition that evokes feelings of chaos. Additional transitions occur when patients with relapsed cancer must decide to pursue conventional care or participate in experimental clinical trials. Individuals with hematologic malignancies (n = 25) and their caregivers (n = 20) were interviewed about their decisions to have an experimental stem cell transplant. Noting that they had "no other choice," participants expressed no regret posttransplant. "Doing something" perhaps helped address the chaos of cancer. This aggressive response to advanced cancer also represented a social imperative that negated the options of living with the cancer or entering palliative care. | Male | Decision Making | Humans | Patient Participation | OK |
| Ronald H. | Behrens |  |  |  |  | Infectious Disease Clinics of North America | 2012-09-00 Sep 2012 | A significant number of travellers sustain travel-related injury or illness, despite receiving pretravel advice. This appears to be due to a combination of inconsistent guidance about risks and recommendations, and partial adherence. This article considers perceptions and attitudes to risk, factors affecting uptake of advice, and features of an effective consultation. A framework is proposed for a pretravel consultation, using a shared decision-making approach. Engaging the traveller as an active participant in maintaining their own health and providing balanced, evidence-based information about risks and benefits is predicted to enhance the effectiveness of the pretravel consultation. | Humans | communication | risk assessment | Professional-Patient Relations | OK |
| Damian | Jenkinson |  |  |  |  | Disability and Rehabilitation | 2014-00-00 2014 | PURPOSE: The aim of this study was to delineate the type of treatment currently provided to rehabilitate the upper limb following stroke in order to inform the treatment protocol for a control arm of a future pragmatic clinical trial of assistive technologies. METHOD: A national survey of stroke teams was conducted. Using open-ended, free script questions, the survey was designed to capture the important components of treatment that are most commonly used to treat patients described as mild, moderate or severely impaired following stroke. Stroke teams were targeted within the 28 geographical areas covered by the Stroke Improvement Networks. RESULT: Treatment descriptions were returned by 53 stroke teams from 20 of the 28 geographical areas. In total, 998 treatment components were provided - 289 for mildly, 403 for moderately and 306 for severely impairment patients following stroke). These captured five substantive treatment categories in terms of most active, moderately active and non-active patient participation in treatment as well as assistive technologies and other. CONCLUSION: From the treatment components described, the highest number of treatment components was listed for the moderately impaired patient. The treatment components used by the units surveyed are quite consistent with the 2012 Clinical Guidelines for Stroke. | | | | | OK |
|  |  |  |  |  |  | The Journal of Medicine and Philosophy | 2014-04-00 Apr 2014 | It has recently been proposed to incorporate the use of a "Patient Preference Predictor" (PPP) into the process of making treatment decisions for incapacitated patients. A PPP would predict which treatment option a given incapacitated patient would most likely prefer, based on the individual's characteristics and information on what treatment preferences are correlated with these characteristics. Including a PPP in the shared decision-making process between clinicians and surrogates has the potential to better realize important ethical goals for making treatment decisions for incapacitated patients. However, developing and implementing a PPP poses significant practical challenges. The present paper discusses these practical challenges and considers ways to address them. | Decision Making | Humans | Questionnaires | patient preference | OK |
| Karen | Fung | Benjamin | Barankin | Robert | Bissonnette | Journal of Cutaneous Medicine and Surgery | 2010-10-00 2010 Sep-Oct | BACKGROUND: Informed shared decision making is a mutual process engaging both doctor and patient and informed by best medical evidence and patient values and preferences. OBJECTIVE: Our aim was to identify the needs of psoriasis patients in decisions on selecting treatment. METHODS: Psoriasis subjects participated in an online survey on decisional role, postdecisional conflict, and treatment awareness. RESULTS: Of 2,622 people invited to participate, 248 completed surveys. Their most recent treatment decision was either made by subjects alone (42%) or physicians alone (28%) or was shared (29%). Subjects perceived that their doctors lacked time to stay abreast of treatments, to provide counseling, and to access appropriate treatments. Deficiencies most frequently identified were information on options, clarification of values, access to physicians, and decision-making skills. Those with a body surface area (BSA) ≥ 3% more frequently indicated that having the skill or ability to make treatment decisions was important. LIMITATIONS: The limitations of this study include sampling, recall, and reporting bias. Percent BSA was not verified. CONCLUSIONS: The multiple deficiencies in support of psoriasis patients in treatment decisions may preclude informed shared decision making. | Decision Making | Humans | Physician-Patient Relations | Cross-Sectional Studies | OK |
| T. | Sensky | C. P. F. | Van Der Staak | C. a. J. | De Jong | The International Journal of Social Psychiatry | 2011-05-00 May 2011 | BACKGROUND: Little is known about the perspectives of either patients or clinicians regarding treatment goals in addiction healthcare. In general, treatment goals involve abstinence or at least reduction of substance use. AIM: To examine and compare the treatment goals indicated by both patients and clinicians at baseline, interim and exit measurement. METHOD: A descriptive study was performed with multiple measurements of treatment goals. Patients (n = 111) and clinicians (n = 20) were recruited from three addiction treatment centres in the Netherlands. A Shared Decision Making Intervention (SDMI) was undertaken to promote and evaluate treatment agreement. RESULTS: Patients identified treatment goals of daytime activities and abstinence or reduced alcohol consumption as most important. Clinicians indicated psychological distress, daytime activities and substance use as most important. Differences between patients and clinicians were found for the treatment goals of physical health (patient > clinician) and psychological distress (clinician > patient). The results further showed that treatment goals of both patients and clinicians become more closely aligned during the course of treatment. CONCLUSION: SDMI provides a method to explore and discuss discrepancy between patients' and clinicians' goals of treatment which leads to convergence. Such convergence is likely to be a necessary prerequisite for positive treatment outcomes. | Male | Humans | Patient Participation | Questionnaires | OK |
| Jan | Dommerholt | Boudewijn | Stegenga | Michel | Wensing | BMC medicine | 2011-00-00 2011 | BACKGROUND: Shoulder pain is a common musculoskeletal problem that is often chronic or recurrent. Myofascial trigger points (MTrPs) cause shoulder pain and are prevalent in patients with shoulder pain. However, few studies have focused on MTrP therapy. The aim of this study was to assess the effectiveness of multimodal treatment of MTrPs in patients with chronic shoulder pain. METHODS: A single-assessor, blinded, randomized, controlled trial was conducted. The intervention group received comprehensive treatment once weekly consisting of manual compression of the MTrPs, manual stretching of the muscles and intermittent cold application with stretching. Patients were instructed to perform muscle-stretching and relaxation exercises at home and received ergonomic recommendations and advice to assume and maintain good posture. The control group remained on the waiting list for 3 months. The Disabilities of Arm, Shoulder and Hand (DASH) questionnaire score (primary outcome), Visual Analogue Scale for Pain (VAS-P), Global Perceived Effect (GPE) scale and the number of muscles with MTrPs were assessed at 6 and 12 weeks in the intervention group and compared with those of a control group. RESULTS: Compared with the control group, the intervention group showed significant improvement (P < 0.05) on the DASH after 12 weeks (mean difference, 7.7; 95% confidence interval (95% CI), 1.2 to 14.2), on the VAS-P1 for current pain (mean difference, 13.8; 95% CI, 2.6 to 25.0), on the VAS-P2 for pain in the past 7 days (mean difference, 10.2; 95% CI, 0.7 to 19.7) and VAS-P3 most severe pain in the past 7 days (mean difference, 13.8; 95% CI, 0.8 to 28.4). After 12 weeks, 55% of the patients in the intervention group reported improvement (from slightly improved to completely recovered) versus 14% in the control group. The mean number of muscles with active MTrPs decreased in the intervention group compared with the control group (mean difference, 2.7; 95% CI, 1.2 to 4.2). CONCLUSIONS: The results of this study show that 12-week comprehensive treatment of MTrPs in shoulder muscles reduces the number of muscles with active MTrPs and is effective in reducing symptoms and improving shoulder function in patients with chronic shoulder pain. TRIAL REGISTRATION NUMBER: ISRCTN: ISRCTN75722066. | Humans | Patient Participation | Questionnaires | Treatment Outcome | OK |
| A. M. | Stiggelbout | R. A. | Nout | M. | Kamps | British Journal of Cancer | 2014-08-12 Aug 12, 2014 | BACKGROUND: Vaginal brachytherapy (VBT) in high-intermediate-risk endometrial cancer (EC) provides a significant reduction in the risk of local cancer recurrence, but without survival benefit and with increased mucosal atrophy. Five-year local control is estimated to be similar for VBT and a watchful waiting policy (WWP), in which patients receive VBT combined with external radiation in case of a recurrence. Our aim was to assess treatment preferences of EC patients and clinicians regarding VBT and WWP, and to evaluate their preferred and perceived involvement in treatment decision making. METHODS: Interviews were held with 95 treated EC patients. The treatment trade-off method was used to assess the minimally desired benefit from VBT in local control. Patients' preferred and perceived involvement in decision making were assessed using a questionnaire. Seventy-seven clinicians completed a questionnaire assessing their minimally desired benefit and preferred involvement in decision making. RESULTS: Minimally desired benefit of VBT was significantly lower for patients than for clinicians (median=0 vs 8%, P<0.001), for irradiated than for non-irradiated patients (median=0 vs 6.5%, P<0.001), and for radiation oncologists than for gynaecologists (median=4 vs 13%, P<0.001). Substantial variation existed within the groups of patients and clinicians. Participants preferred the patient and clinician to share in the decision about VBT. However, irradiated patients indicated low perceived involvement in actual treatment decision making. CONCLUSIONS: We found variations between and within patients and clinicians in minimally desired benefit from VBT. However, the recurrence risk at which patients preferred VBT was low. Our results showed that patients consider active participation in decision making essential. | Decision Making | Humans | Patient Participation | Physician-Patient Relations | OK |
| M. | Bhandari |  |  |  |  | Journal of Long-Term Effects of Medical Implants | 2013-00-00 2013 | INTRODUCTION: Decision boards can be useful in shared decision making by helping patients and their physicians choose among treatment options. Two surgical treatments for early knee osteoarthritis (OA) are high tibial osteotomy (HTO) and the KineSpring® Knee Implant System. The primary objective of this study was to determine patient preferences between these two treatments using a decision board. METHODS: We developed a decision board that presented information on HTO and the KineSpring System for treating knee OA. First, it was presented to 15 individuals for a pilot test and a "scope test." Then it was presented to 81 individuals who were asked to imagine that they had early to midstage knee OA, and this group was administered a complete a series of questions, including their treatment preference and what they would be willing to pay if they elected to use the KineSpring System. Descriptive statistics were calculated and a chi-squared test was conducted to assess any significant differences in patient preferences based on demographic characteristics. RESULTS: Our pilot test confirmed that most participants (87%) agreed that the decision board was easy to understand and helped them in making a decision. Of 81 respondents, the KineSpring System was preferred by 60% (n = 49). Individuals selecting KineSpring would be willing to pay an average of $2,700 to receive it over HTO. CONCLUSIONS: When provided with treatment options and information, 60% of individuals preferred the KineSpring System over HTO. The decision board was well-received as a useful tool for presenting information. | Male | Decision Support Techniques | Humans | Female | OK |
| Frans J. | Mulder | Thierry G. | Guitton | David | Ring | The Journal of Hand Surgery | 2014-11-00 Nov 2014 | PURPOSE: To test the null hypothesis that there are no differences in the priorities and preferences of patients with idiopathic trigger finger (TF) and hand surgeons. METHODS: One hundred five hand surgeons of the Science of Variation Group and 84 patients with TF completed a survey about their priorities and preferences in decision making regarding the management of TF. The questionnaire was structured according the Ottawa Decision Support Framework for the development of a decision aid. RESULTS: Patients desired orthotics more and surgery less than physicians. Patients and physicians disagreed on the main advantage of several treatment options for TFs and on disadvantages of the treatment options. Patients preferred to decide for themselves after receiving advice, whereas physicians preferred a shared decision. Patients preferred booklets, and physicians opted for Internet and video decision aids. CONCLUSIONS: Comparing patients and hand surgeons, there were some differences in treatment preferences and perceived advantages and disadvantages regarding idiopathic TF-differences that might be addressed by a decision aid. CLINICAL RELEVANCE: Information that helps inform patients of their options based on current best evidence might help them understand their own preferences and values, reduce decisional conflict, limit surgeon-to-surgeon variations, and improve health. | | | | | OK |
| M. | Müller-Engelmann | M. | Heinzel-Gutenbrunner | T. | Krones | Methods of Information in Medicine | 2013-00-00 2013 | OBJECTIVES: The OPTION scale ("observing patient involvement in decision making") assesses the extent to which clinicians involve patients in decisions across a range of situations in clinical practice. It so far just covers physician behavior. We intended to modify the scoring of the OPTION scale to incorporate active patient behavior in consultations. METHODS: Modification was done on scoring level, attempting a dyadic, relationship-centred approach in that high ratings can be evoked also by the behaviour of active patients. The German version of the OPTION scale was compared with a modified version by analysing video recordings of primary care consultations dealing with cardiovascular prevention. Fifteen general practitioners provided 40 videotaped consultations. Videos were analysed by two rater pairs and two experts in shared decision making (SDM). RESULTS: Reliability measures of the modified version were lower than those of the original scale. Significant associations of the dichotomised scale with the expert SDM rating as well as with physicians' expertise in SDM were only found for the modified OPTION scale. Receiver Operating Characteristic (ROC) analyses confirmed a valid differentiation between the presence of SDM (yes/no) on total score level, even though the cut-off point was quite low. Standard deviations of the single items in the modified version were higher compared to the original OPTION scale, while the means of total scores were similar. CONCLUSIONS: The original OPTION scale is physician-centered and neglects the activity and a possible self-involvement of the patient. Our modified instruction was able to capture the dyadic element partially. The development of a separate dyadic instrument might be more promising. | | | | | OK |
| J. | Karstanje | J. | Passchier | A. | Tibben | Public Health Genomics | 2010-00-00 2010 | BACKGROUND: Preconception carrier screening for hemoglobinopathies (HbPs) is debated in the Netherlands. OBJECTIVES: Intentions to participate in preconception carrier screening for HbPs as well as informed reproductive options were assessed in 109 Turkish female immigrants. METHOD: Participants completed a structured questionnaire. RESULTS: 83.5% of 109 Turkish female immigrants intended to participate in preconception carrier screening. Intention to participate was related to the desire to reduce uncertainty about having offspring affected by HbPs, but not with socio-demographic variables, risk-estimation and worrying. If the tests concerning carrier status for HbPs were confirmative for both partners, 36.3% (n = 33) of the women with the intention of preconception carrier screening would refrain from getting children of their own, including 14.3% (n = 13) opting for adoption, 37.4% (n = 34) would decide to become pregnant and to opt for prenatal testing on HbPs. Finally, among 37.4% of the women opting for prenatal testing, 30.3% (n = 10) would terminate an affected pregnancy, 18.2% (n = 6) not and 51.5% (n = 17) could not decide (1 missing value). CONCLUSION: A large majority of Turkish female immigrants would participate in preconception carrier screening for HbPs. | Humans | Patient Participation | Questionnaires | Young Adult | OK |
| E. | Rorarius | M. | Sihvo | E. | Isotalo | Journal of Voice: Official Journal of the Voice Foundation | 2011-09-00 Sep 2011 | OBJECTIVE: To study the effects of voice treatment including brief voice therapy for 12 months in two groups of voice patients: organic and functional. DESIGN AND METHODS: A clinical prospective follow-up study with repeated measurements in five phases: medical session, first voice therapy session, last voice therapy session, and 6 months and 12 months after voice therapy. The mean number of voice therapy sessions was 3.4. The main outcome measures were the Voice Activity and Participation Profile (VAPP) and the Symptom Questionnaire. Of consenting patients (n=141) with chronic voice disorders, 46 dropped out during follow-up. Ninety-five patients formed the study group. Forty-one of them received only voice therapy, but the rest of them received combined treatment (medication, amplifiers, and voice massage), but also experienced life events affecting voice. Patients with any laryngeal pathology formed the organic group (n=47), others had a functional voice disorder. RESULTS: Using the improvement criterion that the change of the VAPP score should exceed standard error of measurement, the percentage of individual patients achieving improvement was 47% in the mild, 59% in the moderate, and 75% in the severe disorder groups. Effect size for VAPP total score was 0.89. The positive effect continued to progress after the therapy ended. Patients with functional or organic voice disorder improved almost equally, although minor findings indicate that functional patients benefited more. CONCLUSIONS: Voice treatment had a progressive effect for 1 year in half of our patients. No statistical difference was found between the functional and organic patient groups. | Male | Humans | Patient Participation | Patient Satisfaction | OK |
|  |  |  |  |  |  | Journal of Cardiovascular Electrophysiology | 2013-04-00 Apr 2013 | The use of smart phones has increased dramatically and there are nearly a billion users on 3G and 4G networks worldwide. Nearly 60% of the U.S. population uses smart phones to access the internet, and smart phone sales now surpass those of desktop and laptop computers. The speed of wireless communication technology on 3G and 4G networks and the widespread adoption and use of iOS equipped smart phones (Apple Inc., Cupertino, CA, USA) provide infrastructure for the transmission of wireless biomedical data, including ECG data. These technologies provide an unprecedented opportunity for physicians to continually access data that can be used to detect issues before symptoms occur or to have definitive data when symptoms are present. The technology also greatly empowers and enables the possibility for unprecedented patient participation in their own medical education and health status as well as that of their social network. As patient advocates, physicians and particularly cardiac electrophysiologists should embrace the future and promise of wireless ECG recording, a technology solution that can truly scale across the global population. | Time Factors | Male | Humans | Patient Participation | OK |
| Sarah | McNicol | Mary | Dixon-Woods | Sara | Kenyon | Clinical Trials (London, England) | 2013-02-00 Feb 2013 | BACKGROUND: The implications of offering unblinding to trial participants to treatment arm after trial completion have been little explored. PURPOSE: We sought to explore trial participants' perspectives on whether they would like to be unblinded as to the treatment arm to which they were allocated following involvement in a large randomised controlled trial (RCT). METHODS: We conducted semi-structured interviews with 38 women who had participated in a trial during suspected preterm labour and had received the results of a long-term follow-up study that identified adverse outcomes for children in some of the treatment groups. Participants were sampled purposively. Analysis was based on the constant comparative method. RESULTS: Most women reported that they wanted to know the treatment arm to which they had been allocated. While the primary motive for some was curiosity, many others wanted to know as part of an attempt to understand or explain their child's current health problems. These women were motivated by a search for a coherent causal narrative, even though unblinding was unlikely to be able to meet their aspirations. Some participants identified potential disadvantages in discovering their treatment allocation, including feeling responsible for their child's health status, and some women were very clear that they did not want to know their treatment group. LIMITATIONS: A purposive sample was used and the extent to which it represents the views of all participants in the study is not established. CONCLUSIONS: Important challenges arise in offering to unblind trial participants, whatever the trial results. Participants may need help and support to understand the limitations of the knowledge they gain through being unblinded and to decide whether they wish to know to which treatment arm they were allocated. | Humans | Patient Participation | Questionnaires | Female | OK |
|  |  |  |  |  |  | Patient Education and Counseling | 2011-01-00 Jan 2011 | OBJECTIVE: experts recommend that clinicians tailor their interactions according to each patient's preferred decision style. Because participation is associated with improved clinical outcomes, factors which modify preferred role should be addressed before determining the level at which patients wish to participate. The objective of this study was to determine if certainty related to initiating treatment is related to preferred role in decision making. METHODS: we conducted face-to-face interviews with 142 patients eligible for the treatment of hepatitis C. Preferred role in decision making was measured using the Control Preferences Scale and certainty was measured with 11-point numeric rating scale. RESULTS: subjects who were uncertain whether they wanted to start treatment were more likely to prefer Role 2: "to make the final selection of my treatment after seriously considering my doctor's opinion" over Role 1 "to make the final selection about which treatment I will receive" compared to those who were certain [Adjusted OR (95% CI)=4.9 (1.7-14.5)]. Subjects who were uncertain were also more likely to prefer sharing responsibility for decision making over Role 1 compared to those who were certain [Adjusted OR (95% CI)=3.7 (1.3-10.4)]. CONCLUSIONS: certainty is associated with preferred decision styles. Physicians should ascertain their patients' levels of uncertainty and adjust their input accordingly. PRACTICE IMPLICATION: physicians should ascertain their patients' levels of uncertainty and adjust their input accordingly. | Male | Decision Making | Humans | Patient Participation | OK |
| Sean | Tunis |  |  |  |  | The Oncologist | 2010-00-00 2010 | Patients, clinicians, payers, and policymakers face an environment of significant evidentiary uncertainty as they attempt to achieve maximum value, or the greatest level of benefit possible at a given level of cost in their respective health care decisions. This is particularly true in the area of oncology, for which published evidence from clinical trials is often incongruent with real-world patient care, and a substantial portion of clinical use is for off-label indications that have not been systematically evaluated. It is this uncertainty in the knowledge of the clinical harms and benefits associated with oncology treatments that prevents postregulatory decision makers from making accurate assessments of the value of these treatments. Because of the incentives inherent in the clinical research enterprise, randomized control trials (RCTs) are designed for the specific purpose of regulatory approval and maximizing market penetration. The pursuit of these goals results in RCT study designs that achieve maximal internal validity at the expense of generalizability to diverse real-world patient populations that may have significant comorbidities and other clinically mitigating factors. As such, systematic reviews for the purposes of coverage and treatment decisions often find relevant and high-quality evidence to be limited or nonexistent. For a number of reasons, including frequent off-label use of medications and the expedited approval process for cancer drugs by the U.S. Food and Drug Administration, this situation is exacerbated in the area of oncology. This paper investigates the convergence of incentives and circumstances that lead to widespread uncertainty in oncology and proposes new paradigms for clinical research, including pragmatic clinical trials, methodological guidance, and coverage with evidence development. Each of these initiatives would support the design of clinical research that is more informative for postregulatory decision makers, and would therefore reduce uncertainty and provide greater confidence in conclusions about the value of these treatments. | Decision Making | Humans | Patient Participation | Physician-Patient Relations | OK |
| Stephen | Jan |  |  |  |  | BMC family practice | 2012-00-00 2012 | BACKGROUND: In spite of the potential impact upon population health and expenditure, interventions promoting medication adherence have been found to be of moderate effectiveness and cost effectiveness. Understanding the relative influence of factors affecting patient medication adherence decisions and the characteristics of individuals associated with variation in adherence will lead to a better understanding of how future interventions should be designed and targeted. This study aims to explore medication-taking decisions that may underpin intentional medication non-adherence behaviour amongst a community sample and the relative importance of medication specific factors and patient background characteristics contributing to those decisions. METHODS: A discrete choice experiment conducted through a web-enabled online survey was used to estimate the relative importance of eight medication factors (immediate and long-term medication harms and benefits, cost, regimen, symptom severity, alcohol restrictions) on the preference to continue taking a medication. To reflect more closely what usually occurs in practice, non-disease specific medication and health terms were used to mimic decisions across multiple medications and conditions.161 general community participants, matching the national Australian census data (age, gender) were recruited through an online panel provider (participation rate: 10%) in 2010. RESULTS: Six of the eight factors (i.e. immediate and long-term medication harms and benefits, cost, and regimen) had a significant influence on medication choice. Patient background characteristics did not improve the model. Respondents with private health insurance appeared less sensitive to cost then those without private health insurance. In general, health outcomes, framed as a side-effect, were found to have a greater influence over adherence than outcomes framed as therapeutic benefits. CONCLUSIONS: Medication-taking decisions are the subject of rational choices, influenced by the attributes of treatments and potentially amenable to intervention through education, strategic pricing and the altering of dosing characteristics. Understanding individual treatment preferences is thus an important step to improving adherence support provision in practice. Re-framing future interventions and policies to support rational and informed individual patient choices, is the way forward to realising the full potential health and economic benefits from the efficacious use of medications. | Male | Humans | Patient Participation | Questionnaires | OK |
| Richard M. | Adanu | Paula M. | Lantz |  |  | International Journal of Gynaecology and Obstetrics: The Official Organ of the International Federation of Gynaecology and Obstetrics | 2013-09-00 Sep 2013 | OBJECTIVE: To determine the types of access to care most strongly associated with facility-based delivery among women in Ghana. METHODS: Data relating to the "5 As of Access" framework were extracted from the 2008 Ghana Demographic Health Survey and analyzed using multivariate logistic regression. RESULTS: In all, 55.5% of a weighted sample of 1102 women delivered in a healthcare facility, whereas 45.5% delivered at home. Affordability was the strongest access factor associated with delivery location, with health insurance coverage tripling the odds of facility delivery. Availability, accessibility (except urban residence), acceptability, and social access variables were not significant factors in the final models. Social access variables, including needing permission to seek healthcare and not being involved in decisions regarding healthcare, were associated with a reduced likelihood of facility-based delivery when examined individually. Multivariate analysis suggested that these variables reflected maternal literacy, health insurance coverage, and household wealth, all of which attenuated the effects of social access. CONCLUSION: Affordability was an important determinant of facility delivery in Ghana-even among women with health insurance-but social access variables had a mediating role. | Decision Making | Humans | Patient Participation | Female | OK |
| Jessica | Sage | Carole M. | Lannon | Esi | Morgan Dewitt | Pediatric Rheumatology Online Journal | 2013-00-00 2013 | BACKGROUND: The increase in therapeutic options for juvenile idiopathic arthritis (JIA) has added complexity to treatment decisions. Shared decision making has the potential to help providers and families work together to choose the best possible option for each patient from the array of choices. As part of a needs assessment, prior to design and implementation of shared decision making interventions, we conducted a qualitative assessment of clinicians' current approaches to treatment decision making in JIA. METHODS: Pediatric rheumatology clinicians were recruited from 2 academic children's hospitals affiliated with a quality improvement learning network, using purposive and snowball sampling. Semi-structured interviews elicited how clinicians with prescribing authority (n = 10) interact with families to make treatment decisions. Interviews were audio-recorded and transcribed verbatim. A multi-disciplinary research team used content analysis to analyze the interview data.To validate data from individual interviews and enrich our understanding, we presented the interview results to pediatric rheumatology clinicians attending a learning network meeting (n = 24 from 12 children's hospitals). We then asked the clinicians questions to further identify and discuss areas of variation in the decision-making processes. RESULTS: Clinicians described a decision-making process in which they, rather than the family or other care team members, consistently initiated treatment decisions. Initial treatment options presented to families generally reflected the clinician's preferred treatment approaches, which differed across clinicians. Clinicians used various methods to inform families about treatment options and tailor information according to perceptions of a family's information needs, level of comprehension or mood (e.g. anxiety). The attributes of medication presented to families fell into 4 categories: benefits, risks, logistics and family preferences. Clinicians typically included family members in the decision to initiate JIA treatment after limiting the options to fit the clinical situation and the clinician's own preferences. Family members' preferences were seen as more integral in the decision to stop treatment after symptom remission. CONCLUSIONS: Decision making about initial JIA treatment appears to be largely driven by clinician preferences. Family preferences are more likely to be considered for treatment discontinuation. Opportunities exist to develop, test, and implement tools to facilitate shared decision making in pediatric rheumatology. | | | | | Doublon |
| Jessica | Sage | Carole M. | Lannon | Esi | Morgan Dewitt | Pediatric Rheumatology Online Journal | 2013-00-00 2013 | BACKGROUND: The increase in therapeutic options for juvenile idiopathic arthritis (JIA) has added complexity to treatment decisions. Shared decision making has the potential to help providers and families work together to choose the best possible option for each patient from the array of choices. As part of a needs assessment, prior to design and implementation of shared decision making interventions, we conducted a qualitative assessment of clinicians' current approaches to treatment decision making in JIA. METHODS: Pediatric rheumatology clinicians were recruited from 2 academic children's hospitals affiliated with a quality improvement learning network, using purposive and snowball sampling. Semi-structured interviews elicited how clinicians with prescribing authority (n = 10) interact with families to make treatment decisions. Interviews were audio-recorded and transcribed verbatim. A multi-disciplinary research team used content analysis to analyze the interview data.To validate data from individual interviews and enrich our understanding, we presented the interview results to pediatric rheumatology clinicians attending a learning network meeting (n = 24 from 12 children's hospitals). We then asked the clinicians questions to further identify and discuss areas of variation in the decision-making processes. RESULTS: Clinicians described a decision-making process in which they, rather than the family or other care team members, consistently initiated treatment decisions. Initial treatment options presented to families generally reflected the clinician's preferred treatment approaches, which differed across clinicians. Clinicians used various methods to inform families about treatment options and tailor information according to perceptions of a family's information needs, level of comprehension or mood (e.g. anxiety). The attributes of medication presented to families fell into 4 categories: benefits, risks, logistics and family preferences. Clinicians typically included family members in the decision to initiate JIA treatment after limiting the options to fit the clinical situation and the clinician's own preferences. Family members' preferences were seen as more integral in the decision to stop treatment after symptom remission. CONCLUSIONS: Decision making about initial JIA treatment appears to be largely driven by clinician preferences. Family preferences are more likely to be considered for treatment discontinuation. Opportunities exist to develop, test, and implement tools to facilitate shared decision making in pediatric rheumatology. | | | | | OK |
| Jeeyun | Lee | Seonwoo | Kim | Moon Ki | Choi | BMC cancer | 2012-00-00 2012 | BACKGROUND: In the past few years, the number of clinical trials has increased rapidly in East Asia, especially for gastric and hepatobiliary cancer that are prevalent in Asian populations. However, the actual degree of understanding or perceptions of clinical trials by cancer patients in East Asian countries have seldom been studied. METHODS: Between July 1st and November 30th of 2011, we conducted a prospective study to survey cancer patients regarding their awareness of, and willingness to participate in, a clinical trial. Patients with gastrointestinal/hepatobiliary cancer who visited the Hematology-Oncology outpatient clinic at Samsung Medical Center (SMC) were enrolled. A total of 21 questions were asked including four questions which used the Visual analogue scale (VAS) score. RESULTS: In this survey study, 1,000 patients were asked to participate and 675 patients consented to participate (67.5%). The awareness of clinical trials was substantially higher in patients who had a higher level of education (p<0.001), were married (p=0.004), and had a higher economic status (p=0.001). However, the willingness to participate in a clinical trial was not affected by the level of education or economic status of patients. The most influential factors for patient willingness to participate were a physician recommendation (n=181, 26.8%), limited treatment options (n=178, 26.4%), and expectations of effectiveness of new anti-cancer drugs (n=142, 21.0%). Patients with previous experience in clinical trials had a greater willingness to participate in clinical trials compared to patients without previous experience (p<0.001). CONCLUSIONS: This large patient cohort survey study showed that Korean cancer patients are more aware of clinical trials, but awareness did not translate into willingness to participate. | Male | Humans | Patient Participation | Questionnaires | OK |
| Yankel M. | Kupersztoch | M. Elizabeth | Paulk | Yookyung | Kim | PloS One | 2013-00-00 2013 | BACKGROUND: Culture shapes how people understand illness and death, but few studies examine whether acculturation influences patients' end-of-life treatment preferences and medical care. METHODS AND FINDINGS: In this multi-site, prospective, longitudinal cohort study of terminally-ill cancer patients and their caregivers (n = 171 dyads), trained interviewers administered the United States Acculturation Scale (USAS). The USAS is a 19-item scale developed to assess the degree of "Americanization" in first generation or non-US born caregivers of terminally-ill cancer patients. We evaluated the internal consistency, concurrent, criterion, and content validity of the USAS. We also examined whether caregivers' USAS scores predicted patients' communication, treatment preferences, and end-of-life medical care in multivariable models that corrected for significant confounding influences (e.g. education, country of origin, English proficiency). The USAS measure was internally consistent (Cronbach α = 0.98); and significantly associated with US birthplace (r = 0.66, P<0.0001). USAS scores were predictive of patients' preferences for prognostic information (AOR = 1.31, 95% CI:1.00-1.72), but not comfort asking physicians' questions about care (AOR 1.23, 95% CI:0.87-1.73). They predicted patients' preferences for feeding tubes (AOR = 0.68, 95% CI:0.49-0.99) and wish to avoid dying in an intensive care unit (AOR = 1.36, 95% CI:1.05-1.76). Scores indicating greater acculturation were also associated with increased odds of patient participation in clinical trials (AOR = 2.20, 95% CI:1.28-3.78), compared with lower USAS scores, and greater odds of patients receiving chemotherapy (AOR = 1.59, 95% CI:1.20-2.12). CONCLUSION: The USAS is a reliable and valid measure of "Americanization" associated with advanced cancer patients' end-of-life preferences and care. USAS scores indicating greater caregiver acculturation were associated with increased odds of patient participation in cancer treatment (chemotherapy, clinical trials) compared with lower scores. Future studies should examine the effects of acculturation on end-of-life care to identify patient and provider factors that explain these effects and targets for future interventions to improve care (e.g., by designing more culturally-competent health education materials). | Male | Humans | Physician-Patient Relations | Questionnaires | OK |
|  |  |  |  |  |  |  | 2012-00-00 2012 | Performance measurements are used to gauge and improve the effectiveness and efficiency of institutions, but rarely do the measurement guidelines include instructions to achieve success. As more public institutions and public policies became subject to the uncompromising nature of performance measurement, the need to understand the changes that implementing a performance measurement system bring to public organizations grew ever more important. The implementation of the federal No Child Left Behind Act (NCLB) and the Delaware Student Testing Program (DSTP) provided a universal and level playing field to identify and observe these changes. But what changes were brought and what was the condition of the field? If the performance measurement system was systemically flawed, then the changes observed were suspect as well. To explore this topic, this dissertation presents the organizational changes implemented in Delaware elementary schools in response to federal and state accountability mandates, and in addition this dissertation explores whether the organizational changes differed by the degree of student achievement success. Through looking at these changes through school principal interviews and surveys, this dissertation peered inside the black box between the promise and expectations of performance measures and the resulting outcomes to explore the conceptions and practices of school leadership, the changes to the school-level decision-making process, and the changes in school culture that have resulted from the implementation of a performance measurement/accountability policy. Finally this dissertation seeks to determine the degree to which the DSTP contains the characteristics of an adequate performance measurement system. Since the implementation of the DSTP, the organizational changes implemented in Delaware schools have been real and significant; the findings of this analysis show that principals have become focused on their leadership practices aiming towards becoming instructional leaders and have instituted shared decision making practices. As a possible result of these changes, the climate among teachers has changed and has become more collaborative. However, this dissertation found no appreciable differences between successful schools and less successful school on these dimensions. Finally, this dissertation has found that while the DSTP has several shortcomings it is an adequate performance measurement system. (PsycINFO Database Record (c) 2012 APA, all rights reserved) | Decision Making | Teaching | Delaware elementary schools | expectations | OK |
| Asnat | Groutz | Ami | Amit | Foad | Azem | Patient Education and Counseling | 2012-02-00 Feb 2012 | OBJECTIVES: To investigate the gender preference of Muslim Israeli-Arab women regarding obstetricians/gynecologists, and identify other features that affect their choice. METHODS: The study included 167 responders to an anonymous questionnaire. RESULTS: Around one-half of the responders had no gender preference regarding family physicians, but most (76.6%) preferred a female gynecologist. Likewise, most responders preferred pelvic examinations (85.6%) and pregnancy follow-up (77.8%) by female gynecologists. Additionally, 61.7% preferred consulting female physicians for major obstetrical and gynecological (OB/GYN) problems. The reasons for female preference were embarrassment (67.7%), feeling comfortable with female gynecologists (80.8%) and the notion that female gynecologists are more gentle (68.3%). The three most important factors which affected actual selection, however, were experience (83.8%), knowledge (70.1%) and ability (50.3%), rather than physician gender (29.3%). Multivariate analysis revealed that other qualities and importance of background variables of the gynecologist were independent predictors of gender preference. CONCLUSIONS: Although Muslim Arab-Israeli women express gender bias regarding their preference for gynecologists/obstetricians, personal and professional skills are considered to be more important factors when it comes to actually making a choice. PRACTICE IMPLICATION: We suggest that the ideal obstetrician/gynecologist for these women would be female, though skilled, knowledgeable, and experienced male would be appropriate. | Male | Humans | Patient Participation | Patient Satisfaction | OK |
|  |  |  |  |  |  | Patient Education and Counseling | 2011-03-00 Mar 2011 | OBJECTIVE: To integrate findings of a mixed methods research (MMR) into adolescents' preferences and competencies for communication during consultations, in order to demonstrate the added value of MMR for health communication research. METHODS: Sequential MMR with adolescents (12-19 years) with various chronic conditions in a university hospital. Methods comprised: (1) 31 face-to-face interviews; (2) Q-methodology; (3) 39 observations of outpatient consultations; (4) three focus groups with 27 healthcare providers; (5) web-based questionnaire in 960 adolescents. RESULTS: Adolescents had different preferences regarding health communication, but all wished to be involved as partners. Yet, their actual participation during consultations was low. They often acted as bystanders rather than main characters because their participation was neither requested nor encouraged. Parents filled the gap, to healthcare providers' frustration. The questionnaire confirmed the discrepancy between self-efficacy and self-reported independent behavior during consultations. CONCLUSION: Triadic communication was all but multi-party-talk and adolescents did not act and were not considered as main partners. MMR was of pivotal importance for our understanding. PRACTICE IMPLICATIONS: As chronically ill adolescents need to prepare themselves for transition to adult care, healthcare providers should encourage them to take the lead in communication by initiating independent visits and changing the parents' roles. | Humans | Patient Participation | Patient Satisfaction | Physician-Patient Relations | OK |
|  |  |  |  |  |  | Journal of Evaluation in Clinical Practice | 2012-10-00 Oct 2012 | The recent Mental Capacity Act (2005) sets out a test for assessing a person's capacity to make treatment choices. In some cases, particularly in psychiatry, it is unclear how the criteria ought to be interpreted and applied by clinicians. In this paper, I argue that this uncertainty arises because the concept of capacity employed in the Act, and the diagnostic tools developed to assist its assessment, overlook the inherent normativity of judgements made about whether a person is using or weighing information in the decision-making process. Patients may fail on this criterion to the extent that they do not appear to be handling the information given in an appropriate way, on account of a mental impairment disrupting the way the decision process ought to proceed. Using case law and clinical examples, I describe some of the normative dimensions along which judgements of incapacity can be made, namely epistemic, evaluative and affective dimensions. Such judgements are complex and the normative standards by which a clinician may determine capacity cannot be reduced to a set of criteria. Rather, in recognizing this normativity, clinicians may better understand how clinical judgements are structured and what kinds of assumption may inform their assessment. | Humans | Patient Participation | Physician-Patient Relations | Mental Competency | OK |
| Lori | McLeod | Cheryl | Coon | Ari | Gnanasakthy | Expert Review of Pharmacoeconomics & Outcomes Research | 2013-08-00 Aug 2013 | Release of the US FDA patient-reported outcome (PRO) guidance raised expectations within the pharmaceutical industry for the use of PRO measures in support of labeling claims. The FDA developed the guidance with admirable intent, and the recommendations within this document are based on sound scientific principles. However, implementation of the guidance has been somewhat inconsistent within the Study Endpoints and Label Development (SEALD) and across the various FDA-reviewing divisions. Industry and regulatory bodies need to work toward gaining common ground to best support registration of treatments that could extend patients' lives, reduce symptoms, and/or improve health-related quality of life. PROs are valuable tools in communicating these messages, and realistic implementation of the FDA PRO Guidance may truly facilitate this process. | Humans | Patient Participation | United States | Evidence-based medicine | OK |
| Tara | Lamond | Randi M. | Williams | Marc D. | Schwartz | Behavioral Medicine (Washington, D.C.) | 2010-12-00 2010 Oct-Dec | The survival advantages associated with different treatments for localized prostate cancer (PCa) continue to be uncertain. We evaluated patients' use of an interactive CD-ROM-based decision aid designed to improve informed decision making about PCa treatment. Newly diagnosed, early-stage PCa patients who had not made a treatment decision completed a baseline telephone interview (N = 132), were mailed the CD-ROM, and completed a one-month follow-up interview (N = 120; 91%). Compared to non-users (21%), CD-users (79%) preferred to make an independent rather than a shared treatment decision (OR = 3.5, CI 1.2,10.5). The majority of users (63%-90%) responded positively regarding the length and clarity of the information. Further, 76% reported using the CD as much/more than other information sources. A preference for having less decisional control predicted greater satisfaction with the CD (F[7,87] = 4.75, p < .05). Electronic utilization data revealed that the topics most accessed concerned treatment information and that users spent over an hour using the CD (median = 72 minutes). This electronic educational tool was well-accepted by patients and may be particularly useful for patients who desire less control over their treatment decisions and who are less proactive in seeking information on their own. | Male | Humans | Patient Participation | Questionnaires | OK |
| Mark J. | Mihalyo | James | Schuster | Patricia E. | Deegan | Community Mental Health Journal | 2013-04-00 avril 2013 | Healthcare reform emphasizes patient-centered care and shared decision-making. This study examined the impact on psychotropic adherence of a decision support center and computerized tool designed to empower and activate consumers prior to an outpatient medication management visit. Administrative data were used to identify 1,122 Medicaid-enrolled adults receiving psychotropic medication from community mental health centers over a two-year period from community mental health centers. Multivariate linear regression models were used to examine if tool users had higher rates of 180-day medication adherence than non-users. Older clients, Caucasian clients, those without recent hospitalizations, and those who were Medicaid-eligible due to disability had higher rates of 180-day medication adherence. After controlling for sociodemographics, clinical characteristics, baseline adherence, and secular changes over time, using the computerized tool did not affect adherence to psychotropic medications. The computerized decision tool did not affect medication adherence among clients in outpatient mental health clinics. Additional research should clarify the impact of decision-making tools on other important outcomes such as engagement, patient-prescriber communication, quality of care, self-management, and long-term clinical and functional outcomes. (PsycINFO Database Record (c) 2013 APA, all rights reserved). (journal abstract) | Decision Making | computerized medication | community mental health services | psychotropic medication | Doublon |
| Mark J. | Mihalyo | James | Schuster | Patricia E. | Deegan | Community Mental Health Journal | 2013-04-00 Apr 2013 | Healthcare reform emphasizes patient-centered care and shared decision-making. This study examined the impact on psychotropic adherence of a decision support center and computerized tool designed to empower and activate consumers prior to an outpatient medication management visit. Administrative data were used to identify 1,122 Medicaid-enrolled adults receiving psychotropic medication from community mental health centers over a two-year period from community mental health centers. Multivariate linear regression models were used to examine if tool users had higher rates of 180-day medication adherence than non-users. Older clients, Caucasian clients, those without recent hospitalizations, and those who were Medicaid-eligible due to disability had higher rates of 180-day medication adherence. After controlling for sociodemographics, clinical characteristics, baseline adherence, and secular changes over time, using the computerized tool did not affect adherence to psychotropic medications. The computerized decision tool did not affect medication adherence among clients in outpatient mental health clinics. Additional research should clarify the impact of decision-making tools on other important outcomes such as engagement, patient-prescriber communication, quality of care, self-management, and long-term clinical and functional outcomes. | Male | Decision Support Techniques | Humans | Patient Participation | OK |
| Scott A. | Turske | Terrisca R. | Des Jardins | Hossein | Yarandi | Journal of Medical Internet Research | 2013-00-00 2013 | BACKGROUND: There are an estimated 25.8 million American children and adults, equivalent to 8.3% of the US population, living with diabetes. Diabetes is particularly burdensome on minority populations. The use of mobile technologies for reaching broad populations is a promising approach, given its wide footprint and ability to deliver inexpensive personalized messages, to increase awareness of type 2 diabetes and promote behavior changes targeting risk factors associated with type 2 diabetes. As a part of the Beacon Community Cooperative Agreement Program, txt4health, a public-facing mobile health information service, was launched in 3 Beacon Communities: the Southeast Michigan Beacon Community in Detroit, MI, the Greater Cincinnati Beacon Community in Cincinnati, OH, and the Crescent City Beacon Community in New Orleans, LA. Txt4health is a mobile health information service designed to help people understand their risk for type 2 diabetes and become more informed about the steps they can take to lead healthy lives. OBJECTIVE: The purpose of this investigation was to use the RE-AIM framework to document txt4health reach and adoption by focusing on enrollment and participant engagement in program pilots in Southeast Michigan and Greater Cincinnati. METHODS: We conducted a retrospective records analysis of individual-level txt4health system data from participants in Southeast Michigan and Greater Cincinnati to determine participant usage of txt4health and engagement with the program. RESULTS: Results from the retrospective records analysis revealed that 5570 participants initiated the 2-step enrollment process via 1 of 3 enrollment strategies: text message, website, or directly with Beacon staff who signed participants up via the website. In total, 33.00% (1838/5570) of participants completed the 2-step enrollment process and were fully enrolled in the program. All participants (100.00%, 1620/1620) who enrolled via text message completed the entire 2-step enrollment process versus 5.52% (218/3950) of participants who enrolled via website or a Beacon staff member. Of those who fully enrolled, 71.00% (1305/1838) completed the diabetes risk assessment and 74.27% (1365/1838) set an initial weight loss goal. Overall, 39.06% (718/1838) of participants completed all 14 weeks of the program and 56.26% (1034/1838) dropped out before completing all 14 weeks, with the bulk of dropouts occurring in the first 4 weeks. Length of participation varied greatly, ranging from 0-48.7 weeks (median 8.6, mean 15.8, SD 15.8). Wide variability of participant engagement in regards to weekly weight and physical activity was documented. CONCLUSIONS: Although broadly focused public health text message interventions may have the potential to reach large populations and show high levels of engagement among some users, the level of individual engagement among participants varies widely, suggesting that this type of approach may not be appropriate for all. | Male | Humans | Patient Participation | United States | OK |
|  |  |  |  |  |  | The Patient | 2014-07-22 Jul 22, 2014 | OBJECTIVE: Our aim was to facilitate shared decision making (SDM) during preventive visits by utilizing a web-based survey system to offer colorectal cancer (CRC) and prostate cancer screening decision aids (DAs) to appropriately identified patients prior to the visit. METHODS: Patients completed a web-based questionnaire before their preventive medicine appointment. Age- and gender-appropriate patients completed additional questions to determine eligibility for CRC or prostate-specific antigen (PSA) screening. Eligible patients were offered a choice of video or print DA, and completed questions assessing their knowledge, values, and preferences regarding the screening decision. Responses were summarized and fed forward to clinician and patient reports. RESULTS: Overall, 11,493 CRC and 4,384 PSA questionnaires were completed. Patient responses were used to identify those eligible for cancer-screening DAs: 2,187 (19 %) for CRC and 2,962 (68 %) for PSA; 15 % of eligible patients requested a DA. Many patients declined a DA because they indicated they "already know enough to make their decision" (34 % for CRC, 46 % for PSA). CONCLUSION: A web-based questionnaire provides an efficient means to identify patients eligible for cancer screening decisions and to offer them DAs before an appointment. Pre-visit use of DAs along with reports giving feedback to patients and clinicians provides an opportunity for SDM to occur at the visit. | | | | | OK |
| S. | Borbé | T. | Steinert |  |  | Der Nervenarzt | 2012-05-00 May 2012 | OBJECTIVE: Joint crisis plans in psychiatry are consensus-oriented instruments for patients to state their will in case of a crisis. The aim of this survey is to determine the prevalence, practice and barriers for implementation of joint crisis plans in Germany. METHODS: In spring 2009, 366 psychiatric hospitals in Germany were asked to complete a questionnaire. RESULTS: The return rate was 46.4%; 68% of the participating hospitals offered joint crisis plans. The number of completed joint crisis plans was low (median 2.7/year per clinic). The demand did not increase according to a majority of the hospitals. The main reason for hospitals to abstain from joint crisis plans was low demand. Hospitals providing the option of joint crisis plans reported positive experiences. CONCLUSIONS: A theoretical offer of joint crisis plans is widespread in German psychiatric hospitals. Despite positive experiences, patients do not ask for them frequently. | Humans | Patient Participation | Health Surveys | Advance Directives | OK |
| Claire | Weston | Richard | Baker |  |  | Journal of the Royal Society of Medicine | 2011-10-00 Oct 2011 | OBJECTIVES: To investigate the relationship between patient experience assessed through surveys of random samples of practice populations and intermediate outcome targets in those patients with diabetes, collected in the Quality and Outcomes Framework pay-for-performance scheme. DESIGN: Cross-sectional study. SETTING: The East Midlands region of England. PARTICIPANTS: Six hundred and twenty-nine general practices. MAIN OUTCOME MEASURES: Logistic regression models were used to assess whether practice-level reports of patient experience of access and consultations were associated with achievement of treatment targets (HbA1c of 7.5% and 10% or lower, BP 145/85 mmHg or lower, and cholesterol 5 mmol/L or lower) in people with diabetes. Survey respondent characteristics (ethnicity, age, sex) and practice size, deprivation, and prevalence of diabetes and obesity were also assessed within the models. RESULTS: Patient experience of practice populations explained little of the variation in diabetes treatment targets. In the practice survey, the proportion of respondents who had seen a nurse in the last 6 months was associated with increased likelihood of achieving HbA1c of 7.5%, and being involved in decision-making or having tests and treatment explained were associated with achievement of HbA1c of 10% or less, cholesterol of 5 mmol/L or less, and BP of 145/85 or less. CONCLUSIONS: Although patient experience at practice level should be included in monitoring outcomes, it should not replace monitoring clinical outcomes in diabetes. A mix of clinical and patient experience measures will have to be used to monitor outcomes in general practice. | Male | Decision Making | Humans | Patient Participation | OK |
|  |  |  |  |  |  | Military Medicine | 2013-10-00 octobre 2013 | Objectives: The primary aim of this review was to compare and contrast the principles of the Assessment-Diagnosis-Treatment-Outcomes (ADTO) model with clinical prediction rules, shared decision making, and practice guidelines and provide a framework for validating medical simulations. Methods: Comparing and contrasting the various methods and tools used by the clinical community and patients to help make informed decisions regarding health care options. Results: The ADTO model provides an excellent theoretical framework to analyze and conceptualize the interrelationships between clinicians and patients as they go through the process of determining best practices. However, the ADTO model may be too comprehensive to implement within any given clinical prediction rule or hypothetical-construct-oriented medical simulation, at least at this point in the development of these fields. Conclusions: The way forward for developing substantive medical simulations that will tie together complex constructs is to start at the beginning and plan to make small steps as a basis for establishing the infrastructure that will lead to a giant leap for mankind. (PsycINFO Database Record (c) 2014 APA, all rights reserved). (journal abstract) | Measurement | Decision Making | Treatment | Health Care Services | Doublon |
|  |  |  |  |  |  | Military Medicine | 2013-10-00 Oct 2013 | OBJECTIVES: The primary aim of this review was to compare and contrast the principles of the Assessment-Diagnosis-Treatment-Outcomes (ADTO) model with clinical prediction rules, shared decision making, and practice guidelines and provide a framework for validating medical simulations. METHODS: Comparing and contrasting the various methods and tools used by the clinical community and patients to help make informed decisions regarding health care options. RESULTS: The ADTO model provides an excellent theoretical framework to analyze and conceptualize the interrelationships between clinicians and patients as they go through the process of determining best practices. However, the ADTO model may be too comprehensive to implement within any given clinical prediction rule or hypothetical-construct-oriented medical simulation, at least at this point in the development of these fields. CONCLUSIONS: The way forward for developing substantive medical simulations that will tie together complex constructs is to start at the beginning and plan to make small steps as a basis for establishing the infrastructure that will lead to a giant leap for mankind. | Decision Making | Decision Support Techniques | Humans | patient care | OK |
| Carrie A. | Levin | Floyd J. | Fowler | Angela | Fagerlin | Medical Decision Making: An International Journal of the Society for Medical Decision Making | 2010-10-00 2010 Sep-Oct | BACKGROUND: The rise in Internet use for seeking health information raises questions about the role the Internet may play in how patients make medical decisions. OBJECTIVE: To examine Internet use and perceived importance of different sources of information by patients making 9 specific medical decisions covering prescription medication initiation, cancer screening, and elective surgery. SETTING: National sample of US adults identified by random-digit dialing. DESIGN: Cross-sectional survey conducted between November 2006 and May 2007. PARTICIPANTS: The final sample comprised 2575 English-speaking US adults aged 40 y and older who had either undergone 1 of 9 medical procedures or tests or talked with a health care provider about doing so during the previous 2 y. MEASUREMENTS: Participants indicated if they or other family members used the Internet to seek information related to each of the specific medical decisions and rated how important the health care provider, the Internet (if used), family and friends, and the media (newspapers, magazines, and television) were in providing information to help make the medical decision. RESULTS: Use of the Internet for information related to specific decisions among adults 40 y and older was generally low (28%) but varied across decisions, from 17% for breast cancer screening to 48% for hip/knee replacement. Internet use was higher at younger ages, rising from 14% among those aged 70 y and older to 38% for those aged 40 to 49 y. Internet users consistently rated health care providers as the most influential source of information for medical decisions, followed by the Internet, family and friends, and media. LIMITATIONS: Telephone surveys are limited by coverage and nonresponse. The authors excluded health-related Internet use not associated with the 9 target decisions. CONCLUSIONS: A minority of patients reported using the Internet to make specific common medical decisions, but use varied widely by type of decision. Perhaps reflecting perceived risk and uncertainty, use was lowest for screening decisions and highest for surgical decisions. | Male | Humans | Patient Participation | Questionnaires | OK |
| Anneke T. | Schroen | Craig L. | Slingluff |  |  | Clinical Trials (London, England) | 2013-02-00 Feb 2013 | BACKGROUND: Performing biopsies for correlative studies in cancer trials raises ethical and regulatory concerns and may impact trial accrual negatively. However, strategies to address these concerns remain largely unexplored. PURPOSE: We sought to assess the perceived risk of mandatory tissue biopsies to be performed for research purposes as part of a clinical trial of a melanoma vaccine by administering a pretrial accrual assessment questionnaire in the population of interest. Furthermore, we explored how such survey data may be used to address potential concerns of regulatory and funding organizations that may not be able to assess the risks of those biopsies. Method A total of 91 melanoma patients, similar to potential participants in a melanoma vaccine pilot study, scored their willingness, on a 9-point Likert scale, to participate in vaccine trials involving no skin biopsy versus a skin biopsy resulting in a 3-, 6-, or 12-cm scar. The vaccine trial was performed with skin biopsies leaving a 6-cm scar. Accrual rate was assessed and that accrual was compared to the accrual of two similar vaccine trials without biopsy requirements. RESULTS: A total of 95% of the participants expressed willingness to enter a vaccine trial (likely to highly likely). This proportion decreased to 74%, 63%, and 59%, respectively, for vaccine trials requiring skin biopsy leaving a 3-, 6-, or 12-cm scar. The trial was designed with an estimated 40% decrease in accrual rate compared to prior studies (2 participants expected/month). The resulting trial with a 6-cm biopsy exceeded that accrual rate estimate and had a similar accrual rate to vaccine trials without a biopsy (4.1 vs 2.7-4.6 participants/month). LIMITATIONS: Potential limitations of this study include the exclusion of some questionnaire responses and the post hoc nature of the analysis. CONCLUSION: Willingness to participate in vaccine trials was decreased by the requirement for skin biopsy, but the size of the biopsy was less of a deterrent than expected. Findings from brief surveys may aid in risk assessment during regulatory review, predict acceptability of tissue collection for correlative studies, and support regulatory approval and meeting accrual goals of the study. | Humans | Patient Participation | Questionnaires | Informed Consent | OK |
| Anant R. C. | Patel | Paul W. | Jones | John R. | Hurst | American Journal of Respiratory and Critical Care Medicine | 2012-06-01 Jun 1, 2012 | RATIONALE: The Chronic Obstructive Pulmonary Disease (COPD) Assessment Test (CAT) is an eight-item questionnaire designed to assess and quantify the impact of COPD symptoms on health status. COPD exacerbations impair quality of life and are characterized by worsening respiratory symptoms from the stable state. We hypothesized that CAT scores at exacerbation relate to exacerbation severity as measured by exacerbation duration, lung function impairment, and systemic inflammation. OBJECTIVES: To evaluate the usefulness of the CAT to assess exacerbation severity. METHODS: One hundred sixty-one patients enrolled in the London COPD cohort completed the CAT at baseline (stable state), exacerbation, and during recovery between April 2010 and June 2011. MEASUREMENTS AND MAIN RESULTS: Frequent exacerbators had significantly higher baseline CAT scores than infrequent exacerbators (19.5 ± 6.6 vs. 16.8 ± 8.0, P = 0.025). In 152 exacerbations, CAT scores rose from an average baseline value of 19.4 ± 6.8 to 24.1 ± 7.3 (P < 0.001) at exacerbation. Change in CAT score from baseline to exacerbation onset was significantly but weakly related to change in C-reactive protein (rho = 0.26, P = 0.008) but not to change in fibrinogen (rho = 0.09, P = 0.351) from baseline to exacerbation. At exacerbation, rises in CAT score were significantly associated with falls in FEV(1) (rho = -0.20, P = 0.032). Median recovery time as judged by symptom diary cards was significantly related to the time taken for the CAT score to return to baseline (rho = 0.42, P = 0.012). CONCLUSIONS: The CAT provides a reliable score of exacerbation severity. Baseline CAT scores are elevated in frequent exacerbators. CAT scores increase at exacerbation and reflect severity as determined by lung function and exacerbation duration. | Male | Humans | Patient Participation | Questionnaires | OK |
| Aslaug | Mikkelsen |  |  |  |  | Journal of Clinical Nursing | 2010-07-00 Jul 2010 | AIMS: This study presents development, empirical testing and validation of an instrument measuring service user involvement in in-patient mental health from the mental health professionals' perspective. BACKGROUND: Service user involvement is high on the agenda in European mental health policies. In Norway, focus is on enhanced service user involvement at both the individual and organisational levels of in-patient mental health services. Mental health professionals are in an important position to ensure opportunities for real user involvement in in-patient mental health care. However, there is a need for more empirical knowledge on how mental health professionals attend to service user involvement. DESIGN: Survey. METHODS: A self-report questionnaire was designed and administered to 121 mental health professionals, with 98 responses, working in a community-based mental health centre in western Norway. Factor analysis procedures together with reliability testing were performed. RESULTS: A 30-items instrument was developed. The instrument contains four components/subscales: (1) Democratic patient involvement (mean score 3·74, Cronbach's alpha 0·81), (2) Carer involvement (mean score 3·67, Cronbach's alpha 0·82), (3) Assisted patient involvement (mean score 4·05, Cronbach's alpha 0·78) and (4) Management support (mean score 4·10, Cronbach's alpha 0·75). These subscales were found to be essential to service user involvement in the context of in-patient mental health care. The total mean score for the instrument was 3·88, Cronbach's alpha 0·88. CONCLUSION: Empirical testing of the instrument demonstrates that the measurement of mental health professionals' perception of service user involvement has a reasonable level of construct validity and reliability. RELEVANCE TO CLINICAL PRACTICE: We have developed a measurement instrument with items reflecting essential characteristics to user involvement in in-patient mental health services. We believe that answering this questionnaire on the subject user involvement can act as one step towards enhancing awareness of this issue and to assess user-oriented practices in treatment and services. | Male | Humans | Patient Participation | Questionnaires | OK |
| Per-Olof | Ostergren | A. S. M. Atiqur | Rahman | Nazmul | Bari | BMC health services research | 2012-00-00 2012 | BACKGROUND: Knowledge about the relation between user involvement in the provision of assistive technology and outcomes of assistive technology use is a prerequisite for the development of efficient service delivery strategies. However, current knowledge is limited, particularly from low-income countries where affordability is an issue. The objective was therefore to explore the relation between outcomes of assistive technology use and user involvement in the service delivery process in Bangladesh. METHODS: Using structured interviews, data from 136 users of hearing aids and 149 users of manual wheelchairs were collected. Outcomes were measured using the International Outcome Inventory for Hearing Aids (IOI-HA), which was adapted for wheelchair users. Predictors of user involvement included preference, measurement and training. RESULTS: Users reported outcomes comparable to those found in other high- and low-income countries. User involvement increased the likelihood for reporting better outcomes except for measurement among hearing aid users. CONCLUSIONS: The findings support the provision of assistive technology as a strategy to improve the participation of people with disabilities in society. They also support current policies and guidelines for user-involvement in the service delivery process. Simplified strategies for provision of hearing aids may be explored. | Male | Humans | Patient Participation | Questionnaires | OK |
| Pål | Gjerden |  |  |  |  | Tidsskrift for Den Norske Lægeforening: Tidsskrift for Praktisk Medicin, Ny Række | 2012-03-06 Mar 6, 2012 | | Humans | Patient Participation | Questionnaires | Psychotropic Drugs | OK |
| Adrian | Edwards | France | Légaré | Robert | Newcombe | Patient Education and Counseling | 2013-11-00 novembre 2013 | Objective: To propose a revised Observer OPTION measure of shared decision making. Methods: We analyzed published models to identify the core components of a parsimonious conceptual framework of shared decision making. By using this framework, we developed a revised measure combining data from an observational study of clinical practice in Canada with our experience of using Observer OPTION12 Item. Results: Our conceptual framework for shared decision making composed of justifying deliberative work, followed by the steps of describing options, information exchange, preference elicitation, and preference integration. By excluding items in Observer OPTION12 Item that were seldom observed or not aligned to a robust construct, we propose Observer OPTION5 Item. Conclusion: Although widely used, Observer OPTION12 Item did not give sufficient attention to preference elicitation and integration, and included items that were not specific to a core construct of shared decision making. We attempted to remedy these shortcomings by proposing a shorter, more focused measure. Practice implications: Observer OPTION5 Item requires evaluation; we hope that it will be useful as both a research tool and as a formative measure of clinical practice. (PsycINFO Database Record (c) 2014 APA, all rights reserved). (journal abstract) | Shared decision making | Decision Making | physicians | Psychometrics | OK |
| Matthew | Cooperberg | Michael | Goodman | John J. | Graff | Journal of Comparative Effectiveness Research | 2013-07-00 Jul 2013 | BACKGROUND: While randomized controlled trials represent the highest level of evidence we can generate in comparative effectiveness research, there are clinical scenarios where this type of study design is not feasible. The Comparative Effectiveness Analyses of Surgery and Radiation in localized prostate cancer (CEASAR) study is an observational study designed to compare the effectiveness and harms of different treatments for localized prostate cancer, a clinical scenario in which randomized controlled trials have been difficult to execute and, when completed, have been difficult to generalize to the population at large. METHODS: CEASAR employs a population-based, prospective cohort study design, using tumor registries as cohort inception tools. The primary outcome is quality of life after treatment, measured by validated instruments. Risk adjustment is facilitated by capture of traditional and nontraditional confounders before treatment and by propensity score analysis. RESULTS: We have accrued a diverse, representative cohort of 3691 men in the USA with clinically localized prostate cancer. Half of the men invited to participate enrolled, and 86% of patients who enrolled have completed the 6-month survey. CONCLUSION: Challenging comparative effectiveness research questions can be addressed using well-designed observational studies. The CEASAR study provides an opportunity to determine what treatments work best, for which patients, and in whose hands. | Male | Humans | Patient Participation | Questionnaires | OK |
| Adrian | Edwards | France | Légaré | Robert | Newcombe | Patient Education and Counseling | 2013-11-00 Nov 2013 | OBJECTIVE: To propose a revised Observer OPTION measure of shared decision making. METHODS: We analyzed published models to identify the core components of a parsimonious conceptual framework of shared decision making. By using this framework, we developed a revised measure combining data from an observational study of clinical practice in Canada with our experience of using Observer OPTION(12 Item). RESULTS: Our conceptual framework for shared decision making composed of justifying deliberative work, followed by the steps of describing options, information exchange, preference elicitation, and preference integration. By excluding items in Observer OPTION(12 Item) that were seldom observed or not aligned to a robust construct, we propose Observer OPTION(5 Item). CONCLUSION: Although widely used, Observer OPTION(12 Item) did not give sufficient attention to preference elicitation and integration, and included items that were not specific to a core construct of shared decision making. We attempted to remedy these shortcomings by proposing a shorter, more focused measure. PRACTICE IMPLICATIONS: Observer OPTION(5 Item) requires evaluation; we hope that it will be useful as both a research tool and as a formative measure of clinical practice. | Decision Making | Humans | Psychometrics | Models, Theoretical | Doublon |
| Adrian | Edwards | France | Légaré | Robert | Newcombe | Patient Education and Counseling | 2013-11-00 Nov 2013 | OBJECTIVE: To propose a revised Observer OPTION measure of shared decision making. METHODS: We analyzed published models to identify the core components of a parsimonious conceptual framework of shared decision making. By using this framework, we developed a revised measure combining data from an observational study of clinical practice in Canada with our experience of using Observer OPTION(12 Item). RESULTS: Our conceptual framework for shared decision making composed of justifying deliberative work, followed by the steps of describing options, information exchange, preference elicitation, and preference integration. By excluding items in Observer OPTION(12 Item) that were seldom observed or not aligned to a robust construct, we propose Observer OPTION(5 Item). CONCLUSION: Although widely used, Observer OPTION(12 Item) did not give sufficient attention to preference elicitation and integration, and included items that were not specific to a core construct of shared decision making. We attempted to remedy these shortcomings by proposing a shorter, more focused measure. PRACTICE IMPLICATIONS: Observer OPTION(5 Item) requires evaluation; we hope that it will be useful as both a research tool and as a formative measure of clinical practice. | Decision Making | Humans | Psychometrics | Models, Theoretical | Doublon |
| Andrew | Davis | Kristine | Bordenave | Stephen | Brown | Journal of Clinical Hypertension (Greenwich, Conn.) | 2012-01-00 Jan 2012 | The objective of this study was to determine whether a videoconference-based telehealth network can increase hypertension management knowledge and self-assessed competency among primary care providers (PCPs) working in urban Federally Qualified Health Centers (FQHCs). We created a telehealth network among 6 urban FQHCs and our institution to support a 12-session educational program designed to teach state-of-the-art hypertension management. Each 1-hour session included a brief lecture by a university-based hypertension specialist, case presentations by PCPs, and interactive discussions among the specialist and PCPs. Twelve PCPs (9 intervention and 3 controls) were surveyed at baseline and immediately following the curriculum. The mean number of correct answers on the 26-item hypertension knowledge questionnaire increased in the intervention group (13.11 [standard deviation (SD)]=3.06) to 17.44 [SD=1.59], P<.01) but not among controls (14.33 [SD=3.21] to 13.00 [SD=3.46], P=.06). Similarly, the mean score on a 7-item hypertension management self-assessed competency scale increased in the intervention group (4.68 [SD=0.94] to 5.41 [SD=0.89], P<.01) but not among controls (5.28 [SD=0.43] to 5.62 [SD=0.67], P=.64). This model holds promise for enhancing hypertension care provided by urban FQHC providers. | Male | Humans | Patient Participation | Questionnaires | OK |
| Cayce C. | Hughes | Kenya F. | Hunter | Frances K. | Barg | Patient Education and Counseling | 2011-08-00 Aug 2011 | OBJECTIVE: To compare and contrast notions of ADHD among pediatricians and parents of affected children to understand the perspectives they bring to shared decision making (SDM). METHODS: In this freelisting study, 60 parents of children with ADHD and 30 primary care pediatricians listed words reflecting their understanding of (1) Attention Deficit Hyperactivity Disorder (ADHD), (2) getting/offering help for ADHD, (3) talking to doctors/families about ADHD, and (4) "mental health." Smith's salience score established terms that were salient and cultural consensus analysis identified variation within subgroups of participants. RESULTS: Parents' terms reflected ADHD's effects on the child and family, while clinicians often mentioned school. Lists suggested differing needs and goals for clinicians and subgroups of parents in SDM: "time" for clinicians, "learning" and "understanding" for non-college educated parents, and "comfort" and "relief" for college educated parents. Neither parents nor clinicians framed ADHD in the same way as "mental health." CONCLUSION: Parents and clinicians, who conceptualize ADHD differently, should negotiate a shared understanding of ADHD as a basis for SDM. Treatment discussions should be tailored to encompass families' varied emotional and educational needs. PRACTICE IMPLICATIONS: Fostering SDM in primary care is consonant with notions of ADHD as distinct from mental health. | Male | Decision Making | Humans | Questionnaires | OK |
|  |  |  |  |  |  | The primary care companion for CNS disorders | 2013-00-00 2013 | |  |  |  |  | OK |
| Yves | de Roten | Jean-Nicolas | Despland | Franz | Caspar | Clinical Psychology & Psychotherapy | 2013-02-00 2013 Jan-Feb | A comprehensive understanding of the patient's problems is essential for a constructive therapeutic behaviour, especially in borderline personality disorder (BPD) where difficult interpersonal patterns are persistent. In these circumstances, the use of an integrative case formulation approach such as Plan Analysis, developed by K. Grawe and F. Caspar, can be of help for therapy planning. The focus here is on instrumental relations between behaviours and the hypothetical Plans and motives 'behind' those behaviours. The present qualitative study aimed at setting a prototypical Plan structure for n = 15 patients presenting a Diagnostic and Statistical Manual of Mental Disorders, Fourth Edition, diagnosis of BPD using Plan Analysis. The first psychotherapeutic session of every outpatient was video-taped and evaluated according to the Plan Analysis procedure. Inter-rater reliability was established between two independent raters and was considered sufficient. The detailed prototypical Plan structure of BPD showed two main tendencies: first, the important presence of support-seeking among these patients and second, the will to be in control and to protect oneself. This study confirms the existence of several core similarities in the functioning of patients with BPD. These findings are in line with earlier studies and expand the latter with the aim of contributing to the understanding of BPD psychopathology. Clinical implications are discussed. | Male | Humans | Patient Participation | Questionnaires | OK |
|  |  |  |  |  |  | Current HIV research | 2012-09-00 Sep 2012 | There is a pressing need to find an efficacious HIV vaccine and a concomitant need for the recruitment of participants in efficacy trials. These efforts are hampered, however, by a gap between what respondents say they will do regarding research participation, and whether they actually enroll. The current paper examines the size of this gap and proposes psychological reasons for it. Some reasons include the temporal stability of the intention, the time taken to consider its ramifications and plans to deal with them, and the social forces that affect the intention. From this analysis, recommendations are offered to improve recruitment efforts and the predictive power of expressions of willingness to participate. | Male | Humans | Patient Participation | Female | OK |
| William F. | Fadel | Marianne S. | Matthias | Michelle P. | Salyers | Simulation in Healthcare: Journal of the Society for Simulation in Healthcare | 2014-12-00 Dec 2014 | INTRODUCTION: Sociodemographic differences have been observed in the treatment of extremely premature (periviable) neonates, but the source of this variation is not well understood. We assessed the feasibility of using simulation to test the effect of maternal race and insurance status on shared decision making (SDM) in periviable counseling. METHODS: We conducted a 2 × 2 factorial simulation experiment in which obstetricians and neonatologists counseled 2 consecutive standardized patients diagnosed with ruptured membranes at 23 weeks, counterbalancing race (black/white) and insurance status using random permutation. We assessed verisimilitude of the simulation in semistructured debriefing interviews. We coded physician communication related to resuscitation, mode of delivery, and steroid decisions using a 9-point SDM coding framework and then compared communication scores by standardized patient race and insurer using analysis of variance. RESULTS: Sixteen obstetricians and 15 neonatologists participated; 71% were women, 84% were married, and 75% were parents; 91% of the physicians rated the simulation as highly realistic. Overall, SDM scores were relatively high, with means ranging from 6.4 to 7.9 (of 9). There was a statistically significant interaction between race and insurer for SDM related to steroid use and mode of delivery (P < 0.01 and P = 0.01, respectively). Between-group comparison revealed nonsignificant differences (P = <0.10) between the SDM scores for privately insured black patients versus privately insured white patients, Medicaid-insured white patients versus Medicaid-insured black patients, and privately insured black patients versus Medicaid-insured black patients. CONCLUSIONS: This study confirms that simulation is a feasible method for studying sociodemographic effects on periviable counseling. Shared decision making may occur differentially based on patients' sociodemographic characteristics and deserves further study. | | | | | OK |
|  |  |  |  |  |  | Healthcare Executive | 2011-06-00 2011 May-Jun | | Humans | Patient Participation | Internet | Community-Institutional Relations | OK |
| Melissa | Coulson | Mary | Sarigiannis |  |  | The British Journal of Occupational Therapy | 2014-02-00 février 2014 | Introduction: Measuring change in clients' 'participation', and being able to attribute this change to occupational therapy intervention, is important in the quest to provide evidence to support practice. The Australian Therapy Outcome Measures for Occupational Therapy is an outcome measure that has four domains, one of which is concerned with measuring client participation. An occupational therapy service wanted to determine if client level of participation was improving, as measured by the Australian Therapy Outcome Measures for Occupational Therapy. Method: Data were collected between 2009 and 2012 from three home-based therapy services, using the Australian Therapy Outcome Measures for Occupational Therapy. Data were divided into diagnostic cohorts, and then exploratory data analysis techniques and paired t-tests were performed. Findings: Data from 506 participants were analysed and clients in all diagnostic groups made statistically (p &lt; .05) significant improvements over time in relation to their participation. Conclusion: Most clients in this study improved or maintained their level of participation between admission and discharge, with few clients deteriorating, when measured on the Australian Therapy Outcome Measures for Occupational Therapy scales. These results suggest the home-based therapy service was meeting its goal, and further supports the responsiveness of the Australian Therapy Outcome Measures for Occupational Therapy to detect change in clients' participation status over time. Randomized control trials are needed to determine whether improvements in participation are due to engaging in the occupational therapy program. (PsycINFO Database Record (c) 2014 APA, all rights reserved). (journal abstract) | Client Participation | Clients | treatment outcomes | therapy outcomes | OK |
| Jeuneviette | Bontemps-Jones | Joseph E. | Bauer | Jeanine | Bade | Journal of Medical Internet Research | 2011-00-00 2011 | BACKGROUND: Most smokers attempt to quit on their own even though cessation aids can substantially increase their chances of success. Millions of smokers seek cessation advice on the Internet, so using it to promote cessation products and services is one strategy for increasing demand for treatments. Little is known, however, about what cessation aids these smokers would find most appealing or what predicts their preferences (eg, age, level of dependence, or timing of quit date). OBJECTIVE: The objective of our study was to gain insight into how Internet seekers of cessation information make judgments about their preferences for treatments, and to identify sociodemographic and other predictors of preferences. METHODS: An online survey assessing interest in 9 evidence-based cessation products and services was voluntarily completed by 1196 smokers who visited the American Cancer Society's Great American Smokeout (GASO) webpage. Cluster analysis was conducted on ratings of interest. RESULTS: In total, 48% (572/1196) of respondents were "quite a bit" or "very much" interested in nicotine replacement therapy (NRT), 45% (534/1196) in a website that provides customized quitting advice, and 37% (447/1196) in prescription medications. Only 11.5% (138/1196) indicated similar interest in quitlines, and 17% (208/1196) in receiving customized text messages. Hierarchical agglomerative cluster analysis revealed that interest in treatments formed 3 clusters: interpersonal-supportive methods (eg, telephone counseling, Web-based peer support, and in-person group programs), nonsocial-informational methods (eg, Internet programs, tailored emails, and informational booklets), and pharmacotherapy (NRT, bupropion, and varenicline). Only 5% (60/1196) of smokers were "quite a bit" or "very much" interested in interpersonal-supportive methods compared with 25% (298/1196) for nonsocial-informational methods and 33% (399/1196) for pharmacotherapy. Multivariate analyses and follow-up comparisons indicated that level of interest in pharmacotherapy ("quite a bit or "very much" vs. "not at all") varied as a function of education (n = 575, χ(2) (3) =16.6, P = .001), age (n = 528, χ(2) (3) = 8.2, P = .04), smoking level (n = 514, χ(2) (3) = 9.5, P = .02), and when smokers were planning to quit (n = 607, χ(2) (4) = 34.0, P < .001). Surprisingly, greater age was associated with stronger interest in nonsocial-informational methods (n = 367, χ(2) (3) = 10.8, P = .01). Interest in interpersonal-supportive methods was greater if smokers had used a quitline before (n = 259, χ(2) (1) = 18.3, P < .001), or were planning to quit earlier rather than later (n = 148, χ(2) (1) = 4.9, P = .03). CONCLUSIONS: Smokers accessing the Internet for information on quitting appear to differentiate cessation treatments by how much interpersonal interaction or support the treatment entails. Quitting date, smoking level, and sociodemographic variables can identify smokers with varying levels of interest in the 3 classes of cessation methods identified. These results can potentially be used to more effectively target and increase demand for these treatments among smokers searching the Internet for cessation information. | Humans | Patient Participation | Patient Satisfaction | Questionnaires | OK |
| Alexandra L. | Quittner |  |  |  |  | Paediatric Respiratory Reviews | 2013-09-00 Sep 2013 | Patient-reported outcomes (PROs) have been successfully developed for a variety of chronic respiratory diseases, such as asthma and cystic fibrosis (CF). They have recently been used to evaluate the efficacy of new medications and assess current patient functioning. Although regulatory bodies have favored PROs that measures symptoms, other domains of functioning, such as treatment burden, should be considered. This review examines current guidelines for the development and application of PROs in clinical trials, describes methods for selecting appropriate measures for paediatric populations, and presents a model incorporating PROs into clinical practice. Guidance on interpretation of these measures and graphic presentation of results are illustrated. PROs can serve as the link between the health care provider and patient to foster collaborative and personalized medicine. This model promotes greater patient responsibility, facilitates communication with providers, encourages shared decision-making, and enhances adherence. | Humans | Patient Satisfaction | Physician-Patient Relations | Questionnaires | OK |
| Susie | Gagnon | Sandy | Dunn | Pierre | Pluye | Journal of Evaluation in Clinical Practice | 2011-08-00 Aug 2011 | RATIONALE, AIMS AND OBJECTIVES: Following increased interest in having inter-professional (IP) health care teams engage patients in decision making, we developed a conceptual model for an IP approach to shared decision making (SDM) in primary care. We assessed the validity of the model with stakeholders in Canada. METHODS: In 15 individual interviews and 7 group interviews with 79 stakeholders, we asked them to: (1) propose changes to the IP-SDM model; (2) identify barriers and facilitators to the model's implementation in clinical practice; and (3) assess the model using a theory appraisal questionnaire. We performed a thematic analysis of the transcripts and a descriptive analysis of the questionnaires. RESULTS: Stakeholders suggested placing the patient at its centre; extending the concept of family to include significant others; clarifying outcomes; highlighting the concept of time; merging the micro, meso and macro levels in one figure; and recognizing the influence of the environment and emotions. The most common barriers identified were time constraints, insufficient resources and an imbalance of power among health professionals. The most common facilitators were education and training in inter-professionalism and SDM, motivation to achieve an IP approach to SDM, and mutual knowledge and understanding of disciplinary roles. Most stakeholders considered that the concepts and relationships between the concepts were clear and rated the model as logical, testable, having clear schematic representation, and being relevant to inter-professional collaboration, SDM and primary care. CONCLUSIONS: Stakeholders validated the new IP-SDM model for primary care settings and proposed few modifications. Future research should assess if the model helps implement SDM in IP clinical practice. | Male | Decision Making | Humans | Questionnaires | OK |
|  |  |  |  |  |  | Quality of Life Research: An International Journal of Quality of Life Aspects of Treatment, Care and Rehabilitation | 2013-02-00 Feb 2013 | PURPOSE: To provide evidence for the validity of the Fragebogen zur Lebensqualität multimorbider älterer Menschen (FLQM; English: Quality of life in elders with multimorbidity), a new tool for the assessment of life-satisfaction as a subjective indicator of quality of life in old age. METHODS: The FLQM measures overall life-satisfaction based on those self-generated domains in life that are most important to the respondent. Construct validity (correlations with convergent and divergent scales) and differential validity (subgroup differences with respect to age and limitations in everyday functioning) were analyzed in an urban sample of older adults (n = 299; 54.6% male; Mean (age) = 75.65 years, SD = 6.57). RESULTS: Correlations of FLQM with measures of similar constructs were close (r = 0.35-0.50; p < 0.001). There were no age-group differences. However, subjects with self-reported impairment in functioning scored significantly lower on the FLQM. Also, the relationship between FLQM and self-rated health was close (r = 0.44; p < 0.001). CONCLUSIONS: The FLQM is a valid assessment of older peoples' life-satisfaction. The focus on domains of utmost individual importance seems to link the FLQM to self-rated health more closely than other ratings of global life-satisfaction. Further investigation into the qualitative information obtained by the FLQM is desirable in order to explain individual compositions of life-satisfaction and overall quality of life. | Male | Humans | Patient Participation | Questionnaires | OK |
| Greg | Curnew | Sam | Schulman | Eleanor | Pullenayegum | Journal of Population Therapeutics and Clinical Pharmacology = Journal De La Thérapeutique Des Populations Et De La Pharamcologie Clinique | 2013-00-00 2013 | BACKGROUND: Decision aids have been helpful to support patients in decision-making including anticoagulation. With the introduction of new oral anticoagulants (NOACs), it will be important to assist patients and physicians in shared decision-making about NOACs and warfarin. OBJECTIVES: To validate a patient decision aid (DA) for warfarin versus dabigatran, the first NOAC approved for atrial fibrillation (AF). METHODS: Participants without AF and not taking anticoagulants were recruited for the validation exercise. The decision aid described AF, stroke, and hemorrhagic events in terms of incidence, clinical presentation, and prognosis. Warfarin and dabigatran were then compared on multiple clinical and process outcomes as outlined in the pivotal clinical trial. Our primary outcome was confidence in making a treatment decision, using a decisional conflict scale. Secondary outcomes were change in knowledge scores and ratings of clarity, helpfulness and comprehensiveness. RESULTS: 35 patients (mean age 62.7 [SD 9.68], 37.1% female) participated. After use of the decision aid, the mean total decisional conflict score was low at 18.9 (SD: 14.2). Mean knowledge score improved significantly from 4.60 (SD 1.48) to 6.42 (SD 0.80) out of a total score of 7. Only one participant (2.9%) found the decision aid difficult to understand. All 35 participants rated the DA as helpful for making a decision about anticoagulant treatment for AF. Two participants (5.7%) requested more information on adverse effects of the two drugs. CONCLUSION: Our DA to allow patients to make an informed decision with their physician regarding dabigatran versus warfarin in AF, proved understandable, comprehensive and helpful. | Male | Decision Support Techniques | Humans | Female | OK |
| Elizabeth | Kristjansson | Stephen A. | Kearing | Kate F. | Clay | Patient Education and Counseling | 2010-01-00 janvier 2010 | Objective: The Preparation for Decision Making (PrepDM) scale was developed to evaluate decision processes relating to the preparation of patients for decision making and dialoguing with their practitioners. The objective of this study was to evaluate the scale's psychometric properties. Methods: From July 2005 to March 2006, after viewing a decision aid prescribed during routine clinical care, patients completed a questionnaire including: demographic information, treatment intention, decisional conflict, decision aid acceptability, and the PrepDM scale. Results: Four hundred orthopaedic patients completed the questionnaire. The PrepDM scale showed significant correlation with the informed (r = −0.21, p &lt; 0.01) and support (r = −0.13, p = 0.01) subscales (DCS); and discriminated significantly between patients who did and did not find the decision aid helpful (p &lt; 0.0001). Alpha coefficients for internal consistency ranged from 0.92 to 0.96. The scale is strongly unidimensional (principal components analysis) and Item Response Theory analyses demonstrated that all ten scale items function very well. Conclusion: The psychometric properties of the PrepDM scale are very good. Practice Implications: The scale could allow more comprehensive evaluation of interventions designed to prepare patients for shared-decision making encounters regarding complex health care decisions. (PsycINFO Database Record (c) 2014 APA, all rights reserved). (journal abstract) | Decision Making | Patient Participation | Patient Satisfaction | Physician-Patient Relations | Doublon |
| Elizabeth | Kristjansson | Stephen A. | Kearing | Kate F. | Clay | Patient Education and Counseling | 2010-01-00 Jan 2010 | OBJECTIVE: The Preparation for Decision Making (PrepDM) scale was developed to evaluate decision processes relating to the preparation of patients for decision making and dialoguing with their practitioners. The objective of this study was to evaluate the scale's psychometric properties. METHODS: From July 2005 to March 2006, after viewing a decision aid prescribed during routine clinical care, patients completed a questionnaire including: demographic information, treatment intention, decisional conflict, decision aid acceptability, and the PrepDM scale. RESULTS: Four hundred orthopaedic patients completed the questionnaire. The PrepDM scale showed significant correlation with the informed (r=-0.21, p<0.01) and support (r=-0.13, p=0.01) subscales (DCS); and discriminated significantly between patients who did and did not find the decision aid helpful (p<0.0001). Alpha coefficients for internal consistency ranged from 0.92 to 0.96. The scale is strongly unidimensional (principal components analysis) and Item Response Theory analyses demonstrated that all ten scale items function very well. CONCLUSION: The psychometric properties of the PrepDM scale are very good. PRACTICE IMPLICATIONS: The scale could allow more comprehensive evaluation of interventions designed to prepare patients for shared-decision making encounters regarding complex health care decisions. | Male | Decision Making | Decision Support Techniques | Humans | OK |
| David Y. T. | Chen | Laura | Gross | Karen | Ruth | BJU international | 2014-05-00 May 2014 | OBJECTIVE: To validate six previously identified markers among men at increased risk of prostate cancer (African-American men and those with a family history of prostate cancer) enrolled in the Prostate Cancer Risk Assessment Program (PRAP), a prostate cancer screening study. PATIENTS AND METHODS: Eligibility criteria for PRAP include age 35-69 years with a family history of prostate cancer, African-American ethnicity regardless of family history, and known BRCA gene mutations. The genome-wide association study markers assessed included rs2736098 (5p15.33), rs10993994 (10q11), rs10788160 (10q26), rs11067228 (12q24), rs4430796 (17q12) and rs17632542 (19q13.33). Genotyping methods included either the Taqman(®) single nucleotide polymorphism (SNP) genotyping assay (Applied Biosystems, Foster City, CA, USA) or pyrosequencing. Linear regression models were used to evaluate the association between individual markers and log-transformed baseline PSA levels, while adjusting for potential confounders. RESULTS: A total of 707 participants (37% Caucasian, 63% African-American) with clinical and genotype data were included in the analysis. Rs10788160 (10q26) was strongly associated with PSA levels among Caucasian participants in the high-risk group (P < 0.01), with a 33.2% increase in PSA level with each A-allele carried. Furthermore, rs10993994 (10q11) was found to be associated with PSA level (P = 0.03) in Caucasian men in the high-risk group, with a 15% increase in PSA level with each T-allele carried. A PSA adjustment model based on allele carrier status at rs10788160 and rs10993994 was proposed, specific to high-risk Caucasian men. CONCLUSIONS: Genetic variation at 10q may be particularly important in personalizing the interpretation of PSA level for Caucasian men in the high-risk group. Such information may have clinical relevance in shared decision-making and individualized prostate cancer screening strategies for Caucasian men in the high-risk group, although further study is warranted. | Male | Humans | Aged | Middle Aged | OK |
| Jing | Sun | Jing | Sun | Nicholas | Buys |  | 2013-00-00 2013 | Communication within the dental encounter is paramount to successful clinical outcomes. In this chapter we examine the concept of shared decision-making (SDM) in patients who have received treatment for a single missing tooth. Method: A cross sectional study design was used for the study. Patients were recruited from a university dental clinic and a private dental practice. Exploratory factor analysis was used to identify the dimensions of patient's SDM, their level of dental knowledge and their treatment satisfaction. Results: Exploratory factor analysis showed that 9 factors were extracted from the questionnaire. Overall, the 9 factor structure had a high level of reliability for each individual dimension. Of the 108 participants, only 26.5% were exposed to SDM. Independent sample t tests showed that there was no significant difference between the SDM group and comparison group on levels of denture knowledge, post treatment functional and economic satisfaction. However, a significant difference was seen in regards to knowledge of other treatment alternatives and post treatment aesthetic/social satisfaction, with the SDM group reporting higher levels. Conclusions: The results suggest that a 9 factor solution can be used to assess the patient's experience in the dental encounter. In regards to the use of SDM in the dental encounter, further research is required using a larger sample size from an increased number of dental practices. However, SDM is an important component of clinical dental practice and should be further explored in clinical dentistry. (PsycINFO Database Record (c) 2014 APA, all rights reserved). (chapter) | Decision Making | Psychometrics | test reliability | patients | OK |
| Michel | Rousseau | Stéphane | Turcotte | France | Légaré | Medical Decision Making | 2014-01-00 janvier 2014 | Background: We sought to determine the psychometric properties of SURE, a 4-item checklist designed to screen for clinically significant decisional conflict in clinical practice. Methods: This study was a secondary analysis of a clustered randomized trial assessing the effect of DECISION12, a 2-hour online tutorial followed by a 2-hour interactive workshop on shared decision making, on decisions to use antibiotics for acute respiratory infections. Patients completed SURE and also the Decisional Conflict Scale (DCS), as the gold standard, after consultation. We evaluated internal consistency of SURE using the Kuder- Richardson 20 coefficient (KR-20). We compared DCS and SURE scores using the Spearman correlation coefficient. We assessed sensitivity and specificity of SURE scores (cut-off score ≤ 3 out of 4) by identifying patients with and without clinically significant decisional conflict (DCS score &gt; 37.5 on a scale of 0–100). Results: Of the 712 patients recruited during the trial, 654 completed both tools. SURE scores showed adequate internal consistency (KR-20 coefficient of 0.7). There was a significant correlation between DCS and SURE scores (Spearman’s ρ = 20.45, P &lt; 0.0001). The prevalence of clinically significant decisional conflict as estimated by the DCS was 5.2% (95% CI 3.7–7.3). Sensitivity and specificity of SURE ≤ 3 were 94.1% (95% CI 78.9–99.0) and 89.8% (95% CI 87.1–92.0), respectively. Conclusions: SURE shows adequate psychometric properties in a primary care population with a low prevalence of clinically significant decisional conflict. SURE has the potential to be a useful screening tool for practitioners, responding to the growing need for detecting clinically significant decisional conflict in patients. (PsycINFO Database Record (c) 2014 APA, all rights reserved). (journal abstract) | Measurement | Decision Making | Psychometrics | test validity | Doublon |
| Michel | Rousseau | Stéphane | Turcotte | France | Légaré | Medical Decision Making: An International Journal of the Society for Medical Decision Making | 2014-01-00 Jan 2014 | BACKGROUND: We sought to determine the psychometric properties of SURE, a 4-item checklist designed to screen for clinically significant decisional conflict in clinical practice. METHODS: This study was a secondary analysis of a clustered randomized trial assessing the effect of DECISION+2, a 2-hour online tutorial followed by a 2-hour interactive workshop on shared decision making, on decisions to use antibiotics for acute respiratory infections. Patients completed SURE and also the Decisional Conflict Scale (DCS), as the gold standard, after consultation. We evaluated internal consistency of SURE using the Kuder-Richardson 20 coefficient (KR-20). We compared DCS and SURE scores using the Spearman correlation coefficient. We assessed sensitivity and specificity of SURE scores (cut-off score ≤3 out of 4) by identifying patients with and without clinically significant decisional conflict (DCS score >37.5 on a scale of 0-100). RESULTS: Of the 712 patients recruited during the trial, 654 completed both tools. SURE scores showed adequate internal consistency (KR-20 coefficient of 0.7). There was a significant correlation between DCS and SURE scores (Spearman's ρ = -0.45, P < 0.0001). The prevalence of clinically significant decisional conflict as estimated by the DCS was 5.2% (95% CI 3.7-7.3). Sensitivity and specificity of SURE ≤3 were 94.1% (95% CI 78.9-99.0) and 89.8% (95% CI 87.1-92.0), respectively. CONCLUSIONS: SURE shows adequate psychometric properties in a primary care population with a low prevalence of clinically significant decisional conflict. SURE has the potential to be a useful screening tool for practitioners, responding to the growing need for detecting clinically significant decisional conflict in patients. | Male | Decision Making | Humans | Patient Participation | OK |
| Wolfram J. | Herrmann | Tobias | Freund | Joachim | Szecsenyi | PloS One | 2013-00-00 2013 | The patients' active participation in their medical care is important for patients with chronic diseases. Measurements of patient activation are needed for studies and in clinical practice. This study aims to validate the Patient Activation Measure 13 (PAM13-D) in German-speaking primary care patients. This international cross-sectional multicentre study enrolled consecutively patients from primary care practices in three German-speaking countries: Germany, Austria, and Switzerland. Patients completed the PAM13-D questionnaire. General Self-Efficacy scale (GSE) was used to assess convergent validity. Furthermore Cronbach's alpha was performed to assess internal consistency. Exploratory factor analysis was used to evaluate the underlying factor structure of the items. We included 508 patients from 16 primary care practices in the final analysis. Results were internally consistent, with a Cronbach's alpha of 0.84. Factor analysis revealed one major underlying factor. The mean values of the PAM13-D correlated significantly (r = 0.43) with those of the GSE. The German PAM13 is a reliable and valid measure of patient activation. Thus, it may be useful in primary care clinical practice and research. | Humans | Patient Participation | Questionnaires | patients | OK |
| Judith | Hibbard | Lisa | Johns | Dustin | Wright | Disability and Rehabilitation | 2010-00-00 2010 | PURPOSE: Patient engagement in multiple sclerosis (MS) care can be challenging at times given the unpredictable disease course, wide range of symptoms, variable therapeutic response to treatment and high rates of patient depression. Patient activation, a model for conceptualising patients' involvement in their health care, has been found useful for discerning patient differences in chronic illness management. The purpose of this study was to validate the patient activation measure (PAM-13) in an MS clinic sample. METHODS: This was a survey study of 199 MS clinic patients. Participants completed the PAM-13 along with measures of MS medication adherence, self-efficacy, depression and quality of life. RESULTS: Results from Rasch and correlation analyses indicate that the PAM-13 is reliable and valid for the MS population. Activation was associated with MS self-efficacy, depression and quality of life but not with self-reported medication adherence. Also, participants with relapse-remitting MS, current employment, or high levels of education were more activated than other subgroups. CONCLUSIONS: The PAM-13 is a useful tool for understanding health behaviours in MS. The findings of this study support further clinical consideration and investigation into developing interventions to increase patient activation and improve health outcomes in MS. | Male | Humans | Patient Participation | Questionnaires | OK |
| Sigridur | Gunnarsdottir | |  |  |  | Scandinavian Journal of Caring Sciences | 2013-09-00 Sep 2013 | AIM: The aim of this study was to evaluate the psychometric properties of the Icelandic version of the Modified Patients' Perceived Involvement in Care Scale (M-PICS), I-PICS, an instrument measuring patients' perceptions of pain-related communication with health care providers (HCP). METHODS: The M-PICS was translated into Icelandic according to standard procedures for forward and backward translation. The questionnaire consisted of 20 items measuring four constructs: (i) the degree to which the HCP was perceived as controlling the information-exchange process; (ii) to what extent patients sought or shared information with their HCP; (iii) patients' perceived encouragement to raise questions and discuss their symptoms with their health care provider and (iv) patients' perceived participation in decision-making during the health care visit. The response options for each item ranged from one to five on a 5-point Likert scale, where higher scores indicated higher endorsement. RESULTS: One hundred and forty-nine participants with pain lasting longer than three months (77.2% women; mean age, 49.9 years) completed the questionnaire. To examine the construct validity of the I-PICS, a confirmatory factor analysis was performed, specifying four factors in congruence with the theoretical underpinnings of the original modified scale (M-PICS). Of the 20 items, 19 were retained, and the I-PICS factor structure was for the most part identical to the M-PICS, with the exception of three items that moved between factors and one item that did not fall decisively on one specific factor. Internal consistency (alpha) for the four factors ranged from 0.74 to 0.86 and was 0.86 for the total scale. The mean score on the total I-PICS was 2.67 on a one to five scale, ranging from 1.21 to 4.28 (possible range, 1-5). CONCLUSION: This study supports the four-factor structure of the M-PICS and that the I-PICS is a valid and reliable instrument for assessing patient-HCP communication. | Male | Humans | Patient Participation | Female | OK |
| Sigridur | Gunnarsdottir | |  |  |  | Scandinavian Journal of Caring Sciences | 2013-09-00 septembre 2013 | Aim: The aim of this study was to evaluate the psychometric properties of the Icelandic version of the Modified Patients’ Perceived Involvement in Care Scale (M-PICS), IPICS, an instrument measuring patients’ perceptions of pain-related communication with health care providers (HCP). Methods: The M-PICS was translated into Icelandic according to standard procedures for forward and backward translation. The questionnaire consisted of 20 items measuring four constructs: (i) the degree to which the HCP was perceived as controlling the information-exchange process; (ii) to what extent patients sought or shared information with their HCP; (iii) patients’ perceived encouragement to raise questions and discuss their symptoms with their health care provider and (iv) patients’ perceived participation in decision-making during the health care visit. The response options for each item ranged from one to five on a 5-point Likert scale, where higher scores indicated higher endorsement. Results: One hundred and forty-nine participants with pain lasting longer than three months (77.2% women; mean age, 49.9 years) completed the questionnaire. To examine the construct validity of the I-PICS, a confirmatory factor analysis was performed, specifying four factors in congruence with the theoretical underpinnings of the original modified scale (MPICS). Of the 20 items, 19were retained, and the I-PICS factor structure was for the most part identical to the M-PICS, with the exception of three items that moved between factors and one item that did not fall decisively on one specific factor. Internal consistency (alpha) for the four factors ranged from 0.74 to 0.86 and was 0.86 for the total scale. The mean score on the total I-PICS was 2.67 on a one to five scale, ranging from 1.21 to 4.28 (possible range, 1–5). Conclusion: This study supports the four-factor structure of the M-PICS and that the I-PICS is a valid and reliable instrument for assessing patient–HCP communication. (PsycINFO Database Record (c) 2013 APA, all rights reserved). (journal abstract) | Psychometrics | test validity | test reliability | communication | Doublon |
|  |  |  |  |  |  | Headache | 2010-03-00 Mar 2010 | BACKGROUND: Migraine is a highly prevalent disorder that imposes an important burden of disability to patients and has social and economic impact in developed countries. A good screening tool for migraine diagnosis is useful to improve disease identification and therapeutic approaches, hopefully reducing the burden of migraine. Although Portuguese is currently the sixth most spoken language in the world, no migraine screening instrument exists in Portuguese. OBJECTIVE: To validate the Portuguese version of ID-Migraine. METHODS: Consecutive adults of 2 headache outpatient clinics in Lisbon, Portugal fulfilled the Portuguese version of ID-Migraine before evaluation by a trained neurologist in clinic, blinded to the questionnaire's results. The gold standard was the neurologists' clinical diagnosis, according to the International Classification of Headache Disorders, 2nd edition. A subset of patients was randomly selected to revaluation, in order to determine test-retest reliability. The validity measures of the test were calculated. RESULTS: A total of 142 patients were included, 83.8% of which women, with an age average of 39.2 years. Clinical diagnosis of migraine was made in 63.4% of the patients. The Portuguese version of ID-Migraine presented a sensitivity of 0.94 (95% CI 0.87-0.97), specificity of 0.60 (95% CI 0.46-0.73) and a positive predictive value of 0.80 (95% CI 0.71-0.87). Calculated Cronbachs' alpha was 0.78 and kappa coefficient 0.60. CONCLUSIONS: The Portuguese version of ID-Migraine was of easy and rapid application and well accepted by patients. Its validity measures were identical to the 3 other versions of the same questionnaire - English (original), Italian, and Turkish. The Portuguese version of ID-Migraine is a valid screening tool for migraine, the first that can be used in Portuguese speaking communities although the low literacy rates in some of these countries may prevent its generalized application throughout the world. | Patient Participation | Questionnaires | physicians | Health Surveys | OK |
| Amado | Rivero-Santana | Ausiàs | Cebolla-Martí | Isabelle | Scholl | Health Expectations: An International Journal of Public Participation in Health Care and Health Policy | 2014-03-05 Mar 5, 2014 | OBJECTIVE: To translate and assess the psychometric properties of the 9-item Shared Decision-Making Questionnaire (SDM-Q-9) for measuring patients' perceptions of how clinicians' performance fits the SDM process. DESIGN: Cross-sectional study. SETTING AND PARTICIPANTS: Data were collected in primary care health centres. Patients suffering from chronic diseases and facing a medical decision were included in the study. MEASUREMENTS: The original German SDM-Q-9 was translated to Spanish using the process of cross-cultural adaptation of self-reported measures as the methodological model for Spanish translation. Reliability (internal consistency) and construct validity [exploratory (EFA) and confirmatory factor analysis (CFA)] were assessed. RESULTS: The final Spanish version of the SDM-Q-9 was tested in a primary care sample of 540 patients. The SDM-Q-9 presented adequate reliability and acceptable validity. Internal consistency yielded a Cronbach's alpha of 0.885 for the whole scale. EFA showed a two-factorial solution, and for the CFA, the best solution was obtained with a one-dimensional factor with the item 1 excluded, which produced the best indexes of fit. DISCUSSION AND CONCLUSIONS: The Spanish version of the SDM-Q-9 showed adequate reliability and acceptable validity parameters among primary care patients. The SDM-Q-9 is suitable for use in Spain and other Spanish-speaking countries with similarly organized health-care systems. The use of the SDM-Q-9 may contribute to the evaluation of SDM process from the patient's perspective. | | | | | OK |
| Linda | Cardozo | Dudley | Robinson |  |  | International Urogynecology Journal | 2011-07-00 Jul 2011 | INTRODUCTION AND HYPOTHESIS: The purpose of this study is to assess the validity of patient goal achievement in overactive bladder (OAB). METHODS: Data were taken from a placebo-controlled randomised trial of transdermal oxybutynin and open label extension study. Face validity was assessed using qualitative analysis. Convergent validity was assessed by comparison with objective symptom improvement. Responsiveness was assessed at 4 s, using the standardised effect size. Reliability was assessed between 4 and 12 weeks of treatment. RESULTS: Ninety-six women were randomised. There were moderate correlations (0.50-0.51) between goal achievement and symptom improvement for urinary urgency and urge incontinence. Mean goal achievement demonstrated good reliability (intraclass correlation = 0.82) but low responsiveness (r = 0.14) between transdermal oxybutynin and placebo-treated groups. CONCLUSIONS: Although patient goals have good face validity and can be reliably measured, they have limited convergence with conventional measures of OAB severity and improvement and low responsiveness. | Humans | Patient Participation | Questionnaires | Female | OK |
| Sally W. | Vernon | Patricia D. | Mullen | Robert O. | Morgan | Patient Education and Counseling | 2011-12-00 Dec 2011 | OBJECTIVE: To evaluate the psychometric properties of the 4-factor low literacy Decisional Conflict Scale (DCS-LL) with men eligible for prostate cancer screening (PCS). METHODS: We used baseline (T0; n=149) and post-intervention (T2; n=89) data from a randomized, controlled trial of a PCS decision aid to assess internal consistency reliability and construct, discriminant, and factor validity. RESULTS: There was evidence of excellent internal consistency reliability (α's≥.80) and fair construct validity (most r's≥.40) for the DCS-LL except for the Supported subscale. The DCS-LL was able to discriminate between men who had decided and those who had not. There was evidence for the original 4-factor model at T0 but exploratory analysis suggested a 3-factor solution at T0 and T2 with Informed and Value Clarity as one factor. CONCLUSION: For men eligible for PCS, feeling informed and feeling clear about values may not reflect distinct cognitive processes. Feeling supported may not be a factor contributing to uncertainty. PRACTICE IMPLICATIONS: Research should address whether current DCS subscales best represent the factors that contribute to uncertainty for PCS and for other screening decisions. Research should also explore the influence of health literacy on the factor structure of the DCS-LL. | Male | Decision Making | Decision Support Techniques | Humans | OK |
| Jolanda | Hennink | Caroline G. | Schafrat | Johanna M. A. | Visser-Meily | Disability and Rehabilitation | 2012-00-00 2012 | PURPOSE: There is still a need for a generic participation instrument that measures both objective and subjective participation in adults living in the community and that is feasible for use in rehabilitation practice. The Utrecht Scale for Evaluation of Rehabilitation-Participation (USER-Participation) was developed to satisfy this need, comprising 31 items in three scales: Frequency, Restrictions and Satisfaction. The aim of this study was to examine the validity of this measure. METHODS: Cross-sectional study involving former rehabilitation outpatients from five rehabilitation facilities in the Netherlands (n = 395). The Frenchay Activities Index (FAI), the participation subtotal score of the ICF Measure of Participation and Activities Screener (IMPACT-SP) and the Participation Scale were included as reference measures. RESULTS: Internal consistency of the USER-Participation scales was satisfactory (α 0.70-0.91). Spearman correlations between these scales were between 0.36 and 0.52. Concurrent validity was shown by strong correlations between the Frequency scale and the FAI (0.59), the Restrictions scale and the IMPACT-SP (0.75) and the Satisfaction scale and the Participation Scale (-0.73). Discriminant validity was shown by significant differences in USER-Participation scores between participants with different levels of independence and between participants with different health conditions. CONCLUSION: The USER-Participation appears to be a valid measure to rate objective and subjective participation in persons with physical disabilities. | Male | Humans | Patient Participation | Questionnaires | OK |
|  |  |  |  |  |  | Medical Care | 2012-09-00 Sep 2012 | INTRODUCTION: Validly interpreting scores based on patients' reports of their experiences using scales depends on a valid scoring system. The possibility exists that, although the creators of a scale intended to create a scale measuring a single construct, the questions may seem to measure more than one construct. However, the possibility also exists that the questions do generally measure one construct, but several "nuisance" constructs influence the measurement of the construct indirectly. Unidimensional, multidimensional, and bifactor analytical models offer a method for establishing scale dimensionality and subsequently developing valid scoring systems. In this paper, we take an applied perspective, discuss these models and their implications, and use real data to provide an example. METHODS: We used unidimensional, multidimensional, and bifactor analyses to examine the measurement structure of the Consumer Assessments of Healthcare Providers and Systems Cultural Competence (CAHPS-CC) Survey. Participants came from a 2008 sample of 2 Medicaid managed care plans, in New York and California. RESULTS: Both unidimensional and bifactor models failed to fit the data well. A multidimensional model, with 7 factors corresponding to 7 cultural competence domains fit the data well (root mean square error of approximation=0.064; Tucker-Lewis Index=0.98; comparative fit index=0.97). DISCUSSION: Results indicate that the CAHPS-CC does not seem to measure a single construct. Rather, the CAHPS-CC seems to measure 7 separable domains, suggesting the need for 7 separate scale scores. Our findings highlight the importance of conducting dimensionality analyses. | Humans | Patient Participation | Patient Satisfaction | Physician-Patient Relations | OK |
|  |  |  |  |  |  | Texas Dental Journal | 2012-10-00 Oct 2012 | | Humans | Patient Participation | Policy Making | Risk Management | OK |
| Siamak | Noorbaloochi | Joseph P. | Grill | Somnath | Saha | Journal of General Internal Medicine | 2010-07-00 Jul 2010 | OBJECTIVES: This paper compares estimates of poor health literacy using two widely used assessment tools and assesses the effect of non-response on these estimates. STUDY DESIGN AND SETTING: A total of 4,868 veterans receiving care at four VA medical facilities between 2004 and 2005 were stratified by age and facility and randomly selected for recruitment. Interviewers collected demographic information and conducted assessments of health literacy (both REALM and S-TOFHLA) from 1,796 participants. Prevalence estimates for each assessment were computed. Non-respondents received a brief proxy questionnaire with demographic and self-report literacy questions to assess non-response bias. Available administrative data for non-participants were also used to assess non-response bias. RESULTS: Among the 1,796 patients assessed using the S-TOFHLA, 8% had inadequate and 7% had marginal skills. For the REALM, 4% were categorized with 6th grade skills and 17% with 7-8th grade skills. Adjusting for non-response bias increased the S-TOFHLA prevalence estimates for inadequate and marginal skills to 9.3% and 11.8%, respectively, and the REALM estimates for < or = 6th and 7-8th grade skills to 5.4% and 33.8%, respectively. CONCLUSIONS: Estimates of poor health literacy varied by the assessment used, especially after adjusting for non-response bias. Researchers and clinicians should consider the possible limitations of each assessment when considering the most suitable tool for their purposes. | Male | Humans | Patient Participation | United States | OK |
| Louis A. | Penner | Marci J. | Gleason | Tanina | Foster | Patient Education and Counseling | 2011-01-00 Jan 2011 | OBJECTIVE: to investigate whether patient demographic characteristics and patients' companions influence variation in patient question asking during cancer clinical interactions, thus representing a potential disparity in access to information. METHODS: data included 109 oncologist-patient-companion interactions video recorded at a comprehensive cancer center. Interactions were observed and analyzed using the Karmanos Information Seeking Analysis System (K-ISAS). RESULTS: significant relationships were found between patient race/ethnicity and question asking. Black patients asked fewer questions and a smaller proportion of direct questions (relative to the total frequency of questions) than White patients. Black patients were also less likely to have companions present during the interaction, which resulted in fewer questions asked on Black patients' behalf. CONCLUSION: differences in question asking by Black and White patients suggest that Black patients may receive less information from their oncologists than White patients. PRACTICE/RESEARCH IMPLICATIONS: patients should be encouraged to ask more questions and more direct questions and to bring a companion to the interaction to assist them in gaining information from their physician. Future research is needed to investigate ways to eliminate this potential source of disparities in access to information. | Male | Humans | Patient Participation | Physician-Patient Relations | OK |
| Musu | Sesay | Caroline G. | Tai | Daniel K. | Lee | Cancer | 2014-11-12 Nov 12, 2014 | BACKGROUND: Health care providers often counsel prostate cancer patients about treatment options with medical terminology. However, studies have demonstrated a severe lack of comprehension of these terms, particularly in underserved populations. It was hypothesized that a video-based educational tool would significantly improve the understanding of key terms related to prostate health in a predominantly lower literacy population. METHODS: A software application was developed by various experts, including urologists and human-computer interaction specialists, to serve as a video-based educational tool emphasizing narrated animations to promote understanding of terms related to urinary, bowel, and sexual function. This application was viewed by patients recruited from 2 low-income safety net clinics, where a previously developed survey was administered to assess pre- and postintervention levels of comprehension. RESULTS: Fifty-six patients with a mean literacy level of 7th to 8th grade completed the study. Patients achieved statistically significant improvements in comprehension for the majority of the terms after the video intervention, with notable improvements including the terms incontinence (from 14% to 50%), bowels (from 14% to 46%), and impotence (from 58% to 84%). Patients demonstrated significant gains in their understanding of the function of the prostate (from 11% to 30%) and in their ability to locate the prostate on anatomic drawings (from 50% to 82%). CONCLUSIONS: This video-based educational tool is an effective method for overcoming the severe lack of comprehension of prostate health terminology among patients. The improvements achieved have the potential to enhance patient participation in shared and informed decision making and to support combined visual-audio multimedia as a promising tool for prostate cancer education. Cancer 2014. © 2014 American Cancer Society. | | | | | OK |
|  |  |  |  |  |  | Pharmacoepidemiology and Drug Safety | 2013-10-00 Oct 2013 | PURPOSE: To survey British community pharmacists' views and practices concerning direct patient reporting of ADRs. METHODS: Cross-sectional postal survey of community pharmacists in Britain RESULTS: Of 1096 questionnaires distributed, 297 usable responses were obtained, (27.1%). Respondents' estimates of the frequency of patients reporting a suspected ADR to them had a median of 1.0 per month. Almost a fifth of respondents (19.6%) do not specifically ask patients about ADRs, and 38.7% do not encourage patients to report. Only 18.5% displayed a poster promoting the YC Scheme in their pharmacy, but 57.9% claimed to have patient YCs available. A quarter (24.9%) of respondents considered that ADR reporting should be restricted to health professionals and 14.4% considered that patients were not at all capable of identifying ADRs. CONCLUSIONS: The low response rate and overall results suggest that British community pharmacists may lack interest in and do not promote direct patient reporting. Increased awareness of the benefits and mechanisms of patient reporting may be required to ensure that pharmacists can provide the necessary support to facilitate patient reporting. | Humans | Patient Participation | Questionnaires | Great Britain | OK |
| Lukas | Zimmerli | Susan | Mérillat-Koeneke | Robert | Riener | Journal of Rehabilitation Medicine | 2011-05-00 May 2011 | OBJECTIVE: To examine the effect of various forms of training interventions, with and without virtual reality, on the initiation and maintenance of active participation during robot-assisted gait training. DESIGN: Intervention study at the Rehabilitation Centre Affoltern a. A., University Children's Hospital, Zurich. SUBJECTS: Ten patients (5 males, mean age 12.47 years, standard deviation 1.84 years) with different neurological gait disorders and 14 healthy children (7 males, mean age 11.76 years, standard deviation 2.75 years). METHODS: All participants walked in the driven gait orthosis Lokomat® in 4 different randomly-assigned conditions. Biofeedback values calculated during swing phases were the primary outcome measure and secondary outcomes were derived from a questionnaire assessing the participant's motivation. RESULTS: Findings revealed a significant main effect for training condition in all participants (p < 0.001), for patients (p < 0.05) and for healthy controls (p < 0.01). Overall, both virtual reality-assisted therapy approaches were equally the most effective in initiating the desired active participation in all children, compared with conventional training conditions. Motivation was very high and differed between the groups only in the virtual navigation condition. CONCLUSION: Novel virtual reality-based training conditions represent a valuable approach to enhance active participation during robot-assisted gait training in patients and healthy controls. | Male | Humans | Patient Participation | Questionnaires | OK |
| Christiane | Wachter | Doris | Henne-Bruns | Franz | Porzsolt | BMC medical informatics and decision making | 2012-00-00 2012 | BACKGROUND: A variety of multimodal treatment options are available for colorectal cancer and many patients want to be involved in decisions about their therapies. However, their desire for autonomy is limited by lack of disease-specific knowledge. Visual aids may be helpful tools to present complex data in an easy-to-understand, graphic form to lay persons. The aim of the present study was to evaluate the treatment preferences of healthy persons and patients using visual aids depicting multimodal treatment options for colorectal cancer. METHODS: We designed visual aids for treatment scenarios based on four key studies concerning multimodal treatment of colorectal cancer. The visual aids were composed of diagrams depicting outcome parameters and side effects of two treatment options. They were presented to healthy persons (n = 265) and to patients with colorectal cancer (n = 102). RESULTS: Most patients and healthy persons could make immediate decisions after seeing the diagrams (range: 88%-100%). Patients (79%) chose the intensive-treatment option in the scenario with a clear survival benefit. In scenarios without survival benefit, all groups clearly preferred the milder treatment option (range: 78% - 90%). No preference was seen in the scenario depicting equally intense treatment options with different timing (neoadjuvant vs. adjuvant) but without survival benefit. CONCLUSIONS: Healthy persons' and patients' decisions using visual aids seem to be influenced by quality-of-life aspects rather than recurrence rates especially in situations without survival benefit. In the future visual aids may help to improve the management of patients with colorectal cancer. | Male | Decision Making | Humans | Patient Participation | OK |
| Amal | Durakovic | Jane | Thøgersen | Anne-Kirsten | Midjord | Journal of Cardiopulmonary Rehabilitation and Prevention | 2011-08-00 2011 Jul-Aug | PURPOSE: Vitamin D deficiency is common in patients with chronic obstructive pulmonary disease; however, no study has evaluated the influence of vitamin D status on effects of pulmonary rehabilitation (PR). METHODS: We studied 311 patients, who participated in a 7-week outpatient PR. Vitamin D status was assessed at entry and examined for association with patient characteristics, dropout from PR, and effect on endurance shuttle walk time. RESULTS: Vitamin D level 25 nM was seen in 61 (19.6%) of the patients. They were significantly younger, were more frequently on long-term oxygen therapy, had higher body mass index and fat-free mass index, had worse quality of life score, tended to have lower percent predicted value for forced expiratory volume in the first second of expiration, and more frequently were current smokers. They had a 3-time higher risk of dropout from the PR program (P = .003) compared with patients with normal vitamin D status and a poorer improvement in endurance shuttle walk time (P = .03). DISCUSSION: In conclusion, vitamin D deficiency was significantly associated with higher dropout rates from PR, and there was a tendency toward a poorer improvement in endurance shuttle walk time. | Male | Humans | Patient Participation | Questionnaires | OK |
| Clare | Flach | Emese | Csipke | Helen | Glossop | Journal of Mental Health (Abingdon, England) | 2012-02-00 Feb 2012 | BACKGROUND: Service users express dissatisfaction with inpatient care and their concerns revolve around staff interactions, involvement in treatment decisions, the availability of activities and safety. Traditionally, satisfaction with acute care has been assessed using measures designed by clinicians or academics. AIMS: To develop a patient-reported outcome measure of perceptions of acute care. An innovative participatory methodology was used to involve services users throughout the research process. METHOD: A total of 397 participants were recruited for the study. Focus groups of service users were convened to discuss their experiences and views of acute care. Service user researchers constructed a measure from the qualitative data, which was validated by expert panels of service users and tested for its psychometric properties. RESULTS: Views on Inpatient Care (VOICE) is easy to understand and complete and therefore is suitable for use by service users while in hospital. The 19-item measure has good validity and internal and test-retest reliability. Service users who have been compulsorily admitted have significantly worse perceptions of the inpatient environment. CONCLUSIONS: A participatory methodology has been used to generate a self-report questionnaire measuring service users' perceptions of acute care. VOICE encompasses the issues that service users consider most important and has strong psychometric properties. | Male | Humans | Patient Participation | Patient Satisfaction | OK |
| Mika | Gissler | Stefan Hrafn | Jonsson | Max | Petzold | Journal of Medical Internet Research | 2013-00-00 2013 | BACKGROUND: Survey response rates have been declining over the past decade. The more widespread use of the Internet and Web-based technologies among potential health survey participants suggests that Web-based questionnaires may be an alternative to paper questionnaires in future epidemiological studies. OBJECTIVE: To compare response rates in a population of parents by using 4 different modes of data collection for a questionnaire survey of which 1 involved a nonmonetary incentive. METHODS: A random sample of 3148 parents of Danish children aged 2-17 years were invited to participate in the Danish part of the NordChild 2011 survey on their children's health and welfare. NordChild was conducted in 1984 and 1996 in collaboration with Finland, Iceland, Norway, and Sweden using mailed paper questionnaires only. In 2011, all countries used conventional paper versions only except Denmark where the parents were randomized into 4 groups: (1) 789 received a paper questionnaire only (paper), (2) 786 received the paper questionnaire and a log-in code to the Web-based questionnaire (paper/Web), (3) 787 received a log-in code to the Web-based questionnaire (Web), and (4) 786 received log-in details to the Web-based questionnaire and were given an incentive consisting of a chance to win a tablet computer (Web/tablet). In connection with the first reminder, the nonresponders in the paper, paper/Web, and Web groups were also present with the opportunity to win a tablet computer as a means of motivation. Descriptive analysis was performed using chi-square tests. Odds ratios were used to estimate differences in response rates between the 4 modes. RESULTS: In 2011, 1704 of 3148 (54.13%) respondents answered the Danish questionnaire. The highest response rate was with the paper mode (n=443, 56.2%). The other groups had similar response rates: paper/Web (n=422, 53.7%), Web (n=420, 53.4%), and Web/tablet (n=419, 53.3%) modes. Compared to the paper mode, the odds for response rate in the paper/Web decreased by 9% (OR 0.91, 95% CI 0.74-1.10) and by 11% (OR 0.89, 95% CI 0.73-1.09) in the Web and Web/tablet modes. The total number of responders for NordChild declined from 10,291 of 15,339 (67.09%) in 1984 and 10,667 of 15,254 (69.93%) in 1996 to 7805 of 15,945 (48.95%) in 2011 with similar declines in all 5 Nordic countries. CONCLUSIONS: Web-based questionnaires could replace traditional paper questionnaires with minor effects on response rates and lower costs. The increasing effect on the response rate on participants replying for a nonmonetary incentive could only be estimated within the 2 Web-based questionnaire modes before the first reminder. Alternative platforms to reach higher participation rates in population surveys should reflect the development of electronic devices and the ways in which the population primarily accesses the Internet. | Male | Humans | Questionnaires | Female | Doublon |
| Mika | Gissler | Stefan Hrafn | Jonsson | Max | Petzold | Journal of Medical Internet Research | 2013-08-00 août 2013 | Background: Survey response rates have been declining over the past decade. The more widespread use of the Internet and Web-based technologies among potential health survey participants suggests that Web-based questionnaires may be an alternative to paper questionnaires in future epidemiological studies. Objective: To compare response rates in a population of parents by using 4 different modes of data collection for a questionnaire survey of which 1 involved a nonmonetary incentive. Methods: A random sample of 3148 parents of Danish children aged 2-17 years were invited to participate in the Danish part of the NordChild 2011 survey on their children’s health and welfare. NordChild was conducted in 1984 and 1996 in collaboration with Finland, Iceland, Norway, and Sweden using mailed paper questionnaires only. In 2011, all countries used conventional paper versions only except Denmark where the parents were randomized into 4 groups: (1) 789 received a paper questionnaire only (paper), (2) 786 received the paper questionnaire and a log-in code to the Web-based questionnaire (paper/Web), (3) 787 received a log-in code to the Web-based questionnaire (Web), and (4) 786 received log-in details to the Web-based questionnaire and were given an incentive consisting of a chance to win a tablet computer (Web/tablet). In connection with the first reminder, the nonresponders in the paper, paper/Web, and Web groups were also present with the opportunity to win a tablet computer as a means of motivation. Descriptive analysis was performed using chi-square tests. Odds ratios were used to estimate differences in response rates between the 4 modes. Results: In 2011, 1704 of 3148 (54.13%) respondents answered the Danish questionnaire. The highest response rate was with the paper mode (n = 443, 56.2%). The other groups had similar response rates: paper/Web (n = 422, 53.7%), Web (n = 420, 53.4%), and Web/tablet (n = 419, 53.3%) modes. Compared to the paper mode, the odds for response rate in the paper/Web decreased by 9% (OR 0.91, 95% CI 0.74-1.10) and by 11% (OR 0.89, 95% CI 0.73-1.09) in the Web and Web/tablet modes. The total number of responders for NordChild declined from 10,291 of 15,339 (67.09%) in 1984 and 10,667 of 15,254 (69.93%) in 1996 to 7805 of 15,945 (48.95%) in 2011 with similar declines in all 5 Nordic countries. Conclusions: Web-based questionnaires could replace traditional paper questionnaires with minor effects on response rates and lower costs. The increasing effect on the response rate on participants replying for a nonmonetary incentive could only be estimated within the 2 Web-based questionnaire modes before the first reminder. Alternative platforms to reach higher participation rates in population surveys should reflect the development of electronic devices and the ways in which the population primarily accesses the Internet. (PsycINFO Database Record (c) 2014 APA, all rights reserved). (journal abstract) | Questionnaires | Client Participation | Internet | Surveys | Doublon |
| Mika | Gissler | Stefan Hrafn | Jonsson | Max | Petzold | Journal of Medical Internet Research | 2013-00-00 2013 | BACKGROUND: Survey response rates have been declining over the past decade. The more widespread use of the Internet and Web-based technologies among potential health survey participants suggests that Web-based questionnaires may be an alternative to paper questionnaires in future epidemiological studies. OBJECTIVE: To compare response rates in a population of parents by using 4 different modes of data collection for a questionnaire survey of which 1 involved a nonmonetary incentive. METHODS: A random sample of 3148 parents of Danish children aged 2-17 years were invited to participate in the Danish part of the NordChild 2011 survey on their children's health and welfare. NordChild was conducted in 1984 and 1996 in collaboration with Finland, Iceland, Norway, and Sweden using mailed paper questionnaires only. In 2011, all countries used conventional paper versions only except Denmark where the parents were randomized into 4 groups: (1) 789 received a paper questionnaire only (paper), (2) 786 received the paper questionnaire and a log-in code to the Web-based questionnaire (paper/Web), (3) 787 received a log-in code to the Web-based questionnaire (Web), and (4) 786 received log-in details to the Web-based questionnaire and were given an incentive consisting of a chance to win a tablet computer (Web/tablet). In connection with the first reminder, the nonresponders in the paper, paper/Web, and Web groups were also present with the opportunity to win a tablet computer as a means of motivation. Descriptive analysis was performed using chi-square tests. Odds ratios were used to estimate differences in response rates between the 4 modes. RESULTS: In 2011, 1704 of 3148 (54.13%) respondents answered the Danish questionnaire. The highest response rate was with the paper mode (n=443, 56.2%). The other groups had similar response rates: paper/Web (n=422, 53.7%), Web (n=420, 53.4%), and Web/tablet (n=419, 53.3%) modes. Compared to the paper mode, the odds for response rate in the paper/Web decreased by 9% (OR 0.91, 95% CI 0.74-1.10) and by 11% (OR 0.89, 95% CI 0.73-1.09) in the Web and Web/tablet modes. The total number of responders for NordChild declined from 10,291 of 15,339 (67.09%) in 1984 and 10,667 of 15,254 (69.93%) in 1996 to 7805 of 15,945 (48.95%) in 2011 with similar declines in all 5 Nordic countries. CONCLUSIONS: Web-based questionnaires could replace traditional paper questionnaires with minor effects on response rates and lower costs. The increasing effect on the response rate on participants replying for a nonmonetary incentive could only be estimated within the 2 Web-based questionnaire modes before the first reminder. Alternative platforms to reach higher participation rates in population surveys should reflect the development of electronic devices and the ways in which the population primarily accesses the Internet. | Male | Humans | Questionnaires | Female | OK |
| Anne-Maëlle | Fontenoy | Benoît | le Goff | Yves | Maugars | Joint, Bone, Spine: Revue Du Rhumatisme | 2010-12-00 Dec 2010 | OBJECTIVES: To investigate whether weekly determination of Routine Assessment of Patient Index Data (RAPID) scores 3 and 4 in patients with rheumatoid arthritis (RA) improved the assessment of disease activity and detected additional activity peaks (predictive of additional structural damage). METHODS: Each week for 6 months, 26 patients with RA completed the patient-reported outcome questionnaires RAPID-3 and RAPID-4. During the study period, the treatment regimen for RA remained unchanged in 23 of the 26 patients. RESULTS: RAPID-3 was as informative as RAPID-4. Mean values were 3.85±1.66 (range: 0.72-6.85) and 3.43±1.57 (range: 0.81-6.77), respectively. The areas under the RAPID-3 score curves plotted using only the first and last weeks or all the weeks showed a statistically significant difference in 19 (73%) of the 26 patients. The difference between the highest and lowest RAPID-3 scores was greater than the clinically significant threshold of 1.2 in all 26 patients (mean difference: 2.95±0.71; range: 1.6-5.5). In 13 patients, the RAPID-3 score detected one (one patient) or several (12 patients) activity peaks. Among RAPID-3 score components, the visual analog scale (VAS) pain score had the greatest influence (37% of the total score), followed by the VAS disease-activity score (36%) then by the multidimensional Health Assessment Questionnaire score (27%). Scores were not influenced by patient mood at questionnaire completion. CONCLUSION: Self-evaluation at home using the RAPID-3 score provides additional information that should improve the accuracy of RA monitoring between physician visits and that may help to optimize visit scheduling. | Time Factors | Humans | Patient Participation | Questionnaires | OK |
| Shubha | Krishnamurthy | Anita | Shet | Ayesha | De Costa | PloS One | 2013-00-00 2013 | BACKGROUND: India has the highest number of HIV infected persons in the world after South Africa. Much HIV related behavioral, clinical and laboratory based research is ongoing in India. Yet little is known on Indian HIV patients' knowledge of research, their processes of decision making and motives for participation. We aimed to explore these areas among HIV infected individuals to understand their reasons for participating in research. METHODOLOGY/PRINCIPAL FINDINGS: This is a cross sectional survey among 173 HIV infected adults at a tertiary level hospital in Bangalore, India, done between October 2010 and January 2011. A pre-tested questionnaire was administered to the participants by trained research assistants to assess their knowledge regarding research, willingness to participate, decision making and determinants of participation. Participants were presented with five hypothetical HIV research studies. Each study had a different level of intervention and time commitment. Of respondents, 103(60%), said that research meant 'to discover something new' and 138(80%) were willing to participate in research. A third of the respondents were unaware of their right to refuse participation. Willingness to participate in research varied with level of intervention. It was the lowest for the hypothetical study involving sensitive questions followed by the hypothetical drug trial; and was the highest for the hypothetical cross sectional questionnaire based study (p<0.0015). Individual health benefits and altruism were the primary motives for participation in research and indicate the presence of therapeutic misconception. Women were less likely to make autonomous decisions for participation in interventional studies. CONCLUSIONS/SIGNIFICANCE: Despite a majority willing to participate, over a third of respondents did not have any knowledge of research or the voluntary nature of participation. This has ethical implications. Researchers need to focus on enabling potential research participants understand the concepts of research, promote autonomous decisions, especially by women and restrict therapeutic misconception. | Male | Decision Making | Humans | Patient Participation | OK |
| Marije S. | Koelewijn-van Loon | Sarah | Collins | John | Kirwan | The Journal of Rheumatology | 2014-01-00 Jan 2014 | OBJECTIVE: Since 2002, 58 patients have participated as collaborating partners in 6 Outcome Measures in Rheumatology (OMERACT) conferences. Little is known about how they engage with researchers and how they have influenced conference outcomes. METHODS: A responsive evaluation was carried out, including a thematic document analysis of conference proceedings and gray literature, participant observation, and 38 interviews with patients and professionals representing research, industry, and regulators. Interview transcripts were subjected to an inductive content analysis. RESULTS: The role of patients has evolved from a single focus group in 2002 to full integration in all parts of the conference in 2012. Longterm engagement has made a significant change in the scope and conduct of rheumatology research. It has enriched the research agenda by identifying previously neglected outcome domains such as fatigue, sleep disturbances, and flares, and it has contributed to more patient-relevant outcomes in clinical trials. Facilitating factors have been a strong commitment of the leadership, adequate selection procedure, inclusive conference design, interactive and encouraging moderation style, and self-organized support. The intensity of the program and doubts regarding the representativeness of the patient group were still seen as challenges for the future. CONCLUSION: Making patient participation an integral part of the vision and procedures of OMERACT has significantly contributed to the success of OMERACT. It has changed the perceptions and beliefs of many participants. Full use of patients' experiential knowledge before and during the conference is still challenging. | Humans | Patient Participation | Outcome Assessment (Health Care) | Rheumatology | OK |
| Deena | Elsori | Makhtar | Sarr |  |  | The Practising Midwife | 2014-06-00 Jun 2014 | Evidence suggests that, throughout the world, there is a lack of understanding of the midwife's role. It has previously been found that most pregnant women in Dubai did not understand the role of the midwife and felt safer being cared for by doctors This paper presents findings from a survey conducted in Abu Dhabi, where midwifery is well established, which sought to explore whether the role of the midwife is generally acknowledged and understood. Overall a lack of knowledge of the role and scope of practice of the midwife was evident amongst a third of respondents. Since midwifery care has been shown to provide high quality maternity care, marketing and promoting the role of the midwife both to service users and to other professionals is crucial. | Humans | Patient Participation | Questionnaires | Female | OK |
| Charles | Wolfe |  |  |  |  | Health Expectations: An International Journal of Public Participation in Health Care and Health Policy | 2010-03-00 Mar 2010 | BACKGROUND: Health researchers are encouraged to involve service users as partners in their research. There is a need to increase the evidence base of involvement, including an accumulation of empirical accounts of involvement practices, demonstrating how involvement influences research and refinement of the concept itself. AIMS: To report the development of a pilot study by academic researchers and stroke service users belonging to a user research group to investigate costs of stroke to individuals and families; to reflect on what this example of user involvement achieved and implications for what involvement means. METHODS: We conducted a 2-year ethnographic study that included participant observation, formal and informal interviews with professionals and user group members and documentary analysis. Data were systematically recorded to permit description of processes and reflexive analysis. RESULTS AND CONCLUSIONS: We report on five stages of the research process from service user identification of a research question to interpretation of pilot study findings. Professional researchers led the research process and developed a novel method to involve stroke service users in the development of a questionnaire. Some academic colleagues questioned the value of the proposed investigation as it did not appear to conform to implicit criteria of quality research. We argue that the moral status that user involvement has acquired means that academics' concerns about quality did not prevent the pilot study from being conducted. We suggest that much of what was undertaken might be considered standard good practice in developing new research studies but also identify additional benefits of user involvement. Implications for conceptual development and evaluation are discussed. | Male | Humans | Patient Participation | Questionnaires | OK |
|  |  |  |  |  |  | Journal of Travel Medicine | 2013-08-00 2013 Jul-Aug | | Male | Animals | Humans | Patient Participation | OK |
| Linda | Worrall |  |  |  |  | Ear and Hearing | 2012-02-00 2012 Jan-Feb | OBJECTIVES: Client involvement in health decision making, or shared decision making, is increasingly being advocated. For example, rehabilitation interventions such as hearing aids and communication programs can be presented as options to adults with hearing impairment seeking help for the first time. Our previous research focused on the predictors of intervention decisions when options were presented with a decision aid. However, not all participants took up the intervention they initially decided upon. Although it is interesting to understand what informs adults with hearing impairment's intervention decisions, it is their intervention uptake and outcomes which best represent the ultimate end result of the rehabilitation process. This prospective study investigated the predictors of uptake and of successful outcomes of hearing aids and communication programs in middle-aged and older adults with hearing impairment seeking help for the first time. DESIGN: Using shared decision making, 153 participants with hearing impairment (average of air conduction thresholds at 0.5, 1, 2, and 4 kHz greater than 25 dB HL in at least one ear) aged 50 yr and older were presented with intervention options: hearing aids, communication programs (group or individual), and no intervention. Each participant received a decision aid and had at least 1 wk to consider intervention options before the intervention decision was made. Outcome measures for both hearing aids and communication programs at 3 mo after intervention completion were benefit (measured with the Client-Oriented Scale of Improvement), composite outcomes (measured with the International Outcome Inventory), and reduction in self-reported hearing disability (measured with the Hearing Handicap Questionnaire). Multivariate analysis (logistic and linear regression) identified predictors of intervention uptake and of successful outcomes when all other variables were held constant. RESULTS: Almost a quarter of the 153 participants (24%) did not take up the intervention they initially decided upon: 6 mo after making their intervention decision, 66 participants (43%) obtained hearing aids, 28 participants (18%) completed communication programs, and 59 participants (39%) did not complete an intervention. Seven intervention uptake predictors were identified: (1) application for subsidized hearing services (participants more likely to obtain hearing aids and less likely to complete no intervention); (2) higher socioeconomic status (no intervention less likely); (3) greater communication self-efficacy (hearing aids less likely); (4) greater contemplation stage of change (no intervention less likely); (5) greater hearing disability perceived by others and self (communication programs less likely); (6) greater perceived communication program effectiveness (communication programs more likely); and (7) greater perceived suitability of individual communication program (hearing aids less likely and communication programs more likely). Six predictors of successful intervention outcomes were identified: (1) higher socioeconomic status; (2) greater initial self-reported hearing disability; (3) lower precontemplation stage of change; (4) greater action stage of change; (5) lower chance locus of control; and (6) greater hearing disability perceived by others and self. CONCLUSIONS: Self-reported hearing disability and stages of change are the two most robust predictors of intervention uptake and successful outcomes. Clinicians should offer intervention options and should discuss these predictors when helping adults with hearing impairment make optimal decisions. | Male | Humans | Patient Participation | Female | OK |
| Maureen | Lumb | Penny | Morris | Sue | Sherwin | Health Expectations: An International Journal of Public Participation in Health Care and Health Policy | 2014-06-00 Jun 2014 | BACKGROUND: Involving service users and carers in decisions about their health care is a key feature of health-care practice. Professional health and social care students need to develop skills and attributes to best enable this to happen. AIMS: The aims were to explore service user and carer perceptions of behaviours, attributes and context required to enable shared decision making; to compare these perceptions to those of students and academic staff with a view to utilizing the findings to inform the development of student assessment tools. METHODS: A mixed methods approach was used including action learning groups (ALG) and an iterative process alongside a modified Delphi survey. PARTICIPANTS: The ALGs were from an existing service user and carer network. The survey was sent to sixty students, sixty academics and 30 service users from 16 different professional disciplines, spanning four Universities in England. RESULTS: The collaborative enquiry process and survey identified general agreement that being open and honest, listening, showing respect, giving time and being up to date were important. The qualitative findings identified that individual interpretation was a key factor. An unexpected result was an insight into possible insecurities of students. CONCLUSIONS: The findings indicate that distilling rich qualitative information into a format for student assessment tools could be problematic as the individual context could be lost, it is therefore proposed that the information could be better used as a learning rather than assessment tool. Several of those involved identified how they valued the process and found it beneficial. | Humans | Patient Participation | Questionnaires | patient preference | OK |
| Anik | Giguere | Marie-Pierre | Gagnon | France | Légaré | The Journal of Continuing Education in the Health Professions | 2012-00-00 2012 | INTRODUCTION: Little is known about the factors that influence family physician (FP) participation in continuing professional development (CPD) programs in shared decision making (SDM). We sought to identify the factors that motivate FPs to participate in DECISION+, a CPD program in SDM. METHODS: In 2007-2008, we collected data from 39 FPs who participated in a pilot randomized trial of DECISION+. In 2010, we collected data again from 11 of those participants and from 12 new subjects. Based on the theory of planned behavior, our questionnaire assessed FPs' intentions to participate in a CPD program in SDM and evaluated FPs' attitudes, subjective norms and perceived behavioral control. We also conducted 4 focus groups to explore FPs' salient beliefs. RESULTS: In 2010, FPs' mean intention to participate in a CPD program in SDM was relatively strong (2.6 ± 0.5 on a scale from -3 = "strongly disagree" to +3 = "strongly agree"). Affective attitude was the only factor significantly associated with intention (r = .51, p = .04). FPs identified the attractions of participating in a CPD program in SDM as (1) its interest, (2) the pleasure of learning, and (3) professional stimulation. Facilitators of their participation were (1) a relevant clinical topic, (2) an interactive program, (3) an accessible program, and (4) decision support tools. DISCUSSION: To attract FPs to a CPD program in SDM, CPD developers should make the program interesting, enjoyable, and professionally stimulating. They should choose a clinically relevant topic, ensure that the program is interactive and accessible, and include decision support tools. | Time Factors | Male | Decision Making | Humans | OK |
| Marie Cécile | Dedianne | Christian | Dupraz | Corinne | Perdrix | Patient Education and Counseling | 2012-05-00 May 2012 | OBJECTIVE: To understand patients' perceptions of decision making and identify relationships among decision-making models. METHODS: This qualitative study was made up of four focus group interviews (elderly persons, users of health support groups, students, and rural inhabitants). Participants were asked to report their perceptions of decision making in three written clinical scenarios (hypertension, breast cancer, prostate cancer). The analysis was based on the principles of grounded theory. RESULTS: Most patients perceived decision making as shared decision making, a deliberative question-response interaction with the physician that allowed patients to be experts in obtaining clearer information, participating in the care process, and negotiating compromises with physician preferences. Requesting second opinions allowed patients to maintain control, even within the paternalistic model preferred by elderly persons. Facilitating factors (trust, qualitative non-verbal communication, time to think) and obstacles (serious/emergency situations, perceived inadequate scientific competence, problems making requests, fear of knowing) were also part of shared decision making. CONCLUSION AND PRACTICE IMPLICATIONS: In the global concept of patient-centered care, shared decision making can be flexible and can integrate paternalistic and informative models. Physicians' expertise should be associated with biomedical and relational skills through listening to, informing, and advising patients, and by supporting patients' choices. | Male | Decision Making | Humans | Patient Participation | OK |
| Alexander | Neishtadt |  |  |  |  | The Journal of Family Practice | 2014-04-00 Apr 2014 | PURPOSE: Little is known about patients who present a written list during a medical consultation.In this preliminary study, we sought to examine and characterize patients who use a prepared list. METHODS: The design was an open observational case-controlled study that took place at 2 urban primary care clinics. We enrolled patients consecutively as they arrived with a written list for consultation. Consecutive patients presenting without a list served as the control group. Physician interviews and completed questionnaires provided demographic and medical characteristics of this group and explanations for list preparation. RESULTS: Fifty-four patients presented with a list and were compared with controls. Statistically, patients arriving with a list were significantly more likely to be older and retired, and less likely to be salaried workers or housewives. These patients had more chronic diseases and consumed more long-term medications. They had a greater number of doctor visits in the past year compared with controls, and perceived an increase in memory loss. There were no differences between the groups in terms of psychiatric disease or personality disorders. CONCLUSIONS: Aside from certain demographic and health characteristics, patients who use written lists do not differ substantially from those who don't. They have no discernible ill intention, and the list serves as a memory aid to make the most of the visit. | Male | Humans | Patient Participation | Physician-Patient Relations | OK |
| Heidi | Keller | Lydia | Rosinger | Carsten | Sauer | Medical Decision Making | 2013-01-00 janvier 2013 | Background: Shared decision making (SDM) is often advocated as an ideal for making medical decisions. Until now, however, opinions regarding which treatment situations warrant SDM have not been systematically investigated. The purpose of this study was to examine social norms regarding medical decision making, using a factorial survey design. Methods: The factorial survey applied in this study consisted of 7 situational factors (e.g., the reason for consultation), each with 2 to 3 levels (e.g., prevention and severe disease). These factors were turned into various descriptions of treatment situations. A total of 101 physicians, 115 patients, and 113 members of self-help groups participated in the study. Each participant assessed 10 vignettes using a 5-point scale to indicate who they thought should make the decision in each specific situation. Results: Most assessments across the 3 groups called for a shared decision (39%). Ordered logistic regression analysis demonstrated that, according to study participants, all 7 situational factors (reason for consultation, time frame of negative outcomes, time pressure, number of therapeutic options, side effects, scientific evidence of efficacy, and desire to participate) significantly affected how decisions regarding treatment should be made. The strongest factor was the patient’s desire to participate in decision making (odds ratio = 1.84; P ≤ 0.001), followed by the reason for consultation (odds ratio = 0.69; P ≤ 0.001). Conclusions: This study reveals that there is a general desire for SDM in a variety of treatment situations. Furthermore, based on the responses of our participants, our findings also lay the framework in determining which treatment situations warrant SDM. (PsycINFO Database Record (c) 2013 APA, all rights reserved). (journal abstract) | Shared decision making | Decision Making | Therapeutic Processes | medical decision making | Doublon |
| Heidi | Keller | Lydia | Rosinger | Carsten | Sauer | Medical Decision Making: An International Journal of the Society for Medical Decision Making | 2013-01-00 Jan 2013 | BACKGROUND: Shared decision making (SDM) is often advocated as an ideal for making medical decisions. Until now, however, opinions regarding which treatment situations warrant SDM have not been systematically investigated. The purpose of this study was to examine social norms regarding medical decision making, using a factorial survey design. METHODS: The factorial survey applied in this study consisted of 7 situational factors (e.g., the reason for consultation), each with 2 to 3 levels (e.g., prevention and severe disease). These factors were turned into various descriptions of treatment situations. A total of 101 physicians, 115 patients, and 113 members of self-help groups participated in the study. Each participant assessed 10 vignettes using a 5-point scale to indicate who they thought should make the decision in each specific situation. RESULTS: Most assessments across the 3 groups called for a shared decision (39%). Ordered logistic regression analysis demonstrated that, according to study participants, all 7 situational factors (reason for consultation, time frame of negative outcomes, time pressure, number of therapeutic options, side effects, scientific evidence of efficacy, and desire to participate) significantly affected how decisions regarding treatment should be made. The strongest factor was the patient's desire to participate in decision making (odds ratio = 1.84; P ≤ 0.001), followed by the reason for consultation (odds ratio = 0.69; P ≤ 0.001). CONCLUSIONS: This study reveals that there is a general desire for SDM in a variety of treatment situations. Furthermore, based on the responses of our participants, our findings also lay the framework in determining which treatment situations warrant SDM. | Male | Decision Making | Humans | Questionnaires | OK |
|  |  |  |  |  |  | BMC medical research methodology | 2012-00-00 2012 | BACKGROUND: Randomized controlled trials (RCTs) remain an indispensable form of human experimentation as a vehicle for discovery of new treatments. However, since their inception RCTs have raised ethical concerns. The ethical tension has revolved around "duties to individuals" vs. "societal value" of RCTs. By asking current patients "to sacrifice for the benefit of future patients" we risk subjugating our duties to patients' best interest to the utilitarian goal for the good of others. This tension creates a key dilemma: when is it rational, from the perspective of the trial patients and researchers (as societal representatives of future patients), to enroll in RCTs? METHODS: We employed the trust version of the prisoner's dilemma since interaction between the patient and researcher in the setting of a clinical trial is inherently based on trust. We also took into account that the patient may have regretted his/her decision to participate in the trial, while a researcher may feel guilty because he/she abused the patient's trust. RESULTS: We found that under typical circumstances of clinical research, most patients can be expected not to trust researchers, and most researchers can be expected to abuse the patients' trust. The most significant factor determining trust was the success of experimental or standard treatments, respectively. The more that a researcher believes the experimental treatment will be successful, the more incentive the researcher has to abuse trust. The analysis was sensitive to the assumptions about the utilities related to success and failure of therapies that are tested in RCTs. By varying all variables in the Monte Carlo analysis we found that, on average, the researcher can be expected to honor a patient's trust 41% of the time, while the patient is inclined to trust the researcher 69% of the time. Under assumptions of our model, enrollment into RCTs represents a rational strategy that can meet both patients' and researchers' interests simultaneously 19% of the time. CONCLUSIONS: There is an inherent ethical dilemma in the conduct of RCTs. The factors that hamper full co-operation between patients and researchers in the conduct of RCTs can be best addressed by: a) having more reliable estimates on the probabilities that new vs. established treatments will be successful, b) improving transparency in the clinical trial system to ensure fulfillment of "the social contract" between patients and researchers. | Male | Emotions | Humans | Patient Participation | OK |
| Cees M. P. M. | Hertogh | Marike E. | de Boer | Julian C. | Hughes | Palliative Medicine | 2014-03-00 Mar 2014 | BACKGROUND: Dementia is a life-limiting disease without curative treatments. Patients and families may need palliative care specific to dementia. AIM: To define optimal palliative care in dementia. METHODS: Five-round Delphi study. Based on literature, a core group of 12 experts from 6 countries drafted a set of core domains with salient recommendations for each domain. We invited 89 experts from 27 countries to evaluate these in a two-round online survey with feedback. Consensus was determined according to predefined criteria. The fourth round involved decisions by the core team, and the fifth involved input from the European Association for Palliative Care. RESULTS: A total of 64 (72%) experts from 23 countries evaluated a set of 11 domains and 57 recommendations. There was immediate and full consensus on the following eight domains, including the recommendations: person-centred care, communication and shared decision-making; optimal treatment of symptoms and providing comfort (these two identified as central to care and research); setting care goals and advance planning; continuity of care; psychosocial and spiritual support; family care and involvement; education of the health care team; and societal and ethical issues. After revision, full consensus was additionally reached for prognostication and timely recognition of dying. Recommendations on nutrition and dehydration (avoiding overly aggressive, burdensome or futile treatment) and on dementia stages in relation to care goals (applicability of palliative care) achieved moderate consensus. CONCLUSION: We have provided the first definition of palliative care in dementia based on evidence and consensus, a framework to provide guidance for clinical practice, policy and research. | Humans | Aged | Middle Aged | Adult | OK |
|  |  |  |  |  |  | Disability and Rehabilitation | 2014-00-00 2014 | PURPOSE: The World Health Organization Disability Assessment Schedule (WHODAS 2.0) was considered as a potentially appropriate patient-reported outcome measure (PROM) for community rehabilitation services in the UK. The study explored qualitative aspects of the measure's content and content and construct validity. METHOD: A convenience sample of 10 community rehabilitation service users participated in semi-structured interviews and completed the WHODAS 2.0. Content analysis and a constant comparative method of analysis were applied. Participants' accounts were compared with the measure's content and its underlying construct of disability. RESULTS: Participants' reports of current difficulties were rich with accounts of bodily impairments and activity and participation limitations. WHODAS 2.0 content largely covered those activities that interviewees found difficult. Participants tended to conceptualize disability according to the medical model. The wording of the questionnaire allowed for ambiguity with respect to social perspectives on disability, which resulted in variability of scores. CONCLUSIONS: While WHODAS 2.0 content coverage appears comprehensive, the questionnaire in its current form tends to favor a medical construct of disability. We recommend caution when applying WHODAS 2.0 in contexts such as community rehabilitation, where social aspects of disability may be considered important. Further investigation of the measure's construct validity might be warranted. | Male | Humans | Patient Participation | Female | OK |
|  |  |  |  |  |  | Hospital Peer Review | 2012-04-00 Apr 2012 | | Humans | Patient Participation | United States | Outcome Assessment (Health Care) | OK |
| Sarah | Reiter | Rudolf | Cohen | Markus | Bühner | The Journal of Clinical Psychiatry | 2011-12-00 Dec 2011 | OBJECTIVE: Treatment guidelines for schizophrenia recommend that medical decisions should be shared between patients with schizophrenia and their physicians. Our goal was to determine why some patients want to participate in medical decision making and others do not. METHOD: To identify determinants of participation preferences in schizophrenia patients (ICD-10 criteria) and in a nonpsychiatric comparison group (multiple sclerosis), we undertook a cross-sectional survey in 4 psychiatric and neurologic hospitals in Germany. Inpatients suffering from schizophrenia or multiple sclerosis (but not both) were consecutively recruited (2007-2008), and 203 patients participated in the study (101 with schizophrenia and 102 with multiple sclerosis). Predictors for patients' participation preferences were identified using a structural equation model. RESULTS: Patients' reports about their participation preferences in medical decisions can be predicted to a considerable extent (52% of the variance). For patients with schizophrenia, poor treatment satisfaction (P < .001), negative attitudes toward medication (P < .05), better perceived decision making skills (P < .001), and higher education (P < .01) were related to higher participation preferences. In the comparison group, drug attitudes (P < .05) and education (P < .05) were also shown to be related with participation preferences. CONCLUSIONS: Patients with schizophrenia who want to participate in decision making are often dissatisfied with care or are skeptical toward medication. Patients who judge their decisional capacity as poor or who are poorly educated prefer not to participate in decision making. Future implementation strategies for shared decision making must address how dissatisfied patients can be included in decision making and how patients who currently do not want to share decisions can be enabled, empowered, and motivated for shared decision making. | Male | Decision Making | Humans | Patient Participation | OK |
|  |  |  |  |  |  | Journal of General Internal Medicine | 2012-05-00 May 2012 | BACKGROUND: There is a growing awareness that patients should be more active and effective managers of their health and health care. Recent studies have found patient activation--or having the knowledge, skills, and confidence to manage one's health, to be related to health-related outcomes. These studies have often relied on self-reported outcomes and often have used small samples. OBJECTIVE: To examine the degree to which patient activation is related to a broad range of patient health and utilization outcomes in a large, insured population. DESIGN: Cross-sectional study of patients at Fairview Health Services in Minnesota. Data on patient activation and patient outcomes were derived from the electronic health record, abstracted in December 2010. PATIENTS: A total of 25,047 adult patients were included in the analysis. They all had a primary care visit in the prior six months and completed the patient activation measure as part of an office visit. MAIN MEASURES: The key independent measure was the Patient Activation Measure. We examined 13 patient outcomes across four areas: prevention, unhealthy behaviors, clinical indicators, and costly utilization. RESULTS: In multivariate models, patient activation was related to 12 of 13 patient outcomes in the expected direction. For every additional 10 points in patient activation, the predicted probability of having an ED visit, being obese, or smoking was one percentage point lower. The likelihood of having a breast cancer screen or clinical indicators in the normal range (A1c, HDL, and triglycerides) was one percentage point higher. CONCLUSIONS: This cross sectional study finds that patient activation is strongly related to a broad range of health-related outcomes, which suggests improving activation has great potential. Future work should examine the effectiveness of interventions to support patient activation. | Male | Humans | Patient Participation | Female | OK |
| Maedean | Brown | Cheryl | Ethier | David | Wells | Intensive Care Medicine | 2012-01-00 Jan 2012 | PURPOSE: Consent for research participation in the intensive care unit (ICU) is often obtained from a substitute decision maker (SDM). In this study we explored SDMs' reasons for declining or providing consent for research studies for critically ill adult family members. METHODS: Two questionnaires were developed, one directed at SDMs who agreed to have their relative participate in a research study (AGREE group), and another for SDMs who declined participation (DECLINE group). The questionnaires explored SDMs' opinions about research in general, timing of research approach, the informed consent process, and reasons for agreeing or declining participation. RESULTS: Ninety-six SDMs completed the questionnaire (68 AGREE, 27 DECLINE). There were no differences between AGREE and DECLINE groups with respect to SDM demographics, perceived severity of illness of the patient, or the research study approach. The most common reasons for providing consent were potential for research to help others (91%), research is important for medical progress (88%), and trust in the medical team (87%). The most common reasons for declining consent were SDM was too anxious to consider research (67%), fear that patient would receive experimental treatment (37%), and concern about risks of the study (33%). CONCLUSIONS: SDMs who agree to have a relative participate in an ICU research study are motivated by the potential benefit to the patient and altruism. SDMs who decline research participation, while not generally opposed to research, are fearful of study-related harm or discomfort for the patient, and are too anxious to consider a research study at that time. | Male | Decision Making | Humans | Patient Participation | OK |
|  |  |  |  |  |  | Journal of Medical Internet Research | 2011-00-00 2011 | Active patient and public involvement as partners in their own health care and in the development of health services is key to achieving a health care system that is responsive to patients' needs and values. It promotes better use of the health care system, and improves health outcomes, quality of life and patient satisfaction. By involving patients and health care professionals as partners in the creation and updating of patient health support tools, wikis--highly accessible, interactive vehicles of communication--have the potential to empower users to implement these support tools in daily life. Acknowledging the potential of wikis, and recognizing that they capitalize on the free and open access to information, scientists, opinion leaders and patient advocates have suggested that wikis could help decision-making constituencies improve the delivery of health care. They might also decrease its cost and improve access to knowledge within developing countries. However, little is known about the efficacy of wikis in helping to attain these goals. There is also a need to know more about the intention of patients and health care workers to use wikis, in what circumstances and what factors will influence their use of wikis. In this issue of the Journal of Medical Internet Research, Gupta et al describe how they developed and tested a new wiki-inspired application to improve asthma care. The researchers involved patients with asthma, primary care physicians, pulmonologists and certified asthma educators in the construction of an asthma action plan. Their paper--entitled "WikiBuild: a new online collaboration process for multistakeholder tool development and consensus building"--is the first description of a wiki-inspired technology built to involve patients and health care professionals in the development of a patient support tool. This innovative study has made important contributions toward how wikis could be generalized to involve multiple stakeholders in the development of other knowledge translation tools such as clinical practice guidelines or decision aids. More specifically, Gupta et al have uncovered potential action mechanisms toward increasing usage of these tools by patients and health care professionals. These are decreasing hierarchical influences, increasing usability and adapting a tool to local context. More research is now needed to determine if the use of the resulting wiki-developed plan will actually be higher than a plan developed using other methods. Furthermore, there is also a need to assess the intention of participants to continue using wiki-based processes on an ongoing basis. It is in this dynamic and continuous retroaction loop that the support tool users--both patients and health care professionals--can adapt and improve the product after its real-life shortcomings are revealed and as new evidence becomes available. As such, a wiki would be more than a simple patient support development tool, but could also become a dynamic and interactive repository and delivery tool that would facilitate ongoing and sustainable patient and professional engagement. | Humans | Patient Participation | Internet | Cooperative Behavior | OK |
| Janan | Dietrich | Guy | de Bruyn | Martin | van der Watt | Journal of Acquired Immune Deficiency Syndromes (1999) | 2011-10-01 Oct 1, 2011 | OBJECTIVES: Adolescents may be appropriate for inclusion in biomedical HIV prevention trials. Adolescents' overall willingness to participate (WTP) in biomedical HIV prevention trials was examined, including after the prematurely discontinued phase IIb HVTN 503/Phambili HIV vaccine trial, in Soweto, South Africa. METHODS: An interview-administered cross-sectional survey was conducted among 506 adolescents (16-18 years) between October 2008 and March 2009. The assessment included WTP in HIV prevention trials, sexual and substance use behavior, and related psychosocial constructs. Multivariate logistic regression analyses examined predictors of WTP in biomedical prevention trials. RESULTS: The sample primarily consisted of female participants (n = 298, 59%), and 50% of all participants were sexually active. WTP in general was high (93%), with 75% WTP in a vaccine trial after being informed about the HVTN 503/Phambili trial. Less exposure to stressors [odds ratio (OR): 2.8, confidence interval (CI): 1.3 to 6.3] was associated with adolescents' WTP in HIV biomedical prevention trials overall. Those with less exposure to stressors (OR: 1.7, CI: 1.1 to 2.8) and not sexually active (OR: 2.1, CI: 1.4 to 3.3) were predictive of WTP after the HVTN 503/Phambili trial. A higher number of sexual partners were associated with unwillingness to participate more generally (P = 0.039) and specifically after the HIV vaccine trial (P = 0.0004). CONCLUSIONS: The high level of adolescents' WTP in biomedical prevention trials is encouraging, especially after the prematurely discontinued HVTN 503/Phambili HIV vaccine trial. High-risk youth were less likely to be WTP, although those not yet sexually active were more WTP. Future biomedical HIV prevention trials should address challenges to enrollment of high-risk adolescents who may show less WTP. | Male | Humans | Patient Participation | Female | OK |
| Edward R. | Marcantonio | Marcie B. | Freeman | Bruce E. | Landon | Journal of Cultural Diversity | 2011-00-00 2011 | The objective of this study was to identify racial differences in willingness to participate in a population with previous exposure to clinical research. A survey instrument was administered to community-dwelling whites and African Americans who were voluntarily receiving a lay research and health education newsletter from a local Boston geriatric clinical research institution. The survey instrument assessed willingness to participate in 3 hypothetical clinical trials (diet trial for obesity, medication trial for hypertension [HTN], chemotherapy trial for cancer). Surveys were received from 473 whites and 279 African Americans (53% response rate) with mean age 74 (SD +/- 9). In multivariate models, race was not significantly related to willingness to participate in the multivariate models for any of the 3 trials. Previous trial participation was related to a higher odds of willingness to participate in the diet trial only (OR 1.8, 95% CI 1.2, 2.6). Lower levels of trust in one's primary care physician were associated with a lower odds of willingness to participate in clinical trials for the diet and HTN trials (OR 0.5, 95% CI 0.3, 0.8 and OR 0.6, 95% CI 0.3, 0.9, respectively). These findings suggest that, within populations previously exposed to clinical research, African Americans are no less willing to participate in clinical trials compared to whites. | Male | Humans | Patient Participation | Questionnaires | OK |
|  |  |  |  |  |  | AIDS and behavior | 2012-08-00 Aug 2012 | This article examines willingness to participate in future HIV prevention research among gay and bisexual men in Scotland, UK. Anonymous, self-complete questionnaires and Orasure™ oral fluid samples were collected in commercial gay venues. 1,320 men were eligible for inclusion. 78.2% reported willingness to participate in future HIV prevention research; 64.6% for an HIV vaccine, 57.4% for a behaviour change study, and 53.0% for a rectal microbicide. In multivariate analysis, for HIV vaccine research, greater age, minority ethnicity, and not providing an oral fluid sample were associated with lower willingness; heterosexual orientation and not providing an oral fluid sample were for microbicides; higher education and greater HIV treatment optimism were for behaviour change. STI testing remained associated with being more willing to participate in microbicide research and frequent gay scene use remained associated with being more willing to participate in behaviour change research. Having an STI in the past 12 months remained significantly associated with being willing to participate in all three study types. There were no associations between sexual risk behaviour and willingness. Although most men expressed willingness to participate in future research, recruitment of high-risk men, who have the potential to benefit most, is likely to be more challenging. | Male | Humans | Patient Participation | Questionnaires | OK |
| Zhenhai | Zhou | Shuming | Li | Yingjie | Liu | Vaccine | 2010-06-23 Jun 23, 2010 | HIV vaccine trials require volunteers. Little is known about willingness to participate (WTP) in HIV vaccine trials among Chinese MSM. A survey of 550 MSM was conducted from March to June 2008, in Beijing, China. Data were collected on demographics, behaviors, perceptions about HIV/AIDS and HIV vaccines, and concerns about participation in HIV vaccine clinical trials. Of study participants, 35.8% were definitely willing to participate, 35.1% were probably willing, 16.4% were probably not willing, and 12.7% were definitely not willing. Analyses suggest that perceived family support, perceived protection against HIV infection and fear that participation would result in social distancing were associated with WTP. MSM in China may be good candidates for HIV vaccine trials. Further studies are needed to evaluate actual enrollment. | Male | Humans | Patient Participation | Questionnaires | OK |
| Dongliang | Li | Yingjie | Liu | Stephen W. | Pan | PloS One | 2013-00-00 2013 | BACKGROUND: Early detection of HIV infection enables timely care and treatment. However, many men who have sex with men (MSM) remain unaware of their HIV status because they do not or are unable to access HIV testing services. Oral fluid HIV rapid tests have the potential to increase HIV testing. This study is the first to evaluate willingness to use the oral fluid test among MSM in China. METHODS: A cross-sectional study was conducted in Beijing from July to October, 2012. Data were collected by self-administered questionnaires. RESULTS: Of 262 who participated in the survey, 223(85.1%) reported that they were willing to use the oral fluid HIV rapid test. Willingness to use the oral fluid test was associated with higher education (adjusted odds ratio (AOR): 2.40, 95% confidence interval (CI): 1.13-5.10), lack of unprotected anal intercourse (UAI) with male partners in the past one month (AOR: 2.38; 95% 95%CI: 1.15-4.95), having taken more than 4 HIV tests (AOR: 3.54; 95%CI:1.52-8.28), and having ever heard of the oral fluid HIV rapid test from gay friends or gay organizations (AOR: 3.24, 95%CI: 1.40-7.51). Among those who expressed willingness to use the oral fluid HIV rapid test, the median amount of money they were willing to pay was 8 dollars. Among the 39 participants who were unwilling to use the oral fluid test, 79.5% (31/39) expressed concerns about the accuracy of the oral fluid HIV rapid test results and 17.9%(7/39) reported that they were not familiar with the oral fluid test and did not know how to use such a test. CONCLUSIONS: A high proportion of MSM in Beijing appear to be willing to use the oral fluid HIV rapid test. Appropriate cost and education measures could help improve acceptance of the oral fluid test. | Male | Humans | Patient Participation | Young Adult | OK |
| Jason | Coleman | Yong | Gao | Steriani | Elavsky | Journal of Physical Activity & Health | 2013-03-00 Mar 2013 | BACKGROUND: Ideal approaches to increasing long-term physical activity (PA) adherence in women remain unclear. This study used a longitudinal mixed-methods approach to 1) determine the effectiveness of an 8-month book club intervention for increasing PA participation and self-worth, and reducing barriers at 1-year followup; and 2) identify reasons why completers and noncompleters did or did not maintain PA. METHODS: One year after the cessation of Women Bound to be Active (WBA), completers (participated in posttesting; n = 30) and noncompleters (did not participate in posttesting; n = 22) responded to questionnaires and interviews assessing their body mass index (BMI), current PA participation, barriers, and global self-worth. RESULTS: Compared with noncompleters, completers reported decreases in BMI, higher motivation for PA, higher ratio of benefits to barriers, and more consistent PA. Both groups still reported barriers to PA, especially time; however, completers more often reported strategies for overcoming these barriers. Completers more directly discussed the impact of their improved self-worth on their PA participation. CONCLUSIONS: In the future, a greater focus on time management and self-regulation strategies should be emphasized in PA interventions, specifically those that focus on women. This may help to prevent program and long-term PA attrition. | Humans | Patient Participation | Questionnaires | Female | OK |
| Sandy | Brooks | Suzanne | Tough | Patricia A. | Janssen | BMC pregnancy and childbirth | 2012-00-00 2012 | BACKGROUND: Much attention has been given to the adequacy of prenatal care use in promoting healthy outcomes for women and their infants. Adequacy of use takes into account the timing of initiation of prenatal care and the number of visits. However, there is emerging evidence that the quality of prenatal care may be more important than adequacy of use. The purpose of our study was to explore women's and care providers' perspectives of quality prenatal care to inform the development of items for a new instrument, the Quality of Prenatal Care Questionnaire. We report on the derivation of themes resulting from this first step of questionnaire development. METHODS: A qualitative descriptive approach was used. Semi-structured interviews were conducted with 40 pregnant women and 40 prenatal care providers recruited from five urban centres across Canada. Data were analyzed using inductive open and then pattern coding. The final step of analysis used a deductive approach to assign the emergent themes to broader categories reflective of the study's conceptual framework. RESULTS: The three main categories informed by Donabedian's model of quality health care were structure of care, clinical care processes, and interpersonal care processes. Structure of care themes included access, physical setting, and staff and care provider characteristics. Themes under clinical care processes were health promotion and illness prevention, screening and assessment, information sharing, continuity of care, non-medicalization of pregnancy, and women-centredness. Interpersonal care processes themes were respectful attitude, emotional support, approachable interaction style, and taking time. A recurrent theme woven throughout the data reflected the importance of a meaningful relationship between a woman and her prenatal care provider that was characterized by trust. CONCLUSIONS: While certain aspects of structure of care were identified as being key dimensions of quality prenatal care, clinical and interpersonal care processes emerged as being most essential to quality care. These processes are important as they have a role in mitigating adverse outcomes, promoting involvement of women in their own care, and keeping women engaged in care. The findings suggest key considerations for the planning, delivery, and evaluation of prenatal care. Most notably, care should be woman-centred and embrace shared decision making as an essential element. | Humans | Questionnaires | Female | Adult | OK |
|  |  |  |  |  |  | Journal of the American Academy of Nurse Practitioners | 2010-11-00 Nov 2010 | PURPOSE: To describe women's experiences during decision making about hormonal and nonhormonal therapies during the menopausal transition. DATA SOURCES: Transcripts from 21 semi-structured audio taped interviews with seven peri- and postmenopausal women who had a recently visited a nurse practitioner (NP) and were making a decision about menopausal management. CONCLUSIONS: Decision making was a nonlinear process in which women considered available options, weighed benefits and risks and likely outcomes. Reevaluation of the decision was ongoing. Both internal and external conditions influenced their decisions. Media reports of findings from the Women's Health Initiative study may have influenced some women's perceptions of the risk of using hormones for symptom relief. IMPLICATIONS FOR PRACTICE: Women described caring and empowering consultations with the NPs. They appreciated provision of information, adequate time spent at the visit, and decision support. NPs have a critical role to play in providing women with current research findings about hormone therapy and alternatives for symptom relief, and assisting women with understanding risks and benefits of each possible choice. Both individual and group approaches for decision support should be available to women. The approach of a collaborative partnership in decision making is a model that is congruent with nursing practice. | Decision Making | Decision Support Techniques | Humans | Patient Participation | OK |
| D. K. | Raynor | Karl | Atkin |  |  | Health Policy (Amsterdam, Netherlands) | 2013-10-00 Oct 2013 | Current healthcare policy in the UK has been shaped by two major forces; increasing accountability to evidence-based standards and increasing patient involvement. Shared decision-making brings the patient into prescribing decisions, and guidelines introduce a third decision-maker, the policy maker, into the doctor-patient consultation. This study explored the decision-making processes used by patients and GPs in comparison to local policy makers. METHOD: Qualitative interviews with 8 GPs, 14 patients and 2 PCT Prescribing Advisers, followed by quantitative questionnaires completed by 305 GPs and 533 patients. RESULTS: Patients made individual medicine-taking decisions based on experience, personal financial and human cost, trust and the relational aspects of their interactions with doctors over time. In contrast local implementation of prescribing guidelines was based on consideration of financial costs, efficacy and risks, based on objective clinical evidence at a population level. GPs adopted a mid-position between these two polar views. Guidelines are written from a different perspective to the worldview of patients, and they tend to downplay the criteria most important to patients. This has the potential to have a harmful effect on patients' medicine-taking and adherence. Paradoxically, enforcing the use of guidelines could inhibit the achievement of guideline targets. | Male | Decision Making | Humans | Physician-Patient Relations | Doublon |
| D. K. | Raynor | Karl | Atkin |  |  | Health Policy (Amsterdam, Netherlands) | 2013-10-00 Oct 2013 | Current healthcare policy in the UK has been shaped by two major forces; increasing accountability to evidence-based standards and increasing patient involvement. Shared decision-making brings the patient into prescribing decisions, and guidelines introduce a third decision-maker, the policy maker, into the doctor-patient consultation. This study explored the decision-making processes used by patients and GPs in comparison to local policy makers. METHOD: Qualitative interviews with 8 GPs, 14 patients and 2 PCT Prescribing Advisers, followed by quantitative questionnaires completed by 305 GPs and 533 patients. RESULTS: Patients made individual medicine-taking decisions based on experience, personal financial and human cost, trust and the relational aspects of their interactions with doctors over time. In contrast local implementation of prescribing guidelines was based on consideration of financial costs, efficacy and risks, based on objective clinical evidence at a population level. GPs adopted a mid-position between these two polar views. Guidelines are written from a different perspective to the worldview of patients, and they tend to downplay the criteria most important to patients. This has the potential to have a harmful effect on patients' medicine-taking and adherence. Paradoxically, enforcing the use of guidelines could inhibit the achievement of guideline targets. | Male | Decision Making | Humans | Physician-Patient Relations | OK |
| Anne-Deborah | Bouhnik | Mégane | Meresse | Camille | Cluze | Psycho-Oncology | 2013-11-00 Nov 2013 | OBJECTIVE: The objective of this study is to investigate young breast cancer patients' preferred and actual involvement in decision-making about surgery, chemotherapy, and adjuvant endocrine therapy (AET). METHODS: A total of 442 women aged 18-40 years at the time of the diagnosis participated in the region-wide ELIPPSE40 cohort study (southeastern France). Logistic regression analyses were performed on various factors possibly affecting patients' preferred and perceived involvement in the decisions about their cancer treatment. RESULTS: The women's mean age was 36.8 years at enrollment. Preference for a fully passive role in decision-making was stated by 20.7% of them. It was favored by regular breast surveillance (p = 0.04) and positive experience of being informed about cancer diagnosis (p = 0.02). Patients' preferences were independently associated with their reported involvement in decision-making about surgery (p = 0.01). A fully passive role in decision-making about chemotherapy and AET was more likely to be reported by patients who perceived their involvement in decision-making about surgery as having been fully passive (adjusted odds ratio = 4.8, CI95% [2.7-8.7], and adjusted odds ratio = 9.8, CI95% [3.3-29.2], respectively). This study shows a significant relationship between the use of antidepressants and involvement in decision-making about surgery, and confirms the relationship between impaired quality of life (in the psychological domain) and a fully passive role in decisions about cancer treatment. CONCLUSIONS: Patients' involvement in decision-making about chemotherapy and AET was strongly influenced by their experience of decision-making about surgery, regardless of their tumor stage and history of breast or ovarian cancer. When decisions are being made about surgery, special attention should be paid to facilitating breast cancer patients' involvement in the decision-making. | Decision Making | Humans | Patient Participation | Patient Satisfaction | OK |
|  |  |  |  |  |  | Child Welfare | 2010-00-00 2010 | This paper presents findings from a landmark Australian study investigating the experiences and perspectives of young people in residential care. Data from a representative sample are analyzed to identify young people's satisfaction with various aspects of their residential care experience: their sense of safety, normality, support, comfort in general living environment, participation in decision-making, and improvements in well-being. Findings point to strengths and weaknesses in current service delivery. The vast majority of respondents felt safe and well-treated and satisfied with the care and support provided by staff. Respondents were less commonly satisfied with the care and support provided by caseworkers, their participation in higher order decision-making, their sense of normality, and the amount of contact with their families. Compared with older respondents, younger respondents less commonly expressed satisfaction with various aspects of their care. Similarly, those reporting more placements were less satisfied with their care and support than those reporting fewer placements. | Male | Humans | Patient Participation | Patient Satisfaction | OK |
| Anita | Bundy |  |  |  |  | Disability and Rehabilitation | 2013-00-00 2013 | PURPOSE: To examine the meaningfulness, usefulness and acceptability of a new self-report instrument: "your ideas about participation and environment" (YIPE). METHOD: Cognitive interviewing was employed with 10 adults with disabilities to explore YIPE questions and formatting. Thematic content procedures were used in data analysis. The instrument was revised; participants were re-interviewed and data were re-examined. RESULTS: Participants considered the YIPE as a helpful tool to assist with developing person-centred goals and communicating their needs to health professionals, thus establishing its meaningfulness. They believed it could be useful at various points in the intervention process (e.g. preparation, monitoring, and evaluation). Participants also endorsed the YIPE's computer-based format, thus confirming its acceptability. CONCLUSIONS: The findings support the meaningfulness, acceptability and usefulness of the YIPE and consequently its potential for empowering service users and promoting person-centred care. Using the International Classification of Functioning, Disability and Health as the basis resulted in an instrument that was appropriate for understanding the interrelationship between participation and the supportiveness of the usual environment in which participation generally takes place. IMPLICATIONS FOR REHABILITATION: The YIPE is a useful tool to assist with developing person-centred goals, communicate needs with health professionals and it can be used at various points in a therapy process (preparation, monitoring or evaluation). The interrelationship between participation and the environment is important to consider when measuring functioning. Self-report empowers people to play an active role in interactions with health professionals and promote their own care. | Male | Humans | Patient Participation | Questionnaires | OK |
|  |  |  |  |  |  |  |  |  |  |  |  |  |  |

The first one is intituled "Additional_file1.xls" : List of records identified trough database searching (n=1365) and after removed (n=1255). Description of data : title, author 1 to 5 (first and last name), name of journal, date of publication, abstract, tag 1 to 4, selection's status (included or duplicate).
