# Supplementary material for: Evaluating the quality of shared decision making during the patient-carer encounter: a systematic review of tools
Source: BMC Res Notes. 2016 Aug 2;9:382. doi: 10.1186/s13104-016-2164-6 (PMC4971727; doi:10.1186/s13104-016-2164-6)
Supplement: Supplementary file 2 — 10.1186/s13104-016-2164-6 List of records of 20% and 10 % random sample selected by two independent observers and calcul of coefficients of concordance κ1 and κ2. Description of data: Sheet 1: title, selection’s status NB reading 1 20 % titles (excluded/included), selection’s status LM reading 1 20 % titles (excluded/included), comparison between 2 observers, kappa 1. Sheet 2: title, selection’s status NB reading 2 10 % titles (excluded/included), selection’s status LM reading 2 10 % titles (excluded/included), comparison between 2 observers, selection’s status adjustment after discussions (excluded/included), kappa 2. [file 13104_2016_2164_MOESM2_ESM.docx]

| **Title** | **Excluded(0) /Included(1) LM (Reading 2 ; 10% titles)** | **Excluded(0) /Included(1) NB (Reading 2 ; 10% titles)** | **Comparison between 2 observers (Reading 2)** | **Excluded(0) /Included(1) Adjustement after dicussions** |  |  |  |  |
| --- | --- | --- | --- | --- | --- | --- | --- | --- |
| A latent variable framework for modeling dyadic measures in research on shared decision-making | 1 | 1 | OK |  |  |  |  |  |
| Adaptive Conjoint Analysis as individual preference assessment tool: feasibility through the internet and reliability of preferences | 1 | 1 | OK |  |  |  |  |  |
| Anesthesia and analgesia-related preferences and outcomes of women who have birth plans | 1 | 1 | OK |  |  |  |  |  |
| Differences in information seeking among breast, prostate, and colorectal cancer patients: results from a population-based survey | 1 | 1 | OK |  |  |  |  |  |
| Do the public share practitioners' views about the best evidence? | 1 | 1 | OK |  |  |  |  |  |
| Effects of an interactive tailored patient assessment on patient-clinician communication in cancer care | 1 | 1 | OK |  |  | **Reading 2** |  |  |
| Patient autonomy in multiple sclerosis--possible goals and assessment strategies | 1 | 1 | OK |  |  |  |  |  |
| Patient experiences with public hospital care: first benchmark survey in Hong Kong | 1 | 1 | OK |  |  |  | score juge B (LM) | |
| Patient involvement in teaching and assessing intimate examination skills: a systematic review | 1 | 1 | OK |  |  | score juge A (NB) | score=0 (exclus) | score=1 (inclus) |
| Patient-experienced effect of an active implementation of a disease management programme for COPD - a randomised trial | 1 | 1 | OK |  |  | score=0 (exclus) | 81 (0,6328) | 7 |
| Patients' perspectives of the substitute decision maker: who makes better decisions? | 1 | 1 | OK |  |  | score=1 (inclus) | 11 | 29 (0,2266) |
| Pre-dialysis patients' perceived autonomy, self-esteem and labor participation: associations with illness perceptions and treatment perceptions. A cross-sectional study | 1 | 1 | OK |  |  |  | 92 | 36 |
| Your ideas about participation and environment: a new self-report instrument | 1 | 1 | OK |  |  | **Pobs=0,859** |  |  |
| Are there racial differences in patients' shared decision-making preferences and behaviors among patients with diabetes? | 1 | 1 | OK |  |  |  |  |  |
| Decision dissonance: evaluating an approach to measuring the quality of surgical decision making | 1 | 1 | OK |  |  |  |  |  |
| Development and structural validation of a shortened version of the Participation Scale | 1 | 1 | OK |  |  |  | score juge B (LM) | |
| Development, validation, and results of a survey to measure understanding of cardiopulmonary resuscitation choices among ICU patients and their surrogate decision makers | 1 | 1 | OK |  |  | score juge A (NB) | score=0 (exclus) | score=1 (inclus) |
| Disparities in patient reports of communications to inform decision making in the DECISIONS survey | 1 | 1 | OK |  |  | score=0 (exclus) | 63.25 |  |
| Do physicians understand Type 2 diabetes patients’ perceptions of seriousness; the emotional impact and needs for care improvement? A cross-national survey | 1 | 1 | OK |  |  | score=1 (inclus) |  | 11.25 |
| Exploring patient activation in the clinic: measurement from three perspectives | 1 | 1 | OK |  |  |  |  |  |
| For what am I participating? The need for communication after receiving consent from biobanking project participants: experience in Japan | 1 | 1 | OK |  |  | **Pa=0,621** |  |  |
| Patient involvement in assessing consultation quality: a quantitative study of the Patient Enablement Instrument in Poland | 1 | 1 | OK |  |  |  |  |  |
| Patient involvement in surgical treatment decisions and satisfaction with the treatment results after lumbar intervertebral discectomy | 1 | 1 | OK |  |  |  |  |  |
| Patients' preferences for patient-centered communication: a survey from an outpatient department in rural Sierra Leone | 1 | 1 | OK |  |  | Pobs-Pa | 0.238 |  |
| Risk assessment and clinical decision making for colorectal cancer screening | 1 | 1 | OK |  |  | 1-Pa | 0.379 |  |
| Using freelisting to understand shared decision making in ADHD: parents' and pediatricians' perspectives | 1 | 1 | OK |  |  | kappa (2) | 0.62796834 |  |
| Using the Australian Therapy Outcome Measures for Occupational Therapy (AusTOMs-OT) to measure client participation outcomes | 1 | 1 | OK |  |  |  |  |  |
| Validity of the utrecht scale for evaluation of rehabilitation-participation | 1 | 1 | OK |  |  |  |  |  |
| Worlds apart? An exploration of prescribing and medicine-taking decisions by patients, GPs and local policy makers | 1 | 1 | OK |  |  | LM | nb inclus(1) | 36 |
| African American prostate cancer survivors' treatment decision-making and quality of life | 0 | 1 | FALSE | 1 |  |  | nb total | 128 |
| Attitudes toward concordance in psychiatry: a comparative, cross-sectional study of psychiatric patients and mental health professionals | 0 | 1 | FALSE | 1 |  |  | % | 28.13% |
| Attitudes toward shared decision-making and risk communication practices in residents and their teachers | 0 | 1 | FALSE | 0 |  |  |  |  |
| Breech presentation and choice of mode of childbirth: a qualitative study of women's experiences | 0 | 1 | FALSE | 0 |  |  |  |  |
| Clinical microsystems--a path to improved user involvement] | 0 | 1 | FALSE | 0 |  | NB | nb inclus(1) | 41 |
| Decision preparation, satisfaction and regret in a multi-center sample of men with newly diagnosed localized prostate cancer | 0 | 1 | FALSE | 1 |  |  | nb total | 128 |
| Development of PRIDe: A tool to assess physicians’ preference of role in clinical decision making | 0 | 1 | FALSE | 1 |  |  | % | 32.03% |
| 'It's not my job. I'm the patient not the doctor': patient perspectives on medicines management in the treatment of schizophrenia | 0 | 1 | FALSE | 0 |  |  |  |  |
| Patient participation in hospital wards--health personnel's experience] | 0 | 1 | FALSE | 1 |  |  |  |  |
| Pressure during decision making of continuous sedation in end-of-life situations in Dutch general practice | 0 | 1 | FALSE | 0 |  |  |  |  |
| Using measurement-based care with patient involvement to improve outcomes in depression | 0 | 1 | FALSE | 1 |  |  |  |  |
| Accessibility, usability, and usefulness of a Web-based clinical decision support tool to enhance provider-patient communication around Self-management TO Prevent (STOP) Stroke | 1 | 0 | FALSE | 1 |  |  |  |  |
| Identifying patient information needs about cancer clinical trials using a Question Prompt List | 1 | 0 | FALSE | 1 |  |  |  |  |
| Patients assessing students' assignments; making the patient experience real | 1 | 0 | FALSE | 1 |  |  |  |  |
| Roles of illness attributions and cultural views of cancer in determining participation in cancer-smart lifestyle among Chinese and Western youth in Australia | 1 | 0 | FALSE | 0 |  |  |  |  |
| Supporting treatment decision making in advanced cancer: a randomized trial of a decision aid for patients with advanced colorectal cancer considering chemotherapy | 1 | 0 | FALSE | 0 |  |  |  |  |
| The impact of benign gene expression classifier test results on the endocrinologist-patient decision to operate on patients with thyroid nodules with indeterminate fine-needle aspiration cytopathology | 1 | 0 | FALSE | 0 |  |  |  |  |
| Towards client-centered counseling: development and testing of the WHO Decision-Making Tool | 1 | 0 | FALSE | 1 |  |  |  |  |
| “If I didn’t have anybody, what would I have done?”: Experiences of older adults and their discharge home after lower limb orthopaedic surgery | 0 | 0 | OK |  |  |  |  |  |
| A review of the reporting and handling of missing data in cohort studies with repeated assessment of exposure measures | 0 | 0 | OK |  |  |  |  |  |
| A web-based electronic neurology referral system: a solution for an overburdened healthcare system? | 0 | 0 | OK |  |  |  |  |  |
| Activation among chronically ill older adults with complex medical needs: challenges to supporting effective self-management | 0 | 0 | OK |  |  |  |  |  |
| Adherence to behavioral interventions for urge incontinence when combined with drug therapy: adherence rates, barriers, and predictors | 0 | 0 | OK |  |  |  |  |  |
| Altruism among participants in cancer clinical trials | 0 | 0 | OK |  |  |  |  |  |
| An ontology-based tool for the correspondences between specialist and consumer medical lexicons for the geriatrics domain | 0 | 0 | OK |  |  |  |  |  |
| Arriba-lib: evaluation of an electronic library of decision aids in primary care physicians | 0 | 0 | OK |  |  |  |  |  |
| Assessing the added value of health technologies: reconciling different perspectives | 0 | 0 | OK |  |  |  |  |  |
| Assessing the risk of aortic valve replacement for severe aortic stenosis in the transcatheter valve era | 0 | 0 | OK |  |  |  |  |  |
| Asthma knowledge, attitude, and self-efficacy in Iranian asthmatic patients | 0 | 0 | OK |  |  |  |  |  |
| Attitudes and expectations of patients with neuromuscular diseases about their participation in a clinical trial | 0 | 0 | OK |  |  |  |  |  |
| Barriers to and facilitators of participation of older adults in a placebo-controlled randomized clinical trial | 0 | 0 | OK |  |  |  |  |  |
| Capturing acute toxicity data during lung radiotherapy by using a patient-reported assessment tool | 0 | 0 | OK |  |  |  |  |  |
| Changes of heart: the switch-value method for assessing value uncertainty | 0 | 0 | OK |  |  |  |  |  |
| Clinical implementation of quality of life instruments and prediction tools for localized prostate cancer: results from a national survey of radiation oncologists and urologists | 0 | 0 | OK |  |  |  |  |  |
| Comparing different strategies for colorectal cancer screening in Italy: predictors of patients' participation | 0 | 0 | OK |  |  |  |  |  |
| Deactivation of implanted cardioverter-defibrillators at the end of life: results of the EHRA survey | 0 | 0 | OK |  |  |  |  |  |
| Developing an interactive mobile phone self-report system for self-management of hypertension. Part 1: patient and professional perspectives | 0 | 0 | OK |  |  |  |  |  |
| Development of symptom assessments utilising item response theory and computer-adaptive testing--a practical method based on a systematic review | 0 | 0 | OK |  |  |  |  |  |
| Discussions about clinical trials among patients with newly diagnosed lung and colorectal cancer | 0 | 0 | OK |  |  |  |  |  |
| Do canadian researchers and the lay public prioritize biomedical research outcomes equally? A choice experiment | 0 | 0 | OK |  |  |  |  |  |
| Effect of Wellness Recovery Action Plan (WRAP) participation on psychiatric symptoms, sense of hope, and recovery | 0 | 0 | OK |  |  |  |  |  |
| Elevation of serum fortilin levels is specific for apoptosis and signifies cell death in vivo | 0 | 0 | OK |  |  |  |  |  |
| Emancipatory actions displayed by multi-ethnic women: "Regaining control of my health care" | 0 | 0 | OK |  |  |  |  |  |
| Everyday ethics and help-seeking in early rheumatoid arthritis | 0 | 0 | OK |  |  |  |  |  |
| Exercise participation barrier prevalence and association with exercise participation status in individuals with spinal cord injury | 0 | 0 | OK |  |  |  |  |  |
| Expanding sexually transmitted infection screening among women and men engaging in transactional sex: the feasibility of field-based self-collection | 0 | 0 | OK |  |  |  |  |  |
| Exploring pharmacist-customer communication: the established blood pressure measurement episode | 0 | 0 | OK |  |  |  |  |  |
| Factors affecting acceptance of a Web-based self-referral system | 0 | 0 | OK |  |  |  |  |  |
| Factors influencing patient safety in Sweden: perceptions of patient safety officers in the county councils | 0 | 0 | OK |  |  |  |  |  |
| Facts and figures about patient associations in the Netherlands between 2007 and 2009: review of their activities and aims | 0 | 0 | OK |  |  |  |  |  |
| From quality of life to palliative care in cancerology] | 0 | 0 | OK |  |  |  |  |  |
| Goal setting in inpatient medical rehabilitation: exploring the current practice] | 0 | 0 | OK |  |  |  |  |  |
| Impact of postpartum information about pertussis booster to parents in a university maternity hospital | 0 | 0 | OK |  |  |  |  |  |
| Information for decision making by post-menopausal women with hormone receptor positive early-stage breast cancer considering adjuvant endocrine therapy | 0 | 0 | OK |  |  |  |  |  |
| Integrating patient-reported outcomes in healthcare policy, research and practice | 0 | 0 | OK |  |  |  |  |  |
| Involving patient in the early stages of health technology assessment (HTA): a study protocol | 0 | 0 | OK |  |  |  |  |  |
| Maternal and infant predictors of attendance at Neonatal Follow-Up programmes | 0 | 0 | OK |  |  |  |  |  |
| Measure once, cut twice--adding patient-reported outcome measures to the electronic health record for comparative effectiveness research | 0 | 0 | OK |  |  |  |  |  |
| Measuring clients' perception of functional limitations using the Perceived Functioning & Health questionnaire | 0 | 0 | OK |  |  |  |  |  |
| Measuring pain perceptions and medication taking behavior at the end of life: a pilot study | 0 | 0 | OK |  |  |  |  |  |
| Mental health professionals' attitudes to partnership in medicine taking: a validation study of the Leeds Attitude to Concordance Scale II | 0 | 0 | OK |  |  |  |  |  |
| Multidisciplinary meetings in oncology do not impact the physician-patient relationship] | 0 | 0 | OK |  |  |  |  |  |
| New diagnostic concept of adjustment disorders in psychosomatic outpatients--symptom severity, willingness to change, psychotherapy motivation] | 0 | 0 | OK |  |  |  |  |  |
| Objective measurements of disease severity and diagnostic confirmation in atopic dermatitis and urticaria] | 0 | 0 | OK |  |  |  |  |  |
| One million vascular screening tests a year: a considered perspective | 0 | 0 | OK |  |  |  |  |  |
| Participation and barriers to colorectal cancer screening in Malaysia | 0 | 0 | OK |  |  |  |  |  |
| Patient and provider attitudes toward screening for Down syndrome in a Latin American country where abortion is illegal | 0 | 0 | OK |  |  |  |  |  |
| Patient web empowerment index (PWEI): an index for assessment of healthcare providers' web strategies. Case study: PWEI application in Italy | 0 | 0 | OK |  |  |  |  |  |
| Patient-centered plan-of-care tool for improving clinical outcomes | 0 | 0 | OK |  |  |  |  |  |
| Patient-reported outcomes in left ventricular assist device therapy: a systematic review and recommendations for clinical research and practice | 0 | 0 | OK |  |  |  |  |  |
| Payers test reference pricing and centers of excellence to steer patients to low-price and high-quality providers | 0 | 0 | OK |  |  |  |  |  |
| Potential impact of abrupt opioid therapy discontinuation in the management of chronic pain: a pilot study on patient perspective | 0 | 0 | OK |  |  |  |  |  |
| Predictors of willingness to participate in window-of-opportunity breast trials | 0 | 0 | OK |  |  |  |  |  |
| Pregnant women's perspectives on decision-making when a fetal malformation is detected by ultrasound examination | 0 | 0 | OK |  |  |  |  |  |
| Provider use of collaborative goal setting with glaucoma patients | 0 | 0 | OK |  |  |  |  |  |
| Rehabilitation goals in discharge reports and in patient questionnaires--on the example of endometriosis patients] | 0 | 0 | OK |  |  |  |  |  |
| Role of patient and public participation in health technology assessment and coverage decisions | 0 | 0 | OK |  |  |  |  |  |
| Study to assess the effect of a structured communication approach on quality of life in secure mental health settings (Comquol): study protocol for a pilot cluster randomized trial | 0 | 0 | OK |  |  |  |  |  |
| Synthesis of recommendations for the assessment and management of low back pain from recent clinical practice guidelines | 0 | 0 | OK |  |  |  |  |  |
| Systematic review of internet patient information on colorectal cancer surgery | 0 | 0 | OK |  |  |  |  |  |
| Testing the utility of a cancer clinical trial specific Question Prompt List (QPL-CT) during oncology consultations | 0 | 0 | OK |  |  |  |  |  |
| The facilitating factors and barriers encountered in the adoption of a humanized birth care approach in a highly specialized university affiliated hospital | 0 | 0 | OK |  |  |  |  |  |
| The Functional Living Index-Cancer: estimating its reliability based on clinical trial data | 0 | 0 | OK |  |  |  |  |  |
| The influence of adverse reactions, subjective distress, and anxiety on retention of first-time blood donors | 0 | 0 | OK |  |  |  |  |  |
| The Perceived Efficacy and Goal Setting System (PEGS), part II: evaluation of test-retest reliability and differences between child and parental reports in the Swedish version | 0 | 0 | OK |  |  |  |  |  |
| The rights of psychiatric patients in China: a survey of medical staff and consumers' attitudes toward patient participation in clinical trials | 0 | 0 | OK |  |  |  |  |  |
| Towards a multidimensional patient reported outcome measures assessment: development and validation of a questionnaire for patients with ankylosing spondylitis/spondyloarthritis | 0 | 0 | OK |  |  |  |  |  |
| United states acculturation and cancer patients' end-of-life care | 0 | 0 | OK |  |  |  |  |  |
| Using a population-based observational cohort study to address difficult comparative effectiveness research questions: the CEASAR study | 0 | 0 | OK |  |  |  |  |  |
| Using simulation to assess the influence of race and insurer on shared decision making in periviable counseling | 0 | 0 | OK |  |  |  |  |  |
| Using social media to engage patients: many tools exist to connect, communicate and build loyalty | 0 | 0 | OK |  |  |  |  |  |
| Validation of a new patient-generated questionnaire for quality of life in an urban sample of elder residents | 0 | 0 | OK |  |  |  |  |  |
| Willingness to participate in biomedical HIV prevention studies after the HVTN 503/Phambili trial: a survey conducted among adolescents in Soweto, South Africa | 0 | 0 | OK |  |  |  |  |  |
| Cancer patients and advance directives: a survey of patients in a hematology and oncology outpatient clinic | 0 | 0 | OK |  |  |  |  |  |
| Cesarean delivery by maternal request: surveys of obstetricians | 0 | 0 | OK |  |  |  |  |  |
| Experiences of care reported by adults with traumatic brain injury | 0 | 0 | OK |  |  |  |  |  |
| Patient-Perceived Pressure from Clinicians for Labor Induction and Cesarean Delivery: A Population-Based Survey of U.S. Women | 0 | 0 | OK |  |  |  |  |  |
| Shared decision making coding systems: how do they compare in the oncology context? | 0 | 0 | OK |  |  |  |  |  |
| Understanding treatment decision making in juvenile idiopathic arthritis: a qualitative assessment | 0 | 0 | OK |  |  |  |  |  |

The second one is "Additional_file2.xls" : List of records of 20% and 10 % random sample selected by two independent observers and calcul of coefficients of concordance κ1 and κ2. Description of data : Sheet 1 : title, selection's status NB reading 1 20 % titles (excluded/included), selection's status LM reading 1 20 % titles (excluded/included), comparison between 2 observers, kappa 1. Sheet 2 : title, selection's status NB reading 2 10 % titles (excluded/included), selection's status LM reading 2 10 % titles (excluded/included), comparison between 2 observers, selection's status adjustment after discussions (excluded/included), kappa 2.
