# Supplementary material for: Evaluating the quality of shared decision making during the patient-carer encounter: a systematic review of tools
Source: BMC Res Notes. 2016 Aug 2;9:382. doi: 10.1186/s13104-016-2164-6 (PMC4971727; doi:10.1186/s13104-016-2164-6)
Supplement: Supplementary file 3 — 10.1186/s13104-016-2164-6 List of full-text articles assessed for eligibility (n=86) and full-text articles excluded (n=67). Description of data: title, first author (last name), name of journal, date of publication, abstract, selection’s status (included or excluded), not relevant, study or instrument not accessible, other language, measured construct is not SDM, not about a clinical encounter, too specific. [file 13104_2016_2164_MOESM3_ESM.docx]

| **Title** | **Author1_Last** | **Name of journal** | **Date of publication** | **Abstract** | **Selection status : included (1) / excluded (0)** | **not relevant** | **study or instrument not accessible** | **other language (than english, french, spanish)** | **measured construct is not SDM** | **not about a clinical encounter** | **too specific** |
| --- | --- | --- | --- | --- | --- | --- | --- | --- | --- | --- | --- |
| A coding system to measure elements of shared decision making during psychiatric visits | Salyers | Psychiatric Services (Washington, D.C.) | 2012-08-00 Aug 2012 | OBJECTIVE: Shared decision making is widely recognized to facilitate effective health care. The purpose of this study was to assess the applicability and usefulness of a scale to measure the presence and extent of shared decision making in clinical decisions in psychiatric practice. METHODS: A coding scheme assessing shared decision making in general medical settings was adapted to mental health settings, and a manual for using the scheme was created. Trained raters used the adapted scale to analyze 170 audio-recordings of medication check-up visits with either psychiatrists or nurse practitioners. The scale assessed the level of shared decision making based on the presence of nine specific elements. Interrater reliability was examined, and the frequency with which elements of shared decision making were observed was documented. The association between visit length and extent of shared decision making was also examined. RESULTS: Interrater reliability among three raters on a subset of 20 recordings ranged from 67% to 100% agreement for the presence of each of the nine elements of shared decision making and 100% for the agreement between provider and consumer on decisions made. Of the 170 sessions, 128 (75%) included a clinical decision. Just over half of the decisions (53%) met minimum criteria for shared decision making. Shared decision making was not related to visit length after the analysis controlled for the complexity of the decision. CONCLUSIONS: The rating scale appears to reliably assess shared decision making in psychiatric practice and could be helpful for future research, training, and implementation efforts. | 0 | 1 |  |  |  |  |  |
| A patient self-assessment tool to measure communication behaviors during doctor visits about hypertension | Ashton | Patient Education and Counseling | 2010-11-00 Nov 2010 | OBJECTIVE: To develop a preliminary version of a post-doctor visit self-assessment tool that patients with hypertension can use to evaluate their communication behaviors. High-quality communication between patient and doctor may have a positive effect on blood pressure control in hypertensive patients. Patients' communicative behaviors such as asking questions influence those of doctors, but most existing measurement tools assess doctors' behaviors rather than patients'. METHODS: The tool is intended for use by African American or Caucasian American adults with hypertension, regardless of literacy level. The project included theory-based development of the item pool, usability testing (8 individuals), and cognitive response testing (13 additional individuals). MAIN RESULTS: After multiple iterations, the preliminary version includes 138 items in 7 theory-based domains. CONCLUSION AND PRACTICE IMPLICATIONS: The self-assessment tool is ready for testing of item and scale reliability and validity and consequent item reduction. This tool could prove useful in trials evaluating whether patients with hypertension who learn to be better communicators are more likely to achieve blood pressure control. In addition, because it asks patients to reflect on their use of specific behaviors that can be learned, the tool might also help patients in clinical practice to assume more active roles during their medical interactions. | 0 | 1 |  |  |  |  |  |
| A patient survey for emergency care designed by children, for children | Davies | Archives of Disease in Childhood | 2013-04-00 Apr 2013 | | 0 | 1 |  |  |  |  |  |
| Adaptation, data quality and confirmatory factor analysis of the Danish version of the PACIC questionnaire | Maindal | European Journal of Public Health | 2012-02-00 Feb 2012 | BACKGROUND: The Patient Assessment of Chronic Illness Care (PACIC) 20-item questionnaire measures how chronic care patients perceive their involvement in care. We aimed to adapt the measure into Danish and to assess data quality, internal consistency and the proposed factorial structure. METHODS: The PACIC was translated by a standardised forward-backward procedure, and filled in by 560 patients receiving type 2 diabetes care. Data quality was assessed by mean, median, item response, missing values, floor and ceiling effects, internal consistency (Cronbach's α and average inter-item correlation), item-rest correlations and factorial structure was assessed by confirmatory factor analysis (CFA). RESULTS: The item response was high (missing answers: 0.5-2.9%). Floor effect was 2.7-69.2%, above 15% for 17 items. Ceiling effect was 4.0-40.4%, above 15% for 12 items. The subscales had average inter-item correlations over 0.30 and CFA showed high factor loadings (range 0.67-0.77). All had α over 0.7 and included items with both high and low loadings. The CFA model fit was good for two indices out of six (TLI and SRMR). CONCLUSIONS: Danish PACIC is now available and validated in primary care in a type 2 diabetes population. The psychometric properties were satisfactory apart from ceiling and floor effects. We endorse the proposed five scale structure. All the subscales showed good model fit, and may be used for separate sum scores. | 0 | 1 |  |  |  |  |  |
| Advances in measuring culturally competent care: a confirmatory factor analysis of CAHPS-CC in a safety-net population | Stern | Medical Care | 2012-09-00 Sep 2012 | BACKGROUND: Providing culturally competent care shows promise as a mechanism to reduce health care inequalities. Until the recent development of the Consumer Assessment of Healthcare Providers and Systems Cultural Competency Item Set (CAHPS-CC), no measures capturing patient-level experiences with culturally competent care have been suitable for broad-scale administration. METHODS: We performed confirmatory factor analysis and internal consistency reliability analysis of CAHPS-CC among patients with type 2 diabetes (n=600) receiving primary care in safety-net clinics. CAHPS-CC domains were also correlated with global physician ratings. RESULTS: A 7-factor model demonstrated satisfactory fit (χ²₂₃₁=484.34, P<0.0001) with significant factor loadings at P<0.05. Three domains showed excellent reliability-Doctor Communication-Positive Behaviors (α=0.82), Trust (α=0.77), and Doctor Communication-Health Promotion (α=0.72). Four domains showed inadequate reliability either among Spanish speakers or overall (overall reliabilities listed): Doctor Communication-Negative Behaviors (α=0.54), Equitable Treatment (α=0.69), Doctor Communication-Alternative Medicine (α=0.52), and Shared Decision-Making (α=0.51). CAHPS-CC domains were positively and significantly correlated with global physician rating. CONCLUSIONS: Select CAHPS-CC domains are suitable for broad-scale administration among safety-net patients. Those domains may be used to target quality-improvement efforts focused on providing culturally competent care in safety-net settings. | 0 | 1 |  |  |  |  |  |
| An examination of the validity of EPSCALE using factor analysis | Edgcumbe | Patient Education and Counseling | 2012-04-00 Apr 2012 | OBJECTIVE: To examine the validity and utility of the Explanation and Planning Scale (EPSCALE) instrument, a widely used scale for teaching and assessment of explanation and planning skills used by clinicians during the medical interview. METHODS: Data obtained across 4 OSCE stations during medical student final MB examinations. Exploratory factor analysis, using a single factor and two factor models (based on prior theory) and a six factor empirical model, suggested by parallel analysis. PARTICIPANTS: 124 medical students sitting final MB examinations at the University of Cambridge. RESULTS: A single factor model represented a very poor fit. A two factor model with factors labelled 'Explanation' and 'Planning' produced an improved fit, but the best was seen with a six factor model, with factors which broadly corresponded to the domains of the Calgary-Cambridge guide. CONCLUSIONS: These factor models provide supportive evidence for the construct validity of EPSCALE. PRACTICE IMPLICATIONS: EPSCALE can justifiably be used in the assessment of shared-decision making skills. | 0 | 1 |  |  |  |  |  |
| Applying the theory of planned behaviour to multiple sclerosis patients' decisions on disease modifying therapy--questionnaire concept and validation | Kasper | BMC medical informatics and decision making | 2012-00-00 2012 | BACKGROUND: Patients making important medical decisions need to evaluate complex information in the light of their own beliefs, attitudes and priorities. The process can be considered in terms of the theory of planned behaviour. Decision support technologies aim at helping patients making informed treatment choices. Instruments assessing informed choices need to include risk knowledge, attitude (towards therapy) and actual uptake. However, mechanisms by which decision support achieves its goals are poorly understood.Our aim was therefore to develop and validate an instrument modeling the process of multiple sclerosis (MS) patients' decision making about whether to undergo disease modifying (immuno-)therapies (DMT). METHODS: We constructed a 30-item patient administered questionnaire to access the elaboration of decisions about DMT in MS according to the theory of planned behaviour. MS-patients' belief composites regarding immunotherapy were classified according to the domains "attitude", "subjective social norm" and "control beliefs" and within each domain to either "expectations" or "values" yielding 6 sub-domains. A randomized controlled trial (n = 192) evaluating an evidence based educational intervention tested the instrument's predictive power regarding intention to use immunotherapy and its sensitivity to the intervention. RESULTS: The psychometric properties of the questionnaire were satisfactory (mean item difficulty 62, mean SD 0.9, range 0-3). Responses explain up to 68% of the variability in the intention to use DMT was explained by up to 68% in the total sample. Four weeks after an educational intervention, predictive power was higher in the intervention (IG) compared to the control group (CG) (intention estimate: CG 56% / IG 69%, p = .179; three domains CG 56% / IG 74%, p = .047; six sub-domains CG 64% / IG 78%, p = .073). The IG held more critical beliefs towards immunotherapy (p = .002) and were less willing to comply with social norm (p = .012). CONCLUSIONS: The questionnaire seems to provide a valid way of explaining patients' inherent decision processes and to be sensitive towards varying levels of elaboration. Similar tools based on the theory of planned behaviour could be applied to other decision making scenarios. | 0 |  |  |  | 1 |  |  |
| Assessment of Interprofessional Team Collaboration Scale (AITCS): development and testing of the instrument | Orchard | The Journal of Continuing Education in the Health Professions | 2012-00-00 2012 | INTRODUCTION: Many health professionals believe they practice collaboratively. Providing insight into their actual level of collaboration requires a means to assess practice within health settings. This chapter reports on the development, testing, and refinement process for the Assessment of Interprofessional Team Collaboration Scale (AITCS). There is a paucity of literature and measurement tools addressing interprofessional collaborative team performance and the nature of effective teamwork processes and patient roles within collaborative teams. These gaps limit our knowledge about how health care teams form and function. Instruments are therefore needed to assess collaborative relationships. METHODS: The AITCS, with its 47 items within 4 subscales (partnership, cooperation, coordination, and shared decision making) and assessed on a 5-point Likert scale, was administered to a total of 125 practitioners from 7 health care teams practicing within a variety of settings, in 2 provinces in Canada. RESULTS: Principal components and factor analysis of data resulted in 37 items loading onto 3 factors, explaining 61.02% of the variance. The internal consistency estimates for reliability of each subscale ranged from 0.80 to 0.97, with an overall reliability of 0.98. Thus, the AITCS is a reliable and valid instrument. DISCUSSION: The psychometric analysis of this instrument supports its value in measuring collaboration within teams and when patients are included as team members. The AITCS can be applied to continuing professional education interventions to determine change over time. It has limitations to the Canadian context and within the settings where participants practiced. Further test and retest reliability and longitudinal study application is needed. | 0 |  |  |  |  | 1 |  |
| Assessment of youth-friendly health care: a systematic review of indicators drawn from young people's perspectives | Ambresin | The Journal of Adolescent Health: Official Publication of the Society for Adolescent Medicine | 2013-06-00 Jun 2013 | PURPOSE: To review the literature on young people's perspectives on health care with a view to defining domains and indicators of youth-friendly care. METHODS: Three bibliographic databases were searched to identify studies that purportedly measured young people's perspectives on health care. Each study was assessed to identify the constructs, domains, and indicators of adolescent-friendly health care. RESULTS: Twenty-two studies were identified: 15 used quantitative methods, six used qualitative methods and one used mixed methodology. Eight domains stood out as central to young people's positive experience of care. These were: accessibility of health care; staff attitude; communication; medical competency; guideline-driven care; age appropriate environments; youth involvement in health care; and health outcomes. Staff attitudes, which included notions of respect and friendliness, appeared universally applicable, whereas other domains, such as an appropriate environment including cleanliness, were more specific to particular contexts. CONCLUSION: These eight domains provide a practical framework for assessing how well services are engaging young people. Measures of youth-friendly health care should address universally applicable indicators of youth-friendly care and may benefit from additional questions that are specific to the local health setting. | 0 |  |  |  |  | 1 |  |
| Assessments of the extent to which health-care providers involve patients in decision making: a systematic review of studies using the OPTION instrument | Couët | Health Expectations: An International Journal of Public Participation in Health Care and Health Policy | 2013-03-04 Mar 4, 2013 | BACKGROUND: We have no clear overview of the extent to which health-care providers involve patients in the decision-making process during consultations. The Observing Patient Involvement in Decision Making instrument (OPTION) was designed to assess this. OBJECTIVE: To systematically review studies that used the OPTION instrument to observe the extent to which health-care providers involve patients in decision making across a range of clinical contexts, including different health professions and lengths of consultation. SEARCH STRATEGY: We conducted online literature searches in multiple databases (2001-12) and gathered further data through networking. INCLUSION CRITERIA: (i) OPTION scores as reported outcomes and (ii) health-care providers and patients as study participants. For analysis, we only included studies using the revised scale. DATA EXTRACTION: Extracted data included: (i) study and participant characteristics and (ii) OPTION outcomes (scores, statistical associations and reported psychometric results). We also assessed the quality of OPTION outcomes reporting. MAIN RESULTS: We found 33 eligible studies, 29 of which used the revised scale. Overall, we found low levels of patient-involving behaviours: in cases where no intervention was used to implement shared decision making (SDM), the mean OPTION score was 23 ± 14 (0-100 scale). When assessed, the variables most consistently associated with higher OPTION scores were interventions to implement SDM (n = 8/9) and duration of consultations (n = 8/15). CONCLUSIONS: Whatever the clinical context, few health-care providers consistently attempt to facilitate patient involvement, and even fewer adjust care to patient preferences. However, both SDM interventions and longer consultations could improve this. | 0 | 1 |  |  |  |  |  |
| Attitudes toward concordance in psychiatry: a comparative, cross-sectional study of psychiatric patients and mental health professionals | De las Cuevas | BMC psychiatry | 2012-00-00 2012 | BACKGROUND: Concordance and Shared Decision-Making (SDM) are considered measures of the quality of care that improves communication, promotes patient participation, creates a positive relationship with the healthcare professional, and results in greater adherence with the treatment plan. METHODS: This study compares the attitudes of 225 mental health professionals (125 psychiatrists and 100 psychiatry registrars) and 449 psychiatric outpatients towards SDM and concordance in medicine taking by using the "Leeds Attitude toward Concordance Scale" (LATCon). RESULTS: The internal consistency of the scale was good in all three samples (Cronbach's α: patients = 0.82, psychiatrists = 0.76, and registrars = 0.82). Patients scored significantly lower (1.96 ± 0.48) than professionals (P < .001 in both cases), while no statistically significant differences between psychiatrists (2.32 ± 0.32) and registrars (2.23 ± 0.35) were registered; the three groups showed a positive attitude towards concordance in most indicators. Patients are clearly in favor of being informed and that their views and preferences be taken into account during the decision-making process, although they widely consider that the final decision must be the doctor's responsibility. Among mental health professionals, the broader experience provides a greater conviction of the importance of the patient's decision about treatment. CONCLUSIONS: We observed a positive attitude towards concordance in the field of psychotropic drugs prescription both in professionals and among patients, but further studies are needed to address the extent to which this apparently accepted model is reflected in the daily practice of mental health professionals. | 0 | 1 |  |  |  |  |  |
| Comparing the nine-item Shared Decision-Making Questionnaire to the OPTION Scale - an attempt to establish convergent validity | Scholl | Health Expectations: An International Journal of Public Participation in Health Care and Health Policy | 2012-11-26 Nov 26, 2012 | BACKGROUND: While there has been a clear move towards shared decision-making (SDM) in the last few years, the measurement of SDM-related constructs remains challenging. There has been a call for further psychometric testing of known scales, especially regarding validity aspects. OBJECTIVE: To test convergent validity of the nine-item Shared Decision-Making Questionnaire (SDM-Q-9) by comparing it to the OPTION Scale. DESIGN: Cross-sectional study. SETTING AND PARTICIPANTS: Data were collected in outpatient care practices. Patients suffering from chronic diseases and facing a medical decision were included in the study. METHODS: Consultations were evaluated using the OPTION Scale. Patients completed the SDM-Q-9 after the consultation. First, the internal consistency of both scales and the inter-rater reliability of the OPTION Scale were calculated. To analyse the convergent validity of the SDM-Q-9, correlation between the patient (SDM-Q-9) and expert ratings (OPTION Scale) was calculated. RESULTS: A total of 21 physicians provided analysable data of consultations with 63 patients. Analyses revealed good internal consistency of the SDM-Q-9 and limited internal consistency of the OPTION Scale. Inter-rater reliability of the latter was less than optimal. Association between the total scores of both instruments was weak with a Spearman correlation of r = 0.19 and did not reach statistical significance. DISCUSSION: By the use of the OPTION Scale convergent validity of the SDM-Q-9 could not be established. Several possible explanations for this result are discussed. CONCLUSION: This study shows that the measurement of SDM remains challenging. | 0 | 1 |  |  |  |  |  |
| Completing the third person's perspective on patients' involvement in medical decision-making: approaching the full picture | Kasper | Zeitschrift Für Evidenz, Fortbildung Und Qualität Im Gesundheitswesen | 2012-00-00 2012 | OBJECTIVE: Shared decision making is based on the idea of cooperation and partnership between patients and doctors. In this concept both parties may initiate and perform specific decision-making steps. However, the common observation-based instruments focus solely on doctors' behaviour. Content and quality of information provided to involve patients in medical decisions are hardly considered in evaluation of SDM. This study investigates the advantages of a revised observer inventory taking into account these aspects. METHODS: Based on the OPTION scale, a more comprehensive observation-based inventory was developed, additionally considering both the patient-sided indicators for patient involvement and the criteria of evidence-based patient information. The inventory comprises three scales (doctor, patient, doctor-patient dyad) and 15 indicators each. Rater training and re-analyses of 76 consultations previously analysed using the OPTION scale were conducted. Convergent validities were calculated between the observer-based scales and the patients' ratings on the Shared Decision Making Questionnaire, the Decisional Conflict Scale and the Control Preference Scale. RESULTS: Interrater reliabilities of the revised scales were high (r=.87 to .74) and even higher when only the dyadic perspective was coded (.86). The revised inventory provided additional information on the involvement taking place. No substantive correlations were found between observation-based and patients' subjective judgments. CONCLUSION: The observers' perspective on patient involvement needs to consider patient activities. Inconsistencies of patients' and observers' judgements concerning patient participation need further investigation. | 1 |  |  |  |  |  |  |
| Decision dissonance: evaluating an approach to measuring the quality of surgical decision making | Fowler | Joint Commission Journal on Quality and Patient Safety / Joint Commission Resources | 2013-03-00 Mar 2013 | BACKGROUND: Good decision making has been increasingly cited as a core component of good medical care, and shared decision making is one means of achieving high decision quality. If it is to be a standard, good measures and protocols are needed for assessing the quality of decisions. Consistency with patient goals and concerns is one defining characteristic of a good decision. A new method for evaluating decision quality for major surgical decisions was examined, and a methodology for collecting the needed data was developed. METHODS: For a national probability sample of fee-for-service Medicare beneficiaries who had a coronary artery bypass graft (CABG), a lumpectomy or a mastectomy for breast cancer, or surgery for prostate cancer during the last half of 2008, a mail-survey of selected patients was carried out about one year after the procedures. Patients' goals and concerns, knowledge, key aspects of interactions with clinicians, and feelings about the decisions were assessed. A decision dissonance score was created that measured the extent to which patient ratings of goals ran counter to the treatment received. The construct and predictive validity of the decision dissonance score was then assessed. RESULTS: When data were averaged across all four procedures, patients with more knowledge and those who reported more involvement reported significantly lower Decision Dissonance Scores. Patients with lower Decision Dissonance Scores also reported more confidence in their decisions and feeling more positively about how the treatment turned out, and they were more likely to say that they would make the same decision again. CONCLUSIONS: Surveying discharged surgery patients is a feasible way to evaluate decision making, and Decision Dissonance appears to be a promising approach to validly measuring decision quality. | 0 |  |  |  |  |  | 1 |
| Decision-making preferences among patients with an acute myocardial infarction | Krumholz | JAMA internal medicine | 2013-07-08 Jul 8, 2013 | | 0 |  | 1 |  |  |  |  |
| Determining the validity and reliability of clinical communication assessment tools for dental patients and students | Schönwetter | Journal of Dental Education | 2012-10-00 Oct 2012 | A shortcoming identified in the dental education literature is the scarcity of patient assessment of the quality of communication between student clinicians and patients. This study, the second in a series, attempts to address this scarcity by testing the communication components deemed critical to patients identified in the first article. Two instruments were tested: the Patient Communication Assessment Instrument (PCAI) and the Student Communication Assessment Instrument (SCAI). Item-to-total correlations and Cronbach's alpha were used to determine internal consistency reliability. Construct validity was examined through principal components factor analysis with varimax rotation using a total of 820 participants (410 patients and 410 students), who completed communication skills questionnaires collected in the 2006-07 school year as part of dental and dental hygiene clinical courses. Each component in the assessment instruments demonstrated internal consistency (alpha range=0.779-0.960). Based on a principal components analysis, six new factors were found to be significantly associated with communication skills: being caring and respectful, sharing information, interacting with team members, tending to comfort, professional relationship-building, and appointment preparation/follow-up. Correlational analysis demonstrated a core of critical instrument items to be considered for future assessment of the quality of communication between student clinicians and patients. Adequate estimates of reliability and validity for the PCAI and SCAI were demonstrated. Further research is needed in other countries and cultures to test and confirm the constructs. | 0 |  |  |  |  |  | 1 |
| Developing a dyadic OPTION scale to measure perceptions of shared decision making | Melbourne | Patient Education and Counseling | 2010-02-00 Feb 2010 | OBJECTIVE: Our aim was to develop a measurement which enables research into the interdependent nature of clinical encounters. The prime objective was to develop an instrument capable of assessing the extent to which patients have been involved in (shared) decision making from two viewpoints-that of the patient and the clinician. METHODS: To develop an initial 'dyadic OPTION' instrument, the twelve original third-person items were drafted in passive, first person plural forms. Using this version initially, three rounds of cognitive debriefing interviews were held. These were audio-recorded and analysed at the end of each round and the results used to revise the dyadic OPTION scale. RESULTS: It was possible to modify the observer OPTION instrument into an instrument for completion by both clinicians and patients after a dyadic interaction. Cognitive debriefing revealed five areas of interpretative difficulty. Each item of the observer OPTION scale underwent modification in order to develop a dyadic version of the scale. CONCLUSIONS: The dyadic OPTION scale is acceptable and comprehensible by both clinicians and public respondents. Cognitive debriefing adapted and refined an existing scale and provided confidence that the core constructs of the scale (perceived involvement in decisions making) were understood. PRACTICE IMPLICATIONS: Further validation of the dyadic OPTION scale is required prior to its use in research settings. | 0 | 1 |  |  |  |  |  |
| Developing CollaboRATE: a fast and frugal patient-reported measure of shared decision making in clinical encounters | Elwyn | Patient Education and Counseling | 2013-10-00 Oct 2013 | OBJECTIVE: Measuring the process of shared decision making is a challenge, which constitutes a barrier to research and implementation. The aim of the study was to report the development of CollaboRATE, brief patient-reported measure of shared decision making. METHODS: We used the following stages: (1) item formulation; (2) cognitive interviews; (3) item refinement; and (4) pilot testing of final items. Participants were over 18 years old, recruited from the public areas of the Dartmouth-Hitchcock Medical Center. RESULTS: The key finding of this study is that developing a brief patient-reported measure of shared decision making requires a move away from terms such as 'decisions', 'options' and 'preferences'. Although technically correct, these terms act as barriers. They are often unfamiliar, and they also implicitly assume that patients are willing to take active roles in decision making; whereas patients are often unaware that decisions are required, or have taken place, never mind feel that they could or should have participated in them. CONCLUSION: These methods have allowed us to develop a brief, patient-reported measure of shared decision making that is highly accessible to intended users. PRACTICE IMPLICATIONS: The potential strength of the CollaboRATE will be the ability for completion in less than 30s, and across a range of routine settings. | 0 | 1 |  |  |  |  |  |
| Developing new dental communication skills assessment tools by including patients and other stakeholders | Wener | Journal of Dental Education | 2011-12-00 Dec 2011 | Effectively using patients as teachers to provide authentic feedback is an underused strategy in dental education, but it has potential for integrating the teaching of therapeutic communication skills within the dental clinic setting. This study focuses on the absence of patient input into the design of instruments used to assess students' clinical communication skills and demonstrates how a holistic approach, with input from key stakeholders including patients, was used to produce two such instruments. The development of complementary communication assessment instruments, one for patient use and one for student use, took place in three phases. In Phase I the authors reviewed a sample of existing patient satisfaction surveys; in Phase II they captured input from stakeholders; and Phase III resulted in the generation of the patient communication assessment instrument and the student communication self-assessment instrument. This article highlights communication skill issues relevant to the education of oral health professionals and describes the rationale and process for the development of the first iteration of the patient assessment and student self-assessment clinical communication instruments. | 0 | 1 |  |  |  |  |  |
| Development and evaluation of shared medical decision-making scale for end-of-life patients in Korea] | Jo | Journal of Korean Academy of Nursing | 2012-08-00 Aug 2012 | PURPOSE: The study was done to develop a shared decision-making scale for end-of-life patients in Korea. METHODS: The process included construction of a conceptual framework, generation of initial items, verification of content validity, selection of secondary items, preliminary study, and extraction of final items. The participants were 388 adults who lived in one of 3 Korean metropolitan cities: Seoul, Daegu, or Busan. Item analysis, factor analysis, criterion related validity, and internal consistency were used to analyze the data. Data collection was done from July to October 2011. RESULTS: Thirty-four items were selected for the final scale, and categorized into 7 factors explaining 61.9% of the total variance. The factors were labeled as sharing information (9 items), constructing system (7 items), explanation as a duty (5 items), autonomy (4 items), capturing time (3 items), participation of family (3 items), and human respect (3 items). The scores for the scale were significantly correlated among shared decision-making scale, terminating life support scale, and dignified dying scale. Cronbach's alpha coefficient for the 34 items was .94. CONCLUSION: The above findings indicate that the shared decision-making scale has a good validity and reliability when used for end-of-life patients in Korea. | 0 |  |  | 1 |  |  |  |
| Development and psychometric properties of a five-language multiperspective instrument to assess clinical decision making style in the treatment of people with severe mental illness (CDMS) | Puschner | BMC psychiatry | 2013-00-00 2013 | BACKGROUND: The aim of this study was to develop and evaluate psychometric properties of the Clinical Decision Making Style (CDMS) scale which measures general preferences for decision making as well as preferences regarding the provision of information to the patient from the perspectives of people with severe mental illness and staff. METHODS: A participatory approach was chosen for instrument development which followed 10 sequential steps proposed in a current guideline of good practice for the translation and cultural adaptation of measures. Following item analysis, reliability, validity, and long-term stability of the CDMS were examined using Spearman correlations in a sample of 588 people with severe mental illness and 213 mental health professionals in 6 European countries (Germany, UK, Italy, Denmark, Hungary, and Switzerland). RESULTS: In both patient and staff versions, the two CDMS subscales "Participation in Decision Making" and "Information" reliably measure distinct characteristics of decision making. Validity could be demonstrated to some extent, but needs further investigation. CONCLUSIONS: Together with two other five-language patient- and staff-rated measures developed in the CEDAR study (ISRCTN75841675) - "Clinical Decision Making in Routine Care" and "Clinical Decision Making Involvement and Satisfaction" - the CDMS allows empirical investigation of the complex relation between clinical decision making and outcome in the treatment of people with severe mental illness across Europe. | 0 |  |  |  |  |  | 1 |
| Development and psychometric properties of the Shared Decision Making Questionnaire--physician version (SDM-Q-Doc) | Scholl | Patient Education and Counseling | 2012-08-00 Aug 2012 | OBJECTIVE: To develop and psychometrically test a brief instrument for assessing the physician's perspective of the shared decision-making process in clinical encounters. METHODS: We adapted the 9-item Shared Decision Making Questionnaire (SDM-Q-9) for patients to generate a new version for physicians (SDM-Q-Doc). The physician version was tested in clinical encounters between 29 physicians and 324 patients in German outpatient care contexts. Analyses of the extent to which the instrument was accepted, the reliability of the instrument, and the factorial structure of the scale were performed. RESULTS: Physicians showed a high level of acceptance toward the SDM-Q-Doc. Item discrimination parameters were above .4 for all but one item. An analysis of internal consistency yielded a Cronbach's α of .88. Factor analysis confirmed a one-dimensional structure. CONCLUSION: The results of this study suggest that the SDM-Q-Doc is a well-accepted and reliable instrument for assessing the physician's perspective during SDM processes in clinical encounters. To our knowledge, the SDM-Q-Doc is the first psychometrically tested scale available for assessing the physician's perspective. PRACTICE IMPLICATIONS: The SDM-Q-Doc can be used in studies that analyze the effectiveness of the implementation of SDM and as a quality indicator in quality assurance programs and health service assessments. | 1 |  |  |  |  |  |  |
| Development and validation of a neonatal intensive care parent satisfaction instrument | Latour | Pediatric Critical Care Medicine: A Journal of the Society of Critical Care Medicine and the World Federation of Pediatric Intensive and Critical Care Societies | 2012-09-00 Sep 2012 | OBJECTIVE: To develop and test the psychometric properties of the EMPATHIC-N (EMpowerment of PArents in THe Intensive Care-Neonatology) questionnaire measuring parent satisfaction. DESIGN: A psychometric study testing the reliability and validity of a parent satisfaction questionnaire by applying confirmatory factor analysis including standardized factor loadings and subsequently Cronbach's α reliability estimates across time, congruent validity, and nondifferential validity testing. SETTING: A 30-bed neonatal intensive care unit in a university hospital. PATIENTS: Two cohorts with a total of 441 parents whose child was admitted to the neonatal intensive care unit, January to December 2009. INTERVENTIONS: None. MEASUREMENTS AND MAIN RESULTS: In the first cohort, 220 of 339 (65%) parents responded; in the second cohort, 59 of 102 (58%) parents responded. Structural equation modeling and confirmatory factor analysis resulted in a sufficient model fit of 57 statements within five domains: Information, Care & Treatment, Organization, Parental Participation, and Professional Attitude. Standardized factor loading of these statements were between 0.58 and 0.91. Reliability measures, Cronbach's α, of the domains ranged from 0.82 to 0.95. Reliability across time showed no evidence of statistically significant differences between the domains. Congruent validity was confirmed by a good correlation (p = .01) between the domains and four general satisfaction questions. Nondifferential validity showed no significant effect sizes between the infants' characteristics and the domains, except between ventilated infants and parent participation statements and infants ≥30 wks gestational age and organizational statements. CONCLUSIONS: The EMPATHIC-N questionnaire is a valid quality performance indicator to measure the delivered care as perceived by parents. | 0 |  |  |  | 1 |  |  |
| Development and validation of the Japanese version of the Decisional Conflict Scale to investigate the value of pharmacists' information: a before and after study | Kawaguchi | BMC medical informatics and decision making | 2013-00-00 2013 | BACKGROUND: The information provided in patient-centered care and shared decision-making influences patients' concerns and adherence to treatment. In the decision-making process, patients experience decisional conflict. The Decisional Conflict Scale (DCS) is a 16-item, self-administered questionnaire consisting of 5 subscales developed to assess patients' decisional conflict. This study aimed to develop the Japanese version of the DCS and to clarify the influence of the information provided by pharmacists' on decisional conflict among patients with cancer. METHODS: We developed the Japanese version of the DCS by using the forward-backward translation method. One hundred patients who were recommended a new chemotherapy regimen were recruited. The psychometric properties of the Japanese DCS, including internal consistency, convergent validity, discriminant validity, and construct validity, were examined. We assessed the decisional conflict of patients before and after the pharmacists' provision of information. RESULTS: Ninety-four patients, predominately female, with an average age of 58.1 years were sampled. The scores on the 5 subscales of the DCS showed high internal consistency (Cronbach's alpha = 0.84-0.96). Multi-trait scaling analysis and cluster analysis showed strong validity. The mean total DCS score decreased significantly from 40.2 to 31.7 after patients received information from the pharmacists (p < 0.001, paired t-test). Scores on all 5 subscales, namely, uncertainty, informed, values clarity, support, and effective decision, also significantly improved (p < 0.001 for all categories, paired t-test). CONCLUSIONS: The psychometric properties of the Japanese version of the DCS are considered appropriate for it to be administered to patients with cancer. Pharmacists' provision of information was able to decrease decisional conflict among patients with cancer who were recommended a new chemotherapy regimen. | 1 |  |  |  |  |  |  |
| Development of a Quantitative Measure of Holistic Nursing Care | Kinchen | Journal of Holistic Nursing: Official Journal of the American Holistic Nurses' Association | 2014-12-23 Dec 23, 2014 | Holistic care has long been a defining attribute of nursing practice. From the earliest years of its formal history, nursing has favored a holistic approach in the care of patients, and such an approach has become more important over time. The expansion of nursing's responsibility in delivering comprehensive primary care, the recognition of the importance of relationship-centered care, and the need for evidence-based legitimation of holistic nursing care and practices to insurance companies, policy-makers, health care providers, and patients highlight the need to examine the holistic properties of nursing care. The Holistic Caring Inventory is a theoretically sound, valid, and reliable tool; however, it does not comprehensively address attributes that have come to define holistic nursing care, necessitating the development of a more current instrument to measure the elements of a holistic perspective in nursing care. The development of a current and more comprehensive measure of holistic nursing care may be critical in demonstrating the importance of a holistic approach to patient care that reflects the principles of relationship-based care, shared decision-making, authentic presence, and pattern recognition. | 0 | 1 |  |  |  |  |  |
| Development of a questionnaire to assess communication preferences of patients with chronic illness | Farin | Patient Education and Counseling | 2011-01-00 Jan 2011 | OBJECTIVE: the objectives of the study are to develop a patient-oriented and theory-based questionnaire on the communication preferences of chronically ill patients (KOPRA questionnaire) and to carry out psychometric testing of the instrument. METHODS: following two preliminary studies (focus groups, cognitive interviews), a total of 472 patients with chronic back pain or chronic ischemic heart disease were surveyed. In the main sample (N=333), communication preferences regarding the physician were assessed; for N=89 (or N=50) patients, preferences regarding nursing staff (or therapists) were analyzed. Psychometric testing was done with respect to unidimensionality, fit to an item response theory (IRT) model, and for reliability. The questionnaire was developed and validated in German. RESULTS: In the physician version with a total of 32 items, there are four scales ("Patient participation and patient orientation", "Effective and open communication", "Emotionally supportive communication", and "Communication about personal circumstances") that are unidimensional, fulfill the demands for a 1-parameter IRT model, and are reliable (Cronbach's alpha between .80 and .92). The psychometric properties with respect to nursing staff and therapists are slightly worse. CONCLUSION: the KOPRA questionnaire has good psychometric properties. PRACTICE IMPLICATIONS: clinical use of the questionnaire appears useful to determine patients' communication preferences. | 0 |  |  |  | 1 |  |  |
| Development of a shared decision making coding system for analysis of patient-healthcare provider encounters | Clayman | Patient Education and Counseling | 2012-09-00 Sep 2012 | OBJECTIVES: To describe the development and refinement of a scheme, detail of essential elements and participants in shared decision making (DEEP-SDM), for coding shared decision making (SDM) while reporting on the characteristics of decisions in a sample of patients with metastatic breast cancer. METHODS: The evidence-based patient choice instrument was modified to reflect Makoul and Clayman's integrative model of SDM. Coding was conducted on video recordings of 20 women at the first visit with their medical oncologists after suspicion of disease progression. Noldus Observer XT v.8, a video coding software platform, was used for coding. RESULTS: The sample contained 80 decisions (range: 1-11), divided into 150 decision making segments. Most decisions were physician-led, although patients and physicians initiated similar numbers of decision-making conversations. CONCLUSION: DEEP-SDM facilitates content analysis of encounters between women with metastatic breast cancer and their medical oncologists. Despite the fractured nature of decision making, it is possible to identify decision points and to code each of the essential elements of shared decision making. Further work should include application of DEEP-SDM to non-cancer encounters. PRACTICE IMPLICATIONS: A better understanding of how decisions unfold in the medical encounter can help inform the relationship of SDM to patient-reported outcomes. | 0 | 1 |  |  |  |  |  |
| Development of and field test results for the CAHPS PCMH Survey | Scholle | Medical Care | 2012-11-00 Nov 2012 | OBJECTIVE: To develop and evaluate survey questions that assess processes of care relevant to Patient-Centered Medical Homes (PCMHs). RESEARCH DESIGN: We convened expert panels, reviewed evidence on effective care practices and existing surveys, elicited broad public input, and conducted cognitive interviews and a field test to develop items relevant to PCMHs that could be added to the Consumer Assessment of Healthcare Providers and Systems (CAHPS®) Clinician & Group (CG-CAHPS) 1.0 Survey. Surveys were tested using a 2-contact mail protocol in 10 adults and 33 pediatric practices (both private and community health centers) in Massachusetts. A total of 4875 completed surveys were received (overall response rate of 25%). ANALYSES: We calculated the rate of valid responses for each item. We conducted exploratory factor analyses and estimated item-to-total correlations, individual and site-level reliability, and correlations among proposed multi-item composites. RESULTS: Ten items in 4 new domains (Comprehensiveness, Information, Self-Management Support, and Shared Decision-Making) and 4 items in 2 existing domains (Access and Coordination of Care) were selected to be supplemental items to be used in conjunction with the adult CG-CAHPS 1.0 Survey. For the child version, 4 items in each of 2 new domains (Information and Self-Management Support) and 5 items in existing domains (Access, Comprehensiveness-Prevention, Coordination of Care) were selected. CONCLUSIONS: This study provides support for the reliability and validity of new items to supplement the CG-CAHPS 1.0 Survey to assess aspects of primary care that are important attributes of PCMHs. | 0 | 1 |  |  |  |  |  |
| Development of instruments to measure the quality of breast cancer treatment decisions | Lee | Health Expectations: An International Journal of Public Participation in Health Care and Health Policy | 2010-09-00 Sep 2010 | BACKGROUND: Women with early-stage breast cancer face a multitude of decisions. The quality of a decision can be measured by the extent to which the treatment reflects what is most important to an informed patient. Reliable and valid measures of patients' knowledge and their goals and concerns related to breast cancer treatments are needed to assess the decision quality. OBJECTIVE: To identify a set of key facts and goals relevant to each of three breast cancer treatment decisions (surgery, reconstruction and adjuvant chemotherapy and hormone therapy) and to evaluate the validity of the methods used to identify them. METHODS: Candidate facts and goals were chosen based on evidence review and qualitative studies with breast cancer patients and providers. Cross-sectional surveys of patients and providers were conducted for each decision. The accuracy, importance and completeness of the items were examined. RESULTS: Thirty-eight facts (11-14 per decision) and 27 goals (8-10 per decision) were identified. An average of 17 patients and 21 providers responded to each survey. The sets of facts were accurate and complete for all three decisions. The sets of goals and concerns were important for surgery and reconstruction, but not chemotherapy/hormone therapy. Patients and providers disagreed about the relative importance of several key facts and goals. CONCLUSIONS: Overall, breast cancer patients and providers found the sets of facts and goals accurate, important and complete for three treatment decisions. Because patients' and providers' perspectives are different, it is vital that instrument development should include items reflecting both views. | 0 | 1 |  |  |  |  |  |
| Development of PRIDe: a tool to assess physicians' preference of role in clinical decision making | Giguère | Patient Education and Counseling | 2012-08-00 Aug 2012 | OBJECTIVE: To develop and evaluate items for inclusion in PRIDe (Preferred Role in Decision Making), a new tool to assess changes of role preference among professionals exposed to training in shared decision making (SDM). METHODS: This study was part of a pilot trial to evaluate the effectiveness of SDM training on the doctors' prescription of antibiotics for acute respiratory infections. Thirty-nine family physicians were randomized to immediate exposure to training or to delayed exposure. Potential items for PRIDe and a questionnaire about physicians' intention to engage in SDM were administered at baseline and at follow-up. RESULTS: Following analysis, we retained five items that captured a change in physicians' preference. The items' scores were pooled and the resulting tool showed limited internal consistency (Cronbach's alpha = 0.41) but significant test-retest reliability (immediate group: P = 0.03; delayed group: P = 0.008) and acceptable discriminant validity, with patients involved in decision making more actively after training than before (Fisher's test, P = .02). CONCLUSION: This initial step to develop an evaluation tool to assess changes in doctors' preference of role in decision making following SDM training shows promising results. The next step is to develop more clinical vignettes followed by questions inspired from this analysis. PRACTICE IMPLICATIONS: The PRIDe instrument can be used in the assessment of health professionals' attitude towards shared decision making after training in shared decision making. Additional research is needed to evaluate its validity before it can be recommended for use. | 0 |  |  |  |  |  | 1 |
| Development of the patient approach and views toward healthcare communication (PAV-COM) measure among older adults | Tarn | BMC health services research | 2012-00-00 2012 | BACKGROUND: This study examines the psychometric properties of 9 items on the Patient Activation component of the Medicare Current Beneficiary Survey (MCBS) that assess how patients approach and communicate with their physicians. The MCBS is a nationally representative, cross-sectional survey of Medicare beneficiaries. METHODS: We analyzed MCBS data collected in 2002 and 2005 from 15,165 adults aged 65 and older. Exploratory factor analysis was conducted using maximum likelihood to estimate a polychoric correlation matrix on the 2002 data, and confirmatory factor analysis was performed using the 2005 data. RESULTS: Exploratory factor analysis of the 2002 data showed a 2-factor solution: approach to interactions (5 items) and views about physician's healthcare communication (6 items). Findings were confirmed using the 2005 data. Items were combined to form the Patient Approach and Views toward Healthcare Communication (PAV-COM) scale (range 1 to 100; Cronbach's alpha of 0.75, and item-rest correlations between 0.33 and 0.54). Higher PAV-COM scores were associated with greater fulfillment of preventive health behaviors such as vaccinations and cancer screenings. CONCLUSIONS: The PAV-COM measure is a valid tool for assessing patient approaches and views toward communication with physicians. This measure can be used to evaluate interventions to improve patient participation during healthcare encounters. | 0 |  |  |  | 1 |  |  |
| Diabetes Empowerment Process Scale: development and psychometric testing of the Chinese version | Chen | Journal of Advanced Nursing | 2011-01-00 Jan 2011 | AIM: This paper is a report of the development and psychometric testing of the Chinese version of the Diabetes Empowerment Process Scale. BACKGROUND: In people living with diabetes, empowerment can improve metabolic and psychosocial outcomes. A scale for measuring empowerment processes can also help healthcare professionals to optimize their empowering actions and would improve their interactions with people living with diabetes. METHOD: Based on literature reviews and interviews with people living with diabetes, a 27-item Chinese Diabetes Empowerment Process Scale was developed. Fifteen items were produced after item analysis and content validity testing. To test construct validity, concurrent validity and internal consistency, 211 outpatients living with diabetes completed the Chinese Diabetes Empowerment Process Scale, a Self-care Behaviour Scale and a Diabetes Empowerment Scale. Test-retest reliability was also analysed with 30 patients. The study was conducted in 2008 and 2009 in Taiwan. RESULTS: Confirmatory factor analysis indicated that a second-order factorial model with four subscales and 15 items best fitted the data. The four subscales were Mutual participation, Raising awareness, Providing necessary information and Open communication. Scores on the Chinese Diabetes Empowerment Process Scale correlated statistically significantly with those on the Self-care Behaviour Scale (r=0.21-0.40; P<0.01) and Diabetes Empowerment Scale (r=0.35-0.65; P<0.01). Reliability was supported by acceptable Cronbach's α (range: 0.73-0.91) and test-retest reliability scores (range: 0.75-0.83). CONCLUSION: The Chinese Diabetes Empowerment Process Scale has satisfactory validity and reliability for measuring the empowerment process of health professionals. Further studies are needed to test the applicability of the scale to other populations. | 0 | 1 |  |  |  |  |  |
| Dyadic OPTION: Measuring perceptions of shared decision-making in practice | Melbourne | Patient Education and Counseling | 2011-04-00 Apr 2011 | BACKGROUND: Current models of the medical consultation emphasize shared decision-making (SDM), whereby the expertise of both the doctor and the patient are recognised and seen to equally contribute to the consultation. The evidence regarding the desirability and effectiveness of the SDM approach is often conflicting. It is proposed that the conflicts are due to the nature of assessment, with current assessments from the perspective of an outside observer. AIMS: To empirically assess perceived involvement in the medical consultation using the dyadic OPTION instrument. METHOD: 36 simulated medical consultations were organised between general practitioners and standardized- patients, using the observer OPTION and the newly developed dyadic OPTION instruments. RESULTS: SDM behaviours observed in the consultations were seen to depend on both members of the doctor and patient dyad, rather than each in isolation. Thus a dyadic approach to measurement is supported. CONCLUSIONS: This current study highlights the necessity for a dyadic approach to assessment and introduces a novel research instrument: the dyadic OPTION instrument. | 0 | 1 |  |  |  |  |  |
| Evaluating the psychometric properties of the CAHPS Patient-centered Medical Home survey | Hays | Clinical Therapeutics | 2014-05-00 May 2014 | OBJECTIVE: The goal of this study was to evaluate the reliability and validity of the Consumer Assessment of Healthcare Providers and Systems (CAHPS) Patient-Centered Medical Home (PCMH) survey. METHODS: We conducted a field test of the CAHPS PCMH survey with 2740 adults. We collected information by mail (n = 1746), telephone (n = 672), and from the Web (n = 322) from 6 sites of care affiliated with a West Coast staff model health maintenance organization. RESULTS: An overall response rate of 37% was obtained. Internal consistency reliability estimates for 7 multi-item scales were as follows: access to care, 5 items, α = 0.79; communication with providers, 6 items, α = 0.93; office staff courtesy and respect, 2 items, α = 0.80; shared decision making about medicines, 3 items, α = 0.67; self-management support, 2 items, α = 0.61; attention to mental health issues, 3 items, α = 0.80; and care coordination, 4 items, α = 0.58. The number of responses needed to get reliable information at the site of care level for the composites was generally acceptable (<300 for 0.70 reliability-level) except for self-management support and shared decision making about medicines. Item-scale correlations provided support for distinct composites except for access to care and shared decision making about medicines, which overlapped with the communication with providers scale. Shared decision making and self-management support were significantly, uniquely associated with the global rating of the provider (dependent variable), along with access and communication in a multiple regression model. CONCLUSIONS: This study provides further support for the reliability and validity of the CAHPS PCMH survey, but refinement of the self-management support and shared decision-making scales is needed. The survey can be used to provide information about the performance of different health plans on multiple domains of health care, but future efforts to improve some of the survey items is needed. | 0 | 1 |  |  |  |  |  |
| How does your doctor talk with you? Preliminary validation of a brief patient self-report questionnaire on the quality of physician-patient interaction | Bieber | Journal of Clinical Psychology in Medical Settings | 2010-06-00 Jun 2010 | The quality of physician-patient interaction is increasingly being recognized as an essential component of effective treatment. The present article reports on the development and validation of a brief patient self-report questionnaire (QQPPI) that assesses the quality of physician-patient interactions. Data were gathered from 147 patients and 19 physicians immediately after consultations in a tertiary care outpatient setting. The QQPPI displayed good psychometric properties, with high internal consistency and good item characteristics. The QQPPI total score showed variability between different physicians and was independent of patients' gender, age, and education. The QQPPI featured high correlations with other quality-related measures and was not influenced by social desirability, or patients' clinical characteristics. The QQPPI is a brief patient self-report questionnaire that allows assessment of the quality of physician-patient interactions during routine ambulatory care. It can also be used to evaluate physician communication training programs or for educational purposes. | 0 |  |  |  | 1 |  |  |
| Information-seeking and decision-making preferences among adult orthodontic patients: an elective health care model | Miller | Community Dentistry and Oral Epidemiology | 2011-02-00 Feb 2011 | OBJECTIVES: When it comes to their own health care, adult patients traditionally demonstrate strong information-seeking desire but a somewhat lower desire to make their own treatment decisions in nonelective situations. Little is known about these desires in patients facing elective health care situations. We used the well-tested Autonomy Preferences Index (API) as a base to construct and test our elective Autonomy Preferences Index (eAPI) for both information-seeking and decision-making and analyzed demographic variables on both. METHODS: The eAPI was constructed to mirror the API but uses elective scenarios rather than the API's nonelective scenarios. It was validated using cognitive interviews to determine item intent and comprehension and by Cronbach's alpha. Both the API and eAPI were distributed to 188 active-treatment patients at the Division of Orthodontics, University of Minnesota. API and eAPI items were scored using a 1 (low) to 5 (high) Likert scale of desire. RESULTS: Mean information-seeking desire was universally high (>4, P < 0.001) for both API and eAPI instruments. Mean decision-making (DM) desire was universally low to moderate: API-DM = 2.84 and eAPI-DM = 2.6. Decision-making preferences for nonelective items (API-DM) decreased as the condition severity presented in the vignettes increased: mild = 2.88, moderate = 2.67 and severe = 2.21. Conversely, elective decision-making preferences (eAPI-DM) increased with increasing condition severity: mild = 2.51, moderate = 2.79 and severe = 3.18 (P < 0.001). CONCLUSIONS: Adult patients have universally high information-seeking preferences and moderate to low decision-making preferences regardless of the elective or nonelective nature of their condition. However, as vignette condition severity increases, patients facing nonelective scenarios display progressively less desire for decision-making, whereas patients facing elective scenarios show progressively more decision-making desire. | 0 | 1 |  |  |  |  |  |
| Interpersonal communication from the patient perspective: comparison of primary healthcare evaluation instruments | Beaulieu | Healthcare Policy = Politiques De Santé | 2011-12-00 Dec 2011 | The operational definition of interpersonal communication is "the ability of the provider to elicit and understand patient concerns, to explain healthcare issues and to engage in shared decision-making if desired." OBJECTIVE: To examine how well interpersonal communication is captured in validated instruments that evaluate primary healthcare from the patient's perspective. METHOD: 645 adults with at least one healthcare contact in the previous 12 months responded to instruments that evaluate primary healthcare. Eight subscales measure interpersonal communication: the Primary Care Assessment Survey (PCAS, two subscales); the Components of Primary Care Index (CPCI, one subscale); the first version of the EUROPEP (EUROPEP-I); and the Interpersonal Processes of Care Survey, version II (IPC-II, four subscales). Scores were normalized for descriptive comparison. Exploratory and confirmatory (structural equation) factor analysis examined fit to operational definition, and item response theory analysis examined item performance. RESULTS: Items not pertaining to interpersonal communication were removed from the EUROPEP-I. Most subscales are skewed positively. Normalized mean scores are similar across subscales except for IPC-II Patient-Centred Decision-Making and IPC-II Hurried Communication. All subscales load reasonably well on a single factor, presumed to be interpersonal communication. The best model has three underlying factors corresponding to eliciting (eigenvalue = 26.56), explaining (eigenvalue = 2.45) and decision-making (eigenvalue = 1.34). Both the PCAS Communication and the EUROPEP-I Clinical Behaviour subscales capture all three dimensions. Individual subscales within IPC-II measure each sub-dimension. CONCLUSION: The operational definition is well reflected in the available measures, although shared decision-making is poorly represented. These subscales can be used with confidence in the Canadian context to measure this crucial aspect of patient-centred care. | 0 | 1 |  |  |  |  |  |
| MAPPIN'SDM--The multifocal approach to sharing in shared decision making | Kasper | PLoS ONE | 2012-04-13 avril 13, 2012 | Background: The wide scale permeation of health care by the shared decision making concept (SDM) reflects its relevance and advanced stage of development. An increasing number of studies evaluating the efficacy of SDM use instruments based on various sub-constructs administered from different viewpoints. However, as the concept has never been captured in operable core definition it is quite difficult to link these parts of evidence. This study aims at investigating interrelations of SDM indicators administered from different perspectives. Method: A comprehensive inventory was developed mapping judgements from different perspectives (observer, doctor, patient) and constructs (behavior, perception) referring to three units (doctor, patient, doctor-patient-dyad) and an identical set of SDM-indicators. The inventory adopted the existing approaches, but added additional observer foci (patient and doctor-patient-dyad) and relevant indicators hitherto neglected by existing instruments. The complete inventory comprising a doctor-patient-questionnaire and an observer-instrument was applied to 40 decision consultations from 10 physicians from different medical fields. Convergent validities were calculated on the basis of Pearson correlation coefficients. Results: Reliabilities for all scales were high to excellent. No correlations were found between observer and patients or physicians neither for means nor for single items. Judgements of doctors and patients were moderately related. Correlations between the observer scales and within the subjective perspectives were high. Inter-perspective agreement was not related to SDM performance or patient activity. Conclusion: The study demonstrates the contribution to involvement made by each of the relevant perspectives and emphasizes the need for an inter-subjective approach regarding SDM measurement. (PsycINFO Database Record (c) 2014 APA, all rights reserved). (journal abstract) | 1 |  |  |  |  |  |  |
| Measurement of patient involvement - what do concepts of psychotherapy research contribute? | Strauss | Zeitschrift Für Evidenz, Fortbildung Und Qualität Im Gesundheitswesen | 2012-00-00 2012 | The article comments on Shared Decision Making (SDM) research from a different perspective, i.e. psychotherapy research. Psychotherapy research had to face similar challenges, both conceptual as well as methodological. Meanwhile, the generic model of psychotherapy has helped researchers to put single results into a perspective that also might be helpful to the SDM field. Since SDM research increasingly focuses upon process perspectives directed towards the doctor-patient dyad, we propose measures and constructs from interpersonal theory as useful tools to be introduced into SDM research. A selection of these measures and their potential within SDM research is described. | 0 |  |  |  |  | 1 |  |
| Measurement of shared decision making - a review of instruments | Scholl | Zeitschrift Für Evidenz, Fortbildung Und Qualität Im Gesundheitswesen | 2011-00-00 2011 | The last years have seen a clear move towards shared decision making (SDM) and increased patient involvement in many countries. However, as the field of SDM research is still relatively young, new instruments for the measurement of (shared) decision making (process, outcome and surrounding elements) are constantly being developed. Thus, the aims of this structured review were to give an update on current developments regarding the measurement in the field of SDM, as well as to give a short overview of published and unpublished instruments. We conducted an electronic literature search in PubMed and the Web of Science database, performed hand searches of relevant journals and contacted key authors in the field. We found eight scales that have been subjected to further psychometric testing, eleven new and psychometrically tested instruments and nine developments that are still in the publishing process. The results show that there is a trend towards measuring SDM processes from a dyadic approach (assessing both the patient's and the clinician's perspective). More and more scales have been developed and tested in languages other than English, which indicates the growing research efforts in various countries. While reliability of most scales is good, they differ in their extent of validation. Further psychometric testing is needed, as well as the development of a theoretical measurement framework in order to improve consistency of measured constructs across research groups. | 0 | 1 |  |  |  |  |  |
| Measuring children's decision-making involvement regarding chronic illness management | Miller | Journal of Pediatric Psychology | 2012-04-00 Apr 2012 | OBJECTIVES: To develop a measure of decision-making involvement in children and adolescents with cystic fibrosis, diabetes, and asthma. METHODS: Parent-child dyads completed the Decision-Making Involvement Scale (DMIS) and measures of locus of control and family communication. DMIS items were subjected to exploratory and confirmatory factor analysis (CFA). Temporal stability and construct validity were assessed. RESULTS: The parent form was reduced to 20 items representing five factors. CFA showed that the five factors were an acceptable fit to the parent- and child-report data. Internal consistency values ranged from 0.71 to 0.91. Temporal stability was supported by moderate-substantial intraclass correlation coefficients. DMIS subscales were associated with child age, child locus of control, and family communication. CONCLUSIONS: The DMIS can be used to inform our understanding of the transition to greater independence for illness management. Additional research is needed to examine outcomes of decision-making involvement, including treatment adherence and responsibility. | 0 | 1 |  |  |  |  |  |
| Measuring informed decision making about prostate cancer screening in primary care | Leader | Medical Decision Making: An International Journal of the Society for Medical Decision Making | 2012-04-00 2012 Mar-Apr | PURPOSE: To measure the extent of informed decision making (IDM) about prostate cancer screening in physician-patient encounters, describe the coding process, and assess the reliability of the IDM measure. METHODS: Audiorecoded encounters of 146 older adult men and their primary care physicians were obtained in a randomized controlled trial of mediated decision support related to prostate cancer screening. Each encounter was dual coded for the presence or absence of 9 elements that reflect several important dimensions of IDM, such as information sharing, patient empowerment, and engaging patients in preference clarification. An IDM-9 score (range = 0-9) was determined for each encounter by summing the number of elements that were coded as present. Estimates of coding reliability and internal consistency were calculated. RESULTS: Male patients tended to be white (59%), married (70%), and between the ages of 50 and 59 (70%). Physicians tended to be white (90%), male (74%), and have more than 10 years of practice experience (74%). IDM-9 scores ranged from 0 to 7.5 (mean [SD], 2.7 [2.1]). Reliability (0.90) and internal consistency (0.81) of the IDM-9 were both high. The IDM dimension observed most frequently was information sharing (74%), whereas the dimension least frequently observed was engagement in preference clarification (3.4%). CONCLUSIONS: In physician-patient encounters, the level of IDM concerning prostate cancer screening was low. The use of a dual-coding approach with audiorecorded encounters produced a measure of IDM that was reliable and internally consistent. | 1 |  |  |  |  |  |  |
| Measuring patient participation in surgical treatment decision-making from healthcare professionals' perspective | Heggland | Journal of Clinical Nursing | 2014-02-00 Feb 2014 | AIMS AND OBJECTIVES: To develop, empirical test, and validate an instrument measuring patient participation in surgical treatment decision-making from healthcare professionals' perspective. BACKGROUND: Since the advent of New Public Management in many Western countries, patient participation in healthcare decision-making has been considered to be a best practice. A common notion is that well-educated and well-informed public want to choose their own treatments and providers and want to ask questions about the quality of their health services. DESIGN: Survey. METHODS: A self-report-measuring instrument was designed and administered to 620 healthcare professionals. Items were developed, validated and tested by 451 nurses and physicians working in six surgical wards in a University Hospital in Norway. RESULTS: A 16-item scale with the following four dimensions was developed: information dissemination, formulation of options, integration of information and control. Factor analysis procedures and reliability testing were performed. A one-way, between-groups analysis of variance was conducted to compare doctors' and nurses' opinions on four dimensions of patient participation in surgical treatment decision-making. CONCLUSIONS: This article shows that patient participation in surgical treatment decision-making can be measured by a 16-item scale and four distinct dimensions. The analysis demonstrated a reasonable level of construct validity and reliability. Nurses and physicians have a positive attitude towards patient participation overall, but the two groups differ in the extent to which they accept the idea of patient participation in treatment decision-making. RELEVANCE TO CLINICAL PRACTICE: The instrument can be a tool for managers and healthcare professionals in the implementation of patient participation in clinical practice. Data from the instrument can be useful to identify health services being provided and what areas that could strengthen patient participation. | 0 |  |  |  |  |  | 1 |
| Measuring shared decision making: a review of constructs, measures, and opportunities for cardiovascular care | Sepucha | Circulation. Cardiovascular Quality and Outcomes | 2014-07-00 Jul 2014 | | 0 |  |  |  |  | 1 |  |
| Measuring the participation of elderly patients in the discharge process from hospital: a critical review of existing instruments | Foss | Scandinavian Journal of Caring Sciences | 2010-12-00 Dec 2010 | Measuring patients' experiences has been a major task for health care organisations during the past decade. The discharge process is identified as a vulnerable component of health care in need of assessment, especially when it concerns elderly persons. There are no published reviews or systematic assessment of the existing instruments developed to capture patients' perspective on the discharge process. This study gives a review of existing survey instruments designed to assess patients' perspectives on the discharge process. We used systematic searches for potentially relevant instruments in MEDLINE, PubMed, CINAHL, and the Cochrane Database of Systematic Reviews for English language studies published between 1998 and 2009 was considered to evaluate the patients' perspective on the discharge process. Ten studies were included and assessed according to the established criteria, and the studies presented a total of 47 items related to participation. The review identified only one instrument designed specifically to capture participation in the discharge process. The main focus is on the information flow from the professional to the patient and never vice versa. Few of the instruments studied/analysed to what degree the patients were invited to share their knowledge, and none of the instruments inquired whether, in the patients' experiences, their perspective was taken into account. The major finding of the review is that none of the existing instruments capture the full range of participation, nor do they cover those areas of the discharge process identified by elderly patients themselves as the most essential. | 0 |  |  |  |  | 1 |  |
| Of blind men and elephants: suggesting SDM-MASS as a compound measure for shared decision making integrating patient, physician and observer views | Geiger | Zeitschrift Für Evidenz, Fortbildung Und Qualität Im Gesundheitswesen | 2012-00-00 2012 | OBJECTIVE: Shared decision making (SDM) between patient and physician is an interpersonal process. Most SDM measures use the view of one party (patient, physician or observer) as a proxy to capture this process although these views typically diverge. This study suggests the compound measure SDM(MASS) (SDM Meeting its concept's ASSumptions) integrating these three perspectives in one single index. METHODS: SDM(MASS) was derived theoretically and compared empirically to unilateral perspectives of patients, physicians and observers by application to a data set of 10 physicians (40 consultations) receiving an SDM training. RESULTS: The constituting parts of SDM(MASS) were highly reliable (Cronbach's alpha .94; interrater reliability .74-.87). Unilateral appraisal of training effects was divergent. SDM(MASS) revealed no effect. CONCLUSION: SDM(MASS) combines noteworthy information about SDM processes from different viewpoints and thereby delivers plausible assessments. It could overcome immanent shortcomings of unilateral approaches. However, it is a complex measure needing further validation. | 1 |  |  |  |  |  |  |
| Participation in the decision-making of the patient admitted to an intensive care unit] | Villanueva Ortiz | Medicina Clínica | 2010-03-20 Mar 20, 2010 | | 0 | 1 |  |  |  |  |  |
| Patient involvement in decision making in primary care clinics: development of a measuring tool] | Ruiz Moral | Atencion Primaria / Sociedad Española De Medicina De Familia Y Comunitaria | 2010-05-00 May 2010 | OBJECTIVES: This work aims to explore to what extent Spanish primary care providers involve patients in decisions and describe the development of a suggested tool for assessing patient involvement in these settings. DESIGN: Cross-sectional, development of a measurement tool. SETTING: Primary care clinics. PARTICIPANTS: Family doctors and residents. INTERVENTIONS: Based on a review of the literature and the opinions of primary care doctors, a selection was made of items from a previous scale used to measure general communication skills (CICAA-Patient Centred) and new specific items were added to this to measure involvement. MAIN MEASUREMENTS: The involvement of patients in decision-making was evaluated initially with this tool in 31 different clinical visits and the scale was then reformulated. A pool of 161 interviews was used to complete the process. Some psychometric properties (reliability and internal consistency) were estimated for the different samples and stages of the process. RESULTS: Some degree of patient involvement was found in just 31 visits. Despite this, only in 18 of these (58%) was there some involvement in a discussion about more than one treatment option. The Cohen's kappa values of the CICAA-Decision scale were between 0.48 and 0.94. Cronbach's alpha was 0.60/0.51. The global Intra-class correlation coefficient was 0.96. CONCLUSIONS: The levels of patient involvement were lower than expected. A simple question, such as that defined by one item in particular, and the CICAA-D scale, in general, could be useful to assess patient involvement in decision making in primary care. | 1 |  |  |  |  |  |  |
| Patient participation in clinical encounters: a systematic review to identify self-report measures | Mavis | Health Expectations: An International Journal of Public Participation in Health Care and Health Policy | 2014-03-11 Mar 11, 2014 | BACKGROUND: There is evidence suggesting that active participation of patients in their health care can improve the quality of care and decrease health-care costs. Further, patient reports of their health-care experience are increasingly used to monitor health-care quality. OBJECTIVE: This paper describes a systematic review of peer-reviewed studies to identify measures of patients' active participation in their encounters with health-care providers. METHODS: A systematic literature review was conducted for publications indexed from 1975 to 2011. Of interest were self-reported measures of patient participation that were not limited to a specific health concern. All abstracts were reviewed independently by two authors, and the full paper was considered for those meeting inclusion criteria. MAIN RESULTS: From a review of 4528 citations, ten measures were identified. The approaches to development of the measures varied considerably, as did their study samples and their psychometric quality. DISCUSSION: These measures represented three conceptual frameworks: empowerment and self-efficacy, therapeutic alliance, and consumerism/satisfaction. They provide a more comprehensive perspective of patients' experiences of their provider encounters, and a better understanding patient behaviour enhanced the quality of health-care delivery or improved health outcomes. These measures underscore the continuing challenge of defining patient participation and the multiple theoretical approaches that underlie this form of patient behaviour. CONCLUSIONS: Current interest in quality-related physician report cards gives significant weight to patients' self-reported experiences as one dimension of physician performance. It is critical to identify the specific focus and quality of measures selected for this and research purposes. | 0 | 1 |  |  |  |  |  |
| Patient Participation in Rehabilitation Questionnaire (PPRQ)-development and psychometric evaluation | Lindberg | Spinal Cord | 2013-11-00 Nov 2013 | STUDY DESIGN: A cross-sectional postal questionnaire study. OBJECTIVES: The aim of the study was to evaluate selected psychometric properties of a draft version of the Patient Participation in Rehabilitation Questionnaire (PPRQ) measuring patients' experiences of participation in care and rehabilitation. SETTING: Sweden. METHODS: On the basis of previous qualitative analyses of patient interview data, a 32-item questionnaire covering five domains of participation was developed and sent to 268 persons with spinal cord injury, aged 18-80 years and injured 1-12 years previously. In total, 141 (51%) evaluable questionnaires were returned. Multi-trait analysis was used to assess scaling assumptions by testing item convergent and discriminant validity and internal consistency reliability (Cronbach's α) associated with the hypothesized item-scale structure of the questionnaire. RESULT: Nine items failed to meet scaling assumptions and were omitted. Scaling assumptions were thereafter substantiated for the scales: 'respect and integrity' (6 items); 'planning and decision-making' (4 items); 'information and knowledge' (4 items); 'motivation and encouragement' (5 items); and 'involvement of family' (4 items). Item-scale correlations ranged from 0.67 to 0.85 and most items correlated higher or significantly higher with their hypothesized scale than with other scales. Cronbach's α was 0.89 for all scales. CONCLUSION: The PPRQ appears to adequately assess central aspects of participation in care and rehabilitation from the perspective of patients with spinal cord injury. Further studies using larger samples will be undertaken to confirm the scale structure as well as the sensitivity and responsiveness of the questionnaire. | 0 | 1 |  |  |  |  |  |
| Patient participation in surgical treatment decision making from the patients' perspective: validation of an instrument | Heggland | Nursing Research and Practice | 2012-00-00 2012 | The aim of this paper is to describe the development of a new, brief, easy-to-administer self-reported instrument designed to assess patient participation in decision making in surgical treatment. We describe item generation, psychometric testing, and validity of the instrument. The final scale consisted of four factors: information dissemination (5 items), formulation of options (4 items), integration of information (4 items), and control (3 items). The analysis demonstrated a reasonable level of construct validity and reliability. The instrument applies to patients in surgical wards and can be used to identify the health services that are being provided and the areas that could strengthen patient participation. | 0 |  |  |  |  |  | 1 |
| Patient participation in surgical treatment decision-making can be measured using a 16-item scale with four distinct dimensions | Doherty | Evidence-Based Nursing | 2015-01-00 Jan 2015 | | 0 |  |  |  |  |  | 1 |
| Patients' and observers' perceptions of involvement differ. Validation study on inter-relating measures for shared decision making | Kasper | PloS One | 2011-00-00 2011 | OBJECTIVE: Patient involvement into medical decisions as conceived in the shared decision making method (SDM) is essential in evidence based medicine. However, it is not conclusively evident how best to define, realize and evaluate involvement to enable patients making informed choices. We aimed at investigating the ability of four measures to indicate patient involvement. While use and reporting of these instruments might imply wide overlap regarding the addressed constructs this assumption seems questionable with respect to the diversity of the perspectives from which the assessments are administered. METHODS: The study investigated a nested cohort (N = 79) of a randomized trial evaluating a patient decision aid on immunotherapy for multiple sclerosis. Convergent validities were calculated between observer ratings of videotaped physician-patient consultations (OPTION) and patients' perceptions of the communication (Shared Decision Making Questionnaire, Control Preference Scale & Decisional Conflict Scale). RESULTS: OPTION reliability was high to excellent. Communication performance was low according to OPTION and high according to the three patient administered measures. No correlations were found between observer and patient judges, neither for means nor for single items. Patient report measures showed some moderate correlations. CONCLUSION: Existing SDM measures do not refer to a single construct. A gold standard is missing to decide whether any of these measures has the potential to indicate patient involvement. PRACTICE IMPLICATIONS: Pronounced heterogeneity of the underpinning constructs implies difficulties regarding the interpretation of existing evidence on the efficacy of SDM. Consideration of communication theory and basic definitions of SDM would recommend an inter-subjective focus of measurement. TRIAL REGISTRATION: Controlled-Trials.com ISRCTN25267500. | 0 | 1 |  |  |  |  |  |
| Psychiatric patients' attitudes towards concordance and shared decision making | De las Cuevas | Patient Education and Counseling | 2011-12-00 Dec 2011 | OBJECTIVES: To assess psychiatric outpatients' attitudes towards concordance and shared decision making in a psychiatric setting and to evaluate the role that self-perceived knowledge and beliefs about psychiatric medicines play in those attitudes. METHODS: The Leeds Attitude to Concordance Scale (LATCon) was tested on a sample of 435 psychiatric outpatients. Principal Component Analysis was used to assess the structure of LATCon items. Regression analysis on LATCon scores was performed with sociodemographics, Belief about Medicines Questionnaire (BMQ) subscales, self-perceived knowledge, perceived psychiatrist behaviour and current medications as predictor variables. RESULTS: The LATCon scale showed a good factorial validity, with a monofactorial structure and high internal consistency. Psychiatric outpatients tended to be in agreement with the concept of concordance, but they did not share some relevant aspects of the construct. Cognitive representations of psychiatric medications, assessed by the BMQ subscales, significantly predicted scores on the LATCon scale. CONCLUSION: Psychiatric outpatients show a considerable desire to participate in decision making about their treatment. The Spanish version of the LATCon Scale seems to be a valid instrument. PRACTICE IMPLICATIONS: Psychiatrists must consider their patients' desire to participate in treatment decisions and explore how patients' views about psychiatric medications influence their attitudes towards concordance. | 0 |  |  |  |  | 1 |  |
| Psychometric assessment of the patient activation measure short form (PAM-13) in rural settings | Hung | Quality of Life Research: An International Journal of Quality of Life Aspects of Treatment, Care and Rehabilitation | 2013-04-00 Apr 2013 | PURPOSE: The patient activation measure short form (PAM-13) assesses patients' self-reported health management skills, knowledge, confidence, and motivation. We used item response theory to evaluate the psychometric properties of the PAM-13 utilized in rural settings. METHODS: A Rasch partial credit model analysis was conducted on the PAM-13 instrument using a sample of 812 rural patients recruited by providers and our research staff. Specially, we examined dimensionality, item fit, and quality of measures, category response curves, and item differential functioning. Convergent and divergent validities were also examined. FINDINGS: The PAM-13 instrument has excellent convergent and divergent validities. It is fairly unidimensional, and all items fit the Rasch model well. It has relatively high person and item reliability indices. Majority of the items were free of item differential functioning. There were, however, some issues with ceiling effects. Additionally, there was a lack of responses for category one across all items. CONCLUSIONS: Patient activation measure short form (PAM-13) performs well in some areas, but not all. In general, more items need to be added to cover the upper end of the trait. The four response categories of PAM-13 should be collapsed into three. | 0 | 1 |  |  |  |  |  |
| Psychometric evaluation of the Shared Decision-Making Instrument--Revised | Bartlett | Western Journal of Nursing Research | 2013-02-00 Feb 2013 | The purpose of this study was to evaluate the psychometric properties of the Shared Decision-Making Inventory-Revised (SDMI-R) to measure four constructs (knowledge, attitudes, self-efficacy, and intent) theoretically defined as vital in discussing the human papillomavirus (HPV) disease and vaccine with clients. The SDMI-R was distributed to a sample (N = 1,525) of school nurses. Correlational matrixes denoted moderate to strong correlations, indicating adequate internal reliability. Reliability for the total instrument was satisfactory (α = .874) along with Attitude, Self-Efficacy and Intent subscales .828, .917, .891, respectively. Exploratory factor analysis revealed five components that explained 75.96% of the variance. | 0 |  |  |  |  |  | 1 |
| Psychometric properties of the ‘Skala Kepuasan Interaksi Perubatan-11’ to measure patient satisfaction with physician-patient interaction in Malaysia | Aziz | Family Practice | 2014-04-00 avril 2014 | Background: Patient satisfaction influences the outcomes of the patient-physician encounter. Objective: The objective of this study was to validate the Malay version patient satisfaction (MISS-21) questionnaire using a confirmatory validity approach. Methods: A cross-sectional study was conducted involving 252 patients attending primary health clinic, Hospital Universiti Sains Malaysia. Construct validity (convergent and discriminant) using confirmatory factor analysis and internal consistency were performed after the translation, content validity and face validity processes. Criterion validity was assessed using Pearson correlations with the scale of shared decision making 9-item questionnaire (SDMQ-9). The data was analysed using Analysis of Moment Structure version 19. Results: A total of 252 (100%) outpatients responded to this study. The final model that consists of three domains with 11 items had a good fit; (χ²(df) = 65.805 (32), P &lt; 0.001, Tucker–Lewis indices = 0.902, comparative fit index = 0.927, root mean square error of approximation = 0.061, standardized root mean square residual = 0.058). Composite reliability and average variance extracted of the domains ranged from 0.541 to 0.760 and 0.240 to 0.522, respectively. The SDMQ-9 had a moderate correlation with the total score of the final construct (r = 0.406, P &lt;0.001). Conclusion: The study suggested that the three-factor model with 11 items of the Malay version MISS-21 could be used to assess patient satisfaction on patient-physician interaction in primary health care setting because it is acceptably valid, reliable and simple. The validated Malay version questionnaire was called as ‘Skala Kepuasan Interaksi Perubatan-11’. (PsycINFO Database Record (c) 2014 APA, all rights reserved). (journal abstract) | 0 | 1 |  |  |  |  |  |
| Psychometric properties of the decisional balance for patient choice in substance abuse treatment | Finnell | Issues in Mental Health Nursing | 2011-00-00 2011 | In the context of patient-centered care and the increasing number of evidence-based substance abuse treatments, outpatient substance abuse treatment programs are poised to provide patients with a menu of options. Not all patients will be ready for such an open choice field in substance abuse treatment and they will undoubtedly differ on how they weigh the risks and benefits of having autonomy to choose their own treatment. Given the lack of an existing valid measure to assess this decision making process, this study sought to establish a measure to assess the relative weight that patients give to the pros and cons of choosing their own substance abuse treatment. Construct validity of the Decisional Balance for Patient Choice in Substance Abuse Treatment was assessed in a sample of 231 outpatients using confirmatory factor analysis. As another validity aspect, the use of decisional balance across a continuum of choice options also was investigated. The model fit was acceptable (CFI = 0.904). Internal consistency of the measure was established. The final 22-item measure revealed sound psychometric properties, but further testing is warranted. | 0 |  |  |  | 1 |  |  |
| Psychometric properties of the patient activation measure among multimorbid older adults | Skolasky | Health Services Research | 2011-04-00 Apr 2011 | OBJECTIVES: The Patient Activation Measure (PAM) quantifies the extent to which people are informed about and involved in their health care. Objectives were to determine the psychometric properties of PAM among multimorbid older adults and evaluate a theoretical, four-stage model of patient activation. Methods. A cross-sectional analysis was used to assess the psychometric properties of PAM. Internal consistency was assessed using Cronbach α. Construct validity was evaluated using general linear modeling to compute associations between PAM scores and health-related behaviors, functional status, and health care quality. Latent class analysis was used to evaluate the theoretical four-stage structure of patient activation. STUDY SETTING: Participants in a randomized trial of Guided Care (N = 855), a model of comprehensive health care for older adults with chronic conditions that put them at risk of using health services heavily during the coming year. PRINCIPAL FINDINGS: Higher PAM activation scores and stage were positively associated with higher functional status, health care quality, and adherence to some health behaviors. Latent class analysis supported the multistage theory of patient activation. CONCLUSIONS: The PAM is a reliable, valid, and potentially clinically useful measure of patient activation for multimorbid older adults. | 0 | 1 |  |  |  |  |  |
| Questionnaire for patient participation in emergency departments: development and psychometric testing | Frank | Journal of Advanced Nursing | 2011-03-00 Mar 2011 | AIM: The aim of the study was to develop and test the psychometric properties of a patient participation questionnaire in emergency departments. BACKGROUND: Patient participation is an important indicator of the quality of healthcare. International and national healthcare policy guidelines promote patient participation. While patients cared for in emergency departments generally express dissatisfaction with their care, a review of the literature fails to reveal any scientifically tested instruments for assessing patient participation from the perspective of patients. METHODS: A methodological study was conducted involving a convenience sample of 356 patients recently cared for in emergency departments in Sweden. Data were collected in 2008 and analysed for construct and criterion validity, also homogeneity and stability reliability. RESULTS: A 17-item questionnaire was developed. Two separate factor analyses revealed a distinct 4-factor solution which was labelled: Fight for participation, Requirement for participation, Mutual participation and Participating in getting basic needs satisfied. Criterion validity testing showed 9 out of 20 correlations above 0.30 and, of these, three were moderate correlations of 0.62, 0.63 and 0.70. Cronbach's alpha coefficient ranged from 0.63 to 0.84 and test-retest varied between 0.59 and 0.93. CONCLUSION: The results signify evidence of acceptable validity and reliability, and the questionnaire makes it possible to evaluate patient participation in emergency department caring situations. In addition, it produces data which are usable by a diverse range of healthcare professionals. | 0 |  |  |  | 1 |  |  |
| Reliability and validity of the German version of the OPTION scale | Hirsch | Health Expectations: An International Journal of Public Participation in Health Care and Health Policy | 2012-12-00 Dec 2012 | OBJECTIVE: To examine the psychometric properties of the German version of the 'observing patient involvement' scale (OPTION) by analysing video recordings of primary care consultations dealing with counselling in cardiovascular prevention. DESIGN: Cross-sectional assessment of physician-patient interaction by two rater pairs and two experts in shared decision making (SDM). SETTING: Primary care. PARTICIPANTS: Fifteen general practitioners provided 40 videographed consultations. MEASUREMENTS: Video ratings using the OPTION instrument. RESULTS: Mean differences on item level between the four raters were quite large. Most items were skewed towards minimal levels of shared decision making. Measures of inter-rater association showed low to moderate associations on item level and high associations on total score level. Cronbach-α of the whole scale based on the data of all four raters is 0.90 and therefore on a high level. An oblique factor analysis revealed two factors, but both factors were highly correlated so we can confirm a one-dimensional structure of the instrument. ROC analyses between the rater total scores and dichotomized expert ratings (SDM yes/no) revealed a good discriminability of the OPTION total score. Physicians with more expertise in shared decision making received higher OPTION ratings. CONCLUSIONS: The German version of the OPTION scale is reliable at total score level. Some items need further revision in the direction of more concrete, observable behaviour. We were only able to perform a quasi-validation of the scale. Validity issues need further research efforts. | 1 |  |  |  |  |  |  |
| Shared decision making in oncology: assessing oncologist behaviour in consultations in which adjuvant therapy is considered after primary surgical treatment | Singh | Health Expectations: An International Journal of Public Participation in Health Care and Health Policy | 2010-09-00 Sep 2010 | INTRODUCTION: Shared decision making (SDM) is now considered a desirable goal in health care, yet little is known about current practice in cancer care, and its impact on patient outcomes. This study aimed to develop an oncology-specific coding system for SDM, explore variations in SDM according to patient and disease characteristics, determine the relationship between SDM and patient satisfaction with the consultation, and explore the impact of SDM on patient anxiety. METHODS: Sixty-three medical and radiation oncology consultations with patients with primary cancer involving consideration of adjuvant therapy after surgery were audio-taped, transcribed and coded. Intra and inter-rater reliability of the coding system was 95 and 90% respectively. Patients completed questionnaires before and after the consultation. RESULTS: Construct validity of the SDM coding system was successfully conducted. Oncologists demonstrated on average under 11 of 18 SDM behaviours. Behaviours seeking patient preferences were particularly rare. SDM behaviours were more apparent in consultations involving female breast cancer patients. SDM behaviour scores in combination with patient involvement preference could predict achievement of patient involvement preference but not overall patient satisfaction. Although there was no overall relationship between patient anxiety and SDM scores, it did appear that physicians may change SDM behaviour according to patient factors including anxiety. CONCLUSION: Our findings reinforce the importance of the doctor in facilitating shared decision making in oncology consultations. | 1 |  |  |  |  |  |  |
| Sharing decisions in breast cancer care: Development of the Decision Analysis System for Oncology (DAS-O) to identify shared decision making during treatment consultations | Brown | Health Expectations: An International Journal of Public Participation in Health Care & Health Policy | 2011-03-00 mars 2011 | Background: Shared Decision Making (SDM) is widely accepted as the preferred method for reaching treatment decisions in the oncology setting including those about clinical trial participation: however, there is some disagreement between researchers over the components of SDM. Specific standardized coding systems are needed to help overcome this difficulty. Objective: The first objective was to describe the development of an oncology specific SDM coding system, the DAS-O. The second objective was to provide reliability and validity data supporting the DAS-O. Setting and participants: Consultation data were available from tertiary cancer center out patient oncology clinics in: Australia, New Zealand (ANZ), Switzerland, Germany and Austria (SGA). Patients were women with a confirmed diagnosis of early stage breast cancer. Reliability data were from 18 randomly selected coded transcripts drawn from ANZ and SGA. Concurrent validity data are from 55 (ANZ) consultations. Measurement: Inter and Intra rater reliability data was evaluated using Kappa correlation statistics and correlation coefficients. Correlation coefficients were used to assess concurrent validity between the DAS-O and two other SDM coding systems, OPTION and DSAT. Results: Inter and Intra rater reliability for the system were high with average Kappas of 0.58 and 0.65 respectively. Correlation coefficients between DAS-O and OPTION was 0.73 and &gt; 0.5 for DSAT. (PsycINFO Database Record (c) 2012 APA, all rights reserved). (journal abstract) | 1 |  |  |  |  |  |  |
| Some but not all dyadic measures in shared decision making research have satisfactory psychometric properties | Légaré | Journal of Clinical Epidemiology | 2012-12-00 Dec 2012 | OBJECTIVE: To assess the psychometric properties of dyadic measures for shared decision making (SDM) research. STUDY DESIGN AND SETTING: We conducted an observational cross-sectional study in 17 primary care clinics with physician-patient dyads. We used seven subscales to measure six elements of SDM: (1) defining the problem, presenting options, and discussing pros and cons; (2) clarifying the patient's values and preferences; (3) discussing the patient's self-efficacy; (4) drawing on the doctor's knowledge; (5) verifying the patient's understanding; and (6) assessing the patient's uncertainty. We assessed the reliability and invariance of the factorial structure and considered a measure to be dyadic if the factorial structure of the patient version was similar to that of the physician version and if there was equality of loading (no significant chi-square). RESULTS: We analyzed data for 264 physicians and 269 patients. All measures except one showed adequate reliability (Cronbach alpha, 0.70-0.93) and factorial validity (root mean square error of approximation, 0.000-0.06). However, we found only four measures to be dyadic (P>0.05): the values clarification subscale, perceived behavioral subscale, information-verifying subscale, and uncertainty subscale. CONCLUSION: The subscales for values clarification, perceived behavioral control, information verifying, and uncertainty are appropriate dyadic measures for SDM research and can be used to derive dyadic indices. | 1 |  |  |  |  |  |  |
| The 9-item Shared Decision Making Questionnaire (SDM-Q-9). Development and psychometric properties in a primary care sample | Kriston | Patient Education and Counseling | 2010-07-00 Jul 2010 | OBJECTIVE: To develop and psychometrically test a brief patient-report instrument for measuring Shared Decision Making (SDM) in clinical encounters. METHODS: We revised an existing instrument (Shared Decision Making Questionnaire; SDM-Q), including the generation of new items and changing the response format. A 9-item version (SDM-Q-9) was developed and tested in a German primary care sample of 2351 patients via face validity ratings, investigation of acceptance, as well as factor and reliability analysis. Findings were cross-validated in a randomly selected subsample. RESULTS: The SDM-Q-9 showed face validity and high acceptance. Factor analysis revealed a clearly one-dimensional nature of the underlying construct. Both item difficulties and discrimination indices proved to be appropriate. Internal consistency yielded a Cronbach's alpha of 0.938 in the test sample. CONCLUSION: The SDM-Q-9 is a reliable and well accepted instrument. Generalizability of the findings is limited by the elderly sample living in rural areas of Germany. While the current results are promising, further testing of criterion validity and administration in other populations is necessary. PRACTICE IMPLICATIONS: The SDM-Q-9 can be used in studies investigating the effectiveness of interventions aimed at the implementation of SDM and as a quality indicator in health services assessments. | 1 |  |  |  |  |  |  |
| The assessment of satisfaction with care in the perinatal period | Britton | Journal of Psychosomatic Obstetrics and Gynaecology | 2012-06-00 Jun 2012 | Patient satisfaction is frequently used to evaluate the quality of medical care and to guide the development of health care services. Improved satisfaction is a goal recommended by the Institute of Medicine and the government of the United Kingdom for health care reform. During the perinatal period, dynamic changes in physical and psychological state impose unique challenges in the assessment of satisfaction. This article reviews the measurement of satisfaction with care, together with factors that may influence satisfaction and its measurement during the perinatal period. Recommendations are also provided for further research and development of satisfaction instruments and potential interventions to improve satisfaction with perinatal care. | 0 |  |  |  | 1 |  |  |
| The congruence of patient communication preferences and physician communication behavior in cardiac patients | Farin | Journal of Cardiopulmonary Rehabilitation and Prevention | 2011-12-00 2011 Nov-Dec | BACKGROUND: The purpose of this study was to examine the communication preferences of patients with chronic ischemic heart disease (CIHD) and matching between the preferences and physician communication behavior. Prior to this, psychometric testing was performed on a questionnaire on the perceived communication behavior of the physician (KOVA Questionnaire). METHODS: Patients with CIHD undergoing rehabilitation (N = 342) in Germany were questioned. At the beginning of rehabilitation, patients answered questions about their communication preferences (KOPRA Questionnaire), and at the end of rehabilitation, they answered questions regarding the perceived communication behavior of the physician. Preference-matching values were determined by combining the KOPRA and KOVA items. RESULTS: The KOVA Questionnaire psychometric properties proved to be good. Patients with CIHD indicated clear and open communication and patient participation were especially important. This was followed by emotionally supportive communication and, finally, communication about personal things. Overall, the behavior of physicians corresponded quite closely with the patients' communication preferences. However, preference matching was low (the physicians demonstrated too little of the desired behavior) regarding the open communication of bad news and explanation of treatments. There was relatively high fulfillment of expectations on the part of patients in terms of seeking information from the physician, the physician's explanation of the diagnosis, and regarding aspects of shared decision making. We observed no gender differences, but did identify age-group differences. CONCLUSIONS: The instruments developed (KOPRA and KOVA questionnaires) can be used for communication studies of patients with chronic conditions. However, some patient communication needs seem to require greater consideration from physicians. | 0 |  |  |  | 1 |  |  |
| The decisional conflict scale: moving from the individual to the dyad level | Légaré | Zeitschrift Für Evidenz, Fortbildung Und Qualität Im Gesundheitswesen | 2012-00-00 2012 | Decisional conflict is a central determinant of decision making, particularly in the context of uncertainty. It is also one of the most frequently reported outcomes in studies on decision support interventions. Decisional conflict is defined as personal uncertainty about which option to choose. The Decisional Conflict Scale (DCS) is a self-administered questionnaire that was originally designed to assess decisional conflict in patients. The scale has since been adapted to and tested among health professionals, since decisional conflict as seen by doctors, nurses and other healthcare providers has proven useful in evaluating the quality of the shared decision making (SDM) process. In recent years, however, more and more researchers have found that evaluating the perspectives of the patient and the health professional as interdependent members of a dyad, rather than as two autonomous individuals, offers exciting avenues for developing interventions to improve decision making in the clinical setting. For that reason, the SDM community has increasingly turned its attention to a dyadic approach to SDM. In this paper, we briefly review the history of the Dyadic Decisional Conflict Scale (D-DCS), update its psychometrics based on published work, and propose a research agenda for refining it further. | 0 | 1 |  |  |  |  |  |
| The development and initial validation of a clinical tool for patients' preferences on patient participation - The 4Ps | Eldh | Health Expectations: An International Journal of Public Participation in Health Care and Health Policy | 2014-06-17 Jun 17, 2014 | AIMS: To report on the development and initial testing of a clinical tool, The Patient Preferences for Patient Participation tool (The 4Ps), which will allow patients to depict, prioritize, and evaluate their participation in health care. BACKGROUND: While patient participation is vital for high quality health care, a common definition incorporating all stakeholders' experience is pending. In order to support participation in health care, a tool for determining patients' preferences on participation is proposed, including opportunities to evaluate participation while considering patient preferences. METHODS: Exploratory mixed methods studies informed the development of the tool, and descriptive design guided its initial testing. The 4Ps tool was tested with 21 Swedish researcher experts (REs) and patient experts (PEs) with experience of patient participation. Individual Think Aloud interviews were employed to capture experiences of content, response process, and acceptability. RESULTS: 'The 4Ps' included three sections for the patient to depict, prioritize, and evaluate participation using 12 items corresponding to 'Having Dialogue', 'Sharing Knowledge', 'Planning', and 'Managing Self-care'. The REs and PEs considered 'The 4Ps' comprehensible, and that all items corresponded to the concept of patient participation. The tool was perceived to facilitate patient participation whilst requiring amendments to content and layout. CONCLUSIONS: A tool like The 4Ps provides opportunities for patients to depict participation, and thus supports communication and collaboration. Further patient evaluation is needed to understand the conditions for patient participation. While The 4Ps is promising, revision and testing in clinical practice is required. | 0 |  |  |  | 1 |  |  |
| The German version of the Four Habits Coding Scheme — Association between physicians' communication and shared decision making skills in the medical encounter | Scholl | Patient Education and Counseling | 2014-02-00 février 2014 | Objective: To translate a measure of physicians’ communication skills, the Four Habits Coding Scheme (4HCS), into German, to examine its psychometric properties, and to analyze its association with the OPTION Scale, which assesses physicians’ shared decision making (SDM) behavior. Methods: We performed a secondary data analysis of 67 audio-recorded medical consultations. Reliability, internal consistency, and factorial validity of the translated 4HCS were analyzed. The association with the OPTION Scale was examined using correlation and linear regression. Results: Testing of reliability revealed intraclass correlation coefficients above .70. Results regarding internal consistency and factorial validity were inconclusive. The correlations between the OPTION score and the four dimensions of the 4HCS were .04 (p = .782), −.14 (p = .303), −.15 (p = .279) and .55 (p &lt; .001), respectively. In multiple regression the four dimensions of the 4HCS explained substantial amount of variation in the OPTION scores (R² = .42, P &lt; .001). Conclusion: The measure showed good observer reliability, however further testing is necessary. Due to the strong interrelation of both measures, SDM should be seen in the context of broader communication skills. Practice implications: The 4HCS can be used in research and medical education. Further studies are necessary that investigate SDM within the context of communication skills. (PsycINFO Database Record (c) 2014 APA, all rights reserved). (journal abstract) | 0 |  |  |  | 1 |  |  |
| The importance and complexity of regret in the measurement of ‘good’ decisions: A systematic review and a content analysis of existing assessment instruments | Joseph‐Williams | Health Expectations: An International Journal of Public Participation in Health Care & Health Policy | 2011-03-00 mars 2011 | Background or context: Regret is a common consequence of decisions, including those decisions related to individuals' health. Several assessment instruments have been developed that attempt to measure decision regret. However, recent research has highlighted the complexity of regret. Given its relevance to shared decision making, it is important to understand its conceptualization and the instruments used to measure it. Objectives: To review current conceptions of regret. To systematically identify instruments used to measure decision regret and assess whether they capture recent conceptualizations of regret. Search strategy: Five electronic databases were searched in 2008. Search strategies used a combination of MeSH terms (or database equivalent) and free text searching under the following key headings: 'Decision' and 'regret'and 'measurement'. Follow-up manual searches were also performed. Inclusion criteria: Articles were included if they reported the development and psychometric testing of an instrument designed to measure decision regret, or the use of a previously developed and tested instrument. Main results: Thirty-two articles were included: 10 report the development and validation of an instrument that measures decision regret and 22 report the use of a previously developed and tested instrument. Content analysis found that existing instruments for the measurement of regret do not capture current conceptualizations of regret and they do not enable the construct of regret to be measured comprehensively. Conclusions: Existing instrumentation requires further development. There is also a need to clarify the purpose for using regret assessment instruments as this will, and should, focus their future application. (PsycINFO Database Record (c) 2014 APA, all rights reserved). (journal abstract) | 0 | 1 |  |  |  |  |  |
| The OPTION scale for the assessment of shared decision making (SDM): methodological issues | Nicolai | Zeitschrift Für Evidenz, Fortbildung Und Qualität Im Gesundheitswesen | 2012-00-00 2012 | BACKGROUND: Promoting patient involvement in medical decision making has become a desirable goal in medical consultations. Reliable and valid measures are necessary to evaluate interventions designed to promote shared decision making and to understand determinants and associations. The OPTION ("observing patient involvement") scale is the most prominent observation instrument for assessing the extent to which clinicians actively involve patients in decision making. OBJECTIVE: This paper discusses psychometric and methodological characteristics of the OPTION scale. RESULTS: There is little support for the purported unidimensional structure. Although reliabilities are acceptable, results are highly heterogeneous across studies. There is also little evidence concerning validity. In particular, studies mainly failed to support convergent validity. Additional issues pertain to lack of item independence, restriction of range, and failure to consider dyadic aspects. CONCLUSIONS: Given these findings, a number of methodological and conceptual issues still need to be addressed for the effective measurement of patient involvement. Directions for future research are discussed. | 1 |  |  |  |  |  |  |
| The Patient Enablement Instrument-French version in a family practice setting: a reliability study | Hudon | BMC family practice | 2011-00-00 2011 | BACKGROUND: Patient enablement can be defined as the extent to which a patient is capable of understanding and coping with his or her health issues. This concept is linked to a number of health outcomes such as self-management of chronic diseases and quality of life. The Patient Enablement Instrument (PEI) was designed to measure this concept after a medical consultation. The instrument, in its original form and its translations into several languages, has proven to be reliable and valid. The purpose of this study was to evaluate the reliability of the French version of the PEI (PEI-Fv) in a family practice setting. METHODS: One hundred and ten participants were recruited in a family medicine clinic in the Saguenay region of Quebec (Canada). The PEI-Fv was completed twice, immediately after consultation with a physician (T1) and 2 weeks after the consultation (T2). The internal consistency of the tool was assessed with Cronbach's α and test-retest reliability by intraclass correlation coefficient. RESULTS: The mean score for the PEI-Fv was 5.06 ± 3.97 (95% confidence interval [CI]: 4.30-5.81) at T1 and 4.63 ± 3.90 (95% CI: 3.82-5.44) at T2. Cronbach's α was high at T1 (α1 = 0.93; 95% CI: 0.91-0.95) and T2 (α2 = 0.93; 95% CI: 0.91-0.95). The intraclass correlation coefficient was 0.62 (95% CI: 0.48-0.74), indicating a moderate test-retest reliability. CONCLUSIONS: The internal consistency of the PEI-Fv is excellent. Test-retest reliability was moderate to good. Test-retest reliability should be examined in further studies at a less than 2-week interval to reduce maturation bias. This instrument can be used to measure enablement after consultation in a French-speaking family practice setting. | 0 |  |  |  | 1 |  |  |
| The psychometric properties of CollaboRATE: a fast and frugal patient-reported measure of the shared decision-making process | Barr | Journal of Medical Internet Research | 2014-00-00 2014 | BACKGROUND: Patient-centered health care is a central component of current health policy agendas. Shared decision making (SDM) is considered to be the pinnacle of patient engagement and methods to promote this are becoming commonplace. However, the measurement of SDM continues to prove challenging. Reviews have highlighted the need for a patient-reported measure of SDM that is practical, valid, and reliable to assist implementation efforts. In consultation with patients, we developed CollaboRATE, a 3-item measure of the SDM process. OBJECTIVE: There is a need for scalable patient-reported measure of the SDM process. In the current project, we assessed the psychometric properties of CollaboRATE. METHODS: A representative sample of the US population were recruited online and were randomly allocated to view 1 of 6 simulated doctor-patient encounters in January 2013. Three dimensions of SDM were manipulated in the encounters: (1) explanation of the health issue, (2) elicitation of patient preferences, and (3) integration of patient preferences. Participants then completed CollaboRATE (possible scores 0-100) in addition to 2 other patient-reported measures of SDM: the 9-item Shared Decision Decision Making Questionnaire (SDM-Q-9) and the Doctor Facilitation subscale of the Patient's Perceived Involvement in Care Scale (PICS). A subsample of participants was resurveyed between 7 and 14 days after the initial survey. We assessed CollaboRATE's discriminative, concurrent, and divergent validity, intrarater reliability, and sensitivity to change. RESULTS: The final sample consisted of 1341 participants. CollaboRATE demonstrated discriminative validity, with a significant increase in CollaboRATE score as the number of core dimensions of SDM increased from zero (mean score: 46.0, 95% CI 42.4-49.6) to 3 (mean score 85.8, 95% CI 83.2-88.4). CollaboRATE also demonstrated concurrent validity with other measures of SDM, excellent intrarater reliability, and sensitivity to change; however, divergent validity was not demonstrated. CONCLUSIONS: The fast and frugal nature of CollaboRATE lends itself to routine clinical use. Further assessment of CollaboRATE in real-world settings is required. | 1 |  |  |  |  |  |  |
| The Swiss Health Literacy Survey: development and psychometric properties of a multidimensional instrument to assess competencies for health | Wang | Health Expectations: An International Journal of Public Participation in Health Care and Health Policy | 2014-06-00 Jun 2014 | BACKGROUND: Growing recognition of the role of citizens and patients in health and health care has placed a spotlight on health literacy and patient education. OBJECTIVE: To identify specific competencies for health in definitions of health literacy and patient-centred concepts and empirically test their dimensionality in the general population. METHODS: A thorough review of the literature on health literacy, self-management, patient empowerment, patient education and shared decision making revealed considerable conceptual overlap as competencies for health and identified a corpus of 30 generic competencies for health. A questionnaire containing 127 items covering the 30 competencies was fielded as a telephone interview in German, French and Italian among 1255 respondents randomly selected from the resident population in Switzerland. FINDINGS: Analyses with the software MPlus to model items with mixed response categories showed that the items do not load onto a single factor. Multifactorial models with good fit could be erected for each of five dimensions defined a priori and their corresponding competencies: information and knowledge (four competencies, 17 items), general cognitive skills (four competencies, 17 items), social roles (two competencies, seven items), medical management (four competencies, 27 items) and healthy lifestyle (two competencies, six items). Multiple indicators and multiple causes models identified problematic differential item functioning for only six items belonging to two competencies. CONCLUSIONS: The psychometric analyses of this instrument support broader conceptualization of health literacy not as a single competence but rather as a package of competencies for health. | 0 |  |  |  | 1 |  |  |
| Trying to optimise the German version of the OPTION scale regarding the dyadic aspect of shared decision making | Keller | Methods of Information in Medicine | 2013-00-00 2013 | OBJECTIVES: The OPTION scale ("observing patient involvement in decision making") assesses the extent to which clinicians involve patients in decisions across a range of situations in clinical practice. It so far just covers physician behavior. We intended to modify the scoring of the OPTION scale to incorporate active patient behavior in consultations. METHODS: Modification was done on scoring level, attempting a dyadic, relationship-centred approach in that high ratings can be evoked also by the behaviour of active patients. The German version of the OPTION scale was compared with a modified version by analysing video recordings of primary care consultations dealing with cardiovascular prevention. Fifteen general practitioners provided 40 videotaped consultations. Videos were analysed by two rater pairs and two experts in shared decision making (SDM). RESULTS: Reliability measures of the modified version were lower than those of the original scale. Significant associations of the dichotomised scale with the expert SDM rating as well as with physicians' expertise in SDM were only found for the modified OPTION scale. Receiver Operating Characteristic (ROC) analyses confirmed a valid differentiation between the presence of SDM (yes/no) on total score level, even though the cut-off point was quite low. Standard deviations of the single items in the modified version were higher compared to the original OPTION scale, while the means of total scores were similar. CONCLUSIONS: The original OPTION scale is physician-centered and neglects the activity and a possible self-involvement of the patient. Our modified instruction was able to capture the dyadic element partially. The development of a separate dyadic instrument might be more promising. | 1 |  |  |  |  |  |  |
| User involvement in in-patient mental health services: operationalisation, empirical testing, and validation | Storm | Journal of Clinical Nursing | 2010-07-00 Jul 2010 | AIMS: This study presents development, empirical testing and validation of an instrument measuring service user involvement in in-patient mental health from the mental health professionals' perspective. BACKGROUND: Service user involvement is high on the agenda in European mental health policies. In Norway, focus is on enhanced service user involvement at both the individual and organisational levels of in-patient mental health services. Mental health professionals are in an important position to ensure opportunities for real user involvement in in-patient mental health care. However, there is a need for more empirical knowledge on how mental health professionals attend to service user involvement. DESIGN: Survey. METHODS: A self-report questionnaire was designed and administered to 121 mental health professionals, with 98 responses, working in a community-based mental health centre in western Norway. Factor analysis procedures together with reliability testing were performed. RESULTS: A 30-items instrument was developed. The instrument contains four components/subscales: (1) Democratic patient involvement (mean score 3·74, Cronbach's alpha 0·81), (2) Carer involvement (mean score 3·67, Cronbach's alpha 0·82), (3) Assisted patient involvement (mean score 4·05, Cronbach's alpha 0·78) and (4) Management support (mean score 4·10, Cronbach's alpha 0·75). These subscales were found to be essential to service user involvement in the context of in-patient mental health care. The total mean score for the instrument was 3·88, Cronbach's alpha 0·88. CONCLUSION: Empirical testing of the instrument demonstrates that the measurement of mental health professionals' perception of service user involvement has a reasonable level of construct validity and reliability. RELEVANCE TO CLINICAL PRACTICE: We have developed a measurement instrument with items reflecting essential characteristics to user involvement in in-patient mental health services. We believe that answering this questionnaire on the subject user involvement can act as one step towards enhancing awareness of this issue and to assess user-oriented practices in treatment and services. | 0 |  |  |  | 1 |  |  |
| Using a ‘talk’ model of shared decision making to propose an observation-based measure: Observer OPTION5 Item | Elwyn | Patient Education and Counseling | 2013-11-00 novembre 2013 | Objective: To propose a revised Observer OPTION measure of shared decision making. Methods: We analyzed published models to identify the core components of a parsimonious conceptual framework of shared decision making. By using this framework, we developed a revised measure combining data from an observational study of clinical practice in Canada with our experience of using Observer OPTION12 Item. Results: Our conceptual framework for shared decision making composed of justifying deliberative work, followed by the steps of describing options, information exchange, preference elicitation, and preference integration. By excluding items in Observer OPTION12 Item that were seldom observed or not aligned to a robust construct, we propose Observer OPTION5 Item. Conclusion: Although widely used, Observer OPTION12 Item did not give sufficient attention to preference elicitation and integration, and included items that were not specific to a core construct of shared decision making. We attempted to remedy these shortcomings by proposing a shorter, more focused measure. Practice implications: Observer OPTION5 Item requires evaluation; we hope that it will be useful as both a research tool and as a formative measure of clinical practice. (PsycINFO Database Record (c) 2014 APA, all rights reserved). (journal abstract) | 0 | 1 |  |  |  |  |  |
| Validation of a preparation for decision making scale | Bennett | Patient Education and Counseling | 2010-01-00 Jan 2010 | OBJECTIVE: The Preparation for Decision Making (PrepDM) scale was developed to evaluate decision processes relating to the preparation of patients for decision making and dialoguing with their practitioners. The objective of this study was to evaluate the scale's psychometric properties. METHODS: From July 2005 to March 2006, after viewing a decision aid prescribed during routine clinical care, patients completed a questionnaire including: demographic information, treatment intention, decisional conflict, decision aid acceptability, and the PrepDM scale. RESULTS: Four hundred orthopaedic patients completed the questionnaire. The PrepDM scale showed significant correlation with the informed (r=-0.21, p<0.01) and support (r=-0.13, p=0.01) subscales (DCS); and discriminated significantly between patients who did and did not find the decision aid helpful (p<0.0001). Alpha coefficients for internal consistency ranged from 0.92 to 0.96. The scale is strongly unidimensional (principal components analysis) and Item Response Theory analyses demonstrated that all ten scale items function very well. CONCLUSION: The psychometric properties of the PrepDM scale are very good. PRACTICE IMPLICATIONS: The scale could allow more comprehensive evaluation of interventions designed to prepare patients for shared-decision making encounters regarding complex health care decisions. | 0 |  |  |  |  | 1 |  |
| Validation of shared decision-making questionnaire in the dental encounter | Peters |  | 2013-00-00 2013 | Communication within the dental encounter is paramount to successful clinical outcomes. In this chapter we examine the concept of shared decision-making (SDM) in patients who have received treatment for a single missing tooth. Method: A cross sectional study design was used for the study. Patients were recruited from a university dental clinic and a private dental practice. Exploratory factor analysis was used to identify the dimensions of patient's SDM, their level of dental knowledge and their treatment satisfaction. Results: Exploratory factor analysis showed that 9 factors were extracted from the questionnaire. Overall, the 9 factor structure had a high level of reliability for each individual dimension. Of the 108 participants, only 26.5% were exposed to SDM. Independent sample t tests showed that there was no significant difference between the SDM group and comparison group on levels of denture knowledge, post treatment functional and economic satisfaction. However, a significant difference was seen in regards to knowledge of other treatment alternatives and post treatment aesthetic/social satisfaction, with the SDM group reporting higher levels. Conclusions: The results suggest that a 9 factor solution can be used to assess the patient's experience in the dental encounter. In regards to the use of SDM in the dental encounter, further research is required using a larger sample size from an increased number of dental practices. However, SDM is an important component of clinical dental practice and should be further explored in clinical dentistry. (PsycINFO Database Record (c) 2014 APA, all rights reserved). (chapter) | 0 |  | 1 |  |  |  |  |
| Validation of SURE, a four-item clinical checklist for detecting decisional conflict in patients | Ferron Parayre | Medical Decision Making: An International Journal of the Society for Medical Decision Making | 2014-01-00 Jan 2014 | BACKGROUND: We sought to determine the psychometric properties of SURE, a 4-item checklist designed to screen for clinically significant decisional conflict in clinical practice. METHODS: This study was a secondary analysis of a clustered randomized trial assessing the effect of DECISION+2, a 2-hour online tutorial followed by a 2-hour interactive workshop on shared decision making, on decisions to use antibiotics for acute respiratory infections. Patients completed SURE and also the Decisional Conflict Scale (DCS), as the gold standard, after consultation. We evaluated internal consistency of SURE using the Kuder-Richardson 20 coefficient (KR-20). We compared DCS and SURE scores using the Spearman correlation coefficient. We assessed sensitivity and specificity of SURE scores (cut-off score ≤3 out of 4) by identifying patients with and without clinically significant decisional conflict (DCS score >37.5 on a scale of 0-100). RESULTS: Of the 712 patients recruited during the trial, 654 completed both tools. SURE scores showed adequate internal consistency (KR-20 coefficient of 0.7). There was a significant correlation between DCS and SURE scores (Spearman's ρ = -0.45, P < 0.0001). The prevalence of clinically significant decisional conflict as estimated by the DCS was 5.2% (95% CI 3.7-7.3). Sensitivity and specificity of SURE ≤3 were 94.1% (95% CI 78.9-99.0) and 89.8% (95% CI 87.1-92.0), respectively. CONCLUSIONS: SURE shows adequate psychometric properties in a primary care population with a low prevalence of clinically significant decisional conflict. SURE has the potential to be a useful screening tool for practitioners, responding to the growing need for detecting clinically significant decisional conflict in patients. | 1 |  |  |  |  |  |  |
| Validation of the German version of the patient activation measure 13 (PAM13-D) in an international multicentre study of primary care patients | Brenk-Franz | PloS One | 2013-00-00 2013 | The patients' active participation in their medical care is important for patients with chronic diseases. Measurements of patient activation are needed for studies and in clinical practice. This study aims to validate the Patient Activation Measure 13 (PAM13-D) in German-speaking primary care patients. This international cross-sectional multicentre study enrolled consecutively patients from primary care practices in three German-speaking countries: Germany, Austria, and Switzerland. Patients completed the PAM13-D questionnaire. General Self-Efficacy scale (GSE) was used to assess convergent validity. Furthermore Cronbach's alpha was performed to assess internal consistency. Exploratory factor analysis was used to evaluate the underlying factor structure of the items. We included 508 patients from 16 primary care practices in the final analysis. Results were internally consistent, with a Cronbach's alpha of 0.84. Factor analysis revealed one major underlying factor. The mean values of the PAM13-D correlated significantly (r = 0.43) with those of the GSE. The German PAM13 is a reliable and valid measure of patient activation. Thus, it may be useful in primary care clinical practice and research. | 0 | 1 |  |  |  |  |  |
| Validation of the patient activation measure in a multiple sclerosis clinic sample and implications for care | Stepleman | Disability and Rehabilitation | 2010-00-00 2010 | PURPOSE: Patient engagement in multiple sclerosis (MS) care can be challenging at times given the unpredictable disease course, wide range of symptoms, variable therapeutic response to treatment and high rates of patient depression. Patient activation, a model for conceptualising patients' involvement in their health care, has been found useful for discerning patient differences in chronic illness management. The purpose of this study was to validate the patient activation measure (PAM-13) in an MS clinic sample. METHODS: This was a survey study of 199 MS clinic patients. Participants completed the PAM-13 along with measures of MS medication adherence, self-efficacy, depression and quality of life. RESULTS: Results from Rasch and correlation analyses indicate that the PAM-13 is reliable and valid for the MS population. Activation was associated with MS self-efficacy, depression and quality of life but not with self-reported medication adherence. Also, participants with relapse-remitting MS, current employment, or high levels of education were more activated than other subgroups. CONCLUSIONS: The PAM-13 is a useful tool for understanding health behaviours in MS. The findings of this study support further clinical consideration and investigation into developing interventions to increase patient activation and improve health outcomes in MS. | 0 |  |  |  | 1 |  |  |
| Validation of the patients' perceived involvement in care scale among patients with chronic pain | Jonsdottir | Scandinavian Journal of Caring Sciences | 2013-09-00 Sep 2013 | AIM: The aim of this study was to evaluate the psychometric properties of the Icelandic version of the Modified Patients' Perceived Involvement in Care Scale (M-PICS), I-PICS, an instrument measuring patients' perceptions of pain-related communication with health care providers (HCP). METHODS: The M-PICS was translated into Icelandic according to standard procedures for forward and backward translation. The questionnaire consisted of 20 items measuring four constructs: (i) the degree to which the HCP was perceived as controlling the information-exchange process; (ii) to what extent patients sought or shared information with their HCP; (iii) patients' perceived encouragement to raise questions and discuss their symptoms with their health care provider and (iv) patients' perceived participation in decision-making during the health care visit. The response options for each item ranged from one to five on a 5-point Likert scale, where higher scores indicated higher endorsement. RESULTS: One hundred and forty-nine participants with pain lasting longer than three months (77.2% women; mean age, 49.9 years) completed the questionnaire. To examine the construct validity of the I-PICS, a confirmatory factor analysis was performed, specifying four factors in congruence with the theoretical underpinnings of the original modified scale (M-PICS). Of the 20 items, 19 were retained, and the I-PICS factor structure was for the most part identical to the M-PICS, with the exception of three items that moved between factors and one item that did not fall decisively on one specific factor. Internal consistency (alpha) for the four factors ranged from 0.74 to 0.86 and was 0.86 for the total scale. The mean score on the total I-PICS was 2.67 on a one to five scale, ranging from 1.21 to 4.28 (possible range, 1-5). CONCLUSION: This study supports the four-factor structure of the M-PICS and that the I-PICS is a valid and reliable instrument for assessing patient-HCP communication. | 1 |  |  |  |  |  |  |
| Validation of the Spanish version of the 9-item Shared Decision-Making Questionnaire | De Las Cuevas | Health Expectations: An International Journal of Public Participation in Health Care and Health Policy | 2014-03-05 Mar 5, 2014 | OBJECTIVE: To translate and assess the psychometric properties of the 9-item Shared Decision-Making Questionnaire (SDM-Q-9) for measuring patients' perceptions of how clinicians' performance fits the SDM process. DESIGN: Cross-sectional study. SETTING AND PARTICIPANTS: Data were collected in primary care health centres. Patients suffering from chronic diseases and facing a medical decision were included in the study. MEASUREMENTS: The original German SDM-Q-9 was translated to Spanish using the process of cross-cultural adaptation of self-reported measures as the methodological model for Spanish translation. Reliability (internal consistency) and construct validity [exploratory (EFA) and confirmatory factor analysis (CFA)] were assessed. RESULTS: The final Spanish version of the SDM-Q-9 was tested in a primary care sample of 540 patients. The SDM-Q-9 presented adequate reliability and acceptable validity. Internal consistency yielded a Cronbach's alpha of 0.885 for the whole scale. EFA showed a two-factorial solution, and for the CFA, the best solution was obtained with a one-dimensional factor with the item 1 excluded, which produced the best indexes of fit. DISCUSSION AND CONCLUSIONS: The Spanish version of the SDM-Q-9 showed adequate reliability and acceptable validity parameters among primary care patients. The SDM-Q-9 is suitable for use in Spain and other Spanish-speaking countries with similarly organized health-care systems. The use of the SDM-Q-9 may contribute to the evaluation of SDM process from the patient's perspective. | 1 |  |  |  |  |  |  |
| Validity of a low literacy version of the Decisional Conflict Scale | Linder | Patient Education and Counseling | 2011-12-00 Dec 2011 | OBJECTIVE: To evaluate the psychometric properties of the 4-factor low literacy Decisional Conflict Scale (DCS-LL) with men eligible for prostate cancer screening (PCS). METHODS: We used baseline (T0; n=149) and post-intervention (T2; n=89) data from a randomized, controlled trial of a PCS decision aid to assess internal consistency reliability and construct, discriminant, and factor validity. RESULTS: There was evidence of excellent internal consistency reliability (α's≥.80) and fair construct validity (most r's≥.40) for the DCS-LL except for the Supported subscale. The DCS-LL was able to discriminate between men who had decided and those who had not. There was evidence for the original 4-factor model at T0 but exploratory analysis suggested a 3-factor solution at T0 and T2 with Informed and Value Clarity as one factor. CONCLUSION: For men eligible for PCS, feeling informed and feeling clear about values may not reflect distinct cognitive processes. Feeling supported may not be a factor contributing to uncertainty. PRACTICE IMPLICATIONS: Research should address whether current DCS subscales best represent the factors that contribute to uncertainty for PCS and for other screening decisions. Research should also explore the influence of health literacy on the factor structure of the DCS-LL. | 1 |  |  |  |  |  |  |

The third one is "Additional_file3.xls" : List of full-text articles assessed for eligibility (n=86) and full-text articles excluded (n=67). Description of data : title, first author (last name), name of journal, date of publication, abstract, selection's status (included or excluded), not relevant, study or instrument not accessible, other language, measured construct is not SDM, not about a clinical encounter, too specific.
